# Estimating global injuries morbidity and mortality: methods and data used in the Global Burden of Disease 2017 study

**DOI:** 10.1136/injuryprev-2019-043531

**Published:** 2020-08-24

**Authors:** Spencer L James, Chris D Castle, Zachary V Dingels, Jack T Fox, Erin B Hamilton, Zichen Liu, Nicholas L S Roberts, Dillon O Sylte, Gregory J Bertolacci, Matthew Cunningham, Nathaniel J Henry, Kate E LeGrand, Ahmed Abdelalim, Ibrahim Abdollahpour, Rizwan Suliankatchi Abdulkader, Aidin Abedi, Kedir Hussein Abegaz, Akine Eshete Abosetugn, Abdelrahman I Abushouk, Oladimeji M Adebayo, Jose C Adsuar, Shailesh M Advani, Marcela Agudelo-Botero, Tauseef Ahmad, Muktar Beshir Ahmed, Rushdia Ahmed, Miloud Taki Eddine Aichour, Fares Alahdab, Fahad Mashhour Alanezi, Niguse Meles Alema, Biresaw Wassihun Alemu, Suliman A Alghnam, Beriwan Abdulqadir Ali, Saqib Ali, Cyrus Alinia, Vahid Alipour, Syed Mohamed Aljunid, Amir Almasi-Hashiani, Nihad A Almasri, Khalid Altirkawi, Yasser Sami Abdeldayem Amer, Catalina Liliana Andrei, Alireza Ansari-Moghaddam, Carl Abelardo T Antonio, Davood Anvari, Seth Christopher Yaw Appiah, Jalal Arabloo, Morteza Arab-Zozani, Zohreh Arefi, Olatunde Aremu, Filippo Ariani, Amit Arora, Malke Asaad, Beatriz Paulina Ayala Quintanilla, Getinet Ayano, Martin Amogre Ayanore, Ghasem Azarian, Alaa Badawi, Ashish D Badiye, Atif Amin Baig, Mohan Bairwa, Ahad Bakhtiari, Arun Balachandran, Maciej Banach, Srikanta K Banerjee, Palash Chandra Banik, Amrit Banstola, Suzanne Lyn Barker-Collo, Till Winfried Bärnighausen, Akbar Barzegar, Mohsen Bayati, Shahrzad Bazargan-Hejazi, Neeraj Bedi, Masoud Behzadifar, Habte Belete, Derrick A Bennett, Isabela M Bensenor, Kidanemaryam Berhe, Akshaya Srikanth Bhagavathula, Pankaj Bhardwaj, Anusha Ganapati Bhat, Krittika Bhattacharyya, Zulfiqar A Bhutta, Sadia Bibi, Ali Bijani, Archith Boloor, Guilherme Borges, Rohan Borschmann, Antonio Maria Borzì, Soufiane Boufous, Dejana Braithwaite, Nikolay Ivanovich Briko, Traolach Brugha, Shyam S Budhathoki, Josip Car, Rosario Cárdenas, Félix Carvalho, João Mauricio Castaldelli-Maia, Carlos A Castañeda-Orjuela, Giulio Castelpietra, Ferrán Catalá-López, Ester Cerin, Joht S Chandan, Jens Robert Chapman, Vijay Kumar Chattu, Soosanna Kumary Chattu, Irini Chatziralli, Neha Chaudhary, Daniel Youngwhan Cho, Jee-Young J Choi, Mohiuddin Ahsanul Kabir Chowdhury, Devasahayam J Christopher, Dinh-Toi Chu, Flavia M Cicuttini, João M Coelho, Vera M Costa, Saad M A Dahlawi, Ahmad Daryani, Claudio Alberto Dávila-Cervantes, Diego De Leo, Feleke Mekonnen Demeke, Gebre Teklemariam Demoz, Desalegn Getnet Demsie, Kebede Deribe, Rupak Desai, Mostafa Dianati Nasab, Diana Dias da Silva, Zahra Sadat Dibaji Forooshani, Hoa Thi Do, Kerrie E Doyle, Tim Robert Driscoll, Eleonora Dubljanin, Bereket Duko Adema, Arielle Wilder Eagan, Demelash Abewa Elemineh, Shaimaa I El-Jaafary, Ziad El-Khatib, Christian Lycke Ellingsen, Maysaa El Sayed Zaki, Sharareh Eskandarieh, Oghenowede Eyawo, Pawan Sirwan Faris, Andre Faro, Farshad Farzadfar, Seyed-Mohammad Fereshtehnejad, Eduarda Fernandes, Pietro Ferrara, Florian Fischer, Morenike Oluwatoyin Folayan, Artem Alekseevich Fomenkov, Masoud Foroutan, Joel Msafiri Francis, Richard Charles Franklin, Takeshi Fukumoto, Biniyam Sahiledengle Geberemariyam, Hadush Gebremariam, Ketema Bizuwork Gebremedhin, Leake G Gebremeskel, Gebreamlak Gebremedhn Gebremeskel, Berhe Gebremichael, Getnet Azeze Gedefaw, Birhanu Geta, Agegnehu Bante Getenet, Mansour Ghafourifard, Farhad Ghamari, Reza Ghanei Gheshlagh, Asadollah Gholamian, Syed Amir Gilani, Tiffany K Gill, Amir Hossein Goudarzian, Alessandra C Goulart, Ayman Grada, Michal Grivna, Rafael Alves Guimarães, Yuming Guo, Gaurav Gupta, Juanita A Haagsma, Brian James Hall, Randah R Hamadeh, Samer Hamidi, Demelash Woldeyohannes Handiso, Josep Maria Haro, Amir Hasanzadeh, Shoaib Hassan, Soheil Hassanipour, Hadi Hassankhani, Hamid Yimam Hassen, Rasmus Havmoeller, Delia Hendrie, Fatemeh Heydarpour, Martha Híjar, Hung Chak Ho, Chi Linh Hoang, Michael K Hole, Ramesh Holla, Naznin Hossain, Mehdi Hosseinzadeh, Sorin Hostiuc, Guoqing Hu, Segun Emmanuel Ibitoye, Olayinka Stephen Ilesanmi, Leeberk Raja Inbaraj, Seyed Sina Naghibi Irvani, M Mofizul Islam, Sheikh Mohammed Shariful Islam, Rebecca Q Ivers, Mohammad Ali Jahani, Mihajlo Jakovljevic, Farzad Jalilian, Sudha Jayaraman, Achala Upendra Jayatilleke, Ravi Prakash Jha, Yetunde O John-Akinola, Jost B Jonas, Kelly M Jones, Nitin Joseph, Farahnaz Joukar, Jacek Jerzy Jozwiak, Suresh Banayya Jungari, Mikk Jürisson, Ali Kabir, Amaha Kahsay, Leila R Kalankesh, Rohollah Kalhor, Teshome Abegaz Kamil, Tanuj Kanchan, Neeti Kapoor, Manoochehr Karami, Amir Kasaeian, Hagazi Gebremedhin Kassaye, Taras Kavetskyy, Gbenga A Kayode, Peter Njenga Keiyoro, Abraham Getachew Kelbore, Yousef Saleh Khader, Morteza Abdullatif Khafaie, Nauman Khalid, Ibrahim A Khalil, Rovshan Khalilov, Maseer Khan, Ejaz Ahmad Khan, Junaid Khan, Tripti Khanna, Salman Khazaei, Habibolah Khazaie, Roba Khundkar, Daniel N Kiirithio, Young-Eun Kim, Yun Jin Kim, Daniel Kim, Sezer Kisa, Adnan Kisa, Hamidreza Komaki, Shivakumar K M Kondlahalli, Ali Koolivand, Vladimir Andreevich Korshunov, Ai Koyanagi, Moritz U G Kraemer, Kewal Krishan, Barthelemy Kuate Defo, Burcu Kucuk Bicer, Nuworza Kugbey, Nithin Kumar, Manasi Kumar, Vivek Kumar, Narinder Kumar, Girikumar Kumaresh, Faris Hasan Lami, Van C Lansingh, Savita Lasrado, Arman Latifi, Paolo Lauriola, Carlo La Vecchia, Janet L Leasher, Shaun Wen Huey Lee, Shanshan Li, Xuefeng Liu, Alan D Lopez, Paulo A Lotufo, Ronan A Lyons, Daiane Borges Machado, Mohammed Madadin, Muhammed Magdy Abd El Razek, Narayan Bahadur Mahotra, Marek Majdan, Azeem Majeed, Venkatesh Maled, Deborah Carvalho Malta, Navid Manafi, Amir Manafi, Ana-Laura Manda, Narayana Manjunatha, Fariborz Mansour-Ghanaei, Mohammad Ali Mansournia, Joemer C Maravilla, Amanda J Mason-Jones, Seyedeh Zahra Masoumi, Benjamin Ballard Massenburg, Pallab K Maulik, Man Mohan Mehndiratta, Zeleke Aschalew Melketsedik, Peter T N Memiah, Walter Mendoza, Ritesh G Menezes, Melkamu Merid Mengesha, Tuomo J Meretoja, Atte Meretoja, Hayimro Edemealem Merie, Tomislav Mestrovic, Bartosz Miazgowski, Tomasz Miazgowski, Ted R Miller, G K Mini, Andreea Mirica, Erkin M Mirrakhimov, Mehdi Mirzaei-Alavijeh, Prasanna Mithra, Babak Moazen, Masoud Moghadaszadeh, Efat Mohamadi, Yousef Mohammad, Aso Mohammad Darwesh, Abdollah Mohammadian-Hafshejani, Reza Mohammadpourhodki, Shafiu Mohammed, Jemal Abdu Mohammed, Farnam Mohebi, Mohammad A Mohseni Bandpei, Mariam Molokhia, Lorenzo Monasta, Yoshan Moodley, Masoud Moradi, Ghobad Moradi, Maziar Moradi-Lakeh, Rahmatollah Moradzadeh, Lidia Morawska, Ilais Moreno Velásquez, Shane Douglas Morrison, Tilahun Belete Mossie, Atalay Goshu Muluneh, Kamarul Imran Musa, Ghulam Mustafa, Mehdi Naderi, Ahamarshan Jayaraman Nagarajan, Gurudatta Naik, Mukhammad David Naimzada, Farid Najafi, Vinay Nangia, Bruno Ramos Nascimento, Morteza Naserbakht, Vinod Nayak, Javad Nazari, Duduzile Edith Ndwandwe, Ionut Negoi, Josephine W Ngunjiri, Trang Huyen Nguyen, Cuong Tat Nguyen, Diep Ngoc Nguyen, Huong Lan Thi Nguyen, Rajan Nikbakhsh, Dina Nur Anggraini Ningrum, Chukwudi A Nnaji, Richard Ofori-Asenso, Felix Akpojene Ogbo, Onome Bright Oghenetega, In-Hwan Oh, Andrew T Olagunju, Tinuke O Olagunju, Ahmed Omar Bali, Obinna E Onwujekwe, Heather M Orpana, Erika Ota, Nikita Otstavnov, Stanislav S Otstavnov, Mahesh P A, Jagadish Rao Padubidri, Smita Pakhale, Keyvan Pakshir, Songhomitra Panda-Jonas, Eun-Kee Park, Sangram Kishor Patel, Ashish Pathak, Sanghamitra Pati, Kebreab Paulos, Amy E Peden, Veincent Christian Filipino Pepito, Jeevan Pereira, Michael R Phillips, Roman V Polibin, Suzanne Polinder, Farshad Pourmalek, Akram Pourshams, Hossein Poustchi, Swayam Prakash, Dimas Ria Angga Pribadi, Parul Puri, Zahiruddin Quazi Syed, Navid Rabiee, Mohammad Rabiee, Amir Radfar, Anwar Rafay, Ata Rafiee, Alireza Rafiei, Fakher Rahim, Siavash Rahimi, Muhammad Aziz Rahman, Ali Rajabpour-Sanati, Fatemeh Rajati, Ivo Rakovac, Sowmya J Rao, Vahid Rashedi, Prateek Rastogi, Priya Rathi, Salman Rawaf, Lal Rawal, Reza Rawassizadeh, Vishnu Renjith, Serge Resnikoff, Aziz Rezapour, Ana Isabel Ribeiro, Jennifer Rickard, Carlos Miguel Rios González, Leonardo Roever, Luca Ronfani, Gholamreza Roshandel, Basema Saddik, Hamid Safarpour, Mahdi Safdarian, S Mohammad Sajadi, Payman Salamati, Marwa R Rashad Salem, Hosni Salem, Inbal Salz, Abdallah M Samy, Juan Sanabria, Lidia Sanchez Riera, Milena M Santric Milicevic, Abdur Razzaque Sarker, Arash Sarveazad, Brijesh Sathian, Monika Sawhney, Mehdi Sayyah, David C Schwebel, Soraya Seedat, Subramanian Senthilkumaran, Seyedmojtaba Seyedmousavi, Feng Sha, Faramarz Shaahmadi, Saeed Shahabi, Masood Ali Shaikh, Mehran Shams-Beyranvand, Aziz Sheikh, Mika Shigematsu, Jae Il Shin, Rahman Shiri, Soraya Siabani, Inga Dora Sigfusdottir, Jasvinder A Singh, Pankaj Kumar Singh, Dhirendra Narain Sinha, Amin Soheili, Joan B Soriano, Muluken Bekele Sorrie, Ireneous N Soyiri, Mark A Stokes, Mu'awiyyah Babale Sufiyan, Bryan L Sykes, Rafael Tabarés-Seisdedos, Karen M Tabb, Biruk Wogayehu Taddele, Yonatal Mesfin Tefera, Arash Tehrani-Banihashemi, Gebretsadkan Hintsa Tekulu, Ayenew Kassie Tesema Tesema, Berhe Etsay Tesfay, Rekha Thapar, Mariya Vladimirovna Titova, Kenean Getaneh Tlaye, Hamid Reza Tohidinik, Roman Topor-Madry, Khanh Bao Tran, Bach Xuan Tran, Jaya Prasad Tripathy, Alexander C Tsai, Aristidis Tsatsakis, Lorainne Tudor Car, Irfan Ullah, Saif Ullah, Bhaskaran Unnikrishnan, Era Upadhyay, Olalekan A Uthman, Pascual R Valdez, Tommi Juhani Vasankari, Yousef Veisani, Narayanaswamy Venketasubramanian, Francesco S Violante, Vasily Vlassov, Yasir Waheed, Yuan-Pang Wang, Taweewat Wiangkham, Haileab Fekadu Wolde, Dawit Habte Woldeyes, Temesgen Gebeyehu Wondmeneh, Adam Belay Wondmieneh, Ai-Min Wu, Grant M A Wyper, Rajaram Yadav, Ali Yadollahpour, Yuichiro Yano, Sanni Yaya, Vahid Yazdi-Feyzabadi, Pengpeng Ye, Paul Yip, Engida Yisma, Naohiro Yonemoto, Seok-Jun Yoon, Yoosik Youm, Mustafa Z Younis, Zabihollah Yousefi, Chuanhua Yu, Yong Yu, Telma Zahirian Moghadam, Zoubida Zaidi, Sojib Bin Zaman, Mohammad Zamani, Hamed Zandian, Fatemeh Zarei, Zhi-Jiang Zhang, Yunquan Zhang, Arash Ziapour, Sanjay Zodpey, Rakhi Dandona, Samath Dhamminda Dharmaratne, Simon I Hay, Ali H Mokdad, David M Pigott, Robert C Reiner, Theo Vos

**Affiliations:** 1 Institute for Health Metrics and Evaluation, University of Washington, Seattle, WA, USA; 2 Department of Neurology, Cairo University, Cairo, Egypt; 3 Neuroscience Research Center, Isfahan University of Medical Sciences, Isfahan, Iran; 4 Department of Public Health, Ministry of Health, Riyadh, Saudi Arabia; 5 Department of Orthopaedic Surgery, University of Southern California, Los Angeles, CA, USA; 6 Biostatistics and Health Informatics, Madda Walabu University, Bale Robe, Ethiopia; 7 Radiotherapy Center, Addis Ababa University, Addis Ababa, Ethiopia; 8 Department of Public Health, Debre Berhan University, Debre Berhan, Ethiopia; 9 Cardiovascular Medicine Department, Ain Shams University, Abbasia, Egypt; 10 Department of Medicine, University College Hospital, Ibadan, Nigeria; 11 Sport Science Department, University of Extremadura, Badajoz, Spain; 12 Social Behavioral Research Branch, National Institute of Health, Bethesda, MD, USA; 13 Cancer Prevention and Control, Georgetown University, Washington, DC, USA; 14 School of Medicine, Center for Politics, Population and Health Research, National Autonomous University of Mexico, Mexico City, Mexico; 15 Department of Epidemiology and Health Statistics, Southeast University Nanjing, Nanjing, China; 16 Microbiology Department, Hazara University, Mansehra, Pakistan; 17 Department of Epidemiology, Jimma University, Jimma, Ethiopia; 18 James P Grant School of Public Health, BRAC University, Dhaka, Bangladesh; 19 Health Systems and Population Studies Division, International Centre for Diarrhoeal Disease Research, Dhaka, Bangladesh; 20 Higher National School of Veterinary Medicine, Algiers, Algeria; 21 Evidence Based Practice Center, Mayo Clinic Foundation for Medical Education and Research, Rochester, MN, USA; 22 Department of Computer Sciences, Imam Abdulrehman Bin Faisal University, Dammam, Saudi Arabia; 23 Department of Pharmacy, Adigrat University, Adigrat, Ethiopia; 24 Medicine and Health Science, Arba Minch University, Arba Minch, Ethiopia; 25 Midwifery Department, Arba Minch University, Injbara, Ethiopia; 26 Department of Population Health Research, King Abdullah International Medical Research Center, Riyadh, Saudi Arabia; 27 Medical Technical Institute, Erbil Polytechnic University, Erbil, Iraq; 28 Department of Information Systems, College of Economics and Political Science, Sultan Qaboos University, Muscat, Oman; 29 Department of Health Care Management and Economics, Urmia University of Medical Science, Urmia, Iran; 30 Health Management and Economics Research Center, Iran University of Medical Sciences, Tehran, Iran; 31 Health Economics Department, Iran University of Medical Sciences, Tehran, Iran; 32 Department of Health Policy and Management, Kuwait University, Safat, Kuwait; 33 International Centre for Casemix and Clinical Coding, National University of Malaysia, Bandar Tun Razak, Malaysia; 34 Department of Epidemiology, Arak University of Medical Sciences, Arak, Iran; 35 Physiotherapy Department, The University of Jordan, Amman, Jordan; 36 King Saud University, Riyadh, Saudi Arabia; 37 Clinical Practice Guidelines Unit, King Saud University, Riyadh, Saudi Arabia; 38 Alexandria Center for Evidence-Based Clinical Practice Guidelines, Alexandria University, Alexandria, Egypt; 39 Carol Davila University of Medicine and Pharmacy, Bucharest, Romania; 40 Department of Epidemiology and Biostatistics, Health Promotion Research Center, Zahedan, Iran; 41 Department of Health Policy and Administration, University of the Philippines Manila, Manila, Philippines; 42 Department of Applied Social Sciences, Hong Kong Polytechnic University, Hong Kong, China; 43 Department of Parasitology, Mazandaran University of Medical Sciences, Sari, Iran; 44 Department of Microbiology and Immunology, Iranshahr University of Medical Sciences, Iranshahr, Iran; 45 Department of Sociology and Social Work, Kwame Nkrumah University of Science and Technology, Kumasi, Ghana; 46 Center for International Health, Ludwig Maximilians University, Munich, Germany; 47 Social Determinants of Health Research Center, Birjand University of Medical Sciences, Birjand, Iran; 48 Department of Health Promotion and Education, Tehran University of Medical Sciences, Tehran, Iran; 49 School of Health Sciences, Birmingham City University, Birmingham, UK; 50 Regional Centre for the Analysis of Data on Occupational and Work-related Injuries and Diseases, Local Health Unit Tuscany Centre, Florence, Italy; 51 School of Science and Health, Western Sydney University, Sydney, New South Wales, Australia; 52 Oral Health Services, Sydney Local Health District, Sydney, New South Wales, Australia; 53 Plastic Surgery Department, University of Texas, Houston, TX, USA; 54 The Judith Lumley Centre, La Trobe University, Melbourne, Victoria, Australia; 55 General Office for Research and Technological Transfer, Peruvian National Institute of Health, Lima, Peru; 56 School of Public Health, Curtin University, Perth, Western Australia, Australia; 57 Department of Health Policy Planning and Management, University of Health and Allied Sciences, Ho, Ghana; 58 Department of Environmental Health Engineering, Hamadan University of Medical Sciences, Hamadan, Iran; 59 Public Health Risk Sciences Division, Public Health Agency of Canada, Toronto, Ontario, Canada; 60 Department of Nutritional Sciences, University of Toronto, Toronto, Ontario, Canada; 61 Department of Forensic Science, Government Institute of Forensic Science, Nagpur, India; 62 Biochemistry Unit, Universiti Sultan Zainal Abidin, Kuala Terengganu, Malaysia; 63 School of Health Sciences, Univeristi Sultan Zainal Abidin, Kuala Terengganu, Malaysia; 64 Institute of Health Management Research, Indian Institute of Health Management Research University, Jaipur, India; 65 Department of Epidemiology, Johns Hopkins University, Baltimore, MD, USA; 66 Health Policy and Management Department, Tehran University of Medical Sciences, Tehran, Iran; 67 Department of Demography, University of Groningen, Groningen, Netherlands; 68 Population Research Centre, Institute for Social and Economic Change, Bengaluru, India; 69 Department of Hypertension, Medical University of Lodz, Lodz, Poland; 70 Polish Mothers’ Memorial Hospital Research Institute, Lodz, Poland; 71 School of Health Sciences, Walden University, Minneapolis, MN, USA; 72 Department of Noncommunicable Diseases, Bangladesh University of Health Sciences (BUHS), Dhaka, Bangladesh; 73 Department of Research, Public Health Perspective Nepal, Pokhara-Lekhnath Metropolitan City, Nepal; 74 School of Psychology, University of Auckland, Auckland, New Zealand; 75 Heidelberg Institute of Global Health (HIGH), Heidelberg University, Heidelberg, Germany; 76 T.H. Chan School of Public Health, Harvard University, Boston, MA, USA; 77 Occupational Health Department, Kermanshah University of Medical Sciences, Kermanshah, Iran; 78 Health Human Resources Research Center, Shiraz University of Medical Sciences, Shiraz, Iran; 79 Department of Psychiatry, Charles R. Drew University of Medicine and Science, Los Angeles, CA, USA; 80 Department of Psychiatry and Biobehavioral Sciences, David Geffen School of Medicine, University of California Los Angeles, Los Angeles, CA, USA; 81 Department of Community Medicine, Gandhi Medical College Bhopal, Bhopal, India; 82 Jazan University, Jazan, Saudi Arabia; 83 Social Determinants of Health Research Center, Lorestan University of Medical Sciences, Khorramabad, Iran; 84 Psychiatry Department, Bahir Dar University, Bhair Dar, Ethiopia; 85 Nuffield Department of Population Health, University of Oxford, Oxford, UK; 86 Department of Internal Medicine, University of São Paulo, São Paulo, Brazil; 87 Department of Nutrition and Dietetics, Mekelle University, Mekelle, Ethiopia; 88 Department of Internal Medicine, United Arab Emirates University, Al Ain, United Arab Emirates; 89 Social and Clinical Pharmacy, Charles University, Hradec Kralova, Czech Republic; 90 Department of Community Medicine, All India Institute of Medical Sciences, Nagpur, India; 91 Department of Community Medicine, Datta Meghe Institute of Medical Sciences, Wardha, India; 92 Internal Medicine Department, University of Massachusetts Medical School, Springfield, MA, USA; 93 Department of Statistical and Computational Genomics, National Institute of Biomedical Genomics, Kalyani, India; 94 Department of Statistics, University of Calcutta, Kolkata, India; 95 Centre for Global Child Health, University of Toronto, Toronto, Ontario, Canada; 96 Centre of Excellence in Women and Child Health, Aga Khan University, Karachi, Pakistan; 97 Institute of Soil and Environmental Sciences, University of Agriculture, Faisalabad, Pakistan; 98 Social Determinants of Health Research Center, Babol University of Medical Sciences, Babol, Iran; 99 Department of Internal Medicine, Manipal Academy of Higher Education, Mangalore, India; 100 Department of Epidemiology and Psychosocial Reseach, Ramón de la Fuente Muñiz National Institute of Psychiatry, Mexico City, Mexico; 101 Centre for Adolescent Health, Murdoch Childrens Research Institute, Melbourne, Victoria, Australia; 102 School of Population and Global Health, University of Melbourne, Melbourne, Victoria, Australia; 103 Department of Clinical and Experimental Medicine, University of Catania, Catania, Italy; 104 Transport and Road Safety (TARS) Research Department, University of New South Wales, Sydney, New South Wales, Australia; 105 Division of Hematology and Oncology, Georgetown University, Washington DC, USA; 106 Department of Epidemiology and Evidence Based Medicine, I.M. Sechenov First Moscow State Medical University, Moscow, Russia; 107 Department of Health Sciences, University of Leicester, Leicester, UK; 108 Research Department, Golden Community, Kathmandu, Nepal; 109 Centre for Population Health Sciences, Nanyang Technological University, Singapore, Singapore; 110 Global eHealth Unit, Imperial College London, London, UK; 111 Department of Population and Health, Metropolitan Autonomous University, Mexico City, Mexico; 112 Research Unit on Applied Molecular Biosciences (UCIBIO), University of Porto, Porto, Portugal; 113 Department of Psychiatry, University of São Paulo, São Paulo, Brazil; 114 Colombian National Health Observatory, National Institute of Health, Bogota, Colombia; 115 Epidemiology and Public Health Evaluation Group, National University of Colombia, Bogota, Colombia; 116 Primary Care Services Area, Central Health Directorate, Region Friuli Venezia Giulia, Trieste, Italy; 117 Department of Medicine (DAME), University of Udine, Udine, Italy; 118 National School of Public Health, Carlos III Health Institute, Madrid, Spain; 119 Clinical Epidemiology Program, Ottawa Hospital Research Institute, Ottawa, Ontario, Canada; 120 Mary MacKillop Institute for Health Research, Australian Catholic University, Melbourne, Victoria, Australia; 121 School of Public Health, University of Hong Kong, Hong Kong, China; 122 Institute of Applied Health Research, University of Birmingham, Birmingham, UK; 123 Swedish Neuroscience Institute, Swedish Brain and Spine Specialists, Seattle, WA, USA; 124 Department of Medicine, University of Toronto, Toronto, Ontario, Canada; 125 Department of Public Health, Texila American University, Georgetown, Guyana; 126 2nd Department of Ophthalmology, University of Athens, Haidari, Greece; 127 Ophthalmology Independent Consultant, Athens, Greece; 128 Pediatrics Department, Harvard University, Boston, MA, USA; 129 Neonatology Department, Beth Israel Deaconess Medical Center, Boston, MA, USA; 130 Department of Surgery, Division of Plastic and Reconstructive Surgery, University of Washington, Seattle, WA, USA; 131 Department of Biochemistry and Biomedical Science, Seoul National University Hospital, Seoul, South Korea; 132 Maternal and Child Health Division, International Centre for Diarrhoeal Disease Research, Dhaka, Bangladesh; 133 Department of Epidemiology and Biostatistics, University of South Carolina, Columbia, SC, USA; 134 Department of Pulmonary Medicine, Christian Medical College and Hospital (CMC), Vellore, India; 135 Faculty of Biology, Hanoi National University of Education, Hanoi, Vietnam; 136 School of Public Health and Preventive Medicine, Monash University, Melbourne, Victoria, Australia; 137 Centro Hospitalar Universitário do Porto - Serviço de Oftalmologia, University of Porto, Porto, Portugal; 138 Department of Environmental Health, Imam Abdulrahman Bin Faisal University, Dammam, Saudi Arabia; 139 Toxoplasmosis Research Center, Mazandaran University of Medical Sciences, Sari, Iran; 140 Population and Development, Facultad Latinoamericana de Ciencias Sociales Mexico, Mexico City, Mexico; 141 Australian Institute for Suicide Research and Prevention, Griffith University, Mount Gravatt, Queensland, Australia; 142 Department of Medical Laboratory Sciences, Bahir Dar University, Bahir Dar, Ethiopia; 143 School of Pharmacy, Aksum University, Aksum, Ethiopia; 144 Addis Ababa University, Addis Ababa, Ethiopia; 145 Department of Global Health and Infection, Brighton and Sussex Medical School, Brighton, UK; 146 School of Public Health, Addis Ababa University, Addis Ababa, Ethiopia; 147 Division of Cardiology, Atlanta Veterans Affairs Medical Center, Decatur, GA, USA; 148 Department of Epidemiology, Shiraz University of Medical Sciences, Shiraz, Iran; 149 Faculty of Pharmacy, University of Porto, Porto, Portugal; 150 Tehran University of Medical Sciences, Tehran, Iran; 151 Center of Excellence in Public Health Nutrition, Nguyen Tat Thanh University, Ho Chi Minh City, Vietnam; 152 School of Health and Biomedical Sciences, Royal Melbourne Institute of Technology University, Bundoora, Victoria, Australia; 153 Sydney School of Public Health, University of Sydney, Sydney, New South Wales, Australia; 154 Faculty of Medicine, University of Belgrade, Belgrade, Serbia; 155 Public Health Department, Hawassa University, Hawassa, Ethiopia; 156 Curtin University, Perth, Western Australia, Australia; 157 Department of Global Health and Social Medicine, Harvard University, Boston, MA, USA; 158 Department of Social Services, Tufts Medical Center, Boston, MA, USA; 159 Department of Statistics, Debre Markos University, Debre Markos, Ethiopia; 160 Department of Public Health Sciences, Karolinska Institutet, Stockholm, Sweden; 161 World Health Programme, Université du Québec en Abitibi-Témiscamingue, Rouyn-Noranda, Quebec, Canada; 162 Department of Pathology, Stavanger University Hospital, Stavanger, Norway; 163 Norwegian Institute of Public Health, Oslo, Norway; 164 Department of Clinical Pathology, Mansoura University, Mansoura, Egypt; 165 Multiple Sclerosis Research Center, Tehran University of Medical Sciences, Tehran, Iran; 166 Epidemiology and Population Health, York University, Vancouver, British Columbia, Canada; 167 Faculty of Health Sciences, Simon Fraser University, Burnaby, British Columbia, Canada; 168 Biology Department, Salahaddin University-Erbil, Erbil, Iraq; 169 Department of Biology and Biotechnology “Lazzaro Spallanzani", University of Pavia, Pavia, Italy; 170 Department of Psychology, Federal University of Sergipe, Sao Cristovao, Brazil; 171 Non-communicable Diseases Research Center, Tehran University of Medical Sciences, Tehran, Iran; 172 Department of Neurobiology, Karolinska Institutet, Stockholm, Sweden; 173 Division of Neurology, University of Ottawa, Ottawa, Ontario, Canada; 174 REQUIMTE/LAQV, University of Porto, Porto, Portugal; 175 Research Centre on Public Health (CESP), University of Milan Bicocca, Monza, Italy; 176 Department of Population Medicine and Health Services Research, Bielefeld University, Bielefeld, Germany; 177 Department of Child Dental Health, Obafemi Awolowo University, Ile-Ife, Nigeria; 178 Timiryazev Institute of Plant Physiology, Russian Academy of Sciences, Moscow, Russia; 179 Abadan School of Medical Sciences, Abadan University of Medical Sciences, Abadan, Iran; 180 Department of Family Medicine and Primary Care, University of the Witwatersrand, Johannesburg, South Africa; 181 College of Public Health, Medical and Veterinary Science, James Cook University, Douglas, Queensland, Australia; 182 Royal Life Saving Society, Sydney, New South Wales, Australia; 183 Department of Dermatology, Kobe University, Kobe, Japan; 184 Gene Expression & Regulation Program, The Wistar Institute, Philadelphia, PA, USA; 185 Public Health Department, Madda Walabu University, Bale Robe, Ethiopia; 186 Department of Nursing and Midwifery, Addis Ababa University, Addis Ababa, Ethiopia; 187 Pharmacy Department, Mekelle University, Mekelle, Ethiopia; 188 Department of Nursing, Aksum University, Aksum, Ethiopia; 189 Department of Nursing, Mekelle University, Mekelle, Ethiopia; 190 Public Health, Haramaya University, Harar, Ethiopia; 191 Bahir Dar University, Bahir Dar, Ethiopia; 192 Haramaya University, Dire Dawa, Ethiopia; 193 Department of Pharmacy, Wollo University, Dessie, Ethiopia; 194 Department of Nursing, Arba Minch University, Arba Minch, Ethiopia; 195 Department of Medical Surgery, Tabriz University of Medical Sciences, Tabriz, Iran; 196 Occupational Health Department, Arak University of Medical Sciences, Arak, Iran; 197 Department of Nursing and Midwifery, Kurdistan University of Medical Sciences, Sanandaj, Iran; 198 Science and Research Branch, Islamic Azad University, Tehran, Iran; 199 Young Researchers and Elite Club, Islamic Azad University, Rasht, Iran; 200 Faculty of Allied Health Sciences, The University of Lahore, Lahore, Pakistan; 201 Chairman BOG, Afro-Asian Institute, Lahore, Pakistan; 202 Adelaide Medical School, University of Adelaide, Adelaide, SA, Australia; 203 Nursing and Midwifery Department, Mazandaran University of Medical Sciences, Sari, Iran; 204 Center for Clinical and Epidemiological Research, University of São Paulo, Sao Paulo, Brazil; 205 Internal Medicine Department, University of São Paulo, Sao Paulo, Brazil; 206 Department of Dermatology, Boston University, Boston, MA, USA; 207 Institute of Public Health, United Arab Emirates University, Al Ain, United Arab Emirates; 208 Instituto de Patologia Tropical e Saúde Pública, Federal University of Goias, Goiânia, Brazil; 209 Department of Epidemiology and Biostatistics, Zhengzhou University, Zhengzhou, China; 210 Non-Communicable Diseases (NCD), World Health Organization (WHO), New Delhi, India; 211 Department of Public Health, Erasmus University Medical Center, Rotterdam, Netherlands; 212 Global and Community Mental Health Research Group, University of Macau, Macao, China; 213 Department of Family and Community Medicine, Arabian Gulf University, Manama, Bahrain; 214 School of Health and Environmental Studies, Hamdan Bin Mohammed Smart University, Dubai, United Arab Emirates; 215 Biomedical Research Networking Center for Mental Health Network (CiberSAM), Madrid, Spain; 216 Research and Development Unit, San Juan de Dios Sanitary Park, Sant Boi de Llobregat, Spain; 217 Department of Microbiology, Maragheh University of Medical Sciences, Maragheh, Iran; 218 Department of Microbiology, Tehran University of Medical Sciences, Tehran, Iran; 219 Centre for International Health and Section for Ethics and Health Economics, University of Bergen, Bergen, Norway; 220 Gastrointestinal and Liver Disease Research Center, Guilan University of Medical Sciences, Rasht, Iran; 221 Guilan University of Medical Sciences, Rasht, Iran; 222 School of Nursing and Midwifery, Tabriz University of Medical Sciences, Tabriz, Iran; 223 Independent Consultant, Tabriz, Iran; 224 Department of Public Health, Mizan-Tepi University, Tepi, Ethiopia; 225 Unit of Epidemiology and Social Medicine, University Hospital Antwerp, Wilrijk, Belgium; 226 Department of Clinical Sciences, Karolinska University Hospital, Stockholm, Sweden; 227 Medical Biology Research Center, Kermanshah University of Medical Sciences, Kermanshah, Iran; 228 Research Coordination, AC Environments Foundation, Cuernavaca, Mexico; 229 CISS, National Institute of Public Health, Cuernavaca. Mexico; 230 Department of Urban Planning and Design, University of Hong Kong, Hong Kong, China; 231 Center of Excellence in Behavioral Medicine, Nguyen Tat Thanh University, Ho Chi Minh City, Vietnam; 232 Department of Pediatrics, Dell Medical School, University of Texas Austin, Austin, TX, USA; 233 Kasturba Medical College, Manipal Academy of Higher Education, Manipal, India; 234 Department of Pharmacology and Therapeutics, Dhaka Medical College, Dhaka, Bangladesh; 235 Department of Pharmacology, Bangladesh Industrial Gases Limited, Tangail, Bangladesh; 236 Department of Computer Engineering, Islamic Azad University, Tehran, Iran; 237 Computer Science Department, University of Human Development, Sulaymaniyah, Iraq; 238 Department of Legal Medicine and Bioethics, Carol Davila University of Medicine and Pharmacy, Bucharest, Romania; 239 Clinical Legal Medicine Department, National Institute of Legal Medicine Mina Minovici, Bucharest, Romania; 240 Department of Epidemiology and Health Statistics, Central South University, Changsha, China; 241 Department of Health Promotion and Education, University of Ibadan, Ibadan, Nigeria; 242 Department of Community Medicine, University of Ibadan, Ibadan, Nigeria; 243 Department of Family Medicine, Bangalore Baptist Hospital, Bangalore, India; 244 Research Institute for Endocrine Sciences, Shahid Beheshti University of Medical Sciences, Tehran, Iran; 245 School of Psychology and Public Health, La Trobe University, Bundoora, Melbourne, Victoria, Australia; 246 Institute for Physical Activity and Nutrition, Deakin University, Burwood, Victoria, Australia; 247 Sydney Medical School, University of Sydney, Sydney, New South Wales, Australia; 248 School of Public Health and Community Medicine, University of New South Wales, Sydney, New South Wales, Australia; 249 Faculty of Medicine, Babol University of Medical Sciences, Babol, Iran; 250 Department for Health Care and Public Health, Sechenov First Moscow State Medical University, Moscow, Russia; 251 Social Development & Health Promotion Research Center, Kermanshah University of Medical Sciences, Kermanshah, Iran; 252 Department of Surgery, Virginia Commonwealth University, Richmond, VA, USA; 253 Institute of Medicine, University of Colombo, Colombo, Sri Lanka; 254 Faculty of Graduate Studies, University of Colombo, Colombo, Sri Lanka; 255 Department of Community Medicine, Banaras Hindu University, Varanasi, India; 256 Health Promotion and Education, University of Ibadan, Ibadan, Nigeria; 257 Department of Ophthalmology, Heidelberg University, Mannheim, Germany; 258 Beijing Ophthalmology & Visual Science Key Laboratory, Beijing Tongren Hospital, Beijing, China; 259 Auckland University of Technology, Auckland, New Zealand; 260 Community Medicine Department, Manipal Academy of Higher Education, Mangalore, India; 261 Department of Family Medicine and Public Health, University of Opole, Opole, Poland; 262 School of Health Sciences, Savitribai Phule Pune University, Pune, India; 263 Institute of Family Medicine and Public Health, University of Tartu, Tartu, Estonia; 264 Minimally Invasive Surgery Research Center, Iran University of Medical Sciences, Tehran, Iran; 265 Department of Medical Informatics, Tabriz University of Medical Sciences, Tabriz, Iran; 266 Social Determinants of Health Research Center, Research Institute for Prevention of Non-Communicable Diseases, Qazvin University of Medical Sciences, Qazvin, Iran; 267 Health Services Management Department, Qazvin University of Medical Sciences, Qazvin, Iran; 268 School of Public Health, Department of Health informatics and Health Innovation, A.C.S. Medical College and Hospital, Mekelle, Ethiopia; 269 Department of Forensic Medicine and Toxicology, All India Institute of Medical Sciences, Jodhpur, India; 270 Department of Epidemiology, Hamadan University of Medical Sciences, Hamadan, Iran; 271 Hematology-Oncology and Stem Cell Transplantation Research Center, Tehran University of Medical Sciences, Tehran, Iran; 272 Pars Advanced and Minimally Invasive Medical Manners Research Center, Iran University of Medical Sciences, Tehran, Iran; 273 Department of Applied Physics, The John Paul II Catholic University of Lublin, Lublin Voivodeship, Poland; 274 Department of Biology and Chemistry, Drohobych Ivan Franko State Pedagogical University, Drohobych, Ukraine; 275 International Research Center of Excellence, Institute of Human Virology Nigeria, Abuja, Nigeria; 276 Julius Centre for Health Sciences and Primary Care, Utrecht University, Utrecht, Netherlands; 277 Open, Distance and eLearning Campus, University of Nairobi, Nairobi, Kenya; 278 Department of Dermatology, Wolaita Sodo University, Wolaita Sodo, Ethiopia; 279 Department of Public Health, Jordan University of Science and Technology, Irbid, Jordan; 280 Social Determinants of Health Research Center, Ahvaz Jundishapur University of Medical Sciences, Ahvaz, Iran; 281 School of Food and Agricultural Sciences, University of Management and Technology, Lahore, Pakistan; 282 Department of Global Health, University of Washington, Seattle, WA, USA; 283 Department of Physiology, Baku State University, Baku, Azerbaijan; 284 Epidemiology, Faculty of Public Health and Tropical Medicine, Jazan University, Jazan, Saudi Arabia; 285 Epidemiology and Biostatistics Department, Health Services Academy, Islamabad, Pakistan; 286 Department of Population Studies, International Institute for Population Sciences, Mumbai, India; 287 Department of Health Research, Indian Council of Medical Research, New Delhi, India; 288 Centre for Ethics, Jawahar Lal Nehru University, New Delhi, India; 289 Department of Psychiatry, Kermanshah University of Medical Sciences, Kermanshah, Iran; 290 Nuffield Department of Surgical Sciences, Oxford University Global Surgery Group, University of Oxford, Oxford, UK; 291 Research and Data Solutions, Synotech Consultant, Nairobi, Kenya; 292 Department of Preventive Medicine, Korea University, Seoul, South Korea; 293 School of Medicine, Xiamen University Malaysia, Sepang, Malaysia; 294 Department of Health Sciences, Northeastern University, Boston, MA, USA; 295 Department of Nursing and Health Promotion, Oslo Metropolitan University, Oslo, Norway; 296 School of Health Sciences, Kristiania University College, Oslo, Norway; 297 Neurophysiology Research Center, Hamadan University of Medical Sciences, Hamadan, Iran; 298 Brain Engineering Research Center, Institute for Research in Fundamental Sciences, Tehran, Iran; 299 Public Health Dentistry Department, Krishna Institute of Medical Sciences Deemed to be University, Karad, India; 300 Environmental Health Engineering, Arak University of Medical Sciences, Arak, Iran; 301 CIBERSAM, San Juan de Dios Sanitary Park, Sant Boi de Llobregat, Spain; 302 Catalan Institution for Research and Advanced Studies (ICREA), Barcelona, Spain; 303 Department of Zoology, University of Oxford, Oxford, UK; 304 Harvard Medical School, Harvard University, Boston, MA, USA; 305 Department of Anthropology, Panjab University, Chandigarh, India; 306 Department of Demography, University of Montreal, Montreal, Quebec, Canada; 307 Department of Social and Preventive Medicine, University of Montreal, Montreal, Quebec, Canada; 308 Department of Public Health, Yuksek Ihtisas University, Ankara, Turkey; 309 Department of Public Health, Hacettepe University, Ankara, Turkey; 310 Department of Family and Community Health, University of Health and Allied Sciences, Ho, Ghana; 311 Department of Psychology and Health Promotion, University of KwaZulu-Natal, Durban, South Africa; 312 Community Medicine Department, Kasturba Medical College, Manipal Academy of Higher Education, Mangalore, India; 313 Department of Psychiatry, University of Nairobi, Nairobi, Kenya; 314 Division of Psychology and Language Sciences, University College London, London, UK; 315 Department of Medicine Brigham and Women’s Hospital, Harvard University, Boston, MA, USA; 316 Orthopaedics Department, Base Hospital Lucknow Cantt, Lucknow, India; 317 Mechanical and Industrial Engineering, Indian Institute of Technology, Roorkee, India; 318 Department of Community and Family Medicine, University of Baghdad, Baghdad, Iraq; 319 HelpMeSee, New York, NY, USA; 320 International Relations, Mexican Institute of Ophthalmology, Queretaro, Mexico; 321 Department of Otorhinolaryngology (ENT), Father Muller Medical College, Mangalore, India; 322 Department of Public Health, Maragheh University of Medical Sciences, Maragheh, Iran; 323 Institute of Clinical Physiology, National Research Council, Pisa, Italy; 324 Clinical Medicine and Community Health, University of Milan, Milano, Italy; 325 College of Optometry, Nova Southeastern University, Fort Lauderdale, FL, USA; 326 School of Pharmacy, Monash University, Bandar Sunway, Malaysia; 327 School of Pharmacy, Taylor’s University Lakeside Campus, Subang Jaya, Malaysia; 328 Department of Systems, Populations and Leadership, University of Michigan, Ann Arbor, MI, USA; 329 Department of Health Metrics Sciences, School of Medicine, University of Washington, Seattle, WA, USA; 330 Department of Medicine, University of São Paulo, Sao Paulo, Brazil; 331 Health Data Research UK, Swansea University, Swansea, UK; 332 Center for Integration of Data and Health Knowledge, FIOCRUZ: Cidacs Center for Integration of Data and Health Knowledge, Salvador, Brazil; 333 Faculty of Epidemiology and Population Health, London School of Hygiene & Tropical Medicine, England; 334 Pathology Department, College of Medicine, Imam Abdulrahman Bin Faisal University, Dammam, Saudi Arabia; 335 Ophthalmology Department, Aswan Faculty of Medicine, Aswan, Egypt; 336 Institute of Medicine, Tribhuvan University, Kathmandu, Nepal; 337 Department of Public Health, Trnava University, Trnava, Slovakia; 338 Department of Primary Care and Public Health, Imperial College London, London, UK; 339 Health Education and Research Department, SDM College of Medical Sciences & Hospital, Dharwad, India; 340 Health University, Rajiv Gandhi University of Health Sciences, Bangalore, India; 341 Department of Maternal and Child Nursing and Public Health, Federal University of Minas Gerais, Belo Horizonte, Brazil; 342 Ophthalmology Department, Iran University of Medical Sciences, Tehran, Iran; 343 Ophthalmology Department, University of Manitoba, Winnipeg, Manitoba, Canada; 344 Department of Surgery, University of Virginia, Charlottesville, VA, USA; 345 Surgery Department, Emergency University Hospital Bucharest, Bucharest, Romania; 346 Psychiatry Department, National Institute of Mental Health and Neurosciences, Bengaluru, India; 347 Department of Epidemiology and Biostatistics, Tehran University of Medical Sciences, Tehran, Iran; 348 Institute for Social Science Research, The University of Queensland, Brisbane, Queensland, Australia; 349 Department of Health Sciences, University of York, York, UK; 350 Department of Midwifery-Reproductive Health, Hamadan University of Medical Sciences, Hamadan, Iran; 351 Research Department, The George Institute for Global Health, New Delhi, India; 352 School of Medicine, University of New South Wales, Sydney, New South Wales, Australia; 353 Neurology Department, Janakpuri Super Specialty Hospital Society, New Delhi, India; 354 Department of Neurology, Govind Ballabh Institute of Medical Education and Research, New Delhi, India; 355 Division of Epidemiology and Prevention, Institute of Human Virology, University of Maryland, Baltimore, MD, USA; 356 Peru Country Office, United Nations Population Fund (UNFPA), Lima, Peru; 357 Forensic Medicine Division, Imam Abdulrahman Bin Faisal University, Dammam, Saudi Arabia; 358 Department of Epidemiology and Biostatistics, Haramaya University, Harar, Ethiopia; 359 Breast Surgery Unit, Helsinki University Hospital, Helsinki, Finland; 360 University of Helsinki, Helsinki, Finland; 361 Neurocenter, Helsinki University Hospital, Helsinki, Finland; 362 School of Health Sciences, University of Melbourne, Parkville, Victoria, Australia; 363 Statistics Department, Debre Markos University, Debre Markos, Ethiopia; 364 Clinical Microbiology and Parasitology Unit, Zora Profozic Polyclinic, Zagreb, Croatia; 365 University Centre Varazdin, University North, Varazdin, Croatia; 366 Center for Innovation in Medical Education, Pomeranian Medical University, Szczecin, Poland; 367 Pomeranian Medical University, Szczecin, Poland; 368 Department of Propedeutics of Internal Diseases & Arterial Hypertension, Pomeranian Medical University, Szczecin, Poland; 369 Pacific Institute for Research & Evaluation, Calverton, MD, USA; 370 Achutha Menon Centre for Health Science Studies, Sree Chitra Tirunal Institute for Medical Sciences and Technology, Trivandrum, India; 371 Global Institute of Public Health (GIPH), Ananthapuri Hospitals and Research Centre, Trivandrum, India; 372 Department of Statistics and Econometrics, Bucharest University of Economic Studies, Bucharest, Romania; 373 President’s Office, National Institute of Statistics, Bucharest, Romania; 374 Faculty of Internal Medicine, Kyrgyz State Medical Academy, Bishkek, Kyrgyzstan; 375 Department of Atherosclerosis and Coronary Heart Disease, National Center of Cardiology and Internal Disease, Bishkek, Kyrgyzstan; 376 Heidelberg Institute of Global Health (HIGH), Faculty of Medicine and University Hospital, Heidelberg University, Heidelberg, Germany; 377 Institute of Addiction Research (ISFF), Frankfurt University of Applied Sciences, Frankfurt, Germany; 378 Biotechnology Research Center, Tabriz University of Medical Sciences, Tabriz, Iran; 379 Molecular Medicine Research Center, Tabriz University of Medical Sciences, Tabriz, Iran; 380 Health Equity Research Center, Tehran University of Medical Sciences, Tehran, Iran; 381 Internal Medicine Department, King Saud University, Riyadh, Saudi Arabia; 382 Department of Information Technology, University of Human Development, Sulaymaniyah, Iraq; 383 Department of Epidemiology and Biostatistics, Shahrekord University of Medical Sciences Shahrekord, Iran; 384 Department of Nursing, Shahroud University of Medical Sciences, Shahroud, Iran; 385 Health Systems and Policy Research Unit, Ahmadu Bello University, Zaria, Nigeria; 386 Department of Public Health, Samara University, Samara, Ethiopia; 387 Iran National Institute of Health Research, Tehran University of Medical Sciences, Tehran, Iran; 388 Pediatric Neurorehabilitation Research Center, University of Social Welfare and Rehabilitation Sciences, Tehran, Iran; 389 Faculty of Life Sciences and Medicine, King’s College London, London, UK; 390 Clinical Epidemiology and Public Health Research Unit, Burlo Garofolo Institute for Maternal and Child Health, Trieste, Italy; 391 Department of Public Health Medicine, University of KwaZulu-Natal, Durban, South Africa; 392 Research Center for Environmental Determinants of Health, Kermanshah University of Medical Sciences, Kermanshah, Iran; 393 Kermanshah University of Medical Sciences, Kermanshah, Iran; 394 Social Determinants of Health Research Center, Kurdistan University of Medical Sciences, Sanandaj, Iran; 395 Department of Epidemiology and Biostatistics, Kurdistan University of Medical Sciences, Sanandaj, Iran; 396 Preventive Medicine and Public Health Research Center, Iran University of Medical Sciences, Tehran, Iran; 397 International Laboratory for Air Quality and Health, Queensland University of Technology, Brisbane, Queensland, Australia; 398 Gorgas Memorial Institute for Health Studies, Panama City, Panama; 399 Department of Psychiatry, Badhir Dar University, Ethiopia; 400 Department of Epidemiology and Biostatistics, University of Gondar, Gondar, Ethiopia; 401 School of Medical Sciences, Science University of Malaysia, Kubang Kerian, Malaysia; 402 Department of Pediatric Medicine, Nishtar Medical University, Multan, Pakistan; 403 Department of Pediatrics & Pediatric Pulmonology, Institute of Mother & Child Care, Multan, Pakistan; 404 Clinical Research Development Center, Kermanshah University of Medical Sciences, Kermanshah, Iran; 405 Research and Analytics, Initiative for Financing Health and Human Development, Chennai, India; 406 Research and Analytics, Bioinsilico Technologies, Chennai, India; 407 Department of Epidemiology, University of Alabama at Birmingham, Birmingham, AL, USA; 408 Laboratory of Public Health Indicators Analysis and Health Digitalization, Moscow Institute of Physics and Technology, Dolgoprudny, Russia; 409 Experimental Surgery and Oncology Laboratory, Kursk State Medical University of the Ministry of Health of the Russian Federation, Kursk, Russia; 410 Department of Epidemiology & Biostatistics, Kermanshah University of Medical Sciences, Kermanshah, Iran; 411 Suraj Eye Institute, Nagpur, India; 412 Hospital of the Federal University of Minas Gerais, Federal University of Minas Gerais, Belo Horizonte, Brazil; 413 Mental Health Research Center, IUMS, Tehran, Iran; 414 Preventive Medicine and Public Health Research Center, IUMS, Tehran, Iran; 415 Department of Forensic Medicine and Toxicology, Manipal Academy of Higher Education, Manipal, India; 416 Department of Pediatrics, Arak University of Medical Sciences, Arak, Iran; 417 Iranian Ministry of Health and Medical Education, Tehran, Iran; 418 Cochrane South Africa, South African Medical Research Council, Cape Town, South Africa; 419 Department of General Surgery, Carol Davila University of Medicine and Pharmacy, Bucharest, Romania; 420 Department of General Surgery, Emergency Hospital of Bucharest, Bucharest, Romania; 421 Department of Biological Sciences, University of Embu, Embu, Kenya; 422 Institute for Global Health Innovations, Duy Tan University, Hanoi, Vietnam; 423 Project of ADB, National Institute of Nutrition, Hanoi, Vietnam; 424 Industrial Management Department, Hanoi University of Science and Technology, Hanoi, Vietnam; 425 Department of Pharmacology, Shahid Beheshti University of Medical Sciences, Tehran, Iran; 426 Heidelberg University Hospital, Heidelberg, Germany; 427 Public Health Department, Universitas Negeri Semarang, Kota Semarang, Indonesia; 428 Graduate Institute of Biomedical Informatics, Taipei Medical University, Taipei City, Taiwan; 429 School of Public Health and Family Medicine, University of Cape Town, Cape Town, South Africa; 430 Centre of Cardiovascular Research and Education in Therapeutics, Monash University, Melbourne, Victoria, Australia; 431 Independent Consultant, Accra, Ghana; 432 UCIBIO, University of Porto, Porto, Portugal; 433 Reproductive Health Sciences, Department Obstetrics and Gynecology, University of Ibadan, Ibadan, Nigeria; 434 Department of Preventive Medicine, Kyung Hee University, Dongdaemun-gu, South Korea; 435 Department of Psychiatry and Behavioural Neurosciences, McMaster University, Hamilton, Ontario, Canada; 436 Department of Psychiatry, University of Lagos, Lagos, Nigeria; 437 Department of Pathology and Molecular Medicine, McMaster University, Hamilton, Ontario, Canada; 438 Diplomacy and Public Relations Department, University of Human Development, Sulaimaniyah, Iraq; 439 Department of Pharmacology and Therapeutics, University of Nigeria Nsukka, Enugu, Nigeria; 440 Applied Research Division, Public Health Agency of Canada, Ottawa, Ontario, Canada; 441 School of Psychology, University of Ottawa, Ottawa, Ontario, Canada; 442 Department of Global Health Nursing, St. Luke’s International University, Chuo-ku, Japan; 443 Academic Department, Unium Ltd, Moscow, Russia; 444 Department of Project Management, National Research University Higher School of Economics, Moscow, Russia; 445 Department of Respiratory Medicine, Jagadguru Sri Shivarathreeswara Academy of Health Education and Research, Mysore, India; 446 Department of Forensic Medicine, Manipal Academy of Higher Education, Manipal, India; 447 Department of Medicine, Ottawa Hospital Research Institute, Ottawa Hospital, Ottawa, Ontario, Canada; 448 Parasitology and Mycology Department, Shiraz University of Medical Sciences, Shiraz, Iran; 449 Augenpraxis Jonas, Heidelberg University, Heidelberg, Germany; 450 Department of Medical Humanities and Social Medicine, Kosin University, Busan, South Korea; 451 Research and Evaluation Department, Population Council, New Delhi, India; 452 Indian Institute of Health Management Research University, Jaipur, India; 453 Department of Pediatircs, RD Gardi Medical College, Ujjain, India; 454 Public Health Sciences, Karolinska Institutet, Stockholm, Sweden; 455 Regional Medical Research Centre, Indian Council of Medical Research, Bhubaneswar, India; 456 Department of Midwifery, Wolaita Sodo University, Wolaita Sodo, Ethiopia; 457 School of Public Health and Community Medicine, Faculty of Medicine, University of New South Wales, Sydney, New South Wales, Australia; 458 Center for Research and Innovation, Ateneo De Manila University, Pasig City, Philippines; 459 Department of Orthopedics, Yenepoya Medical College, Mangalore, India; 460 Department of Psychiatry, Department of Epidemiology, Columbia University, New York, NY, USA; 461 Shanghai Mental Health Center, Shanghai Jiao Tong University, Shanghai, China; 462 Department of Epidemiology and Evidence-Based Medicine, Sechenon University, Moscow, Russia; 463 School of Population and Public Health, University of British Columbia, Vancouver, British Columbia, Canada; 464 Digestive Diseases Research Institute, Tehran University of Medical Sciences, Tehran, Iran; 465 Department of Nephrology, Sanjay Gandhi Postgraduate Institute of Medical Sciences, Lucknow, India; 466 Health Sciences Department, Muhammadiyah University of Surakarta, Sukoharjo, Indonesia; 467 Department of Chemistry, Sharif University of Technology, Tehran, Iran; 468 Biomedical Engineering Department, Amirkabir University of Technology, Tehran, Iran; 469 College of Medicine, University of Central Florida, Orlando, FL, USA; 470 College of Graduate Health Sciences, A.T. Still University, Mesa, AZ, USA; 471 Department of Epidemiology & Biostatistics, Contech School of Public Health, Lahore, Pakistan; 472 Department of Medicine, University of Alberta, Edmonton, Alberta, Canada; 473 Department of Immunology, Mazandaran University of Medical Sciences, Sari, Iran; 474 Molecular and Cell Biology Research Center, Mazandaran University of Medical Sciences, Sari, Iran; 475 Thalassemia and Hemoglobinopathy Research Center, Ahvaz Jundishapur University of Medical Sciences, Ahvaz, Iran; 476 Metabolomics and Genomics Research Center, Tehran University of Medical Sciences, Tehran, Iran; 477 Faculty of Medicine, Mazandaran University of Medical Sciences, Sari, Iran; 478 School of Nursing and Healthcare Professions, Federation University Australia, Berwick, Victoria, Australia; 479 School of Nursing and Midwifery, La Trobe University, Melbourne, Victoria, Australia; 480 Faculty of Medicine, Birjand University of Medical Sciences, Birjand, Iran; 481 European Office for the Prevention and Control of Noncommunicable Diseases, World Health Organization (WHO), Moscow, Russia; 482 Department of Oral Pathology, Srinivas Institute of Dental Sciences, Mangalore, India; 483 School of Behavioral Sciences and Mental Health, Tehran Institute of Psychiatry, Tehran, Iran; 484 Academic Public Health Department, Public Health England, London, UK; 485 School of Health, Medical and Applied Sciences, CQ University, Sydney, New South Wales, Australia; 486 Department of Computer Science, Metropolitan College, Boston University, Boston, USA; 487 Neurology Department, Sree Chitra Tirunal Institute for Medical Sciences and Technology, Thiruvananthapuram, India; 488 Brien Holden Vision Institute, Sydney, New South Wales, Australia; 489 Organization for the Prevention of Blindness, Paris, France; 490 EPIUnit - Public Health Institute University Porto (ISPUP), University of Porto, Porto, Portugal; 491 Surgery Department, University of Minnesota, Minneapolis, MN, USA; 492 Surgery Department, University Teaching Hospital of Kigali, Kigali, Rwanda; 493 Research Directorate, Nihon Gakko University, Fernando de la Mora, Paraguay; 494 Research Direction, Universidad Nacional de Caaguazú, Coronel Oviedo, Paraguay; 495 Department of Clinical Research, Federal University of Uberlândia, Uberlândia, Brazil; 496 Golestan Research Center of Gastroenterology and Hepatology, Golestan University of Medical Sciences, Gorgan, Iran; 497 College of Medicine, University of Sharjah, Sharjah, United Arab Emirates; 498 Department of Health in Disasters and Emergencies, Shahid Beheshti University of Medical Sciences, Tehran, Iran; 499 Department of Neuroscience, Iran University of Medical Sciences, Tehran, Iran; 500 Sina Trauma and Surgery Research Center, Tehran University of Medical Sciences, Tehran, Iran; 501 Nanobiotechnology Center, Soran University, Soran, Iraq; 502 Public Health and Community Medicine Department, Cairo University, Giza, Egypt; 503 Urology Department, Cairo University, Giza, Egypt; 504 Health and Disability Intelligence Group, Ministry of Health, Wellington, New Zealand; 505 Department of Entomology, Ain Shams University, Cairo, Egypt; 506 Department of Surgery, Marshall University, Huntington, WV, USA; 507 Department of Nutrition and Preventive Medicine, Case Western Reserve University, Cleveland, OH, USA; 508 Rheumatology Department, University Hospitals Bristol NHS Foundation Trust, Bristol, UK; 509 Institute of Bone and Joint Research, University of Sydney, Syndey, New South Wales, Australia; 510 Institute of Social Medicine, University of Belgrade, Belgrade, Serbia; 511 Centre-School of Public Health and Health Management, University of Belgrade, Belgrade, Serbia; 512 Health Economics, Bangladesh Institute of Development Studies (BIDS), Dhaka, Bangladesh; 513 Colorectal Research Center, Iran University of Medical Sciences, Tehran, Iran; 514 Surgery Department, Hamad Medical Corporation, Doha, Qatar; 515 Faculty of Health & Social Sciences, Bournemouth University, Bournemouth, UK; 516 Department of Public Health Sciences, University of North Carolina at Charlotte, Charlotte, NC, USA; 517 Education Development Center, Faculty Member of Education Development Center, Ahvaz Jundishapur University of Medical Sciences, Ahvaz, Iran; 518 Department of Psychology, University of Alabama at Birmingham, Birmingham, AL, USA; 519 Department of Psychiatry, Stellenbosch University, Cape Town, South Africa; 520 Emergency Department, Manian Medical Centre, Erode, India; 521 Microbiology Service, National Institutes of Health, Bethesda, MD, USA; 522 Center for Biomedical Information Technology, Shenzhen Institutes of Advanced Technology, Chinese Academy of Sciences, Shenzhen, China; 523 Department of Health Promotion and Education, Alborz University of Medical Sciences, Karaj, Iran; 524 Health Policy Research Center, Shiraz University of Medical Sciences, Shiraz, Iran; 525 Independent Consultant, Karachi, Pakistan; 526 School of Medicine, Alborz University of Medical Sciences, Karaj, Iran; 527 Centre for Medical Informatics, University of Edinburgh, Edinburgh, UK; 528 Division of General Internal Medicine, Harvard University, Boston, MA, USA; 529 National Institute of Infectious Diseases, Tokyo, Japan; 530 College of Medicine, Yonsei University, Seodaemun-gu, South Korea; 531 Division of Cardiology, Emory University, Atlanta, GA, USA; 532 Finnish Institute of Occupational Health, Helsinki, Finland; 533 Department of Health Education & Promotion, Kermanshah University of Medical Sciences, Kermanshah, Iran; 534 School of Health, University of Technology Sydney, Sydney, New South Wales, Australia; 535 Department of Psychology, Reykjavik University, Reykjavik, Iceland; 536 Department of Health and Behavior Studies, Columbia University, New York, NY, USA; 537 Department of Medicine, University of Alabama at Birmingham, Birmingham, AL, USA; 538 Medicine Service, US Department of Veterans Affairs (VA), Birmingham, AL, USA; 539 Department of Forensic Medicine, Kathmandu University, Dhulikhel, Nepal; 540 Department of Epidemiology, School of Preventive Oncology, Patna, India; 541 Department of Epidemiology, Healis Sekhsaria Institute for Public Health, Mumbai, India; 542 Medical Surgical Nursing Department, Urmia University of Medical Science, Urmia, Iran; 543 Emergency Nursing Department, Semnan University of Medical Sciences, Semnan, Iran; 544 Hospital Universitario de la Princesa, Autonomous University of Madrid, Madrid, Spain; 545 Centro de Investigación Biomédica en Red Enfermedades Respiratorias (CIBERES), Madrid, Spain; 546 Department of Public Health, Arba Minch University, Arba Minch, Ethiopia; 547 Hull York Medical School, University of Hull, Hull City, UK; 548 Usher Institute of Population Health Sciences and Informatics, University of Edinburgh, Edinburgh, UK; 549 Department of Psychology, Deakin University, Melbourne, Victoria, Australia; 550 Department of Community Medicine, Ahmadu Bello University, Zaria, Nigeria; 551 Department of Criminology, Law and Society, University of California Irvine, Irvine, CA, USA; 552 Department of Medicine, University of Valencia, Valencia, Spain; 553 Carlos III Health Institute, Biomedical Research Networking Center for Mental Health Network (CiberSAM), Madrid, Spain; 554 School of Social Work, University of Illinois, Urbana, IL, USA; 555 Public Health, Arba Minch College of Health Sciences, Arba Minch, Ethiopia; 556 School of Public Health, University of Adelaide, Adelaide, SA, Australia; 557 Department of Environmental Health, Wollo University, Dessie, Ethiopia; 558 Department of Community and Family Medicine, Iran University of Medical Sciences, Tehran, Iran; 559 Department of Pharmacognosy, Mekelle University, Mekelle, Ethiopia; 560 Institute of Public Health, University of Gondar, Gondar, Ethiopia; 561 Department of Public Health, Adigrat University, Adigrat, Ethiopia; 562 Biology Department, Moscow State University, Moscow, Russia; 563 Department of Nursing, Woldia University, Woldia, Ethiopia; 564 HIV/STI Surveillance Research Center, and WHO Collaborating Center for HIV Surveillance, Kerman University of Medical Sciences, Kerman, Iran; 565 Institute of Public Health, Krakow, Poland; 566 The Agency for Health Technology Assessment and Tariff System, Warszawa, Poland; 567 Department of Molecular Medicine and Pathology, University of Auckland, Auckland, New Zealand; 568 Clinical Hematology and Toxicology, Military Medical University, Hanoi, Vietnam; 569 Department of Health Economics, Hanoi Medical University, Hanoi, Vietnam; 570 Department of Psychiatry, Massachusetts General Hospital, Boston, MA, USA; 571 Mbarara University of Science and Technology, Mbarara, Uganda; 572 Department of Medicine, University of Crete, Heraklion, Greece; 573 Lee Kong Chian School of Medicine, Nanyang Technological University, Singapore, Singapore; 574 Gomal Center of Biochemistry and Biotechnology, Gomal University, Dera Ismail Khan, Pakistan; 575 TB Culture Laboratory, Mufti Mehmood Memorial Teaching Hospital, Dera Ismail Khan, Pakistan; 576 Amity Institute of Biotechnology, Amity University Rajasthan, Jaipur, India; 577 Division of Health Sciences, University of Warwick, Coventry, UK; 578 Argentine Society of Medicine, Buenos Aires, Argentina; 579 Velez Sarsfield Hospital, Buenos Aires, Argentina; 580 UKK Institute, Tampere, Finland; 581 Psychosocial Injuries Research Center, Ilam University of Medical Sciences, Ilam, Iran; 582 Raffles Neuroscience Centre, Raffles Hospital, Singapore, Singapore; 583 Yong Loo Lin School of Medicine, National University of Singapore, Singapore, Singapore; 584 Department of Medical and Surgical Sciences, University of Bologna, Bologna, Italy; 585 Occupational Health Unit, Sant’Orsola Malpighi Hospital, Bologna, Italy; 586 Department of Health Care Administration and Economics, National Research University Higher School of Economics, Moscow, Russia; 587 Foundation University Medical College, Foundation University, Islamabad, Pakistan; 588 Department of Physical Therapy, Naresuan University, Meung District, Thailand; 589 Department of Human Anatomy, Histology, and Embryology, Bahir Dar University, Bahir Dar, Ethiopia; 590 Department of Nursing, Wollo University, Dessie, Ethiopia; 591 Department of Orthopaedics, Wenzhou Medical University, Wenzhou, China; 592 Public Health Science Directorate, NHS Health Scotland, Glasgow, Scotland; 593 Medical Physics Department, Ahvaz Jundishapur University of Medical Sciences, Ahvaz, Iran; 594 Department of Preventive Medicine, Northwestern University, Chicago, IL, USA; 595 School of International Development and Global Studies, University of Ottawa, Ottawa, Ontario, Canada; 596 Health Services Management Research Center, Kerman University of Medical Sciences, Kerman, Iran; 597 Department of Health Management, Policy and Economics, Kerman University of Medical Sciences, Kerman, Iran; 598 Division of Injury Prevention and Mental Health Improvement, National Center for Chronic and Noncommunicable Disease Control, Chinese Center for Disease Control and Prevention, Beijing, China; 599 Centre for Suicide Research and Prevention, University of Hong Kong, Hong Kong, China; 600 Department of Social Work and Social Administration, University of Hong Kong, Hong Kong, China; 601 School of Allied Health Sciences, Addis Ababa University, Addis Ababa, Ethiopia; 602 Department of Psychopharmacology, National Center of Neurology and Psychiatry, Tokyo, Japan; 603 Department of Sociology, Yonsei University, Seoul, South Korea; 604 Department of Health Policy & Management, Jackson State University, Jackson, MS, USA; 605 School of Medicine, Tsinghua University, Beijing, China; 606 Department of Environmental Health, Mazandaran University of Medical Sciences, Sari, Iran; 607 Environmental Health, Academy of Medical Science, Sari, Iran; 608 Department of Epidemiology and Biostatistics, Wuhan University, Wuhan, China; 609 Global Health Institute, Wuhan University, Wuhan, China; 610 School of Public Health and Management, Hubei University of Medicine, Shiyan, China; 611 Social Determinants of Health Research Center, Ardabil University of Medical Science, Ardabil, Iran; 612 Department of Epidemiology, University Hospital of Setif, Setif, Algeria; 613 Department of Medicine, School of Clinical Sciences at Monash Health, Monash University, Melbourne, Victoria, Australia; 614 Student Research Committee, Babol University of Medical Sciences, Babol, Iran; 615 Department of Community Medicine, Ardabil University of Medical Science, Ardabil, Iran; 616 Faculty of Medical Sciences, Department of Health Education, Tarbiat Modares University, Tehran, Iran; 617 Department of Preventive Medicine, Wuhan University, Wuhan, China; 618 School of Public Health, Wuhan University of Science and Technology, Wuhan, China; 619 Hubei Province Key Laboratory of Occupational Hazard Identification and Control, Wuhan University of Science and Technology, Wuhan, China; 620 Indian Institute of Public Health, Public Health Foundation of India, Gurugram, India; 621 Public Health Foundation of India, Gurugram, India; 622 Department of Community Medicine, University of Peradeniya, Peradeniya, Sri Lanka

**Keywords:** populations/contexts, methodology, descriptive epidemiology, statistical issues

## Abstract

**Background:**

While there is a long history of measuring death and disability from injuries, modern research methods must account for the wide spectrum of disability that can occur in an injury, and must provide estimates with sufficient demographic, geographical and temporal detail to be useful for policy makers. The Global Burden of Disease (GBD) 2017 study used methods to provide highly detailed estimates of global injury burden that meet these criteria.

**Methods:**

In this study, we report and discuss the methods used in GBD 2017 for injury morbidity and mortality burden estimation. In summary, these methods included estimating cause-specific mortality for every cause of injury, and then estimating incidence for every cause of injury. Non-fatal disability for each cause is then calculated based on the probabilities of suffering from different types of bodily injury experienced.

**Results:**

GBD 2017 produced morbidity and mortality estimates for 38 causes of injury. Estimates were produced in terms of incidence, prevalence, years lived with disability, cause-specific mortality, years of life lost and disability-adjusted life-years for a 28-year period for 22 age groups, 195 countries and both sexes.

**Conclusions:**

GBD 2017 demonstrated a complex and sophisticated series of analytical steps using the largest known database of morbidity and mortality data on injuries. GBD 2017 results should be used to help inform injury prevention policy making and resource allocation. We also identify important avenues for improving injury burden estimation in the future.

## INTRODUCTION

The Global Burden of Disease (GBD) study is a comprehensive assessment of population health loss. GBD has expanded in scope since its original release in 1994 (GBD 1990) and was most recently updated in autumn 2018 (GBD 2017).^1–7^ Each update of the study has provided updated results through the most recent year of data availability as well as increasingly refined detail in terms of locations, age groups and causes. In addition, GBD incorporates new data as well as updated methods for each annual release that represent the expanding complexity of the study. Cumulatively, the increasing volume of data and increasingly sophisticated estimation methods have necessitated near-continual refinements in terms of data processing, statistical modelling, computational storage and processing as well as global collaboration with the over 4000 GBD collaborators in over 140 countries and territories.

Historically, injuries have formed one of the three broad cause groups in the GBD cause hierarchy alongside the other two main groups of health loss (communicable, maternal, neonatal and nutritional diseases; non-communicable diseases). Not surprisingly, there is considerable variation in how morbidity and mortality are estimated across different causes in the GBD hierarchy and study design. The methods for estimating morbidity and mortality from injuries have evolved over time through the most recent release of GBD 2017. Historically, there have been certain challenges in injuries burden estimation, some of which have been addressed and updated over time, and some of which remain as methodological challenges to address as population health measurement develops more sophisticated modelling strategies. For example, methodological challenges that have been identified over the past three decades in population health research have included obtaining data in data-sparse, burden-heavy areas of the world, developing adjustments for ill-defined causes of death, separately estimating *cause* of injury from the bodily harm that results from an injury event and adjusting for known biases in data, such as underestimation in sexual violence data.^3 8 9^ Cumulatively, the global injuries research community has developed a wide array of methodological innovations and advancements to overcome many of these challenges, although undoubtedly the science will continue to advance as higher-quality datasets become available, as modelling methods improve and as computational processing power becomes more accessible to population health research groups around the world.

Many studies have been published based on different releases of the GBD study, ranging from studies on intentional injuries in the eastern Mediterranean to detailed assessments of traumatic brain injury and spinal cord injury disability rates on a global scale.^10 11^ While this array of published GBD injury studies demonstrates a broad spectrum of expert knowledge on specific injuries or specific geographies or both, it is also critical to recognise that population health is a rapidly evolving, collaborative science that has benefited from near-continual improvements even through the current updates being implemented for GBD 2019. As a result, it should benefit the scientific enterprise to focus on publishing the most updated results with perspective on global, demographic and temporal patterns, and on sharing iterative updates on the current state of the science of GBD injuries burden estimation. The goal of this study is to comprehensively review and report methods used for GBD 2017 and associated publications that have gone through extensive collaborator-review and peer-review processes.

## METHODS

### GBD 2017 study

GBD is predicated on the principle that every case of death and disability in the population should be systematically identified and accounted for in the formulation of global disease and injury burden. On the side of mortality, every death that occurs in the population should have one underlying cause of death which can be assigned to a cause in a mutually exclusive, collectively exhaustive hierarchy of diseases and injuries that can cause death. These data can be used in a method described below to calculate cause-specific mortality rates and years of life lost. For morbidity, every non-fatal case of disease or injury should have an amount of disability assigned for some period of time. These data can be used in a process described below to estimate the incidence, prevalence and years lived with disability. Summing morbidity and mortality from some cause form the burden from that cause, expressed as disability-adjusted life-years (DALY). For causes with known risk factors, some portion of this burden may be explained by exposure to that risk factor. Across causes within some population, it is also a principle of GBD that the sum of all cause-specific deaths should equal all-cause mortality in the population, and that rates of incidence, prevalence, remission and cause-specific mortality can be reconciled with one another such that all death and disability in a population is internally consistent across causes and geographies. As examples, the sum of different types of road injury cases must sum up to overall road injuries, and the sum of deaths from different injuries in a given country must sum up to the estimate of all-injury deaths. The principle of internal consistency extends to populations used in GBD, where every birth, death and net migration must be accounted for in the population estimates which form the denominators of GBD results. While there is immense complexity in the process summarised above, it is important to begin with these core principles which govern the computation processes at the heart of GBD burden estimation. A summarised overview of key GBD 2017 methods is also provided in online supplementary appendix 1.

10.1136/injuryprev-2019-043531.supp1 Supplementary data

### GBD study design and hierarchies

GBD study design, including cause-specific methods, is described in a high level of detail in associated publications.^2–7^ In addition to the injury-focused methods described in this paper, it is important to define hierarchies used in the GBD study design. In particular, GBD 2017 was built around a location hierarchy where different subnational locations (eg, US states, India states, China provinces) which form a composite of a national location (eg, the USA, India, China). National locations are aggregated to form GBD regions, which are then aggregated to form GBD super regions. These designations affect the modelling structure and utilisation of location random effects, processes which are described in more detail later. The country-level and regional-level GBD location hierarchy used in GBD 2017 is provided in online supplementary appendix table 1. In addition to locations, GBD processes are conducted to produce estimates for every one of 22 age groups, male and female sex and across 28 years from 1990 to 2017 (inclusive). Age-standardised, all-age and combined sex results are also computed for each GBD result. Exceptions exist to the rules above, for example, self-harm is not permitted to occur in the 0–6 days (early neonatal) age group in the GBD age hierarchy. There are no sex restrictions placed on any GBD injury causes, although these restrictions exist for other GBD causes, such as cancers like prostate, cervical and uterine being related to one sex.

10.1136/injuryprev-2019-043531.supp2 Supplementary data

### GBD injury classification

In the GBD cause hierarchy, injuries are part of the first level of the GBD cause hierarchy, which consists of three broad groups: communicable, maternal, neonatal and nutritional diseases; non-communicable diseases and injuries. Additional levels of the GBD cause hierarchy provide additional detail. The hierarchy of injuries in GBD is provided in table 1. The organisation of the hierarchy has implications both in terms of how results are produced and in terms of analytical and processing steps which are discussed in more detail below. Case definitions including International Classification of Diseases (ICD) codes used to identify injury deaths and cases are provided in table 2.

**Table 1:** Global Burden of Disease cause-of-injury hierarchy

Transport injuries	Unintentional injuries	Self-harm and interpersonal violence	Forces of nature, conflict and terrorism and executions and police conflict
Road injuries	Falls	Self-harm	Exposure to forces of nature
Pedestrian road injuries	Drowning	Self-harm by firearm	Conflict and terrorism
Cyclist road injuries	Fire, heat and hot substances	Self-harm by other specified means	Executions and police conflict
Motorcyclist road injuries	Poisonings	Interpersonal violence	
Motor vehicle road injuries	Poisoning by carbon monoxide	Assault by firearm	
Other road injuries	Poisoning by other means	Assault by sharp object	
Other transport injuries	Exposure to mechanical forces	Assault by other means	
	Unintentional firearm injuries		
	Unintentional suffocation		
	Other exposure to mechanical forces		
	Adverse effects of medical treatment		
	Animal contact		
	Venomous animal contact		
	Non-venomous animal contact		
	Foreign body		
	Pulmonary aspiration and foreign body in airway		
	Foreign body in eyes		
	Foreign body in other body part		
	Environmental heat and cold exposure		
	Other unintentional injuries		

**Table 2:** Case definitions for cause of injury in GBD 2017

Child causes	ICD codes	Case definition (fatal)	Case definition (non-fatal)
Self-harm	**ICD9**: E950-E959 **ICD10**: X60-X64.9, X66-X84.9, Y87.0	Deliberate bodily damage inflicted on oneself resulting in death	Deliberate bodily damage inflicted on oneself with or without intent to kill oneself.
Self-harm by firearm	**ICD9**: E955-E955.9 **ICD10**: X72-X74.9	Deliberate bodily damage inflicted by firearm on oneself resulting in death	Deliberate bodily damage inflicted on oneself by firearm with or without intent to kill oneself.
Self-harm by other specified means	**ICD9**: E950-E954, E956-E958.0, E958.2-E959 **ICD10**: X60-X64.9, X66-X67.9, X69-X71.9, X75- X75.9, X77-X84.9, Y87.0	Deliberate bodily damage inflicted on oneself resulting in death by means of:* Self-poisoning Medication overdose Transport incident Falling from height Hanging/strangulation *(not exhaustive)	Deliberate bodily damage inflicted on oneself with or without intent to kill oneself by means of:* Self-poisoning Medication overdose Transport incident Falling from height Hanging/strangulation *(not exhaustive)
Poisoning	**ICD9**: E850.3-E858.99, E862-E869.99, E929.2 **ICD10**: J70.5, X40-X44.9, X47-X49.9, Y10-Y14.9, Y16-Y19.9	Death resulting from accidental exposure to a non-infectious substance which contacts the body or enters into the body via inhalation, ingestion, injection or absorption and causes deranged physiological function of body and/or cellular injury/death.	Unintentional exposure to a non-infectious substance which contacts the body or enters into the body via inhalation, ingestion, injection or absorption and causes deranged physiological function of body and/or cellular injury/death.
Poisoning by carbon monoxide (CO)	**ICD9**: E862-E862.99, E868-E869.99 **ICD10**: J70.5, X47-X47.9	Death from exposure to carbon monoxide (CO) as identified based on carboxyhemoglobin levels (specified based on smoking status and age) or proximity to a confirmed CO poisoning case.	Non-fatal exposure to CO as identified based on carboxyhemoglobin levels (specified based on smoking status and age) or proximity to a confirmed CO poisoning case.
Poisoning by other means	**ICD9**: E850.3-E858.99, E866-E866.99 **ICD10**: X40-X44.9, X49-X49.9, Y10-Y14.9, Y16-Y19.9	Death resulting from accidental exposure to a non-infectious substance (other than CO) which contacts the body or enters into the body via inhalation, ingestion, injection or absorption and causes deranged physiological function of body and/or cellular injury/death.	Accidental exposure to a non-infectious substance (other than CO) which contacts the body or enters into the body via inhalation, ingestion, injection or absorption and causes deranged physiological function of body and/or cellular injury/death.
Animal contact	**ICD9**: E905-E906.99 **ICD10**: W52.0-W62.9, W64-W64.9, X20-X29.9	Death resulting from unintentionally being attacked, struck, impaled, bitten, stung, crushed, exposed to or stepped on by a non-human animal.	Bodily damage resulting from unintentionally being attacked, butted, impaled, bitten, stung, crushed, exposed to or stepped on by a non-human animal.
Venomous animal contact	**ICD9**: E905-E905.99 **ICD10**: W52.3, X20-X29.9	Death resulting from unintentionally being bitten by, stung by, or exposed to a non-human venomous animal.	Bodily damage resulting from unintentionally being bitten by, stung by or exposed to a non-human venomous or poisonous animal.
Non-venomous animal contact	**ICD9**: E905-E906.99 **ICD10**: W52.0-W62.9, W64-W64.9, X20-X29.9	Death resulting from unintentionally being attacked, struck, impaled, crushed, exposed to or stepped on by a non-human animal.	Bodily damage resulting from unintentionally being attacked, struck, impaled, crushed, exposed to or stepped on by a non-human animal.
Falls	**ICD9**: E880-E886.99, E888-E888.9, E929.3 **ICD10**: W00-W19.9	A sudden movement downwards due to slipping, tripping or other accidental movement which results in a person coming to rest inadvertently on the ground, floor or other lower level, resulting in death.	A sudden movement downward due to slipping, tripping or other accidental movement which results in a person coming to rest inadvertently on the ground, floor or other lower level, resulting in tissue damage.
Drowning	**ICD10:** W65-W70.9, W73-W74.9 **ICD9:** E910-E910.99	Death that occurs as a result of immersion in water or another fluid.	Non-fatal immersion or submersion in water or another fluid, regardless of whether tissue damage has occurred. The subject can be resuscitated and has not suffered brain death.
Fire, heat, and hot substances	**ICD9:** E890-E899.09, E924-E924.99, E929.4 **ICD10:** X00-X06.9, X08-X19.9	Death due to unintentional exposure to substances of high temperature sufficient to cause tissue damage on exposure, including bodily contact with hot liquid, solid or gas such as cooking stoves, smoke, steam, drinks, machinery, appliances, tools, radiators and objects radiating heat energy.	Unintentional exposure to substances of high temperature sufficient to cause tissue damage on exposure, including bodily contact with hot liquid, solid or gas such as cooking stoves, smoke, steam, drinks, machinery, appliances, tools, radiators and objects radiating heat energy.
Road injuries	**ICD9:** E800.3, E801.3, E802.3, E803.3, E804.3, E805.3, E806.3, E807.3, E810.0-E810.6, E811.0-E811.7, E812.0-E812.7, E813.0- E813.7, E814.0-E814.7, E815.0-E815.7, E816.0-E816.7, E817.0-E817.7, E818.0- E818.7, E819.0-E819.7, E820.0-E820.6, E821.0-E821.6, E822.0-E822.7, E823.0- E823.7, E824.0-E824.7, E825.0-E825.7, E826.0-E826.1, E826.3-E826.4, E827.0, E827.3-E827.4, E828.0, E828.4, E829.0- E829.4 **ICD10:** V01-V04.99, V06-V80.929, V82-V82.9, V87.2-V87.3	Interaction with an automobile, motorcycle, pedal cycle or other vehicles resulting in death.	Interaction with an automobile, motorcycle, pedal cycle or other vehicles resulting in bodily damage.
Pedestrian road injuries	**ICD9:** E811.7, E812.7, E813.7, E814.7, E815.7, E816.7, E817.7, E818.7, E819.7, E822.7, E823.7, E824.7, E825.7, E826.0, E827.0, E828.0, E829.0 **ICD10:** V01-V04.99, V06-V09.9	Interaction, as a pedestrian on the road, with an automobile, motorcycle, pedal cycle or other vehicles resulting in death.	Interaction, as a pedestrian on the road, with an automobile, motorcycle, pedal cycle or other vehicles resulting in bodily damage.
Cyclist road injuries	**ICD9:** E800.3, E801.3, E802.3, E803.3, E804.3, E805.3, E806.3, E807.3, E810.6, E811.6, E812.6, E813.6, E814.6, E815.6, E816.6, E817.6, E818.6, E819.6, E820.6, E821.6, E822.6, E823.6, E824.6, E825.6, E826.1 **ICD10:** V10-V19.9	Accident, as a cyclist or passenger on a pedal cycle, resulting in death.	Accident, as a cyclist or passenger on a pedal cycle, resulting in bodily damage.
Motorcyclist road injuries	**ICD9:** E810.2-E810.3, E811.2-E811.3, E812.2- E812.3, E813.2-E813.3, E814.2-E814.3, E815.2-E815.3, E816.2-E816.3, E817.2- E817.3, E818.2-E818.3, E819.2-E819.3, E820.2-E820.3, E821.2-E821.3, E822.2- E822.3, E823.2-E823.3, E824.2-E824.3, E825.2-E825.3 **ICD10:** V20-V29.9	Accident, as a rider on a motorcycle, resulting in death.	Accident, as a rider on a motorcycle, resulting in bodily damage.
Motor vehicle road injuries	**ICD9:** E810.0-E810.1, E811.0-E811.1, E812.0- E812.1, E813.0-E813.1, E814.0-E814.1, E815.0-E815.1, E816.0-E816.1, E817.0- E817.1, E818.0-E818.1, E819.0-E819.1, E820.0-E820.1, E821.0-E821.1, E822.0- E822.1, E823.0-E823.1, E824.0-E824.1, E825.0-E825.1 **ICD10:** V30-V79.9, V87.2-V87.3	Accident, as a driver or passenger in a motor vehicle, resulting in death.	Accident, as a driver or passenger in a motor vehicle, resulting in bodily damage.
Other road injuries	**ICD9:** E810.4-E810.5, E811.4-E811.5, E812.4- E812.5, E813.4-E813.5, E814.4-E814.5, E815.4-E815.5, E816.4-E816.5, E817.4- E817.5, E818.4-E818.5, E819.4-E819.5, E820.4-E820.5, E821.4-E821.5, E822.4- E822.5, E823.4-E823.5, E824.4-E824.5, E825.4-E825.5, E826.3-E826.4, E827.3- E827.4, E828.4, E829.4 **ICD10:** V80-V80.929, V82-V82.9	Death resulting from being a driver or passenger of a vehicle not including automobiles, motorcycles, bicycles (ie, streetcar).	Bodily damage resulting from being a driver or passenger of a vehicle not including automobiles, motorcycles, bicycles (ie, streetcar).
Other transport injuries	**ICD9:** E800-E800.2, E801-E801.2, E802-E802.2, E803-E803.2, E804-E804.2, E805-E805.2, E806-E806.2, E807-E807.2, E810.7, E820.7, E821.7, E826.2, E827.2, E828.2, E830-E838.9, E840-E849.9, E929.1 **ICD10:** V00-V00.898, V05-V05.99, V81-V81.9, V83-V86.99, V88.2-V88.3, V90-V98.8	Interaction with a means of transport other than automobile, motorcycle, pedal cycle or other road vehicles resulting in death.	Interaction with a means of transport other than automobile, motorcycle, pedal cycle or other road vehicles resulting in bodily damage.
Interpersonal violence	**ICD9:** E960-E969 **ICD10:** X85-Y08.9, Y87.1-Y87.2	Death from intentional use of physical force or power, threatened or actual, from another person or group not including military or police forces.	Sustaining bodily harm in terms of tissue damage from intentional use of physical force or power, threatened or actual, from another person or group not including military or police forces.
Physical violence by firearm	**ICD9:** E965-E965.4 **ICD10:** X93-X95.9	Death from intentional use of physical force or power by a firearm from another person or group or community not including military or police forces.	Sustaining bodily harm in terms of tissue damage from intentional use of physical force or power by a firearm from another person or group not including military or police forces.
Physical violence by sharp object	**ICD9:** E966 **ICD10:** X99-X99.9	Death from intentional use of physical force or power by a sharp object from another person or group or community not including military or police forces.	Sustaining bodily harm in terms of tissue damage from intentional use of physical force or power by a sharp object from another person or group not including military or police forces.
Sexual violence	**ICD9:** E960-E960.1 **ICD10:** Y05-Y05.9	NA	Experiencing at least one event of sexual violence in the last year, where sexual violence is defined as any sexual assault, including both penetrative sexual violence (rape) and non-penetrative sexual violence (other forms of unwanted sexual touching).
Physical violence by other means	**ICD9:** E961-E964, E965.5-E965.9, E967-E969 **ICD10:** X85-X92.9, X96-X98.9, Y00-Y04.9, Y06- Y08.9, Y87.1-Y87.2	Death from intentional use of physical force or power by an object other than a firearm or sharp object from another person or group or community not including military or police forces.	Sustaining bodily harm in terms of tissue damage from intentional use of physical force or power by an object other than a firearm or sharp object from another person or group not including military or police forces.
Conflict and terrorism	**ICD9:** E979-E979.9, E990-E999.1 **ICD10:** U00-U03, Y36-Y38.9, Y89.1	Death resulting from the instrumental use of violence by people who identify themselves as members of a group—whether this group is transitory or has a more permanent identity—against another group or set of individuals, in order to achieve political, economic or social objectives.	Bodily harm resulting from the instrumental use of violence by people who identify themselves as members of a group—whether this group is transitory or has a more permanent identity—against another group or set of individuals, in order to achieve political, economic or social objectives.
Executions and police conflict	**ICD9:** E970-E978 **ICD10:** Y35-Y35.93, Y89.0	State-sanctioned executions or police-related altercations leading to death.	State-sanctioned executions or police-related altercations leading to bodily damage.
Exposure to forces of nature	**ICD9:** E907-E909.9 **ICD10:** X33-X38.9	Death resulting from an unforeseen and often sudden natural event such as a hurricane, earthquake, tsunami or tornado.	Bodily damage resulting from an unforeseen and often sudden natural event such as a hurricane, earthquake, tsunami or tornado.
Exposure to mechanical forces	**ICD9:** E913-E913.19, E916-E922.99, E928.1- E928.7 **ICD10:** W20-W38.9, W40-W43.9, W45.0-W45.2, W46-W46.2, W49-W52, W75-W76.9	Unintentional death resulting from contact with or threat of an (in)animate object, human or plant.	Unintentional bodily damage resulting from contact with or threat of an (in)animate object, human or plant.
Unintentional firearm injuries	**ICD9:** E922-E922.99, E928.7 **ICD10:** W32-W34.9	Unintentional death resulting from contact with a firearm.	Unintentional bodily damage resulting from contact with a firearm.
Other exposure to mechanical forces	**ICD9:** E916-E921.99, E928.1-E928.6 **ICD10:** W20-W31.9, W35-W38.9, W40-W43.9, W45.0-W45.2, W46-W46.2, W49-W52	Unintentional death resulting from contact with or threat of an (in)animate object (not including a firearm), human or plant.	Unintentional bodily damage resulting from contact with or threat of an (in)animate object (not including a firearm), human or plant.
Pulmonary aspiration and foreign body in airway	**ICD9:** 770.1–770.18, E911-E912.09, E913.8- E913.99 **ICD10:** W78-W80.9, W83-W84.9	Unintentional death from inhaling, swallowing or aspirating extraneous materials or substance that enters the airway or lungs.	Unintentional bodily damage from inhaling, swallowing or aspirating extraneous materials or substance that enters the airway or lungs.
Foreign body in eyes	**ICD9:** 360.5–360.69, 374.86, 376.6, E914- E914 09 **ICD10:** H02.81-H02.819, H44.6-H44.799	NA	Unintentional damage from extraneous materials or substance in the orbital structure or eye.
Foreign body in other body part	**ICD9:** 709.4, E915-E915.09 **ICD10:** M60.2-M60.28, W44-W45, W45.3-W45.9	Unintentional death from an extraneous material or substance being within the body, not including the airway, lungs or eyes.	Unintentional bodily damage from an extraneous material or substance being within the body, not including the airway, lungs or eyes.

Injuries definition: damage, defined by cellular death, tissue disruption, loss of homeostasis, pain limiting activities of daily living or short-term psychological harm (for cases of sexual violence), inflicted on the body as the direct or indirect result of a physical force, immersion or exposure, which may include interpersonal or self-inflicted forces.

GBD, Global Burden of Disease; ICD, International Classification of Diseases.

GBD separates the concept of cause of injury from nature of injury. Cause of injury (eg, road injuries, falls, drowning) have historically been used for assigning cause of death as opposed to the ‘nature’ of injury, which more directly specifies the pathology that resulted in death. For example, an individual who falls, fractures his or her hip, undergoes surgery and then develops hospital-acquired pneumonia and dies while hospitalised would still have a fall as the underlying cause of death, regardless of whether sepsis or some other disease process leads to death more proximally in the chain of events. In this individual, the ‘nature’ of injury would have been specified as a hip fracture, since it is the bodily injury that would dictate the disability this person experiences. Since it is evident that a hip fracture is more disabling than a mild skin abrasion, it is important for measuring non-fatal burden to consider both the cause and the nature in the formulation of complete injury burden. A full list of nature of injury is provided in table 3.

**Table 3:** GBD nature of injury

**Nature of injury**		
Amputation of lower limbs, bilateral	Fracture of sternum and/or fracture of one or more ribs	Crush injury
Amputation of upper limbs, bilateral	Fracture of vertebral column	Nerve injury
Amputation of fingers (excluding thumb)	Fracture of femur, other than femoral neck	Injury to eyes
Amputation of lower limb, unilateral	Minor TBI	Poisoning requiring urgent care
Amputation of upper limb, unilateral	Moderate/severe TBI	Severe chest injury
Amputation of thumb	Spinal cord lesion at neck level	Internal haemorrhage in abdomen and pelvis
Amputation of toe/toes	Spinal cord lesion below neck level	Effect of different environmental factors
Lower airway burns	Muscle and tendon injuries, including sprains and strains lesser dislocations	Complications following therapeutic procedures
Burns, <20% total burned surface area without lower airway burns	Foreign body in ear	Multiple fractures, dislocations, crashes, wounds, pains and strains
Burns, ≥20% total burned surface area or ≥10% burned surface area if head/neck or hands/wrist involved without lower airway burns	Open wound(s)	
Fracture of clavicle, scapula or humerus	Contusion in any part of the body	
Fracture of face bones	Superficial injury of any part of the body	
Fracture of foot bones except ankle	Dislocation of hip	
Fracture of hand (wrist and other distal part of hand)	Dislocation of knee	
Fracture of hip	Dislocation of shoulder	
Fracture of patella, tibia or fibula or ankle	Foreign body in respiratory system	
Fracture of pelvis	Foreign body in GI and urogenital system	
Fracture of radius and/or ulna	Drowning and non-fatal submersion	
Fracture of skull	Asphyxiation	

GBD, Global Burden of Disease; GI, gastrointestinal; TBI, traumatic brain injury.

### Cause-specific mortality and years of life lost

As described above, cause-specific mortality is measured for every cause of injury in the GBD cause hierarchy with the exception of foreign body in the ear and sexual violence, which undergo only non-fatal burden estimation (described in more detail below). GBD adheres to five general principles for measuring cause-specific mortality, which are described in more detail elsewhere but are summarised as follows.^12^ First, GBD 2017 identifies all available data. For injuries, this includes vital registration (VR), vital registration samples, verbal autopsy (VA), police records and mortuary/hospital data. VR is the preferred data source but is not available in every location in the GBD location hierarchy. Prior VA research has demonstrated that VA is more accurate for certain injury causes than it is for certain diseases.^13^ Police data undergo additional validity checks to ensure that systematic under-reporting does not occur in comparison to VR data, which is described in more detail in a related publication.^6^ The second general principle relevant to injury mortality estimation is maximising comparability and quality of the dataset. For the purposes of injury mortality estimation, this process is largely focused on (1) ensuring appropriate accounting for different ICD code versions used for cause of death data classification over time, (2) redistribution of ill-defined causes of death (described in more detail elsewhere) and (3) processing VA studies into usable data that map to the GBD cause hierarchy.^8 9 12^ The third general principle for injury cause of death models in GBD 2017 is to develop a diverse set of plausible models. This process is conducted via the Cause of Death Ensemble model (CODEm) framework, which is the standard, peer-reviewed cause of death estimation process used extensively in the GBD study. CODEm generates a large set of possible models based on covariates suggested by the modeller based on expert input and literature review (eg, alcohol for road injuries) and then runs every plausible model, which can range into the thousands per cause. These models can be conducted in both rate space and cause fraction space and use an assortment of combinations among the user-selected covariates (table 4). Fourth, the predictive validity of each one of these submodels is tested using test-train holdouts, whereby a specific model is trained on a portion of data and tested on a separate portion to determine out-of-sample predictive validity. Once the submodels are conducted and predictive validity is measured, then an ensemble model is developed out of the submodels. The submodels and the ensemble model are then subject to the fifth principle, which is to choose the best-performing models based on out-of-sample predictive validity. The chosen models may be a single cause model or an ensemble of models. Beyond these processes, which have become automated with expert review in the GBD processing architecture, there is also considerable time required by the analysts, modellers, collaborators and principal investigators who are involved in the GBD study. Such processes also come under expert scrutiny via the GBD Scientific Council and the peer-review process in the annual GBD capstone publications.^2–7^

**Table 4:** Covariates used in GBD cause of death models

**Cause**	**Global or data-rich model**	**Sex**	**Number of covariates used**	**Covariates used**
Transport injuries	Global/Data rich	Male	10	Alcohol (litres per capita), Education (years per capita), Lag distributed income per capita (I$), Population Density (300–500 ppl/sqkm, proportion), Population Density (500–1000 ppl/sqkm, proportion), Rainfall Quintile 5 (proportion), Vehicles–two+four wheels (per capita), Vehicles–two wheels fraction (proportion), Sociodemographic Index, Healthcare Access and Quality Index
Transport injuries	Global/Data rich	Female	10	Alcohol (litres per capita), Education (years per capita), Lag distributed income per capita (I$), Population Density (300–500 ppl/sqkm, proportion), Population Density (500–1000 ppl/sqkm, proportion), Rainfall Quintile 5 (proportion), Vehicles–two+four wheels (per capita), Vehicles–two wheels fraction (proportion), Sociodemographic Index, Healthcare Access and Quality Index
Road injuries	Global/Data rich	Male	13	Alcohol (liters per capita), Education (years per capita), Lag distributed income per capita (I$), Population 15 to 30 (proportion), Population Density (300–500 ppl/sqkm, proportion), Population Density (500–1000 ppl/sqkm, proportion), Vehicles–two+four wheels (per capita), Vehicles–two wheels (per capita), Vehicles - 4 wheels (per capita), Vehicles–two wheels fraction (proportion), Log-transformed summary exposure value (SEV) scalar: Road Inj, Sociodemographic Index, Healthcare Access and Quality Index
Road injuries	Global/Data rich	Female	13	Alcohol (liters per capita), Education (years per capita), Lag distributed income per capita (I$), Population 15 to 30 (proportion), Population Density (300–500 ppl/sqkm, proportion), Population Density (500–1000 ppl/sqkm, proportion), Vehicles–two+four wheels (per capita), Vehicles–two wheels (per capita), Vehicles - 4 wheels (per capita), Vehicles–two wheels fraction (proportion), Log-transformed SEV scalar: Road Inj, Sociodemographic Index, Healthcare access and quality index
Pedestrian road injuries	Global/Data rich	Male	11	Alcohol (liters per capita), Education (years per capita), Population Density (300–500 ppl/sqkm, proportion), Population Density (500–1000 ppl/sqkm, proportion), Rainfall Quintile 5 (proportion), Vehicles–two+four wheels (per capita), Vehicles–two wheels fraction (proportion), Log-transformed SEV scalar: Pedest, Sociodemographic Index, Healthcare Access and Quality Index, Lag distributed income per capita (I$)
Pedestrian road injuries	Global/Data rich	Female	11	Alcohol (liters per capita), Education (years per capita), Population Density (300–500 ppl/sqkm, proportion), Population Density (500–1000 ppl/sqkm, proportion), Rainfall Quintile 5 (proportion), Vehicles–two+four wheels (per capita), Vehicles–two wheels fraction (proportion), Log-transformed SEV scalar: Pedest, Sociodemographic Index, Healthcare Access and Quality Index, Lag distributed income per capita (I$)
Cyclist road injuries	Global/Data rich	Male	10	Alcohol (liters per capita), Education (years per capita), Population Density (300–500 ppl/sqkm, proportion), Population Density (500–1000 ppl/sqkm, proportion), Vehicles–two+four wheels (per capita), Vehicles - two wheels fraction (proportion), Log-transformed SEV scalar: Cyclist, Sociodemographic Index, Healthcare Access and Quality Index, Lag distributed income per capita (I$)
Cyclist road injuries	Global/Data rich	Female	10	Alcohol (liters per capita), Education (years per capita), Population Density (300–500 ppl/sqkm, proportion), Population Density (500–1000 ppl/sqkm, proportion), Vehicles - two+four wheels (per capita), Vehicles–two wheels fraction (proportion), Log-transformed SEV scalar: Cyclist, Sociodemographic Index, Healthcare Access and Quality Index, Lag distributed income per capita (I$)
Motorcyclist road injuries	Global/Data rich	Male	10	Alcohol (liters per capita), Education (years per capita), Population Density (300–500 ppl/sqkm, proportion), Population Density (500–1000 ppl/sqkm, proportion), Rainfall Quintile 5 (proportion), Vehicles–two wheels (per capita), Log-transformed SEV scalar: Mot Cyc, Sociodemographic Index, Healthcare Access and Quality Index, Lag distributed income per capita (I$)
Motorcyclist road injuries	Global/Data rich	Female	10	Alcohol (liters per capita), Education (years per capita), Population Density (300–500 ppl/sqkm, proportion), Population Density (500–1000 ppl/sqkm, proportion), Rainfall Quintile 5 (proportion), Vehicles–two wheels (per capita), Log-transformed SEV scalar: Mot Cyc, Sociodemographic Index, Healthcare Access and Quality Index, Lag distributed income per capita (I$)
Motor vehicle road injuries	Global/Data rich	Male	10	Alcohol (liters per capita), Education (years per capita), Population Density (300–500 ppl/sqkm, proportion), Population Density (500–1000 ppl/sqkm, proportion), Rainfall Quintile 5 (proportion), Vehicles–four wheels (per capita), Log-transformed SEV scalar: Mot Veh, Sociodemographic Index, Healthcare Access and Quality Index, Lag distributed income per capita (I$)
Motor vehicle road injuries	Global/Data rich	Female	10	Alcohol (liters per capita), Education (years per capita), Population Density (300–500 ppl/sqkm, proportion), Population Density (500–1000 ppl/sqkm, proportion), Rainfall Quintile 5 (proportion), Vehicles–four wheels (per capita), Log-transformed SEV scalar: Mot Veh, Sociodemographic Index, Healthcare Access and Quality Index, Lag distributed income per capita (I$)
Other road injuries	Global/Data rich	Male	8	Alcohol (liters per capita), Rainfall Quintile 5 (proportion), Vehicles–two+four wheels (per capita), Vehicles–two wheels fraction (proportion), Log-transformed SEV scalar: Oth Road, Sociodemographic Index, Healthcare Access and Quality Index, Lag distributed income per capita (I$)
Other road injuries	Global/Data rich	Female	8	Alcohol (liters per capita), Rainfall Quintile 5 (proportion), Vehicles–two+four wheels (per capita), Vehicles–two wheels fraction (proportion), Log-transformed SEV scalar: Oth Road, Sociodemographic Index, Healthcare Access and Quality Index, Lag distributed income per capita (I$)
Other transport injuries	Global/Data rich	Male	11	Alcohol (liters per capita), Education (years per capita), Population Density (300–500 ppl/sqkm, proportion), Population Density (500–1000 ppl/sqkm, proportion), Rainfall Quintile 5 (proportion), Vehicles–two+four wheels (per capita), Vehicles–two wheels fraction (proportion), Log-transformed SEV scalar: Oth Trans, Sociodemographic Index, Healthcare Access and Quality Index, Lag distributed income per capita (I$)
Other transport injuries	Global/Data rich	Female	11	Alcohol (liters per capita), Education (years per capita), Population Density (300–500 ppl/sqkm, proportion), Population Density (500–1000 ppl/sqkm, proportion), Rainfall Quintile 5 (proportion), Vehicles–two+four wheels (per capita), Vehicles–two wheels fraction (proportion), Log-transformed SEV scalar: Oth Trans, Sociodemographic Index, Healthcare Access and Quality Index, Lag distributed income per capita (I$)
Falls	Global/Data rich	Male	7	Alcohol (liters per capita), Elevation Over 1500 m (proportion), Log-transformed SEV scalar: Falls, Sociodemographic Index, milk adjusted(g), Healthcare Access and Quality Index, Lag distributed income per capita (I$)
Falls	Global/Data rich	Female	7	Alcohol (liters per capita), Elevation Over 1500 m (proportion), Log-transformed SEV scalar: Falls, Sociodemographic Index, milk adjusted(g), Healthcare Access and Quality Index, Lag distributed income per capita (I$)
Drowning	Global/Data rich	Male	10	Alcohol (liters per capita), Coastal Population within 10 km (proportion), Education (years per capita), Landlocked Nation (binary), Elevation Under 100 m (proportion), Rainfall Quintile 1 (proportion), Rainfall Quintile 5 (proportion), Log-transformed SEV scalar: Drown, Sociodemographic Index, Lag distributed income per capita (I$)
Drowning	Global/Data rich	Female	10	Alcohol (liters per capita), Coastal Population within 10 km (proportion), Education (years per capita), Landlocked Nation (binary), Elevation Under 100 m (proportion), Rainfall Quintile 1 (proportion), Rainfall Quintile 5 (proportion), Log-transformed SEV scalar: Drown, Sociodemographic Index, Lag distributed income per capita (I$)
Fire, heat and hot substances	Global/Data rich	Male	9	Alcohol (liters per capita), Tobacco (cigarettes per capita), Education (years per capita), Indoor Air Pollution (All Cooking Fuels), Population Density (over 1000 ppl/sqkm, proportion), Log-transformed SEV scalar: Fire, Sociodemographic Index, Healthcare Access and Quality Index, Lag distributed income per capita (I$)
Fire, heat and hot substances	Global/Data rich	Female	9	Alcohol (liters per capita), Tobacco (cigarettes per capita), Education (years per capita), Indoor Air Pollution (All Cooking Fuels), Population Density (over 1000 ppl/sqkm, proportion), Log-transformed SEV scalar: Fire, Sociodemographic Index, Healthcare Access and Quality Index, Lag distributed income per capita (I$)
Poisonings	Global/Data rich	Male	8	Education (years per capita), Opium Cultivation (binary), Population Density (over 1000 ppl/sqkm, proportion), Population Density (under 150 ppl/sqkm, proportion), Log-transformed SEV scalar: Poison, Sociodemographic Index, Healthcare Access and Quality Index, Lag distributed income per capita (I$)
Poisonings	Global/Data rich	Female	8	Education (years per capita), Opium Cultivation (binary), Population Density (over 1000 ppl/sqkm, proportion), Population Density (under 150 ppl/sqkm, proportion), Log-transformed SEV scalar: Poison, Sociodemographic Index, Healthcare Access and Quality Index, Lag distributed income per capita (I$)
Poisoning by carbon monoxide	Global/Data rich	Male	4	Education (years per capita), Lag distributed income per capita (I$), Sociodemographic Index, Healthcare Access and Quality Index
Poisoning by carbon monoxide	Global/Data rich	Female	4	Education (years per capita), Lag distributed income per capita (I$), Sociodemographic Index, Healthcare access and quality index
Poisoning by other means	Global/Data rich	Male	4	Education (years per capita), Lag distributed income per capita (I$), Sociodemographic Index, Healthcare access and quality index
Poisoning by other means	Global/Data rich	Female	4	Education (years per capita), Lag distributed income per capita (I$), Sociodemographic Index, Healthcare access and quality index
Exposure to mechanical forces	Global/Data rich	Male	7	Alcohol (liters per capita), Education (years per capita), Population Density (over 1000 ppl/sqkm, proportion), Population Density (under 150 ppl/sqkm, proportion), Sociodemographic Index, Healthcare access and quality index, Lag distributed income per capita (I$)
Exposure to mechanical forces	Global/Data rich	Female	7	Alcohol (liters per capita), Education (years per capita), Population Density (over 1000 ppl/sqkm, proportion), Population Density (under 150 ppl/sqkm, proportion), Sociodemographic Index, Healthcare access and quality index, Lag distributed income per capita (I$)
Unintentional firearm injuries	Global/Data rich	Male	9	Alcohol (liters per capita), Education (years per capita), Health System Access (unitless), Population Density (over 1000 ppl/sqkm, proportion), Population Density (under 150 ppl/sqkm, proportion), Log-transformed SEV scalar: Mech Gun, Sociodemographic Index, Healthcare Access and Quality Index, Lag distributed income per capita (I$)
Unintentional firearm injuries	Global/Data rich	Female	9	Alcohol (liters per capita), Education (years per capita), Health System Access (unitless), Population Density (over 1000 ppl/sqkm, proportion), Population Density (under 150 ppl/sqkm, proportion), Log-transformed SEV scalar: Mech Gun, Sociodemographic Index, Healthcare Access and Quality Index, Lag distributed income per capita (I$)
Other exposure to mechanical forces	Global/Data rich	Male	9	Alcohol (liters per capita), Education (years per capita), Health System Access (unitless), Population Density (over 1000 ppl/sqkm, proportion), Population Density (under 150 ppl/sqkm, proportion), Log-transformed SEV scalar: Oth Mech, Sociodemographic Index, Healthcare Access and Quality Index, Lag distributed income per capita (I$)
Other exposure to mechanical forces	Global/Data rich	Female	9	Alcohol (liters per capita), Education (years per capita), Health System Access (unitless), Population Density (over 1000 ppl/sqkm, proportion), Population Density (under 150 ppl/sqkm, proportion), Log-transformed SEV scalar: Oth Mech, Sociodemographic Index, Healthcare Access and Quality Index, Lag distributed income per capita (I$)
Adverse effects of medical treatment	Global/Data rich	Male	3	Lag distributed income per capita (I$), Sociodemographic Index, Healthcare Access and Quality Index
Adverse effects of medical treatment	Global/Data rich	Female	3	Lag distributed income per capita (I$), Sociodemographic Index, Healthcare Access and Quality Index
Animal contact	Global/Data rich	Male	11	Alcohol (liters per capita), Education (years per capita), Elevation Over 1500 m (proportion), Population 15 to 30 (proportion), Population Density (over 1000 ppl/sqkm, proportion), Population Density (under 150 ppl/sqkm, proportion), Elevation Under 100 m (proportion), Log-transformed SEV scalar: Animal, Sociodemographic Index, Healthcare Access and Quality Index, Lag distributed income per capita (I$)
Animal contact	Global/Data rich	Female	11	Alcohol (liters per capita), Education (years per capita), Elevation Over 1500 m (proportion), Population 15 to 30 (proportion), Population Density (over 1000 ppl/sqkm, proportion), Population Density (under 150 ppl/sqkm, proportion), Elevation Under 100 m (proportion), Log-transformed SEV scalar: Animal, Sociodemographic Index, Healthcare Access and Quality Index, Lag distributed income per capita (I$)
Venomous animal contact	Global/Data rich	Male	10	Alcohol (liters per capita), Education (years per capita), Elevation Over 1500 m (proportion), Population Density (over 1000 ppl/sqkm, proportion), Population Density (under 150 ppl/sqkm, proportion), Elevation Under 100 m (proportion), Log-transformed SEV scalar: Venom, Sociodemographic Index, Healthcare Access and Quality Index, Lag distributed income per capita (I$)
Venomous animal contact	Global/Data rich	Female	10	Alcohol (liters per capita), Education (years per capita), Elevation Over 1500 m (proportion), Population Density (over 1000 ppl/sqkm, proportion), Population Density (under 150 ppl/sqkm, proportion), Elevation Under 100 m (proportion), Log-transformed SEV scalar: Venom, Sociodemographic Index, Healthcare Access and Quality Index, Lag distributed income per capita (I$)
Non-venomous animal contact	Global	Male	6	Alcohol (liters per capita), Education (years per capita), Lag distributed income per capita (I$), Log-transformed SEV scalar: Non Ven, Sociodemographic Index, Healthcare Access and Quality Index
Non-venomous animal contact	Data rich	Male	10	Alcohol (liters per capita), Education (years per capita), Elevation Over 1500 m (proportion), Population Density (over 1000 ppl/sqkm, proportion), Population Density (under 150 ppl/sqkm, proportion), Elevation Under 100 m (proportion), Log-transformed SEV scalar: Non Ven, Sociodemographic Index, Healthcare Access and Quality Index, Lag distributed income per capita (I$)
Non-venomous animal contact	Global/Data rich	Female	10	Alcohol (liters per capita), Education (years per capita), Elevation Over 1500 m (proportion), Population Density (over 1000 ppl/sqkm, proportion), Population Density (under 150 ppl/sqkm, proportion), Elevation Under 100 m (proportion), Log-transformed SEV scalar: Non Ven, Sociodemographic Index, Healthcare Access and Quality Index, Lag distributed income per capita (I$)
Foreign body	Global	Male	10	Education (years per capita), Indoor Air Pollution (All Cooking Fuels), Population Density (over 1000 ppl/sqkm, proportion), Population Over 65 (proportion), Sociodemographic Index, Healthcare Access and Quality Index, Lag distributed income per capita (I$)
Foreign body	Global	Female	10	Education (years per capita), Indoor Air Pollution (All Cooking Fuels), Population Density (over 1000 ppl/sqkm, proportion), Population Over 65 (proportion), Sociodemographic Index, Healthcare Access and Quality Index, Lag distributed income per capita (I$)
Pulmonary aspiration and foreign body in airway	Global/Data rich	Male	6	Alcohol (liters per capita), Lag distributed income per capita (I$), Mean BMI, Log-transformed SEV scalar: F Body Aspn, Sociodemographic Index, Access and Quality Index
Pulmonary aspiration and foreign body in airway	Global	Female	8	Alcohol (liters per capita), Education (years per capita), Mean BMI, Alcohol binge drinker proportion, age-standardised, Log-transformed SEV scalar: F Body Aspn, Sociodemographic Index, Healthcare access and quality index, Lag distributed income per capita (I$)
Pulmonary aspiration and foreign body in airway	Data rich	Female	6	Alcohol (liters per capita), Lag distributed income per capita (I$), Mean BMI, Log-transformed SEV scalar: F Body Aspn, Sociodemographic Index, Healthcare Access and Quality Index
Foreign body in other body part	Global/Data rich	Male	10	Alcohol (liters per capita), Education (years per capita), Elevation Over 1500 m (proportion), Population Density (over 1000 ppl/sqkm, proportion), Population Density (under 150 ppl/sqkm, proportion), Elevation Under 100 m (proportion), Log-transformed SEV scalar: Oth F Body, Sociodemographic Index, Healthcare Access and Quality Index, Lag distributed income per capita (I$)
Foreign body in other body part	Global/Data rich	Female	10	Alcohol (liters per capita), Education (years per capita), Elevation Over 1500 m (proportion), Population Density (over 1000 ppl/sqkm, proportion), Population Density (under 150 ppl/sqkm, proportion), Elevation Under 100 m (proportion), Log-transformed SEV scalar: Oth F Body, Sociodemographic Index, Healthcare Access and Quality Index, Lag distributed income per capita (I$)
Environmental heat and cold exposure	Global/Data rich	Male	11	Education (years per capita), Lag distributed income per capita (I$), Population-weighted mean temperature, Elevation Over 1500 m (proportion), Elevation 500 to 1500 m (proportion), Population Density (150–300 ppl/sqkm, proportion), Rainfall (Quintiles 4–5), Sanitation (proportion with access), 90th percentile climatic temperature in the given country-year, Sociodemographic Index, Healthcare Access and Quality Index
Environmental heat and cold exposure	Global/Data rich	Female	11	Education (years per capita), Lag distributed income per capita (I$), Population-weighted mean temperature, Elevation Over 1500 m (proportion), Elevation 500 to 1500 m (proportion), Population Density (150–300 ppl/sqkm, proportion), Rainfall (Quintiles 4–5), Sanitation (proportion with access), 90th percentile climatic temperature in the given country-year, Sociodemographic Index, Healthcare Access and Quality Index
Other unintentional injuries	Global/Data rich	Male	12	Alcohol (liters per capita), Education (years per capita), Elevation Over 1500 m (proportion), Population Density (over 1000 ppl/sqkm, proportion), Population Density (under 150 ppl/sqkm, proportion), Elevation Under 100 m (proportion), Vehicles–two wheels (per capita), Vehicles–four wheels (per capita), Log-transformed SEV scalar: Oth Unint, Sociodemographic Index, Healthcare Access and Quality Index, Lag distributed income per capita (I$)
Other unintentional injuries	Global/Data rich	Female	12	Alcohol (liters per capita), Education (years per capita), Elevation Over 1500 m (proportion), Population Density (over 1000 ppl/sqkm, proportion), Population Density (under 150 ppl/sqkm, proportion), Elevation Under 100 m (proportion), Vehicles–two wheels (per capita), Vehicles–four wheels (per capita), Log-transformed SEV scalar: Oth Unint, Sociodemographic Index, Healthcare Access and Quality Index, Lag distributed income per capita (I$)
Self-harm	Global	Male	11	Alcohol (liters per capita), Education (years per capita), Population Density (150–300 ppl/sqkm, proportion), Population Density (300–500 ppl/sqkm, proportion), Population Density (500–1000 ppl/sqkm, proportion), Population Density (over 1000 ppl/sqkm, proportion), Population Density (under 150 ppl/sqkm, proportion), Sociodemographic Index, Healthcare Access and Quality Index, Muslim Religion (proportion of population), Lag distributed income per capita (I$)
Self-harm	Global	Female	15	Alcohol (liters per capita), Education (years per capita), Population Density (150–300 ppl/sqkm, proportion), Population Density (300–500 ppl/sqkm, proportion), Population Density (500–1000 ppl/sqkm, proportion), Population Density (over 1000 ppl/sqkm, proportion), Population Density (under 150 ppl/sqkm, proportion), Religion (binary,>50% Muslim), Log-transformed SEV scalar: Self Harm, Sociodemographic Index, Major depressive disorder, Risk of selfharm due to major depressive disorder, Healthcare Access and Quality Index, Non-partner lifetime prevalence of sexual violence (female-only), Lag distributed income per capita (I$)
Self-harm	Data rich	Male	11	Alcohol (liters per capita), Education (years per capita), Population Density (150–300 ppl/sqkm, proportion), Population Density (300–500 ppl/sqkm, proportion), Population Density (500–1000 ppl/sqkm, proportion), Population Density (over 1000 ppl/sqkm, proportion), Population Density (under 150 ppl/sqkm, proportion), Religion (binary,>50% Muslim), Sociodemographic Index, Healthcare Access and Quality Index, Lag distributed income per capita (I$)
Self-harm	Data rich	Female	13	Alcohol (liters per capita), Education (years per capita), Population Density (150–300 ppl/sqkm, proportion), Population Density (300–500 ppl/sqkm, proportion), Population Density (500–1000 ppl/sqkm, proportion), Population Density (over 1000 ppl/sqkm, proportion), Population Density (under 150 ppl/sqkm, proportion), Religion (binary,>50% Muslim), Log-transformed SEV scalar: Self Harm, Sociodemographic Index, Major depressive disorder, Healthcare Access and Quality Index, Lag distributed income per capita (I$)
Self-harm by firearm	Global/Data rich	Male	13	Alcohol (liters per capita), Education (years per capita), Population Density (150–300 ppl/sqkm, proportion), Population Density (300–500 ppl/sqkm, proportion), Population Density (500–1000 ppl/sqkm, proportion), Population Density (over 1000 ppl/sqkm, proportion), Population Density (under 150 ppl/sqkm, proportion), Religion (binary,>50% Muslim), Log-transformed SEV scalar: Self Harm, Sociodemographic Index, Major depressive disorder, Healthcare Access and Quality Index, Lag distributed income per capita (I$)
Self-harm by firearm	Global/Data rich	Female	13	Alcohol (liters per capita), Education (years per capita), Population Density (150–300 ppl/sqkm, proportion), Population Density (300–500 ppl/sqkm, proportion), Population Density (500–1000 ppl/sqkm, proportion), Population Density (over 1000 ppl/sqkm, proportion), Population Density (under 150 ppl/sqkm, proportion), Religion (binary,>50% Muslim), Log-transformed SEV scalar: Self Harm, Sociodemographic Index, Major depressive disorder, Healthcare Access and Quality Index, Lag distributed income per capita (I$)
Self-harm by other specified means	Global/Data rich	Male	13	Alcohol (liters per capita), Education (years per capita), Population Density (150–300 ppl/sqkm, proportion), Population Density (300–500 ppl/sqkm, proportion), Population Density (500–1000 ppl/sqkm, proportion), Population Density (over 1000 ppl/sqkm, proportion), Population Density (under 150 ppl/sqkm, proportion), Religion (binary,>50% Muslim), Log-transformed SEV scalar: Self Harm, Sociodemographic Index, Major depressive disorder, Healthcare Access and Quality Index, Lag distributed income per capita (I$)
Self-harm by other specified means	Global/Data rich	Female	13	Alcohol (liters per capita), Education (years per capita), Population Density (150–300 ppl/sqkm, proportion), Population Density (300–500 ppl/sqkm, proportion), Population Density (500–1000 ppl/sqkm, proportion), Population Density (over 1000 ppl/sqkm, proportion), Population Density (under 150 ppl/sqkm, proportion), Religion (binary,>50% Muslim), Log-transformed SEV scalar: Self Harm, Sociodemographic Index, Major depressive disorder, Healthcare Access and Quality Index, Lag distributed income per capita (I$)
Interpersonal violence	Global/Data rich	Male	8	Alcohol (liters per capita), Education (years per capita), Opium Cultivation (binary), Population Density (over 1000 ppl/sqkm, proportion), Log-transformed SEV scalar: Violence, Sociodemographic Index, Healthcare Access and Quality Index, Lag distributed income per capita (I$)
Interpersonal violence	Global/Data rich	Female	8	Alcohol (liters per capita), Education (years per capita), Opium Cultivation (binary), Population Density (over 1000 ppl/sqkm, proportion), Log-transformed SEV scalar: Violence, Sociodemographic Index, Healthcare Access and Quality Index, Lag distributed income per capita (I$)
Physical violence by firearm	Global/Data rich	Male	8	Alcohol (liters per capita), Education (years per capita), Opium Cultivation (binary), Population Density (over 1000 ppl/sqkm, proportion), Log-transformed SEV scalar: Viol Gun, Sociodemographic Index, Healthcare Access and Quality Index, Lag distributed income per capita (I$)
Physical violence by firearm	Global/Data rich	Female	8	Alcohol (liters per capita), Education (years per capita), Opium Cultivation (binary), Population Density (over 1000 ppl/sqkm, proportion), Log-transformed SEV scalar: Viol Gun, Sociodemographic Index, Healthcare Access and Quality Index, Lag distributed income per capita (I$)
Physical violence by sharp object	Global/Data rich	Male	8	Alcohol (liters per capita), Education (years per capita), Opium Cultivation (binary), Population Density (over 1000 ppl/sqkm, proportion), Log-transformed SEV scalar: Viol Knife, Sociodemographic Index, Healthcare Access and Quality Index, Lag distributed income per capita (I$)
Physical violence by sharp object	Global/Data rich	Female	8	Alcohol (liters per capita), Education (years per capita), Opium Cultivation (binary), Population Density (over 1000 ppl/sqkm, proportion), Log-transformed SEV scalar: Viol Knife, Sociodemographic Index, Healthcare Access and Quality Index, Lag distributed income per capita (I$)
Physical violence by other means	Global/Data rich	Male	8	Alcohol (liters per capita), Education (years per capita), Opium Cultivation (binary), Population Density (over 1000 ppl/sqkm, proportion), Log-transformed SEV scalar: Oth Viol, Sociodemographic Index, Healthcare Access and Quality Index, Lag distributed income per capita (I$)
Physical violence by other means	Global/Data rich	Female	8	Alcohol (liters per capita), Education (years per capita), Opium Cultivation (binary), Population Density (over 1000 ppl/sqkm, proportion), Log-transformed SEV scalar: Oth Viol, Sociodemographic Index, Healthcare Access and Quality Index, Lag distributed income per capita (I$)
Executions and police conflict	Global/Data rich	Male	6	Alcohol (liters per capita), Education (years per capita), Lag distributed income per capita (I$), Population Density (over 1000 ppl/sqkm, proportion), Sociodemographic Index, Healthcare Access and Quality Index
Executions and police conflict	Global/Data rich	Female	6	Alcohol (liters per capita), Education (years per capita), Lag distributed income per capita (I$), Population Density (over 1000 ppl/sqkm, proportion), Sociodemographic Index, Healthcare Access and Quality Index

BMI, body mass index.

**Figure 1:**
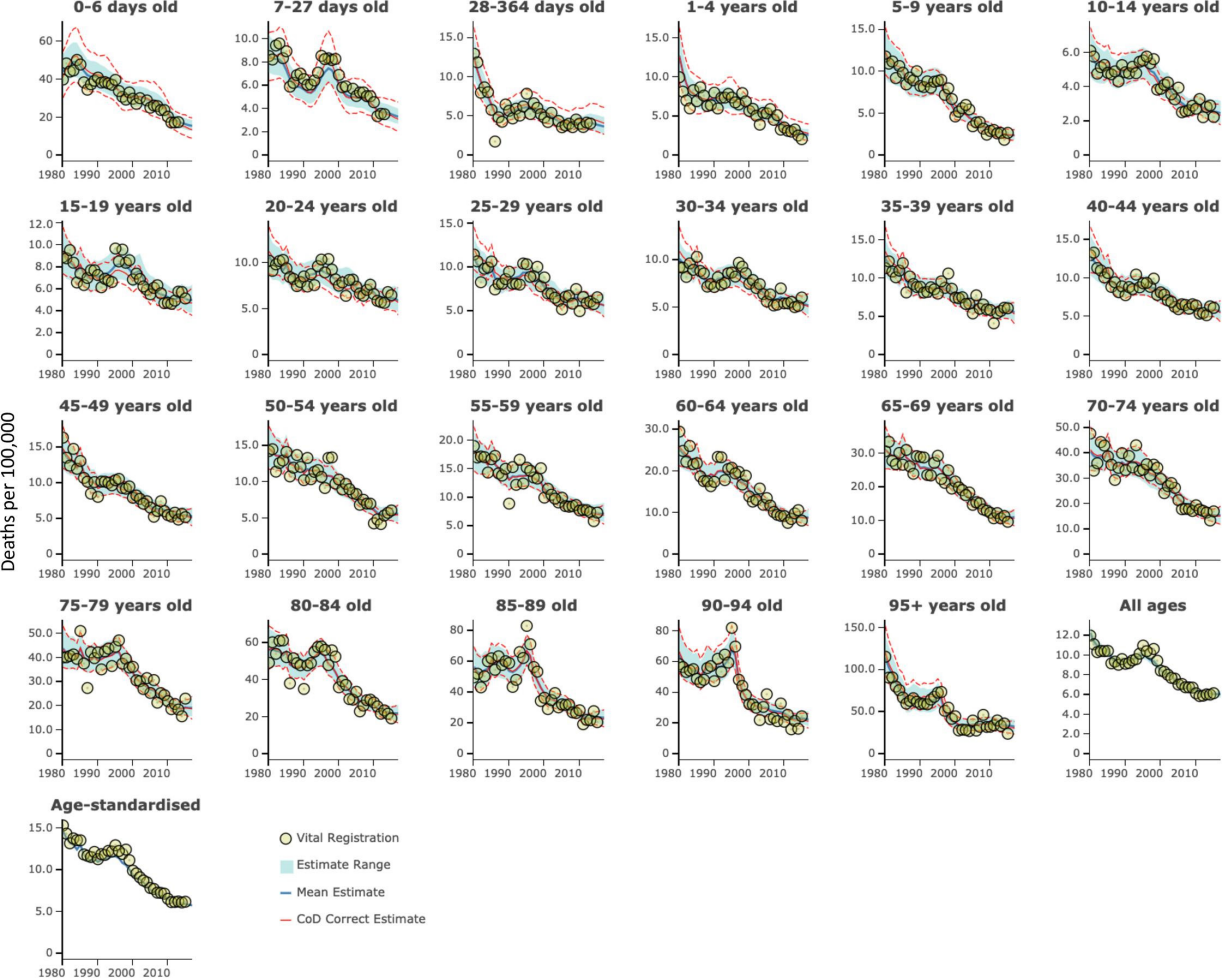
Cause of Death Ensemble model with data points for road injuries in Colombia for females.

**Figure 2:**
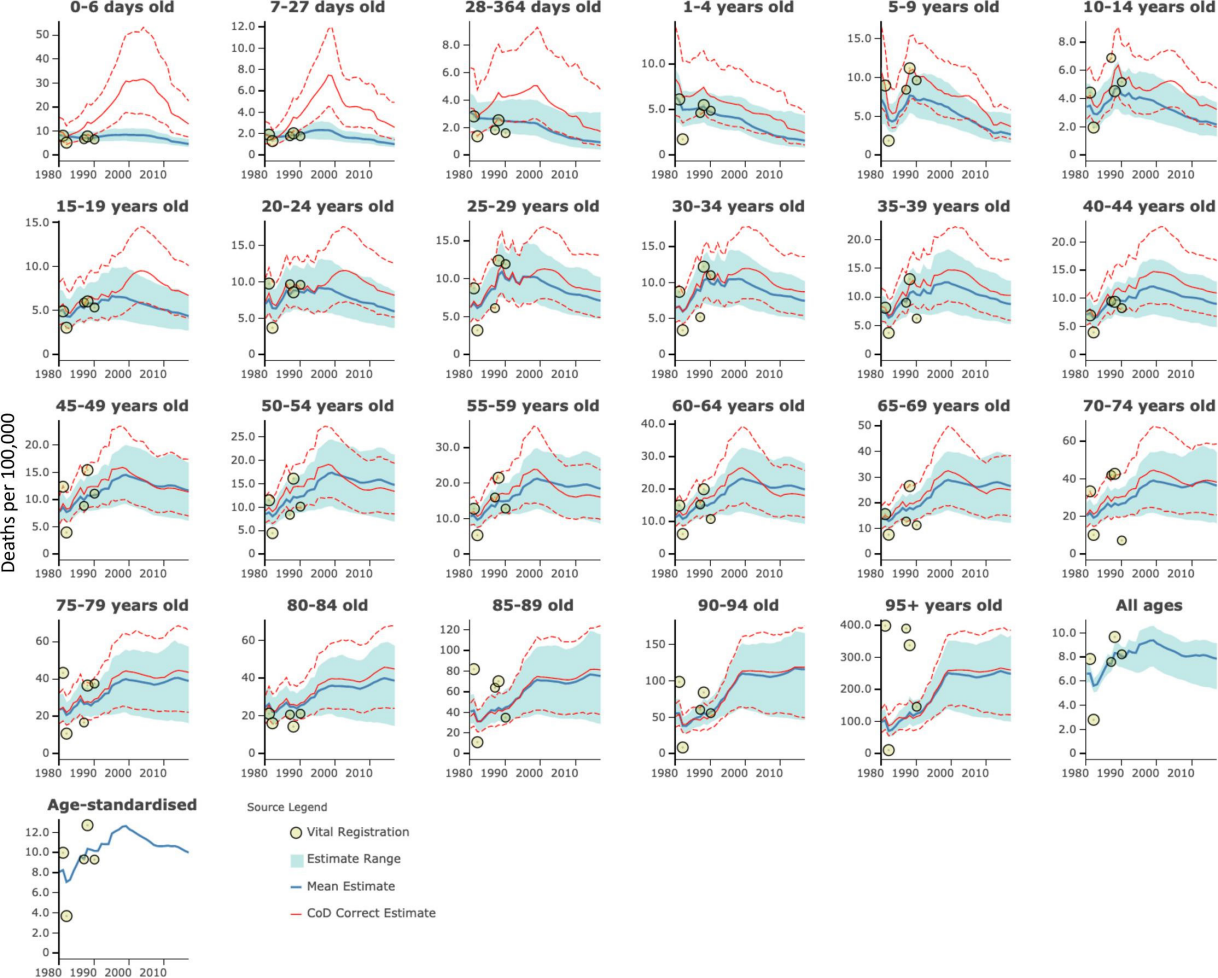
Cause of Death Ensemble model with data points for road injuries in Honduras for females

Once submodels and ensemble models have been conducted for each cause in the GBD cause hierarchy, a process to correct for cause of death rates to ensure internal consistency is conducted. Specifically, each subcause within some overall cause is rescaled such that, for example, every subtype of road injuries sums to road injuries deaths overall, and then road injuries and other transport injuries sum to equal the overall transport injuries cause. As this cascades to the overall cause hierarchy and the overall all-cause mortality rates, cause-specific mortality across all causes ultimately equals the overall mortality in the population. An example of an injuries cause of death model with vital registration data (Colombia, females) is shown in figure 1. A similar model with relatively less data is shown in figure 2 (Honduras, females). While data are absent in more recent years in Honduras, the model is still able to follow temporal trends, age patterns and broader geographical patterns by harnessing signals from covariate-based fixed effects (eg, alcohol consumption per capita) and location-based random effects (eg, the regional trends in Central Latin America and patterns in neighbouring countries). All cause of death models from GBD 2017 are publicly available for review (https://vizhub.healthdata.org/cod/). Cause-specific deaths are converted to cause-specific mortality rates (CSMRs) using GBD populations. Once CSMRs are established, years of life lost (YLLs) are computed as the product of CSMRs and residual life expectancy at the age of death. The residual life expectancy is based on the lowest observed mortality rate for each age across all populations over 5 million. For example, if a death from road injuries occurs at age 25 and the residual life expectancy is 60 years, then there are 60 YLLs attributed to that death. If the death had occurred at age 50 with a residual life expectancy of 38 years, then 38 YLLs would be attributed. Life tables used for GBD 2017 are provided in related publications. 7

### Injury incidence, prevalence and years lived with disability

**Figure 3:**
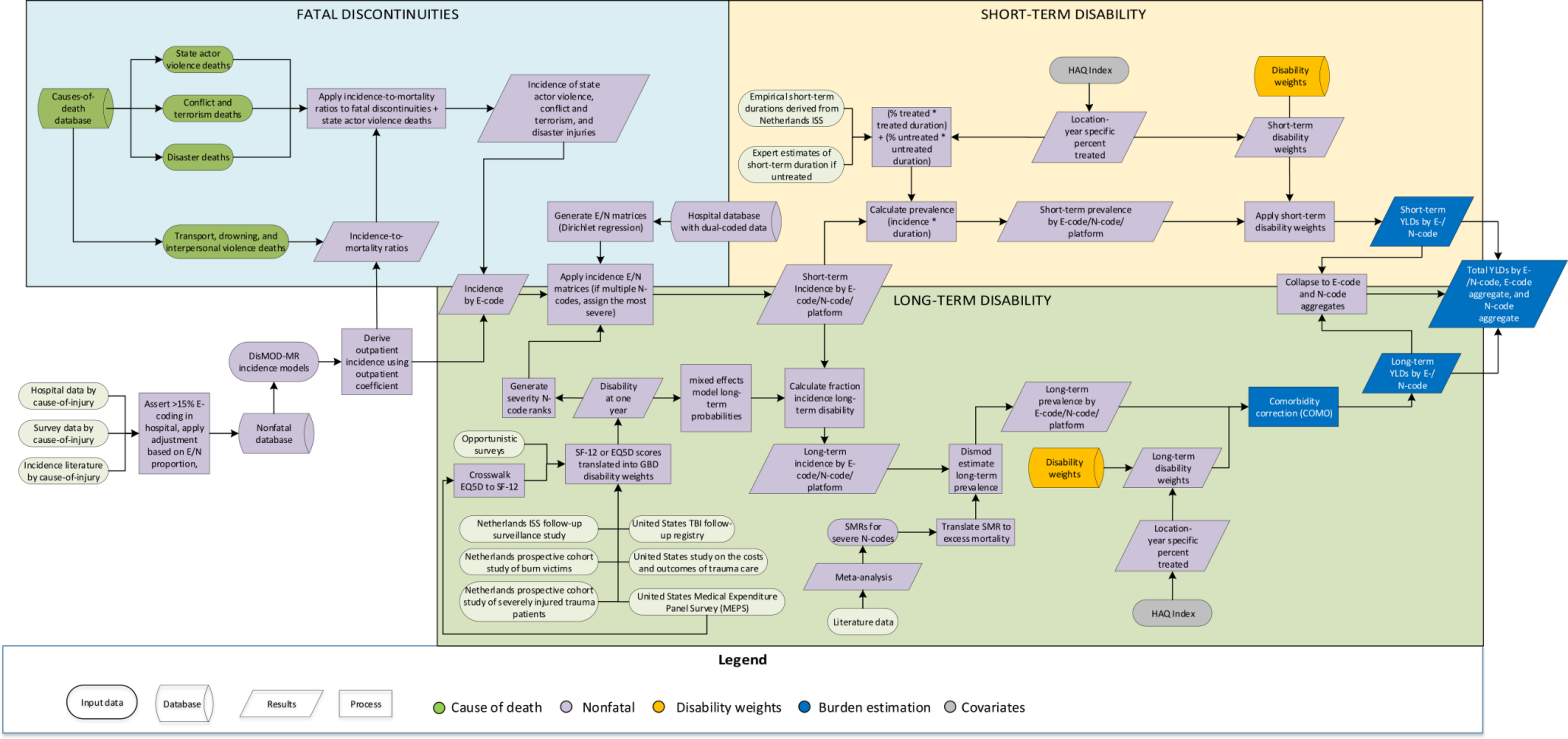
Injuries non-fatal estimation flow chart.

After cause-specific models for each cause of injury in the GBD cause hierarchy are conducted, the non-fatal estimation process is conducted. An overview of this process is depicted in figure 3. In the first stage, we estimate the incidence of injuries warranting medical care using DisMod-MR 2.1 (abbreviated DisMod). DisMod is a meta-regression tool for epidemiological estimation that uses a compartmental model structure whereby a healthy population may become diseased or injured, at which point the individual either remains a prevalent case, goes into remission or dies. DisMod essentially fits differential equations to reconcile the transitions between these different compartments, so that the final posterior estimate for each epidemiological parameter can be explained in the context of the other parameters. Similar to the principles described in CODEm, DisMod uses all available data, ranging from incidence data to cause-specific mortality rates from the corrected CODEm results, to produce estimates for every age, sex, year and location. For the purposes of injuries, we established our case definition for non-fatal injuries as injuries that require medical care. This is a necessary case definition as we do not want to consider minor stumbles and falls, for example, that led to no actual bodily harm as injuries for GBD, since they would not have any associated disability. These models are conducted only for injury *causes* as opposed to the nature of injuries references above. Each data input is designated based on type of data—specifically, inpatient data, outpatient data, surveillance data, survey data and literature studies that are population-representative. We model incidence rates for hospital admissions for injuries, so the non-inpatient data sources get adjusted according to their classification so that the model inputs are consistent as injuries that warranted or received inpatient medical care. The coefficients measured by DisMod that were used for adjustment are provided in table 5. Input data for injury cause incidence models included sources identified as part of systematic reviews conducted in past GBD cycles, new sources identified by the GBD collaborator network and new sources of clinical data and other injuries data obtained by the core injuries burden estimation team at the Institute for Health Metrics and Evaluation at the University of Washington. In addition, CSMRs from the corrected CODEm models described above are used in this stage of DisMod modelling. The list of non-fatal injury sources used in GBD 2017 is provided in online supplementary appendix table 2. The completed DisMod models for inpatient incidence for each cause of injury are publicly available at https://vizhub.healthdata.org/epi/.

10.1136/injuryprev-2019-043531.supp3 Supplementary data

**Table 5:** Covariates and coefficients used in Global Burden of Disease incidence cause models

**Cause**	**Outpatient coefficient**	**Injury receiving formal care, inpatient and outpatient coefficient**	**Injury warranting medical care coefficient**
Animal contact	7.04 (7.03–7.04)	7.56 (6.91–8.31)	
Non-venomous animal contact	2.91 (2.91–2.92)	11.21 (10.1–12.38)	
Venomous animal contact	3.14 (3.01–3.34)	4.09 (3.69–4.5)	
Drowning	0.88 (0.87–0.89)	1.01 (1.0–1.05)	30.42 (15.33–51.11)
Falls	6.91 (6.89–6.94)	5.94 (5.5–6.46)	9.73 (9.28–10.22)
Fire, heat and hot substances	3.53 (3.53–3.56)	7.82 (7.24–8.51)	
Pulmonary aspiration and foreign body in airway	3.37 (3.35–3.43)	15.36 (13.93–16.86)	
Foreign body in eyes	931.4 (923.34–934.49)	302.06 (251.14–365.04)	
Foreign body in other body part	1.97 (1.95–2.01)	20.97 (15.55–26.26)	
Interpersonal violence	6.57 (6.56–6.61)	21.43 (13.6–32.79)	46.97 (39.57–53.62)
Assault by firearm	1.36 (1.29–1.44)	1.27 (1.05–1.6)	53.58 (50.65–54.54)
Assault by sharp object	3.18 (2.92–3.5)	2.38 (1.86–3.22)	37.91 (28.3–50.05)
Assault by other means	5.65 (5.44–5.89)	2.44 (2.02–3.2)	
Exposure to mechanical forces	12.4 (12.0–12.82)	33.3 (30.51–36.23)	
Unintentional firearm injuries	2.71 (2.53–2.9)	4.6 (3.49–6.36)	
Other exposure to mechanical forces	12.62 (12.55–12.85)	30.77 (25.74–36.09)	
Adverse effects of medical treatment	1.06 (1.06–1.06)	19.81 (17.29–26.1)	
Environmental heat and cold exposure	3.91 (3.9–3.94)		17.54 (3.91–49.6)
Other unintentional injuries	13.53 (13.46–13.78)		14.95 (9.62–24.12)
Poisonings	3.96 (3.73–4.19)	3.78 (3.4–4.21)	8.47 (4.41–16.64)
Poisoning by carbon monoxide	5.86 (5.68–5.92)		
Poisoning by other means	4.18 (3.9–4.5)		
Self-harm	2.75 (2.75–2.78)	2.5 (2.2–2.83)	
Self-harm by firearm	2.77 (2.42–3.07)	16.94 (2.81–51.06)	
Self-harm by other specified means	1.5 (1.47–1.51)	6.73 (2.78–19.14)	
Other transport injuries	1.65 (1.6–1.77)	1.01 (1.0–1.03)	
Road injuries	3.77 (3.75–3.78)	6.16 (5.65–6.68)	15.44 (13.25–18.1)
Motorcyclist road injuries	1.94 (1.92–1.99)		
Motor vehicle road injuries	4.48 (4.46–4.48)		
Other road injuries	6.9 (6.89–6.96)		
Cyclist road injuries	4.54 (4.33–4.89)		
Pedestrian road injuries	1.94 (1.94–1.96)	15.78 (7.63–36.6)	

Once an incidence cause model is constructed for each cause of injury, an extensive analytical ‘pipeline’ follows which converts injury cause incidence into years lived with disability. First, inpatient incidence is split into inpatient and outpatient incidence using coefficients empirically measured by DisMod. The outpatient coefficients for each injury cause are also included in table 5. Separate pipelines are then conducted for inpatient and outpatient injury incidence—each step below can be considered to have been run for both streams of data, for each cause of injury. After the coefficient is applied, incidence is adjusted by the excess mortality rate measured by DisMod to essentially remove injury cases that died after the injury occurred. Once these deaths are removed from the incidence pool, the resulting steps are applied to these surviving cases of injury. First, each new case of injury is considered to have 47 possible ‘natures’ of injury that can result. These are the types of bodily injury that are considered to be possible outcomes from a given injury cause. The proportion of new cases of injury that would have some nature of injury as the most disabling outcome is determined based on dual-coded clinical data sources where both the cause and nature of injury were included as ICD codes.^10^ Of note, one limitation of this process is that due to computational demands, it is currently only possible to apportion the most disabling nature of injury for each new case of injury. As such, the probability that each nature of injury is the most disabling nature of injury for some cause of injury is modelled in a Dirichlet regression such that the probabilities sum to 1. In other words, each nature of injury has some probability of being the most disabling injury suffered by the victim of some cause of injury, but if multiple natures of injury occurred, then the less disabling injuries are not captured as part of that injury cause’s disability. This limitation has been recognised as a limitation of GBD injury burden estimation in various peer-reviewed articles and will likely be addressed in future GBD updates as computational efficiency improves.^3 10^

The probability distributions of each cause-nature are computed separately for each age, sex, year and location. At this point, the analytical stage has the age-specific, sex-specific, year-specific, location-specific incidence of a cause-nature combination, for example, the incidence of road injuries that led to a cervical-level spinal cord injury in males aged 20–24 years in 2017 in Stockholm, Sweden. The next step converts these incidence estimates into short-term and long-term injury incidence estimates, where long-term disability is defined as having a lower functional status 1 year postinjury than at the time of injury. These probabilities were measured using long-term follow-up studies.^14–20^ For some natures of injury, such as lower extremity amputation, the probability of being a long-term injury is 1. The probabilities of short-term versus long-term injury for each cause-nature combination are used to split the incidence values into short-term and long-term pipelines. The long-term incidence is then converted to prevalence using the ordinary differential equation solver used in DisMod, which also uses as an input excess mortality estimated for certain natures of injury such as traumatic brain injury and spinal cord injury conducted in a previous systematic review and meta-analysis. The short-term incidence is converted to prevalence by multiplying incidence and duration of injury, where duration of injury was either computed directly from follow-up studies or, in the case of unavailable data, estimated by an expert clinical panel involved in previous iterations of the GBD study. Since access to medical treatment is assumed to affect duration of injury and disability, the GBD Healthcare Access and Quality Index is used to estimate the proportion with and without access to medical treatment on a location-specific basis.^21^ The average duration for short-term injury is therefore calculated as the percentage treated multiplied by treated duration added to the percentage untreated multiplied by the untreated duration. The output from this step is the short-term prevalence of each cause-nature combination. Short-term prevalence is subtracted from long-term prevalence at this stage to avoid double counting the same case of injury. Once short-term and long-term prevalence estimates for each cause-nature are computed, then disability weights as derived by the Salomon *et al* process are assigned to each injury nature.^22^ Short-term disability weights by injury nature are shown in table 6, which does not include amputations since we assume they cause only long-term disability. The full list of long-term disability weights by injury nature, location and year are provided in online supplementary appendix table 3, which does not include foreign body in respiratory system, foreign body in gastrointestinal and urogenital system, foreign body in ear and superficial injury of any part of body, since we assume these natures of injury do not cause long-term disability. After disability weights are assigned to each injury case, years lived with disability for each cause of injury are calculated as the prevalence of each health state multiplied by the corresponding disability weight and then summed across natures of injury for each cause to compute years lived with disability (YLDs) for each age, sex, year and location for that injury cause. YLDs then undergo comorbidity adjustment used across the GBD study whereby comorbid cases of disease and injury in the population are simulated and adjusted disability weights are computed. These processes are described in more detail in GBD literature.^3^ GBD 2017 provided an important methodological update whereby nature of injury results, regardless of cause of injury, could be reviewed in the results from this process; this has enabled more advanced GBD research such as measuring the burden of traumatic brain injury and spinal cord injury, measuring the burden of facial fractures and measuring the burden of hand and finger fractures.^10^

10.1136/injuryprev-2019-043531.supp4 Supplementary data

**Table 6:** Short-term disability weights for each nature of injury

Nature of injury	Short-term disability weight
Spinal cord lesion at neck level	0.7319
Spinal cord lesion below neck level	0.6235
Foreign body in respiratory system	0.4079
Lower airway burns	0.3764
Severe chest Injury	0.3685
Internal haemorrhage in abdomen and pelvis	0.3242
Burns, ≥20% total burned surface area or ≥10% burned surface area if head/neck or hands/wrist involved without lower airway burns	0.3145
Fracture of pelvis	0.2788
Fracture of hip	0.2575
Multiple fractures, dislocations, crashes, wounds, sprains and strains	0.2575
Drowning and non-fatal submersion	0.2471
Asphyxiation	0.2471
Moderate TBI	0.2137
Poisoning requiring urgent care	0.1628
Burns, <20% total burned surface area without lower airway burns	0.1408
Effect of different environmental factors	0.1334
Complications following therapeutic procedures	0.1334
Crush injury	0.1325
Foreign body in GI and urogenital system	0.1143
Dislocation of knee	0.1134
Fracture of femur, other than femoral neck	0.1114
Fracture of vertebral column	0.1106
Minor TBI	0.11
Fracture of sternum and/or fracture of one or more ribs	0.1027
Nerve injury	0.0997
Fracture of skull	0.0714
Fracture of face bones	0.0669
Dislocation of shoulder	0.062
Injury to eyes	0.0543
Fracture of patella, tibia or fibula or ankle	0.0501
Fracture of clavicle, scapula or humerus	0.0349
Fracture of radius and/or ulna	0.0281
Fracture of foot bones except ankle	0.026
Dislocation of hip	0.0159
Foreign body in ear	0.0133
Fracture of hand (wrist and other distal part of hand)	0.0099
Muscle and tendon injuries, including sprains and strains lesser dislocations	0.0075
Contusion in any part of the body	0.0075
Superficial injury of any part of the body	0.0075
Open wound(s)	0.0058

GI, gastrointestinal; TBI, traumatic brain injury.

### Sexual violence

**Figure 4:**
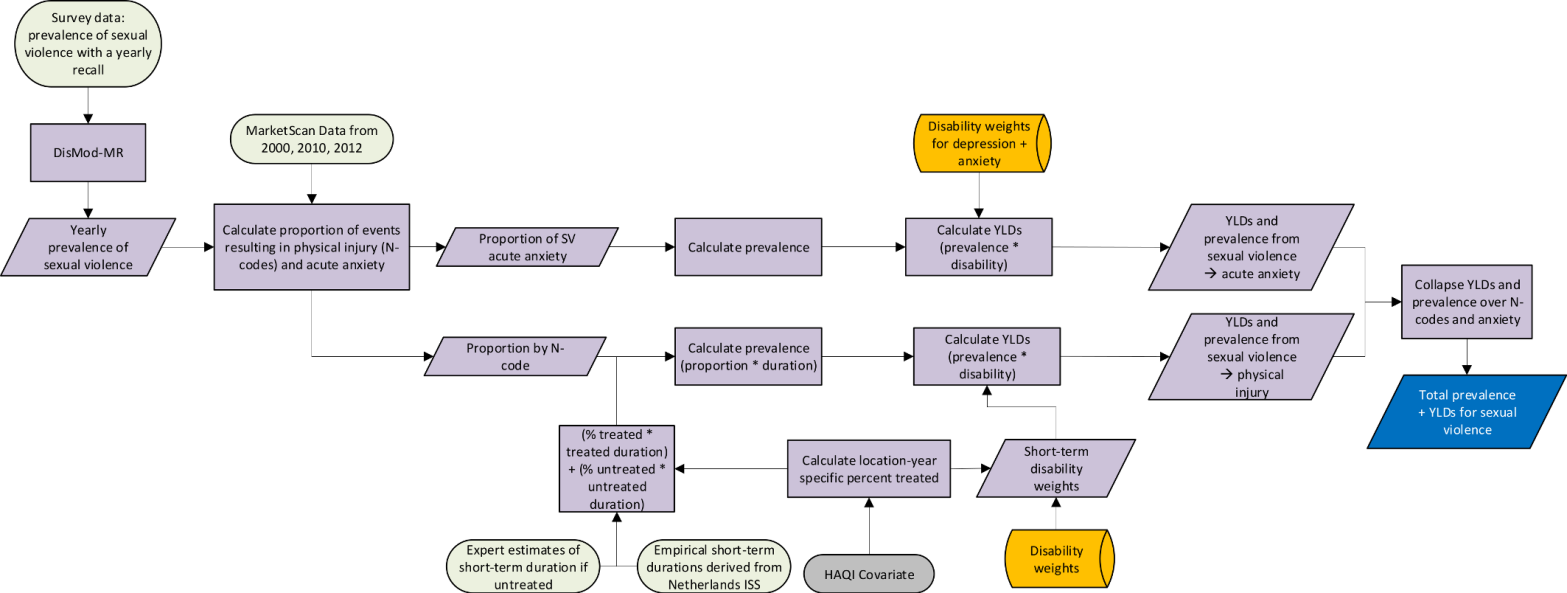
Sexual violence estimation flow chart. HAQI, Healthcare Access and Quality Index.

Sexual violence follows a different analytical pathway than the other causes of injury. This process is shown in figure 4. We used the same study framework as was developed for other injury rates in the GBD 2017 study to estimate the yearly proportion of the population that experienced at least one episode of sexual violence in the past year, using a case definition of any sexual assault including penetrative sexual violence (rape) and non-penetrative sexual violence (other forms of unwanted sexual touching). To inform the sexual violence estimates, we identified data in 93 countries that met the case definition above. This resulted in 263 site-years of data, which mainly were derived from surveys such as Demographic and Health Surveys and Reproductive Health Surveys. Similar to our other injury models, we used DisMod 2.1 to model prevalence. The sexual violence prevalence model used study-level covariates for each type of survey question, for example, we used a study-level covariate to identify surveys that identify penetrative sexual violence only to account for how the overall incidence of sexual violence is greater than this value. This model also used a covariate on alcohol use in litres per capita for each location to help fit the model in data-sparse locations. Once yearly prevalence was measured, sexual violence cases undergo a process by which short-term disability from the physical and psychological harm of sexual violence cases is assigned to each prevalent case; however, long-term sequelae of sexual violence are currently not captured in this process, which has been a known limitation of sexual violence estimation in the GBD framework.

### Disability-adjusted life-years

After estimation of cause-specific mortality and YLLs as well as non-fatal health outcomes estimation including YLDs, DALYs are calculated as the sum of YLLs and YLDs for each cause of injury. YLDs are also calculated for each nature of injury category.

### GATHER statement

GBD 2017 adheres to the Guidelines for Accurate and Transparent Health Estimates Reporting (GATHER). GATHER is described in more detail in online supplementary appendix 2.

10.1136/injuryprev-2019-043531.supp5 Supplementary data

## RESULTS

Results for all GBD 2017 injury estimates are available in associated publications as well as online. Specifically, results by age, sex, year, subnational location and nature of injury can be viewed and downloaded online via the GBD Results Tool (http://ghdx.healthdata.org/gbd-results-tool) and GBD Compare (https://vizhub.healthdata.org/gbd-compare/). These results are available in terms of incidence, prevalence, YLDs, cause-specific mortality, YLLs and DALYs, expressed in counts, rates, and percentages. Analytical code and input datasets are available at http://ghdx.healthdata.org.

### CODEm models

Model performance metrics for each injury cause model in GBD 2017 are provided in table 7. Model performance metrics for CODEm models include root mean square error (RMSE) for in-sample tests and out-of-sample tests, percentage of data points that correctly predict the trend in-sample and out-of-sample and percentage of data points that are present within the 95% uncertainty intervals (UIs) of the model fit. RMSE in-sample is generally better than RMSE out-of-sample, which is an expected result that also demonstrates the importance of performing out-of-sample predictive validity tests. While the correct trend is predicted in approximately one in five models, this may also be related to more dynamic temporal trends in injury mortality patterns over time. In general, most data points exist within the 95% UI of the model fit (mean: 98.5% in-sample, 98.0% out-of-sample).

**Table 7:** Performance metrics for each cause-of-injury CODEm model

Cause	Type	Sex	RMSE in-sample	RMSE out-of-sample	Per cent coverage in-sample	Per cent coverage out-of-sample
Transport injuries	Data rich	Female	0.153062	0.211028	0.999851	0.999395
Transport injuries	Data rich	Male	0.144423	0.202366	0.99978	0.998995
Transport injuries	Global	Female	0.216405	0.338398	0.99951	0.992996
Transport injuries	Global	Male	0.209561	0.327954	0.999347	0.99108
Road injuries	Data rich	Female	0.154916	0.22011	0.999945	0.999642
Road injuries	Data rich	Male	0.147432	0.208989	0.99987	0.999452
Road injuries	Global	Female	0.198002	0.338885	0.999736	0.993674
Road injuries	Global	Male	0.193896	0.321219	0.999332	0.990834
Pedestrian road injuries	Data rich	Female	0.183693	0.327964	0.999776	0.998965
Pedestrian road injuries	Data rich	Male	0.177994	0.323544	0.999688	0.998913
Pedestrian road injuries	Global	Female	0.240151	0.430127	0.999174	0.992328
Pedestrian road injuries	Global	Male	0.247329	0.409191	0.998229	0.990017
Cyclist road injuries	Data rich	Female	0.219965	0.435983	0.999892	0.999106
Cyclist road injuries	Data rich	Male	0.206919	0.500591	0.999876	0.999158
Cyclist road injuries	Global	Female	0.296895	0.528063	0.998384	0.990875
Cyclist road injuries	Global	Male	0.294776	0.527441	0.998702	0.988234
Motorcyclist road injuries	Data rich	Female	0.268406	0.653692	0.999776	0.998805
Motorcyclist road injuries	Data rich	Male	0.195368	0.444714	0.999793	0.998395
Motorcyclist road injuries	Global	Female	0.362655	0.692762	0.998726	0.99082
Motorcyclist road injuries	Global	Male	0.283024	0.502588	0.998804	0.987794
Motor vehicle road injuries	Data rich	Female	0.167766	0.33083	0.99993	0.999335
Motor vehicle road injuries	Data rich	Male	0.160584	0.309726	0.999919	0.999377
Motor vehicle road injuries	Global	Female	0.230946	0.38664	0.99957	0.995355
Motor vehicle road injuries	Global	Male	0.232898	0.378096	0.999353	0.992869
Other road injuries	Data rich	Female	0.408852	1.04171	0.997205	0.970506
Other road injuries	Data rich	Male	0.467256	1.21047	0.994429	0.9463
Other road injuries	Global	Female	0.558784	0.899497	0.994899	0.96375
Other road injuries	Global	Male	0.654189	1.0708	0.984753	0.931697
Other transport injuries	Data rich	Female	0.255843	0.406371	0.999581	0.998655
Other transport injuries	Data rich	Male	0.195575	0.404214	0.999666	0.99863
Other transport injuries	Global	Female	0.31846	0.546918	0.998599	0.991384
Other transport injuries	Global	Male	0.267514	0.49731	0.998444	0.989304
Falls	Data rich	Female	0.162773	0.237492	0.999873	0.999522
Falls	Data rich	Male	0.157114	0.220452	0.999847	0.999492
Falls	Global	Female	0.246877	0.428822	0.99923	0.988577
Falls	Global	Male	0.246101	0.369118	0.999571	0.989585
Drowning	Data rich	Female	0.177905	0.258172	0.999932	0.999782
Drowning	Data rich	Male	0.164617	0.226899	0.999868	0.999373
Drowning	Global	Female	0.238598	0.428467	0.999657	0.992777
Drowning	Global	Male	0.224438	0.361879	0.99961	0.989534
Fire, heat and hot substances	Data rich	Female	0.175426	0.245	0.999962	0.999793
Fire, heat and hot substances	Data rich	Male	0.17054	0.227618	0.999944	0.999737
Fire, heat and hot substances	Global	Female	0.281428	0.401798	0.999483	0.994548
Fire, heat and hot substances	Global	Male	0.289708	0.40982	0.999518	0.99422
Poisonings	Data rich	Female	0.190498	0.283924	0.999901	0.999732
Poisonings	Data rich	Male	0.189747	0.283639	0.999888	0.999668
Poisonings	Global	Female	0.311328	0.515718	0.99918	0.993385
Poisonings	Global	Male	0.323815	0.529806	0.999166	0.992089
Poisoning by carbon monoxide	Data rich	Female	0.255034	0.352342	0.999119	0.998139
Poisoning by carbon monoxide	Data rich	Male	0.234913	0.328692	0.999486	0.998765
Poisoning by carbon monoxide	Global	Female	0.353393	0.688269	0.998372	0.982832
Poisoning by carbon monoxide	Global	Male	0.305615	0.621778	0.999006	0.983458
Poisoning by other means	Data rich	Female	0.208468	0.470199	0.999861	0.998144
Poisoning by other means	Data rich	Male	0.231395	0.543185	0.999871	0.998948
Poisoning by other means	Global	Female	0.284383	0.555132	0.999746	0.989287
Poisoning by other means	Global	Male	0.288098	0.590913	0.999759	0.990146
Exposure to mechanical forces	Data rich	Female	0.171902	0.29354	0.999636	0.99932
Exposure to mechanical forces	Data rich	Male	0.162641	0.259268	0.999605	0.998955
Exposure to mechanical forces	Global	Female	0.398855	0.54379	0.995672	0.987855
Exposure to mechanical forces	Global	Male	0.325975	0.454021	0.995758	0.985214
Unintentional firearm injuries	Data rich	Female	0.207177	0.502831	0.999619	0.999488
Unintentional firearm injuries	Data rich	Male	0.221533	0.49235	0.999306	0.998449
Unintentional firearm injuries	Global	Female	0.354152	0.591674	0.998979	0.991558
Unintentional firearm injuries	Global	Male	0.355798	0.64953	0.996524	0.980841
Other exposure to mechanical forces	Data rich	Female	0.20287	0.436518	0.999912	0.999795
Other exposure to mechanical forces	Data rich	Male	0.170292	0.318704	0.999896	0.999761
Other exposure to mechanical forces	Global	Female	0.406425	0.538089	0.995379	0.98994
Other exposure to mechanical forces	Global	Male	0.361646	0.472713	0.995528	0.988955
Adverse effects of medical treatment	Data rich	Female	0.186809	0.305147	0.999832	0.999511
Adverse effects of medical treatment	Data rich	Male	0.217278	0.342415	0.999833	0.999577
Adverse effects of medical treatment	Global	Female	0.280204	0.430453	0.999698	0.993818
Adverse effects of medical treatment	Global	Male	0.277028	0.431272	0.999573	0.992957
Animal contact	Data rich	Female	0.277226	0.439671	0.999355	0.998642
Animal contact	Data rich	Male	0.231627	0.414921	0.999863	0.999528
Animal contact	Global	Female	0.401714	0.691306	0.998669	0.987713
Animal contact	Global	Male	0.316647	0.623446	0.9991	0.99176
Venomous animal contact	Data rich	Female	0.417726	0.745234	0.960501	0.956152
Venomous animal contact	Data rich	Male	0.401006	0.761481	0.977149	0.97478
Venomous animal contact	Global	Female	0.634642	0.915323	0.965066	0.949503
Venomous animal contact	Global	Male	0.449848	0.839185	0.97819	0.96024
Non-venomous animal contact	Data rich	Female	0.304776	0.593881	0.994547	0.991865
Non-venomous animal contact	Data rich	Male	0.304223	0.529077	0.998929	0.998113
Non-venomous animal contact	Global	Female	0.421204	0.680417	0.995082	0.9848
Non-venomous animal contact	Global	Male	0.471148	0.740524	0.998707	0.990622
Foreign body	Data rich	Female	0.170699	0.275966	0.999937	0.999705
Foreign body	Data rich	Male	0.166161	0.263143	0.999798	0.999305
Foreign body	Global	Female	0.216832	0.401408	0.999535	0.992467
Foreign body	Global	Male	0.227414	0.381598	0.999262	0.989838
Pulmonary aspiration and foreign body in airway	Data rich	Female	0.174424	0.374749	0.999979	0.999572
Pulmonary aspiration and foreign body in airway	Data rich	Male	0.178947	0.34741	0.999928	0.999294
Pulmonary aspiration and foreign body in airway	Global	Female	0.267697	0.416038	0.999413	0.993624
Pulmonary aspiration and foreign body in airway	Global	Male	0.286472	0.422915	0.998089	0.990215
Foreign body in other body part	Data rich	Female	0.31229	0.664465	0.99005	0.987846
Foreign body in other body part	Data rich	Male	0.291172	0.629172	0.993547	0.991666
Foreign body in other body part	Global	Female	0.462299	0.749894	0.98392	0.971743
Foreign body in other body part	Global	Male	0.478614	0.759133	0.984301	0.971436
Other unintentional injuries	Data rich	Female	0.266367	0.450437	0.999612	0.999067
Other unintentional injuries	Data rich	Male	0.228051	0.387409	0.999597	0.998959
Other unintentional injuries	Global	Female	0.354782	0.671813	0.997343	0.984969
Other unintentional injuries	Global	Male	0.301256	0.54085	0.997963	0.985982
Self-harm	Data rich	Female	0.157456	0.236415	0.999699	0.999206
Self-harm	Data rich	Male	0.150967	0.223371	0.999688	0.999011
Self-harm	Global	Female	0.219988	0.370761	0.998551	0.986222
Self-harm	Global	Male	0.203341	0.347213	0.999389	0.979274
Self-harm by firearm	Data rich	Female	0.215778	0.439608	0.992476	0.992525
Self-harm by firearm	Data rich	Male	0.19323	0.402898	0.998082	0.997457
Self-harm by firearm	Global	Female	0.311061	0.642889	0.987894	0.971118
Self-harm by firearm	Global	Male	0.316945	0.590367	0.992646	0.977377
Self-harm by other specified means	Data rich	Female	0.162023	0.345661	0.999855	0.998854
Self-harm by other specified means	Data rich	Male	0.235129	0.322581	0.999898	0.999453
Self-harm by other specified means	Global	Female	0.191636	0.38357	0.999636	0.98601
Self-harm by other specified means	Global	Male	0.192311	0.348953	0.999813	0.986603
Interpersonal violence	Data rich	Female	0.224081	0.294307	0.99863	0.996721
Interpersonal violence	Data rich	Male	0.220852	0.298197	0.998132	0.995665
Interpersonal violence	Global	Female	0.306086	0.450697	0.998456	0.989396
Interpersonal violence	Global	Male	0.307439	0.479452	0.997588	0.981596
Physical violence by firearm	Data rich	Female	0.253283	0.414003	0.998598	0.997318
Physical violence by firearm	Data rich	Male	0.277353	0.501753	0.997843	0.996142
Physical violence by firearm	Global	Female	0.44617	0.621002	0.993619	0.98712
Physical violence by firearm	Global	Male	0.41286	0.679294	0.995867	0.981991
Physical violence by sharp object	Data rich	Female	0.222036	0.393235	0.999815	0.999003
Physical violence by sharp object	Data rich	Male	0.235542	0.463121	0.999796	0.998721
Physical violence by sharp object	Global	Female	0.276474	0.499795	0.999526	0.993622
Physical violence by sharp object	Global	Male	0.332336	0.595217	0.999354	0.990212
Physical violence by other means	Data rich	Female	0.204351	0.336239	0.999954	0.999532
Physical violence by other means	Data rich	Male	0.202192	0.394188	0.999868	0.999051
Physical violence by other means	Global	Female	0.270287	0.410186	0.999719	0.995718
Physical violence by other means	Global	Male	0.285589	0.45387	0.999612	0.992595
Environmental heat and cold exposure	Data rich	Female	0.234754	0.399463	0.999403	0.999073
Environmental heat and cold exposure	Data rich	Male	0.201821	0.309939	0.999658	0.999207
Environmental heat and cold exposure	Global	Female	0.3511	0.639869	0.998595	0.989061
Environmental heat and cold exposure	Global	Male	0.33441	0.528137	0.999336	0.993068
Executions and police conflict	Data rich	Female	0.852242	1.4431	0.49803	0.533053
Executions and police conflict	Data rich	Male	0.970597	1.55607	0.629313	0.628953
Executions and police conflict	Global	Female	1.2422	1.86518	0.541687	0.549016
Executions and police conflict	Global	Male	1.04755	1.95756	0.671496	0.659889

CODEm, Cause of Death Ensemble model.

### Incidence models

Model performance metrics for each injury cause model in GBD 2017 are provided in table 8. These model performance metrics include in-sample coverage and RMSE of estimated results for cause-specific mortality, excess mortality and incidence. There are no performance metrics for CSMR or excess mortality for foreign body in eyes since we do not estimate mortality from this cause of injury. For incidence, the in-sample coverage average was 55.3% across cause-of-injury models and ranged from a low of 26% in falls to a high of 88% in poisoning by carbon monoxide. Incidence RMSE ranged from a low of 1.04 in pedestrian road injuries to a high of 4.86 in foreign body in eye.

**Table 8:** Performance metrics for each cause-of-injury DisMod model

Cause	Cause-specific mortality rate: in-sample coverage	Cause-specific mortality rate: in-sample RMSE	Excess mortality rate: in-sample coverage	Excess mortality rate: in-sample RMSE	Incidence hazard: in-sample coverage	Incidence hazard: in-sample RMSE
Animal contact	0.95	0.96	0.69	1.14	0.40	1.64
Non-venomous animal contact	0.97	0.98	0.74	1.20	0.53	1.40
Venomous animal contact	0.97	1.13	0.74	1.17	0.48	1.31
Drowning	0.91	0.82	0.84	1.40	0.73	1.61
Falls	0.93	0.66	0.71	1.13	0.26	1.77
Fire, heat and hot substances	0.95	0.59	0.67	0.97	0.50	1.16
Pulmonary aspiration and foreign body in airway	0.92	0.93	0.78	1.29	0.65	1.56
Foreign body in eyes					0.83	4.86
Foreign body in other body part	0.96	1.40	0.74	1.31	0.57	1.39
Interpersonal violence	0.89	0.81	0.64	1.11	0.31	1.77
Assault by firearm	0.93	1.96	0.74	1.07	0.69	1.25
Assault by sharp object	0.92	1.50	0.78	1.05	0.57	1.17
Assault by other means	0.90	0.91	0.75	1.10	0.48	1.33
Exposure to mechanical forces	0.92	0.81	0.61	1.23	0.38	2.01
Unintentional firearm injuries	0.95	1.51	0.75	1.13	0.70	1.17
Other exposure to mechanical forces	0.93	0.84	0.66	1.22	0.41	1.94
Adverse effects of medical treatment	0.92	0.71	0.71	1.48	0.37	1.41
Environmental heat and cold exposure	0.94	1.21	0.73	1.54	0.56	1.52
Other unintentional injuries	0.89	1.31	0.51	1.35	0.50	1.67
Poisonings	0.95	0.90	0.76	1.75	0.58	1.90
Poisoning by carbon monoxide	0.95	0.94	0.81	1.11	0.88	1.17
Poisoning by other means	0.95	0.92	0.79	1.89	0.67	2.04
Self-harm	0.98	0.27	0.76	1.02	0.47	1.32
Self-harm by firearm	1.00	1.28	0.89	1.31	0.86	1.35
Self-harm by other specified means	0.98	0.26	0.83	0.96	0.60	1.06
Other transport injuries	0.96	0.99	0.73	1.43	0.63	1.32
Road injuries	0.91	0.47	0.63	1.10	0.27	1.43
Motorcyclist road injuries	0.96	1.07	0.70	1.13	0.54	1.18
Motor vehicle road injuries	0.94	0.55	0.59	1.12	0.48	1.21
Other road injuries	0.99	1.45	0.78	1.16	0.74	1.19
Cyclist road injuries	0.99	1.13	0.73	1.10	0.59	1.09
Pedestrian road injuries	0.92	0.72	0.62	1.02	0.48	1.04

RMSE, root mean square error.

## DISCUSSION

Many considerable advancements have been made in the measurement of global injury burden since early versions of the GBD Study. Novel datasets, sophisticated statistical modelling and global collaboration have all facilitated the advancement of injury burden measurement science. Many more advancements in future updates should be possible as larger datasets become available and as computational power allows for more detailed measurement processes. Continued global collaboration will be an integral component. Suggested priority items for the advancement of injury burden estimation are as follows:

First, while much of the global injury burden occurs in low-income and middle-income countries, these countries are frequently the most data-sparse. GBD has rigorously attempted to collect all available data, including police records and verbal autopsy studies and inpatient and outpatient records; however, it is likely that additional data sources in data-sparse countries exist. Parties who are aware of additional data sources that could be used in the GBD estimation framework should consider joining the GBD collaborator network to contribute new sources of data to be used in future estimation updates.

Second, computational and data limitations make it difficult to account for the full disability that might be experienced in the setting of multiple injuries. For example, if an individual sustains a below-neck spinal injury and an upper extremity amputation, the amputation is not directly accounted for in the prevalence or YLD estimate of the injury cause to which this disability is attributed. This problem quickly grows in complexity, as one can imagine an event like a road injury leading to multiple contusions and abrasions, several fractures in different anatomical sites, a mild traumatic brain injury and a spinal cord injury. There are over 3.6 million permutations of injury if one considers only 10 possible natures of injury, making it difficult to quantitatively measure these relationships by cause of injury and by age, sex, year and location. Future research to address this limitation may focus on simulation studies that model the probability of different comorbid injury combinations to better inform disability weight applications.

Third, more data could be used for nature of injury measurement. Traumatic brain injury and spinal cord injury registries, for example, are not currently directly compatible with the GBD injury estimation framework yet provide rich epidemiological information. Future updates to GBD should focus more attention on incorporating data that measure burden of nature of injury in terms of incidence, prevalence or excess mortality. Incorporating these types of data would require a method to be developed such that estimates were internally consistent across cause-nature distributions. While the methods and data required for this update would be complex, they would represent a large increase in the available data that could be used for GBD injuries estimation.

Fourth, measuring the total burden of sexual violence has proven to be a challenging area of estimation in the GBD framework. As noted in the ‘Methods’ section of this paper, one known limitation is how long-term sequelae and conditions may not be adequately accounted for in sexual violence burden estimation. In order to attribute burden from major depressive disorder, anxiety disorders, self-harm and substance use disorders, measuring the relative risk of developing these conditions for victims of sexual violence would allow for population attributable fractions to be calculated and DALYs from these conditions to be attributed to sexual violence. While the premise of this methodological update is relatively simple, currently there are relatively few studies to inform these relative risks, and conducting and adding such studies in the future would be recognised as a major achievement in GBD research as it would allow for more accurate estimation of lifetime disability caused by sexual violence. This effort would moreover represent an important contribution to research surrounding the Sustainable Development Goals related to sexual violence and women’s rights. 23 24

Fifth, non-fatal injuries from conflict and natural disaster are challenging to estimate because of data sparsity in areas that are afflicted by these events. Fatalities are estimated after such events, but there is still considerable injury burden among the population that survives. Since data collection systems and hospitals may also be destroyed in these events, it becomes difficult to collect adequate non-fatal injury data. Global collaboration should also focus on identifying sources of data on non-fatal and fatal injury cases in conflict and natural disaster events.

It will be important to monitor the effects of implementing these priorities as injury measurement science continues to evolve. Global collaborations including the GBD enterprise should monitor performance statistics and utilisation of results by research groups and ministries to track how improvements to injury measurement progress over time. Scientific dialogue and collaboration must be a major focus, and the GBD enterprise is a good forum to support this kind of data sharing. For example, a collaborative effort between researchers in Vietnam and the Institute for Health Metrics on Evaluation on developing a study on Vietnam injury burden following GBD 2017 led to identifying the use of the Vietnam National Injury Survey, which was then added for estimation in GBD 2019. Increasing data collection standardisation efforts should be emphasised as a priority in all countries, particularly countries where data coverage on injuries is sparse. Ongoing dialogue via scientific publications and international conferences should also continue to serve as a forum to discuss data and methodological updates that can continue to refine the science of injuries estimation in GBD.

## CONCLUSION

Measuring injuries burden in GBD is a complex scientific endeavour that leverages large amounts of data, a complex analytical framework and a global research network. GBD 2017 included more comprehensive detail of injury burden than any other known efforts to date. GBD 2019 and future updates will continue to add detail and refine methods in the interest of providing injury burden estimates that are robust, accurate and timely. Expanded injury data collection efforts will be a critical component of future injury burden estimation.

**What is already known on the subject**

- Global Burden of Disease (GBD) 2017 provided an extensive peer-reviewed assessment of death and disability.
- GBD 2017 methods have been reviewed and updated iteratively as new methods and data become available.
- Measuring injury burden in GBD 2017 is complex due to differences in measuring cause of injury versus nature of injury and the temporal difference between them.

**What this study adds**

- This capstone study details key estimation methods that are used for measuring the global burden of injuries as described in related publications in this journal.
- More detailed methods descriptions and model performance metrics from GBD 2017 are provided in this study than in related studies.
- This study also includes suggested future directions for improving injury burden research.
