# Supplementary material for: Estimating global injuries morbidity and mortality: methods and data used in the Global Burden of Disease 2017 study
Source: Inj Prev. 2020 Aug 24;26(Suppl 1):i125–53. doi: 10.1136/injuryprev-2019-043531 (PMC7571362; doi:10.1136/injuryprev-2019-043531)
Supplement: Supplementary data [file injuryprev-2019-043531supp002.pdf]

Appendix Table 1: Country-level GBD location hierarchy
**Central Europe, Eastern Europe, and Central Asia**
**Central Asia**
Armenia
Azerbaijan
Georgia
Kazakhstan
Kyrgyzstan
Mongolia
Tajikistan
Turkmenistan
Uzbekistan
**Central Europe**
Albania
Bosnia and Herzegovina
Bulgaria
Croatia
Czech Republic
Hungary
Macedonia
Montenegro
Poland
Romania
Serbia
Slovakia

Slovenia
**Eastern Europe**
Belarus
Estonia
Latvia
Lithuania
Moldova
Russia
Ukraine
**High-income**
**Australasia**
Australia
New Zealand
**High-income Asia-Pacific**
Brunei
Japan
South Korea
Singapore
**High-income North America**
Canada
Greenland
USA
**Southern Latin America**
Argentina
Chile

Uruguay
**Western Europe**
Andorra
Austria
Belgium
Cyprus
Denmark
Finland
France
Germany
Greece
Iceland
Ireland
Israel
Italy
Luxembourg
Malta
Netherlands
Norway
Portugal
Spain
Sweden
Switzerland
United Kingdom
**Latin America and Caribbean**

**Andean Latin America**
Bolivia
Ecuador
Peru
**Caribbean**
Antigua and Barbuda
The Bahamas
Barbados
Belize
Bermuda
Cuba
Dominica
Dominican Republic
Grenada
Guyana
Haiti
Jamaica
Puerto Rico
Saint Lucia
Saint Vincent and the Grenadines
Suriname
Trinidad and Tobago
Virgin Islands
**Central Latin America**
Colombia

Costa Rica
El Salvador
Guatemala
Honduras
Mexico
Nicaragua
Panama
Venezuela
**Tropical Latin America**
Brazil
Paraguay
**North Africa and Middle East**
**North Africa and Middle East**
Afghanistan
Algeria
Bahrain
Egypt
Iran
Iraq
Jordan
Kuwait
Lebanon
Libya
Morocco
Palestine

Oman
Qatar
Saudi Arabia
Sudan
Syria
Tunisia
Turkey
United Arab Emirates
Yemen
**South Asia**
**South Asia**
Bangladesh
Bhutan
India
Nepal
Pakistan
**Southeast Asia, East Asia, and Oceania**
**East Asia**
China
North Korea
Taiwan (Province of China)
**Oceania**
American Samoa
Federated States of Micronesia
Fiji

Guam
Kiribati
Marshall Islands
Northern Mariana Islands
Papua New Guinea
Samoa
Solomon Islands
Tonga
Vanuatu
**Southeast Asia**
Cambodia
Indonesia
Laos
Malaysia
Maldives
Mauritius
Myanmar
Philippines
Sri Lanka
Seychelles
Thailand
Timor-Leste
Vietnam
**Sub-Saharan Africa**
**Central sub-Saharan Africa**

Angola
Central African Republic
Congo (Brazzaville)
DR Congo
Equatorial Guinea
Gabon
**Eastern sub-Saharan Africa**
Burundi
Comoros
Djibouti
Eritrea
Ethiopia
Kenya
Madagascar
Malawi
Mozambique
Rwanda
Somalia
South Sudan
Tanzania
Uganda
Zambia
**Southern sub-Saharan Africa**
Botswana
Lesotho

Namibia
South Africa
Swaziland
Zimbabwe
**Western sub-Saharan Africa**
Benin
Burkina Faso
Cameroon
Cape Verde
Chad
Cote d'Ivoire
The Gambia
Ghana
Guinea
Guinea-Bissau
Liberia
Mali
Mauritania
Niger
Nigeria
Sao Tome and Principe
Senegal
Sierra Leone
Togo
