# Supplementary material for: Estimating global injuries morbidity and mortality: methods and data used in the Global Burden of Disease 2017 study
Source: Inj Prev. 2020 Aug 24;26(Suppl 1):i125–53. doi: 10.1136/injuryprev-2019-043531 (PMC7571362; doi:10.1136/injuryprev-2019-043531)
Supplement: Supplementary data [file injuryprev-2019-043531supp003.pdf]

Citation

Centre for Research on the Epidemiology of Disasters (CRED). EM-DAT: The OFDA/CRED International Disaster Database. Brussels, Belgium: Catholic University of Leuven
Norway Hospital Discharge Injury Tabulations 2004 as provided by the Global Burden of Disease 2010 Injury Expert Group. [Unpublished]
Secretariat of Health (Mexico). Mexico Ministry of Health Hospital Discharge Tabulations 2005
Forsén L, Sogaard AJ, Meyer HE, Edna T, Kopjar B. Survival after hip fracture: short-and long-term excess mortality according to age and gender. Osteoporos Int. 1999; 10(1): 73-8
Onarheim H, Vindenes HA. High risk for accidental death in previously burn-injured adults. Burns. 2005; 31(3): 297-301
Centers for Disease Control and Prevention (CDC), Pan American Health Organization (PAHO), Ponce School of Medicine and Health Sciences (Puerto Rico). Zambia - Lusaka Multinational Injury Surveillance Pilot Project 2007 as provided by the Global Burden of Disease 2010 Injury Expert Group
Ministry of Health and Population (Egypt). Egypt National Hospital-Based Injury Surveillance System Tabulations 2005 as provided by the Global Burden of Disease 2010 Injury Expert Group
Portugal Hospital Discharge Injury Tabulations 2004 as provided by the Global Burden of Disease 2010 Injury Expert Group. [Unpublished]
Cameron CM, Purdie DM, Kliewer EV, McClure RJ. Long-term mortality following trauma: 10 year follow-up in a population-based sample of injured adults. J Trauma. 2005; 59(3): 639-46
Moorin R, Miller TR, Hendrie D. Population-based incidence and 5-year survival for hospital-admitted traumatic brain and spinal cord injury, Western Australia, 2003-2008. J Neurol. 2014
Baguley IJ, Nott MT, Howle AA, Simpson GK, Browne S, King AC, Cotter RE, Hodgkinson A. Late mortality after severe traumatic brain injury in New South Wales: a multicentre study. Med J Aust. 2012; 196(1): 40-5
Baguley I, Slewa-Younan S, Lazarus R, Green A. Long-term mortality trends in patients with traumatic brain injury. Brain Inj. 2000; 14(6): 505-12
Colantonio A, Escobar MD, Chipman M, McLellan B, Austin PC, Mirabella G, Ratcliff G. Predictors of postacute mortality following traumatic brain injury in a seriously injured population. J Trauma. 2008; 64(4): 876-82
Middleton JW, Dayton A, Walsh J, Rutkowski SB, Leong G, Duong S. Life expectancy after spinal cord injury: a 50-year study. Spinal Cord. 2012; 50(11): 803–11
Marino RJ, Ditunno JF, Donovan WH, Maynard F. Neurologic recovery after traumatic spinal cord injury: data from the Model Spinal Cord Injury Systems. Arch Phys Med Rehabil. 1999; 80(11): 1391–6
National Center for Health Statistics, Centers for Disease Control and Prevention, United States Census Bureau. United States National Hospital Discharge Survey 1996. Hyattsville, United States: National Center for Health Statistics, Centers for Disease Control and Prevention
National Center for Health Statistics, Centers for Disease Control and Prevention, United States Census Bureau. United States National Hospital Discharge Survey 1997. Hyattsville, United States: National Center for Health Statistics, Centers for Disease Control and Prevention
National Center for Health Statistics, Centers for Disease Control and Prevention, United States Census Bureau. United States National Hospital Discharge Survey 1998. Hyattsville, United States: National Center for Health Statistics, Centers for Disease Control and Prevention
National Center for Health Statistics, Centers for Disease Control and Prevention, United States Census Bureau. United States National Hospital Discharge Survey 1999. Hyattsville, United States: National Center for Health Statistics, Centers for Disease Control and Prevention
National Center for Health Statistics, Centers for Disease Control and Prevention, United States Census Bureau. United States National Hospital Discharge Survey 2000. Hyattsville, United States: National Center for Health Statistics, Centers for Disease Control and Prevention
National Center for Health Statistics, Centers for Disease Control and Prevention, United States Census Bureau. United States National Hospital Discharge Survey 2001. Hyattsville, United States: National Center for Health Statistics, Centers for Disease Control and Prevention
National Center for Health Statistics, Centers for Disease Control and Prevention, United States Census Bureau. United States National Hospital Discharge Survey 2002. Hyattsville, United States: National Center for Health Statistics, Centers for Disease Control and Prevention
National Center for Health Statistics, Centers for Disease Control and Prevention, United States Census Bureau. United States National Hospital Discharge Survey 2003. Hyattsville, United States: National Center for Health Statistics, Centers for Disease Control and Prevention
National Center for Health Statistics, Centers for Disease Control and Prevention, United States Census Bureau. United States National Hospital Discharge Survey 2004. Hyattsville, United States: National Center for Health Statistics, Centers for Disease Control and Prevention
National Center for Health Statistics, Centers for Disease Control and Prevention, United States Census Bureau. United States National Hospital Discharge Survey 2005. Hyattsville, United States: National Center for Health Statistics, Centers for Disease Control and Prevention
National Center for Health Statistics, Centers for Disease Control and Prevention, United States Census Bureau. United States National Hospital Discharge Survey 2006. Hyattsville, United States: National Center for Health Statistics, Centers for Disease Control and Prevention
National Center for Health Statistics, Centers for Disease Control and Prevention, United States Census Bureau. United States National Hospital Discharge Survey 1990. Hyattsville, United States: National Center for Health Statistics, Centers for Disease Control and Prevention
National Center for Health Statistics, Centers for Disease Control and Prevention, United States Census Bureau. United States National Hospital Discharge Survey 1991. Hyattsville, United States: National Center for Health Statistics, Centers for Disease Control and Prevention
National Center for Health Statistics, Centers for Disease Control and Prevention, United States Census Bureau. United States National Hospital Discharge Survey 1992. Hyattsville, United States: National Center for Health Statistics, Centers for Disease Control and Prevention
National Center for Health Statistics, Centers for Disease Control and Prevention, United States Census Bureau. United States National Hospital Discharge Survey 1993. Hyattsville, United States: National Center for Health Statistics, Centers for Disease Control and Prevention
National Center for Health Statistics, Centers for Disease Control and Prevention, United States Census Bureau. United States National Hospital Discharge Survey 1994. Hyattsville, United States: National Center for Health Statistics, Centers for Disease Control and Prevention
National Center for Health Statistics, Centers for Disease Control and Prevention, United States Census Bureau. United States National Hospital Discharge Survey 1995. Hyattsville, United States: National Center for Health Statistics, Centers for Disease Control and Prevention
Centers for Disease Control and Prevention (CDC), Medical University of South Carolina, South Carolina Department of Disabilities and Special Needs, South Carolina Department of Health and Environmental Control. United States - South Carolina Traumatic Brain Injury Follow-up Registry
Ministry of Health and Medical Education (Iran). Iran Hospital Injury Sample Registry Tabulations 2005 as provided by the Global Burden of Disease 2010 Injury Expert Group
Spain Hospital Discharge Injury Tabulations 2000 as provided by the Global Burden of Disease 2010 Injury Expert Group. [Unpublished]
Spain Hospital Discharge Injury Tabulations 2001 as provided by the Global Burden of Disease 2010 Injury Expert Group. [Unpublished]
Spain Hospital Discharge Injury Tabulations 2002 as provided by the Global Burden of Disease 2010 Injury Expert Group. [Unpublished]
Spain Hospital Discharge Injury Tabulations 2003 as provided by the Global Burden of Disease 2010 Injury Expert Group. [Unpublished]
Spain Hospital Discharge Injury Tabulations as provided by the Global Burden of Disease 2010 Injury Expert Group. [Unpublished]
Spain Hospital Discharge Injury Tabulations 2005 as provided by the Global Burden of Disease 2010 Injury Expert Group. [Unpublished]
Spain Hospital Discharge Injury Tabulations 2006 as provided by the Global Burden of Disease 2010 Injury Expert Group. [Unpublished]
Spain Hospital Discharge Injury Tabulations 2007 as provided by the Global Burden of Disease 2010 Injury Expert Group. [Unpublished]
Strauss D, DeVivo MJ, Shavelle R. Long-term mortality risk after spinal cord injury. J Insur Med. 2000; 32: 11-6
Flaada JT, Leibson CL, Mandrekar JN, Diehl N, Perkins PK, Brown AW, Malec JF. Relative risk of mortality after traumatic brain injury: a population-based study of the role of age and injury severity. J Neurotrauma. 2007; 24(3): 435-45
Ratcliff G, Colantonio A, Escobar M, Chase S, Vernich L. Long-term survival following traumatic brain injury. Disabil Rehabil. 2005; 27(6): 305-14
Shavelle R, Strauss D. Comparative mortality of adults with traumatic brain injury in California, 1988--97. J Insur Med. 2000; 32(3): 163-6
Brooks JC, Strauss DJ, Shavelle RM, Paculdo DR, Hammond FM, Harrison-Felix CL. Long-term disability and survival in traumatic brain injury: results from the National Institute on Disability and Rehabilitation Research Model Systems. Arch Phys Med Rehabil. 2013; 94(11): 2203-9
Brown AW, Leibson CL, Malec JF, Perkins PK, Diehl NN, Larson DR. Long-term survival after traumatic brain injury: a population-based analysis. NeuroRehabilitation. 2004; 19(1): 37-43
Harrison-Felix CL, Whiteneck GG, Jha A, DeVivo MJ, Hammond FM, Hart DM. Mortality over four decades after traumatic brain injury rehabilitation: a retrospective cohort study. Arch Phys Med Rehabil. 2009; 90(9): 1506-13
Agency for Healthcare Research and Quality. United States Medical Expenditure Panel Survey 1999 and 2000. Rockville, United States: Agency for Healthcare Research and Quality
Agency for Healthcare Research and Quality. United States Medical Expenditure Panel Survey 2000 and 2001. Rockville, United States: Agency for Healthcare Research and Quality
Agency for Healthcare Research and Quality. United States Medical Expenditure Panel Survey 2001 and 2002. Rockville, United States: Agency for Healthcare Research and Quality
Agency for Healthcare Research and Quality. United States Medical Expenditure Panel Survey 2002 and 2003. Rockville, United States: Agency for Healthcare Research and Quality
Agency for Healthcare Research and Quality. United States Medical Expenditure Panel Survey 2003 and 2004. Rockville, United States: Agency for Healthcare Research and Quality
Agency for Healthcare Research and Quality. United States Medical Expenditure Panel Survey 2004 and 2005. Rockville, United States: Agency for Healthcare Research and Quality
Agency for Healthcare Research and Quality. United States Medical Expenditure Panel Survey 2005 and 2006. Rockville, United States: Agency for Healthcare Research and Quality
Agency for Healthcare Research and Quality. United States Medical Expenditure Panel Survey 2006 and 2007. Rockville, United States: Agency for Healthcare Research and Quality
Agency for Healthcare Research and Quality. United States Medical Expenditure Panel Survey 2007 and 2008. Rockville, United States: Agency for Healthcare Research and Quality
Agency for Healthcare Research and Quality. United States Medical Expenditure Panel Survey 2008 and 2009. Rockville, United States: Agency for Healthcare Research and Quality
Agency for Healthcare Research and Quality. United States Medical Expenditure Panel Survey 2009 and 2010. Rockville, United States: Agency for Healthcare Research and Quality
Agency for Healthcare Research and Quality. United States Medical Expenditure Panel Survey 2010 and 2011. Rockville, United States: Agency for Healthcare Research and Quality
Agency for Healthcare Research and Quality. United States Medical Expenditure Panel Survey 2011 and 2012. Rockville, United States: Agency for Healthcare Research and Quality
Iceland Hospital Discharge Injury Tabulations 2005 as provided by the Global Burden of Disease 2010 Injury Expert Group. [Unpublished]
Sweden Hospital Discharge Injury Tabulations 2004 as provided by the Global Burden of Disease 2010 Injury Expert Group. [Unpublished]
Ministry of Health and Quality of Life (Mauritius). Mauritius Hospital Discharge Injury Tabulations 2003-2007 as provided by the Global Burden of Disease 2010 Injury Expert Group. [Unpublished]
Kanis JA, Oden A, Johnell O, De Laet C, Jonsson B, Oglesby AK. The components of excess mortality after hip fracture. Bone. 2003; 32(5): 468-73
Ministry of Health (Mozambique), World Health Organization (WHO). Mozambique - Maputo Central Hospital Injury Surveillance 2000-2001 as provided by the Global Burden of Disease 2010 Injury Expert Group. [Unpublished]
NHS England. United Kingdom - England Hospital Episode Statistics 2002-2003
NHS England. United Kingdom - England Hospital Episode Statistics 2003-2004
NHS England. United Kingdom - England Hospital Episode Statistics 2004-2005
NHS England. United Kingdom - England Hospital Episode Statistics 2005-2006
NHS England. United Kingdom - England Hospital Episode Statistics 2006-2007
NHS England. United Kingdom - England Hospital Episode Statistics 2007-2008
NHS England. United Kingdom - England Hospital Episode Statistics 2008-2009
NHS England. United Kingdom - England Hospital Episode Statistics 2009-2010
NHS England. United Kingdom - England Hospital Episode Statistics 2010-2011
NHS England. United Kingdom - England Hospital Episode Statistics 2011-2012
NHS England. United Kingdom - England Hospital Episode Statistics 2001-2002
Bulgaria Hospital Discharge Injury Tabulations 2004 as provided by the Global Burden of Disease 2010 Injury Expert Group. [Unpublished]
University of Valle (Colombia). Colombia Cali University of Valle Hospital Injury Surveillance System Tabulations 2006-2009 as provided by the Global Burden of Disease 2010 Injury Expert Group
Slovenia Hospital Discharge Injury Tabulations 2004 as provided by the Global Burden of Disease 2010 Injury Expert Group. [Unpublished]
Frankel HL, Coll JR, Charlifue SW, Whiteneck GG, Gardner BP, Jamous MA, Krishnan KR, Nuseibeh I, Savic G, Sett P. Long-term survival in spinal cord injury: a fifty year investigation. Spinal Cord. 1998; 36(4): 266-74
NHS England. United Kingdom - England Hospital Episode Statistics 2012-2013. Leeds, United Kingdom: NHS Digital
NHS England. United Kingdom - England Hospital Episode Statistics 2013-2014. Leeds, United Kingdom: NHS Digital
NHS England. United Kingdom - England Hospital Episode Statistics 2014-2015. Leeds, United Kingdom: NHS Digital
Chinese Center for Disease Control and Prevention (CCDC), Ministry of Health (China). China National Injury Surveillance System 2006 - China CDC
Chinese Center for Disease Control and Prevention (CCDC), Ministry of Health (China). China National Injury Surveillance System 2007 - China CDC
Chinese Center for Disease Control and Prevention (CCDC), Ministry of Health (China). China National Injury Surveillance System 2008 - China CDC
Chinese Center for Disease Control and Prevention (CCDC), Ministry of Health (China). China National Injury Surveillance System 2009 - China CDC
Chinese Center for Disease Control and Prevention (CCDC), Ministry of Health (China). China National Injury Surveillance System 2010 - China CDC
Chinese Center for Disease Control and Prevention (CCDC), Ministry of Health (China). China National Injury Surveillance System 2011 - China CDC
Chinese Center for Disease Control and Prevention (CCDC), Ministry of Health (China). China National Injury Surveillance System 2012 - China CDC
Chinese Center for Disease Control and Prevention (CCDC). China Zhuhai Study 2006-2007 - China CDC
Chinese Center for Disease Control and Prevention (CCDC). China Injury Comprehensive Surveillance Study 2009-2011 - China CDC
Asian Disaster Reduction Center. Natural Disaster Data Book 2012. Kobe, Japan: Asian Disaster Reduction Center, 2013
The Daily Tribune. RP's Top 10 Deadliest Natural Disasters [Internet]. The Daily Tribune. 2013 Oct 16; Nation
Deutsche Welle. Philippines: A Country Prone to Natural Disasters [Internet]. Deutsche Welle. 2013 Nov 1; World
World Health Organization (WHO). Philippines Floods Situation Report December 2004. Geneva, Switzerland: World Health Organization (WHO), 2004
World Health Organization (WHO). Philippines Natural Disaster Situation Reports. Geneva, Switzerland: World Health Organization (WHO)
World Health Organization Regional Office for the Western Pacific (WPRO-WHO). Philippines - Earthquake hits Negros Oriental, Philippines. Manila, Philippines: World Health Organization Regional Office for the Western Pacific (WPRO-WHO), 2012
World Health Organization Regional Office for the Western Pacific (WPRO-WHO). Philippines - Addressing Health Challenges in the Aftermath of Typhoon Pablo. Manila, Philippines: World Health Organization Regional Office for the Western Pacific (WPRO-WHO), 2012
United Press International. Iran Surveys Damage After Cyclone. United Press International [Internet]. 2007 Jun 9; Top News; [about 1 screen]
Italy Hospital Discharge Injury Tabulations 2003 as provided by the Global Burden of Disease 2010 Injury Expert Group. [Unpublished]
Directorate of Health Statistics and Information, Ministry of Health (Argentina). Argentina Public Hospital Injury Discharges 2007
Directorate of Health Statistics and Information, Ministry of Health (Argentina). Argentina Public Hospital Injury Discharges 2009
National Institute of Epidemiology, National Administration of Laboratories and Health Institutes (ANLIS). Argentina Injury Surveillance System Tabulations 2008 as provided by the Global Burden of Disease 2010 Injury Expert Group. [Unpublished]
Czech Republic Hospital Discharge Injury Tabulations 2004 as provided by the Global Burden of Disease 2010 Injury Expert Group. [Unpublished]
Directorate of Health Statistics and Information, Ministry of Health (Argentina). Argentina Public Hospital Injury Discharges 2011
Directorate of Health Statistics and Information, Ministry of Health (Argentina). Argentina Public Hospital Discharge Statistics 2010
Denmark Hospital Discharge Injury Tabulations 2005 as provided by the Global Burden of Disease 2010 Injury Expert Group. [Unpublished]
Estonia Hospital Discharge Injury Tabulations 2003 as provided by the Global Burden of Disease 2010 Injury Expert Group. [Unpublished]
Hungary Hospital Discharge Injury Tabulations 2004 as provided by the Global Burden of Disease 2010 Injury Expert Group. [Unpublished]
Malta Hospital Discharge Injury Tabulations 2005 as provided by the Global Burden of Disease 2010 Injury Expert Group. [Unpublished]
van Loey NE, van Beeck EF, Faber BW, van de Schoot R, Bremer M. Health-Related Quality of Life After Burns: A Prospective Multicentre Cohort Study With 18 Months Follow-Up. J Trauma. 2011; 72(2): 513-520
Polinder S, van Beeck EF, Essink-Bot ML, Toet H, Looman CW, Mulder S, Meerding WJ. Functional outcome at 2.5, 5, 9, and 24 months after injury in the Netherlands. J Trauma. 2007; 62(1): 133-41
Haagsma JA, Van Beeck EF, Toet H, Polinder S. Posttraumatic stress disorder following injury: trajectories and health-related quality of life. J Depress Anxiety. 2013; http://dx.doi.org/10.4172/2167-1044.S4-002
Consumer Safety Institute (Netherlands). Netherlands Injury Surveillance System 1998
Consumer Safety Institute (Netherlands). Netherlands Injury Surveillance System 1999
Consumer Safety Institute (Netherlands). Netherlands Injury Surveillance System 2000
Consumer Safety Institute (Netherlands). Netherlands Injury Surveillance System 2001
Consumer Safety Institute (Netherlands). Netherlands Injury Surveillance System 2002
Consumer Safety Institute (Netherlands). Netherlands Injury Surveillance System 2003
Consumer Safety Institute (Netherlands). Netherlands Injury Surveillance System 2004
Consumer Safety Institute (Netherlands). Netherlands Injury Surveillance System 2005
Consumer Safety Institute (Netherlands). Netherlands Injury Surveillance System 2006
Consumer Safety Institute (Netherlands). Netherlands Injury Surveillance System 2007
Consumer Safety Institute (Netherlands). Netherlands Injury Surveillance System 2008
Consumer Safety Institute (Netherlands). Netherlands Injury Surveillance System 2009
Consumer Safety Institute (Netherlands). Netherlands Injury Surveillance System 2010
Consumer Safety Institute (Netherlands). Netherlands Injury Surveillance System 2011
Consumer Safety Institute (Netherlands). Netherlands Injury Surveillance System 2012
Ringburg AN, Polinder S, van Ierland MC, Steyerberg EW, van Lieshout EM, Patka P, van Beeck EF, Schipper IB. Prevalence and prognostic factors of disability after major trauma. J Trauma. 2011; 70(4): 916-22
Radboud University Medical Center. Netherlands Radboud University Brain Injury Cohort Study 1998-2011
Dutch Hospital Data (DHD). Netherlands National Medical Registry 1998
Dutch Hospital Data (DHD). Netherlands National Medical Registry 1999
Dutch Hospital Data (DHD). Netherlands National Medical Registry 2000
Dutch Hospital Data (DHD). Netherlands National Medical Registry 2001
Dutch Hospital Data (DHD). Netherlands National Medical Registry 2002
Dutch Hospital Data (DHD). Netherlands National Medical Registry 2003
Dutch Hospital Data (DHD). Netherlands National Medical Registry 2004
Dutch Hospital Data (DHD). Netherlands National Medical Registry 2005
Dutch Hospital Data (DHD). Netherlands National Medical Registry 2006
Dutch Hospital Data (DHD). Netherlands National Medical Registry 2007
Dutch Hospital Data (DHD). Netherlands National Medical Registry 2008
Dutch Hospital Data (DHD). Netherlands National Medical Registry 2009
Dutch Hospital Data (DHD). Netherlands National Medical Registry 2010
Dutch Hospital Data (DHD). Netherlands National Medical Registry 2011
Dutch Hospital Data (DHD). Netherlands National Medical Registry 2012
Latvia Hospital Discharge Injury Tabulations 2004 as provided by the Global Burden of Disease 2010 Injury Expert Group. [Unpublished]
Netherlands Hospital Discharge Injury Tabulations 2005 as provided by the Global Burden of Disease 2010 Injury Expert Group. [Unpublished]
Netherlands Hospital Discharge Injury Tabulations 2004 as provided by the Global Burden of Disease 2010 Injury Expert Group. [Unpublished]
Macedonia Hospital Discharge Injury Tabulations 2005 as provided by the Global Burden of Disease 2010 Injury Expert Group. [Unpublished]
Statistics Indonesia. Indonesia National Socioeconomic Survey 2005
Central Bureau of Statistics (Indonesia). Indonesia National Socioeconomic Survey 1992
Central Bureau of Statistics (Indonesia). Indonesia National Socioeconomic Survey 1993
Central Bureau of Statistics (Indonesia). Indonesia National Socioeconomic Survey 1994
Central Bureau of Statistics (Indonesia), Ministry of Health (Indonesia), United Nations Children's Fund (UNICEF). Indonesia National Socioeconomic Survey 1995
Central Bureau of Statistics (Indonesia), Ministry of Health (Indonesia), United Nations Children's Fund (UNICEF). Indonesia National Socioeconomic Survey 1996
Central Bureau of Statistics (Indonesia), Ministry of Health (Indonesia), United Nations Children's Fund (UNICEF). Indonesia National Socioeconomic Survey 1997

Central Bureau of Statistics (Indonesia), Ministry of Health (Indonesia), World Bank. Indonesia National Socioeconomic Survey 2000
Central Bureau of Statistics (Indonesia), Ministry of Health (Indonesia), World Bank. Indonesia National Socioeconomic Survey 2001
Statistics Indonesia. Indonesia National Socioeconomic Survey 2004
Center for Research and Teaching in Economics (CIDE) (Mexico), Ibero-American University, National Institute of Perinatology (Mexico), National Institute of Statistics and Geography (INEGI) (Mexico). Mexico Family Life Survey 2002
National Institute of Public Health (Mexico). Mexico National Health Survey 1999-2000
National Institute of Public Health (Mexico). Mexico National Survey of Health and Nutrition 2005-2006. Cuernavaca, Mexico: National Institute of Public Health (Mexico)
Macro International, Inc, Ministry of Health (Cambodia), National Institute of Statistics (Cambodia). Cambodia Demographic and Health Survey 2000. Fairfax, United States: ICF International
World Health Organization (WHO). Congo World Health Survey 2003. Geneva, Switzerland: World Health Organization (WHO), 2005
World Health Organization (WHO). France World Health Survey 2003. Geneva, Switzerland: World Health Organization (WHO), 2005
World Health Organization (WHO). Kenya World Health Survey 2004. Geneva, Switzerland: World Health Organization (WHO), 2005
World Health Organization (WHO). Mexico World Health Survey 2002-2003. Geneva, Switzerland: World Health Organization (WHO), 2005
World Health Organization (WHO). Norway World Health Survey 2003. Geneva, Switzerland: World Health Organization (WHO), 2005
World Health Organization (WHO). Vietnam World Health Survey 2002-2003. Geneva, Switzerland: World Health Organization (WHO), 2005
General Statistics Office (Viet Nam), United Nations Development Programme (UNDP), World Bank (WB). Viet Nam Living Standards Measurement Survey 2008. Ha Nội, Viet Nam: General Statistics Office (Viet Nam)
General Statistics Office (Vietnam), United Nations Development Programme (UNDP), World Bank. Vietnam Living Standards Measurement Survey 2006
Statistics Indonesia. Indonesia National Socioeconomic Survey 2010
National Institute of Statistics (Cambodia), Statistics Sweden. Cambodia Socio-Economic Survey 2003-2005. Phnom Penh, Cambodia: National Institute of Statistics (Cambodia)
National Institute of Statistics (Cambodia), Statistics Sweden. Cambodia Socio-Economic Survey 2006-2007. Phnom Penh, Cambodia: National Institute of Statistics (Cambodia)
Pan American Health Organization (PAHO), Center for Demography and Ecology, University of Wisconsin-Madison, Inter-University Consortium for Political and Social Research (ICPSR), College of the Northern Border (COLEF), Research in Health and Demographics (INSAD), National Institute of Medical Sciences and Nutrition Salvador Zubirán. Mexico - Mexico City Survey on Healt
Family Health International, Ministry of Health (Indonesia), National AIDS Commission (KPA), Statistics Indonesia. Indonesia Behavioral Surveillance Survey 2007
Statistics Indonesia. Indonesia National Socioeconomic Survey 2002
Statistics Indonesia. Indonesia National Socioeconomic Survey 2008
Statistics Indonesia. Indonesia National Socioeconomic Survey - Poverty Program Evaluation 2006. Jakarta, Indonesia: Statistics Indonesia
Statistics Indonesia. Indonesia National Socioeconomic Survey - Poverty Program Evaluation 2008-2009. Jakarta, Indonesia: Statistics Indonesia
Central Statistics Organization (Afghanistan), ICF Macro, Indian Institute of Health Management Research (IIHMR), Ministry of Public Health (Afghanistan), World Health Organization Regional Office for the Eastern Mediterranean (EMRO-WHO). Afghanistan Special Demographic and Health Survey 2010. Fairfax, United States: ICF International
California Center for Population Research (CCPR), University of California Los Angeles (UCLA), Center for Research and Teaching in Economics (CIDE) (Mexico), Ibero-American University, National Institute of Public Health (Mexico). Mexico Family Life Survey 2005-2006
Organization for Economic Co-operation and Development (OECD). OECD Health Statistics. Paris, France: Organization for Economic Co-operation and Development (OECD)
National Institute of Public Health (Mexico). Mexico National Survey of Health and Nutrition 2011-2012. Cuernavaca, Mexico: National Institute of Public Health (Mexico)
Statistics Indonesia. Indonesia National Socioeconomic Survey 2011
Healthcare Cost and Utilization Project (HCUP), Agency for Healthcare Research and Quality (AHRQ). United States State Inpatient Databases 2003. Rockville, United States: Healthcare Cost and Utilization Project (HCUP), Agency for Healthcare Research and Quality (AHRQ)
Healthcare Cost and Utilization Project (HCUP), Agency for Healthcare Research and Quality (AHRQ). United States State Inpatient Databases 2004. Rockville, United States: Healthcare Cost and Utilization Project (HCUP), Agency for Healthcare Research and Quality (AHRQ)
Healthcare Cost and Utilization Project (HCUP), Agency for Healthcare Research and Quality (AHRQ). United States State Inpatient Databases 2005. Rockville, United States: Healthcare Cost and Utilization Project (HCUP), Agency for Healthcare Research and Quality (AHRQ)
Healthcare Cost and Utilization Project (HCUP), Agency for Healthcare Research and Quality (AHRQ). United States State Inpatient Databases 2006. Rockville, United States: Healthcare Cost and Utilization Project (HCUP), Agency for Healthcare Research and Quality (AHRQ)
Healthcare Cost and Utilization Project (HCUP), Agency for Healthcare Research and Quality (AHRQ). United States State Inpatient Databases 2007. Rockville, United States: Healthcare Cost and Utilization Project (HCUP), Agency for Healthcare Research and Quality (AHRQ)
Healthcare Cost and Utilization Project (HCUP), Agency for Healthcare Research and Quality (AHRQ). United States State Inpatient Databases 2008. Rockville, United States: Healthcare Cost and Utilization Project (HCUP), Agency for Healthcare Research and Quality (AHRQ)
Ministry of Health (Brazil). Brazil Hospital Information System 1997. Rio de Janeiro, Brazil: Ministry of Health (Brazil)
National Institute of Public Health (Mexico), World Health Organization (WHO). Mexico WHO Study on Global AGEing and Adult Health 2009-2010. Geneva, Switzerland: World Health Organization (WHO), 2011
United Nations Children's Fund (UNICEF). Child Mortality and Injury in Asia. New York, United States: United Nations Children's Fund (UNICEF), 2008. (Innocenti Working Papers: Special Series on Child Injuries Nos. 1-4)
Nguyen TLH, Nguyen THT, Morita S, Sakamoto J. Injury and pre-hospital trauma care in Hanoi, Vietnam. Injury. 2008; 39(9): 1026-33
Hang HM, Ekman R, Bach TT, Byass P, Svanström L. Community-based assessment of unintentional injuries: a pilot study in rural Vietnam. Scand J Public Health Suppl. 2003; 38-44
Hang HM, Bach TT, Byass P. Unintentional injuries over a 1-year period in a rural Vietnamese community: describing an iceberg. Public Health. 2005; 119(6): 466-73
Börsch-Supan, A. (2013). Survey of Health, Ageing and Retirement in Europe (SHARE) Wave 1. Release version: 2.6.0. SHARE-ERIC. Data set. DOI: 10.6103/SHARE.w1.260
Börsch-Supan, A. (2013). Survey of Health, Ageing and Retirement in Europe (SHARE) Wave 2. Release version: 2.6.0. SHARE-ERIC. Data set. DOI: 10.6103/SHARE.w2.260
Börsch-Supan, A. (2013). Survey of Health, Ageing and Retirement in Europe (SHARE) Wave 4. Release version: 1.1.1. SHARE-ERIC. Data set. DOI: 10.6103/SHARE.w4.111
Centers for Disease Control and Prevention (CDC), Kenya Medical Research Institute (KEMRI), Kenya National Bureau of Statistics, Ministry of Public Health and Sanitation (Kenya), National AIDS Control Council (Kenya), National AIDS and STI Control Program (Kenya), National Coordinating Agency for Population and Development (Kenya), National Public Health Laboratory Service
Ministry of Health (Kenya). Kenya National Inpatient Morbidity and Mortality Statistics 1999
Norwegian Directorate of Health. Norway Patient Register 2009
Norwegian Directorate of Health. Norway Patient Register 2010
Norwegian Directorate of Health. Norway Patient Register 2011
Norwegian Directorate of Health. Norway Patient Register 2012
National Institute of Public Health (Mexico), World Health Organization (WHO). Mexico WHO Multi-country Survey Study on Health and Health System Responsiveness 2000-2001. Geneva, Switzerland: World Health Organization (WHO)
Abt Associates Inc., Kenya National Bureau of Statistics, Ministry of Health (Kenya). Kenya Household Health Expenditure and Utilization Survey 2007. Nairobi, Kenya: Kenya National Bureau of Statistics
Center for Research and Teaching in Economics (CIDE) (Mexico), Duke University, Ibero-American University, National Institute of Public Health (Mexico), University of California, Los Angeles (UCLA). Mexico Family Life Survey 2008-2013
Action Africa Help International (AAH-I), Institute for Health Metrics and Evaluation (IHME), Ministry of Medical Services (Kenya), Ministry of Public Health and Sanitation (Kenya). Access, Bottlenecks, Costs, and Equity (ABCE) project in Kenya, 2012. Seattle, United States: Institute for Health Metrics and Evaluation (IHME), 2015
Infectious Diseases Research Collaboration (IDRC), Institute for Health Metrics and Evaluation (IHME), Makerere University, Ministry of Health (Uganda). Access, Bottlenecks, Costs, and Equity (ABCE) project in Uganda, 2012. Seattle, United States: Institute for Health Metrics and Evaluation (IHME), 2015
Kenya National Bureau of Statistics, Ministry of Health (Kenya), World Health Organization (WHO). Kenya STEPS Noncommunicable Disease Risk Factors Survey 2015
National Institute of Statistics (Cambodia), Statistics Sweden. Cambodia Socio-Economic Survey 2007-2008. Phnom Penh, Cambodia: National Institute of Statistics (Cambodia)
Brazilian Institute of Geography and Statistics (IBGE), Ministry of Health (Brazil), Ministry of Planning, Budget, and Management (Brazil). Brazil National Health Survey 2013. Rio de Janeiro, Brazil: Brazilian Institute of Geography and Statistics (IBGE)
Gallup, World Health Organization (WHO). Bahrain WHO Multi-country Survey Study on Health and Health System Responsiveness 2000-2001. Geneva, Switzerland: World Health Organization (WHO)
Erik Consulting, International Research Associates (INRA) Europe, World Health Organization (WHO). France WHO Multi-country Survey Study on Health and Health System Responsiveness 2000-2001. Geneva, Switzerland: World Health Organization (WHO)
Biomedical Engineering Institute, Kaunas University of Technology, Statistics Lithuania, World Health Organization (WHO). Lithuania WHO Multi-country Survey Study on Health and Health System Responsiveness 2000-2001. Geneva, Switzerland: World Health Organization (WHO)
Gallup, World Health Organization (WHO). Oman WHO Multi-country Survey Study on Health and Health System Responsiveness 2000-2001. Geneva, Switzerland: World Health Organization (WHO)
Börsch-Supan, A. (2015). Survey of Health, Ageing and Retirement in Europe (SHARE) Wave 5. Release version: 1.0.0. SHARE-ERIC. Data set. DOI: 10.6103/SHARE.w5.100
Ministry of Health (Brazil). Brazil Hospital Information System 1998-2002
Ministry of Health (Brazil). Brazil Hospital Information System 2003-2007
Ministry of Health (Brazil). Brazil Hospital Information System 2008-2012
Ministry of Health (Brazil). Brazil Hospital Information System 2013-2014
Ministry of Health (Mexico). Mexico Ministry of Health Hospital Discharges 2000-2002
Ministry of Health (Mexico). Mexico Ministry of Health Hospital Discharges 2003-2007
Ministry of Health (Mexico). Mexico Ministry of Health Hospital Discharges 2008-2012
Healthcare Cost and Utilization Project (HCUP), Agency for Healthcare Research and Quality (AHRQ). United States State Inpatient Databases 2003-2007
Healthcare Cost and Utilization Project (HCUP), Agency for Healthcare Research and Quality (AHRQ). United States State Inpatient Databases 2008-2009
Norwegian Directorate of Health. Norway Patient Register 2002
Norwegian Directorate of Health. Norway Patient Register 2003
Healthcare Cost and Utilization Project (HCUP), Agency for Healthcare Research and Quality (AHRQ). United States State Inpatient Databases 2011. Rockville, United States: Healthcare Cost and Utilization Project (HCUP), Agency for Healthcare Research and Quality (AHRQ)
Healthcare Cost and Utilization Project (HCUP), Agency for Healthcare Research and Quality (AHRQ). United States State Inpatient Databases 2012. Rockville, United States: Healthcare Cost and Utilization Project (HCUP), Agency for Healthcare Research and Quality (AHRQ)
Healthcare Cost and Utilization Project (HCUP), Agency for Healthcare Research and Quality (AHRQ). United States State Inpatient Databases 2013. Rockville, United States: Healthcare Cost and Utilization Project (HCUP), Agency for Healthcare Research and Quality (AHRQ)
Healthcare Cost and Utilization Project (HCUP), Agency for Healthcare Research and Quality (AHRQ). United States State Inpatient Databases 2014. Rockville, United States: Healthcare Cost and Utilization Project (HCUP), Agency for Healthcare Research and Quality (AHRQ)
Ministry of Health (Vietnam). Vietnam Hospital Data 2013
World Health Organization Regional Office for Europe (WHO/Europe). European Health for All Database - Inpatient Care Discharges Per 100. Copenhagen, Denmark: World Health Organization Regional Office for Europe (WHO/Europe)
Norwegian Directorate of Health. Norway Patient Register 2013
Norwegian Directorate of Health. Norway Patient Register 2014
Norwegian Directorate of Health. Norway Patient Register 2001
Norwegian Directorate of Health. Norway Patient Register 2000
Norwegian Directorate of Health. Norway Patient Register 1999
Norwegian Directorate of Health. Norway Patient Register 1998
Norwegian Directorate of Health. Norway Patient Register 1997
Norwegian Directorate of Health. Norway Patient Register 1996
Norwegian Directorate of Health. Norway Patient Register 1995
Norwegian Directorate of Health. Norway Patient Register 1994
Norwegian Directorate of Health. Norway Patient Register 1993
Norwegian Directorate of Health. Norway Patient Register 1992
Norwegian Directorate of Health. Norway Patient Register 1991
Norwegian Directorate of Health. Norway Patient Register 1990
Norwegian Directorate of Health. Norway Patient Register 1989
Norwegian Directorate of Health. Norway Patient Register 1988
Norwegian Directorate of Health. Norway Patient Register 1987
Norwegian Directorate of Health. Norway Patient Register 1986
Norwegian Directorate of Health. Norway Patient Register 1985
Norwegian Directorate of Health. Norway Patient Register 1984
Norwegian Directorate of Health. Norway Patient Register 1983
Norwegian Directorate of Health. Norway Patient Register 1982
Norwegian Directorate of Health. Norway Patient Register 1981
Norwegian Directorate of Health. Norway Patient Register 1980
Norwegian Directorate of Health. Norway Patient Register 1979
Norwegian Directorate of Health. Norway Patient Register 1978
Norwegian Directorate of Health. Norway Patient Register 1977
Norwegian Directorate of Health. Norway Patient Register 1975
Norwegian Directorate of Health. Norway Patient Register 1973
Statistics Canada. Canada Community Health Survey 2000-2001. Ottawa, Canada: Statistics Canada, 2003
RAND Corporation, University of Indonesia. Indonesia Family Life Survey 1993-1994. Santa Monica, United States: RAND Corporation
National Institute of Statistics and Censuses (Nicaragua), World Bank. Nicaragua Living Standards Measurement Survey 1993
National Institute of Statistics and Censuses (Nicaragua), World Bank. Nicaragua Living Standards Measurement Survey 1998-1999
Central Statistical Office (Zambia). Zambia Living Conditions Monitoring Survey 2002-2003. Lusaka, Zambia: Central Statistical Office (Zambia)
Central Statistical Office (Zambia). Zambia Living Conditions Monitoring Survey 2004-2005. Lusaka, Zambia: Central Statistical Office (Zambia)
Australian Bureau of Statistics. Australia National Health Survey 1995. Canberra, Australia: Australian Bureau of Statistics
Analytical and Information Center of the Ministry of Health of Uzbekistan, Macro International, Inc, Ministry of Macroeconomics and Statistics (Uzbekistan). Uzbekistan Special Demographic and Health Survey 2002. Fairfax, United States: ICF International
World Health Organization (WHO). Bangladesh World Health Survey 2003. Geneva, Switzerland: World Health Organization (WHO), 2005
World Health Organization (WHO). Burkina Faso World Health Survey 2002-2003. Geneva, Switzerland: World Health Organization (WHO), 2005
World Health Organization (WHO). Dominican Republic World Health Survey 2003. Geneva, Switzerland: World Health Organization (WHO), 2005
World Health Organization (WHO). Germany World Health Survey 2004. Geneva, Switzerland: World Health Organization (WHO), 2005
World Health Organization (WHO). Mali World Health Survey 2003. Geneva, Switzerland: World Health Organization (WHO), 2005
World Health Organization (WHO). Portugal World Health Survey 2003. Geneva, Switzerland: World Health Organization (WHO), 2006
World Health Organization (WHO). Zambia World Health Survey 2003. Geneva, Switzerland: World Health Organization (WHO), 2005
Federal Environment Agency (Germany), Federal Institute for Drugs and Medical Devices (Germany), Max Planck Institute of Psychiatry, Robert Koch Institute. Germany National Health Interview and Examination Survey 1997-1999. Berlin, Germany: Robert Koch Institute, 2000
Centre for Health Promotion Studies, National University of Ireland, Galway, Health Promotion Unit, Department of Health and Children (Ireland). Ireland Survey of Lifestyle Attitudes and Nutrition 1998. Dublin, Ireland: Health Promotion Unit, Department of Health and Children (Ireland)
Qatar Statistics Authority, Supreme Council of Health (Qatar), World Health Organization (WHO). Qatar STEPS Noncommunicable Disease Risk Factors Survey 2012
Health Care International, World Health Organization (WHO). Egypt WHO Multi-country Survey Study on Health and Health System Responsiveness 2000-2001. Geneva, Switzerland: World Health Organization (WHO)
Institute of Health Systems (India), World Health Organization (WHO). India - Andhra Pradesh WHO Multi-country Survey Study on Health and Health System Responsiveness 2000-2001
Ministry of Health and Family Welfare (Bangladesh), The Alliance for Safe Children (TASC), United Nations Children's Fund (UNICEF). Bangladesh Health and Injury Survey 2003
Ministry of Health (Nicaragua), National Institute for Development Information (Nicaragua). Nicaragua National Demographic and Health Survey 2011-2012. Managua, Nicaragua: National Institute for Development Information (Nicaragua)
Robert Koch Institute. Germany Health Update 2009-2010. Berlin, Germany: Robert Koch Institute
Statistics Canada. Canada Community Health Survey 2005. Ottawa, Canada: Statistics Canada
Statistics Canada. Canada Community Health Survey 2007-2008. Ottawa, Canada: Statistics Canada, 2009
Central Statistical Office (Zambia), Churches Health Association of Zambia (CHAZ), Clinton Health Access Initiative (CHAI), Institute for Health Metrics and Evaluation (IHME), Ministry of Health (Zambia), University of Zambia. Access, Bottlenecks, Costs, and Equity (ABCE) project in Zambia, 2011-2012. Seattle, United States: Institute for Health Metrics and Evaluation (IHME), 201
TQA Research, World Health Organization (WHO). Australia WHO Multi-country Survey Study on Health and Health System Responsiveness 2000-2001. Geneva, Switzerland: World Health Organization (WHO)
Environics Research Group, World Health Organization (WHO). Canada WHO Multi-country Survey Study on Health and Health System Responsiveness 2000-2001. Geneva, Switzerland: World Health Organization (WHO)
International Research Associates (INRA) Europe, World Health Organization (WHO). Germany WHO Multi-country Survey Study on Health and Health System Responsiveness 2000-2001. Geneva, Switzerland: World Health Organization (WHO)
Public Opinion Research Center (CBOS) (Poland), World Health Organization (WHO). Poland WHO Multi-country Survey Study on Health and Health System Responsiveness 2000-2001. Geneva, Switzerland: World Health Organization (WHO)
International Research Associates (INRA) Europe, World Health Organization (WHO). Portugal WHO Multi-country Survey Study on Health and Health System Responsiveness 2000-2001. Geneva, Switzerland: World Health Organization (WHO)
Börsch-Supan, A. (2015). Survey of Health, Ageing and Retirement in Europe (SHARE) Wave 5. Release version: 1.0.0. SHARE-ERIC. Data set. DOI: 10.6103/SHARE.w5.100
Concluzia-Prim Center for Survey Methodology (Moldova), Independent Sociology and Information Service (OPINIA) (Moldova), Institute for Advanced Studies (Austria), London School of Hygiene and Tropical Medicine, University of Aberdeen. Moldova Health in Times of Transition Household Survey 2010
TRL Limited (United Kingdom). The Involvement and Impact of Road Crashes on the Poor: Bangladesh and India Case Studies. Workingham, Berkshire, England: TRL Limited (United Kingdom), 2004
Ministry of Rural Development (Mali), National Institute of Statistics (INSTAT) (Mali), World Bank. Mali Agricultural Integrated Economic Survey 2014-2015. Washington DC, United States: World Bank
Ministry of Health (Portugal). Portugal Hospital Inpatient Discharges 2015
Ministry of Health (Poland), National Institute of Public Health-National Institute of Hygiene (NIPH-NIH) (Poland). Poland Hospital Inpatient Discharges 2003
Ministry of Health (Poland), National Institute of Public Health-National Institute of Hygiene (NIPH-NIH) (Poland). Poland Hospital Inpatient Discharges 2004
Federal Statistical Office (Germany). Germany Federal Health Reporting Hospital Discharges 2000
Federal Statistical Office (Germany). Germany Federal Health Reporting Hospital Discharges 2001
Federal Statistical Office (Germany). Germany Federal Health Reporting Hospital Discharges 2002
Federal Statistical Office (Germany). Germany Federal Health Reporting Hospital Discharges 1990
Federal Statistical Office (Germany). Germany Federal Health Reporting Hospital Discharges 1991
Federal Statistical Office (Germany). Germany Federal Health Reporting Hospital Discharges 1992
Federal Statistical Office (Germany). Germany Federal Health Reporting Hospital Discharges 1993
Federal Statistical Office (Germany). Germany Federal Health Reporting Hospital Discharges 1994
Federal Statistical Office (Germany). Germany Federal Health Reporting Hospital Discharges 1995
Federal Statistical Office (Germany). Germany Federal Health Reporting Hospital Discharges 1996
Federal Statistical Office (Germany). Germany Federal Health Reporting Hospital Discharges 1997
Federal Statistical Office (Germany). Germany Federal Health Reporting Hospital Discharges 1998
Federal Statistical Office (Germany). Germany Federal Health Reporting Hospital Discharges 1999

Federal Statistical Office (Germany). Germany Federal Health Reporting Hospital Discharges 2013
Federal Statistical Office (Germany). Germany Federal Health Reporting Hospital Discharges 2014
Statistics Portugal. Portugal Hospital Inpatient Discharges 1985
Statistics Portugal. Portugal Hospital Inpatient Discharges 1986
Statistics Portugal. Portugal Hospital Inpatient Discharges 1987
Statistics Portugal. Portugal Hospital Inpatient Discharges 1988
Statistics Portugal. Portugal Hospital Inpatient Discharges 1989
Statistics Portugal. Portugal Hospital Inpatient Discharges 1990
Statistics Portugal. Portugal Hospital Inpatient Discharges 1991
Statistics Portugal. Portugal Hospital Inpatient Discharges 1992
Statistics Portugal. Portugal Hospital Inpatient Discharges 1993
Statistics Portugal. Portugal Hospital Inpatient Discharges 1994
Statistics Portugal. Portugal Hospital Inpatient Discharges 1995
Statistics Portugal. Portugal Hospital Inpatient Discharges 1996
Statistics Portugal. Portugal Hospital Inpatient Discharges 1997
Statistics Portugal. Portugal Hospital Inpatient Discharges 1998
Statistics Portugal. Portugal Hospital Inpatient Discharges 1999
Statistics Portugal. Portugal Hospital Inpatient Discharges 2000
Statistics Portugal. Portugal Hospital Inpatient Discharges 2001
Statistics Portugal. Portugal Hospital Inpatient Discharges 2002
Statistics Portugal. Portugal Hospital Inpatient Discharges 2003
Statistics Portugal. Portugal Hospital Inpatient Discharges 2004
Statistics Portugal. Portugal Hospital Inpatient Discharges 2005
Statistics Portugal. Portugal Hospital Inpatient Discharges 2006
Statistics Portugal. Portugal Hospital Inpatient Discharges 2007
Statistics Portugal. Portugal Hospital Inpatient Discharges 2008
Statistics Portugal. Portugal Hospital Inpatient Discharges 2009
Statistics Portugal. Portugal Hospital Inpatient Discharges 2010
Statistics Portugal. Portugal Hospital Inpatient Discharges 2011
Statistics Portugal. Portugal Hospital Inpatient Discharges 2012
Statistics Portugal. Portugal Hospital Inpatient Discharges 2013
Statistics Portugal. Portugal Hospital Inpatient Discharges 2014
Ministry of Health (Poland). Poland Hospital Inpatient Discharges 2002
Ministry of Health (Poland). Poland Hospital Inpatient Discharges 2001
Ministry of Health (Poland). Poland Hospital Inpatient Discharges 2000
Ministry of Health (Poland). Poland Hospital Inpatient Discharges 1999
Ministry of Health (Poland). Poland Hospital Inpatient Discharges 1998
Ministry of Health (Poland). Poland Hospital Inpatient Discharges 1997
Ministry of Health (Poland). Poland Hospital Inpatient Discharges 1996
Ministry of Health (Poland). Poland Hospital Inpatient Discharges 1995
Ministry of Health (Poland). Poland Hospital Inpatient Discharges 1994
Ministry of Health (Poland). Poland Hospital Inpatient Discharges 1993
Ministry of Health (Poland). Poland Hospital Inpatient Discharges 1992
Ministry of Health (Poland). Poland Hospital Inpatient Discharges 1991
Ministry of Health (Poland). Poland Hospital Inpatient Discharges 1990
Ministry of Health (Poland). Poland Hospital Inpatient Discharges 1989
Ministry of Health (Poland). Poland Hospital Inpatient Discharges 1988
Ministry of Health (Poland). Poland Hospital Inpatient Discharges 1987
Ministry of Health (Poland). Poland Hospital Inpatient Discharges 1986
Ministry of Health (Poland). Poland Hospital Inpatient Discharges 1985
Ministry of Health (Poland). Poland Hospital Inpatient Discharges 1984
Ministry of Health (Poland). Poland Hospital Inpatient Discharges 1983
Ministry of Health (Poland). Poland Hospital Inpatient Discharges 1982
Ministry of Health (Poland). Poland Hospital Inpatient Discharges 1981
Ministry of Health (Poland). Poland Hospital Inpatient Discharges 1980
Ministry of Health (Poland), Ministry of Health (Poland). Poland Hospital Inpatient Discharges 2013
Ministry of Health (Poland). Poland Hospital Inpatient Discharges 2014
Census and Statistics Directorate (Panama), Ministry of Economy and Finance (Panama), World Bank. Panama Living Standard Measurement Survey 2003. Washington DC, United States: World Bank
Carolina Population Center, University of North Carolina at Chapel Hill, Institute of Sociology, Russian Academy of Sciences, National Research University Higher School of Economics (Russia), ZAO Demoscope. Russia Longitudinal Monitoring Survey of HSE, Round II 1992-1993
Carolina Population Center, University of North Carolina at Chapel Hill, Institute of Sociology, Russian Academy of Sciences, National Research University Higher School of Economics (Russia), ZAO Demoscope. Russia Longitudinal Monitoring Survey of HSE, Round IV 1993-1994
Russia Longitudinal Monitoring Survey (RLMS-HSE), Round VII 1996. National Research University Higher School of Economics, ZAO Demoscope, Carolina Population Center, Univeristy of North Carolina at Chapel Hill, Institute of Sociology, Russian Academy of Sciences
Russia Longitudinal Monitoring Survey (RLMS-HSE), Round VIII 1998-1999. National Research University Higher School of Economics, ZAO Demoscope, Carolina Population Center, Univeristy of North Carolina at Chapel Hill, Institute of Sociology, Russian Academy of Sciences
Russia Longitudinal Monitoring Survey (RLMS-HSE), Round X 2001. National Research University Higher School of Economics, ZAO Demoscope, Carolina Population Center, Univeristy of North Carolina at Chapel Hill, Institute of Sociology, Russian Academy of Sciences
Russia Longitudinal Monitoring Survey (RLMS-HSE), Round XI 2002. National Research University Higher School of Economics, ZAO Demoscope, Carolina Population Center, Univeristy of North Carolina at Chapel Hill, Institute of Sociology, Russian Academy of Sciences
Russia Longitudinal Monitoring Survey (RLMS-HSE), Round XII 2003. National Research University Higher School of Economics, ZAO Demoscope, Carolina Population Center, Univeristy of North Carolina at Chapel Hill, Institute of Sociology, Russian Academy of Sciences
Russia Longitudinal Monitoring Survey (RLMS-HSE), Round XIII 2004. National Research University Higher School of Economics, ZAO Demoscope, Carolina Population Center, Univeristy of North Carolina at Chapel Hill, Institute of Sociology, Russian Academy of Sciences
Russia Longitudinal Monitoring Survey (RLMS-HSE), Round XIV 2005. National Research University Higher School of Economics, ZAO Demoscope, Carolina Population Center, Univeristy of North Carolina at Chapel Hill, Institute of Sociology, Russian Academy of Sciences
Ministry of Health, Social Services and Equality (Spain), National Statistics Institute (Spain). Spain National Health Survey 2006-2007
National Center for Health Statistics, Centers for Disease Control and Prevention, United States Census Bureau. United States National Hospital Ambulatory Medical Care Survey 1992. Hyattsville, United States: National Center for Health Statistics, Centers for Disease Control and Prevention
United States Department of Health and Human Services. Centers for Disease Control and Prevention. National Center for Health Statistics. National Health Interview Survey, 1994: Second Longitudinal Study on Aging, Wave 2, 1997. ICPSR03526-v2. Ann Arbor, MI: Inter-university Consortium for Political and Social Research [distributor], 2007-03-01. http://doi.org/10.3886/ICPSR0
Ministry of Health and Medical Education (Iran), Statistical Centre of Iran. Iran Demographic and Health Survey 2000
World Health Organization (WHO). Ecuador World Health Survey 2003. Geneva, Switzerland: World Health Organization (WHO), 2005
World Health Organization (WHO). Greece World Health Survey 2003. Geneva, Switzerland: World Health Organization (WHO), 2005
World Health Organization (WHO). Laos World Health Survey 2003
World Health Organization (WHO). Malawi World Health Survey 2003. Geneva, Switzerland: World Health Organization (WHO), 2005
World Health Organization (WHO). Mauritania World Health Survey 2003. Geneva, Switzerland: World Health Organization (WHO), 2005
World Health Organization (WHO). Russia World Health Survey 2003. Geneva, Switzerland: World Health Organization (WHO), 2005
World Health Organization (WHO). Spain World Health Survey 2002-2003. Geneva, Switzerland: World Health Organization (WHO), 2005
National Center for Health Statistics (NCHS), Centers for Disease Control and Prevention (CDC). United States National Health and Nutrition Examination Survey 2007-2008. Hyattsville, United States: National Center for Health Statistics (NCHS), Centers for Disease Control and Prevention (CDC), 2009
Central Bureau of Statistics (Sudan), Federal Ministry of Health (Sudan), Government of Sudan, Ministry of Health (South Sudan), Southern Sudan Centre for Census, Statistics and Evaluation. Sudan - South Multiple Indicator Cluster Survey 2010. New York, United States: United Nations Children's Fund (UNICEF), 2015
National Center for Health Statistics (NCHS), Centers for Disease Control and Prevention (CDC), US Census Bureau. United States National Health Interview Survey 1990. Hyattsville, United States: National Center for Health Statistics (NCHS), Centers for Disease Control and Prevention (CDC)
National Center for Health Statistics (NCHS), Centers for Disease Control and Prevention (CDC), US Census Bureau. United States National Health Interview Survey 1991. Hyattsville, United States: National Center for Health Statistics (NCHS), Centers for Disease Control and Prevention (CDC)
National Center for Health Statistics (NCHS), Centers for Disease Control and Prevention (CDC), US Census Bureau. United States National Health Interview Survey 1992. Hyattsville, United States: National Center for Health Statistics (NCHS), Centers for Disease Control and Prevention (CDC)
National Center for Health Statistics (NCHS), Centers for Disease Control and Prevention (CDC), US Census Bureau. United States National Health Interview Survey 1993. Hyattville, United States: National Center for Health Statistics (NCHS), Centers for Disease Control and Prevention (CDC)
National Center for Health Statistics (NCHS), Centers for Disease Control and Prevention (CDC), US Census Bureau. United States National Health Interview Survey 1997. Hyattsville, United States: National Center for Health Statistics (NCHS), Centers for Disease Control and Prevention (CDC)
National Center for Health Statistics (NCHS), Centers for Disease Control and Prevention (CDC), US Census Bureau. United States National Health Interview Survey 1998. Hyattsville, United States: National Center for Health Statistics (NCHS), Centers for Disease Control and Prevention (CDC)
National Center for Health Statistics (NCHS), Centers for Disease Control and Prevention (CDC), US Census Bureau. United States National Health Interview Survey 1999. Hyattsville, United States: National Center for Health Statistics (NCHS), Centers for Disease Control and Prevention (CDC)
National Center for Health Statistics (NCHS), Centers for Disease Control and Prevention (CDC), US Census Bureau. United States National Health Interview Survey 2000. Hyattsville, United States: National Center for Health Statistics (NCHS), Centers for Disease Control and Prevention (CDC)
National Center for Health Statistics (NCHS), Centers for Disease Control and Prevention (CDC), US Census Bureau. United States National Health Interview Survey 2001. Hyattsville, United States: National Center for Health Statistics (NCHS), Centers for Disease Control and Prevention (CDC)
National Center for Health Statistics (NCHS), Centers for Disease Control and Prevention (CDC), US Census Bureau. United States National Health Interview Survey 2002. Hyattsville, United States: National Center for Health Statistics (NCHS), Centers for Disease Control and Prevention (CDC)
National Center for Health Statistics (NCHS), Centers for Disease Control and Prevention (CDC), US Census Bureau. United States National Health Interview Survey 2003. Hyattsville, United States: National Center for Health Statistics (NCHS), Centers for Disease Control and Prevention (CDC)
National Center for Health Statistics (NCHS), Centers for Disease Control and Prevention (CDC), US Census Bureau. United States National Health Interview Survey 2004. Hyattsville, United States: National Center for Health Statistics (NCHS), Centers for Disease Control and Prevention (CDC)
National Center for Health Statistics (NCHS), Centers for Disease Control and Prevention (CDC), US Census Bureau. United States National Health Interview Survey 2006. Hyattsville, United States: National Center for Health Statistics (NCHS), Centers for Disease Control and Prevention (CDC)
National Center for Health Statistics (NCHS), Centers for Disease Control and Prevention (CDC), US Census Bureau. United States National Health Interview Survey 2007. Hyattsville, United States: National Center for Health Statistics (NCHS), Centers for Disease Control and Prevention (CDC)
National Center for Health Statistics (NCHS), Centers for Disease Control and Prevention (CDC), US Census Bureau. United States National Health Interview Survey 2008. Hyattsville, United States: National Center for Health Statistics (NCHS), Centers for Disease Control and Prevention (CDC)
National Center for Health Statistics (NCHS), Centers for Disease Control and Prevention (CDC), US Census Bureau. United States National Health Interview Survey 2009. Hyattsville, United States: National Center for Health Statistics (NCHS), Centers for Disease Control and Prevention (CDC)
National Center for Health Statistics (NCHS), Centers for Disease Control and Prevention (CDC), US Census Bureau. United States National Health Interview Survey 2010. Hyattsville, United States: National Center for Health Statistics (NCHS), Centers for Disease Control and Prevention (CDC)
National Statistical Office of Malawi, World Bank. Malawi Living Standards Measurement Survey 2004-2005
National Center for Health Statistics (NCHS), Centers for Disease Control and Prevention (CDC). United States National Health and Nutrition Examination Survey 2005-2006. Hyattsville, United States: National Center for Health Statistics (NCHS), Centers for Disease Control and Prevention (CDC), 2007
National Center for Health Statistics (NCHS), Centers for Disease Control and Prevention (CDC). United States National Health and Nutrition Examination Survey 2003-2004. Hyattsville, United States: National Center for Health Statistics (NCHS), Centers for Disease Control and Prevention (CDC)
National Center for Health Statistics (NCHS), Centers for Disease Control and Prevention (CDC). United States National Health and Nutrition Examination Survey 2009-2010. Hyattsville, United States: National Center for Health Statistics (NCHS), Centers for Disease Control and Prevention (CDC), 2011
National Center for Health Statistics (NCHS), Centers for Disease Control and Prevention (CDC). United States National Health and Nutrition Examination Survey 1988-1994. Hyattsville, United States: National Center for Health Statistics (NCHS), Centers for Disease Control and Prevention (CDC)
National Center for Health Statistics (NCHS), Centers for Disease Control and Prevention (CDC). United States National Health and Nutrition Examination Survey 2001-2002. Hyattsville, United States: National Center for Health Statistics (NCHS), Centers for Disease Control and Prevention (CDC)
National Center for Health Statistics (NCHS), Centers for Disease Control and Prevention (CDC). United States National Health and Nutrition Examination Survey 1999-2000. Hyattsville, United States: National Center for Health Statistics (NCHS), Centers for Disease Control and Prevention (CDC)
Analytical Sciences, Inc., National Center for Health Statistics, Centers for Disease Control and Prevention, United States Census Bureau. United States National Hospital Ambulatory Medical Care Survey 1993. Hyattsville, United States: National Center for Health Statistics, Centers for Disease Control and Prevention
Analytical Sciences, Inc., National Center for Health Statistics, Centers for Disease Control and Prevention, United States Census Bureau. United States National Hospital Ambulatory Medical Care Survey 1994. Hyattsville, United States: National Center for Health Statistics, Centers for Disease Control and Prevention
Analytical Sciences, Inc., National Center for Health Statistics, Centers for Disease Control and Prevention, United States Census Bureau. United States National Hospital Ambulatory Medical Care Survey 1995. Hyattsville, United States: National Center for Health Statistics, Centers for Disease Control and Prevention
Analytical Sciences, Inc., National Center for Health Statistics, Centers for Disease Control and Prevention, United States Census Bureau. United States National Hospital Ambulatory Medical Care Survey 1996. Hyattsville, United States: National Center for Health Statistics, Centers for Disease Control and Prevention
Analytical Sciences, Inc., National Center for Health Statistics, Centers for Disease Control and Prevention, United States Census Bureau. United States National Hospital Ambulatory Medical Care Survey 1997. Hyattsville, United States: National Center for Health Statistics, Centers for Disease Control and Prevention
Analytical Sciences, Inc., National Center for Health Statistics, Centers for Disease Control and Prevention, United States Census Bureau. United States National Hospital Ambulatory Medical Care Survey 1998. Hyattsville, United States: National Center for Health Statistics, Centers for Disease Control and Prevention
Analytical Sciences, Inc., National Center for Health Statistics, Centers for Disease Control and Prevention, United States Census Bureau. United States National Hospital Ambulatory Medical Care Survey 1999. Hyattsville, United States: National Center for Health Statistics, Centers for Disease Control and Prevention
Constella Group, National Center for Health Statistics, Centers for Disease Control and Prevention, United States Census Bureau. United States National Hospital Ambulatory Medical Care Survey 2002. Hyattsville, United States: National Center for Health Statistics, Centers for Disease Control and Prevention
Analytical Sciences, Inc., National Center for Health Statistics, Centers for Disease Control and Prevention, United States Census Bureau. United States National Hospital Ambulatory Medical Care Survey 2000. Hyattsville, United States: National Center for Health Statistics, Centers for Disease Control and Prevention
Analytical Sciences, Inc., National Center for Health Statistics, Centers for Disease Control and Prevention, United States Census Bureau. United States National Hospital Ambulatory Medical Care Survey 2001. Hyattsville, United States: National Center for Health Statistics, Centers for Disease Control and Prevention
Constella Group, National Center for Health Statistics, Centers for Disease Control and Prevention, United States Census Bureau. United States National Hospital Ambulatory Medical Care Survey 2003. Hyattsville, United States: National Center for Health Statistics, Centers for Disease Control and Prevention
Constella Group, National Center for Health Statistics, Centers for Disease Control and Prevention, United States Census Bureau. United States National Hospital Ambulatory Medical Care Survey 2004. Hyattsville, United States: National Center for Health Statistics, Centers for Disease Control and Prevention
Constella Group, National Center for Health Statistics, Centers for Disease Control and Prevention, United States Census Bureau. United States National Hospital Ambulatory Medical Care Survey 2005. Hyattsville, United States: National Center for Health Statistics, Centers for Disease Control and Prevention
Constella Group, National Center for Health Statistics, Centers for Disease Control and Prevention, United States Census Bureau. United States National Hospital Ambulatory Medical Care Survey 2006. Hyattsville, United States: National Center for Health Statistics, Centers for Disease Control and Prevention
National Center for Health Statistics, Centers for Disease Control and Prevention, SRA International, Inc., United States Census Bureau. United States National Hospital Ambulatory Medical Care Survey 2007. Hyattsville, United States: National Center for Health Statistics, Centers for Disease Control and Prevention
National Center for Health Statistics, Centers for Disease Control and Prevention, SRA International, Inc., United States Census Bureau. United States National Hospital Ambulatory Medical Care Survey 2008. Hyattsville, United States: National Center for Health Statistics, Centers for Disease Control and Prevention
National Center for Health Statistics, Centers for Disease Control and Prevention, SRA International, Inc., United States Census Bureau. United States National Hospital Ambulatory Medical Care Survey 2009. Hyattsville, United States: National Center for Health Statistics, Centers for Disease Control and Prevention
Panel Study of Income Dynamics, 2007 public use dataset. Produced and distributed by the University of Michigan with primary funding from the National Science Foundation, the National Institute of Aging, and the National Institute of Child Health and Human Development. Ann Arbor, MI, (2011)
Panel Study of Income Dynamics, 2005 public use dataset. Produced and distributed by the University of Michigan with primary funding from the National Science Foundation, the National Institute of Aging, and the National Institute of Child Health and Human Development. Ann Arbor, MI, (2011)
National Center for Health Statistics, Centers for Disease Control and Prevention, United States Census Bureau. United States National Hospital Discharge Survey 2007. Hyattsville, United States: National Center for Health Statistics, Centers for Disease Control and Prevention
National Center for Health Statistics, Centers for Disease Control and Prevention, United States Census Bureau. United States National Hospital Discharge Survey 2008. Hyattsville, United States: National Center for Health Statistics, Centers for Disease Control and Prevention
National Center for Health Statistics, Centers for Disease Control and Prevention, United States Census Bureau. United States National Hospital Discharge Survey 2009. Hyattsville, United States: National Center for Health Statistics, Centers for Disease Control and Prevention
National Center for Health Statistics, Centers for Disease Control and Prevention, United States Census Bureau. United States National Hospital Discharge Survey 2010. Hyattsville, United States: National Center for Health Statistics, Centers for Disease Control and Prevention
National Center for Health Statistics, Centers for Disease Control and Prevention, United States Census Bureau. United States National Hospital Discharge Survey 1979. Hyattsville, United States: National Center for Health Statistics, Centers for Disease Control and Prevention
National Center for Health Statistics, Centers for Disease Control and Prevention, United States Census Bureau. United States National Hospital Discharge Survey 1980. Hyattsville, United States: National Center for Health Statistics, Centers for Disease Control and Prevention
National Center for Health Statistics, Centers for Disease Control and Prevention, United States Census Bureau. United States National Hospital Discharge Survey 1981. Hyattsville, United States: National Center for Health Statistics, Centers for Disease Control and Prevention
National Center for Health Statistics, Centers for Disease Control and Prevention, United States Census Bureau. United States National Hospital Discharge Survey 1982. Hyattsville, United States: National Center for Health Statistics, Centers for Disease Control and Prevention
National Center for Health Statistics, Centers for Disease Control and Prevention, United States Census Bureau. United States National Hospital Discharge Survey 1983. Hyattsville, United States: National Center for Health Statistics, Centers for Disease Control and Prevention
National Center for Health Statistics, Centers for Disease Control and Prevention, United States Census Bureau. United States National Hospital Discharge Survey 1984. Hyattsville, United States: National Center for Health Statistics, Centers for Disease Control and Prevention
National Center for Health Statistics, Centers for Disease Control and Prevention, United States Census Bureau. United States National Hospital Discharge Survey 1985. Hyattsville, United States: National Center for Health Statistics, Centers for Disease Control and Prevention
National Center for Health Statistics, Centers for Disease Control and Prevention, United States Census Bureau. United States National Hospital Discharge Survey 1986. Hyattsville, United States: National Center for Health Statistics, Centers for Disease Control and Prevention
National Center for Health Statistics, Centers for Disease Control and Prevention, United States Census Bureau. United States National Hospital Discharge Survey 1987. Hyattsville, United States: National Center for Health Statistics, Centers for Disease Control and Prevention
National Center for Health Statistics, Centers for Disease Control and Prevention, United States Census Bureau. United States National Hospital Discharge Survey 1988. Hyattsville, United States: National Center for Health Statistics, Centers for Disease Control and Prevention
National Center for Health Statistics, Centers for Disease Control and Prevention, United States Census Bureau. United States National Hospital Discharge Survey 1989. Hyattsville, United States: National Center for Health Statistics, Centers for Disease Control and Prevention
National Institute of Statistics and Censuses (Ecuador). Ecuador Hospital Inpatient Discharges 1997. Quito, Ecuador: National Institute of Statistics and Censuses (Ecuador)
National Institute of Statistics and Censuses (Ecuador). Ecuador Hospital Inpatient Discharges 1998. Quito, Ecuador: National Institute of Statistics and Censuses (Ecuador)
National Institute of Statistics and Censuses (Ecuador). Ecuador Hospital Inpatient Discharges 1999. Quito, Ecuador: National Institute of Statistics and Censuses (Ecuador)
National Institute of Statistics and Censuses (Ecuador). Ecuador Hospital Inpatient Discharges 2000. Quito, Ecuador: National Institute of Statistics and Censuses (Ecuador)
National Institute of Statistics and Censuses (Ecuador). Ecuador Hospital Inpatient Discharges 2001. Quito, Ecuador: National Institute of Statistics and Censuses (Ecuador)
National Institute of Statistics and Censuses (Ecuador). Ecuador Hospital Inpatient Discharges 2002. Quito, Ecuador: National Institute of Statistics and Censuses (Ecuador)
National Institute of Statistics and Censuses (Ecuador). Ecuador Hospital Inpatient Discharges 2003. Quito, Ecuador: National Institute of Statistics and Censuses (Ecuador)
National Institute of Statistics and Censuses (Ecuador). Ecuador Hospital Inpatient Discharges 2004. Quito, Ecuador: National Institute of Statistics and Censuses (Ecuador)
National Institute of Statistics and Censuses (Ecuador). Ecuador Hospital Inpatient Discharges 2005. Quito, Ecuador: National Institute of Statistics and Censuses (Ecuador)
National Institute of Statistics and Censuses (Ecuador). Ecuador Hospital Inpatient Discharges 2006. Quito, Ecuador: National Institute of Statistics and Censuses (Ecuador)
National Institute of Statistics and Censuses (Ecuador). Ecuador Hospital Inpatient Discharges 2007. Quito, Ecuador: National Institute of Statistics and Censuses (Ecuador)
National Institute of Statistics and Censuses (Ecuador). Ecuador Hospital Inpatient Discharges 2008. Quito, Ecuador: National Institute of Statistics and Censuses (Ecuador)
National Institute of Statistics and Censuses (Ecuador). Ecuador Hospital Inpatient Discharges 2009. Quito, Ecuador: National Institute of Statistics and Censuses (Ecuador)
National Institute of Statistics and Censuses (Ecuador). Ecuador Hospital Inpatient Discharges 2010. Quito, Ecuador: National Institute of Statistics and Censuses (Ecuador)
National Institute of Statistics and Censuses (Ecuador). Ecuador Hospital Inpatient Discharges 2011. Quito, Ecuador: National Institute of Statistics and Censuses (Ecuador)
National Center for Health Statistics (NCHS), Centers for Disease Control and Prevention (CDC), US Census Bureau. United States National Health Interview Survey 2011. Hyattsville, United States: National Center for Health Statistics (NCHS), Centers for Disease Control and Prevention (CDC)
Ministry of Health and Medical Education (Iran), World Health Organization (WHO). Iran WHO Multi-country Survey Study on Health and Health System Responsiveness 2000-2001. Geneva, Switzerland: World Health Organization (WHO)
National Statistical Office of Malawi. Malawi Integrated Household Survey 2010 (IHS3 - Year: 1). Dataset downloaded from [http://go.worldbank.org/NOXNI9YDS0] on [September 19, 2015]
National Center for Health Statistics, Centers for Disease Control and Prevention. United States National Health and Nutrition Examination Survey 2011-2012. Hyattsville, United States: National Center for Health Statistics, Centers for Disease Control and Prevention, 2013
National Center for Health Statistics, Centers for Disease Control and Prevention, United States Census Bureau. United States National Health Interview Survey 2012. Hyattsville, United States: National Center for Health Statistics, Centers for Disease Control and Prevention, 2013
Russian Academy of Medical Science, World Health Organization (WHO). Russia WHO Study on Global AGEing and Adult Health 2007-2010
National Center for Health Statistics, Centers for Disease Control and Prevention, SRA International, Inc., United States Census Bureau. United States National Hospital Ambulatory Medical Care Survey 2010. Hyattsville, United States: National Center for Health Statistics, Centers for Disease Control and Prevention

National Institute of Statistics and Censuses (Ecuador). Ecuador Hospital Inpatient Discharges 2012. Quito, Ecuador: National Institute of Statistics and Censuses (Ecuador), 2013
Russia Longitudinal Monitoring Survey (RLMS-HSE), Round XVIII 2009. National Research University Higher School of Economics, ZAO Demoscope, Carolina Population Center, Univeristy of North Carolina at Chapel Hill, Institute of Sociology, Russian Academy of Sciences
Russia Longitudinal Monitoring Survey (RLMS-HSE), Round XVII 2008. National Research University Higher School of Economics, ZAO Demoscope, Carolina Population Center, Univeristy of North Carolina at Chapel Hill, Institute of Sociology, Russian Academy of Sciences
Russia Longitudinal Monitoring Survey (RLMS-HSE), Round XVI 2007. National Research University Higher School of Economics, ZAO Demoscope, Carolina Population Center, Univeristy of North Carolina at Chapel Hill, Institute of Sociology, Russian Academy of Sciences
Russia Longitudinal Monitoring Survey (RLMS-HSE), Round XV 2006. National Research University Higher School of Economics, ZAO Demoscope, Carolina Population Center, Univeristy of North Carolina at Chapel Hill, Institute of Sociology, Russian Academy of Sciences
Russia Longitudinal Monitoring Survey (RLMS-HSE), Round VI 1995. National Research University Higher School of Economics, ZAO Demoscope, Carolina Population Center, Univeristy of North Carolina at Chapel Hill, Institute of Sociology, Russian Academy of Sciences
Russia Longitudinal Monitoring Survey (RLMS-HSE), Round V 1994. National Research University Higher School of Economics, ZAO Demoscope, Carolina Population Center, Univeristy of North Carolina at Chapel Hill, Institute of Sociology, Russian Academy of Sciences
Government Delegation for the National Plan on Drugs (Spain), Ministry of Health, Social Services and Equality (Spain). Spain Household Survey on Alcohol and Drugs 2007-2008. Madrid, Spain: Ministry of Health, Social Services and Equality (Spain)
Coggan C, Hooper R, Adams B. Self-reported injury rates in New Zealand. N Z Med J. 2002; 115(61): U167
Petridou E, Dessypris N, Frangakis CE, Belechri M, Mavrou A, Trichopoulos D. Estimating the population burden of injuries: a comparison of household surveys and emergency department surveillance. Epidemiology. 2004; 15(4): 428-32
Ministry of Health, Social Services and Equality (Spain), Sociological Research Center (Spain). Spain Health Barometer Survey 2004. Madrid, Spain: Ministry of Health, Social Services and Equality (Spain), 2004
Eurostat, National Institute of Statistics (Romania). Romania European Health Interview Survey 2008 - Eurostat
National Center for Health Statistics, Centers for Disease Control and Prevention, United States Census Bureau. United States National Health Interview Survey 2013. Hyattsville, United States: National Center for Health Statistics, Centers for Disease Control and Prevention, 2014
National Institute of Statistics and Censuses (Ecuador). Ecuador Hospital Inpatient Discharges 2013. Quito, Ecuador: National Institute of Statistics and Censuses (Ecuador)
National Center for Health Statistics, Centers for Disease Control and Prevention. United States National Health and Nutrition Examination Survey 2013-2014. Hyattsville, United States: National Center for Health Statistics, Centers for Disease Control and Prevention
National Center for Health Statistics, Centers for Disease Control and Prevention, SRA International, Inc., United States Census Bureau. United States National Ambulatory Medical Care Survey 2012. Hyattsville, United States: National Center for Health Statistics, Centers for Disease Control and Prevention
International Research Associates (INRA) Europe, World Health Organization (WHO). Spain WHO Multi-country Survey Study on Health and Health System Responsiveness 2000-2001. Geneva, Switzerland: World Health Organization (WHO)
University of Otago (New Zealand), World Health Organization (WHO). New Zealand WHO Multi-country Survey Study on Health and Health System Responsiveness 2000-2001. Geneva, Switzerland: World Health Organization (WHO)
International Research Associates (INRA) Europe, World Health Organization (WHO). Romania WHO Multi-country Survey Study on Health and Health System Responsiveness 2000-2001. Geneva, Switzerland: World Health Organization (WHO)
International Research Associates (INRA) Europe, World Health Organization (WHO). Russia WHO Multi-country Survey Study on Health and Health System Responsiveness 2000-2001. Geneva, Switzerland: World Health Organization (WHO)
Washington State University, World Health Organization (WHO). United States WHO Multi-country Survey Study on Health and Health System Responsiveness 2000-2001. Geneva, Switzerland: World Health Organization (WHO)
National Center for Health Statistics, Centers for Disease Control and Prevention, SRA International, Inc., United States Census Bureau. United States National Hospital Ambulatory Medical Care Survey 2011. Hyattsville, United States: National Center for Health Statistics, Centers for Disease Control and Prevention
Börsch-Supan, A. (2015). Survey of Health, Ageing and Retirement in Europe (SHARE) Wave 5. Release version: 1.0.0. SHARE-ERIC. Data set. DOI: 10.6103/SHARE.w5.100
National Center for Health Statistics, Centers for Disease Control and Prevention. United States National Health Interview Survey 2014. Hyattsville, United States: National Center for Health Statistics, Centers for Disease Control and Prevention, 2015
Ministry of Health (New Zealand). New Zealand National Minimum Dataset 2000
Ministry of Health (New Zealand). New Zealand National Minimum Dataset 2001
Ministry of Health (New Zealand). New Zealand National Minimum Dataset 2002
Ministry of Health (New Zealand). New Zealand National Minimum Dataset 2003
Ministry of Health (New Zealand). New Zealand National Minimum Dataset 2004
Ministry of Health (New Zealand). New Zealand National Minimum Dataset 2005
Ministry of Health (New Zealand). New Zealand National Minimum Dataset 2006
Ministry of Health (New Zealand). New Zealand National Minimum Dataset 2007. Wellington, New Zealand: Ministry of Health (New Zealand)
Ministry of Health (New Zealand). New Zealand National Minimum Dataset 2008. Wellington, New Zealand: Ministry of Health (New Zealand)
Ministry of Health (New Zealand). New Zealand National Minimum Dataset 2009. Wellington, New Zealand: Ministry of Health (New Zealand)
Ministry of Health (New Zealand). New Zealand National Minimum Dataset 2010. Wellington, New Zealand: Ministry of Health (New Zealand)
Ministry of Health (New Zealand). New Zealand National Minimum Dataset 2011. Wellington, New Zealand: Ministry of Health (New Zealand)
Ministry of Health (New Zealand). New Zealand National Minimum Dataset 2012. Wellington, New Zealand: Ministry of Health (New Zealand)
Ministry of Health (New Zealand). New Zealand National Minimum Dataset 2013. Wellington, New Zealand: Ministry of Health (New Zealand)
Ministry of Health (New Zealand). New Zealand National Minimum Dataset 2014. Wellington, New Zealand: Ministry of Health (New Zealand)
National Statistical Office of Malawi. Malawi Integrated Household Survey 2013 (IHS3 - Year: 2). Dataset downloaded from [http://go.worldbank.org/NOXNI9YDS0] on [September 15, 2015]
Center for Sociological Studies, Lomonosov Moscow State University, Concluzia-Prim Center for Survey Methodology (Moldova), Institute for Advanced Studies (Austria), London School of Hygiene and Tropical Medicine, University of Aberdeen. Russia Health in Times of Transition Household Survey 2010
National Center for Health Statistics (NCHS), Centers for Disease Control and Prevention (CDC). United States National Hospital Discharge Survey 1988-1992
National Center for Health Statistics (NCHS), Centers for Disease Control and Prevention (CDC). United States National Hospital Discharge Survey 1993-1997
National Center for Health Statistics (NCHS), Centers for Disease Control and Prevention (CDC). United States National Hospital Discharge Survey 1998-2002
National Center for Health Statistics (NCHS), Centers for Disease Control and Prevention (CDC). United States National Hospital Discharge Survey 2003-2007
National Center for Health Statistics (NCHS), Centers for Disease Control and Prevention (CDC). United States National Hospital Discharge Survey 2008-2010
National Institute of Statistics and Censuses (Ecuador). Ecuador Hospital Inpatient Discharges 2014. Quito, Ecuador: National Institute of Statistics and Censuses (Ecuador)
Healthcare Cost and Utilization Project (HCUP), Agency for Healthcare Research and Quality (AHRQ). United States Nationwide Inpatient Sample 2013. Rockville, United States: Healthcare Cost and Utilization Project (HCUP), Agency for Healthcare Research and Quality (AHRQ)
United States Department of Health and Human Services. Centers for Disease Control and Prevention. National Center for Health Statistics. National Health Interview Survey, 1994: Second Supplement on Aging. ICPSR02563-v3. Ann Arbor, MI: Inter-university Consortium for Political and Social Research [distributor], 2007-02-12. http://doi.org/10.3886/ICPSR02563.v3
National Center for Health Statistics, Centers for Disease Control and Prevention, SRA International, Inc., United States Census Bureau. United States National Hospital Ambulatory Medical Care Survey 2012. Hyattsville, United States: National Center for Health Statistics, Centers for Disease Control and Prevention
National Center for Health Statistics, Centers for Disease Control and Prevention, SRA International, Inc., United States Census Bureau. United States National Ambulatory Medical Care Survey 2013. Hyattsville, United States: National Center for Health Statistics, Centers for Disease Control and Prevention
National Center for Health Statistics, Centers for Disease Control and Prevention, SRA International, Inc., United States Census Bureau. United States National Hospital Ambulatory Medical Care Survey 2013. Hyattsville, United States: National Center for Health Statistics, Centers for Disease Control and Prevention
Ministry of Health (New Zealand). New Zealand National Minimum Dataset 2000-2002
Ministry of Health (New Zealand). New Zealand National Minimum Dataset 2003-2007
Ministry of Health (New Zealand). New Zealand National Minimum Dataset 2008-2012
Ministry of Health (New Zealand). New Zealand National Minimum Dataset 2013-2014
Ministry of Health (New Zealand). New Zealand National Minimum Dataset 2015. Wellington, New Zealand: Ministry of Health (New Zealand)
Ministry of Health (Romania). Romania Hospital Inpatient Discharges 2000
Ministry of Health (Romania). Romania Hospital Inpatient Discharges 2001
Ministry of Health (Romania). Romania Hospital Inpatient Discharges 2002
Ministry of Health (Romania). Romania Hospital Inpatient Discharges 2003
Ministry of Health (Romania). Romania Hospital Inpatient Discharges 2004
Ministry of Health (Romania). Romania Hospital Inpatient Discharges 2005
National School of Public Health (Greece), World Health Organization (WHO). Greece WHO Multi-country Survey Study on Health and Health System Responsiveness 2000-2001
Healthcare Cost and Utilization Project (HCUP), Agency for Healthcare Research and Quality (AHRQ). United States Nationwide Inpatient Sample 2014. Rockville, United States: Healthcare Cost and Utilization Project (HCUP), Agency for Healthcare Research and Quality (AHRQ)
Healthcare Cost and Utilization Project (HCUP), Agency for Healthcare Research and Quality (AHRQ). United States Nationwide Inpatient Sample 2015. Rockville, United States: Healthcare Cost and Utilization Project (HCUP), Agency for Healthcare Research and Quality (AHRQ)
Ministry of Health (Romania). Romania Hospital Inpatient Discharges 1980
Ministry of Health and Social Security (Spain), National Statistics Institute (Spain). Spain Statistics on Health Establishments Providing Inpatient Care 1972
Ministry of Health and Social Security (Spain), National Statistics Institute (Spain). Spain Statistics on Health Establishments Providing Inpatient Care 1973
Ministry of Health and Social Security (Spain), National Statistics Institute (Spain). Spain Statistics on Health Establishments Providing Inpatient Care 1974
Ministry of Health and Social Security (Spain), National Statistics Institute (Spain). Spain Statistics on Health Establishments Providing Inpatient Care 1975
Ministry of Health and Social Security (Spain), National Statistics Institute (Spain). Spain Statistics on Health Establishments Providing Inpatient Care 1976
Ministry of Health and Social Security (Spain), National Statistics Institute (Spain). Spain Statistics on Health Establishments Providing Inpatient Care 1977
Ministry of Health and Social Security (Spain), National Statistics Institute (Spain). Spain Statistics on Health Establishments Providing Inpatient Care 1978
Ministry of Health and Social Security (Spain), National Statistics Institute (Spain). Spain Statistics on Health Establishments Providing Inpatient Care 1979
Ministry of Health and Social Security (Spain), National Statistics Institute (Spain). Spain Statistics on Health Establishments Providing Inpatient Care 1980
Ministry of Health and Social Security (Spain), National Statistics Institute (Spain). Spain Statistics on Health Establishments Providing Inpatient Care 1981
Ministry of Health and Consumer Affairs (Spain), National Statistics Institute (Spain). Spain Statistics on Health Establishments Providing Inpatient Care 1982
Ministry of Health and Consumer Affairs (Spain), National Statistics Institute (Spain). Spain Statistics on Health Establishments Providing Inpatient Care 1983
Ministry of Health and Consumer Affairs (Spain), National Statistics Institute (Spain). Spain Statistics on Health Establishments Providing Inpatient Care 1984
Ministry of Health and Consumer Affairs (Spain), National Statistics Institute (Spain). Spain Statistics on Health Establishments Providing Inpatient Care 1985
Ministry of Health and Consumer Affairs (Spain), National Statistics Institute (Spain). Spain Statistics on Health Establishments Providing Inpatient Care 1986
Ministry of Health and Consumer Affairs (Spain), National Statistics Institute (Spain). Spain Statistics on Health Establishments Providing Inpatient Care 1987
Ministry of Health and Consumer Affairs (Spain), National Statistics Institute (Spain). Spain Statistics on Health Establishments Providing Inpatient Care 1988
Ministry of Health and Consumer Affairs (Spain), National Statistics Institute (Spain). Spain Statistics on Health Establishments Providing Inpatient Care 1989
Ministry of Health and Consumer Affairs (Spain), National Statistics Institute (Spain). Spain Statistics on Health Establishments Providing Inpatient Care 1990
Ministry of Health and Consumer Affairs (Spain), National Statistics Institute (Spain). Spain Statistics on Health Establishments Providing Inpatient Care 1991
Ministry of Health and Consumer Affairs (Spain), National Statistics Institute (Spain). Spain Statistics on Health Establishments Providing Inpatient Care 1992
Ministry of Health and Consumer Affairs (Spain), National Statistics Institute (Spain). Spain Statistics on Health Establishments Providing Inpatient Care 1993
Ministry of Health and Consumer Affairs (Spain), National Statistics Institute (Spain). Spain Statistics on Health Establishments Providing Inpatient Care 1994
Ministry of Health and Consumer Affairs (Spain), National Statistics Institute (Spain). Spain Statistics on Health Establishments Providing Inpatient Care 1995
Ministry of Health and Consumer Affairs (Spain), National Statistics Institute (Spain). Spain Statistics on Health Establishments Providing Inpatient Care 1996
Ministry of Health and Consumer Affairs (Spain). Spain Statistics on Health Establishments Providing Inpatient Care 1997. Madrid, Spain: Ministry of Health, Social Services and Equality (Spain)
Ministry of Health and Consumer Affairs (Spain). Spain Statistics on Health Establishments Providing Inpatient Care 1998. Madrid, Spain: Ministry of Health, Social Services and Equality (Spain)
Ministry of Health and Consumer Affairs (Spain). Spain Statistics on Health Establishments Providing Inpatient Care 1999. Madrid, Spain: Ministry of Health, Social Services and Equality (Spain)
Ministry of Health and Consumer Affairs (Spain). Spain Statistics on Health Establishments Providing Inpatient Care 2000. Madrid, Spain: Ministry of Health, Social Services and Equality (Spain)
Ministry of Health and Consumer Affairs (Spain). Spain Statistics on Health Establishments Providing Inpatient Care 2001. Madrid, Spain: Ministry of Health, Social Services and Equality (Spain)
Ministry of Health and Consumer Affairs (Spain). Spain Statistics on Health Establishments Providing Inpatient Care 2002. Madrid, Spain: Ministry of Health, Social Services and Equality (Spain)
Ministry of Health and Consumer Affairs (Spain). Spain Statistics on Health Establishments Providing Inpatient Care 2003. Madrid, Spain: Ministry of Health, Social Services and Equality (Spain)
Ministry of Health and Consumer Affairs (Spain). Spain Statistics on Health Establishments Providing Inpatient Care 2004. Madrid, Spain: Ministry of Health, Social Services and Equality (Spain)
Ministry of Health and Consumer Affairs (Spain). Spain Statistics on Health Establishments Providing Inpatient Care 2005. Madrid, Spain: Ministry of Health, Social Services and Equality (Spain)
Ministry of Health and Consumer Affairs (Spain). Spain Statistics on Health Establishments Providing Inpatient Care 2006. Madrid, Spain: Ministry of Health, Social Services and Equality (Spain)
Ministry of Health and Consumer Affairs (Spain). Spain Statistics on Health Establishments Providing Inpatient Care 2007. Madrid, Spain: Ministry of Health, Social Services and Equality (Spain)
Ministry of Health and Consumer Affairs (Spain). Spain Statistics on Health Establishments Providing Inpatient Care 2008. Madrid, Spain: Ministry of Health, Social Services and Equality (Spain)
Ministry of Health and Consumer Affairs (Spain). Spain Statistics on Health Establishments Providing Inpatient Care 2009. Madrid, Spain: Ministry of Health, Social Services and Equality (Spain)
Ministry of Health, Social Services and Equality (Spain). Spain Statistics on Specialized Healthcare Centers 2010. Madrid, Spain: Ministry of Health, Social Services and Equality (Spain)
Ministry of Health, Social Services and Equality (Spain). Spain Statistics on Specialized Healthcare Centers 2011. Madrid, Spain: Ministry of Health, Social Services and Equality (Spain)
Ministry of Health, Social Services and Equality (Spain). Spain Statistics on Specialized Healthcare Centers 2012. Madrid, Spain: Ministry of Health, Social Services and Equality (Spain)
Ministry of Health, Social Services and Equality (Spain). Spain Statistics on Specialized Healthcare Centers 2013. Madrid, Spain: Ministry of Health, Social Services and Equality (Spain)
Ministry of Health, Social Services and Equality (Spain). Spain Statistics on Specialized Healthcare Centers 2014. Madrid, Spain: Ministry of Health, Social Services and Equality (Spain)
National Center for Health Statistics, Centers for Disease Control and Prevention, SRA International, Inc., United States Census Bureau. United States National Hospital Ambulatory Medical Care Survey 2014. Hyattsville, United States: National Center for Health Statistics, Centers for Disease Control and Prevention
Ministry of Health (Romania). Romania Hospital Inpatient Discharges 1985
Ministry of Health (Romania). Romania Hospital Inpatient Discharges 1986
Ministry of Health (Romania). Romania Hospital Inpatient Discharges 1987
Ministry of Health (Romania). Romania Hospital Inpatient Discharges 1988
Ministry of Health (Romania). Romania Hospital Inpatient Discharges 1989
Ministry of Health (Romania). Romania Hospital Inpatient Discharges 1990
Ministry of Health (Romania). Romania Hospital Inpatient Discharges 1991
Ministry of Health (Romania). Romania Hospital Inpatient Discharges 1992
Ministry of Health (Romania). Romania Hospital Inpatient Discharges 1993
Ministry of Health (Romania). Romania Hospital Inpatient Discharges 1994
Ministry of Health (Romania). Romania Hospital Inpatient Discharges 1995
Ministry of Health (Romania). Romania Hospital Inpatient Discharges 1996
Ministry of Health (Romania). Romania Hospital Inpatient Discharges 1997
Ministry of Health (Romania). Romania Hospital Inpatient Discharges 1998
Ministry of Health (Romania). Romania Hospital Inpatient Discharges 1999
Ministry of Health (Romania). Romania Hospital Inpatient Discharges 2013
Accident Compensation Corporation (New Zealand). New Zealand Accident Compensation Corporation Injury Claims 2015
Accident Compensation Corporation (New Zealand). New Zealand Accident Compensation Corporation Injury Claims 2016
Accident Compensation Corporation (New Zealand). New Zealand Accident Compensation Corporation Injury Claims 2017
Accident Compensation Corporation (New Zealand). New Zealand Accident Compensation Corporation Injury Claims 2014
Accident Compensation Corporation (New Zealand). New Zealand Accident Compensation Corporation Injury Claims 2013
Accident Compensation Corporation (New Zealand). New Zealand Accident Compensation Corporation Injury Claims 2012
Accident Compensation Corporation (New Zealand). New Zealand Accident Compensation Corporation Injury Claims 2011
Accident Compensation Corporation (New Zealand). New Zealand Accident Compensation Corporation Injury Claims 2010
Accident Compensation Corporation (New Zealand). New Zealand Accident Compensation Corporation Injury Claims 2009
Accident Compensation Corporation (New Zealand). New Zealand Accident Compensation Corporation Injury Claims 2008
Bureau of Statistics (Guyana), World Bank. Guyana Living Standards Measurement Survey 1992-1993
Ministry of Statistics and Programme Implementation (India). India National Sample Survey Round 52 1995-1996. New Delhi, India: Ministry of Statistics and Programme Implementation (India)
Ministry of Statistics and Programme Implementation (India). India National Sample Survey Round 60 2004. New Delhi, India: Ministry of Statistics and Programme Implementation (India)
National Institute of Statistics and Informatics (Peru), World Bank (WB). Peru Living Standards Measurement Survey 1990
National Institute of Statistics and Informatics (Peru), World Bank. Peru Living Standards Measurement Survey 1991. Washington DC, United States: World Bank
National Institute of Statistics and Informatics (Peru), World Bank (WB). Peru Living Standards Measurement Survey 1994
Ministry of Social Affairs (Serbia), World Bank. Yugoslavia, Federal Republic - Serbia Living Standards Measurement Survey 2002. Washington DC, United States: World Bank
Ministry of Social Affairs (Serbia), World Bank. Serbia and Montenegro - Serbia Living Standards Measurement Survey 2003. Washington DC, United States: World Bank
International Institute for Population Sciences (India), World Health Organization (WHO). India World Health Survey 2003. Geneva, Switzerland: World Health Organization (WHO), 2005
World Health Organization (WHO). Malaysia World Health Survey 2003. Geneva, Switzerland: World Health Organization (WHO), 2005
World Health Organization (WHO). Mauritius World Health Survey 2003. Geneva, Switzerland: World Health Organization (WHO), 2005
World Health Organization (WHO). Sweden World Health Survey 2003. Geneva, Switzerland: World Health Organization (WHO), 2005
World Health Organization (WHO). Ukraine World Health Survey 2002-2003. Geneva, Switzerland: World Health Organization (WHO), 2005
National Institute of Statistics and Informatics (INEI) (Peru), United Nations Economic Commission for Latin America and the Caribbean (CEPAL), Institute of Research for Development (France). Peru National Household Survey 2007. Lima, Peru: National Institute of Statistics and Informatics (INEI) (Peru)
National Institute of Statistics and Informatics (INEI) (Peru), United Nations Economic Commission for Latin America and the Caribbean (CEPAL), Institute of Research for Development (France). Peru National Household Survey 2008. Lima, Peru: National Institute of Statistics and Informatics (INEI) (Peru)
National Institute of Statistics and Informatics (INEI) (Peru). Peru National Household Survey 2009. Lima, Peru: National Institute of Statistics and Informatics (INEI) (Peru)
National Institute of Statistics and Informatics (INEI) (Peru). Peru National Household Survey 2010. Lima, Peru: National Institute of Statistics and Informatics (INEI) (Peru)
National Institute of Statistics and Informatics (INEI) (Peru). Peru National Household Survey, Second Quarter 1998. Lima, Peru: National Institute of Statistics and Informatics (INEI) (Peru)
National Institute of Statistics and Informatics (INEI) (Peru). Peru National Household Survey, Second Quarter 1999. Lima, Peru: National Institute of Statistics and Informatics (INEI) (Peru)
National Institute of Statistics (Albania), World Bank (WB). Albania Living Standards Measurement Survey 2002. Washington DC, United States: World Bank (WB)
National Institute of Statistics (Albania), World Bank (WB). Albania Living Standards Measurement Survey 2005. Washington DC, United States: World Bank (WB)
National Institute of Statistics and Informatics (INEI) (Peru). Peru National Household Survey, Second Quarter 2000. Lima, Peru: National Institute of Statistics and Informatics (INEI) (Peru)
Ministry of Social Affairs (Serbia), World Bank. Serbia Living Standards Measurement Survey 2007. Washington DC, United States: World Bank

National Institute of Statistics and Informatics (INEI) (Peru). Peru National Household Survey 2005. Lima, Peru: National Institute of Statistics and Informatics (INEI) (Peru)
National Institute of Statistics and Informatics (INEI) (Peru). Peru National Household Survey 2006. Lima, Peru: National Institute of Statistics and Informatics (INEI) (Peru)
International Institute for Population Sciences (India), World Health Organization (WHO). India WHO Study on Global Ageing and Adult Health 2007. Geneva, Switzerland: World Health Organization (WHO), 2007
Ministry of Health (Syria), World Health Organization (WHO). Syria WHO Multi-country Survey Study on Health and Health System Responsiveness 2000-2001
National Institute of Statistics (Niger), World Bank. Niger National Survey on Household Living Conditions and Agriculture 2011-2012
Statistics Sweden, Swedish National Institute of Public Health. Sweden National Survey of Public Health 2009
Statistics Sweden, Swedish National Institute of Public Health. Sweden National Survey of Public Health 2007
Institute for Public Health, Ministry of Health (Malaysia). Malaysia National Health And Morbidity Survey 2006. Kuala Lumpur, Malaysia: Institute for Public Health, Ministry of Health (Malaysia)
National Board of Health and Welfare (Sweden). Sweden National Patient Register 1998. Stockholm, Sweden: National Board of Health and Welfare (Sweden)
National Board of Health and Welfare (Sweden). Sweden National Patient Register 1999. Stockholm, Sweden: National Board of Health and Welfare (Sweden)
Statistics Sweden, Swedish National Institute of Public Health. Sweden National Survey of Public Health 2008
Maziak W, Ward KD, Rastam S. Injuries in Aleppo, Syria; first population-based estimates and characterization of predominant types. BMC Public Health. 2006; 63
Statistics Sweden, Swedish National Institute of Public Health. Sweden National Survey of Public Health 2012
Statistics Sweden, Swedish National Institute of Public Health. Sweden National Survey of Public Health 2010
Statistics Sweden, Swedish National Institute of Public Health. Sweden National Survey of Public Health 2004
Statistics Sweden, Swedish National Institute of Public Health. Sweden National Survey of Public Health 2005
Statistics Sweden, Swedish National Institute of Public Health. Sweden National Survey of Public Health 2011
Trinity College Dublin. Ireland Longitudinal Study on Ageing 2009-2011. Dublin, Ireland: Irish Social Science Data Archive, University College Dublin
Statistics Sweden, Swedish National Institute of Public Health. Sweden National Survey of Public Health 2013
Public Health Agency of Sweden, Statistics Sweden. Sweden National Survey of Public Health 2014
International Research Associates (INRA) Europe, World Health Organization (WHO). Iceland WHO Multi-country Survey Study on Health and Health System Responsiveness 2000-2001. Geneva, Switzerland: World Health Organization (WHO)
International Research Associates (INRA) Europe, World Health Organization (WHO). Sweden WHO Multi-country Survey Study on Health and Health System Responsiveness 2000-2001. Geneva, Switzerland: World Health Organization (WHO)
Kiev International Institute of Sociology, World Health Organization (WHO). Ukraine WHO Multi-country Survey Study on Health and Health System Responsiveness 2000-2001. Geneva, Switzerland: World Health Organization (WHO)
Gallup, World Health Organization (WHO). Venezuela WHO Multi-country Survey Study on Health and Health System Responsiveness 2000-2001. Geneva, Switzerland: World Health Organization (WHO)
Börsch-Supan, A. (2015). Survey of Health, Ageing and Retirement in Europe (SHARE) Wave 5. Release version: 1.0.0. SHARE-ERIC. Data set. DOI: 10.6103/SHARE.w5.100
Government of India, Ministry of Statistics and Programme Implementation (India). India National Sample Survey Round 71 2014. New Delhi, India: Ministry of Statistics and Programme Implementation (India)
Armenian Sociological Association, Concluzia-Prim Center for Survey Methodology (Moldova), Institute for Advanced Studies (Austria), London School of Hygiene and Tropical Medicine, University of Aberdeen. Armenia Health in Times of Transition Household Survey 2010
Concluzia-Prim Center for Survey Methodology (Moldova), East-Ukranian Foundation For Social Research, Institute for Advanced Studies (Austria), London School of Hygiene and Tropical Medicine, University of Aberdeen. Ukraine Health in Times of Transition Household Survey 2010
Institute for Public Health, Ministry of Health (Malaysia). Malaysia National Health and Morbidity Survey 2011
Institute of Public Health of Serbia. Serbia National Hospital Discharge Database 2012
Department of Health, Social Services and Public Safety (Northern Ireland), Information Centre for Health and Social Care, NHS, NHS England, NHS Health Scotland, NHS Wales. United Kingdom Hospital Patient and Discharge Data 2006
Directorate of Health (Iceland). Iceland Hospital Data Registry 2008
Dandona R, Kumar GA, Ameer MA, Ahmed GM, Dandona L. Incidence and burden of road traffic injuries in urban India [Unpublished data]. Inj Prev. 2008; 14(6): 354-9
Mirkazemi R, Kar A. A population-based study on road traffic injuries in Pune City, India [Unpublished data]. Traffic Inj Prev. 2014; 15(4): 379-85
Ministry of Health (Albania). Albania Inpatient Care Discharges per 100 1993
Ministry of Health (Albania). Albania Inpatient Care Discharges per 100 1994
Ministry of Health (Albania). Albania Inpatient Care Discharges per 100 1995
Ministry of Health (Albania). Albania Inpatient Care Discharges per 100 1996
Ministry of Health (Albania). Albania Inpatient Care Discharges per 100 1997
Ministry of Health (Albania). Albania Inpatient Care Discharges per 100 1998
Ministry of Health (Albania). Albania Inpatient Care Discharges per 100 1999
Ministry of Health (Albania). Albania Inpatient Care Discharges per 100 2000
Ministry of Health (Albania). Albania Inpatient Care Discharges per 100 2001
Ministry of Health (Albania). Albania Inpatient Care Discharges per 100 2002
Ministry of Health (Albania). Albania Inpatient Care Discharges per 100 2003
Ministry of Health (Albania). Albania Inpatient Care Discharges per 100 2004
Ministry of Health (Albania). Albania Inpatient Care Discharges per 100 2005
Ministry of Health (Albania). Albania Inpatient Care Discharges per 100 2006
Ministry of Health (Albania). Albania Inpatient Care Discharges per 100 2007
Ministry of Health (Albania). Albania Inpatient Care Discharges per 100 2008
Ministry of Health (Albania). Albania Inpatient Care Discharges per 100 2009
Ministry of Health (Albania). Albania Inpatient Care Discharges per 100 2010
Ministry of Health (Albania). Albania Inpatient Care Discharges per 100 2011
Ministry of Health (Albania). Albania Inpatient Care Discharges per 100 2012
Ministry of Health (Albania). Albania Inpatient Care Discharges per 100 2013
Directorate of Health (Iceland). Iceland Hospital Data Registry 2010
Directorate of Health (Iceland). Iceland Hospital Data Registry 2011
Directorate of Health (Iceland). Iceland Hospital Data Registry 2012
Directorate of Health (Iceland). Iceland Hospital Data Registry 2013
Directorate of Health (Iceland). Iceland Hospital Data Registry 2014
Directorate of Health (Iceland). Iceland Hospital Data Registry 1999
Directorate of Health (Iceland). Iceland Hospital Data Registry 1995
Directorate of Health (Iceland). Iceland Hospital Data Registry 1994
Directorate of Health (Iceland). Iceland Hospital Data Registry 1993
Directorate of Health (Iceland). Iceland Hospital Data Registry 1992
Directorate of Health (Iceland). Iceland Hospital Data Registry 1991
Directorate of Health (Iceland). Iceland Hospital Data Registry 1990
Directorate of Health (Iceland). Iceland Hospital Data Registry 1989
Directorate of Health (Iceland). Iceland Hospital Data Registry 1988
Ministry of Health (Armenia). Armenia Inpatient Care Discharges per 100 1980
Ministry of Health (Armenia). Armenia Inpatient Care Discharges per 100 1981
Ministry of Health (Armenia). Armenia Inpatient Care Discharges per 100 1982
Ministry of Health (Armenia). Armenia Inpatient Care Discharges per 100 1983
Ministry of Health (Armenia). Armenia Inpatient Care Discharges per 100 1984
Ministry of Health (Armenia). Armenia Inpatient Care Discharges per 100 1985
Ministry of Health (Armenia). Armenia Inpatient Care Discharges per 100 1986
Ministry of Health (Armenia). Armenia Inpatient Care Discharges per 100 1987
Ministry of Health (Armenia). Armenia Inpatient Care Discharges per 100 1988
Ministry of Health (Armenia). Armenia Inpatient Care Discharges per 100 1989
Ministry of Health (Armenia). Armenia Inpatient Care Discharges per 100 1990
Ministry of Health (Armenia). Armenia Inpatient Care Discharges per 100 1991
Ministry of Health (Armenia). Armenia Inpatient Care Discharges per 100 1992
Ministry of Health (Armenia). Armenia Inpatient Care Discharges per 100 1993
Ministry of Health (Armenia). Armenia Inpatient Care Discharges per 100 1994
Ministry of Health (Armenia). Armenia Inpatient Care Discharges per 100 1995
Ministry of Health (Armenia). Armenia Inpatient Care Discharges per 100 1996
Ministry of Health (Armenia). Armenia Inpatient Care Discharges per 100 1997
Ministry of Health (Armenia). Armenia Inpatient Care Discharges per 100 1998
Ministry of Health (Armenia). Armenia Inpatient Care Discharges per 100 1999
Ministry of Health (Armenia). Armenia Inpatient Care Discharges per 100 2000
Ministry of Health (Armenia). Armenia Inpatient Care Discharges per 100 2001
Ministry of Health (Armenia). Armenia Inpatient Care Discharges per 100 2002
Ministry of Health (Armenia). Armenia Inpatient Care Discharges per 100 2003
Ministry of Health (Armenia). Armenia Inpatient Care Discharges per 100 2004
Ministry of Health (Armenia). Armenia Inpatient Care Discharges per 100 2005
Ministry of Health (Armenia). Armenia Inpatient Care Discharges per 100 2006
Ministry of Health (Armenia). Armenia Inpatient Care Discharges per 100 2007
Ministry of Health (Armenia). Armenia Inpatient Care Discharges per 100 2008
Ministry of Health (Armenia). Armenia Inpatient Care Discharges per 100 2009
Ministry of Health (Armenia). Armenia Inpatient Care Discharges per 100 2010
Ministry of Health (Armenia). Armenia Inpatient Care Discharges per 100 2011
Ministry of Health (Armenia). Armenia Inpatient Care Discharges per 100 2012
Ministry of Health (Armenia). Armenia Inpatient Care Discharges per 100 2013
Ministry of Health (Armenia). Armenia Inpatient Care Discharges per 100 2014
Center for Health Statistics, Ministry of Health (Ukraine). Ukraine Inpatient Care Discharges per 100 1980
Center for Health Statistics, Ministry of Health (Ukraine). Ukraine Inpatient Care Discharges per 100 1981
Center for Health Statistics, Ministry of Health (Ukraine). Ukraine Inpatient Care Discharges per 100 1982
Center for Health Statistics, Ministry of Health (Ukraine). Ukraine Inpatient Care Discharges per 100 1983
Center for Health Statistics, Ministry of Health (Ukraine). Ukraine Inpatient Care Discharges per 100 1984
Center for Health Statistics, Ministry of Health (Ukraine). Ukraine Inpatient Care Discharges per 100 1985
Center for Health Statistics, Ministry of Health (Ukraine). Ukraine Inpatient Care Discharges per 100 1986
Center for Health Statistics, Ministry of Health (Ukraine). Ukraine Inpatient Care Discharges per 100 1987
Center for Health Statistics, Ministry of Health (Ukraine). Ukraine Inpatient Care Discharges per 100 1988
Center for Health Statistics, Ministry of Health (Ukraine). Ukraine Inpatient Care Discharges per 100 1989
Center for Health Statistics, Ministry of Health (Ukraine). Ukraine Inpatient Care Discharges per 100 1990
Center for Health Statistics, Ministry of Health (Ukraine). Ukraine Inpatient Care Discharges per 100 1991
Center for Health Statistics, Ministry of Health (Ukraine). Ukraine Inpatient Care Discharges per 100 1992
Center for Health Statistics, Ministry of Health (Ukraine). Ukraine Inpatient Care Discharges per 100 1993
Center for Health Statistics, Ministry of Health (Ukraine). Ukraine Inpatient Care Discharges per 100 1994
Center for Health Statistics, Ministry of Health (Ukraine). Ukraine Inpatient Care Discharges per 100 1995
Center for Health Statistics, Ministry of Health (Ukraine). Ukraine Inpatient Care Discharges per 100 1996
Center for Health Statistics, Ministry of Health (Ukraine). Ukraine Inpatient Care Discharges per 100 1997
Center for Health Statistics, Ministry of Health (Ukraine). Ukraine Inpatient Care Discharges per 100 1998
Center for Health Statistics, Ministry of Health (Ukraine). Ukraine Inpatient Care Discharges per 100 1999
Center for Health Statistics, Ministry of Health (Ukraine). Ukraine Inpatient Care Discharges per 100 2000
Center for Health Statistics, Ministry of Health (Ukraine). Ukraine Inpatient Care Discharges per 100 2001
Center for Health Statistics, Ministry of Health (Ukraine). Ukraine Inpatient Care Discharges per 100 2002
Center for Health Statistics, Ministry of Health (Ukraine). Ukraine Inpatient Care Discharges per 100 2003
Center for Health Statistics, Ministry of Health (Ukraine). Ukraine Inpatient Care Discharges per 100 2004
Center for Health Statistics, Ministry of Health (Ukraine). Ukraine Inpatient Care Discharges per 100 2005
Center for Health Statistics, Ministry of Health (Ukraine). Ukraine Inpatient Care Discharges per 100 2006
Center for Health Statistics, Ministry of Health (Ukraine). Ukraine Inpatient Care Discharges per 100 2007
Center for Health Statistics, Ministry of Health (Ukraine). Ukraine Inpatient Care Discharges per 100 2008
Center for Health Statistics, Ministry of Health (Ukraine). Ukraine Inpatient Care Discharges per 100 2009
Center for Health Statistics, Ministry of Health (Ukraine). Ukraine Inpatient Care Discharges per 100 2010
Center for Health Statistics, Ministry of Health (Ukraine). Ukraine Inpatient Care Discharges per 100 2011
Center for Health Statistics, Ministry of Health (Ukraine). Ukraine Inpatient Care Discharges per 100 2012
Center for Health Statistics, Ministry of Health (Ukraine). Ukraine Inpatient Care Discharges per 100 2013
Institute of Public Health of Serbia. Serbia Inpatient Care Discharges Per 100 2010
Institute of Public Health of Serbia. Serbia Inpatient Care Discharges Per 100 2011
Institute of Public Health of Serbia. Serbia Inpatient Care Discharges Per 100 2013
Institute of Public Health of Serbia. Serbia Inpatient Care Discharges Per 100 2014
Department of Health, Social Services and Public Safety (Northern Ireland), Information Centre for Health and Social Care, NHS, NHS England, NHS Health Scotland, NHS Wales. United Kingdom Hospital Patient and Discharge Data 2012
Department of Health, Social Services and Public Safety (Northern Ireland), Information Centre for Health and Social Care, NHS, NHS England, NHS Health Scotland, NHS Wales. United Kingdom Hospital Patient and Discharge Data 2013
Department of Health, Social Services and Public Safety (Northern Ireland), Information Centre for Health and Social Care, NHS, NHS England, NHS Health Scotland, NHS Wales. United Kingdom Hospital Patient and Discharge Data 2014
National Board of Health and Welfare (Sweden). Sweden National Patient Register 1997. Stockholm, Sweden: National Board of Health and Welfare (Sweden)
National Board of Health and Welfare (Sweden). Sweden National Patient Register 1996. Stockholm, Sweden: National Board of Health and Welfare (Sweden)
National Board of Health and Welfare (Sweden). Sweden National Patient Register 1995. Stockholm, Sweden: National Board of Health and Welfare (Sweden)
National Board of Health and Welfare (Sweden). Sweden National Patient Register 1994. Stockholm, Sweden: National Board of Health and Welfare (Sweden)
National Board of Health and Welfare (Sweden). Sweden National Patient Register 1993. Stockholm, Sweden: National Board of Health and Welfare (Sweden)
National Board of Health and Welfare (Sweden). Sweden National Patient Register 1992. Stockholm, Sweden: National Board of Health and Welfare (Sweden)
National Board of Health and Welfare (Sweden). Sweden National Patient Register 1991. Stockholm, Sweden: National Board of Health and Welfare (Sweden)
National Board of Health and Welfare (Sweden). Sweden National Patient Register 1990. Stockholm, Sweden: National Board of Health and Welfare (Sweden)
National Board of Health and Welfare (Sweden). Sweden National Patient Register 1989. Stockholm, Sweden: National Board of Health and Welfare (Sweden)
National Board of Health and Welfare (Sweden). Sweden National Patient Register 1988. Stockholm, Sweden: National Board of Health and Welfare (Sweden)
National Board of Health and Welfare (Sweden). Sweden National Patient Register 1987. Stockholm, Sweden: National Board of Health and Welfare (Sweden)
National Board of Health and Welfare (Sweden). Sweden National Patient Register 2013. Stockholm, Sweden: National Board of Health and Welfare (Sweden)
National Board of Health and Welfare (Sweden). Sweden National Patient Register 2014. Stockholm, Sweden: National Board of Health and Welfare (Sweden)
World Health Organization Regional Office for Europe (WHO/Europe). European Hospital Morbidity Database, 2008. Copenhagen, Denmark: World Health Organization Regional Office for Europe (WHO/Europe)
Macro International, Inc, National Institute of Statistics (INE) (Mozambique). Mozambique Demographic and Health Survey 2003-2004. Fairfax, United States: ICF International

World Health Organization (WHO). Bosnia and Herzegovina World Health Survey 2003. Geneva, Switzerland: World Health Organization (WHO), 2005
World Health Organization (WHO). Chad World Health Survey 2003. Geneva, Switzerland: World Health Organization (WHO), 2005
World Health Organization (WHO). Ireland World Health Survey 2003. Geneva, Switzerland: World Health Organization (WHO), 2005
World Health Organization (WHO). Nepal World Health Survey 2003. Geneva, Switzerland: World Health Organization (WHO), 2005
World Health Organization (WHO). Slovakia World Health Survey 2003. Geneva, Switzerland: World Health Organization (WHO), 2005
World Health Organization (WHO). Tunisia World Health Survey 2003. Geneva, Switzerland: World Health Organization (WHO), 2005
Centers for Disease Control and Prevention (CDC), Ministry of Health (Jordan), World Health Organization (WHO). Jordan STEPS Noncommunicable Disease Risk Factors Survey 2007
Agency for Statistics (Bosnia and Herzegovina), Institute of Statistics (Republic of Srpska), Federal Office of Statistics (Bosnia and Herzegovina), Swedish International Development Agency (SIDA), UK Department for International Development (DFID), United Nations Development Programme (UNDP), European Commission (EC), Government of Japan, World Bank (WB). Bosnia a
Agency for Statistics (Bosnia and Herzegovina), Institute of Statistics (Republic of Srpska), Federal Office of Statistics (Bosnia and Herzegovina), Independent Bureau for Humanitarian Issues (IBHI), Birks Sinclair and Associates, LTD, Institute for Social and Economic Research, University of Essex. Bosnia and Herzegovina Living Standards Measurement Survey 2002. Washington, D
Agency for Statistics (Bosnia and Herzegovina), Birks Sinclair and Associates, LTD, Federal Office of Statistics (Federation of Bosnia and Herzegovina), Independent Bureau for Humanitarian Issues (IBHI), Institute for Social and Economic Research, University of Essex, Institute of Statistics (Republic of Srpska). Bosnia and Herzegovina Living Standards Measurement Survey 2004-2
Public Health Authority of the Slovak Republic, World Health Organization (WHO). Slovakia WHO Multi-country Survey Study on Health and Health System Responsiveness 2000
University of Ibadan (Nigeria), World Health Organization (WHO). Nigeria WHO Multi-country Survey Study on Health and Health System Responsiveness 2000-2001
Labinjo M, Juillard C, Kobusingye OC, Hyder AA. The burden of road traffic injuries in Nigeria: results of a population-based survey. Inj Prev. 2009; 15(3): 157-62
National Bureau of Statistics (Nigeria). Nigeria Living Standards Survey 2008-2010. Abuja, Nigeria: National Bureau of Statistics (Nigeria)
Trinity College Dublin. Ireland Longitudinal Study on Ageing 2012-2013. Dublin, Ireland: Irish Social Science Data Archive, University College Dublin
LINK Institute for Market and Social Research (Switzerland), World Health Organization (WHO). Switzerland WHO Multi-country Survey Study on Health and Health System Responsiveness 2000-2001. Geneva, Switzerland: World Health Organization (WHO)
International Research Associates (INRA) Europe, World Health Organization (WHO). Ireland WHO Multi-country Survey Study on Health and Health System Responsiveness 2000-2001. Geneva, Switzerland: World Health Organization (WHO)
Gallup, World Health Organization (WHO). Jordan WHO Multi-country Survey Study on Health and Health System Responsiveness 2000-2001. Geneva, Switzerland: World Health Organization (WHO)
Börsch-Supan, A. (2015). Survey of Health, Ageing and Retirement in Europe (SHARE) Wave 5. Release version: 1.0.0. SHARE-ERIC. Data set. DOI: 10.6103/SHARE.w5.100
Concluzia-Prim Center for Survey Methodology (Moldova), Institute for Advanced Studies (Austria), London School of Hygiene and Tropical Medicine, SIAR Research and Consulting (Azerbaijan), University of Aberdeen. Azerbaijan Health in Times of Transition Household Survey 2010
Department of Health Services, Ministry of Health and Population (Nepal). Nepal Hospital Inpatient Discharges 2010-2012
Department of Health and Children (Ireland), Economic and Social Research Institute (ESRI) (Ireland). Ireland Hospital Inpatient Enquiry 2000
Department of Health (Ireland), Economic and Social Research Institute (ESRI) (Ireland). Ireland Hospital Inpatient Enquiry 1980
Department of Health (Ireland), Economic and Social Research Institute (ESRI) (Ireland). Ireland Hospital Inpatient Enquiry 1981
Department of Health (Ireland), Economic and Social Research Institute (ESRI) (Ireland). Ireland Hospital Inpatient Enquiry 1982
Department of Health (Ireland), Economic and Social Research Institute (ESRI) (Ireland). Ireland Hospital Inpatient Enquiry 1983
Department of Health (Ireland), Economic and Social Research Institute (ESRI) (Ireland). Ireland Hospital Inpatient Enquiry 1984
Department of Health (Ireland), Economic and Social Research Institute (ESRI) (Ireland). Ireland Hospital Inpatient Enquiry 1985
Department of Health (Ireland), Economic and Social Research Institute (ESRI) (Ireland). Ireland Hospital Inpatient Enquiry 1986
Department of Health (Ireland), Economic and Social Research Institute (ESRI) (Ireland). Ireland Hospital Inpatient Enquiry 1987
Department of Health (Ireland), Economic and Social Research Institute (ESRI) (Ireland). Ireland Hospital Inpatient Enquiry 1988
Department of Health (Ireland), Economic and Social Research Institute (ESRI) (Ireland). Ireland Hospital Inpatient Enquiry 1989
Department of Health (Ireland), Economic and Social Research Institute (ESRI) (Ireland). Ireland Hospital Inpatient Enquiry 1990
Department of Health (Ireland), Economic and Social Research Institute (ESRI) (Ireland). Ireland Hospital Inpatient Enquiry 1991
Department of Health (Ireland), Economic and Social Research Institute (ESRI) (Ireland). Ireland Hospital Inpatient Enquiry 1992
Department of Health (Ireland), Economic and Social Research Institute (ESRI) (Ireland). Ireland Hospital Inpatient Enquiry 1993
Department of Health (Ireland), Economic and Social Research Institute (ESRI) (Ireland). Ireland Hospital Inpatient Enquiry 1994
Department of Health (Ireland), Economic and Social Research Institute (ESRI) (Ireland). Ireland Hospital Inpatient Enquiry 1995
Department of Health (Ireland), Economic and Social Research Institute (ESRI) (Ireland). Ireland Hospital Inpatient Enquiry 1996
Department of Health and Children (Ireland), Economic and Social Research Institute (ESRI) (Ireland). Ireland Hospital Inpatient Enquiry 1997
Department of Health and Children (Ireland), Economic and Social Research Institute (ESRI) (Ireland). Ireland Hospital Inpatient Enquiry 1998
Department of Health and Children (Ireland), Economic and Social Research Institute (ESRI) (Ireland). Ireland Hospital Inpatient Enquiry 1999
Economic and Social Research Institute (ESRI) (Ireland), Health Service Executive (HSE) (Ireland). Ireland Hospital Inpatient Enquiry 2013
Economic and Social Research Institute (ESRI) (Ireland), Health Service Executive (HSE) (Ireland). Ireland Hospital Inpatient Enquiry 2014
Federal Statistical Office (Switzerland). Switzerland Medical Statistics of Hospitals 1997
Federal Statistical Office (Switzerland). Switzerland Medical Statistics of Hospitals 1998
Federal Statistical Office (Switzerland). Switzerland Medical Statistics of Hospitals 2013
Federal Statistical Office (Switzerland). Switzerland Medical Statistics of Hospitals 2014
National Statistical Institute of Bulgaria. Bulgaria Living Standards Measurement Survey 2003. Washington DC, United States: World Bank
Administrative Department of Science, Technology, and Innovation (Colombia), Center for Development Projects, Pontifical Xavierian University, Ministry of Social Protection (Colombia), Specialized Information Systems. Colombia National Health Survey 2007-2008
Planning Institute of Jamaica, Statistical Institute of Jamaica. Jamaica Survey of Living Conditions 1988. Kingston, Jamaica: Planning Institute of Jamaica
Statistical Institute of Jamaica. Jamaica Survey of Living Conditions 1990. Washington DC, United States: World Bank
Planning Institute of Jamaica, Statistical Institute of Jamaica. Jamaica Survey of Living Conditions 1992
Planning Institute of Jamaica, Statistical Institute of Jamaica. Jamaica Survey of Living Conditions 1991
World Health Organization (WHO). Austria World Health Survey 2003. Geneva, Switzerland: World Health Organization (WHO), 2005
Center for Scientific and Technological Information, Oswaldo Cruz Foundation and World Health Organization (WHO). Brazil World Health Survey 2003. Geneva, Switzerland: World Health Organization (WHO), 2005
World Health Organization (WHO). Côte d'Ivoire World Health Survey 2003. Geneva, Switzerland: World Health Organization (WHO), 2005
World Health Organization (WHO). Georgia World Health Survey 2003. Geneva, Switzerland: World Health Organization (WHO), 2005
World Health Organization (WHO). Israel World Health Survey 2003. Geneva, Switzerland: World Health Organization (WHO), 2005
World Health Organization (WHO). Myanmar World Health Survey 2003. Geneva, Switzerland: World Health Organization (WHO), 2005
World Health Organization (WHO). Namibia World Health Survey 2003. Geneva, Switzerland: World Health Organization (WHO), 2005
World Health Organization (WHO). Pakistan World Health Survey 2003-2004. Geneva, Switzerland: World Health Organization (WHO), 2005
World Health Organization (WHO). Slovenia World Health Survey 2003. Geneva, Switzerland: World Health Organization (WHO), 2005
World Health Organization (WHO). Turkey World Health Survey 2003. Geneva, Switzerland: World Health Organization (WHO), 2005
Planning Institute of Jamaica, Statistical Institute of Jamaica. Jamaica Survey of Living Conditions 1993
Planning Institute of Jamaica, Statistical Institute of Jamaica. Jamaica Survey of Living Conditions 1994
Planning Institute of Jamaica, Statistical Institute of Jamaica. Jamaica Survey of Living Conditions 1995
Planning Institute of Jamaica, Statistical Institute of Jamaica. Jamaica Survey of Living Conditions 1996
Planning Institute of Jamaica, Statistical Institute of Jamaica. Jamaica Survey of Living Conditions 1997
Planning Institute of Jamaica, Statistical Institute of Jamaica. Jamaica Survey of Living Conditions 1999
Planning Institute of Jamaica, Statistical Institute of Jamaica. Jamaica Survey of Living Conditions 2000
TNS BBSS, World Bank. Bulgaria Living Standards Measurement Survey 2001. Washington DC, United States: World Bank
National Administrative Department of Statistics (Colombia). Colombia National Quality of Life Survey 1997. Bogotá, Colombia: National Administrative Department of Statistics (Colombia)
National Administrative Department of Statistics (Colombia). Colombia National Quality of Life Survey 2008. Bogotá, Colombia: National Administrative Department of Statistics (Colombia)
National Administrative Department of Statistics (Colombia). Colombia National Quality of Life Survey 2010. Bogotá, Colombia: National Administrative Department of Statistics (Colombia), 2012
Healthcare Cost and Utilization Project (HCUP), Agency for Healthcare Research and Quality (AHRQ). United States State Inpatient Databases 2009. Rockville, United States: Healthcare Cost and Utilization Project (HCUP), Agency for Healthcare Research and Quality (AHRQ)
Pontifical Xavierian University, World Health Organization (WHO). Colombia WHO Multi-country Survey Study on Health and Health System Responsiveness 2000-2001. Geneva, Switzerland: World Health Organization (WHO)
AMATEM (Turkey), Plaza Ltd. Research, World Health Organization (WHO). Turkey WHO Multi-country Survey Study on Health and Health System Responsiveness 2000-2001. Geneva, Switzerland: World Health Organization (WHO)
Institute for Polling and Marketing (Georgia), World Health Organization (WHO). Georgia WHO Multi-country Survey Study on Health and Health System Responsiveness 2000-2001
Turkish Statistical Institute. Turkey Health Interview Survey 2010. Ankara, Turkey: Turkish Statistical Institute
Statistics Austria, World Health Organization (WHO). Austria WHO Multi-country Survey Study on Health and Health System Responsiveness 2000-2001. Geneva, Switzerland: World Health Organization (WHO)
Federal Ministry of Health (Austria), Statistics Austria. Austria Hospital Inpatient Discharges 1989. Vienna, Austria: Statistics Austria
Fatmi Z, Hadden WC, Razzak JA, Qureshi HI, Hyder AA, Pappas G. Incidence, patterns and severity of reported unintentional injuries in Pakistan for persons five years and older: results of the National Health Survey of Pakistan 1990-94. BMC Public Health. 2007; 152
Eurostat, Ministry of Health (Bulgaria), National Statistical Institute of Bulgaria. Bulgaria European Health Interview Survey 2008
National Institute of Statistics of Rwanda. Rwanda Integrated Household Living Conditions Survey 2010-2011. Kigali, Rwanda: National Institute of Statistics of Rwanda
Health Institute (São Paulo, Brazil), State University of Campinas, São Paulo Municipal Health Department, São Paulo State University, University of São Paulo. Brazil - São Paulo Health Survey 2008-2009
Federal Ministry of Health (Austria), Statistics Austria. Austria Hospital Inpatient Discharges 2013. Vienna, Austria: Statistics Austria
International Research Associates (INRA) Europe, World Health Organization (WHO). Bulgaria WHO Multi-country Survey Study on Health and Health System Responsiveness 2000-2001. Geneva, Switzerland: World Health Organization (WHO)
Börsch-Supan, A. (2015). Survey of Health, Ageing and Retirement in Europe (SHARE) Wave 5. Release version: 1.0.0. SHARE-ERIC. Data set. DOI: 10.6103/SHARE.w5.100
Börsch-Supan, A. (2015). Survey of Health, Ageing and Retirement in Europe (SHARE) Wave 5. Release version: 1.0.0. SHARE-ERIC. Data set. DOI: 10.6103/SHARE.w5.100
Börsch-Supan, A. (2015). Survey of Health, Ageing and Retirement in Europe (SHARE) Wave 5. Release version: 1.0.0. SHARE-ERIC. Data set. DOI: 10.6103/SHARE.w5.100
Concluzia-Prim Center for Survey Methodology (Moldova), Georgia Opinion Research Business International (GORBI), Institute for Advanced Studies (Austria), London School of Hygiene and Tropical Medicine, University of Aberdeen. Georgia Health in Times of Transition Household Survey 2010
Ministry of Labor and Social Policy (Bulgaria), National Statistical Institute of Bulgaria, TNS Gallup, World Bank. Bulgaria Multitopic Household Survey 2007. Washington DC, United States: World Bank
Federal Ministry of Health (Austria), Statistics Austria. Austria Hospital Inpatient Discharges 2014. Vienna, Austria: Statistics Austria
National Institute of Public Health (Slovenia). Slovenia National Hospital Health Care Statistics Database 2004
National Institute of Public Health (Slovenia). Slovenia National Hospital Health Care Statistics Database 2005
National Institute of Public Health (Slovenia). Slovenia National Hospital Health Care Statistics Database 2006
National Institute of Public Health (Slovenia). Slovenia National Hospital Health Care Statistics Database 2007
National Institute of Public Health (Slovenia). Slovenia National Hospital Health Care Statistics Database 2008
General Directorate of Curative Services, Ministry of Health (Turkey). Turkey Hospital Inpatient Discharges 2007
General Directorate of Curative Services, Ministry of Health (Turkey). Turkey Hospital Inpatient Discharges 2008
General Directorate of Curative Services, Ministry of Health (Turkey). Turkey Hospital Inpatient Discharges 2009
General Directorate of Curative Services, Ministry of Health (Turkey). Turkey Hospital Inpatient Discharges 2010
General Directorate of Curative Services, Ministry of Health (Turkey). Turkey Hospital Inpatient Discharges 2011
National Center for Disease Control and Public Health (Georgia). Georgia Inpatient Care Discharges per 100 1991
National Center for Disease Control and Public Health (Georgia). Georgia Inpatient Care Discharges per 100 1992
National Center for Disease Control and Public Health (Georgia). Georgia Inpatient Care Discharges per 100 1993
National Center for Disease Control and Public Health (Georgia). Georgia Inpatient Care Discharges per 100 1994
National Center for Disease Control and Public Health (Georgia). Georgia Inpatient Care Discharges per 100 1995
National Center for Disease Control and Public Health (Georgia). Georgia Inpatient Care Discharges per 100 1996
National Center for Disease Control and Public Health (Georgia). Georgia Inpatient Care Discharges per 100 1997
National Center for Disease Control and Public Health (Georgia). Georgia Inpatient Care Discharges per 100 1998
National Center for Disease Control and Public Health (Georgia). Georgia Inpatient Care Discharges per 100 1999
National Center for Disease Control and Public Health (Georgia). Georgia Inpatient Care Discharges per 100 2000
National Center for Disease Control and Public Health (Georgia). Georgia Inpatient Care Discharges per 100 2001
National Center for Disease Control and Public Health (Georgia). Georgia Inpatient Care Discharges per 100 2002
National Center for Disease Control and Public Health (Georgia). Georgia Inpatient Care Discharges per 100 2003
National Center for Disease Control and Public Health (Georgia). Georgia Inpatient Care Discharges per 100 2004
National Center for Disease Control and Public Health (Georgia). Georgia Inpatient Care Discharges per 100 2005
National Center for Disease Control and Public Health (Georgia). Georgia Inpatient Care Discharges per 100 2006
National Center for Disease Control and Public Health (Georgia). Georgia Inpatient Care Discharges per 100 2007
National Center for Disease Control and Public Health (Georgia). Georgia Inpatient Care Discharges per 100 2008
National Center for Disease Control and Public Health (Georgia). Georgia Inpatient Care Discharges per 100 2009
National Center for Disease Control and Public Health (Georgia). Georgia Inpatient Care Discharges per 100 2010
National Center for Disease Control and Public Health (Georgia). Georgia Inpatient Care Discharges per 100 2011
National Center for Disease Control and Public Health (Georgia). Georgia Inpatient Care Discharges per 100 2012
National Center for Disease Control and Public Health (Georgia). Georgia Inpatient Care Discharges per 100 2013
National Center for Disease Control and Public Health (Georgia). Georgia Inpatient Care Discharges per 100 2014
Ministry of Health (Israel). Israel National Hospital Discharge Database 2011
Ministry of Health (Israel). Israel National Hospital Discharge Database 2012
Ministry of Health (Israel). Israel National Hospital Discharge Database 2013
Ministry of Health (Israel). Israel National Hospital Discharge Database 2014
National Center for Disease Control and Public Health (Georgia). Georgia Inpatient Care Discharges per 100 1980
National Center for Disease Control and Public Health (Georgia). Georgia Inpatient Care Discharges per 100 1985
National Center for Disease Control and Public Health (Georgia). Georgia Inpatient Care Discharges per 100 1986
National Center for Disease Control and Public Health (Georgia). Georgia Inpatient Care Discharges per 100 1987
National Center for Disease Control and Public Health (Georgia). Georgia Inpatient Care Discharges per 100 1988
National Center for Disease Control and Public Health (Georgia). Georgia Inpatient Care Discharges per 100 1989
National Center for Disease Control and Public Health (Georgia). Georgia Inpatient Care Discharges per 100 1990
General Directorate of Curative Services, Ministry of Health (Turkey). Turkey Inpatient Care Discharges per 100 1980
General Directorate of Curative Services, Ministry of Health (Turkey). Turkey Inpatient Care Discharges per 100 1981
General Directorate of Curative Services, Ministry of Health (Turkey). Turkey Inpatient Care Discharges per 100 1982
General Directorate of Curative Services, Ministry of Health (Turkey). Turkey Inpatient Care Discharges per 100 1983
General Directorate of Curative Services, Ministry of Health (Turkey). Turkey Inpatient Care Discharges per 100 1984
General Directorate of Curative Services, Ministry of Health (Turkey). Turkey Inpatient Care Discharges per 100 1985
General Directorate of Curative Services, Ministry of Health (Turkey). Turkey Inpatient Care Discharges per 100 1986
General Directorate of Curative Services, Ministry of Health (Turkey). Turkey Inpatient Care Discharges per 100 1987
General Directorate of Curative Services, Ministry of Health (Turkey). Turkey Inpatient Care Discharges per 100 1988
General Directorate of Curative Services, Ministry of Health (Turkey). Turkey Inpatient Care Discharges per 100 1989
General Directorate of Curative Services, Ministry of Health (Turkey). Turkey Inpatient Care Discharges per 100 1990
General Directorate of Curative Services, Ministry of Health (Turkey). Turkey Inpatient Care Discharges per 100 1991
General Directorate of Curative Services, Ministry of Health (Turkey). Turkey Inpatient Care Discharges per 100 1992
General Directorate of Curative Services, Ministry of Health (Turkey). Turkey Inpatient Care Discharges per 100 1993
General Directorate of Curative Services, Ministry of Health (Turkey). Turkey Inpatient Care Discharges per 100 1994
General Directorate of Curative Services, Ministry of Health (Turkey). Turkey Inpatient Care Discharges per 100 1995
General Directorate of Curative Services, Ministry of Health (Turkey). Turkey Inpatient Care Discharges per 100 1996
General Directorate of Curative Services, Ministry of Health (Turkey). Turkey Inpatient Care Discharges per 100 1997
General Directorate of Curative Services, Ministry of Health (Turkey). Turkey Inpatient Care Discharges per 100 1998
General Directorate of Curative Services, Ministry of Health (Turkey). Turkey Inpatient Care Discharges per 100 1999
General Directorate of Curative Services, Ministry of Health (Turkey). Turkey Inpatient Care Discharges per 100 2000

General Directorate of Curative Services, Ministry of Health (Turkey). Turkey Inpatient Care Discharges per 100 2001
General Directorate of Curative Services, Ministry of Health (Turkey). Turkey Inpatient Care Discharges per 100 2002
General Directorate of Curative Services, Ministry of Health (Turkey). Turkey Inpatient Care Discharges per 100 2003
General Directorate of Curative Services, Ministry of Health (Turkey). Turkey Inpatient Care Discharges per 100 2004
General Directorate of Curative Services, Ministry of Health (Turkey). Turkey Inpatient Care Discharges per 100 2005
General Directorate of Curative Services, Ministry of Health (Turkey). Turkey Inpatient Care Discharges per 100 2006
General Directorate of Curative Services, Ministry of Health (Turkey). Turkey Inpatient Care Discharges per 100 2012
General Directorate of Curative Services, Ministry of Health (Turkey). Turkey Inpatient Care Discharges per 100 2013
General Directorate of Curative Services, Ministry of Health (Turkey). Turkey Inpatient Care Discharges per 100 2014
National Institute of Public Health (Slovenia). Yugoslavia - Slovenia National Hospital Health Care Statistics Database 1980
National Institute of Public Health (Slovenia). Yugoslavia - Slovenia National Hospital Health Care Statistics Database 1986
National Institute of Public Health (Slovenia). Yugoslavia - Slovenia National Hospital Health Care Statistics Database 1985
National Institute of Public Health (Slovenia). Yugoslavia - Slovenia National Hospital Health Care Statistics Database 1987
National Institute of Public Health (Slovenia). Yugoslavia - Slovenia National Hospital Health Care Statistics Database 1988
National Institute of Public Health (Slovenia). Slovenia National Hospital Health Care Statistics Database 1989
National Institute of Public Health (Slovenia). Yugoslavia - Slovenia National Hospital Health Care Statistics Database 1990
National Institute of Public Health (Slovenia). Slovenia National Hospital Health Care Statistics Database 1991
National Institute of Public Health (Slovenia). Slovenia National Hospital Health Care Statistics Database 1992
National Institute of Public Health (Slovenia). Slovenia National Hospital Health Care Statistics Database 1993
National Institute of Public Health (Slovenia). Slovenia National Hospital Health Care Statistics Database 1994
National Institute of Public Health (Slovenia). Slovenia National Hospital Health Care Statistics Database 1995
National Institute of Public Health (Slovenia). Slovenia National Hospital Health Care Statistics Database 1996
National Institute of Public Health (Slovenia). Slovenia National Hospital Health Care Statistics Database 1997
National Institute of Public Health (Slovenia). Slovenia National Hospital Health Care Statistics Database 1998
National Institute of Public Health (Slovenia). Slovenia National Hospital Health Care Statistics Database 1999
National Institute of Public Health (Slovenia). Slovenia National Hospital Health Care Statistics Database 2000
National Institute of Public Health (Slovenia). Slovenia National Hospital Health Care Statistics Database 2001
National Institute of Public Health (Slovenia). Slovenia National Hospital Health Care Statistics Database 2002
National Institute of Public Health (Slovenia). Slovenia National Hospital Health Care Statistics Database 2003
National Institute of Public Health (Slovenia). Slovenia National Hospital Health Care Statistics Database 2013
National Institute of Public Health (Slovenia). Slovenia National Hospital Health Care Statistics Database 2014
National Center for Disease Control and Public Health (Georgia). Georgia Hospital Data 2013-2014
Bahamas Department of Statistics, Ministry of Health (Bahamas). Bahamas Living Conditions Survey 2001. Nassau, The Bahamas: Bahamas Department of Statistics
Agency of the Republic of Kazakhstan on Statistics, World Bank. Kazakhstan Living Standards Measurement Survey 1996. Washington DC, United States: World Bank
Central Statistical Service (South Africa). South Africa October Household Survey 1996
World Health Organization (WHO). Belgium World Health Survey 2002. Geneva, Switzerland: World Health Organization (WHO), 2005
World Health Organization (WHO). China World Health Survey 2002. Geneva, Switzerland: World Health Organization (WHO), 2005
World Health Organization (WHO). Comoros World Health Survey 2003. Geneva, Switzerland: World Health Organization (WHO), 2005
World Health Organization (WHO). Croatia World Health Survey 2003. Geneva, Switzerland: World Health Organization (WHO), 2005
World Health Organization (WHO). Italy World Health Survey 2003. Geneva, Switzerland: World Health Organization (WHO), 2005
World Health Organization (WHO). Kazakhstan World Health Survey 2002-2003. Geneva, Switzerland: World Health Organization (WHO), 2005
World Health Organization (WHO). Paraguay World Health Survey 2002-2003. Geneva, Switzerland: World Health Organization (WHO), 2005
World Health Organization (WHO). Philippines World Health Survey 2003. Geneva, Switzerland: World Health Organization (WHO), 2005
World Health Organization (WHO). Senegal World Health Survey 2003. Geneva, Switzerland: World Health Organization (WHO), 2005
World Health Organization (WHO). South Africa World Health Survey 2002-2003. Geneva, Switzerland: World Health Organization (WHO), 2005
World Health Organization (WHO). United Arab Emirates World Health Survey 2003. Geneva, Switzerland: World Health Organization (WHO), 2005
Ministry of Health (China), National Center for Chronic and Noncommunicable Disease Control and Prevention (China), World Health Organization (WHO). China WHO Study on Global AGEing and Adult Health 2007-2010
Carolina Population Center, University of North Carolina at Chapel Hill, Chinese Center for Disease Control and Prevention (CCDC). China Health and Nutrition Survey. Chapel Hill, United States: Carolina Population Center, University of North Carolina at Chapel Hill
Ministry of Public Health (Lebanon), World Health Organization (WHO). Lebanon WHO Multi-country Survey Study on Health and Health System Responsiveness 2000-2001
Institute of Social Medicine and Health Policy, Shandong University, Shandong University School of Medicine, World Health Organization (WHO). China WHO Multi-country Survey Study on Health and Health System Responsiveness 2000-2001
Central Statistical Service (South Africa). South Africa October Household Survey 1994
Department of Health (South Africa), Human Sciences Research Council, World Health Organization (WHO). South Africa WHO Study on Global AGEing and Adult Health 2007-2008. Geneva, Switzerland: World Health Organization (WHO)
China Center for Economic Research, Peking University. China Health and Retirement Longitudinal Study Pilot Resurvey 2012. Beijing, China: China Center for Economic Research, Peking University
Human Sciences Research Council, South African Medical Research Council. South Africa National Health and Nutrition Examination Survey 2012
Central American Population Center, University of Costa Rica. Costa Rica Survey of Family Health Services and Expenses 2008. San José, Costa Rica: Central American Population Center, University of Costa Rica
Gallup, World Health Organization (WHO). United Arab Emirates WHO Multi-country Survey Study on Health and Health System Responsiveness 2000-2001. Geneva, Switzerland: World Health Organization (WHO)
International Research Associates (INRA) Europe, World Health Organization (WHO). Belgium WHO Multi-country Survey Study on Health and Health System Responsiveness 2000-2001. Geneva, Switzerland: World Health Organization (WHO)
Gallup, World Health Organization (WHO). Costa Rica WHO Multi-country Survey Study on Health and Health System Responsiveness 2000-2001. Geneva, Switzerland: World Health Organization (WHO)
Market, Media, and Public Opinion Research (Croatia), World Health Organization (WHO). Croatia WHO Multi-country Survey Study on Health and Health System Responsiveness 2000-2001. Geneva, Switzerland: World Health Organization (WHO)
International Research Associates (INRA) Europe, World Health Organization (WHO). Italy WHO Multi-country Survey Study on Health and Health System Responsiveness 2000-2001. Geneva, Switzerland: World Health Organization (WHO)
Börsch-Supan, A. (2015). Survey of Health, Ageing and Retirement in Europe (SHARE) Wave 5. Release version: 1.0.0. SHARE-ERIC. Data set. DOI: 10.6103/SHARE.w5.100
Börsch-Supan, A. (2015). Survey of Health, Ageing and Retirement in Europe (SHARE) Wave 5. Release version: 1.0.0. SHARE-ERIC. Data set. DOI: 10.6103/SHARE.w5.100
Chinese Center for Disease Control and Prevention (CCDC), Ministry of Health (China). China National Injury Surveillance System 2013
Chinese Center for Disease Control and Prevention (CCDC), Ministry of Health (China). China National Injury Surveillance System 2014
Center for Study of Public Opinion (Kazakhstan), Concluzia-Prim Center for Survey Methodology (Moldova), Institute for Advanced Studies (Austria), London School of Hygiene and Tropical Medicine, University of Aberdeen. Kazakhstan Health in Times of Transition Household Survey 2010
Philippine Health Insurance Corporation. Philippine Health Insurance Corporation Claims 2013-2016
Ministry of Health (Italy). Italy National Hospital Discharge Database 2012
Federal Public Service Health, Food Chain Safety, and Environment (Belgium). Belgium Minimum Clinical Summary 2000
Federal Public Service Health, Food Chain Safety, and Environment (Belgium). Belgium Minimum Clinical Summary 2001
Federal Public Service Health, Food Chain Safety, and Environment (Belgium). Belgium Minimum Clinical Summary 2002
Federal Public Service Health, Food Chain Safety, and Environment (Belgium). Belgium Minimum Clinical Summary 2005
Federal Public Service Health, Food Chain Safety, and Environment (Belgium). Belgium Minimum Clinical Summary 2006
Federal Public Service Health, Food Chain Safety, and Environment (Belgium). Belgium Minimum Clinical Summary 2007
Federal Public Service Health, Food Chain Safety, and Environment (Belgium). Belgium Minimum Hospital Summary 2010
Federal Public Service Health, Food Chain Safety, and Environment (Belgium). Belgium Minimum Hospital Summary 2011
Federal Public Service Health, Food Chain Safety, and Environment (Belgium). Belgium Minimum Hospital Summary 2012
Federal Public Service Health, Food Chain Safety, and Environment (Belgium). Belgium Minimum Hospital Summary 2013
Ministry of Health (Italy). Italy National Hospital Discharge Database 1970
Ministry of Health (Italy). Italy National Hospital Discharge Database 1971
Ministry of Health (Italy). Italy National Hospital Discharge Database 1972
Ministry of Health (Italy). Italy National Hospital Discharge Database 1973
Ministry of Health (Italy). Italy National Hospital Discharge Database 1974
Ministry of Health (Italy). Italy National Hospital Discharge Database 1975
Ministry of Health (Italy). Italy National Hospital Discharge Database 1976
Ministry of Health (Italy). Italy National Hospital Discharge Database 1977
Ministry of Health (Italy). Italy National Hospital Discharge Database 1978
Ministry of Health (Italy). Italy National Hospital Discharge Database 1979
Ministry of Health (Italy). Italy National Hospital Discharge Database 1980
Ministry of Health (Italy). Italy National Hospital Discharge Database 1981
Ministry of Health (Italy). Italy National Hospital Discharge Database 1982
Ministry of Health (Italy). Italy National Hospital Discharge Database 1983
Ministry of Health (Italy). Italy National Hospital Discharge Database 1984
Ministry of Health (Italy). Italy National Hospital Discharge Database 1985
Ministry of Health (Italy). Italy National Hospital Discharge Database 1986
Ministry of Health (Italy). Italy National Hospital Discharge Database 1987
Ministry of Health (Italy). Italy National Hospital Discharge Database 1988
Ministry of Health (Italy). Italy National Hospital Discharge Database 1989
Ministry of Health (Italy). Italy National Hospital Discharge Database 1990
Ministry of Health (Italy). Italy National Hospital Discharge Database 1991
Ministry of Health (Italy). Italy National Hospital Discharge Database 1992
Ministry of Health (Italy). Italy National Hospital Discharge Database 1993
Ministry of Health (Italy). Italy National Hospital Discharge Database 1994
Ministry of Health (Italy). Italy National Hospital Discharge Database 1995
Ministry of Health (Italy). Italy National Hospital Discharge Database 1996
Ministry of Health (Italy). Italy National Hospital Discharge Database 1997
Ministry of Health (Italy). Italy National Hospital Discharge Database 1998
Ministry of Health (Italy). Italy National Hospital Discharge Database 1999
Ministry of Health (Italy). Italy National Hospital Discharge Database 2000
Ministry of Health (Italy). Italy National Hospital Discharge Database 2013
Ministry of Health (Italy). Italy National Hospital Discharge Database 2014
National Bureau of Statistics of China. China Statistical Yearbook 2015. Beijing, China: National Bureau of Statistics of China
Ministry of Health (Italy). Italy Hospital Inpatient Discharges 2013-2016
Ghana Statistical Service. Ghana Living Standards Measurement Survey 1991-1992. Accra, Ghana: Ghana Statistical Service
Ghana Statistical Service. Ghana Living Standards Survey 1998-1999
Ghana Statistical Service. Ghana Living Standards Measurement Survey 2005-2006. Accra, Ghana: Ghana Statistical Service
Institute of Sociology, Russian Academy of Sciences, Paragon Research, University of North Carolina, World Bank. Kyrgyzstan Living Standards Measurement Survey 1993. Washington DC, United States: World Bank
World Health Organization (WHO). Czech Republic World Health Survey 2002-2003. Geneva, Switzerland: World Health Organization (WHO), 2005
World Health Organization (WHO). Ghana World Health Survey 2003. Geneva, Switzerland: World Health Organization (WHO), 2005
World Health Organization (WHO). Luxembourg World Health Survey 2003. Geneva, Switzerland: World Health Organization (WHO), 2005
World Health Organization (WHO). Sri Lanka World Health Survey 2003. Geneva, Switzerland: World Health Organization (WHO), 2005
World Health Organization (WHO). Swaziland World Health Survey 2003. Geneva, Switzerland: World Health Organization (WHO), 2005
Pan American Health Organization (PAHO), Center for Demography and Ecology, University of Wisconsin-Madison, Inter-University Consortium for Political and Social Research (ICPSR), Chronic Disease Research Centre (CDRC), University of the West Indies. Barbados - Bridgetown Survey on Health, Well-Being, and Aging in Latin America and the Caribbean 1999-2000. Ann Arbor,
National Statistical Committee of the Kyrgyz Republic, Research Triangle Institute, Inc. (RTI), World Bank. Kyrgyzstan Living Standards Measurement Survey 1997. Washington DC, United States: World Bank
National Statistical Committee of the Kyrgyz Republic, Research Triangle Institute, Inc. (RTI), World Bank. Kyrgyzstan Living Standards Measurement Survey 1998. Washington DC, United States: World Bank
Ghana Health Service, Ministry of Health (Ghana), University of Ghana, World Health Organization (WHO). Ghana WHO Study on Global AGEing and Adult Health 2007-2008
Gallup, World Health Organization (WHO). Argentina WHO Multi-country Survey Study on Health and Health System Responsiveness 2000-2001. Geneva, Switzerland: World Health Organization (WHO)
Mock CN, Forjuoh SN, Rivara FP. Epidemiology of transport-related injuries in Ghana. Accid Anal Prev. 1999; 31(4): 359-70
Eurostat, Institute of Health Information and Statistics of the Czech Republic. Czech Republic European Health Interview Survey 2008
Statistical Service of Cyprus (CYSTAT). Cyprus European Health Interview Survey 2008
General Administration of Statistics and Censuses (El Salvador), Ministry of Economy (El Salvador). El Salvador Multipurpose Household Survey 2013. San Salvador, El Salvador: General Administration of Statistics and Censuses (El Salvador)
Ghana Statistical Service, World Bank. Ghana Living Standards Measurement Survey 2012-2013. Accra, Ghana: Ghana Statistical Service
MEMRB International, World Health Organization (WHO). Cyprus WHO Multi-country Survey Study on Health and Health System Responsiveness 2000-2001. Geneva, Switzerland: World Health Organization (WHO)
Institute of Health Information and Statistics of the Czech Republic, International Research Associates (INRA) Europe, World Health Organization (WHO). Czech Republic WHO Multi-country Survey Study on Health and Health System Responsiveness 2000-2001. Geneva, Switzerland: World Health Organization (WHO)
National Statistical Committee of the Kyrgyz Republic, SIAR Research and Consulting (Kyrgyzstan), World Health Organization (WHO). Kyrgyzstan WHO Multi-country Survey Study on Health and Health System Responsiveness 2000-2001. Geneva, Switzerland: World Health Organization (WHO)
International Research Associates (INRA) Europe, World Health Organization (WHO). Luxembourg WHO Multi-country Survey Study on Health and Health System Responsiveness 2000-2001. Geneva, Switzerland: World Health Organization (WHO)
Börsch-Supan, A. (2015). Survey of Health, Ageing and Retirement in Europe (SHARE) Wave 5. Release version: 1.0.0. SHARE-ERIC. Data set. DOI: 10.6103/SHARE.w5.100
Börsch-Supan, A. (2015). Survey of Health, Ageing and Retirement in Europe (SHARE) Wave 5. Release version: 1.0.0. SHARE-ERIC. Data set. DOI: 10.6103/SHARE.w5.100
Belarusian State University, Concluzia-Prim Center for Survey Methodology (Moldova), Institute for Advanced Studies (Austria), London School of Hygiene and Tropical Medicine, University of Aberdeen. Belarus Health in Times of Transition Household Survey 2010
Concluzia-Prim Center for Survey Methodology (Moldova), Institute for Advanced Studies (Austria), International Centre for Sociological, Political and Social Psychological Research (Kyrgyzstan), London School of Hygiene and Tropical Medicine, University of Aberdeen. Kyrgyzstan Health in Times of Transition Household Survey 2011
General Administration of Statistics and Censuses (El Salvador), Ministry of Economy (El Salvador). El Salvador Multipurpose Household Survey 2014. San Salvador, El Salvador: General Administration of Statistics and Censuses (El Salvador)
Department of Economics, University of Chile, Ministry of Planning (Chile). Chile National Socioeconomic Characterization Survey 1992. Santiago, Chile: Ministry of Social Development (Chile)
Department of Economics, University of Chile, Ministry of Planning (Chile). Chile National Socioeconomic Characterization Survey 1994. Santiago, Chile: Ministry of Social Development (Chile)
Department of Economics, University of Chile, Ministry of Planning (Chile). Chile National Socioeconomic Characterization Survey 1996. Santiago, Chile: Ministry of Social Development (Chile)
Department of Economics, University of Chile, Ministry of Planning (Chile). Chile National Socioeconomic Characterization Survey 1998. Santiago, Chile: Ministry of Social Development (Chile)
Department of Economics, University of Chile, Ministry of Planning (Chile). Chile National Socioeconomic Characterization Survey 2000. Santiago, Chile: Ministry of Social Development (Chile)
Ministry of Public Health (Thailand). Thailand National Health and Examination Survey 2003-2004
World Health Organization (WHO). Denmark World Health Survey 2003. Geneva, Switzerland: World Health Organization (WHO), 2005
World Health Organization (WHO). Estonia World Health Survey 2003. Geneva, Switzerland: World Health Organization (WHO), 2005
World Health Organization (WHO). Guatemala World Health Survey 2003. Geneva, Switzerland: World Health Organization (WHO), 2005
World Health Organization (WHO). Hungary World Health Survey 2003. Geneva, Switzerland: World Health Organization (WHO), 2005
World Health Organization (WHO). Morocco World Health Survey 2003. Geneva, Switzerland: World Health Organization (WHO), 2005
World Health Organization (WHO). Zimbabwe World Health Survey 2003. Geneva, Switzerland: World Health Organization (WHO), 2005
Department of Economics, University of Chile, Ministry of Planning (Chile). Chile National Socioeconomic Characterization Survey 2003. Santiago, Chile: Ministry of Social Development (Chile)
Pan American Health Organization (PAHO), Center for Demography and Ecology, University of Wisconsin-Madison, Inter-University Consortium for Political and Social Research (ICPSR), Institute of Nutrition and Food Technology (INTA), University of Chile, Center for Geriatrics and Gerontology, Pontifical Catholic University of Chile. Chile - Santiago Survey on Health, Well-Being, a[illegible]
Directorate of Statistics of the High Commission for Planning (Morocco), World Bank. Morocco Living Standards Measurement Survey 1990-1991
Department of Economics, University of Chile, Ministry of Planning (Chile). Chile National Socioeconomic Characterization Survey 2006. Santiago, Chile: Ministry of Social Development (Chile)
Gallup, Health Promotion Research Institute (Hungary). Hungary National Population Health Survey 2000
Ministry of Health (Chile). Chile Hospital Discharges 2001. Santiago, Chile: Ministry of Health (Chile)
Ministry of Health (Chile). Chile Hospital Discharges 2002. Santiago, Chile: Ministry of Health (Chile)
Ministry of Health (Chile). Chile Hospital Discharges 2003. Santiago, Chile: Ministry of Health (Chile)

Ministry of Health (Chile). Chile Hospital Discharges 2004. Santiago, Chile: Ministry of Health (Chile)
Ministry of Health (Chile). Chile Hospital Discharges 2005. Santiago, Chile: Ministry of Health (Chile)
Ministry of Health (Chile). Chile Hospital Discharges 2006. Santiago, Chile: Ministry of Health (Chile)
Ministry of Health (Chile). Chile Hospital Discharges 2007. Santiago, Chile: Ministry of Health (Chile)
Ministry of Health (Chile). Chile Hospital Discharges 2008. Santiago, Chile: Ministry of Health (Chile)
Ministry of Health (Chile). Chile Hospital Discharges 2009. Santiago, Chile: Ministry of Health (Chile)
Ministry of Health (Chile). Chile Hospital Discharges 2010. Santiago, Chile: Ministry of Health (Chile)
Ministry of Health (Chile). Chile Hospital Discharges 2011. Santiago, Chile: Ministry of Health (Chile)
Institute of Health Research, Chulalongkorn University (Thailand), Ministry of Public Health (Thailand), The Alliance for Safe Children (TASC), United Nations Children's Fund (UNICEF). Thailand National Injury Survey 2003-2004
Ministry of Planning (Chile), Social Observatory, Alberto Hurtado University. Chile National Socioeconomic Characterization Survey 2009. Santiago, Chile: Ministry of Social Development (Chile)
Eurostat, Hungarian Central Statistical Office (HCSO). Hungary European Health Interview Survey 2009 - Eurostat
Ministry of Health (Chile). Chile Hospital Discharges 2012. Santiago, Chile: Ministry of Health (Chile)
University of Concepcion (Chile), World Health Organization (WHO). Chile WHO Multi-country Survey Study on Health and Health System Responsiveness 2000-2001. Geneva, Switzerland: World Health Organization (WHO)
Statistics Denmark, World Health Organization (WHO). Denmark WHO Multi-country Survey Study on Health and Health System Responsiveness 2000-2001. Geneva, Switzerland: World Health Organization (WHO)
International Research Associates (INRA) Europe, World Health Organization (WHO). Estonia WHO Multi-country Survey Study on Health and Health System Responsiveness 2000-2001. Geneva, Switzerland: World Health Organization (WHO)
Szonda Ipsos, World Health Organization (WHO). Hungary WHO Multi-country Survey Study on Health and Health System Responsiveness 2000-2001. Geneva, Switzerland: World Health Organization (WHO)
Graduate School of Public Health, Seoul National University, World Health Organization (WHO). South Korea WHO Multi-country Survey Study on Health and Health System Responsiveness 2000-2001. Geneva, Switzerland: World Health Organization (WHO)
Gallup, World Health Organization (WHO). Morocco WHO Multi-country Survey Study on Health and Health System Responsiveness 2000-2001. Geneva, Switzerland: World Health Organization (WHO)
International Research Associates (INRA) Europe, World Health Organization (WHO). Malta WHO Multi-country Survey Study on Health and Health System Responsiveness 2000-2001. Geneva, Switzerland: World Health Organization (WHO)
Mahidol University, World Health Organization (WHO). Thailand WHO Multi-country Survey Study on Health and Health System Responsiveness 2000-2001. Geneva, Switzerland: World Health Organization (WHO)
Börsch-Supan, A. (2015). Survey of Health, Ageing and Retirement in Europe (SHARE) Wave 5. Release version: 1.0.0. SHARE-ERIC. Data set. DOI: 10.6103/SHARE.w5.100
Börsch-Supan, A. (2015). Survey of Health, Ageing and Retirement in Europe (SHARE) Wave 5. Release version: 1.0.0. SHARE-ERIC. Data set. DOI: 10.6103/SHARE.w5.100
National Office of Statistics (Cuba). Cuba Statistical Yearbook 2012. Havana, Cuba: National Office of Statistics (Cuba)
Ministry of Health (Chile). Chile Hospital Discharges 2001-2002
Ministry of Health (Chile). Chile Hospital Discharges 2003-2007
Ministry of Health (Chile). Chile Hospital Discharges 2008-2012
Danish Health and Medicines Authority. Denmark National Patient Registry 2003
Danish Health and Medicines Authority. Denmark National Patient Registry 2004
Danish Health and Medicines Authority. Denmark National Patient Registry 2005
Danish Health and Medicines Authority. Denmark National Patient Registry 2006
Danish Health and Medicines Authority. Denmark National Patient Registry 2007
Danish Health and Medicines Authority. Denmark National Patient Registry 2008
Danish Health and Medicines Authority. Denmark National Patient Registry 2009
Ministry of Social Affairs (Estonia), National Institute for Health Development (Estonia). Estonia Hospital Inpatient Discharges 2003
Ministry of Social Affairs (Estonia), National Institute for Health Development (Estonia). Estonia Hospital Inpatient Discharges 2004
Ministry of Social Affairs (Estonia), National Institute for Health Development (Estonia). Estonia Hospital Inpatient Discharges 2005
Ministry of Social Affairs (Estonia), National Institute for Health Development (Estonia). Estonia Hospital Inpatient Discharges 2006
Ministry of Social Affairs (Estonia), National Institute for Health Development (Estonia). Estonia Hospital Inpatient Discharges 2007
Ministry of Social Affairs (Estonia), National Institute for Health Development (Estonia). Estonia Hospital Inpatient Discharges 2008
Ministry of Social Affairs (Estonia), National Institute for Health Development (Estonia). Estonia Hospital Inpatient Discharges 2009
Ministry of Social Affairs (Estonia), National Institute for Health Development (Estonia). Estonia Hospital Inpatient Discharges 2010
Ministry of Social Affairs (Estonia), National Institute for Health Development (Estonia). Estonia Hospital Inpatient Discharges 2011
Center for Health Care Information (GYOGYINFOK) (Hungary). Hungary Hospital Inpatient Discharges 1990
National Institute for Health Development (Estonia). Estonia Hospital Inpatient Discharges 2012
National Institute for Health Development (Estonia). Estonia Hospital Inpatient Discharges 2013
National Institute for Health Development (Estonia). Estonia Hospital Inpatient Discharges 2014
Institute of Experimental and Clinical Medicine (Estonia). Estonia Hospital Inpatient Discharges 1999
Institute of Experimental and Clinical Medicine (Estonia). Estonia Hospital Inpatient Discharges 1998
Institute of Experimental and Clinical Medicine (Estonia). Estonia Hospital Inpatient Discharges 1997
Institute of Experimental and Clinical Medicine (Estonia). Estonia Hospital Inpatient Discharges 1996
Institute of Experimental and Clinical Medicine (Estonia). Estonia Hospital Inpatient Discharges 1995
Institute of Experimental and Clinical Medicine (Estonia). Estonia Hospital Inpatient Discharges 1994
Institute of Experimental and Clinical Medicine (Estonia). Estonia Hospital Inpatient Discharges 1993
Institute of Experimental and Clinical Medicine (Estonia). Estonia Hospital Inpatient Discharges 1992
Institute of Experimental and Clinical Medicine (Estonia). Estonia Hospital Inpatient Discharges 1991
Institute of Experimental and Clinical Medicine (Estonia). Estonia Hospital Inpatient Discharges 1990
Institute of Experimental and Clinical Medicine (Estonia). Estonia Hospital Inpatient Discharges 1989
Institute of Experimental and Clinical Medicine (Estonia). Estonia Hospital Inpatient Discharges 1988
Institute of Experimental and Clinical Medicine (Estonia). Estonia Hospital Inpatient Discharges 1987
Institute of Experimental and Clinical Medicine (Estonia). Estonia Hospital Inpatient Discharges 1986
Institute of Experimental and Clinical Medicine (Estonia). Estonia Hospital Inpatient Discharges 1985
Institute of Experimental and Clinical Medicine (Estonia). Estonia Hospital Inpatient Discharges 1980
Center for Health Care Information (GYOGYINFOK) (Hungary). Hungary Hospital Inpatient Discharges 1991
Center for Health Care Information (GYOGYINFOK) (Hungary). Hungary Hospital Inpatient Discharges 1992
Center for Health Care Information (GYOGYINFOK) (Hungary). Hungary Hospital Inpatient Discharges 1993
Center for Health Care Information (GYOGYINFOK) (Hungary). Hungary Hospital Inpatient Discharges 1994
Center for Health Care Information (GYOGYINFOK) (Hungary). Hungary Hospital Inpatient Discharges 1995
Center for Health Care Information (GYOGYINFOK) (Hungary). Hungary Hospital Inpatient Discharges 1996
Center for Health Care Information (GYOGYINFOK) (Hungary). Hungary Hospital Inpatient Discharges 1997
Center for Health Care Information (GYOGYINFOK) (Hungary). Hungary Hospital Inpatient Discharges 1998
Center for Health Care Information (GYOGYINFOK) (Hungary). Hungary Hospital Inpatient Discharges 1999
Center for Health Care Information (GYOGYINFOK) (Hungary). Hungary Hospital Inpatient Discharges 2000
Center for Health Care Information (GYOGYINFOK) (Hungary). Hungary Hospital Inpatient Discharges 2001
Center for Health Care Information (GYOGYINFOK) (Hungary). Hungary Hospital Inpatient Discharges 2002
Center for Health Care Information (GYOGYINFOK) (Hungary). Hungary Hospital Inpatient Discharges 2003
National Institute for Strategic Health Research (ESKI) (Hungary). Hungary Hospital Inpatient Discharges 2013
National Institute for Strategic Health Research (ESKI) (Hungary). Hungary Hospital Inpatient Discharges 2014
Danish Health and Medicines Authority. Denmark National Patient Registry 1979
Danish Health and Medicines Authority. Denmark National Patient Registry 1980
Danish Health and Medicines Authority. Denmark National Patient Registry 1981
Danish Health and Medicines Authority. Denmark National Patient Registry 1982
Danish Health and Medicines Authority. Denmark National Patient Registry 1983
Danish Health and Medicines Authority. Denmark National Patient Registry 1984
Danish Health and Medicines Authority. Denmark National Patient Registry 1985
Danish Health and Medicines Authority. Denmark National Patient Registry 1986
Danish Health and Medicines Authority. Denmark National Patient Registry 1987
Danish Health and Medicines Authority. Denmark National Patient Registry 1988
Danish Health and Medicines Authority. Denmark National Patient Registry 1989
Danish Health and Medicines Authority. Denmark National Patient Registry 1990
Danish Health and Medicines Authority. Denmark National Patient Registry 1991
Danish Health and Medicines Authority. Denmark National Patient Registry 1992
Danish Health and Medicines Authority. Denmark National Patient Registry 1993
Danish Health and Medicines Authority. Denmark National Patient Registry 1994
Danish Health and Medicines Authority. Denmark National Patient Registry 1995
Danish Health and Medicines Authority. Denmark National Patient Registry 1996
Danish Health and Medicines Authority. Denmark National Patient Registry 1997
Danish Health and Medicines Authority. Denmark National Patient Registry 1998
Danish Health and Medicines Authority. Denmark National Patient Registry 1999
Danish Health and Medicines Authority. Denmark National Patient Registry 2000
Danish Health and Medicines Authority. Denmark National Patient Registry 2001
Danish Health and Medicines Authority. Denmark National Patient Registry 2002
Danish Health and Medicines Authority. Denmark National Patient Registry 2010
Danish Health and Medicines Authority. Denmark National Patient Registry 2011
Danish Health and Medicines Authority. Denmark National Patient Registry 2012
Danish Health and Medicines Authority. Denmark National Patient Registry 2013
National State Statistical Agency (Tajikistan), World Bank. Tajikistan Living Standards Measurement Survey 2003
National State Statistical Agency (Tajikistan), World Bank. Tajikistan Living Standards Measurement Survey 2007
Planning Commission (Tanzania), University of Dar es Salaam, World Bank. Tanzania Living Standards Measurement Study 1993-1994. Washington DC, United States: World Bank
National Statistics Directorate (Timor-Leste), World Bank. Timor-Leste Living Standards and Measurement Survey 2001. Washington DC, United States: World Bank
Palestinian Central Bureau of Statistics. Palestine Demographic and Health Survey 2004
World Health Organization (WHO). Ethiopia World Health Survey 2003. Geneva, Switzerland: World Health Organization (WHO), 2005
World Health Organization (WHO). Finland World Health Survey 2004. Geneva, Switzerland: World Health Organization (WHO), 2005
World Health Organization (WHO). Latvia World Health Survey 2003. Geneva, Switzerland: World Health Organization (WHO), 2005
World Health Organization (WHO). Netherlands World Health Survey 2004. Geneva, Switzerland: World Health Organization (WHO), 2005
World Health Organization (WHO). United Kingdom World Health Survey 2004. Geneva, Switzerland: World Health Organization (WHO), 2005
World Health Organization (WHO). Uruguay World Health Survey 2002-2003. Geneva, Switzerland: World Health Organization (WHO), 2005
National State Statistical Agency (Tajikistan), World Bank. Tajikistan Living Standards Measurement Survey 2009
National Statistics Directorate (Timor-Leste), World Bank. Timor-Leste Living Standards and Measurement Survey 2007-2008. Washington DC, United States: World Bank
National Bureau of Statistics (Tanzania). Tanzania Living Standards Measurement Study - Integrated Surveys on Agriculture 2010-2011. Dar es Salaam, Tanzania: National Bureau of Statistics (Tanzania)
Jimma University, World Health Organization (WHO). Ethiopia Jimma Community Injury Survey 2006
Central Statistical Bureau (Latvia), Eurostat. Latvia European Health Interview Survey 2008 - Eurostat
International Research Associates (INRA) Europe, National Research and Development Center for Welfare and Health (STAKES) (Finland), World Health Organization (WHO). Finland WHO Multi-country Survey Study on Health and Health System Responsiveness 2000-2001. Geneva, Switzerland: World Health Organization (WHO)
National Centre for Social Research (NatCen), World Health Organization (WHO). United Kingdom WHO Multi-country Survey Study on Health and Health System Responsiveness 2000-2001. Geneva, Switzerland: World Health Organization (WHO)
Gallup, World Health Organization (WHO). Latvia WHO Multi-country Survey Study on Health and Health System Responsiveness 2000-2001. Geneva, Switzerland: World Health Organization (WHO)
International Research Associates (INRA) Europe, Netherlands Organisation for Applied Scientific Research (TNO), World Health Organization (WHO). Netherlands WHO Multi-country Survey Study on Health and Health System Responsiveness 2000-2001. Geneva, Switzerland: World Health Organization (WHO)
University of the West Indies, World Health Organization (WHO). Trinidad and Tobago WHO Multi-country Survey Study on Health and Health System Responsiveness 2000-2001. Geneva, Switzerland: World Health Organization (WHO)
Börsch-Supan, A. (2015). Survey of Health, Ageing and Retirement in Europe (SHARE) Wave 5. Release version: 1.0.0. SHARE-ERIC. Data set. DOI: 10.6103/SHARE.w5.100
Centre for Disease Prevention and Control (Latvia), Riga Stradiņš University. Latvia Health Behavior Among the Adult Population 2014
Ethiopian Public Health Institute (EPHI), World Health Organization (WHO). Ethiopia STEPS Noncommunicable Disease Risk Factors Survey 2015
Ministry of Health of the Republic of Latvia. Latvia Hospital Inpatient Discharges 2004
Ministry of Health of the Republic of Latvia. Latvia Hospital Inpatient Discharges 2006
Ministry of Health of the Republic of Latvia. Latvia Hospital Inpatient Discharges 2007
Ministry of Health of the Republic of Latvia. Latvia Hospital Inpatient Discharges 2008
Ministry of Health of the Republic of Latvia. Latvia Hospital Inpatient Discharges 2010
Ministry of Health of the Republic of Latvia, National Health Service (Latvia). Latvia Hospital Inpatient Discharges 2011
Ministry of Health of the Republic of Latvia, National Health Service (Latvia). Latvia Hospital Inpatient Discharges 2012
Institute of Public Health (Macedonia). Macedonia Hospital Inpatient Discharges 2000
Institute of Public Health (Macedonia). Macedonia Hospital Inpatient Discharges 2001
Institute of Public Health (Macedonia). Macedonia Hospital Inpatient Discharges 2002
Institute of Public Health (Macedonia). Macedonia Hospital Inpatient Discharges 2004
National Public Health Institute (Finland). Finland Hospital Discharge Register 1988
National Public Health Institute (Finland). Finland Hospital Discharge Register 1989
National Public Health Institute (Finland). Finland Hospital Discharge Register 1990
National Public Health Institute (Finland). Finland Hospital Discharge Register 1991
National Public Health Institute (Finland). Finland Hospital Discharge Register 1992
National Public Health Institute (Finland). Finland Hospital Discharge Register 1993
Ministry of Health of the Republic of Latvia. Latvia Hospital Inpatient Discharges 1980
Institute of Public Health (Macedonia). Macedonia Hospital Inpatient Discharges 1980
Dutch Hospital Data (DHD). Netherlands National Medical Registry 1990
Dutch Hospital Data (DHD). Netherlands National Medical Registry 1991
Dutch Hospital Data (DHD). Netherlands National Medical Registry 1992
Dutch Hospital Data (DHD). Netherlands National Medical Registry 1993
Dutch Hospital Data (DHD). Netherlands National Medical Registry 1994
Dutch Hospital Data (DHD). Netherlands National Medical Registry 1995
Dutch Hospital Data (DHD). Netherlands National Medical Registry 1996
Dutch Hospital Data (DHD). Netherlands National Medical Registry 1997
National Public Health Institute (Finland). Finland Hospital Discharge Register 1994
National Public Health Institute (Finland). Finland Hospital Discharge Register 1995
National Public Health Institute (Finland). Finland Hospital Discharge Register 1996
National Public Health Institute (Finland). Finland Hospital Discharge Register 1997

National Public Health Institute (Finland). Finland Hospital Discharge Register 1998
National Public Health Institute (Finland). Finland Hospital Discharge Register 1999
National Institute for Health and Welfare (THL) (Finland). Finland Hospital Discharge Register 2013
National Institute for Health and Welfare (THL) (Finland). Finland Hospital Discharge Register 2014
Ministry of Health of the Republic of Latvia. Latvia Hospital Inpatient Discharges 1981
Ministry of Health of the Republic of Latvia. Latvia Hospital Inpatient Discharges 1982
Ministry of Health of the Republic of Latvia. Latvia Hospital Inpatient Discharges 1983
Ministry of Health of the Republic of Latvia. Latvia Hospital Inpatient Discharges 1984
Ministry of Health of the Republic of Latvia. Latvia Hospital Inpatient Discharges 1985
Ministry of Health of the Republic of Latvia. Latvia Hospital Inpatient Discharges 1986
Ministry of Health of the Republic of Latvia. Latvia Hospital Inpatient Discharges 1987
Ministry of Health of the Republic of Latvia. Latvia Hospital Inpatient Discharges 1988
Ministry of Health of the Republic of Latvia. Latvia Hospital Inpatient Discharges 1989
Ministry of Health of the Republic of Latvia. Latvia Hospital Inpatient Discharges 1990
Ministry of Health of the Republic of Latvia. Latvia Hospital Inpatient Discharges 1991
Ministry of Health of the Republic of Latvia. Latvia Hospital Inpatient Discharges 1992
Ministry of Health of the Republic of Latvia. Latvia Hospital Inpatient Discharges 1993
Ministry of Health of the Republic of Latvia. Latvia Hospital Inpatient Discharges 1994
Ministry of Health of the Republic of Latvia. Latvia Hospital Inpatient Discharges 1995
Ministry of Health of the Republic of Latvia. Latvia Hospital Inpatient Discharges 1996
Ministry of Health of the Republic of Latvia. Latvia Hospital Inpatient Discharges 1997
Ministry of Health of the Republic of Latvia. Latvia Hospital Inpatient Discharges 1998
Ministry of Health of the Republic of Latvia. Latvia Hospital Inpatient Discharges 1999
Ministry of Health of the Republic of Latvia. Latvia Hospital Inpatient Discharges 2000
Ministry of Health of the Republic of Latvia. Latvia Hospital Inpatient Discharges 2001
Ministry of Health of the Republic of Latvia. Latvia Hospital Inpatient Discharges 2002
Ministry of Health of the Republic of Latvia. Latvia Hospital Inpatient Discharges 2003
Ministry of Health of the Republic of Latvia, National Health Service (Latvia). Latvia Hospital Inpatient Discharges 2013
Ministry of Health of the Republic of Latvia, National Health Service (Latvia). Latvia Hospital Inpatient Discharges 2014
Institute of Public Health (Macedonia). Macedonia Hospital Inpatient Discharges 1982
Institute of Public Health (Macedonia). Macedonia Hospital Inpatient Discharges 1984
Institute of Public Health (Macedonia). Macedonia Hospital Inpatient Discharges 1985
Institute of Public Health (Macedonia). Macedonia Hospital Inpatient Discharges 1986
Institute of Public Health (Macedonia). Macedonia Hospital Inpatient Discharges 1987
Institute of Public Health (Macedonia). Macedonia Hospital Inpatient Discharges 1989
Institute of Public Health (Macedonia). Macedonia Hospital Inpatient Discharges 1990
Institute of Public Health (Macedonia). Macedonia Hospital Inpatient Discharges 1991
Institute of Public Health (Macedonia). Macedonia Hospital Inpatient Discharges 1992
Institute of Public Health (Macedonia). Macedonia Hospital Inpatient Discharges 1993
Institute of Public Health (Macedonia). Macedonia Hospital Inpatient Discharges 1995
Institute of Public Health (Macedonia). Macedonia Hospital Inpatient Discharges 1996
Institute of Public Health (Macedonia). Macedonia Hospital Inpatient Discharges 1997
Institute of Public Health (Macedonia). Macedonia Hospital Inpatient Discharges 2012
Department of Statistics (Mexico). Mexico General Population and Housing Census 1980
Centers for Disease Control and Prevention (CDC). United States Behavioral Risk Factor Surveillance System 2003. Atlanta, Georgia: CDC, US Department of Health and Human Services
Centers for Disease Control and Prevention (CDC). United States Behavioral Risk Factor Surveillance System 2006. Atlanta, Georgia: CDC, US Department of Health and Human Services
Centers for Disease Control and Prevention (CDC). United States Behavioral Risk Factor Surveillance System 2008. Atlanta, Georgia: CDC, US Department of Health and Human Services
Centers for Disease Control and Prevention (CDC). United States Behavioral Risk Factor Surveillance System 2012. Atlanta, Georgia: CDC, US Department of Health and Human Services, 2013
Hanoi School of Public Health, The Alliance for Safe Children (TASC), United Nations Children's Fund (UNICEF). Vietnam Multi-Center Injury Survey 2001
Ministry of Health (Indonesia). Indonesia Integrated Hospital Data 2013. Jakarta, Indonesia: Ministry of Health (Indonesia), 2014
Abolhassani F, Moayyeri A, Naghavi M, Soltani A, Larijani B, Shalmani HT. Incidence and characteristics of falls leading to hip fracture in Iranian population. Bone. 2006; 39(2): 408-13
Saadat S, Mafi M, Sharif-Alhoseini M. Population based estimates of non-fatal injuries in the capital of Iran. BMC Public Health. 2011; 608
Rezapur-Shahkolai F, Naghavi M, Vaez M, Shokouhi M, Laflamme L. Injury incidence, healthcare consumption and avenues for prevention: a household survey on injury in rural Twiserkan, Iran. Public Health. 2009; 123(5): 384-9
Stalin P, Senthilvel V, Kanimozhy K, Singh Z, Rajkamal R, Purty AJ. Burden, distribution and impact of domestic accidents in a semi-urban area of coastal Tamil Nadu, India. Int J Inj Contr Saf Promot. 2015; 22(1): 11–5
Dandona R, Kumar GA, Ivers R, Joshi R, Neal B, Dandona L. Characteristics of non-fatal fall injuries in rural India. Inj Prev. 2010; 16(3): 166–71
Bachani AM, Ghaffar A, Hyder AA. Burden of fall injuries in Pakistan--analysis of the National Injury Survey of Pakistan. East Mediterr Health J. 2011; 17(5): 375-81
Department of Health (Hong Kong). Hong Kong Injury Survey 2008
Chien W-C, Lai C-H, Chung C-H, Lin C-H. A retrospective population-based data analyses of unintentional fall mortality and hospitalisation in Taiwan during 2005-2007. Int J Inj Contr Saf Promot. 2013; 20(1): 50-8
Verma PK, Tewari KN. Epidemiology of Road Traffic Injuries in Delhi Result of a Survey. Regional Health Forum. 2004; 8(1): 1-10
Panjeshahin MR, Lari AR, Talei A, Shamsnia J, Alaghehbandan R. Epidemiology and mortality of burns in the South West of Iran. Burns. 2001; 27(3): 219-26
ICF Macro, Institute of Public Health (Albania), Institute of Statistics (Albania). Albania Demographic and Health Survey 2008-2009. Fairfax, United States: ICF International, 2009
Norwegian Directorate of Health. Norway Patient Register 2008-2012
Norwegian Directorate of Health. Norway Control and Payment of Health Reimbursement (KUHR) Database 2006
Norwegian Directorate of Health. Norway Control and Payment of Health Reimbursement (KUHR) Database 2007
Norwegian Directorate of Health. Norway Control and Payment of Health Reimbursement (KUHR) Database 2008
Norwegian Directorate of Health. Norway Control and Payment of Health Reimbursement (KUHR) Database 2009
Norwegian Directorate of Health. Norway Control and Payment of Health Reimbursement (KUHR) Database 2010
Norwegian Directorate of Health. Norway Control and Payment of Health Reimbursement (KUHR) Database 2011
Norwegian Directorate of Health. Norway Control and Payment of Health Reimbursement (KUHR) Database 2012
Norwegian Directorate of Health. Norway Control and Payment of Health Reimbursement (KUHR) Database 2013
Norwegian Directorate of Health. Norway Control and Payment of Health Reimbursement (KUHR) Database 2014
Norwegian Directorate of Health. Norway Control and Payment of Health Reimbursement (KUHR) Database 2015
Ministry of Health, Labour and Welfare (Japan). Japan Diagnosis Procedure Combination Database 2010-2012
Ministry of Health, Labour and Welfare (Japan). Japan Diagnosis Procedure Combination Database 2013-2015
Federal Statistical Office (Germany). Germany Hospital Discharges by Diagnosis 2009. Wiesbaden, Germany: Federal Statistical Office (Germany), 2011
Hamad Medical Corporation (Qatar). Qatar - Annual Inpatients Discharge Abstract: Hamad General Hospital 2002. Doha, Qatar: Hamad Medical Corporation (Qatar)
Hamad Medical Corporation (Qatar). Qatar - Annual Inpatients Discharge Abstract: Hamad General Hospital and Women's Hospital 2003. Doha, Qatar: Hamad Medical Corporation (Qatar)
National Institute of Statistics and Censuses (Ecuador). Ecuador Hospital Inpatient Discharges 1993-1997
National Institute of Statistics and Censuses (Ecuador). Ecuador Hospital Inpatient Discharges 1998-2002
National Institute of Statistics and Censuses (Ecuador). Ecuador Hospital Inpatient Discharges 2003-2007
National Institute of Statistics and Censuses (Ecuador). Ecuador Hospital Inpatient Discharges 2008-2012
National Institute of Statistics and Censuses (Ecuador). Ecuador Hospital Inpatient Discharges 2013-2014
Ministry of Health and Medical Education (Iran). Iran Hospital Data 2001-2010
National Board of Health and Welfare (Sweden). Sweden National Patient Register 2001. Stockholm, Sweden: National Board of Health and Welfare (Sweden)
National Board of Health and Welfare (Sweden). Sweden National Patient Register 2002. Stockholm, Sweden: National Board of Health and Welfare (Sweden)
National Board of Health and Welfare (Sweden). Sweden National Patient Register 2003. Stockholm, Sweden: National Board of Health and Welfare (Sweden)
National Board of Health and Welfare (Sweden). Sweden National Patient Register 2004. Stockholm, Sweden: National Board of Health and Welfare (Sweden)
National Board of Health and Welfare (Sweden). Sweden National Patient Register 2005. Stockholm, Sweden: National Board of Health and Welfare (Sweden)
National Board of Health and Welfare (Sweden). Sweden National Patient Register 2006. Stockholm, Sweden: National Board of Health and Welfare (Sweden)
National Board of Health and Welfare (Sweden). Sweden National Patient Register 2007. Stockholm, Sweden: National Board of Health and Welfare (Sweden)
National Board of Health and Welfare (Sweden). Sweden National Patient Register 2008. Stockholm, Sweden: National Board of Health and Welfare (Sweden)
National Board of Health and Welfare (Sweden). Sweden National Patient Register 2009. Stockholm, Sweden: National Board of Health and Welfare (Sweden)
National Board of Health and Welfare (Sweden). Sweden National Patient Register 2010. Stockholm, Sweden: National Board of Health and Welfare (Sweden)
National Board of Health and Welfare (Sweden). Sweden National Patient Register 2011. Stockholm, Sweden: National Board of Health and Welfare (Sweden)
National Board of Health and Welfare (Sweden). Sweden National Patient Register 2012. Stockholm, Sweden: National Board of Health and Welfare (Sweden)
National Board of Health and Welfare (Sweden). Sweden National Patient Register 1998-2002
National Board of Health and Welfare (Sweden). Sweden National Patient Register 2003-2007
National Board of Health and Welfare (Sweden). Sweden National Patient Register 2008-2012
Nazareth Hospital, Shillong. India - Shillong Nazareth Hospital Inpatient Discharges 2014
Nazareth Hospital, Shillong, JSS Hospital, Mysore, King George's Medical University (India). India Hospital Inpatient Data 2014-2017.
Department of Health Services, Ministry of Health and Population (Nepal). Nepal Hospital Inpatient Discharges 2013-2014
Ministry of Health (Jordan). Jordan Al-Bashir Hospital Discharges 2016
Federal Ministry of Health (Austria), Statistics Austria. Austria Hospital Inpatient Discharges 1989-1992
Federal Ministry of Health (Austria), Statistics Austria. Austria Hospital Inpatient Discharges 1993-1997
Federal Ministry of Health (Austria), Statistics Austria. Austria Hospital Inpatient Discharges 1998-2002
Federal Ministry of Health (Austria), Statistics Austria. Austria Hospital Inpatient Discharges 2003-2007
Federal Ministry of Health (Austria), Statistics Austria. Austria Hospital Inpatient Discharges 2008-2012
NHS England. United Kingdom - England Hospital Episode Statistics 2003-2007
NHS England. United Kingdom - England Hospital Episode Statistics 2001-2002
NHS England. United Kingdom - England Hospital Episode Statistics 2008-2012
Federal Ministry of Health (Austria), Statistics Austria. Austria Hospital Inpatient Discharges 2013-2014
Ministry of Health (Turkey). Turkey Diagnosis-Related Group Hospital Inpatient Database 2011-2012
NHS England. United Kingdom - England Hospital Episode Statistics 2013-2014
Ministry of Health (Italy). Italy Hospital Inpatient Discharges 2005-2007
Ministry of Health (Italy). Italy Hospital Inpatient Discharges 2008-2012
Center for Health Statistics and Information, National Health and Family Planning Commission (China), Shanghai Municipal Center for Disease Control and Prevention (Shanghai CDC), Shanghai Health Information Center. China Hospital Inpatient Data 2013-2016
Tercero F, Andersson R, Peña R, Rocha J, Castro N. The epidemiology of moderate and severe injuries in a Nicaraguan community: a household-based survey. Public Health. 2006; 120(2): 106-14
National Council Against Addictions (Mexico), National Institute of Psychiatry Ramón de la Fuente Muñiz (Mexico), National Institute of Public Health (Mexico). Mexico National Addiction Survey 2008. Cuernavaca, Mexico: National Institute of Public Health (Mexico)
National Center for the Prevention and Control of Addictions (Mexico), National Council Against Addictions (Mexico), National Institute of Psychiatry Ramón de la Fuente Muñiz (Mexico), National Institute of Public Health (Mexico). Mexico National Addiction Survey 2011
Nguyen TV, Dalman C, Le TC, Nguyen TV, Tran NV, Allebeck P. Suicide attempt in a rural area of Vietnam: Incidence, methods used and access to mental health care. Int J Ment Health Syst. 2010; 4(1): 3
Department of Health (South Africa), Macro International, Inc, South African Medical Research Council. South Africa Demographic and Health Survey 1998. Fairfax, United States: ICF International
Macro International, Inc, National Institute of Public Health (Cambodia), National Institute of Statistics (Cambodia). Cambodia Demographic and Health Survey 2005-2006. Fairfax, United States: ICF International
Centers for Disease Control and Prevention (CDC), Central Bureau of Statistics (Kenya), Macro International, Inc, Ministry of Health (Kenya), National Council for Population and Development (Kenya). Kenya Demographic and Health Survey 2003. Fairfax, United States: ICF International
Liberia Institute for Statistics and Geo-information Services (LISGIS), Macro International, Inc. Liberia Demographic and Health Survey 2006-2007. Fairfax, United States: ICF International
Macro International, Inc, Uganda Bureau of Statistics. Uganda Demographic and Health Survey 2006. Fairfax, United States: ICF International
ICF Macro, Kenya Medical Research Institute (KEMRI), Kenya National Bureau of Statistics, Ministry of Public Health and Sanitation (Kenya), National AIDS and STI Control Programme (NASCOP) (Kenya), National Aids Control Council (NACC), National Coordinating Agency for Population and Development (Kenya). Kenya Demographic and Health Survey 2008-2009. Fairfax, United S
Centers for Disease Control and Prevention (CDC). United States Behavioral Risk Factor Surveillance System 2005. Atlanta, Georgia: CDC, US Department of Health and Human Services
Centers for Disease Control and Prevention (CDC), ICF Macro, Ministry of Health (Uganda), Uganda Bureau of Statistics, Uganda Virus Research Institute. Uganda AIDS Indicator Survey 2011. Fairfax, United States: ICF International
ICF Macro, Uganda Bureau of Statistics. Uganda Demographic and Health Survey 2011. Fairfax, United States: ICF International
Centers for Disease Control and Prevention (CDC), Kenya National Bureau of Statistics, United Nations Children's Fund (UNICEF). Kenya Violence Against Children Study 2010. Nairobi, Kenya: Kenya National Bureau of Statistics
European Union Agency for Fundamental Rights. European Union Violence Against Women Study 2012
Central Statistics Organization (Afghanistan), ICF International, Ministry of Public Health (Afghanistan). Afghanistan Demographic and Health Survey 2015-2016. Fairfax, United States: ICF International, 2017
ICF International, Ministry of Health (Cambodia), National Institute of Statistics (Cambodia). Cambodia Demographic and Health Survey 2014. Fairfax, United States: ICF International, 2017
ICF International, Kenya Medical Research Institute (KEMRI), Kenya National Bureau of Statistics, Ministry of Health (Kenya), National AIDS Control Council (Kenya), National Council for Population and Development (Kenya). Kenya Demographic and Health Survey 2014. Fairfax, United States: ICF International
Division of Reproductive Health, Centers for Disease Control and Prevention (CDC), National Institute for Development Information (Nicaragua). Nicaragua Reproductive Health Survey 2006-2007. Managua, Nicaragua: National Institute for Development Information (Nicaragua)
Macro International, Inc, Mitra and Associates, National Institute of Population Research and Training (NIPORT). Bangladesh Demographic and Health Survey 2007. Fairfax, United States: ICF International, 2009
Macro International, Inc, Ministry of Health and Sports (Bolivia), National Institute of Statistics (Bolivia). Bolivia Demographic and Health Survey 2008. Fairfax, United States: ICF International
ICF Macro, Ministry of Health (Burkina Faso), National Institute of Statistics and Demography (Burkina Faso). Burkina Faso Demographic and Health Survey 2010-2011. Fairfax, United States: ICF International
Center for Social and Demographic Studies (Dominican Republic) (CESDEM), Macro International, Inc. Dominican Republic Demographic and Health Survey 2002. Fairfax, United States: ICF International
Center for Social and Demographic Studies (Dominican Republic) (CESDEM), Macro International, Inc. Dominican Republic Demographic and Health Survey 2007. Fairfax, United States: ICF International
El-Zanaty and Associates, Macro International, Inc, Ministry of Health and Population (Egypt), Population Council (Egypt). Egypt Demographic and Health Survey 2005. Fairfax, United States: ICF International
Macro International, Inc, National Scientific and Applied Center for Preventive Medicine (Moldova). Moldova Demographic and Health Survey 2005. Fairfax, United States: ICF International
Macro International, Inc, Ministry of Health (Nicaragua), National Institute of Statistics and Censuses (Nicaragua). Nicaragua Demographic and Health Survey 1997-1998. Fairfax, United States: ICF International
Central Board of Health (Zambia), Central Statistical Office (Zambia), Macro International, Inc. Zambia Demographic and Health Survey 2001-2002. Fairfax, United States: ICF International
Central Statistical Office (Zambia), Macro International, Inc. Zambia Demographic and Health Survey 2007. Fairfax, United States: ICF International
Central Statistical Office (Zambia), Department for International Development (DFiD) (United Kingdom), Family Health International, MEASURE Evaluation Project, Carolina Population Center, University of North Carolina, Ministry of Health (Zambia), University of Zambia, Zambian National AIDS Council. Zambia Sexual Behavior Survey 2005. Lusaka, Zambia: Central Statistical Of
Central Statistical Office (Zambia), MEASURE Evaluation Project, Carolina Population Center, University of North Carolina, Ministry of Health (Zambia), University of Zambia, Zambian National AIDS Council. Zambia Sexual Behavior Survey 2009
ICF International, Ministry of Planning and Monitoring Implementation of the Revolution of Modernity (Congo, DR), Ministry of Public Health (Congo, DR), National Institute of Statistics (Congo, DR). Democratic Republic of the Congo Demographic and Health Survey 2013-2014. Fairfax, United States: ICF International, 2014
ICF International, INFO-STAT (Mali), Ministry of Health (Mali), National Institute of Statistics (INSTAT) (Mali), Planning and Statistics Unit, Ministry of Health (Mali). Mali Demographic and Health Survey 2012-2013. Fairfax, United States: ICF International, 2014
Central Statistical Office (Zambia), ICF International, Ministry of Health (Zambia), Tropical Diseases Research Centre, University Teaching Hospital (Zambia), University of Zambia. Zambia Demographic and Health Survey 2013-2014. Fairfax, United States: ICF International
Center for Social and Demographic Studies (Dominican Republic) (CESDEM), ICF International, Ministry of Public Health and Social Assistance (Dominican Republic). Dominican Republic Demographic and Health Survey 2013. Fairfax, United States: ICF International, 2014
El-Zanaty and Associates, ICF International, Ministry of Health and Population (Egypt). Egypt Demographic and Health Survey 2014. Fairfax, United States: ICF International

Division of Reproductive Health-Centers for Disease Control and Prevention (CDC) and Romanian Association of Public Health and Health Management. (2001) Romania Reproductive Health Survey 1999. Atlanta, United States: Centers for Disease Control and Prevention (CDC)
Macro International, Inc, National Institute of Statistics (Cameroon). Cameroon Demographic and Health Survey 2004. Fairfax, United States: ICF International
Macro International, Inc, National Statistical Office of Malawi. Malawi Demographic and Health Survey 2004-2005. Fairfax, United States: ICF International
ICF Macro, National Statistical Office of Malawi. Malawi Demographic and Health Survey 2010. Fairfax, United States: ICF International
Center for Studies of Population and Social Development (CEPAR) (Ecuador), Division of Reproductive Health-Centers for Disease Control and Prevention (CDC). Ecuador Reproductive Health Survey 1999. Atlanta, United States: Centers for Disease Control and Prevention (CDC), 2001
Center for Studies of Population and Social Development (CEPAR) (Ecuador) and Division of Reproductive Health-Centers for Disease Control and Prevention (CDC). (2005) Ecuador Reproductive Health Survey 2004. Quito, Ecuador: CEPAR
National Institute on Alcohol Abuse and Alcoholism (NIAAA), National Institutes of Health (NIH), U.S. Department of Health and Human Services. United States National Epidemiologic Survey on Alcohol and Related Conditions 2004-2005

Alfred Rusescu Institute for Mother and Child Care, John Snow, Inc., Ministry of Health (Romania), Swiss Agency for Development and Cooperation, United Nations Children's Fund (UNICEF), United Nations Population Fund (UNFPA), United States Agency for International Development (USAID), World Health Organization (WHO). Romania Reproductive Health Survey 2004
Aarhus University, Addiction Switzerland Research Institute, Alcohol Research Group, Public Health Institute, Centre for Addiction and Mental Health (Canada), Centre for Alcohol Policy Research, Turning Point Alcohol and Drug Centre (Australia), Kettil Bruun Society for Social and Epidemiological Research on Alcohol, University of North Dakota. United States Gender, Alcohol and
Emory University and Centers for Disease Control & Prevention Collaboration, ICF International, Ministry of Health (Malawi), National Statistical Office of Malawi. Malawi Demographic and Health Survey 2015-2016. Fairfax, United States: ICF International, 2017
International Institute for Population Sciences (India), Macro International, Inc. India Demographic and Health Survey 2005-2006. Fairfax, United States: ICF International
Macro International, Inc, State Statistical Committee (Ukraine), Ukrainian Center for Social Reforms (UCSR). Ukraine Demographic and Health Survey 2007. Fairfax, United States: ICF International
Albania Institute of Public Health (IPH), Ministry of Health (Albania), National Institute of Statistics (Albania), and Centers for Disease Control and Prevention. (2005) Albania Reproductive Health Survey 2001. Atlanta, United States: Centers for Disease Control and Prevention (CDC)
General Directorate of Statistics (Gabon), ICF International, Ministry of Economy, Employment and Sustainable Development (Gabon), Ministry of Health (Gabon). Gabon Demographic and Health Survey 2012. Fairfax, United States: ICF International, 2013
Kiribati National Statistics Office, Ministry of Internal and Social Affairs (Kiribati), Secretariat of the Pacific Community (SPC), United Nations Children's Fund (UNICEF), World Health Organization (WHO). Kiribati Family Health and Support Study 2008
Ministry of Health (Botswana), National AIDS Coordinating Agency (Botswana), Statistics Botswana. Botswana AIDS Impact Survey 2013. Gaborone, Botswana: Statistics Botswana, 2015
ICF International, Ministry of Health (Peru), National Institute of Statistics and Informatics (Peru), National Police of Peru (PNP). Peru Continuous Demographic and Health Survey 2014 - INEI. Lima, Peru: National Institute of Statistics and Informatics (Peru), 2015
Nybergh L, Taft C, Enander V, Krantz G. Self-reported exposure to intimate partner violence among women and men in Sweden: results from a population-based survey. BMC Public Health. 2013; 13: 845
Macro International, Inc, State Statistical Committee of Azerbaijan. Azerbaijan Demographic and Health Survey 2006. Fairfax, United States: ICF International
Haitian Institute of Childhood (IHE), Macro International, Inc. Haiti Demographic and Health Survey 2000. Fairfax, United States: ICF International
Haitian Institute of Childhood (IHE), Haitian Institute of Statistics and Informatics, Macro International, Inc. Haiti Demographic and Health Survey 2005-2006. Fairfax, United States: ICF International
Department of Statistics (Jordan), Macro International, Inc. Jordan Demographic and Health Survey 2007. Fairfax, United States: ICF International
ICF Macro, Ministry of Health and Population (Nepal), New ERA. Nepal Demographic and Health Survey 2011. Fairfax, United States: ICF International
Macro International, Inc, National Population Commission of Nigeria. Nigeria Demographic and Health Survey 2008. Fairfax, United States: ICF International, 2009
Adventist Development and Relief Agency (ADRA), Azerbaijan Ministry of Health, State Statistical Committee of Azerbaijan, and Centers for Disease Control and Prevention (CDC). (2003) Azerbaijan Reproductive Health Survey 2001. Atlanta, United States: Centers for Disease Control and Prevention (CDC)
Centers for Disease Control and Prevention (CDC). United States Behavioral Risk Factor Surveillance System 2007. Atlanta, Georgia: CDC, US Department of Health and Human Services
ICF Macro, Manhica Health Research Center (CISM), Ministry of Health (Mozambique), National Institute of Statistics (INE) (Mozambique). Mozambique Demographic and Health Survey 2011. Fairfax, United States: ICF International
Centers for Disease Control and Prevention (CDC), Haitian Institute of Childhood (IHE), Haitian Institute of Statistics and Informatics, Macro International, Inc. Haiti Demographic and Health Survey 2012. Fairfax, United States: ICF International
ICF International, National Population Commission of Nigeria. Nigeria Demographic and Health Survey 2013. Fairfax, United States: ICF International
Department of Statistics (Jordan), ICF International. Jordan Demographic and Health Survey 2012. Fairfax, United States: ICF International
Lamichhane P, Puri M, Tamang J, Dulal B. Women's Status and Violence against Young Married Women in Rural Nepal. BMC Womens Health. 2011; 19
ICF International, National Institute of Statistical, Economic and Demographic Studies (Chad). Chad Demographic and Health Survey 2014-2015. Fairfax, United States: ICF International, 2016
TNS-BMRB Scotland, Scottish Government. (2010). Scottish Crime and Justice Survey, 2008-2009. [data collection]. UK Data Service. SN: 6362, http://dx.doi.org/10.5255/UKDA-SN-6362-1
TNS-BMRB Scotland and Scottish Government, Scottish Crime and Justice Survey, 2009-2010 [computer file]. Colchester, Essex: UK Data Archive [distributor], August 2011. SN: 6685, http://dx.doi.org/10.5255/UKDA-SN-6685-1
TNS BMRB Scotland and Scottish Government, Scottish Crime and Justice Survey, 2010-2011 [computer file]. Colchester, Essex: UK Data Archive [distributor], March 2013. SN: 7229, http://dx.doi.org/10.5255/UKDA-SN-7229-1
ICF International, Ministry of the Fight Against AIDS (Côte d'Ivoire), National Institute of Statistics (Côte d'Ivoire). Côte d'Ivoire Demographic and Health Survey 2011-2012. Fairfax, United States: ICF International
Macro International, Inc, National Institute of Statistics of Rwanda. Rwanda Demographic and Health Survey 2005. Fairfax, United States: ICF International
ICF Macro, Profamilia (Colombia). Colombia Demographic and Health Survey 2009-2010. Fairfax, United States: ICF International, 2011
ICF Macro, Ministry of Health (Sao Tome and Principe), National Institute of Statistics (Sao Tome and Principe). Sao Tome and Principe Demographic and Health Survey 2008-2009. Fairfax, United States: ICF International
Georgia Center for Disease Control (NCDC), Georgia Ministry of Labor, Health and Social Affairs (MOLHSA), Division of Reproductive Health-Centers for Disease Control and Prevention (CDC). (2001) Georgia Reproductive Health Survey 1999-2000. Atlanta, United States: Centers for Disease Control and Prevention (CDC)
Georgia Center for Disease Control (NCDC), Georgian Ministry of Labor Health and Social Affairs (MOLHSA), Division of Reproductive Health, Centers for Disease Control and Prevention (CDC). Georgia Reproductive Health Survey 2005. Atlanta, United States: Centers for Disease Control and Prevention (CDC)
ICF Macro, Ministry of Health (Rwanda), National Institute of Statistics of Rwanda. Rwanda Demographic and Health Survey 2010-2011. Fairfax, United States: ICF International
Division of Reproductive Health, Centers for Disease Control and Prevention (CDC), Georgia Ministry of Labor, Health and Social Affairs, National Center for Disease Control and Public Health (Georgia), National Statistics Office of Georgia (GeoStat). Georgia Reproductive Health Survey 2010-2011
BNB Consulting (Turkey), ICON-INSTITUTE Consulting Group, Institute of Population Studies, Hacettepe University. Turkey National Research on Domestic Violence Against Women 2008
ICF International, Ministry of Health and Social Services (Namibia), Namibia Institute of Pathology, Namibia Statistics Agency. Namibia Demographic and Health Survey 2013. Fairfax, United States: ICF International
ICF International, Ministry of Health and Sports (Myanmar). Myanmar Demographic and Health Survey 2015-2016. Fairfax, United States: ICF International, 2017
ICF International, Ministry of Health (Rwanda), National Institute of Statistics of Rwanda. Rwanda Demographic and Health Survey 2014-2015. Fairfax, United States: ICF International, 2016
Umubyeyi A, Mogren I, Ntaganira J, Krantz G. Women are considerably more exposed to intimate partner violence than men in Rwanda: results from a population-based, cross-sectional study. BMC Womens Health. 2014; 14: 99
Institute of Population Studies, Hacettepe University, Ministry of Family and Social Policies (Turkey). Turkey National Research on Domestic Violence Against Women 2014
Division of Reproductive Health-Centers for Disease Control and Prevention (CDC). (2005): Paraguay Reproductive Health Survey 2004. Asunción, Paraguay, Paraguayan Center for Population Studies (CEPEP)
Macro International, Inc, National Statistics Office (Philippines). Philippines Demographic and Health Survey 2008. Fairfax, United States: ICF International, 2010
Ministry of Health and Prevention (Senegal), Research Center for Human Development (Senegal). Senegal Demographic and Health Survey 2005. Fairfax, United States: ICF International
Paraguay Center for Population Studies (CEPEP). Paraguay Reproductive Health Survey 2008. Asunción, Paraguay: Paraguayan Center for Population Studies (CEPEP)
National Opinion Research Center, University of Chicago (NORC), Renmin University, Beijing, Peking Union Medical College, Beijing, University of North Carolina. China Health and Family Life Survey 1999-2000. Chicago, USA: Population Research Center, University of Chicago
Agency of the Republic of Kazakhstan on Statistics, United Nations Children's Fund (UNICEF). Kazakhstan Multiple Indicator Cluster Survey 2010-2011. New York, United States: United Nations Children's Fund (UNICEF), 2013
General Directorate of Statistics and Forecasting (Comoros), ICF International. Comoros Demographic and Health Survey 2012-2013. Fairfax, United States: ICF International
Gambia Bureau of Statistics (GBOS), ICF International, Ministry of Health and Social Welfare (Gambia). Gambia Demographic and Health Survey 2013. Fairfax, United States: ICF International, 2015
ICF International, Philippines Statistics Authority. Philippines Demographic and Health Survey 2013. Fairfax, United States: ICF International, 2014
Jewkes R, Penn-Kekana L, Levin J, Ratsaka M, Schrieber M. Prevalence of emotional, physical and sexual abuse of women in three South African provinces. S Afr Med J. 2001; 91(5): 421-8
Center for AIDS Development, Research and Evaluation (CADRE) (South Africa), Geospace International (South Africa), Global Clinical and Viral Laboratory (South Africa), Human Sciences Research Council, Maphume Research Services, National Institute for Communicable Diseases (South Africa), South African Medical Research Council. South Africa National HIV Prevalence, Incic
Xu X, Zhu F, O'Campo P, Koenig MA, Mock V, Campbell J. Prevalence of and Risk Factors for Intimate Partner Violence in China. Am J Public Health. 2005; 95(1): 78–85
Ghana Statistical Service, Macro International, Inc, Ministry of Health (Ghana). Ghana Demographic and Health Survey 2008. Fairfax, United States: ICF International
Asociación Demográfica Salvadoreña (ADS), Division of Reproductive Health-Centers for Disease Control and Prevention (CDC). (2004) El Salvador Reproductive Health Survey 2002-2003. San Salvador, El Salvador: ADS
Asociación Demográfica Salvadoreña (ADS), Division of Reproductive Health-Centers for Disease Control and Prevention (CDC). (2009) El Salvador Reproductive Health Survey 2008. San Salvador, El Salvador: ADS
ICF International, Ministry of Health (Kyrgyzstan), National Statistical Committee of the Kyrgyz Republic. Kyrgyzstan Demographic and Health Survey 2012. Fairfax, United States: ICF International
Ministry of Women Affairs (Samoa), Secretariat of the Pacific Community (SPC). Samoa Family Health and Safety Study 2000
ICF International, Ministry of Health and Sanitation (Sierra Leone), Statistics Sierra Leone. Sierra Leone Demographic and Health Survey 2013. Fairfax, United States: ICF International, 2014
Macro International, Inc, National Statistics Directorate (Guinea). Guinea Demographic and Health Survey 2005. Fairfax, United States: ICF International
Central Statistical Office (Zimbabwe), Macro International, Inc. Zimbabwe Demographic and Health Survey 2005-2006. Fairfax, United States: ICF International
Guatemala Ministry of Health and Social Assistance, University of Valle, Division of Reproductive Health-Centers for Disease Control and Prevention (CDC). (2003) Guatemala Reproductive Health Survey 2002. Atlanta, United States: Centers for Disease Control and Prevention (CDC)
ICF Macro, Zimbabwe National Statistics Agency. Zimbabwe Demographic and Health Survey 2010-2011. Calverton, United States: ICF Macro, 2012
Directorate General of Statistics and National Accounts (Togo), ICF International, Ministry of Health (Togo), Ministry of Planning, Development and Zoning (Togo). Togo Demographic and Health Survey 2013-2014. Fairfax, United States: ICF International, 2015
ICF International, Institute of Nutrition of Central America and Panama, Ministry of Public Health and Social Assistance (Guatemala), National Statistics Institute (Guatemala), Secretary of Planning and Programming of the Presidency (Segeplán) (Guatemala). Guatemala Demographic and Health Survey 2014-2015. Fairfax, United States: ICF International, 2017
ICF International, National Microbiology Reference Laboratory, Harare Central Hospital (NMRL) (Zimbabwe), Zimbabwe National Statistics Agency. Zimbabwe Demographic and Health Survey 2015. Fairfax, United States: ICF International, 2016
Laanpere M, Ringmets I, Part K, Karro H. Intimate partner violence and sexual health outcomes: a population-based study among 16-44-year-old women in Estonia. Eur J Public Health. 2013; 23(4): 688-93
United Nations Children's Fund (UNICEF). Central African Republic Multiple Indicator Cluster Survey 2006. New York, United States: United Nations Children's Fund (UNICEF)
Macro International, Inc, National Institute of Statistics (Honduras), Secretary of Health (Honduras). Honduras Demographic and Health Survey 2005-2006. Fairfax, United States: ICF International
ICF Macro, Ministry of Finance (Timor-Leste), National Statistics Directorate (Timor-Leste). Timor-Leste Demographic and Health Survey 2009-2010. Fairfax, United States: ICF International
ICF Macro, National Bureau of Statistics (Tanzania). Tanzania Demographic and Health Survey 2009-2010. Fairfax, United States: ICF International
Honduras Family Planning Association (ASHONPLAFA), Ministry of Health (Honduras), and Division of Reproductive Health-Centers for Disease Control and Prevention (CDC). Honduras Reproductive Health Survey 2001. Tegucigalpa, Honduras: Honduras Family Planning Association (ASHONPLAFA)
ICF International, Ministry of Health (Tajikistan), Statistical Agency under the President of the Republic of Tajikistan. Tajikistan Demographic and Health Survey 2012. Fairfax, United States: ICF International, 2013
ICF Macro, National Institute of Statistics (Honduras). Honduras Demographic and Health Survey 2011-2012. Fairfax, United States: ICF International
Ma'a Fafine mo e Famili (Tonga). Tonga National Study on Domestic Violence Against Women 2008-2009
National Centre for Social Research and University of Leicester, Adult Psychiatric Morbidity Survey, 2007 [computer file]. 3rd Edition. Colchester, Essex: UK Data Archive [distributor], January 2011. SN: 6379, http://dx.doi.org/10.5255/UKDA-SN-6379-1
ICF International, Ministry of Health (Zanzibar), Ministry of Health, Community Development, Gender, Elderly and Children (MoHCDEC) (Tanzania), National Bureau of Statistics (Tanzania), Office of Chief Government Statistician (OCGS-Zanzibar). Tanzania Demographic and Health Survey 2015-2016. Fairfax, United States: ICF International, 2016
