# Supplementary material for: Estimating global injuries morbidity and mortality: methods and data used in the Global Burden of Disease 2017 study
Source: Inj Prev. 2020 Aug 24;26(Suppl 1):i125–53. doi: 10.1136/injuryprev-2019-043531 (PMC7571362; doi:10.1136/injuryprev-2019-043531)
Supplement: Supplementary data [file injuryprev-2019-043531supp004.pdf]

Location	Year	Nature of injury	Long-term disability weight
Democratic People's Republic of Korea	1990	Amputation of lower limbs, bilateral	0.2089
Democratic People's Republic of Korea	1995	Amputation of lower limbs, bilateral	0.2053
Democratic People's Republic of Korea	2000	Amputation of lower limbs, bilateral	0.2157
Democratic People's Republic of Korea	2005	Amputation of lower limbs, bilateral	0.2148
Democratic People's Republic of Korea	2010	Amputation of lower limbs, bilateral	0.2031
Democratic People's Republic of Korea	2017	Amputation of lower limbs, bilateral	0.195
Democratic People's Republic of Korea	1990	Lower airway burns	0.2252
Democratic People's Republic of Korea	1995	Lower airway burns	0.2252
Democratic People's Republic of Korea	2000	Lower airway burns	0.2252
Democratic People's Republic of Korea	2005	Lower airway burns	0.2252
Democratic People's Republic of Korea	2010	Lower airway burns	0.2252
Democratic People's Republic of Korea	2017	Lower airway burns	0.2252
Democratic People's Republic of Korea	1990	Dislocation of hip	0.0159
Democratic People's Republic of Korea	1995	Dislocation of hip	0.0159
Democratic People's Republic of Korea	2000	Dislocation of hip	0.0159
Democratic People's Republic of Korea	2005	Dislocation of hip	0.0159
Democratic People's Republic of Korea	2010	Dislocation of hip	0.0159
Democratic People's Republic of Korea	2017	Dislocation of hip	0.0159
Democratic People's Republic of Korea	1990	Dislocation of knee	0.1134
Democratic People's Republic of Korea	1995	Dislocation of knee	0.1134
Democratic People's Republic of Korea	2000	Dislocation of knee	0.1134
Democratic People's Republic of Korea	2005	Dislocation of knee	0.1134
Democratic People's Republic of Korea	2010	Dislocation of knee	0.1134
Democratic People's Republic of Korea	2017	Dislocation of knee	0.1134
Democratic People's Republic of Korea	1990	Dislocation of shoulder	0.062
Democratic People's Republic of Korea	1995	Dislocation of shoulder	0.062
Democratic People's Republic of Korea	2000	Dislocation of shoulder	0.062
Democratic People's Republic of Korea	2005	Dislocation of shoulder	0.062
Democratic People's Republic of Korea	2010	Dislocation of shoulder	0.062
Democratic People's Republic of Korea	2017	Dislocation of shoulder	0.062
Democratic People's Republic of Korea	1990	Muscle and tendon injuries, including sprains and strains lesser dislocations	0.0075
Democratic People's Republic of Korea	1995	Muscle and tendon injuries, including sprains and strains lesser dislocations	0.0075
Democratic People's Republic of Korea	2000	Muscle and tendon injuries, including sprains and strains lesser dislocations	0.0075
Democratic People's Republic of Korea	2005	Muscle and tendon injuries, including sprains and strains lesser dislocations	0.0075
Democratic People's Republic of Korea	2010	Muscle and tendon injuries, including sprains and strains lesser dislocations	0.0075
Democratic People's Republic of Korea	2017	Muscle and tendon injuries, including sprains and strains lesser dislocations	0.0075
Democratic People's Republic of Korea	1990	Fracture of clavicle, scapula, or humerus	0.0349
Democratic People's Republic of Korea	1995	Fracture of clavicle, scapula, or humerus	0.0349
Democratic People's Republic of Korea	2000	Fracture of clavicle, scapula, or humerus	0.0349
Democratic People's Republic of Korea	2005	Fracture of clavicle, scapula, or humerus	0.0349
Democratic People's Republic of Korea	2010	Fracture of clavicle, scapula, or humerus	0.0349
Democratic People's Republic of Korea	2017	Fracture of clavicle, scapula, or humerus	0.0349
Democratic People's Republic of Korea	1990	Fracture of face bones	0.0669
Democratic People's Republic of Korea	1995	Fracture of face bones	0.0669
Democratic People's Republic of Korea	2000	Fracture of face bones	0.0669
Democratic People's Republic of Korea	2005	Fracture of face bones	0.0669
Democratic People's Republic of Korea	2010	Fracture of face bones	0.0669
Democratic People's Republic of Korea	2017	Fracture of face bones	0.0669
Democratic People's Republic of Korea	1990	Fracture of foot bones except ankle	0.0264
Democratic People's Republic of Korea	1995	Fracture of foot bones except ankle	0.0264
Democratic People's Republic of Korea	2000	Fracture of foot bones except ankle	0.0264
Democratic People's Republic of Korea	2005	Fracture of foot bones except ankle	0.0264
Democratic People's Republic of Korea	2010	Fracture of foot bones except ankle	0.0264
Democratic People's Republic of Korea	2017	Fracture of foot bones except ankle	0.0264
Democratic People's Republic of Korea	1990	Fracture of hand (wrist and other distal part of hand)	0.0138
Democratic People's Republic of Korea	1995	Fracture of hand (wrist and other distal part of hand)	0.0138
Democratic People's Republic of Korea	2000	Fracture of hand (wrist and other distal part of hand)	0.0138
Democratic People's Republic of Korea	2005	Fracture of hand (wrist and other distal part of hand)	0.0138
Democratic People's Republic of Korea	2010	Fracture of hand (wrist and other distal part of hand)	0.0138
Democratic People's Republic of Korea	2017	Fracture of hand (wrist and other distal part of hand)	0.0138
Democratic People's Republic of Korea	1990	Fracture of hip	0.1709
Democratic People's Republic of Korea	1995	Fracture of hip	0.1662
Democratic People's Republic of Korea	2000	Fracture of hip	0.1799
Democratic People's Republic of Korea	2005	Fracture of hip	0.1788
Democratic People's Republic of Korea	2010	Fracture of hip	0.1633
Democratic People's Republic of Korea	2017	Fracture of hip	0.1526
Democratic People's Republic of Korea	1990	Amputation of upper limbs, bilateral	0.2046
Democratic People's Republic of Korea	1995	Amputation of upper limbs, bilateral	0.1997
Democratic People's Republic of Korea	2000	Amputation of upper limbs, bilateral	0.2139
Democratic People's Republic of Korea	2005	Amputation of upper limbs, bilateral	0.2127
Democratic People's Republic of Korea	2010	Amputation of upper limbs, bilateral	0.1967
Democratic People's Republic of Korea	2017	Amputation of upper limbs, bilateral	0.1857
Democratic People's Republic of Korea	1990	Fracture of patella, tibia or fibula, or ankle	0.0554
Democratic People's Republic of Korea	1995	Fracture of patella, tibia or fibula, or ankle	0.0554
Democratic People's Republic of Korea	2000	Fracture of patella, tibia or fibula, or ankle	0.0554
Democratic People's Republic of Korea	2005	Fracture of patella, tibia or fibula, or ankle	0.0554
Democratic People's Republic of Korea	2010	Fracture of patella, tibia or fibula, or ankle	0.0554
Democratic People's Republic of Korea	2017	Fracture of patella, tibia or fibula, or ankle	0.0554
Democratic People's Republic of Korea	1990	Fracture of pelvis	0.1823
Democratic People's Republic of Korea	1995	Fracture of pelvis	0.1823
Democratic People's Republic of Korea	2000	Fracture of pelvis	0.1823
Democratic People's Republic of Korea	2005	Fracture of pelvis	0.1823
Democratic People's Republic of Korea	2010	Fracture of pelvis	0.1823
Democratic People's Republic of Korea	2017	Fracture of pelvis	0.1823
Democratic People's Republic of Korea	1990	Fracture of radius and/or ulna	0.0435
Democratic People's Republic of Korea	1995	Fracture of radius and/or ulna	0.0435
Democratic People's Republic of Korea	2000	Fracture of radius and/or ulna	0.0435
Democratic People's Republic of Korea	2005	Fracture of radius and/or ulna	0.0435
Democratic People's Republic of Korea	2010	Fracture of radius and/or ulna	0.0435
Democratic People's Republic of Korea	2017	Fracture of radius and/or ulna	0.0435
Democratic People's Republic of Korea	1990	Fracture of skull	0.0714
Democratic People's Republic of Korea	1995	Fracture of skull	0.0714
Democratic People's Republic of Korea	2000	Fracture of skull	0.0714
Democratic People's Republic of Korea	2005	Fracture of skull	0.0714
Democratic People's Republic of Korea	2010	Fracture of skull	0.0714
Democratic People's Republic of Korea	2017	Fracture of skull	0.0714
Democratic People's Republic of Korea	1990	Fracture of sternum and/or fracture of one or more ribs	0.1027
Democratic People's Republic of Korea	1995	Fracture of sternum and/or fracture of one or more ribs	0.1027
Democratic People's Republic of Korea	2000	Fracture of sternum and/or fracture of one or more ribs	0.1027
Democratic People's Republic of Korea	2005	Fracture of sternum and/or fracture of one or more ribs	0.1027
Democratic People's Republic of Korea	2010	Fracture of sternum and/or fracture of one or more ribs	0.1027
Democratic People's Republic of Korea	2017	Fracture of sternum and/or fracture of one or more ribs	0.1027
Democratic People's Republic of Korea	1990	Fracture of vertebral column	0.1106
Democratic People's Republic of Korea	1995	Fracture of vertebral column	0.1106
Democratic People's Republic of Korea	2000	Fracture of vertebral column	0.1106
Democratic People's Republic of Korea	2005	Fracture of vertebral column	0.1106
Democratic People's Republic of Korea	2010	Fracture of vertebral column	0.1106
Democratic People's Republic of Korea	2017	Fracture of vertebral column	0.1106
Democratic People's Republic of Korea	1990	Fracture of femur, other than femoral neck	0.042
Democratic People's Republic of Korea	1995	Fracture of femur, other than femoral neck	0.042
Democratic People's Republic of Korea	2000	Fracture of femur, other than femoral neck	0.042
Democratic People's Republic of Korea	2005	Fracture of femur, other than femoral neck	0.042
Democratic People's Republic of Korea	2010	Fracture of femur, other than femoral neck	0.042
Democratic People's Republic of Korea	2017	Fracture of femur, other than femoral neck	0.042
Democratic People's Republic of Korea	1990	Minor TBI	0.1319
Democratic People's Republic of Korea	1995	Minor TBI	0.1319
Democratic People's Republic of Korea	2000	Minor TBI	0.1319
Democratic People's Republic of Korea	2005	Minor TBI	0.1319
Democratic People's Republic of Korea	2010	Minor TBI	0.1319
Democratic People's Republic of Korea	2017	Minor TBI	0.1319
Democratic People's Republic of Korea	1990	Moderate TBI	0.1637
Democratic People's Republic of Korea	1995	Moderate TBI	0.1637
Democratic People's Republic of Korea	2000	Moderate TBI	0.1637
Democratic People's Republic of Korea	2005	Moderate TBI	0.1637
Democratic People's Republic of Korea	2010	Moderate TBI	0.1637
Democratic People's Republic of Korea	2017	Moderate TBI	0.1637
Democratic People's Republic of Korea	1990	Amputation of fingers (excluding thumb)	0.0047
Democratic People's Republic of Korea	1995	Amputation of fingers (excluding thumb)	0.0047
Democratic People's Republic of Korea	2000	Amputation of fingers (excluding thumb)	0.0047
Democratic People's Republic of Korea	2005	Amputation of fingers (excluding thumb)	0.0047
Democratic People's Republic of Korea	2010	Amputation of fingers (excluding thumb)	0.0047
Democratic People's Republic of Korea	2017	Amputation of fingers (excluding thumb)	0.0047
Democratic People's Republic of Korea	1990	Spinal cord lesion at neck level	0.451
Democratic People's Republic of Korea	1995	Spinal cord lesion at neck level	0.4494
Democratic People's Republic of Korea	2000	Spinal cord lesion at neck level	0.4541
Democratic People's Republic of Korea	2005	Spinal cord lesion at neck level	0.4537
Democratic People's Republic of Korea	2010	Spinal cord lesion at neck level	0.4483
Democratic People's Republic of Korea	2017	Spinal cord lesion at neck level	0.4446
Democratic People's Republic of Korea	1990	Spinal cord lesion below neck level	0.2603
Democratic People's Republic of Korea	1995	Spinal cord lesion below neck level	0.2569
Democratic People's Republic of Korea	2000	Spinal cord lesion below neck level	0.2669
Democratic People's Republic of Korea	2005	Spinal cord lesion below neck level	0.266
Democratic People's Republic of Korea	2010	Spinal cord lesion below neck level	0.2547
Democratic People's Republic of Korea	2017	Spinal cord lesion below neck level	0.247
Democratic People's Republic of Korea	1990	Drowning and nonfatal submersion	0.2471
Democratic People's Republic of Korea	1995	Drowning and nonfatal submersion	0.2471
Democratic People's Republic of Korea	2000	Drowning and nonfatal submersion	0.2471
Democratic People's Republic of Korea	2005	Drowning and nonfatal submersion	0.2471
Democratic People's Republic of Korea	2010	Drowning and nonfatal submersion	0.2471
Democratic People's Republic of Korea	2017	Drowning and nonfatal submersion	0.2471
Democratic People's Republic of Korea	1990	Asphyxiation	0.2471
Democratic People's Republic of Korea	1995	Asphyxiation	0.2471
Democratic People's Republic of Korea	2000	Asphyxiation	0.2471
Democratic People's Republic of Korea	2005	Asphyxiation	0.2471
Democratic People's Republic of Korea	2010	Asphyxiation	0.2471
Democratic People's Republic of Korea	2017	Asphyxiation	0.2471
Democratic People's Republic of Korea	1990	Crush injury	0.1325
Democratic People's Republic of Korea	1995	Crush injury	0.1325
Democratic People's Republic of Korea	2000	Crush injury	0.1325
Democratic People's Republic of Korea	2005	Crush injury	0.1325
Democratic People's Republic of Korea	2010	Crush injury	0.1325
Democratic People's Republic of Korea	2017	Crush injury	0.1325
Democratic People's Republic of Korea	1990	Nerve injury	0.1129
Democratic People's Republic of Korea	1995	Nerve injury	0.1129
Democratic People's Republic of Korea	2000	Nerve injury	0.1129
Democratic People's Republic of Korea	2005	Nerve injury	0.1129
Democratic People's Republic of Korea	2010	Nerve injury	0.1129
Democratic People's Republic of Korea	2017	Nerve injury	0.1129
Democratic People's Republic of Korea	1990	Injury to eyes	0.0165
Democratic People's Republic of Korea	1995	Injury to eyes	0.0165
Democratic People's Republic of Korea	2000	Injury to eyes	0.0165
Democratic People's Republic of Korea	2005	Injury to eyes	0.0165
Democratic People's Republic of Korea	2010	Injury to eyes	0.0165
Democratic People's Republic of Korea	2017	Injury to eyes	0.0165
Democratic People's Republic of Korea	1990	Amputation of lower limb, unilateral	0.0831
Democratic People's Republic of Korea	1995	Amputation of lower limb, unilateral	0.0813
Democratic People's Republic of Korea	2000	Amputation of lower limb, unilateral	0.0866
Democratic People's Republic of Korea	2005	Amputation of lower limb, unilateral	0.0862
Democratic People's Republic of Korea	2010	Amputation of lower limb, unilateral	0.0801
Democratic People's Republic of Korea	2017	Amputation of lower limb, unilateral	0.076
Democratic People's Republic of Korea	1990	Open wound(s)	0.011
Democratic People's Republic of Korea	1995	Open wound(s)	0.011
Democratic People's Republic of Korea	2000	Open wound(s)	0.011
Democratic People's Republic of Korea	2005	Open wound(s)	0.011
Democratic People's Republic of Korea	2010	Open wound(s)	0.011
Democratic People's Republic of Korea	2017	Open wound(s)	0.011
Democratic People's Republic of Korea	1990	Poisoning requiring urgent care	0.1628
Democratic People's Republic of Korea	1995	Poisoning requiring urgent care	0.1628
Democratic People's Republic of Korea	2000	Poisoning requiring urgent care	0.1628
Democratic People's Republic of Korea	2005	Poisoning requiring urgent care	0.1628
Democratic People's Republic of Korea	2010	Poisoning requiring urgent care	0.1628
Democratic People's Republic of Korea	2017	Poisoning requiring urgent care	0.1628
Democratic People's Republic of Korea	1990	Severe chest injury	0.0474
Democratic People's Republic of Korea	1995	Severe chest injury	0.0474
Democratic People's Republic of Korea	2000	Severe chest injury	0.0474
Democratic People's Republic of Korea	2005	Severe chest injury	0.0474
Democratic People's Republic of Korea	2010	Severe chest injury	0.0474
Democratic People's Republic of Korea	2017	Severe chest injury	0.0474
Democratic People's Republic of Korea	1990	Internal hemorrhage in abdomen and pelvis	0.1143
Democratic People's Republic of Korea	1995	Internal hemorrhage in abdomen and pelvis	0.1143
Democratic People's Republic of Korea	2000	Internal hemorrhage in abdomen and pelvis	0.1143
Democratic People's Republic of Korea	2005	Internal hemorrhage in abdomen and pelvis	0.1143
Democratic People's Republic of Korea	2010	Internal hemorrhage in abdomen and pelvis	0.1143
Democratic People's Republic of Korea	2017	Internal hemorrhage in abdomen and pelvis	0.1143
Democratic People's Republic of Korea	1990	Contusion in any part of the body	0.0075
Democratic People's Republic of Korea	1995	Contusion in any part of the body	0.0075
Democratic People's Republic of Korea	2000	Contusion in any part of the body	0.0075
Democratic People's Republic of Korea	2005	Contusion in any part of the body	0.0075
Democratic People's Republic of Korea	2010	Contusion in any part of the body	0.0075
Democratic People's Republic of Korea	2017	Contusion in any part of the body	0.0075
Democratic People's Republic of Korea	1990	Effect of different environmental factors	0.049
Democratic People's Republic of Korea	1995	Effect of different environmental factors	0.049
Democratic People's Republic of Korea	2000	Effect of different environmental factors	0.049
Democratic People's Republic of Korea	2005	Effect of different environmental factors	0.049
Democratic People's Republic of Korea	2010	Effect of different environmental factors	0.049
Democratic People's Republic of Korea	2017	Effect of different environmental factors	0.049
Democratic People's Republic of Korea	1990	Complications following therapeutic procedures	0.0506
Democratic People's Republic of Korea	1995	Complications following therapeutic procedures	0.0506
Democratic People's Republic of Korea	2000	Complications following therapeutic procedures	0.0506
Democratic People's Republic of Korea	2005	Complications following therapeutic procedures	0.0506
Democratic People's Republic of Korea	2010	Complications following therapeutic procedures	0.0506
Democratic People's Republic of Korea	2017	Complications following therapeutic procedures	0.0506
Democratic People's Republic of Korea	1990	Multiple fractures, dislocations, crashes, wounds, sprains, and strains	0.1709
Democratic People's Republic of Korea	1995	Multiple fractures, dislocations, crashes, wounds, sprains, and strains	0.1662
Democratic People's Republic of Korea	2000	Multiple fractures, dislocations, crashes, wounds, sprains, and strains	0.1799
Democratic People's Republic of Korea	2005	Multiple fractures, dislocations, crashes, wounds, sprains, and strains	0.1788
Democratic People's Republic of Korea	2010	Multiple fractures, dislocations, crashes, wounds, sprains, and strains	0.1633
Democratic People's Republic of Korea	2017	Multiple fractures, dislocations, crashes, wounds, sprains, and strains	0.1526
Democratic People's Republic of Korea	1990	Amputation of upper limb, unilateral	0.065
Democratic People's Republic of Korea	1995	Amputation of upper limb, unilateral	0.0639
Democratic People's Republic of Korea	2000	Amputation of upper limb, unilateral	0.067
Democratic People's Republic of Korea	2005	Amputation of upper limb, unilateral	0.0668
Democratic People's Republic of Korea	2010	Amputation of upper limb, unilateral	0.0632
Democratic People's Republic of Korea	2017	Amputation of upper limb, unilateral	0.0608
Democratic People's Republic of Korea	1990	Amputation of thumb	0.0114
Democratic People's Republic of Korea	1995	Amputation of thumb	0.0114
Democratic People's Republic of Korea	2000	Amputation of thumb	0.0114
Democratic People's Republic of Korea	2005	Amputation of thumb	0.0114
Democratic People's Republic of Korea	2010	Amputation of thumb	0.0114
Democratic People's Republic of Korea	2017	Amputation of thumb	0.0114
Democratic People's Republic of Korea	1990	Amputation of toe/toes	0.0059
Democratic People's Republic of Korea	1995	Amputation of toe/toes	0.0059
Democratic People's Republic of Korea	2000	Amputation of toe/toes	0.0059
Democratic People's Republic of Korea	2005	Amputation of toe/toes	0.0059
Democratic People's Republic of Korea	2010	Amputation of toe/toes	0.0059
Democratic People's Republic of Korea	2017	Amputation of toe/toes	0.0059
Democratic People's Republic of Korea	1990	Burns, <20% total burned surface area without lower airway burns	0.0163
Democratic People's Republic of Korea	1995	Burns, <20% total burned surface area without lower airway burns	0.0163
Democratic People's Republic of Korea	2000	Burns, <20% total burned surface area without lower airway burns	0.0163
Democratic People's Republic of Korea	2005	Burns, <20% total burned surface area without lower airway burns	0.0163
Democratic People's Republic of Korea	2010	Burns, <20% total burned surface area without lower airway burns	0.0163
Democratic People's Republic of Korea	2017	Burns, <20% total burned surface area without lower airway burns	0.0163
Democratic People's Republic of Korea	1990	Burns, >=20% total burned surface area or >= 10% burned surface area if head/neck or hands/wrist involved w/o lower airway burns	0.24
Democratic People's Republic of Korea	1995	Burns, >=20% total burned surface area or >= 10% burned surface area if head/neck or hands/wrist involved w/o lower airway burns	0.2356
Democratic People's Republic of Korea	2000	Burns, >=20% total burned surface area or >= 10% burned surface area if head/neck or hands/wrist involved w/o lower airway burns	0.2484
Democratic People's Republic of Korea	2005	Burns, >=20% total burned surface area or >= 10% burned surface area if head/neck or hands/wrist involved w/o lower airway burns	0.2473
Democratic People's Republic of Korea	2010	Burns, >=20% total burned surface area or >= 10% burned surface area if head/neck or hands/wrist involved w/o lower airway burns	0.2329
Democratic People's Republic of Korea	2017	Burns, >=20% total burned surface area or >= 10% burned surface area if head/neck or hands/wrist involved w/o lower airway burns	0.223
Taiwan (Province of China)	1990	Amputation of lower limbs, bilateral	0.172
Taiwan (Province of China)	1995	Amputation of lower limbs, bilateral	0.1552
Taiwan (Province of China)	2000	Amputation of lower limbs, bilateral	0.134
Taiwan (Province of China)	2005	Amputation of lower limbs, bilateral	0.1232
Taiwan (Province of China)	2010	Amputation of lower limbs, bilateral	0.1232
Taiwan (Province of China)	2017	Amputation of lower limbs, bilateral	0.1232
Taiwan (Province of China)	1990	Lower airway burns	0.2252
Taiwan (Province of China)	1995	Lower airway burns	0.2252
Taiwan (Province of China)	2000	Lower airway burns	0.2252
Taiwan (Province of China)	2005	Lower airway burns	0.2252
Taiwan (Province of China)	2010	Lower airway burns	0.2252
Taiwan (Province of China)	2017	Lower airway burns	0.2252
Taiwan (Province of China)	1990	Dislocation of hip	0.0159
Taiwan (Province of China)	1995	Dislocation of hip	0.0159
Taiwan (Province of China)	2000	Dislocation of hip	0.0159
Taiwan (Province of China)	2005	Dislocation of hip	0.0159
Taiwan (Province of China)	2010	Dislocation of hip	0.0159
Taiwan (Province of China)	2017	Dislocation of hip	0.0159
Taiwan (Province of China)	1990	Dislocation of knee	0.1134
Taiwan (Province of China)	1995	Dislocation of knee	0.1134
Taiwan (Province of China)	2000	Dislocation of knee	0.1134
Taiwan (Province of China)	2005	Dislocation of knee	0.1134
Taiwan (Province of China)	2010	Dislocation of knee	0.1134
Taiwan (Province of China)	2017	Dislocation of knee	0.1134
Taiwan (Province of China)	1990	Dislocation of shoulder	0.062
Taiwan (Province of China)	1995	Dislocation of shoulder	0.062
Taiwan (Province of China)	2000	Dislocation of shoulder	0.062
Taiwan (Province of China)	2005	Dislocation of shoulder	0.062
Taiwan (Province of China)	2010	Dislocation of shoulder	0.062
Taiwan (Province of China)	2017	Dislocation of shoulder	0.062
Taiwan (Province of China)	1990	Muscle and tendon injuries, including sprains and strains lesser dislocations	0.0075
Taiwan (Province of China)	1995	Muscle and tendon injuries, including sprains and strains lesser dislocations	0.0075
Taiwan (Province of China)	2000	Muscle and tendon injuries, including sprains and strains lesser dislocations	0.0075
Taiwan (Province of China)	2005	Muscle and tendon injuries, including sprains and strains lesser dislocations	0.0075
Taiwan (Province of China)	2010	Muscle and tendon injuries, including sprains and strains lesser dislocations	0.0075
Taiwan (Province of China)	2017	Muscle and tendon injuries, including sprains and strains lesser dislocations	0.0075
Taiwan (Province of China)	1990	Fracture of clavicle, scapula, or humerus	0.0349
Taiwan (Province of China)	1995	Fracture of clavicle, scapula, or humerus	0.0349
Taiwan (Province of China)	2000	Fracture of clavicle, scapula, or humerus	0.0349
Taiwan (Province of China)	2005	Fracture of clavicle, scapula, or humerus	0.0349
Taiwan (Province of China)	2010	Fracture of clavicle, scapula, or humerus	0.0349
Taiwan (Province of China)	2017	Fracture of clavicle, scapula, or humerus	0.0349
Taiwan (Province of China)	1990	Fracture of face bones	0.0669
Taiwan (Province of China)	1995	Fracture of face bones	0.0669
Taiwan (Province of China)	2000	Fracture of face bones	0.0669
Taiwan (Province of China)	2005	Fracture of face bones	0.0669
Taiwan (Province of China)	2010	Fracture of face bones	0.0669
Taiwan (Province of China)	2017	Fracture of face bones	0.0669
Taiwan (Province of China)	1990	Fracture of foot bones except ankle	0.0264
Taiwan (Province of China)	1995	Fracture of foot bones except ankle	0.0264
Taiwan (Province of China)	2000	Fracture of foot bones except ankle	0.0264
Taiwan (Province of China)	2005	Fracture of foot bones except ankle	0.0264
Taiwan (Province of China)	2010	Fracture of foot bones except ankle	0.0264
Taiwan (Province of China)	2017	Fracture of foot bones except ankle	0.0264
Taiwan (Province of China)	1990	Fracture of hand (wrist and other distal part of hand)	0.0138
Taiwan (Province of China)	1995	Fracture of hand (wrist and other distal part of hand)	0.0138
Taiwan (Province of China)	2000	Fracture of hand (wrist and other distal part of hand)	0.0138
Taiwan (Province of China)	2005	Fracture of hand (wrist and other distal part of hand)	0.0138
Taiwan (Province of China)	2010	Fracture of hand (wrist and other distal part of hand)	0.0138
Taiwan (Province of China)	2017	Fracture of hand (wrist and other distal part of hand)	0.0138
Taiwan (Province of China)	1990	Fracture of hip	0.1221
Taiwan (Province of China)	1995	Fracture of hip	0.1
Taiwan (Province of China)	2000	Fracture of hip	0.0719
Taiwan (Province of China)	2005	Fracture of hip	0.0577
Taiwan (Province of China)	2010	Fracture of hip	0.0577
Taiwan (Province of China)	2017	Fracture of hip	0.0577
Taiwan (Province of China)	1990	Amputation of upper limbs, bilateral	0.1542
Taiwan (Province of China)	1995	Amputation of upper limbs, bilateral	0.1313
Taiwan (Province of China)	2000	Amputation of upper limbs, bilateral	0.1023
Taiwan (Province of China)	2005	Amputation of upper limbs, bilateral	0.0876
Taiwan (Province of China)	2010	Amputation of upper limbs, bilateral	0.0876
Taiwan (Province of China)	2017	Amputation of upper limbs, bilateral	0.0876
Taiwan (Province of China)	1990	Fracture of patella, tibia or fibula, or ankle	0.0554
Taiwan (Province of China)	1995	Fracture of patella, tibia or fibula, or ankle	0.0554
Taiwan (Province of China)	2000	Fracture of patella, tibia or fibula, or ankle	0.0554
Taiwan (Province of China)	2005	Fracture of patella, tibia or fibula, or ankle	0.0554
Taiwan (Province of China)	2010	Fracture of patella, tibia or fibula, or ankle	0.0554
Taiwan (Province of China)	2017	Fracture of patella, tibia or fibula, or ankle	0.0554
Taiwan (Province of China)	1990	Fracture of pelvis	0.1823
Taiwan (Province of China)	1995	Fracture of pelvis	0.1823
Taiwan (Province of China)	2000	Fracture of pelvis	0.1823
Taiwan (Province of China)	2005	Fracture of pelvis	0.1823
Taiwan (Province of China)	2010	Fracture of pelvis	0.1823
Taiwan (Province of China)	2017	Fracture of pelvis	0.1823
Taiwan (Province of China)	1990	Fracture of radius and/or ulna	0.0435
Taiwan (Province of China)	1995	Fracture of radius and/or ulna	0.0435
Taiwan (Province of China)	2000	Fracture of radius and/or ulna	0.0435
Taiwan (Province of China)	2005	Fracture of radius and/or ulna	0.0435
Taiwan (Province of China)	2010	Fracture of radius and/or ulna	0.0435
Taiwan (Province of China)	2017	Fracture of radius and/or ulna	0.0435
Taiwan (Province of China)	1990	Fracture of skull	0.0714
Taiwan (Province of China)	1995	Fracture of skull	0.0714
Taiwan (Province of China)	2000	Fracture of skull	0.0714
Taiwan (Province of China)	2005	Fracture of skull	0.0714
Taiwan (Province of China)	2010	Fracture of skull	0.0714
Taiwan (Province of China)	2017	Fracture of skull	0.0714
Taiwan (Province of China)	1990	Fracture of sternum and/or fracture of one or more ribs	0.1027
Taiwan (Province of China)	1995	Fracture of sternum and/or fracture of one or more ribs	0.1027
Taiwan (Province of China)	2000	Fracture of sternum and/or fracture of one or more ribs	0.1027
Taiwan (Province of China)	2005	Fracture of sternum and/or fracture of one or more ribs	0.1027
Taiwan (Province of China)	2010	Fracture of sternum and/or fracture of one or more ribs	0.1027
Taiwan (Province of China)	2017	Fracture of sternum and/or fracture of one or more ribs	0.1027
Taiwan (Province of China)	1990	Fracture of vertebral column	0.1106
Taiwan (Province of China)	1995	Fracture of vertebral column	0.1106
Taiwan (Province of China)	2000	Fracture of vertebral column	0.1106
Taiwan (Province of China)	2005	Fracture of vertebral column	0.1106
Taiwan (Province of China)	2010	Fracture of vertebral column	0.1106
Taiwan (Province of China)	2017	Fracture of vertebral column	0.1106
Taiwan (Province of China)	1990	Fracture of femur, other than femoral neck	0.042
Taiwan (Province of China)	1995	Fracture of femur, other than femoral neck	0.042
Taiwan (Province of China)	2000	Fracture of femur, other than femoral neck	0.042
Taiwan (Province of China)	2005	Fracture of femur, other than femoral neck	0.042
Taiwan (Province of China)	2010	Fracture of femur, other than femoral neck	0.042
Taiwan (Province of China)	2017	Fracture of femur, other than femoral neck	0.042
Taiwan (Province of China)	1990	Minor TBI	0.1319
Taiwan (Province of China)	1995	Minor TBI	0.1319
Taiwan (Province of China)	2000	Minor TBI	0.1319
Taiwan (Province of China)	2005	Minor TBI	0.1319
Taiwan (Province of China)	2010	Minor TBI	0.1319
Taiwan (Province of China)	2017	Minor TBI	0.1319
Taiwan (Province of China)	1990	Moderate TBI	0.1637
Taiwan (Province of China)	1995	Moderate TBI	0.1637
Taiwan (Province of China)	2000	Moderate TBI	0.1637
Taiwan (Province of China)	2005	Moderate TBI	0.1637
Taiwan (Province of China)	2010	Moderate TBI	0.1637
Taiwan (Province of China)	2017	Moderate TBI	0.1637
Taiwan (Province of China)	1990	Amputation of fingers (excluding thumb)	0.0047
Taiwan (Province of China)	1995	Amputation of fingers (excluding thumb)	0.0047
Taiwan (Province of China)	2000	Amputation of fingers (excluding thumb)	0.0047
Taiwan (Province of China)	2005	Amputation of fingers (excluding thumb)	0.0047
Taiwan (Province of China)	2010	Amputation of fingers (excluding thumb)	0.0047
Taiwan (Province of China)	2017	Amputation of fingers (excluding thumb)	0.0047
Taiwan (Province of China)	1990	Spinal cord lesion at neck level	0.4341
Taiwan (Province of China)	1995	Spinal cord lesion at neck level	0.4264
Taiwan (Province of China)	2000	Spinal cord lesion at neck level	0.4167
Taiwan (Province of China)	2005	Spinal cord lesion at neck level	0.4118
Taiwan (Province of China)	2010	Spinal cord lesion at neck level	0.4118
Taiwan (Province of China)	2017	Spinal cord lesion at neck level	0.4118
Taiwan (Province of China)	1990	Spinal cord lesion below neck level	0.2247
Taiwan (Province of China)	1995	Spinal cord lesion below neck level	0.2085
Taiwan (Province of China)	2000	Spinal cord lesion below neck level	0.188
Taiwan (Province of China)	2005	Spinal cord lesion below neck level	0.1777
Taiwan (Province of China)	2010	Spinal cord lesion below neck level	0.1777
Taiwan (Province of China)	2017	Spinal cord lesion below neck level	0.1777
Taiwan (Province of China)	1990	Drowning and nonfatal submersion	0.2471
Taiwan (Province of China)	1995	Drowning and nonfatal submersion	0.2471
Taiwan (Province of China)	2000	Drowning and nonfatal submersion	0.2471
Taiwan (Province of China)	2005	Drowning and nonfatal submersion	0.2471
Taiwan (Province of China)	2010	Drowning and nonfatal submersion	0.2471
Taiwan (Province of China)	2017	Drowning and nonfatal submersion	0.2471
Taiwan (Province of China)	1990	Asphyxiation	0.2471
Taiwan (Province of China)	1995	Asphyxiation	0.2471
Taiwan (Province of China)	2000	Asphyxiation	0.2471
Taiwan (Province of China)	2005	Asphyxiation	0.2471
Taiwan (Province of China)	2010	Asphyxiation	0.2471
Taiwan (Province of China)	2017	Asphyxiation	0.2471
Taiwan (Province of China)	1990	Crush injury	0.1325
Taiwan (Province of China)	1995	Crush injury	0.1325
Taiwan (Province of China)	2000	Crush injury	0.1325
Taiwan (Province of China)	2005	Crush injury	0.1325
Taiwan (Province of China)	2010	Crush injury	0.1325
Taiwan (Province of China)	2017	Crush injury	0.1325
Taiwan (Province of China)	1990	Nerve injury	0.1129
Taiwan (Province of China)	1995	Nerve injury	0.1129
Taiwan (Province of China)	2000	Nerve injury	0.1129
Taiwan (Province of China)	2005	Nerve injury	0.1129
Taiwan (Province of China)	2010	Nerve injury	0.1129
Taiwan (Province of China)	2017	Nerve injury	0.1129
Taiwan (Province of China)	1990	Injury to eyes	0.0165
Taiwan (Province of China)	1995	Injury to eyes	0.0165
Taiwan (Province of China)	2000	Injury to eyes	0.0165
Taiwan (Province of China)	2005	Injury to eyes	0.0165

Taiwan (Province of China)	2010	Injury to eyes	0.0165
Taiwan (Province of China)	2017	Injury to eyes	0.0165
Taiwan (Province of China)	1990	Amputation of lower limb, unilateral	0.0641
Taiwan (Province of China)	1995	Amputation of lower limb, unilateral	0.0554
Taiwan (Province of China)	2000	Amputation of lower limb, unilateral	0.0445
Taiwan (Province of China)	2005	Amputation of lower limb, unilateral	0.039
Taiwan (Province of China)	2010	Amputation of lower limb, unilateral	0.039
Taiwan (Province of China)	2017	Amputation of lower limb, unilateral	0.039
Taiwan (Province of China)	1990	Open wound(s)	0.011
Taiwan (Province of China)	1995	Open wound(s)	0.011
Taiwan (Province of China)	2000	Open wound(s)	0.011
Taiwan (Province of China)	2005	Open wound(s)	0.011
Taiwan (Province of China)	2010	Open wound(s)	0.011
Taiwan (Province of China)	2017	Open wound(s)	0.011
Taiwan (Province of China)	1990	Poisoning requiring urgent care	0.1628
Taiwan (Province of China)	1995	Poisoning requiring urgent care	0.1628
Taiwan (Province of China)	2000	Poisoning requiring urgent care	0.1628
Taiwan (Province of China)	2005	Poisoning requiring urgent care	0.1628
Taiwan (Province of China)	2010	Poisoning requiring urgent care	0.1628
Taiwan (Province of China)	2017	Poisoning requiring urgent care	0.1628
Taiwan (Province of China)	1990	Severe chest injury	0.0474
Taiwan (Province of China)	1995	Severe chest injury	0.0474
Taiwan (Province of China)	2000	Severe chest injury	0.0474
Taiwan (Province of China)	2005	Severe chest injury	0.0474
Taiwan (Province of China)	2010	Severe chest injury	0.0474
Taiwan (Province of China)	2017	Severe chest injury	0.0474
Taiwan (Province of China)	1990	Internal hemorrhage in abdomen and pelvis	0.1143
Taiwan (Province of China)	1995	Internal hemorrhage in abdomen and pelvis	0.1143
Taiwan (Province of China)	2000	Internal hemorrhage in abdomen and pelvis	0.1143
Taiwan (Province of China)	2005	Internal hemorrhage in abdomen and pelvis	0.1143
Taiwan (Province of China)	2010	Internal hemorrhage in abdomen and pelvis	0.1143
Taiwan (Province of China)	2017	Internal hemorrhage in abdomen and pelvis	0.1143
Taiwan (Province of China)	1990	Contusion in any part of the body	0.0075
Taiwan (Province of China)	1995	Contusion in any part of the body	0.0075
Taiwan (Province of China)	2000	Contusion in any part of the body	0.0075
Taiwan (Province of China)	2005	Contusion in any part of the body	0.0075
Taiwan (Province of China)	2010	Contusion in any part of the body	0.0075
Taiwan (Province of China)	2017	Contusion in any part of the body	0.0075
Taiwan (Province of China)	1990	Effect of different environmental factors	0.049
Taiwan (Province of China)	1995	Effect of different environmental factors	0.049
Taiwan (Province of China)	2000	Effect of different environmental factors	0.049
Taiwan (Province of China)	2005	Effect of different environmental factors	0.049
Taiwan (Province of China)	2010	Effect of different environmental factors	0.049
Taiwan (Province of China)	2017	Effect of different environmental factors	0.049
Taiwan (Province of China)	1990	Complications following therapeutic procedures	0.0506
Taiwan (Province of China)	1995	Complications following therapeutic procedures	0.0506
Taiwan (Province of China)	2000	Complications following therapeutic procedures	0.0506
Taiwan (Province of China)	2005	Complications following therapeutic procedures	0.0506
Taiwan (Province of China)	2010	Complications following therapeutic procedures	0.0506
Taiwan (Province of China)	2017	Complications following therapeutic procedures	0.0506
Taiwan (Province of China)	1990	Multiple fractures, dislocations, crashes, wounds, sprains, and strains	0.1221
Taiwan (Province of China)	1995	Multiple fractures, dislocations, crashes, wounds, sprains, and strains	0.1
Taiwan (Province of China)	2000	Multiple fractures, dislocations, crashes, wounds, sprains, and strains	0.0719
Taiwan (Province of China)	2005	Multiple fractures, dislocations, crashes, wounds, sprains, and strains	0.0577
Taiwan (Province of China)	2010	Multiple fractures, dislocations, crashes, wounds, sprains, and strains	0.0577
Taiwan (Province of China)	2017	Multiple fractures, dislocations, crashes, wounds, sprains, and strains	0.0577
Taiwan (Province of China)	1990	Amputation of upper limb, unilateral	0.0538
Taiwan (Province of China)	1995	Amputation of upper limb, unilateral	0.0487
Taiwan (Province of China)	2000	Amputation of upper limb, unilateral	0.0422
Taiwan (Province of China)	2005	Amputation of upper limb, unilateral	0.039
Taiwan (Province of China)	2010	Amputation of upper limb, unilateral	0.039
Taiwan (Province of China)	2017	Amputation of upper limb, unilateral	0.039
Taiwan (Province of China)	1990	Amputation of thumb	0.0114
Taiwan (Province of China)	1995	Amputation of thumb	0.0114
Taiwan (Province of China)	2000	Amputation of thumb	0.0114
Taiwan (Province of China)	2005	Amputation of thumb	0.0114
Taiwan (Province of China)	2010	Amputation of thumb	0.0114
Taiwan (Province of China)	2017	Amputation of thumb	0.0114
Taiwan (Province of China)	1990	Amputation of toe/toes	0.0059
Taiwan (Province of China)	1995	Amputation of toe/toes	0.0059
Taiwan (Province of China)	2000	Amputation of toe/toes	0.0059
Taiwan (Province of China)	2005	Amputation of toe/toes	0.0059
Taiwan (Province of China)	2010	Amputation of toe/toes	0.0059
Taiwan (Province of China)	2017	Amputation of toe/toes	0.0059
Taiwan (Province of China)	1990	Burns, <20% total burned surface area without lower airway burns	0.0163
Taiwan (Province of China)	1995	Burns, <20% total burned surface area without lower airway burns	0.0163
Taiwan (Province of China)	2000	Burns, <20% total burned surface area without lower airway burns	0.0163
Taiwan (Province of China)	2005	Burns, <20% total burned surface area without lower airway burns	0.0163
Taiwan (Province of China)	2010	Burns, <20% total burned surface area without lower airway burns	0.0163
Taiwan (Province of China)	2017	Burns, <20% total burned surface area without lower airway burns	0.0163
Taiwan (Province of China)	1990	Burns, >=20% total burned surface area or >= 10% burned surface area if head/neck or hands/wrist involved w/o lower airway burns	0.1946
Taiwan (Province of China)	1995	Burns, >=20% total burned surface area or >= 10% burned surface area if head/neck or hands/wrist involved w/o lower airway burns	0.1739
Taiwan (Province of China)	2000	Burns, >=20% total burned surface area or >= 10% burned surface area if head/neck or hands/wrist involved w/o lower airway burns	0.1478
Taiwan (Province of China)	2005	Burns, >=20% total burned surface area or >= 10% burned surface area if head/neck or hands/wrist involved w/o lower airway burns	0.1346
Taiwan (Province of China)	2010	Burns, >=20% total burned surface area or >= 10% burned surface area if head/neck or hands/wrist involved w/o lower airway burns	0.1346
Taiwan (Province of China)	2017	Burns, >=20% total burned surface area or >= 10% burned surface area if head/neck or hands/wrist involved w/o lower airway burns	0.1346
Cambodia	1990	Amputation of lower limbs, bilateral	0.3077
Cambodia	1995	Amputation of lower limbs, bilateral	0.3047
Cambodia	2000	Amputation of lower limbs, bilateral	0.2978
Cambodia	2005	Amputation of lower limbs, bilateral	0.278
Cambodia	2010	Amputation of lower limbs, bilateral	0.2657
Cambodia	2017	Amputation of lower limbs, bilateral	0.2403
Cambodia	1990	Lower airway burns	0.2252
Cambodia	1995	Lower airway burns	0.2252
Cambodia	2000	Lower airway burns	0.2252
Cambodia	2005	Lower airway burns	0.2252
Cambodia	2010	Lower airway burns	0.2252
Cambodia	2017	Lower airway burns	0.2252
Cambodia	1990	Dislocation of hip	0.0159
Cambodia	1995	Dislocation of hip	0.0159
Cambodia	2000	Dislocation of hip	0.0159
Cambodia	2005	Dislocation of hip	0.0159
Cambodia	2010	Dislocation of hip	0.0159
Cambodia	2017	Dislocation of hip	0.0159
Cambodia	1990	Dislocation of knee	0.1134
Cambodia	1995	Dislocation of knee	0.1134
Cambodia	2000	Dislocation of knee	0.1134
Cambodia	2005	Dislocation of knee	0.1134
Cambodia	2010	Dislocation of knee	0.1134
Cambodia	2017	Dislocation of knee	0.1134
Cambodia	1990	Dislocation of shoulder	0.062
Cambodia	1995	Dislocation of shoulder	0.062
Cambodia	2000	Dislocation of shoulder	0.062
Cambodia	2005	Dislocation of shoulder	0.062
Cambodia	2010	Dislocation of shoulder	0.062
Cambodia	2017	Dislocation of shoulder	0.062
Cambodia	1990	Muscle and tendon injuries, including sprains and strains lesser dislocations	0.0075
Cambodia	1995	Muscle and tendon injuries, including sprains and strains lesser dislocations	0.0075
Cambodia	2000	Muscle and tendon injuries, including sprains and strains lesser dislocations	0.0075
Cambodia	2005	Muscle and tendon injuries, including sprains and strains lesser dislocations	0.0075
Cambodia	2010	Muscle and tendon injuries, including sprains and strains lesser dislocations	0.0075
Cambodia	2017	Muscle and tendon injuries, including sprains and strains lesser dislocations	0.0075
Cambodia	1990	Fracture of clavicle, scapula, or humerus	0.0349
Cambodia	1995	Fracture of clavicle, scapula, or humerus	0.0349
Cambodia	2000	Fracture of clavicle, scapula, or humerus	0.0349
Cambodia	2005	Fracture of clavicle, scapula, or humerus	0.0349
Cambodia	2010	Fracture of clavicle, scapula, or humerus	0.0349
Cambodia	2017	Fracture of clavicle, scapula, or humerus	0.0349
Cambodia	1990	Fracture of face bones	0.0669
Cambodia	1995	Fracture of face bones	0.0669
Cambodia	2000	Fracture of face bones	0.0669
Cambodia	2005	Fracture of face bones	0.0669
Cambodia	2010	Fracture of face bones	0.0669
Cambodia	2017	Fracture of face bones	0.0669
Cambodia	1990	Fracture of foot bones except ankle	0.0264
Cambodia	1995	Fracture of foot bones except ankle	0.0264
Cambodia	2000	Fracture of foot bones except ankle	0.0264
Cambodia	2005	Fracture of foot bones except ankle	0.0264
Cambodia	2010	Fracture of foot bones except ankle	0.0264
Cambodia	2017	Fracture of foot bones except ankle	0.0264
Cambodia	1990	Fracture of hand (wrist and other distal part of hand)	0.0138
Cambodia	1995	Fracture of hand (wrist and other distal part of hand)	0.0138
Cambodia	2000	Fracture of hand (wrist and other distal part of hand)	0.0138
Cambodia	2005	Fracture of hand (wrist and other distal part of hand)	0.0138
Cambodia	2010	Fracture of hand (wrist and other distal part of hand)	0.0138
Cambodia	2017	Fracture of hand (wrist and other distal part of hand)	0.0138
Cambodia	1990	Fracture of hip	0.3017
Cambodia	1995	Fracture of hip	0.2976
Cambodia	2000	Fracture of hip	0.2886
Cambodia	2005	Fracture of hip	0.2624
Cambodia	2010	Fracture of hip	0.2461
Cambodia	2017	Fracture of hip	0.2126
Cambodia	1990	Amputation of upper limbs, bilateral	0.3396
Cambodia	1995	Amputation of upper limbs, bilateral	0.3354
Cambodia	2000	Amputation of upper limbs, bilateral	0.326
Cambodia	2005	Amputation of upper limbs, bilateral	0.299
Cambodia	2010	Amputation of upper limbs, bilateral	0.2822
Cambodia	2017	Amputation of upper limbs, bilateral	0.2476
Cambodia	1990	Fracture of patella, tibia or fibula, or ankle	0.0554
Cambodia	1995	Fracture of patella, tibia or fibula, or ankle	0.0554
Cambodia	2000	Fracture of patella, tibia or fibula, or ankle	0.0554
Cambodia	2005	Fracture of patella, tibia or fibula, or ankle	0.0554
Cambodia	2010	Fracture of patella, tibia or fibula, or ankle	0.0554
Cambodia	2017	Fracture of patella, tibia or fibula, or ankle	0.0554
Cambodia	1990	Fracture of pelvis	0.1823
Cambodia	1995	Fracture of pelvis	0.1823
Cambodia	2000	Fracture of pelvis	0.1823
Cambodia	2005	Fracture of pelvis	0.1823
Cambodia	2010	Fracture of pelvis	0.1823
Cambodia	2017	Fracture of pelvis	0.1823
Cambodia	1990	Fracture of radius and/or ulna	0.0435
Cambodia	1995	Fracture of radius and/or ulna	0.0435
Cambodia	2000	Fracture of radius and/or ulna	0.0435
Cambodia	2005	Fracture of radius and/or ulna	0.0435
Cambodia	2010	Fracture of radius and/or ulna	0.0435
Cambodia	2017	Fracture of radius and/or ulna	0.0435
Cambodia	1990	Fracture of skull	0.0714
Cambodia	1995	Fracture of skull	0.0714
Cambodia	2000	Fracture of skull	0.0714
Cambodia	2005	Fracture of skull	0.0714
Cambodia	2010	Fracture of skull	0.0714
Cambodia	2017	Fracture of skull	0.0714
Cambodia	1990	Fracture of sternum and/or fracture of one or more ribs	0.1027
Cambodia	1995	Fracture of sternum and/or fracture of one or more ribs	0.1027
Cambodia	2000	Fracture of sternum and/or fracture of one or more ribs	0.1027
Cambodia	2005	Fracture of sternum and/or fracture of one or more ribs	0.1027
Cambodia	2010	Fracture of sternum and/or fracture of one or more ribs	0.1027
Cambodia	2017	Fracture of sternum and/or fracture of one or more ribs	0.1027
Cambodia	1990	Fracture of vertebral column	0.1106
Cambodia	1995	Fracture of vertebral column	0.1106
Cambodia	2000	Fracture of vertebral column	0.1106
Cambodia	2005	Fracture of vertebral column	0.1106
Cambodia	2010	Fracture of vertebral column	0.1106
Cambodia	2017	Fracture of vertebral column	0.1106
Cambodia	1990	Fracture of femur, other than femoral neck	0.042
Cambodia	1995	Fracture of femur, other than femoral neck	0.042
Cambodia	2000	Fracture of femur, other than femoral neck	0.042
Cambodia	2005	Fracture of femur, other than femoral neck	0.042
Cambodia	2010	Fracture of femur, other than femoral neck	0.042
Cambodia	2017	Fracture of femur, other than femoral neck	0.042
Cambodia	1990	Minor TBI	0.1319
Cambodia	1995	Minor TBI	0.1319
Cambodia	2000	Minor TBI	0.1319
Cambodia	2005	Minor TBI	0.1319
Cambodia	2010	Minor TBI	0.1319
Cambodia	2017	Minor TBI	0.1319
Cambodia	1990	Moderate TBI	0.1637
Cambodia	1995	Moderate TBI	0.1637
Cambodia	2000	Moderate TBI	0.1637
Cambodia	2005	Moderate TBI	0.1637
Cambodia	2010	Moderate TBI	0.1637
Cambodia	2017	Moderate TBI	0.1637
Cambodia	1990	Amputation of fingers (excluding thumb)	0.0047
Cambodia	1995	Amputation of fingers (excluding thumb)	0.0047
Cambodia	2000	Amputation of fingers (excluding thumb)	0.0047
Cambodia	2005	Amputation of fingers (excluding thumb)	0.0047
Cambodia	2010	Amputation of fingers (excluding thumb)	0.0047
Cambodia	2017	Amputation of fingers (excluding thumb)	0.0047
Cambodia	1990	Spinal cord lesion at neck level	0.4962
Cambodia	1995	Spinal cord lesion at neck level	0.4948
Cambodia	2000	Spinal cord lesion at neck level	0.4917
Cambodia	2005	Spinal cord lesion at neck level	0.4826
Cambodia	2010	Spinal cord lesion at neck level	0.477
Cambodia	2017	Spinal cord lesion at neck level	0.4654
Cambodia	1990	Spinal cord lesion below neck level	0.3558
Cambodia	1995	Spinal cord lesion below neck level	0.3528
Cambodia	2000	Spinal cord lesion below neck level	0.3462
Cambodia	2005	Spinal cord lesion below neck level	0.3271
Cambodia	2010	Spinal cord lesion below neck level	0.3152
Cambodia	2017	Spinal cord lesion below neck level	0.2907
Cambodia	1990	Drowning and nonfatal submersion	0.2471
Cambodia	1995	Drowning and nonfatal submersion	0.2471
Cambodia	2000	Drowning and nonfatal submersion	0.2471
Cambodia	2005	Drowning and nonfatal submersion	0.2471
Cambodia	2010	Drowning and nonfatal submersion	0.2471
Cambodia	2017	Drowning and nonfatal submersion	0.2471
Cambodia	1990	Asphyxiation	0.2471
Cambodia	1995	Asphyxiation	0.2471
Cambodia	2000	Asphyxiation	0.2471
Cambodia	2005	Asphyxiation	0.2471
Cambodia	2010	Asphyxiation	0.2471
Cambodia	2017	Asphyxiation	0.2471
Cambodia	1990	Crush injury	0.1325
Cambodia	1995	Crush injury	0.1325
Cambodia	2000	Crush injury	0.1325
Cambodia	2005	Crush injury	0.1325
Cambodia	2010	Crush injury	0.1325
Cambodia	2017	Crush injury	0.1325
Cambodia	1990	Nerve injury	0.1129
Cambodia	1995	Nerve injury	0.1129
Cambodia	2000	Nerve injury	0.1129
Cambodia	2005	Nerve injury	0.1129
Cambodia	2010	Nerve injury	0.1129
Cambodia	2017	Nerve injury	0.1129
Cambodia	1990	Injury to eyes	0.0165
Cambodia	1995	Injury to eyes	0.0165
Cambodia	2000	Injury to eyes	0.0165
Cambodia	2005	Injury to eyes	0.0165
Cambodia	2010	Injury to eyes	0.0165
Cambodia	2017	Injury to eyes	0.0165
Cambodia	1990	Amputation of lower limb, unilateral	0.1341
Cambodia	1995	Amputation of lower limb, unilateral	0.1325
Cambodia	2000	Amputation of lower limb, unilateral	0.1289
Cambodia	2005	Amputation of lower limb, unilateral	0.1188
Cambodia	2010	Amputation of lower limb, unilateral	0.1124
Cambodia	2017	Amputation of lower limb, unilateral	0.0993
Cambodia	1990	Open wound(s)	0.011
Cambodia	1995	Open wound(s)	0.011
Cambodia	2000	Open wound(s)	0.011
Cambodia	2005	Open wound(s)	0.011
Cambodia	2010	Open wound(s)	0.011
Cambodia	2017	Open wound(s)	0.011
Cambodia	1990	Poisoning requiring urgent care	0.1628
Cambodia	1995	Poisoning requiring urgent care	0.1628
Cambodia	2000	Poisoning requiring urgent care	0.1628
Cambodia	2005	Poisoning requiring urgent care	0.1628
Cambodia	2010	Poisoning requiring urgent care	0.1628
Cambodia	2017	Poisoning requiring urgent care	0.1628
Cambodia	1990	Severe chest injury	0.0474
Cambodia	1995	Severe chest injury	0.0474
Cambodia	2000	Severe chest injury	0.0474
Cambodia	2005	Severe chest injury	0.0474
Cambodia	2010	Severe chest injury	0.0474
Cambodia	2017	Severe chest injury	0.0474
Cambodia	1990	Internal hemorrhage in abdomen and pelvis	0.1143
Cambodia	1995	Internal hemorrhage in abdomen and pelvis	0.1143
Cambodia	2000	Internal hemorrhage in abdomen and pelvis	0.1143
Cambodia	2005	Internal hemorrhage in abdomen and pelvis	0.1143
Cambodia	2010	Internal hemorrhage in abdomen and pelvis	0.1143
Cambodia	2017	Internal hemorrhage in abdomen and pelvis	0.1143
Cambodia	1990	Contusion in any part of the body	0.0075
Cambodia	1995	Contusion in any part of the body	0.0075
Cambodia	2000	Contusion in any part of the body	0.0075
Cambodia	2005	Contusion in any part of the body	0.0075
Cambodia	2010	Contusion in any part of the body	0.0075
Cambodia	2017	Contusion in any part of the body	0.0075
Cambodia	1990	Effect of different environmental factors	0.049
Cambodia	1995	Effect of different environmental factors	0.049
Cambodia	2000	Effect of different environmental factors	0.049
Cambodia	2005	Effect of different environmental factors	0.049
Cambodia	2010	Effect of different environmental factors	0.049
Cambodia	2017	Effect of different environmental factors	0.049
Cambodia	1990	Complications following therapeutic procedures	0.0506
Cambodia	1995	Complications following therapeutic procedures	0.0506
Cambodia	2000	Complications following therapeutic procedures	0.0506
Cambodia	2005	Complications following therapeutic procedures	0.0506
Cambodia	2010	Complications following therapeutic procedures	0.0506
Cambodia	2017	Complications following therapeutic procedures	0.0506
Cambodia	1990	Multiple fractures, dislocations, crashes, wounds, sprains, and strains	0.3017
Cambodia	1995	Multiple fractures, dislocations, crashes, wounds, sprains, and strains	0.2976
Cambodia	2000	Multiple fractures, dislocations, crashes, wounds, sprains, and strains	0.2886
Cambodia	2005	Multiple fractures, dislocations, crashes, wounds, sprains, and strains	0.2624
Cambodia	2010	Multiple fractures, dislocations, crashes, wounds, sprains, and strains	0.2461
Cambodia	2017	Multiple fractures, dislocations, crashes, wounds, sprains, and strains	0.2126
Cambodia	1990	Amputation of upper limb, unilateral	0.095
Cambodia	1995	Amputation of upper limb, unilateral	0.094
Cambodia	2000	Amputation of upper limb, unilateral	0.092
Cambodia	2005	Amputation of upper limb, unilateral	0.086
Cambodia	2010	Amputation of upper limb, unilateral	0.0822
Cambodia	2017	Amputation of upper limb, unilateral	0.0745
Cambodia	1990	Amputation of thumb	0.0114
Cambodia	1995	Amputation of thumb	0.0114
Cambodia	2000	Amputation of thumb	0.0114
Cambodia	2005	Amputation of thumb	0.0114
Cambodia	2010	Amputation of thumb	0.0114
Cambodia	2017	Amputation of thumb	0.0114
Cambodia	1990	Amputation of toe/toes	0.0059
Cambodia	1995	Amputation of toe/toes	0.0059
Cambodia	2000	Amputation of toe/toes	0.0059
Cambodia	2005	Amputation of toe/toes	0.0059
Cambodia	2010	Amputation of toe/toes	0.0059
Cambodia	2017	Amputation of toe/toes	0.0059
Cambodia	1990	Burns, <20% total burned surface area without lower airway burns	0.0163
Cambodia	1995	Burns, <20% total burned surface area without lower airway burns	0.0163
Cambodia	2000	Burns, <20% total burned surface area without lower airway burns	0.0163
Cambodia	2005	Burns, <20% total burned surface area without lower airway burns	0.0163
Cambodia	2010	Burns, <20% total burned surface area without lower airway burns	0.0163
Cambodia	2017	Burns, <20% total burned surface area without lower airway burns	0.0163
Cambodia	1990	Burns, >=20% total burned surface area or >= 10% burned surface area if head/neck or hands/wrist involved w/o lower airway burns	0.3618
Cambodia	1995	Burns, >=20% total burned surface area or >= 10% burned surface area if head/neck or hands/wrist involved w/o lower airway burns	0.358
Cambodia	2000	Burns, >=20% total burned surface area or >= 10% burned surface area if head/neck or hands/wrist involved w/o lower airway burns	0.3496
Cambodia	2005	Burns, >=20% total burned surface area or >= 10% burned surface area if head/neck or hands/wrist involved w/o lower airway burns	0.3252
Cambodia	2010	Burns, >=20% total burned surface area or >= 10% burned surface area if head/neck or hands/wrist involved w/o lower airway burns	0.31
Cambodia	2017	Burns, >=20% total burned surface area or >= 10% burned surface area if head/neck or hands/wrist involved w/o lower airway burns	0.2788
Lao People's Democratic Republic	1990	Amputation of lower limbs, bilateral	0.3155
Lao People's Democratic Republic	1995	Amputation of lower limbs, bilateral	0.31
Lao People's Democratic Republic	2000	Amputation of lower limbs, bilateral	0.3026
Lao People's Democratic Republic	2005	Amputation of lower limbs, bilateral	0.2918
Lao People's Democratic Republic	2010	Amputation of lower limbs, bilateral	0.2742
Lao People's Democratic Republic	2017	Amputation of lower limbs, bilateral	0.2495
Lao People's Democratic Republic	1990	Lower airway burns	0.2252
Lao People's Democratic Republic	1995	Lower airway burns	0.2252
Lao People's Democratic Republic	2000	Lower airway burns	0.2252
Lao People's Democratic Republic	2005	Lower airway burns	0.2252
Lao People's Democratic Republic	2010	Lower airway burns	0.2252
Lao People's Democratic Republic	2017	Lower airway burns	0.2252
Lao People's Democratic Republic	1990	Dislocation of hip	0.0159
Lao People's Democratic Republic	1995	Dislocation of hip	0.0159
Lao People's Democratic Republic	2000	Dislocation of hip	0.0159
Lao People's Democratic Republic	2005	Dislocation of hip	0.0159
Lao People's Democratic Republic	2010	Dislocation of hip	0.0159
Lao People's Democratic Republic	2017	Dislocation of hip	0.0159
Lao People's Democratic Republic	1990	Dislocation of knee	0.1134
Lao People's Democratic Republic	1995	Dislocation of knee	0.1134
Lao People's Democratic Republic	2000	Dislocation of knee	0.1134
Lao People's Democratic Republic	2005	Dislocation of knee	0.1134
Lao People's Democratic Republic	2010	Dislocation of knee	0.1134
Lao People's Democratic Republic	2017	Dislocation of knee	0.1134
Lao People's Democratic Republic	1990	Dislocation of shoulder	0.062
Lao People's Democratic Republic	1995	Dislocation of shoulder	0.062
Lao People's Democratic Republic	2000	Dislocation of shoulder	0.062
Lao People's Democratic Republic	2005	Dislocation of shoulder	0.062
Lao People's Democratic Republic	2010	Dislocation of shoulder	0.062
Lao People's Democratic Republic	2017	Dislocation of shoulder	0.062
Lao People's Democratic Republic	1990	Muscle and tendon injuries, including sprains and strains lesser dislocations	0.0075
Lao People's Democratic Republic	1995	Muscle and tendon injuries, including sprains and strains lesser dislocations	0.0075
Lao People's Democratic Republic	2000	Muscle and tendon injuries, including sprains and strains lesser dislocations	0.0075
Lao People's Democratic Republic	2005	Muscle and tendon injuries, including sprains and strains lesser dislocations	0.0075
Lao People's Democratic Republic	2010	Muscle and tendon injuries, including sprains and strains lesser dislocations	0.0075
Lao People's Democratic Republic	2017	Muscle and tendon injuries, including sprains and strains lesser dislocations	0.0075
Lao People's Democratic Republic	1990	Fracture of clavicle, scapula, or humerus	0.0349
Lao People's Democratic Republic	1995	Fracture of clavicle, scapula, or humerus	0.0349
Lao People's Democratic Republic	2000	Fracture of clavicle, scapula, or humerus	0.0349
Lao People's Democratic Republic	2005	Fracture of clavicle, scapula, or humerus	0.0349
Lao People's Democratic Republic	2010	Fracture of clavicle, scapula, or humerus	0.0349
Lao People's Democratic Republic	2017	Fracture of clavicle, scapula, or humerus	0.0349
Lao People's Democratic Republic	1990	Fracture of face bones	0.0669
Lao People's Democratic Republic	1995	Fracture of face bones	0.0669
Lao People's Democratic Republic	2000	Fracture of face bones	0.0669
Lao People's Democratic Republic	2005	Fracture of face bones	0.0669
Lao People's Democratic Republic	2010	Fracture of face bones	0.0669
Lao People's Democratic Republic	2017	Fracture of face bones	0.0669
Lao People's Democratic Republic	1990	Fracture of foot bones except ankle	0.0264
Lao People's Democratic Republic	1995	Fracture of foot bones except ankle	0.0264
Lao People's Democratic Republic	2000	Fracture of foot bones except ankle	0.0264
Lao People's Democratic Republic	2005	Fracture of foot bones except ankle	0.0264
Lao People's Democratic Republic	2010	Fracture of foot bones except ankle	0.0264
Lao People's Democratic Republic	2017	Fracture of foot bones except ankle	0.0264
Lao People's Democratic Republic	1990	Fracture of hand (wrist and other distal part of hand)	0.0138
Lao People's Democratic Republic	1995	Fracture of hand (wrist and other distal part of hand)	0.0138
Lao People's Democratic Republic	2000	Fracture of hand (wrist and other distal part of hand)	0.0138
Lao People's Democratic Republic	2005	Fracture of hand (wrist and other distal part of hand)	0.0138
Lao People's Democratic Republic	2010	Fracture of hand (wrist and other distal part of hand)	0.0138
Lao People's Democratic Republic	2017	Fracture of hand (wrist and other distal part of hand)	0.0138
Lao People's Democratic Republic	1990	Fracture of hip	0.3119
Lao People's Democratic Republic	1995	Fracture of hip	0.3047
Lao People's Democratic Republic	2000	Fracture of hip	0.2949
Lao People's Democratic Republic	2005	Fracture of hip	0.2807
Lao People's Democratic Republic	2010	Fracture of hip	0.2573
Lao People's Democratic Republic	2017	Fracture of hip	0.2246
Lao People's Democratic Republic	1990	Amputation of upper limbs, bilateral	0.3501
Lao People's Democratic Republic	1995	Amputation of upper limbs, bilateral	0.3427
Lao People's Democratic Republic	2000	Amputation of upper limbs, bilateral	0.3326
Lao People's Democratic Republic	2005	Amputation of upper limbs, bilateral	0.3179
Lao People's Democratic Republic	2010	Amputation of upper limbs, bilateral	0.2938
Lao People's Democratic Republic	2017	Amputation of upper limbs, bilateral	0.26
Lao People's Democratic Republic	1990	Fracture of patella, tibia or fibula, or ankle	0.0554
Lao People's Democratic Republic	1995	Fracture of patella, tibia or fibula, or ankle	0.0554
Lao People's Democratic Republic	2000	Fracture of patella, tibia or fibula, or ankle	0.0554
Lao People's Democratic Republic	2005	Fracture of patella, tibia or fibula, or ankle	0.0554
Lao People's Democratic Republic	2010	Fracture of patella, tibia or fibula, or ankle	0.0554
Lao People's Democratic Republic	2017	Fracture of patella, tibia or fibula, or ankle	0.0554
Lao People's Democratic Republic	1990	Fracture of pelvis	0.1823
Lao People's Democratic Republic	1995	Fracture of pelvis	0.1823
Lao People's Democratic Republic	2000	Fracture of pelvis	0.1823
Lao People's Democratic Republic	2005	Fracture of pelvis	0.1823
Lao People's Democratic Republic	2010	Fracture of pelvis	0.1823
Lao People's Democratic Republic	2017	Fracture of pelvis	0.1823
Lao People's Democratic Republic	1990	Fracture of radius and/or ulna	0.0435
Lao People's Democratic Republic	1995	Fracture of radius and/or ulna	0.0435
Lao People's Democratic Republic	2000	Fracture of radius and/or ulna	0.0435

Lao People's Democratic Republic	2005	Fracture of radius and/or ulna	0.0435
Lao People's Democratic Republic	2010	Fracture of radius and/or ulna	0.0435
Lao People's Democratic Republic	2017	Fracture of radius and/or ulna	0.0435
Lao People's Democratic Republic	1990	Fracture of skull	0.0714
Lao People's Democratic Republic	1995	Fracture of skull	0.0714
Lao People's Democratic Republic	2000	Fracture of skull	0.0714
Lao People's Democratic Republic	2005	Fracture of skull	0.0714
Lao People's Democratic Republic	2010	Fracture of skull	0.0714
Lao People's Democratic Republic	2017	Fracture of skull	0.0714
Lao People's Democratic Republic	1990	Fracture of sternum and/or fracture of one or more ribs	0.1027
Lao People's Democratic Republic	1995	Fracture of sternum and/or fracture of one or more ribs	0.1027
Lao People's Democratic Republic	2000	Fracture of sternum and/or fracture of one or more ribs	0.1027
Lao People's Democratic Republic	2005	Fracture of sternum and/or fracture of one or more ribs	0.1027
Lao People's Democratic Republic	2010	Fracture of sternum and/or fracture of one or more ribs	0.1027
Lao People's Democratic Republic	2017	Fracture of sternum and/or fracture of one or more ribs	0.1027
Lao People's Democratic Republic	1990	Fracture of vertebral column	0.1106
Lao People's Democratic Republic	1995	Fracture of vertebral column	0.1106
Lao People's Democratic Republic	2000	Fracture of vertebral column	0.1106
Lao People's Democratic Republic	2005	Fracture of vertebral column	0.1106
Lao People's Democratic Republic	2010	Fracture of vertebral column	0.1106
Lao People's Democratic Republic	2017	Fracture of vertebral column	0.1106
Lao People's Democratic Republic	1990	Fracture of femur, other than femoral neck	0.042
Lao People's Democratic Republic	1995	Fracture of femur, other than femoral neck	0.042
Lao People's Democratic Republic	2000	Fracture of femur, other than femoral neck	0.042
Lao People's Democratic Republic	2005	Fracture of femur, other than femoral neck	0.042
Lao People's Democratic Republic	2010	Fracture of femur, other than femoral neck	0.042
Lao People's Democratic Republic	2017	Fracture of femur, other than femoral neck	0.042
Lao People's Democratic Republic	1990	Minor TBI	0.1319
Lao People's Democratic Republic	1995	Minor TBI	0.1319
Lao People's Democratic Republic	2000	Minor TBI	0.1319
Lao People's Democratic Republic	2005	Minor TBI	0.1319
Lao People's Democratic Republic	2010	Minor TBI	0.1319
Lao People's Democratic Republic	2017	Minor TBI	0.1319
Lao People's Democratic Republic	1990	Moderate TBI	0.1637
Lao People's Democratic Republic	1995	Moderate TBI	0.1637
Lao People's Democratic Republic	2000	Moderate TBI	0.1637
Lao People's Democratic Republic	2005	Moderate TBI	0.1637
Lao People's Democratic Republic	2010	Moderate TBI	0.1637
Lao People's Democratic Republic	2017	Moderate TBI	0.1637
Lao People's Democratic Republic	1990	Amputation of fingers (excluding thumb)	0.0047
Lao People's Democratic Republic	1995	Amputation of fingers (excluding thumb)	0.0047
Lao People's Democratic Republic	2000	Amputation of fingers (excluding thumb)	0.0047
Lao People's Democratic Republic	2005	Amputation of fingers (excluding thumb)	0.0047
Lao People's Democratic Republic	2010	Amputation of fingers (excluding thumb)	0.0047
Lao People's Democratic Republic	2017	Amputation of fingers (excluding thumb)	0.0047
Lao People's Democratic Republic	1990	Spinal cord lesion at neck level	0.4997
Lao People's Democratic Republic	1995	Spinal cord lesion at neck level	0.4973
Lao People's Democratic Republic	2000	Spinal cord lesion at neck level	0.4938
Lao People's Democratic Republic	2005	Spinal cord lesion at neck level	0.4889
Lao People's Democratic Republic	2010	Spinal cord lesion at neck level	0.4809
Lao People's Democratic Republic	2017	Spinal cord lesion at neck level	0.4696
Lao People's Democratic Republic	1990	Spinal cord lesion below neck level	0.3632
Lao People's Democratic Republic	1995	Spinal cord lesion below neck level	0.358
Lao People's Democratic Republic	2000	Spinal cord lesion below neck level	0.3508
Lao People's Democratic Republic	2005	Spinal cord lesion below neck level	0.3404
Lao People's Democratic Republic	2010	Spinal cord lesion below neck level	0.3234
Lao People's Democratic Republic	2017	Spinal cord lesion below neck level	0.2995
Lao People's Democratic Republic	1990	Drowning and nonfatal submersion	0.2471
Lao People's Democratic Republic	1995	Drowning and nonfatal submersion	0.2471
Lao People's Democratic Republic	2000	Drowning and nonfatal submersion	0.2471
Lao People's Democratic Republic	2005	Drowning and nonfatal submersion	0.2471
Lao People's Democratic Republic	2010	Drowning and nonfatal submersion	0.2471
Lao People's Democratic Republic	2017	Drowning and nonfatal submersion	0.2471
Lao People's Democratic Republic	1990	Asphyxiation	0.2471
Lao People's Democratic Republic	1995	Asphyxiation	0.2471
Lao People's Democratic Republic	2000	Asphyxiation	0.2471
Lao People's Democratic Republic	2005	Asphyxiation	0.2471
Lao People's Democratic Republic	2010	Asphyxiation	0.2471
Lao People's Democratic Republic	2017	Asphyxiation	0.2471
Lao People's Democratic Republic	1990	Crush injury	0.1325
Lao People's Democratic Republic	1995	Crush injury	0.1325
Lao People's Democratic Republic	2000	Crush injury	0.1325
Lao People's Democratic Republic	2005	Crush injury	0.1325
Lao People's Democratic Republic	2010	Crush injury	0.1325
Lao People's Democratic Republic	2017	Crush injury	0.1325
Lao People's Democratic Republic	1990	Nerve injury	0.1129
Lao People's Democratic Republic	1995	Nerve injury	0.1129
Lao People's Democratic Republic	2000	Nerve injury	0.1129
Lao People's Democratic Republic	2005	Nerve injury	0.1129
Lao People's Democratic Republic	2010	Nerve injury	0.1129
Lao People's Democratic Republic	2017	Nerve injury	0.1129
Lao People's Democratic Republic	1990	Injury to eyes	0.0165
Lao People's Democratic Republic	1995	Injury to eyes	0.0165
Lao People's Democratic Republic	2000	Injury to eyes	0.0165
Lao People's Democratic Republic	2005	Injury to eyes	0.0165
Lao People's Democratic Republic	2010	Injury to eyes	0.0165
Lao People's Democratic Republic	2017	Injury to eyes	0.0165
Lao People's Democratic Republic	1990	Amputation of lower limb, unilateral	0.138
Lao People's Democratic Republic	1995	Amputation of lower limb, unilateral	0.1352
Lao People's Democratic Republic	2000	Amputation of lower limb, unilateral	0.1314
Lao People's Democratic Republic	2005	Amputation of lower limb, unilateral	0.1259
Lao People's Democratic Republic	2010	Amputation of lower limb, unilateral	0.1168
Lao People's Democratic Republic	2017	Amputation of lower limb, unilateral	0.104
Lao People's Democratic Republic	1990	Open wound(s)	0.011
Lao People's Democratic Republic	1995	Open wound(s)	0.011
Lao People's Democratic Republic	2000	Open wound(s)	0.011
Lao People's Democratic Republic	2005	Open wound(s)	0.011
Lao People's Democratic Republic	2010	Open wound(s)	0.011
Lao People's Democratic Republic	2017	Open wound(s)	0.011
Lao People's Democratic Republic	1990	Poisoning requiring urgent care	0.1628
Lao People's Democratic Republic	1995	Poisoning requiring urgent care	0.1628
Lao People's Democratic Republic	2000	Poisoning requiring urgent care	0.1628
Lao People's Democratic Republic	2005	Poisoning requiring urgent care	0.1628
Lao People's Democratic Republic	2010	Poisoning requiring urgent care	0.1628
Lao People's Democratic Republic	2017	Poisoning requiring urgent care	0.1628
Lao People's Democratic Republic	1990	Severe chest injury	0.0474
Lao People's Democratic Republic	1995	Severe chest injury	0.0474
Lao People's Democratic Republic	2000	Severe chest injury	0.0474
Lao People's Democratic Republic	2005	Severe chest injury	0.0474
Lao People's Democratic Republic	2010	Severe chest injury	0.0474
Lao People's Democratic Republic	2017	Severe chest injury	0.0474
Lao People's Democratic Republic	1990	Internal hemorrhage in abdomen and pelvis	0.1143
Lao People's Democratic Republic	1995	Internal hemorrhage in abdomen and pelvis	0.1143
Lao People's Democratic Republic	2000	Internal hemorrhage in abdomen and pelvis	0.1143
Lao People's Democratic Republic	2005	Internal hemorrhage in abdomen and pelvis	0.1143
Lao People's Democratic Republic	2010	Internal hemorrhage in abdomen and pelvis	0.1143
Lao People's Democratic Republic	2017	Internal hemorrhage in abdomen and pelvis	0.1143
Lao People's Democratic Republic	1990	Contusion in any part of the body	0.0075
Lao People's Democratic Republic	1995	Contusion in any part of the body	0.0075
Lao People's Democratic Republic	2000	Contusion in any part of the body	0.0075
Lao People's Democratic Republic	2005	Contusion in any part of the body	0.0075
Lao People's Democratic Republic	2010	Contusion in any part of the body	0.0075
Lao People's Democratic Republic	2017	Contusion in any part of the body	0.0075
Lao People's Democratic Republic	1990	Effect of different environmental factors	0.049
Lao People's Democratic Republic	1995	Effect of different environmental factors	0.049
Lao People's Democratic Republic	2000	Effect of different environmental factors	0.049
Lao People's Democratic Republic	2005	Effect of different environmental factors	0.049
Lao People's Democratic Republic	2010	Effect of different environmental factors	0.049
Lao People's Democratic Republic	2017	Effect of different environmental factors	0.049
Lao People's Democratic Republic	1990	Complications following therapeutic procedures	0.0506
Lao People's Democratic Republic	1995	Complications following therapeutic procedures	0.0506
Lao People's Democratic Republic	2000	Complications following therapeutic procedures	0.0506
Lao People's Democratic Republic	2005	Complications following therapeutic procedures	0.0506
Lao People's Democratic Republic	2010	Complications following therapeutic procedures	0.0506
Lao People's Democratic Republic	2017	Complications following therapeutic procedures	0.0506
Lao People's Democratic Republic	1990	Multiple fractures, dislocations, crashes, wounds, sprains, and strains	0.3119
Lao People's Democratic Republic	1995	Multiple fractures, dislocations, crashes, wounds, sprains, and strains	0.3047
Lao People's Democratic Republic	2000	Multiple fractures, dislocations, crashes, wounds, sprains, and strains	0.2949
Lao People's Democratic Republic	2005	Multiple fractures, dislocations, crashes, wounds, sprains, and strains	0.2807
Lao People's Democratic Republic	2010	Multiple fractures, dislocations, crashes, wounds, sprains, and strains	0.2573
Lao People's Democratic Republic	2017	Multiple fractures, dislocations, crashes, wounds, sprains, and strains	0.2246
Lao People's Democratic Republic	1990	Amputation of upper limb, unilateral	0.0973
Lao People's Democratic Republic	1995	Amputation of upper limb, unilateral	0.0957
Lao People's Democratic Republic	2000	Amputation of upper limb, unilateral	0.0934
Lao People's Democratic Republic	2005	Amputation of upper limb, unilateral	0.0901
Lao People's Democratic Republic	2010	Amputation of upper limb, unilateral	0.0848
Lao People's Democratic Republic	2017	Amputation of upper limb, unilateral	0.0773
Lao People's Democratic Republic	1990	Amputation of thumb	0.0114
Lao People's Democratic Republic	1995	Amputation of thumb	0.0114
Lao People's Democratic Republic	2000	Amputation of thumb	0.0114
Lao People's Democratic Republic	2005	Amputation of thumb	0.0114
Lao People's Democratic Republic	2010	Amputation of thumb	0.0114
Lao People's Democratic Republic	2017	Amputation of thumb	0.0114
Lao People's Democratic Republic	1990	Amputation of toe/toes	0.0059
Lao People's Democratic Republic	1995	Amputation of toe/toes	0.0059
Lao People's Democratic Republic	2000	Amputation of toe/toes	0.0059
Lao People's Democratic Republic	2005	Amputation of toe/toes	0.0059
Lao People's Democratic Republic	2010	Amputation of toe/toes	0.0059
Lao People's Democratic Republic	2017	Amputation of toe/toes	0.0059
Lao People's Democratic Republic	1990	Burns, <20% total burned surface area without lower airway burns	0.0163
Lao People's Democratic Republic	1995	Burns, <20% total burned surface area without lower airway burns	0.0163
Lao People's Democratic Republic	2000	Burns, <20% total burned surface area without lower airway burns	0.0163
Lao People's Democratic Republic	2005	Burns, <20% total burned surface area without lower airway burns	0.0163
Lao People's Democratic Republic	2010	Burns, <20% total burned surface area without lower airway burns	0.0163
Lao People's Democratic Republic	2017	Burns, <20% total burned surface area without lower airway burns	0.0163
Lao People's Democratic Republic	1990	Burns, >=20% total burned surface area or >= 10% burned surface area if head/neck or hands/wrist involved w/o lower airway burns	0.3713
Lao People's Democratic Republic	1995	Burns, >=20% total burned surface area or >= 10% burned surface area if head/neck or hands/wrist involved w/o lower airway burns	0.3646
Lao People's Democratic Republic	2000	Burns, >=20% total burned surface area or >= 10% burned surface area if head/neck or hands/wrist involved w/o lower airway burns	0.3555
Lao People's Democratic Republic	2005	Burns, >=20% total burned surface area or >= 10% burned surface area if head/neck or hands/wrist involved w/o lower airway burns	0.3422
Lao People's Democratic Republic	2010	Burns, >=20% total burned surface area or >= 10% burned surface area if head/neck or hands/wrist involved w/o lower airway burns	0.3205
Lao People's Democratic Republic	2017	Burns, >=20% total burned surface area or >= 10% burned surface area if head/neck or hands/wrist involved w/o lower airway burns	0.29
Malaysia	1990	Amputation of lower limbs, bilateral	0.227
Malaysia	1995	Amputation of lower limbs, bilateral	0.2103
Malaysia	2000	Amputation of lower limbs, bilateral	0.1926
Malaysia	2005	Amputation of lower limbs, bilateral	0.1765
Malaysia	2010	Amputation of lower limbs, bilateral	0.1673
Malaysia	2017	Amputation of lower limbs, bilateral	0.1445
Malaysia	1990	Lower airway burns	0.2252
Malaysia	1995	Lower airway burns	0.2252
Malaysia	2000	Lower airway burns	0.2252
Malaysia	2005	Lower airway burns	0.2252
Malaysia	2010	Lower airway burns	0.2252
Malaysia	2017	Lower airway burns	0.2252
Malaysia	1990	Dislocation of hip	0.0159
Malaysia	1995	Dislocation of hip	0.0159
Malaysia	2000	Dislocation of hip	0.0159
Malaysia	2005	Dislocation of hip	0.0159
Malaysia	2010	Dislocation of hip	0.0159
Malaysia	2017	Dislocation of hip	0.0159
Malaysia	1990	Dislocation of knee	0.1134
Malaysia	1995	Dislocation of knee	0.1134
Malaysia	2000	Dislocation of knee	0.1134
Malaysia	2005	Dislocation of knee	0.1134
Malaysia	2010	Dislocation of knee	0.1134
Malaysia	2017	Dislocation of knee	0.1134
Malaysia	1990	Dislocation of shoulder	0.062
Malaysia	1995	Dislocation of shoulder	0.062
Malaysia	2000	Dislocation of shoulder	0.062
Malaysia	2005	Dislocation of shoulder	0.062
Malaysia	2010	Dislocation of shoulder	0.062
Malaysia	2017	Dislocation of shoulder	0.062
Malaysia	1990	Muscle and tendon injuries, including sprains and strains lesser dislocations	0.0075
Malaysia	1995	Muscle and tendon injuries, including sprains and strains lesser dislocations	0.0075
Malaysia	2000	Muscle and tendon injuries, including sprains and strains lesser dislocations	0.0075
Malaysia	2005	Muscle and tendon injuries, including sprains and strains lesser dislocations	0.0075
Malaysia	2010	Muscle and tendon injuries, including sprains and strains lesser dislocations	0.0075
Malaysia	2017	Muscle and tendon injuries, including sprains and strains lesser dislocations	0.0075
Malaysia	1990	Fracture of clavicle, scapula, or humerus	0.0349
Malaysia	1995	Fracture of clavicle, scapula, or humerus	0.0349
Malaysia	2000	Fracture of clavicle, scapula, or humerus	0.0349
Malaysia	2005	Fracture of clavicle, scapula, or humerus	0.0349
Malaysia	2010	Fracture of clavicle, scapula, or humerus	0.0349
Malaysia	2017	Fracture of clavicle, scapula, or humerus	0.0349
Malaysia	1990	Fracture of face bones	0.0669
Malaysia	1995	Fracture of face bones	0.0669
Malaysia	2000	Fracture of face bones	0.0669
Malaysia	2005	Fracture of face bones	0.0669
Malaysia	2010	Fracture of face bones	0.0669
Malaysia	2017	Fracture of face bones	0.0669
Malaysia	1990	Fracture of foot bones except ankle	0.0264
Malaysia	1995	Fracture of foot bones except ankle	0.0264
Malaysia	2000	Fracture of foot bones except ankle	0.0264
Malaysia	2005	Fracture of foot bones except ankle	0.0264
Malaysia	2010	Fracture of foot bones except ankle	0.0264
Malaysia	2017	Fracture of foot bones except ankle	0.0264
Malaysia	1990	Fracture of hand (wrist and other distal part of hand)	0.0138
Malaysia	1995	Fracture of hand (wrist and other distal part of hand)	0.0138
Malaysia	2000	Fracture of hand (wrist and other distal part of hand)	0.0138
Malaysia	2005	Fracture of hand (wrist and other distal part of hand)	0.0138
Malaysia	2010	Fracture of hand (wrist and other distal part of hand)	0.0138
Malaysia	2017	Fracture of hand (wrist and other distal part of hand)	0.0138
Malaysia	1990	Fracture of hip	0.1948
Malaysia	1995	Fracture of hip	0.1729
Malaysia	2000	Fracture of hip	0.1495
Malaysia	2005	Fracture of hip	0.1281
Malaysia	2010	Fracture of hip	0.116
Malaysia	2017	Fracture of hip	0.0858
Malaysia	1990	Amputation of upper limbs, bilateral	0.2293
Malaysia	1995	Amputation of upper limbs, bilateral	0.2066
Malaysia	2000	Amputation of upper limbs, bilateral	0.1824
Malaysia	2005	Amputation of upper limbs, bilateral	0.1604
Malaysia	2010	Amputation of upper limbs, bilateral	0.1479
Malaysia	2017	Amputation of upper limbs, bilateral	0.1167
Malaysia	1990	Fracture of patella, tibia or fibula, or ankle	0.0554
Malaysia	1995	Fracture of patella, tibia or fibula, or ankle	0.0554
Malaysia	2000	Fracture of patella, tibia or fibula, or ankle	0.0554
Malaysia	2005	Fracture of patella, tibia or fibula, or ankle	0.0554
Malaysia	2010	Fracture of patella, tibia or fibula, or ankle	0.0554
Malaysia	2017	Fracture of patella, tibia or fibula, or ankle	0.0554
Malaysia	1990	Fracture of pelvis	0.1823
Malaysia	1995	Fracture of pelvis	0.1823
Malaysia	2000	Fracture of pelvis	0.1823
Malaysia	2005	Fracture of pelvis	0.1823
Malaysia	2010	Fracture of pelvis	0.1823
Malaysia	2017	Fracture of pelvis	0.1823
Malaysia	1990	Fracture of radius and/or ulna	0.0435
Malaysia	1995	Fracture of radius and/or ulna	0.0435
Malaysia	2000	Fracture of radius and/or ulna	0.0435
Malaysia	2005	Fracture of radius and/or ulna	0.0435
Malaysia	2010	Fracture of radius and/or ulna	0.0435
Malaysia	2017	Fracture of radius and/or ulna	0.0435
Malaysia	1990	Fracture of skull	0.0714
Malaysia	1995	Fracture of skull	0.0714
Malaysia	2000	Fracture of skull	0.0714
Malaysia	2005	Fracture of skull	0.0714
Malaysia	2010	Fracture of skull	0.0714
Malaysia	2017	Fracture of skull	0.0714
Malaysia	1990	Fracture of sternum and/or fracture of one or more ribs	0.1027
Malaysia	1995	Fracture of sternum and/or fracture of one or more ribs	0.1027
Malaysia	2000	Fracture of sternum and/or fracture of one or more ribs	0.1027
Malaysia	2005	Fracture of sternum and/or fracture of one or more ribs	0.1027
Malaysia	2010	Fracture of sternum and/or fracture of one or more ribs	0.1027
Malaysia	2017	Fracture of sternum and/or fracture of one or more ribs	0.1027
Malaysia	1990	Fracture of vertebral column	0.1106
Malaysia	1995	Fracture of vertebral column	0.1106
Malaysia	2000	Fracture of vertebral column	0.1106
Malaysia	2005	Fracture of vertebral column	0.1106
Malaysia	2010	Fracture of vertebral column	0.1106
Malaysia	2017	Fracture of vertebral column	0.1106
Malaysia	1990	Fracture of femur, other than femoral neck	0.042
Malaysia	1995	Fracture of femur, other than femoral neck	0.042
Malaysia	2000	Fracture of femur, other than femoral neck	0.042
Malaysia	2005	Fracture of femur, other than femoral neck	0.042
Malaysia	2010	Fracture of femur, other than femoral neck	0.042
Malaysia	2017	Fracture of femur, other than femoral neck	0.042
Malaysia	1990	Minor TBI	0.1319
Malaysia	1995	Minor TBI	0.1319
Malaysia	2000	Minor TBI	0.1319
Malaysia	2005	Minor TBI	0.1319
Malaysia	2010	Minor TBI	0.1319
Malaysia	2017	Minor TBI	0.1319
Malaysia	1990	Moderate TBI	0.1637
Malaysia	1995	Moderate TBI	0.1637
Malaysia	2000	Moderate TBI	0.1637
Malaysia	2005	Moderate TBI	0.1637
Malaysia	2010	Moderate TBI	0.1637
Malaysia	2017	Moderate TBI	0.1637
Malaysia	1990	Amputation of fingers (excluding thumb)	0.0047
Malaysia	1995	Amputation of fingers (excluding thumb)	0.0047
Malaysia	2000	Amputation of fingers (excluding thumb)	0.0047
Malaysia	2005	Amputation of fingers (excluding thumb)	0.0047
Malaysia	2010	Amputation of fingers (excluding thumb)	0.0047
Malaysia	2017	Amputation of fingers (excluding thumb)	0.0047
Malaysia	1990	Spinal cord lesion at neck level	0.4593
Malaysia	1995	Spinal cord lesion at neck level	0.4516
Malaysia	2000	Spinal cord lesion at neck level	0.4436
Malaysia	2005	Spinal cord lesion at neck level	0.4362
Malaysia	2010	Spinal cord lesion at neck level	0.432
Malaysia	2017	Spinal cord lesion at neck level	0.4216
Malaysia	1990	Spinal cord lesion below neck level	0.2778
Malaysia	1995	Spinal cord lesion below neck level	0.2617
Malaysia	2000	Spinal cord lesion below neck level	0.2446
Malaysia	2005	Spinal cord lesion below neck level	0.2291
Malaysia	2010	Spinal cord lesion below neck level	0.2202
Malaysia	2017	Spinal cord lesion below neck level	0.1982
Malaysia	1990	Drowning and nonfatal submersion	0.2471
Malaysia	1995	Drowning and nonfatal submersion	0.2471
Malaysia	2000	Drowning and nonfatal submersion	0.2471
Malaysia	2005	Drowning and nonfatal submersion	0.2471
Malaysia	2010	Drowning and nonfatal submersion	0.2471
Malaysia	2017	Drowning and nonfatal submersion	0.2471
Malaysia	1990	Asphyxiation	0.2471
Malaysia	1995	Asphyxiation	0.2471
Malaysia	2000	Asphyxiation	0.2471
Malaysia	2005	Asphyxiation	0.2471
Malaysia	2010	Asphyxiation	0.2471
Malaysia	2017	Asphyxiation	0.2471
Malaysia	1990	Crush injury	0.1325
Malaysia	1995	Crush injury	0.1325
Malaysia	2000	Crush injury	0.1325
Malaysia	2005	Crush injury	0.1325
Malaysia	2010	Crush injury	0.1325
Malaysia	2017	Crush injury	0.1325
Malaysia	1990	Nerve injury	0.1129
Malaysia	1995	Nerve injury	0.1129
Malaysia	2000	Nerve injury	0.1129
Malaysia	2005	Nerve injury	0.1129
Malaysia	2010	Nerve injury	0.1129
Malaysia	2017	Nerve injury	0.1129
Malaysia	1990	Injury to eyes	0.0165
Malaysia	1995	Injury to eyes	0.0165
Malaysia	2000	Injury to eyes	0.0165
Malaysia	2005	Injury to eyes	0.0165
Malaysia	2010	Injury to eyes	0.0165
Malaysia	2017	Injury to eyes	0.0165
Malaysia	1990	Amputation of lower limb, unilateral	0.0924
Malaysia	1995	Amputation of lower limb, unilateral	0.0838
Malaysia	2000	Amputation of lower limb, unilateral	0.0747
Malaysia	2005	Amputation of lower limb, unilateral	0.0664
Malaysia	2010	Amputation of lower limb, unilateral	0.0617
Malaysia	2017	Amputation of lower limb, unilateral	0.0499
Malaysia	1990	Open wound(s)	0.011
Malaysia	1995	Open wound(s)	0.011
Malaysia	2000	Open wound(s)	0.011
Malaysia	2005	Open wound(s)	0.011
Malaysia	2010	Open wound(s)	0.011
Malaysia	2017	Open wound(s)	0.011
Malaysia	1990	Poisoning requiring urgent care	0.1628
Malaysia	1995	Poisoning requiring urgent care	0.1628
Malaysia	2000	Poisoning requiring urgent care	0.1628
Malaysia	2005	Poisoning requiring urgent care	0.1628
Malaysia	2010	Poisoning requiring urgent care	0.1628
Malaysia	2017	Poisoning requiring urgent care	0.1628
Malaysia	1990	Severe chest injury	0.0474
Malaysia	1995	Severe chest injury	0.0474
Malaysia	2000	Severe chest injury	0.0474
Malaysia	2005	Severe chest injury	0.0474
Malaysia	2010	Severe chest injury	0.0474
Malaysia	2017	Severe chest injury	0.0474
Malaysia	1990	Internal hemorrhage in abdomen and pelvis	0.1143
Malaysia	1995	Internal hemorrhage in abdomen and pelvis	0.1143
Malaysia	2000	Internal hemorrhage in abdomen and pelvis	0.1143
Malaysia	2005	Internal hemorrhage in abdomen and pelvis	0.1143
Malaysia	2010	Internal hemorrhage in abdomen and pelvis	0.1143
Malaysia	2017	Internal hemorrhage in abdomen and pelvis	0.1143
Malaysia	1990	Contusion in any part of the body	0.0075
Malaysia	1995	Contusion in any part of the body	0.0075
Malaysia	2000	Contusion in any part of the body	0.0075
Malaysia	2005	Contusion in any part of the body	0.0075
Malaysia	2010	Contusion in any part of the body	0.0075
Malaysia	2017	Contusion in any part of the body	0.0075
Malaysia	1990	Effect of different environmental factors	0.049
Malaysia	1995	Effect of different environmental factors	0.049
Malaysia	2000	Effect of different environmental factors	0.049
Malaysia	2005	Effect of different environmental factors	0.049
Malaysia	2010	Effect of different environmental factors	0.049
Malaysia	2017	Effect of different environmental factors	0.049
Malaysia	1990	Complications following therapeutic procedures	0.0506
Malaysia	1995	Complications following therapeutic procedures	0.0506
Malaysia	2000	Complications following therapeutic procedures	0.0506
Malaysia	2005	Complications following therapeutic procedures	0.0506
Malaysia	2010	Complications following therapeutic procedures	0.0506
Malaysia	2017	Complications following therapeutic procedures	0.0506
Malaysia	1990	Multiple fractures, dislocations, crashes, wounds, sprains, and strains	0.1948
Malaysia	1995	Multiple fractures, dislocations, crashes, wounds, sprains, and strains	0.1729
Malaysia	2000	Multiple fractures, dislocations, crashes, wounds, sprains, and strains	0.1495
Malaysia	2005	Multiple fractures, dislocations, crashes, wounds, sprains, and strains	0.1281
Malaysia	2010	Multiple fractures, dislocations, crashes, wounds, sprains, and strains	0.116
Malaysia	2017	Multiple fractures, dislocations, crashes, wounds, sprains, and strains	0.0858
Malaysia	1990	Amputation of upper limb, unilateral	0.0705
Malaysia	1995	Amputation of upper limb, unilateral	0.0654
Malaysia	2000	Amputation of upper limb, unilateral	0.06
Malaysia	2005	Amputation of upper limb, unilateral	0.0551
Malaysia	2010	Amputation of upper limb, unilateral	0.0524
Malaysia	2017	Amputation of upper limb, unilateral	0.0454
Malaysia	1990	Amputation of thumb	0.0114
Malaysia	1995	Amputation of thumb	0.0114
Malaysia	2000	Amputation of thumb	0.0114
Malaysia	2005	Amputation of thumb	0.0114
Malaysia	2010	Amputation of thumb	0.0114
Malaysia	2017	Amputation of thumb	0.0114
Malaysia	1990	Amputation of toe/toes	0.0059
Malaysia	1995	Amputation of toe/toes	0.0059
Malaysia	2000	Amputation of toe/toes	0.0059
Malaysia	2005	Amputation of toe/toes	0.0059
Malaysia	2010	Amputation of toe/toes	0.0059
Malaysia	2017	Amputation of toe/toes	0.0059
Malaysia	1990	Burns, <20% total burned surface area without lower airway burns	0.0163
Malaysia	1995	Burns, <20% total burned surface area without lower airway burns	0.0163
Malaysia	2000	Burns, <20% total burned surface area without lower airway burns	0.0163
Malaysia	2005	Burns, <20% total burned surface area without lower airway burns	0.0163
Malaysia	2010	Burns, <20% total burned surface area without lower airway burns	0.0163
Malaysia	2017	Burns, <20% total burned surface area without lower airway burns	0.0163
Malaysia	1990	Burns, >=20% total burned surface area or >= 10% burned surface area if head/neck or hands/wrist involved w/o lower airway burns	0.2623
Malaysia	1995	Burns, >=20% total burned surface area or >= 10% burned surface area if head/neck or hands/wrist involved w/o lower airway burns	0.2418
Malaysia	2000	Burns, >=20% total burned surface area or >= 10% burned surface area if head/neck or hands/wrist involved w/o lower airway burns	0.22
Malaysia	2005	Burns, >=20% total burned surface area or >= 10% burned surface area if head/neck or hands/wrist involved w/o lower airway burns	0.2002
Malaysia	2010	Burns, >=20% total burned surface area or >= 10% burned surface area if head/neck or hands/wrist involved w/o lower airway burns	0.1889
Malaysia	2017	Burns, >=20% total burned surface area or >= 10% burned surface area if head/neck or hands/wrist involved w/o lower airway burns	0.1608
Maldives	1990	Amputation of lower limbs, bilateral	0.2495
Maldives	1995	Amputation of lower limbs, bilateral	0.2266

Maldives	2000	Amputation of lower limbs, bilateral	0.1993
Maldives	2005	Amputation of lower limbs, bilateral	0.1801
Maldives	2010	Amputation of lower limbs, bilateral	0.1563
Maldives	2017	Amputation of lower limbs, bilateral	0.1362
Maldives	1990	Lower airway burns	0.2252
Maldives	1995	Lower airway burns	0.2252
Maldives	2000	Lower airway burns	0.2252
Maldives	2005	Lower airway burns	0.2252
Maldives	2010	Lower airway burns	0.2252
Maldives	2017	Lower airway burns	0.2252
Maldives	1990	Dislocation of hip	0.0159
Maldives	1995	Dislocation of hip	0.0159
Maldives	2000	Dislocation of hip	0.0159
Maldives	2005	Dislocation of hip	0.0159
Maldives	2010	Dislocation of hip	0.0159
Maldives	2017	Dislocation of hip	0.0159
Maldives	1990	Dislocation of knee	0.1134
Maldives	1995	Dislocation of knee	0.1134
Maldives	2000	Dislocation of knee	0.1134
Maldives	2005	Dislocation of knee	0.1134
Maldives	2010	Dislocation of knee	0.1134
Maldives	2017	Dislocation of knee	0.1134
Maldives	1990	Dislocation of shoulder	0.062
Maldives	1995	Dislocation of shoulder	0.062
Maldives	2000	Dislocation of shoulder	0.062
Maldives	2005	Dislocation of shoulder	0.062
Maldives	2010	Dislocation of shoulder	0.062
Maldives	2017	Dislocation of shoulder	0.062
Maldives	1990	Muscle and tendon injuries, including sprains and strains lesser dislocations	0.0075
Maldives	1995	Muscle and tendon injuries, including sprains and strains lesser dislocations	0.0075
Maldives	2000	Muscle and tendon injuries, including sprains and strains lesser dislocations	0.0075
Maldives	2005	Muscle and tendon injuries, including sprains and strains lesser dislocations	0.0075
Maldives	2010	Muscle and tendon injuries, including sprains and strains lesser dislocations	0.0075
Maldives	2017	Muscle and tendon injuries, including sprains and strains lesser dislocations	0.0075
Maldives	1990	Fracture of clavicle, scapula, or humerus	0.0349
Maldives	1995	Fracture of clavicle, scapula, or humerus	0.0349
Maldives	2000	Fracture of clavicle, scapula, or humerus	0.0349
Maldives	2005	Fracture of clavicle, scapula, or humerus	0.0349
Maldives	2010	Fracture of clavicle, scapula, or humerus	0.0349
Maldives	2017	Fracture of clavicle, scapula, or humerus	0.0349
Maldives	1990	Fracture of face bones	0.0669
Maldives	1995	Fracture of face bones	0.0669
Maldives	2000	Fracture of face bones	0.0669
Maldives	2005	Fracture of face bones	0.0669
Maldives	2010	Fracture of face bones	0.0669
Maldives	2017	Fracture of face bones	0.0669
Maldives	1990	Fracture of foot bones except ankle	0.0264
Maldives	1995	Fracture of foot bones except ankle	0.0264
Maldives	2000	Fracture of foot bones except ankle	0.0264
Maldives	2005	Fracture of foot bones except ankle	0.0264
Maldives	2010	Fracture of foot bones except ankle	0.0264
Maldives	2017	Fracture of foot bones except ankle	0.0264
Maldives	1990	Fracture of hand (wrist and other distal part of hand)	0.0138
Maldives	1995	Fracture of hand (wrist and other distal part of hand)	0.0138
Maldives	2000	Fracture of hand (wrist and other distal part of hand)	0.0138
Maldives	2005	Fracture of hand (wrist and other distal part of hand)	0.0138
Maldives	2010	Fracture of hand (wrist and other distal part of hand)	0.0138
Maldives	2017	Fracture of hand (wrist and other distal part of hand)	0.0138
Maldives	1990	Fracture of hip	0.2247
Maldives	1995	Fracture of hip	0.1944
Maldives	2000	Fracture of hip	0.1583
Maldives	2005	Fracture of hip	0.1328
Maldives	2010	Fracture of hip	0.1013
Maldives	2017	Fracture of hip	0.0747
Maldives	1990	Amputation of upper limbs, bilateral	0.2601
Maldives	1995	Amputation of upper limbs, bilateral	0.2288
Maldives	2000	Amputation of upper limbs, bilateral	0.1915
Maldives	2005	Amputation of upper limbs, bilateral	0.1652
Maldives	2010	Amputation of upper limbs, bilateral	0.1327
Maldives	2017	Amputation of upper limbs, bilateral	0.1053
Maldives	1990	Fracture of patella, tibia or fibula, or ankle	0.0554
Maldives	1995	Fracture of patella, tibia or fibula, or ankle	0.0554
Maldives	2000	Fracture of patella, tibia or fibula, or ankle	0.0554
Maldives	2005	Fracture of patella, tibia or fibula, or ankle	0.0554
Maldives	2010	Fracture of patella, tibia or fibula, or ankle	0.0554
Maldives	2017	Fracture of patella, tibia or fibula, or ankle	0.0554
Maldives	1990	Fracture of pelvis	0.1823
Maldives	1995	Fracture of pelvis	0.1823
Maldives	2000	Fracture of pelvis	0.1823
Maldives	2005	Fracture of pelvis	0.1823
Maldives	2010	Fracture of pelvis	0.1823
Maldives	2017	Fracture of pelvis	0.1823
Maldives	1990	Fracture of radius and/or ulna	0.0435
Maldives	1995	Fracture of radius and/or ulna	0.0435
Maldives	2000	Fracture of radius and/or ulna	0.0435
Maldives	2005	Fracture of radius and/or ulna	0.0435
Maldives	2010	Fracture of radius and/or ulna	0.0435
Maldives	2017	Fracture of radius and/or ulna	0.0435
Maldives	1990	Fracture of skull	0.0714
Maldives	1995	Fracture of skull	0.0714
Maldives	2000	Fracture of skull	0.0714
Maldives	2005	Fracture of skull	0.0714
Maldives	2010	Fracture of skull	0.0714
Maldives	2017	Fracture of skull	0.0714
Maldives	1990	Fracture of sternum and/or fracture of one or more ribs	0.1027
Maldives	1995	Fracture of sternum and/or fracture of one or more ribs	0.1027
Maldives	2000	Fracture of sternum and/or fracture of one or more ribs	0.1027
Maldives	2005	Fracture of sternum and/or fracture of one or more ribs	0.1027
Maldives	2010	Fracture of sternum and/or fracture of one or more ribs	0.1027
Maldives	2017	Fracture of sternum and/or fracture of one or more ribs	0.1027
Maldives	1990	Fracture of vertebral column	0.1106
Maldives	1995	Fracture of vertebral column	0.1106
Maldives	2000	Fracture of vertebral column	0.1106
Maldives	2005	Fracture of vertebral column	0.1106
Maldives	2010	Fracture of vertebral column	0.1106
Maldives	2017	Fracture of vertebral column	0.1106
Maldives	1990	Fracture of femur, other than femoral neck	0.042
Maldives	1995	Fracture of femur, other than femoral neck	0.042
Maldives	2000	Fracture of femur, other than femoral neck	0.042
Maldives	2005	Fracture of femur, other than femoral neck	0.042
Maldives	2010	Fracture of femur, other than femoral neck	0.042
Maldives	2017	Fracture of femur, other than femoral neck	0.042
Maldives	1990	Minor TBI	0.1319
Maldives	1995	Minor TBI	0.1319
Maldives	2000	Minor TBI	0.1319
Maldives	2005	Minor TBI	0.1319
Maldives	2010	Minor TBI	0.1319
Maldives	2017	Minor TBI	0.1319
Maldives	1990	Moderate TBI	0.1637
Maldives	1995	Moderate TBI	0.1637
Maldives	2000	Moderate TBI	0.1637
Maldives	2005	Moderate TBI	0.1637
Maldives	2010	Moderate TBI	0.1637
Maldives	2017	Moderate TBI	0.1637
Maldives	1990	Amputation of fingers (excluding thumb)	0.0047
Maldives	1995	Amputation of fingers (excluding thumb)	0.0047
Maldives	2000	Amputation of fingers (excluding thumb)	0.0047
Maldives	2005	Amputation of fingers (excluding thumb)	0.0047
Maldives	2010	Amputation of fingers (excluding thumb)	0.0047
Maldives	2017	Amputation of fingers (excluding thumb)	0.0047
Maldives	1990	Spinal cord lesion at neck level	0.4696
Maldives	1995	Spinal cord lesion at neck level	0.4591
Maldives	2000	Spinal cord lesion at neck level	0.4466
Maldives	2005	Spinal cord lesion at neck level	0.4378
Maldives	2010	Spinal cord lesion at neck level	0.4269
Maldives	2017	Spinal cord lesion at neck level	0.4177
Maldives	1990	Spinal cord lesion below neck level	0.2996
Maldives	1995	Spinal cord lesion below neck level	0.2775
Maldives	2000	Spinal cord lesion below neck level	0.2511
Maldives	2005	Spinal cord lesion below neck level	0.2325
Maldives	2010	Spinal cord lesion below neck level	0.2095
Maldives	2017	Spinal cord lesion below neck level	0.1901
Maldives	1990	Drowning and nonfatal submersion	0.2471
Maldives	1995	Drowning and nonfatal submersion	0.2471
Maldives	2000	Drowning and nonfatal submersion	0.2471
Maldives	2005	Drowning and nonfatal submersion	0.2471
Maldives	2010	Drowning and nonfatal submersion	0.2471
Maldives	2017	Drowning and nonfatal submersion	0.2471
Maldives	1990	Asphyxiation	0.2471
Maldives	1995	Asphyxiation	0.2471
Maldives	2000	Asphyxiation	0.2471
Maldives	2005	Asphyxiation	0.2471
Maldives	2010	Asphyxiation	0.2471
Maldives	2017	Asphyxiation	0.2471
Maldives	1990	Crush injury	0.1325
Maldives	1995	Crush injury	0.1325
Maldives	2000	Crush injury	0.1325
Maldives	2005	Crush injury	0.1325
Maldives	2010	Crush injury	0.1325
Maldives	2017	Crush injury	0.1325
Maldives	1990	Nerve injury	0.1129
Maldives	1995	Nerve injury	0.1129
Maldives	2000	Nerve injury	0.1129
Maldives	2005	Nerve injury	0.1129
Maldives	2010	Nerve injury	0.1129
Maldives	2017	Nerve injury	0.1129
Maldives	1990	Injury to eyes	0.0165
Maldives	1995	Injury to eyes	0.0165
Maldives	2000	Injury to eyes	0.0165
Maldives	2005	Injury to eyes	0.0165
Maldives	2010	Injury to eyes	0.0165
Maldives	2017	Injury to eyes	0.0165
Maldives	1990	Amputation of lower limb, unilateral	0.1041
Maldives	1995	Amputation of lower limb, unilateral	0.0923
Maldives	2000	Amputation of lower limb, unilateral	0.0782
Maldives	2005	Amputation of lower limb, unilateral	0.0682
Maldives	2010	Amputation of lower limb, unilateral	0.056
Maldives	2017	Amputation of lower limb, unilateral	0.0456
Maldives	1990	Open wound(s)	0.011
Maldives	1995	Open wound(s)	0.011
Maldives	2000	Open wound(s)	0.011
Maldives	2005	Open wound(s)	0.011
Maldives	2010	Open wound(s)	0.011
Maldives	2017	Open wound(s)	0.011
Maldives	1990	Poisoning requiring urgent care	0.1628
Maldives	1995	Poisoning requiring urgent care	0.1628
Maldives	2000	Poisoning requiring urgent care	0.1628
Maldives	2005	Poisoning requiring urgent care	0.1628
Maldives	2010	Poisoning requiring urgent care	0.1628
Maldives	2017	Poisoning requiring urgent care	0.1628
Maldives	1990	Severe chest injury	0.0474
Maldives	1995	Severe chest injury	0.0474
Maldives	2000	Severe chest injury	0.0474
Maldives	2005	Severe chest injury	0.0474
Maldives	2010	Severe chest injury	0.0474
Maldives	2017	Severe chest injury	0.0474
Maldives	1990	Internal hemorrhage in abdomen and pelvis	0.1143
Maldives	1995	Internal hemorrhage in abdomen and pelvis	0.1143
Maldives	2000	Internal hemorrhage in abdomen and pelvis	0.1143
Maldives	2005	Internal hemorrhage in abdomen and pelvis	0.1143
Maldives	2010	Internal hemorrhage in abdomen and pelvis	0.1143
Maldives	2017	Internal hemorrhage in abdomen and pelvis	0.1143
Maldives	1990	Contusion in any part of the body	0.0075
Maldives	1995	Contusion in any part of the body	0.0075
Maldives	2000	Contusion in any part of the body	0.0075
Maldives	2005	Contusion in any part of the body	0.0075
Maldives	2010	Contusion in any part of the body	0.0075
Maldives	2017	Contusion in any part of the body	0.0075
Maldives	1990	Effect of different environmental factors	0.049
Maldives	1995	Effect of different environmental factors	0.049
Maldives	2000	Effect of different environmental factors	0.049
Maldives	2005	Effect of different environmental factors	0.049
Maldives	2010	Effect of different environmental factors	0.049
Maldives	2017	Effect of different environmental factors	0.049
Maldives	1990	Complications following therapeutic procedures	0.0506
Maldives	1995	Complications following therapeutic procedures	0.0506
Maldives	2000	Complications following therapeutic procedures	0.0506
Maldives	2005	Complications following therapeutic procedures	0.0506
Maldives	2010	Complications following therapeutic procedures	0.0506
Maldives	2017	Complications following therapeutic procedures	0.0506
Maldives	1990	Multiple fractures, dislocations, crashes, wounds, sprains, and strains	0.2247
Maldives	1995	Multiple fractures, dislocations, crashes, wounds, sprains, and strains	0.1944
Maldives	2000	Multiple fractures, dislocations, crashes, wounds, sprains, and strains	0.1583
Maldives	2005	Multiple fractures, dislocations, crashes, wounds, sprains, and strains	0.1328
Maldives	2010	Multiple fractures, dislocations, crashes, wounds, sprains, and strains	0.1013
Maldives	2017	Multiple fractures, dislocations, crashes, wounds, sprains, and strains	0.0747
Maldives	1990	Amputation of upper limb, unilateral	0.0773
Maldives	1995	Amputation of upper limb, unilateral	0.0704
Maldives	2000	Amputation of upper limb, unilateral	0.0621
Maldives	2005	Amputation of upper limb, unilateral	0.0562
Maldives	2010	Amputation of upper limb, unilateral	0.049
Maldives	2017	Amputation of upper limb, unilateral	0.0429
Maldives	1990	Amputation of thumb	0.0114
Maldives	1995	Amputation of thumb	0.0114
Maldives	2000	Amputation of thumb	0.0114
Maldives	2005	Amputation of thumb	0.0114
Maldives	2010	Amputation of thumb	0.0114
Maldives	2017	Amputation of thumb	0.0114
Maldives	1990	Amputation of toe/toes	0.0059
Maldives	1995	Amputation of toe/toes	0.0059
Maldives	2000	Amputation of toe/toes	0.0059
Maldives	2005	Amputation of toe/toes	0.0059
Maldives	2010	Amputation of toe/toes	0.0059
Maldives	2017	Amputation of toe/toes	0.0059
Maldives	1990	Burns, <20% total burned surface area without lower airway burns	0.0163
Maldives	1995	Burns, <20% total burned surface area without lower airway burns	0.0163
Maldives	2000	Burns, <20% total burned surface area without lower airway burns	0.0163
Maldives	2005	Burns, <20% total burned surface area without lower airway burns	0.0163
Maldives	2010	Burns, <20% total burned surface area without lower airway burns	0.0163
Maldives	2017	Burns, <20% total burned surface area without lower airway burns	0.0163
Maldives	1990	Burns, >=20% total burned surface area or >= 10% burned surface area if head/neck or hands/wrist involved w/o lower airway burns	0.2901
Maldives	1995	Burns, >=20% total burned surface area or >= 10% burned surface area if head/neck or hands/wrist involved w/o lower airway burns	0.2619
Maldives	2000	Burns, >=20% total burned surface area or >= 10% burned surface area if head/neck or hands/wrist involved w/o lower airway burns	0.2283
Maldives	2005	Burns, >=20% total burned surface area or >= 10% burned surface area if head/neck or hands/wrist involved w/o lower airway burns	0.2045
Maldives	2010	Burns, >=20% total burned surface area or >= 10% burned surface area if head/neck or hands/wrist involved w/o lower airway burns	0.1752
Maldives	2017	Burns, >=20% total burned surface area or >= 10% burned surface area if head/neck or hands/wrist involved w/o lower airway burns	0.1505
Myanmar	1990	Amputation of lower limbs, bilateral	0.3089
Myanmar	1995	Amputation of lower limbs, bilateral	0.305
Myanmar	2000	Amputation of lower limbs, bilateral	0.2982
Myanmar	2005	Amputation of lower limbs, bilateral	0.2837
Myanmar	2010	Amputation of lower limbs, bilateral	0.2645
Myanmar	2017	Amputation of lower limbs, bilateral	0.2327
Myanmar	1990	Lower airway burns	0.2252
Myanmar	1995	Lower airway burns	0.2252
Myanmar	2000	Lower airway burns	0.2252
Myanmar	2005	Lower airway burns	0.2252
Myanmar	2010	Lower airway burns	0.2252
Myanmar	2017	Lower airway burns	0.2252
Myanmar	1990	Dislocation of hip	0.0159
Myanmar	1995	Dislocation of hip	0.0159
Myanmar	2000	Dislocation of hip	0.0159
Myanmar	2005	Dislocation of hip	0.0159
Myanmar	2010	Dislocation of hip	0.0159
Myanmar	2017	Dislocation of hip	0.0159
Myanmar	1990	Dislocation of knee	0.1134
Myanmar	1995	Dislocation of knee	0.1134
Myanmar	2000	Dislocation of knee	0.1134
Myanmar	2005	Dislocation of knee	0.1134
Myanmar	2010	Dislocation of knee	0.1134
Myanmar	2017	Dislocation of knee	0.1134
Myanmar	1990	Dislocation of shoulder	0.062
Myanmar	1995	Dislocation of shoulder	0.062
Myanmar	2000	Dislocation of shoulder	0.062
Myanmar	2005	Dislocation of shoulder	0.062
Myanmar	2010	Dislocation of shoulder	0.062
Myanmar	2017	Dislocation of shoulder	0.062
Myanmar	1990	Muscle and tendon injuries, including sprains and strains lesser dislocations	0.0075
Myanmar	1995	Muscle and tendon injuries, including sprains and strains lesser dislocations	0.0075
Myanmar	2000	Muscle and tendon injuries, including sprains and strains lesser dislocations	0.0075
Myanmar	2005	Muscle and tendon injuries, including sprains and strains lesser dislocations	0.0075
Myanmar	2010	Muscle and tendon injuries, including sprains and strains lesser dislocations	0.0075
Myanmar	2017	Muscle and tendon injuries, including sprains and strains lesser dislocations	0.0075
Myanmar	1990	Fracture of clavicle, scapula, or humerus	0.0349
Myanmar	1995	Fracture of clavicle, scapula, or humerus	0.0349
Myanmar	2000	Fracture of clavicle, scapula, or humerus	0.0349
Myanmar	2005	Fracture of clavicle, scapula, or humerus	0.0349
Myanmar	2010	Fracture of clavicle, scapula, or humerus	0.0349
Myanmar	2017	Fracture of clavicle, scapula, or humerus	0.0349
Myanmar	1990	Fracture of face bones	0.0669
Myanmar	1995	Fracture of face bones	0.0669
Myanmar	2000	Fracture of face bones	0.0669
Myanmar	2005	Fracture of face bones	0.0669
Myanmar	2010	Fracture of face bones	0.0669
Myanmar	2017	Fracture of face bones	0.0669
Myanmar	1990	Fracture of foot bones except ankle	0.0264
Myanmar	1995	Fracture of foot bones except ankle	0.0264
Myanmar	2000	Fracture of foot bones except ankle	0.0264
Myanmar	2005	Fracture of foot bones except ankle	0.0264
Myanmar	2010	Fracture of foot bones except ankle	0.0264
Myanmar	2017	Fracture of foot bones except ankle	0.0264
Myanmar	1990	Fracture of hand (wrist and other distal part of hand)	0.0138
Myanmar	1995	Fracture of hand (wrist and other distal part of hand)	0.0138
Myanmar	2000	Fracture of hand (wrist and other distal part of hand)	0.0138
Myanmar	2005	Fracture of hand (wrist and other distal part of hand)	0.0138
Myanmar	2010	Fracture of hand (wrist and other distal part of hand)	0.0138
Myanmar	2017	Fracture of hand (wrist and other distal part of hand)	0.0138
Myanmar	1990	Fracture of hip	0.3032
Myanmar	1995	Fracture of hip	0.2981
Myanmar	2000	Fracture of hip	0.2891
Myanmar	2005	Fracture of hip	0.27
Myanmar	2010	Fracture of hip	0.2445
Myanmar	2017	Fracture of hip	0.2024
Myanmar	1990	Amputation of upper limbs, bilateral	0.3411
Myanmar	1995	Amputation of upper limbs, bilateral	0.3359
Myanmar	2000	Amputation of upper limbs, bilateral	0.3266
Myanmar	2005	Amputation of upper limbs, bilateral	0.3068
Myanmar	2010	Amputation of upper limbs, bilateral	0.2805
Myanmar	2017	Amputation of upper limbs, bilateral	0.2371
Myanmar	1990	Fracture of patella, tibia or fibula, or ankle	0.0554
Myanmar	1995	Fracture of patella, tibia or fibula, or ankle	0.0554
Myanmar	2000	Fracture of patella, tibia or fibula, or ankle	0.0554
Myanmar	2005	Fracture of patella, tibia or fibula, or ankle	0.0554
Myanmar	2010	Fracture of patella, tibia or fibula, or ankle	0.0554
Myanmar	2017	Fracture of patella, tibia or fibula, or ankle	0.0554
Myanmar	1990	Fracture of pelvis	0.1823
Myanmar	1995	Fracture of pelvis	0.1823
Myanmar	2000	Fracture of pelvis	0.1823
Myanmar	2005	Fracture of pelvis	0.1823
Myanmar	2010	Fracture of pelvis	0.1823
Myanmar	2017	Fracture of pelvis	0.1823
Myanmar	1990	Fracture of radius and/or ulna	0.0435
Myanmar	1995	Fracture of radius and/or ulna	0.0435
Myanmar	2000	Fracture of radius and/or ulna	0.0435
Myanmar	2005	Fracture of radius and/or ulna	0.0435
Myanmar	2010	Fracture of radius and/or ulna	0.0435
Myanmar	2017	Fracture of radius and/or ulna	0.0435
Myanmar	1990	Fracture of skull	0.0714
Myanmar	1995	Fracture of skull	0.0714
Myanmar	2000	Fracture of skull	0.0714
Myanmar	2005	Fracture of skull	0.0714
Myanmar	2010	Fracture of skull	0.0714
Myanmar	2017	Fracture of skull	0.0714
Myanmar	1990	Fracture of sternum and/or fracture of one or more ribs	0.1027
Myanmar	1995	Fracture of sternum and/or fracture of one or more ribs	0.1027
Myanmar	2000	Fracture of sternum and/or fracture of one or more ribs	0.1027
Myanmar	2005	Fracture of sternum and/or fracture of one or more ribs	0.1027
Myanmar	2010	Fracture of sternum and/or fracture of one or more ribs	0.1027
Myanmar	2017	Fracture of sternum and/or fracture of one or more ribs	0.1027
Myanmar	1990	Fracture of vertebral column	0.1106
Myanmar	1995	Fracture of vertebral column	0.1106
Myanmar	2000	Fracture of vertebral column	0.1106
Myanmar	2005	Fracture of vertebral column	0.1106
Myanmar	2010	Fracture of vertebral column	0.1106
Myanmar	2017	Fracture of vertebral column	0.1106
Myanmar	1990	Fracture of femur, other than femoral neck	0.042
Myanmar	1995	Fracture of femur, other than femoral neck	0.042
Myanmar	2000	Fracture of femur, other than femoral neck	0.042
Myanmar	2005	Fracture of femur, other than femoral neck	0.042
Myanmar	2010	Fracture of femur, other than femoral neck	0.042
Myanmar	2017	Fracture of femur, other than femoral neck	0.042
Myanmar	1990	Minor TBI	0.1319
Myanmar	1995	Minor TBI	0.1319
Myanmar	2000	Minor TBI	0.1319
Myanmar	2005	Minor TBI	0.1319
Myanmar	2010	Minor TBI	0.1319
Myanmar	2017	Minor TBI	0.1319
Myanmar	1990	Moderate TBI	0.1637
Myanmar	1995	Moderate TBI	0.1637
Myanmar	2000	Moderate TBI	0.1637
Myanmar	2005	Moderate TBI	0.1637
Myanmar	2010	Moderate TBI	0.1637
Myanmar	2017	Moderate TBI	0.1637
Myanmar	1990	Amputation of fingers (excluding thumb)	0.0047
Myanmar	1995	Amputation of fingers (excluding thumb)	0.0047
Myanmar	2000	Amputation of fingers (excluding thumb)	0.0047
Myanmar	2005	Amputation of fingers (excluding thumb)	0.0047
Myanmar	2010	Amputation of fingers (excluding thumb)	0.0047
Myanmar	2017	Amputation of fingers (excluding thumb)	0.0047
Myanmar	1990	Spinal cord lesion at neck level	0.4967
Myanmar	1995	Spinal cord lesion at neck level	0.4949
Myanmar	2000	Spinal cord lesion at neck level	0.4918
Myanmar	2005	Spinal cord lesion at neck level	0.4852
Myanmar	2010	Spinal cord lesion at neck level	0.4764
Myanmar	2017	Spinal cord lesion at neck level	0.4619
Myanmar	1990	Spinal cord lesion below neck level	0.3569
Myanmar	1995	Spinal cord lesion below neck level	0.3531
Myanmar	2000	Spinal cord lesion below neck level	0.3465
Myanmar	2005	Spinal cord lesion below neck level	0.3326
Myanmar	2010	Spinal cord lesion below neck level	0.314
Myanmar	2017	Spinal cord lesion below neck level	0.2833
Myanmar	1990	Drowning and nonfatal submersion	0.2471
Myanmar	1995	Drowning and nonfatal submersion	0.2471
Myanmar	2000	Drowning and nonfatal submersion	0.2471
Myanmar	2005	Drowning and nonfatal submersion	0.2471
Myanmar	2010	Drowning and nonfatal submersion	0.2471
Myanmar	2017	Drowning and nonfatal submersion	0.2471
Myanmar	1990	Asphyxiation	0.2471
Myanmar	1995	Asphyxiation	0.2471
Myanmar	2000	Asphyxiation	0.2471
Myanmar	2005	Asphyxiation	0.2471
Myanmar	2010	Asphyxiation	0.2471
Myanmar	2017	Asphyxiation	0.2471
Myanmar	1990	Crush injury	0.1325
Myanmar	1995	Crush injury	0.1325
Myanmar	2000	Crush injury	0.1325
Myanmar	2005	Crush injury	0.1325
Myanmar	2010	Crush injury	0.1325
Myanmar	2017	Crush injury	0.1325
Myanmar	1990	Nerve injury	0.1129
Myanmar	1995	Nerve injury	0.1129
Myanmar	2000	Nerve injury	0.1129
Myanmar	2005	Nerve injury	0.1129
Myanmar	2010	Nerve injury	0.1129
Myanmar	2017	Nerve injury	0.1129
Myanmar	1990	Injury to eyes	0.0165
Myanmar	1995	Injury to eyes	0.0165
Myanmar	2000	Injury to eyes	0.0165
Myanmar	2005	Injury to eyes	0.0165
Myanmar	2010	Injury to eyes	0.0165
Myanmar	2017	Injury to eyes	0.0165
Myanmar	1990	Amputation of lower limb, unilateral	0.1346

Myanmar	1995	Amputation of lower limb, unilateral	0.1327
Myanmar	2000	Amputation of lower limb, unilateral	0.1292
Myanmar	2005	Amputation of lower limb, unilateral	0.1217
Myanmar	2010	Amputation of lower limb, unilateral	0.1118
Myanmar	2017	Amputation of lower limb, unilateral	0.0954
Myanmar	1990	Open wound(s)	0.011
Myanmar	1995	Open wound(s)	0.011
Myanmar	2000	Open wound(s)	0.011
Myanmar	2005	Open wound(s)	0.011
Myanmar	2010	Open wound(s)	0.011
Myanmar	2017	Open wound(s)	0.011
Myanmar	1990	Poisoning requiring urgent care	0.1628
Myanmar	1995	Poisoning requiring urgent care	0.1628
Myanmar	2000	Poisoning requiring urgent care	0.1628
Myanmar	2005	Poisoning requiring urgent care	0.1628
Myanmar	2010	Poisoning requiring urgent care	0.1628
Myanmar	2017	Poisoning requiring urgent care	0.1628
Myanmar	1990	Severe chest injury	0.0474
Myanmar	1995	Severe chest injury	0.0474
Myanmar	2000	Severe chest injury	0.0474
Myanmar	2005	Severe chest injury	0.0474
Myanmar	2010	Severe chest injury	0.0474
Myanmar	2017	Severe chest injury	0.0474
Myanmar	1990	Internal hemorrhage in abdomen and pelvis	0.1143
Myanmar	1995	Internal hemorrhage in abdomen and pelvis	0.1143
Myanmar	2000	Internal hemorrhage in abdomen and pelvis	0.1143
Myanmar	2005	Internal hemorrhage in abdomen and pelvis	0.1143
Myanmar	2010	Internal hemorrhage in abdomen and pelvis	0.1143
Myanmar	2017	Internal hemorrhage in abdomen and pelvis	0.1143
Myanmar	1990	Contusion in any part of the body	0.0075
Myanmar	1995	Contusion in any part of the body	0.0075
Myanmar	2000	Contusion in any part of the body	0.0075
Myanmar	2005	Contusion in any part of the body	0.0075
Myanmar	2010	Contusion in any part of the body	0.0075
Myanmar	2017	Contusion in any part of the body	0.0075
Myanmar	1990	Effect of different environmental factors	0.049
Myanmar	1995	Effect of different environmental factors	0.049
Myanmar	2000	Effect of different environmental factors	0.049
Myanmar	2005	Effect of different environmental factors	0.049
Myanmar	2010	Effect of different environmental factors	0.049
Myanmar	2017	Effect of different environmental factors	0.049
Myanmar	1990	Complications following therapeutic procedures	0.0506
Myanmar	1995	Complications following therapeutic procedures	0.0506
Myanmar	2000	Complications following therapeutic procedures	0.0506
Myanmar	2005	Complications following therapeutic procedures	0.0506
Myanmar	2010	Complications following therapeutic procedures	0.0506
Myanmar	2017	Complications following therapeutic procedures	0.0506
Myanmar	1990	Multiple fractures, dislocations, crashes, wounds, sprains, and strains	0.3032
Myanmar	1995	Multiple fractures, dislocations, crashes, wounds, sprains, and strains	0.2981
Myanmar	2000	Multiple fractures, dislocations, crashes, wounds, sprains, and strains	0.2891
Myanmar	2005	Multiple fractures, dislocations, crashes, wounds, sprains, and strains	0.27
Myanmar	2010	Multiple fractures, dislocations, crashes, wounds, sprains, and strains	0.2445
Myanmar	2017	Multiple fractures, dislocations, crashes, wounds, sprains, and strains	0.2024
Myanmar	1990	Amputation of upper limb, unilateral	0.0953
Myanmar	1995	Amputation of upper limb, unilateral	0.0941
Myanmar	2000	Amputation of upper limb, unilateral	0.0921
Myanmar	2005	Amputation of upper limb, unilateral	0.0877
Myanmar	2010	Amputation of upper limb, unilateral	0.0818
Myanmar	2017	Amputation of upper limb, unilateral	0.0722
Myanmar	1990	Amputation of thumb	0.0114
Myanmar	1995	Amputation of thumb	0.0114
Myanmar	2000	Amputation of thumb	0.0114
Myanmar	2005	Amputation of thumb	0.0114
Myanmar	2010	Amputation of thumb	0.0114
Myanmar	2017	Amputation of thumb	0.0114
Myanmar	1990	Amputation of toe/toes	0.0059
Myanmar	1995	Amputation of toe/toes	0.0059
Myanmar	2000	Amputation of toe/toes	0.0059
Myanmar	2005	Amputation of toe/toes	0.0059
Myanmar	2010	Amputation of toe/toes	0.0059
Myanmar	2017	Amputation of toe/toes	0.0059
Myanmar	1990	Burns, <20% total burned surface area without lower airway burns	0.0163
Myanmar	1995	Burns, <20% total burned surface area without lower airway burns	0.0163
Myanmar	2000	Burns, <20% total burned surface area without lower airway burns	0.0163
Myanmar	2005	Burns, <20% total burned surface area without lower airway burns	0.0163
Myanmar	2010	Burns, <20% total burned surface area without lower airway burns	0.0163
Myanmar	2017	Burns, <20% total burned surface area without lower airway burns	0.0163
Myanmar	1990	Burns, >=20% total burned surface area or >= 10% burned surface area if head/neck or hands/wrist involved w/o lower airway burns	0.3632
Myanmar	1995	Burns, >=20% total burned surface area or >= 10% burned surface area if head/neck or hands/wrist involved w/o lower airway burns	0.3584
Myanmar	2000	Burns, >=20% total burned surface area or >= 10% burned surface area if head/neck or hands/wrist involved w/o lower airway burns	0.3501
Myanmar	2005	Burns, >=20% total burned surface area or >= 10% burned surface area if head/neck or hands/wrist involved w/o lower airway burns	0.3323
Myanmar	2010	Burns, >=20% total burned surface area or >= 10% burned surface area if head/neck or hands/wrist involved w/o lower airway burns	0.3085
Myanmar	2017	Burns, >=20% total burned surface area or >= 10% burned surface area if head/neck or hands/wrist involved w/o lower airway burns	0.2693
Philippines	1990	Amputation of lower limbs, bilateral	0.2447
Philippines	1995	Amputation of lower limbs, bilateral	0.2381
Philippines	2000	Amputation of lower limbs, bilateral	0.2321
Philippines	2005	Amputation of lower limbs, bilateral	0.2253
Philippines	2010	Amputation of lower limbs, bilateral	0.2152
Philippines	2017	Amputation of lower limbs, bilateral	0.2013
Philippines	1990	Lower airway burns	0.2252
Philippines	1995	Lower airway burns	0.2252
Philippines	2000	Lower airway burns	0.2252
Philippines	2005	Lower airway burns	0.2252
Philippines	2010	Lower airway burns	0.2252
Philippines	2017	Lower airway burns	0.2252
Philippines	1990	Dislocation of hip	0.0159
Philippines	1995	Dislocation of hip	0.0159
Philippines	2000	Dislocation of hip	0.0159
Philippines	2005	Dislocation of hip	0.0159
Philippines	2010	Dislocation of hip	0.0159
Philippines	2017	Dislocation of hip	0.0159
Philippines	1990	Dislocation of knee	0.1134
Philippines	1995	Dislocation of knee	0.1134
Philippines	2000	Dislocation of knee	0.1134
Philippines	2005	Dislocation of knee	0.1134
Philippines	2010	Dislocation of knee	0.1134
Philippines	2017	Dislocation of knee	0.1134
Philippines	1990	Dislocation of shoulder	0.062
Philippines	1995	Dislocation of shoulder	0.062
Philippines	2000	Dislocation of shoulder	0.062
Philippines	2005	Dislocation of shoulder	0.062
Philippines	2010	Dislocation of shoulder	0.062
Philippines	2017	Dislocation of shoulder	0.062
Philippines	1990	Muscle and tendon injuries, including sprains and strains lesser dislocations	0.0075
Philippines	1995	Muscle and tendon injuries, including sprains and strains lesser dislocations	0.0075
Philippines	2000	Muscle and tendon injuries, including sprains and strains lesser dislocations	0.0075
Philippines	2005	Muscle and tendon injuries, including sprains and strains lesser dislocations	0.0075
Philippines	2010	Muscle and tendon injuries, including sprains and strains lesser dislocations	0.0075
Philippines	2017	Muscle and tendon injuries, including sprains and strains lesser dislocations	0.0075
Philippines	1990	Fracture of clavicle, scapula, or humerus	0.0349
Philippines	1995	Fracture of clavicle, scapula, or humerus	0.0349
Philippines	2000	Fracture of clavicle, scapula, or humerus	0.0349
Philippines	2005	Fracture of clavicle, scapula, or humerus	0.0349
Philippines	2010	Fracture of clavicle, scapula, or humerus	0.0349
Philippines	2017	Fracture of clavicle, scapula, or humerus	0.0349
Philippines	1990	Fracture of face bones	0.0669
Philippines	1995	Fracture of face bones	0.0669
Philippines	2000	Fracture of face bones	0.0669
Philippines	2005	Fracture of face bones	0.0669
Philippines	2010	Fracture of face bones	0.0669
Philippines	2017	Fracture of face bones	0.0669
Philippines	1990	Fracture of foot bones except ankle	0.0264
Philippines	1995	Fracture of foot bones except ankle	0.0264
Philippines	2000	Fracture of foot bones except ankle	0.0264
Philippines	2005	Fracture of foot bones except ankle	0.0264
Philippines	2010	Fracture of foot bones except ankle	0.0264
Philippines	2017	Fracture of foot bones except ankle	0.0264
Philippines	1990	Fracture of hand (wrist and other distal part of hand)	0.0138
Philippines	1995	Fracture of hand (wrist and other distal part of hand)	0.0138
Philippines	2000	Fracture of hand (wrist and other distal part of hand)	0.0138
Philippines	2005	Fracture of hand (wrist and other distal part of hand)	0.0138
Philippines	2010	Fracture of hand (wrist and other distal part of hand)	0.0138
Philippines	2017	Fracture of hand (wrist and other distal part of hand)	0.0138
Philippines	1990	Fracture of hip	0.2183
Philippines	1995	Fracture of hip	0.2096
Philippines	2000	Fracture of hip	0.2016
Philippines	2005	Fracture of hip	0.1926
Philippines	2010	Fracture of hip	0.1793
Philippines	2017	Fracture of hip	0.1609
Philippines	1990	Amputation of upper limbs, bilateral	0.2535
Philippines	1995	Amputation of upper limbs, bilateral	0.2445
Philippines	2000	Amputation of upper limbs, bilateral	0.2363
Philippines	2005	Amputation of upper limbs, bilateral	0.227
Philippines	2010	Amputation of upper limbs, bilateral	0.2132
Philippines	2017	Amputation of upper limbs, bilateral	0.1942
Philippines	1990	Fracture of patella, tibia or fibula, or ankle	0.0554
Philippines	1995	Fracture of patella, tibia or fibula, or ankle	0.0554
Philippines	2000	Fracture of patella, tibia or fibula, or ankle	0.0554
Philippines	2005	Fracture of patella, tibia or fibula, or ankle	0.0554
Philippines	2010	Fracture of patella, tibia or fibula, or ankle	0.0554
Philippines	2017	Fracture of patella, tibia or fibula, or ankle	0.0554
Philippines	1990	Fracture of pelvis	0.1823
Philippines	1995	Fracture of pelvis	0.1823
Philippines	2000	Fracture of pelvis	0.1823
Philippines	2005	Fracture of pelvis	0.1823
Philippines	2010	Fracture of pelvis	0.1823
Philippines	2017	Fracture of pelvis	0.1823
Philippines	1990	Fracture of radius and/or ulna	0.0435
Philippines	1995	Fracture of radius and/or ulna	0.0435
Philippines	2000	Fracture of radius and/or ulna	0.0435
Philippines	2005	Fracture of radius and/or ulna	0.0435
Philippines	2010	Fracture of radius and/or ulna	0.0435
Philippines	2017	Fracture of radius and/or ulna	0.0435
Philippines	1990	Fracture of skull	0.0714
Philippines	1995	Fracture of skull	0.0714
Philippines	2000	Fracture of skull	0.0714
Philippines	2005	Fracture of skull	0.0714
Philippines	2010	Fracture of skull	0.0714
Philippines	2017	Fracture of skull	0.0714
Philippines	1990	Fracture of sternum and/or fracture of one or more ribs	0.1027
Philippines	1995	Fracture of sternum and/or fracture of one or more ribs	0.1027
Philippines	2000	Fracture of sternum and/or fracture of one or more ribs	0.1027
Philippines	2005	Fracture of sternum and/or fracture of one or more ribs	0.1027
Philippines	2010	Fracture of sternum and/or fracture of one or more ribs	0.1027
Philippines	2017	Fracture of sternum and/or fracture of one or more ribs	0.1027
Philippines	1990	Fracture of vertebral column	0.1106
Philippines	1995	Fracture of vertebral column	0.1106
Philippines	2000	Fracture of vertebral column	0.1106
Philippines	2005	Fracture of vertebral column	0.1106
Philippines	2010	Fracture of vertebral column	0.1106
Philippines	2017	Fracture of vertebral column	0.1106
Philippines	1990	Fracture of femur, other than femoral neck	0.042
Philippines	1995	Fracture of femur, other than femoral neck	0.042
Philippines	2000	Fracture of femur, other than femoral neck	0.042
Philippines	2005	Fracture of femur, other than femoral neck	0.042
Philippines	2010	Fracture of femur, other than femoral neck	0.042
Philippines	2017	Fracture of femur, other than femoral neck	0.042
Philippines	1990	Minor TBI	0.1319
Philippines	1995	Minor TBI	0.1319
Philippines	2000	Minor TBI	0.1319
Philippines	2005	Minor TBI	0.1319
Philippines	2010	Minor TBI	0.1319
Philippines	2017	Minor TBI	0.1319
Philippines	1990	Moderate TBI	0.1637
Philippines	1995	Moderate TBI	0.1637
Philippines	2000	Moderate TBI	0.1637
Philippines	2005	Moderate TBI	0.1637
Philippines	2010	Moderate TBI	0.1637
Philippines	2017	Moderate TBI	0.1637
Philippines	1990	Amputation of fingers (excluding thumb)	0.0047
Philippines	1995	Amputation of fingers (excluding thumb)	0.0047
Philippines	2000	Amputation of fingers (excluding thumb)	0.0047
Philippines	2005	Amputation of fingers (excluding thumb)	0.0047
Philippines	2010	Amputation of fingers (excluding thumb)	0.0047
Philippines	2017	Amputation of fingers (excluding thumb)	0.0047
Philippines	1990	Spinal cord lesion at neck level	0.4674
Philippines	1995	Spinal cord lesion at neck level	0.4643
Philippines	2000	Spinal cord lesion at neck level	0.4616
Philippines	2005	Spinal cord lesion at neck level	0.4585
Philippines	2010	Spinal cord lesion at neck level	0.4539
Philippines	2017	Spinal cord lesion at neck level	0.4475
Philippines	1990	Spinal cord lesion below neck level	0.2949
Philippines	1995	Spinal cord lesion below neck level	0.2885
Philippines	2000	Spinal cord lesion below neck level	0.2827
Philippines	2005	Spinal cord lesion below neck level	0.2762
Philippines	2010	Spinal cord lesion below neck level	0.2664
Philippines	2017	Spinal cord lesion below neck level	0.253
Philippines	1990	Drowning and nonfatal submersion	0.2471
Philippines	1995	Drowning and nonfatal submersion	0.2471
Philippines	2000	Drowning and nonfatal submersion	0.2471
Philippines	2005	Drowning and nonfatal submersion	0.2471
Philippines	2010	Drowning and nonfatal submersion	0.2471
Philippines	2017	Drowning and nonfatal submersion	0.2471
Philippines	1990	Asphyxiation	0.2471
Philippines	1995	Asphyxiation	0.2471
Philippines	2000	Asphyxiation	0.2471
Philippines	2005	Asphyxiation	0.2471
Philippines	2010	Asphyxiation	0.2471
Philippines	2017	Asphyxiation	0.2471
Philippines	1990	Crush injury	0.1325
Philippines	1995	Crush injury	0.1325
Philippines	2000	Crush injury	0.1325
Philippines	2005	Crush injury	0.1325
Philippines	2010	Crush injury	0.1325
Philippines	2017	Crush injury	0.1325
Philippines	1990	Nerve injury	0.1129
Philippines	1995	Nerve injury	0.1129
Philippines	2000	Nerve injury	0.1129
Philippines	2005	Nerve injury	0.1129
Philippines	2010	Nerve injury	0.1129
Philippines	2017	Nerve injury	0.1129
Philippines	1990	Injury to eyes	0.0165
Philippines	1995	Injury to eyes	0.0165
Philippines	2000	Injury to eyes	0.0165
Philippines	2005	Injury to eyes	0.0165
Philippines	2010	Injury to eyes	0.0165
Philippines	2017	Injury to eyes	0.0165
Philippines	1990	Amputation of lower limb, unilateral	0.1016
Philippines	1995	Amputation of lower limb, unilateral	0.0981
Philippines	2000	Amputation of lower limb, unilateral	0.0951
Philippines	2005	Amputation of lower limb, unilateral	0.0916
Philippines	2010	Amputation of lower limb, unilateral	0.0863
Philippines	2017	Amputation of lower limb, unilateral	0.0792
Philippines	1990	Open wound(s)	0.011
Philippines	1995	Open wound(s)	0.011
Philippines	2000	Open wound(s)	0.011
Philippines	2005	Open wound(s)	0.011
Philippines	2010	Open wound(s)	0.011
Philippines	2017	Open wound(s)	0.011
Philippines	1990	Poisoning requiring urgent care	0.1628
Philippines	1995	Poisoning requiring urgent care	0.1628
Philippines	2000	Poisoning requiring urgent care	0.1628
Philippines	2005	Poisoning requiring urgent care	0.1628
Philippines	2010	Poisoning requiring urgent care	0.1628
Philippines	2017	Poisoning requiring urgent care	0.1628
Philippines	1990	Severe chest injury	0.0474
Philippines	1995	Severe chest injury	0.0474
Philippines	2000	Severe chest injury	0.0474
Philippines	2005	Severe chest injury	0.0474
Philippines	2010	Severe chest injury	0.0474
Philippines	2017	Severe chest injury	0.0474
Philippines	1990	Internal hemorrhage in abdomen and pelvis	0.1143
Philippines	1995	Internal hemorrhage in abdomen and pelvis	0.1143
Philippines	2000	Internal hemorrhage in abdomen and pelvis	0.1143
Philippines	2005	Internal hemorrhage in abdomen and pelvis	0.1143
Philippines	2010	Internal hemorrhage in abdomen and pelvis	0.1143
Philippines	2017	Internal hemorrhage in abdomen and pelvis	0.1143
Philippines	1990	Contusion in any part of the body	0.0075
Philippines	1995	Contusion in any part of the body	0.0075
Philippines	2000	Contusion in any part of the body	0.0075
Philippines	2005	Contusion in any part of the body	0.0075
Philippines	2010	Contusion in any part of the body	0.0075
Philippines	2017	Contusion in any part of the body	0.0075
Philippines	1990	Effect of different environmental factors	0.049
Philippines	1995	Effect of different environmental factors	0.049
Philippines	2000	Effect of different environmental factors	0.049
Philippines	2005	Effect of different environmental factors	0.049
Philippines	2010	Effect of different environmental factors	0.049
Philippines	2017	Effect of different environmental factors	0.049
Philippines	1990	Complications following therapeutic procedures	0.0506
Philippines	1995	Complications following therapeutic procedures	0.0506
Philippines	2000	Complications following therapeutic procedures	0.0506
Philippines	2005	Complications following therapeutic procedures	0.0506
Philippines	2010	Complications following therapeutic procedures	0.0506
Philippines	2017	Complications following therapeutic procedures	0.0506
Philippines	1990	Multiple fractures, dislocations, crashes, wounds, sprains, and strains	0.2183
Philippines	1995	Multiple fractures, dislocations, crashes, wounds, sprains, and strains	0.2096
Philippines	2000	Multiple fractures, dislocations, crashes, wounds, sprains, and strains	0.2016
Philippines	2005	Multiple fractures, dislocations, crashes, wounds, sprains, and strains	0.1926
Philippines	2010	Multiple fractures, dislocations, crashes, wounds, sprains, and strains	0.1793
Philippines	2017	Multiple fractures, dislocations, crashes, wounds, sprains, and strains	0.1609
Philippines	1990	Amputation of upper limb, unilateral	0.0758
Philippines	1995	Amputation of upper limb, unilateral	0.0738
Philippines	2000	Amputation of upper limb, unilateral	0.072
Philippines	2005	Amputation of upper limb, unilateral	0.0699
Philippines	2010	Amputation of upper limb, unilateral	0.0669
Philippines	2017	Amputation of upper limb, unilateral	0.0627
Philippines	1990	Amputation of thumb	0.0114
Philippines	1995	Amputation of thumb	0.0114
Philippines	2000	Amputation of thumb	0.0114
Philippines	2005	Amputation of thumb	0.0114
Philippines	2010	Amputation of thumb	0.0114
Philippines	2017	Amputation of thumb	0.0114
Philippines	1990	Amputation of toe/toes	0.0059
Philippines	1995	Amputation of toe/toes	0.0059
Philippines	2000	Amputation of toe/toes	0.0059
Philippines	2005	Amputation of toe/toes	0.0059
Philippines	2010	Amputation of toe/toes	0.0059
Philippines	2017	Amputation of toe/toes	0.0059
Philippines	1990	Burns, <20% total burned surface area without lower airway burns	0.0163
Philippines	1995	Burns, <20% total burned surface area without lower airway burns	0.0163
Philippines	2000	Burns, <20% total burned surface area without lower airway burns	0.0163
Philippines	2005	Burns, <20% total burned surface area without lower airway burns	0.0163
Philippines	2010	Burns, <20% total burned surface area without lower airway burns	0.0163
Philippines	2017	Burns, <20% total burned surface area without lower airway burns	0.0163
Philippines	1990	Burns, >=20% total burned surface area or >= 10% burned surface area if head/neck or hands/wrist involved w/o lower airway burns	0.2842
Philippines	1995	Burns, >=20% total burned surface area or >= 10% burned surface area if head/neck or hands/wrist involved w/o lower airway burns	0.276
Philippines	2000	Burns, >=20% total burned surface area or >= 10% burned surface area if head/neck or hands/wrist involved w/o lower airway burns	0.2686
Philippines	2005	Burns, >=20% total burned surface area or >= 10% burned surface area if head/neck or hands/wrist involved w/o lower airway burns	0.2602
Philippines	2010	Burns, >=20% total burned surface area or >= 10% burned surface area if head/neck or hands/wrist involved w/o lower airway burns	0.2478
Philippines	2017	Burns, >=20% total burned surface area or >= 10% burned surface area if head/neck or hands/wrist involved w/o lower airway burns	0.2307
Sri Lanka	1990	Amputation of lower limbs, bilateral	0.2162
Sri Lanka	1995	Amputation of lower limbs, bilateral	0.2009
Sri Lanka	2000	Amputation of lower limbs, bilateral	0.1923
Sri Lanka	2005	Amputation of lower limbs, bilateral	0.1758
Sri Lanka	2010	Amputation of lower limbs, bilateral	0.1578
Sri Lanka	2017	Amputation of lower limbs, bilateral	0.1357
Sri Lanka	1990	Lower airway burns	0.2252
Sri Lanka	1995	Lower airway burns	0.2252
Sri Lanka	2000	Lower airway burns	0.2252
Sri Lanka	2005	Lower airway burns	0.2252
Sri Lanka	2010	Lower airway burns	0.2252
Sri Lanka	2017	Lower airway burns	0.2252
Sri Lanka	1990	Dislocation of hip	0.0159
Sri Lanka	1995	Dislocation of hip	0.0159
Sri Lanka	2000	Dislocation of hip	0.0159
Sri Lanka	2005	Dislocation of hip	0.0159
Sri Lanka	2010	Dislocation of hip	0.0159
Sri Lanka	2017	Dislocation of hip	0.0159
Sri Lanka	1990	Dislocation of knee	0.1134
Sri Lanka	1995	Dislocation of knee	0.1134
Sri Lanka	2000	Dislocation of knee	0.1134
Sri Lanka	2005	Dislocation of knee	0.1134
Sri Lanka	2010	Dislocation of knee	0.1134
Sri Lanka	2017	Dislocation of knee	0.1134
Sri Lanka	1990	Dislocation of shoulder	0.062
Sri Lanka	1995	Dislocation of shoulder	0.062
Sri Lanka	2000	Dislocation of shoulder	0.062
Sri Lanka	2005	Dislocation of shoulder	0.062
Sri Lanka	2010	Dislocation of shoulder	0.062
Sri Lanka	2017	Dislocation of shoulder	0.062
Sri Lanka	1990	Muscle and tendon injuries, including sprains and strains lesser dislocations	0.0075
Sri Lanka	1995	Muscle and tendon injuries, including sprains and strains lesser dislocations	0.0075
Sri Lanka	2000	Muscle and tendon injuries, including sprains and strains lesser dislocations	0.0075
Sri Lanka	2005	Muscle and tendon injuries, including sprains and strains lesser dislocations	0.0075
Sri Lanka	2010	Muscle and tendon injuries, including sprains and strains lesser dislocations	0.0075
Sri Lanka	2017	Muscle and tendon injuries, including sprains and strains lesser dislocations	0.0075
Sri Lanka	1990	Fracture of clavicle, scapula, or humerus	0.0349
Sri Lanka	1995	Fracture of clavicle, scapula, or humerus	0.0349
Sri Lanka	2000	Fracture of clavicle, scapula, or humerus	0.0349
Sri Lanka	2005	Fracture of clavicle, scapula, or humerus	0.0349
Sri Lanka	2010	Fracture of clavicle, scapula, or humerus	0.0349
Sri Lanka	2017	Fracture of clavicle, scapula, or humerus	0.0349
Sri Lanka	1990	Fracture of face bones	0.0669
Sri Lanka	1995	Fracture of face bones	0.0669
Sri Lanka	2000	Fracture of face bones	0.0669
Sri Lanka	2005	Fracture of face bones	0.0669
Sri Lanka	2010	Fracture of face bones	0.0669
Sri Lanka	2017	Fracture of face bones	0.0669
Sri Lanka	1990	Fracture of foot bones except ankle	0.0264
Sri Lanka	1995	Fracture of foot bones except ankle	0.0264
Sri Lanka	2000	Fracture of foot bones except ankle	0.0264
Sri Lanka	2005	Fracture of foot bones except ankle	0.0264
Sri Lanka	2010	Fracture of foot bones except ankle	0.0264
Sri Lanka	2017	Fracture of foot bones except ankle	0.0264
Sri Lanka	1990	Fracture of hand (wrist and other distal part of hand)	0.0138
Sri Lanka	1995	Fracture of hand (wrist and other distal part of hand)	0.0138
Sri Lanka	2000	Fracture of hand (wrist and other distal part of hand)	0.0138
Sri Lanka	2005	Fracture of hand (wrist and other distal part of hand)	0.0138
Sri Lanka	2010	Fracture of hand (wrist and other distal part of hand)	0.0138
Sri Lanka	2017	Fracture of hand (wrist and other distal part of hand)	0.0138
Sri Lanka	1990	Fracture of hip	0.1806
Sri Lanka	1995	Fracture of hip	0.1603
Sri Lanka	2000	Fracture of hip	0.149
Sri Lanka	2005	Fracture of hip	0.1271
Sri Lanka	2010	Fracture of hip	0.1034
Sri Lanka	2017	Fracture of hip	0.0742
Sri Lanka	1990	Amputation of upper limbs, bilateral	0.2145
Sri Lanka	1995	Amputation of upper limbs, bilateral	0.1936
Sri Lanka	2000	Amputation of upper limbs, bilateral	0.1819
Sri Lanka	2005	Amputation of upper limbs, bilateral	0.1584
Sri Lanka	2010	Amputation of upper limbs, bilateral	0.1349
Sri Lanka	2017	Amputation of upper limbs, bilateral	0.1047
Sri Lanka	1990	Fracture of patella, tibia or fibula, or ankle	0.0554
Sri Lanka	1995	Fracture of patella, tibia or fibula, or ankle	0.0554
Sri Lanka	2000	Fracture of patella, tibia or fibula, or ankle	0.0554
Sri Lanka	2005	Fracture of patella, tibia or fibula, or ankle	0.0554
Sri Lanka	2010	Fracture of patella, tibia or fibula, or ankle	0.0554
Sri Lanka	2017	Fracture of patella, tibia or fibula, or ankle	0.0554
Sri Lanka	1990	Fracture of pelvis	0.1823
Sri Lanka	1995	Fracture of pelvis	0.1823
Sri Lanka	2000	Fracture of pelvis	0.1823
Sri Lanka	2005	Fracture of pelvis	0.1823
Sri Lanka	2010	Fracture of pelvis	0.1823
Sri Lanka	2017	Fracture of pelvis	0.1823
Sri Lanka	1990	Fracture of radius and/or ulna	0.0435
Sri Lanka	1995	Fracture of radius and/or ulna	0.0435
Sri Lanka	2000	Fracture of radius and/or ulna	0.0435
Sri Lanka	2005	Fracture of radius and/or ulna	0.0435
Sri Lanka	2010	Fracture of radius and/or ulna	0.0435
Sri Lanka	2017	Fracture of radius and/or ulna	0.0435

Sri Lanka	1990	Fracture of skull	0.0714
Sri Lanka	1995	Fracture of skull	0.0714
Sri Lanka	2000	Fracture of skull	0.0714
Sri Lanka	2005	Fracture of skull	0.0714
Sri Lanka	2010	Fracture of skull	0.0714
Sri Lanka	2017	Fracture of skull	0.0714
Sri Lanka	1990	Fracture of sternum and/or fracture of one or more ribs	0.1027
Sri Lanka	1995	Fracture of sternum and/or fracture of one or more ribs	0.1027
Sri Lanka	2000	Fracture of sternum and/or fracture of one or more ribs	0.1027
Sri Lanka	2005	Fracture of sternum and/or fracture of one or more ribs	0.1027
Sri Lanka	2010	Fracture of sternum and/or fracture of one or more ribs	0.1027
Sri Lanka	2017	Fracture of sternum and/or fracture of one or more ribs	0.1027
Sri Lanka	1990	Fracture of vertebral column	0.1106
Sri Lanka	1995	Fracture of vertebral column	0.1106
Sri Lanka	2000	Fracture of vertebral column	0.1106
Sri Lanka	2005	Fracture of vertebral column	0.1106
Sri Lanka	2010	Fracture of vertebral column	0.1106
Sri Lanka	2017	Fracture of vertebral column	0.1106
Sri Lanka	1990	Fracture of femur, other than femoral neck	0.042
Sri Lanka	1995	Fracture of femur, other than femoral neck	0.042
Sri Lanka	2000	Fracture of femur, other than femoral neck	0.042
Sri Lanka	2005	Fracture of femur, other than femoral neck	0.042
Sri Lanka	2010	Fracture of femur, other than femoral neck	0.042
Sri Lanka	2017	Fracture of femur, other than femoral neck	0.042
Sri Lanka	1990	Minor TBI	0.1319
Sri Lanka	1995	Minor TBI	0.1319
Sri Lanka	2000	Minor TBI	0.1319
Sri Lanka	2005	Minor TBI	0.1319
Sri Lanka	2010	Minor TBI	0.1319
Sri Lanka	2017	Minor TBI	0.1319
Sri Lanka	1990	Moderate TBI	0.1637
Sri Lanka	1995	Moderate TBI	0.1637
Sri Lanka	2000	Moderate TBI	0.1637
Sri Lanka	2005	Moderate TBI	0.1637
Sri Lanka	2010	Moderate TBI	0.1637
Sri Lanka	2017	Moderate TBI	0.1637
Sri Lanka	1990	Amputation of fingers (excluding thumb)	0.0047
Sri Lanka	1995	Amputation of fingers (excluding thumb)	0.0047
Sri Lanka	2000	Amputation of fingers (excluding thumb)	0.0047
Sri Lanka	2005	Amputation of fingers (excluding thumb)	0.0047
Sri Lanka	2010	Amputation of fingers (excluding thumb)	0.0047
Sri Lanka	2017	Amputation of fingers (excluding thumb)	0.0047
Sri Lanka	1990	Spinal cord lesion at neck level	0.4543
Sri Lanka	1995	Spinal cord lesion at neck level	0.4473
Sri Lanka	2000	Spinal cord lesion at neck level	0.4434
Sri Lanka	2005	Spinal cord lesion at neck level	0.4358
Sri Lanka	2010	Spinal cord lesion at neck level	0.4276
Sri Lanka	2017	Spinal cord lesion at neck level	0.4175
Sri Lanka	1990	Spinal cord lesion below neck level	0.2673
Sri Lanka	1995	Spinal cord lesion below neck level	0.2526
Sri Lanka	2000	Spinal cord lesion below neck level	0.2443
Sri Lanka	2005	Spinal cord lesion below neck level	0.2284
Sri Lanka	2010	Spinal cord lesion below neck level	0.211
Sri Lanka	2017	Spinal cord lesion below neck level	0.1897
Sri Lanka	1990	Drowning and nonfatal submersion	0.2471
Sri Lanka	1995	Drowning and nonfatal submersion	0.2471
Sri Lanka	2000	Drowning and nonfatal submersion	0.2471
Sri Lanka	2005	Drowning and nonfatal submersion	0.2471
Sri Lanka	2010	Drowning and nonfatal submersion	0.2471
Sri Lanka	2017	Drowning and nonfatal submersion	0.2471
Sri Lanka	1990	Asphyxiation	0.2471
Sri Lanka	1995	Asphyxiation	0.2471
Sri Lanka	2000	Asphyxiation	0.2471
Sri Lanka	2005	Asphyxiation	0.2471
Sri Lanka	2010	Asphyxiation	0.2471
Sri Lanka	2017	Asphyxiation	0.2471
Sri Lanka	1990	Crush injury	0.1325
Sri Lanka	1995	Crush injury	0.1325
Sri Lanka	2000	Crush injury	0.1325
Sri Lanka	2005	Crush injury	0.1325
Sri Lanka	2010	Crush injury	0.1325
Sri Lanka	2017	Crush injury	0.1325
Sri Lanka	1990	Nerve injury	0.1129
Sri Lanka	1995	Nerve injury	0.1129
Sri Lanka	2000	Nerve injury	0.1129
Sri Lanka	2005	Nerve injury	0.1129
Sri Lanka	2010	Nerve injury	0.1129
Sri Lanka	2017	Nerve injury	0.1129
Sri Lanka	1990	Injury to eyes	0.0165
Sri Lanka	1995	Injury to eyes	0.0165
Sri Lanka	2000	Injury to eyes	0.0165
Sri Lanka	2005	Injury to eyes	0.0165
Sri Lanka	2010	Injury to eyes	0.0165
Sri Lanka	2017	Injury to eyes	0.0165
Sri Lanka	1990	Amputation of lower limb, unilateral	0.0869
Sri Lanka	1995	Amputation of lower limb, unilateral	0.079
Sri Lanka	2000	Amputation of lower limb, unilateral	0.0745
Sri Lanka	2005	Amputation of lower limb, unilateral	0.066
Sri Lanka	2010	Amputation of lower limb, unilateral	0.0568
Sri Lanka	2017	Amputation of lower limb, unilateral	0.0454
Sri Lanka	1990	Open wound(s)	0.011
Sri Lanka	1995	Open wound(s)	0.011
Sri Lanka	2000	Open wound(s)	0.011
Sri Lanka	2005	Open wound(s)	0.011
Sri Lanka	2010	Open wound(s)	0.011
Sri Lanka	2017	Open wound(s)	0.011
Sri Lanka	1990	Poisoning requiring urgent care	0.1628
Sri Lanka	1995	Poisoning requiring urgent care	0.1628
Sri Lanka	2000	Poisoning requiring urgent care	0.1628
Sri Lanka	2005	Poisoning requiring urgent care	0.1628
Sri Lanka	2010	Poisoning requiring urgent care	0.1628
Sri Lanka	2017	Poisoning requiring urgent care	0.1628
Sri Lanka	1990	Severe chest injury	0.0474
Sri Lanka	1995	Severe chest injury	0.0474
Sri Lanka	2000	Severe chest injury	0.0474
Sri Lanka	2005	Severe chest injury	0.0474
Sri Lanka	2010	Severe chest injury	0.0474
Sri Lanka	2017	Severe chest injury	0.0474
Sri Lanka	1990	Internal hemorrhage in abdomen and pelvis	0.1143
Sri Lanka	1995	Internal hemorrhage in abdomen and pelvis	0.1143
Sri Lanka	2000	Internal hemorrhage in abdomen and pelvis	0.1143
Sri Lanka	2005	Internal hemorrhage in abdomen and pelvis	0.1143
Sri Lanka	2010	Internal hemorrhage in abdomen and pelvis	0.1143
Sri Lanka	2017	Internal hemorrhage in abdomen and pelvis	0.1143
Sri Lanka	1990	Contusion in any part of the body	0.0075
Sri Lanka	1995	Contusion in any part of the body	0.0075
Sri Lanka	2000	Contusion in any part of the body	0.0075
Sri Lanka	2005	Contusion in any part of the body	0.0075
Sri Lanka	2010	Contusion in any part of the body	0.0075
Sri Lanka	2017	Contusion in any part of the body	0.0075
Sri Lanka	1990	Effect of different environmental factors	0.049
Sri Lanka	1995	Effect of different environmental factors	0.049
Sri Lanka	2000	Effect of different environmental factors	0.049
Sri Lanka	2005	Effect of different environmental factors	0.049
Sri Lanka	2010	Effect of different environmental factors	0.049
Sri Lanka	2017	Effect of different environmental factors	0.049
Sri Lanka	1990	Complications following therapeutic procedures	0.0506
Sri Lanka	1995	Complications following therapeutic procedures	0.0506
Sri Lanka	2000	Complications following therapeutic procedures	0.0506
Sri Lanka	2005	Complications following therapeutic procedures	0.0506
Sri Lanka	2010	Complications following therapeutic procedures	0.0506
Sri Lanka	2017	Complications following therapeutic procedures	0.0506
Sri Lanka	1990	Multiple fractures, dislocations, crashes, wounds, sprains, and strains	0.1806
Sri Lanka	1995	Multiple fractures, dislocations, crashes, wounds, sprains, and strains	0.1603
Sri Lanka	2000	Multiple fractures, dislocations, crashes, wounds, sprains, and strains	0.149
Sri Lanka	2005	Multiple fractures, dislocations, crashes, wounds, sprains, and strains	0.1271
Sri Lanka	2010	Multiple fractures, dislocations, crashes, wounds, sprains, and strains	0.1034
Sri Lanka	2017	Multiple fractures, dislocations, crashes, wounds, sprains, and strains	0.0742
Sri Lanka	1990	Amputation of upper limb, unilateral	0.0672
Sri Lanka	1995	Amputation of upper limb, unilateral	0.0625
Sri Lanka	2000	Amputation of upper limb, unilateral	0.0589
Sri Lanka	2005	Amputation of upper limb, unilateral	0.0549
Sri Lanka	2010	Amputation of upper limb, unilateral	0.0495
Sri Lanka	2017	Amputation of upper limb, unilateral	0.0428
Sri Lanka	1990	Amputation of thumb	0.0114
Sri Lanka	1995	Amputation of thumb	0.0114
Sri Lanka	2000	Amputation of thumb	0.0114
Sri Lanka	2005	Amputation of thumb	0.0114
Sri Lanka	2010	Amputation of thumb	0.0114
Sri Lanka	2017	Amputation of thumb	0.0114
Sri Lanka	1990	Amputation of toe/toes	0.0059
Sri Lanka	1995	Amputation of toe/toes	0.0059
Sri Lanka	2000	Amputation of toe/toes	0.0059
Sri Lanka	2005	Amputation of toe/toes	0.0059
Sri Lanka	2010	Amputation of toe/toes	0.0059
Sri Lanka	2017	Amputation of toe/toes	0.0059
Sri Lanka	1990	Burns, <20% total burned surface area without lower airway burns	0.0163
Sri Lanka	1995	Burns, <20% total burned surface area without lower airway burns	0.0163
Sri Lanka	2000	Burns, <20% total burned surface area without lower airway burns	0.0163
Sri Lanka	2005	Burns, <20% total burned surface area without lower airway burns	0.0163
Sri Lanka	2010	Burns, <20% total burned surface area without lower airway burns	0.0163
Sri Lanka	2017	Burns, <20% total burned surface area without lower airway burns	0.0163
Sri Lanka	1990	Burns, >=20% total burned surface area or >= 10% burned surface area if head/neck or hands/wrist involved w/o lower airway burns	0.249
Sri Lanka	1995	Burns, >=20% total burned surface area or >= 10% burned surface area if head/neck or hands/wrist involved w/o lower airway burns	0.2302
Sri Lanka	2000	Burns, >=20% total burned surface area or >= 10% burned surface area if head/neck or hands/wrist involved w/o lower airway burns	0.2196
Sri Lanka	2005	Burns, >=20% total burned surface area or >= 10% burned surface area if head/neck or hands/wrist involved w/o lower airway burns	0.1993
Sri Lanka	2010	Burns, >=20% total burned surface area or >= 10% burned surface area if head/neck or hands/wrist involved w/o lower airway burns	0.1772
Sri Lanka	2017	Burns, >=20% total burned surface area or >= 10% burned surface area if head/neck or hands/wrist involved w/o lower airway burns	0.1499
Thailand	1990	Amputation of lower limbs, bilateral	0.2264
Thailand	1995	Amputation of lower limbs, bilateral	0.2099
Thailand	2000	Amputation of lower limbs, bilateral	0.1916
Thailand	2005	Amputation of lower limbs, bilateral	0.1719
Thailand	2010	Amputation of lower limbs, bilateral	0.1571
Thailand	2017	Amputation of lower limbs, bilateral	0.1401
Thailand	1990	Lower airway burns	0.2252
Thailand	1995	Lower airway burns	0.2252
Thailand	2000	Lower airway burns	0.2252
Thailand	2005	Lower airway burns	0.2252
Thailand	2010	Lower airway burns	0.2252
Thailand	2017	Lower airway burns	0.2252
Thailand	1990	Dislocation of hip	0.0159
Thailand	1995	Dislocation of hip	0.0159
Thailand	2000	Dislocation of hip	0.0159
Thailand	2005	Dislocation of hip	0.0159
Thailand	2010	Dislocation of hip	0.0159
Thailand	2017	Dislocation of hip	0.0159
Thailand	1990	Dislocation of knee	0.1134
Thailand	1995	Dislocation of knee	0.1134
Thailand	2000	Dislocation of knee	0.1134
Thailand	2005	Dislocation of knee	0.1134
Thailand	2010	Dislocation of knee	0.1134
Thailand	2017	Dislocation of knee	0.1134
Thailand	1990	Dislocation of shoulder	0.062
Thailand	1995	Dislocation of shoulder	0.062
Thailand	2000	Dislocation of shoulder	0.062
Thailand	2005	Dislocation of shoulder	0.062
Thailand	2010	Dislocation of shoulder	0.062
Thailand	2017	Dislocation of shoulder	0.062
Thailand	1990	Muscle and tendon injuries, including sprains and strains lesser dislocations	0.0075
Thailand	1995	Muscle and tendon injuries, including sprains and strains lesser dislocations	0.0075
Thailand	2000	Muscle and tendon injuries, including sprains and strains lesser dislocations	0.0075
Thailand	2005	Muscle and tendon injuries, including sprains and strains lesser dislocations	0.0075
Thailand	2010	Muscle and tendon injuries, including sprains and strains lesser dislocations	0.0075
Thailand	2017	Muscle and tendon injuries, including sprains and strains lesser dislocations	0.0075
Thailand	1990	Fracture of clavicle, scapula, or humerus	0.0349
Thailand	1995	Fracture of clavicle, scapula, or humerus	0.0349
Thailand	2000	Fracture of clavicle, scapula, or humerus	0.0349
Thailand	2005	Fracture of clavicle, scapula, or humerus	0.0349
Thailand	2010	Fracture of clavicle, scapula, or humerus	0.0349
Thailand	2017	Fracture of clavicle, scapula, or humerus	0.0349
Thailand	1990	Fracture of face bones	0.0669
Thailand	1995	Fracture of face bones	0.0669
Thailand	2000	Fracture of face bones	0.0669
Thailand	2005	Fracture of face bones	0.0669
Thailand	2010	Fracture of face bones	0.0669
Thailand	2017	Fracture of face bones	0.0669
Thailand	1990	Fracture of foot bones except ankle	0.0264
Thailand	1995	Fracture of foot bones except ankle	0.0264
Thailand	2000	Fracture of foot bones except ankle	0.0264
Thailand	2005	Fracture of foot bones except ankle	0.0264
Thailand	2010	Fracture of foot bones except ankle	0.0264
Thailand	2017	Fracture of foot bones except ankle	0.0264
Thailand	1990	Fracture of hand (wrist and other distal part of hand)	0.0138
Thailand	1995	Fracture of hand (wrist and other distal part of hand)	0.0138
Thailand	2000	Fracture of hand (wrist and other distal part of hand)	0.0138
Thailand	2005	Fracture of hand (wrist and other distal part of hand)	0.0138
Thailand	2010	Fracture of hand (wrist and other distal part of hand)	0.0138
Thailand	2017	Fracture of hand (wrist and other distal part of hand)	0.0138
Thailand	1990	Fracture of hip	0.1941
Thailand	1995	Fracture of hip	0.1723
Thailand	2000	Fracture of hip	0.1481
Thailand	2005	Fracture of hip	0.122
Thailand	2010	Fracture of hip	0.1025
Thailand	2017	Fracture of hip	0.0799
Thailand	1990	Amputation of upper limbs, bilateral	0.2285
Thailand	1995	Amputation of upper limbs, bilateral	0.206
Thailand	2000	Amputation of upper limbs, bilateral	0.181
Thailand	2005	Amputation of upper limbs, bilateral	0.1541
Thailand	2010	Amputation of upper limbs, bilateral	0.1339
Thailand	2017	Amputation of upper limbs, bilateral	0.1106
Thailand	1990	Fracture of patella, tibia or fibula, or ankle	0.0554
Thailand	1995	Fracture of patella, tibia or fibula, or ankle	0.0554
Thailand	2000	Fracture of patella, tibia or fibula, or ankle	0.0554
Thailand	2005	Fracture of patella, tibia or fibula, or ankle	0.0554
Thailand	2010	Fracture of patella, tibia or fibula, or ankle	0.0554
Thailand	2017	Fracture of patella, tibia or fibula, or ankle	0.0554
Thailand	1990	Fracture of pelvis	0.1823
Thailand	1995	Fracture of pelvis	0.1823
Thailand	2000	Fracture of pelvis	0.1823
Thailand	2005	Fracture of pelvis	0.1823
Thailand	2010	Fracture of pelvis	0.1823
Thailand	2017	Fracture of pelvis	0.1823
Thailand	1990	Fracture of radius and/or ulna	0.0435
Thailand	1995	Fracture of radius and/or ulna	0.0435
Thailand	2000	Fracture of radius and/or ulna	0.0435
Thailand	2005	Fracture of radius and/or ulna	0.0435
Thailand	2010	Fracture of radius and/or ulna	0.0435
Thailand	2017	Fracture of radius and/or ulna	0.0435
Thailand	1990	Fracture of skull	0.0714
Thailand	1995	Fracture of skull	0.0714
Thailand	2000	Fracture of skull	0.0714
Thailand	2005	Fracture of skull	0.0714
Thailand	2010	Fracture of skull	0.0714
Thailand	2017	Fracture of skull	0.0714
Thailand	1990	Fracture of sternum and/or fracture of one or more ribs	0.1027
Thailand	1995	Fracture of sternum and/or fracture of one or more ribs	0.1027
Thailand	2000	Fracture of sternum and/or fracture of one or more ribs	0.1027
Thailand	2005	Fracture of sternum and/or fracture of one or more ribs	0.1027
Thailand	2010	Fracture of sternum and/or fracture of one or more ribs	0.1027
Thailand	2017	Fracture of sternum and/or fracture of one or more ribs	0.1027
Thailand	1990	Fracture of vertebral column	0.1106
Thailand	1995	Fracture of vertebral column	0.1106
Thailand	2000	Fracture of vertebral column	0.1106
Thailand	2005	Fracture of vertebral column	0.1106
Thailand	2010	Fracture of vertebral column	0.1106
Thailand	2017	Fracture of vertebral column	0.1106
Thailand	1990	Fracture of femur, other than femoral neck	0.042
Thailand	1995	Fracture of femur, other than femoral neck	0.042
Thailand	2000	Fracture of femur, other than femoral neck	0.042
Thailand	2005	Fracture of femur, other than femoral neck	0.042
Thailand	2010	Fracture of femur, other than femoral neck	0.042
Thailand	2017	Fracture of femur, other than femoral neck	0.042
Thailand	1990	Minor TBI	0.1319
Thailand	1995	Minor TBI	0.1319
Thailand	2000	Minor TBI	0.1319
Thailand	2005	Minor TBI	0.1319
Thailand	2010	Minor TBI	0.1319
Thailand	2017	Minor TBI	0.1319
Thailand	1990	Moderate TBI	0.1637
Thailand	1995	Moderate TBI	0.1637
Thailand	2000	Moderate TBI	0.1637
Thailand	2005	Moderate TBI	0.1637
Thailand	2010	Moderate TBI	0.1637
Thailand	2017	Moderate TBI	0.1637
Thailand	1990	Amputation of fingers (excluding thumb)	0.0047
Thailand	1995	Amputation of fingers (excluding thumb)	0.0047
Thailand	2000	Amputation of fingers (excluding thumb)	0.0047
Thailand	2005	Amputation of fingers (excluding thumb)	0.0047
Thailand	2010	Amputation of fingers (excluding thumb)	0.0047
Thailand	2017	Amputation of fingers (excluding thumb)	0.0047
Thailand	1990	Spinal cord lesion at neck level	0.459
Thailand	1995	Spinal cord lesion at neck level	0.4514
Thailand	2000	Spinal cord lesion at neck level	0.4431
Thailand	2005	Spinal cord lesion at neck level	0.4341
Thailand	2010	Spinal cord lesion at neck level	0.4273
Thailand	2017	Spinal cord lesion at neck level	0.4195
Thailand	1990	Spinal cord lesion below neck level	0.2773
Thailand	1995	Spinal cord lesion below neck level	0.2613
Thailand	2000	Spinal cord lesion below neck level	0.2437
Thailand	2005	Spinal cord lesion below neck level	0.2246
Thailand	2010	Spinal cord lesion below neck level	0.2104
Thailand	2017	Spinal cord lesion below neck level	0.1939
Thailand	1990	Drowning and nonfatal submersion	0.2471
Thailand	1995	Drowning and nonfatal submersion	0.2471
Thailand	2000	Drowning and nonfatal submersion	0.2471
Thailand	2005	Drowning and nonfatal submersion	0.2471
Thailand	2010	Drowning and nonfatal submersion	0.2471
Thailand	2017	Drowning and nonfatal submersion	0.2471
Thailand	1990	Asphyxiation	0.2471
Thailand	1995	Asphyxiation	0.2471
Thailand	2000	Asphyxiation	0.2471
Thailand	2005	Asphyxiation	0.2471
Thailand	2010	Asphyxiation	0.2471
Thailand	2017	Asphyxiation	0.2471
Thailand	1990	Crush injury	0.1325
Thailand	1995	Crush injury	0.1325
Thailand	2000	Crush injury	0.1325
Thailand	2005	Crush injury	0.1325
Thailand	2010	Crush injury	0.1325
Thailand	2017	Crush injury	0.1325
Thailand	1990	Nerve injury	0.1129
Thailand	1995	Nerve injury	0.1129
Thailand	2000	Nerve injury	0.1129
Thailand	2005	Nerve injury	0.1129
Thailand	2010	Nerve injury	0.1129
Thailand	2017	Nerve injury	0.1129
Thailand	1990	Injury to eyes	0.0165
Thailand	1995	Injury to eyes	0.0165
Thailand	2000	Injury to eyes	0.0165
Thailand	2005	Injury to eyes	0.0165
Thailand	2010	Injury to eyes	0.0165
Thailand	2017	Injury to eyes	0.0165
Thailand	1990	Amputation of lower limb, unilateral	0.0921
Thailand	1995	Amputation of lower limb, unilateral	0.0836
Thailand	2000	Amputation of lower limb, unilateral	0.0742
Thailand	2005	Amputation of lower limb, unilateral	0.064
Thailand	2010	Amputation of lower limb, unilateral	0.0564
Thailand	2017	Amputation of lower limb, unilateral	0.0476
Thailand	1990	Open wound(s)	0.011
Thailand	1995	Open wound(s)	0.011
Thailand	2000	Open wound(s)	0.011
Thailand	2005	Open wound(s)	0.011
Thailand	2010	Open wound(s)	0.011
Thailand	2017	Open wound(s)	0.011
Thailand	1990	Poisoning requiring urgent care	0.1628
Thailand	1995	Poisoning requiring urgent care	0.1628
Thailand	2000	Poisoning requiring urgent care	0.1628
Thailand	2005	Poisoning requiring urgent care	0.1628
Thailand	2010	Poisoning requiring urgent care	0.1628
Thailand	2017	Poisoning requiring urgent care	0.1628
Thailand	1990	Severe chest injury	0.0474
Thailand	1995	Severe chest injury	0.0474
Thailand	2000	Severe chest injury	0.0474
Thailand	2005	Severe chest injury	0.0474
Thailand	2010	Severe chest injury	0.0474
Thailand	2017	Severe chest injury	0.0474
Thailand	1990	Internal hemorrhage in abdomen and pelvis	0.1143
Thailand	1995	Internal hemorrhage in abdomen and pelvis	0.1143
Thailand	2000	Internal hemorrhage in abdomen and pelvis	0.1143
Thailand	2005	Internal hemorrhage in abdomen and pelvis	0.1143
Thailand	2010	Internal hemorrhage in abdomen and pelvis	0.1143
Thailand	2017	Internal hemorrhage in abdomen and pelvis	0.1143
Thailand	1990	Contusion in any part of the body	0.0075
Thailand	1995	Contusion in any part of the body	0.0075
Thailand	2000	Contusion in any part of the body	0.0075
Thailand	2005	Contusion in any part of the body	0.0075
Thailand	2010	Contusion in any part of the body	0.0075
Thailand	2017	Contusion in any part of the body	0.0075
Thailand	1990	Effect of different environmental factors	0.049
Thailand	1995	Effect of different environmental factors	0.049
Thailand	2000	Effect of different environmental factors	0.049
Thailand	2005	Effect of different environmental factors	0.049
Thailand	2010	Effect of different environmental factors	0.049
Thailand	2017	Effect of different environmental factors	0.049
Thailand	1990	Complications following therapeutic procedures	0.0506
Thailand	1995	Complications following therapeutic procedures	0.0506
Thailand	2000	Complications following therapeutic procedures	0.0506
Thailand	2005	Complications following therapeutic procedures	0.0506
Thailand	2010	Complications following therapeutic procedures	0.0506
Thailand	2017	Complications following therapeutic procedures	0.0506
Thailand	1990	Multiple fractures, dislocations, crashes, wounds, sprains, and strains	0.1941
Thailand	1995	Multiple fractures, dislocations, crashes, wounds, sprains, and strains	0.1723
Thailand	2000	Multiple fractures, dislocations, crashes, wounds, sprains, and strains	0.1481
Thailand	2005	Multiple fractures, dislocations, crashes, wounds, sprains, and strains	0.122
Thailand	2010	Multiple fractures, dislocations, crashes, wounds, sprains, and strains	0.1025
Thailand	2017	Multiple fractures, dislocations, crashes, wounds, sprains, and strains	0.0799
Thailand	1990	Amputation of upper limb, unilateral	0.0703
Thailand	1995	Amputation of upper limb, unilateral	0.0653
Thailand	2000	Amputation of upper limb, unilateral	0.0597
Thailand	2005	Amputation of upper limb, unilateral	0.0537
Thailand	2010	Amputation of upper limb, unilateral	0.0493
Thailand	2017	Amputation of upper limb, unilateral	0.0441
Thailand	1990	Amputation of thumb	0.0114
Thailand	1995	Amputation of thumb	0.0114
Thailand	2000	Amputation of thumb	0.0114
Thailand	2005	Amputation of thumb	0.0114
Thailand	2010	Amputation of thumb	0.0114
Thailand	2017	Amputation of thumb	0.0114
Thailand	1990	Amputation of toe/toes	0.0059
Thailand	1995	Amputation of toe/toes	0.0059
Thailand	2000	Amputation of toe/toes	0.0059
Thailand	2005	Amputation of toe/toes	0.0059
Thailand	2010	Amputation of toe/toes	0.0059
Thailand	2017	Amputation of toe/toes	0.0059
Thailand	1990	Burns, <20% total burned surface area without lower airway burns	0.0163
Thailand	1995	Burns, <20% total burned surface area without lower airway burns	0.0163
Thailand	2000	Burns, <20% total burned surface area without lower airway burns	0.0163
Thailand	2005	Burns, <20% total burned surface area without lower airway burns	0.0163
Thailand	2010	Burns, <20% total burned surface area without lower airway burns	0.0163
Thailand	2017	Burns, <20% total burned surface area without lower airway burns	0.0163
Thailand	1990	Burns, >=20% total burned surface area or >= 10% burned surface area if head/neck or hands/wrist involved w/o lower airway burns	0.2616
Thailand	1995	Burns, >=20% total burned surface area or >= 10% burned surface area if head/neck or hands/wrist involved w/o lower airway burns	0.2413
Thailand	2000	Burns, >=20% total burned surface area or >= 10% burned surface area if head/neck or hands/wrist involved w/o lower airway burns	0.2188
Thailand	2005	Burns, >=20% total burned surface area or >= 10% burned surface area if head/neck or hands/wrist involved w/o lower airway burns	0.1945
Thailand	2010	Burns, >=20% total burned surface area or >= 10% burned surface area if head/neck or hands/wrist involved w/o lower airway burns	0.1763
Thailand	2017	Burns, >=20% total burned surface area or >= 10% burned surface area if head/neck or hands/wrist involved w/o lower airway burns	0.1553
Timor-Leste	1990	Amputation of lower limbs, bilateral	0.3012
Timor-Leste	1995	Amputation of lower limbs, bilateral	0.2921
Timor-Leste	2000	Amputation of lower limbs, bilateral	0.2835
Timor-Leste	2005	Amputation of lower limbs, bilateral	0.2671
Timor-Leste	2010	Amputation of lower limbs, bilateral	0.2442

Timor-Leste	2017	Amputation of lower limbs, bilateral	0.2288
Timor-Leste	1990	Lower airway burns	0.2252
Timor-Leste	1995	Lower airway burns	0.2252
Timor-Leste	2000	Lower airway burns	0.2252
Timor-Leste	2005	Lower airway burns	0.2252
Timor-Leste	2010	Lower airway burns	0.2252
Timor-Leste	2017	Lower airway burns	0.2252
Timor-Leste	1990	Dislocation of hip	0.0159
Timor-Leste	1995	Dislocation of hip	0.0159
Timor-Leste	2000	Dislocation of hip	0.0159
Timor-Leste	2005	Dislocation of hip	0.0159
Timor-Leste	2010	Dislocation of hip	0.0159
Timor-Leste	2017	Dislocation of hip	0.0159
Timor-Leste	1990	Dislocation of knee	0.1134
Timor-Leste	1995	Dislocation of knee	0.1134
Timor-Leste	2000	Dislocation of knee	0.1134
Timor-Leste	2005	Dislocation of knee	0.1134
Timor-Leste	2010	Dislocation of knee	0.1134
Timor-Leste	2017	Dislocation of knee	0.1134
Timor-Leste	1990	Dislocation of shoulder	0.062
Timor-Leste	1995	Dislocation of shoulder	0.062
Timor-Leste	2000	Dislocation of shoulder	0.062
Timor-Leste	2005	Dislocation of shoulder	0.062
Timor-Leste	2010	Dislocation of shoulder	0.062
Timor-Leste	2017	Dislocation of shoulder	0.062
Timor-Leste	1990	Muscle and tendon injuries, including sprains and strains lesser dislocations	0.0075
Timor-Leste	1995	Muscle and tendon injuries, including sprains and strains lesser dislocations	0.0075
Timor-Leste	2000	Muscle and tendon injuries, including sprains and strains lesser dislocations	0.0075
Timor-Leste	2005	Muscle and tendon injuries, including sprains and strains lesser dislocations	0.0075
Timor-Leste	2010	Muscle and tendon injuries, including sprains and strains lesser dislocations	0.0075
Timor-Leste	2017	Muscle and tendon injuries, including sprains and strains lesser dislocations	0.0075
Timor-Leste	1990	Fracture of clavicle, scapula, or humerus	0.0349
Timor-Leste	1995	Fracture of clavicle, scapula, or humerus	0.0349
Timor-Leste	2000	Fracture of clavicle, scapula, or humerus	0.0349
Timor-Leste	2005	Fracture of clavicle, scapula, or humerus	0.0349
Timor-Leste	2010	Fracture of clavicle, scapula, or humerus	0.0349
Timor-Leste	2017	Fracture of clavicle, scapula, or humerus	0.0349
Timor-Leste	1990	Fracture of face bones	0.0669
Timor-Leste	1995	Fracture of face bones	0.0669
Timor-Leste	2000	Fracture of face bones	0.0669
Timor-Leste	2005	Fracture of face bones	0.0669
Timor-Leste	2010	Fracture of face bones	0.0669
Timor-Leste	2017	Fracture of face bones	0.0669
Timor-Leste	1990	Fracture of foot bones except ankle	0.0264
Timor-Leste	1995	Fracture of foot bones except ankle	0.0264
Timor-Leste	2000	Fracture of foot bones except ankle	0.0264
Timor-Leste	2005	Fracture of foot bones except ankle	0.0264
Timor-Leste	2010	Fracture of foot bones except ankle	0.0264
Timor-Leste	2017	Fracture of foot bones except ankle	0.0264
Timor-Leste	1990	Fracture of hand (wrist and other distal part of hand)	0.0138
Timor-Leste	1995	Fracture of hand (wrist and other distal part of hand)	0.0138
Timor-Leste	2000	Fracture of hand (wrist and other distal part of hand)	0.0138
Timor-Leste	2005	Fracture of hand (wrist and other distal part of hand)	0.0138
Timor-Leste	2010	Fracture of hand (wrist and other distal part of hand)	0.0138
Timor-Leste	2017	Fracture of hand (wrist and other distal part of hand)	0.0138
Timor-Leste	1990	Fracture of hip	0.2931
Timor-Leste	1995	Fracture of hip	0.281
Timor-Leste	2000	Fracture of hip	0.2696
Timor-Leste	2005	Fracture of hip	0.248
Timor-Leste	2010	Fracture of hip	0.2177
Timor-Leste	2017	Fracture of hip	0.1973
Timor-Leste	1990	Amputation of upper limbs, bilateral	0.3307
Timor-Leste	1995	Amputation of upper limbs, bilateral	0.3183
Timor-Leste	2000	Amputation of upper limbs, bilateral	0.3065
Timor-Leste	2005	Amputation of upper limbs, bilateral	0.2842
Timor-Leste	2010	Amputation of upper limbs, bilateral	0.2529
Timor-Leste	2017	Amputation of upper limbs, bilateral	0.2318
Timor-Leste	1990	Fracture of patella, tibia or fibula, or ankle	0.0554
Timor-Leste	1995	Fracture of patella, tibia or fibula, or ankle	0.0554
Timor-Leste	2000	Fracture of patella, tibia or fibula, or ankle	0.0554
Timor-Leste	2005	Fracture of patella, tibia or fibula, or ankle	0.0554
Timor-Leste	2010	Fracture of patella, tibia or fibula, or ankle	0.0554
Timor-Leste	2017	Fracture of patella, tibia or fibula, or ankle	0.0554
Timor-Leste	1990	Fracture of pelvis	0.1823
Timor-Leste	1995	Fracture of pelvis	0.1823
Timor-Leste	2000	Fracture of pelvis	0.1823
Timor-Leste	2005	Fracture of pelvis	0.1823
Timor-Leste	2010	Fracture of pelvis	0.1823
Timor-Leste	2017	Fracture of pelvis	0.1823
Timor-Leste	1990	Fracture of radius and/or ulna	0.0435
Timor-Leste	1995	Fracture of radius and/or ulna	0.0435
Timor-Leste	2000	Fracture of radius and/or ulna	0.0435
Timor-Leste	2005	Fracture of radius and/or ulna	0.0435
Timor-Leste	2010	Fracture of radius and/or ulna	0.0435
Timor-Leste	2017	Fracture of radius and/or ulna	0.0435
Timor-Leste	1990	Fracture of skull	0.0714
Timor-Leste	1995	Fracture of skull	0.0714
Timor-Leste	2000	Fracture of skull	0.0714
Timor-Leste	2005	Fracture of skull	0.0714
Timor-Leste	2010	Fracture of skull	0.0714
Timor-Leste	2017	Fracture of skull	0.0714
Timor-Leste	1990	Fracture of sternum and/or fracture of one or more ribs	0.1027
Timor-Leste	1995	Fracture of sternum and/or fracture of one or more ribs	0.1027
Timor-Leste	2000	Fracture of sternum and/or fracture of one or more ribs	0.1027
Timor-Leste	2005	Fracture of sternum and/or fracture of one or more ribs	0.1027
Timor-Leste	2010	Fracture of sternum and/or fracture of one or more ribs	0.1027
Timor-Leste	2017	Fracture of sternum and/or fracture of one or more ribs	0.1027
Timor-Leste	1990	Fracture of vertebral column	0.1106
Timor-Leste	1995	Fracture of vertebral column	0.1106
Timor-Leste	2000	Fracture of vertebral column	0.1106
Timor-Leste	2005	Fracture of vertebral column	0.1106
Timor-Leste	2010	Fracture of vertebral column	0.1106
Timor-Leste	2017	Fracture of vertebral column	0.1106
Timor-Leste	1990	Fracture of femur, other than femoral neck	0.042
Timor-Leste	1995	Fracture of femur, other than femoral neck	0.042
Timor-Leste	2000	Fracture of femur, other than femoral neck	0.042
Timor-Leste	2005	Fracture of femur, other than femoral neck	0.042
Timor-Leste	2010	Fracture of femur, other than femoral neck	0.042
Timor-Leste	2017	Fracture of femur, other than femoral neck	0.042
Timor-Leste	1990	Minor TBI	0.1319
Timor-Leste	1995	Minor TBI	0.1319
Timor-Leste	2000	Minor TBI	0.1319
Timor-Leste	2005	Minor TBI	0.1319
Timor-Leste	2010	Minor TBI	0.1319
Timor-Leste	2017	Minor TBI	0.1319
Timor-Leste	1990	Moderate TBI	0.1637
Timor-Leste	1995	Moderate TBI	0.1637
Timor-Leste	2000	Moderate TBI	0.1637
Timor-Leste	2005	Moderate TBI	0.1637
Timor-Leste	2010	Moderate TBI	0.1637
Timor-Leste	2017	Moderate TBI	0.1637
Timor-Leste	1990	Amputation of fingers (excluding thumb)	0.0047
Timor-Leste	1995	Amputation of fingers (excluding thumb)	0.0047
Timor-Leste	2000	Amputation of fingers (excluding thumb)	0.0047
Timor-Leste	2005	Amputation of fingers (excluding thumb)	0.0047
Timor-Leste	2010	Amputation of fingers (excluding thumb)	0.0047
Timor-Leste	2017	Amputation of fingers (excluding thumb)	0.0047
Timor-Leste	1990	Spinal cord lesion at neck level	0.4932
Timor-Leste	1995	Spinal cord lesion at neck level	0.4891
Timor-Leste	2000	Spinal cord lesion at neck level	0.4851
Timor-Leste	2005	Spinal cord lesion at neck level	0.4776
Timor-Leste	2010	Spinal cord lesion at neck level	0.4672
Timor-Leste	2017	Spinal cord lesion at neck level	0.4601
Timor-Leste	1990	Spinal cord lesion below neck level	0.3495
Timor-Leste	1995	Spinal cord lesion below neck level	0.3407
Timor-Leste	2000	Spinal cord lesion below neck level	0.3323
Timor-Leste	2005	Spinal cord lesion below neck level	0.3166
Timor-Leste	2010	Spinal cord lesion below neck level	0.2944
Timor-Leste	2017	Spinal cord lesion below neck level	0.2796
Timor-Leste	1990	Drowning and nonfatal submersion	0.2471
Timor-Leste	1995	Drowning and nonfatal submersion	0.2471
Timor-Leste	2000	Drowning and nonfatal submersion	0.2471
Timor-Leste	2005	Drowning and nonfatal submersion	0.2471
Timor-Leste	2010	Drowning and nonfatal submersion	0.2471
Timor-Leste	2017	Drowning and nonfatal submersion	0.2471
Timor-Leste	1990	Asphyxiation	0.2471
Timor-Leste	1995	Asphyxiation	0.2471
Timor-Leste	2000	Asphyxiation	0.2471
Timor-Leste	2005	Asphyxiation	0.2471
Timor-Leste	2010	Asphyxiation	0.2471
Timor-Leste	2017	Asphyxiation	0.2471
Timor-Leste	1990	Crush injury	0.1325
Timor-Leste	1995	Crush injury	0.1325
Timor-Leste	2000	Crush injury	0.1325
Timor-Leste	2005	Crush injury	0.1325
Timor-Leste	2010	Crush injury	0.1325
Timor-Leste	2017	Crush injury	0.1325
Timor-Leste	1990	Nerve injury	0.1129
Timor-Leste	1995	Nerve injury	0.1129
Timor-Leste	2000	Nerve injury	0.1129
Timor-Leste	2005	Nerve injury	0.1129
Timor-Leste	2010	Nerve injury	0.1129
Timor-Leste	2017	Nerve injury	0.1129
Timor-Leste	1990	Injury to eyes	0.0165
Timor-Leste	1995	Injury to eyes	0.0165
Timor-Leste	2000	Injury to eyes	0.0165
Timor-Leste	2005	Injury to eyes	0.0165
Timor-Leste	2010	Injury to eyes	0.0165
Timor-Leste	2017	Injury to eyes	0.0165
Timor-Leste	1990	Amputation of lower limb, unilateral	0.1307
Timor-Leste	1995	Amputation of lower limb, unilateral	0.126
Timor-Leste	2000	Amputation of lower limb, unilateral	0.1216
Timor-Leste	2005	Amputation of lower limb, unilateral	0.1131
Timor-Leste	2010	Amputation of lower limb, unilateral	0.1013
Timor-Leste	2017	Amputation of lower limb, unilateral	0.0934
Timor-Leste	1990	Open wound(s)	0.011
Timor-Leste	1995	Open wound(s)	0.011
Timor-Leste	2000	Open wound(s)	0.011
Timor-Leste	2005	Open wound(s)	0.011
Timor-Leste	2010	Open wound(s)	0.011
Timor-Leste	2017	Open wound(s)	0.011
Timor-Leste	1990	Poisoning requiring urgent care	0.1628
Timor-Leste	1995	Poisoning requiring urgent care	0.1628
Timor-Leste	2000	Poisoning requiring urgent care	0.1628
Timor-Leste	2005	Poisoning requiring urgent care	0.1628
Timor-Leste	2010	Poisoning requiring urgent care	0.1628
Timor-Leste	2017	Poisoning requiring urgent care	0.1628
Timor-Leste	1990	Severe chest injury	0.0474
Timor-Leste	1995	Severe chest injury	0.0474
Timor-Leste	2000	Severe chest injury	0.0474
Timor-Leste	2005	Severe chest injury	0.0474
Timor-Leste	2010	Severe chest injury	0.0474
Timor-Leste	2017	Severe chest injury	0.0474
Timor-Leste	1990	Internal hemorrhage in abdomen and pelvis	0.1143
Timor-Leste	1995	Internal hemorrhage in abdomen and pelvis	0.1143
Timor-Leste	2000	Internal hemorrhage in abdomen and pelvis	0.1143
Timor-Leste	2005	Internal hemorrhage in abdomen and pelvis	0.1143
Timor-Leste	2010	Internal hemorrhage in abdomen and pelvis	0.1143
Timor-Leste	2017	Internal hemorrhage in abdomen and pelvis	0.1143
Timor-Leste	1990	Contusion in any part of the body	0.0075
Timor-Leste	1995	Contusion in any part of the body	0.0075
Timor-Leste	2000	Contusion in any part of the body	0.0075
Timor-Leste	2005	Contusion in any part of the body	0.0075
Timor-Leste	2010	Contusion in any part of the body	0.0075
Timor-Leste	2017	Contusion in any part of the body	0.0075
Timor-Leste	1990	Effect of different environmental factors	0.049
Timor-Leste	1995	Effect of different environmental factors	0.049
Timor-Leste	2000	Effect of different environmental factors	0.049
Timor-Leste	2005	Effect of different environmental factors	0.049
Timor-Leste	2010	Effect of different environmental factors	0.049
Timor-Leste	2017	Effect of different environmental factors	0.049
Timor-Leste	1990	Complications following therapeutic procedures	0.0506
Timor-Leste	1995	Complications following therapeutic procedures	0.0506
Timor-Leste	2000	Complications following therapeutic procedures	0.0506
Timor-Leste	2005	Complications following therapeutic procedures	0.0506
Timor-Leste	2010	Complications following therapeutic procedures	0.0506
Timor-Leste	2017	Complications following therapeutic procedures	0.0506
Timor-Leste	1990	Multiple fractures, dislocations, crashes, wounds, sprains, and strains	0.2931
Timor-Leste	1995	Multiple fractures, dislocations, crashes, wounds, sprains, and strains	0.281
Timor-Leste	2000	Multiple fractures, dislocations, crashes, wounds, sprains, and strains	0.2696
Timor-Leste	2005	Multiple fractures, dislocations, crashes, wounds, sprains, and strains	0.248
Timor-Leste	2010	Multiple fractures, dislocations, crashes, wounds, sprains, and strains	0.2177
Timor-Leste	2017	Multiple fractures, dislocations, crashes, wounds, sprains, and strains	0.1973
Timor-Leste	1990	Amputation of upper limb, unilateral	0.093
Timor-Leste	1995	Amputation of upper limb, unilateral	0.0902
Timor-Leste	2000	Amputation of upper limb, unilateral	0.0876
Timor-Leste	2005	Amputation of upper limb, unilateral	0.0827
Timor-Leste	2010	Amputation of upper limb, unilateral	0.0757
Timor-Leste	2017	Amputation of upper limb, unilateral	0.071
Timor-Leste	1990	Amputation of thumb	0.0114
Timor-Leste	1995	Amputation of thumb	0.0114
Timor-Leste	2000	Amputation of thumb	0.0114
Timor-Leste	2005	Amputation of thumb	0.0114
Timor-Leste	2010	Amputation of thumb	0.0114
Timor-Leste	2017	Amputation of thumb	0.0114
Timor-Leste	1990	Amputation of toe/toes	0.0059
Timor-Leste	1995	Amputation of toe/toes	0.0059
Timor-Leste	2000	Amputation of toe/toes	0.0059
Timor-Leste	2005	Amputation of toe/toes	0.0059
Timor-Leste	2010	Amputation of toe/toes	0.0059
Timor-Leste	2017	Amputation of toe/toes	0.0059
Timor-Leste	1990	Burns, <20% total burned surface area without lower airway burns	0.0163
Timor-Leste	1995	Burns, <20% total burned surface area without lower airway burns	0.0163
Timor-Leste	2000	Burns, <20% total burned surface area without lower airway burns	0.0163
Timor-Leste	2005	Burns, <20% total burned surface area without lower airway burns	0.0163
Timor-Leste	2010	Burns, <20% total burned surface area without lower airway burns	0.0163
Timor-Leste	2017	Burns, <20% total burned surface area without lower airway burns	0.0163
Timor-Leste	1990	Burns, >=20% total burned surface area or >= 10% burned surface area if head/neck or hands/wrist involved w/o lower airway burns	0.3538
Timor-Leste	1995	Burns, >=20% total burned surface area or >= 10% burned surface area if head/neck or hands/wrist involved w/o lower airway burns	0.3426
Timor-Leste	2000	Burns, >=20% total burned surface area or >= 10% burned surface area if head/neck or hands/wrist involved w/o lower airway burns	0.3319
Timor-Leste	2005	Burns, >=20% total burned surface area or >= 10% burned surface area if head/neck or hands/wrist involved w/o lower airway burns	0.3118
Timor-Leste	2010	Burns, >=20% total burned surface area or >= 10% burned surface area if head/neck or hands/wrist involved w/o lower airway burns	0.2836
Timor-Leste	2017	Burns, >=20% total burned surface area or >= 10% burned surface area if head/neck or hands/wrist involved w/o lower airway burns	0.2646
Socialist Republic of Viet Nam	1990	Amputation of lower limbs, bilateral	0.2529
Socialist Republic of Viet Nam	1995	Amputation of lower limbs, bilateral	0.2396
Socialist Republic of Viet Nam	2000	Amputation of lower limbs, bilateral	0.225
Socialist Republic of Viet Nam	2005	Amputation of lower limbs, bilateral	0.2077
Socialist Republic of Viet Nam	2010	Amputation of lower limbs, bilateral	0.1916
Socialist Republic of Viet Nam	2017	Amputation of lower limbs, bilateral	0.1701
Socialist Republic of Viet Nam	1990	Lower airway burns	0.2252
Socialist Republic of Viet Nam	1995	Lower airway burns	0.2252
Socialist Republic of Viet Nam	2000	Lower airway burns	0.2252
Socialist Republic of Viet Nam	2005	Lower airway burns	0.2252
Socialist Republic of Viet Nam	2010	Lower airway burns	0.2252
Socialist Republic of Viet Nam	2017	Lower airway burns	0.2252
Socialist Republic of Viet Nam	1990	Dislocation of hip	0.0159
Socialist Republic of Viet Nam	1995	Dislocation of hip	0.0159
Socialist Republic of Viet Nam	2000	Dislocation of hip	0.0159
Socialist Republic of Viet Nam	2005	Dislocation of hip	0.0159
Socialist Republic of Viet Nam	2010	Dislocation of hip	0.0159
Socialist Republic of Viet Nam	2017	Dislocation of hip	0.0159
Socialist Republic of Viet Nam	1990	Dislocation of knee	0.1134
Socialist Republic of Viet Nam	1995	Dislocation of knee	0.1134
Socialist Republic of Viet Nam	2000	Dislocation of knee	0.1134
Socialist Republic of Viet Nam	2005	Dislocation of knee	0.1134
Socialist Republic of Viet Nam	2010	Dislocation of knee	0.1134
Socialist Republic of Viet Nam	2017	Dislocation of knee	0.1134
Socialist Republic of Viet Nam	1990	Dislocation of shoulder	0.062
Socialist Republic of Viet Nam	1995	Dislocation of shoulder	0.062
Socialist Republic of Viet Nam	2000	Dislocation of shoulder	0.062
Socialist Republic of Viet Nam	2005	Dislocation of shoulder	0.062
Socialist Republic of Viet Nam	2010	Dislocation of shoulder	0.062
Socialist Republic of Viet Nam	2017	Dislocation of shoulder	0.062
Socialist Republic of Viet Nam	1990	Muscle and tendon injuries, including sprains and strains lesser dislocations	0.0075
Socialist Republic of Viet Nam	1995	Muscle and tendon injuries, including sprains and strains lesser dislocations	0.0075
Socialist Republic of Viet Nam	2000	Muscle and tendon injuries, including sprains and strains lesser dislocations	0.0075
Socialist Republic of Viet Nam	2005	Muscle and tendon injuries, including sprains and strains lesser dislocations	0.0075
Socialist Republic of Viet Nam	2010	Muscle and tendon injuries, including sprains and strains lesser dislocations	0.0075
Socialist Republic of Viet Nam	2017	Muscle and tendon injuries, including sprains and strains lesser dislocations	0.0075
Socialist Republic of Viet Nam	1990	Fracture of clavicle, scapula, or humerus	0.0349
Socialist Republic of Viet Nam	1995	Fracture of clavicle, scapula, or humerus	0.0349
Socialist Republic of Viet Nam	2000	Fracture of clavicle, scapula, or humerus	0.0349
Socialist Republic of Viet Nam	2005	Fracture of clavicle, scapula, or humerus	0.0349
Socialist Republic of Viet Nam	2010	Fracture of clavicle, scapula, or humerus	0.0349
Socialist Republic of Viet Nam	2017	Fracture of clavicle, scapula, or humerus	0.0349
Socialist Republic of Viet Nam	1990	Fracture of face bones	0.0669
Socialist Republic of Viet Nam	1995	Fracture of face bones	0.0669
Socialist Republic of Viet Nam	2000	Fracture of face bones	0.0669
Socialist Republic of Viet Nam	2005	Fracture of face bones	0.0669
Socialist Republic of Viet Nam	2010	Fracture of face bones	0.0669
Socialist Republic of Viet Nam	2017	Fracture of face bones	0.0669
Socialist Republic of Viet Nam	1990	Fracture of foot bones except ankle	0.0264
Socialist Republic of Viet Nam	1995	Fracture of foot bones except ankle	0.0264
Socialist Republic of Viet Nam	2000	Fracture of foot bones except ankle	0.0264
Socialist Republic of Viet Nam	2005	Fracture of foot bones except ankle	0.0264
Socialist Republic of Viet Nam	2010	Fracture of foot bones except ankle	0.0264
Socialist Republic of Viet Nam	2017	Fracture of foot bones except ankle	0.0264
Socialist Republic of Viet Nam	1990	Fracture of hand (wrist and other distal part of hand)	0.0138
Socialist Republic of Viet Nam	1995	Fracture of hand (wrist and other distal part of hand)	0.0138
Socialist Republic of Viet Nam	2000	Fracture of hand (wrist and other distal part of hand)	0.0138
Socialist Republic of Viet Nam	2005	Fracture of hand (wrist and other distal part of hand)	0.0138
Socialist Republic of Viet Nam	2010	Fracture of hand (wrist and other distal part of hand)	0.0138
Socialist Republic of Viet Nam	2017	Fracture of hand (wrist and other distal part of hand)	0.0138
Socialist Republic of Viet Nam	1990	Fracture of hip	0.2291
Socialist Republic of Viet Nam	1995	Fracture of hip	0.2116
Socialist Republic of Viet Nam	2000	Fracture of hip	0.1922
Socialist Republic of Viet Nam	2005	Fracture of hip	0.1694
Socialist Republic of Viet Nam	2010	Fracture of hip	0.148
Socialist Republic of Viet Nam	2017	Fracture of hip	0.1197
Socialist Republic of Viet Nam	1990	Amputation of upper limbs, bilateral	0.2647
Socialist Republic of Viet Nam	1995	Amputation of upper limbs, bilateral	0.2465
Socialist Republic of Viet Nam	2000	Amputation of upper limbs, bilateral	0.2266
Socialist Republic of Viet Nam	2005	Amputation of upper limbs, bilateral	0.203
Socialist Republic of Viet Nam	2010	Amputation of upper limbs, bilateral	0.1809
Socialist Republic of Viet Nam	2017	Amputation of upper limbs, bilateral	0.1517
Socialist Republic of Viet Nam	1990	Fracture of patella, tibia or fibula, or ankle	0.0554
Socialist Republic of Viet Nam	1995	Fracture of patella, tibia or fibula, or ankle	0.0554
Socialist Republic of Viet Nam	2000	Fracture of patella, tibia or fibula, or ankle	0.0554
Socialist Republic of Viet Nam	2005	Fracture of patella, tibia or fibula, or ankle	0.0554
Socialist Republic of Viet Nam	2010	Fracture of patella, tibia or fibula, or ankle	0.0554
Socialist Republic of Viet Nam	2017	Fracture of patella, tibia or fibula, or ankle	0.0554
Socialist Republic of Viet Nam	1990	Fracture of pelvis	0.1823
Socialist Republic of Viet Nam	1995	Fracture of pelvis	0.1823
Socialist Republic of Viet Nam	2000	Fracture of pelvis	0.1823
Socialist Republic of Viet Nam	2005	Fracture of pelvis	0.1823
Socialist Republic of Viet Nam	2010	Fracture of pelvis	0.1823
Socialist Republic of Viet Nam	2017	Fracture of pelvis	0.1823
Socialist Republic of Viet Nam	1990	Fracture of radius and/or ulna	0.0435
Socialist Republic of Viet Nam	1995	Fracture of radius and/or ulna	0.0435
Socialist Republic of Viet Nam	2000	Fracture of radius and/or ulna	0.0435
Socialist Republic of Viet Nam	2005	Fracture of radius and/or ulna	0.0435
Socialist Republic of Viet Nam	2010	Fracture of radius and/or ulna	0.0435
Socialist Republic of Viet Nam	2017	Fracture of radius and/or ulna	0.0435
Socialist Republic of Viet Nam	1990	Fracture of skull	0.0714
Socialist Republic of Viet Nam	1995	Fracture of skull	0.0714
Socialist Republic of Viet Nam	2000	Fracture of skull	0.0714
Socialist Republic of Viet Nam	2005	Fracture of skull	0.0714
Socialist Republic of Viet Nam	2010	Fracture of skull	0.0714
Socialist Republic of Viet Nam	2017	Fracture of skull	0.0714
Socialist Republic of Viet Nam	1990	Fracture of sternum and/or fracture of one or more ribs	0.1027
Socialist Republic of Viet Nam	1995	Fracture of sternum and/or fracture of one or more ribs	0.1027
Socialist Republic of Viet Nam	2000	Fracture of sternum and/or fracture of one or more ribs	0.1027
Socialist Republic of Viet Nam	2005	Fracture of sternum and/or fracture of one or more ribs	0.1027
Socialist Republic of Viet Nam	2010	Fracture of sternum and/or fracture of one or more ribs	0.1027
Socialist Republic of Viet Nam	2017	Fracture of sternum and/or fracture of one or more ribs	0.1027
Socialist Republic of Viet Nam	1990	Fracture of vertebral column	0.1106
Socialist Republic of Viet Nam	1995	Fracture of vertebral column	0.1106
Socialist Republic of Viet Nam	2000	Fracture of vertebral column	0.1106
Socialist Republic of Viet Nam	2005	Fracture of vertebral column	0.1106
Socialist Republic of Viet Nam	2010	Fracture of vertebral column	0.1106
Socialist Republic of Viet Nam	2017	Fracture of vertebral column	0.1106
Socialist Republic of Viet Nam	1990	Fracture of femur, other than femoral neck	0.042
Socialist Republic of Viet Nam	1995	Fracture of femur, other than femoral neck	0.042
Socialist Republic of Viet Nam	2000	Fracture of femur, other than femoral neck	0.042
Socialist Republic of Viet Nam	2005	Fracture of femur, other than femoral neck	0.042
Socialist Republic of Viet Nam	2010	Fracture of femur, other than femoral neck	0.042
Socialist Republic of Viet Nam	2017	Fracture of femur, other than femoral neck	0.042
Socialist Republic of Viet Nam	1990	Minor TBI	0.1319
Socialist Republic of Viet Nam	1995	Minor TBI	0.1319
Socialist Republic of Viet Nam	2000	Minor TBI	0.1319
Socialist Republic of Viet Nam	2005	Minor TBI	0.1319
Socialist Republic of Viet Nam	2010	Minor TBI	0.1319
Socialist Republic of Viet Nam	2017	Minor TBI	0.1319
Socialist Republic of Viet Nam	1990	Moderate TBI	0.1637
Socialist Republic of Viet Nam	1995	Moderate TBI	0.1637
Socialist Republic of Viet Nam	2000	Moderate TBI	0.1637
Socialist Republic of Viet Nam	2005	Moderate TBI	0.1637
Socialist Republic of Viet Nam	2010	Moderate TBI	0.1637
Socialist Republic of Viet Nam	2017	Moderate TBI	0.1637
Socialist Republic of Viet Nam	1990	Amputation of fingers (excluding thumb)	0.0047
Socialist Republic of Viet Nam	1995	Amputation of fingers (excluding thumb)	0.0047
Socialist Republic of Viet Nam	2000	Amputation of fingers (excluding thumb)	0.0047
Socialist Republic of Viet Nam	2005	Amputation of fingers (excluding thumb)	0.0047
Socialist Republic of Viet Nam	2010	Amputation of fingers (excluding thumb)	0.0047
Socialist Republic of Viet Nam	2017	Amputation of fingers (excluding thumb)	0.0047
Socialist Republic of Viet Nam	1990	Spinal cord lesion at neck level	0.4711
Socialist Republic of Viet Nam	1995	Spinal cord lesion at neck level	0.465
Socialist Republic of Viet Nam	2000	Spinal cord lesion at neck level	0.4584
Socialist Republic of Viet Nam	2005	Spinal cord lesion at neck level	0.4504
Socialist Republic of Viet Nam	2010	Spinal cord lesion at neck level	0.4431
Socialist Republic of Viet Nam	2017	Spinal cord lesion at neck level	0.4333
Socialist Republic of Viet Nam	1990	Spinal cord lesion below neck level	0.3028
Socialist Republic of Viet Nam	1995	Spinal cord lesion below neck level	0.29
Socialist Republic of Viet Nam	2000	Spinal cord lesion below neck level	0.2759
Socialist Republic of Viet Nam	2005	Spinal cord lesion below neck level	0.2592
Socialist Republic of Viet Nam	2010	Spinal cord lesion below neck level	0.2436
Socialist Republic of Viet Nam	2017	Spinal cord lesion below neck level	0.2229
Socialist Republic of Viet Nam	1990	Drowning and nonfatal submersion	0.2471
Socialist Republic of Viet Nam	1995	Drowning and nonfatal submersion	0.2471
Socialist Republic of Viet Nam	2000	Drowning and nonfatal submersion	0.2471
Socialist Republic of Viet Nam	2005	Drowning and nonfatal submersion	0.2471
Socialist Republic of Viet Nam	2010	Drowning and nonfatal submersion	0.2471
Socialist Republic of Viet Nam	2017	Drowning and nonfatal submersion	0.2471
Socialist Republic of Viet Nam	1990	Asphyxiation	0.2471
Socialist Republic of Viet Nam	1995	Asphyxiation	0.2471
Socialist Republic of Viet Nam	2000	Asphyxiation	0.2471
Socialist Republic of Viet Nam	2005	Asphyxiation	0.2471
Socialist Republic of Viet Nam	2010	Asphyxiation	0.2471
Socialist Republic of Viet Nam	2017	Asphyxiation	0.2471
Socialist Republic of Viet Nam	1990	Crush injury	0.1325
Socialist Republic of Viet Nam	1995	Crush injury	0.1325
Socialist Republic of Viet Nam	2000	Crush injury	0.1325
Socialist Republic of Viet Nam	2005	Crush injury	0.1325
Socialist Republic of Viet Nam	2010	Crush injury	0.1325
Socialist Republic of Viet Nam	2017	Crush injury	0.1325
Socialist Republic of Viet Nam	1990	Nerve injury	0.1129
Socialist Republic of Viet Nam	1995	Nerve injury	0.1129
Socialist Republic of Viet Nam	2000	Nerve injury	0.1129
Socialist Republic of Viet Nam	2005	Nerve injury	0.1129
Socialist Republic of Viet Nam	2010	Nerve injury	0.1129
Socialist Republic of Viet Nam	2017	Nerve injury	0.1129
Socialist Republic of Viet Nam	1990	Injury to eyes	0.0165
Socialist Republic of Viet Nam	1995	Injury to eyes	0.0165
Socialist Republic of Viet Nam	2000	Injury to eyes	0.0165
Socialist Republic of Viet Nam	2005	Injury to eyes	0.0165
Socialist Republic of Viet Nam	2010	Injury to eyes	0.0165
Socialist Republic of Viet Nam	2017	Injury to eyes	0.0165
Socialist Republic of Viet Nam	1990	Amputation of lower limb, unilateral	0.1058
Socialist Republic of Viet Nam	1995	Amputation of lower limb, unilateral	0.0989
Socialist Republic of Viet Nam	2000	Amputation of lower limb, unilateral	0.0914
Socialist Republic of Viet Nam	2005	Amputation of lower limb, unilateral	0.0825

Socialist Republic of Viet Nam	2010	Amputation of lower limb, unilateral	0.0742
Socialist Republic of Viet Nam	2017	Amputation of lower limb, unilateral	0.0631
Socialist Republic of Viet Nam	1990	Open wound(s)	0.011
Socialist Republic of Viet Nam	1995	Open wound(s)	0.011
Socialist Republic of Viet Nam	2000	Open wound(s)	0.011
Socialist Republic of Viet Nam	2005	Open wound(s)	0.011
Socialist Republic of Viet Nam	2010	Open wound(s)	0.011
Socialist Republic of Viet Nam	2017	Open wound(s)	0.011
Socialist Republic of Viet Nam	1990	Poisoning requiring urgent care	0.1628
Socialist Republic of Viet Nam	1995	Poisoning requiring urgent care	0.1628
Socialist Republic of Viet Nam	2000	Poisoning requiring urgent care	0.1628
Socialist Republic of Viet Nam	2005	Poisoning requiring urgent care	0.1628
Socialist Republic of Viet Nam	2010	Poisoning requiring urgent care	0.1628
Socialist Republic of Viet Nam	2017	Poisoning requiring urgent care	0.1628
Socialist Republic of Viet Nam	1990	Severe chest injury	0.0474
Socialist Republic of Viet Nam	1995	Severe chest injury	0.0474
Socialist Republic of Viet Nam	2000	Severe chest injury	0.0474
Socialist Republic of Viet Nam	2005	Severe chest injury	0.0474
Socialist Republic of Viet Nam	2010	Severe chest injury	0.0474
Socialist Republic of Viet Nam	2017	Severe chest injury	0.0474
Socialist Republic of Viet Nam	1990	Internal hemorrhage in abdomen and pelvis	0.1143
Socialist Republic of Viet Nam	1995	Internal hemorrhage in abdomen and pelvis	0.1143
Socialist Republic of Viet Nam	2000	Internal hemorrhage in abdomen and pelvis	0.1143
Socialist Republic of Viet Nam	2005	Internal hemorrhage in abdomen and pelvis	0.1143
Socialist Republic of Viet Nam	2010	Internal hemorrhage in abdomen and pelvis	0.1143
Socialist Republic of Viet Nam	2017	Internal hemorrhage in abdomen and pelvis	0.1143
Socialist Republic of Viet Nam	1990	Contusion in any part of the body	0.0075
Socialist Republic of Viet Nam	1995	Contusion in any part of the body	0.0075
Socialist Republic of Viet Nam	2000	Contusion in any part of the body	0.0075
Socialist Republic of Viet Nam	2005	Contusion in any part of the body	0.0075
Socialist Republic of Viet Nam	2010	Contusion in any part of the body	0.0075
Socialist Republic of Viet Nam	2017	Contusion in any part of the body	0.0075
Socialist Republic of Viet Nam	1990	Effect of different environmental factors	0.049
Socialist Republic of Viet Nam	1995	Effect of different environmental factors	0.049
Socialist Republic of Viet Nam	2000	Effect of different environmental factors	0.049
Socialist Republic of Viet Nam	2005	Effect of different environmental factors	0.049
Socialist Republic of Viet Nam	2010	Effect of different environmental factors	0.049
Socialist Republic of Viet Nam	2017	Effect of different environmental factors	0.049
Socialist Republic of Viet Nam	1990	Complications following therapeutic procedures	0.0506
Socialist Republic of Viet Nam	1995	Complications following therapeutic procedures	0.0506
Socialist Republic of Viet Nam	2000	Complications following therapeutic procedures	0.0506
Socialist Republic of Viet Nam	2005	Complications following therapeutic procedures	0.0506
Socialist Republic of Viet Nam	2010	Complications following therapeutic procedures	0.0506
Socialist Republic of Viet Nam	2017	Complications following therapeutic procedures	0.0506
Socialist Republic of Viet Nam	1990	Multiple fractures, dislocations, crashes, wounds, sprains, and strains	0.2291
Socialist Republic of Viet Nam	1995	Multiple fractures, dislocations, crashes, wounds, sprains, and strains	0.2116
Socialist Republic of Viet Nam	2000	Multiple fractures, dislocations, crashes, wounds, sprains, and strains	0.1922
Socialist Republic of Viet Nam	2005	Multiple fractures, dislocations, crashes, wounds, sprains, and strains	0.1694
Socialist Republic of Viet Nam	2010	Multiple fractures, dislocations, crashes, wounds, sprains, and strains	0.148
Socialist Republic of Viet Nam	2017	Multiple fractures, dislocations, crashes, wounds, sprains, and strains	0.1197
Socialist Republic of Viet Nam	1990	Amputation of upper limb, unilateral	0.0783
Socialist Republic of Viet Nam	1995	Amputation of upper limb, unilateral	0.0743
Socialist Republic of Viet Nam	2000	Amputation of upper limb, unilateral	0.0699
Socialist Republic of Viet Nam	2005	Amputation of upper limb, unilateral	0.0646
Socialist Republic of Viet Nam	2010	Amputation of upper limb, unilateral	0.0597
Socialist Republic of Viet Nam	2017	Amputation of upper limb, unilateral	0.0532
Socialist Republic of Viet Nam	1990	Amputation of thumb	0.0114
Socialist Republic of Viet Nam	1995	Amputation of thumb	0.0114
Socialist Republic of Viet Nam	2000	Amputation of thumb	0.0114
Socialist Republic of Viet Nam	2005	Amputation of thumb	0.0114
Socialist Republic of Viet Nam	2010	Amputation of thumb	0.0114
Socialist Republic of Viet Nam	2017	Amputation of thumb	0.0114
Socialist Republic of Viet Nam	1990	Amputation of toe/toes	0.0059
Socialist Republic of Viet Nam	1995	Amputation of toe/toes	0.0059
Socialist Republic of Viet Nam	2000	Amputation of toe/toes	0.0059
Socialist Republic of Viet Nam	2005	Amputation of toe/toes	0.0059
Socialist Republic of Viet Nam	2010	Amputation of toe/toes	0.0059
Socialist Republic of Viet Nam	2017	Amputation of toe/toes	0.0059
Socialist Republic of Viet Nam	1990	Burns, <20% total burned surface area without lower airway burns	0.0163
Socialist Republic of Viet Nam	1995	Burns, <20% total burned surface area without lower airway burns	0.0163
Socialist Republic of Viet Nam	2000	Burns, <20% total burned surface area without lower airway burns	0.0163
Socialist Republic of Viet Nam	2005	Burns, <20% total burned surface area without lower airway burns	0.0163
Socialist Republic of Viet Nam	2010	Burns, <20% total burned surface area without lower airway burns	0.0163
Socialist Republic of Viet Nam	2017	Burns, <20% total burned surface area without lower airway burns	0.0163
Socialist Republic of Viet Nam	1990	Burns, >=20% total burned surface area or >= 10% burned surface area if head/neck or hands/wrist involved w/o lower airway burns	0.2942
Socialist Republic of Viet Nam	1995	Burns, >=20% total burned surface area or >= 10% burned surface area if head/neck or hands/wrist involved w/o lower airway burns	0.2779
Socialist Republic of Viet Nam	2000	Burns, >=20% total burned surface area or >= 10% burned surface area if head/neck or hands/wrist involved w/o lower airway burns	0.2599
Socialist Republic of Viet Nam	2005	Burns, >=20% total burned surface area or >= 10% burned surface area if head/neck or hands/wrist involved w/o lower airway burns	0.2386
Socialist Republic of Viet Nam	2010	Burns, >=20% total burned surface area or >= 10% burned surface area if head/neck or hands/wrist involved w/o lower airway burns	0.2187
Socialist Republic of Viet Nam	2017	Burns, >=20% total burned surface area or >= 10% burned surface area if head/neck or hands/wrist involved w/o lower airway burns	0.1923
Fiji	1990	Amputation of lower limbs, bilateral	0.2379
Fiji	1995	Amputation of lower limbs, bilateral	0.2343
Fiji	2000	Amputation of lower limbs, bilateral	0.2305
Fiji	2005	Amputation of lower limbs, bilateral	0.2281
Fiji	2010	Amputation of lower limbs, bilateral	0.2231
Fiji	2017	Amputation of lower limbs, bilateral	0.2129
Fiji	1990	Lower airway burns	0.2252
Fiji	1995	Lower airway burns	0.2252
Fiji	2000	Lower airway burns	0.2252
Fiji	2005	Lower airway burns	0.2252
Fiji	2010	Lower airway burns	0.2252
Fiji	2017	Lower airway burns	0.2252
Fiji	1990	Dislocation of hip	0.0159
Fiji	1995	Dislocation of hip	0.0159
Fiji	2000	Dislocation of hip	0.0159
Fiji	2005	Dislocation of hip	0.0159
Fiji	2010	Dislocation of hip	0.0159
Fiji	2017	Dislocation of hip	0.0159
Fiji	1990	Dislocation of knee	0.1134
Fiji	1995	Dislocation of knee	0.1134
Fiji	2000	Dislocation of knee	0.1134
Fiji	2005	Dislocation of knee	0.1134
Fiji	2010	Dislocation of knee	0.1134
Fiji	2017	Dislocation of knee	0.1134
Fiji	1990	Dislocation of shoulder	0.062
Fiji	1995	Dislocation of shoulder	0.062
Fiji	2000	Dislocation of shoulder	0.062
Fiji	2005	Dislocation of shoulder	0.062
Fiji	2010	Dislocation of shoulder	0.062
Fiji	2017	Dislocation of shoulder	0.062
Fiji	1990	Muscle and tendon injuries, including sprains and strains lesser dislocations	0.0075
Fiji	1995	Muscle and tendon injuries, including sprains and strains lesser dislocations	0.0075
Fiji	2000	Muscle and tendon injuries, including sprains and strains lesser dislocations	0.0075
Fiji	2005	Muscle and tendon injuries, including sprains and strains lesser dislocations	0.0075
Fiji	2010	Muscle and tendon injuries, including sprains and strains lesser dislocations	0.0075
Fiji	2017	Muscle and tendon injuries, including sprains and strains lesser dislocations	0.0075
Fiji	1990	Fracture of clavicle, scapula, or humerus	0.0349
Fiji	1995	Fracture of clavicle, scapula, or humerus	0.0349
Fiji	2000	Fracture of clavicle, scapula, or humerus	0.0349
Fiji	2005	Fracture of clavicle, scapula, or humerus	0.0349
Fiji	2010	Fracture of clavicle, scapula, or humerus	0.0349
Fiji	2017	Fracture of clavicle, scapula, or humerus	0.0349
Fiji	1990	Fracture of face bones	0.0669
Fiji	1995	Fracture of face bones	0.0669
Fiji	2000	Fracture of face bones	0.0669
Fiji	2005	Fracture of face bones	0.0669
Fiji	2010	Fracture of face bones	0.0669
Fiji	2017	Fracture of face bones	0.0669
Fiji	1990	Fracture of foot bones except ankle	0.0264
Fiji	1995	Fracture of foot bones except ankle	0.0264
Fiji	2000	Fracture of foot bones except ankle	0.0264
Fiji	2005	Fracture of foot bones except ankle	0.0264
Fiji	2010	Fracture of foot bones except ankle	0.0264
Fiji	2017	Fracture of foot bones except ankle	0.0264
Fiji	1990	Fracture of hand (wrist and other distal part of hand)	0.0138
Fiji	1995	Fracture of hand (wrist and other distal part of hand)	0.0138
Fiji	2000	Fracture of hand (wrist and other distal part of hand)	0.0138
Fiji	2005	Fracture of hand (wrist and other distal part of hand)	0.0138
Fiji	2010	Fracture of hand (wrist and other distal part of hand)	0.0138
Fiji	2017	Fracture of hand (wrist and other distal part of hand)	0.0138
Fiji	1990	Fracture of hip	0.2093
Fiji	1995	Fracture of hip	0.2045
Fiji	2000	Fracture of hip	0.1996
Fiji	2005	Fracture of hip	0.1964
Fiji	2010	Fracture of hip	0.1898
Fiji	2017	Fracture of hip	0.1763
Fiji	1990	Amputation of upper limbs, bilateral	0.2442
Fiji	1995	Amputation of upper limbs, bilateral	0.2392
Fiji	2000	Amputation of upper limbs, bilateral	0.2342
Fiji	2005	Amputation of upper limbs, bilateral	0.2309
Fiji	2010	Amputation of upper limbs, bilateral	0.224
Fiji	2017	Amputation of upper limbs, bilateral	0.2101
Fiji	1990	Fracture of patella, tibia or fibula, or ankle	0.0554
Fiji	1995	Fracture of patella, tibia or fibula, or ankle	0.0554
Fiji	2000	Fracture of patella, tibia or fibula, or ankle	0.0554
Fiji	2005	Fracture of patella, tibia or fibula, or ankle	0.0554
Fiji	2010	Fracture of patella, tibia or fibula, or ankle	0.0554
Fiji	2017	Fracture of patella, tibia or fibula, or ankle	0.0554
Fiji	1990	Fracture of pelvis	0.1823
Fiji	1995	Fracture of pelvis	0.1823
Fiji	2000	Fracture of pelvis	0.1823
Fiji	2005	Fracture of pelvis	0.1823
Fiji	2010	Fracture of pelvis	0.1823
Fiji	2017	Fracture of pelvis	0.1823
Fiji	1990	Fracture of radius and/or ulna	0.0435
Fiji	1995	Fracture of radius and/or ulna	0.0435
Fiji	2000	Fracture of radius and/or ulna	0.0435
Fiji	2005	Fracture of radius and/or ulna	0.0435
Fiji	2010	Fracture of radius and/or ulna	0.0435
Fiji	2017	Fracture of radius and/or ulna	0.0435
Fiji	1990	Fracture of skull	0.0714
Fiji	1995	Fracture of skull	0.0714
Fiji	2000	Fracture of skull	0.0714
Fiji	2005	Fracture of skull	0.0714
Fiji	2010	Fracture of skull	0.0714
Fiji	2017	Fracture of skull	0.0714
Fiji	1990	Fracture of sternum and/or fracture of one or more ribs	0.1027
Fiji	1995	Fracture of sternum and/or fracture of one or more ribs	0.1027
Fiji	2000	Fracture of sternum and/or fracture of one or more ribs	0.1027
Fiji	2005	Fracture of sternum and/or fracture of one or more ribs	0.1027
Fiji	2010	Fracture of sternum and/or fracture of one or more ribs	0.1027
Fiji	2017	Fracture of sternum and/or fracture of one or more ribs	0.1027
Fiji	1990	Fracture of vertebral column	0.1106
Fiji	1995	Fracture of vertebral column	0.1106
Fiji	2000	Fracture of vertebral column	0.1106
Fiji	2005	Fracture of vertebral column	0.1106
Fiji	2010	Fracture of vertebral column	0.1106
Fiji	2017	Fracture of vertebral column	0.1106
Fiji	1990	Fracture of femur, other than femoral neck	0.042
Fiji	1995	Fracture of femur, other than femoral neck	0.042
Fiji	2000	Fracture of femur, other than femoral neck	0.042
Fiji	2005	Fracture of femur, other than femoral neck	0.042
Fiji	2010	Fracture of femur, other than femoral neck	0.042
Fiji	2017	Fracture of femur, other than femoral neck	0.042
Fiji	1990	Minor TBI	0.1319
Fiji	1995	Minor TBI	0.1319
Fiji	2000	Minor TBI	0.1319
Fiji	2005	Minor TBI	0.1319
Fiji	2010	Minor TBI	0.1319
Fiji	2017	Minor TBI	0.1319
Fiji	1990	Moderate TBI	0.1637
Fiji	1995	Moderate TBI	0.1637
Fiji	2000	Moderate TBI	0.1637
Fiji	2005	Moderate TBI	0.1637
Fiji	2010	Moderate TBI	0.1637
Fiji	2017	Moderate TBI	0.1637
Fiji	1990	Amputation of fingers (excluding thumb)	0.0047
Fiji	1995	Amputation of fingers (excluding thumb)	0.0047
Fiji	2000	Amputation of fingers (excluding thumb)	0.0047
Fiji	2005	Amputation of fingers (excluding thumb)	0.0047
Fiji	2010	Amputation of fingers (excluding thumb)	0.0047
Fiji	2017	Amputation of fingers (excluding thumb)	0.0047
Fiji	1990	Spinal cord lesion at neck level	0.4642
Fiji	1995	Spinal cord lesion at neck level	0.4626
Fiji	2000	Spinal cord lesion at neck level	0.4609
Fiji	2005	Spinal cord lesion at neck level	0.4598
Fiji	2010	Spinal cord lesion at neck level	0.4575
Fiji	2017	Spinal cord lesion at neck level	0.4528
Fiji	1990	Spinal cord lesion below neck level	0.2883
Fiji	1995	Spinal cord lesion below neck level	0.2848
Fiji	2000	Spinal cord lesion below neck level	0.2812
Fiji	2005	Spinal cord lesion below neck level	0.2789
Fiji	2010	Spinal cord lesion below neck level	0.2741
Fiji	2017	Spinal cord lesion below neck level	0.2642
Fiji	1990	Drowning and nonfatal submersion	0.2471
Fiji	1995	Drowning and nonfatal submersion	0.2471
Fiji	2000	Drowning and nonfatal submersion	0.2471
Fiji	2005	Drowning and nonfatal submersion	0.2471
Fiji	2010	Drowning and nonfatal submersion	0.2471
Fiji	2017	Drowning and nonfatal submersion	0.2471
Fiji	1990	Asphyxiation	0.2471
Fiji	1995	Asphyxiation	0.2471
Fiji	2000	Asphyxiation	0.2471
Fiji	2005	Asphyxiation	0.2471
Fiji	2010	Asphyxiation	0.2471
Fiji	2017	Asphyxiation	0.2471
Fiji	1990	Crush injury	0.1325
Fiji	1995	Crush injury	0.1325
Fiji	2000	Crush injury	0.1325
Fiji	2005	Crush injury	0.1325
Fiji	2010	Crush injury	0.1325
Fiji	2017	Crush injury	0.1325
Fiji	1990	Nerve injury	0.1129
Fiji	1995	Nerve injury	0.1129
Fiji	2000	Nerve injury	0.1129
Fiji	2005	Nerve injury	0.1129
Fiji	2010	Nerve injury	0.1129
Fiji	2017	Nerve injury	0.1129
Fiji	1990	Injury to eyes	0.0165
Fiji	1995	Injury to eyes	0.0165
Fiji	2000	Injury to eyes	0.0165
Fiji	2005	Injury to eyes	0.0165
Fiji	2010	Injury to eyes	0.0165
Fiji	2017	Injury to eyes	0.0165
Fiji	1990	Amputation of lower limb, unilateral	0.098
Fiji	1995	Amputation of lower limb, unilateral	0.0962
Fiji	2000	Amputation of lower limb, unilateral	0.0943
Fiji	2005	Amputation of lower limb, unilateral	0.093
Fiji	2010	Amputation of lower limb, unilateral	0.0904
Fiji	2017	Amputation of lower limb, unilateral	0.0852
Fiji	1990	Open wound(s)	0.011
Fiji	1995	Open wound(s)	0.011
Fiji	2000	Open wound(s)	0.011
Fiji	2005	Open wound(s)	0.011
Fiji	2010	Open wound(s)	0.011
Fiji	2017	Open wound(s)	0.011
Fiji	1990	Poisoning requiring urgent care	0.1628
Fiji	1995	Poisoning requiring urgent care	0.1628
Fiji	2000	Poisoning requiring urgent care	0.1628
Fiji	2005	Poisoning requiring urgent care	0.1628
Fiji	2010	Poisoning requiring urgent care	0.1628
Fiji	2017	Poisoning requiring urgent care	0.1628
Fiji	1990	Severe chest injury	0.0474
Fiji	1995	Severe chest injury	0.0474
Fiji	2000	Severe chest injury	0.0474
Fiji	2005	Severe chest injury	0.0474
Fiji	2010	Severe chest injury	0.0474
Fiji	2017	Severe chest injury	0.0474
Fiji	1990	Internal hemorrhage in abdomen and pelvis	0.1143
Fiji	1995	Internal hemorrhage in abdomen and pelvis	0.1143
Fiji	2000	Internal hemorrhage in abdomen and pelvis	0.1143
Fiji	2005	Internal hemorrhage in abdomen and pelvis	0.1143
Fiji	2010	Internal hemorrhage in abdomen and pelvis	0.1143
Fiji	2017	Internal hemorrhage in abdomen and pelvis	0.1143
Fiji	1990	Contusion in any part of the body	0.0075
Fiji	1995	Contusion in any part of the body	0.0075
Fiji	2000	Contusion in any part of the body	0.0075
Fiji	2005	Contusion in any part of the body	0.0075
Fiji	2010	Contusion in any part of the body	0.0075
Fiji	2017	Contusion in any part of the body	0.0075
Fiji	1990	Effect of different environmental factors	0.049
Fiji	1995	Effect of different environmental factors	0.049
Fiji	2000	Effect of different environmental factors	0.049
Fiji	2005	Effect of different environmental factors	0.049
Fiji	2010	Effect of different environmental factors	0.049
Fiji	2017	Effect of different environmental factors	0.049
Fiji	1990	Complications following therapeutic procedures	0.0506
Fiji	1995	Complications following therapeutic procedures	0.0506
Fiji	2000	Complications following therapeutic procedures	0.0506
Fiji	2005	Complications following therapeutic procedures	0.0506
Fiji	2010	Complications following therapeutic procedures	0.0506
Fiji	2017	Complications following therapeutic procedures	0.0506
Fiji	1990	Multiple fractures, dislocations, crashes, wounds, sprains, and strains	0.2093
Fiji	1995	Multiple fractures, dislocations, crashes, wounds, sprains, and strains	0.2045
Fiji	2000	Multiple fractures, dislocations, crashes, wounds, sprains, and strains	0.1996
Fiji	2005	Multiple fractures, dislocations, crashes, wounds, sprains, and strains	0.1964
Fiji	2010	Multiple fractures, dislocations, crashes, wounds, sprains, and strains	0.1898
Fiji	2017	Multiple fractures, dislocations, crashes, wounds, sprains, and strains	0.1763
Fiji	1990	Amputation of upper limb, unilateral	0.0738
Fiji	1995	Amputation of upper limb, unilateral	0.0727
Fiji	2000	Amputation of upper limb, unilateral	0.0715
Fiji	2005	Amputation of upper limb, unilateral	0.0708
Fiji	2010	Amputation of upper limb, unilateral	0.0693
Fiji	2017	Amputation of upper limb, unilateral	0.0662
Fiji	1990	Amputation of thumb	0.0114
Fiji	1995	Amputation of thumb	0.0114
Fiji	2000	Amputation of thumb	0.0114
Fiji	2005	Amputation of thumb	0.0114
Fiji	2010	Amputation of thumb	0.0114
Fiji	2017	Amputation of thumb	0.0114
Fiji	1990	Amputation of toe/toes	0.0059
Fiji	1995	Amputation of toe/toes	0.0059
Fiji	2000	Amputation of toe/toes	0.0059
Fiji	2005	Amputation of toe/toes	0.0059
Fiji	2010	Amputation of toe/toes	0.0059
Fiji	2017	Amputation of toe/toes	0.0059
Fiji	1990	Burns, <20% total burned surface area without lower airway burns	0.0163
Fiji	1995	Burns, <20% total burned surface area without lower airway burns	0.0163
Fiji	2000	Burns, <20% total burned surface area without lower airway burns	0.0163
Fiji	2005	Burns, <20% total burned surface area without lower airway burns	0.0163
Fiji	2010	Burns, <20% total burned surface area without lower airway burns	0.0163
Fiji	2017	Burns, <20% total burned surface area without lower airway burns	0.0163
Fiji	1990	Burns, >=20% total burned surface area or >= 10% burned surface area if head/neck or hands/wrist involved w/o lower airway burns	0.2757
Fiji	1995	Burns, >=20% total burned surface area or >= 10% burned surface area if head/neck or hands/wrist involved w/o lower airway burns	0.2713
Fiji	2000	Burns, >=20% total burned surface area or >= 10% burned surface area if head/neck or hands/wrist involved w/o lower airway burns	0.2667
Fiji	2005	Burns, >=20% total burned surface area or >= 10% burned surface area if head/neck or hands/wrist involved w/o lower airway burns	0.2638
Fiji	2010	Burns, >=20% total burned surface area or >= 10% burned surface area if head/neck or hands/wrist involved w/o lower airway burns	0.2576
Fiji	2017	Burns, >=20% total burned surface area or >= 10% burned surface area if head/neck or hands/wrist involved w/o lower airway burns	0.245
Kiribati	1990	Amputation of lower limbs, bilateral	0.3077
Kiribati	1995	Amputation of lower limbs, bilateral	0.305
Kiribati	2000	Amputation of lower limbs, bilateral	0.2987
Kiribati	2005	Amputation of lower limbs, bilateral	0.2957
Kiribati	2010	Amputation of lower limbs, bilateral	0.2903
Kiribati	2017	Amputation of lower limbs, bilateral	0.2851
Kiribati	1990	Lower airway burns	0.2252
Kiribati	1995	Lower airway burns	0.2252
Kiribati	2000	Lower airway burns	0.2252
Kiribati	2005	Lower airway burns	0.2252
Kiribati	2010	Lower airway burns	0.2252
Kiribati	2017	Lower airway burns	0.2252
Kiribati	1990	Dislocation of hip	0.0159
Kiribati	1995	Dislocation of hip	0.0159
Kiribati	2000	Dislocation of hip	0.0159
Kiribati	2005	Dislocation of hip	0.0159
Kiribati	2010	Dislocation of hip	0.0159
Kiribati	2017	Dislocation of hip	0.0159
Kiribati	1990	Dislocation of knee	0.1134
Kiribati	1995	Dislocation of knee	0.1134
Kiribati	2000	Dislocation of knee	0.1134
Kiribati	2005	Dislocation of knee	0.1134
Kiribati	2010	Dislocation of knee	0.1134
Kiribati	2017	Dislocation of knee	0.1134
Kiribati	1990	Dislocation of shoulder	0.062
Kiribati	1995	Dislocation of shoulder	0.062
Kiribati	2000	Dislocation of shoulder	0.062
Kiribati	2005	Dislocation of shoulder	0.062
Kiribati	2010	Dislocation of shoulder	0.062
Kiribati	2017	Dislocation of shoulder	0.062
Kiribati	1990	Muscle and tendon injuries, including sprains and strains lesser dislocations	0.0075
Kiribati	1995	Muscle and tendon injuries, including sprains and strains lesser dislocations	0.0075
Kiribati	2000	Muscle and tendon injuries, including sprains and strains lesser dislocations	0.0075
Kiribati	2005	Muscle and tendon injuries, including sprains and strains lesser dislocations	0.0075
Kiribati	2010	Muscle and tendon injuries, including sprains and strains lesser dislocations	0.0075
Kiribati	2017	Muscle and tendon injuries, including sprains and strains lesser dislocations	0.0075
Kiribati	1990	Fracture of clavicle, scapula, or humerus	0.0349
Kiribati	1995	Fracture of clavicle, scapula, or humerus	0.0349
Kiribati	2000	Fracture of clavicle, scapula, or humerus	0.0349
Kiribati	2005	Fracture of clavicle, scapula, or humerus	0.0349
Kiribati	2010	Fracture of clavicle, scapula, or humerus	0.0349
Kiribati	2017	Fracture of clavicle, scapula, or humerus	0.0349
Kiribati	1990	Fracture of face bones	0.0669
Kiribati	1995	Fracture of face bones	0.0669
Kiribati	2000	Fracture of face bones	0.0669
Kiribati	2005	Fracture of face bones	0.0669
Kiribati	2010	Fracture of face bones	0.0669
Kiribati	2017	Fracture of face bones	0.0669
Kiribati	1990	Fracture of foot bones except ankle	0.0264
Kiribati	1995	Fracture of foot bones except ankle	0.0264
Kiribati	2000	Fracture of foot bones except ankle	0.0264
Kiribati	2005	Fracture of foot bones except ankle	0.0264
Kiribati	2010	Fracture of foot bones except ankle	0.0264
Kiribati	2017	Fracture of foot bones except ankle	0.0264
Kiribati	1990	Fracture of hand (wrist and other distal part of hand)	0.0138
Kiribati	1995	Fracture of hand (wrist and other distal part of hand)	0.0138
Kiribati	2000	Fracture of hand (wrist and other distal part of hand)	0.0138
Kiribati	2005	Fracture of hand (wrist and other distal part of hand)	0.0138
Kiribati	2010	Fracture of hand (wrist and other distal part of hand)	0.0138
Kiribati	2017	Fracture of hand (wrist and other distal part of hand)	0.0138
Kiribati	1990	Fracture of hip	0.3017
Kiribati	1995	Fracture of hip	0.2981
Kiribati	2000	Fracture of hip	0.2897
Kiribati	2005	Fracture of hip	0.2857
Kiribati	2010	Fracture of hip	0.2787
Kiribati	2017	Fracture of hip	0.2718
Kiribati	1990	Amputation of upper limbs, bilateral	0.3396
Kiribati	1995	Amputation of upper limbs, bilateral	0.3359
Kiribati	2000	Amputation of upper limbs, bilateral	0.3272
Kiribati	2005	Amputation of upper limbs, bilateral	0.3231
Kiribati	2010	Amputation of upper limbs, bilateral	0.3158
Kiribati	2017	Amputation of upper limbs, bilateral	0.3087
Kiribati	1990	Fracture of patella, tibia or fibula, or ankle	0.0554
Kiribati	1995	Fracture of patella, tibia or fibula, or ankle	0.0554
Kiribati	2000	Fracture of patella, tibia or fibula, or ankle	0.0554
Kiribati	2005	Fracture of patella, tibia or fibula, or ankle	0.0554
Kiribati	2010	Fracture of patella, tibia or fibula, or ankle	0.0554
Kiribati	2017	Fracture of patella, tibia or fibula, or ankle	0.0554
Kiribati	1990	Fracture of pelvis	0.1823
Kiribati	1995	Fracture of pelvis	0.1823
Kiribati	2000	Fracture of pelvis	0.1823
Kiribati	2005	Fracture of pelvis	0.1823
Kiribati	2010	Fracture of pelvis	0.1823
Kiribati	2017	Fracture of pelvis	0.1823
Kiribati	1990	Fracture of radius and/or ulna	0.0435
Kiribati	1995	Fracture of radius and/or ulna	0.0435
Kiribati	2000	Fracture of radius and/or ulna	0.0435
Kiribati	2005	Fracture of radius and/or ulna	0.0435
Kiribati	2010	Fracture of radius and/or ulna	0.0435
Kiribati	2017	Fracture of radius and/or ulna	0.0435
Kiribati	1990	Fracture of skull	0.0714
Kiribati	1995	Fracture of skull	0.0714
Kiribati	2000	Fracture of skull	0.0714

Kiribati	2005	Fracture of skull	0.0714
Kiribati	2010	Fracture of skull	0.0714
Kiribati	2017	Fracture of skull	0.0714
Kiribati	1990	Fracture of sternum and/or fracture of one or more ribs	0.1027
Kiribati	1995	Fracture of sternum and/or fracture of one or more ribs	0.1027
Kiribati	2000	Fracture of sternum and/or fracture of one or more ribs	0.1027
Kiribati	2005	Fracture of sternum and/or fracture of one or more ribs	0.1027
Kiribati	2010	Fracture of sternum and/or fracture of one or more ribs	0.1027
Kiribati	2017	Fracture of sternum and/or fracture of one or more ribs	0.1027
Kiribati	1990	Fracture of vertebral column	0.1106
Kiribati	1995	Fracture of vertebral column	0.1106
Kiribati	2000	Fracture of vertebral column	0.1106
Kiribati	2005	Fracture of vertebral column	0.1106
Kiribati	2010	Fracture of vertebral column	0.1106
Kiribati	2017	Fracture of vertebral column	0.1106
Kiribati	1990	Fracture of femur, other than femoral neck	0.042
Kiribati	1995	Fracture of femur, other than femoral neck	0.042
Kiribati	2000	Fracture of femur, other than femoral neck	0.042
Kiribati	2005	Fracture of femur, other than femoral neck	0.042
Kiribati	2010	Fracture of femur, other than femoral neck	0.042
Kiribati	2017	Fracture of femur, other than femoral neck	0.042
Kiribati	1990	Minor TBI	0.1319
Kiribati	1995	Minor TBI	0.1319
Kiribati	2000	Minor TBI	0.1319
Kiribati	2005	Minor TBI	0.1319
Kiribati	2010	Minor TBI	0.1319
Kiribati	2017	Minor TBI	0.1319
Kiribati	1990	Moderate TBI	0.1637
Kiribati	1995	Moderate TBI	0.1637
Kiribati	2000	Moderate TBI	0.1637
Kiribati	2005	Moderate TBI	0.1637
Kiribati	2010	Moderate TBI	0.1637
Kiribati	2017	Moderate TBI	0.1637
Kiribati	1990	Amputation of fingers (excluding thumb)	0.0047
Kiribati	1995	Amputation of fingers (excluding thumb)	0.0047
Kiribati	2000	Amputation of fingers (excluding thumb)	0.0047
Kiribati	2005	Amputation of fingers (excluding thumb)	0.0047
Kiribati	2010	Amputation of fingers (excluding thumb)	0.0047
Kiribati	2017	Amputation of fingers (excluding thumb)	0.0047
Kiribati	1990	Spinal cord lesion at neck level	0.4962
Kiribati	1995	Spinal cord lesion at neck level	0.495
Kiribati	2000	Spinal cord lesion at neck level	0.4921
Kiribati	2005	Spinal cord lesion at neck level	0.4907
Kiribati	2010	Spinal cord lesion at neck level	0.4882
Kiribati	2017	Spinal cord lesion at neck level	0.4859
Kiribati	1990	Spinal cord lesion below neck level	0.3558
Kiribati	1995	Spinal cord lesion below neck level	0.3531
Kiribati	2000	Spinal cord lesion below neck level	0.347
Kiribati	2005	Spinal cord lesion below neck level	0.3441
Kiribati	2010	Spinal cord lesion below neck level	0.3389
Kiribati	2017	Spinal cord lesion below neck level	0.3339
Kiribati	1990	Drowning and nonfatal submersion	0.2471
Kiribati	1995	Drowning and nonfatal submersion	0.2471
Kiribati	2000	Drowning and nonfatal submersion	0.2471
Kiribati	2005	Drowning and nonfatal submersion	0.2471
Kiribati	2010	Drowning and nonfatal submersion	0.2471
Kiribati	2017	Drowning and nonfatal submersion	0.2471
Kiribati	1990	Asphyxiation	0.2471
Kiribati	1995	Asphyxiation	0.2471
Kiribati	2000	Asphyxiation	0.2471
Kiribati	2005	Asphyxiation	0.2471
Kiribati	2010	Asphyxiation	0.2471
Kiribati	2017	Asphyxiation	0.2471
Kiribati	1990	Crush injury	0.1325
Kiribati	1995	Crush injury	0.1325
Kiribati	2000	Crush injury	0.1325
Kiribati	2005	Crush injury	0.1325
Kiribati	2010	Crush injury	0.1325
Kiribati	2017	Crush injury	0.1325
Kiribati	1990	Nerve injury	0.1129
Kiribati	1995	Nerve injury	0.1129
Kiribati	2000	Nerve injury	0.1129
Kiribati	2005	Nerve injury	0.1129
Kiribati	2010	Nerve injury	0.1129
Kiribati	2017	Nerve injury	0.1129
Kiribati	1990	Injury to eyes	0.0165
Kiribati	1995	Injury to eyes	0.0165
Kiribati	2000	Injury to eyes	0.0165
Kiribati	2005	Injury to eyes	0.0165
Kiribati	2010	Injury to eyes	0.0165
Kiribati	2017	Injury to eyes	0.0165
Kiribati	1990	Amputation of lower limb, unilateral	0.1341
Kiribati	1995	Amputation of lower limb, unilateral	0.1327
Kiribati	2000	Amputation of lower limb, unilateral	0.1294
Kiribati	2005	Amputation of lower limb, unilateral	0.1278
Kiribati	2010	Amputation of lower limb, unilateral	0.1251
Kiribati	2017	Amputation of lower limb, unilateral	0.1224
Kiribati	1990	Open wound(s)	0.011
Kiribati	1995	Open wound(s)	0.011
Kiribati	2000	Open wound(s)	0.011
Kiribati	2005	Open wound(s)	0.011
Kiribati	2010	Open wound(s)	0.011
Kiribati	2017	Open wound(s)	0.011
Kiribati	1990	Poisoning requiring urgent care	0.1628
Kiribati	1995	Poisoning requiring urgent care	0.1628
Kiribati	2000	Poisoning requiring urgent care	0.1628
Kiribati	2005	Poisoning requiring urgent care	0.1628
Kiribati	2010	Poisoning requiring urgent care	0.1628
Kiribati	2017	Poisoning requiring urgent care	0.1628
Kiribati	1990	Severe chest injury	0.0474
Kiribati	1995	Severe chest injury	0.0474
Kiribati	2000	Severe chest injury	0.0474
Kiribati	2005	Severe chest injury	0.0474
Kiribati	2010	Severe chest injury	0.0474
Kiribati	2017	Severe chest injury	0.0474
Kiribati	1990	Internal hemorrhage in abdomen and pelvis	0.1143
Kiribati	1995	Internal hemorrhage in abdomen and pelvis	0.1143
Kiribati	2000	Internal hemorrhage in abdomen and pelvis	0.1143
Kiribati	2005	Internal hemorrhage in abdomen and pelvis	0.1143
Kiribati	2010	Internal hemorrhage in abdomen and pelvis	0.1143
Kiribati	2017	Internal hemorrhage in abdomen and pelvis	0.1143
Kiribati	1990	Contusion in any part of the body	0.0075
Kiribati	1995	Contusion in any part of the body	0.0075
Kiribati	2000	Contusion in any part of the body	0.0075
Kiribati	2005	Contusion in any part of the body	0.0075
Kiribati	2010	Contusion in any part of the body	0.0075
Kiribati	2017	Contusion in any part of the body	0.0075
Kiribati	1990	Effect of different environmental factors	0.049
Kiribati	1995	Effect of different environmental factors	0.049
Kiribati	2000	Effect of different environmental factors	0.049
Kiribati	2005	Effect of different environmental factors	0.049
Kiribati	2010	Effect of different environmental factors	0.049
Kiribati	2017	Effect of different environmental factors	0.049
Kiribati	1990	Complications following therapeutic procedures	0.0506
Kiribati	1995	Complications following therapeutic procedures	0.0506
Kiribati	2000	Complications following therapeutic procedures	0.0506
Kiribati	2005	Complications following therapeutic procedures	0.0506
Kiribati	2010	Complications following therapeutic procedures	0.0506
Kiribati	2017	Complications following therapeutic procedures	0.0506
Kiribati	1990	Multiple fractures, dislocations, crashes, wounds, sprains, and strains	0.3017
Kiribati	1995	Multiple fractures, dislocations, crashes, wounds, sprains, and strains	0.2981
Kiribati	2000	Multiple fractures, dislocations, crashes, wounds, sprains, and strains	0.2897
Kiribati	2005	Multiple fractures, dislocations, crashes, wounds, sprains, and strains	0.2857
Kiribati	2010	Multiple fractures, dislocations, crashes, wounds, sprains, and strains	0.2787
Kiribati	2017	Multiple fractures, dislocations, crashes, wounds, sprains, and strains	0.2718
Kiribati	1990	Amputation of upper limb, unilateral	0.095
Kiribati	1995	Amputation of upper limb, unilateral	0.0941
Kiribati	2000	Amputation of upper limb, unilateral	0.0922
Kiribati	2005	Amputation of upper limb, unilateral	0.0913
Kiribati	2010	Amputation of upper limb, unilateral	0.0897
Kiribati	2017	Amputation of upper limb, unilateral	0.0881
Kiribati	1990	Amputation of thumb	0.0114
Kiribati	1995	Amputation of thumb	0.0114
Kiribati	2000	Amputation of thumb	0.0114
Kiribati	2005	Amputation of thumb	0.0114
Kiribati	2010	Amputation of thumb	0.0114
Kiribati	2017	Amputation of thumb	0.0114
Kiribati	1990	Amputation of toe/toes	0.0059
Kiribati	1995	Amputation of toe/toes	0.0059
Kiribati	2000	Amputation of toe/toes	0.0059
Kiribati	2005	Amputation of toe/toes	0.0059
Kiribati	2010	Amputation of toe/toes	0.0059
Kiribati	2017	Amputation of toe/toes	0.0059
Kiribati	1990	Burns, <20% total burned surface area without lower airway burns	0.0163
Kiribati	1995	Burns, <20% total burned surface area without lower airway burns	0.0163
Kiribati	2000	Burns, <20% total burned surface area without lower airway burns	0.0163
Kiribati	2005	Burns, <20% total burned surface area without lower airway burns	0.0163
Kiribati	2010	Burns, <20% total burned surface area without lower airway burns	0.0163
Kiribati	2017	Burns, <20% total burned surface area without lower airway burns	0.0163
Kiribati	1990	Burns, >=20% total burned surface area or >= 10% burned surface area if head/neck or hands/wrist involved w/o lower airway burns	0.3618
Kiribati	1995	Burns, >=20% total burned surface area or >= 10% burned surface area if head/neck or hands/wrist involved w/o lower airway burns	0.3585
Kiribati	2000	Burns, >=20% total burned surface area or >= 10% burned surface area if head/neck or hands/wrist involved w/o lower airway burns	0.3507
Kiribati	2005	Burns, >=20% total burned surface area or >= 10% burned surface area if head/neck or hands/wrist involved w/o lower airway burns	0.347
Kiribati	2010	Burns, >=20% total burned surface area or >= 10% burned surface area if head/neck or hands/wrist involved w/o lower airway burns	0.3404
Kiribati	2017	Burns, >=20% total burned surface area or >= 10% burned surface area if head/neck or hands/wrist involved w/o lower airway burns	0.3339
Marshall Islands	1990	Amputation of lower limbs, bilateral	0.2644
Marshall Islands	1995	Amputation of lower limbs, bilateral	0.2597
Marshall Islands	2000	Amputation of lower limbs, bilateral	0.2585
Marshall Islands	2005	Amputation of lower limbs, bilateral	0.2507
Marshall Islands	2010	Amputation of lower limbs, bilateral	0.2433
Marshall Islands	2017	Amputation of lower limbs, bilateral	0.2296
Marshall Islands	1990	Lower airway burns	0.2252
Marshall Islands	1995	Lower airway burns	0.2252
Marshall Islands	2000	Lower airway burns	0.2252
Marshall Islands	2005	Lower airway burns	0.2252
Marshall Islands	2010	Lower airway burns	0.2252
Marshall Islands	2017	Lower airway burns	0.2252
Marshall Islands	1990	Dislocation of hip	0.0159
Marshall Islands	1995	Dislocation of hip	0.0159
Marshall Islands	2000	Dislocation of hip	0.0159
Marshall Islands	2005	Dislocation of hip	0.0159
Marshall Islands	2010	Dislocation of hip	0.0159
Marshall Islands	2017	Dislocation of hip	0.0159
Marshall Islands	1990	Dislocation of knee	0.1134
Marshall Islands	1995	Dislocation of knee	0.1134
Marshall Islands	2000	Dislocation of knee	0.1134
Marshall Islands	2005	Dislocation of knee	0.1134
Marshall Islands	2010	Dislocation of knee	0.1134
Marshall Islands	2017	Dislocation of knee	0.1134
Marshall Islands	1990	Dislocation of shoulder	0.062
Marshall Islands	1995	Dislocation of shoulder	0.062
Marshall Islands	2000	Dislocation of shoulder	0.062
Marshall Islands	2005	Dislocation of shoulder	0.062
Marshall Islands	2010	Dislocation of shoulder	0.062
Marshall Islands	2017	Dislocation of shoulder	0.062
Marshall Islands	1990	Muscle and tendon injuries, including sprains and strains lesser dislocations	0.0075
Marshall Islands	1995	Muscle and tendon injuries, including sprains and strains lesser dislocations	0.0075
Marshall Islands	2000	Muscle and tendon injuries, including sprains and strains lesser dislocations	0.0075
Marshall Islands	2005	Muscle and tendon injuries, including sprains and strains lesser dislocations	0.0075
Marshall Islands	2010	Muscle and tendon injuries, including sprains and strains lesser dislocations	0.0075
Marshall Islands	2017	Muscle and tendon injuries, including sprains and strains lesser dislocations	0.0075
Marshall Islands	1990	Fracture of clavicle, scapula, or humerus	0.0349
Marshall Islands	1995	Fracture of clavicle, scapula, or humerus	0.0349
Marshall Islands	2000	Fracture of clavicle, scapula, or humerus	0.0349
Marshall Islands	2005	Fracture of clavicle, scapula, or humerus	0.0349
Marshall Islands	2010	Fracture of clavicle, scapula, or humerus	0.0349
Marshall Islands	2017	Fracture of clavicle, scapula, or humerus	0.0349
Marshall Islands	1990	Fracture of face bones	0.0669
Marshall Islands	1995	Fracture of face bones	0.0669
Marshall Islands	2000	Fracture of face bones	0.0669
Marshall Islands	2005	Fracture of face bones	0.0669
Marshall Islands	2010	Fracture of face bones	0.0669
Marshall Islands	2017	Fracture of face bones	0.0669
Marshall Islands	1990	Fracture of foot bones except ankle	0.0264
Marshall Islands	1995	Fracture of foot bones except ankle	0.0264
Marshall Islands	2000	Fracture of foot bones except ankle	0.0264
Marshall Islands	2005	Fracture of foot bones except ankle	0.0264
Marshall Islands	2010	Fracture of foot bones except ankle	0.0264
Marshall Islands	2017	Fracture of foot bones except ankle	0.0264
Marshall Islands	1990	Fracture of hand (wrist and other distal part of hand)	0.0138
Marshall Islands	1995	Fracture of hand (wrist and other distal part of hand)	0.0138
Marshall Islands	2000	Fracture of hand (wrist and other distal part of hand)	0.0138
Marshall Islands	2005	Fracture of hand (wrist and other distal part of hand)	0.0138
Marshall Islands	2010	Fracture of hand (wrist and other distal part of hand)	0.0138
Marshall Islands	2017	Fracture of hand (wrist and other distal part of hand)	0.0138
Marshall Islands	1990	Fracture of hip	0.2445
Marshall Islands	1995	Fracture of hip	0.2381
Marshall Islands	2000	Fracture of hip	0.2379
Marshall Islands	2005	Fracture of hip	0.2262
Marshall Islands	2010	Fracture of hip	0.2164
Marshall Islands	2017	Fracture of hip	0.1983
Marshall Islands	1990	Amputation of upper limbs, bilateral	0.2805
Marshall Islands	1995	Amputation of upper limbs, bilateral	0.274
Marshall Islands	2000	Amputation of upper limbs, bilateral	0.2737
Marshall Islands	2005	Amputation of upper limbs, bilateral	0.2617
Marshall Islands	2010	Amputation of upper limbs, bilateral	0.2516
Marshall Islands	2017	Amputation of upper limbs, bilateral	0.2328
Marshall Islands	1990	Fracture of patella, tibia or fibula, or ankle	0.0554
Marshall Islands	1995	Fracture of patella, tibia or fibula, or ankle	0.0554
Marshall Islands	2000	Fracture of patella, tibia or fibula, or ankle	0.0554
Marshall Islands	2005	Fracture of patella, tibia or fibula, or ankle	0.0554
Marshall Islands	2010	Fracture of patella, tibia or fibula, or ankle	0.0554
Marshall Islands	2017	Fracture of patella, tibia or fibula, or ankle	0.0554
Marshall Islands	1990	Fracture of pelvis	0.1823
Marshall Islands	1995	Fracture of pelvis	0.1823
Marshall Islands	2000	Fracture of pelvis	0.1823
Marshall Islands	2005	Fracture of pelvis	0.1823
Marshall Islands	2010	Fracture of pelvis	0.1823
Marshall Islands	2017	Fracture of pelvis	0.1823
Marshall Islands	1990	Fracture of radius and/or ulna	0.0435
Marshall Islands	1995	Fracture of radius and/or ulna	0.0435
Marshall Islands	2000	Fracture of radius and/or ulna	0.0435
Marshall Islands	2005	Fracture of radius and/or ulna	0.0435
Marshall Islands	2010	Fracture of radius and/or ulna	0.0435
Marshall Islands	2017	Fracture of radius and/or ulna	0.0435
Marshall Islands	1990	Fracture of skull	0.0714
Marshall Islands	1995	Fracture of skull	0.0714
Marshall Islands	2000	Fracture of skull	0.0714
Marshall Islands	2005	Fracture of skull	0.0714
Marshall Islands	2010	Fracture of skull	0.0714
Marshall Islands	2017	Fracture of skull	0.0714
Marshall Islands	1990	Fracture of sternum and/or fracture of one or more ribs	0.1027
Marshall Islands	1995	Fracture of sternum and/or fracture of one or more ribs	0.1027
Marshall Islands	2000	Fracture of sternum and/or fracture of one or more ribs	0.1027
Marshall Islands	2005	Fracture of sternum and/or fracture of one or more ribs	0.1027
Marshall Islands	2010	Fracture of sternum and/or fracture of one or more ribs	0.1027
Marshall Islands	2017	Fracture of sternum and/or fracture of one or more ribs	0.1027
Marshall Islands	1990	Fracture of vertebral column	0.1106
Marshall Islands	1995	Fracture of vertebral column	0.1106
Marshall Islands	2000	Fracture of vertebral column	0.1106
Marshall Islands	2005	Fracture of vertebral column	0.1106
Marshall Islands	2010	Fracture of vertebral column	0.1106
Marshall Islands	2017	Fracture of vertebral column	0.1106
Marshall Islands	1990	Fracture of femur, other than femoral neck	0.042
Marshall Islands	1995	Fracture of femur, other than femoral neck	0.042
Marshall Islands	2000	Fracture of femur, other than femoral neck	0.042
Marshall Islands	2005	Fracture of femur, other than femoral neck	0.042
Marshall Islands	2010	Fracture of femur, other than femoral neck	0.042
Marshall Islands	2017	Fracture of femur, other than femoral neck	0.042
Marshall Islands	1990	Minor TBI	0.1319
Marshall Islands	1995	Minor TBI	0.1319
Marshall Islands	2000	Minor TBI	0.1319
Marshall Islands	2005	Minor TBI	0.1319
Marshall Islands	2010	Minor TBI	0.1319
Marshall Islands	2017	Minor TBI	0.1319
Marshall Islands	1990	Moderate TBI	0.1637
Marshall Islands	1995	Moderate TBI	0.1637
Marshall Islands	2000	Moderate TBI	0.1637
Marshall Islands	2005	Moderate TBI	0.1637
Marshall Islands	2010	Moderate TBI	0.1637
Marshall Islands	2017	Moderate TBI	0.1637
Marshall Islands	1990	Amputation of fingers (excluding thumb)	0.0047
Marshall Islands	1995	Amputation of fingers (excluding thumb)	0.0047
Marshall Islands	2000	Amputation of fingers (excluding thumb)	0.0047
Marshall Islands	2005	Amputation of fingers (excluding thumb)	0.0047
Marshall Islands	2010	Amputation of fingers (excluding thumb)	0.0047
Marshall Islands	2017	Amputation of fingers (excluding thumb)	0.0047
Marshall Islands	1990	Spinal cord lesion at neck level	0.4764
Marshall Islands	1995	Spinal cord lesion at neck level	0.4742
Marshall Islands	2000	Spinal cord lesion at neck level	0.4741
Marshall Islands	2005	Spinal cord lesion at neck level	0.4701
Marshall Islands	2010	Spinal cord lesion at neck level	0.4667
Marshall Islands	2017	Spinal cord lesion at neck level	0.4604
Marshall Islands	1990	Spinal cord lesion below neck level	0.314
Marshall Islands	1995	Spinal cord lesion below neck level	0.3094
Marshall Islands	2000	Spinal cord lesion below neck level	0.3092
Marshall Islands	2005	Spinal cord lesion below neck level	0.3007
Marshall Islands	2010	Spinal cord lesion below neck level	0.2935
Marshall Islands	2017	Spinal cord lesion below neck level	0.2803
Marshall Islands	1990	Drowning and nonfatal submersion	0.2471
Marshall Islands	1995	Drowning and nonfatal submersion	0.2471
Marshall Islands	2000	Drowning and nonfatal submersion	0.2471
Marshall Islands	2005	Drowning and nonfatal submersion	0.2471
Marshall Islands	2010	Drowning and nonfatal submersion	0.2471
Marshall Islands	2017	Drowning and nonfatal submersion	0.2471
Marshall Islands	1990	Asphyxiation	0.2471
Marshall Islands	1995	Asphyxiation	0.2471
Marshall Islands	2000	Asphyxiation	0.2471
Marshall Islands	2005	Asphyxiation	0.2471
Marshall Islands	2010	Asphyxiation	0.2471
Marshall Islands	2017	Asphyxiation	0.2471
Marshall Islands	1990	Crush injury	0.1325
Marshall Islands	1995	Crush injury	0.1325
Marshall Islands	2000	Crush injury	0.1325
Marshall Islands	2005	Crush injury	0.1325
Marshall Islands	2010	Crush injury	0.1325
Marshall Islands	2017	Crush injury	0.1325
Marshall Islands	1990	Nerve injury	0.1129
Marshall Islands	1995	Nerve injury	0.1129
Marshall Islands	2000	Nerve injury	0.1129
Marshall Islands	2005	Nerve injury	0.1129
Marshall Islands	2010	Nerve injury	0.1129
Marshall Islands	2017	Nerve injury	0.1129
Marshall Islands	1990	Injury to eyes	0.0165
Marshall Islands	1995	Injury to eyes	0.0165
Marshall Islands	2000	Injury to eyes	0.0165
Marshall Islands	2005	Injury to eyes	0.0165
Marshall Islands	2010	Injury to eyes	0.0165
Marshall Islands	2017	Injury to eyes	0.0165
Marshall Islands	1990	Amputation of lower limb, unilateral	0.1118
Marshall Islands	1995	Amputation of lower limb, unilateral	0.1093
Marshall Islands	2000	Amputation of lower limb, unilateral	0.1092
Marshall Islands	2005	Amputation of lower limb, unilateral	0.1047
Marshall Islands	2010	Amputation of lower limb, unilateral	0.1008
Marshall Islands	2017	Amputation of lower limb, unilateral	0.0938
Marshall Islands	1990	Open wound(s)	0.011
Marshall Islands	1995	Open wound(s)	0.011
Marshall Islands	2000	Open wound(s)	0.011
Marshall Islands	2005	Open wound(s)	0.011
Marshall Islands	2010	Open wound(s)	0.011
Marshall Islands	2017	Open wound(s)	0.011
Marshall Islands	1990	Poisoning requiring urgent care	0.1628
Marshall Islands	1995	Poisoning requiring urgent care	0.1628
Marshall Islands	2000	Poisoning requiring urgent care	0.1628
Marshall Islands	2005	Poisoning requiring urgent care	0.1628
Marshall Islands	2010	Poisoning requiring urgent care	0.1628
Marshall Islands	2017	Poisoning requiring urgent care	0.1628
Marshall Islands	1990	Severe chest injury	0.0474
Marshall Islands	1995	Severe chest injury	0.0474
Marshall Islands	2000	Severe chest injury	0.0474
Marshall Islands	2005	Severe chest injury	0.0474
Marshall Islands	2010	Severe chest injury	0.0474
Marshall Islands	2017	Severe chest injury	0.0474
Marshall Islands	1990	Internal hemorrhage in abdomen and pelvis	0.1143
Marshall Islands	1995	Internal hemorrhage in abdomen and pelvis	0.1143
Marshall Islands	2000	Internal hemorrhage in abdomen and pelvis	0.1143
Marshall Islands	2005	Internal hemorrhage in abdomen and pelvis	0.1143
Marshall Islands	2010	Internal hemorrhage in abdomen and pelvis	0.1143
Marshall Islands	2017	Internal hemorrhage in abdomen and pelvis	0.1143
Marshall Islands	1990	Contusion in any part of the body	0.0075
Marshall Islands	1995	Contusion in any part of the body	0.0075
Marshall Islands	2000	Contusion in any part of the body	0.0075
Marshall Islands	2005	Contusion in any part of the body	0.0075
Marshall Islands	2010	Contusion in any part of the body	0.0075
Marshall Islands	2017	Contusion in any part of the body	0.0075
Marshall Islands	1990	Effect of different environmental factors	0.049
Marshall Islands	1995	Effect of different environmental factors	0.049
Marshall Islands	2000	Effect of different environmental factors	0.049
Marshall Islands	2005	Effect of different environmental factors	0.049
Marshall Islands	2010	Effect of different environmental factors	0.049
Marshall Islands	2017	Effect of different environmental factors	0.049
Marshall Islands	1990	Complications following therapeutic procedures	0.0506
Marshall Islands	1995	Complications following therapeutic procedures	0.0506
Marshall Islands	2000	Complications following therapeutic procedures	0.0506
Marshall Islands	2005	Complications following therapeutic procedures	0.0506
Marshall Islands	2010	Complications following therapeutic procedures	0.0506
Marshall Islands	2017	Complications following therapeutic procedures	0.0506
Marshall Islands	1990	Multiple fractures, dislocations, crashes, wounds, sprains, and strains	0.2445
Marshall Islands	1995	Multiple fractures, dislocations, crashes, wounds, sprains, and strains	0.2381
Marshall Islands	2000	Multiple fractures, dislocations, crashes, wounds, sprains, and strains	0.2379
Marshall Islands	2005	Multiple fractures, dislocations, crashes, wounds, sprains, and strains	0.2262
Marshall Islands	2010	Multiple fractures, dislocations, crashes, wounds, sprains, and strains	0.2164
Marshall Islands	2017	Multiple fractures, dislocations, crashes, wounds, sprains, and strains	0.1983
Marshall Islands	1990	Amputation of upper limb, unilateral	0.0818
Marshall Islands	1995	Amputation of upper limb, unilateral	0.0804
Marshall Islands	2000	Amputation of upper limb, unilateral	0.0803
Marshall Islands	2005	Amputation of upper limb, unilateral	0.0777
Marshall Islands	2010	Amputation of upper limb, unilateral	0.0754
Marshall Islands	2017	Amputation of upper limb, unilateral	0.0712
Marshall Islands	1990	Amputation of thumb	0.0114
Marshall Islands	1995	Amputation of thumb	0.0114
Marshall Islands	2000	Amputation of thumb	0.0114
Marshall Islands	2005	Amputation of thumb	0.0114
Marshall Islands	2010	Amputation of thumb	0.0114
Marshall Islands	2017	Amputation of thumb	0.0114
Marshall Islands	1990	Amputation of toe/toes	0.0059
Marshall Islands	1995	Amputation of toe/toes	0.0059
Marshall Islands	2000	Amputation of toe/toes	0.0059
Marshall Islands	2005	Amputation of toe/toes	0.0059
Marshall Islands	2010	Amputation of toe/toes	0.0059
Marshall Islands	2017	Amputation of toe/toes	0.0059
Marshall Islands	1990	Burns, <20% total burned surface area without lower airway burns	0.0163
Marshall Islands	1995	Burns, <20% total burned surface area without lower airway burns	0.0163
Marshall Islands	2000	Burns, <20% total burned surface area without lower airway burns	0.0163
Marshall Islands	2005	Burns, <20% total burned surface area without lower airway burns	0.0163
Marshall Islands	2010	Burns, <20% total burned surface area without lower airway burns	0.0163
Marshall Islands	2017	Burns, <20% total burned surface area without lower airway burns	0.0163
Marshall Islands	1990	Burns, >=20% total burned surface area or >= 10% burned surface area if head/neck or hands/wrist involved w/o lower airway burns	0.3085
Marshall Islands	1995	Burns, >=20% total burned surface area or >= 10% burned surface area if head/neck or hands/wrist involved w/o lower airway burns	0.3026
Marshall Islands	2000	Burns, >=20% total burned surface area or >= 10% burned surface area if head/neck or hands/wrist involved w/o lower airway burns	0.3024
Marshall Islands	2005	Burns, >=20% total burned surface area or >= 10% burned surface area if head/neck or hands/wrist involved w/o lower airway burns	0.2915
Marshall Islands	2010	Burns, >=20% total burned surface area or >= 10% burned surface area if head/neck or hands/wrist involved w/o lower airway burns	0.2824
Marshall Islands	2017	Burns, >=20% total burned surface area or >= 10% burned surface area if head/neck or hands/wrist involved w/o lower airway burns	0.2655
Federated States of Micronesia	1990	Amputation of lower limbs, bilateral	0.282
Federated States of Micronesia	1995	Amputation of lower limbs, bilateral	0.2727
Federated States of Micronesia	2000	Amputation of lower limbs, bilateral	0.2666
Federated States of Micronesia	2005	Amputation of lower limbs, bilateral	0.2505
Federated States of Micronesia	2010	Amputation of lower limbs, bilateral	0.2477
Federated States of Micronesia	2017	Amputation of lower limbs, bilateral	0.2346
Federated States of Micronesia	1990	Lower airway burns	0.2252
Federated States of Micronesia	1995	Lower airway burns	0.2252

Federated States of Micronesia	2000	Lower airway burns	0.2252
Federated States of Micronesia	2005	Lower airway burns	0.2252
Federated States of Micronesia	2010	Lower airway burns	0.2252
Federated States of Micronesia	2017	Lower airway burns	0.2252
Federated States of Micronesia	1990	Dislocation of hip	0.0159
Federated States of Micronesia	1995	Dislocation of hip	0.0159
Federated States of Micronesia	2000	Dislocation of hip	0.0159
Federated States of Micronesia	2005	Dislocation of hip	0.0159
Federated States of Micronesia	2010	Dislocation of hip	0.0159
Federated States of Micronesia	2017	Dislocation of hip	0.0159
Federated States of Micronesia	1990	Dislocation of knee	0.1134
Federated States of Micronesia	1995	Dislocation of knee	0.1134
Federated States of Micronesia	2000	Dislocation of knee	0.1134
Federated States of Micronesia	2005	Dislocation of knee	0.1134
Federated States of Micronesia	2010	Dislocation of knee	0.1134
Federated States of Micronesia	2017	Dislocation of knee	0.1134
Federated States of Micronesia	1990	Dislocation of shoulder	0.062
Federated States of Micronesia	1995	Dislocation of shoulder	0.062
Federated States of Micronesia	2000	Dislocation of shoulder	0.062
Federated States of Micronesia	2005	Dislocation of shoulder	0.062
Federated States of Micronesia	2010	Dislocation of shoulder	0.062
Federated States of Micronesia	2017	Dislocation of shoulder	0.062
Federated States of Micronesia	1990	Muscle and tendon injuries, including sprains and strains lesser dislocations	0.0075
Federated States of Micronesia	1995	Muscle and tendon injuries, including sprains and strains lesser dislocations	0.0075
Federated States of Micronesia	2000	Muscle and tendon injuries, including sprains and strains lesser dislocations	0.0075
Federated States of Micronesia	2005	Muscle and tendon injuries, including sprains and strains lesser dislocations	0.0075
Federated States of Micronesia	2010	Muscle and tendon injuries, including sprains and strains lesser dislocations	0.0075
Federated States of Micronesia	2017	Muscle and tendon injuries, including sprains and strains lesser dislocations	0.0075
Federated States of Micronesia	1990	Fracture of clavicle, scapula, or humerus	0.0349
Federated States of Micronesia	1995	Fracture of clavicle, scapula, or humerus	0.0349
Federated States of Micronesia	2000	Fracture of clavicle, scapula, or humerus	0.0349
Federated States of Micronesia	2005	Fracture of clavicle, scapula, or humerus	0.0349
Federated States of Micronesia	2010	Fracture of clavicle, scapula, or humerus	0.0349
Federated States of Micronesia	2017	Fracture of clavicle, scapula, or humerus	0.0349
Federated States of Micronesia	1990	Fracture of face bones	0.0669
Federated States of Micronesia	1995	Fracture of face bones	0.0669
Federated States of Micronesia	2000	Fracture of face bones	0.0669
Federated States of Micronesia	2005	Fracture of face bones	0.0669
Federated States of Micronesia	2010	Fracture of face bones	0.0669
Federated States of Micronesia	2017	Fracture of face bones	0.0669
Federated States of Micronesia	1990	Fracture of foot bones except ankle	0.0264
Federated States of Micronesia	1995	Fracture of foot bones except ankle	0.0264
Federated States of Micronesia	2000	Fracture of foot bones except ankle	0.0264
Federated States of Micronesia	2005	Fracture of foot bones except ankle	0.0264
Federated States of Micronesia	2010	Fracture of foot bones except ankle	0.0264
Federated States of Micronesia	2017	Fracture of foot bones except ankle	0.0264
Federated States of Micronesia	1990	Fracture of hand (wrist and other distal part of hand)	0.0138
Federated States of Micronesia	1995	Fracture of hand (wrist and other distal part of hand)	0.0138
Federated States of Micronesia	2000	Fracture of hand (wrist and other distal part of hand)	0.0138
Federated States of Micronesia	2005	Fracture of hand (wrist and other distal part of hand)	0.0138
Federated States of Micronesia	2010	Fracture of hand (wrist and other distal part of hand)	0.0138
Federated States of Micronesia	2017	Fracture of hand (wrist and other distal part of hand)	0.0138
Federated States of Micronesia	1990	Fracture of hip	0.2676
Federated States of Micronesia	1995	Fracture of hip	0.2554
Federated States of Micronesia	2000	Fracture of hip	0.2473
Federated States of Micronesia	2005	Fracture of hip	0.2261
Federated States of Micronesia	2010	Fracture of hip	0.2223
Federated States of Micronesia	2017	Fracture of hip	0.2049
Federated States of Micronesia	1990	Amputation of upper limbs, bilateral	0.3044
Federated States of Micronesia	1995	Amputation of upper limbs, bilateral	0.2918
Federated States of Micronesia	2000	Amputation of upper limbs, bilateral	0.2835
Federated States of Micronesia	2005	Amputation of upper limbs, bilateral	0.2615
Federated States of Micronesia	2010	Amputation of upper limbs, bilateral	0.2576
Federated States of Micronesia	2017	Amputation of upper limbs, bilateral	0.2397
Federated States of Micronesia	1990	Fracture of patella, tibia or fibula, or ankle	0.0554
Federated States of Micronesia	1995	Fracture of patella, tibia or fibula, or ankle	0.0554
Federated States of Micronesia	2000	Fracture of patella, tibia or fibula, or ankle	0.0554
Federated States of Micronesia	2005	Fracture of patella, tibia or fibula, or ankle	0.0554
Federated States of Micronesia	2010	Fracture of patella, tibia or fibula, or ankle	0.0554
Federated States of Micronesia	2017	Fracture of patella, tibia or fibula, or ankle	0.0554
Federated States of Micronesia	1990	Fracture of pelvis	0.1823
Federated States of Micronesia	1995	Fracture of pelvis	0.1823
Federated States of Micronesia	2000	Fracture of pelvis	0.1823
Federated States of Micronesia	2005	Fracture of pelvis	0.1823
Federated States of Micronesia	2010	Fracture of pelvis	0.1823
Federated States of Micronesia	2017	Fracture of pelvis	0.1823
Federated States of Micronesia	1990	Fracture of radius and/or ulna	0.0435
Federated States of Micronesia	1995	Fracture of radius and/or ulna	0.0435
Federated States of Micronesia	2000	Fracture of radius and/or ulna	0.0435
Federated States of Micronesia	2005	Fracture of radius and/or ulna	0.0435
Federated States of Micronesia	2010	Fracture of radius and/or ulna	0.0435
Federated States of Micronesia	2017	Fracture of radius and/or ulna	0.0435
Federated States of Micronesia	1990	Fracture of skull	0.0714
Federated States of Micronesia	1995	Fracture of skull	0.0714
Federated States of Micronesia	2000	Fracture of skull	0.0714
Federated States of Micronesia	2005	Fracture of skull	0.0714
Federated States of Micronesia	2010	Fracture of skull	0.0714
Federated States of Micronesia	2017	Fracture of skull	0.0714
Federated States of Micronesia	1990	Fracture of sternum and/or fracture of one or more ribs	0.1027
Federated States of Micronesia	1995	Fracture of sternum and/or fracture of one or more ribs	0.1027
Federated States of Micronesia	2000	Fracture of sternum and/or fracture of one or more ribs	0.1027
Federated States of Micronesia	2005	Fracture of sternum and/or fracture of one or more ribs	0.1027
Federated States of Micronesia	2010	Fracture of sternum and/or fracture of one or more ribs	0.1027
Federated States of Micronesia	2017	Fracture of sternum and/or fracture of one or more ribs	0.1027
Federated States of Micronesia	1990	Fracture of vertebral column	0.1106
Federated States of Micronesia	1995	Fracture of vertebral column	0.1106
Federated States of Micronesia	2000	Fracture of vertebral column	0.1106
Federated States of Micronesia	2005	Fracture of vertebral column	0.1106
Federated States of Micronesia	2010	Fracture of vertebral column	0.1106
Federated States of Micronesia	2017	Fracture of vertebral column	0.1106
Federated States of Micronesia	1990	Fracture of femur, other than femoral neck	0.042
Federated States of Micronesia	1995	Fracture of femur, other than femoral neck	0.042
Federated States of Micronesia	2000	Fracture of femur, other than femoral neck	0.042
Federated States of Micronesia	2005	Fracture of femur, other than femoral neck	0.042
Federated States of Micronesia	2010	Fracture of femur, other than femoral neck	0.042
Federated States of Micronesia	2017	Fracture of femur, other than femoral neck	0.042
Federated States of Micronesia	1990	Minor TBI	0.1319
Federated States of Micronesia	1995	Minor TBI	0.1319
Federated States of Micronesia	2000	Minor TBI	0.1319
Federated States of Micronesia	2005	Minor TBI	0.1319
Federated States of Micronesia	2010	Minor TBI	0.1319
Federated States of Micronesia	2017	Minor TBI	0.1319
Federated States of Micronesia	1990	Moderate TBI	0.1637
Federated States of Micronesia	1995	Moderate TBI	0.1637
Federated States of Micronesia	2000	Moderate TBI	0.1637
Federated States of Micronesia	2005	Moderate TBI	0.1637
Federated States of Micronesia	2010	Moderate TBI	0.1637
Federated States of Micronesia	2017	Moderate TBI	0.1637
Federated States of Micronesia	1990	Amputation of fingers (excluding thumb)	0.0047
Federated States of Micronesia	1995	Amputation of fingers (excluding thumb)	0.0047
Federated States of Micronesia	2000	Amputation of fingers (excluding thumb)	0.0047
Federated States of Micronesia	2005	Amputation of fingers (excluding thumb)	0.0047
Federated States of Micronesia	2010	Amputation of fingers (excluding thumb)	0.0047
Federated States of Micronesia	2017	Amputation of fingers (excluding thumb)	0.0047
Federated States of Micronesia	1990	Spinal cord lesion at neck level	0.4844
Federated States of Micronesia	1995	Spinal cord lesion at neck level	0.4802
Federated States of Micronesia	2000	Spinal cord lesion at neck level	0.4774
Federated States of Micronesia	2005	Spinal cord lesion at neck level	0.47
Federated States of Micronesia	2010	Spinal cord lesion at neck level	0.4687
Federated States of Micronesia	2017	Spinal cord lesion at neck level	0.4627
Federated States of Micronesia	1990	Spinal cord lesion below neck level	0.3309
Federated States of Micronesia	1995	Spinal cord lesion below neck level	0.322
Federated States of Micronesia	2000	Spinal cord lesion below neck level	0.3161
Federated States of Micronesia	2005	Spinal cord lesion below neck level	0.3006
Federated States of Micronesia	2010	Spinal cord lesion below neck level	0.2978
Federated States of Micronesia	2017	Spinal cord lesion below neck level	0.2851
Federated States of Micronesia	1990	Drowning and nonfatal submersion	0.2471
Federated States of Micronesia	1995	Drowning and nonfatal submersion	0.2471
Federated States of Micronesia	2000	Drowning and nonfatal submersion	0.2471
Federated States of Micronesia	2005	Drowning and nonfatal submersion	0.2471
Federated States of Micronesia	2010	Drowning and nonfatal submersion	0.2471
Federated States of Micronesia	2017	Drowning and nonfatal submersion	0.2471
Federated States of Micronesia	1990	Asphyxiation	0.2471
Federated States of Micronesia	1995	Asphyxiation	0.2471
Federated States of Micronesia	2000	Asphyxiation	0.2471
Federated States of Micronesia	2005	Asphyxiation	0.2471
Federated States of Micronesia	2010	Asphyxiation	0.2471
Federated States of Micronesia	2017	Asphyxiation	0.2471
Federated States of Micronesia	1990	Crush injury	0.1325
Federated States of Micronesia	1995	Crush injury	0.1325
Federated States of Micronesia	2000	Crush injury	0.1325
Federated States of Micronesia	2005	Crush injury	0.1325
Federated States of Micronesia	2010	Crush injury	0.1325
Federated States of Micronesia	2017	Crush injury	0.1325
Federated States of Micronesia	1990	Nerve injury	0.1129
Federated States of Micronesia	1995	Nerve injury	0.1129
Federated States of Micronesia	2000	Nerve injury	0.1129
Federated States of Micronesia	2005	Nerve injury	0.1129
Federated States of Micronesia	2010	Nerve injury	0.1129
Federated States of Micronesia	2017	Nerve injury	0.1129
Federated States of Micronesia	1990	Injury to eyes	0.0165
Federated States of Micronesia	1995	Injury to eyes	0.0165
Federated States of Micronesia	2000	Injury to eyes	0.0165
Federated States of Micronesia	2005	Injury to eyes	0.0165
Federated States of Micronesia	2010	Injury to eyes	0.0165
Federated States of Micronesia	2017	Injury to eyes	0.0165
Federated States of Micronesia	1990	Amputation of lower limb, unilateral	0.1208
Federated States of Micronesia	1995	Amputation of lower limb, unilateral	0.116
Federated States of Micronesia	2000	Amputation of lower limb, unilateral	0.1129
Federated States of Micronesia	2005	Amputation of lower limb, unilateral	0.1046
Federated States of Micronesia	2010	Amputation of lower limb, unilateral	0.1031
Federated States of Micronesia	2017	Amputation of lower limb, unilateral	0.0963
Federated States of Micronesia	1990	Open wound(s)	0.011
Federated States of Micronesia	1995	Open wound(s)	0.011
Federated States of Micronesia	2000	Open wound(s)	0.011
Federated States of Micronesia	2005	Open wound(s)	0.011
Federated States of Micronesia	2010	Open wound(s)	0.011
Federated States of Micronesia	2017	Open wound(s)	0.011
Federated States of Micronesia	1990	Poisoning requiring urgent care	0.1628
Federated States of Micronesia	1995	Poisoning requiring urgent care	0.1628
Federated States of Micronesia	2000	Poisoning requiring urgent care	0.1628
Federated States of Micronesia	2005	Poisoning requiring urgent care	0.1628
Federated States of Micronesia	2010	Poisoning requiring urgent care	0.1628
Federated States of Micronesia	2017	Poisoning requiring urgent care	0.1628
Federated States of Micronesia	1990	Severe chest injury	0.0474
Federated States of Micronesia	1995	Severe chest injury	0.0474
Federated States of Micronesia	2000	Severe chest injury	0.0474
Federated States of Micronesia	2005	Severe chest injury	0.0474
Federated States of Micronesia	2010	Severe chest injury	0.0474
Federated States of Micronesia	2017	Severe chest injury	0.0474
Federated States of Micronesia	1990	Internal hemorrhage in abdomen and pelvis	0.1143
Federated States of Micronesia	1995	Internal hemorrhage in abdomen and pelvis	0.1143
Federated States of Micronesia	2000	Internal hemorrhage in abdomen and pelvis	0.1143
Federated States of Micronesia	2005	Internal hemorrhage in abdomen and pelvis	0.1143
Federated States of Micronesia	2010	Internal hemorrhage in abdomen and pelvis	0.1143
Federated States of Micronesia	2017	Internal hemorrhage in abdomen and pelvis	0.1143
Federated States of Micronesia	1990	Contusion in any part of the body	0.0075
Federated States of Micronesia	1995	Contusion in any part of the body	0.0075
Federated States of Micronesia	2000	Contusion in any part of the body	0.0075
Federated States of Micronesia	2005	Contusion in any part of the body	0.0075
Federated States of Micronesia	2010	Contusion in any part of the body	0.0075
Federated States of Micronesia	2017	Contusion in any part of the body	0.0075
Federated States of Micronesia	1990	Effect of different environmental factors	0.049
Federated States of Micronesia	1995	Effect of different environmental factors	0.049
Federated States of Micronesia	2000	Effect of different environmental factors	0.049
Federated States of Micronesia	2005	Effect of different environmental factors	0.049
Federated States of Micronesia	2010	Effect of different environmental factors	0.049
Federated States of Micronesia	2017	Effect of different environmental factors	0.049
Federated States of Micronesia	1990	Complications following therapeutic procedures	0.0506
Federated States of Micronesia	1995	Complications following therapeutic procedures	0.0506
Federated States of Micronesia	2000	Complications following therapeutic procedures	0.0506
Federated States of Micronesia	2005	Complications following therapeutic procedures	0.0506
Federated States of Micronesia	2010	Complications following therapeutic procedures	0.0506
Federated States of Micronesia	2017	Complications following therapeutic procedures	0.0506
Federated States of Micronesia	1990	Multiple fractures, dislocations, crashes, wounds, sprains, and strains	0.2676
Federated States of Micronesia	1995	Multiple fractures, dislocations, crashes, wounds, sprains, and strains	0.2554
Federated States of Micronesia	2000	Multiple fractures, dislocations, crashes, wounds, sprains, and strains	0.2473
Federated States of Micronesia	2005	Multiple fractures, dislocations, crashes, wounds, sprains, and strains	0.2261
Federated States of Micronesia	2010	Multiple fractures, dislocations, crashes, wounds, sprains, and strains	0.2223
Federated States of Micronesia	2017	Multiple fractures, dislocations, crashes, wounds, sprains, and strains	0.2049
Federated States of Micronesia	1990	Amputation of upper limb, unilateral	0.0871
Federated States of Micronesia	1995	Amputation of upper limb, unilateral	0.0843
Federated States of Micronesia	2000	Amputation of upper limb, unilateral	0.0825
Federated States of Micronesia	2005	Amputation of upper limb, unilateral	0.0776
Federated States of Micronesia	2010	Amputation of upper limb, unilateral	0.0767
Federated States of Micronesia	2017	Amputation of upper limb, unilateral	0.0728
Federated States of Micronesia	1990	Amputation of thumb	0.0114
Federated States of Micronesia	1995	Amputation of thumb	0.0114
Federated States of Micronesia	2000	Amputation of thumb	0.0114
Federated States of Micronesia	2005	Amputation of thumb	0.0114
Federated States of Micronesia	2010	Amputation of thumb	0.0114
Federated States of Micronesia	2017	Amputation of thumb	0.0114
Federated States of Micronesia	1990	Amputation of toe/toes	0.0059
Federated States of Micronesia	1995	Amputation of toe/toes	0.0059
Federated States of Micronesia	2000	Amputation of toe/toes	0.0059
Federated States of Micronesia	2005	Amputation of toe/toes	0.0059
Federated States of Micronesia	2010	Amputation of toe/toes	0.0059
Federated States of Micronesia	2017	Amputation of toe/toes	0.0059
Federated States of Micronesia	1990	Burns, <20% total burned surface area without lower airway burns	0.0163
Federated States of Micronesia	1995	Burns, <20% total burned surface area without lower airway burns	0.0163
Federated States of Micronesia	2000	Burns, <20% total burned surface area without lower airway burns	0.0163
Federated States of Micronesia	2005	Burns, <20% total burned surface area without lower airway burns	0.0163
Federated States of Micronesia	2010	Burns, <20% total burned surface area without lower airway burns	0.0163
Federated States of Micronesia	2017	Burns, <20% total burned surface area without lower airway burns	0.0163
Federated States of Micronesia	1990	Burns, >=20% total burned surface area or >= 10% burned surface area if head/neck or hands/wrist involved w/o lower airway burns	0.3301
Federated States of Micronesia	1995	Burns, >=20% total burned surface area or >= 10% burned surface area if head/neck or hands/wrist involved w/o lower airway burns	0.3187
Federated States of Micronesia	2000	Burns, >=20% total burned surface area or >= 10% burned surface area if head/neck or hands/wrist involved w/o lower airway burns	0.3112
Federated States of Micronesia	2005	Burns, >=20% total burned surface area or >= 10% burned surface area if head/neck or hands/wrist involved w/o lower airway burns	0.2914
Federated States of Micronesia	2010	Burns, >=20% total burned surface area or >= 10% burned surface area if head/neck or hands/wrist involved w/o lower airway burns	0.2879
Federated States of Micronesia	2017	Burns, >=20% total burned surface area or >= 10% burned surface area if head/neck or hands/wrist involved w/o lower airway burns	0.2717
Papua New Guinea	1990	Amputation of lower limbs, bilateral	0.2991
Papua New Guinea	1995	Amputation of lower limbs, bilateral	0.2922
Papua New Guinea	2000	Amputation of lower limbs, bilateral	0.2804
Papua New Guinea	2005	Amputation of lower limbs, bilateral	0.2794
Papua New Guinea	2010	Amputation of lower limbs, bilateral	0.2785
Papua New Guinea	2017	Amputation of lower limbs, bilateral	0.2672
Papua New Guinea	1990	Lower airway burns	0.2252
Papua New Guinea	1995	Lower airway burns	0.2252
Papua New Guinea	2000	Lower airway burns	0.2252
Papua New Guinea	2005	Lower airway burns	0.2252
Papua New Guinea	2010	Lower airway burns	0.2252
Papua New Guinea	2017	Lower airway burns	0.2252
Papua New Guinea	1990	Dislocation of hip	0.0159
Papua New Guinea	1995	Dislocation of hip	0.0159
Papua New Guinea	2000	Dislocation of hip	0.0159
Papua New Guinea	2005	Dislocation of hip	0.0159
Papua New Guinea	2010	Dislocation of hip	0.0159
Papua New Guinea	2017	Dislocation of hip	0.0159
Papua New Guinea	1990	Dislocation of knee	0.1134
Papua New Guinea	1995	Dislocation of knee	0.1134
Papua New Guinea	2000	Dislocation of knee	0.1134
Papua New Guinea	2005	Dislocation of knee	0.1134
Papua New Guinea	2010	Dislocation of knee	0.1134
Papua New Guinea	2017	Dislocation of knee	0.1134
Papua New Guinea	1990	Dislocation of shoulder	0.062
Papua New Guinea	1995	Dislocation of shoulder	0.062
Papua New Guinea	2000	Dislocation of shoulder	0.062
Papua New Guinea	2005	Dislocation of shoulder	0.062
Papua New Guinea	2010	Dislocation of shoulder	0.062
Papua New Guinea	2017	Dislocation of shoulder	0.062
Papua New Guinea	1990	Muscle and tendon injuries, including sprains and strains lesser dislocations	0.0075
Papua New Guinea	1995	Muscle and tendon injuries, including sprains and strains lesser dislocations	0.0075
Papua New Guinea	2000	Muscle and tendon injuries, including sprains and strains lesser dislocations	0.0075
Papua New Guinea	2005	Muscle and tendon injuries, including sprains and strains lesser dislocations	0.0075
Papua New Guinea	2010	Muscle and tendon injuries, including sprains and strains lesser dislocations	0.0075
Papua New Guinea	2017	Muscle and tendon injuries, including sprains and strains lesser dislocations	0.0075
Papua New Guinea	1990	Fracture of clavicle, scapula, or humerus	0.0349
Papua New Guinea	1995	Fracture of clavicle, scapula, or humerus	0.0349
Papua New Guinea	2000	Fracture of clavicle, scapula, or humerus	0.0349
Papua New Guinea	2005	Fracture of clavicle, scapula, or humerus	0.0349
Papua New Guinea	2010	Fracture of clavicle, scapula, or humerus	0.0349
Papua New Guinea	2017	Fracture of clavicle, scapula, or humerus	0.0349
Papua New Guinea	1990	Fracture of face bones	0.0669
Papua New Guinea	1995	Fracture of face bones	0.0669
Papua New Guinea	2000	Fracture of face bones	0.0669
Papua New Guinea	2005	Fracture of face bones	0.0669
Papua New Guinea	2010	Fracture of face bones	0.0669
Papua New Guinea	2017	Fracture of face bones	0.0669
Papua New Guinea	1990	Fracture of foot bones except ankle	0.0264
Papua New Guinea	1995	Fracture of foot bones except ankle	0.0264
Papua New Guinea	2000	Fracture of foot bones except ankle	0.0264
Papua New Guinea	2005	Fracture of foot bones except ankle	0.0264
Papua New Guinea	2010	Fracture of foot bones except ankle	0.0264
Papua New Guinea	2017	Fracture of foot bones except ankle	0.0264
Papua New Guinea	1990	Fracture of hand (wrist and other distal part of hand)	0.0138
Papua New Guinea	1995	Fracture of hand (wrist and other distal part of hand)	0.0138
Papua New Guinea	2000	Fracture of hand (wrist and other distal part of hand)	0.0138
Papua New Guinea	2005	Fracture of hand (wrist and other distal part of hand)	0.0138
Papua New Guinea	2010	Fracture of hand (wrist and other distal part of hand)	0.0138
Papua New Guinea	2017	Fracture of hand (wrist and other distal part of hand)	0.0138
Papua New Guinea	1990	Fracture of hip	0.2903
Papua New Guinea	1995	Fracture of hip	0.2811
Papua New Guinea	2000	Fracture of hip	0.2656
Papua New Guinea	2005	Fracture of hip	0.2642
Papua New Guinea	2010	Fracture of hip	0.2631
Papua New Guinea	2017	Fracture of hip	0.248
Papua New Guinea	1990	Amputation of upper limbs, bilateral	0.3278
Papua New Guinea	1995	Amputation of upper limbs, bilateral	0.3184
Papua New Guinea	2000	Amputation of upper limbs, bilateral	0.3023
Papua New Guinea	2005	Amputation of upper limbs, bilateral	0.3009
Papua New Guinea	2010	Amputation of upper limbs, bilateral	0.2997
Papua New Guinea	2017	Amputation of upper limbs, bilateral	0.2842
Papua New Guinea	1990	Fracture of patella, tibia or fibula, or ankle	0.0554
Papua New Guinea	1995	Fracture of patella, tibia or fibula, or ankle	0.0554
Papua New Guinea	2000	Fracture of patella, tibia or fibula, or ankle	0.0554
Papua New Guinea	2005	Fracture of patella, tibia or fibula, or ankle	0.0554
Papua New Guinea	2010	Fracture of patella, tibia or fibula, or ankle	0.0554
Papua New Guinea	2017	Fracture of patella, tibia or fibula, or ankle	0.0554
Papua New Guinea	1990	Fracture of pelvis	0.1823
Papua New Guinea	1995	Fracture of pelvis	0.1823
Papua New Guinea	2000	Fracture of pelvis	0.1823
Papua New Guinea	2005	Fracture of pelvis	0.1823
Papua New Guinea	2010	Fracture of pelvis	0.1823
Papua New Guinea	2017	Fracture of pelvis	0.1823
Papua New Guinea	1990	Fracture of radius and/or ulna	0.0435
Papua New Guinea	1995	Fracture of radius and/or ulna	0.0435
Papua New Guinea	2000	Fracture of radius and/or ulna	0.0435
Papua New Guinea	2005	Fracture of radius and/or ulna	0.0435
Papua New Guinea	2010	Fracture of radius and/or ulna	0.0435
Papua New Guinea	2017	Fracture of radius and/or ulna	0.0435
Papua New Guinea	1990	Fracture of skull	0.0714
Papua New Guinea	1995	Fracture of skull	0.0714
Papua New Guinea	2000	Fracture of skull	0.0714
Papua New Guinea	2005	Fracture of skull	0.0714
Papua New Guinea	2010	Fracture of skull	0.0714
Papua New Guinea	2017	Fracture of skull	0.0714
Papua New Guinea	1990	Fracture of sternum and/or fracture of one or more ribs	0.1027
Papua New Guinea	1995	Fracture of sternum and/or fracture of one or more ribs	0.1027
Papua New Guinea	2000	Fracture of sternum and/or fracture of one or more ribs	0.1027
Papua New Guinea	2005	Fracture of sternum and/or fracture of one or more ribs	0.1027
Papua New Guinea	2010	Fracture of sternum and/or fracture of one or more ribs	0.1027
Papua New Guinea	2017	Fracture of sternum and/or fracture of one or more ribs	0.1027
Papua New Guinea	1990	Fracture of vertebral column	0.1106
Papua New Guinea	1995	Fracture of vertebral column	0.1106
Papua New Guinea	2000	Fracture of vertebral column	0.1106
Papua New Guinea	2005	Fracture of vertebral column	0.1106
Papua New Guinea	2010	Fracture of vertebral column	0.1106
Papua New Guinea	2017	Fracture of vertebral column	0.1106
Papua New Guinea	1990	Fracture of femur, other than femoral neck	0.042
Papua New Guinea	1995	Fracture of femur, other than femoral neck	0.042
Papua New Guinea	2000	Fracture of femur, other than femoral neck	0.042
Papua New Guinea	2005	Fracture of femur, other than femoral neck	0.042
Papua New Guinea	2010	Fracture of femur, other than femoral neck	0.042
Papua New Guinea	2017	Fracture of femur, other than femoral neck	0.042
Papua New Guinea	1990	Minor TBI	0.1319
Papua New Guinea	1995	Minor TBI	0.1319
Papua New Guinea	2000	Minor TBI	0.1319
Papua New Guinea	2005	Minor TBI	0.1319
Papua New Guinea	2010	Minor TBI	0.1319
Papua New Guinea	2017	Minor TBI	0.1319
Papua New Guinea	1990	Moderate TBI	0.1637
Papua New Guinea	1995	Moderate TBI	0.1637
Papua New Guinea	2000	Moderate TBI	0.1637
Papua New Guinea	2005	Moderate TBI	0.1637
Papua New Guinea	2010	Moderate TBI	0.1637
Papua New Guinea	2017	Moderate TBI	0.1637
Papua New Guinea	1990	Amputation of fingers (excluding thumb)	0.0047
Papua New Guinea	1995	Amputation of fingers (excluding thumb)	0.0047
Papua New Guinea	2000	Amputation of fingers (excluding thumb)	0.0047
Papua New Guinea	2005	Amputation of fingers (excluding thumb)	0.0047
Papua New Guinea	2010	Amputation of fingers (excluding thumb)	0.0047
Papua New Guinea	2017	Amputation of fingers (excluding thumb)	0.0047
Papua New Guinea	1990	Spinal cord lesion at neck level	0.4923
Papua New Guinea	1995	Spinal cord lesion at neck level	0.4891
Papua New Guinea	2000	Spinal cord lesion at neck level	0.4837
Papua New Guinea	2005	Spinal cord lesion at neck level	0.4832
Papua New Guinea	2010	Spinal cord lesion at neck level	0.4828
Papua New Guinea	2017	Spinal cord lesion at neck level	0.4777
Papua New Guinea	1990	Spinal cord lesion below neck level	0.3474
Papua New Guinea	1995	Spinal cord lesion below neck level	0.3407
Papua New Guinea	2000	Spinal cord lesion below neck level	0.3294
Papua New Guinea	2005	Spinal cord lesion below neck level	0.3284
Papua New Guinea	2010	Spinal cord lesion below neck level	0.3276
Papua New Guinea	2017	Spinal cord lesion below neck level	0.3166
Papua New Guinea	1990	Drowning and nonfatal submersion	0.2471
Papua New Guinea	1995	Drowning and nonfatal submersion	0.2471
Papua New Guinea	2000	Drowning and nonfatal submersion	0.2471
Papua New Guinea	2005	Drowning and nonfatal submersion	0.2471
Papua New Guinea	2010	Drowning and nonfatal submersion	0.2471
Papua New Guinea	2017	Drowning and nonfatal submersion	0.2471
Papua New Guinea	1990	Asphyxiation	0.2471
Papua New Guinea	1995	Asphyxiation	0.2471
Papua New Guinea	2000	Asphyxiation	0.2471
Papua New Guinea	2005	Asphyxiation	0.2471
Papua New Guinea	2010	Asphyxiation	0.2471
Papua New Guinea	2017	Asphyxiation	0.2471
Papua New Guinea	1990	Crush injury	0.1325
Papua New Guinea	1995	Crush injury	0.1325
Papua New Guinea	2000	Crush injury	0.1325
Papua New Guinea	2005	Crush injury	0.1325
Papua New Guinea	2010	Crush injury	0.1325
Papua New Guinea	2017	Crush injury	0.1325
Papua New Guinea	1990	Nerve injury	0.1129
Papua New Guinea	1995	Nerve injury	0.1129
Papua New Guinea	2000	Nerve injury	0.1129
Papua New Guinea	2005	Nerve injury	0.1129
Papua New Guinea	2010	Nerve injury	0.1129
Papua New Guinea	2017	Nerve injury	0.1129
Papua New Guinea	1990	Injury to eyes	0.0165
Papua New Guinea	1995	Injury to eyes	0.0165
Papua New Guinea	2000	Injury to eyes	0.0165
Papua New Guinea	2005	Injury to eyes	0.0165
Papua New Guinea	2010	Injury to eyes	0.0165
Papua New Guinea	2017	Injury to eyes	0.0165
Papua New Guinea	1990	Amputation of lower limb, unilateral	0.1296
Papua New Guinea	1995	Amputation of lower limb, unilateral	0.126
Papua New Guinea	2000	Amputation of lower limb, unilateral	0.12
Papua New Guinea	2005	Amputation of lower limb, unilateral	0.1194
Papua New Guinea	2010	Amputation of lower limb, unilateral	0.119
Papua New Guinea	2017	Amputation of lower limb, unilateral	0.1132
Papua New Guinea	1990	Open wound(s)	0.011

Papua New Guinea	1995	Open wound(s)	0.011
Papua New Guinea	2000	Open wound(s)	0.011
Papua New Guinea	2005	Open wound(s)	0.011
Papua New Guinea	2010	Open wound(s)	0.011
Papua New Guinea	2017	Open wound(s)	0.011
Papua New Guinea	1990	Poisoning requiring urgent care	0.1628
Papua New Guinea	1995	Poisoning requiring urgent care	0.1628
Papua New Guinea	2000	Poisoning requiring urgent care	0.1628
Papua New Guinea	2005	Poisoning requiring urgent care	0.1628
Papua New Guinea	2010	Poisoning requiring urgent care	0.1628
Papua New Guinea	2017	Poisoning requiring urgent care	0.1628
Papua New Guinea	1990	Severe chest injury	0.0474
Papua New Guinea	1995	Severe chest injury	0.0474
Papua New Guinea	2000	Severe chest injury	0.0474
Papua New Guinea	2005	Severe chest injury	0.0474
Papua New Guinea	2010	Severe chest injury	0.0474
Papua New Guinea	2017	Severe chest injury	0.0474
Papua New Guinea	1990	Internal hemorrhage in abdomen and pelvis	0.1143
Papua New Guinea	1995	Internal hemorrhage in abdomen and pelvis	0.1143
Papua New Guinea	2000	Internal hemorrhage in abdomen and pelvis	0.1143
Papua New Guinea	2005	Internal hemorrhage in abdomen and pelvis	0.1143
Papua New Guinea	2010	Internal hemorrhage in abdomen and pelvis	0.1143
Papua New Guinea	2017	Internal hemorrhage in abdomen and pelvis	0.1143
Papua New Guinea	1990	Contusion in any part of the body	0.0075
Papua New Guinea	1995	Contusion in any part of the body	0.0075
Papua New Guinea	2000	Contusion in any part of the body	0.0075
Papua New Guinea	2005	Contusion in any part of the body	0.0075
Papua New Guinea	2010	Contusion in any part of the body	0.0075
Papua New Guinea	2017	Contusion in any part of the body	0.0075
Papua New Guinea	1990	Effect of different environmental factors	0.049
Papua New Guinea	1995	Effect of different environmental factors	0.049
Papua New Guinea	2000	Effect of different environmental factors	0.049
Papua New Guinea	2005	Effect of different environmental factors	0.049
Papua New Guinea	2010	Effect of different environmental factors	0.049
Papua New Guinea	2017	Effect of different environmental factors	0.049
Papua New Guinea	1990	Complications following therapeutic procedures	0.0506
Papua New Guinea	1995	Complications following therapeutic procedures	0.0506
Papua New Guinea	2000	Complications following therapeutic procedures	0.0506
Papua New Guinea	2005	Complications following therapeutic procedures	0.0506
Papua New Guinea	2010	Complications following therapeutic procedures	0.0506
Papua New Guinea	2017	Complications following therapeutic procedures	0.0506
Papua New Guinea	1990	Multiple fractures, dislocations, crashes, wounds, sprains, and strains	0.2903
Papua New Guinea	1995	Multiple fractures, dislocations, crashes, wounds, sprains, and strains	0.2811
Papua New Guinea	2000	Multiple fractures, dislocations, crashes, wounds, sprains, and strains	0.2656
Papua New Guinea	2005	Multiple fractures, dislocations, crashes, wounds, sprains, and strains	0.2642
Papua New Guinea	2010	Multiple fractures, dislocations, crashes, wounds, sprains, and strains	0.2631
Papua New Guinea	2017	Multiple fractures, dislocations, crashes, wounds, sprains, and strains	0.248
Papua New Guinea	1990	Amputation of upper limb, unilateral	0.0923
Papua New Guinea	1995	Amputation of upper limb, unilateral	0.0902
Papua New Guinea	2000	Amputation of upper limb, unilateral	0.0867
Papua New Guinea	2005	Amputation of upper limb, unilateral	0.0864
Papua New Guinea	2010	Amputation of upper limb, unilateral	0.0861
Papua New Guinea	2017	Amputation of upper limb, unilateral	0.0827
Papua New Guinea	1990	Amputation of thumb	0.0114
Papua New Guinea	1995	Amputation of thumb	0.0114
Papua New Guinea	2000	Amputation of thumb	0.0114
Papua New Guinea	2005	Amputation of thumb	0.0114
Papua New Guinea	2010	Amputation of thumb	0.0114
Papua New Guinea	2017	Amputation of thumb	0.0114
Papua New Guinea	1990	Amputation of toe/toes	0.0059
Papua New Guinea	1995	Amputation of toe/toes	0.0059
Papua New Guinea	2000	Amputation of toe/toes	0.0059
Papua New Guinea	2005	Amputation of toe/toes	0.0059
Papua New Guinea	2010	Amputation of toe/toes	0.0059
Papua New Guinea	2017	Amputation of toe/toes	0.0059
Papua New Guinea	1990	Burns, <20% total burned surface area without lower airway burns	0.0163
Papua New Guinea	1995	Burns, <20% total burned surface area without lower airway burns	0.0163
Papua New Guinea	2000	Burns, <20% total burned surface area without lower airway burns	0.0163
Papua New Guinea	2005	Burns, <20% total burned surface area without lower airway burns	0.0163
Papua New Guinea	2010	Burns, <20% total burned surface area without lower airway burns	0.0163
Papua New Guinea	2017	Burns, <20% total burned surface area without lower airway burns	0.0163
Papua New Guinea	1990	Burns, >=20% total burned surface area or >= 10% burned surface area if head/neck or hands/wrist involved w/o lower airway burns	0.3512
Papua New Guinea	1995	Burns, >=20% total burned surface area or >= 10% burned surface area if head/neck or hands/wrist involved w/o lower airway burns	0.3427
Papua New Guinea	2000	Burns, >=20% total burned surface area or >= 10% burned surface area if head/neck or hands/wrist involved w/o lower airway burns	0.3282
Papua New Guinea	2005	Burns, >=20% total burned surface area or >= 10% burned surface area if head/neck or hands/wrist involved w/o lower airway burns	0.3269
Papua New Guinea	2010	Burns, >=20% total burned surface area or >= 10% burned surface area if head/neck or hands/wrist involved w/o lower airway burns	0.3259
Papua New Guinea	2017	Burns, >=20% total burned surface area or >= 10% burned surface area if head/neck or hands/wrist involved w/o lower airway burns	0.3119
Samoa	1990	Amputation of lower limbs, bilateral	0.2501
Samoa	1995	Amputation of lower limbs, bilateral	0.2414
Samoa	2000	Amputation of lower limbs, bilateral	0.2292
Samoa	2005	Amputation of lower limbs, bilateral	0.2225
Samoa	2010	Amputation of lower limbs, bilateral	0.2188
Samoa	2017	Amputation of lower limbs, bilateral	0.2142
Samoa	1990	Lower airway burns	0.2252
Samoa	1995	Lower airway burns	0.2252
Samoa	2000	Lower airway burns	0.2252
Samoa	2005	Lower airway burns	0.2252
Samoa	2010	Lower airway burns	0.2252
Samoa	2017	Lower airway burns	0.2252
Samoa	1990	Dislocation of hip	0.0159
Samoa	1995	Dislocation of hip	0.0159
Samoa	2000	Dislocation of hip	0.0159
Samoa	2005	Dislocation of hip	0.0159
Samoa	2010	Dislocation of hip	0.0159
Samoa	2017	Dislocation of hip	0.0159
Samoa	1990	Dislocation of knee	0.1134
Samoa	1995	Dislocation of knee	0.1134
Samoa	2000	Dislocation of knee	0.1134
Samoa	2005	Dislocation of knee	0.1134
Samoa	2010	Dislocation of knee	0.1134
Samoa	2017	Dislocation of knee	0.1134
Samoa	1990	Dislocation of shoulder	0.062
Samoa	1995	Dislocation of shoulder	0.062
Samoa	2000	Dislocation of shoulder	0.062
Samoa	2005	Dislocation of shoulder	0.062
Samoa	2010	Dislocation of shoulder	0.062
Samoa	2017	Dislocation of shoulder	0.062
Samoa	1990	Muscle and tendon injuries, including sprains and strains lesser dislocations	0.0075
Samoa	1995	Muscle and tendon injuries, including sprains and strains lesser dislocations	0.0075
Samoa	2000	Muscle and tendon injuries, including sprains and strains lesser dislocations	0.0075
Samoa	2005	Muscle and tendon injuries, including sprains and strains lesser dislocations	0.0075
Samoa	2010	Muscle and tendon injuries, including sprains and strains lesser dislocations	0.0075
Samoa	2017	Muscle and tendon injuries, including sprains and strains lesser dislocations	0.0075
Samoa	1990	Fracture of clavicle, scapula, or humerus	0.0349
Samoa	1995	Fracture of clavicle, scapula, or humerus	0.0349
Samoa	2000	Fracture of clavicle, scapula, or humerus	0.0349
Samoa	2005	Fracture of clavicle, scapula, or humerus	0.0349
Samoa	2010	Fracture of clavicle, scapula, or humerus	0.0349
Samoa	2017	Fracture of clavicle, scapula, or humerus	0.0349
Samoa	1990	Fracture of face bones	0.0669
Samoa	1995	Fracture of face bones	0.0669
Samoa	2000	Fracture of face bones	0.0669
Samoa	2005	Fracture of face bones	0.0669
Samoa	2010	Fracture of face bones	0.0669
Samoa	2017	Fracture of face bones	0.0669
Samoa	1990	Fracture of foot bones except ankle	0.0264
Samoa	1995	Fracture of foot bones except ankle	0.0264
Samoa	2000	Fracture of foot bones except ankle	0.0264
Samoa	2005	Fracture of foot bones except ankle	0.0264
Samoa	2010	Fracture of foot bones except ankle	0.0264
Samoa	2017	Fracture of foot bones except ankle	0.0264
Samoa	1990	Fracture of hand (wrist and other distal part of hand)	0.0138
Samoa	1995	Fracture of hand (wrist and other distal part of hand)	0.0138
Samoa	2000	Fracture of hand (wrist and other distal part of hand)	0.0138
Samoa	2005	Fracture of hand (wrist and other distal part of hand)	0.0138
Samoa	2010	Fracture of hand (wrist and other distal part of hand)	0.0138
Samoa	2017	Fracture of hand (wrist and other distal part of hand)	0.0138
Samoa	1990	Fracture of hip	0.2255
Samoa	1995	Fracture of hip	0.2139
Samoa	2000	Fracture of hip	0.1979
Samoa	2005	Fracture of hip	0.1889
Samoa	2010	Fracture of hip	0.184
Samoa	2017	Fracture of hip	0.178
Samoa	1990	Amputation of upper limbs, bilateral	0.261
Samoa	1995	Amputation of upper limbs, bilateral	0.249
Samoa	2000	Amputation of upper limbs, bilateral	0.2324
Samoa	2005	Amputation of upper limbs, bilateral	0.2232
Samoa	2010	Amputation of upper limbs, bilateral	0.2181
Samoa	2017	Amputation of upper limbs, bilateral	0.2119
Samoa	1990	Fracture of patella, tibia or fibula, or ankle	0.0554
Samoa	1995	Fracture of patella, tibia or fibula, or ankle	0.0554
Samoa	2000	Fracture of patella, tibia or fibula, or ankle	0.0554
Samoa	2005	Fracture of patella, tibia or fibula, or ankle	0.0554
Samoa	2010	Fracture of patella, tibia or fibula, or ankle	0.0554
Samoa	2017	Fracture of patella, tibia or fibula, or ankle	0.0554
Samoa	1990	Fracture of pelvis	0.1823
Samoa	1995	Fracture of pelvis	0.1823
Samoa	2000	Fracture of pelvis	0.1823
Samoa	2005	Fracture of pelvis	0.1823
Samoa	2010	Fracture of pelvis	0.1823
Samoa	2017	Fracture of pelvis	0.1823
Samoa	1990	Fracture of radius and/or ulna	0.0435
Samoa	1995	Fracture of radius and/or ulna	0.0435
Samoa	2000	Fracture of radius and/or ulna	0.0435
Samoa	2005	Fracture of radius and/or ulna	0.0435
Samoa	2010	Fracture of radius and/or ulna	0.0435
Samoa	2017	Fracture of radius and/or ulna	0.0435
Samoa	1990	Fracture of skull	0.0714
Samoa	1995	Fracture of skull	0.0714
Samoa	2000	Fracture of skull	0.0714
Samoa	2005	Fracture of skull	0.0714
Samoa	2010	Fracture of skull	0.0714
Samoa	2017	Fracture of skull	0.0714
Samoa	1990	Fracture of sternum and/or fracture of one or more ribs	0.1027
Samoa	1995	Fracture of sternum and/or fracture of one or more ribs	0.1027
Samoa	2000	Fracture of sternum and/or fracture of one or more ribs	0.1027
Samoa	2005	Fracture of sternum and/or fracture of one or more ribs	0.1027
Samoa	2010	Fracture of sternum and/or fracture of one or more ribs	0.1027
Samoa	2017	Fracture of sternum and/or fracture of one or more ribs	0.1027
Samoa	1990	Fracture of vertebral column	0.1106
Samoa	1995	Fracture of vertebral column	0.1106
Samoa	2000	Fracture of vertebral column	0.1106
Samoa	2005	Fracture of vertebral column	0.1106
Samoa	2010	Fracture of vertebral column	0.1106
Samoa	2017	Fracture of vertebral column	0.1106
Samoa	1990	Fracture of femur, other than femoral neck	0.042
Samoa	1995	Fracture of femur, other than femoral neck	0.042
Samoa	2000	Fracture of femur, other than femoral neck	0.042
Samoa	2005	Fracture of femur, other than femoral neck	0.042
Samoa	2010	Fracture of femur, other than femoral neck	0.042
Samoa	2017	Fracture of femur, other than femoral neck	0.042
Samoa	1990	Minor TBI	0.1319
Samoa	1995	Minor TBI	0.1319
Samoa	2000	Minor TBI	0.1319
Samoa	2005	Minor TBI	0.1319
Samoa	2010	Minor TBI	0.1319
Samoa	2017	Minor TBI	0.1319
Samoa	1990	Moderate TBI	0.1637
Samoa	1995	Moderate TBI	0.1637
Samoa	2000	Moderate TBI	0.1637
Samoa	2005	Moderate TBI	0.1637
Samoa	2010	Moderate TBI	0.1637
Samoa	2017	Moderate TBI	0.1637
Samoa	1990	Amputation of fingers (excluding thumb)	0.0047
Samoa	1995	Amputation of fingers (excluding thumb)	0.0047
Samoa	2000	Amputation of fingers (excluding thumb)	0.0047
Samoa	2005	Amputation of fingers (excluding thumb)	0.0047
Samoa	2010	Amputation of fingers (excluding thumb)	0.0047
Samoa	2017	Amputation of fingers (excluding thumb)	0.0047
Samoa	1990	Spinal cord lesion at neck level	0.4699
Samoa	1995	Spinal cord lesion at neck level	0.4658
Samoa	2000	Spinal cord lesion at neck level	0.4603
Samoa	2005	Spinal cord lesion at neck level	0.4572
Samoa	2010	Spinal cord lesion at neck level	0.4555
Samoa	2017	Spinal cord lesion at neck level	0.4534
Samoa	1990	Spinal cord lesion below neck level	0.3002
Samoa	1995	Spinal cord lesion below neck level	0.2917
Samoa	2000	Spinal cord lesion below neck level	0.28
Samoa	2005	Spinal cord lesion below neck level	0.2735
Samoa	2010	Spinal cord lesion below neck level	0.2699
Samoa	2017	Spinal cord lesion below neck level	0.2655
Samoa	1990	Drowning and nonfatal submersion	0.2471
Samoa	1995	Drowning and nonfatal submersion	0.2471
Samoa	2000	Drowning and nonfatal submersion	0.2471
Samoa	2005	Drowning and nonfatal submersion	0.2471
Samoa	2010	Drowning and nonfatal submersion	0.2471
Samoa	2017	Drowning and nonfatal submersion	0.2471
Samoa	1990	Asphyxiation	0.2471
Samoa	1995	Asphyxiation	0.2471
Samoa	2000	Asphyxiation	0.2471
Samoa	2005	Asphyxiation	0.2471
Samoa	2010	Asphyxiation	0.2471
Samoa	2017	Asphyxiation	0.2471
Samoa	1990	Crush injury	0.1325
Samoa	1995	Crush injury	0.1325
Samoa	2000	Crush injury	0.1325
Samoa	2005	Crush injury	0.1325
Samoa	2010	Crush injury	0.1325
Samoa	2017	Crush injury	0.1325
Samoa	1990	Nerve injury	0.1129
Samoa	1995	Nerve injury	0.1129
Samoa	2000	Nerve injury	0.1129
Samoa	2005	Nerve injury	0.1129
Samoa	2010	Nerve injury	0.1129
Samoa	2017	Nerve injury	0.1129
Samoa	1990	Injury to eyes	0.0165
Samoa	1995	Injury to eyes	0.0165
Samoa	2000	Injury to eyes	0.0165
Samoa	2005	Injury to eyes	0.0165
Samoa	2010	Injury to eyes	0.0165
Samoa	2017	Injury to eyes	0.0165
Samoa	1990	Amputation of lower limb, unilateral	0.1044
Samoa	1995	Amputation of lower limb, unilateral	0.0998
Samoa	2000	Amputation of lower limb, unilateral	0.0936
Samoa	2005	Amputation of lower limb, unilateral	0.0901
Samoa	2010	Amputation of lower limb, unilateral	0.0882
Samoa	2017	Amputation of lower limb, unilateral	0.0859
Samoa	1990	Open wound(s)	0.011
Samoa	1995	Open wound(s)	0.011
Samoa	2000	Open wound(s)	0.011
Samoa	2005	Open wound(s)	0.011
Samoa	2010	Open wound(s)	0.011
Samoa	2017	Open wound(s)	0.011
Samoa	1990	Poisoning requiring urgent care	0.1628
Samoa	1995	Poisoning requiring urgent care	0.1628
Samoa	2000	Poisoning requiring urgent care	0.1628
Samoa	2005	Poisoning requiring urgent care	0.1628
Samoa	2010	Poisoning requiring urgent care	0.1628
Samoa	2017	Poisoning requiring urgent care	0.1628
Samoa	1990	Severe chest injury	0.0474
Samoa	1995	Severe chest injury	0.0474
Samoa	2000	Severe chest injury	0.0474
Samoa	2005	Severe chest injury	0.0474
Samoa	2010	Severe chest injury	0.0474
Samoa	2017	Severe chest injury	0.0474
Samoa	1990	Internal hemorrhage in abdomen and pelvis	0.1143
Samoa	1995	Internal hemorrhage in abdomen and pelvis	0.1143
Samoa	2000	Internal hemorrhage in abdomen and pelvis	0.1143
Samoa	2005	Internal hemorrhage in abdomen and pelvis	0.1143
Samoa	2010	Internal hemorrhage in abdomen and pelvis	0.1143
Samoa	2017	Internal hemorrhage in abdomen and pelvis	0.1143
Samoa	1990	Contusion in any part of the body	0.0075
Samoa	1995	Contusion in any part of the body	0.0075
Samoa	2000	Contusion in any part of the body	0.0075
Samoa	2005	Contusion in any part of the body	0.0075
Samoa	2010	Contusion in any part of the body	0.0075
Samoa	2017	Contusion in any part of the body	0.0075
Samoa	1990	Effect of different environmental factors	0.049
Samoa	1995	Effect of different environmental factors	0.049
Samoa	2000	Effect of different environmental factors	0.049
Samoa	2005	Effect of different environmental factors	0.049
Samoa	2010	Effect of different environmental factors	0.049
Samoa	2017	Effect of different environmental factors	0.049
Samoa	1990	Complications following therapeutic procedures	0.0506
Samoa	1995	Complications following therapeutic procedures	0.0506
Samoa	2000	Complications following therapeutic procedures	0.0506
Samoa	2005	Complications following therapeutic procedures	0.0506
Samoa	2010	Complications following therapeutic procedures	0.0506
Samoa	2017	Complications following therapeutic procedures	0.0506
Samoa	1990	Multiple fractures, dislocations, crashes, wounds, sprains, and strains	0.2255
Samoa	1995	Multiple fractures, dislocations, crashes, wounds, sprains, and strains	0.2139
Samoa	2000	Multiple fractures, dislocations, crashes, wounds, sprains, and strains	0.1979
Samoa	2005	Multiple fractures, dislocations, crashes, wounds, sprains, and strains	0.1889
Samoa	2010	Multiple fractures, dislocations, crashes, wounds, sprains, and strains	0.184
Samoa	2017	Multiple fractures, dislocations, crashes, wounds, sprains, and strains	0.178
Samoa	1990	Amputation of upper limb, unilateral	0.0775
Samoa	1995	Amputation of upper limb, unilateral	0.0748
Samoa	2000	Amputation of upper limb, unilateral	0.0711
Samoa	2005	Amputation of upper limb, unilateral	0.0691
Samoa	2010	Amputation of upper limb, unilateral	0.068
Samoa	2017	Amputation of upper limb, unilateral	0.0666
Samoa	1990	Amputation of thumb	0.0114
Samoa	1995	Amputation of thumb	0.0114
Samoa	2000	Amputation of thumb	0.0114
Samoa	2005	Amputation of thumb	0.0114
Samoa	2010	Amputation of thumb	0.0114
Samoa	2017	Amputation of thumb	0.0114
Samoa	1990	Amputation of toe/toes	0.0059
Samoa	1995	Amputation of toe/toes	0.0059
Samoa	2000	Amputation of toe/toes	0.0059
Samoa	2005	Amputation of toe/toes	0.0059
Samoa	2010	Amputation of toe/toes	0.0059
Samoa	2017	Amputation of toe/toes	0.0059
Samoa	1990	Burns, <20% total burned surface area without lower airway burns	0.0163
Samoa	1995	Burns, <20% total burned surface area without lower airway burns	0.0163
Samoa	2000	Burns, <20% total burned surface area without lower airway burns	0.0163
Samoa	2005	Burns, <20% total burned surface area without lower airway burns	0.0163
Samoa	2010	Burns, <20% total burned surface area without lower airway burns	0.0163
Samoa	2017	Burns, <20% total burned surface area without lower airway burns	0.0163
Samoa	1990	Burns, >=20% total burned surface area or >= 10% burned surface area if head/neck or hands/wrist involved w/o lower airway burns	0.2909
Samoa	1995	Burns, >=20% total burned surface area or >= 10% burned surface area if head/neck or hands/wrist involved w/o lower airway burns	0.2801
Samoa	2000	Burns, >=20% total burned surface area or >= 10% burned surface area if head/neck or hands/wrist involved w/o lower airway burns	0.2651
Samoa	2005	Burns, >=20% total burned surface area or >= 10% burned surface area if head/neck or hands/wrist involved w/o lower airway burns	0.2568
Samoa	2010	Burns, >=20% total burned surface area or >= 10% burned surface area if head/neck or hands/wrist involved w/o lower airway burns	0.2522
Samoa	2017	Burns, >=20% total burned surface area or >= 10% burned surface area if head/neck or hands/wrist involved w/o lower airway burns	0.2466
Solomon Islands	1990	Amputation of lower limbs, bilateral	0.2864
Solomon Islands	1995	Amputation of lower limbs, bilateral	0.2795
Solomon Islands	2000	Amputation of lower limbs, bilateral	0.2707
Solomon Islands	2005	Amputation of lower limbs, bilateral	0.278
Solomon Islands	2010	Amputation of lower limbs, bilateral	0.2789
Solomon Islands	2017	Amputation of lower limbs, bilateral	0.2651
Solomon Islands	1990	Lower airway burns	0.2252
Solomon Islands	1995	Lower airway burns	0.2252
Solomon Islands	2000	Lower airway burns	0.2252
Solomon Islands	2005	Lower airway burns	0.2252
Solomon Islands	2010	Lower airway burns	0.2252
Solomon Islands	2017	Lower airway burns	0.2252
Solomon Islands	1990	Dislocation of hip	0.0159
Solomon Islands	1995	Dislocation of hip	0.0159
Solomon Islands	2000	Dislocation of hip	0.0159
Solomon Islands	2005	Dislocation of hip	0.0159
Solomon Islands	2010	Dislocation of hip	0.0159
Solomon Islands	2017	Dislocation of hip	0.0159
Solomon Islands	1990	Dislocation of knee	0.1134
Solomon Islands	1995	Dislocation of knee	0.1134
Solomon Islands	2000	Dislocation of knee	0.1134
Solomon Islands	2005	Dislocation of knee	0.1134
Solomon Islands	2010	Dislocation of knee	0.1134
Solomon Islands	2017	Dislocation of knee	0.1134
Solomon Islands	1990	Dislocation of shoulder	0.062
Solomon Islands	1995	Dislocation of shoulder	0.062
Solomon Islands	2000	Dislocation of shoulder	0.062
Solomon Islands	2005	Dislocation of shoulder	0.062
Solomon Islands	2010	Dislocation of shoulder	0.062
Solomon Islands	2017	Dislocation of shoulder	0.062
Solomon Islands	1990	Muscle and tendon injuries, including sprains and strains lesser dislocations	0.0075
Solomon Islands	1995	Muscle and tendon injuries, including sprains and strains lesser dislocations	0.0075
Solomon Islands	2000	Muscle and tendon injuries, including sprains and strains lesser dislocations	0.0075
Solomon Islands	2005	Muscle and tendon injuries, including sprains and strains lesser dislocations	0.0075
Solomon Islands	2010	Muscle and tendon injuries, including sprains and strains lesser dislocations	0.0075
Solomon Islands	2017	Muscle and tendon injuries, including sprains and strains lesser dislocations	0.0075
Solomon Islands	1990	Fracture of clavicle, scapula, or humerus	0.0349
Solomon Islands	1995	Fracture of clavicle, scapula, or humerus	0.0349
Solomon Islands	2000	Fracture of clavicle, scapula, or humerus	0.0349
Solomon Islands	2005	Fracture of clavicle, scapula, or humerus	0.0349
Solomon Islands	2010	Fracture of clavicle, scapula, or humerus	0.0349
Solomon Islands	2017	Fracture of clavicle, scapula, or humerus	0.0349
Solomon Islands	1990	Fracture of face bones	0.0669
Solomon Islands	1995	Fracture of face bones	0.0669
Solomon Islands	2000	Fracture of face bones	0.0669
Solomon Islands	2005	Fracture of face bones	0.0669
Solomon Islands	2010	Fracture of face bones	0.0669
Solomon Islands	2017	Fracture of face bones	0.0669
Solomon Islands	1990	Fracture of foot bones except ankle	0.0264
Solomon Islands	1995	Fracture of foot bones except ankle	0.0264
Solomon Islands	2000	Fracture of foot bones except ankle	0.0264
Solomon Islands	2005	Fracture of foot bones except ankle	0.0264
Solomon Islands	2010	Fracture of foot bones except ankle	0.0264
Solomon Islands	2017	Fracture of foot bones except ankle	0.0264
Solomon Islands	1990	Fracture of hand (wrist and other distal part of hand)	0.0138
Solomon Islands	1995	Fracture of hand (wrist and other distal part of hand)	0.0138
Solomon Islands	2000	Fracture of hand (wrist and other distal part of hand)	0.0138
Solomon Islands	2005	Fracture of hand (wrist and other distal part of hand)	0.0138
Solomon Islands	2010	Fracture of hand (wrist and other distal part of hand)	0.0138
Solomon Islands	2017	Fracture of hand (wrist and other distal part of hand)	0.0138
Solomon Islands	1990	Fracture of hip	0.2734
Solomon Islands	1995	Fracture of hip	0.2643
Solomon Islands	2000	Fracture of hip	0.2527
Solomon Islands	2005	Fracture of hip	0.2624
Solomon Islands	2010	Fracture of hip	0.2635
Solomon Islands	2017	Fracture of hip	0.2453
Solomon Islands	1990	Amputation of upper limbs, bilateral	0.3104
Solomon Islands	1995	Amputation of upper limbs, bilateral	0.301
Solomon Islands	2000	Amputation of upper limbs, bilateral	0.289
Solomon Islands	2005	Amputation of upper limbs, bilateral	0.299
Solomon Islands	2010	Amputation of upper limbs, bilateral	0.3002
Solomon Islands	2017	Amputation of upper limbs, bilateral	0.2813
Solomon Islands	1990	Fracture of patella, tibia or fibula, or ankle	0.0554
Solomon Islands	1995	Fracture of patella, tibia or fibula, or ankle	0.0554
Solomon Islands	2000	Fracture of patella, tibia or fibula, or ankle	0.0554
Solomon Islands	2005	Fracture of patella, tibia or fibula, or ankle	0.0554
Solomon Islands	2010	Fracture of patella, tibia or fibula, or ankle	0.0554
Solomon Islands	2017	Fracture of patella, tibia or fibula, or ankle	0.0554
Solomon Islands	1990	Fracture of pelvis	0.1823
Solomon Islands	1995	Fracture of pelvis	0.1823
Solomon Islands	2000	Fracture of pelvis	0.1823
Solomon Islands	2005	Fracture of pelvis	0.1823
Solomon Islands	2010	Fracture of pelvis	0.1823
Solomon Islands	2017	Fracture of pelvis	0.1823
Solomon Islands	1990	Fracture of radius and/or ulna	0.0435
Solomon Islands	1995	Fracture of radius and/or ulna	0.0435
Solomon Islands	2000	Fracture of radius and/or ulna	0.0435
Solomon Islands	2005	Fracture of radius and/or ulna	0.0435
Solomon Islands	2010	Fracture of radius and/or ulna	0.0435
Solomon Islands	2017	Fracture of radius and/or ulna	0.0435
Solomon Islands	1990	Fracture of skull	0.0714
Solomon Islands	1995	Fracture of skull	0.0714
Solomon Islands	2000	Fracture of skull	0.0714
Solomon Islands	2005	Fracture of skull	0.0714
Solomon Islands	2010	Fracture of skull	0.0714
Solomon Islands	2017	Fracture of skull	0.0714

Solomon Islands	1990	Fracture of sternum and/or fracture of one or more ribs	0.1027
Solomon Islands	1995	Fracture of sternum and/or fracture of one or more ribs	0.1027
Solomon Islands	2000	Fracture of sternum and/or fracture of one or more ribs	0.1027
Solomon Islands	2005	Fracture of sternum and/or fracture of one or more ribs	0.1027
Solomon Islands	2010	Fracture of sternum and/or fracture of one or more ribs	0.1027
Solomon Islands	2017	Fracture of sternum and/or fracture of one or more ribs	0.1027
Solomon Islands	1990	Fracture of vertebral column	0.1106
Solomon Islands	1995	Fracture of vertebral column	0.1106
Solomon Islands	2000	Fracture of vertebral column	0.1106
Solomon Islands	2005	Fracture of vertebral column	0.1106
Solomon Islands	2010	Fracture of vertebral column	0.1106
Solomon Islands	2017	Fracture of vertebral column	0.1106
Solomon Islands	1990	Fracture of femur, other than femoral neck	0.042
Solomon Islands	1995	Fracture of femur, other than femoral neck	0.042
Solomon Islands	2000	Fracture of femur, other than femoral neck	0.042
Solomon Islands	2005	Fracture of femur, other than femoral neck	0.042
Solomon Islands	2010	Fracture of femur, other than femoral neck	0.042
Solomon Islands	2017	Fracture of femur, other than femoral neck	0.042
Solomon Islands	1990	Minor TBI	0.1319
Solomon Islands	1995	Minor TBI	0.1319
Solomon Islands	2000	Minor TBI	0.1319
Solomon Islands	2005	Minor TBI	0.1319
Solomon Islands	2010	Minor TBI	0.1319
Solomon Islands	2017	Minor TBI	0.1319
Solomon Islands	1990	Moderate TBI	0.1637
Solomon Islands	1995	Moderate TBI	0.1637
Solomon Islands	2000	Moderate TBI	0.1637
Solomon Islands	2005	Moderate TBI	0.1637
Solomon Islands	2010	Moderate TBI	0.1637
Solomon Islands	2017	Moderate TBI	0.1637
Solomon Islands	1990	Amputation of fingers (excluding thumb)	0.0047
Solomon Islands	1995	Amputation of fingers (excluding thumb)	0.0047
Solomon Islands	2000	Amputation of fingers (excluding thumb)	0.0047
Solomon Islands	2005	Amputation of fingers (excluding thumb)	0.0047
Solomon Islands	2010	Amputation of fingers (excluding thumb)	0.0047
Solomon Islands	2017	Amputation of fingers (excluding thumb)	0.0047
Solomon Islands	1990	Spinal cord lesion at neck level	0.4864
Solomon Islands	1995	Spinal cord lesion at neck level	0.4833
Solomon Islands	2000	Spinal cord lesion at neck level	0.4793
Solomon Islands	2005	Spinal cord lesion at neck level	0.4826
Solomon Islands	2010	Spinal cord lesion at neck level	0.483
Solomon Islands	2017	Spinal cord lesion at neck level	0.4767
Solomon Islands	1990	Spinal cord lesion below neck level	0.3351
Solomon Islands	1995	Spinal cord lesion below neck level	0.3285
Solomon Islands	2000	Spinal cord lesion below neck level	0.32
Solomon Islands	2005	Spinal cord lesion below neck level	0.327
Solomon Islands	2010	Spinal cord lesion below neck level	0.3279
Solomon Islands	2017	Spinal cord lesion below neck level	0.3146
Solomon Islands	1990	Drowning and nonfatal submersion	0.2471
Solomon Islands	1995	Drowning and nonfatal submersion	0.2471
Solomon Islands	2000	Drowning and nonfatal submersion	0.2471
Solomon Islands	2005	Drowning and nonfatal submersion	0.2471
Solomon Islands	2010	Drowning and nonfatal submersion	0.2471
Solomon Islands	2017	Drowning and nonfatal submersion	0.2471
Solomon Islands	1990	Asphyxiation	0.2471
Solomon Islands	1995	Asphyxiation	0.2471
Solomon Islands	2000	Asphyxiation	0.2471
Solomon Islands	2005	Asphyxiation	0.2471
Solomon Islands	2010	Asphyxiation	0.2471
Solomon Islands	2017	Asphyxiation	0.2471
Solomon Islands	1990	Crush injury	0.1325
Solomon Islands	1995	Crush injury	0.1325
Solomon Islands	2000	Crush injury	0.1325
Solomon Islands	2005	Crush injury	0.1325
Solomon Islands	2010	Crush injury	0.1325
Solomon Islands	2017	Crush injury	0.1325
Solomon Islands	1990	Nerve injury	0.1129
Solomon Islands	1995	Nerve injury	0.1129
Solomon Islands	2000	Nerve injury	0.1129
Solomon Islands	2005	Nerve injury	0.1129
Solomon Islands	2010	Nerve injury	0.1129
Solomon Islands	2017	Nerve injury	0.1129
Solomon Islands	1990	Injury to eyes	0.0165
Solomon Islands	1995	Injury to eyes	0.0165
Solomon Islands	2000	Injury to eyes	0.0165
Solomon Islands	2005	Injury to eyes	0.0165
Solomon Islands	2010	Injury to eyes	0.0165
Solomon Islands	2017	Injury to eyes	0.0165
Solomon Islands	1990	Amputation of lower limb, unilateral	0.1231
Solomon Islands	1995	Amputation of lower limb, unilateral	0.1195
Solomon Islands	2000	Amputation of lower limb, unilateral	0.115
Solomon Islands	2005	Amputation of lower limb, unilateral	0.1187
Solomon Islands	2010	Amputation of lower limb, unilateral	0.1192
Solomon Islands	2017	Amputation of lower limb, unilateral	0.1121
Solomon Islands	1990	Open wound(s)	0.011
Solomon Islands	1995	Open wound(s)	0.011
Solomon Islands	2000	Open wound(s)	0.011
Solomon Islands	2005	Open wound(s)	0.011
Solomon Islands	2010	Open wound(s)	0.011
Solomon Islands	2017	Open wound(s)	0.011
Solomon Islands	1990	Poisoning requiring urgent care	0.1628
Solomon Islands	1995	Poisoning requiring urgent care	0.1628
Solomon Islands	2000	Poisoning requiring urgent care	0.1628
Solomon Islands	2005	Poisoning requiring urgent care	0.1628
Solomon Islands	2010	Poisoning requiring urgent care	0.1628
Solomon Islands	2017	Poisoning requiring urgent care	0.1628
Solomon Islands	1990	Severe chest injury	0.0474
Solomon Islands	1995	Severe chest injury	0.0474
Solomon Islands	2000	Severe chest injury	0.0474
Solomon Islands	2005	Severe chest injury	0.0474
Solomon Islands	2010	Severe chest injury	0.0474
Solomon Islands	2017	Severe chest injury	0.0474
Solomon Islands	1990	Internal hemorrhage in abdomen and pelvis	0.1143
Solomon Islands	1995	Internal hemorrhage in abdomen and pelvis	0.1143
Solomon Islands	2000	Internal hemorrhage in abdomen and pelvis	0.1143
Solomon Islands	2005	Internal hemorrhage in abdomen and pelvis	0.1143
Solomon Islands	2010	Internal hemorrhage in abdomen and pelvis	0.1143
Solomon Islands	2017	Internal hemorrhage in abdomen and pelvis	0.1143
Solomon Islands	1990	Contusion in any part of the body	0.0075
Solomon Islands	1995	Contusion in any part of the body	0.0075
Solomon Islands	2000	Contusion in any part of the body	0.0075
Solomon Islands	2005	Contusion in any part of the body	0.0075
Solomon Islands	2010	Contusion in any part of the body	0.0075
Solomon Islands	2017	Contusion in any part of the body	0.0075
Solomon Islands	1990	Effect of different environmental factors	0.049
Solomon Islands	1995	Effect of different environmental factors	0.049
Solomon Islands	2000	Effect of different environmental factors	0.049
Solomon Islands	2005	Effect of different environmental factors	0.049
Solomon Islands	2010	Effect of different environmental factors	0.049
Solomon Islands	2017	Effect of different environmental factors	0.049
Solomon Islands	1990	Complications following therapeutic procedures	0.0506
Solomon Islands	1995	Complications following therapeutic procedures	0.0506
Solomon Islands	2000	Complications following therapeutic procedures	0.0506
Solomon Islands	2005	Complications following therapeutic procedures	0.0506
Solomon Islands	2010	Complications following therapeutic procedures	0.0506
Solomon Islands	2017	Complications following therapeutic procedures	0.0506
Solomon Islands	1990	Multiple fractures, dislocations, crashes, wounds, sprains, and strains	0.2734
Solomon Islands	1995	Multiple fractures, dislocations, crashes, wounds, sprains, and strains	0.2643
Solomon Islands	2000	Multiple fractures, dislocations, crashes, wounds, sprains, and strains	0.2527
Solomon Islands	2005	Multiple fractures, dislocations, crashes, wounds, sprains, and strains	0.2624
Solomon Islands	2010	Multiple fractures, dislocations, crashes, wounds, sprains, and strains	0.2635
Solomon Islands	2017	Multiple fractures, dislocations, crashes, wounds, sprains, and strains	0.2453
Solomon Islands	1990	Amputation of upper limb, unilateral	0.0885
Solomon Islands	1995	Amputation of upper limb, unilateral	0.0864
Solomon Islands	2000	Amputation of upper limb, unilateral	0.0837
Solomon Islands	2005	Amputation of upper limb, unilateral	0.0859
Solomon Islands	2010	Amputation of upper limb, unilateral	0.0862
Solomon Islands	2017	Amputation of upper limb, unilateral	0.082
Solomon Islands	1990	Amputation of thumb	0.0114
Solomon Islands	1995	Amputation of thumb	0.0114
Solomon Islands	2000	Amputation of thumb	0.0114
Solomon Islands	2005	Amputation of thumb	0.0114
Solomon Islands	2010	Amputation of thumb	0.0114
Solomon Islands	2017	Amputation of thumb	0.0114
Solomon Islands	1990	Amputation of toe/toes	0.0059
Solomon Islands	1995	Amputation of toe/toes	0.0059
Solomon Islands	2000	Amputation of toe/toes	0.0059
Solomon Islands	2005	Amputation of toe/toes	0.0059
Solomon Islands	2010	Amputation of toe/toes	0.0059
Solomon Islands	2017	Amputation of toe/toes	0.0059
Solomon Islands	1990	Burns, <20% total burned surface area without lower airway burns	0.0163
Solomon Islands	1995	Burns, <20% total burned surface area without lower airway burns	0.0163
Solomon Islands	2000	Burns, <20% total burned surface area without lower airway burns	0.0163
Solomon Islands	2005	Burns, <20% total burned surface area without lower airway burns	0.0163
Solomon Islands	2010	Burns, <20% total burned surface area without lower airway burns	0.0163
Solomon Islands	2017	Burns, <20% total burned surface area without lower airway burns	0.0163
Solomon Islands	1990	Burns, >=20% total burned surface area or >= 10% burned surface area if head/neck or hands/wrist involved w/o lower airway burns	0.3355
Solomon Islands	1995	Burns, >=20% total burned surface area or >= 10% burned surface area if head/neck or hands/wrist involved w/o lower airway burns	0.327
Solomon Islands	2000	Burns, >=20% total burned surface area or >= 10% burned surface area if head/neck or hands/wrist involved w/o lower airway burns	0.3162
Solomon Islands	2005	Burns, >=20% total burned surface area or >= 10% burned surface area if head/neck or hands/wrist involved w/o lower airway burns	0.3252
Solomon Islands	2010	Burns, >=20% total burned surface area or >= 10% burned surface area if head/neck or hands/wrist involved w/o lower airway burns	0.3263
Solomon Islands	2017	Burns, >=20% total burned surface area or >= 10% burned surface area if head/neck or hands/wrist involved w/o lower airway burns	0.3093
Tonga	1990	Amputation of lower limbs, bilateral	0.247
Tonga	1995	Amputation of lower limbs, bilateral	0.243
Tonga	2000	Amputation of lower limbs, bilateral	0.2322
Tonga	2005	Amputation of lower limbs, bilateral	0.2248
Tonga	2010	Amputation of lower limbs, bilateral	0.2186
Tonga	2017	Amputation of lower limbs, bilateral	0.2072
Tonga	1990	Lower airway burns	0.2252
Tonga	1995	Lower airway burns	0.2252
Tonga	2000	Lower airway burns	0.2252
Tonga	2005	Lower airway burns	0.2252
Tonga	2010	Lower airway burns	0.2252
Tonga	2017	Lower airway burns	0.2252
Tonga	1990	Dislocation of hip	0.0159
Tonga	1995	Dislocation of hip	0.0159
Tonga	2000	Dislocation of hip	0.0159
Tonga	2005	Dislocation of hip	0.0159
Tonga	2010	Dislocation of hip	0.0159
Tonga	2017	Dislocation of hip	0.0159
Tonga	1990	Dislocation of knee	0.1134
Tonga	1995	Dislocation of knee	0.1134
Tonga	2000	Dislocation of knee	0.1134
Tonga	2005	Dislocation of knee	0.1134
Tonga	2010	Dislocation of knee	0.1134
Tonga	2017	Dislocation of knee	0.1134
Tonga	1990	Dislocation of shoulder	0.062
Tonga	1995	Dislocation of shoulder	0.062
Tonga	2000	Dislocation of shoulder	0.062
Tonga	2005	Dislocation of shoulder	0.062
Tonga	2010	Dislocation of shoulder	0.062
Tonga	2017	Dislocation of shoulder	0.062
Tonga	1990	Muscle and tendon injuries, including sprains and strains lesser dislocations	0.0075
Tonga	1995	Muscle and tendon injuries, including sprains and strains lesser dislocations	0.0075
Tonga	2000	Muscle and tendon injuries, including sprains and strains lesser dislocations	0.0075
Tonga	2005	Muscle and tendon injuries, including sprains and strains lesser dislocations	0.0075
Tonga	2010	Muscle and tendon injuries, including sprains and strains lesser dislocations	0.0075
Tonga	2017	Muscle and tendon injuries, including sprains and strains lesser dislocations	0.0075
Tonga	1990	Fracture of clavicle, scapula, or humerus	0.0349
Tonga	1995	Fracture of clavicle, scapula, or humerus	0.0349
Tonga	2000	Fracture of clavicle, scapula, or humerus	0.0349
Tonga	2005	Fracture of clavicle, scapula, or humerus	0.0349
Tonga	2010	Fracture of clavicle, scapula, or humerus	0.0349
Tonga	2017	Fracture of clavicle, scapula, or humerus	0.0349
Tonga	1990	Fracture of face bones	0.0669
Tonga	1995	Fracture of face bones	0.0669
Tonga	2000	Fracture of face bones	0.0669
Tonga	2005	Fracture of face bones	0.0669
Tonga	2010	Fracture of face bones	0.0669
Tonga	2017	Fracture of face bones	0.0669
Tonga	1990	Fracture of foot bones except ankle	0.0264
Tonga	1995	Fracture of foot bones except ankle	0.0264
Tonga	2000	Fracture of foot bones except ankle	0.0264
Tonga	2005	Fracture of foot bones except ankle	0.0264
Tonga	2010	Fracture of foot bones except ankle	0.0264
Tonga	2017	Fracture of foot bones except ankle	0.0264
Tonga	1990	Fracture of hand (wrist and other distal part of hand)	0.0138
Tonga	1995	Fracture of hand (wrist and other distal part of hand)	0.0138
Tonga	2000	Fracture of hand (wrist and other distal part of hand)	0.0138
Tonga	2005	Fracture of hand (wrist and other distal part of hand)	0.0138
Tonga	2010	Fracture of hand (wrist and other distal part of hand)	0.0138
Tonga	2017	Fracture of hand (wrist and other distal part of hand)	0.0138
Tonga	1990	Fracture of hip	0.2214
Tonga	1995	Fracture of hip	0.2161
Tonga	2000	Fracture of hip	0.2018
Tonga	2005	Fracture of hip	0.1921
Tonga	2010	Fracture of hip	0.1838
Tonga	2017	Fracture of hip	0.1688
Tonga	1990	Amputation of upper limbs, bilateral	0.2567
Tonga	1995	Amputation of upper limbs, bilateral	0.2512
Tonga	2000	Amputation of upper limbs, bilateral	0.2364
Tonga	2005	Amputation of upper limbs, bilateral	0.2264
Tonga	2010	Amputation of upper limbs, bilateral	0.2179
Tonga	2017	Amputation of upper limbs, bilateral	0.2024
Tonga	1990	Fracture of patella, tibia or fibula, or ankle	0.0554
Tonga	1995	Fracture of patella, tibia or fibula, or ankle	0.0554
Tonga	2000	Fracture of patella, tibia or fibula, or ankle	0.0554
Tonga	2005	Fracture of patella, tibia or fibula, or ankle	0.0554
Tonga	2010	Fracture of patella, tibia or fibula, or ankle	0.0554
Tonga	2017	Fracture of patella, tibia or fibula, or ankle	0.0554
Tonga	1990	Fracture of pelvis	0.1823
Tonga	1995	Fracture of pelvis	0.1823
Tonga	2000	Fracture of pelvis	0.1823
Tonga	2005	Fracture of pelvis	0.1823
Tonga	2010	Fracture of pelvis	0.1823
Tonga	2017	Fracture of pelvis	0.1823
Tonga	1990	Fracture of radius and/or ulna	0.0435
Tonga	1995	Fracture of radius and/or ulna	0.0435
Tonga	2000	Fracture of radius and/or ulna	0.0435
Tonga	2005	Fracture of radius and/or ulna	0.0435
Tonga	2010	Fracture of radius and/or ulna	0.0435
Tonga	2017	Fracture of radius and/or ulna	0.0435
Tonga	1990	Fracture of skull	0.0714
Tonga	1995	Fracture of skull	0.0714
Tonga	2000	Fracture of skull	0.0714
Tonga	2005	Fracture of skull	0.0714
Tonga	2010	Fracture of skull	0.0714
Tonga	2017	Fracture of skull	0.0714
Tonga	1990	Fracture of sternum and/or fracture of one or more ribs	0.1027
Tonga	1995	Fracture of sternum and/or fracture of one or more ribs	0.1027
Tonga	2000	Fracture of sternum and/or fracture of one or more ribs	0.1027
Tonga	2005	Fracture of sternum and/or fracture of one or more ribs	0.1027
Tonga	2010	Fracture of sternum and/or fracture of one or more ribs	0.1027
Tonga	2017	Fracture of sternum and/or fracture of one or more ribs	0.1027
Tonga	1990	Fracture of vertebral column	0.1106
Tonga	1995	Fracture of vertebral column	0.1106
Tonga	2000	Fracture of vertebral column	0.1106
Tonga	2005	Fracture of vertebral column	0.1106
Tonga	2010	Fracture of vertebral column	0.1106
Tonga	2017	Fracture of vertebral column	0.1106
Tonga	1990	Fracture of femur, other than femoral neck	0.042
Tonga	1995	Fracture of femur, other than femoral neck	0.042
Tonga	2000	Fracture of femur, other than femoral neck	0.042
Tonga	2005	Fracture of femur, other than femoral neck	0.042
Tonga	2010	Fracture of femur, other than femoral neck	0.042
Tonga	2017	Fracture of femur, other than femoral neck	0.042
Tonga	1990	Minor TBI	0.1319
Tonga	1995	Minor TBI	0.1319
Tonga	2000	Minor TBI	0.1319
Tonga	2005	Minor TBI	0.1319
Tonga	2010	Minor TBI	0.1319
Tonga	2017	Minor TBI	0.1319
Tonga	1990	Moderate TBI	0.1637
Tonga	1995	Moderate TBI	0.1637
Tonga	2000	Moderate TBI	0.1637
Tonga	2005	Moderate TBI	0.1637
Tonga	2010	Moderate TBI	0.1637
Tonga	2017	Moderate TBI	0.1637
Tonga	1990	Amputation of fingers (excluding thumb)	0.0047
Tonga	1995	Amputation of fingers (excluding thumb)	0.0047
Tonga	2000	Amputation of fingers (excluding thumb)	0.0047
Tonga	2005	Amputation of fingers (excluding thumb)	0.0047
Tonga	2010	Amputation of fingers (excluding thumb)	0.0047
Tonga	2017	Amputation of fingers (excluding thumb)	0.0047
Tonga	1990	Spinal cord lesion at neck level	0.4684
Tonga	1995	Spinal cord lesion at neck level	0.4666
Tonga	2000	Spinal cord lesion at neck level	0.4616
Tonga	2005	Spinal cord lesion at neck level	0.4583
Tonga	2010	Spinal cord lesion at neck level	0.4554
Tonga	2017	Spinal cord lesion at neck level	0.4502
Tonga	1990	Spinal cord lesion below neck level	0.2972
Tonga	1995	Spinal cord lesion below neck level	0.2933
Tonga	2000	Spinal cord lesion below neck level	0.2828
Tonga	2005	Spinal cord lesion below neck level	0.2757
Tonga	2010	Spinal cord lesion below neck level	0.2697
Tonga	2017	Spinal cord lesion below neck level	0.2587
Tonga	1990	Drowning and nonfatal submersion	0.2471
Tonga	1995	Drowning and nonfatal submersion	0.2471
Tonga	2000	Drowning and nonfatal submersion	0.2471
Tonga	2005	Drowning and nonfatal submersion	0.2471
Tonga	2010	Drowning and nonfatal submersion	0.2471
Tonga	2017	Drowning and nonfatal submersion	0.2471
Tonga	1990	Asphyxiation	0.2471
Tonga	1995	Asphyxiation	0.2471
Tonga	2000	Asphyxiation	0.2471
Tonga	2005	Asphyxiation	0.2471
Tonga	2010	Asphyxiation	0.2471
Tonga	2017	Asphyxiation	0.2471
Tonga	1990	Crush injury	0.1325
Tonga	1995	Crush injury	0.1325
Tonga	2000	Crush injury	0.1325
Tonga	2005	Crush injury	0.1325
Tonga	2010	Crush injury	0.1325
Tonga	2017	Crush injury	0.1325
Tonga	1990	Nerve injury	0.1129
Tonga	1995	Nerve injury	0.1129
Tonga	2000	Nerve injury	0.1129
Tonga	2005	Nerve injury	0.1129
Tonga	2010	Nerve injury	0.1129
Tonga	2017	Nerve injury	0.1129
Tonga	1990	Injury to eyes	0.0165
Tonga	1995	Injury to eyes	0.0165
Tonga	2000	Injury to eyes	0.0165
Tonga	2005	Injury to eyes	0.0165
Tonga	2010	Injury to eyes	0.0165
Tonga	2017	Injury to eyes	0.0165
Tonga	1990	Amputation of lower limb, unilateral	0.1028
Tonga	1995	Amputation of lower limb, unilateral	0.1007
Tonga	2000	Amputation of lower limb, unilateral	0.0951
Tonga	2005	Amputation of lower limb, unilateral	0.0913
Tonga	2010	Amputation of lower limb, unilateral	0.0881
Tonga	2017	Amputation of lower limb, unilateral	0.0823
Tonga	1990	Open wound(s)	0.011
Tonga	1995	Open wound(s)	0.011
Tonga	2000	Open wound(s)	0.011
Tonga	2005	Open wound(s)	0.011
Tonga	2010	Open wound(s)	0.011
Tonga	2017	Open wound(s)	0.011
Tonga	1990	Poisoning requiring urgent care	0.1628
Tonga	1995	Poisoning requiring urgent care	0.1628
Tonga	2000	Poisoning requiring urgent care	0.1628
Tonga	2005	Poisoning requiring urgent care	0.1628
Tonga	2010	Poisoning requiring urgent care	0.1628
Tonga	2017	Poisoning requiring urgent care	0.1628
Tonga	1990	Severe chest injury	0.0474
Tonga	1995	Severe chest injury	0.0474
Tonga	2000	Severe chest injury	0.0474
Tonga	2005	Severe chest injury	0.0474
Tonga	2010	Severe chest injury	0.0474
Tonga	2017	Severe chest injury	0.0474
Tonga	1990	Internal hemorrhage in abdomen and pelvis	0.1143
Tonga	1995	Internal hemorrhage in abdomen and pelvis	0.1143
Tonga	2000	Internal hemorrhage in abdomen and pelvis	0.1143
Tonga	2005	Internal hemorrhage in abdomen and pelvis	0.1143
Tonga	2010	Internal hemorrhage in abdomen and pelvis	0.1143
Tonga	2017	Internal hemorrhage in abdomen and pelvis	0.1143
Tonga	1990	Contusion in any part of the body	0.0075
Tonga	1995	Contusion in any part of the body	0.0075
Tonga	2000	Contusion in any part of the body	0.0075
Tonga	2005	Contusion in any part of the body	0.0075
Tonga	2010	Contusion in any part of the body	0.0075
Tonga	2017	Contusion in any part of the body	0.0075
Tonga	1990	Effect of different environmental factors	0.049
Tonga	1995	Effect of different environmental factors	0.049
Tonga	2000	Effect of different environmental factors	0.049
Tonga	2005	Effect of different environmental factors	0.049
Tonga	2010	Effect of different environmental factors	0.049
Tonga	2017	Effect of different environmental factors	0.049
Tonga	1990	Complications following therapeutic procedures	0.0506
Tonga	1995	Complications following therapeutic procedures	0.0506
Tonga	2000	Complications following therapeutic procedures	0.0506
Tonga	2005	Complications following therapeutic procedures	0.0506
Tonga	2010	Complications following therapeutic procedures	0.0506
Tonga	2017	Complications following therapeutic procedures	0.0506
Tonga	1990	Multiple fractures, dislocations, crashes, wounds, sprains, and strains	0.2214
Tonga	1995	Multiple fractures, dislocations, crashes, wounds, sprains, and strains	0.2161
Tonga	2000	Multiple fractures, dislocations, crashes, wounds, sprains, and strains	0.2018
Tonga	2005	Multiple fractures, dislocations, crashes, wounds, sprains, and strains	0.1921
Tonga	2010	Multiple fractures, dislocations, crashes, wounds, sprains, and strains	0.1838
Tonga	2017	Multiple fractures, dislocations, crashes, wounds, sprains, and strains	0.1688
Tonga	1990	Amputation of upper limb, unilateral	0.0765
Tonga	1995	Amputation of upper limb, unilateral	0.0753
Tonga	2000	Amputation of upper limb, unilateral	0.072
Tonga	2005	Amputation of upper limb, unilateral	0.0698
Tonga	2010	Amputation of upper limb, unilateral	0.0679
Tonga	2017	Amputation of upper limb, unilateral	0.0645
Tonga	1990	Amputation of thumb	0.0114
Tonga	1995	Amputation of thumb	0.0114
Tonga	2000	Amputation of thumb	0.0114
Tonga	2005	Amputation of thumb	0.0114
Tonga	2010	Amputation of thumb	0.0114
Tonga	2017	Amputation of thumb	0.0114
Tonga	1990	Amputation of toe/toes	0.0059
Tonga	1995	Amputation of toe/toes	0.0059
Tonga	2000	Amputation of toe/toes	0.0059
Tonga	2005	Amputation of toe/toes	0.0059
Tonga	2010	Amputation of toe/toes	0.0059
Tonga	2017	Amputation of toe/toes	0.0059
Tonga	1990	Burns, <20% total burned surface area without lower airway burns	0.0163
Tonga	1995	Burns, <20% total burned surface area without lower airway burns	0.0163
Tonga	2000	Burns, <20% total burned surface area without lower airway burns	0.0163
Tonga	2005	Burns, <20% total burned surface area without lower airway burns	0.0163
Tonga	2010	Burns, <20% total burned surface area without lower airway burns	0.0163
Tonga	2017	Burns, <20% total burned surface area without lower airway burns	0.0163
Tonga	1990	Burns, >=20% total burned surface area or >= 10% burned surface area if head/neck or hands/wrist involved w/o lower airway burns	0.287
Tonga	1995	Burns, >=20% total burned surface area or >= 10% burned surface area if head/neck or hands/wrist involved w/o lower airway burns	0.2821
Tonga	2000	Burns, >=20% total burned surface area or >= 10% burned surface area if head/neck or hands/wrist involved w/o lower airway burns	0.2687
Tonga	2005	Burns, >=20% total burned surface area or >= 10% burned surface area if head/neck or hands/wrist involved w/o lower airway burns	0.2597
Tonga	2010	Burns, >=20% total burned surface area or >= 10% burned surface area if head/neck or hands/wrist involved w/o lower airway burns	0.2521
Tonga	2017	Burns, >=20% total burned surface area or >= 10% burned surface area if head/neck or hands/wrist involved w/o lower airway burns	0.238
Vanuatu	1990	Amputation of lower limbs, bilateral	0.2812
Vanuatu	1995	Amputation of lower limbs, bilateral	0.2819
Vanuatu	2000	Amputation of lower limbs, bilateral	0.2795
Vanuatu	2005	Amputation of lower limbs, bilateral	0.28
Vanuatu	2010	Amputation of lower limbs, bilateral	0.2736
Vanuatu	2017	Amputation of lower limbs, bilateral	0.2656
Vanuatu	1990	Lower airway burns	0.2252
Vanuatu	1995	Lower airway burns	0.2252
Vanuatu	2000	Lower airway burns	0.2252
Vanuatu	2005	Lower airway burns	0.2252
Vanuatu	2010	Lower airway burns	0.2252

Vanuatu	2017	Lower airway burns	0.2252
Vanuatu	1990	Dislocation of hip	0.0159
Vanuatu	1995	Dislocation of hip	0.0159
Vanuatu	2000	Dislocation of hip	0.0159
Vanuatu	2005	Dislocation of hip	0.0159
Vanuatu	2010	Dislocation of hip	0.0159
Vanuatu	2017	Dislocation of hip	0.0159
Vanuatu	1990	Dislocation of knee	0.1134
Vanuatu	1995	Dislocation of knee	0.1134
Vanuatu	2000	Dislocation of knee	0.1134
Vanuatu	2005	Dislocation of knee	0.1134
Vanuatu	2010	Dislocation of knee	0.1134
Vanuatu	2017	Dislocation of knee	0.1134
Vanuatu	1990	Dislocation of shoulder	0.062
Vanuatu	1995	Dislocation of shoulder	0.062
Vanuatu	2000	Dislocation of shoulder	0.062
Vanuatu	2005	Dislocation of shoulder	0.062
Vanuatu	2010	Dislocation of shoulder	0.062
Vanuatu	2017	Dislocation of shoulder	0.062
Vanuatu	1990	Muscle and tendon injuries, including sprains and strains lesser dislocations	0.0075
Vanuatu	1995	Muscle and tendon injuries, including sprains and strains lesser dislocations	0.0075
Vanuatu	2000	Muscle and tendon injuries, including sprains and strains lesser dislocations	0.0075
Vanuatu	2005	Muscle and tendon injuries, including sprains and strains lesser dislocations	0.0075
Vanuatu	2010	Muscle and tendon injuries, including sprains and strains lesser dislocations	0.0075
Vanuatu	2017	Muscle and tendon injuries, including sprains and strains lesser dislocations	0.0075
Vanuatu	1990	Fracture of clavicle, scapula, or humerus	0.0349
Vanuatu	1995	Fracture of clavicle, scapula, or humerus	0.0349
Vanuatu	2000	Fracture of clavicle, scapula, or humerus	0.0349
Vanuatu	2005	Fracture of clavicle, scapula, or humerus	0.0349
Vanuatu	2010	Fracture of clavicle, scapula, or humerus	0.0349
Vanuatu	2017	Fracture of clavicle, scapula, or humerus	0.0349
Vanuatu	1990	Fracture of face bones	0.0669
Vanuatu	1995	Fracture of face bones	0.0669
Vanuatu	2000	Fracture of face bones	0.0669
Vanuatu	2005	Fracture of face bones	0.0669
Vanuatu	2010	Fracture of face bones	0.0669
Vanuatu	2017	Fracture of face bones	0.0669
Vanuatu	1990	Fracture of foot bones except ankle	0.0264
Vanuatu	1995	Fracture of foot bones except ankle	0.0264
Vanuatu	2000	Fracture of foot bones except ankle	0.0264
Vanuatu	2005	Fracture of foot bones except ankle	0.0264
Vanuatu	2010	Fracture of foot bones except ankle	0.0264
Vanuatu	2017	Fracture of foot bones except ankle	0.0264
Vanuatu	1990	Fracture of hand (wrist and other distal part of hand)	0.0138
Vanuatu	1995	Fracture of hand (wrist and other distal part of hand)	0.0138
Vanuatu	2000	Fracture of hand (wrist and other distal part of hand)	0.0138
Vanuatu	2005	Fracture of hand (wrist and other distal part of hand)	0.0138
Vanuatu	2010	Fracture of hand (wrist and other distal part of hand)	0.0138
Vanuatu	2017	Fracture of hand (wrist and other distal part of hand)	0.0138
Vanuatu	1990	Fracture of hip	0.2666
Vanuatu	1995	Fracture of hip	0.2675
Vanuatu	2000	Fracture of hip	0.2643
Vanuatu	2005	Fracture of hip	0.265
Vanuatu	2010	Fracture of hip	0.2566
Vanuatu	2017	Fracture of hip	0.246
Vanuatu	1990	Amputation of upper limbs, bilateral	0.3034
Vanuatu	1995	Amputation of upper limbs, bilateral	0.3043
Vanuatu	2000	Amputation of upper limbs, bilateral	0.301
Vanuatu	2005	Amputation of upper limbs, bilateral	0.3017
Vanuatu	2010	Amputation of upper limbs, bilateral	0.293
Vanuatu	2017	Amputation of upper limbs, bilateral	0.2821
Vanuatu	1990	Fracture of patella, tibia or fibula, or ankle	0.0554
Vanuatu	1995	Fracture of patella, tibia or fibula, or ankle	0.0554
Vanuatu	2000	Fracture of patella, tibia or fibula, or ankle	0.0554
Vanuatu	2005	Fracture of patella, tibia or fibula, or ankle	0.0554
Vanuatu	2010	Fracture of patella, tibia or fibula, or ankle	0.0554
Vanuatu	2017	Fracture of patella, tibia or fibula, or ankle	0.0554
Vanuatu	1990	Fracture of pelvis	0.1823
Vanuatu	1995	Fracture of pelvis	0.1823
Vanuatu	2000	Fracture of pelvis	0.1823
Vanuatu	2005	Fracture of pelvis	0.1823
Vanuatu	2010	Fracture of pelvis	0.1823
Vanuatu	2017	Fracture of pelvis	0.1823
Vanuatu	1990	Fracture of radius and/or ulna	0.0435
Vanuatu	1995	Fracture of radius and/or ulna	0.0435
Vanuatu	2000	Fracture of radius and/or ulna	0.0435
Vanuatu	2005	Fracture of radius and/or ulna	0.0435
Vanuatu	2010	Fracture of radius and/or ulna	0.0435
Vanuatu	2017	Fracture of radius and/or ulna	0.0435
Vanuatu	1990	Fracture of skull	0.0714
Vanuatu	1995	Fracture of skull	0.0714
Vanuatu	2000	Fracture of skull	0.0714
Vanuatu	2005	Fracture of skull	0.0714
Vanuatu	2010	Fracture of skull	0.0714
Vanuatu	2017	Fracture of skull	0.0714
Vanuatu	1990	Fracture of sternum and/or fracture of one or more ribs	0.1027
Vanuatu	1995	Fracture of sternum and/or fracture of one or more ribs	0.1027
Vanuatu	2000	Fracture of sternum and/or fracture of one or more ribs	0.1027
Vanuatu	2005	Fracture of sternum and/or fracture of one or more ribs	0.1027
Vanuatu	2010	Fracture of sternum and/or fracture of one or more ribs	0.1027
Vanuatu	2017	Fracture of sternum and/or fracture of one or more ribs	0.1027
Vanuatu	1990	Fracture of vertebral column	0.1106
Vanuatu	1995	Fracture of vertebral column	0.1106
Vanuatu	2000	Fracture of vertebral column	0.1106
Vanuatu	2005	Fracture of vertebral column	0.1106
Vanuatu	2010	Fracture of vertebral column	0.1106
Vanuatu	2017	Fracture of vertebral column	0.1106
Vanuatu	1990	Fracture of femur, other than femoral neck	0.042
Vanuatu	1995	Fracture of femur, other than femoral neck	0.042
Vanuatu	2000	Fracture of femur, other than femoral neck	0.042
Vanuatu	2005	Fracture of femur, other than femoral neck	0.042
Vanuatu	2010	Fracture of femur, other than femoral neck	0.042
Vanuatu	2017	Fracture of femur, other than femoral neck	0.042
Vanuatu	1990	Minor TBI	0.1319
Vanuatu	1995	Minor TBI	0.1319
Vanuatu	2000	Minor TBI	0.1319
Vanuatu	2005	Minor TBI	0.1319
Vanuatu	2010	Minor TBI	0.1319
Vanuatu	2017	Minor TBI	0.1319
Vanuatu	1990	Moderate TBI	0.1637
Vanuatu	1995	Moderate TBI	0.1637
Vanuatu	2000	Moderate TBI	0.1637
Vanuatu	2005	Moderate TBI	0.1637
Vanuatu	2010	Moderate TBI	0.1637
Vanuatu	2017	Moderate TBI	0.1637
Vanuatu	1990	Amputation of fingers (excluding thumb)	0.0047
Vanuatu	1995	Amputation of fingers (excluding thumb)	0.0047
Vanuatu	2000	Amputation of fingers (excluding thumb)	0.0047
Vanuatu	2005	Amputation of fingers (excluding thumb)	0.0047
Vanuatu	2010	Amputation of fingers (excluding thumb)	0.0047
Vanuatu	2017	Amputation of fingers (excluding thumb)	0.0047
Vanuatu	1990	Spinal cord lesion at neck level	0.4841
Vanuatu	1995	Spinal cord lesion at neck level	0.4844
Vanuatu	2000	Spinal cord lesion at neck level	0.4833
Vanuatu	2005	Spinal cord lesion at neck level	0.4835
Vanuatu	2010	Spinal cord lesion at neck level	0.4806
Vanuatu	2017	Spinal cord lesion at neck level	0.4769
Vanuatu	1990	Spinal cord lesion below neck level	0.3302
Vanuatu	1995	Spinal cord lesion below neck level	0.3308
Vanuatu	2000	Spinal cord lesion below neck level	0.3285
Vanuatu	2005	Spinal cord lesion below neck level	0.329
Vanuatu	2010	Spinal cord lesion below neck level	0.3228
Vanuatu	2017	Spinal cord lesion below neck level	0.3151
Vanuatu	1990	Drowning and nonfatal submersion	0.2471
Vanuatu	1995	Drowning and nonfatal submersion	0.2471
Vanuatu	2000	Drowning and nonfatal submersion	0.2471
Vanuatu	2005	Drowning and nonfatal submersion	0.2471
Vanuatu	2010	Drowning and nonfatal submersion	0.2471
Vanuatu	2017	Drowning and nonfatal submersion	0.2471
Vanuatu	1990	Asphyxiation	0.2471
Vanuatu	1995	Asphyxiation	0.2471
Vanuatu	2000	Asphyxiation	0.2471
Vanuatu	2005	Asphyxiation	0.2471
Vanuatu	2010	Asphyxiation	0.2471
Vanuatu	2017	Asphyxiation	0.2471
Vanuatu	1990	Crush injury	0.1325
Vanuatu	1995	Crush injury	0.1325
Vanuatu	2000	Crush injury	0.1325
Vanuatu	2005	Crush injury	0.1325
Vanuatu	2010	Crush injury	0.1325
Vanuatu	2017	Crush injury	0.1325
Vanuatu	1990	Nerve injury	0.1129
Vanuatu	1995	Nerve injury	0.1129
Vanuatu	2000	Nerve injury	0.1129
Vanuatu	2005	Nerve injury	0.1129
Vanuatu	2010	Nerve injury	0.1129
Vanuatu	2017	Nerve injury	0.1129
Vanuatu	1990	Injury to eyes	0.0165
Vanuatu	1995	Injury to eyes	0.0165
Vanuatu	2000	Injury to eyes	0.0165
Vanuatu	2005	Injury to eyes	0.0165
Vanuatu	2010	Injury to eyes	0.0165
Vanuatu	2017	Injury to eyes	0.0165
Vanuatu	1990	Amputation of lower limb, unilateral	0.1204
Vanuatu	1995	Amputation of lower limb, unilateral	0.1207
Vanuatu	2000	Amputation of lower limb, unilateral	0.1195
Vanuatu	2005	Amputation of lower limb, unilateral	0.1198
Vanuatu	2010	Amputation of lower limb, unilateral	0.1165
Vanuatu	2017	Amputation of lower limb, unilateral	0.1124
Vanuatu	1990	Open wound(s)	0.011
Vanuatu	1995	Open wound(s)	0.011
Vanuatu	2000	Open wound(s)	0.011
Vanuatu	2005	Open wound(s)	0.011
Vanuatu	2010	Open wound(s)	0.011
Vanuatu	2017	Open wound(s)	0.011
Vanuatu	1990	Poisoning requiring urgent care	0.1628
Vanuatu	1995	Poisoning requiring urgent care	0.1628
Vanuatu	2000	Poisoning requiring urgent care	0.1628
Vanuatu	2005	Poisoning requiring urgent care	0.1628
Vanuatu	2010	Poisoning requiring urgent care	0.1628
Vanuatu	2017	Poisoning requiring urgent care	0.1628
Vanuatu	1990	Severe chest injury	0.0474
Vanuatu	1995	Severe chest injury	0.0474
Vanuatu	2000	Severe chest injury	0.0474
Vanuatu	2005	Severe chest injury	0.0474
Vanuatu	2010	Severe chest injury	0.0474
Vanuatu	2017	Severe chest injury	0.0474
Vanuatu	1990	Internal hemorrhage in abdomen and pelvis	0.1143
Vanuatu	1995	Internal hemorrhage in abdomen and pelvis	0.1143
Vanuatu	2000	Internal hemorrhage in abdomen and pelvis	0.1143
Vanuatu	2005	Internal hemorrhage in abdomen and pelvis	0.1143
Vanuatu	2010	Internal hemorrhage in abdomen and pelvis	0.1143
Vanuatu	2017	Internal hemorrhage in abdomen and pelvis	0.1143
Vanuatu	1990	Contusion in any part of the body	0.0075
Vanuatu	1995	Contusion in any part of the body	0.0075
Vanuatu	2000	Contusion in any part of the body	0.0075
Vanuatu	2005	Contusion in any part of the body	0.0075
Vanuatu	2010	Contusion in any part of the body	0.0075
Vanuatu	2017	Contusion in any part of the body	0.0075
Vanuatu	1990	Effect of different environmental factors	0.049
Vanuatu	1995	Effect of different environmental factors	0.049
Vanuatu	2000	Effect of different environmental factors	0.049
Vanuatu	2005	Effect of different environmental factors	0.049
Vanuatu	2010	Effect of different environmental factors	0.049
Vanuatu	2017	Effect of different environmental factors	0.049
Vanuatu	1990	Complications following therapeutic procedures	0.0506
Vanuatu	1995	Complications following therapeutic procedures	0.0506
Vanuatu	2000	Complications following therapeutic procedures	0.0506
Vanuatu	2005	Complications following therapeutic procedures	0.0506
Vanuatu	2010	Complications following therapeutic procedures	0.0506
Vanuatu	2017	Complications following therapeutic procedures	0.0506
Vanuatu	1990	Multiple fractures, dislocations, crashes, wounds, sprains, and strains	0.2666
Vanuatu	1995	Multiple fractures, dislocations, crashes, wounds, sprains, and strains	0.2675
Vanuatu	2000	Multiple fractures, dislocations, crashes, wounds, sprains, and strains	0.2643
Vanuatu	2005	Multiple fractures, dislocations, crashes, wounds, sprains, and strains	0.265
Vanuatu	2010	Multiple fractures, dislocations, crashes, wounds, sprains, and strains	0.2566
Vanuatu	2017	Multiple fractures, dislocations, crashes, wounds, sprains, and strains	0.246
Vanuatu	1990	Amputation of upper limb, unilateral	0.0869
Vanuatu	1995	Amputation of upper limb, unilateral	0.0871
Vanuatu	2000	Amputation of upper limb, unilateral	0.0864
Vanuatu	2005	Amputation of upper limb, unilateral	0.0866
Vanuatu	2010	Amputation of upper limb, unilateral	0.0846
Vanuatu	2017	Amputation of upper limb, unilateral	0.0822
Vanuatu	1990	Amputation of thumb	0.0114
Vanuatu	1995	Amputation of thumb	0.0114
Vanuatu	2000	Amputation of thumb	0.0114
Vanuatu	2005	Amputation of thumb	0.0114
Vanuatu	2010	Amputation of thumb	0.0114
Vanuatu	2017	Amputation of thumb	0.0114
Vanuatu	1990	Amputation of toe/toes	0.0059
Vanuatu	1995	Amputation of toe/toes	0.0059
Vanuatu	2000	Amputation of toe/toes	0.0059
Vanuatu	2005	Amputation of toe/toes	0.0059
Vanuatu	2010	Amputation of toe/toes	0.0059
Vanuatu	2017	Amputation of toe/toes	0.0059
Vanuatu	1990	Burns, <20% total burned surface area without lower airway burns	0.0163
Vanuatu	1995	Burns, <20% total burned surface area without lower airway burns	0.0163
Vanuatu	2000	Burns, <20% total burned surface area without lower airway burns	0.0163
Vanuatu	2005	Burns, <20% total burned surface area without lower airway burns	0.0163
Vanuatu	2010	Burns, <20% total burned surface area without lower airway burns	0.0163
Vanuatu	2017	Burns, <20% total burned surface area without lower airway burns	0.0163
Vanuatu	1990	Burns, >=20% total burned surface area or >= 10% burned surface area if head/neck or hands/wrist involved w/o lower airway burns	0.3291
Vanuatu	1995	Burns, >=20% total burned surface area or >= 10% burned surface area if head/neck or hands/wrist involved w/o lower airway burns	0.33
Vanuatu	2000	Burns, >=20% total burned surface area or >= 10% burned surface area if head/neck or hands/wrist involved w/o lower airway burns	0.327
Vanuatu	2005	Burns, >=20% total burned surface area or >= 10% burned surface area if head/neck or hands/wrist involved w/o lower airway burns	0.3277
Vanuatu	2010	Burns, >=20% total burned surface area or >= 10% burned surface area if head/neck or hands/wrist involved w/o lower airway burns	0.3198
Vanuatu	2017	Burns, >=20% total burned surface area or >= 10% burned surface area if head/neck or hands/wrist involved w/o lower airway burns	0.3099
Armenia	1990	Amputation of lower limbs, bilateral	0.1887
Armenia	1995	Amputation of lower limbs, bilateral	0.1887
Armenia	2000	Amputation of lower limbs, bilateral	0.1781
Armenia	2005	Amputation of lower limbs, bilateral	0.1668
Armenia	2010	Amputation of lower limbs, bilateral	0.1542
Armenia	2017	Amputation of lower limbs, bilateral	0.1365
Armenia	1990	Lower airway burns	0.2252
Armenia	1995	Lower airway burns	0.2252
Armenia	2000	Lower airway burns	0.2252
Armenia	2005	Lower airway burns	0.2252
Armenia	2010	Lower airway burns	0.2252
Armenia	2017	Lower airway burns	0.2252
Armenia	1990	Dislocation of hip	0.0159
Armenia	1995	Dislocation of hip	0.0159
Armenia	2000	Dislocation of hip	0.0159
Armenia	2005	Dislocation of hip	0.0159
Armenia	2010	Dislocation of hip	0.0159
Armenia	2017	Dislocation of hip	0.0159
Armenia	1990	Dislocation of knee	0.1134
Armenia	1995	Dislocation of knee	0.1134
Armenia	2000	Dislocation of knee	0.1134
Armenia	2005	Dislocation of knee	0.1134
Armenia	2010	Dislocation of knee	0.1134
Armenia	2017	Dislocation of knee	0.1134
Armenia	1990	Dislocation of shoulder	0.062
Armenia	1995	Dislocation of shoulder	0.062
Armenia	2000	Dislocation of shoulder	0.062
Armenia	2005	Dislocation of shoulder	0.062
Armenia	2010	Dislocation of shoulder	0.062
Armenia	2017	Dislocation of shoulder	0.062
Armenia	1990	Muscle and tendon injuries, including sprains and strains lesser dislocations	0.0075
Armenia	1995	Muscle and tendon injuries, including sprains and strains lesser dislocations	0.0075
Armenia	2000	Muscle and tendon injuries, including sprains and strains lesser dislocations	0.0075
Armenia	2005	Muscle and tendon injuries, including sprains and strains lesser dislocations	0.0075
Armenia	2010	Muscle and tendon injuries, including sprains and strains lesser dislocations	0.0075
Armenia	2017	Muscle and tendon injuries, including sprains and strains lesser dislocations	0.0075
Armenia	1990	Fracture of clavicle, scapula, or humerus	0.0349
Armenia	1995	Fracture of clavicle, scapula, or humerus	0.0349
Armenia	2000	Fracture of clavicle, scapula, or humerus	0.0349
Armenia	2005	Fracture of clavicle, scapula, or humerus	0.0349
Armenia	2010	Fracture of clavicle, scapula, or humerus	0.0349
Armenia	2017	Fracture of clavicle, scapula, or humerus	0.0349
Armenia	1990	Fracture of face bones	0.0669
Armenia	1995	Fracture of face bones	0.0669
Armenia	2000	Fracture of face bones	0.0669
Armenia	2005	Fracture of face bones	0.0669
Armenia	2010	Fracture of face bones	0.0669
Armenia	2017	Fracture of face bones	0.0669
Armenia	1990	Fracture of foot bones except ankle	0.0264
Armenia	1995	Fracture of foot bones except ankle	0.0264
Armenia	2000	Fracture of foot bones except ankle	0.0264
Armenia	2005	Fracture of foot bones except ankle	0.0264
Armenia	2010	Fracture of foot bones except ankle	0.0264
Armenia	2017	Fracture of foot bones except ankle	0.0264
Armenia	1990	Fracture of hand (wrist and other distal part of hand)	0.0138
Armenia	1995	Fracture of hand (wrist and other distal part of hand)	0.0138
Armenia	2000	Fracture of hand (wrist and other distal part of hand)	0.0138
Armenia	2005	Fracture of hand (wrist and other distal part of hand)	0.0138
Armenia	2010	Fracture of hand (wrist and other distal part of hand)	0.0138
Armenia	2017	Fracture of hand (wrist and other distal part of hand)	0.0138
Armenia	1990	Fracture of hip	0.1442
Armenia	1995	Fracture of hip	0.1442
Armenia	2000	Fracture of hip	0.1303
Armenia	2005	Fracture of hip	0.1153
Armenia	2010	Fracture of hip	0.0986
Armenia	2017	Fracture of hip	0.0752
Armenia	1990	Amputation of upper limbs, bilateral	0.1769
Armenia	1995	Amputation of upper limbs, bilateral	0.177
Armenia	2000	Amputation of upper limbs, bilateral	0.1626
Armenia	2005	Amputation of upper limbs, bilateral	0.1472
Armenia	2010	Amputation of upper limbs, bilateral	0.1298
Armenia	2017	Amputation of upper limbs, bilateral	0.1058
Armenia	1990	Fracture of patella, tibia or fibula, or ankle	0.0554
Armenia	1995	Fracture of patella, tibia or fibula, or ankle	0.0554
Armenia	2000	Fracture of patella, tibia or fibula, or ankle	0.0554
Armenia	2005	Fracture of patella, tibia or fibula, or ankle	0.0554
Armenia	2010	Fracture of patella, tibia or fibula, or ankle	0.0554
Armenia	2017	Fracture of patella, tibia or fibula, or ankle	0.0554
Armenia	1990	Fracture of pelvis	0.1823
Armenia	1995	Fracture of pelvis	0.1823
Armenia	2000	Fracture of pelvis	0.1823
Armenia	2005	Fracture of pelvis	0.1823
Armenia	2010	Fracture of pelvis	0.1823
Armenia	2017	Fracture of pelvis	0.1823
Armenia	1990	Fracture of radius and/or ulna	0.0435
Armenia	1995	Fracture of radius and/or ulna	0.0435
Armenia	2000	Fracture of radius and/or ulna	0.0435
Armenia	2005	Fracture of radius and/or ulna	0.0435
Armenia	2010	Fracture of radius and/or ulna	0.0435
Armenia	2017	Fracture of radius and/or ulna	0.0435
Armenia	1990	Fracture of skull	0.0714
Armenia	1995	Fracture of skull	0.0714
Armenia	2000	Fracture of skull	0.0714
Armenia	2005	Fracture of skull	0.0714
Armenia	2010	Fracture of skull	0.0714
Armenia	2017	Fracture of skull	0.0714
Armenia	1990	Fracture of sternum and/or fracture of one or more ribs	0.1027
Armenia	1995	Fracture of sternum and/or fracture of one or more ribs	0.1027
Armenia	2000	Fracture of sternum and/or fracture of one or more ribs	0.1027
Armenia	2005	Fracture of sternum and/or fracture of one or more ribs	0.1027
Armenia	2010	Fracture of sternum and/or fracture of one or more ribs	0.1027
Armenia	2017	Fracture of sternum and/or fracture of one or more ribs	0.1027
Armenia	1990	Fracture of vertebral column	0.1106
Armenia	1995	Fracture of vertebral column	0.1106
Armenia	2000	Fracture of vertebral column	0.1106
Armenia	2005	Fracture of vertebral column	0.1106
Armenia	2010	Fracture of vertebral column	0.1106
Armenia	2017	Fracture of vertebral column	0.1106
Armenia	1990	Fracture of femur, other than femoral neck	0.042
Armenia	1995	Fracture of femur, other than femoral neck	0.042
Armenia	2000	Fracture of femur, other than femoral neck	0.042
Armenia	2005	Fracture of femur, other than femoral neck	0.042
Armenia	2010	Fracture of femur, other than femoral neck	0.042
Armenia	2017	Fracture of femur, other than femoral neck	0.042
Armenia	1990	Minor TBI	0.1319
Armenia	1995	Minor TBI	0.1319
Armenia	2000	Minor TBI	0.1319
Armenia	2005	Minor TBI	0.1319
Armenia	2010	Minor TBI	0.1319
Armenia	2017	Minor TBI	0.1319
Armenia	1990	Moderate TBI	0.1637
Armenia	1995	Moderate TBI	0.1637
Armenia	2000	Moderate TBI	0.1637
Armenia	2005	Moderate TBI	0.1637
Armenia	2010	Moderate TBI	0.1637
Armenia	2017	Moderate TBI	0.1637
Armenia	1990	Amputation of fingers (excluding thumb)	0.0047
Armenia	1995	Amputation of fingers (excluding thumb)	0.0047
Armenia	2000	Amputation of fingers (excluding thumb)	0.0047
Armenia	2005	Amputation of fingers (excluding thumb)	0.0047
Armenia	2010	Amputation of fingers (excluding thumb)	0.0047
Armenia	2017	Amputation of fingers (excluding thumb)	0.0047
Armenia	1990	Spinal cord lesion at neck level	0.4417
Armenia	1995	Spinal cord lesion at neck level	0.4417
Armenia	2000	Spinal cord lesion at neck level	0.4369
Armenia	2005	Spinal cord lesion at neck level	0.4317
Armenia	2010	Spinal cord lesion at neck level	0.426
Armenia	2017	Spinal cord lesion at neck level	0.4179
Armenia	1990	Spinal cord lesion below neck level	0.2408
Armenia	1995	Spinal cord lesion below neck level	0.2408
Armenia	2000	Spinal cord lesion below neck level	0.2307
Armenia	2005	Spinal cord lesion below neck level	0.2197
Armenia	2010	Spinal cord lesion below neck level	0.2075
Armenia	2017	Spinal cord lesion below neck level	0.1905
Armenia	1990	Drowning and nonfatal submersion	0.2471
Armenia	1995	Drowning and nonfatal submersion	0.2471
Armenia	2000	Drowning and nonfatal submersion	0.2471
Armenia	2005	Drowning and nonfatal submersion	0.2471
Armenia	2010	Drowning and nonfatal submersion	0.2471
Armenia	2017	Drowning and nonfatal submersion	0.2471
Armenia	1990	Asphyxiation	0.2471
Armenia	1995	Asphyxiation	0.2471
Armenia	2000	Asphyxiation	0.2471
Armenia	2005	Asphyxiation	0.2471
Armenia	2010	Asphyxiation	0.2471
Armenia	2017	Asphyxiation	0.2471
Armenia	1990	Crush injury	0.1325
Armenia	1995	Crush injury	0.1325
Armenia	2000	Crush injury	0.1325
Armenia	2005	Crush injury	0.1325
Armenia	2010	Crush injury	0.1325
Armenia	2017	Crush injury	0.1325
Armenia	1990	Nerve injury	0.1129
Armenia	1995	Nerve injury	0.1129
Armenia	2000	Nerve injury	0.1129
Armenia	2005	Nerve injury	0.1129
Armenia	2010	Nerve injury	0.1129
Armenia	2017	Nerve injury	0.1129
Armenia	1990	Injury to eyes	0.0165
Armenia	1995	Injury to eyes	0.0165
Armenia	2000	Injury to eyes	0.0165
Armenia	2005	Injury to eyes	0.0165
Armenia	2010	Injury to eyes	0.0165
Armenia	2017	Injury to eyes	0.0165
Armenia	1990	Amputation of lower limb, unilateral	0.0727
Armenia	1995	Amputation of lower limb, unilateral	0.0727
Armenia	2000	Amputation of lower limb, unilateral	0.0673
Armenia	2005	Amputation of lower limb, unilateral	0.0614
Armenia	2010	Amputation of lower limb, unilateral	0.0549
Armenia	2017	Amputation of lower limb, unilateral	0.0458
Armenia	1990	Open wound(s)	0.011
Armenia	1995	Open wound(s)	0.011
Armenia	2000	Open wound(s)	0.011
Armenia	2005	Open wound(s)	0.011

Armenia	2010	Open wound(s)	0.011
Armenia	2017	Open wound(s)	0.011
Armenia	1990	Poisoning requiring urgent care	0.1628
Armenia	1995	Poisoning requiring urgent care	0.1628
Armenia	2000	Poisoning requiring urgent care	0.1628
Armenia	2005	Poisoning requiring urgent care	0.1628
Armenia	2010	Poisoning requiring urgent care	0.1628
Armenia	2017	Poisoning requiring urgent care	0.1628
Armenia	1990	Severe chest injury	0.0474
Armenia	1995	Severe chest injury	0.0474
Armenia	2000	Severe chest injury	0.0474
Armenia	2005	Severe chest injury	0.0474
Armenia	2010	Severe chest injury	0.0474
Armenia	2017	Severe chest injury	0.0474
Armenia	1990	Internal hemorrhage in abdomen and pelvis	0.1143
Armenia	1995	Internal hemorrhage in abdomen and pelvis	0.1143
Armenia	2000	Internal hemorrhage in abdomen and pelvis	0.1143
Armenia	2005	Internal hemorrhage in abdomen and pelvis	0.1143
Armenia	2010	Internal hemorrhage in abdomen and pelvis	0.1143
Armenia	2017	Internal hemorrhage in abdomen and pelvis	0.1143
Armenia	1990	Contusion in any part of the body	0.0075
Armenia	1995	Contusion in any part of the body	0.0075
Armenia	2000	Contusion in any part of the body	0.0075
Armenia	2005	Contusion in any part of the body	0.0075
Armenia	2010	Contusion in any part of the body	0.0075
Armenia	2017	Contusion in any part of the body	0.0075
Armenia	1990	Effect of different environmental factors	0.049
Armenia	1995	Effect of different environmental factors	0.049
Armenia	2000	Effect of different environmental factors	0.049
Armenia	2005	Effect of different environmental factors	0.049
Armenia	2010	Effect of different environmental factors	0.049
Armenia	2017	Effect of different environmental factors	0.049
Armenia	1990	Complications following therapeutic procedures	0.0506
Armenia	1995	Complications following therapeutic procedures	0.0506
Armenia	2000	Complications following therapeutic procedures	0.0506
Armenia	2005	Complications following therapeutic procedures	0.0506
Armenia	2010	Complications following therapeutic procedures	0.0506
Armenia	2017	Complications following therapeutic procedures	0.0506
Armenia	1990	Multiple fractures, dislocations, crashes, wounds, sprains, and strains	0.1442
Armenia	1995	Multiple fractures, dislocations, crashes, wounds, sprains, and strains	0.1442
Armenia	2000	Multiple fractures, dislocations, crashes, wounds, sprains, and strains	0.1303
Armenia	2005	Multiple fractures, dislocations, crashes, wounds, sprains, and strains	0.1153
Armenia	2010	Multiple fractures, dislocations, crashes, wounds, sprains, and strains	0.0986
Armenia	2017	Multiple fractures, dislocations, crashes, wounds, sprains, and strains	0.0752
Armenia	1990	Amputation of upper limb, unilateral	0.0588
Armenia	1995	Amputation of upper limb, unilateral	0.0588
Armenia	2000	Amputation of upper limb, unilateral	0.0556
Armenia	2005	Amputation of upper limb, unilateral	0.0522
Armenia	2010	Amputation of upper limb, unilateral	0.0484
Armenia	2017	Amputation of upper limb, unilateral	0.043
Armenia	1990	Amputation of thumb	0.0114
Armenia	1995	Amputation of thumb	0.0114
Armenia	2000	Amputation of thumb	0.0114
Armenia	2005	Amputation of thumb	0.0114
Armenia	2010	Amputation of thumb	0.0114
Armenia	2017	Amputation of thumb	0.0114
Armenia	1990	Amputation of toe/toes	0.0059
Armenia	1995	Amputation of toe/toes	0.0059
Armenia	2000	Amputation of toe/toes	0.0059
Armenia	2005	Amputation of toe/toes	0.0059
Armenia	2010	Amputation of toe/toes	0.0059
Armenia	2017	Amputation of toe/toes	0.0059
Armenia	1990	Burns, <20% total burned surface area without lower airway burns	0.0163
Armenia	1995	Burns, <20% total burned surface area without lower airway burns	0.0163
Armenia	2000	Burns, <20% total burned surface area without lower airway burns	0.0163
Armenia	2005	Burns, <20% total burned surface area without lower airway burns	0.0163
Armenia	2010	Burns, <20% total burned surface area without lower airway burns	0.0163
Armenia	2017	Burns, <20% total burned surface area without lower airway burns	0.0163
Armenia	1990	Burns, >=20% total burned surface area or >= 10% burned surface area if head/neck or hands/wrist involved w/o lower airway burns	0.2151
Armenia	1995	Burns, >=20% total burned surface area or >= 10% burned surface area if head/neck or hands/wrist involved w/o lower airway burns	0.2151
Armenia	2000	Burns, >=20% total burned surface area or >= 10% burned surface area if head/neck or hands/wrist involved w/o lower airway burns	0.2022
Armenia	2005	Burns, >=20% total burned surface area or >= 10% burned surface area if head/neck or hands/wrist involved w/o lower airway burns	0.1883
Armenia	2010	Burns, >=20% total burned surface area or >= 10% burned surface area if head/neck or hands/wrist involved w/o lower airway burns	0.1726
Armenia	2017	Burns, >=20% total burned surface area or >= 10% burned surface area if head/neck or hands/wrist involved w/o lower airway burns	0.1509
Azerbaijan	1990	Amputation of lower limbs, bilateral	0.2092
Azerbaijan	1995	Amputation of lower limbs, bilateral	0.2065
Azerbaijan	2000	Amputation of lower limbs, bilateral	0.202
Azerbaijan	2005	Amputation of lower limbs, bilateral	0.1983
Azerbaijan	2010	Amputation of lower limbs, bilateral	0.1759
Azerbaijan	2017	Amputation of lower limbs, bilateral	0.1535
Azerbaijan	1990	Lower airway burns	0.2252
Azerbaijan	1995	Lower airway burns	0.2252
Azerbaijan	2000	Lower airway burns	0.2252
Azerbaijan	2005	Lower airway burns	0.2252
Azerbaijan	2010	Lower airway burns	0.2252
Azerbaijan	2017	Lower airway burns	0.2252
Azerbaijan	1990	Dislocation of hip	0.0159
Azerbaijan	1995	Dislocation of hip	0.0159
Azerbaijan	2000	Dislocation of hip	0.0159
Azerbaijan	2005	Dislocation of hip	0.0159
Azerbaijan	2010	Dislocation of hip	0.0159
Azerbaijan	2017	Dislocation of hip	0.0159
Azerbaijan	1990	Dislocation of knee	0.1134
Azerbaijan	1995	Dislocation of knee	0.1134
Azerbaijan	2000	Dislocation of knee	0.1134
Azerbaijan	2005	Dislocation of knee	0.1134
Azerbaijan	2010	Dislocation of knee	0.1134
Azerbaijan	2017	Dislocation of knee	0.1134
Azerbaijan	1990	Dislocation of shoulder	0.062
Azerbaijan	1995	Dislocation of shoulder	0.062
Azerbaijan	2000	Dislocation of shoulder	0.062
Azerbaijan	2005	Dislocation of shoulder	0.062
Azerbaijan	2010	Dislocation of shoulder	0.062
Azerbaijan	2017	Dislocation of shoulder	0.062
Azerbaijan	1990	Muscle and tendon injuries, including sprains and strains lesser dislocations	0.0075
Azerbaijan	1995	Muscle and tendon injuries, including sprains and strains lesser dislocations	0.0075
Azerbaijan	2000	Muscle and tendon injuries, including sprains and strains lesser dislocations	0.0075
Azerbaijan	2005	Muscle and tendon injuries, including sprains and strains lesser dislocations	0.0075
Azerbaijan	2010	Muscle and tendon injuries, including sprains and strains lesser dislocations	0.0075
Azerbaijan	2017	Muscle and tendon injuries, including sprains and strains lesser dislocations	0.0075
Azerbaijan	1990	Fracture of clavicle, scapula, or humerus	0.0349
Azerbaijan	1995	Fracture of clavicle, scapula, or humerus	0.0349
Azerbaijan	2000	Fracture of clavicle, scapula, or humerus	0.0349
Azerbaijan	2005	Fracture of clavicle, scapula, or humerus	0.0349
Azerbaijan	2010	Fracture of clavicle, scapula, or humerus	0.0349
Azerbaijan	2017	Fracture of clavicle, scapula, or humerus	0.0349
Azerbaijan	1990	Fracture of face bones	0.0669
Azerbaijan	1995	Fracture of face bones	0.0669
Azerbaijan	2000	Fracture of face bones	0.0669
Azerbaijan	2005	Fracture of face bones	0.0669
Azerbaijan	2010	Fracture of face bones	0.0669
Azerbaijan	2017	Fracture of face bones	0.0669
Azerbaijan	1990	Fracture of foot bones except ankle	0.0264
Azerbaijan	1995	Fracture of foot bones except ankle	0.0264
Azerbaijan	2000	Fracture of foot bones except ankle	0.0264
Azerbaijan	2005	Fracture of foot bones except ankle	0.0264
Azerbaijan	2010	Fracture of foot bones except ankle	0.0264
Azerbaijan	2017	Fracture of foot bones except ankle	0.0264
Azerbaijan	1990	Fracture of hand (wrist and other distal part of hand)	0.0138
Azerbaijan	1995	Fracture of hand (wrist and other distal part of hand)	0.0138
Azerbaijan	2000	Fracture of hand (wrist and other distal part of hand)	0.0138
Azerbaijan	2005	Fracture of hand (wrist and other distal part of hand)	0.0138
Azerbaijan	2010	Fracture of hand (wrist and other distal part of hand)	0.0138
Azerbaijan	2017	Fracture of hand (wrist and other distal part of hand)	0.0138
Azerbaijan	1990	Fracture of hip	0.1714
Azerbaijan	1995	Fracture of hip	0.1678
Azerbaijan	2000	Fracture of hip	0.1619
Azerbaijan	2005	Fracture of hip	0.157
Azerbaijan	2010	Fracture of hip	0.1273
Azerbaijan	2017	Fracture of hip	0.0977
Azerbaijan	1990	Amputation of upper limbs, bilateral	0.205
Azerbaijan	1995	Amputation of upper limbs, bilateral	0.2014
Azerbaijan	2000	Amputation of upper limbs, bilateral	0.1953
Azerbaijan	2005	Amputation of upper limbs, bilateral	0.1902
Azerbaijan	2010	Amputation of upper limbs, bilateral	0.1596
Azerbaijan	2017	Amputation of upper limbs, bilateral	0.129
Azerbaijan	1990	Fracture of patella, tibia or fibula, or ankle	0.0554
Azerbaijan	1995	Fracture of patella, tibia or fibula, or ankle	0.0554
Azerbaijan	2000	Fracture of patella, tibia or fibula, or ankle	0.0554
Azerbaijan	2005	Fracture of patella, tibia or fibula, or ankle	0.0554
Azerbaijan	2010	Fracture of patella, tibia or fibula, or ankle	0.0554
Azerbaijan	2017	Fracture of patella, tibia or fibula, or ankle	0.0554
Azerbaijan	1990	Fracture of pelvis	0.1823
Azerbaijan	1995	Fracture of pelvis	0.1823
Azerbaijan	2000	Fracture of pelvis	0.1823
Azerbaijan	2005	Fracture of pelvis	0.1823
Azerbaijan	2010	Fracture of pelvis	0.1823
Azerbaijan	2017	Fracture of pelvis	0.1823
Azerbaijan	1990	Fracture of radius and/or ulna	0.0435
Azerbaijan	1995	Fracture of radius and/or ulna	0.0435
Azerbaijan	2000	Fracture of radius and/or ulna	0.0435
Azerbaijan	2005	Fracture of radius and/or ulna	0.0435
Azerbaijan	2010	Fracture of radius and/or ulna	0.0435
Azerbaijan	2017	Fracture of radius and/or ulna	0.0435
Azerbaijan	1990	Fracture of skull	0.0714
Azerbaijan	1995	Fracture of skull	0.0714
Azerbaijan	2000	Fracture of skull	0.0714
Azerbaijan	2005	Fracture of skull	0.0714
Azerbaijan	2010	Fracture of skull	0.0714
Azerbaijan	2017	Fracture of skull	0.0714
Azerbaijan	1990	Fracture of sternum and/or fracture of one or more ribs	0.1027
Azerbaijan	1995	Fracture of sternum and/or fracture of one or more ribs	0.1027
Azerbaijan	2000	Fracture of sternum and/or fracture of one or more ribs	0.1027
Azerbaijan	2005	Fracture of sternum and/or fracture of one or more ribs	0.1027
Azerbaijan	2010	Fracture of sternum and/or fracture of one or more ribs	0.1027
Azerbaijan	2017	Fracture of sternum and/or fracture of one or more ribs	0.1027
Azerbaijan	1990	Fracture of vertebral column	0.1106
Azerbaijan	1995	Fracture of vertebral column	0.1106
Azerbaijan	2000	Fracture of vertebral column	0.1106
Azerbaijan	2005	Fracture of vertebral column	0.1106
Azerbaijan	2010	Fracture of vertebral column	0.1106
Azerbaijan	2017	Fracture of vertebral column	0.1106
Azerbaijan	1990	Fracture of femur, other than femoral neck	0.042
Azerbaijan	1995	Fracture of femur, other than femoral neck	0.042
Azerbaijan	2000	Fracture of femur, other than femoral neck	0.042
Azerbaijan	2005	Fracture of femur, other than femoral neck	0.042
Azerbaijan	2010	Fracture of femur, other than femoral neck	0.042
Azerbaijan	2017	Fracture of femur, other than femoral neck	0.042
Azerbaijan	1990	Minor TBI	0.1319
Azerbaijan	1995	Minor TBI	0.1319
Azerbaijan	2000	Minor TBI	0.1319
Azerbaijan	2005	Minor TBI	0.1319
Azerbaijan	2010	Minor TBI	0.1319
Azerbaijan	2017	Minor TBI	0.1319
Azerbaijan	1990	Moderate TBI	0.1637
Azerbaijan	1995	Moderate TBI	0.1637
Azerbaijan	2000	Moderate TBI	0.1637
Azerbaijan	2005	Moderate TBI	0.1637
Azerbaijan	2010	Moderate TBI	0.1637
Azerbaijan	2017	Moderate TBI	0.1637
Azerbaijan	1990	Amputation of fingers (excluding thumb)	0.0047
Azerbaijan	1995	Amputation of fingers (excluding thumb)	0.0047
Azerbaijan	2000	Amputation of fingers (excluding thumb)	0.0047
Azerbaijan	2005	Amputation of fingers (excluding thumb)	0.0047
Azerbaijan	2010	Amputation of fingers (excluding thumb)	0.0047
Azerbaijan	2017	Amputation of fingers (excluding thumb)	0.0047
Azerbaijan	1990	Spinal cord lesion at neck level	0.4511
Azerbaijan	1995	Spinal cord lesion at neck level	0.4499
Azerbaijan	2000	Spinal cord lesion at neck level	0.4479
Azerbaijan	2005	Spinal cord lesion at neck level	0.4462
Azerbaijan	2010	Spinal cord lesion at neck level	0.4359
Azerbaijan	2017	Spinal cord lesion at neck level	0.4257
Azerbaijan	1990	Spinal cord lesion below neck level	0.2606
Azerbaijan	1995	Spinal cord lesion below neck level	0.2581
Azerbaijan	2000	Spinal cord lesion below neck level	0.2537
Azerbaijan	2005	Spinal cord lesion below neck level	0.2502
Azerbaijan	2010	Spinal cord lesion below neck level	0.2285
Azerbaijan	2017	Spinal cord lesion below neck level	0.2069
Azerbaijan	1990	Drowning and nonfatal submersion	0.2471
Azerbaijan	1995	Drowning and nonfatal submersion	0.2471
Azerbaijan	2000	Drowning and nonfatal submersion	0.2471
Azerbaijan	2005	Drowning and nonfatal submersion	0.2471
Azerbaijan	2010	Drowning and nonfatal submersion	0.2471
Azerbaijan	2017	Drowning and nonfatal submersion	0.2471
Azerbaijan	1990	Asphyxiation	0.2471
Azerbaijan	1995	Asphyxiation	0.2471
Azerbaijan	2000	Asphyxiation	0.2471
Azerbaijan	2005	Asphyxiation	0.2471
Azerbaijan	2010	Asphyxiation	0.2471
Azerbaijan	2017	Asphyxiation	0.2471
Azerbaijan	1990	Crush injury	0.1325
Azerbaijan	1995	Crush injury	0.1325
Azerbaijan	2000	Crush injury	0.1325
Azerbaijan	2005	Crush injury	0.1325
Azerbaijan	2010	Crush injury	0.1325
Azerbaijan	2017	Crush injury	0.1325
Azerbaijan	1990	Nerve injury	0.1129
Azerbaijan	1995	Nerve injury	0.1129
Azerbaijan	2000	Nerve injury	0.1129
Azerbaijan	2005	Nerve injury	0.1129
Azerbaijan	2010	Nerve injury	0.1129
Azerbaijan	2017	Nerve injury	0.1129
Azerbaijan	1990	Injury to eyes	0.0165
Azerbaijan	1995	Injury to eyes	0.0165
Azerbaijan	2000	Injury to eyes	0.0165
Azerbaijan	2005	Injury to eyes	0.0165
Azerbaijan	2010	Injury to eyes	0.0165
Azerbaijan	2017	Injury to eyes	0.0165
Azerbaijan	1990	Amputation of lower limb, unilateral	0.0833
Azerbaijan	1995	Amputation of lower limb, unilateral	0.0819
Azerbaijan	2000	Amputation of lower limb, unilateral	0.0796
Azerbaijan	2005	Amputation of lower limb, unilateral	0.0777
Azerbaijan	2010	Amputation of lower limb, unilateral	0.0661
Azerbaijan	2017	Amputation of lower limb, unilateral	0.0546
Azerbaijan	1990	Open wound(s)	0.011
Azerbaijan	1995	Open wound(s)	0.011
Azerbaijan	2000	Open wound(s)	0.011
Azerbaijan	2005	Open wound(s)	0.011
Azerbaijan	2010	Open wound(s)	0.011
Azerbaijan	2017	Open wound(s)	0.011
Azerbaijan	1990	Poisoning requiring urgent care	0.1628
Azerbaijan	1995	Poisoning requiring urgent care	0.1628
Azerbaijan	2000	Poisoning requiring urgent care	0.1628
Azerbaijan	2005	Poisoning requiring urgent care	0.1628
Azerbaijan	2010	Poisoning requiring urgent care	0.1628
Azerbaijan	2017	Poisoning requiring urgent care	0.1628
Azerbaijan	1990	Severe chest injury	0.0474
Azerbaijan	1995	Severe chest injury	0.0474
Azerbaijan	2000	Severe chest injury	0.0474
Azerbaijan	2005	Severe chest injury	0.0474
Azerbaijan	2010	Severe chest injury	0.0474
Azerbaijan	2017	Severe chest injury	0.0474
Azerbaijan	1990	Internal hemorrhage in abdomen and pelvis	0.1143
Azerbaijan	1995	Internal hemorrhage in abdomen and pelvis	0.1143
Azerbaijan	2000	Internal hemorrhage in abdomen and pelvis	0.1143
Azerbaijan	2005	Internal hemorrhage in abdomen and pelvis	0.1143
Azerbaijan	2010	Internal hemorrhage in abdomen and pelvis	0.1143
Azerbaijan	2017	Internal hemorrhage in abdomen and pelvis	0.1143
Azerbaijan	1990	Contusion in any part of the body	0.0075
Azerbaijan	1995	Contusion in any part of the body	0.0075
Azerbaijan	2000	Contusion in any part of the body	0.0075
Azerbaijan	2005	Contusion in any part of the body	0.0075
Azerbaijan	2010	Contusion in any part of the body	0.0075
Azerbaijan	2017	Contusion in any part of the body	0.0075
Azerbaijan	1990	Effect of different environmental factors	0.049
Azerbaijan	1995	Effect of different environmental factors	0.049
Azerbaijan	2000	Effect of different environmental factors	0.049
Azerbaijan	2005	Effect of different environmental factors	0.049
Azerbaijan	2010	Effect of different environmental factors	0.049
Azerbaijan	2017	Effect of different environmental factors	0.049
Azerbaijan	1990	Complications following therapeutic procedures	0.0506
Azerbaijan	1995	Complications following therapeutic procedures	0.0506
Azerbaijan	2000	Complications following therapeutic procedures	0.0506
Azerbaijan	2005	Complications following therapeutic procedures	0.0506
Azerbaijan	2010	Complications following therapeutic procedures	0.0506
Azerbaijan	2017	Complications following therapeutic procedures	0.0506
Azerbaijan	1990	Multiple fractures, dislocations, crashes, wounds, sprains, and strains	0.1714
Azerbaijan	1995	Multiple fractures, dislocations, crashes, wounds, sprains, and strains	0.1678
Azerbaijan	2000	Multiple fractures, dislocations, crashes, wounds, sprains, and strains	0.1619
Azerbaijan	2005	Multiple fractures, dislocations, crashes, wounds, sprains, and strains	0.157
Azerbaijan	2010	Multiple fractures, dislocations, crashes, wounds, sprains, and strains	0.1273
Azerbaijan	2017	Multiple fractures, dislocations, crashes, wounds, sprains, and strains	0.0977
Azerbaijan	1990	Amputation of upper limb, unilateral	0.0651
Azerbaijan	1995	Amputation of upper limb, unilateral	0.0643
Azerbaijan	2000	Amputation of upper limb, unilateral	0.0629
Azerbaijan	2005	Amputation of upper limb, unilateral	0.0618
Azerbaijan	2010	Amputation of upper limb, unilateral	0.055
Azerbaijan	2017	Amputation of upper limb, unilateral	0.0482
Azerbaijan	1990	Amputation of thumb	0.0114
Azerbaijan	1995	Amputation of thumb	0.0114
Azerbaijan	2000	Amputation of thumb	0.0114
Azerbaijan	2005	Amputation of thumb	0.0114
Azerbaijan	2010	Amputation of thumb	0.0114
Azerbaijan	2017	Amputation of thumb	0.0114
Azerbaijan	1990	Amputation of toe/toes	0.0059
Azerbaijan	1995	Amputation of toe/toes	0.0059
Azerbaijan	2000	Amputation of toe/toes	0.0059
Azerbaijan	2005	Amputation of toe/toes	0.0059
Azerbaijan	2010	Amputation of toe/toes	0.0059
Azerbaijan	2017	Amputation of toe/toes	0.0059
Azerbaijan	1990	Burns, <20% total burned surface area without lower airway burns	0.0163
Azerbaijan	1995	Burns, <20% total burned surface area without lower airway burns	0.0163
Azerbaijan	2000	Burns, <20% total burned surface area without lower airway burns	0.0163
Azerbaijan	2005	Burns, <20% total burned surface area without lower airway burns	0.0163
Azerbaijan	2010	Burns, <20% total burned surface area without lower airway burns	0.0163
Azerbaijan	2017	Burns, <20% total burned surface area without lower airway burns	0.0163
Azerbaijan	1990	Burns, >=20% total burned surface area or >= 10% burned surface area if head/neck or hands/wrist involved w/o lower airway burns	0.2404
Azerbaijan	1995	Burns, >=20% total burned surface area or >= 10% burned surface area if head/neck or hands/wrist involved w/o lower airway burns	0.2371
Azerbaijan	2000	Burns, >=20% total burned surface area or >= 10% burned surface area if head/neck or hands/wrist involved w/o lower airway burns	0.2316
Azerbaijan	2005	Burns, >=20% total burned surface area or >= 10% burned surface area if head/neck or hands/wrist involved w/o lower airway burns	0.2271
Azerbaijan	2010	Burns, >=20% total burned surface area or >= 10% burned surface area if head/neck or hands/wrist involved w/o lower airway burns	0.1995
Azerbaijan	2017	Burns, >=20% total burned surface area or >= 10% burned surface area if head/neck or hands/wrist involved w/o lower airway burns	0.1719
Georgia	1990	Amputation of lower limbs, bilateral	0.1695
Georgia	1995	Amputation of lower limbs, bilateral	0.1639
Georgia	2000	Amputation of lower limbs, bilateral	0.1633
Georgia	2005	Amputation of lower limbs, bilateral	0.1619
Georgia	2010	Amputation of lower limbs, bilateral	0.1589
Georgia	2017	Amputation of lower limbs, bilateral	0.148
Georgia	1990	Lower airway burns	0.2252
Georgia	1995	Lower airway burns	0.2252
Georgia	2000	Lower airway burns	0.2252
Georgia	2005	Lower airway burns	0.2252
Georgia	2010	Lower airway burns	0.2252
Georgia	2017	Lower airway burns	0.2252
Georgia	1990	Dislocation of hip	0.0159
Georgia	1995	Dislocation of hip	0.0159
Georgia	2000	Dislocation of hip	0.0159
Georgia	2005	Dislocation of hip	0.0159
Georgia	2010	Dislocation of hip	0.0159
Georgia	2017	Dislocation of hip	0.0159
Georgia	1990	Dislocation of knee	0.1134
Georgia	1995	Dislocation of knee	0.1134
Georgia	2000	Dislocation of knee	0.1134
Georgia	2005	Dislocation of knee	0.1134
Georgia	2010	Dislocation of knee	0.1134
Georgia	2017	Dislocation of knee	0.1134
Georgia	1990	Dislocation of shoulder	0.062
Georgia	1995	Dislocation of shoulder	0.062
Georgia	2000	Dislocation of shoulder	0.062
Georgia	2005	Dislocation of shoulder	0.062
Georgia	2010	Dislocation of shoulder	0.062
Georgia	2017	Dislocation of shoulder	0.062
Georgia	1990	Muscle and tendon injuries, including sprains and strains lesser dislocations	0.0075
Georgia	1995	Muscle and tendon injuries, including sprains and strains lesser dislocations	0.0075
Georgia	2000	Muscle and tendon injuries, including sprains and strains lesser dislocations	0.0075
Georgia	2005	Muscle and tendon injuries, including sprains and strains lesser dislocations	0.0075
Georgia	2010	Muscle and tendon injuries, including sprains and strains lesser dislocations	0.0075
Georgia	2017	Muscle and tendon injuries, including sprains and strains lesser dislocations	0.0075
Georgia	1990	Fracture of clavicle, scapula, or humerus	0.0349
Georgia	1995	Fracture of clavicle, scapula, or humerus	0.0349
Georgia	2000	Fracture of clavicle, scapula, or humerus	0.0349
Georgia	2005	Fracture of clavicle, scapula, or humerus	0.0349
Georgia	2010	Fracture of clavicle, scapula, or humerus	0.0349
Georgia	2017	Fracture of clavicle, scapula, or humerus	0.0349
Georgia	1990	Fracture of face bones	0.0669
Georgia	1995	Fracture of face bones	0.0669
Georgia	2000	Fracture of face bones	0.0669
Georgia	2005	Fracture of face bones	0.0669
Georgia	2010	Fracture of face bones	0.0669
Georgia	2017	Fracture of face bones	0.0669
Georgia	1990	Fracture of foot bones except ankle	0.0264
Georgia	1995	Fracture of foot bones except ankle	0.0264
Georgia	2000	Fracture of foot bones except ankle	0.0264
Georgia	2005	Fracture of foot bones except ankle	0.0264
Georgia	2010	Fracture of foot bones except ankle	0.0264
Georgia	2017	Fracture of foot bones except ankle	0.0264
Georgia	1990	Fracture of hand (wrist and other distal part of hand)	0.0138
Georgia	1995	Fracture of hand (wrist and other distal part of hand)	0.0138
Georgia	2000	Fracture of hand (wrist and other distal part of hand)	0.0138
Georgia	2005	Fracture of hand (wrist and other distal part of hand)	0.0138
Georgia	2010	Fracture of hand (wrist and other distal part of hand)	0.0138
Georgia	2017	Fracture of hand (wrist and other distal part of hand)	0.0138
Georgia	1990	Fracture of hip	0.1188
Georgia	1995	Fracture of hip	0.1115
Georgia	2000	Fracture of hip	0.1106
Georgia	2005	Fracture of hip	0.1088
Georgia	2010	Fracture of hip	0.1048
Georgia	2017	Fracture of hip	0.0904
Georgia	1990	Amputation of upper limbs, bilateral	0.1508
Georgia	1995	Amputation of upper limbs, bilateral	0.1432
Georgia	2000	Amputation of upper limbs, bilateral	0.1423
Georgia	2005	Amputation of upper limbs, bilateral	0.1404
Georgia	2010	Amputation of upper limbs, bilateral	0.1363
Georgia	2017	Amputation of upper limbs, bilateral	0.1214
Georgia	1990	Fracture of patella, tibia or fibula, or ankle	0.0554
Georgia	1995	Fracture of patella, tibia or fibula, or ankle	0.0554
Georgia	2000	Fracture of patella, tibia or fibula, or ankle	0.0554
Georgia	2005	Fracture of patella, tibia or fibula, or ankle	0.0554
Georgia	2010	Fracture of patella, tibia or fibula, or ankle	0.0554
Georgia	2017	Fracture of patella, tibia or fibula, or ankle	0.0554
Georgia	1990	Fracture of pelvis	0.1823
Georgia	1995	Fracture of pelvis	0.1823
Georgia	2000	Fracture of pelvis	0.1823
Georgia	2005	Fracture of pelvis	0.1823
Georgia	2010	Fracture of pelvis	0.1823
Georgia	2017	Fracture of pelvis	0.1823
Georgia	1990	Fracture of radius and/or ulna	0.0435
Georgia	1995	Fracture of radius and/or ulna	0.0435
Georgia	2000	Fracture of radius and/or ulna	0.0435
Georgia	2005	Fracture of radius and/or ulna	0.0435
Georgia	2010	Fracture of radius and/or ulna	0.0435
Georgia	2017	Fracture of radius and/or ulna	0.0435
Georgia	1990	Fracture of skull	0.0714
Georgia	1995	Fracture of skull	0.0714
Georgia	2000	Fracture of skull	0.0714
Georgia	2005	Fracture of skull	0.0714
Georgia	2010	Fracture of skull	0.0714
Georgia	2017	Fracture of skull	0.0714
Georgia	1990	Fracture of sternum and/or fracture of one or more ribs	0.1027
Georgia	1995	Fracture of sternum and/or fracture of one or more ribs	0.1027
Georgia	2000	Fracture of sternum and/or fracture of one or more ribs	0.1027

Georgia	2005	Fracture of sternum and/or fracture of one or more ribs	0.1027
Georgia	2010	Fracture of sternum and/or fracture of one or more ribs	0.1027
Georgia	2017	Fracture of sternum and/or fracture of one or more ribs	0.1027
Georgia	1990	Fracture of vertebral column	0.1106
Georgia	1995	Fracture of vertebral column	0.1106
Georgia	2000	Fracture of vertebral column	0.1106
Georgia	2005	Fracture of vertebral column	0.1106
Georgia	2010	Fracture of vertebral column	0.1106
Georgia	2017	Fracture of vertebral column	0.1106
Georgia	1990	Fracture of femur, other than femoral neck	0.042
Georgia	1995	Fracture of femur, other than femoral neck	0.042
Georgia	2000	Fracture of femur, other than femoral neck	0.042
Georgia	2005	Fracture of femur, other than femoral neck	0.042
Georgia	2010	Fracture of femur, other than femoral neck	0.042
Georgia	2017	Fracture of femur, other than femoral neck	0.042
Georgia	1990	Minor TBI	0.1319
Georgia	1995	Minor TBI	0.1319
Georgia	2000	Minor TBI	0.1319
Georgia	2005	Minor TBI	0.1319
Georgia	2010	Minor TBI	0.1319
Georgia	2017	Minor TBI	0.1319
Georgia	1990	Moderate TBI	0.1637
Georgia	1995	Moderate TBI	0.1637
Georgia	2000	Moderate TBI	0.1637
Georgia	2005	Moderate TBI	0.1637
Georgia	2010	Moderate TBI	0.1637
Georgia	2017	Moderate TBI	0.1637
Georgia	1990	Amputation of fingers (excluding thumb)	0.0047
Georgia	1995	Amputation of fingers (excluding thumb)	0.0047
Georgia	2000	Amputation of fingers (excluding thumb)	0.0047
Georgia	2005	Amputation of fingers (excluding thumb)	0.0047
Georgia	2010	Amputation of fingers (excluding thumb)	0.0047
Georgia	2017	Amputation of fingers (excluding thumb)	0.0047
Georgia	1990	Spinal cord lesion at neck level	0.433
Georgia	1995	Spinal cord lesion at neck level	0.4304
Georgia	2000	Spinal cord lesion at neck level	0.4301
Georgia	2005	Spinal cord lesion at neck level	0.4295
Georgia	2010	Spinal cord lesion at neck level	0.4281
Georgia	2017	Spinal cord lesion at neck level	0.4231
Georgia	1990	Spinal cord lesion below neck level	0.2223
Georgia	1995	Spinal cord lesion below neck level	0.2169
Georgia	2000	Spinal cord lesion below neck level	0.2163
Georgia	2005	Spinal cord lesion below neck level	0.215
Georgia	2010	Spinal cord lesion below neck level	0.2121
Georgia	2017	Spinal cord lesion below neck level	0.2015
Georgia	1990	Drowning and nonfatal submersion	0.2471
Georgia	1995	Drowning and nonfatal submersion	0.2471
Georgia	2000	Drowning and nonfatal submersion	0.2471
Georgia	2005	Drowning and nonfatal submersion	0.2471
Georgia	2010	Drowning and nonfatal submersion	0.2471
Georgia	2017	Drowning and nonfatal submersion	0.2471
Georgia	1990	Asphyxiation	0.2471
Georgia	1995	Asphyxiation	0.2471
Georgia	2000	Asphyxiation	0.2471
Georgia	2005	Asphyxiation	0.2471
Georgia	2010	Asphyxiation	0.2471
Georgia	2017	Asphyxiation	0.2471
Georgia	1990	Crush injury	0.1325
Georgia	1995	Crush injury	0.1325
Georgia	2000	Crush injury	0.1325
Georgia	2005	Crush injury	0.1325
Georgia	2010	Crush injury	0.1325
Georgia	2017	Crush injury	0.1325
Georgia	1990	Nerve injury	0.1129
Georgia	1995	Nerve injury	0.1129
Georgia	2000	Nerve injury	0.1129
Georgia	2005	Nerve injury	0.1129
Georgia	2010	Nerve injury	0.1129
Georgia	2017	Nerve injury	0.1129
Georgia	1990	Injury to eyes	0.0165
Georgia	1995	Injury to eyes	0.0165
Georgia	2000	Injury to eyes	0.0165
Georgia	2005	Injury to eyes	0.0165
Georgia	2010	Injury to eyes	0.0165
Georgia	2017	Injury to eyes	0.0165
Georgia	1990	Amputation of lower limb, unilateral	0.0628
Georgia	1995	Amputation of lower limb, unilateral	0.0599
Georgia	2000	Amputation of lower limb, unilateral	0.0596
Georgia	2005	Amputation of lower limb, unilateral	0.0589
Georgia	2010	Amputation of lower limb, unilateral	0.0573
Georgia	2017	Amputation of lower limb, unilateral	0.0517
Georgia	1990	Open wound(s)	0.011
Georgia	1995	Open wound(s)	0.011
Georgia	2000	Open wound(s)	0.011
Georgia	2005	Open wound(s)	0.011
Georgia	2010	Open wound(s)	0.011
Georgia	2017	Open wound(s)	0.011
Georgia	1990	Poisoning requiring urgent care	0.1628
Georgia	1995	Poisoning requiring urgent care	0.1628
Georgia	2000	Poisoning requiring urgent care	0.1628
Georgia	2005	Poisoning requiring urgent care	0.1628
Georgia	2010	Poisoning requiring urgent care	0.1628
Georgia	2017	Poisoning requiring urgent care	0.1628
Georgia	1990	Severe chest injury	0.0474
Georgia	1995	Severe chest injury	0.0474
Georgia	2000	Severe chest injury	0.0474
Georgia	2005	Severe chest injury	0.0474
Georgia	2010	Severe chest injury	0.0474
Georgia	2017	Severe chest injury	0.0474
Georgia	1990	Internal hemorrhage in abdomen and pelvis	0.1143
Georgia	1995	Internal hemorrhage in abdomen and pelvis	0.1143
Georgia	2000	Internal hemorrhage in abdomen and pelvis	0.1143
Georgia	2005	Internal hemorrhage in abdomen and pelvis	0.1143
Georgia	2010	Internal hemorrhage in abdomen and pelvis	0.1143
Georgia	2017	Internal hemorrhage in abdomen and pelvis	0.1143
Georgia	1990	Contusion in any part of the body	0.0075
Georgia	1995	Contusion in any part of the body	0.0075
Georgia	2000	Contusion in any part of the body	0.0075
Georgia	2005	Contusion in any part of the body	0.0075
Georgia	2010	Contusion in any part of the body	0.0075
Georgia	2017	Contusion in any part of the body	0.0075
Georgia	1990	Effect of different environmental factors	0.049
Georgia	1995	Effect of different environmental factors	0.049
Georgia	2000	Effect of different environmental factors	0.049
Georgia	2005	Effect of different environmental factors	0.049
Georgia	2010	Effect of different environmental factors	0.049
Georgia	2017	Effect of different environmental factors	0.049
Georgia	1990	Complications following therapeutic procedures	0.0506
Georgia	1995	Complications following therapeutic procedures	0.0506
Georgia	2000	Complications following therapeutic procedures	0.0506
Georgia	2005	Complications following therapeutic procedures	0.0506
Georgia	2010	Complications following therapeutic procedures	0.0506
Georgia	2017	Complications following therapeutic procedures	0.0506
Georgia	1990	Multiple fractures, dislocations, crashes, wounds, sprains, and strains	0.1188
Georgia	1995	Multiple fractures, dislocations, crashes, wounds, sprains, and strains	0.1115
Georgia	2000	Multiple fractures, dislocations, crashes, wounds, sprains, and strains	0.1106
Georgia	2005	Multiple fractures, dislocations, crashes, wounds, sprains, and strains	0.1088
Georgia	2010	Multiple fractures, dislocations, crashes, wounds, sprains, and strains	0.1048
Georgia	2017	Multiple fractures, dislocations, crashes, wounds, sprains, and strains	0.0904
Georgia	1990	Amputation of upper limb, unilateral	0.053
Georgia	1995	Amputation of upper limb, unilateral	0.0513
Georgia	2000	Amputation of upper limb, unilateral	0.0511
Georgia	2005	Amputation of upper limb, unilateral	0.0507
Georgia	2010	Amputation of upper limb, unilateral	0.0498
Georgia	2017	Amputation of upper limb, unilateral	0.0465
Georgia	1990	Amputation of thumb	0.0114
Georgia	1995	Amputation of thumb	0.0114
Georgia	2000	Amputation of thumb	0.0114
Georgia	2005	Amputation of thumb	0.0114
Georgia	2010	Amputation of thumb	0.0114
Georgia	2017	Amputation of thumb	0.0114
Georgia	1990	Amputation of toe/toes	0.0059
Georgia	1995	Amputation of toe/toes	0.0059
Georgia	2000	Amputation of toe/toes	0.0059
Georgia	2005	Amputation of toe/toes	0.0059
Georgia	2010	Amputation of toe/toes	0.0059
Georgia	2017	Amputation of toe/toes	0.0059
Georgia	1990	Burns, <20% total burned surface area without lower airway burns	0.0163
Georgia	1995	Burns, <20% total burned surface area without lower airway burns	0.0163
Georgia	2000	Burns, <20% total burned surface area without lower airway burns	0.0163
Georgia	2005	Burns, <20% total burned surface area without lower airway burns	0.0163
Georgia	2010	Burns, <20% total burned surface area without lower airway burns	0.0163
Georgia	2017	Burns, <20% total burned surface area without lower airway burns	0.0163
Georgia	1990	Burns, >=20% total burned surface area or >= 10% burned surface area if head/neck or hands/wrist involved w/o lower airway burns	0.1915
Georgia	1995	Burns, >=20% total burned surface area or >= 10% burned surface area if head/neck or hands/wrist involved w/o lower airway burns	0.1847
Georgia	2000	Burns, >=20% total burned surface area or >= 10% burned surface area if head/neck or hands/wrist involved w/o lower airway burns	0.1839
Georgia	2005	Burns, >=20% total burned surface area or >= 10% burned surface area if head/neck or hands/wrist involved w/o lower airway burns	0.1822
Georgia	2010	Burns, >=20% total burned surface area or >= 10% burned surface area if head/neck or hands/wrist involved w/o lower airway burns	0.1785
Georgia	2017	Burns, >=20% total burned surface area or >= 10% burned surface area if head/neck or hands/wrist involved w/o lower airway burns	0.165
Kazakhstan	1990	Amputation of lower limbs, bilateral	0.1895
Kazakhstan	1995	Amputation of lower limbs, bilateral	0.1974
Kazakhstan	2000	Amputation of lower limbs, bilateral	0.194
Kazakhstan	2005	Amputation of lower limbs, bilateral	0.1854
Kazakhstan	2010	Amputation of lower limbs, bilateral	0.1666
Kazakhstan	2017	Amputation of lower limbs, bilateral	0.1414
Kazakhstan	1990	Lower airway burns	0.2252
Kazakhstan	1995	Lower airway burns	0.2252
Kazakhstan	2000	Lower airway burns	0.2252
Kazakhstan	2005	Lower airway burns	0.2252
Kazakhstan	2010	Lower airway burns	0.2252
Kazakhstan	2017	Lower airway burns	0.2252
Kazakhstan	1990	Dislocation of hip	0.0159
Kazakhstan	1995	Dislocation of hip	0.0159
Kazakhstan	2000	Dislocation of hip	0.0159
Kazakhstan	2005	Dislocation of hip	0.0159
Kazakhstan	2010	Dislocation of hip	0.0159
Kazakhstan	2017	Dislocation of hip	0.0159
Kazakhstan	1990	Dislocation of knee	0.1134
Kazakhstan	1995	Dislocation of knee	0.1134
Kazakhstan	2000	Dislocation of knee	0.1134
Kazakhstan	2005	Dislocation of knee	0.1134
Kazakhstan	2010	Dislocation of knee	0.1134
Kazakhstan	2017	Dislocation of knee	0.1134
Kazakhstan	1990	Dislocation of shoulder	0.062
Kazakhstan	1995	Dislocation of shoulder	0.062
Kazakhstan	2000	Dislocation of shoulder	0.062
Kazakhstan	2005	Dislocation of shoulder	0.062
Kazakhstan	2010	Dislocation of shoulder	0.062
Kazakhstan	2017	Dislocation of shoulder	0.062
Kazakhstan	1990	Muscle and tendon injuries, including sprains and strains lesser dislocations	0.0075
Kazakhstan	1995	Muscle and tendon injuries, including sprains and strains lesser dislocations	0.0075
Kazakhstan	2000	Muscle and tendon injuries, including sprains and strains lesser dislocations	0.0075
Kazakhstan	2005	Muscle and tendon injuries, including sprains and strains lesser dislocations	0.0075
Kazakhstan	2010	Muscle and tendon injuries, including sprains and strains lesser dislocations	0.0075
Kazakhstan	2017	Muscle and tendon injuries, including sprains and strains lesser dislocations	0.0075
Kazakhstan	1990	Fracture of clavicle, scapula, or humerus	0.0349
Kazakhstan	1995	Fracture of clavicle, scapula, or humerus	0.0349
Kazakhstan	2000	Fracture of clavicle, scapula, or humerus	0.0349
Kazakhstan	2005	Fracture of clavicle, scapula, or humerus	0.0349
Kazakhstan	2010	Fracture of clavicle, scapula, or humerus	0.0349
Kazakhstan	2017	Fracture of clavicle, scapula, or humerus	0.0349
Kazakhstan	1990	Fracture of face bones	0.0669
Kazakhstan	1995	Fracture of face bones	0.0669
Kazakhstan	2000	Fracture of face bones	0.0669
Kazakhstan	2005	Fracture of face bones	0.0669
Kazakhstan	2010	Fracture of face bones	0.0669
Kazakhstan	2017	Fracture of face bones	0.0669
Kazakhstan	1990	Fracture of foot bones except ankle	0.0264
Kazakhstan	1995	Fracture of foot bones except ankle	0.0264
Kazakhstan	2000	Fracture of foot bones except ankle	0.0264
Kazakhstan	2005	Fracture of foot bones except ankle	0.0264
Kazakhstan	2010	Fracture of foot bones except ankle	0.0264
Kazakhstan	2017	Fracture of foot bones except ankle	0.0264
Kazakhstan	1990	Fracture of hand (wrist and other distal part of hand)	0.0138
Kazakhstan	1995	Fracture of hand (wrist and other distal part of hand)	0.0138
Kazakhstan	2000	Fracture of hand (wrist and other distal part of hand)	0.0138
Kazakhstan	2005	Fracture of hand (wrist and other distal part of hand)	0.0138
Kazakhstan	2010	Fracture of hand (wrist and other distal part of hand)	0.0138
Kazakhstan	2017	Fracture of hand (wrist and other distal part of hand)	0.0138
Kazakhstan	1990	Fracture of hip	0.1454
Kazakhstan	1995	Fracture of hip	0.1558
Kazakhstan	2000	Fracture of hip	0.1512
Kazakhstan	2005	Fracture of hip	0.1399
Kazakhstan	2010	Fracture of hip	0.115
Kazakhstan	2017	Fracture of hip	0.0817
Kazakhstan	1990	Amputation of upper limbs, bilateral	0.1782
Kazakhstan	1995	Amputation of upper limbs, bilateral	0.1889
Kazakhstan	2000	Amputation of upper limbs, bilateral	0.1842
Kazakhstan	2005	Amputation of upper limbs, bilateral	0.1726
Kazakhstan	2010	Amputation of upper limbs, bilateral	0.1468
Kazakhstan	2017	Amputation of upper limbs, bilateral	0.1124
Kazakhstan	1990	Fracture of patella, tibia or fibula, or ankle	0.0554
Kazakhstan	1995	Fracture of patella, tibia or fibula, or ankle	0.0554
Kazakhstan	2000	Fracture of patella, tibia or fibula, or ankle	0.0554
Kazakhstan	2005	Fracture of patella, tibia or fibula, or ankle	0.0554
Kazakhstan	2010	Fracture of patella, tibia or fibula, or ankle	0.0554
Kazakhstan	2017	Fracture of patella, tibia or fibula, or ankle	0.0554
Kazakhstan	1990	Fracture of pelvis	0.1823
Kazakhstan	1995	Fracture of pelvis	0.1823
Kazakhstan	2000	Fracture of pelvis	0.1823
Kazakhstan	2005	Fracture of pelvis	0.1823
Kazakhstan	2010	Fracture of pelvis	0.1823
Kazakhstan	2017	Fracture of pelvis	0.1823
Kazakhstan	1990	Fracture of radius and/or ulna	0.0435
Kazakhstan	1995	Fracture of radius and/or ulna	0.0435
Kazakhstan	2000	Fracture of radius and/or ulna	0.0435
Kazakhstan	2005	Fracture of radius and/or ulna	0.0435
Kazakhstan	2010	Fracture of radius and/or ulna	0.0435
Kazakhstan	2017	Fracture of radius and/or ulna	0.0435
Kazakhstan	1990	Fracture of skull	0.0714
Kazakhstan	1995	Fracture of skull	0.0714
Kazakhstan	2000	Fracture of skull	0.0714
Kazakhstan	2005	Fracture of skull	0.0714
Kazakhstan	2010	Fracture of skull	0.0714
Kazakhstan	2017	Fracture of skull	0.0714
Kazakhstan	1990	Fracture of sternum and/or fracture of one or more ribs	0.1027
Kazakhstan	1995	Fracture of sternum and/or fracture of one or more ribs	0.1027
Kazakhstan	2000	Fracture of sternum and/or fracture of one or more ribs	0.1027
Kazakhstan	2005	Fracture of sternum and/or fracture of one or more ribs	0.1027
Kazakhstan	2010	Fracture of sternum and/or fracture of one or more ribs	0.1027
Kazakhstan	2017	Fracture of sternum and/or fracture of one or more ribs	0.1027
Kazakhstan	1990	Fracture of vertebral column	0.1106
Kazakhstan	1995	Fracture of vertebral column	0.1106
Kazakhstan	2000	Fracture of vertebral column	0.1106
Kazakhstan	2005	Fracture of vertebral column	0.1106
Kazakhstan	2010	Fracture of vertebral column	0.1106
Kazakhstan	2017	Fracture of vertebral column	0.1106
Kazakhstan	1990	Fracture of femur, other than femoral neck	0.042
Kazakhstan	1995	Fracture of femur, other than femoral neck	0.042
Kazakhstan	2000	Fracture of femur, other than femoral neck	0.042
Kazakhstan	2005	Fracture of femur, other than femoral neck	0.042
Kazakhstan	2010	Fracture of femur, other than femoral neck	0.042
Kazakhstan	2017	Fracture of femur, other than femoral neck	0.042
Kazakhstan	1990	Minor TBI	0.1319
Kazakhstan	1995	Minor TBI	0.1319
Kazakhstan	2000	Minor TBI	0.1319
Kazakhstan	2005	Minor TBI	0.1319
Kazakhstan	2010	Minor TBI	0.1319
Kazakhstan	2017	Minor TBI	0.1319
Kazakhstan	1990	Moderate TBI	0.1637
Kazakhstan	1995	Moderate TBI	0.1637
Kazakhstan	2000	Moderate TBI	0.1637
Kazakhstan	2005	Moderate TBI	0.1637
Kazakhstan	2010	Moderate TBI	0.1637
Kazakhstan	2017	Moderate TBI	0.1637
Kazakhstan	1990	Amputation of fingers (excluding thumb)	0.0047
Kazakhstan	1995	Amputation of fingers (excluding thumb)	0.0047
Kazakhstan	2000	Amputation of fingers (excluding thumb)	0.0047
Kazakhstan	2005	Amputation of fingers (excluding thumb)	0.0047
Kazakhstan	2010	Amputation of fingers (excluding thumb)	0.0047
Kazakhstan	2017	Amputation of fingers (excluding thumb)	0.0047
Kazakhstan	1990	Spinal cord lesion at neck level	0.4421
Kazakhstan	1995	Spinal cord lesion at neck level	0.4457
Kazakhstan	2000	Spinal cord lesion at neck level	0.4442
Kazakhstan	2005	Spinal cord lesion at neck level	0.4403
Kazakhstan	2010	Spinal cord lesion at neck level	0.4316
Kazakhstan	2017	Spinal cord lesion at neck level	0.4201
Kazakhstan	1990	Spinal cord lesion below neck level	0.2417
Kazakhstan	1995	Spinal cord lesion below neck level	0.2492
Kazakhstan	2000	Spinal cord lesion below neck level	0.2459
Kazakhstan	2005	Spinal cord lesion below neck level	0.2377
Kazakhstan	2010	Spinal cord lesion below neck level	0.2195
Kazakhstan	2017	Spinal cord lesion below neck level	0.1952
Kazakhstan	1990	Drowning and nonfatal submersion	0.2471
Kazakhstan	1995	Drowning and nonfatal submersion	0.2471
Kazakhstan	2000	Drowning and nonfatal submersion	0.2471
Kazakhstan	2005	Drowning and nonfatal submersion	0.2471
Kazakhstan	2010	Drowning and nonfatal submersion	0.2471
Kazakhstan	2017	Drowning and nonfatal submersion	0.2471
Kazakhstan	1990	Asphyxiation	0.2471
Kazakhstan	1995	Asphyxiation	0.2471
Kazakhstan	2000	Asphyxiation	0.2471
Kazakhstan	2005	Asphyxiation	0.2471
Kazakhstan	2010	Asphyxiation	0.2471
Kazakhstan	2017	Asphyxiation	0.2471
Kazakhstan	1990	Crush injury	0.1325
Kazakhstan	1995	Crush injury	0.1325
Kazakhstan	2000	Crush injury	0.1325
Kazakhstan	2005	Crush injury	0.1325
Kazakhstan	2010	Crush injury	0.1325
Kazakhstan	2017	Crush injury	0.1325
Kazakhstan	1990	Nerve injury	0.1129
Kazakhstan	1995	Nerve injury	0.1129
Kazakhstan	2000	Nerve injury	0.1129
Kazakhstan	2005	Nerve injury	0.1129
Kazakhstan	2010	Nerve injury	0.1129
Kazakhstan	2017	Nerve injury	0.1129
Kazakhstan	1990	Injury to eyes	0.0165
Kazakhstan	1995	Injury to eyes	0.0165
Kazakhstan	2000	Injury to eyes	0.0165
Kazakhstan	2005	Injury to eyes	0.0165
Kazakhstan	2010	Injury to eyes	0.0165
Kazakhstan	2017	Injury to eyes	0.0165
Kazakhstan	1990	Amputation of lower limb, unilateral	0.0731
Kazakhstan	1995	Amputation of lower limb, unilateral	0.0772
Kazakhstan	2000	Amputation of lower limb, unilateral	0.0754
Kazakhstan	2005	Amputation of lower limb, unilateral	0.071
Kazakhstan	2010	Amputation of lower limb, unilateral	0.0613
Kazakhstan	2017	Amputation of lower limb, unilateral	0.0483
Kazakhstan	1990	Open wound(s)	0.011
Kazakhstan	1995	Open wound(s)	0.011
Kazakhstan	2000	Open wound(s)	0.011
Kazakhstan	2005	Open wound(s)	0.011
Kazakhstan	2010	Open wound(s)	0.011
Kazakhstan	2017	Open wound(s)	0.011
Kazakhstan	1990	Poisoning requiring urgent care	0.1628
Kazakhstan	1995	Poisoning requiring urgent care	0.1628
Kazakhstan	2000	Poisoning requiring urgent care	0.1628
Kazakhstan	2005	Poisoning requiring urgent care	0.1628
Kazakhstan	2010	Poisoning requiring urgent care	0.1628
Kazakhstan	2017	Poisoning requiring urgent care	0.1628
Kazakhstan	1990	Severe chest injury	0.0474
Kazakhstan	1995	Severe chest injury	0.0474
Kazakhstan	2000	Severe chest injury	0.0474
Kazakhstan	2005	Severe chest injury	0.0474
Kazakhstan	2010	Severe chest injury	0.0474
Kazakhstan	2017	Severe chest injury	0.0474
Kazakhstan	1990	Internal hemorrhage in abdomen and pelvis	0.1143
Kazakhstan	1995	Internal hemorrhage in abdomen and pelvis	0.1143
Kazakhstan	2000	Internal hemorrhage in abdomen and pelvis	0.1143
Kazakhstan	2005	Internal hemorrhage in abdomen and pelvis	0.1143
Kazakhstan	2010	Internal hemorrhage in abdomen and pelvis	0.1143
Kazakhstan	2017	Internal hemorrhage in abdomen and pelvis	0.1143
Kazakhstan	1990	Contusion in any part of the body	0.0075
Kazakhstan	1995	Contusion in any part of the body	0.0075
Kazakhstan	2000	Contusion in any part of the body	0.0075
Kazakhstan	2005	Contusion in any part of the body	0.0075
Kazakhstan	2010	Contusion in any part of the body	0.0075
Kazakhstan	2017	Contusion in any part of the body	0.0075
Kazakhstan	1990	Effect of different environmental factors	0.049
Kazakhstan	1995	Effect of different environmental factors	0.049
Kazakhstan	2000	Effect of different environmental factors	0.049
Kazakhstan	2005	Effect of different environmental factors	0.049
Kazakhstan	2010	Effect of different environmental factors	0.049
Kazakhstan	2017	Effect of different environmental factors	0.049
Kazakhstan	1990	Complications following therapeutic procedures	0.0506
Kazakhstan	1995	Complications following therapeutic procedures	0.0506
Kazakhstan	2000	Complications following therapeutic procedures	0.0506
Kazakhstan	2005	Complications following therapeutic procedures	0.0506
Kazakhstan	2010	Complications following therapeutic procedures	0.0506
Kazakhstan	2017	Complications following therapeutic procedures	0.0506
Kazakhstan	1990	Multiple fractures, dislocations, crashes, wounds, sprains, and strains	0.1454
Kazakhstan	1995	Multiple fractures, dislocations, crashes, wounds, sprains, and strains	0.1558
Kazakhstan	2000	Multiple fractures, dislocations, crashes, wounds, sprains, and strains	0.1512
Kazakhstan	2005	Multiple fractures, dislocations, crashes, wounds, sprains, and strains	0.1399
Kazakhstan	2010	Multiple fractures, dislocations, crashes, wounds, sprains, and strains	0.115
Kazakhstan	2017	Multiple fractures, dislocations, crashes, wounds, sprains, and strains	0.0817
Kazakhstan	1990	Amputation of upper limb, unilateral	0.0591
Kazakhstan	1995	Amputation of upper limb, unilateral	0.0615
Kazakhstan	2000	Amputation of upper limb, unilateral	0.0604
Kazakhstan	2005	Amputation of upper limb, unilateral	0.0579
Kazakhstan	2010	Amputation of upper limb, unilateral	0.0521
Kazakhstan	2017	Amputation of upper limb, unilateral	0.0445
Kazakhstan	1990	Amputation of thumb	0.0114
Kazakhstan	1995	Amputation of thumb	0.0114
Kazakhstan	2000	Amputation of thumb	0.0114
Kazakhstan	2005	Amputation of thumb	0.0114
Kazakhstan	2010	Amputation of thumb	0.0114
Kazakhstan	2017	Amputation of thumb	0.0114
Kazakhstan	1990	Amputation of toe/toes	0.0059
Kazakhstan	1995	Amputation of toe/toes	0.0059
Kazakhstan	2000	Amputation of toe/toes	0.0059
Kazakhstan	2005	Amputation of toe/toes	0.0059
Kazakhstan	2010	Amputation of toe/toes	0.0059
Kazakhstan	2017	Amputation of toe/toes	0.0059
Kazakhstan	1990	Burns, <20% total burned surface area without lower airway burns	0.0163
Kazakhstan	1995	Burns, <20% total burned surface area without lower airway burns	0.0163
Kazakhstan	2000	Burns, <20% total burned surface area without lower airway burns	0.0163
Kazakhstan	2005	Burns, <20% total burned surface area without lower airway burns	0.0163
Kazakhstan	2010	Burns, <20% total burned surface area without lower airway burns	0.0163
Kazakhstan	2017	Burns, <20% total burned surface area without lower airway burns	0.0163
Kazakhstan	1990	Burns, >=20% total burned surface area or >= 10% burned surface area if head/neck or hands/wrist involved w/o lower airway burns	0.2162
Kazakhstan	1995	Burns, >=20% total burned surface area or >= 10% burned surface area if head/neck or hands/wrist involved w/o lower airway burns	0.2259
Kazakhstan	2000	Burns, >=20% total burned surface area or >= 10% burned surface area if head/neck or hands/wrist involved w/o lower airway burns	0.2217
Kazakhstan	2005	Burns, >=20% total burned surface area or >= 10% burned surface area if head/neck or hands/wrist involved w/o lower airway burns	0.2112
Kazakhstan	2010	Burns, >=20% total burned surface area or >= 10% burned surface area if head/neck or hands/wrist involved w/o lower airway burns	0.188
Kazakhstan	2017	Burns, >=20% total burned surface area or >= 10% burned surface area if head/neck or hands/wrist involved w/o lower airway burns	0.1569
Kyrgyzstan	1990	Amputation of lower limbs, bilateral	0.205
Kyrgyzstan	1995	Amputation of lower limbs, bilateral	0.2068
Kyrgyzstan	2000	Amputation of lower limbs, bilateral	0.1995
Kyrgyzstan	2005	Amputation of lower limbs, bilateral	0.1925
Kyrgyzstan	2010	Amputation of lower limbs, bilateral	0.1838
Kyrgyzstan	2017	Amputation of lower limbs, bilateral	0.1704
Kyrgyzstan	1990	Lower airway burns	0.2252
Kyrgyzstan	1995	Lower airway burns	0.2252
Kyrgyzstan	2000	Lower airway burns	0.2252
Kyrgyzstan	2005	Lower airway burns	0.2252
Kyrgyzstan	2010	Lower airway burns	0.2252
Kyrgyzstan	2017	Lower airway burns	0.2252
Kyrgyzstan	1990	Dislocation of hip	0.0159
Kyrgyzstan	1995	Dislocation of hip	0.0159

Kyrgyzstan	2000	Dislocation of hip	0.0159
Kyrgyzstan	2005	Dislocation of hip	0.0159
Kyrgyzstan	2010	Dislocation of hip	0.0159
Kyrgyzstan	2017	Dislocation of hip	0.0159
Kyrgyzstan	1990	Dislocation of knee	0.1134
Kyrgyzstan	1995	Dislocation of knee	0.1134
Kyrgyzstan	2000	Dislocation of knee	0.1134
Kyrgyzstan	2005	Dislocation of knee	0.1134
Kyrgyzstan	2010	Dislocation of knee	0.1134
Kyrgyzstan	2017	Dislocation of knee	0.1134
Kyrgyzstan	1990	Dislocation of shoulder	0.062
Kyrgyzstan	1995	Dislocation of shoulder	0.062
Kyrgyzstan	2000	Dislocation of shoulder	0.062
Kyrgyzstan	2005	Dislocation of shoulder	0.062
Kyrgyzstan	2010	Dislocation of shoulder	0.062
Kyrgyzstan	2017	Dislocation of shoulder	0.062
Kyrgyzstan	1990	Muscle and tendon injuries, including sprains and strains lesser dislocations	0.0075
Kyrgyzstan	1995	Muscle and tendon injuries, including sprains and strains lesser dislocations	0.0075
Kyrgyzstan	2000	Muscle and tendon injuries, including sprains and strains lesser dislocations	0.0075
Kyrgyzstan	2005	Muscle and tendon injuries, including sprains and strains lesser dislocations	0.0075
Kyrgyzstan	2010	Muscle and tendon injuries, including sprains and strains lesser dislocations	0.0075
Kyrgyzstan	2017	Muscle and tendon injuries, including sprains and strains lesser dislocations	0.0075
Kyrgyzstan	1990	Fracture of clavicle, scapula, or humerus	0.0349
Kyrgyzstan	1995	Fracture of clavicle, scapula, or humerus	0.0349
Kyrgyzstan	2000	Fracture of clavicle, scapula, or humerus	0.0349
Kyrgyzstan	2005	Fracture of clavicle, scapula, or humerus	0.0349
Kyrgyzstan	2010	Fracture of clavicle, scapula, or humerus	0.0349
Kyrgyzstan	2017	Fracture of clavicle, scapula, or humerus	0.0349
Kyrgyzstan	1990	Fracture of face bones	0.0669
Kyrgyzstan	1995	Fracture of face bones	0.0669
Kyrgyzstan	2000	Fracture of face bones	0.0669
Kyrgyzstan	2005	Fracture of face bones	0.0669
Kyrgyzstan	2010	Fracture of face bones	0.0669
Kyrgyzstan	2017	Fracture of face bones	0.0669
Kyrgyzstan	1990	Fracture of foot bones except ankle	0.0264
Kyrgyzstan	1995	Fracture of foot bones except ankle	0.0264
Kyrgyzstan	2000	Fracture of foot bones except ankle	0.0264
Kyrgyzstan	2005	Fracture of foot bones except ankle	0.0264
Kyrgyzstan	2010	Fracture of foot bones except ankle	0.0264
Kyrgyzstan	2017	Fracture of foot bones except ankle	0.0264
Kyrgyzstan	1990	Fracture of hand (wrist and other distal part of hand)	0.0138
Kyrgyzstan	1995	Fracture of hand (wrist and other distal part of hand)	0.0138
Kyrgyzstan	2000	Fracture of hand (wrist and other distal part of hand)	0.0138
Kyrgyzstan	2005	Fracture of hand (wrist and other distal part of hand)	0.0138
Kyrgyzstan	2010	Fracture of hand (wrist and other distal part of hand)	0.0138
Kyrgyzstan	2017	Fracture of hand (wrist and other distal part of hand)	0.0138
Kyrgyzstan	1990	Fracture of hip	0.1658
Kyrgyzstan	1995	Fracture of hip	0.1682
Kyrgyzstan	2000	Fracture of hip	0.1585
Kyrgyzstan	2005	Fracture of hip	0.1493
Kyrgyzstan	2010	Fracture of hip	0.1377
Kyrgyzstan	2017	Fracture of hip	0.1201
Kyrgyzstan	1990	Amputation of upper limbs, bilateral	0.1993
Kyrgyzstan	1995	Amputation of upper limbs, bilateral	0.2017
Kyrgyzstan	2000	Amputation of upper limbs, bilateral	0.1918
Kyrgyzstan	2005	Amputation of upper limbs, bilateral	0.1822
Kyrgyzstan	2010	Amputation of upper limbs, bilateral	0.1703
Kyrgyzstan	2017	Amputation of upper limbs, bilateral	0.1521
Kyrgyzstan	1990	Fracture of patella, tibia or fibula, or ankle	0.0554
Kyrgyzstan	1995	Fracture of patella, tibia or fibula, or ankle	0.0554
Kyrgyzstan	2000	Fracture of patella, tibia or fibula, or ankle	0.0554
Kyrgyzstan	2005	Fracture of patella, tibia or fibula, or ankle	0.0554
Kyrgyzstan	2010	Fracture of patella, tibia or fibula, or ankle	0.0554
Kyrgyzstan	2017	Fracture of patella, tibia or fibula, or ankle	0.0554
Kyrgyzstan	1990	Fracture of pelvis	0.1823
Kyrgyzstan	1995	Fracture of pelvis	0.1823
Kyrgyzstan	2000	Fracture of pelvis	0.1823
Kyrgyzstan	2005	Fracture of pelvis	0.1823
Kyrgyzstan	2010	Fracture of pelvis	0.1823
Kyrgyzstan	2017	Fracture of pelvis	0.1823
Kyrgyzstan	1990	Fracture of radius and/or ulna	0.0435
Kyrgyzstan	1995	Fracture of radius and/or ulna	0.0435
Kyrgyzstan	2000	Fracture of radius and/or ulna	0.0435
Kyrgyzstan	2005	Fracture of radius and/or ulna	0.0435
Kyrgyzstan	2010	Fracture of radius and/or ulna	0.0435
Kyrgyzstan	2017	Fracture of radius and/or ulna	0.0435
Kyrgyzstan	1990	Fracture of skull	0.0714
Kyrgyzstan	1995	Fracture of skull	0.0714
Kyrgyzstan	2000	Fracture of skull	0.0714
Kyrgyzstan	2005	Fracture of skull	0.0714
Kyrgyzstan	2010	Fracture of skull	0.0714
Kyrgyzstan	2017	Fracture of skull	0.0714
Kyrgyzstan	1990	Fracture of sternum and/or fracture of one or more ribs	0.1027
Kyrgyzstan	1995	Fracture of sternum and/or fracture of one or more ribs	0.1027
Kyrgyzstan	2000	Fracture of sternum and/or fracture of one or more ribs	0.1027
Kyrgyzstan	2005	Fracture of sternum and/or fracture of one or more ribs	0.1027
Kyrgyzstan	2010	Fracture of sternum and/or fracture of one or more ribs	0.1027
Kyrgyzstan	2017	Fracture of sternum and/or fracture of one or more ribs	0.1027
Kyrgyzstan	1990	Fracture of vertebral column	0.1106
Kyrgyzstan	1995	Fracture of vertebral column	0.1106
Kyrgyzstan	2000	Fracture of vertebral column	0.1106
Kyrgyzstan	2005	Fracture of vertebral column	0.1106
Kyrgyzstan	2010	Fracture of vertebral column	0.1106
Kyrgyzstan	2017	Fracture of vertebral column	0.1106
Kyrgyzstan	1990	Fracture of femur, other than femoral neck	0.042
Kyrgyzstan	1995	Fracture of femur, other than femoral neck	0.042
Kyrgyzstan	2000	Fracture of femur, other than femoral neck	0.042
Kyrgyzstan	2005	Fracture of femur, other than femoral neck	0.042
Kyrgyzstan	2010	Fracture of femur, other than femoral neck	0.042
Kyrgyzstan	2017	Fracture of femur, other than femoral neck	0.042
Kyrgyzstan	1990	Minor TBI	0.1319
Kyrgyzstan	1995	Minor TBI	0.1319
Kyrgyzstan	2000	Minor TBI	0.1319
Kyrgyzstan	2005	Minor TBI	0.1319
Kyrgyzstan	2010	Minor TBI	0.1319
Kyrgyzstan	2017	Minor TBI	0.1319
Kyrgyzstan	1990	Moderate TBI	0.1637
Kyrgyzstan	1995	Moderate TBI	0.1637
Kyrgyzstan	2000	Moderate TBI	0.1637
Kyrgyzstan	2005	Moderate TBI	0.1637
Kyrgyzstan	2010	Moderate TBI	0.1637
Kyrgyzstan	2017	Moderate TBI	0.1637
Kyrgyzstan	1990	Amputation of fingers (excluding thumb)	0.0047
Kyrgyzstan	1995	Amputation of fingers (excluding thumb)	0.0047
Kyrgyzstan	2000	Amputation of fingers (excluding thumb)	0.0047
Kyrgyzstan	2005	Amputation of fingers (excluding thumb)	0.0047
Kyrgyzstan	2010	Amputation of fingers (excluding thumb)	0.0047
Kyrgyzstan	2017	Amputation of fingers (excluding thumb)	0.0047
Kyrgyzstan	1990	Spinal cord lesion at neck level	0.4492
Kyrgyzstan	1995	Spinal cord lesion at neck level	0.45
Kyrgyzstan	2000	Spinal cord lesion at neck level	0.4467
Kyrgyzstan	2005	Spinal cord lesion at neck level	0.4435
Kyrgyzstan	2010	Spinal cord lesion at neck level	0.4395
Kyrgyzstan	2017	Spinal cord lesion at neck level	0.4334
Kyrgyzstan	1990	Spinal cord lesion below neck level	0.2566
Kyrgyzstan	1995	Spinal cord lesion below neck level	0.2583
Kyrgyzstan	2000	Spinal cord lesion below neck level	0.2513
Kyrgyzstan	2005	Spinal cord lesion below neck level	0.2445
Kyrgyzstan	2010	Spinal cord lesion below neck level	0.2361
Kyrgyzstan	2017	Spinal cord lesion below neck level	0.2232
Kyrgyzstan	1990	Drowning and nonfatal submersion	0.2471
Kyrgyzstan	1995	Drowning and nonfatal submersion	0.2471
Kyrgyzstan	2000	Drowning and nonfatal submersion	0.2471
Kyrgyzstan	2005	Drowning and nonfatal submersion	0.2471
Kyrgyzstan	2010	Drowning and nonfatal submersion	0.2471
Kyrgyzstan	2017	Drowning and nonfatal submersion	0.2471
Kyrgyzstan	1990	Asphyxiation	0.2471
Kyrgyzstan	1995	Asphyxiation	0.2471
Kyrgyzstan	2000	Asphyxiation	0.2471
Kyrgyzstan	2005	Asphyxiation	0.2471
Kyrgyzstan	2010	Asphyxiation	0.2471
Kyrgyzstan	2017	Asphyxiation	0.2471
Kyrgyzstan	1990	Crush injury	0.1325
Kyrgyzstan	1995	Crush injury	0.1325
Kyrgyzstan	2000	Crush injury	0.1325
Kyrgyzstan	2005	Crush injury	0.1325
Kyrgyzstan	2010	Crush injury	0.1325
Kyrgyzstan	2017	Crush injury	0.1325
Kyrgyzstan	1990	Nerve injury	0.1129
Kyrgyzstan	1995	Nerve injury	0.1129
Kyrgyzstan	2000	Nerve injury	0.1129
Kyrgyzstan	2005	Nerve injury	0.1129
Kyrgyzstan	2010	Nerve injury	0.1129
Kyrgyzstan	2017	Nerve injury	0.1129
Kyrgyzstan	1990	Injury to eyes	0.0165
Kyrgyzstan	1995	Injury to eyes	0.0165
Kyrgyzstan	2000	Injury to eyes	0.0165
Kyrgyzstan	2005	Injury to eyes	0.0165
Kyrgyzstan	2010	Injury to eyes	0.0165
Kyrgyzstan	2017	Injury to eyes	0.0165
Kyrgyzstan	1990	Amputation of lower limb, unilateral	0.0811
Kyrgyzstan	1995	Amputation of lower limb, unilateral	0.082
Kyrgyzstan	2000	Amputation of lower limb, unilateral	0.0783
Kyrgyzstan	2005	Amputation of lower limb, unilateral	0.0746
Kyrgyzstan	2010	Amputation of lower limb, unilateral	0.0702
Kyrgyzstan	2017	Amputation of lower limb, unilateral	0.0633
Kyrgyzstan	1990	Open wound(s)	0.011
Kyrgyzstan	1995	Open wound(s)	0.011
Kyrgyzstan	2000	Open wound(s)	0.011
Kyrgyzstan	2005	Open wound(s)	0.011
Kyrgyzstan	2010	Open wound(s)	0.011
Kyrgyzstan	2017	Open wound(s)	0.011
Kyrgyzstan	1990	Poisoning requiring urgent care	0.1628
Kyrgyzstan	1995	Poisoning requiring urgent care	0.1628
Kyrgyzstan	2000	Poisoning requiring urgent care	0.1628
Kyrgyzstan	2005	Poisoning requiring urgent care	0.1628
Kyrgyzstan	2010	Poisoning requiring urgent care	0.1628
Kyrgyzstan	2017	Poisoning requiring urgent care	0.1628
Kyrgyzstan	1990	Severe chest injury	0.0474
Kyrgyzstan	1995	Severe chest injury	0.0474
Kyrgyzstan	2000	Severe chest injury	0.0474
Kyrgyzstan	2005	Severe chest injury	0.0474
Kyrgyzstan	2010	Severe chest injury	0.0474
Kyrgyzstan	2017	Severe chest injury	0.0474
Kyrgyzstan	1990	Internal hemorrhage in abdomen and pelvis	0.1143
Kyrgyzstan	1995	Internal hemorrhage in abdomen and pelvis	0.1143
Kyrgyzstan	2000	Internal hemorrhage in abdomen and pelvis	0.1143
Kyrgyzstan	2005	Internal hemorrhage in abdomen and pelvis	0.1143
Kyrgyzstan	2010	Internal hemorrhage in abdomen and pelvis	0.1143
Kyrgyzstan	2017	Internal hemorrhage in abdomen and pelvis	0.1143
Kyrgyzstan	1990	Contusion in any part of the body	0.0075
Kyrgyzstan	1995	Contusion in any part of the body	0.0075
Kyrgyzstan	2000	Contusion in any part of the body	0.0075
Kyrgyzstan	2005	Contusion in any part of the body	0.0075
Kyrgyzstan	2010	Contusion in any part of the body	0.0075
Kyrgyzstan	2017	Contusion in any part of the body	0.0075
Kyrgyzstan	1990	Effect of different environmental factors	0.049
Kyrgyzstan	1995	Effect of different environmental factors	0.049
Kyrgyzstan	2000	Effect of different environmental factors	0.049
Kyrgyzstan	2005	Effect of different environmental factors	0.049
Kyrgyzstan	2010	Effect of different environmental factors	0.049
Kyrgyzstan	2017	Effect of different environmental factors	0.049
Kyrgyzstan	1990	Complications following therapeutic procedures	0.0506
Kyrgyzstan	1995	Complications following therapeutic procedures	0.0506
Kyrgyzstan	2000	Complications following therapeutic procedures	0.0506
Kyrgyzstan	2005	Complications following therapeutic procedures	0.0506
Kyrgyzstan	2010	Complications following therapeutic procedures	0.0506
Kyrgyzstan	2017	Complications following therapeutic procedures	0.0506
Kyrgyzstan	1990	Multiple fractures, dislocations, crashes, wounds, sprains, and strains	0.1658
Kyrgyzstan	1995	Multiple fractures, dislocations, crashes, wounds, sprains, and strains	0.1682
Kyrgyzstan	2000	Multiple fractures, dislocations, crashes, wounds, sprains, and strains	0.1585
Kyrgyzstan	2005	Multiple fractures, dislocations, crashes, wounds, sprains, and strains	0.1493
Kyrgyzstan	2010	Multiple fractures, dislocations, crashes, wounds, sprains, and strains	0.1377
Kyrgyzstan	2017	Multiple fractures, dislocations, crashes, wounds, sprains, and strains	0.1201
Kyrgyzstan	1990	Amputation of upper limb, unilateral	0.0638
Kyrgyzstan	1995	Amputation of upper limb, unilateral	0.0643
Kyrgyzstan	2000	Amputation of upper limb, unilateral	0.0621
Kyrgyzstan	2005	Amputation of upper limb, unilateral	0.06
Kyrgyzstan	2010	Amputation of upper limb, unilateral	0.0573
Kyrgyzstan	2017	Amputation of upper limb, unilateral	0.0533
Kyrgyzstan	1990	Amputation of thumb	0.0114
Kyrgyzstan	1995	Amputation of thumb	0.0114
Kyrgyzstan	2000	Amputation of thumb	0.0114
Kyrgyzstan	2005	Amputation of thumb	0.0114
Kyrgyzstan	2010	Amputation of thumb	0.0114
Kyrgyzstan	2017	Amputation of thumb	0.0114
Kyrgyzstan	1990	Amputation of toe/toes	0.0059
Kyrgyzstan	1995	Amputation of toe/toes	0.0059
Kyrgyzstan	2000	Amputation of toe/toes	0.0059
Kyrgyzstan	2005	Amputation of toe/toes	0.0059
Kyrgyzstan	2010	Amputation of toe/toes	0.0059
Kyrgyzstan	2017	Amputation of toe/toes	0.0059
Kyrgyzstan	1990	Burns, <20% total burned surface area without lower airway burns	0.0163
Kyrgyzstan	1995	Burns, <20% total burned surface area without lower airway burns	0.0163
Kyrgyzstan	2000	Burns, <20% total burned surface area without lower airway burns	0.0163
Kyrgyzstan	2005	Burns, <20% total burned surface area without lower airway burns	0.0163
Kyrgyzstan	2010	Burns, <20% total burned surface area without lower airway burns	0.0163
Kyrgyzstan	2017	Burns, <20% total burned surface area without lower airway burns	0.0163
Kyrgyzstan	1990	Burns, >=20% total burned surface area or >= 10% burned surface area if head/neck or hands/wrist involved w/o lower airway burns	0.2353
Kyrgyzstan	1995	Burns, >=20% total burned surface area or >= 10% burned surface area if head/neck or hands/wrist involved w/o lower airway burns	0.2375
Kyrgyzstan	2000	Burns, >=20% total burned surface area or >= 10% burned surface area if head/neck or hands/wrist involved w/o lower airway burns	0.2285
Kyrgyzstan	2005	Burns, >=20% total burned surface area or >= 10% burned surface area if head/neck or hands/wrist involved w/o lower airway burns	0.2198
Kyrgyzstan	2010	Burns, >=20% total burned surface area or >= 10% burned surface area if head/neck or hands/wrist involved w/o lower airway burns	0.2091
Kyrgyzstan	2017	Burns, >=20% total burned surface area or >= 10% burned surface area if head/neck or hands/wrist involved w/o lower airway burns	0.1927
Mongolia	1990	Amputation of lower limbs, bilateral	0.253
Mongolia	1995	Amputation of lower limbs, bilateral	0.2532
Mongolia	2000	Amputation of lower limbs, bilateral	0.2447
Mongolia	2005	Amputation of lower limbs, bilateral	0.2176
Mongolia	2010	Amputation of lower limbs, bilateral	0.1997
Mongolia	2017	Amputation of lower limbs, bilateral	0.1946
Mongolia	1990	Lower airway burns	0.2252
Mongolia	1995	Lower airway burns	0.2252
Mongolia	2000	Lower airway burns	0.2252
Mongolia	2005	Lower airway burns	0.2252
Mongolia	2010	Lower airway burns	0.2252
Mongolia	2017	Lower airway burns	0.2252
Mongolia	1990	Dislocation of hip	0.0159
Mongolia	1995	Dislocation of hip	0.0159
Mongolia	2000	Dislocation of hip	0.0159
Mongolia	2005	Dislocation of hip	0.0159
Mongolia	2010	Dislocation of hip	0.0159
Mongolia	2017	Dislocation of hip	0.0159
Mongolia	1990	Dislocation of knee	0.1134
Mongolia	1995	Dislocation of knee	0.1134
Mongolia	2000	Dislocation of knee	0.1134
Mongolia	2005	Dislocation of knee	0.1134
Mongolia	2010	Dislocation of knee	0.1134
Mongolia	2017	Dislocation of knee	0.1134
Mongolia	1990	Dislocation of shoulder	0.062
Mongolia	1995	Dislocation of shoulder	0.062
Mongolia	2000	Dislocation of shoulder	0.062
Mongolia	2005	Dislocation of shoulder	0.062
Mongolia	2010	Dislocation of shoulder	0.062
Mongolia	2017	Dislocation of shoulder	0.062
Mongolia	1990	Muscle and tendon injuries, including sprains and strains lesser dislocations	0.0075
Mongolia	1995	Muscle and tendon injuries, including sprains and strains lesser dislocations	0.0075
Mongolia	2000	Muscle and tendon injuries, including sprains and strains lesser dislocations	0.0075
Mongolia	2005	Muscle and tendon injuries, including sprains and strains lesser dislocations	0.0075
Mongolia	2010	Muscle and tendon injuries, including sprains and strains lesser dislocations	0.0075
Mongolia	2017	Muscle and tendon injuries, including sprains and strains lesser dislocations	0.0075
Mongolia	1990	Fracture of clavicle, scapula, or humerus	0.0349
Mongolia	1995	Fracture of clavicle, scapula, or humerus	0.0349
Mongolia	2000	Fracture of clavicle, scapula, or humerus	0.0349
Mongolia	2005	Fracture of clavicle, scapula, or humerus	0.0349
Mongolia	2010	Fracture of clavicle, scapula, or humerus	0.0349
Mongolia	2017	Fracture of clavicle, scapula, or humerus	0.0349
Mongolia	1990	Fracture of face bones	0.0669
Mongolia	1995	Fracture of face bones	0.0669
Mongolia	2000	Fracture of face bones	0.0669
Mongolia	2005	Fracture of face bones	0.0669
Mongolia	2010	Fracture of face bones	0.0669
Mongolia	2017	Fracture of face bones	0.0669
Mongolia	1990	Fracture of foot bones except ankle	0.0264
Mongolia	1995	Fracture of foot bones except ankle	0.0264
Mongolia	2000	Fracture of foot bones except ankle	0.0264
Mongolia	2005	Fracture of foot bones except ankle	0.0264
Mongolia	2010	Fracture of foot bones except ankle	0.0264
Mongolia	2017	Fracture of foot bones except ankle	0.0264
Mongolia	1990	Fracture of hand (wrist and other distal part of hand)	0.0138
Mongolia	1995	Fracture of hand (wrist and other distal part of hand)	0.0138
Mongolia	2000	Fracture of hand (wrist and other distal part of hand)	0.0138
Mongolia	2005	Fracture of hand (wrist and other distal part of hand)	0.0138
Mongolia	2010	Fracture of hand (wrist and other distal part of hand)	0.0138
Mongolia	2017	Fracture of hand (wrist and other distal part of hand)	0.0138
Mongolia	1990	Fracture of hip	0.2293
Mongolia	1995	Fracture of hip	0.2296
Mongolia	2000	Fracture of hip	0.2183
Mongolia	2005	Fracture of hip	0.1825
Mongolia	2010	Fracture of hip	0.1589
Mongolia	2017	Fracture of hip	0.1521
Mongolia	1990	Amputation of upper limbs, bilateral	0.2648
Mongolia	1995	Amputation of upper limbs, bilateral	0.2651
Mongolia	2000	Amputation of upper limbs, bilateral	0.2535
Mongolia	2005	Amputation of upper limbs, bilateral	0.2165
Mongolia	2010	Amputation of upper limbs, bilateral	0.1921
Mongolia	2017	Amputation of upper limbs, bilateral	0.1851
Mongolia	1990	Fracture of patella, tibia or fibula, or ankle	0.0554
Mongolia	1995	Fracture of patella, tibia or fibula, or ankle	0.0554
Mongolia	2000	Fracture of patella, tibia or fibula, or ankle	0.0554
Mongolia	2005	Fracture of patella, tibia or fibula, or ankle	0.0554
Mongolia	2010	Fracture of patella, tibia or fibula, or ankle	0.0554
Mongolia	2017	Fracture of patella, tibia or fibula, or ankle	0.0554
Mongolia	1990	Fracture of pelvis	0.1823
Mongolia	1995	Fracture of pelvis	0.1823
Mongolia	2000	Fracture of pelvis	0.1823
Mongolia	2005	Fracture of pelvis	0.1823
Mongolia	2010	Fracture of pelvis	0.1823
Mongolia	2017	Fracture of pelvis	0.1823
Mongolia	1990	Fracture of radius and/or ulna	0.0435
Mongolia	1995	Fracture of radius and/or ulna	0.0435
Mongolia	2000	Fracture of radius and/or ulna	0.0435
Mongolia	2005	Fracture of radius and/or ulna	0.0435
Mongolia	2010	Fracture of radius and/or ulna	0.0435
Mongolia	2017	Fracture of radius and/or ulna	0.0435
Mongolia	1990	Fracture of skull	0.0714
Mongolia	1995	Fracture of skull	0.0714
Mongolia	2000	Fracture of skull	0.0714
Mongolia	2005	Fracture of skull	0.0714
Mongolia	2010	Fracture of skull	0.0714
Mongolia	2017	Fracture of skull	0.0714
Mongolia	1990	Fracture of sternum and/or fracture of one or more ribs	0.1027
Mongolia	1995	Fracture of sternum and/or fracture of one or more ribs	0.1027
Mongolia	2000	Fracture of sternum and/or fracture of one or more ribs	0.1027
Mongolia	2005	Fracture of sternum and/or fracture of one or more ribs	0.1027
Mongolia	2010	Fracture of sternum and/or fracture of one or more ribs	0.1027
Mongolia	2017	Fracture of sternum and/or fracture of one or more ribs	0.1027
Mongolia	1990	Fracture of vertebral column	0.1106
Mongolia	1995	Fracture of vertebral column	0.1106
Mongolia	2000	Fracture of vertebral column	0.1106
Mongolia	2005	Fracture of vertebral column	0.1106
Mongolia	2010	Fracture of vertebral column	0.1106
Mongolia	2017	Fracture of vertebral column	0.1106
Mongolia	1990	Fracture of femur, other than femoral neck	0.042
Mongolia	1995	Fracture of femur, other than femoral neck	0.042
Mongolia	2000	Fracture of femur, other than femoral neck	0.042
Mongolia	2005	Fracture of femur, other than femoral neck	0.042
Mongolia	2010	Fracture of femur, other than femoral neck	0.042
Mongolia	2017	Fracture of femur, other than femoral neck	0.042
Mongolia	1990	Minor TBI	0.1319
Mongolia	1995	Minor TBI	0.1319
Mongolia	2000	Minor TBI	0.1319
Mongolia	2005	Minor TBI	0.1319
Mongolia	2010	Minor TBI	0.1319
Mongolia	2017	Minor TBI	0.1319
Mongolia	1990	Moderate TBI	0.1637
Mongolia	1995	Moderate TBI	0.1637
Mongolia	2000	Moderate TBI	0.1637
Mongolia	2005	Moderate TBI	0.1637
Mongolia	2010	Moderate TBI	0.1637
Mongolia	2017	Moderate TBI	0.1637
Mongolia	1990	Amputation of fingers (excluding thumb)	0.0047
Mongolia	1995	Amputation of fingers (excluding thumb)	0.0047
Mongolia	2000	Amputation of fingers (excluding thumb)	0.0047
Mongolia	2005	Amputation of fingers (excluding thumb)	0.0047
Mongolia	2010	Amputation of fingers (excluding thumb)	0.0047
Mongolia	2017	Amputation of fingers (excluding thumb)	0.0047
Mongolia	1990	Spinal cord lesion at neck level	0.4712
Mongolia	1995	Spinal cord lesion at neck level	0.4713
Mongolia	2000	Spinal cord lesion at neck level	0.4674
Mongolia	2005	Spinal cord lesion at neck level	0.455
Mongolia	2010	Spinal cord lesion at neck level	0.4468
Mongolia	2017	Spinal cord lesion at neck level	0.4445
Mongolia	1990	Spinal cord lesion below neck level	0.3029
Mongolia	1995	Spinal cord lesion below neck level	0.3031
Mongolia	2000	Spinal cord lesion below neck level	0.2949
Mongolia	2005	Spinal cord lesion below neck level	0.2688
Mongolia	2010	Spinal cord lesion below neck level	0.2515
Mongolia	2017	Spinal cord lesion below neck level	0.2466
Mongolia	1990	Drowning and nonfatal submersion	0.2471
Mongolia	1995	Drowning and nonfatal submersion	0.2471
Mongolia	2000	Drowning and nonfatal submersion	0.2471
Mongolia	2005	Drowning and nonfatal submersion	0.2471
Mongolia	2010	Drowning and nonfatal submersion	0.2471
Mongolia	2017	Drowning and nonfatal submersion	0.2471
Mongolia	1990	Asphyxiation	0.2471
Mongolia	1995	Asphyxiation	0.2471
Mongolia	2000	Asphyxiation	0.2471
Mongolia	2005	Asphyxiation	0.2471
Mongolia	2010	Asphyxiation	0.2471
Mongolia	2017	Asphyxiation	0.2471
Mongolia	1990	Crush injury	0.1325
Mongolia	1995	Crush injury	0.1325
Mongolia	2000	Crush injury	0.1325
Mongolia	2005	Crush injury	0.1325
Mongolia	2010	Crush injury	0.1325
Mongolia	2017	Crush injury	0.1325
Mongolia	1990	Nerve injury	0.1129
Mongolia	1995	Nerve injury	0.1129
Mongolia	2000	Nerve injury	0.1129
Mongolia	2005	Nerve injury	0.1129
Mongolia	2010	Nerve injury	0.1129
Mongolia	2017	Nerve injury	0.1129
Mongolia	1990	Injury to eyes	0.0165
Mongolia	1995	Injury to eyes	0.0165
Mongolia	2000	Injury to eyes	0.0165
Mongolia	2005	Injury to eyes	0.0165
Mongolia	2010	Injury to eyes	0.0165
Mongolia	2017	Injury to eyes	0.0165
Mongolia	1990	Amputation of lower limb, unilateral	0.1058
Mongolia	1995	Amputation of lower limb, unilateral	0.106
Mongolia	2000	Amputation of lower limb, unilateral	0.1016
Mongolia	2005	Amputation of lower limb, unilateral	0.0876
Mongolia	2010	Amputation of lower limb, unilateral	0.0784
Mongolia	2017	Amputation of lower limb, unilateral	0.0758
Mongolia	1990	Open wound(s)	0.011
Mongolia	1995	Open wound(s)	0.011
Mongolia	2000	Open wound(s)	0.011
Mongolia	2005	Open wound(s)	0.011
Mongolia	2010	Open wound(s)	0.011
Mongolia	2017	Open wound(s)	0.011
Mongolia	1990	Poisoning requiring urgent care	0.1628

Mongolia	1995	Poisoning requiring urgent care	0.1628
Mongolia	2000	Poisoning requiring urgent care	0.1628
Mongolia	2005	Poisoning requiring urgent care	0.1628
Mongolia	2010	Poisoning requiring urgent care	0.1628
Mongolia	2017	Poisoning requiring urgent care	0.1628
Mongolia	1990	Severe chest injury	0.0474
Mongolia	1995	Severe chest injury	0.0474
Mongolia	2000	Severe chest injury	0.0474
Mongolia	2005	Severe chest injury	0.0474
Mongolia	2010	Severe chest injury	0.0474
Mongolia	2017	Severe chest injury	0.0474
Mongolia	1990	Internal hemorrhage in abdomen and pelvis	0.1143
Mongolia	1995	Internal hemorrhage in abdomen and pelvis	0.1143
Mongolia	2000	Internal hemorrhage in abdomen and pelvis	0.1143
Mongolia	2005	Internal hemorrhage in abdomen and pelvis	0.1143
Mongolia	2010	Internal hemorrhage in abdomen and pelvis	0.1143
Mongolia	2017	Internal hemorrhage in abdomen and pelvis	0.1143
Mongolia	1990	Contusion in any part of the body	0.0075
Mongolia	1995	Contusion in any part of the body	0.0075
Mongolia	2000	Contusion in any part of the body	0.0075
Mongolia	2005	Contusion in any part of the body	0.0075
Mongolia	2010	Contusion in any part of the body	0.0075
Mongolia	2017	Contusion in any part of the body	0.0075
Mongolia	1990	Effect of different environmental factors	0.049
Mongolia	1995	Effect of different environmental factors	0.049
Mongolia	2000	Effect of different environmental factors	0.049
Mongolia	2005	Effect of different environmental factors	0.049
Mongolia	2010	Effect of different environmental factors	0.049
Mongolia	2017	Effect of different environmental factors	0.049
Mongolia	1990	Complications following therapeutic procedures	0.0506
Mongolia	1995	Complications following therapeutic procedures	0.0506
Mongolia	2000	Complications following therapeutic procedures	0.0506
Mongolia	2005	Complications following therapeutic procedures	0.0506
Mongolia	2010	Complications following therapeutic procedures	0.0506
Mongolia	2017	Complications following therapeutic procedures	0.0506
Mongolia	1990	Multiple fractures, dislocations, crashes, wounds, sprains, and strains	0.2293
Mongolia	1995	Multiple fractures, dislocations, crashes, wounds, sprains, and strains	0.2296
Mongolia	2000	Multiple fractures, dislocations, crashes, wounds, sprains, and strains	0.2183
Mongolia	2005	Multiple fractures, dislocations, crashes, wounds, sprains, and strains	0.1825
Mongolia	2010	Multiple fractures, dislocations, crashes, wounds, sprains, and strains	0.1589
Mongolia	2017	Multiple fractures, dislocations, crashes, wounds, sprains, and strains	0.1521
Mongolia	1990	Amputation of upper limb, unilateral	0.0784
Mongolia	1995	Amputation of upper limb, unilateral	0.0784
Mongolia	2000	Amputation of upper limb, unilateral	0.0758
Mongolia	2005	Amputation of upper limb, unilateral	0.0676
Mongolia	2010	Amputation of upper limb, unilateral	0.0622
Mongolia	2017	Amputation of upper limb, unilateral	0.0606
Mongolia	1990	Amputation of thumb	0.0114
Mongolia	1995	Amputation of thumb	0.0114
Mongolia	2000	Amputation of thumb	0.0114
Mongolia	2005	Amputation of thumb	0.0114
Mongolia	2010	Amputation of thumb	0.0114
Mongolia	2017	Amputation of thumb	0.0114
Mongolia	1990	Amputation of toe/toes	0.0059
Mongolia	1995	Amputation of toe/toes	0.0059
Mongolia	2000	Amputation of toe/toes	0.0059
Mongolia	2005	Amputation of toe/toes	0.0059
Mongolia	2010	Amputation of toe/toes	0.0059
Mongolia	2017	Amputation of toe/toes	0.0059
Mongolia	1990	Burns, <20% total burned surface area without lower airway burns	0.0163
Mongolia	1995	Burns, <20% total burned surface area without lower airway burns	0.0163
Mongolia	2000	Burns, <20% total burned surface area without lower airway burns	0.0163
Mongolia	2005	Burns, <20% total burned surface area without lower airway burns	0.0163
Mongolia	2010	Burns, <20% total burned surface area without lower airway burns	0.0163
Mongolia	2017	Burns, <20% total burned surface area without lower airway burns	0.0163
Mongolia	1990	Burns, >=20% total burned surface area or >= 10% burned surface area if head/neck or hands/wrist involved w/o lower airway burns	0.2944
Mongolia	1995	Burns, >=20% total burned surface area or >= 10% burned surface area if head/neck or hands/wrist involved w/o lower airway burns	0.2946
Mongolia	2000	Burns, >=20% total burned surface area or >= 10% burned surface area if head/neck or hands/wrist involved w/o lower airway burns	0.2842
Mongolia	2005	Burns, >=20% total burned surface area or >= 10% burned surface area if head/neck or hands/wrist involved w/o lower airway burns	0.2508
Mongolia	2010	Burns, >=20% total burned surface area or >= 10% burned surface area if head/neck or hands/wrist involved w/o lower airway burns	0.2288
Mongolia	2017	Burns, >=20% total burned surface area or >= 10% burned surface area if head/neck or hands/wrist involved w/o lower airway burns	0.2225
Tajikistan	1990	Amputation of lower limbs, bilateral	0.237
Tajikistan	1995	Amputation of lower limbs, bilateral	0.2324
Tajikistan	2000	Amputation of lower limbs, bilateral	0.2331
Tajikistan	2005	Amputation of lower limbs, bilateral	0.2227
Tajikistan	2010	Amputation of lower limbs, bilateral	0.2127
Tajikistan	2017	Amputation of lower limbs, bilateral	0.2005
Tajikistan	1990	Lower airway burns	0.2252
Tajikistan	1995	Lower airway burns	0.2252
Tajikistan	2000	Lower airway burns	0.2252
Tajikistan	2005	Lower airway burns	0.2252
Tajikistan	2010	Lower airway burns	0.2252
Tajikistan	2017	Lower airway burns	0.2252
Tajikistan	1990	Dislocation of hip	0.0159
Tajikistan	1995	Dislocation of hip	0.0159
Tajikistan	2000	Dislocation of hip	0.0159
Tajikistan	2005	Dislocation of hip	0.0159
Tajikistan	2010	Dislocation of hip	0.0159
Tajikistan	2017	Dislocation of hip	0.0159
Tajikistan	1990	Dislocation of knee	0.1134
Tajikistan	1995	Dislocation of knee	0.1134
Tajikistan	2000	Dislocation of knee	0.1134
Tajikistan	2005	Dislocation of knee	0.1134
Tajikistan	2010	Dislocation of knee	0.1134
Tajikistan	2017	Dislocation of knee	0.1134
Tajikistan	1990	Dislocation of shoulder	0.062
Tajikistan	1995	Dislocation of shoulder	0.062
Tajikistan	2000	Dislocation of shoulder	0.062
Tajikistan	2005	Dislocation of shoulder	0.062
Tajikistan	2010	Dislocation of shoulder	0.062
Tajikistan	2017	Dislocation of shoulder	0.062
Tajikistan	1990	Muscle and tendon injuries, including sprains and strains lesser dislocations	0.0075
Tajikistan	1995	Muscle and tendon injuries, including sprains and strains lesser dislocations	0.0075
Tajikistan	2000	Muscle and tendon injuries, including sprains and strains lesser dislocations	0.0075
Tajikistan	2005	Muscle and tendon injuries, including sprains and strains lesser dislocations	0.0075
Tajikistan	2010	Muscle and tendon injuries, including sprains and strains lesser dislocations	0.0075
Tajikistan	2017	Muscle and tendon injuries, including sprains and strains lesser dislocations	0.0075
Tajikistan	1990	Fracture of clavicle, scapula, or humerus	0.0349
Tajikistan	1995	Fracture of clavicle, scapula, or humerus	0.0349
Tajikistan	2000	Fracture of clavicle, scapula, or humerus	0.0349
Tajikistan	2005	Fracture of clavicle, scapula, or humerus	0.0349
Tajikistan	2010	Fracture of clavicle, scapula, or humerus	0.0349
Tajikistan	2017	Fracture of clavicle, scapula, or humerus	0.0349
Tajikistan	1990	Fracture of face bones	0.0669
Tajikistan	1995	Fracture of face bones	0.0669
Tajikistan	2000	Fracture of face bones	0.0669
Tajikistan	2005	Fracture of face bones	0.0669
Tajikistan	2010	Fracture of face bones	0.0669
Tajikistan	2017	Fracture of face bones	0.0669
Tajikistan	1990	Fracture of foot bones except ankle	0.0264
Tajikistan	1995	Fracture of foot bones except ankle	0.0264
Tajikistan	2000	Fracture of foot bones except ankle	0.0264
Tajikistan	2005	Fracture of foot bones except ankle	0.0264
Tajikistan	2010	Fracture of foot bones except ankle	0.0264
Tajikistan	2017	Fracture of foot bones except ankle	0.0264
Tajikistan	1990	Fracture of hand (wrist and other distal part of hand)	0.0138
Tajikistan	1995	Fracture of hand (wrist and other distal part of hand)	0.0138
Tajikistan	2000	Fracture of hand (wrist and other distal part of hand)	0.0138
Tajikistan	2005	Fracture of hand (wrist and other distal part of hand)	0.0138
Tajikistan	2010	Fracture of hand (wrist and other distal part of hand)	0.0138
Tajikistan	2017	Fracture of hand (wrist and other distal part of hand)	0.0138
Tajikistan	1990	Fracture of hip	0.2082
Tajikistan	1995	Fracture of hip	0.2021
Tajikistan	2000	Fracture of hip	0.203
Tajikistan	2005	Fracture of hip	0.1892
Tajikistan	2010	Fracture of hip	0.176
Tajikistan	2017	Fracture of hip	0.1599
Tajikistan	1990	Amputation of upper limbs, bilateral	0.243
Tajikistan	1995	Amputation of upper limbs, bilateral	0.2367
Tajikistan	2000	Amputation of upper limbs, bilateral	0.2377
Tajikistan	2005	Amputation of upper limbs, bilateral	0.2235
Tajikistan	2010	Amputation of upper limbs, bilateral	0.2098
Tajikistan	2017	Amputation of upper limbs, bilateral	0.1932
Tajikistan	1990	Fracture of patella, tibia or fibula, or ankle	0.0554
Tajikistan	1995	Fracture of patella, tibia or fibula, or ankle	0.0554
Tajikistan	2000	Fracture of patella, tibia or fibula, or ankle	0.0554
Tajikistan	2005	Fracture of patella, tibia or fibula, or ankle	0.0554
Tajikistan	2010	Fracture of patella, tibia or fibula, or ankle	0.0554
Tajikistan	2017	Fracture of patella, tibia or fibula, or ankle	0.0554
Tajikistan	1990	Fracture of pelvis	0.1823
Tajikistan	1995	Fracture of pelvis	0.1823
Tajikistan	2000	Fracture of pelvis	0.1823
Tajikistan	2005	Fracture of pelvis	0.1823
Tajikistan	2010	Fracture of pelvis	0.1823
Tajikistan	2017	Fracture of pelvis	0.1823
Tajikistan	1990	Fracture of radius and/or ulna	0.0435
Tajikistan	1995	Fracture of radius and/or ulna	0.0435
Tajikistan	2000	Fracture of radius and/or ulna	0.0435
Tajikistan	2005	Fracture of radius and/or ulna	0.0435
Tajikistan	2010	Fracture of radius and/or ulna	0.0435
Tajikistan	2017	Fracture of radius and/or ulna	0.0435
Tajikistan	1990	Fracture of skull	0.0714
Tajikistan	1995	Fracture of skull	0.0714
Tajikistan	2000	Fracture of skull	0.0714
Tajikistan	2005	Fracture of skull	0.0714
Tajikistan	2010	Fracture of skull	0.0714
Tajikistan	2017	Fracture of skull	0.0714
Tajikistan	1990	Fracture of sternum and/or fracture of one or more ribs	0.1027
Tajikistan	1995	Fracture of sternum and/or fracture of one or more ribs	0.1027
Tajikistan	2000	Fracture of sternum and/or fracture of one or more ribs	0.1027
Tajikistan	2005	Fracture of sternum and/or fracture of one or more ribs	0.1027
Tajikistan	2010	Fracture of sternum and/or fracture of one or more ribs	0.1027
Tajikistan	2017	Fracture of sternum and/or fracture of one or more ribs	0.1027
Tajikistan	1990	Fracture of vertebral column	0.1106
Tajikistan	1995	Fracture of vertebral column	0.1106
Tajikistan	2000	Fracture of vertebral column	0.1106
Tajikistan	2005	Fracture of vertebral column	0.1106
Tajikistan	2010	Fracture of vertebral column	0.1106
Tajikistan	2017	Fracture of vertebral column	0.1106
Tajikistan	1990	Fracture of femur, other than femoral neck	0.042
Tajikistan	1995	Fracture of femur, other than femoral neck	0.042
Tajikistan	2000	Fracture of femur, other than femoral neck	0.042
Tajikistan	2005	Fracture of femur, other than femoral neck	0.042
Tajikistan	2010	Fracture of femur, other than femoral neck	0.042
Tajikistan	2017	Fracture of femur, other than femoral neck	0.042
Tajikistan	1990	Minor TBI	0.1319
Tajikistan	1995	Minor TBI	0.1319
Tajikistan	2000	Minor TBI	0.1319
Tajikistan	2005	Minor TBI	0.1319
Tajikistan	2010	Minor TBI	0.1319
Tajikistan	2017	Minor TBI	0.1319
Tajikistan	1990	Moderate TBI	0.1637
Tajikistan	1995	Moderate TBI	0.1637
Tajikistan	2000	Moderate TBI	0.1637
Tajikistan	2005	Moderate TBI	0.1637
Tajikistan	2010	Moderate TBI	0.1637
Tajikistan	2017	Moderate TBI	0.1637
Tajikistan	1990	Amputation of fingers (excluding thumb)	0.0047
Tajikistan	1995	Amputation of fingers (excluding thumb)	0.0047
Tajikistan	2000	Amputation of fingers (excluding thumb)	0.0047
Tajikistan	2005	Amputation of fingers (excluding thumb)	0.0047
Tajikistan	2010	Amputation of fingers (excluding thumb)	0.0047
Tajikistan	2017	Amputation of fingers (excluding thumb)	0.0047
Tajikistan	1990	Spinal cord lesion at neck level	0.4639
Tajikistan	1995	Spinal cord lesion at neck level	0.4618
Tajikistan	2000	Spinal cord lesion at neck level	0.4621
Tajikistan	2005	Spinal cord lesion at neck level	0.4573
Tajikistan	2010	Spinal cord lesion at neck level	0.4527
Tajikistan	2017	Spinal cord lesion at neck level	0.4471
Tajikistan	1990	Spinal cord lesion below neck level	0.2875
Tajikistan	1995	Spinal cord lesion below neck level	0.283
Tajikistan	2000	Spinal cord lesion below neck level	0.2837
Tajikistan	2005	Spinal cord lesion below neck level	0.2737
Tajikistan	2010	Spinal cord lesion below neck level	0.264
Tajikistan	2017	Spinal cord lesion below neck level	0.2522
Tajikistan	1990	Drowning and nonfatal submersion	0.2471
Tajikistan	1995	Drowning and nonfatal submersion	0.2471
Tajikistan	2000	Drowning and nonfatal submersion	0.2471
Tajikistan	2005	Drowning and nonfatal submersion	0.2471
Tajikistan	2010	Drowning and nonfatal submersion	0.2471
Tajikistan	2017	Drowning and nonfatal submersion	0.2471
Tajikistan	1990	Asphyxiation	0.2471
Tajikistan	1995	Asphyxiation	0.2471
Tajikistan	2000	Asphyxiation	0.2471
Tajikistan	2005	Asphyxiation	0.2471
Tajikistan	2010	Asphyxiation	0.2471
Tajikistan	2017	Asphyxiation	0.2471
Tajikistan	1990	Crush injury	0.1325
Tajikistan	1995	Crush injury	0.1325
Tajikistan	2000	Crush injury	0.1325
Tajikistan	2005	Crush injury	0.1325
Tajikistan	2010	Crush injury	0.1325
Tajikistan	2017	Crush injury	0.1325
Tajikistan	1990	Nerve injury	0.1129
Tajikistan	1995	Nerve injury	0.1129
Tajikistan	2000	Nerve injury	0.1129
Tajikistan	2005	Nerve injury	0.1129
Tajikistan	2010	Nerve injury	0.1129
Tajikistan	2017	Nerve injury	0.1129
Tajikistan	1990	Injury to eyes	0.0165
Tajikistan	1995	Injury to eyes	0.0165
Tajikistan	2000	Injury to eyes	0.0165
Tajikistan	2005	Injury to eyes	0.0165
Tajikistan	2010	Injury to eyes	0.0165
Tajikistan	2017	Injury to eyes	0.0165
Tajikistan	1990	Amputation of lower limb, unilateral	0.0976
Tajikistan	1995	Amputation of lower limb, unilateral	0.0952
Tajikistan	2000	Amputation of lower limb, unilateral	0.0956
Tajikistan	2005	Amputation of lower limb, unilateral	0.0902
Tajikistan	2010	Amputation of lower limb, unilateral	0.0851
Tajikistan	2017	Amputation of lower limb, unilateral	0.0788
Tajikistan	1990	Open wound(s)	0.011
Tajikistan	1995	Open wound(s)	0.011
Tajikistan	2000	Open wound(s)	0.011
Tajikistan	2005	Open wound(s)	0.011
Tajikistan	2010	Open wound(s)	0.011
Tajikistan	2017	Open wound(s)	0.011
Tajikistan	1990	Poisoning requiring urgent care	0.1628
Tajikistan	1995	Poisoning requiring urgent care	0.1628
Tajikistan	2000	Poisoning requiring urgent care	0.1628
Tajikistan	2005	Poisoning requiring urgent care	0.1628
Tajikistan	2010	Poisoning requiring urgent care	0.1628
Tajikistan	2017	Poisoning requiring urgent care	0.1628
Tajikistan	1990	Severe chest injury	0.0474
Tajikistan	1995	Severe chest injury	0.0474
Tajikistan	2000	Severe chest injury	0.0474
Tajikistan	2005	Severe chest injury	0.0474
Tajikistan	2010	Severe chest injury	0.0474
Tajikistan	2017	Severe chest injury	0.0474
Tajikistan	1990	Internal hemorrhage in abdomen and pelvis	0.1143
Tajikistan	1995	Internal hemorrhage in abdomen and pelvis	0.1143
Tajikistan	2000	Internal hemorrhage in abdomen and pelvis	0.1143
Tajikistan	2005	Internal hemorrhage in abdomen and pelvis	0.1143
Tajikistan	2010	Internal hemorrhage in abdomen and pelvis	0.1143
Tajikistan	2017	Internal hemorrhage in abdomen and pelvis	0.1143
Tajikistan	1990	Contusion in any part of the body	0.0075
Tajikistan	1995	Contusion in any part of the body	0.0075
Tajikistan	2000	Contusion in any part of the body	0.0075
Tajikistan	2005	Contusion in any part of the body	0.0075
Tajikistan	2010	Contusion in any part of the body	0.0075
Tajikistan	2017	Contusion in any part of the body	0.0075
Tajikistan	1990	Effect of different environmental factors	0.049
Tajikistan	1995	Effect of different environmental factors	0.049
Tajikistan	2000	Effect of different environmental factors	0.049
Tajikistan	2005	Effect of different environmental factors	0.049
Tajikistan	2010	Effect of different environmental factors	0.049
Tajikistan	2017	Effect of different environmental factors	0.049
Tajikistan	1990	Complications following therapeutic procedures	0.0506
Tajikistan	1995	Complications following therapeutic procedures	0.0506
Tajikistan	2000	Complications following therapeutic procedures	0.0506
Tajikistan	2005	Complications following therapeutic procedures	0.0506
Tajikistan	2010	Complications following therapeutic procedures	0.0506
Tajikistan	2017	Complications following therapeutic procedures	0.0506
Tajikistan	1990	Multiple fractures, dislocations, crashes, wounds, sprains, and strains	0.2082
Tajikistan	1995	Multiple fractures, dislocations, crashes, wounds, sprains, and strains	0.2021
Tajikistan	2000	Multiple fractures, dislocations, crashes, wounds, sprains, and strains	0.203
Tajikistan	2005	Multiple fractures, dislocations, crashes, wounds, sprains, and strains	0.1892
Tajikistan	2010	Multiple fractures, dislocations, crashes, wounds, sprains, and strains	0.176
Tajikistan	2017	Multiple fractures, dislocations, crashes, wounds, sprains, and strains	0.1599
Tajikistan	1990	Amputation of upper limb, unilateral	0.0735
Tajikistan	1995	Amputation of upper limb, unilateral	0.0721
Tajikistan	2000	Amputation of upper limb, unilateral	0.0723
Tajikistan	2005	Amputation of upper limb, unilateral	0.0692
Tajikistan	2010	Amputation of upper limb, unilateral	0.0661
Tajikistan	2017	Amputation of upper limb, unilateral	0.0624
Tajikistan	1990	Amputation of thumb	0.0114
Tajikistan	1995	Amputation of thumb	0.0114
Tajikistan	2000	Amputation of thumb	0.0114
Tajikistan	2005	Amputation of thumb	0.0114
Tajikistan	2010	Amputation of thumb	0.0114
Tajikistan	2017	Amputation of thumb	0.0114
Tajikistan	1990	Amputation of toe/toes	0.0059
Tajikistan	1995	Amputation of toe/toes	0.0059
Tajikistan	2000	Amputation of toe/toes	0.0059
Tajikistan	2005	Amputation of toe/toes	0.0059
Tajikistan	2010	Amputation of toe/toes	0.0059
Tajikistan	2017	Amputation of toe/toes	0.0059
Tajikistan	1990	Burns, <20% total burned surface area without lower airway burns	0.0163
Tajikistan	1995	Burns, <20% total burned surface area without lower airway burns	0.0163
Tajikistan	2000	Burns, <20% total burned surface area without lower airway burns	0.0163
Tajikistan	2005	Burns, <20% total burned surface area without lower airway burns	0.0163
Tajikistan	2010	Burns, <20% total burned surface area without lower airway burns	0.0163
Tajikistan	2017	Burns, <20% total burned surface area without lower airway burns	0.0163
Tajikistan	1990	Burns, >=20% total burned surface area or >= 10% burned surface area if head/neck or hands/wrist involved w/o lower airway burns	0.2747
Tajikistan	1995	Burns, >=20% total burned surface area or >= 10% burned surface area if head/neck or hands/wrist involved w/o lower airway burns	0.269
Tajikistan	2000	Burns, >=20% total burned surface area or >= 10% burned surface area if head/neck or hands/wrist involved w/o lower airway burns	0.2699
Tajikistan	2005	Burns, >=20% total burned surface area or >= 10% burned surface area if head/neck or hands/wrist involved w/o lower airway burns	0.2571
Tajikistan	2010	Burns, >=20% total burned surface area or >= 10% burned surface area if head/neck or hands/wrist involved w/o lower airway burns	0.2447
Tajikistan	2017	Burns, >=20% total burned surface area or >= 10% burned surface area if head/neck or hands/wrist involved w/o lower airway burns	0.2297
Turkmenistan	1990	Amputation of lower limbs, bilateral	0.2232
Turkmenistan	1995	Amputation of lower limbs, bilateral	0.2176
Turkmenistan	2000	Amputation of lower limbs, bilateral	0.2114
Turkmenistan	2005	Amputation of lower limbs, bilateral	0.2029
Turkmenistan	2010	Amputation of lower limbs, bilateral	0.1878
Turkmenistan	2017	Amputation of lower limbs, bilateral	0.166
Turkmenistan	1990	Lower airway burns	0.2252
Turkmenistan	1995	Lower airway burns	0.2252
Turkmenistan	2000	Lower airway burns	0.2252
Turkmenistan	2005	Lower airway burns	0.2252
Turkmenistan	2010	Lower airway burns	0.2252
Turkmenistan	2017	Lower airway burns	0.2252
Turkmenistan	1990	Dislocation of hip	0.0159
Turkmenistan	1995	Dislocation of hip	0.0159
Turkmenistan	2000	Dislocation of hip	0.0159
Turkmenistan	2005	Dislocation of hip	0.0159
Turkmenistan	2010	Dislocation of hip	0.0159
Turkmenistan	2017	Dislocation of hip	0.0159
Turkmenistan	1990	Dislocation of knee	0.1134
Turkmenistan	1995	Dislocation of knee	0.1134
Turkmenistan	2000	Dislocation of knee	0.1134
Turkmenistan	2005	Dislocation of knee	0.1134
Turkmenistan	2010	Dislocation of knee	0.1134
Turkmenistan	2017	Dislocation of knee	0.1134
Turkmenistan	1990	Dislocation of shoulder	0.062
Turkmenistan	1995	Dislocation of shoulder	0.062
Turkmenistan	2000	Dislocation of shoulder	0.062
Turkmenistan	2005	Dislocation of shoulder	0.062
Turkmenistan	2010	Dislocation of shoulder	0.062
Turkmenistan	2017	Dislocation of shoulder	0.062
Turkmenistan	1990	Muscle and tendon injuries, including sprains and strains lesser dislocations	0.0075
Turkmenistan	1995	Muscle and tendon injuries, including sprains and strains lesser dislocations	0.0075
Turkmenistan	2000	Muscle and tendon injuries, including sprains and strains lesser dislocations	0.0075
Turkmenistan	2005	Muscle and tendon injuries, including sprains and strains lesser dislocations	0.0075
Turkmenistan	2010	Muscle and tendon injuries, including sprains and strains lesser dislocations	0.0075
Turkmenistan	2017	Muscle and tendon injuries, including sprains and strains lesser dislocations	0.0075
Turkmenistan	1990	Fracture of clavicle, scapula, or humerus	0.0349
Turkmenistan	1995	Fracture of clavicle, scapula, or humerus	0.0349
Turkmenistan	2000	Fracture of clavicle, scapula, or humerus	0.0349
Turkmenistan	2005	Fracture of clavicle, scapula, or humerus	0.0349
Turkmenistan	2010	Fracture of clavicle, scapula, or humerus	0.0349
Turkmenistan	2017	Fracture of clavicle, scapula, or humerus	0.0349
Turkmenistan	1990	Fracture of face bones	0.0669
Turkmenistan	1995	Fracture of face bones	0.0669
Turkmenistan	2000	Fracture of face bones	0.0669
Turkmenistan	2005	Fracture of face bones	0.0669
Turkmenistan	2010	Fracture of face bones	0.0669
Turkmenistan	2017	Fracture of face bones	0.0669
Turkmenistan	1990	Fracture of foot bones except ankle	0.0264
Turkmenistan	1995	Fracture of foot bones except ankle	0.0264
Turkmenistan	2000	Fracture of foot bones except ankle	0.0264
Turkmenistan	2005	Fracture of foot bones except ankle	0.0264
Turkmenistan	2010	Fracture of foot bones except ankle	0.0264
Turkmenistan	2017	Fracture of foot bones except ankle	0.0264
Turkmenistan	1990	Fracture of hand (wrist and other distal part of hand)	0.0138
Turkmenistan	1995	Fracture of hand (wrist and other distal part of hand)	0.0138
Turkmenistan	2000	Fracture of hand (wrist and other distal part of hand)	0.0138
Turkmenistan	2005	Fracture of hand (wrist and other distal part of hand)	0.0138
Turkmenistan	2010	Fracture of hand (wrist and other distal part of hand)	0.0138
Turkmenistan	2017	Fracture of hand (wrist and other distal part of hand)	0.0138
Turkmenistan	1990	Fracture of hip	0.1899
Turkmenistan	1995	Fracture of hip	0.1825
Turkmenistan	2000	Fracture of hip	0.1743
Turkmenistan	2005	Fracture of hip	0.163
Turkmenistan	2010	Fracture of hip	0.143
Turkmenistan	2017	Fracture of hip	0.1142
Turkmenistan	1990	Amputation of upper limbs, bilateral	0.2241
Turkmenistan	1995	Amputation of upper limbs, bilateral	0.2165
Turkmenistan	2000	Amputation of upper limbs, bilateral	0.2081
Turkmenistan	2005	Amputation of upper limbs, bilateral	0.1964
Turkmenistan	2010	Amputation of upper limbs, bilateral	0.1757
Turkmenistan	2017	Amputation of upper limbs, bilateral	0.146
Turkmenistan	1990	Fracture of patella, tibia or fibula, or ankle	0.0554
Turkmenistan	1995	Fracture of patella, tibia or fibula, or ankle	0.0554
Turkmenistan	2000	Fracture of patella, tibia or fibula, or ankle	0.0554
Turkmenistan	2005	Fracture of patella, tibia or fibula, or ankle	0.0554
Turkmenistan	2010	Fracture of patella, tibia or fibula, or ankle	0.0554
Turkmenistan	2017	Fracture of patella, tibia or fibula, or ankle	0.0554
Turkmenistan	1990	Fracture of pelvis	0.1823
Turkmenistan	1995	Fracture of pelvis	0.1823
Turkmenistan	2000	Fracture of pelvis	0.1823
Turkmenistan	2005	Fracture of pelvis	0.1823
Turkmenistan	2010	Fracture of pelvis	0.1823
Turkmenistan	2017	Fracture of pelvis	0.1823
Turkmenistan	1990	Fracture of radius and/or ulna	0.0435
Turkmenistan	1995	Fracture of radius and/or ulna	0.0435
Turkmenistan	2000	Fracture of radius and/or ulna	0.0435
Turkmenistan	2005	Fracture of radius and/or ulna	0.0435
Turkmenistan	2010	Fracture of radius and/or ulna	0.0435
Turkmenistan	2017	Fracture of radius and/or ulna	0.0435
Turkmenistan	1990	Fracture of skull	0.0714
Turkmenistan	1995	Fracture of skull	0.0714
Turkmenistan	2000	Fracture of skull	0.0714
Turkmenistan	2005	Fracture of skull	0.0714
Turkmenistan	2010	Fracture of skull	0.0714
Turkmenistan	2017	Fracture of skull	0.0714
Turkmenistan	1990	Fracture of sternum and/or fracture of one or more ribs	0.1027
Turkmenistan	1995	Fracture of sternum and/or fracture of one or more ribs	0.1027
Turkmenistan	2000	Fracture of sternum and/or fracture of one or more ribs	0.1027
Turkmenistan	2005	Fracture of sternum and/or fracture of one or more ribs	0.1027
Turkmenistan	2010	Fracture of sternum and/or fracture of one or more ribs	0.1027
Turkmenistan	2017	Fracture of sternum and/or fracture of one or more ribs	0.1027

Turkmenistan	1990	Fracture of vertebral column	0.1106
Turkmenistan	1995	Fracture of vertebral column	0.1106
Turkmenistan	2000	Fracture of vertebral column	0.1106
Turkmenistan	2005	Fracture of vertebral column	0.1106
Turkmenistan	2010	Fracture of vertebral column	0.1106
Turkmenistan	2017	Fracture of vertebral column	0.1106
Turkmenistan	1990	Fracture of femur, other than femoral neck	0.042
Turkmenistan	1995	Fracture of femur, other than femoral neck	0.042
Turkmenistan	2000	Fracture of femur, other than femoral neck	0.042
Turkmenistan	2005	Fracture of femur, other than femoral neck	0.042
Turkmenistan	2010	Fracture of femur, other than femoral neck	0.042
Turkmenistan	2017	Fracture of femur, other than femoral neck	0.042
Turkmenistan	1990	Minor TBI	0.1319
Turkmenistan	1995	Minor TBI	0.1319
Turkmenistan	2000	Minor TBI	0.1319
Turkmenistan	2005	Minor TBI	0.1319
Turkmenistan	2010	Minor TBI	0.1319
Turkmenistan	2017	Minor TBI	0.1319
Turkmenistan	1990	Moderate TBI	0.1637
Turkmenistan	1995	Moderate TBI	0.1637
Turkmenistan	2000	Moderate TBI	0.1637
Turkmenistan	2005	Moderate TBI	0.1637
Turkmenistan	2010	Moderate TBI	0.1637
Turkmenistan	2017	Moderate TBI	0.1637
Turkmenistan	1990	Amputation of fingers (excluding thumb)	0.0047
Turkmenistan	1995	Amputation of fingers (excluding thumb)	0.0047
Turkmenistan	2000	Amputation of fingers (excluding thumb)	0.0047
Turkmenistan	2005	Amputation of fingers (excluding thumb)	0.0047
Turkmenistan	2010	Amputation of fingers (excluding thumb)	0.0047
Turkmenistan	2017	Amputation of fingers (excluding thumb)	0.0047
Turkmenistan	1990	Spinal cord lesion at neck level	0.4575
Turkmenistan	1995	Spinal cord lesion at neck level	0.455
Turkmenistan	2000	Spinal cord lesion at neck level	0.4522
Turkmenistan	2005	Spinal cord lesion at neck level	0.4482
Turkmenistan	2010	Spinal cord lesion at neck level	0.4413
Turkmenistan	2017	Spinal cord lesion at neck level	0.4313
Turkmenistan	1990	Spinal cord lesion below neck level	0.2742
Turkmenistan	1995	Spinal cord lesion below neck level	0.2688
Turkmenistan	2000	Spinal cord lesion below neck level	0.2628
Turkmenistan	2005	Spinal cord lesion below neck level	0.2545
Turkmenistan	2010	Spinal cord lesion below neck level	0.2399
Turkmenistan	2017	Spinal cord lesion below neck level	0.2189
Turkmenistan	1990	Drowning and nonfatal submersion	0.2471
Turkmenistan	1995	Drowning and nonfatal submersion	0.2471
Turkmenistan	2000	Drowning and nonfatal submersion	0.2471
Turkmenistan	2005	Drowning and nonfatal submersion	0.2471
Turkmenistan	2010	Drowning and nonfatal submersion	0.2471
Turkmenistan	2017	Drowning and nonfatal submersion	0.2471
Turkmenistan	1990	Asphyxiation	0.2471
Turkmenistan	1995	Asphyxiation	0.2471
Turkmenistan	2000	Asphyxiation	0.2471
Turkmenistan	2005	Asphyxiation	0.2471
Turkmenistan	2010	Asphyxiation	0.2471
Turkmenistan	2017	Asphyxiation	0.2471
Turkmenistan	1990	Crush injury	0.1325
Turkmenistan	1995	Crush injury	0.1325
Turkmenistan	2000	Crush injury	0.1325
Turkmenistan	2005	Crush injury	0.1325
Turkmenistan	2010	Crush injury	0.1325
Turkmenistan	2017	Crush injury	0.1325
Turkmenistan	1990	Nerve injury	0.1129
Turkmenistan	1995	Nerve injury	0.1129
Turkmenistan	2000	Nerve injury	0.1129
Turkmenistan	2005	Nerve injury	0.1129
Turkmenistan	2010	Nerve injury	0.1129
Turkmenistan	2017	Nerve injury	0.1129
Turkmenistan	1990	Injury to eyes	0.0165
Turkmenistan	1995	Injury to eyes	0.0165
Turkmenistan	2000	Injury to eyes	0.0165
Turkmenistan	2005	Injury to eyes	0.0165
Turkmenistan	2010	Injury to eyes	0.0165
Turkmenistan	2017	Injury to eyes	0.0165
Turkmenistan	1990	Amputation of lower limb, unilateral	0.0905
Turkmenistan	1995	Amputation of lower limb, unilateral	0.0876
Turkmenistan	2000	Amputation of lower limb, unilateral	0.0844
Turkmenistan	2005	Amputation of lower limb, unilateral	0.08
Turkmenistan	2010	Amputation of lower limb, unilateral	0.0722
Turkmenistan	2017	Amputation of lower limb, unilateral	0.061
Turkmenistan	1990	Open wound(s)	0.011
Turkmenistan	1995	Open wound(s)	0.011
Turkmenistan	2000	Open wound(s)	0.011
Turkmenistan	2005	Open wound(s)	0.011
Turkmenistan	2010	Open wound(s)	0.011
Turkmenistan	2017	Open wound(s)	0.011
Turkmenistan	1990	Poisoning requiring urgent care	0.1628
Turkmenistan	1995	Poisoning requiring urgent care	0.1628
Turkmenistan	2000	Poisoning requiring urgent care	0.1628
Turkmenistan	2005	Poisoning requiring urgent care	0.1628
Turkmenistan	2010	Poisoning requiring urgent care	0.1628
Turkmenistan	2017	Poisoning requiring urgent care	0.1628
Turkmenistan	1990	Severe chest injury	0.0474
Turkmenistan	1995	Severe chest injury	0.0474
Turkmenistan	2000	Severe chest injury	0.0474
Turkmenistan	2005	Severe chest injury	0.0474
Turkmenistan	2010	Severe chest injury	0.0474
Turkmenistan	2017	Severe chest injury	0.0474
Turkmenistan	1990	Internal hemorrhage in abdomen and pelvis	0.1143
Turkmenistan	1995	Internal hemorrhage in abdomen and pelvis	0.1143
Turkmenistan	2000	Internal hemorrhage in abdomen and pelvis	0.1143
Turkmenistan	2005	Internal hemorrhage in abdomen and pelvis	0.1143
Turkmenistan	2010	Internal hemorrhage in abdomen and pelvis	0.1143
Turkmenistan	2017	Internal hemorrhage in abdomen and pelvis	0.1143
Turkmenistan	1990	Contusion in any part of the body	0.0075
Turkmenistan	1995	Contusion in any part of the body	0.0075
Turkmenistan	2000	Contusion in any part of the body	0.0075
Turkmenistan	2005	Contusion in any part of the body	0.0075
Turkmenistan	2010	Contusion in any part of the body	0.0075
Turkmenistan	2017	Contusion in any part of the body	0.0075
Turkmenistan	1990	Effect of different environmental factors	0.049
Turkmenistan	1995	Effect of different environmental factors	0.049
Turkmenistan	2000	Effect of different environmental factors	0.049
Turkmenistan	2005	Effect of different environmental factors	0.049
Turkmenistan	2010	Effect of different environmental factors	0.049
Turkmenistan	2017	Effect of different environmental factors	0.049
Turkmenistan	1990	Complications following therapeutic procedures	0.0506
Turkmenistan	1995	Complications following therapeutic procedures	0.0506
Turkmenistan	2000	Complications following therapeutic procedures	0.0506
Turkmenistan	2005	Complications following therapeutic procedures	0.0506
Turkmenistan	2010	Complications following therapeutic procedures	0.0506
Turkmenistan	2017	Complications following therapeutic procedures	0.0506
Turkmenistan	1990	Multiple fractures, dislocations, crashes, wounds, sprains, and strains	0.1899
Turkmenistan	1995	Multiple fractures, dislocations, crashes, wounds, sprains, and strains	0.1825
Turkmenistan	2000	Multiple fractures, dislocations, crashes, wounds, sprains, and strains	0.1743
Turkmenistan	2005	Multiple fractures, dislocations, crashes, wounds, sprains, and strains	0.163
Turkmenistan	2010	Multiple fractures, dislocations, crashes, wounds, sprains, and strains	0.143
Turkmenistan	2017	Multiple fractures, dislocations, crashes, wounds, sprains, and strains	0.1142
Turkmenistan	1990	Amputation of upper limb, unilateral	0.0693
Turkmenistan	1995	Amputation of upper limb, unilateral	0.0676
Turkmenistan	2000	Amputation of upper limb, unilateral	0.0657
Turkmenistan	2005	Amputation of upper limb, unilateral	0.0631
Turkmenistan	2010	Amputation of upper limb, unilateral	0.0586
Turkmenistan	2017	Amputation of upper limb, unilateral	0.0519
Turkmenistan	1990	Amputation of thumb	0.0114
Turkmenistan	1995	Amputation of thumb	0.0114
Turkmenistan	2000	Amputation of thumb	0.0114
Turkmenistan	2005	Amputation of thumb	0.0114
Turkmenistan	2010	Amputation of thumb	0.0114
Turkmenistan	2017	Amputation of thumb	0.0114
Turkmenistan	1990	Amputation of toe/toes	0.0059
Turkmenistan	1995	Amputation of toe/toes	0.0059
Turkmenistan	2000	Amputation of toe/toes	0.0059
Turkmenistan	2005	Amputation of toe/toes	0.0059
Turkmenistan	2010	Amputation of toe/toes	0.0059
Turkmenistan	2017	Amputation of toe/toes	0.0059
Turkmenistan	1990	Burns, <20% total burned surface area without lower airway burns	0.0163
Turkmenistan	1995	Burns, <20% total burned surface area without lower airway burns	0.0163
Turkmenistan	2000	Burns, <20% total burned surface area without lower airway burns	0.0163
Turkmenistan	2005	Burns, <20% total burned surface area without lower airway burns	0.0163
Turkmenistan	2010	Burns, <20% total burned surface area without lower airway burns	0.0163
Turkmenistan	2017	Burns, <20% total burned surface area without lower airway burns	0.0163
Turkmenistan	1990	Burns, >=20% total burned surface area or >= 10% burned surface area if head/neck or hands/wrist involved w/o lower airway burns	0.2577
Turkmenistan	1995	Burns, >=20% total burned surface area or >= 10% burned surface area if head/neck or hands/wrist involved w/o lower airway burns	0.2508
Turkmenistan	2000	Burns, >=20% total burned surface area or >= 10% burned surface area if head/neck or hands/wrist involved w/o lower airway burns	0.2432
Turkmenistan	2005	Burns, >=20% total burned surface area or >= 10% burned surface area if head/neck or hands/wrist involved w/o lower airway burns	0.2327
Turkmenistan	2010	Burns, >=20% total burned surface area or >= 10% burned surface area if head/neck or hands/wrist involved w/o lower airway burns	0.214
Turkmenistan	2017	Burns, >=20% total burned surface area or >= 10% burned surface area if head/neck or hands/wrist involved w/o lower airway burns	0.1872
Uzbekistan	1990	Amputation of lower limbs, bilateral	0.2064
Uzbekistan	1995	Amputation of lower limbs, bilateral	0.2051
Uzbekistan	2000	Amputation of lower limbs, bilateral	0.1979
Uzbekistan	2005	Amputation of lower limbs, bilateral	0.1887
Uzbekistan	2010	Amputation of lower limbs, bilateral	0.1758
Uzbekistan	2017	Amputation of lower limbs, bilateral	0.1623
Uzbekistan	1990	Lower airway burns	0.2252
Uzbekistan	1995	Lower airway burns	0.2252
Uzbekistan	2000	Lower airway burns	0.2252
Uzbekistan	2005	Lower airway burns	0.2252
Uzbekistan	2010	Lower airway burns	0.2252
Uzbekistan	2017	Lower airway burns	0.2252
Uzbekistan	1990	Dislocation of hip	0.0159
Uzbekistan	1995	Dislocation of hip	0.0159
Uzbekistan	2000	Dislocation of hip	0.0159
Uzbekistan	2005	Dislocation of hip	0.0159
Uzbekistan	2010	Dislocation of hip	0.0159
Uzbekistan	2017	Dislocation of hip	0.0159
Uzbekistan	1990	Dislocation of knee	0.1134
Uzbekistan	1995	Dislocation of knee	0.1134
Uzbekistan	2000	Dislocation of knee	0.1134
Uzbekistan	2005	Dislocation of knee	0.1134
Uzbekistan	2010	Dislocation of knee	0.1134
Uzbekistan	2017	Dislocation of knee	0.1134
Uzbekistan	1990	Dislocation of shoulder	0.062
Uzbekistan	1995	Dislocation of shoulder	0.062
Uzbekistan	2000	Dislocation of shoulder	0.062
Uzbekistan	2005	Dislocation of shoulder	0.062
Uzbekistan	2010	Dislocation of shoulder	0.062
Uzbekistan	2017	Dislocation of shoulder	0.062
Uzbekistan	1990	Muscle and tendon injuries, including sprains and strains lesser dislocations	0.0075
Uzbekistan	1995	Muscle and tendon injuries, including sprains and strains lesser dislocations	0.0075
Uzbekistan	2000	Muscle and tendon injuries, including sprains and strains lesser dislocations	0.0075
Uzbekistan	2005	Muscle and tendon injuries, including sprains and strains lesser dislocations	0.0075
Uzbekistan	2010	Muscle and tendon injuries, including sprains and strains lesser dislocations	0.0075
Uzbekistan	2017	Muscle and tendon injuries, including sprains and strains lesser dislocations	0.0075
Uzbekistan	1990	Fracture of clavicle, scapula, or humerus	0.0349
Uzbekistan	1995	Fracture of clavicle, scapula, or humerus	0.0349
Uzbekistan	2000	Fracture of clavicle, scapula, or humerus	0.0349
Uzbekistan	2005	Fracture of clavicle, scapula, or humerus	0.0349
Uzbekistan	2010	Fracture of clavicle, scapula, or humerus	0.0349
Uzbekistan	2017	Fracture of clavicle, scapula, or humerus	0.0349
Uzbekistan	1990	Fracture of face bones	0.0669
Uzbekistan	1995	Fracture of face bones	0.0669
Uzbekistan	2000	Fracture of face bones	0.0669
Uzbekistan	2005	Fracture of face bones	0.0669
Uzbekistan	2010	Fracture of face bones	0.0669
Uzbekistan	2017	Fracture of face bones	0.0669
Uzbekistan	1990	Fracture of foot bones except ankle	0.0264
Uzbekistan	1995	Fracture of foot bones except ankle	0.0264
Uzbekistan	2000	Fracture of foot bones except ankle	0.0264
Uzbekistan	2005	Fracture of foot bones except ankle	0.0264
Uzbekistan	2010	Fracture of foot bones except ankle	0.0264
Uzbekistan	2017	Fracture of foot bones except ankle	0.0264
Uzbekistan	1990	Fracture of hand (wrist and other distal part of hand)	0.0138
Uzbekistan	1995	Fracture of hand (wrist and other distal part of hand)	0.0138
Uzbekistan	2000	Fracture of hand (wrist and other distal part of hand)	0.0138
Uzbekistan	2005	Fracture of hand (wrist and other distal part of hand)	0.0138
Uzbekistan	2010	Fracture of hand (wrist and other distal part of hand)	0.0138
Uzbekistan	2017	Fracture of hand (wrist and other distal part of hand)	0.0138
Uzbekistan	1990	Fracture of hip	0.1677
Uzbekistan	1995	Fracture of hip	0.166
Uzbekistan	2000	Fracture of hip	0.1564
Uzbekistan	2005	Fracture of hip	0.1442
Uzbekistan	2010	Fracture of hip	0.1272
Uzbekistan	2017	Fracture of hip	0.1093
Uzbekistan	1990	Amputation of upper limbs, bilateral	0.2012
Uzbekistan	1995	Amputation of upper limbs, bilateral	0.1995
Uzbekistan	2000	Amputation of upper limbs, bilateral	0.1896
Uzbekistan	2005	Amputation of upper limbs, bilateral	0.177
Uzbekistan	2010	Amputation of upper limbs, bilateral	0.1584
Uzbekistan	2017	Amputation of upper limbs, bilateral	0.1409
Uzbekistan	1990	Fracture of patella, tibia or fibula, or ankle	0.0554
Uzbekistan	1995	Fracture of patella, tibia or fibula, or ankle	0.0554
Uzbekistan	2000	Fracture of patella, tibia or fibula, or ankle	0.0554
Uzbekistan	2005	Fracture of patella, tibia or fibula, or ankle	0.0554
Uzbekistan	2010	Fracture of patella, tibia or fibula, or ankle	0.0554
Uzbekistan	2017	Fracture of patella, tibia or fibula, or ankle	0.0554
Uzbekistan	1990	Fracture of pelvis	0.1823
Uzbekistan	1995	Fracture of pelvis	0.1823
Uzbekistan	2000	Fracture of pelvis	0.1823
Uzbekistan	2005	Fracture of pelvis	0.1823
Uzbekistan	2010	Fracture of pelvis	0.1823
Uzbekistan	2017	Fracture of pelvis	0.1823
Uzbekistan	1990	Fracture of radius and/or ulna	0.0435
Uzbekistan	1995	Fracture of radius and/or ulna	0.0435
Uzbekistan	2000	Fracture of radius and/or ulna	0.0435
Uzbekistan	2005	Fracture of radius and/or ulna	0.0435
Uzbekistan	2010	Fracture of radius and/or ulna	0.0435
Uzbekistan	2017	Fracture of radius and/or ulna	0.0435
Uzbekistan	1990	Fracture of skull	0.0714
Uzbekistan	1995	Fracture of skull	0.0714
Uzbekistan	2000	Fracture of skull	0.0714
Uzbekistan	2005	Fracture of skull	0.0714
Uzbekistan	2010	Fracture of skull	0.0714
Uzbekistan	2017	Fracture of skull	0.0714
Uzbekistan	1990	Fracture of sternum and/or fracture of one or more ribs	0.1027
Uzbekistan	1995	Fracture of sternum and/or fracture of one or more ribs	0.1027
Uzbekistan	2000	Fracture of sternum and/or fracture of one or more ribs	0.1027
Uzbekistan	2005	Fracture of sternum and/or fracture of one or more ribs	0.1027
Uzbekistan	2010	Fracture of sternum and/or fracture of one or more ribs	0.1027
Uzbekistan	2017	Fracture of sternum and/or fracture of one or more ribs	0.1027
Uzbekistan	1990	Fracture of vertebral column	0.1106
Uzbekistan	1995	Fracture of vertebral column	0.1106
Uzbekistan	2000	Fracture of vertebral column	0.1106
Uzbekistan	2005	Fracture of vertebral column	0.1106
Uzbekistan	2010	Fracture of vertebral column	0.1106
Uzbekistan	2017	Fracture of vertebral column	0.1106
Uzbekistan	1990	Fracture of femur, other than femoral neck	0.042
Uzbekistan	1995	Fracture of femur, other than femoral neck	0.042
Uzbekistan	2000	Fracture of femur, other than femoral neck	0.042
Uzbekistan	2005	Fracture of femur, other than femoral neck	0.042
Uzbekistan	2010	Fracture of femur, other than femoral neck	0.042
Uzbekistan	2017	Fracture of femur, other than femoral neck	0.042
Uzbekistan	1990	Minor TBI	0.1319
Uzbekistan	1995	Minor TBI	0.1319
Uzbekistan	2000	Minor TBI	0.1319
Uzbekistan	2005	Minor TBI	0.1319
Uzbekistan	2010	Minor TBI	0.1319
Uzbekistan	2017	Minor TBI	0.1319
Uzbekistan	1990	Moderate TBI	0.1637
Uzbekistan	1995	Moderate TBI	0.1637
Uzbekistan	2000	Moderate TBI	0.1637
Uzbekistan	2005	Moderate TBI	0.1637
Uzbekistan	2010	Moderate TBI	0.1637
Uzbekistan	2017	Moderate TBI	0.1637
Uzbekistan	1990	Amputation of fingers (excluding thumb)	0.0047
Uzbekistan	1995	Amputation of fingers (excluding thumb)	0.0047
Uzbekistan	2000	Amputation of fingers (excluding thumb)	0.0047
Uzbekistan	2005	Amputation of fingers (excluding thumb)	0.0047
Uzbekistan	2010	Amputation of fingers (excluding thumb)	0.0047
Uzbekistan	2017	Amputation of fingers (excluding thumb)	0.0047
Uzbekistan	1990	Spinal cord lesion at neck level	0.4499
Uzbekistan	1995	Spinal cord lesion at neck level	0.4493
Uzbekistan	2000	Spinal cord lesion at neck level	0.4459
Uzbekistan	2005	Spinal cord lesion at neck level	0.4418
Uzbekistan	2010	Spinal cord lesion at neck level	0.4359
Uzbekistan	2017	Spinal cord lesion at neck level	0.4297
Uzbekistan	1990	Spinal cord lesion below neck level	0.2579
Uzbekistan	1995	Spinal cord lesion below neck level	0.2567
Uzbekistan	2000	Spinal cord lesion below neck level	0.2497
Uzbekistan	2005	Spinal cord lesion below neck level	0.2409
Uzbekistan	2010	Spinal cord lesion below neck level	0.2284
Uzbekistan	2017	Spinal cord lesion below neck level	0.2153
Uzbekistan	1990	Drowning and nonfatal submersion	0.2471
Uzbekistan	1995	Drowning and nonfatal submersion	0.2471
Uzbekistan	2000	Drowning and nonfatal submersion	0.2471
Uzbekistan	2005	Drowning and nonfatal submersion	0.2471
Uzbekistan	2010	Drowning and nonfatal submersion	0.2471
Uzbekistan	2017	Drowning and nonfatal submersion	0.2471
Uzbekistan	1990	Asphyxiation	0.2471
Uzbekistan	1995	Asphyxiation	0.2471
Uzbekistan	2000	Asphyxiation	0.2471
Uzbekistan	2005	Asphyxiation	0.2471
Uzbekistan	2010	Asphyxiation	0.2471
Uzbekistan	2017	Asphyxiation	0.2471
Uzbekistan	1990	Crush injury	0.1325
Uzbekistan	1995	Crush injury	0.1325
Uzbekistan	2000	Crush injury	0.1325
Uzbekistan	2005	Crush injury	0.1325
Uzbekistan	2010	Crush injury	0.1325
Uzbekistan	2017	Crush injury	0.1325
Uzbekistan	1990	Nerve injury	0.1129
Uzbekistan	1995	Nerve injury	0.1129
Uzbekistan	2000	Nerve injury	0.1129
Uzbekistan	2005	Nerve injury	0.1129
Uzbekistan	2010	Nerve injury	0.1129
Uzbekistan	2017	Nerve injury	0.1129
Uzbekistan	1990	Injury to eyes	0.0165
Uzbekistan	1995	Injury to eyes	0.0165
Uzbekistan	2000	Injury to eyes	0.0165
Uzbekistan	2005	Injury to eyes	0.0165
Uzbekistan	2010	Injury to eyes	0.0165
Uzbekistan	2017	Injury to eyes	0.0165
Uzbekistan	1990	Amputation of lower limb, unilateral	0.0818
Uzbekistan	1995	Amputation of lower limb, unilateral	0.0812
Uzbekistan	2000	Amputation of lower limb, unilateral	0.0774
Uzbekistan	2005	Amputation of lower limb, unilateral	0.0727
Uzbekistan	2010	Amputation of lower limb, unilateral	0.066
Uzbekistan	2017	Amputation of lower limb, unilateral	0.0591
Uzbekistan	1990	Open wound(s)	0.011
Uzbekistan	1995	Open wound(s)	0.011
Uzbekistan	2000	Open wound(s)	0.011
Uzbekistan	2005	Open wound(s)	0.011
Uzbekistan	2010	Open wound(s)	0.011
Uzbekistan	2017	Open wound(s)	0.011
Uzbekistan	1990	Poisoning requiring urgent care	0.1628
Uzbekistan	1995	Poisoning requiring urgent care	0.1628
Uzbekistan	2000	Poisoning requiring urgent care	0.1628
Uzbekistan	2005	Poisoning requiring urgent care	0.1628
Uzbekistan	2010	Poisoning requiring urgent care	0.1628
Uzbekistan	2017	Poisoning requiring urgent care	0.1628
Uzbekistan	1990	Severe chest injury	0.0474
Uzbekistan	1995	Severe chest injury	0.0474
Uzbekistan	2000	Severe chest injury	0.0474
Uzbekistan	2005	Severe chest injury	0.0474
Uzbekistan	2010	Severe chest injury	0.0474
Uzbekistan	2017	Severe chest injury	0.0474
Uzbekistan	1990	Internal hemorrhage in abdomen and pelvis	0.1143
Uzbekistan	1995	Internal hemorrhage in abdomen and pelvis	0.1143
Uzbekistan	2000	Internal hemorrhage in abdomen and pelvis	0.1143
Uzbekistan	2005	Internal hemorrhage in abdomen and pelvis	0.1143
Uzbekistan	2010	Internal hemorrhage in abdomen and pelvis	0.1143
Uzbekistan	2017	Internal hemorrhage in abdomen and pelvis	0.1143
Uzbekistan	1990	Contusion in any part of the body	0.0075
Uzbekistan	1995	Contusion in any part of the body	0.0075
Uzbekistan	2000	Contusion in any part of the body	0.0075
Uzbekistan	2005	Contusion in any part of the body	0.0075
Uzbekistan	2010	Contusion in any part of the body	0.0075
Uzbekistan	2017	Contusion in any part of the body	0.0075
Uzbekistan	1990	Effect of different environmental factors	0.049
Uzbekistan	1995	Effect of different environmental factors	0.049
Uzbekistan	2000	Effect of different environmental factors	0.049
Uzbekistan	2005	Effect of different environmental factors	0.049
Uzbekistan	2010	Effect of different environmental factors	0.049
Uzbekistan	2017	Effect of different environmental factors	0.049
Uzbekistan	1990	Complications following therapeutic procedures	0.0506
Uzbekistan	1995	Complications following therapeutic procedures	0.0506
Uzbekistan	2000	Complications following therapeutic procedures	0.0506
Uzbekistan	2005	Complications following therapeutic procedures	0.0506
Uzbekistan	2010	Complications following therapeutic procedures	0.0506
Uzbekistan	2017	Complications following therapeutic procedures	0.0506
Uzbekistan	1990	Multiple fractures, dislocations, crashes, wounds, sprains, and strains	0.1677
Uzbekistan	1995	Multiple fractures, dislocations, crashes, wounds, sprains, and strains	0.166
Uzbekistan	2000	Multiple fractures, dislocations, crashes, wounds, sprains, and strains	0.1564
Uzbekistan	2005	Multiple fractures, dislocations, crashes, wounds, sprains, and strains	0.1442
Uzbekistan	2010	Multiple fractures, dislocations, crashes, wounds, sprains, and strains	0.1272
Uzbekistan	2017	Multiple fractures, dislocations, crashes, wounds, sprains, and strains	0.1093
Uzbekistan	1990	Amputation of upper limb, unilateral	0.0642
Uzbekistan	1995	Amputation of upper limb, unilateral	0.0638
Uzbekistan	2000	Amputation of upper limb, unilateral	0.0616
Uzbekistan	2005	Amputation of upper limb, unilateral	0.0588
Uzbekistan	2010	Amputation of upper limb, unilateral	0.0549
Uzbekistan	2017	Amputation of upper limb, unilateral	0.0508
Uzbekistan	1990	Amputation of thumb	0.0114
Uzbekistan	1995	Amputation of thumb	0.0114
Uzbekistan	2000	Amputation of thumb	0.0114
Uzbekistan	2005	Amputation of thumb	0.0114
Uzbekistan	2010	Amputation of thumb	0.0114
Uzbekistan	2017	Amputation of thumb	0.0114
Uzbekistan	1990	Amputation of toe/toes	0.0059
Uzbekistan	1995	Amputation of toe/toes	0.0059
Uzbekistan	2000	Amputation of toe/toes	0.0059
Uzbekistan	2005	Amputation of toe/toes	0.0059
Uzbekistan	2010	Amputation of toe/toes	0.0059
Uzbekistan	2017	Amputation of toe/toes	0.0059
Uzbekistan	1990	Burns, <20% total burned surface area without lower airway burns	0.0163
Uzbekistan	1995	Burns, <20% total burned surface area without lower airway burns	0.0163
Uzbekistan	2000	Burns, <20% total burned surface area without lower airway burns	0.0163
Uzbekistan	2005	Burns, <20% total burned surface area without lower airway burns	0.0163
Uzbekistan	2010	Burns, <20% total burned surface area without lower airway burns	0.0163
Uzbekistan	2017	Burns, <20% total burned surface area without lower airway burns	0.0163
Uzbekistan	1990	Burns, >=20% total burned surface area or >= 10% burned surface area if head/neck or hands/wrist involved w/o lower airway burns	0.237
Uzbekistan	1995	Burns, >=20% total burned surface area or >= 10% burned surface area if head/neck or hands/wrist involved w/o lower airway burns	0.2354
Uzbekistan	2000	Burns, >=20% total burned surface area or >= 10% burned surface area if head/neck or hands/wrist involved w/o lower airway burns	0.2265
Uzbekistan	2005	Burns, >=20% total burned surface area or >= 10% burned surface area if head/neck or hands/wrist involved w/o lower airway burns	0.2152
Uzbekistan	2010	Burns, >=20% total burned surface area or >= 10% burned surface area if head/neck or hands/wrist involved w/o lower airway burns	0.1993
Uzbekistan	2017	Burns, >=20% total burned surface area or >= 10% burned surface area if head/neck or hands/wrist involved w/o lower airway burns	0.1826
Albania	1990	Amputation of lower limbs, bilateral	0.1911
Albania	1995	Amputation of lower limbs, bilateral	0.1729
Albania	2000	Amputation of lower limbs, bilateral	0.1615
Albania	2005	Amputation of lower limbs, bilateral	0.1476
Albania	2010	Amputation of lower limbs, bilateral	0.1324
Albania	2017	Amputation of lower limbs, bilateral	0.1233
Albania	1990	Lower airway burns	0.2252
Albania	1995	Lower airway burns	0.2252
Albania	2000	Lower airway burns	0.2252
Albania	2005	Lower airway burns	0.2252
Albania	2010	Lower airway burns	0.2252
Albania	2017	Lower airway burns	0.2252
Albania	1990	Dislocation of hip	0.0159
Albania	1995	Dislocation of hip	0.0159
Albania	2000	Dislocation of hip	0.0159
Albania	2005	Dislocation of hip	0.0159
Albania	2010	Dislocation of hip	0.0159

Albania	2017	Dislocation of hip	0.0159
Albania	1990	Dislocation of knee	0.1134
Albania	1995	Dislocation of knee	0.1134
Albania	2000	Dislocation of knee	0.1134
Albania	2005	Dislocation of knee	0.1134
Albania	2010	Dislocation of knee	0.1134
Albania	2017	Dislocation of knee	0.1134
Albania	1990	Dislocation of shoulder	0.062
Albania	1995	Dislocation of shoulder	0.062
Albania	2000	Dislocation of shoulder	0.062
Albania	2005	Dislocation of shoulder	0.062
Albania	2010	Dislocation of shoulder	0.062
Albania	2017	Dislocation of shoulder	0.062
Albania	1990	Muscle and tendon injuries, including sprains and strains lesser dislocations	0.0075
Albania	1995	Muscle and tendon injuries, including sprains and strains lesser dislocations	0.0075
Albania	2000	Muscle and tendon injuries, including sprains and strains lesser dislocations	0.0075
Albania	2005	Muscle and tendon injuries, including sprains and strains lesser dislocations	0.0075
Albania	2010	Muscle and tendon injuries, including sprains and strains lesser dislocations	0.0075
Albania	2017	Muscle and tendon injuries, including sprains and strains lesser dislocations	0.0075
Albania	1990	Fracture of clavicle, scapula, or humerus	0.0349
Albania	1995	Fracture of clavicle, scapula, or humerus	0.0349
Albania	2000	Fracture of clavicle, scapula, or humerus	0.0349
Albania	2005	Fracture of clavicle, scapula, or humerus	0.0349
Albania	2010	Fracture of clavicle, scapula, or humerus	0.0349
Albania	2017	Fracture of clavicle, scapula, or humerus	0.0349
Albania	1990	Fracture of face bones	0.0669
Albania	1995	Fracture of face bones	0.0669
Albania	2000	Fracture of face bones	0.0669
Albania	2005	Fracture of face bones	0.0669
Albania	2010	Fracture of face bones	0.0669
Albania	2017	Fracture of face bones	0.0669
Albania	1990	Fracture of foot bones except ankle	0.0264
Albania	1995	Fracture of foot bones except ankle	0.0264
Albania	2000	Fracture of foot bones except ankle	0.0264
Albania	2005	Fracture of foot bones except ankle	0.0264
Albania	2010	Fracture of foot bones except ankle	0.0264
Albania	2017	Fracture of foot bones except ankle	0.0264
Albania	1990	Fracture of hand (wrist and other distal part of hand)	0.0138
Albania	1995	Fracture of hand (wrist and other distal part of hand)	0.0138
Albania	2000	Fracture of hand (wrist and other distal part of hand)	0.0138
Albania	2005	Fracture of hand (wrist and other distal part of hand)	0.0138
Albania	2010	Fracture of hand (wrist and other distal part of hand)	0.0138
Albania	2017	Fracture of hand (wrist and other distal part of hand)	0.0138
Albania	1990	Fracture of hip	0.1474
Albania	1995	Fracture of hip	0.1234
Albania	2000	Fracture of hip	0.1083
Albania	2005	Fracture of hip	0.0899
Albania	2010	Fracture of hip	0.0698
Albania	2017	Fracture of hip	0.0577
Albania	1990	Amputation of upper limbs, bilateral	0.1803
Albania	1995	Amputation of upper limbs, bilateral	0.1555
Albania	2000	Amputation of upper limbs, bilateral	0.1399
Albania	2005	Amputation of upper limbs, bilateral	0.1209
Albania	2010	Amputation of upper limbs, bilateral	0.1002
Albania	2017	Amputation of upper limbs, bilateral	0.0877
Albania	1990	Fracture of patella, tibia or fibula, or ankle	0.0554
Albania	1995	Fracture of patella, tibia or fibula, or ankle	0.0554
Albania	2000	Fracture of patella, tibia or fibula, or ankle	0.0554
Albania	2005	Fracture of patella, tibia or fibula, or ankle	0.0554
Albania	2010	Fracture of patella, tibia or fibula, or ankle	0.0554
Albania	2017	Fracture of patella, tibia or fibula, or ankle	0.0554
Albania	1990	Fracture of pelvis	0.1823
Albania	1995	Fracture of pelvis	0.1823
Albania	2000	Fracture of pelvis	0.1823
Albania	2005	Fracture of pelvis	0.1823
Albania	2010	Fracture of pelvis	0.1823
Albania	2017	Fracture of pelvis	0.1823
Albania	1990	Fracture of radius and/or ulna	0.0435
Albania	1995	Fracture of radius and/or ulna	0.0435
Albania	2000	Fracture of radius and/or ulna	0.0435
Albania	2005	Fracture of radius and/or ulna	0.0435
Albania	2010	Fracture of radius and/or ulna	0.0435
Albania	2017	Fracture of radius and/or ulna	0.0435
Albania	1990	Fracture of skull	0.0714
Albania	1995	Fracture of skull	0.0714
Albania	2000	Fracture of skull	0.0714
Albania	2005	Fracture of skull	0.0714
Albania	2010	Fracture of skull	0.0714
Albania	2017	Fracture of skull	0.0714
Albania	1990	Fracture of sternum and/or fracture of one or more ribs	0.1027
Albania	1995	Fracture of sternum and/or fracture of one or more ribs	0.1027
Albania	2000	Fracture of sternum and/or fracture of one or more ribs	0.1027
Albania	2005	Fracture of sternum and/or fracture of one or more ribs	0.1027
Albania	2010	Fracture of sternum and/or fracture of one or more ribs	0.1027
Albania	2017	Fracture of sternum and/or fracture of one or more ribs	0.1027
Albania	1990	Fracture of vertebral column	0.1106
Albania	1995	Fracture of vertebral column	0.1106
Albania	2000	Fracture of vertebral column	0.1106
Albania	2005	Fracture of vertebral column	0.1106
Albania	2010	Fracture of vertebral column	0.1106
Albania	2017	Fracture of vertebral column	0.1106
Albania	1990	Fracture of femur, other than femoral neck	0.042
Albania	1995	Fracture of femur, other than femoral neck	0.042
Albania	2000	Fracture of femur, other than femoral neck	0.042
Albania	2005	Fracture of femur, other than femoral neck	0.042
Albania	2010	Fracture of femur, other than femoral neck	0.042
Albania	2017	Fracture of femur, other than femoral neck	0.042
Albania	1990	Minor TBI	0.1319
Albania	1995	Minor TBI	0.1319
Albania	2000	Minor TBI	0.1319
Albania	2005	Minor TBI	0.1319
Albania	2010	Minor TBI	0.1319
Albania	2017	Minor TBI	0.1319
Albania	1990	Moderate TBI	0.1637
Albania	1995	Moderate TBI	0.1637
Albania	2000	Moderate TBI	0.1637
Albania	2005	Moderate TBI	0.1637
Albania	2010	Moderate TBI	0.1637
Albania	2017	Moderate TBI	0.1637
Albania	1990	Amputation of fingers (excluding thumb)	0.0047
Albania	1995	Amputation of fingers (excluding thumb)	0.0047
Albania	2000	Amputation of fingers (excluding thumb)	0.0047
Albania	2005	Amputation of fingers (excluding thumb)	0.0047
Albania	2010	Amputation of fingers (excluding thumb)	0.0047
Albania	2017	Amputation of fingers (excluding thumb)	0.0047
Albania	1990	Spinal cord lesion at neck level	0.4429
Albania	1995	Spinal cord lesion at neck level	0.4345
Albania	2000	Spinal cord lesion at neck level	0.4293
Albania	2005	Spinal cord lesion at neck level	0.423
Albania	2010	Spinal cord lesion at neck level	0.416
Albania	2017	Spinal cord lesion at neck level	0.4118
Albania	1990	Spinal cord lesion below neck level	0.2432
Albania	1995	Spinal cord lesion below neck level	0.2256
Albania	2000	Spinal cord lesion below neck level	0.2146
Albania	2005	Spinal cord lesion below neck level	0.2012
Albania	2010	Spinal cord lesion below neck level	0.1865
Albania	2017	Spinal cord lesion below neck level	0.1777
Albania	1990	Drowning and nonfatal submersion	0.2471
Albania	1995	Drowning and nonfatal submersion	0.2471
Albania	2000	Drowning and nonfatal submersion	0.2471
Albania	2005	Drowning and nonfatal submersion	0.2471
Albania	2010	Drowning and nonfatal submersion	0.2471
Albania	2017	Drowning and nonfatal submersion	0.2471
Albania	1990	Asphyxiation	0.2471
Albania	1995	Asphyxiation	0.2471
Albania	2000	Asphyxiation	0.2471
Albania	2005	Asphyxiation	0.2471
Albania	2010	Asphyxiation	0.2471
Albania	2017	Asphyxiation	0.2471
Albania	1990	Crush injury	0.1325
Albania	1995	Crush injury	0.1325
Albania	2000	Crush injury	0.1325
Albania	2005	Crush injury	0.1325
Albania	2010	Crush injury	0.1325
Albania	2017	Crush injury	0.1325
Albania	1990	Nerve injury	0.1129
Albania	1995	Nerve injury	0.1129
Albania	2000	Nerve injury	0.1129
Albania	2005	Nerve injury	0.1129
Albania	2010	Nerve injury	0.1129
Albania	2017	Nerve injury	0.1129
Albania	1990	Injury to eyes	0.0165
Albania	1995	Injury to eyes	0.0165
Albania	2000	Injury to eyes	0.0165
Albania	2005	Injury to eyes	0.0165
Albania	2010	Injury to eyes	0.0165
Albania	2017	Injury to eyes	0.0165
Albania	1990	Amputation of lower limb, unilateral	0.0739
Albania	1995	Amputation of lower limb, unilateral	0.0646
Albania	2000	Amputation of lower limb, unilateral	0.0587
Albania	2005	Amputation of lower limb, unilateral	0.0515
Albania	2010	Amputation of lower limb, unilateral	0.0437
Albania	2017	Amputation of lower limb, unilateral	0.039
Albania	1990	Open wound(s)	0.011
Albania	1995	Open wound(s)	0.011
Albania	2000	Open wound(s)	0.011
Albania	2005	Open wound(s)	0.011
Albania	2010	Open wound(s)	0.011
Albania	2017	Open wound(s)	0.011
Albania	1990	Poisoning requiring urgent care	0.1628
Albania	1995	Poisoning requiring urgent care	0.1628
Albania	2000	Poisoning requiring urgent care	0.1628
Albania	2005	Poisoning requiring urgent care	0.1628
Albania	2010	Poisoning requiring urgent care	0.1628
Albania	2017	Poisoning requiring urgent care	0.1628
Albania	1990	Severe chest injury	0.0474
Albania	1995	Severe chest injury	0.0474
Albania	2000	Severe chest injury	0.0474
Albania	2005	Severe chest injury	0.0474
Albania	2010	Severe chest injury	0.0474
Albania	2017	Severe chest injury	0.0474
Albania	1990	Internal hemorrhage in abdomen and pelvis	0.1143
Albania	1995	Internal hemorrhage in abdomen and pelvis	0.1143
Albania	2000	Internal hemorrhage in abdomen and pelvis	0.1143
Albania	2005	Internal hemorrhage in abdomen and pelvis	0.1143
Albania	2010	Internal hemorrhage in abdomen and pelvis	0.1143
Albania	2017	Internal hemorrhage in abdomen and pelvis	0.1143
Albania	1990	Contusion in any part of the body	0.0075
Albania	1995	Contusion in any part of the body	0.0075
Albania	2000	Contusion in any part of the body	0.0075
Albania	2005	Contusion in any part of the body	0.0075
Albania	2010	Contusion in any part of the body	0.0075
Albania	2017	Contusion in any part of the body	0.0075
Albania	1990	Effect of different environmental factors	0.049
Albania	1995	Effect of different environmental factors	0.049
Albania	2000	Effect of different environmental factors	0.049
Albania	2005	Effect of different environmental factors	0.049
Albania	2010	Effect of different environmental factors	0.049
Albania	2017	Effect of different environmental factors	0.049
Albania	1990	Complications following therapeutic procedures	0.0506
Albania	1995	Complications following therapeutic procedures	0.0506
Albania	2000	Complications following therapeutic procedures	0.0506
Albania	2005	Complications following therapeutic procedures	0.0506
Albania	2010	Complications following therapeutic procedures	0.0506
Albania	2017	Complications following therapeutic procedures	0.0506
Albania	1990	Multiple fractures, dislocations, crashes, wounds, sprains, and strains	0.1474
Albania	1995	Multiple fractures, dislocations, crashes, wounds, sprains, and strains	0.1234
Albania	2000	Multiple fractures, dislocations, crashes, wounds, sprains, and strains	0.1083
Albania	2005	Multiple fractures, dislocations, crashes, wounds, sprains, and strains	0.0899
Albania	2010	Multiple fractures, dislocations, crashes, wounds, sprains, and strains	0.0698
Albania	2017	Multiple fractures, dislocations, crashes, wounds, sprains, and strains	0.0577
Albania	1990	Amputation of upper limb, unilateral	0.0586
Albania	1995	Amputation of upper limb, unilateral	0.0541
Albania	2000	Amputation of upper limb, unilateral	0.0506
Albania	2005	Amputation of upper limb, unilateral	0.0464
Albania	2010	Amputation of upper limb, unilateral	0.0418
Albania	2017	Amputation of upper limb, unilateral	0.039
Albania	1990	Amputation of thumb	0.0114
Albania	1995	Amputation of thumb	0.0114
Albania	2000	Amputation of thumb	0.0114
Albania	2005	Amputation of thumb	0.0114
Albania	2010	Amputation of thumb	0.0114
Albania	2017	Amputation of thumb	0.0114
Albania	1990	Amputation of toe/toes	0.0059
Albania	1995	Amputation of toe/toes	0.0059
Albania	2000	Amputation of toe/toes	0.0059
Albania	2005	Amputation of toe/toes	0.0059
Albania	2010	Amputation of toe/toes	0.0059
Albania	2017	Amputation of toe/toes	0.0059
Albania	1990	Burns, <20% total burned surface area without lower airway burns	0.0163
Albania	1995	Burns, <20% total burned surface area without lower airway burns	0.0163
Albania	2000	Burns, <20% total burned surface area without lower airway burns	0.0163
Albania	2005	Burns, <20% total burned surface area without lower airway burns	0.0163
Albania	2010	Burns, <20% total burned surface area without lower airway burns	0.0163
Albania	2017	Burns, <20% total burned surface area without lower airway burns	0.0163
Albania	1990	Burns, >=20% total burned surface area or >= 10% burned surface area if head/neck or hands/wrist involved w/o lower airway burns	0.2181
Albania	1995	Burns, >=20% total burned surface area or >= 10% burned surface area if head/neck or hands/wrist involved w/o lower airway burns	0.1958
Albania	2000	Burns, >=20% total burned surface area or >= 10% burned surface area if head/neck or hands/wrist involved w/o lower airway burns	0.1817
Albania	2005	Burns, >=20% total burned surface area or >= 10% burned surface area if head/neck or hands/wrist involved w/o lower airway burns	0.1646
Albania	2010	Burns, >=20% total burned surface area or >= 10% burned surface area if head/neck or hands/wrist involved w/o lower airway burns	0.1459
Albania	2017	Burns, >=20% total burned surface area or >= 10% burned surface area if head/neck or hands/wrist involved w/o lower airway burns	0.1346
Bosnia and Herzegovina	1990	Amputation of lower limbs, bilateral	0.1997
Bosnia and Herzegovina	1995	Amputation of lower limbs, bilateral	0.1978
Bosnia and Herzegovina	2000	Amputation of lower limbs, bilateral	0.169
Bosnia and Herzegovina	2005	Amputation of lower limbs, bilateral	0.1542
Bosnia and Herzegovina	2010	Amputation of lower limbs, bilateral	0.1416
Bosnia and Herzegovina	2017	Amputation of lower limbs, bilateral	0.1316
Bosnia and Herzegovina	1990	Lower airway burns	0.2252
Bosnia and Herzegovina	1995	Lower airway burns	0.2252
Bosnia and Herzegovina	2000	Lower airway burns	0.2252
Bosnia and Herzegovina	2005	Lower airway burns	0.2252
Bosnia and Herzegovina	2010	Lower airway burns	0.2252
Bosnia and Herzegovina	2017	Lower airway burns	0.2252
Bosnia and Herzegovina	1990	Dislocation of hip	0.0159
Bosnia and Herzegovina	1995	Dislocation of hip	0.0159
Bosnia and Herzegovina	2000	Dislocation of hip	0.0159
Bosnia and Herzegovina	2005	Dislocation of hip	0.0159
Bosnia and Herzegovina	2010	Dislocation of hip	0.0159
Bosnia and Herzegovina	2017	Dislocation of hip	0.0159
Bosnia and Herzegovina	1990	Dislocation of knee	0.1134
Bosnia and Herzegovina	1995	Dislocation of knee	0.1134
Bosnia and Herzegovina	2000	Dislocation of knee	0.1134
Bosnia and Herzegovina	2005	Dislocation of knee	0.1134
Bosnia and Herzegovina	2010	Dislocation of knee	0.1134
Bosnia and Herzegovina	2017	Dislocation of knee	0.1134
Bosnia and Herzegovina	1990	Dislocation of shoulder	0.062
Bosnia and Herzegovina	1995	Dislocation of shoulder	0.062
Bosnia and Herzegovina	2000	Dislocation of shoulder	0.062
Bosnia and Herzegovina	2005	Dislocation of shoulder	0.062
Bosnia and Herzegovina	2010	Dislocation of shoulder	0.062
Bosnia and Herzegovina	2017	Dislocation of shoulder	0.062
Bosnia and Herzegovina	1990	Muscle and tendon injuries, including sprains and strains lesser dislocations	0.0075
Bosnia and Herzegovina	1995	Muscle and tendon injuries, including sprains and strains lesser dislocations	0.0075
Bosnia and Herzegovina	2000	Muscle and tendon injuries, including sprains and strains lesser dislocations	0.0075
Bosnia and Herzegovina	2005	Muscle and tendon injuries, including sprains and strains lesser dislocations	0.0075
Bosnia and Herzegovina	2010	Muscle and tendon injuries, including sprains and strains lesser dislocations	0.0075
Bosnia and Herzegovina	2017	Muscle and tendon injuries, including sprains and strains lesser dislocations	0.0075
Bosnia and Herzegovina	1990	Fracture of clavicle, scapula, or humerus	0.0349
Bosnia and Herzegovina	1995	Fracture of clavicle, scapula, or humerus	0.0349
Bosnia and Herzegovina	2000	Fracture of clavicle, scapula, or humerus	0.0349
Bosnia and Herzegovina	2005	Fracture of clavicle, scapula, or humerus	0.0349
Bosnia and Herzegovina	2010	Fracture of clavicle, scapula, or humerus	0.0349
Bosnia and Herzegovina	2017	Fracture of clavicle, scapula, or humerus	0.0349
Bosnia and Herzegovina	1990	Fracture of face bones	0.0669
Bosnia and Herzegovina	1995	Fracture of face bones	0.0669
Bosnia and Herzegovina	2000	Fracture of face bones	0.0669
Bosnia and Herzegovina	2005	Fracture of face bones	0.0669
Bosnia and Herzegovina	2010	Fracture of face bones	0.0669
Bosnia and Herzegovina	2017	Fracture of face bones	0.0669
Bosnia and Herzegovina	1990	Fracture of foot bones except ankle	0.0264
Bosnia and Herzegovina	1995	Fracture of foot bones except ankle	0.0264
Bosnia and Herzegovina	2000	Fracture of foot bones except ankle	0.0264
Bosnia and Herzegovina	2005	Fracture of foot bones except ankle	0.0264
Bosnia and Herzegovina	2010	Fracture of foot bones except ankle	0.0264
Bosnia and Herzegovina	2017	Fracture of foot bones except ankle	0.0264
Bosnia and Herzegovina	1990	Fracture of hand (wrist and other distal part of hand)	0.0138
Bosnia and Herzegovina	1995	Fracture of hand (wrist and other distal part of hand)	0.0138
Bosnia and Herzegovina	2000	Fracture of hand (wrist and other distal part of hand)	0.0138
Bosnia and Herzegovina	2005	Fracture of hand (wrist and other distal part of hand)	0.0138
Bosnia and Herzegovina	2010	Fracture of hand (wrist and other distal part of hand)	0.0138
Bosnia and Herzegovina	2017	Fracture of hand (wrist and other distal part of hand)	0.0138
Bosnia and Herzegovina	1990	Fracture of hip	0.1588
Bosnia and Herzegovina	1995	Fracture of hip	0.1562
Bosnia and Herzegovina	2000	Fracture of hip	0.1182
Bosnia and Herzegovina	2005	Fracture of hip	0.0986
Bosnia and Herzegovina	2010	Fracture of hip	0.082
Bosnia and Herzegovina	2017	Fracture of hip	0.0687
Bosnia and Herzegovina	1990	Amputation of upper limbs, bilateral	0.1921
Bosnia and Herzegovina	1995	Amputation of upper limbs, bilateral	0.1894
Bosnia and Herzegovina	2000	Amputation of upper limbs, bilateral	0.1501
Bosnia and Herzegovina	2005	Amputation of upper limbs, bilateral	0.1299
Bosnia and Herzegovina	2010	Amputation of upper limbs, bilateral	0.1127
Bosnia and Herzegovina	2017	Amputation of upper limbs, bilateral	0.0991
Bosnia and Herzegovina	1990	Fracture of patella, tibia or fibula, or ankle	0.0554
Bosnia and Herzegovina	1995	Fracture of patella, tibia or fibula, or ankle	0.0554
Bosnia and Herzegovina	2000	Fracture of patella, tibia or fibula, or ankle	0.0554
Bosnia and Herzegovina	2005	Fracture of patella, tibia or fibula, or ankle	0.0554
Bosnia and Herzegovina	2010	Fracture of patella, tibia or fibula, or ankle	0.0554
Bosnia and Herzegovina	2017	Fracture of patella, tibia or fibula, or ankle	0.0554
Bosnia and Herzegovina	1990	Fracture of pelvis	0.1823
Bosnia and Herzegovina	1995	Fracture of pelvis	0.1823
Bosnia and Herzegovina	2000	Fracture of pelvis	0.1823
Bosnia and Herzegovina	2005	Fracture of pelvis	0.1823
Bosnia and Herzegovina	2010	Fracture of pelvis	0.1823
Bosnia and Herzegovina	2017	Fracture of pelvis	0.1823
Bosnia and Herzegovina	1990	Fracture of radius and/or ulna	0.0435
Bosnia and Herzegovina	1995	Fracture of radius and/or ulna	0.0435
Bosnia and Herzegovina	2000	Fracture of radius and/or ulna	0.0435
Bosnia and Herzegovina	2005	Fracture of radius and/or ulna	0.0435
Bosnia and Herzegovina	2010	Fracture of radius and/or ulna	0.0435
Bosnia and Herzegovina	2017	Fracture of radius and/or ulna	0.0435
Bosnia and Herzegovina	1990	Fracture of skull	0.0714
Bosnia and Herzegovina	1995	Fracture of skull	0.0714
Bosnia and Herzegovina	2000	Fracture of skull	0.0714
Bosnia and Herzegovina	2005	Fracture of skull	0.0714
Bosnia and Herzegovina	2010	Fracture of skull	0.0714
Bosnia and Herzegovina	2017	Fracture of skull	0.0714
Bosnia and Herzegovina	1990	Fracture of sternum and/or fracture of one or more ribs	0.1027
Bosnia and Herzegovina	1995	Fracture of sternum and/or fracture of one or more ribs	0.1027
Bosnia and Herzegovina	2000	Fracture of sternum and/or fracture of one or more ribs	0.1027
Bosnia and Herzegovina	2005	Fracture of sternum and/or fracture of one or more ribs	0.1027
Bosnia and Herzegovina	2010	Fracture of sternum and/or fracture of one or more ribs	0.1027
Bosnia and Herzegovina	2017	Fracture of sternum and/or fracture of one or more ribs	0.1027
Bosnia and Herzegovina	1990	Fracture of vertebral column	0.1106
Bosnia and Herzegovina	1995	Fracture of vertebral column	0.1106
Bosnia and Herzegovina	2000	Fracture of vertebral column	0.1106
Bosnia and Herzegovina	2005	Fracture of vertebral column	0.1106
Bosnia and Herzegovina	2010	Fracture of vertebral column	0.1106
Bosnia and Herzegovina	2017	Fracture of vertebral column	0.1106
Bosnia and Herzegovina	1990	Fracture of femur, other than femoral neck	0.042
Bosnia and Herzegovina	1995	Fracture of femur, other than femoral neck	0.042
Bosnia and Herzegovina	2000	Fracture of femur, other than femoral neck	0.042
Bosnia and Herzegovina	2005	Fracture of femur, other than femoral neck	0.042
Bosnia and Herzegovina	2010	Fracture of femur, other than femoral neck	0.042
Bosnia and Herzegovina	2017	Fracture of femur, other than femoral neck	0.042
Bosnia and Herzegovina	1990	Minor TBI	0.1319
Bosnia and Herzegovina	1995	Minor TBI	0.1319
Bosnia and Herzegovina	2000	Minor TBI	0.1319
Bosnia and Herzegovina	2005	Minor TBI	0.1319
Bosnia and Herzegovina	2010	Minor TBI	0.1319
Bosnia and Herzegovina	2017	Minor TBI	0.1319
Bosnia and Herzegovina	1990	Moderate TBI	0.1637
Bosnia and Herzegovina	1995	Moderate TBI	0.1637
Bosnia and Herzegovina	2000	Moderate TBI	0.1637
Bosnia and Herzegovina	2005	Moderate TBI	0.1637
Bosnia and Herzegovina	2010	Moderate TBI	0.1637
Bosnia and Herzegovina	2017	Moderate TBI	0.1637
Bosnia and Herzegovina	1990	Amputation of fingers (excluding thumb)	0.0047
Bosnia and Herzegovina	1995	Amputation of fingers (excluding thumb)	0.0047
Bosnia and Herzegovina	2000	Amputation of fingers (excluding thumb)	0.0047
Bosnia and Herzegovina	2005	Amputation of fingers (excluding thumb)	0.0047
Bosnia and Herzegovina	2010	Amputation of fingers (excluding thumb)	0.0047
Bosnia and Herzegovina	2017	Amputation of fingers (excluding thumb)	0.0047
Bosnia and Herzegovina	1990	Spinal cord lesion at neck level	0.4468
Bosnia and Herzegovina	1995	Spinal cord lesion at neck level	0.4459
Bosnia and Herzegovina	2000	Spinal cord lesion at neck level	0.4328
Bosnia and Herzegovina	2005	Spinal cord lesion at neck level	0.426
Bosnia and Herzegovina	2010	Spinal cord lesion at neck level	0.4202
Bosnia and Herzegovina	2017	Spinal cord lesion at neck level	0.4156
Bosnia and Herzegovina	1990	Spinal cord lesion below neck level	0.2515
Bosnia and Herzegovina	1995	Spinal cord lesion below neck level	0.2496
Bosnia and Herzegovina	2000	Spinal cord lesion below neck level	0.2218
Bosnia and Herzegovina	2005	Spinal cord lesion below neck level	0.2075
Bosnia and Herzegovina	2010	Spinal cord lesion below neck level	0.1954
Bosnia and Herzegovina	2017	Spinal cord lesion below neck level	0.1857
Bosnia and Herzegovina	1990	Drowning and nonfatal submersion	0.2471
Bosnia and Herzegovina	1995	Drowning and nonfatal submersion	0.2471
Bosnia and Herzegovina	2000	Drowning and nonfatal submersion	0.2471
Bosnia and Herzegovina	2005	Drowning and nonfatal submersion	0.2471
Bosnia and Herzegovina	2010	Drowning and nonfatal submersion	0.2471
Bosnia and Herzegovina	2017	Drowning and nonfatal submersion	0.2471
Bosnia and Herzegovina	1990	Asphyxiation	0.2471
Bosnia and Herzegovina	1995	Asphyxiation	0.2471
Bosnia and Herzegovina	2000	Asphyxiation	0.2471
Bosnia and Herzegovina	2005	Asphyxiation	0.2471
Bosnia and Herzegovina	2010	Asphyxiation	0.2471
Bosnia and Herzegovina	2017	Asphyxiation	0.2471
Bosnia and Herzegovina	1990	Crush injury	0.1325
Bosnia and Herzegovina	1995	Crush injury	0.1325
Bosnia and Herzegovina	2000	Crush injury	0.1325
Bosnia and Herzegovina	2005	Crush injury	0.1325
Bosnia and Herzegovina	2010	Crush injury	0.1325
Bosnia and Herzegovina	2017	Crush injury	0.1325
Bosnia and Herzegovina	1990	Nerve injury	0.1129
Bosnia and Herzegovina	1995	Nerve injury	0.1129
Bosnia and Herzegovina	2000	Nerve injury	0.1129
Bosnia and Herzegovina	2005	Nerve injury	0.1129
Bosnia and Herzegovina	2010	Nerve injury	0.1129
Bosnia and Herzegovina	2017	Nerve injury	0.1129
Bosnia and Herzegovina	1990	Injury to eyes	0.0165
Bosnia and Herzegovina	1995	Injury to eyes	0.0165
Bosnia and Herzegovina	2000	Injury to eyes	0.0165
Bosnia and Herzegovina	2005	Injury to eyes	0.0165
Bosnia and Herzegovina	2010	Injury to eyes	0.0165
Bosnia and Herzegovina	2017	Injury to eyes	0.0165
Bosnia and Herzegovina	1990	Amputation of lower limb, unilateral	0.0784
Bosnia and Herzegovina	1995	Amputation of lower limb, unilateral	0.0774
Bosnia and Herzegovina	2000	Amputation of lower limb, unilateral	0.0625
Bosnia and Herzegovina	2005	Amputation of lower limb, unilateral	0.0549
Bosnia and Herzegovina	2010	Amputation of lower limb, unilateral	0.0484
Bosnia and Herzegovina	2017	Amputation of lower limb, unilateral	0.0433
Bosnia and Herzegovina	1990	Open wound(s)	0.011
Bosnia and Herzegovina	1995	Open wound(s)	0.011
Bosnia and Herzegovina	2000	Open wound(s)	0.011
Bosnia and Herzegovina	2005	Open wound(s)	0.011
Bosnia and Herzegovina	2010	Open wound(s)	0.011
Bosnia and Herzegovina	2017	Open wound(s)	0.011
Bosnia and Herzegovina	1990	Poisoning requiring urgent care	0.1628
Bosnia and Herzegovina	1995	Poisoning requiring urgent care	0.1628
Bosnia and Herzegovina	2000	Poisoning requiring urgent care	0.1628
Bosnia and Herzegovina	2005	Poisoning requiring urgent care	0.1628

Bosnia and Herzegovina	2010	Poisoning requiring urgent care	0.1628
Bosnia and Herzegovina	2017	Poisoning requiring urgent care	0.1628
Bosnia and Herzegovina	1990	Severe chest injury	0.0474
Bosnia and Herzegovina	1995	Severe chest injury	0.0474
Bosnia and Herzegovina	2000	Severe chest injury	0.0474
Bosnia and Herzegovina	2005	Severe chest injury	0.0474
Bosnia and Herzegovina	2010	Severe chest injury	0.0474
Bosnia and Herzegovina	2017	Severe chest injury	0.0474
Bosnia and Herzegovina	1990	Internal hemorrhage in abdomen and pelvis	0.1143
Bosnia and Herzegovina	1995	Internal hemorrhage in abdomen and pelvis	0.1143
Bosnia and Herzegovina	2000	Internal hemorrhage in abdomen and pelvis	0.1143
Bosnia and Herzegovina	2005	Internal hemorrhage in abdomen and pelvis	0.1143
Bosnia and Herzegovina	2010	Internal hemorrhage in abdomen and pelvis	0.1143
Bosnia and Herzegovina	2017	Internal hemorrhage in abdomen and pelvis	0.1143
Bosnia and Herzegovina	1990	Contusion in any part of the body	0.0075
Bosnia and Herzegovina	1995	Contusion in any part of the body	0.0075
Bosnia and Herzegovina	2000	Contusion in any part of the body	0.0075
Bosnia and Herzegovina	2005	Contusion in any part of the body	0.0075
Bosnia and Herzegovina	2010	Contusion in any part of the body	0.0075
Bosnia and Herzegovina	2017	Contusion in any part of the body	0.0075
Bosnia and Herzegovina	1990	Effect of different environmental factors	0.049
Bosnia and Herzegovina	1995	Effect of different environmental factors	0.049
Bosnia and Herzegovina	2000	Effect of different environmental factors	0.049
Bosnia and Herzegovina	2005	Effect of different environmental factors	0.049
Bosnia and Herzegovina	2010	Effect of different environmental factors	0.049
Bosnia and Herzegovina	2017	Effect of different environmental factors	0.049
Bosnia and Herzegovina	1990	Complications following therapeutic procedures	0.0506
Bosnia and Herzegovina	1995	Complications following therapeutic procedures	0.0506
Bosnia and Herzegovina	2000	Complications following therapeutic procedures	0.0506
Bosnia and Herzegovina	2005	Complications following therapeutic procedures	0.0506
Bosnia and Herzegovina	2010	Complications following therapeutic procedures	0.0506
Bosnia and Herzegovina	2017	Complications following therapeutic procedures	0.0506
Bosnia and Herzegovina	1990	Multiple fractures, dislocations, crashes, wounds, sprains, and strains	0.1588
Bosnia and Herzegovina	1995	Multiple fractures, dislocations, crashes, wounds, sprains, and strains	0.1562
Bosnia and Herzegovina	2000	Multiple fractures, dislocations, crashes, wounds, sprains, and strains	0.1182
Bosnia and Herzegovina	2005	Multiple fractures, dislocations, crashes, wounds, sprains, and strains	0.0986
Bosnia and Herzegovina	2010	Multiple fractures, dislocations, crashes, wounds, sprains, and strains	0.082
Bosnia and Herzegovina	2017	Multiple fractures, dislocations, crashes, wounds, sprains, and strains	0.0687
Bosnia and Herzegovina	1990	Amputation of upper limb, unilateral	0.0622
Bosnia and Herzegovina	1995	Amputation of upper limb, unilateral	0.0616
Bosnia and Herzegovina	2000	Amputation of upper limb, unilateral	0.0529
Bosnia and Herzegovina	2005	Amputation of upper limb, unilateral	0.0484
Bosnia and Herzegovina	2010	Amputation of upper limb, unilateral	0.0446
Bosnia and Herzegovina	2017	Amputation of upper limb, unilateral	0.0415
Bosnia and Herzegovina	1990	Amputation of thumb	0.0114
Bosnia and Herzegovina	1995	Amputation of thumb	0.0114
Bosnia and Herzegovina	2000	Amputation of thumb	0.0114
Bosnia and Herzegovina	2005	Amputation of thumb	0.0114
Bosnia and Herzegovina	2010	Amputation of thumb	0.0114
Bosnia and Herzegovina	2017	Amputation of thumb	0.0114
Bosnia and Herzegovina	1990	Amputation of toe/toes	0.0059
Bosnia and Herzegovina	1995	Amputation of toe/toes	0.0059
Bosnia and Herzegovina	2000	Amputation of toe/toes	0.0059
Bosnia and Herzegovina	2005	Amputation of toe/toes	0.0059
Bosnia and Herzegovina	2010	Amputation of toe/toes	0.0059
Bosnia and Herzegovina	2017	Amputation of toe/toes	0.0059
Bosnia and Herzegovina	1990	Burns, <20% total burned surface area without lower airway burns	0.0163
Bosnia and Herzegovina	1995	Burns, <20% total burned surface area without lower airway burns	0.0163
Bosnia and Herzegovina	2000	Burns, <20% total burned surface area without lower airway burns	0.0163
Bosnia and Herzegovina	2005	Burns, <20% total burned surface area without lower airway burns	0.0163
Bosnia and Herzegovina	2010	Burns, <20% total burned surface area without lower airway burns	0.0163
Bosnia and Herzegovina	2017	Burns, <20% total burned surface area without lower airway burns	0.0163
Bosnia and Herzegovina	1990	Burns, >=20% total burned surface area or >= 10% burned surface area if head/neck or hands/wrist involved w/o lower airway burns	0.2288
Bosnia and Herzegovina	1995	Burns, >=20% total burned surface area or >= 10% burned surface area if head/neck or hands/wrist involved w/o lower airway burns	0.2264
Bosnia and Herzegovina	2000	Burns, >=20% total burned surface area or >= 10% burned surface area if head/neck or hands/wrist involved w/o lower airway burns	0.1909
Bosnia and Herzegovina	2005	Burns, >=20% total burned surface area or >= 10% burned surface area if head/neck or hands/wrist involved w/o lower airway burns	0.1727
Bosnia and Herzegovina	2010	Burns, >=20% total burned surface area or >= 10% burned surface area if head/neck or hands/wrist involved w/o lower airway burns	0.1572
Bosnia and Herzegovina	2017	Burns, >=20% total burned surface area or >= 10% burned surface area if head/neck or hands/wrist involved w/o lower airway burns	0.1449
Bulgaria	1990	Amputation of lower limbs, bilateral	0.1571
Bulgaria	1995	Amputation of lower limbs, bilateral	0.1527
Bulgaria	2000	Amputation of lower limbs, bilateral	0.1475
Bulgaria	2005	Amputation of lower limbs, bilateral	0.1379
Bulgaria	2010	Amputation of lower limbs, bilateral	0.1283
Bulgaria	2017	Amputation of lower limbs, bilateral	0.1232
Bulgaria	1990	Lower airway burns	0.2252
Bulgaria	1995	Lower airway burns	0.2252
Bulgaria	2000	Lower airway burns	0.2252
Bulgaria	2005	Lower airway burns	0.2252
Bulgaria	2010	Lower airway burns	0.2252
Bulgaria	2017	Lower airway burns	0.2252
Bulgaria	1990	Dislocation of hip	0.0159
Bulgaria	1995	Dislocation of hip	0.0159
Bulgaria	2000	Dislocation of hip	0.0159
Bulgaria	2005	Dislocation of hip	0.0159
Bulgaria	2010	Dislocation of hip	0.0159
Bulgaria	2017	Dislocation of hip	0.0159
Bulgaria	1990	Dislocation of knee	0.1134
Bulgaria	1995	Dislocation of knee	0.1134
Bulgaria	2000	Dislocation of knee	0.1134
Bulgaria	2005	Dislocation of knee	0.1134
Bulgaria	2010	Dislocation of knee	0.1134
Bulgaria	2017	Dislocation of knee	0.1134
Bulgaria	1990	Dislocation of shoulder	0.062
Bulgaria	1995	Dislocation of shoulder	0.062
Bulgaria	2000	Dislocation of shoulder	0.062
Bulgaria	2005	Dislocation of shoulder	0.062
Bulgaria	2010	Dislocation of shoulder	0.062
Bulgaria	2017	Dislocation of shoulder	0.062
Bulgaria	1990	Muscle and tendon injuries, including sprains and strains lesser dislocations	0.0075
Bulgaria	1995	Muscle and tendon injuries, including sprains and strains lesser dislocations	0.0075
Bulgaria	2000	Muscle and tendon injuries, including sprains and strains lesser dislocations	0.0075
Bulgaria	2005	Muscle and tendon injuries, including sprains and strains lesser dislocations	0.0075
Bulgaria	2010	Muscle and tendon injuries, including sprains and strains lesser dislocations	0.0075
Bulgaria	2017	Muscle and tendon injuries, including sprains and strains lesser dislocations	0.0075
Bulgaria	1990	Fracture of clavicle, scapula, or humerus	0.0349
Bulgaria	1995	Fracture of clavicle, scapula, or humerus	0.0349
Bulgaria	2000	Fracture of clavicle, scapula, or humerus	0.0349
Bulgaria	2005	Fracture of clavicle, scapula, or humerus	0.0349
Bulgaria	2010	Fracture of clavicle, scapula, or humerus	0.0349
Bulgaria	2017	Fracture of clavicle, scapula, or humerus	0.0349
Bulgaria	1990	Fracture of face bones	0.0669
Bulgaria	1995	Fracture of face bones	0.0669
Bulgaria	2000	Fracture of face bones	0.0669
Bulgaria	2005	Fracture of face bones	0.0669
Bulgaria	2010	Fracture of face bones	0.0669
Bulgaria	2017	Fracture of face bones	0.0669
Bulgaria	1990	Fracture of foot bones except ankle	0.0264
Bulgaria	1995	Fracture of foot bones except ankle	0.0264
Bulgaria	2000	Fracture of foot bones except ankle	0.0264
Bulgaria	2005	Fracture of foot bones except ankle	0.0264
Bulgaria	2010	Fracture of foot bones except ankle	0.0264
Bulgaria	2017	Fracture of foot bones except ankle	0.0264
Bulgaria	1990	Fracture of hand (wrist and other distal part of hand)	0.0138
Bulgaria	1995	Fracture of hand (wrist and other distal part of hand)	0.0138
Bulgaria	2000	Fracture of hand (wrist and other distal part of hand)	0.0138
Bulgaria	2005	Fracture of hand (wrist and other distal part of hand)	0.0138
Bulgaria	2010	Fracture of hand (wrist and other distal part of hand)	0.0138
Bulgaria	2017	Fracture of hand (wrist and other distal part of hand)	0.0138
Bulgaria	1990	Fracture of hip	0.1024
Bulgaria	1995	Fracture of hip	0.0967
Bulgaria	2000	Fracture of hip	0.0897
Bulgaria	2005	Fracture of hip	0.0771
Bulgaria	2010	Fracture of hip	0.0644
Bulgaria	2017	Fracture of hip	0.0577
Bulgaria	1990	Amputation of upper limbs, bilateral	0.1338
Bulgaria	1995	Amputation of upper limbs, bilateral	0.1279
Bulgaria	2000	Amputation of upper limbs, bilateral	0.1207
Bulgaria	2005	Amputation of upper limbs, bilateral	0.1077
Bulgaria	2010	Amputation of upper limbs, bilateral	0.0946
Bulgaria	2017	Amputation of upper limbs, bilateral	0.0876
Bulgaria	1990	Fracture of patella, tibia or fibula, or ankle	0.0554
Bulgaria	1995	Fracture of patella, tibia or fibula, or ankle	0.0554
Bulgaria	2000	Fracture of patella, tibia or fibula, or ankle	0.0554
Bulgaria	2005	Fracture of patella, tibia or fibula, or ankle	0.0554
Bulgaria	2010	Fracture of patella, tibia or fibula, or ankle	0.0554
Bulgaria	2017	Fracture of patella, tibia or fibula, or ankle	0.0554
Bulgaria	1990	Fracture of pelvis	0.1823
Bulgaria	1995	Fracture of pelvis	0.1823
Bulgaria	2000	Fracture of pelvis	0.1823
Bulgaria	2005	Fracture of pelvis	0.1823
Bulgaria	2010	Fracture of pelvis	0.1823
Bulgaria	2017	Fracture of pelvis	0.1823
Bulgaria	1990	Fracture of radius and/or ulna	0.0435
Bulgaria	1995	Fracture of radius and/or ulna	0.0435
Bulgaria	2000	Fracture of radius and/or ulna	0.0435
Bulgaria	2005	Fracture of radius and/or ulna	0.0435
Bulgaria	2010	Fracture of radius and/or ulna	0.0435
Bulgaria	2017	Fracture of radius and/or ulna	0.0435
Bulgaria	1990	Fracture of skull	0.0714
Bulgaria	1995	Fracture of skull	0.0714
Bulgaria	2000	Fracture of skull	0.0714
Bulgaria	2005	Fracture of skull	0.0714
Bulgaria	2010	Fracture of skull	0.0714
Bulgaria	2017	Fracture of skull	0.0714
Bulgaria	1990	Fracture of sternum and/or fracture of one or more ribs	0.1027
Bulgaria	1995	Fracture of sternum and/or fracture of one or more ribs	0.1027
Bulgaria	2000	Fracture of sternum and/or fracture of one or more ribs	0.1027
Bulgaria	2005	Fracture of sternum and/or fracture of one or more ribs	0.1027
Bulgaria	2010	Fracture of sternum and/or fracture of one or more ribs	0.1027
Bulgaria	2017	Fracture of sternum and/or fracture of one or more ribs	0.1027
Bulgaria	1990	Fracture of vertebral column	0.1106
Bulgaria	1995	Fracture of vertebral column	0.1106
Bulgaria	2000	Fracture of vertebral column	0.1106
Bulgaria	2005	Fracture of vertebral column	0.1106
Bulgaria	2010	Fracture of vertebral column	0.1106
Bulgaria	2017	Fracture of vertebral column	0.1106
Bulgaria	1990	Fracture of femur, other than femoral neck	0.042
Bulgaria	1995	Fracture of femur, other than femoral neck	0.042
Bulgaria	2000	Fracture of femur, other than femoral neck	0.042
Bulgaria	2005	Fracture of femur, other than femoral neck	0.042
Bulgaria	2010	Fracture of femur, other than femoral neck	0.042
Bulgaria	2017	Fracture of femur, other than femoral neck	0.042
Bulgaria	1990	Minor TBI	0.1319
Bulgaria	1995	Minor TBI	0.1319
Bulgaria	2000	Minor TBI	0.1319
Bulgaria	2005	Minor TBI	0.1319
Bulgaria	2010	Minor TBI	0.1319
Bulgaria	2017	Minor TBI	0.1319
Bulgaria	1990	Moderate TBI	0.1637
Bulgaria	1995	Moderate TBI	0.1637
Bulgaria	2000	Moderate TBI	0.1637
Bulgaria	2005	Moderate TBI	0.1637
Bulgaria	2010	Moderate TBI	0.1637
Bulgaria	2017	Moderate TBI	0.1637
Bulgaria	1990	Amputation of fingers (excluding thumb)	0.0047
Bulgaria	1995	Amputation of fingers (excluding thumb)	0.0047
Bulgaria	2000	Amputation of fingers (excluding thumb)	0.0047
Bulgaria	2005	Amputation of fingers (excluding thumb)	0.0047
Bulgaria	2010	Amputation of fingers (excluding thumb)	0.0047
Bulgaria	2017	Amputation of fingers (excluding thumb)	0.0047
Bulgaria	1990	Spinal cord lesion at neck level	0.4273
Bulgaria	1995	Spinal cord lesion at neck level	0.4253
Bulgaria	2000	Spinal cord lesion at neck level	0.4229
Bulgaria	2005	Spinal cord lesion at neck level	0.4185
Bulgaria	2010	Spinal cord lesion at neck level	0.4141
Bulgaria	2017	Spinal cord lesion at neck level	0.4118
Bulgaria	1990	Spinal cord lesion below neck level	0.2103
Bulgaria	1995	Spinal cord lesion below neck level	0.2061
Bulgaria	2000	Spinal cord lesion below neck level	0.201
Bulgaria	2005	Spinal cord lesion below neck level	0.1918
Bulgaria	2010	Spinal cord lesion below neck level	0.1826
Bulgaria	2017	Spinal cord lesion below neck level	0.1777
Bulgaria	1990	Drowning and nonfatal submersion	0.2471
Bulgaria	1995	Drowning and nonfatal submersion	0.2471
Bulgaria	2000	Drowning and nonfatal submersion	0.2471
Bulgaria	2005	Drowning and nonfatal submersion	0.2471
Bulgaria	2010	Drowning and nonfatal submersion	0.2471
Bulgaria	2017	Drowning and nonfatal submersion	0.2471
Bulgaria	1990	Asphyxiation	0.2471
Bulgaria	1995	Asphyxiation	0.2471
Bulgaria	2000	Asphyxiation	0.2471
Bulgaria	2005	Asphyxiation	0.2471
Bulgaria	2010	Asphyxiation	0.2471
Bulgaria	2017	Asphyxiation	0.2471
Bulgaria	1990	Crush injury	0.1325
Bulgaria	1995	Crush injury	0.1325
Bulgaria	2000	Crush injury	0.1325
Bulgaria	2005	Crush injury	0.1325
Bulgaria	2010	Crush injury	0.1325
Bulgaria	2017	Crush injury	0.1325
Bulgaria	1990	Nerve injury	0.1129
Bulgaria	1995	Nerve injury	0.1129
Bulgaria	2000	Nerve injury	0.1129
Bulgaria	2005	Nerve injury	0.1129
Bulgaria	2010	Nerve injury	0.1129
Bulgaria	2017	Nerve injury	0.1129
Bulgaria	1990	Injury to eyes	0.0165
Bulgaria	1995	Injury to eyes	0.0165
Bulgaria	2000	Injury to eyes	0.0165
Bulgaria	2005	Injury to eyes	0.0165
Bulgaria	2010	Injury to eyes	0.0165
Bulgaria	2017	Injury to eyes	0.0165
Bulgaria	1990	Amputation of lower limb, unilateral	0.0564
Bulgaria	1995	Amputation of lower limb, unilateral	0.0542
Bulgaria	2000	Amputation of lower limb, unilateral	0.0514
Bulgaria	2005	Amputation of lower limb, unilateral	0.0465
Bulgaria	2010	Amputation of lower limb, unilateral	0.0416
Bulgaria	2017	Amputation of lower limb, unilateral	0.039
Bulgaria	1990	Open wound(s)	0.011
Bulgaria	1995	Open wound(s)	0.011
Bulgaria	2000	Open wound(s)	0.011
Bulgaria	2005	Open wound(s)	0.011
Bulgaria	2010	Open wound(s)	0.011
Bulgaria	2017	Open wound(s)	0.011
Bulgaria	1990	Poisoning requiring urgent care	0.1628
Bulgaria	1995	Poisoning requiring urgent care	0.1628
Bulgaria	2000	Poisoning requiring urgent care	0.1628
Bulgaria	2005	Poisoning requiring urgent care	0.1628
Bulgaria	2010	Poisoning requiring urgent care	0.1628
Bulgaria	2017	Poisoning requiring urgent care	0.1628
Bulgaria	1990	Severe chest injury	0.0474
Bulgaria	1995	Severe chest injury	0.0474
Bulgaria	2000	Severe chest injury	0.0474
Bulgaria	2005	Severe chest injury	0.0474
Bulgaria	2010	Severe chest injury	0.0474
Bulgaria	2017	Severe chest injury	0.0474
Bulgaria	1990	Internal hemorrhage in abdomen and pelvis	0.1143
Bulgaria	1995	Internal hemorrhage in abdomen and pelvis	0.1143
Bulgaria	2000	Internal hemorrhage in abdomen and pelvis	0.1143
Bulgaria	2005	Internal hemorrhage in abdomen and pelvis	0.1143
Bulgaria	2010	Internal hemorrhage in abdomen and pelvis	0.1143
Bulgaria	2017	Internal hemorrhage in abdomen and pelvis	0.1143
Bulgaria	1990	Contusion in any part of the body	0.0075
Bulgaria	1995	Contusion in any part of the body	0.0075
Bulgaria	2000	Contusion in any part of the body	0.0075
Bulgaria	2005	Contusion in any part of the body	0.0075
Bulgaria	2010	Contusion in any part of the body	0.0075
Bulgaria	2017	Contusion in any part of the body	0.0075
Bulgaria	1990	Effect of different environmental factors	0.049
Bulgaria	1995	Effect of different environmental factors	0.049
Bulgaria	2000	Effect of different environmental factors	0.049
Bulgaria	2005	Effect of different environmental factors	0.049
Bulgaria	2010	Effect of different environmental factors	0.049
Bulgaria	2017	Effect of different environmental factors	0.049
Bulgaria	1990	Complications following therapeutic procedures	0.0506
Bulgaria	1995	Complications following therapeutic procedures	0.0506
Bulgaria	2000	Complications following therapeutic procedures	0.0506
Bulgaria	2005	Complications following therapeutic procedures	0.0506
Bulgaria	2010	Complications following therapeutic procedures	0.0506
Bulgaria	2017	Complications following therapeutic procedures	0.0506
Bulgaria	1990	Multiple fractures, dislocations, crashes, wounds, sprains, and strains	0.1024
Bulgaria	1995	Multiple fractures, dislocations, crashes, wounds, sprains, and strains	0.0967
Bulgaria	2000	Multiple fractures, dislocations, crashes, wounds, sprains, and strains	0.0897
Bulgaria	2005	Multiple fractures, dislocations, crashes, wounds, sprains, and strains	0.0771
Bulgaria	2010	Multiple fractures, dislocations, crashes, wounds, sprains, and strains	0.0644
Bulgaria	2017	Multiple fractures, dislocations, crashes, wounds, sprains, and strains	0.0577
Bulgaria	1990	Amputation of upper limb, unilateral	0.0492
Bulgaria	1995	Amputation of upper limb, unilateral	0.0479
Bulgaria	2000	Amputation of upper limb, unilateral	0.0463
Bulgaria	2005	Amputation of upper limb, unilateral	0.0434
Bulgaria	2010	Amputation of upper limb, unilateral	0.0405
Bulgaria	2017	Amputation of upper limb, unilateral	0.039
Bulgaria	1990	Amputation of thumb	0.0114
Bulgaria	1995	Amputation of thumb	0.0114
Bulgaria	2000	Amputation of thumb	0.0114
Bulgaria	2005	Amputation of thumb	0.0114
Bulgaria	2010	Amputation of thumb	0.0114
Bulgaria	2017	Amputation of thumb	0.0114
Bulgaria	1990	Amputation of toe/toes	0.0059
Bulgaria	1995	Amputation of toe/toes	0.0059
Bulgaria	2000	Amputation of toe/toes	0.0059
Bulgaria	2005	Amputation of toe/toes	0.0059
Bulgaria	2010	Amputation of toe/toes	0.0059
Bulgaria	2017	Amputation of toe/toes	0.0059
Bulgaria	1990	Burns, <20% total burned surface area without lower airway burns	0.0163
Bulgaria	1995	Burns, <20% total burned surface area without lower airway burns	0.0163
Bulgaria	2000	Burns, <20% total burned surface area without lower airway burns	0.0163
Bulgaria	2005	Burns, <20% total burned surface area without lower airway burns	0.0163
Bulgaria	2010	Burns, <20% total burned surface area without lower airway burns	0.0163
Bulgaria	2017	Burns, <20% total burned surface area without lower airway burns	0.0163
Bulgaria	1990	Burns, >=20% total burned surface area or >= 10% burned surface area if head/neck or hands/wrist involved w/o lower airway burns	0.1762
Bulgaria	1995	Burns, >=20% total burned surface area or >= 10% burned surface area if head/neck or hands/wrist involved w/o lower airway burns	0.1709
Bulgaria	2000	Burns, >=20% total burned surface area or >= 10% burned surface area if head/neck or hands/wrist involved w/o lower airway burns	0.1644
Bulgaria	2005	Burns, >=20% total burned surface area or >= 10% burned surface area if head/neck or hands/wrist involved w/o lower airway burns	0.1526
Bulgaria	2010	Burns, >=20% total burned surface area or >= 10% burned surface area if head/neck or hands/wrist involved w/o lower airway burns	0.1408
Bulgaria	2017	Burns, >=20% total burned surface area or >= 10% burned surface area if head/neck or hands/wrist involved w/o lower airway burns	0.1346
Croatia	1990	Amputation of lower limbs, bilateral	0.1271
Croatia	1995	Amputation of lower limbs, bilateral	0.1246
Croatia	2000	Amputation of lower limbs, bilateral	0.1232
Croatia	2005	Amputation of lower limbs, bilateral	0.1232
Croatia	2010	Amputation of lower limbs, bilateral	0.1232
Croatia	2017	Amputation of lower limbs, bilateral	0.1232
Croatia	1990	Lower airway burns	0.2252
Croatia	1995	Lower airway burns	0.2252
Croatia	2000	Lower airway burns	0.2252
Croatia	2005	Lower airway burns	0.2252
Croatia	2010	Lower airway burns	0.2252
Croatia	2017	Lower airway burns	0.2252
Croatia	1990	Dislocation of hip	0.0159
Croatia	1995	Dislocation of hip	0.0159
Croatia	2000	Dislocation of hip	0.0159
Croatia	2005	Dislocation of hip	0.0159
Croatia	2010	Dislocation of hip	0.0159
Croatia	2017	Dislocation of hip	0.0159
Croatia	1990	Dislocation of knee	0.1134
Croatia	1995	Dislocation of knee	0.1134
Croatia	2000	Dislocation of knee	0.1134
Croatia	2005	Dislocation of knee	0.1134
Croatia	2010	Dislocation of knee	0.1134
Croatia	2017	Dislocation of knee	0.1134
Croatia	1990	Dislocation of shoulder	0.062
Croatia	1995	Dislocation of shoulder	0.062
Croatia	2000	Dislocation of shoulder	0.062
Croatia	2005	Dislocation of shoulder	0.062
Croatia	2010	Dislocation of shoulder	0.062
Croatia	2017	Dislocation of shoulder	0.062
Croatia	1990	Muscle and tendon injuries, including sprains and strains lesser dislocations	0.0075
Croatia	1995	Muscle and tendon injuries, including sprains and strains lesser dislocations	0.0075
Croatia	2000	Muscle and tendon injuries, including sprains and strains lesser dislocations	0.0075
Croatia	2005	Muscle and tendon injuries, including sprains and strains lesser dislocations	0.0075
Croatia	2010	Muscle and tendon injuries, including sprains and strains lesser dislocations	0.0075
Croatia	2017	Muscle and tendon injuries, including sprains and strains lesser dislocations	0.0075
Croatia	1990	Fracture of clavicle, scapula, or humerus	0.0349
Croatia	1995	Fracture of clavicle, scapula, or humerus	0.0349
Croatia	2000	Fracture of clavicle, scapula, or humerus	0.0349
Croatia	2005	Fracture of clavicle, scapula, or humerus	0.0349
Croatia	2010	Fracture of clavicle, scapula, or humerus	0.0349
Croatia	2017	Fracture of clavicle, scapula, or humerus	0.0349
Croatia	1990	Fracture of face bones	0.0669
Croatia	1995	Fracture of face bones	0.0669
Croatia	2000	Fracture of face bones	0.0669
Croatia	2005	Fracture of face bones	0.0669
Croatia	2010	Fracture of face bones	0.0669
Croatia	2017	Fracture of face bones	0.0669
Croatia	1990	Fracture of foot bones except ankle	0.0264
Croatia	1995	Fracture of foot bones except ankle	0.0264
Croatia	2000	Fracture of foot bones except ankle	0.0264
Croatia	2005	Fracture of foot bones except ankle	0.0264
Croatia	2010	Fracture of foot bones except ankle	0.0264
Croatia	2017	Fracture of foot bones except ankle	0.0264
Croatia	1990	Fracture of hand (wrist and other distal part of hand)	0.0138
Croatia	1995	Fracture of hand (wrist and other distal part of hand)	0.0138
Croatia	2000	Fracture of hand (wrist and other distal part of hand)	0.0138
Croatia	2005	Fracture of hand (wrist and other distal part of hand)	0.0138
Croatia	2010	Fracture of hand (wrist and other distal part of hand)	0.0138
Croatia	2017	Fracture of hand (wrist and other distal part of hand)	0.0138
Croatia	1990	Fracture of hip	0.0628
Croatia	1995	Fracture of hip	0.0595
Croatia	2000	Fracture of hip	0.0577
Croatia	2005	Fracture of hip	0.0577
Croatia	2010	Fracture of hip	0.0577
Croatia	2017	Fracture of hip	0.0577
Croatia	1990	Amputation of upper limbs, bilateral	0.0929
Croatia	1995	Amputation of upper limbs, bilateral	0.0895
Croatia	2000	Amputation of upper limbs, bilateral	0.0876
Croatia	2005	Amputation of upper limbs, bilateral	0.0876
Croatia	2010	Amputation of upper limbs, bilateral	0.0876
Croatia	2017	Amputation of upper limbs, bilateral	0.0876
Croatia	1990	Fracture of patella, tibia or fibula, or ankle	0.0554
Croatia	1995	Fracture of patella, tibia or fibula, or ankle	0.0554
Croatia	2000	Fracture of patella, tibia or fibula, or ankle	0.0554
Croatia	2005	Fracture of patella, tibia or fibula, or ankle	0.0554
Croatia	2010	Fracture of patella, tibia or fibula, or ankle	0.0554
Croatia	2017	Fracture of patella, tibia or fibula, or ankle	0.0554
Croatia	1990	Fracture of pelvis	0.1823
Croatia	1995	Fracture of pelvis	0.1823
Croatia	2000	Fracture of pelvis	0.1823
Croatia	2005	Fracture of pelvis	0.1823
Croatia	2010	Fracture of pelvis	0.1823
Croatia	2017	Fracture of pelvis	0.1823
Croatia	1990	Fracture of radius and/or ulna	0.0435
Croatia	1995	Fracture of radius and/or ulna	0.0435
Croatia	2000	Fracture of radius and/or ulna	0.0435
Croatia	2005	Fracture of radius and/or ulna	0.0435
Croatia	2010	Fracture of radius and/or ulna	0.0435
Croatia	2017	Fracture of radius and/or ulna	0.0435
Croatia	1990	Fracture of skull	0.0714
Croatia	1995	Fracture of skull	0.0714
Croatia	2000	Fracture of skull	0.0714
Croatia	2005	Fracture of skull	0.0714
Croatia	2010	Fracture of skull	0.0714
Croatia	2017	Fracture of skull	0.0714
Croatia	1990	Fracture of sternum and/or fracture of one or more ribs	0.1027
Croatia	1995	Fracture of sternum and/or fracture of one or more ribs	0.1027
Croatia	2000	Fracture of sternum and/or fracture of one or more ribs	0.1027
Croatia	2005	Fracture of sternum and/or fracture of one or more ribs	0.1027
Croatia	2010	Fracture of sternum and/or fracture of one or more ribs	0.1027
Croatia	2017	Fracture of sternum and/or fracture of one or more ribs	0.1027
Croatia	1990	Fracture of vertebral column	0.1106
Croatia	1995	Fracture of vertebral column	0.1106
Croatia	2000	Fracture of vertebral column	0.1106

Croatia	2005	Fracture of vertebral column	0.1106
Croatia	2010	Fracture of vertebral column	0.1106
Croatia	2017	Fracture of vertebral column	0.1106
Croatia	1990	Fracture of femur, other than femoral neck	0.042
Croatia	1995	Fracture of femur, other than femoral neck	0.042
Croatia	2000	Fracture of femur, other than femoral neck	0.042
Croatia	2005	Fracture of femur, other than femoral neck	0.042
Croatia	2010	Fracture of femur, other than femoral neck	0.042
Croatia	2017	Fracture of femur, other than femoral neck	0.042
Croatia	1990	Minor TBI	0.1319
Croatia	1995	Minor TBI	0.1319
Croatia	2000	Minor TBI	0.1319
Croatia	2005	Minor TBI	0.1319
Croatia	2010	Minor TBI	0.1319
Croatia	2017	Minor TBI	0.1319
Croatia	1990	Moderate TBI	0.1637
Croatia	1995	Moderate TBI	0.1637
Croatia	2000	Moderate TBI	0.1637
Croatia	2005	Moderate TBI	0.1637
Croatia	2010	Moderate TBI	0.1637
Croatia	2017	Moderate TBI	0.1637
Croatia	1990	Amputation of fingers (excluding thumb)	0.0047
Croatia	1995	Amputation of fingers (excluding thumb)	0.0047
Croatia	2000	Amputation of fingers (excluding thumb)	0.0047
Croatia	2005	Amputation of fingers (excluding thumb)	0.0047
Croatia	2010	Amputation of fingers (excluding thumb)	0.0047
Croatia	2017	Amputation of fingers (excluding thumb)	0.0047
Croatia	1990	Spinal cord lesion at neck level	0.4136
Croatia	1995	Spinal cord lesion at neck level	0.4125
Croatia	2000	Spinal cord lesion at neck level	0.4118
Croatia	2005	Spinal cord lesion at neck level	0.4118
Croatia	2010	Spinal cord lesion at neck level	0.4118
Croatia	2017	Spinal cord lesion at neck level	0.4118
Croatia	1990	Spinal cord lesion below neck level	0.1814
Croatia	1995	Spinal cord lesion below neck level	0.179
Croatia	2000	Spinal cord lesion below neck level	0.1777
Croatia	2005	Spinal cord lesion below neck level	0.1777
Croatia	2010	Spinal cord lesion below neck level	0.1777
Croatia	2017	Spinal cord lesion below neck level	0.1777
Croatia	1990	Drowning and nonfatal submersion	0.2471
Croatia	1995	Drowning and nonfatal submersion	0.2471
Croatia	2000	Drowning and nonfatal submersion	0.2471
Croatia	2005	Drowning and nonfatal submersion	0.2471
Croatia	2010	Drowning and nonfatal submersion	0.2471
Croatia	2017	Drowning and nonfatal submersion	0.2471
Croatia	1990	Asphyxiation	0.2471
Croatia	1995	Asphyxiation	0.2471
Croatia	2000	Asphyxiation	0.2471
Croatia	2005	Asphyxiation	0.2471
Croatia	2010	Asphyxiation	0.2471
Croatia	2017	Asphyxiation	0.2471
Croatia	1990	Crush injury	0.1325
Croatia	1995	Crush injury	0.1325
Croatia	2000	Crush injury	0.1325
Croatia	2005	Crush injury	0.1325
Croatia	2010	Crush injury	0.1325
Croatia	2017	Crush injury	0.1325
Croatia	1990	Nerve injury	0.1129
Croatia	1995	Nerve injury	0.1129
Croatia	2000	Nerve injury	0.1129
Croatia	2005	Nerve injury	0.1129
Croatia	2010	Nerve injury	0.1129
Croatia	2017	Nerve injury	0.1129
Croatia	1990	Injury to eyes	0.0165
Croatia	1995	Injury to eyes	0.0165
Croatia	2000	Injury to eyes	0.0165
Croatia	2005	Injury to eyes	0.0165
Croatia	2010	Injury to eyes	0.0165
Croatia	2017	Injury to eyes	0.0165
Croatia	1990	Amputation of lower limb, unilateral	0.041
Croatia	1995	Amputation of lower limb, unilateral	0.0397
Croatia	2000	Amputation of lower limb, unilateral	0.039
Croatia	2005	Amputation of lower limb, unilateral	0.039
Croatia	2010	Amputation of lower limb, unilateral	0.039
Croatia	2017	Amputation of lower limb, unilateral	0.039
Croatia	1990	Open wound(s)	0.011
Croatia	1995	Open wound(s)	0.011
Croatia	2000	Open wound(s)	0.011
Croatia	2005	Open wound(s)	0.011
Croatia	2010	Open wound(s)	0.011
Croatia	2017	Open wound(s)	0.011
Croatia	1990	Poisoning requiring urgent care	0.1628
Croatia	1995	Poisoning requiring urgent care	0.1628
Croatia	2000	Poisoning requiring urgent care	0.1628
Croatia	2005	Poisoning requiring urgent care	0.1628
Croatia	2010	Poisoning requiring urgent care	0.1628
Croatia	2017	Poisoning requiring urgent care	0.1628
Croatia	1990	Severe chest injury	0.0474
Croatia	1995	Severe chest injury	0.0474
Croatia	2000	Severe chest injury	0.0474
Croatia	2005	Severe chest injury	0.0474
Croatia	2010	Severe chest injury	0.0474
Croatia	2017	Severe chest injury	0.0474
Croatia	1990	Internal hemorrhage in abdomen and pelvis	0.1143
Croatia	1995	Internal hemorrhage in abdomen and pelvis	0.1143
Croatia	2000	Internal hemorrhage in abdomen and pelvis	0.1143
Croatia	2005	Internal hemorrhage in abdomen and pelvis	0.1143
Croatia	2010	Internal hemorrhage in abdomen and pelvis	0.1143
Croatia	2017	Internal hemorrhage in abdomen and pelvis	0.1143
Croatia	1990	Contusion in any part of the body	0.0075
Croatia	1995	Contusion in any part of the body	0.0075
Croatia	2000	Contusion in any part of the body	0.0075
Croatia	2005	Contusion in any part of the body	0.0075
Croatia	2010	Contusion in any part of the body	0.0075
Croatia	2017	Contusion in any part of the body	0.0075
Croatia	1990	Effect of different environmental factors	0.049
Croatia	1995	Effect of different environmental factors	0.049
Croatia	2000	Effect of different environmental factors	0.049
Croatia	2005	Effect of different environmental factors	0.049
Croatia	2010	Effect of different environmental factors	0.049
Croatia	2017	Effect of different environmental factors	0.049
Croatia	1990	Complications following therapeutic procedures	0.0506
Croatia	1995	Complications following therapeutic procedures	0.0506
Croatia	2000	Complications following therapeutic procedures	0.0506
Croatia	2005	Complications following therapeutic procedures	0.0506
Croatia	2010	Complications following therapeutic procedures	0.0506
Croatia	2017	Complications following therapeutic procedures	0.0506
Croatia	1990	Multiple fractures, dislocations, crashes, wounds, sprains, and strains	0.0628
Croatia	1995	Multiple fractures, dislocations, crashes, wounds, sprains, and strains	0.0595
Croatia	2000	Multiple fractures, dislocations, crashes, wounds, sprains, and strains	0.0577
Croatia	2005	Multiple fractures, dislocations, crashes, wounds, sprains, and strains	0.0577
Croatia	2010	Multiple fractures, dislocations, crashes, wounds, sprains, and strains	0.0577
Croatia	2017	Multiple fractures, dislocations, crashes, wounds, sprains, and strains	0.0577
Croatia	1990	Amputation of upper limb, unilateral	0.0402
Croatia	1995	Amputation of upper limb, unilateral	0.0394
Croatia	2000	Amputation of upper limb, unilateral	0.039
Croatia	2005	Amputation of upper limb, unilateral	0.039
Croatia	2010	Amputation of upper limb, unilateral	0.039
Croatia	2017	Amputation of upper limb, unilateral	0.039
Croatia	1990	Amputation of thumb	0.0114
Croatia	1995	Amputation of thumb	0.0114
Croatia	2000	Amputation of thumb	0.0114
Croatia	2005	Amputation of thumb	0.0114
Croatia	2010	Amputation of thumb	0.0114
Croatia	2017	Amputation of thumb	0.0114
Croatia	1990	Amputation of toe/toes	0.0059
Croatia	1995	Amputation of toe/toes	0.0059
Croatia	2000	Amputation of toe/toes	0.0059
Croatia	2005	Amputation of toe/toes	0.0059
Croatia	2010	Amputation of toe/toes	0.0059
Croatia	2017	Amputation of toe/toes	0.0059
Croatia	1990	Burns, <20% total burned surface area without lower airway burns	0.0163
Croatia	1995	Burns, <20% total burned surface area without lower airway burns	0.0163
Croatia	2000	Burns, <20% total burned surface area without lower airway burns	0.0163
Croatia	2005	Burns, <20% total burned surface area without lower airway burns	0.0163
Croatia	2010	Burns, <20% total burned surface area without lower airway burns	0.0163
Croatia	2017	Burns, <20% total burned surface area without lower airway burns	0.0163
Croatia	1990	Burns, >=20% total burned surface area or >= 10% burned surface area if head/neck or hands/wrist involved w/o lower airway burns	0.1394
Croatia	1995	Burns, >=20% total burned surface area or >= 10% burned surface area if head/neck or hands/wrist involved w/o lower airway burns	0.1363
Croatia	2000	Burns, >=20% total burned surface area or >= 10% burned surface area if head/neck or hands/wrist involved w/o lower airway burns	0.1346
Croatia	2005	Burns, >=20% total burned surface area or >= 10% burned surface area if head/neck or hands/wrist involved w/o lower airway burns	0.1346
Croatia	2010	Burns, >=20% total burned surface area or >= 10% burned surface area if head/neck or hands/wrist involved w/o lower airway burns	0.1346
Croatia	2017	Burns, >=20% total burned surface area or >= 10% burned surface area if head/neck or hands/wrist involved w/o lower airway burns	0.1346
Czech Republic	1990	Amputation of lower limbs, bilateral	0.1329
Czech Republic	1995	Amputation of lower limbs, bilateral	0.1232
Czech Republic	2000	Amputation of lower limbs, bilateral	0.1232
Czech Republic	2005	Amputation of lower limbs, bilateral	0.1232
Czech Republic	2010	Amputation of lower limbs, bilateral	0.1232
Czech Republic	2017	Amputation of lower limbs, bilateral	0.1232
Czech Republic	1990	Lower airway burns	0.2252
Czech Republic	1995	Lower airway burns	0.2252
Czech Republic	2000	Lower airway burns	0.2252
Czech Republic	2005	Lower airway burns	0.2252
Czech Republic	2010	Lower airway burns	0.2252
Czech Republic	2017	Lower airway burns	0.2252
Czech Republic	1990	Dislocation of hip	0.0159
Czech Republic	1995	Dislocation of hip	0.0159
Czech Republic	2000	Dislocation of hip	0.0159
Czech Republic	2005	Dislocation of hip	0.0159
Czech Republic	2010	Dislocation of hip	0.0159
Czech Republic	2017	Dislocation of hip	0.0159
Czech Republic	1990	Dislocation of knee	0.1134
Czech Republic	1995	Dislocation of knee	0.1134
Czech Republic	2000	Dislocation of knee	0.1134
Czech Republic	2005	Dislocation of knee	0.1134
Czech Republic	2010	Dislocation of knee	0.1134
Czech Republic	2017	Dislocation of knee	0.1134
Czech Republic	1990	Dislocation of shoulder	0.062
Czech Republic	1995	Dislocation of shoulder	0.062
Czech Republic	2000	Dislocation of shoulder	0.062
Czech Republic	2005	Dislocation of shoulder	0.062
Czech Republic	2010	Dislocation of shoulder	0.062
Czech Republic	2017	Dislocation of shoulder	0.062
Czech Republic	1990	Muscle and tendon injuries, including sprains and strains lesser dislocations	0.0075
Czech Republic	1995	Muscle and tendon injuries, including sprains and strains lesser dislocations	0.0075
Czech Republic	2000	Muscle and tendon injuries, including sprains and strains lesser dislocations	0.0075
Czech Republic	2005	Muscle and tendon injuries, including sprains and strains lesser dislocations	0.0075
Czech Republic	2010	Muscle and tendon injuries, including sprains and strains lesser dislocations	0.0075
Czech Republic	2017	Muscle and tendon injuries, including sprains and strains lesser dislocations	0.0075
Czech Republic	1990	Fracture of clavicle, scapula, or humerus	0.0349
Czech Republic	1995	Fracture of clavicle, scapula, or humerus	0.0349
Czech Republic	2000	Fracture of clavicle, scapula, or humerus	0.0349
Czech Republic	2005	Fracture of clavicle, scapula, or humerus	0.0349
Czech Republic	2010	Fracture of clavicle, scapula, or humerus	0.0349
Czech Republic	2017	Fracture of clavicle, scapula, or humerus	0.0349
Czech Republic	1990	Fracture of face bones	0.0669
Czech Republic	1995	Fracture of face bones	0.0669
Czech Republic	2000	Fracture of face bones	0.0669
Czech Republic	2005	Fracture of face bones	0.0669
Czech Republic	2010	Fracture of face bones	0.0669
Czech Republic	2017	Fracture of face bones	0.0669
Czech Republic	1990	Fracture of foot bones except ankle	0.0264
Czech Republic	1995	Fracture of foot bones except ankle	0.0264
Czech Republic	2000	Fracture of foot bones except ankle	0.0264
Czech Republic	2005	Fracture of foot bones except ankle	0.0264
Czech Republic	2010	Fracture of foot bones except ankle	0.0264
Czech Republic	2017	Fracture of foot bones except ankle	0.0264
Czech Republic	1990	Fracture of hand (wrist and other distal part of hand)	0.0138
Czech Republic	1995	Fracture of hand (wrist and other distal part of hand)	0.0138
Czech Republic	2000	Fracture of hand (wrist and other distal part of hand)	0.0138
Czech Republic	2005	Fracture of hand (wrist and other distal part of hand)	0.0138
Czech Republic	2010	Fracture of hand (wrist and other distal part of hand)	0.0138
Czech Republic	2017	Fracture of hand (wrist and other distal part of hand)	0.0138
Czech Republic	1990	Fracture of hip	0.0704
Czech Republic	1995	Fracture of hip	0.0577
Czech Republic	2000	Fracture of hip	0.0577
Czech Republic	2005	Fracture of hip	0.0577
Czech Republic	2010	Fracture of hip	0.0577
Czech Republic	2017	Fracture of hip	0.0577
Czech Republic	1990	Amputation of upper limbs, bilateral	0.1008
Czech Republic	1995	Amputation of upper limbs, bilateral	0.0876
Czech Republic	2000	Amputation of upper limbs, bilateral	0.0876
Czech Republic	2005	Amputation of upper limbs, bilateral	0.0876
Czech Republic	2010	Amputation of upper limbs, bilateral	0.0876
Czech Republic	2017	Amputation of upper limbs, bilateral	0.0876
Czech Republic	1990	Fracture of patella, tibia or fibula, or ankle	0.0554
Czech Republic	1995	Fracture of patella, tibia or fibula, or ankle	0.0554
Czech Republic	2000	Fracture of patella, tibia or fibula, or ankle	0.0554
Czech Republic	2005	Fracture of patella, tibia or fibula, or ankle	0.0554
Czech Republic	2010	Fracture of patella, tibia or fibula, or ankle	0.0554
Czech Republic	2017	Fracture of patella, tibia or fibula, or ankle	0.0554
Czech Republic	1990	Fracture of pelvis	0.1823
Czech Republic	1995	Fracture of pelvis	0.1823
Czech Republic	2000	Fracture of pelvis	0.1823
Czech Republic	2005	Fracture of pelvis	0.1823
Czech Republic	2010	Fracture of pelvis	0.1823
Czech Republic	2017	Fracture of pelvis	0.1823
Czech Republic	1990	Fracture of radius and/or ulna	0.0435
Czech Republic	1995	Fracture of radius and/or ulna	0.0435
Czech Republic	2000	Fracture of radius and/or ulna	0.0435
Czech Republic	2005	Fracture of radius and/or ulna	0.0435
Czech Republic	2010	Fracture of radius and/or ulna	0.0435
Czech Republic	2017	Fracture of radius and/or ulna	0.0435
Czech Republic	1990	Fracture of skull	0.0714
Czech Republic	1995	Fracture of skull	0.0714
Czech Republic	2000	Fracture of skull	0.0714
Czech Republic	2005	Fracture of skull	0.0714
Czech Republic	2010	Fracture of skull	0.0714
Czech Republic	2017	Fracture of skull	0.0714
Czech Republic	1990	Fracture of sternum and/or fracture of one or more ribs	0.1027
Czech Republic	1995	Fracture of sternum and/or fracture of one or more ribs	0.1027
Czech Republic	2000	Fracture of sternum and/or fracture of one or more ribs	0.1027
Czech Republic	2005	Fracture of sternum and/or fracture of one or more ribs	0.1027
Czech Republic	2010	Fracture of sternum and/or fracture of one or more ribs	0.1027
Czech Republic	2017	Fracture of sternum and/or fracture of one or more ribs	0.1027
Czech Republic	1990	Fracture of vertebral column	0.1106
Czech Republic	1995	Fracture of vertebral column	0.1106
Czech Republic	2000	Fracture of vertebral column	0.1106
Czech Republic	2005	Fracture of vertebral column	0.1106
Czech Republic	2010	Fracture of vertebral column	0.1106
Czech Republic	2017	Fracture of vertebral column	0.1106
Czech Republic	1990	Fracture of femur, other than femoral neck	0.042
Czech Republic	1995	Fracture of femur, other than femoral neck	0.042
Czech Republic	2000	Fracture of femur, other than femoral neck	0.042
Czech Republic	2005	Fracture of femur, other than femoral neck	0.042
Czech Republic	2010	Fracture of femur, other than femoral neck	0.042
Czech Republic	2017	Fracture of femur, other than femoral neck	0.042
Czech Republic	1990	Minor TBI	0.1319
Czech Republic	1995	Minor TBI	0.1319
Czech Republic	2000	Minor TBI	0.1319
Czech Republic	2005	Minor TBI	0.1319
Czech Republic	2010	Minor TBI	0.1319
Czech Republic	2017	Minor TBI	0.1319
Czech Republic	1990	Moderate TBI	0.1637
Czech Republic	1995	Moderate TBI	0.1637
Czech Republic	2000	Moderate TBI	0.1637
Czech Republic	2005	Moderate TBI	0.1637
Czech Republic	2010	Moderate TBI	0.1637
Czech Republic	2017	Moderate TBI	0.1637
Czech Republic	1990	Amputation of fingers (excluding thumb)	0.0047
Czech Republic	1995	Amputation of fingers (excluding thumb)	0.0047
Czech Republic	2000	Amputation of fingers (excluding thumb)	0.0047
Czech Republic	2005	Amputation of fingers (excluding thumb)	0.0047
Czech Republic	2010	Amputation of fingers (excluding thumb)	0.0047
Czech Republic	2017	Amputation of fingers (excluding thumb)	0.0047
Czech Republic	1990	Spinal cord lesion at neck level	0.4162
Czech Republic	1995	Spinal cord lesion at neck level	0.4118
Czech Republic	2000	Spinal cord lesion at neck level	0.4118
Czech Republic	2005	Spinal cord lesion at neck level	0.4118
Czech Republic	2010	Spinal cord lesion at neck level	0.4118
Czech Republic	2017	Spinal cord lesion at neck level	0.4118
Czech Republic	1990	Spinal cord lesion below neck level	0.187
Czech Republic	1995	Spinal cord lesion below neck level	0.1777
Czech Republic	2000	Spinal cord lesion below neck level	0.1777
Czech Republic	2005	Spinal cord lesion below neck level	0.1777
Czech Republic	2010	Spinal cord lesion below neck level	0.1777
Czech Republic	2017	Spinal cord lesion below neck level	0.1777
Czech Republic	1990	Drowning and nonfatal submersion	0.2471
Czech Republic	1995	Drowning and nonfatal submersion	0.2471
Czech Republic	2000	Drowning and nonfatal submersion	0.2471
Czech Republic	2005	Drowning and nonfatal submersion	0.2471
Czech Republic	2010	Drowning and nonfatal submersion	0.2471
Czech Republic	2017	Drowning and nonfatal submersion	0.2471
Czech Republic	1990	Asphyxiation	0.2471
Czech Republic	1995	Asphyxiation	0.2471
Czech Republic	2000	Asphyxiation	0.2471
Czech Republic	2005	Asphyxiation	0.2471
Czech Republic	2010	Asphyxiation	0.2471
Czech Republic	2017	Asphyxiation	0.2471
Czech Republic	1990	Crush injury	0.1325
Czech Republic	1995	Crush injury	0.1325
Czech Republic	2000	Crush injury	0.1325
Czech Republic	2005	Crush injury	0.1325
Czech Republic	2010	Crush injury	0.1325
Czech Republic	2017	Crush injury	0.1325
Czech Republic	1990	Nerve injury	0.1129
Czech Republic	1995	Nerve injury	0.1129
Czech Republic	2000	Nerve injury	0.1129
Czech Republic	2005	Nerve injury	0.1129
Czech Republic	2010	Nerve injury	0.1129
Czech Republic	2017	Nerve injury	0.1129
Czech Republic	1990	Injury to eyes	0.0165
Czech Republic	1995	Injury to eyes	0.0165
Czech Republic	2000	Injury to eyes	0.0165
Czech Republic	2005	Injury to eyes	0.0165
Czech Republic	2010	Injury to eyes	0.0165
Czech Republic	2017	Injury to eyes	0.0165
Czech Republic	1990	Amputation of lower limb, unilateral	0.0439
Czech Republic	1995	Amputation of lower limb, unilateral	0.039
Czech Republic	2000	Amputation of lower limb, unilateral	0.039
Czech Republic	2005	Amputation of lower limb, unilateral	0.039
Czech Republic	2010	Amputation of lower limb, unilateral	0.039
Czech Republic	2017	Amputation of lower limb, unilateral	0.039
Czech Republic	1990	Open wound(s)	0.011
Czech Republic	1995	Open wound(s)	0.011
Czech Republic	2000	Open wound(s)	0.011
Czech Republic	2005	Open wound(s)	0.011
Czech Republic	2010	Open wound(s)	0.011
Czech Republic	2017	Open wound(s)	0.011
Czech Republic	1990	Poisoning requiring urgent care	0.1628
Czech Republic	1995	Poisoning requiring urgent care	0.1628
Czech Republic	2000	Poisoning requiring urgent care	0.1628
Czech Republic	2005	Poisoning requiring urgent care	0.1628
Czech Republic	2010	Poisoning requiring urgent care	0.1628
Czech Republic	2017	Poisoning requiring urgent care	0.1628
Czech Republic	1990	Severe chest injury	0.0474
Czech Republic	1995	Severe chest injury	0.0474
Czech Republic	2000	Severe chest injury	0.0474
Czech Republic	2005	Severe chest injury	0.0474
Czech Republic	2010	Severe chest injury	0.0474
Czech Republic	2017	Severe chest injury	0.0474
Czech Republic	1990	Internal hemorrhage in abdomen and pelvis	0.1143
Czech Republic	1995	Internal hemorrhage in abdomen and pelvis	0.1143
Czech Republic	2000	Internal hemorrhage in abdomen and pelvis	0.1143
Czech Republic	2005	Internal hemorrhage in abdomen and pelvis	0.1143
Czech Republic	2010	Internal hemorrhage in abdomen and pelvis	0.1143
Czech Republic	2017	Internal hemorrhage in abdomen and pelvis	0.1143
Czech Republic	1990	Contusion in any part of the body	0.0075
Czech Republic	1995	Contusion in any part of the body	0.0075
Czech Republic	2000	Contusion in any part of the body	0.0075
Czech Republic	2005	Contusion in any part of the body	0.0075
Czech Republic	2010	Contusion in any part of the body	0.0075
Czech Republic	2017	Contusion in any part of the body	0.0075
Czech Republic	1990	Effect of different environmental factors	0.049
Czech Republic	1995	Effect of different environmental factors	0.049
Czech Republic	2000	Effect of different environmental factors	0.049
Czech Republic	2005	Effect of different environmental factors	0.049
Czech Republic	2010	Effect of different environmental factors	0.049
Czech Republic	2017	Effect of different environmental factors	0.049
Czech Republic	1990	Complications following therapeutic procedures	0.0506
Czech Republic	1995	Complications following therapeutic procedures	0.0506
Czech Republic	2000	Complications following therapeutic procedures	0.0506
Czech Republic	2005	Complications following therapeutic procedures	0.0506
Czech Republic	2010	Complications following therapeutic procedures	0.0506
Czech Republic	2017	Complications following therapeutic procedures	0.0506
Czech Republic	1990	Multiple fractures, dislocations, crashes, wounds, sprains, and strains	0.0704
Czech Republic	1995	Multiple fractures, dislocations, crashes, wounds, sprains, and strains	0.0577
Czech Republic	2000	Multiple fractures, dislocations, crashes, wounds, sprains, and strains	0.0577
Czech Republic	2005	Multiple fractures, dislocations, crashes, wounds, sprains, and strains	0.0577
Czech Republic	2010	Multiple fractures, dislocations, crashes, wounds, sprains, and strains	0.0577
Czech Republic	2017	Multiple fractures, dislocations, crashes, wounds, sprains, and strains	0.0577
Czech Republic	1990	Amputation of upper limb, unilateral	0.0419
Czech Republic	1995	Amputation of upper limb, unilateral	0.039
Czech Republic	2000	Amputation of upper limb, unilateral	0.039
Czech Republic	2005	Amputation of upper limb, unilateral	0.039
Czech Republic	2010	Amputation of upper limb, unilateral	0.039
Czech Republic	2017	Amputation of upper limb, unilateral	0.039
Czech Republic	1990	Amputation of thumb	0.0114
Czech Republic	1995	Amputation of thumb	0.0114
Czech Republic	2000	Amputation of thumb	0.0114
Czech Republic	2005	Amputation of thumb	0.0114
Czech Republic	2010	Amputation of thumb	0.0114
Czech Republic	2017	Amputation of thumb	0.0114
Czech Republic	1990	Amputation of toe/toes	0.0059
Czech Republic	1995	Amputation of toe/toes	0.0059
Czech Republic	2000	Amputation of toe/toes	0.0059
Czech Republic	2005	Amputation of toe/toes	0.0059
Czech Republic	2010	Amputation of toe/toes	0.0059
Czech Republic	2017	Amputation of toe/toes	0.0059
Czech Republic	1990	Burns, <20% total burned surface area without lower airway burns	0.0163
Czech Republic	1995	Burns, <20% total burned surface area without lower airway burns	0.0163
Czech Republic	2000	Burns, <20% total burned surface area without lower airway burns	0.0163
Czech Republic	2005	Burns, <20% total burned surface area without lower airway burns	0.0163
Czech Republic	2010	Burns, <20% total burned surface area without lower airway burns	0.0163
Czech Republic	2017	Burns, <20% total burned surface area without lower airway burns	0.0163
Czech Republic	1990	Burns, >=20% total burned surface area or >= 10% burned surface area if head/neck or hands/wrist involved w/o lower airway burns	0.1464
Czech Republic	1995	Burns, >=20% total burned surface area or >= 10% burned surface area if head/neck or hands/wrist involved w/o lower airway burns	0.1346
Czech Republic	2000	Burns, >=20% total burned surface area or >= 10% burned surface area if head/neck or hands/wrist involved w/o lower airway burns	0.1346
Czech Republic	2005	Burns, >=20% total burned surface area or >= 10% burned surface area if head/neck or hands/wrist involved w/o lower airway burns	0.1346
Czech Republic	2010	Burns, >=20% total burned surface area or >= 10% burned surface area if head/neck or hands/wrist involved w/o lower airway burns	0.1346
Czech Republic	2017	Burns, >=20% total burned surface area or >= 10% burned surface area if head/neck or hands/wrist involved w/o lower airway burns	0.1346
Hungary	1990	Amputation of lower limbs, bilateral	0.1522
Hungary	1995	Amputation of lower limbs, bilateral	0.139
Hungary	2000	Amputation of lower limbs, bilateral	0.125
Hungary	2005	Amputation of lower limbs, bilateral	0.1232
Hungary	2010	Amputation of lower limbs, bilateral	0.1232
Hungary	2017	Amputation of lower limbs, bilateral	0.1232
Hungary	1990	Lower airway burns	0.2252
Hungary	1995	Lower airway burns	0.2252
Hungary	2000	Lower airway burns	0.2252
Hungary	2005	Lower airway burns	0.2252
Hungary	2010	Lower airway burns	0.2252
Hungary	2017	Lower airway burns	0.2252
Hungary	1990	Dislocation of hip	0.0159
Hungary	1995	Dislocation of hip	0.0159
Hungary	2000	Dislocation of hip	0.0159
Hungary	2005	Dislocation of hip	0.0159
Hungary	2010	Dislocation of hip	0.0159
Hungary	2017	Dislocation of hip	0.0159
Hungary	1990	Dislocation of knee	0.1134
Hungary	1995	Dislocation of knee	0.1134

Hungary	2000	Dislocation of knee	0.1134
Hungary	2005	Dislocation of knee	0.1134
Hungary	2010	Dislocation of knee	0.1134
Hungary	2017	Dislocation of knee	0.1134
Hungary	1990	Dislocation of shoulder	0.062
Hungary	1995	Dislocation of shoulder	0.062
Hungary	2000	Dislocation of shoulder	0.062
Hungary	2005	Dislocation of shoulder	0.062
Hungary	2010	Dislocation of shoulder	0.062
Hungary	2017	Dislocation of shoulder	0.062
Hungary	1990	Muscle and tendon injuries, including sprains and strains lesser dislocations	0.0075
Hungary	1995	Muscle and tendon injuries, including sprains and strains lesser dislocations	0.0075
Hungary	2000	Muscle and tendon injuries, including sprains and strains lesser dislocations	0.0075
Hungary	2005	Muscle and tendon injuries, including sprains and strains lesser dislocations	0.0075
Hungary	2010	Muscle and tendon injuries, including sprains and strains lesser dislocations	0.0075
Hungary	2017	Muscle and tendon injuries, including sprains and strains lesser dislocations	0.0075
Hungary	1990	Fracture of clavicle, scapula, or humerus	0.0349
Hungary	1995	Fracture of clavicle, scapula, or humerus	0.0349
Hungary	2000	Fracture of clavicle, scapula, or humerus	0.0349
Hungary	2005	Fracture of clavicle, scapula, or humerus	0.0349
Hungary	2010	Fracture of clavicle, scapula, or humerus	0.0349
Hungary	2017	Fracture of clavicle, scapula, or humerus	0.0349
Hungary	1990	Fracture of face bones	0.0669
Hungary	1995	Fracture of face bones	0.0669
Hungary	2000	Fracture of face bones	0.0669
Hungary	2005	Fracture of face bones	0.0669
Hungary	2010	Fracture of face bones	0.0669
Hungary	2017	Fracture of face bones	0.0669
Hungary	1990	Fracture of foot bones except ankle	0.0264
Hungary	1995	Fracture of foot bones except ankle	0.0264
Hungary	2000	Fracture of foot bones except ankle	0.0264
Hungary	2005	Fracture of foot bones except ankle	0.0264
Hungary	2010	Fracture of foot bones except ankle	0.0264
Hungary	2017	Fracture of foot bones except ankle	0.0264
Hungary	1990	Fracture of hand (wrist and other distal part of hand)	0.0138
Hungary	1995	Fracture of hand (wrist and other distal part of hand)	0.0138
Hungary	2000	Fracture of hand (wrist and other distal part of hand)	0.0138
Hungary	2005	Fracture of hand (wrist and other distal part of hand)	0.0138
Hungary	2010	Fracture of hand (wrist and other distal part of hand)	0.0138
Hungary	2017	Fracture of hand (wrist and other distal part of hand)	0.0138
Hungary	1990	Fracture of hip	0.0959
Hungary	1995	Fracture of hip	0.0785
Hungary	2000	Fracture of hip	0.06
Hungary	2005	Fracture of hip	0.0577
Hungary	2010	Fracture of hip	0.0577
Hungary	2017	Fracture of hip	0.0577
Hungary	1990	Amputation of upper limbs, bilateral	0.1271
Hungary	1995	Amputation of upper limbs, bilateral	0.1092
Hungary	2000	Amputation of upper limbs, bilateral	0.09
Hungary	2005	Amputation of upper limbs, bilateral	0.0876
Hungary	2010	Amputation of upper limbs, bilateral	0.0876
Hungary	2017	Amputation of upper limbs, bilateral	0.0876
Hungary	1990	Fracture of patella, tibia or fibula, or ankle	0.0554
Hungary	1995	Fracture of patella, tibia or fibula, or ankle	0.0554
Hungary	2000	Fracture of patella, tibia or fibula, or ankle	0.0554
Hungary	2005	Fracture of patella, tibia or fibula, or ankle	0.0554
Hungary	2010	Fracture of patella, tibia or fibula, or ankle	0.0554
Hungary	2017	Fracture of patella, tibia or fibula, or ankle	0.0554
Hungary	1990	Fracture of pelvis	0.1823
Hungary	1995	Fracture of pelvis	0.1823
Hungary	2000	Fracture of pelvis	0.1823
Hungary	2005	Fracture of pelvis	0.1823
Hungary	2010	Fracture of pelvis	0.1823
Hungary	2017	Fracture of pelvis	0.1823
Hungary	1990	Fracture of radius and/or ulna	0.0435
Hungary	1995	Fracture of radius and/or ulna	0.0435
Hungary	2000	Fracture of radius and/or ulna	0.0435
Hungary	2005	Fracture of radius and/or ulna	0.0435
Hungary	2010	Fracture of radius and/or ulna	0.0435
Hungary	2017	Fracture of radius and/or ulna	0.0435
Hungary	1990	Fracture of skull	0.0714
Hungary	1995	Fracture of skull	0.0714
Hungary	2000	Fracture of skull	0.0714
Hungary	2005	Fracture of skull	0.0714
Hungary	2010	Fracture of skull	0.0714
Hungary	2017	Fracture of skull	0.0714
Hungary	1990	Fracture of sternum and/or fracture of one or more ribs	0.1027
Hungary	1995	Fracture of sternum and/or fracture of one or more ribs	0.1027
Hungary	2000	Fracture of sternum and/or fracture of one or more ribs	0.1027
Hungary	2005	Fracture of sternum and/or fracture of one or more ribs	0.1027
Hungary	2010	Fracture of sternum and/or fracture of one or more ribs	0.1027
Hungary	2017	Fracture of sternum and/or fracture of one or more ribs	0.1027
Hungary	1990	Fracture of vertebral column	0.1106
Hungary	1995	Fracture of vertebral column	0.1106
Hungary	2000	Fracture of vertebral column	0.1106
Hungary	2005	Fracture of vertebral column	0.1106
Hungary	2010	Fracture of vertebral column	0.1106
Hungary	2017	Fracture of vertebral column	0.1106
Hungary	1990	Fracture of femur, other than femoral neck	0.042
Hungary	1995	Fracture of femur, other than femoral neck	0.042
Hungary	2000	Fracture of femur, other than femoral neck	0.042
Hungary	2005	Fracture of femur, other than femoral neck	0.042
Hungary	2010	Fracture of femur, other than femoral neck	0.042
Hungary	2017	Fracture of femur, other than femoral neck	0.042
Hungary	1990	Minor TBI	0.1319
Hungary	1995	Minor TBI	0.1319
Hungary	2000	Minor TBI	0.1319
Hungary	2005	Minor TBI	0.1319
Hungary	2010	Minor TBI	0.1319
Hungary	2017	Minor TBI	0.1319
Hungary	1990	Moderate TBI	0.1637
Hungary	1995	Moderate TBI	0.1637
Hungary	2000	Moderate TBI	0.1637
Hungary	2005	Moderate TBI	0.1637
Hungary	2010	Moderate TBI	0.1637
Hungary	2017	Moderate TBI	0.1637
Hungary	1990	Amputation of fingers (excluding thumb)	0.0047
Hungary	1995	Amputation of fingers (excluding thumb)	0.0047
Hungary	2000	Amputation of fingers (excluding thumb)	0.0047
Hungary	2005	Amputation of fingers (excluding thumb)	0.0047
Hungary	2010	Amputation of fingers (excluding thumb)	0.0047
Hungary	2017	Amputation of fingers (excluding thumb)	0.0047
Hungary	1990	Spinal cord lesion at neck level	0.425
Hungary	1995	Spinal cord lesion at neck level	0.419
Hungary	2000	Spinal cord lesion at neck level	0.4126
Hungary	2005	Spinal cord lesion at neck level	0.4118
Hungary	2010	Spinal cord lesion at neck level	0.4118
Hungary	2017	Spinal cord lesion at neck level	0.4118
Hungary	1990	Spinal cord lesion below neck level	0.2056
Hungary	1995	Spinal cord lesion below neck level	0.1929
Hungary	2000	Spinal cord lesion below neck level	0.1793
Hungary	2005	Spinal cord lesion below neck level	0.1777
Hungary	2010	Spinal cord lesion below neck level	0.1777
Hungary	2017	Spinal cord lesion below neck level	0.1777
Hungary	1990	Drowning and nonfatal submersion	0.2471
Hungary	1995	Drowning and nonfatal submersion	0.2471
Hungary	2000	Drowning and nonfatal submersion	0.2471
Hungary	2005	Drowning and nonfatal submersion	0.2471
Hungary	2010	Drowning and nonfatal submersion	0.2471
Hungary	2017	Drowning and nonfatal submersion	0.2471
Hungary	1990	Asphyxiation	0.2471
Hungary	1995	Asphyxiation	0.2471
Hungary	2000	Asphyxiation	0.2471
Hungary	2005	Asphyxiation	0.2471
Hungary	2010	Asphyxiation	0.2471
Hungary	2017	Asphyxiation	0.2471
Hungary	1990	Crush injury	0.1325
Hungary	1995	Crush injury	0.1325
Hungary	2000	Crush injury	0.1325
Hungary	2005	Crush injury	0.1325
Hungary	2010	Crush injury	0.1325
Hungary	2017	Crush injury	0.1325
Hungary	1990	Nerve injury	0.1129
Hungary	1995	Nerve injury	0.1129
Hungary	2000	Nerve injury	0.1129
Hungary	2005	Nerve injury	0.1129
Hungary	2010	Nerve injury	0.1129
Hungary	2017	Nerve injury	0.1129
Hungary	1990	Injury to eyes	0.0165
Hungary	1995	Injury to eyes	0.0165
Hungary	2000	Injury to eyes	0.0165
Hungary	2005	Injury to eyes	0.0165
Hungary	2010	Injury to eyes	0.0165
Hungary	2017	Injury to eyes	0.0165
Hungary	1990	Amputation of lower limb, unilateral	0.0539
Hungary	1995	Amputation of lower limb, unilateral	0.0471
Hungary	2000	Amputation of lower limb, unilateral	0.0399
Hungary	2005	Amputation of lower limb, unilateral	0.039
Hungary	2010	Amputation of lower limb, unilateral	0.039
Hungary	2017	Amputation of lower limb, unilateral	0.039
Hungary	1990	Open wound(s)	0.011
Hungary	1995	Open wound(s)	0.011
Hungary	2000	Open wound(s)	0.011
Hungary	2005	Open wound(s)	0.011
Hungary	2010	Open wound(s)	0.011
Hungary	2017	Open wound(s)	0.011
Hungary	1990	Poisoning requiring urgent care	0.1628
Hungary	1995	Poisoning requiring urgent care	0.1628
Hungary	2000	Poisoning requiring urgent care	0.1628
Hungary	2005	Poisoning requiring urgent care	0.1628
Hungary	2010	Poisoning requiring urgent care	0.1628
Hungary	2017	Poisoning requiring urgent care	0.1628
Hungary	1990	Severe chest injury	0.0474
Hungary	1995	Severe chest injury	0.0474
Hungary	2000	Severe chest injury	0.0474
Hungary	2005	Severe chest injury	0.0474
Hungary	2010	Severe chest injury	0.0474
Hungary	2017	Severe chest injury	0.0474
Hungary	1990	Internal hemorrhage in abdomen and pelvis	0.1143
Hungary	1995	Internal hemorrhage in abdomen and pelvis	0.1143
Hungary	2000	Internal hemorrhage in abdomen and pelvis	0.1143
Hungary	2005	Internal hemorrhage in abdomen and pelvis	0.1143
Hungary	2010	Internal hemorrhage in abdomen and pelvis	0.1143
Hungary	2017	Internal hemorrhage in abdomen and pelvis	0.1143
Hungary	1990	Contusion in any part of the body	0.0075
Hungary	1995	Contusion in any part of the body	0.0075
Hungary	2000	Contusion in any part of the body	0.0075
Hungary	2005	Contusion in any part of the body	0.0075
Hungary	2010	Contusion in any part of the body	0.0075
Hungary	2017	Contusion in any part of the body	0.0075
Hungary	1990	Effect of different environmental factors	0.049
Hungary	1995	Effect of different environmental factors	0.049
Hungary	2000	Effect of different environmental factors	0.049
Hungary	2005	Effect of different environmental factors	0.049
Hungary	2010	Effect of different environmental factors	0.049
Hungary	2017	Effect of different environmental factors	0.049
Hungary	1990	Complications following therapeutic procedures	0.0506
Hungary	1995	Complications following therapeutic procedures	0.0506
Hungary	2000	Complications following therapeutic procedures	0.0506
Hungary	2005	Complications following therapeutic procedures	0.0506
Hungary	2010	Complications following therapeutic procedures	0.0506
Hungary	2017	Complications following therapeutic procedures	0.0506
Hungary	1990	Multiple fractures, dislocations, crashes, wounds, sprains, and strains	0.0959
Hungary	1995	Multiple fractures, dislocations, crashes, wounds, sprains, and strains	0.0785
Hungary	2000	Multiple fractures, dislocations, crashes, wounds, sprains, and strains	0.06
Hungary	2005	Multiple fractures, dislocations, crashes, wounds, sprains, and strains	0.0577
Hungary	2010	Multiple fractures, dislocations, crashes, wounds, sprains, and strains	0.0577
Hungary	2017	Multiple fractures, dislocations, crashes, wounds, sprains, and strains	0.0577
Hungary	1990	Amputation of upper limb, unilateral	0.0478
Hungary	1995	Amputation of upper limb, unilateral	0.0438
Hungary	2000	Amputation of upper limb, unilateral	0.0395
Hungary	2005	Amputation of upper limb, unilateral	0.039
Hungary	2010	Amputation of upper limb, unilateral	0.039
Hungary	2017	Amputation of upper limb, unilateral	0.039
Hungary	1990	Amputation of thumb	0.0114
Hungary	1995	Amputation of thumb	0.0114
Hungary	2000	Amputation of thumb	0.0114
Hungary	2005	Amputation of thumb	0.0114
Hungary	2010	Amputation of thumb	0.0114
Hungary	2017	Amputation of thumb	0.0114
Hungary	1990	Amputation of toe/toes	0.0059
Hungary	1995	Amputation of toe/toes	0.0059
Hungary	2000	Amputation of toe/toes	0.0059
Hungary	2005	Amputation of toe/toes	0.0059
Hungary	2010	Amputation of toe/toes	0.0059
Hungary	2017	Amputation of toe/toes	0.0059
Hungary	1990	Burns, <20% total burned surface area without lower airway burns	0.0163
Hungary	1995	Burns, <20% total burned surface area without lower airway burns	0.0163
Hungary	2000	Burns, <20% total burned surface area without lower airway burns	0.0163
Hungary	2005	Burns, <20% total burned surface area without lower airway burns	0.0163
Hungary	2010	Burns, <20% total burned surface area without lower airway burns	0.0163
Hungary	2017	Burns, <20% total burned surface area without lower airway burns	0.0163
Hungary	1990	Burns, >=20% total burned surface area or >= 10% burned surface area if head/neck or hands/wrist involved w/o lower airway burns	0.1702
Hungary	1995	Burns, >=20% total burned surface area or >= 10% burned surface area if head/neck or hands/wrist involved w/o lower airway burns	0.154
Hungary	2000	Burns, >=20% total burned surface area or >= 10% burned surface area if head/neck or hands/wrist involved w/o lower airway burns	0.1367
Hungary	2005	Burns, >=20% total burned surface area or >= 10% burned surface area if head/neck or hands/wrist involved w/o lower airway burns	0.1346
Hungary	2010	Burns, >=20% total burned surface area or >= 10% burned surface area if head/neck or hands/wrist involved w/o lower airway burns	0.1346
Hungary	2017	Burns, >=20% total burned surface area or >= 10% burned surface area if head/neck or hands/wrist involved w/o lower airway burns	0.1346
North Macedonia	1990	Amputation of lower limbs, bilateral	0.1761
North Macedonia	1995	Amputation of lower limbs, bilateral	0.1686
North Macedonia	2000	Amputation of lower limbs, bilateral	0.1562
North Macedonia	2005	Amputation of lower limbs, bilateral	0.1443
North Macedonia	2010	Amputation of lower limbs, bilateral	0.1301
North Macedonia	2017	Amputation of lower limbs, bilateral	0.1234
North Macedonia	1990	Lower airway burns	0.2252
North Macedonia	1995	Lower airway burns	0.2252
North Macedonia	2000	Lower airway burns	0.2252
North Macedonia	2005	Lower airway burns	0.2252
North Macedonia	2010	Lower airway burns	0.2252
North Macedonia	2017	Lower airway burns	0.2252
North Macedonia	1990	Dislocation of hip	0.0159
North Macedonia	1995	Dislocation of hip	0.0159
North Macedonia	2000	Dislocation of hip	0.0159
North Macedonia	2005	Dislocation of hip	0.0159
North Macedonia	2010	Dislocation of hip	0.0159
North Macedonia	2017	Dislocation of hip	0.0159
North Macedonia	1990	Dislocation of knee	0.1134
North Macedonia	1995	Dislocation of knee	0.1134
North Macedonia	2000	Dislocation of knee	0.1134
North Macedonia	2005	Dislocation of knee	0.1134
North Macedonia	2010	Dislocation of knee	0.1134
North Macedonia	2017	Dislocation of knee	0.1134
North Macedonia	1990	Dislocation of shoulder	0.062
North Macedonia	1995	Dislocation of shoulder	0.062
North Macedonia	2000	Dislocation of shoulder	0.062
North Macedonia	2005	Dislocation of shoulder	0.062
North Macedonia	2010	Dislocation of shoulder	0.062
North Macedonia	2017	Dislocation of shoulder	0.062
North Macedonia	1990	Muscle and tendon injuries, including sprains and strains lesser dislocations	0.0075
North Macedonia	1995	Muscle and tendon injuries, including sprains and strains lesser dislocations	0.0075
North Macedonia	2000	Muscle and tendon injuries, including sprains and strains lesser dislocations	0.0075
North Macedonia	2005	Muscle and tendon injuries, including sprains and strains lesser dislocations	0.0075
North Macedonia	2010	Muscle and tendon injuries, including sprains and strains lesser dislocations	0.0075
North Macedonia	2017	Muscle and tendon injuries, including sprains and strains lesser dislocations	0.0075
North Macedonia	1990	Fracture of clavicle, scapula, or humerus	0.0349
North Macedonia	1995	Fracture of clavicle, scapula, or humerus	0.0349
North Macedonia	2000	Fracture of clavicle, scapula, or humerus	0.0349
North Macedonia	2005	Fracture of clavicle, scapula, or humerus	0.0349
North Macedonia	2010	Fracture of clavicle, scapula, or humerus	0.0349
North Macedonia	2017	Fracture of clavicle, scapula, or humerus	0.0349
North Macedonia	1990	Fracture of face bones	0.0669
North Macedonia	1995	Fracture of face bones	0.0669
North Macedonia	2000	Fracture of face bones	0.0669
North Macedonia	2005	Fracture of face bones	0.0669
North Macedonia	2010	Fracture of face bones	0.0669
North Macedonia	2017	Fracture of face bones	0.0669
North Macedonia	1990	Fracture of foot bones except ankle	0.0264
North Macedonia	1995	Fracture of foot bones except ankle	0.0264
North Macedonia	2000	Fracture of foot bones except ankle	0.0264
North Macedonia	2005	Fracture of foot bones except ankle	0.0264
North Macedonia	2010	Fracture of foot bones except ankle	0.0264
North Macedonia	2017	Fracture of foot bones except ankle	0.0264
North Macedonia	1990	Fracture of hand (wrist and other distal part of hand)	0.0138
North Macedonia	1995	Fracture of hand (wrist and other distal part of hand)	0.0138
North Macedonia	2000	Fracture of hand (wrist and other distal part of hand)	0.0138
North Macedonia	2005	Fracture of hand (wrist and other distal part of hand)	0.0138
North Macedonia	2010	Fracture of hand (wrist and other distal part of hand)	0.0138
North Macedonia	2017	Fracture of hand (wrist and other distal part of hand)	0.0138
North Macedonia	1990	Fracture of hip	0.1275
North Macedonia	1995	Fracture of hip	0.1176
North Macedonia	2000	Fracture of hip	0.1012
North Macedonia	2005	Fracture of hip	0.0856
North Macedonia	2010	Fracture of hip	0.0667
North Macedonia	2017	Fracture of hip	0.0579
North Macedonia	1990	Amputation of upper limbs, bilateral	0.1588
North Macedonia	1995	Amputation of upper limbs, bilateral	0.1495
North Macedonia	2000	Amputation of upper limbs, bilateral	0.1326
North Macedonia	2005	Amputation of upper limbs, bilateral	0.1164
North Macedonia	2010	Amputation of upper limbs, bilateral	0.097
North Macedonia	2017	Amputation of upper limbs, bilateral	0.0879
North Macedonia	1990	Fracture of patella, tibia or fibula, or ankle	0.0554
North Macedonia	1995	Fracture of patella, tibia or fibula, or ankle	0.0554
North Macedonia	2000	Fracture of patella, tibia or fibula, or ankle	0.0554
North Macedonia	2005	Fracture of patella, tibia or fibula, or ankle	0.0554
North Macedonia	2010	Fracture of patella, tibia or fibula, or ankle	0.0554
North Macedonia	2017	Fracture of patella, tibia or fibula, or ankle	0.0554
North Macedonia	1990	Fracture of pelvis	0.1823
North Macedonia	1995	Fracture of pelvis	0.1823
North Macedonia	2000	Fracture of pelvis	0.1823
North Macedonia	2005	Fracture of pelvis	0.1823
North Macedonia	2010	Fracture of pelvis	0.1823
North Macedonia	2017	Fracture of pelvis	0.1823
North Macedonia	1990	Fracture of radius and/or ulna	0.0435
North Macedonia	1995	Fracture of radius and/or ulna	0.0435
North Macedonia	2000	Fracture of radius and/or ulna	0.0435
North Macedonia	2005	Fracture of radius and/or ulna	0.0435
North Macedonia	2010	Fracture of radius and/or ulna	0.0435
North Macedonia	2017	Fracture of radius and/or ulna	0.0435
North Macedonia	1990	Fracture of skull	0.0714
North Macedonia	1995	Fracture of skull	0.0714
North Macedonia	2000	Fracture of skull	0.0714
North Macedonia	2005	Fracture of skull	0.0714
North Macedonia	2010	Fracture of skull	0.0714
North Macedonia	2017	Fracture of skull	0.0714
North Macedonia	1990	Fracture of sternum and/or fracture of one or more ribs	0.1027
North Macedonia	1995	Fracture of sternum and/or fracture of one or more ribs	0.1027
North Macedonia	2000	Fracture of sternum and/or fracture of one or more ribs	0.1027
North Macedonia	2005	Fracture of sternum and/or fracture of one or more ribs	0.1027
North Macedonia	2010	Fracture of sternum and/or fracture of one or more ribs	0.1027
North Macedonia	2017	Fracture of sternum and/or fracture of one or more ribs	0.1027
North Macedonia	1990	Fracture of vertebral column	0.1106
North Macedonia	1995	Fracture of vertebral column	0.1106
North Macedonia	2000	Fracture of vertebral column	0.1106
North Macedonia	2005	Fracture of vertebral column	0.1106
North Macedonia	2010	Fracture of vertebral column	0.1106
North Macedonia	2017	Fracture of vertebral column	0.1106
North Macedonia	1990	Fracture of femur, other than femoral neck	0.042
North Macedonia	1995	Fracture of femur, other than femoral neck	0.042
North Macedonia	2000	Fracture of femur, other than femoral neck	0.042
North Macedonia	2005	Fracture of femur, other than femoral neck	0.042
North Macedonia	2010	Fracture of femur, other than femoral neck	0.042
North Macedonia	2017	Fracture of femur, other than femoral neck	0.042
North Macedonia	1990	Minor TBI	0.1319
North Macedonia	1995	Minor TBI	0.1319
North Macedonia	2000	Minor TBI	0.1319
North Macedonia	2005	Minor TBI	0.1319
North Macedonia	2010	Minor TBI	0.1319
North Macedonia	2017	Minor TBI	0.1319
North Macedonia	1990	Moderate TBI	0.1637
North Macedonia	1995	Moderate TBI	0.1637
North Macedonia	2000	Moderate TBI	0.1637
North Macedonia	2005	Moderate TBI	0.1637
North Macedonia	2010	Moderate TBI	0.1637
North Macedonia	2017	Moderate TBI	0.1637
North Macedonia	1990	Amputation of fingers (excluding thumb)	0.0047
North Macedonia	1995	Amputation of fingers (excluding thumb)	0.0047
North Macedonia	2000	Amputation of fingers (excluding thumb)	0.0047
North Macedonia	2005	Amputation of fingers (excluding thumb)	0.0047
North Macedonia	2010	Amputation of fingers (excluding thumb)	0.0047
North Macedonia	2017	Amputation of fingers (excluding thumb)	0.0047
North Macedonia	1990	Spinal cord lesion at neck level	0.436
North Macedonia	1995	Spinal cord lesion at neck level	0.4325
North Macedonia	2000	Spinal cord lesion at neck level	0.4269
North Macedonia	2005	Spinal cord lesion at neck level	0.4215
North Macedonia	2010	Spinal cord lesion at neck level	0.4149
North Macedonia	2017	Spinal cord lesion at neck level	0.4119
North Macedonia	1990	Spinal cord lesion below neck level	0.2287
North Macedonia	1995	Spinal cord lesion below neck level	0.2214
North Macedonia	2000	Spinal cord lesion below neck level	0.2095
North Macedonia	2005	Spinal cord lesion below neck level	0.198
North Macedonia	2010	Spinal cord lesion below neck level	0.1843
North Macedonia	2017	Spinal cord lesion below neck level	0.1778
North Macedonia	1990	Drowning and nonfatal submersion	0.2471
North Macedonia	1995	Drowning and nonfatal submersion	0.2471
North Macedonia	2000	Drowning and nonfatal submersion	0.2471
North Macedonia	2005	Drowning and nonfatal submersion	0.2471
North Macedonia	2010	Drowning and nonfatal submersion	0.2471
North Macedonia	2017	Drowning and nonfatal submersion	0.2471
North Macedonia	1990	Asphyxiation	0.2471
North Macedonia	1995	Asphyxiation	0.2471
North Macedonia	2000	Asphyxiation	0.2471
North Macedonia	2005	Asphyxiation	0.2471
North Macedonia	2010	Asphyxiation	0.2471
North Macedonia	2017	Asphyxiation	0.2471
North Macedonia	1990	Crush injury	0.1325
North Macedonia	1995	Crush injury	0.1325
North Macedonia	2000	Crush injury	0.1325
North Macedonia	2005	Crush injury	0.1325
North Macedonia	2010	Crush injury	0.1325
North Macedonia	2017	Crush injury	0.1325
North Macedonia	1990	Nerve injury	0.1129
North Macedonia	1995	Nerve injury	0.1129
North Macedonia	2000	Nerve injury	0.1129
North Macedonia	2005	Nerve injury	0.1129
North Macedonia	2010	Nerve injury	0.1129
North Macedonia	2017	Nerve injury	0.1129
North Macedonia	1990	Injury to eyes	0.0165
North Macedonia	1995	Injury to eyes	0.0165
North Macedonia	2000	Injury to eyes	0.0165
North Macedonia	2005	Injury to eyes	0.0165
North Macedonia	2010	Injury to eyes	0.0165
North Macedonia	2017	Injury to eyes	0.0165
North Macedonia	1990	Amputation of lower limb, unilateral	0.0662
North Macedonia	1995	Amputation of lower limb, unilateral	0.0623
North Macedonia	2000	Amputation of lower limb, unilateral	0.0559
North Macedonia	2005	Amputation of lower limb, unilateral	0.0498
North Macedonia	2010	Amputation of lower limb, unilateral	0.0425
North Macedonia	2017	Amputation of lower limb, unilateral	0.039
North Macedonia	1990	Open wound(s)	0.011
North Macedonia	1995	Open wound(s)	0.011
North Macedonia	2000	Open wound(s)	0.011
North Macedonia	2005	Open wound(s)	0.011
North Macedonia	2010	Open wound(s)	0.011
North Macedonia	2017	Open wound(s)	0.011
North Macedonia	1990	Poisoning requiring urgent care	0.1628
North Macedonia	1995	Poisoning requiring urgent care	0.1628
North Macedonia	2000	Poisoning requiring urgent care	0.1628
North Macedonia	2005	Poisoning requiring urgent care	0.1628
North Macedonia	2010	Poisoning requiring urgent care	0.1628
North Macedonia	2017	Poisoning requiring urgent care	0.1628
North Macedonia	1990	Severe chest injury	0.0474

North Macedonia	1995	Severe chest injury	0.0474
North Macedonia	2000	Severe chest injury	0.0474
North Macedonia	2005	Severe chest injury	0.0474
North Macedonia	2010	Severe chest injury	0.0474
North Macedonia	2017	Severe chest injury	0.0474
North Macedonia	1990	Internal hemorrhage in abdomen and pelvis	0.1143
North Macedonia	1995	Internal hemorrhage in abdomen and pelvis	0.1143
North Macedonia	2000	Internal hemorrhage in abdomen and pelvis	0.1143
North Macedonia	2005	Internal hemorrhage in abdomen and pelvis	0.1143
North Macedonia	2010	Internal hemorrhage in abdomen and pelvis	0.1143
North Macedonia	2017	Internal hemorrhage in abdomen and pelvis	0.1143
North Macedonia	1990	Contusion in any part of the body	0.0075
North Macedonia	1995	Contusion in any part of the body	0.0075
North Macedonia	2000	Contusion in any part of the body	0.0075
North Macedonia	2005	Contusion in any part of the body	0.0075
North Macedonia	2010	Contusion in any part of the body	0.0075
North Macedonia	2017	Contusion in any part of the body	0.0075
North Macedonia	1990	Effect of different environmental factors	0.049
North Macedonia	1995	Effect of different environmental factors	0.049
North Macedonia	2000	Effect of different environmental factors	0.049
North Macedonia	2005	Effect of different environmental factors	0.049
North Macedonia	2010	Effect of different environmental factors	0.049
North Macedonia	2017	Effect of different environmental factors	0.049
North Macedonia	1990	Complications following therapeutic procedures	0.0506
North Macedonia	1995	Complications following therapeutic procedures	0.0506
North Macedonia	2000	Complications following therapeutic procedures	0.0506
North Macedonia	2005	Complications following therapeutic procedures	0.0506
North Macedonia	2010	Complications following therapeutic procedures	0.0506
North Macedonia	2017	Complications following therapeutic procedures	0.0506
North Macedonia	1990	Multiple fractures, dislocations, crashes, wounds, sprains, and strains	0.1275
North Macedonia	1995	Multiple fractures, dislocations, crashes, wounds, sprains, and strains	0.1176
North Macedonia	2000	Multiple fractures, dislocations, crashes, wounds, sprains, and strains	0.1012
North Macedonia	2005	Multiple fractures, dislocations, crashes, wounds, sprains, and strains	0.0856
North Macedonia	2010	Multiple fractures, dislocations, crashes, wounds, sprains, and strains	0.0667
North Macedonia	2017	Multiple fractures, dislocations, crashes, wounds, sprains, and strains	0.0579
North Macedonia	1990	Amputation of upper limb, unilateral	0.055
North Macedonia	1995	Amputation of upper limb, unilateral	0.0527
North Macedonia	2000	Amputation of upper limb, unilateral	0.049
North Macedonia	2005	Amputation of upper limb, unilateral	0.0454
North Macedonia	2010	Amputation of upper limb, unilateral	0.0411
North Macedonia	2017	Amputation of upper limb, unilateral	0.039
North Macedonia	1990	Amputation of thumb	0.0114
North Macedonia	1995	Amputation of thumb	0.0114
North Macedonia	2000	Amputation of thumb	0.0114
North Macedonia	2005	Amputation of thumb	0.0114
North Macedonia	2010	Amputation of thumb	0.0114
North Macedonia	2017	Amputation of thumb	0.0114
North Macedonia	1990	Amputation of toe/toes	0.0059
North Macedonia	1995	Amputation of toe/toes	0.0059
North Macedonia	2000	Amputation of toe/toes	0.0059
North Macedonia	2005	Amputation of toe/toes	0.0059
North Macedonia	2010	Amputation of toe/toes	0.0059
North Macedonia	2017	Amputation of toe/toes	0.0059
North Macedonia	1990	Burns, <20% total burned surface area without lower airway burns	0.0163
North Macedonia	1995	Burns, <20% total burned surface area without lower airway burns	0.0163
North Macedonia	2000	Burns, <20% total burned surface area without lower airway burns	0.0163
North Macedonia	2005	Burns, <20% total burned surface area without lower airway burns	0.0163
North Macedonia	2010	Burns, <20% total burned surface area without lower airway burns	0.0163
North Macedonia	2017	Burns, <20% total burned surface area without lower airway burns	0.0163
North Macedonia	1990	Burns, >=20% total burned surface area or >= 10% burned surface area if head/neck or hands/wrist involved w/o lower airway burns	0.1996
North Macedonia	1995	Burns, >=20% total burned surface area or >= 10% burned surface area if head/neck or hands/wrist involved w/o lower airway burns	0.1904
North Macedonia	2000	Burns, >=20% total burned surface area or >= 10% burned surface area if head/neck or hands/wrist involved w/o lower airway burns	0.1751
North Macedonia	2005	Burns, >=20% total burned surface area or >= 10% burned surface area if head/neck or hands/wrist involved w/o lower airway burns	0.1605
North Macedonia	2010	Burns, >=20% total burned surface area or >= 10% burned surface area if head/neck or hands/wrist involved w/o lower airway burns	0.143
North Macedonia	2017	Burns, >=20% total burned surface area or >= 10% burned surface area if head/neck or hands/wrist involved w/o lower airway burns	0.1348
Montenegro	1990	Amputation of lower limbs, bilateral	0.1428
Montenegro	1995	Amputation of lower limbs, bilateral	0.1414
Montenegro	2000	Amputation of lower limbs, bilateral	0.1389
Montenegro	2005	Amputation of lower limbs, bilateral	0.1259
Montenegro	2010	Amputation of lower limbs, bilateral	0.1232
Montenegro	2017	Amputation of lower limbs, bilateral	0.1232
Montenegro	1990	Lower airway burns	0.2252
Montenegro	1995	Lower airway burns	0.2252
Montenegro	2000	Lower airway burns	0.2252
Montenegro	2005	Lower airway burns	0.2252
Montenegro	2010	Lower airway burns	0.2252
Montenegro	2017	Lower airway burns	0.2252
Montenegro	1990	Dislocation of hip	0.0159
Montenegro	1995	Dislocation of hip	0.0159
Montenegro	2000	Dislocation of hip	0.0159
Montenegro	2005	Dislocation of hip	0.0159
Montenegro	2010	Dislocation of hip	0.0159
Montenegro	2017	Dislocation of hip	0.0159
Montenegro	1990	Dislocation of knee	0.1134
Montenegro	1995	Dislocation of knee	0.1134
Montenegro	2000	Dislocation of knee	0.1134
Montenegro	2005	Dislocation of knee	0.1134
Montenegro	2010	Dislocation of knee	0.1134
Montenegro	2017	Dislocation of knee	0.1134
Montenegro	1990	Dislocation of shoulder	0.062
Montenegro	1995	Dislocation of shoulder	0.062
Montenegro	2000	Dislocation of shoulder	0.062
Montenegro	2005	Dislocation of shoulder	0.062
Montenegro	2010	Dislocation of shoulder	0.062
Montenegro	2017	Dislocation of shoulder	0.062
Montenegro	1990	Muscle and tendon injuries, including sprains and strains lesser dislocations	0.0075
Montenegro	1995	Muscle and tendon injuries, including sprains and strains lesser dislocations	0.0075
Montenegro	2000	Muscle and tendon injuries, including sprains and strains lesser dislocations	0.0075
Montenegro	2005	Muscle and tendon injuries, including sprains and strains lesser dislocations	0.0075
Montenegro	2010	Muscle and tendon injuries, including sprains and strains lesser dislocations	0.0075
Montenegro	2017	Muscle and tendon injuries, including sprains and strains lesser dislocations	0.0075
Montenegro	1990	Fracture of clavicle, scapula, or humerus	0.0349
Montenegro	1995	Fracture of clavicle, scapula, or humerus	0.0349
Montenegro	2000	Fracture of clavicle, scapula, or humerus	0.0349
Montenegro	2005	Fracture of clavicle, scapula, or humerus	0.0349
Montenegro	2010	Fracture of clavicle, scapula, or humerus	0.0349
Montenegro	2017	Fracture of clavicle, scapula, or humerus	0.0349
Montenegro	1990	Fracture of face bones	0.0669
Montenegro	1995	Fracture of face bones	0.0669
Montenegro	2000	Fracture of face bones	0.0669
Montenegro	2005	Fracture of face bones	0.0669
Montenegro	2010	Fracture of face bones	0.0669
Montenegro	2017	Fracture of face bones	0.0669
Montenegro	1990	Fracture of foot bones except ankle	0.0264
Montenegro	1995	Fracture of foot bones except ankle	0.0264
Montenegro	2000	Fracture of foot bones except ankle	0.0264
Montenegro	2005	Fracture of foot bones except ankle	0.0264
Montenegro	2010	Fracture of foot bones except ankle	0.0264
Montenegro	2017	Fracture of foot bones except ankle	0.0264
Montenegro	1990	Fracture of hand (wrist and other distal part of hand)	0.0138
Montenegro	1995	Fracture of hand (wrist and other distal part of hand)	0.0138
Montenegro	2000	Fracture of hand (wrist and other distal part of hand)	0.0138
Montenegro	2005	Fracture of hand (wrist and other distal part of hand)	0.0138
Montenegro	2010	Fracture of hand (wrist and other distal part of hand)	0.0138
Montenegro	2017	Fracture of hand (wrist and other distal part of hand)	0.0138
Montenegro	1990	Fracture of hip	0.0836
Montenegro	1995	Fracture of hip	0.0817
Montenegro	2000	Fracture of hip	0.0783
Montenegro	2005	Fracture of hip	0.0612
Montenegro	2010	Fracture of hip	0.0577
Montenegro	2017	Fracture of hip	0.0577
Montenegro	1990	Amputation of upper limbs, bilateral	0.1144
Montenegro	1995	Amputation of upper limbs, bilateral	0.1125
Montenegro	2000	Amputation of upper limbs, bilateral	0.109
Montenegro	2005	Amputation of upper limbs, bilateral	0.0912
Montenegro	2010	Amputation of upper limbs, bilateral	0.0876
Montenegro	2017	Amputation of upper limbs, bilateral	0.0876
Montenegro	1990	Fracture of patella, tibia or fibula, or ankle	0.0554
Montenegro	1995	Fracture of patella, tibia or fibula, or ankle	0.0554
Montenegro	2000	Fracture of patella, tibia or fibula, or ankle	0.0554
Montenegro	2005	Fracture of patella, tibia or fibula, or ankle	0.0554
Montenegro	2010	Fracture of patella, tibia or fibula, or ankle	0.0554
Montenegro	2017	Fracture of patella, tibia or fibula, or ankle	0.0554
Montenegro	1990	Fracture of pelvis	0.1823
Montenegro	1995	Fracture of pelvis	0.1823
Montenegro	2000	Fracture of pelvis	0.1823
Montenegro	2005	Fracture of pelvis	0.1823
Montenegro	2010	Fracture of pelvis	0.1823
Montenegro	2017	Fracture of pelvis	0.1823
Montenegro	1990	Fracture of radius and/or ulna	0.0435
Montenegro	1995	Fracture of radius and/or ulna	0.0435
Montenegro	2000	Fracture of radius and/or ulna	0.0435
Montenegro	2005	Fracture of radius and/or ulna	0.0435
Montenegro	2010	Fracture of radius and/or ulna	0.0435
Montenegro	2017	Fracture of radius and/or ulna	0.0435
Montenegro	1990	Fracture of skull	0.0714
Montenegro	1995	Fracture of skull	0.0714
Montenegro	2000	Fracture of skull	0.0714
Montenegro	2005	Fracture of skull	0.0714
Montenegro	2010	Fracture of skull	0.0714
Montenegro	2017	Fracture of skull	0.0714
Montenegro	1990	Fracture of sternum and/or fracture of one or more ribs	0.1027
Montenegro	1995	Fracture of sternum and/or fracture of one or more ribs	0.1027
Montenegro	2000	Fracture of sternum and/or fracture of one or more ribs	0.1027
Montenegro	2005	Fracture of sternum and/or fracture of one or more ribs	0.1027
Montenegro	2010	Fracture of sternum and/or fracture of one or more ribs	0.1027
Montenegro	2017	Fracture of sternum and/or fracture of one or more ribs	0.1027
Montenegro	1990	Fracture of vertebral column	0.1106
Montenegro	1995	Fracture of vertebral column	0.1106
Montenegro	2000	Fracture of vertebral column	0.1106
Montenegro	2005	Fracture of vertebral column	0.1106
Montenegro	2010	Fracture of vertebral column	0.1106
Montenegro	2017	Fracture of vertebral column	0.1106
Montenegro	1990	Fracture of femur, other than femoral neck	0.042
Montenegro	1995	Fracture of femur, other than femoral neck	0.042
Montenegro	2000	Fracture of femur, other than femoral neck	0.042
Montenegro	2005	Fracture of femur, other than femoral neck	0.042
Montenegro	2010	Fracture of femur, other than femoral neck	0.042
Montenegro	2017	Fracture of femur, other than femoral neck	0.042
Montenegro	1990	Minor TBI	0.1319
Montenegro	1995	Minor TBI	0.1319
Montenegro	2000	Minor TBI	0.1319
Montenegro	2005	Minor TBI	0.1319
Montenegro	2010	Minor TBI	0.1319
Montenegro	2017	Minor TBI	0.1319
Montenegro	1990	Moderate TBI	0.1637
Montenegro	1995	Moderate TBI	0.1637
Montenegro	2000	Moderate TBI	0.1637
Montenegro	2005	Moderate TBI	0.1637
Montenegro	2010	Moderate TBI	0.1637
Montenegro	2017	Moderate TBI	0.1637
Montenegro	1990	Amputation of fingers (excluding thumb)	0.0047
Montenegro	1995	Amputation of fingers (excluding thumb)	0.0047
Montenegro	2000	Amputation of fingers (excluding thumb)	0.0047
Montenegro	2005	Amputation of fingers (excluding thumb)	0.0047
Montenegro	2010	Amputation of fingers (excluding thumb)	0.0047
Montenegro	2017	Amputation of fingers (excluding thumb)	0.0047
Montenegro	1990	Spinal cord lesion at neck level	0.4208
Montenegro	1995	Spinal cord lesion at neck level	0.4201
Montenegro	2000	Spinal cord lesion at neck level	0.419
Montenegro	2005	Spinal cord lesion at neck level	0.413
Montenegro	2010	Spinal cord lesion at neck level	0.4118
Montenegro	2017	Spinal cord lesion at neck level	0.4118
Montenegro	1990	Spinal cord lesion below neck level	0.1966
Montenegro	1995	Spinal cord lesion below neck level	0.1952
Montenegro	2000	Spinal cord lesion below neck level	0.1927
Montenegro	2005	Spinal cord lesion below neck level	0.1802
Montenegro	2010	Spinal cord lesion below neck level	0.1777
Montenegro	2017	Spinal cord lesion below neck level	0.1777
Montenegro	1990	Drowning and nonfatal submersion	0.2471
Montenegro	1995	Drowning and nonfatal submersion	0.2471
Montenegro	2000	Drowning and nonfatal submersion	0.2471
Montenegro	2005	Drowning and nonfatal submersion	0.2471
Montenegro	2010	Drowning and nonfatal submersion	0.2471
Montenegro	2017	Drowning and nonfatal submersion	0.2471
Montenegro	1990	Asphyxiation	0.2471
Montenegro	1995	Asphyxiation	0.2471
Montenegro	2000	Asphyxiation	0.2471
Montenegro	2005	Asphyxiation	0.2471
Montenegro	2010	Asphyxiation	0.2471
Montenegro	2017	Asphyxiation	0.2471
Montenegro	1990	Crush injury	0.1325
Montenegro	1995	Crush injury	0.1325
Montenegro	2000	Crush injury	0.1325
Montenegro	2005	Crush injury	0.1325
Montenegro	2010	Crush injury	0.1325
Montenegro	2017	Crush injury	0.1325
Montenegro	1990	Nerve injury	0.1129
Montenegro	1995	Nerve injury	0.1129
Montenegro	2000	Nerve injury	0.1129
Montenegro	2005	Nerve injury	0.1129
Montenegro	2010	Nerve injury	0.1129
Montenegro	2017	Nerve injury	0.1129
Montenegro	1990	Injury to eyes	0.0165
Montenegro	1995	Injury to eyes	0.0165
Montenegro	2000	Injury to eyes	0.0165
Montenegro	2005	Injury to eyes	0.0165
Montenegro	2010	Injury to eyes	0.0165
Montenegro	2017	Injury to eyes	0.0165
Montenegro	1990	Amputation of lower limb, unilateral	0.049
Montenegro	1995	Amputation of lower limb, unilateral	0.0483
Montenegro	2000	Amputation of lower limb, unilateral	0.047
Montenegro	2005	Amputation of lower limb, unilateral	0.0403
Montenegro	2010	Amputation of lower limb, unilateral	0.039
Montenegro	2017	Amputation of lower limb, unilateral	0.039
Montenegro	1990	Open wound(s)	0.011
Montenegro	1995	Open wound(s)	0.011
Montenegro	2000	Open wound(s)	0.011
Montenegro	2005	Open wound(s)	0.011
Montenegro	2010	Open wound(s)	0.011
Montenegro	2017	Open wound(s)	0.011
Montenegro	1990	Poisoning requiring urgent care	0.1628
Montenegro	1995	Poisoning requiring urgent care	0.1628
Montenegro	2000	Poisoning requiring urgent care	0.1628
Montenegro	2005	Poisoning requiring urgent care	0.1628
Montenegro	2010	Poisoning requiring urgent care	0.1628
Montenegro	2017	Poisoning requiring urgent care	0.1628
Montenegro	1990	Severe chest injury	0.0474
Montenegro	1995	Severe chest injury	0.0474
Montenegro	2000	Severe chest injury	0.0474
Montenegro	2005	Severe chest injury	0.0474
Montenegro	2010	Severe chest injury	0.0474
Montenegro	2017	Severe chest injury	0.0474
Montenegro	1990	Internal hemorrhage in abdomen and pelvis	0.1143
Montenegro	1995	Internal hemorrhage in abdomen and pelvis	0.1143
Montenegro	2000	Internal hemorrhage in abdomen and pelvis	0.1143
Montenegro	2005	Internal hemorrhage in abdomen and pelvis	0.1143
Montenegro	2010	Internal hemorrhage in abdomen and pelvis	0.1143
Montenegro	2017	Internal hemorrhage in abdomen and pelvis	0.1143
Montenegro	1990	Contusion in any part of the body	0.0075
Montenegro	1995	Contusion in any part of the body	0.0075
Montenegro	2000	Contusion in any part of the body	0.0075
Montenegro	2005	Contusion in any part of the body	0.0075
Montenegro	2010	Contusion in any part of the body	0.0075
Montenegro	2017	Contusion in any part of the body	0.0075
Montenegro	1990	Effect of different environmental factors	0.049
Montenegro	1995	Effect of different environmental factors	0.049
Montenegro	2000	Effect of different environmental factors	0.049
Montenegro	2005	Effect of different environmental factors	0.049
Montenegro	2010	Effect of different environmental factors	0.049
Montenegro	2017	Effect of different environmental factors	0.049
Montenegro	1990	Complications following therapeutic procedures	0.0506
Montenegro	1995	Complications following therapeutic procedures	0.0506
Montenegro	2000	Complications following therapeutic procedures	0.0506
Montenegro	2005	Complications following therapeutic procedures	0.0506
Montenegro	2010	Complications following therapeutic procedures	0.0506
Montenegro	2017	Complications following therapeutic procedures	0.0506
Montenegro	1990	Multiple fractures, dislocations, crashes, wounds, sprains, and strains	0.0836
Montenegro	1995	Multiple fractures, dislocations, crashes, wounds, sprains, and strains	0.0817
Montenegro	2000	Multiple fractures, dislocations, crashes, wounds, sprains, and strains	0.0783
Montenegro	2005	Multiple fractures, dislocations, crashes, wounds, sprains, and strains	0.0612
Montenegro	2010	Multiple fractures, dislocations, crashes, wounds, sprains, and strains	0.0577
Montenegro	2017	Multiple fractures, dislocations, crashes, wounds, sprains, and strains	0.0577
Montenegro	1990	Amputation of upper limb, unilateral	0.0449
Montenegro	1995	Amputation of upper limb, unilateral	0.0445
Montenegro	2000	Amputation of upper limb, unilateral	0.0437
Montenegro	2005	Amputation of upper limb, unilateral	0.0398
Montenegro	2010	Amputation of upper limb, unilateral	0.039
Montenegro	2017	Amputation of upper limb, unilateral	0.039
Montenegro	1990	Amputation of thumb	0.0114
Montenegro	1995	Amputation of thumb	0.0114
Montenegro	2000	Amputation of thumb	0.0114
Montenegro	2005	Amputation of thumb	0.0114
Montenegro	2010	Amputation of thumb	0.0114
Montenegro	2017	Amputation of thumb	0.0114
Montenegro	1990	Amputation of toe/toes	0.0059
Montenegro	1995	Amputation of toe/toes	0.0059
Montenegro	2000	Amputation of toe/toes	0.0059
Montenegro	2005	Amputation of toe/toes	0.0059
Montenegro	2010	Amputation of toe/toes	0.0059
Montenegro	2017	Amputation of toe/toes	0.0059
Montenegro	1990	Burns, <20% total burned surface area without lower airway burns	0.0163
Montenegro	1995	Burns, <20% total burned surface area without lower airway burns	0.0163
Montenegro	2000	Burns, <20% total burned surface area without lower airway burns	0.0163
Montenegro	2005	Burns, <20% total burned surface area without lower airway burns	0.0163
Montenegro	2010	Burns, <20% total burned surface area without lower airway burns	0.0163
Montenegro	2017	Burns, <20% total burned surface area without lower airway burns	0.0163
Montenegro	1990	Burns, >=20% total burned surface area or >= 10% burned surface area if head/neck or hands/wrist involved w/o lower airway burns	0.1587
Montenegro	1995	Burns, >=20% total burned surface area or >= 10% burned surface area if head/neck or hands/wrist involved w/o lower airway burns	0.157
Montenegro	2000	Burns, >=20% total burned surface area or >= 10% burned surface area if head/neck or hands/wrist involved w/o lower airway burns	0.1538
Montenegro	2005	Burns, >=20% total burned surface area or >= 10% burned surface area if head/neck or hands/wrist involved w/o lower airway burns	0.1378
Montenegro	2010	Burns, >=20% total burned surface area or >= 10% burned surface area if head/neck or hands/wrist involved w/o lower airway burns	0.1346
Montenegro	2017	Burns, >=20% total burned surface area or >= 10% burned surface area if head/neck or hands/wrist involved w/o lower airway burns	0.1346
Poland	1990	Amputation of lower limbs, bilateral	0.1706
Poland	1995	Amputation of lower limbs, bilateral	0.1574
Poland	2000	Amputation of lower limbs, bilateral	0.1379
Poland	2005	Amputation of lower limbs, bilateral	0.1247
Poland	2010	Amputation of lower limbs, bilateral	0.1232
Poland	2017	Amputation of lower limbs, bilateral	0.1232
Poland	1990	Lower airway burns	0.2252
Poland	1995	Lower airway burns	0.2252
Poland	2000	Lower airway burns	0.2252
Poland	2005	Lower airway burns	0.2252
Poland	2010	Lower airway burns	0.2252
Poland	2017	Lower airway burns	0.2252
Poland	1990	Dislocation of hip	0.0159
Poland	1995	Dislocation of hip	0.0159
Poland	2000	Dislocation of hip	0.0159
Poland	2005	Dislocation of hip	0.0159
Poland	2010	Dislocation of hip	0.0159
Poland	2017	Dislocation of hip	0.0159
Poland	1990	Dislocation of knee	0.1134
Poland	1995	Dislocation of knee	0.1134
Poland	2000	Dislocation of knee	0.1134
Poland	2005	Dislocation of knee	0.1134
Poland	2010	Dislocation of knee	0.1134
Poland	2017	Dislocation of knee	0.1134
Poland	1990	Dislocation of shoulder	0.062
Poland	1995	Dislocation of shoulder	0.062
Poland	2000	Dislocation of shoulder	0.062
Poland	2005	Dislocation of shoulder	0.062
Poland	2010	Dislocation of shoulder	0.062
Poland	2017	Dislocation of shoulder	0.062
Poland	1990	Muscle and tendon injuries, including sprains and strains lesser dislocations	0.0075
Poland	1995	Muscle and tendon injuries, including sprains and strains lesser dislocations	0.0075
Poland	2000	Muscle and tendon injuries, including sprains and strains lesser dislocations	0.0075
Poland	2005	Muscle and tendon injuries, including sprains and strains lesser dislocations	0.0075
Poland	2010	Muscle and tendon injuries, including sprains and strains lesser dislocations	0.0075
Poland	2017	Muscle and tendon injuries, including sprains and strains lesser dislocations	0.0075
Poland	1990	Fracture of clavicle, scapula, or humerus	0.0349
Poland	1995	Fracture of clavicle, scapula, or humerus	0.0349
Poland	2000	Fracture of clavicle, scapula, or humerus	0.0349
Poland	2005	Fracture of clavicle, scapula, or humerus	0.0349
Poland	2010	Fracture of clavicle, scapula, or humerus	0.0349
Poland	2017	Fracture of clavicle, scapula, or humerus	0.0349
Poland	1990	Fracture of face bones	0.0669
Poland	1995	Fracture of face bones	0.0669
Poland	2000	Fracture of face bones	0.0669
Poland	2005	Fracture of face bones	0.0669
Poland	2010	Fracture of face bones	0.0669
Poland	2017	Fracture of face bones	0.0669
Poland	1990	Fracture of foot bones except ankle	0.0264
Poland	1995	Fracture of foot bones except ankle	0.0264
Poland	2000	Fracture of foot bones except ankle	0.0264
Poland	2005	Fracture of foot bones except ankle	0.0264
Poland	2010	Fracture of foot bones except ankle	0.0264
Poland	2017	Fracture of foot bones except ankle	0.0264
Poland	1990	Fracture of hand (wrist and other distal part of hand)	0.0138
Poland	1995	Fracture of hand (wrist and other distal part of hand)	0.0138
Poland	2000	Fracture of hand (wrist and other distal part of hand)	0.0138
Poland	2005	Fracture of hand (wrist and other distal part of hand)	0.0138
Poland	2010	Fracture of hand (wrist and other distal part of hand)	0.0138
Poland	2017	Fracture of hand (wrist and other distal part of hand)	0.0138
Poland	1990	Fracture of hip	0.1203
Poland	1995	Fracture of hip	0.1029
Poland	2000	Fracture of hip	0.077
Poland	2005	Fracture of hip	0.0596
Poland	2010	Fracture of hip	0.0577
Poland	2017	Fracture of hip	0.0577
Poland	1990	Amputation of upper limbs, bilateral	0.1523
Poland	1995	Amputation of upper limbs, bilateral	0.1343
Poland	2000	Amputation of upper limbs, bilateral	0.1076
Poland	2005	Amputation of upper limbs, bilateral	0.0896
Poland	2010	Amputation of upper limbs, bilateral	0.0876
Poland	2017	Amputation of upper limbs, bilateral	0.0876
Poland	1990	Fracture of patella, tibia or fibula, or ankle	0.0554
Poland	1995	Fracture of patella, tibia or fibula, or ankle	0.0554
Poland	2000	Fracture of patella, tibia or fibula, or ankle	0.0554
Poland	2005	Fracture of patella, tibia or fibula, or ankle	0.0554
Poland	2010	Fracture of patella, tibia or fibula, or ankle	0.0554
Poland	2017	Fracture of patella, tibia or fibula, or ankle	0.0554
Poland	1990	Fracture of pelvis	0.1823
Poland	1995	Fracture of pelvis	0.1823
Poland	2000	Fracture of pelvis	0.1823
Poland	2005	Fracture of pelvis	0.1823
Poland	2010	Fracture of pelvis	0.1823
Poland	2017	Fracture of pelvis	0.1823
Poland	1990	Fracture of radius and/or ulna	0.0435
Poland	1995	Fracture of radius and/or ulna	0.0435
Poland	2000	Fracture of radius and/or ulna	0.0435
Poland	2005	Fracture of radius and/or ulna	0.0435
Poland	2010	Fracture of radius and/or ulna	0.0435
Poland	2017	Fracture of radius and/or ulna	0.0435
Poland	1990	Fracture of skull	0.0714
Poland	1995	Fracture of skull	0.0714
Poland	2000	Fracture of skull	0.0714
Poland	2005	Fracture of skull	0.0714
Poland	2010	Fracture of skull	0.0714
Poland	2017	Fracture of skull	0.0714
Poland	1990	Fracture of sternum and/or fracture of one or more ribs	0.1027
Poland	1995	Fracture of sternum and/or fracture of one or more ribs	0.1027
Poland	2000	Fracture of sternum and/or fracture of one or more ribs	0.1027
Poland	2005	Fracture of sternum and/or fracture of one or more ribs	0.1027
Poland	2010	Fracture of sternum and/or fracture of one or more ribs	0.1027
Poland	2017	Fracture of sternum and/or fracture of one or more ribs	0.1027
Poland	1990	Fracture of vertebral column	0.1106
Poland	1995	Fracture of vertebral column	0.1106
Poland	2000	Fracture of vertebral column	0.1106
Poland	2005	Fracture of vertebral column	0.1106
Poland	2010	Fracture of vertebral column	0.1106
Poland	2017	Fracture of vertebral column	0.1106

Poland	1990	Fracture of femur, other than femoral neck	0.042
Poland	1995	Fracture of femur, other than femoral neck	0.042
Poland	2000	Fracture of femur, other than femoral neck	0.042
Poland	2005	Fracture of femur, other than femoral neck	0.042
Poland	2010	Fracture of femur, other than femoral neck	0.042
Poland	2017	Fracture of femur, other than femoral neck	0.042
Poland	1990	Minor TBI	0.1319
Poland	1995	Minor TBI	0.1319
Poland	2000	Minor TBI	0.1319
Poland	2005	Minor TBI	0.1319
Poland	2010	Minor TBI	0.1319
Poland	2017	Minor TBI	0.1319
Poland	1990	Moderate TBI	0.1637
Poland	1995	Moderate TBI	0.1637
Poland	2000	Moderate TBI	0.1637
Poland	2005	Moderate TBI	0.1637
Poland	2010	Moderate TBI	0.1637
Poland	2017	Moderate TBI	0.1637
Poland	1990	Amputation of fingers (excluding thumb)	0.0047
Poland	1995	Amputation of fingers (excluding thumb)	0.0047
Poland	2000	Amputation of fingers (excluding thumb)	0.0047
Poland	2005	Amputation of fingers (excluding thumb)	0.0047
Poland	2010	Amputation of fingers (excluding thumb)	0.0047
Poland	2017	Amputation of fingers (excluding thumb)	0.0047
Poland	1990	Spinal cord lesion at neck level	0.4335
Poland	1995	Spinal cord lesion at neck level	0.4274
Poland	2000	Spinal cord lesion at neck level	0.4185
Poland	2005	Spinal cord lesion at neck level	0.4125
Poland	2010	Spinal cord lesion at neck level	0.4118
Poland	2017	Spinal cord lesion at neck level	0.4118
Poland	1990	Spinal cord lesion below neck level	0.2234
Poland	1995	Spinal cord lesion below neck level	0.2106
Poland	2000	Spinal cord lesion below neck level	0.1918
Poland	2005	Spinal cord lesion below neck level	0.1791
Poland	2010	Spinal cord lesion below neck level	0.1777
Poland	2017	Spinal cord lesion below neck level	0.1777
Poland	1990	Drowning and nonfatal submersion	0.2471
Poland	1995	Drowning and nonfatal submersion	0.2471
Poland	2000	Drowning and nonfatal submersion	0.2471
Poland	2005	Drowning and nonfatal submersion	0.2471
Poland	2010	Drowning and nonfatal submersion	0.2471
Poland	2017	Drowning and nonfatal submersion	0.2471
Poland	1990	Asphyxiation	0.2471
Poland	1995	Asphyxiation	0.2471
Poland	2000	Asphyxiation	0.2471
Poland	2005	Asphyxiation	0.2471
Poland	2010	Asphyxiation	0.2471
Poland	2017	Asphyxiation	0.2471
Poland	1990	Crush injury	0.1325
Poland	1995	Crush injury	0.1325
Poland	2000	Crush injury	0.1325
Poland	2005	Crush injury	0.1325
Poland	2010	Crush injury	0.1325
Poland	2017	Crush injury	0.1325
Poland	1990	Nerve injury	0.1129
Poland	1995	Nerve injury	0.1129
Poland	2000	Nerve injury	0.1129
Poland	2005	Nerve injury	0.1129
Poland	2010	Nerve injury	0.1129
Poland	2017	Nerve injury	0.1129
Poland	1990	Injury to eyes	0.0165
Poland	1995	Injury to eyes	0.0165
Poland	2000	Injury to eyes	0.0165
Poland	2005	Injury to eyes	0.0165
Poland	2010	Injury to eyes	0.0165
Poland	2017	Injury to eyes	0.0165
Poland	1990	Amputation of lower limb, unilateral	0.0634
Poland	1995	Amputation of lower limb, unilateral	0.0566
Poland	2000	Amputation of lower limb, unilateral	0.0465
Poland	2005	Amputation of lower limb, unilateral	0.0397
Poland	2010	Amputation of lower limb, unilateral	0.039
Poland	2017	Amputation of lower limb, unilateral	0.039
Poland	1990	Open wound(s)	0.011
Poland	1995	Open wound(s)	0.011
Poland	2000	Open wound(s)	0.011
Poland	2005	Open wound(s)	0.011
Poland	2010	Open wound(s)	0.011
Poland	2017	Open wound(s)	0.011
Poland	1990	Poisoning requiring urgent care	0.1628
Poland	1995	Poisoning requiring urgent care	0.1628
Poland	2000	Poisoning requiring urgent care	0.1628
Poland	2005	Poisoning requiring urgent care	0.1628
Poland	2010	Poisoning requiring urgent care	0.1628
Poland	2017	Poisoning requiring urgent care	0.1628
Poland	1990	Severe chest injury	0.0474
Poland	1995	Severe chest injury	0.0474
Poland	2000	Severe chest injury	0.0474
Poland	2005	Severe chest injury	0.0474
Poland	2010	Severe chest injury	0.0474
Poland	2017	Severe chest injury	0.0474
Poland	1990	Internal hemorrhage in abdomen and pelvis	0.1143
Poland	1995	Internal hemorrhage in abdomen and pelvis	0.1143
Poland	2000	Internal hemorrhage in abdomen and pelvis	0.1143
Poland	2005	Internal hemorrhage in abdomen and pelvis	0.1143
Poland	2010	Internal hemorrhage in abdomen and pelvis	0.1143
Poland	2017	Internal hemorrhage in abdomen and pelvis	0.1143
Poland	1990	Contusion in any part of the body	0.0075
Poland	1995	Contusion in any part of the body	0.0075
Poland	2000	Contusion in any part of the body	0.0075
Poland	2005	Contusion in any part of the body	0.0075
Poland	2010	Contusion in any part of the body	0.0075
Poland	2017	Contusion in any part of the body	0.0075
Poland	1990	Effect of different environmental factors	0.049
Poland	1995	Effect of different environmental factors	0.049
Poland	2000	Effect of different environmental factors	0.049
Poland	2005	Effect of different environmental factors	0.049
Poland	2010	Effect of different environmental factors	0.049
Poland	2017	Effect of different environmental factors	0.049
Poland	1990	Complications following therapeutic procedures	0.0506
Poland	1995	Complications following therapeutic procedures	0.0506
Poland	2000	Complications following therapeutic procedures	0.0506
Poland	2005	Complications following therapeutic procedures	0.0506
Poland	2010	Complications following therapeutic procedures	0.0506
Poland	2017	Complications following therapeutic procedures	0.0506
Poland	1990	Multiple fractures, dislocations, crashes, wounds, sprains, and strains	0.1203
Poland	1995	Multiple fractures, dislocations, crashes, wounds, sprains, and strains	0.1029
Poland	2000	Multiple fractures, dislocations, crashes, wounds, sprains, and strains	0.077
Poland	2005	Multiple fractures, dislocations, crashes, wounds, sprains, and strains	0.0596
Poland	2010	Multiple fractures, dislocations, crashes, wounds, sprains, and strains	0.0577
Poland	2017	Multiple fractures, dislocations, crashes, wounds, sprains, and strains	0.0577
Poland	1990	Amputation of upper limb, unilateral	0.0534
Poland	1995	Amputation of upper limb, unilateral	0.0494
Poland	2000	Amputation of upper limb, unilateral	0.0434
Poland	2005	Amputation of upper limb, unilateral	0.0394
Poland	2010	Amputation of upper limb, unilateral	0.039
Poland	2017	Amputation of upper limb, unilateral	0.039
Poland	1990	Amputation of thumb	0.0114
Poland	1995	Amputation of thumb	0.0114
Poland	2000	Amputation of thumb	0.0114
Poland	2005	Amputation of thumb	0.0114
Poland	2010	Amputation of thumb	0.0114
Poland	2017	Amputation of thumb	0.0114
Poland	1990	Amputation of toe/toes	0.0059
Poland	1995	Amputation of toe/toes	0.0059
Poland	2000	Amputation of toe/toes	0.0059
Poland	2005	Amputation of toe/toes	0.0059
Poland	2010	Amputation of toe/toes	0.0059
Poland	2017	Amputation of toe/toes	0.0059
Poland	1990	Burns, <20% total burned surface area without lower airway burns	0.0163
Poland	1995	Burns, <20% total burned surface area without lower airway burns	0.0163
Poland	2000	Burns, <20% total burned surface area without lower airway burns	0.0163
Poland	2005	Burns, <20% total burned surface area without lower airway burns	0.0163
Poland	2010	Burns, <20% total burned surface area without lower airway burns	0.0163
Poland	2017	Burns, <20% total burned surface area without lower airway burns	0.0163
Poland	1990	Burns, >=20% total burned surface area or >= 10% burned surface area if head/neck or hands/wrist involved w/o lower airway burns	0.1929
Poland	1995	Burns, >=20% total burned surface area or >= 10% burned surface area if head/neck or hands/wrist involved w/o lower airway burns	0.1766
Poland	2000	Burns, >=20% total burned surface area or >= 10% burned surface area if head/neck or hands/wrist involved w/o lower airway burns	0.1526
Poland	2005	Burns, >=20% total burned surface area or >= 10% burned surface area if head/neck or hands/wrist involved w/o lower airway burns	0.1364
Poland	2010	Burns, >=20% total burned surface area or >= 10% burned surface area if head/neck or hands/wrist involved w/o lower airway burns	0.1346
Poland	2017	Burns, >=20% total burned surface area or >= 10% burned surface area if head/neck or hands/wrist involved w/o lower airway burns	0.1346
Romania	1990	Amputation of lower limbs, bilateral	0.1768
Romania	1995	Amputation of lower limbs, bilateral	0.1665
Romania	2000	Amputation of lower limbs, bilateral	0.1512
Romania	2005	Amputation of lower limbs, bilateral	0.1379
Romania	2010	Amputation of lower limbs, bilateral	0.1256
Romania	2017	Amputation of lower limbs, bilateral	0.1232
Romania	1990	Lower airway burns	0.2252
Romania	1995	Lower airway burns	0.2252
Romania	2000	Lower airway burns	0.2252
Romania	2005	Lower airway burns	0.2252
Romania	2010	Lower airway burns	0.2252
Romania	2017	Lower airway burns	0.2252
Romania	1990	Dislocation of hip	0.0159
Romania	1995	Dislocation of hip	0.0159
Romania	2000	Dislocation of hip	0.0159
Romania	2005	Dislocation of hip	0.0159
Romania	2010	Dislocation of hip	0.0159
Romania	2017	Dislocation of hip	0.0159
Romania	1990	Dislocation of knee	0.1134
Romania	1995	Dislocation of knee	0.1134
Romania	2000	Dislocation of knee	0.1134
Romania	2005	Dislocation of knee	0.1134
Romania	2010	Dislocation of knee	0.1134
Romania	2017	Dislocation of knee	0.1134
Romania	1990	Dislocation of shoulder	0.062
Romania	1995	Dislocation of shoulder	0.062
Romania	2000	Dislocation of shoulder	0.062
Romania	2005	Dislocation of shoulder	0.062
Romania	2010	Dislocation of shoulder	0.062
Romania	2017	Dislocation of shoulder	0.062
Romania	1990	Muscle and tendon injuries, including sprains and strains lesser dislocations	0.0075
Romania	1995	Muscle and tendon injuries, including sprains and strains lesser dislocations	0.0075
Romania	2000	Muscle and tendon injuries, including sprains and strains lesser dislocations	0.0075
Romania	2005	Muscle and tendon injuries, including sprains and strains lesser dislocations	0.0075
Romania	2010	Muscle and tendon injuries, including sprains and strains lesser dislocations	0.0075
Romania	2017	Muscle and tendon injuries, including sprains and strains lesser dislocations	0.0075
Romania	1990	Fracture of clavicle, scapula, or humerus	0.0349
Romania	1995	Fracture of clavicle, scapula, or humerus	0.0349
Romania	2000	Fracture of clavicle, scapula, or humerus	0.0349
Romania	2005	Fracture of clavicle, scapula, or humerus	0.0349
Romania	2010	Fracture of clavicle, scapula, or humerus	0.0349
Romania	2017	Fracture of clavicle, scapula, or humerus	0.0349
Romania	1990	Fracture of face bones	0.0669
Romania	1995	Fracture of face bones	0.0669
Romania	2000	Fracture of face bones	0.0669
Romania	2005	Fracture of face bones	0.0669
Romania	2010	Fracture of face bones	0.0669
Romania	2017	Fracture of face bones	0.0669
Romania	1990	Fracture of foot bones except ankle	0.0264
Romania	1995	Fracture of foot bones except ankle	0.0264
Romania	2000	Fracture of foot bones except ankle	0.0264
Romania	2005	Fracture of foot bones except ankle	0.0264
Romania	2010	Fracture of foot bones except ankle	0.0264
Romania	2017	Fracture of foot bones except ankle	0.0264
Romania	1990	Fracture of hand (wrist and other distal part of hand)	0.0138
Romania	1995	Fracture of hand (wrist and other distal part of hand)	0.0138
Romania	2000	Fracture of hand (wrist and other distal part of hand)	0.0138
Romania	2005	Fracture of hand (wrist and other distal part of hand)	0.0138
Romania	2010	Fracture of hand (wrist and other distal part of hand)	0.0138
Romania	2017	Fracture of hand (wrist and other distal part of hand)	0.0138
Romania	1990	Fracture of hip	0.1286
Romania	1995	Fracture of hip	0.1149
Romania	2000	Fracture of hip	0.0947
Romania	2005	Fracture of hip	0.0771
Romania	2010	Fracture of hip	0.0607
Romania	2017	Fracture of hip	0.0577
Romania	1990	Amputation of upper limbs, bilateral	0.1608
Romania	1995	Amputation of upper limbs, bilateral	0.1467
Romania	2000	Amputation of upper limbs, bilateral	0.1259
Romania	2005	Amputation of upper limbs, bilateral	0.1076
Romania	2010	Amputation of upper limbs, bilateral	0.0908
Romania	2017	Amputation of upper limbs, bilateral	0.0876
Romania	1990	Fracture of patella, tibia or fibula, or ankle	0.0554
Romania	1995	Fracture of patella, tibia or fibula, or ankle	0.0554
Romania	2000	Fracture of patella, tibia or fibula, or ankle	0.0554
Romania	2005	Fracture of patella, tibia or fibula, or ankle	0.0554
Romania	2010	Fracture of patella, tibia or fibula, or ankle	0.0554
Romania	2017	Fracture of patella, tibia or fibula, or ankle	0.0554
Romania	1990	Fracture of pelvis	0.1823
Romania	1995	Fracture of pelvis	0.1823
Romania	2000	Fracture of pelvis	0.1823
Romania	2005	Fracture of pelvis	0.1823
Romania	2010	Fracture of pelvis	0.1823
Romania	2017	Fracture of pelvis	0.1823
Romania	1990	Fracture of radius and/or ulna	0.0435
Romania	1995	Fracture of radius and/or ulna	0.0435
Romania	2000	Fracture of radius and/or ulna	0.0435
Romania	2005	Fracture of radius and/or ulna	0.0435
Romania	2010	Fracture of radius and/or ulna	0.0435
Romania	2017	Fracture of radius and/or ulna	0.0435
Romania	1990	Fracture of skull	0.0714
Romania	1995	Fracture of skull	0.0714
Romania	2000	Fracture of skull	0.0714
Romania	2005	Fracture of skull	0.0714
Romania	2010	Fracture of skull	0.0714
Romania	2017	Fracture of skull	0.0714
Romania	1990	Fracture of sternum and/or fracture of one or more ribs	0.1027
Romania	1995	Fracture of sternum and/or fracture of one or more ribs	0.1027
Romania	2000	Fracture of sternum and/or fracture of one or more ribs	0.1027
Romania	2005	Fracture of sternum and/or fracture of one or more ribs	0.1027
Romania	2010	Fracture of sternum and/or fracture of one or more ribs	0.1027
Romania	2017	Fracture of sternum and/or fracture of one or more ribs	0.1027
Romania	1990	Fracture of vertebral column	0.1106
Romania	1995	Fracture of vertebral column	0.1106
Romania	2000	Fracture of vertebral column	0.1106
Romania	2005	Fracture of vertebral column	0.1106
Romania	2010	Fracture of vertebral column	0.1106
Romania	2017	Fracture of vertebral column	0.1106
Romania	1990	Fracture of femur, other than femoral neck	0.042
Romania	1995	Fracture of femur, other than femoral neck	0.042
Romania	2000	Fracture of femur, other than femoral neck	0.042
Romania	2005	Fracture of femur, other than femoral neck	0.042
Romania	2010	Fracture of femur, other than femoral neck	0.042
Romania	2017	Fracture of femur, other than femoral neck	0.042
Romania	1990	Minor TBI	0.1319
Romania	1995	Minor TBI	0.1319
Romania	2000	Minor TBI	0.1319
Romania	2005	Minor TBI	0.1319
Romania	2010	Minor TBI	0.1319
Romania	2017	Minor TBI	0.1319
Romania	1990	Moderate TBI	0.1637
Romania	1995	Moderate TBI	0.1637
Romania	2000	Moderate TBI	0.1637
Romania	2005	Moderate TBI	0.1637
Romania	2010	Moderate TBI	0.1637
Romania	2017	Moderate TBI	0.1637
Romania	1990	Amputation of fingers (excluding thumb)	0.0047
Romania	1995	Amputation of fingers (excluding thumb)	0.0047
Romania	2000	Amputation of fingers (excluding thumb)	0.0047
Romania	2005	Amputation of fingers (excluding thumb)	0.0047
Romania	2010	Amputation of fingers (excluding thumb)	0.0047
Romania	2017	Amputation of fingers (excluding thumb)	0.0047
Romania	1990	Spinal cord lesion at neck level	0.4363
Romania	1995	Spinal cord lesion at neck level	0.4316
Romania	2000	Spinal cord lesion at neck level	0.4246
Romania	2005	Spinal cord lesion at neck level	0.4185
Romania	2010	Spinal cord lesion at neck level	0.4129
Romania	2017	Spinal cord lesion at neck level	0.4118
Romania	1990	Spinal cord lesion below neck level	0.2294
Romania	1995	Spinal cord lesion below neck level	0.2194
Romania	2000	Spinal cord lesion below neck level	0.2047
Romania	2005	Spinal cord lesion below neck level	0.1918
Romania	2010	Spinal cord lesion below neck level	0.1799
Romania	2017	Spinal cord lesion below neck level	0.1777
Romania	1990	Drowning and nonfatal submersion	0.2471
Romania	1995	Drowning and nonfatal submersion	0.2471
Romania	2000	Drowning and nonfatal submersion	0.2471
Romania	2005	Drowning and nonfatal submersion	0.2471
Romania	2010	Drowning and nonfatal submersion	0.2471
Romania	2017	Drowning and nonfatal submersion	0.2471
Romania	1990	Asphyxiation	0.2471
Romania	1995	Asphyxiation	0.2471
Romania	2000	Asphyxiation	0.2471
Romania	2005	Asphyxiation	0.2471
Romania	2010	Asphyxiation	0.2471
Romania	2017	Asphyxiation	0.2471
Romania	1990	Crush injury	0.1325
Romania	1995	Crush injury	0.1325
Romania	2000	Crush injury	0.1325
Romania	2005	Crush injury	0.1325
Romania	2010	Crush injury	0.1325
Romania	2017	Crush injury	0.1325
Romania	1990	Nerve injury	0.1129
Romania	1995	Nerve injury	0.1129
Romania	2000	Nerve injury	0.1129
Romania	2005	Nerve injury	0.1129
Romania	2010	Nerve injury	0.1129
Romania	2017	Nerve injury	0.1129
Romania	1990	Injury to eyes	0.0165
Romania	1995	Injury to eyes	0.0165
Romania	2000	Injury to eyes	0.0165
Romania	2005	Injury to eyes	0.0165
Romania	2010	Injury to eyes	0.0165
Romania	2017	Injury to eyes	0.0165
Romania	1990	Amputation of lower limb, unilateral	0.0666
Romania	1995	Amputation of lower limb, unilateral	0.0613
Romania	2000	Amputation of lower limb, unilateral	0.0534
Romania	2005	Amputation of lower limb, unilateral	0.0465
Romania	2010	Amputation of lower limb, unilateral	0.0401
Romania	2017	Amputation of lower limb, unilateral	0.039
Romania	1990	Open wound(s)	0.011
Romania	1995	Open wound(s)	0.011
Romania	2000	Open wound(s)	0.011
Romania	2005	Open wound(s)	0.011
Romania	2010	Open wound(s)	0.011
Romania	2017	Open wound(s)	0.011
Romania	1990	Poisoning requiring urgent care	0.1628
Romania	1995	Poisoning requiring urgent care	0.1628
Romania	2000	Poisoning requiring urgent care	0.1628
Romania	2005	Poisoning requiring urgent care	0.1628
Romania	2010	Poisoning requiring urgent care	0.1628
Romania	2017	Poisoning requiring urgent care	0.1628
Romania	1990	Severe chest injury	0.0474
Romania	1995	Severe chest injury	0.0474
Romania	2000	Severe chest injury	0.0474
Romania	2005	Severe chest injury	0.0474
Romania	2010	Severe chest injury	0.0474
Romania	2017	Severe chest injury	0.0474
Romania	1990	Internal hemorrhage in abdomen and pelvis	0.1143
Romania	1995	Internal hemorrhage in abdomen and pelvis	0.1143
Romania	2000	Internal hemorrhage in abdomen and pelvis	0.1143
Romania	2005	Internal hemorrhage in abdomen and pelvis	0.1143
Romania	2010	Internal hemorrhage in abdomen and pelvis	0.1143
Romania	2017	Internal hemorrhage in abdomen and pelvis	0.1143
Romania	1990	Contusion in any part of the body	0.0075
Romania	1995	Contusion in any part of the body	0.0075
Romania	2000	Contusion in any part of the body	0.0075
Romania	2005	Contusion in any part of the body	0.0075
Romania	2010	Contusion in any part of the body	0.0075
Romania	2017	Contusion in any part of the body	0.0075
Romania	1990	Effect of different environmental factors	0.049
Romania	1995	Effect of different environmental factors	0.049
Romania	2000	Effect of different environmental factors	0.049
Romania	2005	Effect of different environmental factors	0.049
Romania	2010	Effect of different environmental factors	0.049
Romania	2017	Effect of different environmental factors	0.049
Romania	1990	Complications following therapeutic procedures	0.0506
Romania	1995	Complications following therapeutic procedures	0.0506
Romania	2000	Complications following therapeutic procedures	0.0506
Romania	2005	Complications following therapeutic procedures	0.0506
Romania	2010	Complications following therapeutic procedures	0.0506
Romania	2017	Complications following therapeutic procedures	0.0506
Romania	1990	Multiple fractures, dislocations, crashes, wounds, sprains, and strains	0.1286
Romania	1995	Multiple fractures, dislocations, crashes, wounds, sprains, and strains	0.1149
Romania	2000	Multiple fractures, dislocations, crashes, wounds, sprains, and strains	0.0947
Romania	2005	Multiple fractures, dislocations, crashes, wounds, sprains, and strains	0.0771
Romania	2010	Multiple fractures, dislocations, crashes, wounds, sprains, and strains	0.0607
Romania	2017	Multiple fractures, dislocations, crashes, wounds, sprains, and strains	0.0577
Romania	1990	Amputation of upper limb, unilateral	0.0552
Romania	1995	Amputation of upper limb, unilateral	0.0521
Romania	2000	Amputation of upper limb, unilateral	0.0475
Romania	2005	Amputation of upper limb, unilateral	0.0434
Romania	2010	Amputation of upper limb, unilateral	0.0397
Romania	2017	Amputation of upper limb, unilateral	0.039
Romania	1990	Amputation of thumb	0.0114
Romania	1995	Amputation of thumb	0.0114
Romania	2000	Amputation of thumb	0.0114
Romania	2005	Amputation of thumb	0.0114
Romania	2010	Amputation of thumb	0.0114
Romania	2017	Amputation of thumb	0.0114
Romania	1990	Amputation of toe/toes	0.0059
Romania	1995	Amputation of toe/toes	0.0059
Romania	2000	Amputation of toe/toes	0.0059
Romania	2005	Amputation of toe/toes	0.0059
Romania	2010	Amputation of toe/toes	0.0059
Romania	2017	Amputation of toe/toes	0.0059
Romania	1990	Burns, <20% total burned surface area without lower airway burns	0.0163
Romania	1995	Burns, <20% total burned surface area without lower airway burns	0.0163
Romania	2000	Burns, <20% total burned surface area without lower airway burns	0.0163
Romania	2005	Burns, <20% total burned surface area without lower airway burns	0.0163
Romania	2010	Burns, <20% total burned surface area without lower airway burns	0.0163
Romania	2017	Burns, <20% total burned surface area without lower airway burns	0.0163
Romania	1990	Burns, >=20% total burned surface area or >= 10% burned surface area if head/neck or hands/wrist involved w/o lower airway burns	0.2006
Romania	1995	Burns, >=20% total burned surface area or >= 10% burned surface area if head/neck or hands/wrist involved w/o lower airway burns	0.1879
Romania	2000	Burns, >=20% total burned surface area or >= 10% burned surface area if head/neck or hands/wrist involved w/o lower airway burns	0.1691
Romania	2005	Burns, >=20% total burned surface area or >= 10% burned surface area if head/neck or hands/wrist involved w/o lower airway burns	0.1526
Romania	2010	Burns, >=20% total burned surface area or >= 10% burned surface area if head/neck or hands/wrist involved w/o lower airway burns	0.1374
Romania	2017	Burns, >=20% total burned surface area or >= 10% burned surface area if head/neck or hands/wrist involved w/o lower airway burns	0.1346
Serbia	1990	Amputation of lower limbs, bilateral	0.158
Serbia	1995	Amputation of lower limbs, bilateral	0.159
Serbia	2000	Amputation of lower limbs, bilateral	0.1509
Serbia	2005	Amputation of lower limbs, bilateral	0.1396
Serbia	2010	Amputation of lower limbs, bilateral	0.126
Serbia	2017	Amputation of lower limbs, bilateral	0.1232
Serbia	1990	Lower airway burns	0.2252
Serbia	1995	Lower airway burns	0.2252
Serbia	2000	Lower airway burns	0.2252
Serbia	2005	Lower airway burns	0.2252
Serbia	2010	Lower airway burns	0.2252
Serbia	2017	Lower airway burns	0.2252
Serbia	1990	Dislocation of hip	0.0159
Serbia	1995	Dislocation of hip	0.0159
Serbia	2000	Dislocation of hip	0.0159
Serbia	2005	Dislocation of hip	0.0159
Serbia	2010	Dislocation of hip	0.0159
Serbia	2017	Dislocation of hip	0.0159
Serbia	1990	Dislocation of knee	0.1134
Serbia	1995	Dislocation of knee	0.1134
Serbia	2000	Dislocation of knee	0.1134
Serbia	2005	Dislocation of knee	0.1134
Serbia	2010	Dislocation of knee	0.1134

Serbia	2017	Dislocation of knee	0.1134
Serbia	1990	Dislocation of shoulder	0.062
Serbia	1995	Dislocation of shoulder	0.062
Serbia	2000	Dislocation of shoulder	0.062
Serbia	2005	Dislocation of shoulder	0.062
Serbia	2010	Dislocation of shoulder	0.062
Serbia	2017	Dislocation of shoulder	0.062
Serbia	1990	Muscle and tendon injuries, including sprains and strains lesser dislocations	0.0075
Serbia	1995	Muscle and tendon injuries, including sprains and strains lesser dislocations	0.0075
Serbia	2000	Muscle and tendon injuries, including sprains and strains lesser dislocations	0.0075
Serbia	2005	Muscle and tendon injuries, including sprains and strains lesser dislocations	0.0075
Serbia	2010	Muscle and tendon injuries, including sprains and strains lesser dislocations	0.0075
Serbia	2017	Muscle and tendon injuries, including sprains and strains lesser dislocations	0.0075
Serbia	1990	Fracture of clavicle, scapula, or humerus	0.0349
Serbia	1995	Fracture of clavicle, scapula, or humerus	0.0349
Serbia	2000	Fracture of clavicle, scapula, or humerus	0.0349
Serbia	2005	Fracture of clavicle, scapula, or humerus	0.0349
Serbia	2010	Fracture of clavicle, scapula, or humerus	0.0349
Serbia	2017	Fracture of clavicle, scapula, or humerus	0.0349
Serbia	1990	Fracture of face bones	0.0669
Serbia	1995	Fracture of face bones	0.0669
Serbia	2000	Fracture of face bones	0.0669
Serbia	2005	Fracture of face bones	0.0669
Serbia	2010	Fracture of face bones	0.0669
Serbia	2017	Fracture of face bones	0.0669
Serbia	1990	Fracture of foot bones except ankle	0.0264
Serbia	1995	Fracture of foot bones except ankle	0.0264
Serbia	2000	Fracture of foot bones except ankle	0.0264
Serbia	2005	Fracture of foot bones except ankle	0.0264
Serbia	2010	Fracture of foot bones except ankle	0.0264
Serbia	2017	Fracture of foot bones except ankle	0.0264
Serbia	1990	Fracture of hand (wrist and other distal part of hand)	0.0138
Serbia	1995	Fracture of hand (wrist and other distal part of hand)	0.0138
Serbia	2000	Fracture of hand (wrist and other distal part of hand)	0.0138
Serbia	2005	Fracture of hand (wrist and other distal part of hand)	0.0138
Serbia	2010	Fracture of hand (wrist and other distal part of hand)	0.0138
Serbia	2017	Fracture of hand (wrist and other distal part of hand)	0.0138
Serbia	1990	Fracture of hip	0.1037
Serbia	1995	Fracture of hip	0.105
Serbia	2000	Fracture of hip	0.0942
Serbia	2005	Fracture of hip	0.0792
Serbia	2010	Fracture of hip	0.0613
Serbia	2017	Fracture of hip	0.0577
Serbia	1990	Amputation of upper limbs, bilateral	0.1351
Serbia	1995	Amputation of upper limbs, bilateral	0.1365
Serbia	2000	Amputation of upper limbs, bilateral	0.1254
Serbia	2005	Amputation of upper limbs, bilateral	0.1099
Serbia	2010	Amputation of upper limbs, bilateral	0.0914
Serbia	2017	Amputation of upper limbs, bilateral	0.0876
Serbia	1990	Fracture of patella, tibia or fibula, or ankle	0.0554
Serbia	1995	Fracture of patella, tibia or fibula, or ankle	0.0554
Serbia	2000	Fracture of patella, tibia or fibula, or ankle	0.0554
Serbia	2005	Fracture of patella, tibia or fibula, or ankle	0.0554
Serbia	2010	Fracture of patella, tibia or fibula, or ankle	0.0554
Serbia	2017	Fracture of patella, tibia or fibula, or ankle	0.0554
Serbia	1990	Fracture of pelvis	0.1823
Serbia	1995	Fracture of pelvis	0.1823
Serbia	2000	Fracture of pelvis	0.1823
Serbia	2005	Fracture of pelvis	0.1823
Serbia	2010	Fracture of pelvis	0.1823
Serbia	2017	Fracture of pelvis	0.1823
Serbia	1990	Fracture of radius and/or ulna	0.0435
Serbia	1995	Fracture of radius and/or ulna	0.0435
Serbia	2000	Fracture of radius and/or ulna	0.0435
Serbia	2005	Fracture of radius and/or ulna	0.0435
Serbia	2010	Fracture of radius and/or ulna	0.0435
Serbia	2017	Fracture of radius and/or ulna	0.0435
Serbia	1990	Fracture of skull	0.0714
Serbia	1995	Fracture of skull	0.0714
Serbia	2000	Fracture of skull	0.0714
Serbia	2005	Fracture of skull	0.0714
Serbia	2010	Fracture of skull	0.0714
Serbia	2017	Fracture of skull	0.0714
Serbia	1990	Fracture of sternum and/or fracture of one or more ribs	0.1027
Serbia	1995	Fracture of sternum and/or fracture of one or more ribs	0.1027
Serbia	2000	Fracture of sternum and/or fracture of one or more ribs	0.1027
Serbia	2005	Fracture of sternum and/or fracture of one or more ribs	0.1027
Serbia	2010	Fracture of sternum and/or fracture of one or more ribs	0.1027
Serbia	2017	Fracture of sternum and/or fracture of one or more ribs	0.1027
Serbia	1990	Fracture of vertebral column	0.1106
Serbia	1995	Fracture of vertebral column	0.1106
Serbia	2000	Fracture of vertebral column	0.1106
Serbia	2005	Fracture of vertebral column	0.1106
Serbia	2010	Fracture of vertebral column	0.1106
Serbia	2017	Fracture of vertebral column	0.1106
Serbia	1990	Fracture of femur, other than femoral neck	0.042
Serbia	1995	Fracture of femur, other than femoral neck	0.042
Serbia	2000	Fracture of femur, other than femoral neck	0.042
Serbia	2005	Fracture of femur, other than femoral neck	0.042
Serbia	2010	Fracture of femur, other than femoral neck	0.042
Serbia	2017	Fracture of femur, other than femoral neck	0.042
Serbia	1990	Minor TBI	0.1319
Serbia	1995	Minor TBI	0.1319
Serbia	2000	Minor TBI	0.1319
Serbia	2005	Minor TBI	0.1319
Serbia	2010	Minor TBI	0.1319
Serbia	2017	Minor TBI	0.1319
Serbia	1990	Moderate TBI	0.1637
Serbia	1995	Moderate TBI	0.1637
Serbia	2000	Moderate TBI	0.1637
Serbia	2005	Moderate TBI	0.1637
Serbia	2010	Moderate TBI	0.1637
Serbia	2017	Moderate TBI	0.1637
Serbia	1990	Amputation of fingers (excluding thumb)	0.0047
Serbia	1995	Amputation of fingers (excluding thumb)	0.0047
Serbia	2000	Amputation of fingers (excluding thumb)	0.0047
Serbia	2005	Amputation of fingers (excluding thumb)	0.0047
Serbia	2010	Amputation of fingers (excluding thumb)	0.0047
Serbia	2017	Amputation of fingers (excluding thumb)	0.0047
Serbia	1990	Spinal cord lesion at neck level	0.4277
Serbia	1995	Spinal cord lesion at neck level	0.4282
Serbia	2000	Spinal cord lesion at neck level	0.4245
Serbia	2005	Spinal cord lesion at neck level	0.4193
Serbia	2010	Spinal cord lesion at neck level	0.4131
Serbia	2017	Spinal cord lesion at neck level	0.4118
Serbia	1990	Spinal cord lesion below neck level	0.2112
Serbia	1995	Spinal cord lesion below neck level	0.2122
Serbia	2000	Spinal cord lesion below neck level	0.2043
Serbia	2005	Spinal cord lesion below neck level	0.1934
Serbia	2010	Spinal cord lesion below neck level	0.1803
Serbia	2017	Spinal cord lesion below neck level	0.1777
Serbia	1990	Drowning and nonfatal submersion	0.2471
Serbia	1995	Drowning and nonfatal submersion	0.2471
Serbia	2000	Drowning and nonfatal submersion	0.2471
Serbia	2005	Drowning and nonfatal submersion	0.2471
Serbia	2010	Drowning and nonfatal submersion	0.2471
Serbia	2017	Drowning and nonfatal submersion	0.2471
Serbia	1990	Asphyxiation	0.2471
Serbia	1995	Asphyxiation	0.2471
Serbia	2000	Asphyxiation	0.2471
Serbia	2005	Asphyxiation	0.2471
Serbia	2010	Asphyxiation	0.2471
Serbia	2017	Asphyxiation	0.2471
Serbia	1990	Crush injury	0.1325
Serbia	1995	Crush injury	0.1325
Serbia	2000	Crush injury	0.1325
Serbia	2005	Crush injury	0.1325
Serbia	2010	Crush injury	0.1325
Serbia	2017	Crush injury	0.1325
Serbia	1990	Nerve injury	0.1129
Serbia	1995	Nerve injury	0.1129
Serbia	2000	Nerve injury	0.1129
Serbia	2005	Nerve injury	0.1129
Serbia	2010	Nerve injury	0.1129
Serbia	2017	Nerve injury	0.1129
Serbia	1990	Injury to eyes	0.0165
Serbia	1995	Injury to eyes	0.0165
Serbia	2000	Injury to eyes	0.0165
Serbia	2005	Injury to eyes	0.0165
Serbia	2010	Injury to eyes	0.0165
Serbia	2017	Injury to eyes	0.0165
Serbia	1990	Amputation of lower limb, unilateral	0.0569
Serbia	1995	Amputation of lower limb, unilateral	0.0574
Serbia	2000	Amputation of lower limb, unilateral	0.0532
Serbia	2005	Amputation of lower limb, unilateral	0.0474
Serbia	2010	Amputation of lower limb, unilateral	0.0404
Serbia	2017	Amputation of lower limb, unilateral	0.039
Serbia	1990	Open wound(s)	0.011
Serbia	1995	Open wound(s)	0.011
Serbia	2000	Open wound(s)	0.011
Serbia	2005	Open wound(s)	0.011
Serbia	2010	Open wound(s)	0.011
Serbia	2017	Open wound(s)	0.011
Serbia	1990	Poisoning requiring urgent care	0.1628
Serbia	1995	Poisoning requiring urgent care	0.1628
Serbia	2000	Poisoning requiring urgent care	0.1628
Serbia	2005	Poisoning requiring urgent care	0.1628
Serbia	2010	Poisoning requiring urgent care	0.1628
Serbia	2017	Poisoning requiring urgent care	0.1628
Serbia	1990	Severe chest injury	0.0474
Serbia	1995	Severe chest injury	0.0474
Serbia	2000	Severe chest injury	0.0474
Serbia	2005	Severe chest injury	0.0474
Serbia	2010	Severe chest injury	0.0474
Serbia	2017	Severe chest injury	0.0474
Serbia	1990	Internal hemorrhage in abdomen and pelvis	0.1143
Serbia	1995	Internal hemorrhage in abdomen and pelvis	0.1143
Serbia	2000	Internal hemorrhage in abdomen and pelvis	0.1143
Serbia	2005	Internal hemorrhage in abdomen and pelvis	0.1143
Serbia	2010	Internal hemorrhage in abdomen and pelvis	0.1143
Serbia	2017	Internal hemorrhage in abdomen and pelvis	0.1143
Serbia	1990	Contusion in any part of the body	0.0075
Serbia	1995	Contusion in any part of the body	0.0075
Serbia	2000	Contusion in any part of the body	0.0075
Serbia	2005	Contusion in any part of the body	0.0075
Serbia	2010	Contusion in any part of the body	0.0075
Serbia	2017	Contusion in any part of the body	0.0075
Serbia	1990	Effect of different environmental factors	0.049
Serbia	1995	Effect of different environmental factors	0.049
Serbia	2000	Effect of different environmental factors	0.049
Serbia	2005	Effect of different environmental factors	0.049
Serbia	2010	Effect of different environmental factors	0.049
Serbia	2017	Effect of different environmental factors	0.049
Serbia	1990	Complications following therapeutic procedures	0.0506
Serbia	1995	Complications following therapeutic procedures	0.0506
Serbia	2000	Complications following therapeutic procedures	0.0506
Serbia	2005	Complications following therapeutic procedures	0.0506
Serbia	2010	Complications following therapeutic procedures	0.0506
Serbia	2017	Complications following therapeutic procedures	0.0506
Serbia	1990	Multiple fractures, dislocations, crashes, wounds, sprains, and strains	0.1037
Serbia	1995	Multiple fractures, dislocations, crashes, wounds, sprains, and strains	0.105
Serbia	2000	Multiple fractures, dislocations, crashes, wounds, sprains, and strains	0.0942
Serbia	2005	Multiple fractures, dislocations, crashes, wounds, sprains, and strains	0.0792
Serbia	2010	Multiple fractures, dislocations, crashes, wounds, sprains, and strains	0.0613
Serbia	2017	Multiple fractures, dislocations, crashes, wounds, sprains, and strains	0.0577
Serbia	1990	Amputation of upper limb, unilateral	0.0495
Serbia	1995	Amputation of upper limb, unilateral	0.0498
Serbia	2000	Amputation of upper limb, unilateral	0.0474
Serbia	2005	Amputation of upper limb, unilateral	0.0439
Serbia	2010	Amputation of upper limb, unilateral	0.0398
Serbia	2017	Amputation of upper limb, unilateral	0.039
Serbia	1990	Amputation of thumb	0.0114
Serbia	1995	Amputation of thumb	0.0114
Serbia	2000	Amputation of thumb	0.0114
Serbia	2005	Amputation of thumb	0.0114
Serbia	2010	Amputation of thumb	0.0114
Serbia	2017	Amputation of thumb	0.0114
Serbia	1990	Amputation of toe/toes	0.0059
Serbia	1995	Amputation of toe/toes	0.0059
Serbia	2000	Amputation of toe/toes	0.0059
Serbia	2005	Amputation of toe/toes	0.0059
Serbia	2010	Amputation of toe/toes	0.0059
Serbia	2017	Amputation of toe/toes	0.0059
Serbia	1990	Burns, <20% total burned surface area without lower airway burns	0.0163
Serbia	1995	Burns, <20% total burned surface area without lower airway burns	0.0163
Serbia	2000	Burns, <20% total burned surface area without lower airway burns	0.0163
Serbia	2005	Burns, <20% total burned surface area without lower airway burns	0.0163
Serbia	2010	Burns, <20% total burned surface area without lower airway burns	0.0163
Serbia	2017	Burns, <20% total burned surface area without lower airway burns	0.0163
Serbia	1990	Burns, >=20% total burned surface area or >= 10% burned surface area if head/neck or hands/wrist involved w/o lower airway burns	0.1774
Serbia	1995	Burns, >=20% total burned surface area or >= 10% burned surface area if head/neck or hands/wrist involved w/o lower airway burns	0.1786
Serbia	2000	Burns, >=20% total burned surface area or >= 10% burned surface area if head/neck or hands/wrist involved w/o lower airway burns	0.1686
Serbia	2005	Burns, >=20% total burned surface area or >= 10% burned surface area if head/neck or hands/wrist involved w/o lower airway burns	0.1547
Serbia	2010	Burns, >=20% total burned surface area or >= 10% burned surface area if head/neck or hands/wrist involved w/o lower airway burns	0.1379
Serbia	2017	Burns, >=20% total burned surface area or >= 10% burned surface area if head/neck or hands/wrist involved w/o lower airway burns	0.1346
Slovakia	1990	Amputation of lower limbs, bilateral	0.1477
Slovakia	1995	Amputation of lower limbs, bilateral	0.1352
Slovakia	2000	Amputation of lower limbs, bilateral	0.1279
Slovakia	2005	Amputation of lower limbs, bilateral	0.1232
Slovakia	2010	Amputation of lower limbs, bilateral	0.1232
Slovakia	2017	Amputation of lower limbs, bilateral	0.1232
Slovakia	1990	Lower airway burns	0.2252
Slovakia	1995	Lower airway burns	0.2252
Slovakia	2000	Lower airway burns	0.2252
Slovakia	2005	Lower airway burns	0.2252
Slovakia	2010	Lower airway burns	0.2252
Slovakia	2017	Lower airway burns	0.2252
Slovakia	1990	Dislocation of hip	0.0159
Slovakia	1995	Dislocation of hip	0.0159
Slovakia	2000	Dislocation of hip	0.0159
Slovakia	2005	Dislocation of hip	0.0159
Slovakia	2010	Dislocation of hip	0.0159
Slovakia	2017	Dislocation of hip	0.0159
Slovakia	1990	Dislocation of knee	0.1134
Slovakia	1995	Dislocation of knee	0.1134
Slovakia	2000	Dislocation of knee	0.1134
Slovakia	2005	Dislocation of knee	0.1134
Slovakia	2010	Dislocation of knee	0.1134
Slovakia	2017	Dislocation of knee	0.1134
Slovakia	1990	Dislocation of shoulder	0.062
Slovakia	1995	Dislocation of shoulder	0.062
Slovakia	2000	Dislocation of shoulder	0.062
Slovakia	2005	Dislocation of shoulder	0.062
Slovakia	2010	Dislocation of shoulder	0.062
Slovakia	2017	Dislocation of shoulder	0.062
Slovakia	1990	Muscle and tendon injuries, including sprains and strains lesser dislocations	0.0075
Slovakia	1995	Muscle and tendon injuries, including sprains and strains lesser dislocations	0.0075
Slovakia	2000	Muscle and tendon injuries, including sprains and strains lesser dislocations	0.0075
Slovakia	2005	Muscle and tendon injuries, including sprains and strains lesser dislocations	0.0075
Slovakia	2010	Muscle and tendon injuries, including sprains and strains lesser dislocations	0.0075
Slovakia	2017	Muscle and tendon injuries, including sprains and strains lesser dislocations	0.0075
Slovakia	1990	Fracture of clavicle, scapula, or humerus	0.0349
Slovakia	1995	Fracture of clavicle, scapula, or humerus	0.0349
Slovakia	2000	Fracture of clavicle, scapula, or humerus	0.0349
Slovakia	2005	Fracture of clavicle, scapula, or humerus	0.0349
Slovakia	2010	Fracture of clavicle, scapula, or humerus	0.0349
Slovakia	2017	Fracture of clavicle, scapula, or humerus	0.0349
Slovakia	1990	Fracture of face bones	0.0669
Slovakia	1995	Fracture of face bones	0.0669
Slovakia	2000	Fracture of face bones	0.0669
Slovakia	2005	Fracture of face bones	0.0669
Slovakia	2010	Fracture of face bones	0.0669
Slovakia	2017	Fracture of face bones	0.0669
Slovakia	1990	Fracture of foot bones except ankle	0.0264
Slovakia	1995	Fracture of foot bones except ankle	0.0264
Slovakia	2000	Fracture of foot bones except ankle	0.0264
Slovakia	2005	Fracture of foot bones except ankle	0.0264
Slovakia	2010	Fracture of foot bones except ankle	0.0264
Slovakia	2017	Fracture of foot bones except ankle	0.0264
Slovakia	1990	Fracture of hand (wrist and other distal part of hand)	0.0138
Slovakia	1995	Fracture of hand (wrist and other distal part of hand)	0.0138
Slovakia	2000	Fracture of hand (wrist and other distal part of hand)	0.0138
Slovakia	2005	Fracture of hand (wrist and other distal part of hand)	0.0138
Slovakia	2010	Fracture of hand (wrist and other distal part of hand)	0.0138
Slovakia	2017	Fracture of hand (wrist and other distal part of hand)	0.0138
Slovakia	1990	Fracture of hip	0.0901
Slovakia	1995	Fracture of hip	0.0735
Slovakia	2000	Fracture of hip	0.0638
Slovakia	2005	Fracture of hip	0.0577
Slovakia	2010	Fracture of hip	0.0577
Slovakia	2017	Fracture of hip	0.0577
Slovakia	1990	Amputation of upper limbs, bilateral	0.1211
Slovakia	1995	Amputation of upper limbs, bilateral	0.1039
Slovakia	2000	Amputation of upper limbs, bilateral	0.094
Slovakia	2005	Amputation of upper limbs, bilateral	0.0876
Slovakia	2010	Amputation of upper limbs, bilateral	0.0876
Slovakia	2017	Amputation of upper limbs, bilateral	0.0876
Slovakia	1990	Fracture of patella, tibia or fibula, or ankle	0.0554
Slovakia	1995	Fracture of patella, tibia or fibula, or ankle	0.0554
Slovakia	2000	Fracture of patella, tibia or fibula, or ankle	0.0554
Slovakia	2005	Fracture of patella, tibia or fibula, or ankle	0.0554
Slovakia	2010	Fracture of patella, tibia or fibula, or ankle	0.0554
Slovakia	2017	Fracture of patella, tibia or fibula, or ankle	0.0554
Slovakia	1990	Fracture of pelvis	0.1823
Slovakia	1995	Fracture of pelvis	0.1823
Slovakia	2000	Fracture of pelvis	0.1823
Slovakia	2005	Fracture of pelvis	0.1823
Slovakia	2010	Fracture of pelvis	0.1823
Slovakia	2017	Fracture of pelvis	0.1823
Slovakia	1990	Fracture of radius and/or ulna	0.0435
Slovakia	1995	Fracture of radius and/or ulna	0.0435
Slovakia	2000	Fracture of radius and/or ulna	0.0435
Slovakia	2005	Fracture of radius and/or ulna	0.0435
Slovakia	2010	Fracture of radius and/or ulna	0.0435
Slovakia	2017	Fracture of radius and/or ulna	0.0435
Slovakia	1990	Fracture of skull	0.0714
Slovakia	1995	Fracture of skull	0.0714
Slovakia	2000	Fracture of skull	0.0714
Slovakia	2005	Fracture of skull	0.0714
Slovakia	2010	Fracture of skull	0.0714
Slovakia	2017	Fracture of skull	0.0714
Slovakia	1990	Fracture of sternum and/or fracture of one or more ribs	0.1027
Slovakia	1995	Fracture of sternum and/or fracture of one or more ribs	0.1027
Slovakia	2000	Fracture of sternum and/or fracture of one or more ribs	0.1027
Slovakia	2005	Fracture of sternum and/or fracture of one or more ribs	0.1027
Slovakia	2010	Fracture of sternum and/or fracture of one or more ribs	0.1027
Slovakia	2017	Fracture of sternum and/or fracture of one or more ribs	0.1027
Slovakia	1990	Fracture of vertebral column	0.1106
Slovakia	1995	Fracture of vertebral column	0.1106
Slovakia	2000	Fracture of vertebral column	0.1106
Slovakia	2005	Fracture of vertebral column	0.1106
Slovakia	2010	Fracture of vertebral column	0.1106
Slovakia	2017	Fracture of vertebral column	0.1106
Slovakia	1990	Fracture of femur, other than femoral neck	0.042
Slovakia	1995	Fracture of femur, other than femoral neck	0.042
Slovakia	2000	Fracture of femur, other than femoral neck	0.042
Slovakia	2005	Fracture of femur, other than femoral neck	0.042
Slovakia	2010	Fracture of femur, other than femoral neck	0.042
Slovakia	2017	Fracture of femur, other than femoral neck	0.042
Slovakia	1990	Minor TBI	0.1319
Slovakia	1995	Minor TBI	0.1319
Slovakia	2000	Minor TBI	0.1319
Slovakia	2005	Minor TBI	0.1319
Slovakia	2010	Minor TBI	0.1319
Slovakia	2017	Minor TBI	0.1319
Slovakia	1990	Moderate TBI	0.1637
Slovakia	1995	Moderate TBI	0.1637
Slovakia	2000	Moderate TBI	0.1637
Slovakia	2005	Moderate TBI	0.1637
Slovakia	2010	Moderate TBI	0.1637
Slovakia	2017	Moderate TBI	0.1637
Slovakia	1990	Amputation of fingers (excluding thumb)	0.0047
Slovakia	1995	Amputation of fingers (excluding thumb)	0.0047
Slovakia	2000	Amputation of fingers (excluding thumb)	0.0047
Slovakia	2005	Amputation of fingers (excluding thumb)	0.0047
Slovakia	2010	Amputation of fingers (excluding thumb)	0.0047
Slovakia	2017	Amputation of fingers (excluding thumb)	0.0047
Slovakia	1990	Spinal cord lesion at neck level	0.423
Slovakia	1995	Spinal cord lesion at neck level	0.4173
Slovakia	2000	Spinal cord lesion at neck level	0.4139
Slovakia	2005	Spinal cord lesion at neck level	0.4118
Slovakia	2010	Spinal cord lesion at neck level	0.4118
Slovakia	2017	Spinal cord lesion at neck level	0.4118
Slovakia	1990	Spinal cord lesion below neck level	0.2013
Slovakia	1995	Spinal cord lesion below neck level	0.1892
Slovakia	2000	Spinal cord lesion below neck level	0.1822
Slovakia	2005	Spinal cord lesion below neck level	0.1777
Slovakia	2010	Spinal cord lesion below neck level	0.1777
Slovakia	2017	Spinal cord lesion below neck level	0.1777
Slovakia	1990	Drowning and nonfatal submersion	0.2471
Slovakia	1995	Drowning and nonfatal submersion	0.2471
Slovakia	2000	Drowning and nonfatal submersion	0.2471
Slovakia	2005	Drowning and nonfatal submersion	0.2471
Slovakia	2010	Drowning and nonfatal submersion	0.2471
Slovakia	2017	Drowning and nonfatal submersion	0.2471
Slovakia	1990	Asphyxiation	0.2471
Slovakia	1995	Asphyxiation	0.2471
Slovakia	2000	Asphyxiation	0.2471
Slovakia	2005	Asphyxiation	0.2471
Slovakia	2010	Asphyxiation	0.2471
Slovakia	2017	Asphyxiation	0.2471
Slovakia	1990	Crush injury	0.1325
Slovakia	1995	Crush injury	0.1325
Slovakia	2000	Crush injury	0.1325
Slovakia	2005	Crush injury	0.1325
Slovakia	2010	Crush injury	0.1325
Slovakia	2017	Crush injury	0.1325
Slovakia	1990	Nerve injury	0.1129
Slovakia	1995	Nerve injury	0.1129
Slovakia	2000	Nerve injury	0.1129
Slovakia	2005	Nerve injury	0.1129
Slovakia	2010	Nerve injury	0.1129
Slovakia	2017	Nerve injury	0.1129
Slovakia	1990	Injury to eyes	0.0165
Slovakia	1995	Injury to eyes	0.0165
Slovakia	2000	Injury to eyes	0.0165
Slovakia	2005	Injury to eyes	0.0165
Slovakia	2010	Injury to eyes	0.0165
Slovakia	2017	Injury to eyes	0.0165
Slovakia	1990	Amputation of lower limb, unilateral	0.0516
Slovakia	1995	Amputation of lower limb, unilateral	0.0451
Slovakia	2000	Amputation of lower limb, unilateral	0.0414
Slovakia	2005	Amputation of lower limb, unilateral	0.039
Slovakia	2010	Amputation of lower limb, unilateral	0.039
Slovakia	2017	Amputation of lower limb, unilateral	0.039
Slovakia	1990	Open wound(s)	0.011
Slovakia	1995	Open wound(s)	0.011
Slovakia	2000	Open wound(s)	0.011
Slovakia	2005	Open wound(s)	0.011
Slovakia	2010	Open wound(s)	0.011
Slovakia	2017	Open wound(s)	0.011
Slovakia	1990	Poisoning requiring urgent care	0.1628
Slovakia	1995	Poisoning requiring urgent care	0.1628
Slovakia	2000	Poisoning requiring urgent care	0.1628
Slovakia	2005	Poisoning requiring urgent care	0.1628
Slovakia	2010	Poisoning requiring urgent care	0.1628
Slovakia	2017	Poisoning requiring urgent care	0.1628
Slovakia	1990	Severe chest injury	0.0474
Slovakia	1995	Severe chest injury	0.0474
Slovakia	2000	Severe chest injury	0.0474
Slovakia	2005	Severe chest injury	0.0474

Slovakia	2010	Severe chest injury	0.0474
Slovakia	2017	Severe chest injury	0.0474
Slovakia	1990	Internal hemorrhage in abdomen and pelvis	0.1143
Slovakia	1995	Internal hemorrhage in abdomen and pelvis	0.1143
Slovakia	2000	Internal hemorrhage in abdomen and pelvis	0.1143
Slovakia	2005	Internal hemorrhage in abdomen and pelvis	0.1143
Slovakia	2010	Internal hemorrhage in abdomen and pelvis	0.1143
Slovakia	2017	Internal hemorrhage in abdomen and pelvis	0.1143
Slovakia	1990	Contusion in any part of the body	0.0075
Slovakia	1995	Contusion in any part of the body	0.0075
Slovakia	2000	Contusion in any part of the body	0.0075
Slovakia	2005	Contusion in any part of the body	0.0075
Slovakia	2010	Contusion in any part of the body	0.0075
Slovakia	2017	Contusion in any part of the body	0.0075
Slovakia	1990	Effect of different environmental factors	0.049
Slovakia	1995	Effect of different environmental factors	0.049
Slovakia	2000	Effect of different environmental factors	0.049
Slovakia	2005	Effect of different environmental factors	0.049
Slovakia	2010	Effect of different environmental factors	0.049
Slovakia	2017	Effect of different environmental factors	0.049
Slovakia	1990	Complications following therapeutic procedures	0.0506
Slovakia	1995	Complications following therapeutic procedures	0.0506
Slovakia	2000	Complications following therapeutic procedures	0.0506
Slovakia	2005	Complications following therapeutic procedures	0.0506
Slovakia	2010	Complications following therapeutic procedures	0.0506
Slovakia	2017	Complications following therapeutic procedures	0.0506
Slovakia	1990	Multiple fractures, dislocations, crashes, wounds, sprains, and strains	0.0901
Slovakia	1995	Multiple fractures, dislocations, crashes, wounds, sprains, and strains	0.0735
Slovakia	2000	Multiple fractures, dislocations, crashes, wounds, sprains, and strains	0.0638
Slovakia	2005	Multiple fractures, dislocations, crashes, wounds, sprains, and strains	0.0577
Slovakia	2010	Multiple fractures, dislocations, crashes, wounds, sprains, and strains	0.0577
Slovakia	2017	Multiple fractures, dislocations, crashes, wounds, sprains, and strains	0.0577
Slovakia	1990	Amputation of upper limb, unilateral	0.0464
Slovakia	1995	Amputation of upper limb, unilateral	0.0426
Slovakia	2000	Amputation of upper limb, unilateral	0.0404
Slovakia	2005	Amputation of upper limb, unilateral	0.039
Slovakia	2010	Amputation of upper limb, unilateral	0.039
Slovakia	2017	Amputation of upper limb, unilateral	0.039
Slovakia	1990	Amputation of thumb	0.0114
Slovakia	1995	Amputation of thumb	0.0114
Slovakia	2000	Amputation of thumb	0.0114
Slovakia	2005	Amputation of thumb	0.0114
Slovakia	2010	Amputation of thumb	0.0114
Slovakia	2017	Amputation of thumb	0.0114
Slovakia	1990	Amputation of toe/toes	0.0059
Slovakia	1995	Amputation of toe/toes	0.0059
Slovakia	2000	Amputation of toe/toes	0.0059
Slovakia	2005	Amputation of toe/toes	0.0059
Slovakia	2010	Amputation of toe/toes	0.0059
Slovakia	2017	Amputation of toe/toes	0.0059
Slovakia	1990	Burns, <20% total burned surface area without lower airway burns	0.0163
Slovakia	1995	Burns, <20% total burned surface area without lower airway burns	0.0163
Slovakia	2000	Burns, <20% total burned surface area without lower airway burns	0.0163
Slovakia	2005	Burns, <20% total burned surface area without lower airway burns	0.0163
Slovakia	2010	Burns, <20% total burned surface area without lower airway burns	0.0163
Slovakia	2017	Burns, <20% total burned surface area without lower airway burns	0.0163
Slovakia	1990	Burns, >=20% total burned surface area or >= 10% burned surface area if head/neck or hands/wrist involved w/o lower airway burns	0.1647
Slovakia	1995	Burns, >=20% total burned surface area or >= 10% burned surface area if head/neck or hands/wrist involved w/o lower airway burns	0.1493
Slovakia	2000	Burns, >=20% total burned surface area or >= 10% burned surface area if head/neck or hands/wrist involved w/o lower airway burns	0.1403
Slovakia	2005	Burns, >=20% total burned surface area or >= 10% burned surface area if head/neck or hands/wrist involved w/o lower airway burns	0.1346
Slovakia	2010	Burns, >=20% total burned surface area or >= 10% burned surface area if head/neck or hands/wrist involved w/o lower airway burns	0.1346
Slovakia	2017	Burns, >=20% total burned surface area or >= 10% burned surface area if head/neck or hands/wrist involved w/o lower airway burns	0.1346
Slovenia	1990	Amputation of lower limbs, bilateral	0.1261
Slovenia	1995	Amputation of lower limbs, bilateral	0.1232
Slovenia	2000	Amputation of lower limbs, bilateral	0.1232
Slovenia	2005	Amputation of lower limbs, bilateral	0.1232
Slovenia	2010	Amputation of lower limbs, bilateral	0.1232
Slovenia	2017	Amputation of lower limbs, bilateral	0.1232
Slovenia	1990	Lower airway burns	0.2252
Slovenia	1995	Lower airway burns	0.2252
Slovenia	2000	Lower airway burns	0.2252
Slovenia	2005	Lower airway burns	0.2252
Slovenia	2010	Lower airway burns	0.2252
Slovenia	2017	Lower airway burns	0.2252
Slovenia	1990	Dislocation of hip	0.0159
Slovenia	1995	Dislocation of hip	0.0159
Slovenia	2000	Dislocation of hip	0.0159
Slovenia	2005	Dislocation of hip	0.0159
Slovenia	2010	Dislocation of hip	0.0159
Slovenia	2017	Dislocation of hip	0.0159
Slovenia	1990	Dislocation of knee	0.1134
Slovenia	1995	Dislocation of knee	0.1134
Slovenia	2000	Dislocation of knee	0.1134
Slovenia	2005	Dislocation of knee	0.1134
Slovenia	2010	Dislocation of knee	0.1134
Slovenia	2017	Dislocation of knee	0.1134
Slovenia	1990	Dislocation of shoulder	0.062
Slovenia	1995	Dislocation of shoulder	0.062
Slovenia	2000	Dislocation of shoulder	0.062
Slovenia	2005	Dislocation of shoulder	0.062
Slovenia	2010	Dislocation of shoulder	0.062
Slovenia	2017	Dislocation of shoulder	0.062
Slovenia	1990	Muscle and tendon injuries, including sprains and strains lesser dislocations	0.0075
Slovenia	1995	Muscle and tendon injuries, including sprains and strains lesser dislocations	0.0075
Slovenia	2000	Muscle and tendon injuries, including sprains and strains lesser dislocations	0.0075
Slovenia	2005	Muscle and tendon injuries, including sprains and strains lesser dislocations	0.0075
Slovenia	2010	Muscle and tendon injuries, including sprains and strains lesser dislocations	0.0075
Slovenia	2017	Muscle and tendon injuries, including sprains and strains lesser dislocations	0.0075
Slovenia	1990	Fracture of clavicle, scapula, or humerus	0.0349
Slovenia	1995	Fracture of clavicle, scapula, or humerus	0.0349
Slovenia	2000	Fracture of clavicle, scapula, or humerus	0.0349
Slovenia	2005	Fracture of clavicle, scapula, or humerus	0.0349
Slovenia	2010	Fracture of clavicle, scapula, or humerus	0.0349
Slovenia	2017	Fracture of clavicle, scapula, or humerus	0.0349
Slovenia	1990	Fracture of face bones	0.0669
Slovenia	1995	Fracture of face bones	0.0669
Slovenia	2000	Fracture of face bones	0.0669
Slovenia	2005	Fracture of face bones	0.0669
Slovenia	2010	Fracture of face bones	0.0669
Slovenia	2017	Fracture of face bones	0.0669
Slovenia	1990	Fracture of foot bones except ankle	0.0264
Slovenia	1995	Fracture of foot bones except ankle	0.0264
Slovenia	2000	Fracture of foot bones except ankle	0.0264
Slovenia	2005	Fracture of foot bones except ankle	0.0264
Slovenia	2010	Fracture of foot bones except ankle	0.0264
Slovenia	2017	Fracture of foot bones except ankle	0.0264
Slovenia	1990	Fracture of hand (wrist and other distal part of hand)	0.0138
Slovenia	1995	Fracture of hand (wrist and other distal part of hand)	0.0138
Slovenia	2000	Fracture of hand (wrist and other distal part of hand)	0.0138
Slovenia	2005	Fracture of hand (wrist and other distal part of hand)	0.0138
Slovenia	2010	Fracture of hand (wrist and other distal part of hand)	0.0138
Slovenia	2017	Fracture of hand (wrist and other distal part of hand)	0.0138
Slovenia	1990	Fracture of hip	0.0615
Slovenia	1995	Fracture of hip	0.0577
Slovenia	2000	Fracture of hip	0.0577
Slovenia	2005	Fracture of hip	0.0577
Slovenia	2010	Fracture of hip	0.0577
Slovenia	2017	Fracture of hip	0.0577
Slovenia	1990	Amputation of upper limbs, bilateral	0.0916
Slovenia	1995	Amputation of upper limbs, bilateral	0.0876
Slovenia	2000	Amputation of upper limbs, bilateral	0.0876
Slovenia	2005	Amputation of upper limbs, bilateral	0.0876
Slovenia	2010	Amputation of upper limbs, bilateral	0.0876
Slovenia	2017	Amputation of upper limbs, bilateral	0.0876
Slovenia	1990	Fracture of patella, tibia or fibula, or ankle	0.0554
Slovenia	1995	Fracture of patella, tibia or fibula, or ankle	0.0554
Slovenia	2000	Fracture of patella, tibia or fibula, or ankle	0.0554
Slovenia	2005	Fracture of patella, tibia or fibula, or ankle	0.0554
Slovenia	2010	Fracture of patella, tibia or fibula, or ankle	0.0554
Slovenia	2017	Fracture of patella, tibia or fibula, or ankle	0.0554
Slovenia	1990	Fracture of pelvis	0.1823
Slovenia	1995	Fracture of pelvis	0.1823
Slovenia	2000	Fracture of pelvis	0.1823
Slovenia	2005	Fracture of pelvis	0.1823
Slovenia	2010	Fracture of pelvis	0.1823
Slovenia	2017	Fracture of pelvis	0.1823
Slovenia	1990	Fracture of radius and/or ulna	0.0435
Slovenia	1995	Fracture of radius and/or ulna	0.0435
Slovenia	2000	Fracture of radius and/or ulna	0.0435
Slovenia	2005	Fracture of radius and/or ulna	0.0435
Slovenia	2010	Fracture of radius and/or ulna	0.0435
Slovenia	2017	Fracture of radius and/or ulna	0.0435
Slovenia	1990	Fracture of skull	0.0714
Slovenia	1995	Fracture of skull	0.0714
Slovenia	2000	Fracture of skull	0.0714
Slovenia	2005	Fracture of skull	0.0714
Slovenia	2010	Fracture of skull	0.0714
Slovenia	2017	Fracture of skull	0.0714
Slovenia	1990	Fracture of sternum and/or fracture of one or more ribs	0.1027
Slovenia	1995	Fracture of sternum and/or fracture of one or more ribs	0.1027
Slovenia	2000	Fracture of sternum and/or fracture of one or more ribs	0.1027
Slovenia	2005	Fracture of sternum and/or fracture of one or more ribs	0.1027
Slovenia	2010	Fracture of sternum and/or fracture of one or more ribs	0.1027
Slovenia	2017	Fracture of sternum and/or fracture of one or more ribs	0.1027
Slovenia	1990	Fracture of vertebral column	0.1106
Slovenia	1995	Fracture of vertebral column	0.1106
Slovenia	2000	Fracture of vertebral column	0.1106
Slovenia	2005	Fracture of vertebral column	0.1106
Slovenia	2010	Fracture of vertebral column	0.1106
Slovenia	2017	Fracture of vertebral column	0.1106
Slovenia	1990	Fracture of femur, other than femoral neck	0.042
Slovenia	1995	Fracture of femur, other than femoral neck	0.042
Slovenia	2000	Fracture of femur, other than femoral neck	0.042
Slovenia	2005	Fracture of femur, other than femoral neck	0.042
Slovenia	2010	Fracture of femur, other than femoral neck	0.042
Slovenia	2017	Fracture of femur, other than femoral neck	0.042
Slovenia	1990	Minor TBI	0.1319
Slovenia	1995	Minor TBI	0.1319
Slovenia	2000	Minor TBI	0.1319
Slovenia	2005	Minor TBI	0.1319
Slovenia	2010	Minor TBI	0.1319
Slovenia	2017	Minor TBI	0.1319
Slovenia	1990	Moderate TBI	0.1637
Slovenia	1995	Moderate TBI	0.1637
Slovenia	2000	Moderate TBI	0.1637
Slovenia	2005	Moderate TBI	0.1637
Slovenia	2010	Moderate TBI	0.1637
Slovenia	2017	Moderate TBI	0.1637
Slovenia	1990	Amputation of fingers (excluding thumb)	0.0047
Slovenia	1995	Amputation of fingers (excluding thumb)	0.0047
Slovenia	2000	Amputation of fingers (excluding thumb)	0.0047
Slovenia	2005	Amputation of fingers (excluding thumb)	0.0047
Slovenia	2010	Amputation of fingers (excluding thumb)	0.0047
Slovenia	2017	Amputation of fingers (excluding thumb)	0.0047
Slovenia	1990	Spinal cord lesion at neck level	0.4131
Slovenia	1995	Spinal cord lesion at neck level	0.4118
Slovenia	2000	Spinal cord lesion at neck level	0.4118
Slovenia	2005	Spinal cord lesion at neck level	0.4118
Slovenia	2010	Spinal cord lesion at neck level	0.4118
Slovenia	2017	Spinal cord lesion at neck level	0.4118
Slovenia	1990	Spinal cord lesion below neck level	0.1804
Slovenia	1995	Spinal cord lesion below neck level	0.1777
Slovenia	2000	Spinal cord lesion below neck level	0.1777
Slovenia	2005	Spinal cord lesion below neck level	0.1777
Slovenia	2010	Spinal cord lesion below neck level	0.1777
Slovenia	2017	Spinal cord lesion below neck level	0.1777
Slovenia	1990	Drowning and nonfatal submersion	0.2471
Slovenia	1995	Drowning and nonfatal submersion	0.2471
Slovenia	2000	Drowning and nonfatal submersion	0.2471
Slovenia	2005	Drowning and nonfatal submersion	0.2471
Slovenia	2010	Drowning and nonfatal submersion	0.2471
Slovenia	2017	Drowning and nonfatal submersion	0.2471
Slovenia	1990	Asphyxiation	0.2471
Slovenia	1995	Asphyxiation	0.2471
Slovenia	2000	Asphyxiation	0.2471
Slovenia	2005	Asphyxiation	0.2471
Slovenia	2010	Asphyxiation	0.2471
Slovenia	2017	Asphyxiation	0.2471
Slovenia	1990	Crush injury	0.1325
Slovenia	1995	Crush injury	0.1325
Slovenia	2000	Crush injury	0.1325
Slovenia	2005	Crush injury	0.1325
Slovenia	2010	Crush injury	0.1325
Slovenia	2017	Crush injury	0.1325
Slovenia	1990	Nerve injury	0.1129
Slovenia	1995	Nerve injury	0.1129
Slovenia	2000	Nerve injury	0.1129
Slovenia	2005	Nerve injury	0.1129
Slovenia	2010	Nerve injury	0.1129
Slovenia	2017	Nerve injury	0.1129
Slovenia	1990	Injury to eyes	0.0165
Slovenia	1995	Injury to eyes	0.0165
Slovenia	2000	Injury to eyes	0.0165
Slovenia	2005	Injury to eyes	0.0165
Slovenia	2010	Injury to eyes	0.0165
Slovenia	2017	Injury to eyes	0.0165
Slovenia	1990	Amputation of lower limb, unilateral	0.0404
Slovenia	1995	Amputation of lower limb, unilateral	0.039
Slovenia	2000	Amputation of lower limb, unilateral	0.039
Slovenia	2005	Amputation of lower limb, unilateral	0.039
Slovenia	2010	Amputation of lower limb, unilateral	0.039
Slovenia	2017	Amputation of lower limb, unilateral	0.039
Slovenia	1990	Open wound(s)	0.011
Slovenia	1995	Open wound(s)	0.011
Slovenia	2000	Open wound(s)	0.011
Slovenia	2005	Open wound(s)	0.011
Slovenia	2010	Open wound(s)	0.011
Slovenia	2017	Open wound(s)	0.011
Slovenia	1990	Poisoning requiring urgent care	0.1628
Slovenia	1995	Poisoning requiring urgent care	0.1628
Slovenia	2000	Poisoning requiring urgent care	0.1628
Slovenia	2005	Poisoning requiring urgent care	0.1628
Slovenia	2010	Poisoning requiring urgent care	0.1628
Slovenia	2017	Poisoning requiring urgent care	0.1628
Slovenia	1990	Severe chest injury	0.0474
Slovenia	1995	Severe chest injury	0.0474
Slovenia	2000	Severe chest injury	0.0474
Slovenia	2005	Severe chest injury	0.0474
Slovenia	2010	Severe chest injury	0.0474
Slovenia	2017	Severe chest injury	0.0474
Slovenia	1990	Internal hemorrhage in abdomen and pelvis	0.1143
Slovenia	1995	Internal hemorrhage in abdomen and pelvis	0.1143
Slovenia	2000	Internal hemorrhage in abdomen and pelvis	0.1143
Slovenia	2005	Internal hemorrhage in abdomen and pelvis	0.1143
Slovenia	2010	Internal hemorrhage in abdomen and pelvis	0.1143
Slovenia	2017	Internal hemorrhage in abdomen and pelvis	0.1143
Slovenia	1990	Contusion in any part of the body	0.0075
Slovenia	1995	Contusion in any part of the body	0.0075
Slovenia	2000	Contusion in any part of the body	0.0075
Slovenia	2005	Contusion in any part of the body	0.0075
Slovenia	2010	Contusion in any part of the body	0.0075
Slovenia	2017	Contusion in any part of the body	0.0075
Slovenia	1990	Effect of different environmental factors	0.049
Slovenia	1995	Effect of different environmental factors	0.049
Slovenia	2000	Effect of different environmental factors	0.049
Slovenia	2005	Effect of different environmental factors	0.049
Slovenia	2010	Effect of different environmental factors	0.049
Slovenia	2017	Effect of different environmental factors	0.049
Slovenia	1990	Complications following therapeutic procedures	0.0506
Slovenia	1995	Complications following therapeutic procedures	0.0506
Slovenia	2000	Complications following therapeutic procedures	0.0506
Slovenia	2005	Complications following therapeutic procedures	0.0506
Slovenia	2010	Complications following therapeutic procedures	0.0506
Slovenia	2017	Complications following therapeutic procedures	0.0506
Slovenia	1990	Multiple fractures, dislocations, crashes, wounds, sprains, and strains	0.0615
Slovenia	1995	Multiple fractures, dislocations, crashes, wounds, sprains, and strains	0.0577
Slovenia	2000	Multiple fractures, dislocations, crashes, wounds, sprains, and strains	0.0577
Slovenia	2005	Multiple fractures, dislocations, crashes, wounds, sprains, and strains	0.0577
Slovenia	2010	Multiple fractures, dislocations, crashes, wounds, sprains, and strains	0.0577
Slovenia	2017	Multiple fractures, dislocations, crashes, wounds, sprains, and strains	0.0577
Slovenia	1990	Amputation of upper limb, unilateral	0.0399
Slovenia	1995	Amputation of upper limb, unilateral	0.039
Slovenia	2000	Amputation of upper limb, unilateral	0.039
Slovenia	2005	Amputation of upper limb, unilateral	0.039
Slovenia	2010	Amputation of upper limb, unilateral	0.039
Slovenia	2017	Amputation of upper limb, unilateral	0.039
Slovenia	1990	Amputation of thumb	0.0114
Slovenia	1995	Amputation of thumb	0.0114
Slovenia	2000	Amputation of thumb	0.0114
Slovenia	2005	Amputation of thumb	0.0114
Slovenia	2010	Amputation of thumb	0.0114
Slovenia	2017	Amputation of thumb	0.0114
Slovenia	1990	Amputation of toe/toes	0.0059
Slovenia	1995	Amputation of toe/toes	0.0059
Slovenia	2000	Amputation of toe/toes	0.0059
Slovenia	2005	Amputation of toe/toes	0.0059
Slovenia	2010	Amputation of toe/toes	0.0059
Slovenia	2017	Amputation of toe/toes	0.0059
Slovenia	1990	Burns, <20% total burned surface area without lower airway burns	0.0163
Slovenia	1995	Burns, <20% total burned surface area without lower airway burns	0.0163
Slovenia	2000	Burns, <20% total burned surface area without lower airway burns	0.0163
Slovenia	2005	Burns, <20% total burned surface area without lower airway burns	0.0163
Slovenia	2010	Burns, <20% total burned surface area without lower airway burns	0.0163
Slovenia	2017	Burns, <20% total burned surface area without lower airway burns	0.0163
Slovenia	1990	Burns, >=20% total burned surface area or >= 10% burned surface area if head/neck or hands/wrist involved w/o lower airway burns	0.1381
Slovenia	1995	Burns, >=20% total burned surface area or >= 10% burned surface area if head/neck or hands/wrist involved w/o lower airway burns	0.1346
Slovenia	2000	Burns, >=20% total burned surface area or >= 10% burned surface area if head/neck or hands/wrist involved w/o lower airway burns	0.1346
Slovenia	2005	Burns, >=20% total burned surface area or >= 10% burned surface area if head/neck or hands/wrist involved w/o lower airway burns	0.1346
Slovenia	2010	Burns, >=20% total burned surface area or >= 10% burned surface area if head/neck or hands/wrist involved w/o lower airway burns	0.1346
Slovenia	2017	Burns, >=20% total burned surface area or >= 10% burned surface area if head/neck or hands/wrist involved w/o lower airway burns	0.1346
Belarus	1990	Amputation of lower limbs, bilateral	0.1581
Belarus	1995	Amputation of lower limbs, bilateral	0.1581
Belarus	2000	Amputation of lower limbs, bilateral	0.1538
Belarus	2005	Amputation of lower limbs, bilateral	0.1441
Belarus	2010	Amputation of lower limbs, bilateral	0.1291
Belarus	2017	Amputation of lower limbs, bilateral	0.1232
Belarus	1990	Lower airway burns	0.2252
Belarus	1995	Lower airway burns	0.2252
Belarus	2000	Lower airway burns	0.2252
Belarus	2005	Lower airway burns	0.2252
Belarus	2010	Lower airway burns	0.2252
Belarus	2017	Lower airway burns	0.2252
Belarus	1990	Dislocation of hip	0.0159
Belarus	1995	Dislocation of hip	0.0159
Belarus	2000	Dislocation of hip	0.0159
Belarus	2005	Dislocation of hip	0.0159
Belarus	2010	Dislocation of hip	0.0159
Belarus	2017	Dislocation of hip	0.0159
Belarus	1990	Dislocation of knee	0.1134
Belarus	1995	Dislocation of knee	0.1134
Belarus	2000	Dislocation of knee	0.1134
Belarus	2005	Dislocation of knee	0.1134
Belarus	2010	Dislocation of knee	0.1134
Belarus	2017	Dislocation of knee	0.1134
Belarus	1990	Dislocation of shoulder	0.062
Belarus	1995	Dislocation of shoulder	0.062
Belarus	2000	Dislocation of shoulder	0.062
Belarus	2005	Dislocation of shoulder	0.062
Belarus	2010	Dislocation of shoulder	0.062
Belarus	2017	Dislocation of shoulder	0.062
Belarus	1990	Muscle and tendon injuries, including sprains and strains lesser dislocations	0.0075
Belarus	1995	Muscle and tendon injuries, including sprains and strains lesser dislocations	0.0075
Belarus	2000	Muscle and tendon injuries, including sprains and strains lesser dislocations	0.0075
Belarus	2005	Muscle and tendon injuries, including sprains and strains lesser dislocations	0.0075
Belarus	2010	Muscle and tendon injuries, including sprains and strains lesser dislocations	0.0075
Belarus	2017	Muscle and tendon injuries, including sprains and strains lesser dislocations	0.0075
Belarus	1990	Fracture of clavicle, scapula, or humerus	0.0349
Belarus	1995	Fracture of clavicle, scapula, or humerus	0.0349
Belarus	2000	Fracture of clavicle, scapula, or humerus	0.0349
Belarus	2005	Fracture of clavicle, scapula, or humerus	0.0349
Belarus	2010	Fracture of clavicle, scapula, or humerus	0.0349
Belarus	2017	Fracture of clavicle, scapula, or humerus	0.0349
Belarus	1990	Fracture of face bones	0.0669
Belarus	1995	Fracture of face bones	0.0669
Belarus	2000	Fracture of face bones	0.0669
Belarus	2005	Fracture of face bones	0.0669
Belarus	2010	Fracture of face bones	0.0669
Belarus	2017	Fracture of face bones	0.0669
Belarus	1990	Fracture of foot bones except ankle	0.0264
Belarus	1995	Fracture of foot bones except ankle	0.0264
Belarus	2000	Fracture of foot bones except ankle	0.0264
Belarus	2005	Fracture of foot bones except ankle	0.0264
Belarus	2010	Fracture of foot bones except ankle	0.0264
Belarus	2017	Fracture of foot bones except ankle	0.0264
Belarus	1990	Fracture of hand (wrist and other distal part of hand)	0.0138
Belarus	1995	Fracture of hand (wrist and other distal part of hand)	0.0138
Belarus	2000	Fracture of hand (wrist and other distal part of hand)	0.0138
Belarus	2005	Fracture of hand (wrist and other distal part of hand)	0.0138
Belarus	2010	Fracture of hand (wrist and other distal part of hand)	0.0138
Belarus	2017	Fracture of hand (wrist and other distal part of hand)	0.0138
Belarus	1990	Fracture of hip	0.1038
Belarus	1995	Fracture of hip	0.1038
Belarus	2000	Fracture of hip	0.098
Belarus	2005	Fracture of hip	0.0853
Belarus	2010	Fracture of hip	0.0654
Belarus	2017	Fracture of hip	0.0577
Belarus	1990	Amputation of upper limbs, bilateral	0.1352
Belarus	1995	Amputation of upper limbs, bilateral	0.1353
Belarus	2000	Amputation of upper limbs, bilateral	0.1293
Belarus	2005	Amputation of upper limbs, bilateral	0.1161
Belarus	2010	Amputation of upper limbs, bilateral	0.0957
Belarus	2017	Amputation of upper limbs, bilateral	0.0876
Belarus	1990	Fracture of patella, tibia or fibula, or ankle	0.0554
Belarus	1995	Fracture of patella, tibia or fibula, or ankle	0.0554
Belarus	2000	Fracture of patella, tibia or fibula, or ankle	0.0554
Belarus	2005	Fracture of patella, tibia or fibula, or ankle	0.0554
Belarus	2010	Fracture of patella, tibia or fibula, or ankle	0.0554
Belarus	2017	Fracture of patella, tibia or fibula, or ankle	0.0554
Belarus	1990	Fracture of pelvis	0.1823
Belarus	1995	Fracture of pelvis	0.1823
Belarus	2000	Fracture of pelvis	0.1823
Belarus	2005	Fracture of pelvis	0.1823
Belarus	2010	Fracture of pelvis	0.1823
Belarus	2017	Fracture of pelvis	0.1823
Belarus	1990	Fracture of radius and/or ulna	0.0435
Belarus	1995	Fracture of radius and/or ulna	0.0435
Belarus	2000	Fracture of radius and/or ulna	0.0435
Belarus	2005	Fracture of radius and/or ulna	0.0435
Belarus	2010	Fracture of radius and/or ulna	0.0435
Belarus	2017	Fracture of radius and/or ulna	0.0435
Belarus	1990	Fracture of skull	0.0714
Belarus	1995	Fracture of skull	0.0714
Belarus	2000	Fracture of skull	0.0714
Belarus	2005	Fracture of skull	0.0714
Belarus	2010	Fracture of skull	0.0714
Belarus	2017	Fracture of skull	0.0714
Belarus	1990	Fracture of sternum and/or fracture of one or more ribs	0.1027
Belarus	1995	Fracture of sternum and/or fracture of one or more ribs	0.1027
Belarus	2000	Fracture of sternum and/or fracture of one or more ribs	0.1027
Belarus	2005	Fracture of sternum and/or fracture of one or more ribs	0.1027
Belarus	2010	Fracture of sternum and/or fracture of one or more ribs	0.1027
Belarus	2017	Fracture of sternum and/or fracture of one or more ribs	0.1027
Belarus	1990	Fracture of vertebral column	0.1106
Belarus	1995	Fracture of vertebral column	0.1106
Belarus	2000	Fracture of vertebral column	0.1106
Belarus	2005	Fracture of vertebral column	0.1106
Belarus	2010	Fracture of vertebral column	0.1106
Belarus	2017	Fracture of vertebral column	0.1106
Belarus	1990	Fracture of femur, other than femoral neck	0.042
Belarus	1995	Fracture of femur, other than femoral neck	0.042
Belarus	2000	Fracture of femur, other than femoral neck	0.042

Belarus	2005	Fracture of femur, other than femoral neck	0.042
Belarus	2010	Fracture of femur, other than femoral neck	0.042
Belarus	2017	Fracture of femur, other than femoral neck	0.042
Belarus	1990	Minor TBI	0.1319
Belarus	1995	Minor TBI	0.1319
Belarus	2000	Minor TBI	0.1319
Belarus	2005	Minor TBI	0.1319
Belarus	2010	Minor TBI	0.1319
Belarus	2017	Minor TBI	0.1319
Belarus	1990	Moderate TBI	0.1637
Belarus	1995	Moderate TBI	0.1637
Belarus	2000	Moderate TBI	0.1637
Belarus	2005	Moderate TBI	0.1637
Belarus	2010	Moderate TBI	0.1637
Belarus	2017	Moderate TBI	0.1637
Belarus	1990	Amputation of fingers (excluding thumb)	0.0047
Belarus	1995	Amputation of fingers (excluding thumb)	0.0047
Belarus	2000	Amputation of fingers (excluding thumb)	0.0047
Belarus	2005	Amputation of fingers (excluding thumb)	0.0047
Belarus	2010	Amputation of fingers (excluding thumb)	0.0047
Belarus	2017	Amputation of fingers (excluding thumb)	0.0047
Belarus	1990	Spinal cord lesion at neck level	0.4278
Belarus	1995	Spinal cord lesion at neck level	0.4278
Belarus	2000	Spinal cord lesion at neck level	0.4258
Belarus	2005	Spinal cord lesion at neck level	0.4214
Belarus	2010	Spinal cord lesion at neck level	0.4145
Belarus	2017	Spinal cord lesion at neck level	0.4118
Belarus	1990	Spinal cord lesion below neck level	0.2113
Belarus	1995	Spinal cord lesion below neck level	0.2113
Belarus	2000	Spinal cord lesion below neck level	0.2071
Belarus	2005	Spinal cord lesion below neck level	0.1978
Belarus	2010	Spinal cord lesion below neck level	0.1833
Belarus	2017	Spinal cord lesion below neck level	0.1777
Belarus	1990	Drowning and nonfatal submersion	0.2471
Belarus	1995	Drowning and nonfatal submersion	0.2471
Belarus	2000	Drowning and nonfatal submersion	0.2471
Belarus	2005	Drowning and nonfatal submersion	0.2471
Belarus	2010	Drowning and nonfatal submersion	0.2471
Belarus	2017	Drowning and nonfatal submersion	0.2471
Belarus	1990	Asphyxiation	0.2471
Belarus	1995	Asphyxiation	0.2471
Belarus	2000	Asphyxiation	0.2471
Belarus	2005	Asphyxiation	0.2471
Belarus	2010	Asphyxiation	0.2471
Belarus	2017	Asphyxiation	0.2471
Belarus	1990	Crush injury	0.1325
Belarus	1995	Crush injury	0.1325
Belarus	2000	Crush injury	0.1325
Belarus	2005	Crush injury	0.1325
Belarus	2010	Crush injury	0.1325
Belarus	2017	Crush injury	0.1325
Belarus	1990	Nerve injury	0.1129
Belarus	1995	Nerve injury	0.1129
Belarus	2000	Nerve injury	0.1129
Belarus	2005	Nerve injury	0.1129
Belarus	2010	Nerve injury	0.1129
Belarus	2017	Nerve injury	0.1129
Belarus	1990	Injury to eyes	0.0165
Belarus	1995	Injury to eyes	0.0165
Belarus	2000	Injury to eyes	0.0165
Belarus	2005	Injury to eyes	0.0165
Belarus	2010	Injury to eyes	0.0165
Belarus	2017	Injury to eyes	0.0165
Belarus	1990	Amputation of lower limb, unilateral	0.0569
Belarus	1995	Amputation of lower limb, unilateral	0.0569
Belarus	2000	Amputation of lower limb, unilateral	0.0547
Belarus	2005	Amputation of lower limb, unilateral	0.0497
Belarus	2010	Amputation of lower limb, unilateral	0.042
Belarus	2017	Amputation of lower limb, unilateral	0.039
Belarus	1990	Open wound(s)	0.011
Belarus	1995	Open wound(s)	0.011
Belarus	2000	Open wound(s)	0.011
Belarus	2005	Open wound(s)	0.011
Belarus	2010	Open wound(s)	0.011
Belarus	2017	Open wound(s)	0.011
Belarus	1990	Poisoning requiring urgent care	0.1628
Belarus	1995	Poisoning requiring urgent care	0.1628
Belarus	2000	Poisoning requiring urgent care	0.1628
Belarus	2005	Poisoning requiring urgent care	0.1628
Belarus	2010	Poisoning requiring urgent care	0.1628
Belarus	2017	Poisoning requiring urgent care	0.1628
Belarus	1990	Severe chest injury	0.0474
Belarus	1995	Severe chest injury	0.0474
Belarus	2000	Severe chest injury	0.0474
Belarus	2005	Severe chest injury	0.0474
Belarus	2010	Severe chest injury	0.0474
Belarus	2017	Severe chest injury	0.0474
Belarus	1990	Internal hemorrhage in abdomen and pelvis	0.1143
Belarus	1995	Internal hemorrhage in abdomen and pelvis	0.1143
Belarus	2000	Internal hemorrhage in abdomen and pelvis	0.1143
Belarus	2005	Internal hemorrhage in abdomen and pelvis	0.1143
Belarus	2010	Internal hemorrhage in abdomen and pelvis	0.1143
Belarus	2017	Internal hemorrhage in abdomen and pelvis	0.1143
Belarus	1990	Contusion in any part of the body	0.0075
Belarus	1995	Contusion in any part of the body	0.0075
Belarus	2000	Contusion in any part of the body	0.0075
Belarus	2005	Contusion in any part of the body	0.0075
Belarus	2010	Contusion in any part of the body	0.0075
Belarus	2017	Contusion in any part of the body	0.0075
Belarus	1990	Effect of different environmental factors	0.049
Belarus	1995	Effect of different environmental factors	0.049
Belarus	2000	Effect of different environmental factors	0.049
Belarus	2005	Effect of different environmental factors	0.049
Belarus	2010	Effect of different environmental factors	0.049
Belarus	2017	Effect of different environmental factors	0.049
Belarus	1990	Complications following therapeutic procedures	0.0506
Belarus	1995	Complications following therapeutic procedures	0.0506
Belarus	2000	Complications following therapeutic procedures	0.0506
Belarus	2005	Complications following therapeutic procedures	0.0506
Belarus	2010	Complications following therapeutic procedures	0.0506
Belarus	2017	Complications following therapeutic procedures	0.0506
Belarus	1990	Multiple fractures, dislocations, crashes, wounds, sprains, and strains	0.1038
Belarus	1995	Multiple fractures, dislocations, crashes, wounds, sprains, and strains	0.1038
Belarus	2000	Multiple fractures, dislocations, crashes, wounds, sprains, and strains	0.098
Belarus	2005	Multiple fractures, dislocations, crashes, wounds, sprains, and strains	0.0853
Belarus	2010	Multiple fractures, dislocations, crashes, wounds, sprains, and strains	0.0654
Belarus	2017	Multiple fractures, dislocations, crashes, wounds, sprains, and strains	0.0577
Belarus	1990	Amputation of upper limb, unilateral	0.0496
Belarus	1995	Amputation of upper limb, unilateral	0.0496
Belarus	2000	Amputation of upper limb, unilateral	0.0482
Belarus	2005	Amputation of upper limb, unilateral	0.0453
Belarus	2010	Amputation of upper limb, unilateral	0.0408
Belarus	2017	Amputation of upper limb, unilateral	0.039
Belarus	1990	Amputation of thumb	0.0114
Belarus	1995	Amputation of thumb	0.0114
Belarus	2000	Amputation of thumb	0.0114
Belarus	2005	Amputation of thumb	0.0114
Belarus	2010	Amputation of thumb	0.0114
Belarus	2017	Amputation of thumb	0.0114
Belarus	1990	Amputation of toe/toes	0.0059
Belarus	1995	Amputation of toe/toes	0.0059
Belarus	2000	Amputation of toe/toes	0.0059
Belarus	2005	Amputation of toe/toes	0.0059
Belarus	2010	Amputation of toe/toes	0.0059
Belarus	2017	Amputation of toe/toes	0.0059
Belarus	1990	Burns, <20% total burned surface area without lower airway burns	0.0163
Belarus	1995	Burns, <20% total burned surface area without lower airway burns	0.0163
Belarus	2000	Burns, <20% total burned surface area without lower airway burns	0.0163
Belarus	2005	Burns, <20% total burned surface area without lower airway burns	0.0163
Belarus	2010	Burns, <20% total burned surface area without lower airway burns	0.0163
Belarus	2017	Burns, <20% total burned surface area without lower airway burns	0.0163
Belarus	1990	Burns, >=20% total burned surface area or >= 10% burned surface area if head/neck or hands/wrist involved w/o lower airway burns	0.1775
Belarus	1995	Burns, >=20% total burned surface area or >= 10% burned surface area if head/neck or hands/wrist involved w/o lower airway burns	0.1775
Belarus	2000	Burns, >=20% total burned surface area or >= 10% burned surface area if head/neck or hands/wrist involved w/o lower airway burns	0.1722
Belarus	2005	Burns, >=20% total burned surface area or >= 10% burned surface area if head/neck or hands/wrist involved w/o lower airway burns	0.1603
Belarus	2010	Burns, >=20% total burned surface area or >= 10% burned surface area if head/neck or hands/wrist involved w/o lower airway burns	0.1418
Belarus	2017	Burns, >=20% total burned surface area or >= 10% burned surface area if head/neck or hands/wrist involved w/o lower airway burns	0.1346
Estonia	1990	Amputation of lower limbs, bilateral	0.147
Estonia	1995	Amputation of lower limbs, bilateral	0.15
Estonia	2000	Amputation of lower limbs, bilateral	0.1353
Estonia	2005	Amputation of lower limbs, bilateral	0.1232
Estonia	2010	Amputation of lower limbs, bilateral	0.1232
Estonia	2017	Amputation of lower limbs, bilateral	0.1232
Estonia	1990	Lower airway burns	0.2252
Estonia	1995	Lower airway burns	0.2252
Estonia	2000	Lower airway burns	0.2252
Estonia	2005	Lower airway burns	0.2252
Estonia	2010	Lower airway burns	0.2252
Estonia	2017	Lower airway burns	0.2252
Estonia	1990	Dislocation of hip	0.0159
Estonia	1995	Dislocation of hip	0.0159
Estonia	2000	Dislocation of hip	0.0159
Estonia	2005	Dislocation of hip	0.0159
Estonia	2010	Dislocation of hip	0.0159
Estonia	2017	Dislocation of hip	0.0159
Estonia	1990	Dislocation of knee	0.1134
Estonia	1995	Dislocation of knee	0.1134
Estonia	2000	Dislocation of knee	0.1134
Estonia	2005	Dislocation of knee	0.1134
Estonia	2010	Dislocation of knee	0.1134
Estonia	2017	Dislocation of knee	0.1134
Estonia	1990	Dislocation of shoulder	0.062
Estonia	1995	Dislocation of shoulder	0.062
Estonia	2000	Dislocation of shoulder	0.062
Estonia	2005	Dislocation of shoulder	0.062
Estonia	2010	Dislocation of shoulder	0.062
Estonia	2017	Dislocation of shoulder	0.062
Estonia	1990	Muscle and tendon injuries, including sprains and strains lesser dislocations	0.0075
Estonia	1995	Muscle and tendon injuries, including sprains and strains lesser dislocations	0.0075
Estonia	2000	Muscle and tendon injuries, including sprains and strains lesser dislocations	0.0075
Estonia	2005	Muscle and tendon injuries, including sprains and strains lesser dislocations	0.0075
Estonia	2010	Muscle and tendon injuries, including sprains and strains lesser dislocations	0.0075
Estonia	2017	Muscle and tendon injuries, including sprains and strains lesser dislocations	0.0075
Estonia	1990	Fracture of clavicle, scapula, or humerus	0.0349
Estonia	1995	Fracture of clavicle, scapula, or humerus	0.0349
Estonia	2000	Fracture of clavicle, scapula, or humerus	0.0349
Estonia	2005	Fracture of clavicle, scapula, or humerus	0.0349
Estonia	2010	Fracture of clavicle, scapula, or humerus	0.0349
Estonia	2017	Fracture of clavicle, scapula, or humerus	0.0349
Estonia	1990	Fracture of face bones	0.0669
Estonia	1995	Fracture of face bones	0.0669
Estonia	2000	Fracture of face bones	0.0669
Estonia	2005	Fracture of face bones	0.0669
Estonia	2010	Fracture of face bones	0.0669
Estonia	2017	Fracture of face bones	0.0669
Estonia	1990	Fracture of foot bones except ankle	0.0264
Estonia	1995	Fracture of foot bones except ankle	0.0264
Estonia	2000	Fracture of foot bones except ankle	0.0264
Estonia	2005	Fracture of foot bones except ankle	0.0264
Estonia	2010	Fracture of foot bones except ankle	0.0264
Estonia	2017	Fracture of foot bones except ankle	0.0264
Estonia	1990	Fracture of hand (wrist and other distal part of hand)	0.0138
Estonia	1995	Fracture of hand (wrist and other distal part of hand)	0.0138
Estonia	2000	Fracture of hand (wrist and other distal part of hand)	0.0138
Estonia	2005	Fracture of hand (wrist and other distal part of hand)	0.0138
Estonia	2010	Fracture of hand (wrist and other distal part of hand)	0.0138
Estonia	2017	Fracture of hand (wrist and other distal part of hand)	0.0138
Estonia	1990	Fracture of hip	0.089
Estonia	1995	Fracture of hip	0.093
Estonia	2000	Fracture of hip	0.0735
Estonia	2005	Fracture of hip	0.0577
Estonia	2010	Fracture of hip	0.0577
Estonia	2017	Fracture of hip	0.0577
Estonia	1990	Amputation of upper limbs, bilateral	0.12
Estonia	1995	Amputation of upper limbs, bilateral	0.1241
Estonia	2000	Amputation of upper limbs, bilateral	0.104
Estonia	2005	Amputation of upper limbs, bilateral	0.0876
Estonia	2010	Amputation of upper limbs, bilateral	0.0876
Estonia	2017	Amputation of upper limbs, bilateral	0.0876
Estonia	1990	Fracture of patella, tibia or fibula, or ankle	0.0554
Estonia	1995	Fracture of patella, tibia or fibula, or ankle	0.0554
Estonia	2000	Fracture of patella, tibia or fibula, or ankle	0.0554
Estonia	2005	Fracture of patella, tibia or fibula, or ankle	0.0554
Estonia	2010	Fracture of patella, tibia or fibula, or ankle	0.0554
Estonia	2017	Fracture of patella, tibia or fibula, or ankle	0.0554
Estonia	1990	Fracture of pelvis	0.1823
Estonia	1995	Fracture of pelvis	0.1823
Estonia	2000	Fracture of pelvis	0.1823
Estonia	2005	Fracture of pelvis	0.1823
Estonia	2010	Fracture of pelvis	0.1823
Estonia	2017	Fracture of pelvis	0.1823
Estonia	1990	Fracture of radius and/or ulna	0.0435
Estonia	1995	Fracture of radius and/or ulna	0.0435
Estonia	2000	Fracture of radius and/or ulna	0.0435
Estonia	2005	Fracture of radius and/or ulna	0.0435
Estonia	2010	Fracture of radius and/or ulna	0.0435
Estonia	2017	Fracture of radius and/or ulna	0.0435
Estonia	1990	Fracture of skull	0.0714
Estonia	1995	Fracture of skull	0.0714
Estonia	2000	Fracture of skull	0.0714
Estonia	2005	Fracture of skull	0.0714
Estonia	2010	Fracture of skull	0.0714
Estonia	2017	Fracture of skull	0.0714
Estonia	1990	Fracture of sternum and/or fracture of one or more ribs	0.1027
Estonia	1995	Fracture of sternum and/or fracture of one or more ribs	0.1027
Estonia	2000	Fracture of sternum and/or fracture of one or more ribs	0.1027
Estonia	2005	Fracture of sternum and/or fracture of one or more ribs	0.1027
Estonia	2010	Fracture of sternum and/or fracture of one or more ribs	0.1027
Estonia	2017	Fracture of sternum and/or fracture of one or more ribs	0.1027
Estonia	1990	Fracture of vertebral column	0.1106
Estonia	1995	Fracture of vertebral column	0.1106
Estonia	2000	Fracture of vertebral column	0.1106
Estonia	2005	Fracture of vertebral column	0.1106
Estonia	2010	Fracture of vertebral column	0.1106
Estonia	2017	Fracture of vertebral column	0.1106
Estonia	1990	Fracture of femur, other than femoral neck	0.042
Estonia	1995	Fracture of femur, other than femoral neck	0.042
Estonia	2000	Fracture of femur, other than femoral neck	0.042
Estonia	2005	Fracture of femur, other than femoral neck	0.042
Estonia	2010	Fracture of femur, other than femoral neck	0.042
Estonia	2017	Fracture of femur, other than femoral neck	0.042
Estonia	1990	Minor TBI	0.1319
Estonia	1995	Minor TBI	0.1319
Estonia	2000	Minor TBI	0.1319
Estonia	2005	Minor TBI	0.1319
Estonia	2010	Minor TBI	0.1319
Estonia	2017	Minor TBI	0.1319
Estonia	1990	Moderate TBI	0.1637
Estonia	1995	Moderate TBI	0.1637
Estonia	2000	Moderate TBI	0.1637
Estonia	2005	Moderate TBI	0.1637
Estonia	2010	Moderate TBI	0.1637
Estonia	2017	Moderate TBI	0.1637
Estonia	1990	Amputation of fingers (excluding thumb)	0.0047
Estonia	1995	Amputation of fingers (excluding thumb)	0.0047
Estonia	2000	Amputation of fingers (excluding thumb)	0.0047
Estonia	2005	Amputation of fingers (excluding thumb)	0.0047
Estonia	2010	Amputation of fingers (excluding thumb)	0.0047
Estonia	2017	Amputation of fingers (excluding thumb)	0.0047
Estonia	1990	Spinal cord lesion at neck level	0.4227
Estonia	1995	Spinal cord lesion at neck level	0.424
Estonia	2000	Spinal cord lesion at neck level	0.4173
Estonia	2005	Spinal cord lesion at neck level	0.4118
Estonia	2010	Spinal cord lesion at neck level	0.4118
Estonia	2017	Spinal cord lesion at neck level	0.4118
Estonia	1990	Spinal cord lesion below neck level	0.2005
Estonia	1995	Spinal cord lesion below neck level	0.2035
Estonia	2000	Spinal cord lesion below neck level	0.1893
Estonia	2005	Spinal cord lesion below neck level	0.1777
Estonia	2010	Spinal cord lesion below neck level	0.1777
Estonia	2017	Spinal cord lesion below neck level	0.1777
Estonia	1990	Drowning and nonfatal submersion	0.2471
Estonia	1995	Drowning and nonfatal submersion	0.2471
Estonia	2000	Drowning and nonfatal submersion	0.2471
Estonia	2005	Drowning and nonfatal submersion	0.2471
Estonia	2010	Drowning and nonfatal submersion	0.2471
Estonia	2017	Drowning and nonfatal submersion	0.2471
Estonia	1990	Asphyxiation	0.2471
Estonia	1995	Asphyxiation	0.2471
Estonia	2000	Asphyxiation	0.2471
Estonia	2005	Asphyxiation	0.2471
Estonia	2010	Asphyxiation	0.2471
Estonia	2017	Asphyxiation	0.2471
Estonia	1990	Crush injury	0.1325
Estonia	1995	Crush injury	0.1325
Estonia	2000	Crush injury	0.1325
Estonia	2005	Crush injury	0.1325
Estonia	2010	Crush injury	0.1325
Estonia	2017	Crush injury	0.1325
Estonia	1990	Nerve injury	0.1129
Estonia	1995	Nerve injury	0.1129
Estonia	2000	Nerve injury	0.1129
Estonia	2005	Nerve injury	0.1129
Estonia	2010	Nerve injury	0.1129
Estonia	2017	Nerve injury	0.1129
Estonia	1990	Injury to eyes	0.0165
Estonia	1995	Injury to eyes	0.0165
Estonia	2000	Injury to eyes	0.0165
Estonia	2005	Injury to eyes	0.0165
Estonia	2010	Injury to eyes	0.0165
Estonia	2017	Injury to eyes	0.0165
Estonia	1990	Amputation of lower limb, unilateral	0.0512
Estonia	1995	Amputation of lower limb, unilateral	0.0527
Estonia	2000	Amputation of lower limb, unilateral	0.0451
Estonia	2005	Amputation of lower limb, unilateral	0.039
Estonia	2010	Amputation of lower limb, unilateral	0.039
Estonia	2017	Amputation of lower limb, unilateral	0.039
Estonia	1990	Open wound(s)	0.011
Estonia	1995	Open wound(s)	0.011
Estonia	2000	Open wound(s)	0.011
Estonia	2005	Open wound(s)	0.011
Estonia	2010	Open wound(s)	0.011
Estonia	2017	Open wound(s)	0.011
Estonia	1990	Poisoning requiring urgent care	0.1628
Estonia	1995	Poisoning requiring urgent care	0.1628
Estonia	2000	Poisoning requiring urgent care	0.1628
Estonia	2005	Poisoning requiring urgent care	0.1628
Estonia	2010	Poisoning requiring urgent care	0.1628
Estonia	2017	Poisoning requiring urgent care	0.1628
Estonia	1990	Severe chest injury	0.0474
Estonia	1995	Severe chest injury	0.0474
Estonia	2000	Severe chest injury	0.0474
Estonia	2005	Severe chest injury	0.0474
Estonia	2010	Severe chest injury	0.0474
Estonia	2017	Severe chest injury	0.0474
Estonia	1990	Internal hemorrhage in abdomen and pelvis	0.1143
Estonia	1995	Internal hemorrhage in abdomen and pelvis	0.1143
Estonia	2000	Internal hemorrhage in abdomen and pelvis	0.1143
Estonia	2005	Internal hemorrhage in abdomen and pelvis	0.1143
Estonia	2010	Internal hemorrhage in abdomen and pelvis	0.1143
Estonia	2017	Internal hemorrhage in abdomen and pelvis	0.1143
Estonia	1990	Contusion in any part of the body	0.0075
Estonia	1995	Contusion in any part of the body	0.0075
Estonia	2000	Contusion in any part of the body	0.0075
Estonia	2005	Contusion in any part of the body	0.0075
Estonia	2010	Contusion in any part of the body	0.0075
Estonia	2017	Contusion in any part of the body	0.0075
Estonia	1990	Effect of different environmental factors	0.049
Estonia	1995	Effect of different environmental factors	0.049
Estonia	2000	Effect of different environmental factors	0.049
Estonia	2005	Effect of different environmental factors	0.049
Estonia	2010	Effect of different environmental factors	0.049
Estonia	2017	Effect of different environmental factors	0.049
Estonia	1990	Complications following therapeutic procedures	0.0506
Estonia	1995	Complications following therapeutic procedures	0.0506
Estonia	2000	Complications following therapeutic procedures	0.0506
Estonia	2005	Complications following therapeutic procedures	0.0506
Estonia	2010	Complications following therapeutic procedures	0.0506
Estonia	2017	Complications following therapeutic procedures	0.0506
Estonia	1990	Multiple fractures, dislocations, crashes, wounds, sprains, and strains	0.089
Estonia	1995	Multiple fractures, dislocations, crashes, wounds, sprains, and strains	0.093
Estonia	2000	Multiple fractures, dislocations, crashes, wounds, sprains, and strains	0.0735
Estonia	2005	Multiple fractures, dislocations, crashes, wounds, sprains, and strains	0.0577
Estonia	2010	Multiple fractures, dislocations, crashes, wounds, sprains, and strains	0.0577
Estonia	2017	Multiple fractures, dislocations, crashes, wounds, sprains, and strains	0.0577
Estonia	1990	Amputation of upper limb, unilateral	0.0462
Estonia	1995	Amputation of upper limb, unilateral	0.0471
Estonia	2000	Amputation of upper limb, unilateral	0.0426
Estonia	2005	Amputation of upper limb, unilateral	0.039
Estonia	2010	Amputation of upper limb, unilateral	0.039
Estonia	2017	Amputation of upper limb, unilateral	0.039
Estonia	1990	Amputation of thumb	0.0114
Estonia	1995	Amputation of thumb	0.0114
Estonia	2000	Amputation of thumb	0.0114
Estonia	2005	Amputation of thumb	0.0114
Estonia	2010	Amputation of thumb	0.0114
Estonia	2017	Amputation of thumb	0.0114
Estonia	1990	Amputation of toe/toes	0.0059
Estonia	1995	Amputation of toe/toes	0.0059
Estonia	2000	Amputation of toe/toes	0.0059
Estonia	2005	Amputation of toe/toes	0.0059
Estonia	2010	Amputation of toe/toes	0.0059
Estonia	2017	Amputation of toe/toes	0.0059
Estonia	1990	Burns, <20% total burned surface area without lower airway burns	0.0163
Estonia	1995	Burns, <20% total burned surface area without lower airway burns	0.0163
Estonia	2000	Burns, <20% total burned surface area without lower airway burns	0.0163
Estonia	2005	Burns, <20% total burned surface area without lower airway burns	0.0163
Estonia	2010	Burns, <20% total burned surface area without lower airway burns	0.0163
Estonia	2017	Burns, <20% total burned surface area without lower airway burns	0.0163
Estonia	1990	Burns, >=20% total burned surface area or >= 10% burned surface area if head/neck or hands/wrist involved w/o lower airway burns	0.1638
Estonia	1995	Burns, >=20% total burned surface area or >= 10% burned surface area if head/neck or hands/wrist involved w/o lower airway burns	0.1675
Estonia	2000	Burns, >=20% total burned surface area or >= 10% burned surface area if head/neck or hands/wrist involved w/o lower airway burns	0.1494
Estonia	2005	Burns, >=20% total burned surface area or >= 10% burned surface area if head/neck or hands/wrist involved w/o lower airway burns	0.1346
Estonia	2010	Burns, >=20% total burned surface area or >= 10% burned surface area if head/neck or hands/wrist involved w/o lower airway burns	0.1346
Estonia	2017	Burns, >=20% total burned surface area or >= 10% burned surface area if head/neck or hands/wrist involved w/o lower airway burns	0.1346
Latvia	1990	Amputation of lower limbs, bilateral	0.1506
Latvia	1995	Amputation of lower limbs, bilateral	0.157
Latvia	2000	Amputation of lower limbs, bilateral	0.1431
Latvia	2005	Amputation of lower limbs, bilateral	0.1295
Latvia	2010	Amputation of lower limbs, bilateral	0.1232
Latvia	2017	Amputation of lower limbs, bilateral	0.1232
Latvia	1990	Lower airway burns	0.2252
Latvia	1995	Lower airway burns	0.2252
Latvia	2000	Lower airway burns	0.2252
Latvia	2005	Lower airway burns	0.2252
Latvia	2010	Lower airway burns	0.2252
Latvia	2017	Lower airway burns	0.2252
Latvia	1990	Dislocation of hip	0.0159
Latvia	1995	Dislocation of hip	0.0159
Latvia	2000	Dislocation of hip	0.0159
Latvia	2005	Dislocation of hip	0.0159
Latvia	2010	Dislocation of hip	0.0159
Latvia	2017	Dislocation of hip	0.0159
Latvia	1990	Dislocation of knee	0.1134
Latvia	1995	Dislocation of knee	0.1134
Latvia	2000	Dislocation of knee	0.1134
Latvia	2005	Dislocation of knee	0.1134
Latvia	2010	Dislocation of knee	0.1134
Latvia	2017	Dislocation of knee	0.1134
Latvia	1990	Dislocation of shoulder	0.062
Latvia	1995	Dislocation of shoulder	0.062

Latvia	2000	Dislocation of shoulder	0.062
Latvia	2005	Dislocation of shoulder	0.062
Latvia	2010	Dislocation of shoulder	0.062
Latvia	2017	Dislocation of shoulder	0.062
Latvia	1990	Muscle and tendon injuries, including sprains and strains lesser dislocations	0.0075
Latvia	1995	Muscle and tendon injuries, including sprains and strains lesser dislocations	0.0075
Latvia	2000	Muscle and tendon injuries, including sprains and strains lesser dislocations	0.0075
Latvia	2005	Muscle and tendon injuries, including sprains and strains lesser dislocations	0.0075
Latvia	2010	Muscle and tendon injuries, including sprains and strains lesser dislocations	0.0075
Latvia	2017	Muscle and tendon injuries, including sprains and strains lesser dislocations	0.0075
Latvia	1990	Fracture of clavicle, scapula, or humerus	0.0349
Latvia	1995	Fracture of clavicle, scapula, or humerus	0.0349
Latvia	2000	Fracture of clavicle, scapula, or humerus	0.0349
Latvia	2005	Fracture of clavicle, scapula, or humerus	0.0349
Latvia	2010	Fracture of clavicle, scapula, or humerus	0.0349
Latvia	2017	Fracture of clavicle, scapula, or humerus	0.0349
Latvia	1990	Fracture of face bones	0.0669
Latvia	1995	Fracture of face bones	0.0669
Latvia	2000	Fracture of face bones	0.0669
Latvia	2005	Fracture of face bones	0.0669
Latvia	2010	Fracture of face bones	0.0669
Latvia	2017	Fracture of face bones	0.0669
Latvia	1990	Fracture of foot bones except ankle	0.0264
Latvia	1995	Fracture of foot bones except ankle	0.0264
Latvia	2000	Fracture of foot bones except ankle	0.0264
Latvia	2005	Fracture of foot bones except ankle	0.0264
Latvia	2010	Fracture of foot bones except ankle	0.0264
Latvia	2017	Fracture of foot bones except ankle	0.0264
Latvia	1990	Fracture of hand (wrist and other distal part of hand)	0.0138
Latvia	1995	Fracture of hand (wrist and other distal part of hand)	0.0138
Latvia	2000	Fracture of hand (wrist and other distal part of hand)	0.0138
Latvia	2005	Fracture of hand (wrist and other distal part of hand)	0.0138
Latvia	2010	Fracture of hand (wrist and other distal part of hand)	0.0138
Latvia	2017	Fracture of hand (wrist and other distal part of hand)	0.0138
Latvia	1990	Fracture of hip	0.0938
Latvia	1995	Fracture of hip	0.1024
Latvia	2000	Fracture of hip	0.0839
Latvia	2005	Fracture of hip	0.0659
Latvia	2010	Fracture of hip	0.0577
Latvia	2017	Fracture of hip	0.0577
Latvia	1990	Amputation of upper limbs, bilateral	0.1249
Latvia	1995	Amputation of upper limbs, bilateral	0.1338
Latvia	2000	Amputation of upper limbs, bilateral	0.1147
Latvia	2005	Amputation of upper limbs, bilateral	0.0962
Latvia	2010	Amputation of upper limbs, bilateral	0.0876
Latvia	2017	Amputation of upper limbs, bilateral	0.0876
Latvia	1990	Fracture of patella, tibia or fibula, or ankle	0.0554
Latvia	1995	Fracture of patella, tibia or fibula, or ankle	0.0554
Latvia	2000	Fracture of patella, tibia or fibula, or ankle	0.0554
Latvia	2005	Fracture of patella, tibia or fibula, or ankle	0.0554
Latvia	2010	Fracture of patella, tibia or fibula, or ankle	0.0554
Latvia	2017	Fracture of patella, tibia or fibula, or ankle	0.0554
Latvia	1990	Fracture of pelvis	0.1823
Latvia	1995	Fracture of pelvis	0.1823
Latvia	2000	Fracture of pelvis	0.1823
Latvia	2005	Fracture of pelvis	0.1823
Latvia	2010	Fracture of pelvis	0.1823
Latvia	2017	Fracture of pelvis	0.1823
Latvia	1990	Fracture of radius and/or ulna	0.0435
Latvia	1995	Fracture of radius and/or ulna	0.0435
Latvia	2000	Fracture of radius and/or ulna	0.0435
Latvia	2005	Fracture of radius and/or ulna	0.0435
Latvia	2010	Fracture of radius and/or ulna	0.0435
Latvia	2017	Fracture of radius and/or ulna	0.0435
Latvia	1990	Fracture of skull	0.0714
Latvia	1995	Fracture of skull	0.0714
Latvia	2000	Fracture of skull	0.0714
Latvia	2005	Fracture of skull	0.0714
Latvia	2010	Fracture of skull	0.0714
Latvia	2017	Fracture of skull	0.0714
Latvia	1990	Fracture of sternum and/or fracture of one or more ribs	0.1027
Latvia	1995	Fracture of sternum and/or fracture of one or more ribs	0.1027
Latvia	2000	Fracture of sternum and/or fracture of one or more ribs	0.1027
Latvia	2005	Fracture of sternum and/or fracture of one or more ribs	0.1027
Latvia	2010	Fracture of sternum and/or fracture of one or more ribs	0.1027
Latvia	2017	Fracture of sternum and/or fracture of one or more ribs	0.1027
Latvia	1990	Fracture of vertebral column	0.1106
Latvia	1995	Fracture of vertebral column	0.1106
Latvia	2000	Fracture of vertebral column	0.1106
Latvia	2005	Fracture of vertebral column	0.1106
Latvia	2010	Fracture of vertebral column	0.1106
Latvia	2017	Fracture of vertebral column	0.1106
Latvia	1990	Fracture of femur, other than femoral neck	0.042
Latvia	1995	Fracture of femur, other than femoral neck	0.042
Latvia	2000	Fracture of femur, other than femoral neck	0.042
Latvia	2005	Fracture of femur, other than femoral neck	0.042
Latvia	2010	Fracture of femur, other than femoral neck	0.042
Latvia	2017	Fracture of femur, other than femoral neck	0.042
Latvia	1990	Minor TBI	0.1319
Latvia	1995	Minor TBI	0.1319
Latvia	2000	Minor TBI	0.1319
Latvia	2005	Minor TBI	0.1319
Latvia	2010	Minor TBI	0.1319
Latvia	2017	Minor TBI	0.1319
Latvia	1990	Moderate TBI	0.1637
Latvia	1995	Moderate TBI	0.1637
Latvia	2000	Moderate TBI	0.1637
Latvia	2005	Moderate TBI	0.1637
Latvia	2010	Moderate TBI	0.1637
Latvia	2017	Moderate TBI	0.1637
Latvia	1990	Amputation of fingers (excluding thumb)	0.0047
Latvia	1995	Amputation of fingers (excluding thumb)	0.0047
Latvia	2000	Amputation of fingers (excluding thumb)	0.0047
Latvia	2005	Amputation of fingers (excluding thumb)	0.0047
Latvia	2010	Amputation of fingers (excluding thumb)	0.0047
Latvia	2017	Amputation of fingers (excluding thumb)	0.0047
Latvia	1990	Spinal cord lesion at neck level	0.4243
Latvia	1995	Spinal cord lesion at neck level	0.4273
Latvia	2000	Spinal cord lesion at neck level	0.4209
Latvia	2005	Spinal cord lesion at neck level	0.4147
Latvia	2010	Spinal cord lesion at neck level	0.4118
Latvia	2017	Spinal cord lesion at neck level	0.4118
Latvia	1990	Spinal cord lesion below neck level	0.204
Latvia	1995	Spinal cord lesion below neck level	0.2103
Latvia	2000	Spinal cord lesion below neck level	0.1968
Latvia	2005	Spinal cord lesion below neck level	0.1837
Latvia	2010	Spinal cord lesion below neck level	0.1777
Latvia	2017	Spinal cord lesion below neck level	0.1777
Latvia	1990	Drowning and nonfatal submersion	0.2471
Latvia	1995	Drowning and nonfatal submersion	0.2471
Latvia	2000	Drowning and nonfatal submersion	0.2471
Latvia	2005	Drowning and nonfatal submersion	0.2471
Latvia	2010	Drowning and nonfatal submersion	0.2471
Latvia	2017	Drowning and nonfatal submersion	0.2471
Latvia	1990	Asphyxiation	0.2471
Latvia	1995	Asphyxiation	0.2471
Latvia	2000	Asphyxiation	0.2471
Latvia	2005	Asphyxiation	0.2471
Latvia	2010	Asphyxiation	0.2471
Latvia	2017	Asphyxiation	0.2471
Latvia	1990	Crush injury	0.1325
Latvia	1995	Crush injury	0.1325
Latvia	2000	Crush injury	0.1325
Latvia	2005	Crush injury	0.1325
Latvia	2010	Crush injury	0.1325
Latvia	2017	Crush injury	0.1325
Latvia	1990	Nerve injury	0.1129
Latvia	1995	Nerve injury	0.1129
Latvia	2000	Nerve injury	0.1129
Latvia	2005	Nerve injury	0.1129
Latvia	2010	Nerve injury	0.1129
Latvia	2017	Nerve injury	0.1129
Latvia	1990	Injury to eyes	0.0165
Latvia	1995	Injury to eyes	0.0165
Latvia	2000	Injury to eyes	0.0165
Latvia	2005	Injury to eyes	0.0165
Latvia	2010	Injury to eyes	0.0165
Latvia	2017	Injury to eyes	0.0165
Latvia	1990	Amputation of lower limb, unilateral	0.053
Latvia	1995	Amputation of lower limb, unilateral	0.0564
Latvia	2000	Amputation of lower limb, unilateral	0.0492
Latvia	2005	Amputation of lower limb, unilateral	0.0422
Latvia	2010	Amputation of lower limb, unilateral	0.039
Latvia	2017	Amputation of lower limb, unilateral	0.039
Latvia	1990	Open wound(s)	0.011
Latvia	1995	Open wound(s)	0.011
Latvia	2000	Open wound(s)	0.011
Latvia	2005	Open wound(s)	0.011
Latvia	2010	Open wound(s)	0.011
Latvia	2017	Open wound(s)	0.011
Latvia	1990	Poisoning requiring urgent care	0.1628
Latvia	1995	Poisoning requiring urgent care	0.1628
Latvia	2000	Poisoning requiring urgent care	0.1628
Latvia	2005	Poisoning requiring urgent care	0.1628
Latvia	2010	Poisoning requiring urgent care	0.1628
Latvia	2017	Poisoning requiring urgent care	0.1628
Latvia	1990	Severe chest injury	0.0474
Latvia	1995	Severe chest injury	0.0474
Latvia	2000	Severe chest injury	0.0474
Latvia	2005	Severe chest injury	0.0474
Latvia	2010	Severe chest injury	0.0474
Latvia	2017	Severe chest injury	0.0474
Latvia	1990	Internal hemorrhage in abdomen and pelvis	0.1143
Latvia	1995	Internal hemorrhage in abdomen and pelvis	0.1143
Latvia	2000	Internal hemorrhage in abdomen and pelvis	0.1143
Latvia	2005	Internal hemorrhage in abdomen and pelvis	0.1143
Latvia	2010	Internal hemorrhage in abdomen and pelvis	0.1143
Latvia	2017	Internal hemorrhage in abdomen and pelvis	0.1143
Latvia	1990	Contusion in any part of the body	0.0075
Latvia	1995	Contusion in any part of the body	0.0075
Latvia	2000	Contusion in any part of the body	0.0075
Latvia	2005	Contusion in any part of the body	0.0075
Latvia	2010	Contusion in any part of the body	0.0075
Latvia	2017	Contusion in any part of the body	0.0075
Latvia	1990	Effect of different environmental factors	0.049
Latvia	1995	Effect of different environmental factors	0.049
Latvia	2000	Effect of different environmental factors	0.049
Latvia	2005	Effect of different environmental factors	0.049
Latvia	2010	Effect of different environmental factors	0.049
Latvia	2017	Effect of different environmental factors	0.049
Latvia	1990	Complications following therapeutic procedures	0.0506
Latvia	1995	Complications following therapeutic procedures	0.0506
Latvia	2000	Complications following therapeutic procedures	0.0506
Latvia	2005	Complications following therapeutic procedures	0.0506
Latvia	2010	Complications following therapeutic procedures	0.0506
Latvia	2017	Complications following therapeutic procedures	0.0506
Latvia	1990	Multiple fractures, dislocations, crashes, wounds, sprains, and strains	0.0938
Latvia	1995	Multiple fractures, dislocations, crashes, wounds, sprains, and strains	0.1024
Latvia	2000	Multiple fractures, dislocations, crashes, wounds, sprains, and strains	0.0839
Latvia	2005	Multiple fractures, dislocations, crashes, wounds, sprains, and strains	0.0659
Latvia	2010	Multiple fractures, dislocations, crashes, wounds, sprains, and strains	0.0577
Latvia	2017	Multiple fractures, dislocations, crashes, wounds, sprains, and strains	0.0577
Latvia	1990	Amputation of upper limb, unilateral	0.0473
Latvia	1995	Amputation of upper limb, unilateral	0.0492
Latvia	2000	Amputation of upper limb, unilateral	0.045
Latvia	2005	Amputation of upper limb, unilateral	0.0409
Latvia	2010	Amputation of upper limb, unilateral	0.039
Latvia	2017	Amputation of upper limb, unilateral	0.039
Latvia	1990	Amputation of thumb	0.0114
Latvia	1995	Amputation of thumb	0.0114
Latvia	2000	Amputation of thumb	0.0114
Latvia	2005	Amputation of thumb	0.0114
Latvia	2010	Amputation of thumb	0.0114
Latvia	2017	Amputation of thumb	0.0114
Latvia	1990	Amputation of toe/toes	0.0059
Latvia	1995	Amputation of toe/toes	0.0059
Latvia	2000	Amputation of toe/toes	0.0059
Latvia	2005	Amputation of toe/toes	0.0059
Latvia	2010	Amputation of toe/toes	0.0059
Latvia	2017	Amputation of toe/toes	0.0059
Latvia	1990	Burns, <20% total burned surface area without lower airway burns	0.0163
Latvia	1995	Burns, <20% total burned surface area without lower airway burns	0.0163
Latvia	2000	Burns, <20% total burned surface area without lower airway burns	0.0163
Latvia	2005	Burns, <20% total burned surface area without lower airway burns	0.0163
Latvia	2010	Burns, <20% total burned surface area without lower airway burns	0.0163
Latvia	2017	Burns, <20% total burned surface area without lower airway burns	0.0163
Latvia	1990	Burns, >=20% total burned surface area or >= 10% burned surface area if head/neck or hands/wrist involved w/o lower airway burns	0.1682
Latvia	1995	Burns, >=20% total burned surface area or >= 10% burned surface area if head/neck or hands/wrist involved w/o lower airway burns	0.1762
Latvia	2000	Burns, >=20% total burned surface area or >= 10% burned surface area if head/neck or hands/wrist involved w/o lower airway burns	0.159
Latvia	2005	Burns, >=20% total burned surface area or >= 10% burned surface area if head/neck or hands/wrist involved w/o lower airway burns	0.1423
Latvia	2010	Burns, >=20% total burned surface area or >= 10% burned surface area if head/neck or hands/wrist involved w/o lower airway burns	0.1346
Latvia	2017	Burns, >=20% total burned surface area or >= 10% burned surface area if head/neck or hands/wrist involved w/o lower airway burns	0.1346
Lithuania	1990	Amputation of lower limbs, bilateral	0.1434
Lithuania	1995	Amputation of lower limbs, bilateral	0.1471
Lithuania	2000	Amputation of lower limbs, bilateral	0.1342
Lithuania	2005	Amputation of lower limbs, bilateral	0.1275
Lithuania	2010	Amputation of lower limbs, bilateral	0.1232
Lithuania	2017	Amputation of lower limbs, bilateral	0.1232
Lithuania	1990	Lower airway burns	0.2252
Lithuania	1995	Lower airway burns	0.2252
Lithuania	2000	Lower airway burns	0.2252
Lithuania	2005	Lower airway burns	0.2252
Lithuania	2010	Lower airway burns	0.2252
Lithuania	2017	Lower airway burns	0.2252
Lithuania	1990	Dislocation of hip	0.0159
Lithuania	1995	Dislocation of hip	0.0159
Lithuania	2000	Dislocation of hip	0.0159
Lithuania	2005	Dislocation of hip	0.0159
Lithuania	2010	Dislocation of hip	0.0159
Lithuania	2017	Dislocation of hip	0.0159
Lithuania	1990	Dislocation of knee	0.1134
Lithuania	1995	Dislocation of knee	0.1134
Lithuania	2000	Dislocation of knee	0.1134
Lithuania	2005	Dislocation of knee	0.1134
Lithuania	2010	Dislocation of knee	0.1134
Lithuania	2017	Dislocation of knee	0.1134
Lithuania	1990	Dislocation of shoulder	0.062
Lithuania	1995	Dislocation of shoulder	0.062
Lithuania	2000	Dislocation of shoulder	0.062
Lithuania	2005	Dislocation of shoulder	0.062
Lithuania	2010	Dislocation of shoulder	0.062
Lithuania	2017	Dislocation of shoulder	0.062
Lithuania	1990	Muscle and tendon injuries, including sprains and strains lesser dislocations	0.0075
Lithuania	1995	Muscle and tendon injuries, including sprains and strains lesser dislocations	0.0075
Lithuania	2000	Muscle and tendon injuries, including sprains and strains lesser dislocations	0.0075
Lithuania	2005	Muscle and tendon injuries, including sprains and strains lesser dislocations	0.0075
Lithuania	2010	Muscle and tendon injuries, including sprains and strains lesser dislocations	0.0075
Lithuania	2017	Muscle and tendon injuries, including sprains and strains lesser dislocations	0.0075
Lithuania	1990	Fracture of clavicle, scapula, or humerus	0.0349
Lithuania	1995	Fracture of clavicle, scapula, or humerus	0.0349
Lithuania	2000	Fracture of clavicle, scapula, or humerus	0.0349
Lithuania	2005	Fracture of clavicle, scapula, or humerus	0.0349
Lithuania	2010	Fracture of clavicle, scapula, or humerus	0.0349
Lithuania	2017	Fracture of clavicle, scapula, or humerus	0.0349
Lithuania	1990	Fracture of face bones	0.0669
Lithuania	1995	Fracture of face bones	0.0669
Lithuania	2000	Fracture of face bones	0.0669
Lithuania	2005	Fracture of face bones	0.0669
Lithuania	2010	Fracture of face bones	0.0669
Lithuania	2017	Fracture of face bones	0.0669
Lithuania	1990	Fracture of foot bones except ankle	0.0264
Lithuania	1995	Fracture of foot bones except ankle	0.0264
Lithuania	2000	Fracture of foot bones except ankle	0.0264
Lithuania	2005	Fracture of foot bones except ankle	0.0264
Lithuania	2010	Fracture of foot bones except ankle	0.0264
Lithuania	2017	Fracture of foot bones except ankle	0.0264
Lithuania	1990	Fracture of hand (wrist and other distal part of hand)	0.0138
Lithuania	1995	Fracture of hand (wrist and other distal part of hand)	0.0138
Lithuania	2000	Fracture of hand (wrist and other distal part of hand)	0.0138
Lithuania	2005	Fracture of hand (wrist and other distal part of hand)	0.0138
Lithuania	2010	Fracture of hand (wrist and other distal part of hand)	0.0138
Lithuania	2017	Fracture of hand (wrist and other distal part of hand)	0.0138
Lithuania	1990	Fracture of hip	0.0843
Lithuania	1995	Fracture of hip	0.0892
Lithuania	2000	Fracture of hip	0.0722
Lithuania	2005	Fracture of hip	0.0633
Lithuania	2010	Fracture of hip	0.0577
Lithuania	2017	Fracture of hip	0.0577
Lithuania	1990	Amputation of upper limbs, bilateral	0.1151
Lithuania	1995	Amputation of upper limbs, bilateral	0.1202
Lithuania	2000	Amputation of upper limbs, bilateral	0.1026
Lithuania	2005	Amputation of upper limbs, bilateral	0.0934
Lithuania	2010	Amputation of upper limbs, bilateral	0.0876
Lithuania	2017	Amputation of upper limbs, bilateral	0.0876
Lithuania	1990	Fracture of patella, tibia or fibula, or ankle	0.0554
Lithuania	1995	Fracture of patella, tibia or fibula, or ankle	0.0554
Lithuania	2000	Fracture of patella, tibia or fibula, or ankle	0.0554
Lithuania	2005	Fracture of patella, tibia or fibula, or ankle	0.0554
Lithuania	2010	Fracture of patella, tibia or fibula, or ankle	0.0554
Lithuania	2017	Fracture of patella, tibia or fibula, or ankle	0.0554
Lithuania	1990	Fracture of pelvis	0.1823
Lithuania	1995	Fracture of pelvis	0.1823
Lithuania	2000	Fracture of pelvis	0.1823
Lithuania	2005	Fracture of pelvis	0.1823
Lithuania	2010	Fracture of pelvis	0.1823
Lithuania	2017	Fracture of pelvis	0.1823
Lithuania	1990	Fracture of radius and/or ulna	0.0435
Lithuania	1995	Fracture of radius and/or ulna	0.0435
Lithuania	2000	Fracture of radius and/or ulna	0.0435
Lithuania	2005	Fracture of radius and/or ulna	0.0435
Lithuania	2010	Fracture of radius and/or ulna	0.0435
Lithuania	2017	Fracture of radius and/or ulna	0.0435
Lithuania	1990	Fracture of skull	0.0714
Lithuania	1995	Fracture of skull	0.0714
Lithuania	2000	Fracture of skull	0.0714
Lithuania	2005	Fracture of skull	0.0714
Lithuania	2010	Fracture of skull	0.0714
Lithuania	2017	Fracture of skull	0.0714
Lithuania	1990	Fracture of sternum and/or fracture of one or more ribs	0.1027
Lithuania	1995	Fracture of sternum and/or fracture of one or more ribs	0.1027
Lithuania	2000	Fracture of sternum and/or fracture of one or more ribs	0.1027
Lithuania	2005	Fracture of sternum and/or fracture of one or more ribs	0.1027
Lithuania	2010	Fracture of sternum and/or fracture of one or more ribs	0.1027
Lithuania	2017	Fracture of sternum and/or fracture of one or more ribs	0.1027
Lithuania	1990	Fracture of vertebral column	0.1106
Lithuania	1995	Fracture of vertebral column	0.1106
Lithuania	2000	Fracture of vertebral column	0.1106
Lithuania	2005	Fracture of vertebral column	0.1106
Lithuania	2010	Fracture of vertebral column	0.1106
Lithuania	2017	Fracture of vertebral column	0.1106
Lithuania	1990	Fracture of femur, other than femoral neck	0.042
Lithuania	1995	Fracture of femur, other than femoral neck	0.042
Lithuania	2000	Fracture of femur, other than femoral neck	0.042
Lithuania	2005	Fracture of femur, other than femoral neck	0.042
Lithuania	2010	Fracture of femur, other than femoral neck	0.042
Lithuania	2017	Fracture of femur, other than femoral neck	0.042
Lithuania	1990	Minor TBI	0.1319
Lithuania	1995	Minor TBI	0.1319
Lithuania	2000	Minor TBI	0.1319
Lithuania	2005	Minor TBI	0.1319
Lithuania	2010	Minor TBI	0.1319
Lithuania	2017	Minor TBI	0.1319
Lithuania	1990	Moderate TBI	0.1637
Lithuania	1995	Moderate TBI	0.1637
Lithuania	2000	Moderate TBI	0.1637
Lithuania	2005	Moderate TBI	0.1637
Lithuania	2010	Moderate TBI	0.1637
Lithuania	2017	Moderate TBI	0.1637
Lithuania	1990	Amputation of fingers (excluding thumb)	0.0047
Lithuania	1995	Amputation of fingers (excluding thumb)	0.0047
Lithuania	2000	Amputation of fingers (excluding thumb)	0.0047
Lithuania	2005	Amputation of fingers (excluding thumb)	0.0047
Lithuania	2010	Amputation of fingers (excluding thumb)	0.0047
Lithuania	2017	Amputation of fingers (excluding thumb)	0.0047
Lithuania	1990	Spinal cord lesion at neck level	0.421
Lithuania	1995	Spinal cord lesion at neck level	0.4227
Lithuania	2000	Spinal cord lesion at neck level	0.4169
Lithuania	2005	Spinal cord lesion at neck level	0.4138
Lithuania	2010	Spinal cord lesion at neck level	0.4118
Lithuania	2017	Spinal cord lesion at neck level	0.4118
Lithuania	1990	Spinal cord lesion below neck level	0.1971
Lithuania	1995	Spinal cord lesion below neck level	0.2007
Lithuania	2000	Spinal cord lesion below neck level	0.1883
Lithuania	2005	Spinal cord lesion below neck level	0.1818
Lithuania	2010	Spinal cord lesion below neck level	0.1777
Lithuania	2017	Spinal cord lesion below neck level	0.1777
Lithuania	1990	Drowning and nonfatal submersion	0.2471
Lithuania	1995	Drowning and nonfatal submersion	0.2471
Lithuania	2000	Drowning and nonfatal submersion	0.2471
Lithuania	2005	Drowning and nonfatal submersion	0.2471
Lithuania	2010	Drowning and nonfatal submersion	0.2471
Lithuania	2017	Drowning and nonfatal submersion	0.2471
Lithuania	1990	Asphyxiation	0.2471
Lithuania	1995	Asphyxiation	0.2471
Lithuania	2000	Asphyxiation	0.2471
Lithuania	2005	Asphyxiation	0.2471
Lithuania	2010	Asphyxiation	0.2471
Lithuania	2017	Asphyxiation	0.2471
Lithuania	1990	Crush injury	0.1325
Lithuania	1995	Crush injury	0.1325
Lithuania	2000	Crush injury	0.1325
Lithuania	2005	Crush injury	0.1325
Lithuania	2010	Crush injury	0.1325
Lithuania	2017	Crush injury	0.1325
Lithuania	1990	Nerve injury	0.1129
Lithuania	1995	Nerve injury	0.1129
Lithuania	2000	Nerve injury	0.1129
Lithuania	2005	Nerve injury	0.1129
Lithuania	2010	Nerve injury	0.1129
Lithuania	2017	Nerve injury	0.1129
Lithuania	1990	Injury to eyes	0.0165
Lithuania	1995	Injury to eyes	0.0165
Lithuania	2000	Injury to eyes	0.0165
Lithuania	2005	Injury to eyes	0.0165
Lithuania	2010	Injury to eyes	0.0165
Lithuania	2017	Injury to eyes	0.0165
Lithuania	1990	Amputation of lower limb, unilateral	0.0493
Lithuania	1995	Amputation of lower limb, unilateral	0.0512
Lithuania	2000	Amputation of lower limb, unilateral	0.0446
Lithuania	2005	Amputation of lower limb, unilateral	0.0412
Lithuania	2010	Amputation of lower limb, unilateral	0.039
Lithuania	2017	Amputation of lower limb, unilateral	0.039
Lithuania	1990	Open wound(s)	0.011
Lithuania	1995	Open wound(s)	0.011
Lithuania	2000	Open wound(s)	0.011
Lithuania	2005	Open wound(s)	0.011
Lithuania	2010	Open wound(s)	0.011
Lithuania	2017	Open wound(s)	0.011
Lithuania	1990	Poisoning requiring urgent care	0.1628
Lithuania	1995	Poisoning requiring urgent care	0.1628
Lithuania	2000	Poisoning requiring urgent care	0.1628
Lithuania	2005	Poisoning requiring urgent care	0.1628
Lithuania	2010	Poisoning requiring urgent care	0.1628
Lithuania	2017	Poisoning requiring urgent care	0.1628
Lithuania	1990	Severe chest injury	0.0474
Lithuania	1995	Severe chest injury	0.0474
Lithuania	2000	Severe chest injury	0.0474
Lithuania	2005	Severe chest injury	0.0474
Lithuania	2010	Severe chest injury	0.0474
Lithuania	2017	Severe chest injury	0.0474
Lithuania	1990	Internal hemorrhage in abdomen and pelvis	0.1143

Lithuania	1995	Internal hemorrhage in abdomen and pelvis	0.1143
Lithuania	2000	Internal hemorrhage in abdomen and pelvis	0.1143
Lithuania	2005	Internal hemorrhage in abdomen and pelvis	0.1143
Lithuania	2010	Internal hemorrhage in abdomen and pelvis	0.1143
Lithuania	2017	Internal hemorrhage in abdomen and pelvis	0.1143
Lithuania	1990	Contusion in any part of the body	0.0075
Lithuania	1995	Contusion in any part of the body	0.0075
Lithuania	2000	Contusion in any part of the body	0.0075
Lithuania	2005	Contusion in any part of the body	0.0075
Lithuania	2010	Contusion in any part of the body	0.0075
Lithuania	2017	Contusion in any part of the body	0.0075
Lithuania	1990	Effect of different environmental factors	0.049
Lithuania	1995	Effect of different environmental factors	0.049
Lithuania	2000	Effect of different environmental factors	0.049
Lithuania	2005	Effect of different environmental factors	0.049
Lithuania	2010	Effect of different environmental factors	0.049
Lithuania	2017	Effect of different environmental factors	0.049
Lithuania	1990	Complications following therapeutic procedures	0.0506
Lithuania	1995	Complications following therapeutic procedures	0.0506
Lithuania	2000	Complications following therapeutic procedures	0.0506
Lithuania	2005	Complications following therapeutic procedures	0.0506
Lithuania	2010	Complications following therapeutic procedures	0.0506
Lithuania	2017	Complications following therapeutic procedures	0.0506
Lithuania	1990	Multiple fractures, dislocations, crashes, wounds, sprains, and strains	0.0843
Lithuania	1995	Multiple fractures, dislocations, crashes, wounds, sprains, and strains	0.0892
Lithuania	2000	Multiple fractures, dislocations, crashes, wounds, sprains, and strains	0.0722
Lithuania	2005	Multiple fractures, dislocations, crashes, wounds, sprains, and strains	0.0633
Lithuania	2010	Multiple fractures, dislocations, crashes, wounds, sprains, and strains	0.0577
Lithuania	2017	Multiple fractures, dislocations, crashes, wounds, sprains, and strains	0.0577
Lithuania	1990	Amputation of upper limb, unilateral	0.0451
Lithuania	1995	Amputation of upper limb, unilateral	0.0462
Lithuania	2000	Amputation of upper limb, unilateral	0.0423
Lithuania	2005	Amputation of upper limb, unilateral	0.0403
Lithuania	2010	Amputation of upper limb, unilateral	0.039
Lithuania	2017	Amputation of upper limb, unilateral	0.039
Lithuania	1990	Amputation of thumb	0.0114
Lithuania	1995	Amputation of thumb	0.0114
Lithuania	2000	Amputation of thumb	0.0114
Lithuania	2005	Amputation of thumb	0.0114
Lithuania	2010	Amputation of thumb	0.0114
Lithuania	2017	Amputation of thumb	0.0114
Lithuania	1990	Amputation of toe/toes	0.0059
Lithuania	1995	Amputation of toe/toes	0.0059
Lithuania	2000	Amputation of toe/toes	0.0059
Lithuania	2005	Amputation of toe/toes	0.0059
Lithuania	2010	Amputation of toe/toes	0.0059
Lithuania	2017	Amputation of toe/toes	0.0059
Lithuania	1990	Burns, <20% total burned surface area without lower airway burns	0.0163
Lithuania	1995	Burns, <20% total burned surface area without lower airway burns	0.0163
Lithuania	2000	Burns, <20% total burned surface area without lower airway burns	0.0163
Lithuania	2005	Burns, <20% total burned surface area without lower airway burns	0.0163
Lithuania	2010	Burns, <20% total burned surface area without lower airway burns	0.0163
Lithuania	2017	Burns, <20% total burned surface area without lower airway burns	0.0163
Lithuania	1990	Burns, >=20% total burned surface area or >= 10% burned surface area if head/neck or hands/wrist involved w/o lower airway burns	0.1593
Lithuania	1995	Burns, >=20% total burned surface area or >= 10% burned surface area if head/neck or hands/wrist involved w/o lower airway burns	0.1639
Lithuania	2000	Burns, >=20% total burned surface area or >= 10% burned surface area if head/neck or hands/wrist involved w/o lower airway burns	0.1481
Lithuania	2005	Burns, >=20% total burned surface area or >= 10% burned surface area if head/neck or hands/wrist involved w/o lower airway burns	0.1398
Lithuania	2010	Burns, >=20% total burned surface area or >= 10% burned surface area if head/neck or hands/wrist involved w/o lower airway burns	0.1346
Lithuania	2017	Burns, >=20% total burned surface area or >= 10% burned surface area if head/neck or hands/wrist involved w/o lower airway burns	0.1346
Republic of Moldova	1990	Amputation of lower limbs, bilateral	0.1861
Republic of Moldova	1995	Amputation of lower limbs, bilateral	0.1904
Republic of Moldova	2000	Amputation of lower limbs, bilateral	0.1812
Republic of Moldova	2005	Amputation of lower limbs, bilateral	0.1714
Republic of Moldova	2010	Amputation of lower limbs, bilateral	0.1638
Republic of Moldova	2017	Amputation of lower limbs, bilateral	0.1474
Republic of Moldova	1990	Lower airway burns	0.2252
Republic of Moldova	1995	Lower airway burns	0.2252
Republic of Moldova	2000	Lower airway burns	0.2252
Republic of Moldova	2005	Lower airway burns	0.2252
Republic of Moldova	2010	Lower airway burns	0.2252
Republic of Moldova	2017	Lower airway burns	0.2252
Republic of Moldova	1990	Dislocation of hip	0.0159
Republic of Moldova	1995	Dislocation of hip	0.0159
Republic of Moldova	2000	Dislocation of hip	0.0159
Republic of Moldova	2005	Dislocation of hip	0.0159
Republic of Moldova	2010	Dislocation of hip	0.0159
Republic of Moldova	2017	Dislocation of hip	0.0159
Republic of Moldova	1990	Dislocation of knee	0.1134
Republic of Moldova	1995	Dislocation of knee	0.1134
Republic of Moldova	2000	Dislocation of knee	0.1134
Republic of Moldova	2005	Dislocation of knee	0.1134
Republic of Moldova	2010	Dislocation of knee	0.1134
Republic of Moldova	2017	Dislocation of knee	0.1134
Republic of Moldova	1990	Dislocation of shoulder	0.062
Republic of Moldova	1995	Dislocation of shoulder	0.062
Republic of Moldova	2000	Dislocation of shoulder	0.062
Republic of Moldova	2005	Dislocation of shoulder	0.062
Republic of Moldova	2010	Dislocation of shoulder	0.062
Republic of Moldova	2017	Dislocation of shoulder	0.062
Republic of Moldova	1990	Muscle and tendon injuries, including sprains and strains lesser dislocations	0.0075
Republic of Moldova	1995	Muscle and tendon injuries, including sprains and strains lesser dislocations	0.0075
Republic of Moldova	2000	Muscle and tendon injuries, including sprains and strains lesser dislocations	0.0075
Republic of Moldova	2005	Muscle and tendon injuries, including sprains and strains lesser dislocations	0.0075
Republic of Moldova	2010	Muscle and tendon injuries, including sprains and strains lesser dislocations	0.0075
Republic of Moldova	2017	Muscle and tendon injuries, including sprains and strains lesser dislocations	0.0075
Republic of Moldova	1990	Fracture of clavicle, scapula, or humerus	0.0349
Republic of Moldova	1995	Fracture of clavicle, scapula, or humerus	0.0349
Republic of Moldova	2000	Fracture of clavicle, scapula, or humerus	0.0349
Republic of Moldova	2005	Fracture of clavicle, scapula, or humerus	0.0349
Republic of Moldova	2010	Fracture of clavicle, scapula, or humerus	0.0349
Republic of Moldova	2017	Fracture of clavicle, scapula, or humerus	0.0349
Republic of Moldova	1990	Fracture of face bones	0.0669
Republic of Moldova	1995	Fracture of face bones	0.0669
Republic of Moldova	2000	Fracture of face bones	0.0669
Republic of Moldova	2005	Fracture of face bones	0.0669
Republic of Moldova	2010	Fracture of face bones	0.0669
Republic of Moldova	2017	Fracture of face bones	0.0669
Republic of Moldova	1990	Fracture of foot bones except ankle	0.0264
Republic of Moldova	1995	Fracture of foot bones except ankle	0.0264
Republic of Moldova	2000	Fracture of foot bones except ankle	0.0264
Republic of Moldova	2005	Fracture of foot bones except ankle	0.0264
Republic of Moldova	2010	Fracture of foot bones except ankle	0.0264
Republic of Moldova	2017	Fracture of foot bones except ankle	0.0264
Republic of Moldova	1990	Fracture of hand (wrist and other distal part of hand)	0.0138
Republic of Moldova	1995	Fracture of hand (wrist and other distal part of hand)	0.0138
Republic of Moldova	2000	Fracture of hand (wrist and other distal part of hand)	0.0138
Republic of Moldova	2005	Fracture of hand (wrist and other distal part of hand)	0.0138
Republic of Moldova	2010	Fracture of hand (wrist and other distal part of hand)	0.0138
Republic of Moldova	2017	Fracture of hand (wrist and other distal part of hand)	0.0138
Republic of Moldova	1990	Fracture of hip	0.1408
Republic of Moldova	1995	Fracture of hip	0.1465
Republic of Moldova	2000	Fracture of hip	0.1344
Republic of Moldova	2005	Fracture of hip	0.1214
Republic of Moldova	2010	Fracture of hip	0.1113
Republic of Moldova	2017	Fracture of hip	0.0896
Republic of Moldova	1990	Amputation of upper limbs, bilateral	0.1735
Republic of Moldova	1995	Amputation of upper limbs, bilateral	0.1793
Republic of Moldova	2000	Amputation of upper limbs, bilateral	0.1668
Republic of Moldova	2005	Amputation of upper limbs, bilateral	0.1534
Republic of Moldova	2010	Amputation of upper limbs, bilateral	0.143
Republic of Moldova	2017	Amputation of upper limbs, bilateral	0.1206
Republic of Moldova	1990	Fracture of patella, tibia or fibula, or ankle	0.0554
Republic of Moldova	1995	Fracture of patella, tibia or fibula, or ankle	0.0554
Republic of Moldova	2000	Fracture of patella, tibia or fibula, or ankle	0.0554
Republic of Moldova	2005	Fracture of patella, tibia or fibula, or ankle	0.0554
Republic of Moldova	2010	Fracture of patella, tibia or fibula, or ankle	0.0554
Republic of Moldova	2017	Fracture of patella, tibia or fibula, or ankle	0.0554
Republic of Moldova	1990	Fracture of pelvis	0.1823
Republic of Moldova	1995	Fracture of pelvis	0.1823
Republic of Moldova	2000	Fracture of pelvis	0.1823
Republic of Moldova	2005	Fracture of pelvis	0.1823
Republic of Moldova	2010	Fracture of pelvis	0.1823
Republic of Moldova	2017	Fracture of pelvis	0.1823
Republic of Moldova	1990	Fracture of radius and/or ulna	0.0435
Republic of Moldova	1995	Fracture of radius and/or ulna	0.0435
Republic of Moldova	2000	Fracture of radius and/or ulna	0.0435
Republic of Moldova	2005	Fracture of radius and/or ulna	0.0435
Republic of Moldova	2010	Fracture of radius and/or ulna	0.0435
Republic of Moldova	2017	Fracture of radius and/or ulna	0.0435
Republic of Moldova	1990	Fracture of skull	0.0714
Republic of Moldova	1995	Fracture of skull	0.0714
Republic of Moldova	2000	Fracture of skull	0.0714
Republic of Moldova	2005	Fracture of skull	0.0714
Republic of Moldova	2010	Fracture of skull	0.0714
Republic of Moldova	2017	Fracture of skull	0.0714
Republic of Moldova	1990	Fracture of sternum and/or fracture of one or more ribs	0.1027
Republic of Moldova	1995	Fracture of sternum and/or fracture of one or more ribs	0.1027
Republic of Moldova	2000	Fracture of sternum and/or fracture of one or more ribs	0.1027
Republic of Moldova	2005	Fracture of sternum and/or fracture of one or more ribs	0.1027
Republic of Moldova	2010	Fracture of sternum and/or fracture of one or more ribs	0.1027
Republic of Moldova	2017	Fracture of sternum and/or fracture of one or more ribs	0.1027
Republic of Moldova	1990	Fracture of vertebral column	0.1106
Republic of Moldova	1995	Fracture of vertebral column	0.1106
Republic of Moldova	2000	Fracture of vertebral column	0.1106
Republic of Moldova	2005	Fracture of vertebral column	0.1106
Republic of Moldova	2010	Fracture of vertebral column	0.1106
Republic of Moldova	2017	Fracture of vertebral column	0.1106
Republic of Moldova	1990	Fracture of femur, other than femoral neck	0.042
Republic of Moldova	1995	Fracture of femur, other than femoral neck	0.042
Republic of Moldova	2000	Fracture of femur, other than femoral neck	0.042
Republic of Moldova	2005	Fracture of femur, other than femoral neck	0.042
Republic of Moldova	2010	Fracture of femur, other than femoral neck	0.042
Republic of Moldova	2017	Fracture of femur, other than femoral neck	0.042
Republic of Moldova	1990	Minor TBI	0.1319
Republic of Moldova	1995	Minor TBI	0.1319
Republic of Moldova	2000	Minor TBI	0.1319
Republic of Moldova	2005	Minor TBI	0.1319
Republic of Moldova	2010	Minor TBI	0.1319
Republic of Moldova	2017	Minor TBI	0.1319
Republic of Moldova	1990	Moderate TBI	0.1637
Republic of Moldova	1995	Moderate TBI	0.1637
Republic of Moldova	2000	Moderate TBI	0.1637
Republic of Moldova	2005	Moderate TBI	0.1637
Republic of Moldova	2010	Moderate TBI	0.1637
Republic of Moldova	2017	Moderate TBI	0.1637
Republic of Moldova	1990	Amputation of fingers (excluding thumb)	0.0047
Republic of Moldova	1995	Amputation of fingers (excluding thumb)	0.0047
Republic of Moldova	2000	Amputation of fingers (excluding thumb)	0.0047
Republic of Moldova	2005	Amputation of fingers (excluding thumb)	0.0047
Republic of Moldova	2010	Amputation of fingers (excluding thumb)	0.0047
Republic of Moldova	2017	Amputation of fingers (excluding thumb)	0.0047
Republic of Moldova	1990	Spinal cord lesion at neck level	0.4406
Republic of Moldova	1995	Spinal cord lesion at neck level	0.4425
Republic of Moldova	2000	Spinal cord lesion at neck level	0.4383
Republic of Moldova	2005	Spinal cord lesion at neck level	0.4339
Republic of Moldova	2010	Spinal cord lesion at neck level	0.4304
Republic of Moldova	2017	Spinal cord lesion at neck level	0.4229
Republic of Moldova	1990	Spinal cord lesion below neck level	0.2384
Republic of Moldova	1995	Spinal cord lesion below neck level	0.2425
Republic of Moldova	2000	Spinal cord lesion below neck level	0.2336
Republic of Moldova	2005	Spinal cord lesion below neck level	0.2242
Republic of Moldova	2010	Spinal cord lesion below neck level	0.2168
Republic of Moldova	2017	Spinal cord lesion below neck level	0.201
Republic of Moldova	1990	Drowning and nonfatal submersion	0.2471
Republic of Moldova	1995	Drowning and nonfatal submersion	0.2471
Republic of Moldova	2000	Drowning and nonfatal submersion	0.2471
Republic of Moldova	2005	Drowning and nonfatal submersion	0.2471
Republic of Moldova	2010	Drowning and nonfatal submersion	0.2471
Republic of Moldova	2017	Drowning and nonfatal submersion	0.2471
Republic of Moldova	1990	Asphyxiation	0.2471
Republic of Moldova	1995	Asphyxiation	0.2471
Republic of Moldova	2000	Asphyxiation	0.2471
Republic of Moldova	2005	Asphyxiation	0.2471
Republic of Moldova	2010	Asphyxiation	0.2471
Republic of Moldova	2017	Asphyxiation	0.2471
Republic of Moldova	1990	Crush injury	0.1325
Republic of Moldova	1995	Crush injury	0.1325
Republic of Moldova	2000	Crush injury	0.1325
Republic of Moldova	2005	Crush injury	0.1325
Republic of Moldova	2010	Crush injury	0.1325
Republic of Moldova	2017	Crush injury	0.1325
Republic of Moldova	1990	Nerve injury	0.1129
Republic of Moldova	1995	Nerve injury	0.1129
Republic of Moldova	2000	Nerve injury	0.1129
Republic of Moldova	2005	Nerve injury	0.1129
Republic of Moldova	2010	Nerve injury	0.1129
Republic of Moldova	2017	Nerve injury	0.1129
Republic of Moldova	1990	Injury to eyes	0.0165
Republic of Moldova	1995	Injury to eyes	0.0165
Republic of Moldova	2000	Injury to eyes	0.0165
Republic of Moldova	2005	Injury to eyes	0.0165
Republic of Moldova	2010	Injury to eyes	0.0165
Republic of Moldova	2017	Injury to eyes	0.0165
Republic of Moldova	1990	Amputation of lower limb, unilateral	0.0714
Republic of Moldova	1995	Amputation of lower limb, unilateral	0.0736
Republic of Moldova	2000	Amputation of lower limb, unilateral	0.0689
Republic of Moldova	2005	Amputation of lower limb, unilateral	0.0638
Republic of Moldova	2010	Amputation of lower limb, unilateral	0.0589
Republic of Moldova	2017	Amputation of lower limb, unilateral	0.0514
Republic of Moldova	1990	Open wound(s)	0.011
Republic of Moldova	1995	Open wound(s)	0.011
Republic of Moldova	2000	Open wound(s)	0.011
Republic of Moldova	2005	Open wound(s)	0.011
Republic of Moldova	2010	Open wound(s)	0.011
Republic of Moldova	2017	Open wound(s)	0.011
Republic of Moldova	1990	Poisoning requiring urgent care	0.1628
Republic of Moldova	1995	Poisoning requiring urgent care	0.1628
Republic of Moldova	2000	Poisoning requiring urgent care	0.1628
Republic of Moldova	2005	Poisoning requiring urgent care	0.1628
Republic of Moldova	2010	Poisoning requiring urgent care	0.1628
Republic of Moldova	2017	Poisoning requiring urgent care	0.1628
Republic of Moldova	1990	Severe chest injury	0.0474
Republic of Moldova	1995	Severe chest injury	0.0474
Republic of Moldova	2000	Severe chest injury	0.0474
Republic of Moldova	2005	Severe chest injury	0.0474
Republic of Moldova	2010	Severe chest injury	0.0474
Republic of Moldova	2017	Severe chest injury	0.0474
Republic of Moldova	1990	Internal hemorrhage in abdomen and pelvis	0.1143
Republic of Moldova	1995	Internal hemorrhage in abdomen and pelvis	0.1143
Republic of Moldova	2000	Internal hemorrhage in abdomen and pelvis	0.1143
Republic of Moldova	2005	Internal hemorrhage in abdomen and pelvis	0.1143
Republic of Moldova	2010	Internal hemorrhage in abdomen and pelvis	0.1143
Republic of Moldova	2017	Internal hemorrhage in abdomen and pelvis	0.1143
Republic of Moldova	1990	Contusion in any part of the body	0.0075
Republic of Moldova	1995	Contusion in any part of the body	0.0075
Republic of Moldova	2000	Contusion in any part of the body	0.0075
Republic of Moldova	2005	Contusion in any part of the body	0.0075
Republic of Moldova	2010	Contusion in any part of the body	0.0075
Republic of Moldova	2017	Contusion in any part of the body	0.0075
Republic of Moldova	1990	Effect of different environmental factors	0.049
Republic of Moldova	1995	Effect of different environmental factors	0.049
Republic of Moldova	2000	Effect of different environmental factors	0.049
Republic of Moldova	2005	Effect of different environmental factors	0.049
Republic of Moldova	2010	Effect of different environmental factors	0.049
Republic of Moldova	2017	Effect of different environmental factors	0.049
Republic of Moldova	1990	Complications following therapeutic procedures	0.0506
Republic of Moldova	1995	Complications following therapeutic procedures	0.0506
Republic of Moldova	2000	Complications following therapeutic procedures	0.0506
Republic of Moldova	2005	Complications following therapeutic procedures	0.0506
Republic of Moldova	2010	Complications following therapeutic procedures	0.0506
Republic of Moldova	2017	Complications following therapeutic procedures	0.0506
Republic of Moldova	1990	Multiple fractures, dislocations, crashes, wounds, sprains, and strains	0.1408
Republic of Moldova	1995	Multiple fractures, dislocations, crashes, wounds, sprains, and strains	0.1465
Republic of Moldova	2000	Multiple fractures, dislocations, crashes, wounds, sprains, and strains	0.1344
Republic of Moldova	2005	Multiple fractures, dislocations, crashes, wounds, sprains, and strains	0.1214
Republic of Moldova	2010	Multiple fractures, dislocations, crashes, wounds, sprains, and strains	0.1113
Republic of Moldova	2017	Multiple fractures, dislocations, crashes, wounds, sprains, and strains	0.0896
Republic of Moldova	1990	Amputation of upper limb, unilateral	0.0581
Republic of Moldova	1995	Amputation of upper limb, unilateral	0.0584
Republic of Moldova	2000	Amputation of upper limb, unilateral	0.0566
Republic of Moldova	2005	Amputation of upper limb, unilateral	0.0536
Republic of Moldova	2010	Amputation of upper limb, unilateral	0.0513
Republic of Moldova	2017	Amputation of upper limb, unilateral	0.0463
Republic of Moldova	1990	Amputation of thumb	0.0114
Republic of Moldova	1995	Amputation of thumb	0.0114
Republic of Moldova	2000	Amputation of thumb	0.0114
Republic of Moldova	2005	Amputation of thumb	0.0114
Republic of Moldova	2010	Amputation of thumb	0.0114
Republic of Moldova	2017	Amputation of thumb	0.0114
Republic of Moldova	1990	Amputation of toe/toes	0.0059
Republic of Moldova	1995	Amputation of toe/toes	0.0059
Republic of Moldova	2000	Amputation of toe/toes	0.0059
Republic of Moldova	2005	Amputation of toe/toes	0.0059
Republic of Moldova	2010	Amputation of toe/toes	0.0059
Republic of Moldova	2017	Amputation of toe/toes	0.0059
Republic of Moldova	1990	Burns, <20% total burned surface area without lower airway burns	0.0163
Republic of Moldova	1995	Burns, <20% total burned surface area without lower airway burns	0.0163
Republic of Moldova	2000	Burns, <20% total burned surface area without lower airway burns	0.0163
Republic of Moldova	2005	Burns, <20% total burned surface area without lower airway burns	0.0163
Republic of Moldova	2010	Burns, <20% total burned surface area without lower airway burns	0.0163
Republic of Moldova	2017	Burns, <20% total burned surface area without lower airway burns	0.0163
Republic of Moldova	1990	Burns, >=20% total burned surface area or >= 10% burned surface area if head/neck or hands/wrist involved w/o lower airway burns	0.212
Republic of Moldova	1995	Burns, >=20% total burned surface area or >= 10% burned surface area if head/neck or hands/wrist involved w/o lower airway burns	0.2173
Republic of Moldova	2000	Burns, >=20% total burned surface area or >= 10% burned surface area if head/neck or hands/wrist involved w/o lower airway burns	0.206
Republic of Moldova	2005	Burns, >=20% total burned surface area or >= 10% burned surface area if head/neck or hands/wrist involved w/o lower airway burns	0.1939
Republic of Moldova	2010	Burns, >=20% total burned surface area or >= 10% burned surface area if head/neck or hands/wrist involved w/o lower airway burns	0.1845
Republic of Moldova	2017	Burns, >=20% total burned surface area or >= 10% burned surface area if head/neck or hands/wrist involved w/o lower airway burns	0.1643
Brunei Darussalam	1990	Amputation of lower limbs, bilateral	0.1641
Brunei Darussalam	1995	Amputation of lower limbs, bilateral	0.1533
Brunei Darussalam	2000	Amputation of lower limbs, bilateral	0.1402
Brunei Darussalam	2005	Amputation of lower limbs, bilateral	0.1289
Brunei Darussalam	2010	Amputation of lower limbs, bilateral	0.1233
Brunei Darussalam	2017	Amputation of lower limbs, bilateral	0.1232
Brunei Darussalam	1990	Lower airway burns	0.2252
Brunei Darussalam	1995	Lower airway burns	0.2252
Brunei Darussalam	2000	Lower airway burns	0.2252
Brunei Darussalam	2005	Lower airway burns	0.2252
Brunei Darussalam	2010	Lower airway burns	0.2252
Brunei Darussalam	2017	Lower airway burns	0.2252
Brunei Darussalam	1990	Dislocation of hip	0.0159
Brunei Darussalam	1995	Dislocation of hip	0.0159
Brunei Darussalam	2000	Dislocation of hip	0.0159
Brunei Darussalam	2005	Dislocation of hip	0.0159
Brunei Darussalam	2010	Dislocation of hip	0.0159
Brunei Darussalam	2017	Dislocation of hip	0.0159
Brunei Darussalam	1990	Dislocation of knee	0.1134
Brunei Darussalam	1995	Dislocation of knee	0.1134
Brunei Darussalam	2000	Dislocation of knee	0.1134
Brunei Darussalam	2005	Dislocation of knee	0.1134
Brunei Darussalam	2010	Dislocation of knee	0.1134
Brunei Darussalam	2017	Dislocation of knee	0.1134
Brunei Darussalam	1990	Dislocation of shoulder	0.062
Brunei Darussalam	1995	Dislocation of shoulder	0.062
Brunei Darussalam	2000	Dislocation of shoulder	0.062
Brunei Darussalam	2005	Dislocation of shoulder	0.062
Brunei Darussalam	2010	Dislocation of shoulder	0.062
Brunei Darussalam	2017	Dislocation of shoulder	0.062
Brunei Darussalam	1990	Muscle and tendon injuries, including sprains and strains lesser dislocations	0.0075
Brunei Darussalam	1995	Muscle and tendon injuries, including sprains and strains lesser dislocations	0.0075
Brunei Darussalam	2000	Muscle and tendon injuries, including sprains and strains lesser dislocations	0.0075
Brunei Darussalam	2005	Muscle and tendon injuries, including sprains and strains lesser dislocations	0.0075
Brunei Darussalam	2010	Muscle and tendon injuries, including sprains and strains lesser dislocations	0.0075
Brunei Darussalam	2017	Muscle and tendon injuries, including sprains and strains lesser dislocations	0.0075
Brunei Darussalam	1990	Fracture of clavicle, scapula, or humerus	0.0349
Brunei Darussalam	1995	Fracture of clavicle, scapula, or humerus	0.0349
Brunei Darussalam	2000	Fracture of clavicle, scapula, or humerus	0.0349
Brunei Darussalam	2005	Fracture of clavicle, scapula, or humerus	0.0349
Brunei Darussalam	2010	Fracture of clavicle, scapula, or humerus	0.0349
Brunei Darussalam	2017	Fracture of clavicle, scapula, or humerus	0.0349
Brunei Darussalam	1990	Fracture of face bones	0.0669
Brunei Darussalam	1995	Fracture of face bones	0.0669
Brunei Darussalam	2000	Fracture of face bones	0.0669
Brunei Darussalam	2005	Fracture of face bones	0.0669
Brunei Darussalam	2010	Fracture of face bones	0.0669
Brunei Darussalam	2017	Fracture of face bones	0.0669
Brunei Darussalam	1990	Fracture of foot bones except ankle	0.0264
Brunei Darussalam	1995	Fracture of foot bones except ankle	0.0264
Brunei Darussalam	2000	Fracture of foot bones except ankle	0.0264
Brunei Darussalam	2005	Fracture of foot bones except ankle	0.0264
Brunei Darussalam	2010	Fracture of foot bones except ankle	0.0264
Brunei Darussalam	2017	Fracture of foot bones except ankle	0.0264
Brunei Darussalam	1990	Fracture of hand (wrist and other distal part of hand)	0.0138
Brunei Darussalam	1995	Fracture of hand (wrist and other distal part of hand)	0.0138
Brunei Darussalam	2000	Fracture of hand (wrist and other distal part of hand)	0.0138
Brunei Darussalam	2005	Fracture of hand (wrist and other distal part of hand)	0.0138
Brunei Darussalam	2010	Fracture of hand (wrist and other distal part of hand)	0.0138
Brunei Darussalam	2017	Fracture of hand (wrist and other distal part of hand)	0.0138
Brunei Darussalam	1990	Fracture of hip	0.1118
Brunei Darussalam	1995	Fracture of hip	0.0974
Brunei Darussalam	2000	Fracture of hip	0.0801
Brunei Darussalam	2005	Fracture of hip	0.0651
Brunei Darussalam	2010	Fracture of hip	0.0577
Brunei Darussalam	2017	Fracture of hip	0.0577
Brunei Darussalam	1990	Amputation of upper limbs, bilateral	0.1435
Brunei Darussalam	1995	Amputation of upper limbs, bilateral	0.1287
Brunei Darussalam	2000	Amputation of upper limbs, bilateral	0.1108
Brunei Darussalam	2005	Amputation of upper limbs, bilateral	0.0953
Brunei Darussalam	2010	Amputation of upper limbs, bilateral	0.0877
Brunei Darussalam	2017	Amputation of upper limbs, bilateral	0.0876
Brunei Darussalam	1990	Fracture of patella, tibia or fibula, or ankle	0.0554
Brunei Darussalam	1995	Fracture of patella, tibia or fibula, or ankle	0.0554
Brunei Darussalam	2000	Fracture of patella, tibia or fibula, or ankle	0.0554
Brunei Darussalam	2005	Fracture of patella, tibia or fibula, or ankle	0.0554
Brunei Darussalam	2010	Fracture of patella, tibia or fibula, or ankle	0.0554
Brunei Darussalam	2017	Fracture of patella, tibia or fibula, or ankle	0.0554
Brunei Darussalam	1990	Fracture of pelvis	0.1823
Brunei Darussalam	1995	Fracture of pelvis	0.1823
Brunei Darussalam	2000	Fracture of pelvis	0.1823
Brunei Darussalam	2005	Fracture of pelvis	0.1823
Brunei Darussalam	2010	Fracture of pelvis	0.1823
Brunei Darussalam	2017	Fracture of pelvis	0.1823
Brunei Darussalam	1990	Fracture of radius and/or ulna	0.0435
Brunei Darussalam	1995	Fracture of radius and/or ulna	0.0435
Brunei Darussalam	2000	Fracture of radius and/or ulna	0.0435
Brunei Darussalam	2005	Fracture of radius and/or ulna	0.0435
Brunei Darussalam	2010	Fracture of radius and/or ulna	0.0435
Brunei Darussalam	2017	Fracture of radius and/or ulna	0.0435
Brunei Darussalam	1990	Fracture of skull	0.0714
Brunei Darussalam	1995	Fracture of skull	0.0714
Brunei Darussalam	2000	Fracture of skull	0.0714
Brunei Darussalam	2005	Fracture of skull	0.0714
Brunei Darussalam	2010	Fracture of skull	0.0714
Brunei Darussalam	2017	Fracture of skull	0.0714
Brunei Darussalam	1990	Fracture of sternum and/or fracture of one or more ribs	0.1027
Brunei Darussalam	1995	Fracture of sternum and/or fracture of one or more ribs	0.1027
Brunei Darussalam	2000	Fracture of sternum and/or fracture of one or more ribs	0.1027
Brunei Darussalam	2005	Fracture of sternum and/or fracture of one or more ribs	0.1027
Brunei Darussalam	2010	Fracture of sternum and/or fracture of one or more ribs	0.1027
Brunei Darussalam	2017	Fracture of sternum and/or fracture of one or more ribs	0.1027
Brunei Darussalam	1990	Fracture of vertebral column	0.1106
Brunei Darussalam	1995	Fracture of vertebral column	0.1106
Brunei Darussalam	2000	Fracture of vertebral column	0.1106
Brunei Darussalam	2005	Fracture of vertebral column	0.1106
Brunei Darussalam	2010	Fracture of vertebral column	0.1106
Brunei Darussalam	2017	Fracture of vertebral column	0.1106
Brunei Darussalam	1990	Fracture of femur, other than femoral neck	0.042
Brunei Darussalam	1995	Fracture of femur, other than femoral neck	0.042
Brunei Darussalam	2000	Fracture of femur, other than femoral neck	0.042
Brunei Darussalam	2005	Fracture of femur, other than femoral neck	0.042
Brunei Darussalam	2010	Fracture of femur, other than femoral neck	0.042
Brunei Darussalam	2017	Fracture of femur, other than femoral neck	0.042

Brunei Darussalam	1990	Minor TBI	0.1319
Brunei Darussalam	1995	Minor TBI	0.1319
Brunei Darussalam	2000	Minor TBI	0.1319
Brunei Darussalam	2005	Minor TBI	0.1319
Brunei Darussalam	2010	Minor TBI	0.1319
Brunei Darussalam	2017	Minor TBI	0.1319
Brunei Darussalam	1990	Moderate TBI	0.1637
Brunei Darussalam	1995	Moderate TBI	0.1637
Brunei Darussalam	2000	Moderate TBI	0.1637
Brunei Darussalam	2005	Moderate TBI	0.1637
Brunei Darussalam	2010	Moderate TBI	0.1637
Brunei Darussalam	2017	Moderate TBI	0.1637
Brunei Darussalam	1990	Amputation of fingers (excluding thumb)	0.0047
Brunei Darussalam	1995	Amputation of fingers (excluding thumb)	0.0047
Brunei Darussalam	2000	Amputation of fingers (excluding thumb)	0.0047
Brunei Darussalam	2005	Amputation of fingers (excluding thumb)	0.0047
Brunei Darussalam	2010	Amputation of fingers (excluding thumb)	0.0047
Brunei Darussalam	2017	Amputation of fingers (excluding thumb)	0.0047
Brunei Darussalam	1990	Spinal cord lesion at neck level	0.4305
Brunei Darussalam	1995	Spinal cord lesion at neck level	0.4256
Brunei Darussalam	2000	Spinal cord lesion at neck level	0.4196
Brunei Darussalam	2005	Spinal cord lesion at neck level	0.4144
Brunei Darussalam	2010	Spinal cord lesion at neck level	0.4118
Brunei Darussalam	2017	Spinal cord lesion at neck level	0.4118
Brunei Darussalam	1990	Spinal cord lesion below neck level	0.2171
Brunei Darussalam	1995	Spinal cord lesion below neck level	0.2067
Brunei Darussalam	2000	Spinal cord lesion below neck level	0.1941
Brunei Darussalam	2005	Spinal cord lesion below neck level	0.1831
Brunei Darussalam	2010	Spinal cord lesion below neck level	0.1777
Brunei Darussalam	2017	Spinal cord lesion below neck level	0.1777
Brunei Darussalam	1990	Drowning and nonfatal submersion	0.2471
Brunei Darussalam	1995	Drowning and nonfatal submersion	0.2471
Brunei Darussalam	2000	Drowning and nonfatal submersion	0.2471
Brunei Darussalam	2005	Drowning and nonfatal submersion	0.2471
Brunei Darussalam	2010	Drowning and nonfatal submersion	0.2471
Brunei Darussalam	2017	Drowning and nonfatal submersion	0.2471
Brunei Darussalam	1990	Asphyxiation	0.2471
Brunei Darussalam	1995	Asphyxiation	0.2471
Brunei Darussalam	2000	Asphyxiation	0.2471
Brunei Darussalam	2005	Asphyxiation	0.2471
Brunei Darussalam	2010	Asphyxiation	0.2471
Brunei Darussalam	2017	Asphyxiation	0.2471
Brunei Darussalam	1990	Crush injury	0.1325
Brunei Darussalam	1995	Crush injury	0.1325
Brunei Darussalam	2000	Crush injury	0.1325
Brunei Darussalam	2005	Crush injury	0.1325
Brunei Darussalam	2010	Crush injury	0.1325
Brunei Darussalam	2017	Crush injury	0.1325
Brunei Darussalam	1990	Nerve injury	0.1129
Brunei Darussalam	1995	Nerve injury	0.1129
Brunei Darussalam	2000	Nerve injury	0.1129
Brunei Darussalam	2005	Nerve injury	0.1129
Brunei Darussalam	2010	Nerve injury	0.1129
Brunei Darussalam	2017	Nerve injury	0.1129
Brunei Darussalam	1990	Injury to eyes	0.0165
Brunei Darussalam	1995	Injury to eyes	0.0165
Brunei Darussalam	2000	Injury to eyes	0.0165
Brunei Darussalam	2005	Injury to eyes	0.0165
Brunei Darussalam	2010	Injury to eyes	0.0165
Brunei Darussalam	2017	Injury to eyes	0.0165
Brunei Darussalam	1990	Amputation of lower limb, unilateral	0.06
Brunei Darussalam	1995	Amputation of lower limb, unilateral	0.0545
Brunei Darussalam	2000	Amputation of lower limb, unilateral	0.0477
Brunei Darussalam	2005	Amputation of lower limb, unilateral	0.0419
Brunei Darussalam	2010	Amputation of lower limb, unilateral	0.039
Brunei Darussalam	2017	Amputation of lower limb, unilateral	0.039
Brunei Darussalam	1990	Open wound(s)	0.011
Brunei Darussalam	1995	Open wound(s)	0.011
Brunei Darussalam	2000	Open wound(s)	0.011
Brunei Darussalam	2005	Open wound(s)	0.011
Brunei Darussalam	2010	Open wound(s)	0.011
Brunei Darussalam	2017	Open wound(s)	0.011
Brunei Darussalam	1990	Poisoning requiring urgent care	0.1628
Brunei Darussalam	1995	Poisoning requiring urgent care	0.1628
Brunei Darussalam	2000	Poisoning requiring urgent care	0.1628
Brunei Darussalam	2005	Poisoning requiring urgent care	0.1628
Brunei Darussalam	2010	Poisoning requiring urgent care	0.1628
Brunei Darussalam	2017	Poisoning requiring urgent care	0.1628
Brunei Darussalam	1990	Severe chest injury	0.0474
Brunei Darussalam	1995	Severe chest injury	0.0474
Brunei Darussalam	2000	Severe chest injury	0.0474
Brunei Darussalam	2005	Severe chest injury	0.0474
Brunei Darussalam	2010	Severe chest injury	0.0474
Brunei Darussalam	2017	Severe chest injury	0.0474
Brunei Darussalam	1990	Internal hemorrhage in abdomen and pelvis	0.1143
Brunei Darussalam	1995	Internal hemorrhage in abdomen and pelvis	0.1143
Brunei Darussalam	2000	Internal hemorrhage in abdomen and pelvis	0.1143
Brunei Darussalam	2005	Internal hemorrhage in abdomen and pelvis	0.1143
Brunei Darussalam	2010	Internal hemorrhage in abdomen and pelvis	0.1143
Brunei Darussalam	2017	Internal hemorrhage in abdomen and pelvis	0.1143
Brunei Darussalam	1990	Contusion in any part of the body	0.0075
Brunei Darussalam	1995	Contusion in any part of the body	0.0075
Brunei Darussalam	2000	Contusion in any part of the body	0.0075
Brunei Darussalam	2005	Contusion in any part of the body	0.0075
Brunei Darussalam	2010	Contusion in any part of the body	0.0075
Brunei Darussalam	2017	Contusion in any part of the body	0.0075
Brunei Darussalam	1990	Effect of different environmental factors	0.049
Brunei Darussalam	1995	Effect of different environmental factors	0.049
Brunei Darussalam	2000	Effect of different environmental factors	0.049
Brunei Darussalam	2005	Effect of different environmental factors	0.049
Brunei Darussalam	2010	Effect of different environmental factors	0.049
Brunei Darussalam	2017	Effect of different environmental factors	0.049
Brunei Darussalam	1990	Complications following therapeutic procedures	0.0506
Brunei Darussalam	1995	Complications following therapeutic procedures	0.0506
Brunei Darussalam	2000	Complications following therapeutic procedures	0.0506
Brunei Darussalam	2005	Complications following therapeutic procedures	0.0506
Brunei Darussalam	2010	Complications following therapeutic procedures	0.0506
Brunei Darussalam	2017	Complications following therapeutic procedures	0.0506
Brunei Darussalam	1990	Multiple fractures, dislocations, crashes, wounds, sprains, and strains	0.1118
Brunei Darussalam	1995	Multiple fractures, dislocations, crashes, wounds, sprains, and strains	0.0974
Brunei Darussalam	2000	Multiple fractures, dislocations, crashes, wounds, sprains, and strains	0.0801
Brunei Darussalam	2005	Multiple fractures, dislocations, crashes, wounds, sprains, and strains	0.0651
Brunei Darussalam	2010	Multiple fractures, dislocations, crashes, wounds, sprains, and strains	0.0577
Brunei Darussalam	2017	Multiple fractures, dislocations, crashes, wounds, sprains, and strains	0.0577
Brunei Darussalam	1990	Amputation of upper limb, unilateral	0.0514
Brunei Darussalam	1995	Amputation of upper limb, unilateral	0.0481
Brunei Darussalam	2000	Amputation of upper limb, unilateral	0.0441
Brunei Darussalam	2005	Amputation of upper limb, unilateral	0.0407
Brunei Darussalam	2010	Amputation of upper limb, unilateral	0.039
Brunei Darussalam	2017	Amputation of upper limb, unilateral	0.039
Brunei Darussalam	1990	Amputation of thumb	0.0114
Brunei Darussalam	1995	Amputation of thumb	0.0114
Brunei Darussalam	2000	Amputation of thumb	0.0114
Brunei Darussalam	2005	Amputation of thumb	0.0114
Brunei Darussalam	2010	Amputation of thumb	0.0114
Brunei Darussalam	2017	Amputation of thumb	0.0114
Brunei Darussalam	1990	Amputation of toe/toes	0.0059
Brunei Darussalam	1995	Amputation of toe/toes	0.0059
Brunei Darussalam	2000	Amputation of toe/toes	0.0059
Brunei Darussalam	2005	Amputation of toe/toes	0.0059
Brunei Darussalam	2010	Amputation of toe/toes	0.0059
Brunei Darussalam	2017	Amputation of toe/toes	0.0059
Brunei Darussalam	1990	Burns, <20% total burned surface area without lower airway burns	0.0163
Brunei Darussalam	1995	Burns, <20% total burned surface area without lower airway burns	0.0163
Brunei Darussalam	2000	Burns, <20% total burned surface area without lower airway burns	0.0163
Brunei Darussalam	2005	Burns, <20% total burned surface area without lower airway burns	0.0163
Brunei Darussalam	2010	Burns, <20% total burned surface area without lower airway burns	0.0163
Brunei Darussalam	2017	Burns, <20% total burned surface area without lower airway burns	0.0163
Brunei Darussalam	1990	Burns, >=20% total burned surface area or >= 10% burned surface area if head/neck or hands/wrist involved w/o lower airway burns	0.1849
Brunei Darussalam	1995	Burns, >=20% total burned surface area or >= 10% burned surface area if head/neck or hands/wrist involved w/o lower airway burns	0.1716
Brunei Darussalam	2000	Burns, >=20% total burned surface area or >= 10% burned surface area if head/neck or hands/wrist involved w/o lower airway burns	0.1555
Brunei Darussalam	2005	Burns, >=20% total burned surface area or >= 10% burned surface area if head/neck or hands/wrist involved w/o lower airway burns	0.1415
Brunei Darussalam	2010	Burns, >=20% total burned surface area or >= 10% burned surface area if head/neck or hands/wrist involved w/o lower airway burns	0.1346
Brunei Darussalam	2017	Burns, >=20% total burned surface area or >= 10% burned surface area if head/neck or hands/wrist involved w/o lower airway burns	0.1346
Republic of Korea	1990	Amputation of lower limbs, bilateral	0.1752
Republic of Korea	1995	Amputation of lower limbs, bilateral	0.1374
Republic of Korea	2000	Amputation of lower limbs, bilateral	0.1241
Republic of Korea	2005	Amputation of lower limbs, bilateral	0.1232
Republic of Korea	2010	Amputation of lower limbs, bilateral	0.1232
Republic of Korea	2017	Amputation of lower limbs, bilateral	0.1232
Republic of Korea	1990	Lower airway burns	0.2252
Republic of Korea	1995	Lower airway burns	0.2252
Republic of Korea	2000	Lower airway burns	0.2252
Republic of Korea	2005	Lower airway burns	0.2252
Republic of Korea	2010	Lower airway burns	0.2252
Republic of Korea	2017	Lower airway burns	0.2252
Republic of Korea	1990	Dislocation of hip	0.0159
Republic of Korea	1995	Dislocation of hip	0.0159
Republic of Korea	2000	Dislocation of hip	0.0159
Republic of Korea	2005	Dislocation of hip	0.0159
Republic of Korea	2010	Dislocation of hip	0.0159
Republic of Korea	2017	Dislocation of hip	0.0159
Republic of Korea	1990	Dislocation of knee	0.1134
Republic of Korea	1995	Dislocation of knee	0.1134
Republic of Korea	2000	Dislocation of knee	0.1134
Republic of Korea	2005	Dislocation of knee	0.1134
Republic of Korea	2010	Dislocation of knee	0.1134
Republic of Korea	2017	Dislocation of knee	0.1134
Republic of Korea	1990	Dislocation of shoulder	0.062
Republic of Korea	1995	Dislocation of shoulder	0.062
Republic of Korea	2000	Dislocation of shoulder	0.062
Republic of Korea	2005	Dislocation of shoulder	0.062
Republic of Korea	2010	Dislocation of shoulder	0.062
Republic of Korea	2017	Dislocation of shoulder	0.062
Republic of Korea	1990	Muscle and tendon injuries, including sprains and strains lesser dislocations	0.0075
Republic of Korea	1995	Muscle and tendon injuries, including sprains and strains lesser dislocations	0.0075
Republic of Korea	2000	Muscle and tendon injuries, including sprains and strains lesser dislocations	0.0075
Republic of Korea	2005	Muscle and tendon injuries, including sprains and strains lesser dislocations	0.0075
Republic of Korea	2010	Muscle and tendon injuries, including sprains and strains lesser dislocations	0.0075
Republic of Korea	2017	Muscle and tendon injuries, including sprains and strains lesser dislocations	0.0075
Republic of Korea	1990	Fracture of clavicle, scapula, or humerus	0.0349
Republic of Korea	1995	Fracture of clavicle, scapula, or humerus	0.0349
Republic of Korea	2000	Fracture of clavicle, scapula, or humerus	0.0349
Republic of Korea	2005	Fracture of clavicle, scapula, or humerus	0.0349
Republic of Korea	2010	Fracture of clavicle, scapula, or humerus	0.0349
Republic of Korea	2017	Fracture of clavicle, scapula, or humerus	0.0349
Republic of Korea	1990	Fracture of face bones	0.0669
Republic of Korea	1995	Fracture of face bones	0.0669
Republic of Korea	2000	Fracture of face bones	0.0669
Republic of Korea	2005	Fracture of face bones	0.0669
Republic of Korea	2010	Fracture of face bones	0.0669
Republic of Korea	2017	Fracture of face bones	0.0669
Republic of Korea	1990	Fracture of foot bones except ankle	0.0264
Republic of Korea	1995	Fracture of foot bones except ankle	0.0264
Republic of Korea	2000	Fracture of foot bones except ankle	0.0264
Republic of Korea	2005	Fracture of foot bones except ankle	0.0264
Republic of Korea	2010	Fracture of foot bones except ankle	0.0264
Republic of Korea	2017	Fracture of foot bones except ankle	0.0264
Republic of Korea	1990	Fracture of hand (wrist and other distal part of hand)	0.0138
Republic of Korea	1995	Fracture of hand (wrist and other distal part of hand)	0.0138
Republic of Korea	2000	Fracture of hand (wrist and other distal part of hand)	0.0138
Republic of Korea	2005	Fracture of hand (wrist and other distal part of hand)	0.0138
Republic of Korea	2010	Fracture of hand (wrist and other distal part of hand)	0.0138
Republic of Korea	2017	Fracture of hand (wrist and other distal part of hand)	0.0138
Republic of Korea	1990	Fracture of hip	0.1263
Republic of Korea	1995	Fracture of hip	0.0765
Republic of Korea	2000	Fracture of hip	0.0588
Republic of Korea	2005	Fracture of hip	0.0577
Republic of Korea	2010	Fracture of hip	0.0577
Republic of Korea	2017	Fracture of hip	0.0577
Republic of Korea	1990	Amputation of upper limbs, bilateral	0.1585
Republic of Korea	1995	Amputation of upper limbs, bilateral	0.107
Republic of Korea	2000	Amputation of upper limbs, bilateral	0.0888
Republic of Korea	2005	Amputation of upper limbs, bilateral	0.0876
Republic of Korea	2010	Amputation of upper limbs, bilateral	0.0876
Republic of Korea	2017	Amputation of upper limbs, bilateral	0.0876
Republic of Korea	1990	Fracture of patella, tibia or fibula, or ankle	0.0554
Republic of Korea	1995	Fracture of patella, tibia or fibula, or ankle	0.0554
Republic of Korea	2000	Fracture of patella, tibia or fibula, or ankle	0.0554
Republic of Korea	2005	Fracture of patella, tibia or fibula, or ankle	0.0554
Republic of Korea	2010	Fracture of patella, tibia or fibula, or ankle	0.0554
Republic of Korea	2017	Fracture of patella, tibia or fibula, or ankle	0.0554
Republic of Korea	1990	Fracture of pelvis	0.1823
Republic of Korea	1995	Fracture of pelvis	0.1823
Republic of Korea	2000	Fracture of pelvis	0.1823
Republic of Korea	2005	Fracture of pelvis	0.1823
Republic of Korea	2010	Fracture of pelvis	0.1823
Republic of Korea	2017	Fracture of pelvis	0.1823
Republic of Korea	1990	Fracture of radius and/or ulna	0.0435
Republic of Korea	1995	Fracture of radius and/or ulna	0.0435
Republic of Korea	2000	Fracture of radius and/or ulna	0.0435
Republic of Korea	2005	Fracture of radius and/or ulna	0.0435
Republic of Korea	2010	Fracture of radius and/or ulna	0.0435
Republic of Korea	2017	Fracture of radius and/or ulna	0.0435
Republic of Korea	1990	Fracture of skull	0.0714
Republic of Korea	1995	Fracture of skull	0.0714
Republic of Korea	2000	Fracture of skull	0.0714
Republic of Korea	2005	Fracture of skull	0.0714
Republic of Korea	2010	Fracture of skull	0.0714
Republic of Korea	2017	Fracture of skull	0.0714
Republic of Korea	1990	Fracture of sternum and/or fracture of one or more ribs	0.1027
Republic of Korea	1995	Fracture of sternum and/or fracture of one or more ribs	0.1027
Republic of Korea	2000	Fracture of sternum and/or fracture of one or more ribs	0.1027
Republic of Korea	2005	Fracture of sternum and/or fracture of one or more ribs	0.1027
Republic of Korea	2010	Fracture of sternum and/or fracture of one or more ribs	0.1027
Republic of Korea	2017	Fracture of sternum and/or fracture of one or more ribs	0.1027
Republic of Korea	1990	Fracture of vertebral column	0.1106
Republic of Korea	1995	Fracture of vertebral column	0.1106
Republic of Korea	2000	Fracture of vertebral column	0.1106
Republic of Korea	2005	Fracture of vertebral column	0.1106
Republic of Korea	2010	Fracture of vertebral column	0.1106
Republic of Korea	2017	Fracture of vertebral column	0.1106
Republic of Korea	1990	Fracture of femur, other than femoral neck	0.042
Republic of Korea	1995	Fracture of femur, other than femoral neck	0.042
Republic of Korea	2000	Fracture of femur, other than femoral neck	0.042
Republic of Korea	2005	Fracture of femur, other than femoral neck	0.042
Republic of Korea	2010	Fracture of femur, other than femoral neck	0.042
Republic of Korea	2017	Fracture of femur, other than femoral neck	0.042
Republic of Korea	1990	Minor TBI	0.1319
Republic of Korea	1995	Minor TBI	0.1319
Republic of Korea	2000	Minor TBI	0.1319
Republic of Korea	2005	Minor TBI	0.1319
Republic of Korea	2010	Minor TBI	0.1319
Republic of Korea	2017	Minor TBI	0.1319
Republic of Korea	1990	Moderate TBI	0.1637
Republic of Korea	1995	Moderate TBI	0.1637
Republic of Korea	2000	Moderate TBI	0.1637
Republic of Korea	2005	Moderate TBI	0.1637
Republic of Korea	2010	Moderate TBI	0.1637
Republic of Korea	2017	Moderate TBI	0.1637
Republic of Korea	1990	Amputation of fingers (excluding thumb)	0.0047
Republic of Korea	1995	Amputation of fingers (excluding thumb)	0.0047
Republic of Korea	2000	Amputation of fingers (excluding thumb)	0.0047
Republic of Korea	2005	Amputation of fingers (excluding thumb)	0.0047
Republic of Korea	2010	Amputation of fingers (excluding thumb)	0.0047
Republic of Korea	2017	Amputation of fingers (excluding thumb)	0.0047
Republic of Korea	1990	Spinal cord lesion at neck level	0.4356
Republic of Korea	1995	Spinal cord lesion at neck level	0.4183
Republic of Korea	2000	Spinal cord lesion at neck level	0.4122
Republic of Korea	2005	Spinal cord lesion at neck level	0.4118
Republic of Korea	2010	Spinal cord lesion at neck level	0.4118
Republic of Korea	2017	Spinal cord lesion at neck level	0.4118
Republic of Korea	1990	Spinal cord lesion below neck level	0.2278
Republic of Korea	1995	Spinal cord lesion below neck level	0.1914
Republic of Korea	2000	Spinal cord lesion below neck level	0.1785
Republic of Korea	2005	Spinal cord lesion below neck level	0.1777
Republic of Korea	2010	Spinal cord lesion below neck level	0.1777
Republic of Korea	2017	Spinal cord lesion below neck level	0.1777
Republic of Korea	1990	Drowning and nonfatal submersion	0.2471
Republic of Korea	1995	Drowning and nonfatal submersion	0.2471
Republic of Korea	2000	Drowning and nonfatal submersion	0.2471
Republic of Korea	2005	Drowning and nonfatal submersion	0.2471
Republic of Korea	2010	Drowning and nonfatal submersion	0.2471
Republic of Korea	2017	Drowning and nonfatal submersion	0.2471
Republic of Korea	1990	Asphyxiation	0.2471
Republic of Korea	1995	Asphyxiation	0.2471
Republic of Korea	2000	Asphyxiation	0.2471
Republic of Korea	2005	Asphyxiation	0.2471
Republic of Korea	2010	Asphyxiation	0.2471
Republic of Korea	2017	Asphyxiation	0.2471
Republic of Korea	1990	Crush injury	0.1325
Republic of Korea	1995	Crush injury	0.1325
Republic of Korea	2000	Crush injury	0.1325
Republic of Korea	2005	Crush injury	0.1325
Republic of Korea	2010	Crush injury	0.1325
Republic of Korea	2017	Crush injury	0.1325
Republic of Korea	1990	Nerve injury	0.1129
Republic of Korea	1995	Nerve injury	0.1129
Republic of Korea	2000	Nerve injury	0.1129
Republic of Korea	2005	Nerve injury	0.1129
Republic of Korea	2010	Nerve injury	0.1129
Republic of Korea	2017	Nerve injury	0.1129
Republic of Korea	1990	Injury to eyes	0.0165
Republic of Korea	1995	Injury to eyes	0.0165
Republic of Korea	2000	Injury to eyes	0.0165
Republic of Korea	2005	Injury to eyes	0.0165
Republic of Korea	2010	Injury to eyes	0.0165
Republic of Korea	2017	Injury to eyes	0.0165
Republic of Korea	1990	Amputation of lower limb, unilateral	0.0657
Republic of Korea	1995	Amputation of lower limb, unilateral	0.0463
Republic of Korea	2000	Amputation of lower limb, unilateral	0.0394
Republic of Korea	2005	Amputation of lower limb, unilateral	0.039
Republic of Korea	2010	Amputation of lower limb, unilateral	0.039
Republic of Korea	2017	Amputation of lower limb, unilateral	0.039
Republic of Korea	1990	Open wound(s)	0.011
Republic of Korea	1995	Open wound(s)	0.011
Republic of Korea	2000	Open wound(s)	0.011
Republic of Korea	2005	Open wound(s)	0.011
Republic of Korea	2010	Open wound(s)	0.011
Republic of Korea	2017	Open wound(s)	0.011
Republic of Korea	1990	Poisoning requiring urgent care	0.1628
Republic of Korea	1995	Poisoning requiring urgent care	0.1628
Republic of Korea	2000	Poisoning requiring urgent care	0.1628
Republic of Korea	2005	Poisoning requiring urgent care	0.1628
Republic of Korea	2010	Poisoning requiring urgent care	0.1628
Republic of Korea	2017	Poisoning requiring urgent care	0.1628
Republic of Korea	1990	Severe chest injury	0.0474
Republic of Korea	1995	Severe chest injury	0.0474
Republic of Korea	2000	Severe chest injury	0.0474
Republic of Korea	2005	Severe chest injury	0.0474
Republic of Korea	2010	Severe chest injury	0.0474
Republic of Korea	2017	Severe chest injury	0.0474
Republic of Korea	1990	Internal hemorrhage in abdomen and pelvis	0.1143
Republic of Korea	1995	Internal hemorrhage in abdomen and pelvis	0.1143
Republic of Korea	2000	Internal hemorrhage in abdomen and pelvis	0.1143
Republic of Korea	2005	Internal hemorrhage in abdomen and pelvis	0.1143
Republic of Korea	2010	Internal hemorrhage in abdomen and pelvis	0.1143
Republic of Korea	2017	Internal hemorrhage in abdomen and pelvis	0.1143
Republic of Korea	1990	Contusion in any part of the body	0.0075
Republic of Korea	1995	Contusion in any part of the body	0.0075
Republic of Korea	2000	Contusion in any part of the body	0.0075
Republic of Korea	2005	Contusion in any part of the body	0.0075
Republic of Korea	2010	Contusion in any part of the body	0.0075
Republic of Korea	2017	Contusion in any part of the body	0.0075
Republic of Korea	1990	Effect of different environmental factors	0.049
Republic of Korea	1995	Effect of different environmental factors	0.049
Republic of Korea	2000	Effect of different environmental factors	0.049
Republic of Korea	2005	Effect of different environmental factors	0.049
Republic of Korea	2010	Effect of different environmental factors	0.049
Republic of Korea	2017	Effect of different environmental factors	0.049
Republic of Korea	1990	Complications following therapeutic procedures	0.0506
Republic of Korea	1995	Complications following therapeutic procedures	0.0506
Republic of Korea	2000	Complications following therapeutic procedures	0.0506
Republic of Korea	2005	Complications following therapeutic procedures	0.0506
Republic of Korea	2010	Complications following therapeutic procedures	0.0506
Republic of Korea	2017	Complications following therapeutic procedures	0.0506
Republic of Korea	1990	Multiple fractures, dislocations, crashes, wounds, sprains, and strains	0.1263
Republic of Korea	1995	Multiple fractures, dislocations, crashes, wounds, sprains, and strains	0.0765
Republic of Korea	2000	Multiple fractures, dislocations, crashes, wounds, sprains, and strains	0.0588
Republic of Korea	2005	Multiple fractures, dislocations, crashes, wounds, sprains, and strains	0.0577
Republic of Korea	2010	Multiple fractures, dislocations, crashes, wounds, sprains, and strains	0.0577
Republic of Korea	2017	Multiple fractures, dislocations, crashes, wounds, sprains, and strains	0.0577
Republic of Korea	1990	Amputation of upper limb, unilateral	0.0547
Republic of Korea	1995	Amputation of upper limb, unilateral	0.0433
Republic of Korea	2000	Amputation of upper limb, unilateral	0.0392
Republic of Korea	2005	Amputation of upper limb, unilateral	0.039
Republic of Korea	2010	Amputation of upper limb, unilateral	0.039
Republic of Korea	2017	Amputation of upper limb, unilateral	0.039
Republic of Korea	1990	Amputation of thumb	0.0114
Republic of Korea	1995	Amputation of thumb	0.0114
Republic of Korea	2000	Amputation of thumb	0.0114
Republic of Korea	2005	Amputation of thumb	0.0114
Republic of Korea	2010	Amputation of thumb	0.0114
Republic of Korea	2017	Amputation of thumb	0.0114
Republic of Korea	1990	Amputation of toe/toes	0.0059
Republic of Korea	1995	Amputation of toe/toes	0.0059
Republic of Korea	2000	Amputation of toe/toes	0.0059
Republic of Korea	2005	Amputation of toe/toes	0.0059
Republic of Korea	2010	Amputation of toe/toes	0.0059
Republic of Korea	2017	Amputation of toe/toes	0.0059
Republic of Korea	1990	Burns, <20% total burned surface area without lower airway burns	0.0163
Republic of Korea	1995	Burns, <20% total burned surface area without lower airway burns	0.0163
Republic of Korea	2000	Burns, <20% total burned surface area without lower airway burns	0.0163
Republic of Korea	2005	Burns, <20% total burned surface area without lower airway burns	0.0163
Republic of Korea	2010	Burns, <20% total burned surface area without lower airway burns	0.0163
Republic of Korea	2017	Burns, <20% total burned surface area without lower airway burns	0.0163
Republic of Korea	1990	Burns, >=20% total burned surface area or >= 10% burned surface area if head/neck or hands/wrist involved w/o lower airway burns	0.1985
Republic of Korea	1995	Burns, >=20% total burned surface area or >= 10% burned surface area if head/neck or hands/wrist involved w/o lower airway burns	0.1521
Republic of Korea	2000	Burns, >=20% total burned surface area or >= 10% burned surface area if head/neck or hands/wrist involved w/o lower airway burns	0.1356
Republic of Korea	2005	Burns, >=20% total burned surface area or >= 10% burned surface area if head/neck or hands/wrist involved w/o lower airway burns	0.1346
Republic of Korea	2010	Burns, >=20% total burned surface area or >= 10% burned surface area if head/neck or hands/wrist involved w/o lower airway burns	0.1346
Republic of Korea	2017	Burns, >=20% total burned surface area or >= 10% burned surface area if head/neck or hands/wrist involved w/o lower airway burns	0.1346
Singapore	1990	Amputation of lower limbs, bilateral	0.1427
Singapore	1995	Amputation of lower limbs, bilateral	0.125
Singapore	2000	Amputation of lower limbs, bilateral	0.1232
Singapore	2005	Amputation of lower limbs, bilateral	0.1232
Singapore	2010	Amputation of lower limbs, bilateral	0.1232
Singapore	2017	Amputation of lower limbs, bilateral	0.1232
Singapore	1990	Lower airway burns	0.2252
Singapore	1995	Lower airway burns	0.2252
Singapore	2000	Lower airway burns	0.2252
Singapore	2005	Lower airway burns	0.2252
Singapore	2010	Lower airway burns	0.2252
Singapore	2017	Lower airway burns	0.2252
Singapore	1990	Dislocation of hip	0.0159
Singapore	1995	Dislocation of hip	0.0159
Singapore	2000	Dislocation of hip	0.0159
Singapore	2005	Dislocation of hip	0.0159
Singapore	2010	Dislocation of hip	0.0159
Singapore	2017	Dislocation of hip	0.0159
Singapore	1990	Dislocation of knee	0.1134
Singapore	1995	Dislocation of knee	0.1134
Singapore	2000	Dislocation of knee	0.1134
Singapore	2005	Dislocation of knee	0.1134
Singapore	2010	Dislocation of knee	0.1134
Singapore	2017	Dislocation of knee	0.1134
Singapore	1990	Dislocation of shoulder	0.062
Singapore	1995	Dislocation of shoulder	0.062
Singapore	2000	Dislocation of shoulder	0.062
Singapore	2005	Dislocation of shoulder	0.062
Singapore	2010	Dislocation of shoulder	0.062

Singapore	2017	Dislocation of shoulder	0.062
Singapore	1990	Muscle and tendon injuries, including sprains and strains lesser dislocations	0.0075
Singapore	1995	Muscle and tendon injuries, including sprains and strains lesser dislocations	0.0075
Singapore	2000	Muscle and tendon injuries, including sprains and strains lesser dislocations	0.0075
Singapore	2005	Muscle and tendon injuries, including sprains and strains lesser dislocations	0.0075
Singapore	2010	Muscle and tendon injuries, including sprains and strains lesser dislocations	0.0075
Singapore	2017	Muscle and tendon injuries, including sprains and strains lesser dislocations	0.0075
Singapore	1990	Fracture of clavicle, scapula, or humerus	0.0349
Singapore	1995	Fracture of clavicle, scapula, or humerus	0.0349
Singapore	2000	Fracture of clavicle, scapula, or humerus	0.0349
Singapore	2005	Fracture of clavicle, scapula, or humerus	0.0349
Singapore	2010	Fracture of clavicle, scapula, or humerus	0.0349
Singapore	2017	Fracture of clavicle, scapula, or humerus	0.0349
Singapore	1990	Fracture of face bones	0.0669
Singapore	1995	Fracture of face bones	0.0669
Singapore	2000	Fracture of face bones	0.0669
Singapore	2005	Fracture of face bones	0.0669
Singapore	2010	Fracture of face bones	0.0669
Singapore	2017	Fracture of face bones	0.0669
Singapore	1990	Fracture of foot bones except ankle	0.0264
Singapore	1995	Fracture of foot bones except ankle	0.0264
Singapore	2000	Fracture of foot bones except ankle	0.0264
Singapore	2005	Fracture of foot bones except ankle	0.0264
Singapore	2010	Fracture of foot bones except ankle	0.0264
Singapore	2017	Fracture of foot bones except ankle	0.0264
Singapore	1990	Fracture of hand (wrist and other distal part of hand)	0.0138
Singapore	1995	Fracture of hand (wrist and other distal part of hand)	0.0138
Singapore	2000	Fracture of hand (wrist and other distal part of hand)	0.0138
Singapore	2005	Fracture of hand (wrist and other distal part of hand)	0.0138
Singapore	2010	Fracture of hand (wrist and other distal part of hand)	0.0138
Singapore	2017	Fracture of hand (wrist and other distal part of hand)	0.0138
Singapore	1990	Fracture of hip	0.0834
Singapore	1995	Fracture of hip	0.06
Singapore	2000	Fracture of hip	0.0577
Singapore	2005	Fracture of hip	0.0577
Singapore	2010	Fracture of hip	0.0577
Singapore	2017	Fracture of hip	0.0577
Singapore	1990	Amputation of upper limbs, bilateral	0.1142
Singapore	1995	Amputation of upper limbs, bilateral	0.0901
Singapore	2000	Amputation of upper limbs, bilateral	0.0876
Singapore	2005	Amputation of upper limbs, bilateral	0.0876
Singapore	2010	Amputation of upper limbs, bilateral	0.0876
Singapore	2017	Amputation of upper limbs, bilateral	0.0876
Singapore	1990	Fracture of patella, tibia or fibula, or ankle	0.0554
Singapore	1995	Fracture of patella, tibia or fibula, or ankle	0.0554
Singapore	2000	Fracture of patella, tibia or fibula, or ankle	0.0554
Singapore	2005	Fracture of patella, tibia or fibula, or ankle	0.0554
Singapore	2010	Fracture of patella, tibia or fibula, or ankle	0.0554
Singapore	2017	Fracture of patella, tibia or fibula, or ankle	0.0554
Singapore	1990	Fracture of pelvis	0.1823
Singapore	1995	Fracture of pelvis	0.1823
Singapore	2000	Fracture of pelvis	0.1823
Singapore	2005	Fracture of pelvis	0.1823
Singapore	2010	Fracture of pelvis	0.1823
Singapore	2017	Fracture of pelvis	0.1823
Singapore	1990	Fracture of radius and/or ulna	0.0435
Singapore	1995	Fracture of radius and/or ulna	0.0435
Singapore	2000	Fracture of radius and/or ulna	0.0435
Singapore	2005	Fracture of radius and/or ulna	0.0435
Singapore	2010	Fracture of radius and/or ulna	0.0435
Singapore	2017	Fracture of radius and/or ulna	0.0435
Singapore	1990	Fracture of skull	0.0714
Singapore	1995	Fracture of skull	0.0714
Singapore	2000	Fracture of skull	0.0714
Singapore	2005	Fracture of skull	0.0714
Singapore	2010	Fracture of skull	0.0714
Singapore	2017	Fracture of skull	0.0714
Singapore	1990	Fracture of sternum and/or fracture of one or more ribs	0.1027
Singapore	1995	Fracture of sternum and/or fracture of one or more ribs	0.1027
Singapore	2000	Fracture of sternum and/or fracture of one or more ribs	0.1027
Singapore	2005	Fracture of sternum and/or fracture of one or more ribs	0.1027
Singapore	2010	Fracture of sternum and/or fracture of one or more ribs	0.1027
Singapore	2017	Fracture of sternum and/or fracture of one or more ribs	0.1027
Singapore	1990	Fracture of vertebral column	0.1106
Singapore	1995	Fracture of vertebral column	0.1106
Singapore	2000	Fracture of vertebral column	0.1106
Singapore	2005	Fracture of vertebral column	0.1106
Singapore	2010	Fracture of vertebral column	0.1106
Singapore	2017	Fracture of vertebral column	0.1106
Singapore	1990	Fracture of femur, other than femoral neck	0.042
Singapore	1995	Fracture of femur, other than femoral neck	0.042
Singapore	2000	Fracture of femur, other than femoral neck	0.042
Singapore	2005	Fracture of femur, other than femoral neck	0.042
Singapore	2010	Fracture of femur, other than femoral neck	0.042
Singapore	2017	Fracture of femur, other than femoral neck	0.042
Singapore	1990	Minor TBI	0.1319
Singapore	1995	Minor TBI	0.1319
Singapore	2000	Minor TBI	0.1319
Singapore	2005	Minor TBI	0.1319
Singapore	2010	Minor TBI	0.1319
Singapore	2017	Minor TBI	0.1319
Singapore	1990	Moderate TBI	0.1637
Singapore	1995	Moderate TBI	0.1637
Singapore	2000	Moderate TBI	0.1637
Singapore	2005	Moderate TBI	0.1637
Singapore	2010	Moderate TBI	0.1637
Singapore	2017	Moderate TBI	0.1637
Singapore	1990	Amputation of fingers (excluding thumb)	0.0047
Singapore	1995	Amputation of fingers (excluding thumb)	0.0047
Singapore	2000	Amputation of fingers (excluding thumb)	0.0047
Singapore	2005	Amputation of fingers (excluding thumb)	0.0047
Singapore	2010	Amputation of fingers (excluding thumb)	0.0047
Singapore	2017	Amputation of fingers (excluding thumb)	0.0047
Singapore	1990	Spinal cord lesion at neck level	0.4207
Singapore	1995	Spinal cord lesion at neck level	0.4126
Singapore	2000	Spinal cord lesion at neck level	0.4118
Singapore	2005	Spinal cord lesion at neck level	0.4118
Singapore	2010	Spinal cord lesion at neck level	0.4118
Singapore	2017	Spinal cord lesion at neck level	0.4118
Singapore	1990	Spinal cord lesion below neck level	0.1965
Singapore	1995	Spinal cord lesion below neck level	0.1794
Singapore	2000	Spinal cord lesion below neck level	0.1777
Singapore	2005	Spinal cord lesion below neck level	0.1777
Singapore	2010	Spinal cord lesion below neck level	0.1777
Singapore	2017	Spinal cord lesion below neck level	0.1777
Singapore	1990	Drowning and nonfatal submersion	0.2471
Singapore	1995	Drowning and nonfatal submersion	0.2471
Singapore	2000	Drowning and nonfatal submersion	0.2471
Singapore	2005	Drowning and nonfatal submersion	0.2471
Singapore	2010	Drowning and nonfatal submersion	0.2471
Singapore	2017	Drowning and nonfatal submersion	0.2471
Singapore	1990	Asphyxiation	0.2471
Singapore	1995	Asphyxiation	0.2471
Singapore	2000	Asphyxiation	0.2471
Singapore	2005	Asphyxiation	0.2471
Singapore	2010	Asphyxiation	0.2471
Singapore	2017	Asphyxiation	0.2471
Singapore	1990	Crush injury	0.1325
Singapore	1995	Crush injury	0.1325
Singapore	2000	Crush injury	0.1325
Singapore	2005	Crush injury	0.1325
Singapore	2010	Crush injury	0.1325
Singapore	2017	Crush injury	0.1325
Singapore	1990	Nerve injury	0.1129
Singapore	1995	Nerve injury	0.1129
Singapore	2000	Nerve injury	0.1129
Singapore	2005	Nerve injury	0.1129
Singapore	2010	Nerve injury	0.1129
Singapore	2017	Nerve injury	0.1129
Singapore	1990	Injury to eyes	0.0165
Singapore	1995	Injury to eyes	0.0165
Singapore	2000	Injury to eyes	0.0165
Singapore	2005	Injury to eyes	0.0165
Singapore	2010	Injury to eyes	0.0165
Singapore	2017	Injury to eyes	0.0165
Singapore	1990	Amputation of lower limb, unilateral	0.049
Singapore	1995	Amputation of lower limb, unilateral	0.0399
Singapore	2000	Amputation of lower limb, unilateral	0.039
Singapore	2005	Amputation of lower limb, unilateral	0.039
Singapore	2010	Amputation of lower limb, unilateral	0.039
Singapore	2017	Amputation of lower limb, unilateral	0.039
Singapore	1990	Open wound(s)	0.011
Singapore	1995	Open wound(s)	0.011
Singapore	2000	Open wound(s)	0.011
Singapore	2005	Open wound(s)	0.011
Singapore	2010	Open wound(s)	0.011
Singapore	2017	Open wound(s)	0.011
Singapore	1990	Poisoning requiring urgent care	0.1628
Singapore	1995	Poisoning requiring urgent care	0.1628
Singapore	2000	Poisoning requiring urgent care	0.1628
Singapore	2005	Poisoning requiring urgent care	0.1628
Singapore	2010	Poisoning requiring urgent care	0.1628
Singapore	2017	Poisoning requiring urgent care	0.1628
Singapore	1990	Severe chest injury	0.0474
Singapore	1995	Severe chest injury	0.0474
Singapore	2000	Severe chest injury	0.0474
Singapore	2005	Severe chest injury	0.0474
Singapore	2010	Severe chest injury	0.0474
Singapore	2017	Severe chest injury	0.0474
Singapore	1990	Internal hemorrhage in abdomen and pelvis	0.1143
Singapore	1995	Internal hemorrhage in abdomen and pelvis	0.1143
Singapore	2000	Internal hemorrhage in abdomen and pelvis	0.1143
Singapore	2005	Internal hemorrhage in abdomen and pelvis	0.1143
Singapore	2010	Internal hemorrhage in abdomen and pelvis	0.1143
Singapore	2017	Internal hemorrhage in abdomen and pelvis	0.1143
Singapore	1990	Contusion in any part of the body	0.0075
Singapore	1995	Contusion in any part of the body	0.0075
Singapore	2000	Contusion in any part of the body	0.0075
Singapore	2005	Contusion in any part of the body	0.0075
Singapore	2010	Contusion in any part of the body	0.0075
Singapore	2017	Contusion in any part of the body	0.0075
Singapore	1990	Effect of different environmental factors	0.049
Singapore	1995	Effect of different environmental factors	0.049
Singapore	2000	Effect of different environmental factors	0.049
Singapore	2005	Effect of different environmental factors	0.049
Singapore	2010	Effect of different environmental factors	0.049
Singapore	2017	Effect of different environmental factors	0.049
Singapore	1990	Complications following therapeutic procedures	0.0506
Singapore	1995	Complications following therapeutic procedures	0.0506
Singapore	2000	Complications following therapeutic procedures	0.0506
Singapore	2005	Complications following therapeutic procedures	0.0506
Singapore	2010	Complications following therapeutic procedures	0.0506
Singapore	2017	Complications following therapeutic procedures	0.0506
Singapore	1990	Multiple fractures, dislocations, crashes, wounds, sprains, and strains	0.0834
Singapore	1995	Multiple fractures, dislocations, crashes, wounds, sprains, and strains	0.06
Singapore	2000	Multiple fractures, dislocations, crashes, wounds, sprains, and strains	0.0577
Singapore	2005	Multiple fractures, dislocations, crashes, wounds, sprains, and strains	0.0577
Singapore	2010	Multiple fractures, dislocations, crashes, wounds, sprains, and strains	0.0577
Singapore	2017	Multiple fractures, dislocations, crashes, wounds, sprains, and strains	0.0577
Singapore	1990	Amputation of upper limb, unilateral	0.0449
Singapore	1995	Amputation of upper limb, unilateral	0.0395
Singapore	2000	Amputation of upper limb, unilateral	0.039
Singapore	2005	Amputation of upper limb, unilateral	0.039
Singapore	2010	Amputation of upper limb, unilateral	0.039
Singapore	2017	Amputation of upper limb, unilateral	0.039
Singapore	1990	Amputation of thumb	0.0114
Singapore	1995	Amputation of thumb	0.0114
Singapore	2000	Amputation of thumb	0.0114
Singapore	2005	Amputation of thumb	0.0114
Singapore	2010	Amputation of thumb	0.0114
Singapore	2017	Amputation of thumb	0.0114
Singapore	1990	Amputation of toe/toes	0.0059
Singapore	1995	Amputation of toe/toes	0.0059
Singapore	2000	Amputation of toe/toes	0.0059
Singapore	2005	Amputation of toe/toes	0.0059
Singapore	2010	Amputation of toe/toes	0.0059
Singapore	2017	Amputation of toe/toes	0.0059
Singapore	1990	Burns, <20% total burned surface area without lower airway burns	0.0163
Singapore	1995	Burns, <20% total burned surface area without lower airway burns	0.0163
Singapore	2000	Burns, <20% total burned surface area without lower airway burns	0.0163
Singapore	2005	Burns, <20% total burned surface area without lower airway burns	0.0163
Singapore	2010	Burns, <20% total burned surface area without lower airway burns	0.0163
Singapore	2017	Burns, <20% total burned surface area without lower airway burns	0.0163
Singapore	1990	Burns, >=20% total burned surface area or >= 10% burned surface area if head/neck or hands/wrist involved w/o lower airway burns	0.1586
Singapore	1995	Burns, >=20% total burned surface area or >= 10% burned surface area if head/neck or hands/wrist involved w/o lower airway burns	0.1368
Singapore	2000	Burns, >=20% total burned surface area or >= 10% burned surface area if head/neck or hands/wrist involved w/o lower airway burns	0.1346
Singapore	2005	Burns, >=20% total burned surface area or >= 10% burned surface area if head/neck or hands/wrist involved w/o lower airway burns	0.1346
Singapore	2010	Burns, >=20% total burned surface area or >= 10% burned surface area if head/neck or hands/wrist involved w/o lower airway burns	0.1346
Singapore	2017	Burns, >=20% total burned surface area or >= 10% burned surface area if head/neck or hands/wrist involved w/o lower airway burns	0.1346
Australia	1990	Amputation of lower limbs, bilateral	0.1232
Australia	1995	Amputation of lower limbs, bilateral	0.1232
Australia	2000	Amputation of lower limbs, bilateral	0.1232
Australia	2005	Amputation of lower limbs, bilateral	0.1232
Australia	2010	Amputation of lower limbs, bilateral	0.1232
Australia	2017	Amputation of lower limbs, bilateral	0.1232
Australia	1990	Lower airway burns	0.2252
Australia	1995	Lower airway burns	0.2252
Australia	2000	Lower airway burns	0.2252
Australia	2005	Lower airway burns	0.2252
Australia	2010	Lower airway burns	0.2252
Australia	2017	Lower airway burns	0.2252
Australia	1990	Dislocation of hip	0.0159
Australia	1995	Dislocation of hip	0.0159
Australia	2000	Dislocation of hip	0.0159
Australia	2005	Dislocation of hip	0.0159
Australia	2010	Dislocation of hip	0.0159
Australia	2017	Dislocation of hip	0.0159
Australia	1990	Dislocation of knee	0.1134
Australia	1995	Dislocation of knee	0.1134
Australia	2000	Dislocation of knee	0.1134
Australia	2005	Dislocation of knee	0.1134
Australia	2010	Dislocation of knee	0.1134
Australia	2017	Dislocation of knee	0.1134
Australia	1990	Dislocation of shoulder	0.062
Australia	1995	Dislocation of shoulder	0.062
Australia	2000	Dislocation of shoulder	0.062
Australia	2005	Dislocation of shoulder	0.062
Australia	2010	Dislocation of shoulder	0.062
Australia	2017	Dislocation of shoulder	0.062
Australia	1990	Muscle and tendon injuries, including sprains and strains lesser dislocations	0.0075
Australia	1995	Muscle and tendon injuries, including sprains and strains lesser dislocations	0.0075
Australia	2000	Muscle and tendon injuries, including sprains and strains lesser dislocations	0.0075
Australia	2005	Muscle and tendon injuries, including sprains and strains lesser dislocations	0.0075
Australia	2010	Muscle and tendon injuries, including sprains and strains lesser dislocations	0.0075
Australia	2017	Muscle and tendon injuries, including sprains and strains lesser dislocations	0.0075
Australia	1990	Fracture of clavicle, scapula, or humerus	0.0349
Australia	1995	Fracture of clavicle, scapula, or humerus	0.0349
Australia	2000	Fracture of clavicle, scapula, or humerus	0.0349
Australia	2005	Fracture of clavicle, scapula, or humerus	0.0349
Australia	2010	Fracture of clavicle, scapula, or humerus	0.0349
Australia	2017	Fracture of clavicle, scapula, or humerus	0.0349
Australia	1990	Fracture of face bones	0.0669
Australia	1995	Fracture of face bones	0.0669
Australia	2000	Fracture of face bones	0.0669
Australia	2005	Fracture of face bones	0.0669
Australia	2010	Fracture of face bones	0.0669
Australia	2017	Fracture of face bones	0.0669
Australia	1990	Fracture of foot bones except ankle	0.0264
Australia	1995	Fracture of foot bones except ankle	0.0264
Australia	2000	Fracture of foot bones except ankle	0.0264
Australia	2005	Fracture of foot bones except ankle	0.0264
Australia	2010	Fracture of foot bones except ankle	0.0264
Australia	2017	Fracture of foot bones except ankle	0.0264
Australia	1990	Fracture of hand (wrist and other distal part of hand)	0.0138
Australia	1995	Fracture of hand (wrist and other distal part of hand)	0.0138
Australia	2000	Fracture of hand (wrist and other distal part of hand)	0.0138
Australia	2005	Fracture of hand (wrist and other distal part of hand)	0.0138
Australia	2010	Fracture of hand (wrist and other distal part of hand)	0.0138
Australia	2017	Fracture of hand (wrist and other distal part of hand)	0.0138
Australia	1990	Fracture of hip	0.0577
Australia	1995	Fracture of hip	0.0577
Australia	2000	Fracture of hip	0.0577
Australia	2005	Fracture of hip	0.0577
Australia	2010	Fracture of hip	0.0577
Australia	2017	Fracture of hip	0.0577
Australia	1990	Amputation of upper limbs, bilateral	0.0876
Australia	1995	Amputation of upper limbs, bilateral	0.0876
Australia	2000	Amputation of upper limbs, bilateral	0.0876
Australia	2005	Amputation of upper limbs, bilateral	0.0876
Australia	2010	Amputation of upper limbs, bilateral	0.0876
Australia	2017	Amputation of upper limbs, bilateral	0.0876
Australia	1990	Fracture of patella, tibia or fibula, or ankle	0.0554
Australia	1995	Fracture of patella, tibia or fibula, or ankle	0.0554
Australia	2000	Fracture of patella, tibia or fibula, or ankle	0.0554
Australia	2005	Fracture of patella, tibia or fibula, or ankle	0.0554
Australia	2010	Fracture of patella, tibia or fibula, or ankle	0.0554
Australia	2017	Fracture of patella, tibia or fibula, or ankle	0.0554
Australia	1990	Fracture of pelvis	0.1823
Australia	1995	Fracture of pelvis	0.1823
Australia	2000	Fracture of pelvis	0.1823
Australia	2005	Fracture of pelvis	0.1823
Australia	2010	Fracture of pelvis	0.1823
Australia	2017	Fracture of pelvis	0.1823
Australia	1990	Fracture of radius and/or ulna	0.0435
Australia	1995	Fracture of radius and/or ulna	0.0435
Australia	2000	Fracture of radius and/or ulna	0.0435
Australia	2005	Fracture of radius and/or ulna	0.0435
Australia	2010	Fracture of radius and/or ulna	0.0435
Australia	2017	Fracture of radius and/or ulna	0.0435
Australia	1990	Fracture of skull	0.0714
Australia	1995	Fracture of skull	0.0714
Australia	2000	Fracture of skull	0.0714
Australia	2005	Fracture of skull	0.0714
Australia	2010	Fracture of skull	0.0714
Australia	2017	Fracture of skull	0.0714
Australia	1990	Fracture of sternum and/or fracture of one or more ribs	0.1027
Australia	1995	Fracture of sternum and/or fracture of one or more ribs	0.1027
Australia	2000	Fracture of sternum and/or fracture of one or more ribs	0.1027
Australia	2005	Fracture of sternum and/or fracture of one or more ribs	0.1027
Australia	2010	Fracture of sternum and/or fracture of one or more ribs	0.1027
Australia	2017	Fracture of sternum and/or fracture of one or more ribs	0.1027
Australia	1990	Fracture of vertebral column	0.1106
Australia	1995	Fracture of vertebral column	0.1106
Australia	2000	Fracture of vertebral column	0.1106
Australia	2005	Fracture of vertebral column	0.1106
Australia	2010	Fracture of vertebral column	0.1106
Australia	2017	Fracture of vertebral column	0.1106
Australia	1990	Fracture of femur, other than femoral neck	0.042
Australia	1995	Fracture of femur, other than femoral neck	0.042
Australia	2000	Fracture of femur, other than femoral neck	0.042
Australia	2005	Fracture of femur, other than femoral neck	0.042
Australia	2010	Fracture of femur, other than femoral neck	0.042
Australia	2017	Fracture of femur, other than femoral neck	0.042
Australia	1990	Minor TBI	0.1319
Australia	1995	Minor TBI	0.1319
Australia	2000	Minor TBI	0.1319
Australia	2005	Minor TBI	0.1319
Australia	2010	Minor TBI	0.1319
Australia	2017	Minor TBI	0.1319
Australia	1990	Moderate TBI	0.1637
Australia	1995	Moderate TBI	0.1637
Australia	2000	Moderate TBI	0.1637
Australia	2005	Moderate TBI	0.1637
Australia	2010	Moderate TBI	0.1637
Australia	2017	Moderate TBI	0.1637
Australia	1990	Amputation of fingers (excluding thumb)	0.0047
Australia	1995	Amputation of fingers (excluding thumb)	0.0047
Australia	2000	Amputation of fingers (excluding thumb)	0.0047
Australia	2005	Amputation of fingers (excluding thumb)	0.0047
Australia	2010	Amputation of fingers (excluding thumb)	0.0047
Australia	2017	Amputation of fingers (excluding thumb)	0.0047
Australia	1990	Spinal cord lesion at neck level	0.4118
Australia	1995	Spinal cord lesion at neck level	0.4118
Australia	2000	Spinal cord lesion at neck level	0.4118
Australia	2005	Spinal cord lesion at neck level	0.4118
Australia	2010	Spinal cord lesion at neck level	0.4118
Australia	2017	Spinal cord lesion at neck level	0.4118
Australia	1990	Spinal cord lesion below neck level	0.1777
Australia	1995	Spinal cord lesion below neck level	0.1777
Australia	2000	Spinal cord lesion below neck level	0.1777
Australia	2005	Spinal cord lesion below neck level	0.1777
Australia	2010	Spinal cord lesion below neck level	0.1777
Australia	2017	Spinal cord lesion below neck level	0.1777
Australia	1990	Drowning and nonfatal submersion	0.2471
Australia	1995	Drowning and nonfatal submersion	0.2471
Australia	2000	Drowning and nonfatal submersion	0.2471
Australia	2005	Drowning and nonfatal submersion	0.2471
Australia	2010	Drowning and nonfatal submersion	0.2471
Australia	2017	Drowning and nonfatal submersion	0.2471
Australia	1990	Asphyxiation	0.2471
Australia	1995	Asphyxiation	0.2471
Australia	2000	Asphyxiation	0.2471
Australia	2005	Asphyxiation	0.2471
Australia	2010	Asphyxiation	0.2471
Australia	2017	Asphyxiation	0.2471
Australia	1990	Crush injury	0.1325
Australia	1995	Crush injury	0.1325
Australia	2000	Crush injury	0.1325
Australia	2005	Crush injury	0.1325
Australia	2010	Crush injury	0.1325
Australia	2017	Crush injury	0.1325
Australia	1990	Nerve injury	0.1129
Australia	1995	Nerve injury	0.1129
Australia	2000	Nerve injury	0.1129
Australia	2005	Nerve injury	0.1129
Australia	2010	Nerve injury	0.1129
Australia	2017	Nerve injury	0.1129
Australia	1990	Injury to eyes	0.0165
Australia	1995	Injury to eyes	0.0165
Australia	2000	Injury to eyes	0.0165
Australia	2005	Injury to eyes	0.0165
Australia	2010	Injury to eyes	0.0165
Australia	2017	Injury to eyes	0.0165
Australia	1990	Amputation of lower limb, unilateral	0.039
Australia	1995	Amputation of lower limb, unilateral	0.039
Australia	2000	Amputation of lower limb, unilateral	0.039
Australia	2005	Amputation of lower limb, unilateral	0.039
Australia	2010	Amputation of lower limb, unilateral	0.039
Australia	2017	Amputation of lower limb, unilateral	0.039
Australia	1990	Open wound(s)	0.011
Australia	1995	Open wound(s)	0.011
Australia	2000	Open wound(s)	0.011
Australia	2005	Open wound(s)	0.011
Australia	2010	Open wound(s)	0.011
Australia	2017	Open wound(s)	0.011
Australia	1990	Poisoning requiring urgent care	0.1628
Australia	1995	Poisoning requiring urgent care	0.1628
Australia	2000	Poisoning requiring urgent care	0.1628
Australia	2005	Poisoning requiring urgent care	0.1628
Australia	2010	Poisoning requiring urgent care	0.1628
Australia	2017	Poisoning requiring urgent care	0.1628
Australia	1990	Severe chest injury	0.0474
Australia	1995	Severe chest injury	0.0474
Australia	2000	Severe chest injury	0.0474
Australia	2005	Severe chest injury	0.0474
Australia	2010	Severe chest injury	0.0474
Australia	2017	Severe chest injury	0.0474
Australia	1990	Internal hemorrhage in abdomen and pelvis	0.1143
Australia	1995	Internal hemorrhage in abdomen and pelvis	0.1143
Australia	2000	Internal hemorrhage in abdomen and pelvis	0.1143
Australia	2005	Internal hemorrhage in abdomen and pelvis	0.1143

Australia	2010	Internal hemorrhage in abdomen and pelvis	0.1143
Australia	2017	Internal hemorrhage in abdomen and pelvis	0.1143
Australia	1990	Contusion in any part of the body	0.0075
Australia	1995	Contusion in any part of the body	0.0075
Australia	2000	Contusion in any part of the body	0.0075
Australia	2005	Contusion in any part of the body	0.0075
Australia	2010	Contusion in any part of the body	0.0075
Australia	2017	Contusion in any part of the body	0.0075
Australia	1990	Effect of different environmental factors	0.049
Australia	1995	Effect of different environmental factors	0.049
Australia	2000	Effect of different environmental factors	0.049
Australia	2005	Effect of different environmental factors	0.049
Australia	2010	Effect of different environmental factors	0.049
Australia	2017	Effect of different environmental factors	0.049
Australia	1990	Complications following therapeutic procedures	0.0506
Australia	1995	Complications following therapeutic procedures	0.0506
Australia	2000	Complications following therapeutic procedures	0.0506
Australia	2005	Complications following therapeutic procedures	0.0506
Australia	2010	Complications following therapeutic procedures	0.0506
Australia	2017	Complications following therapeutic procedures	0.0506
Australia	1990	Multiple fractures, dislocations, crashes, wounds, sprains, and strains	0.0577
Australia	1995	Multiple fractures, dislocations, crashes, wounds, sprains, and strains	0.0577
Australia	2000	Multiple fractures, dislocations, crashes, wounds, sprains, and strains	0.0577
Australia	2005	Multiple fractures, dislocations, crashes, wounds, sprains, and strains	0.0577
Australia	2010	Multiple fractures, dislocations, crashes, wounds, sprains, and strains	0.0577
Australia	2017	Multiple fractures, dislocations, crashes, wounds, sprains, and strains	0.0577
Australia	1990	Amputation of upper limb, unilateral	0.039
Australia	1995	Amputation of upper limb, unilateral	0.039
Australia	2000	Amputation of upper limb, unilateral	0.039
Australia	2005	Amputation of upper limb, unilateral	0.039
Australia	2010	Amputation of upper limb, unilateral	0.039
Australia	2017	Amputation of upper limb, unilateral	0.039
Australia	1990	Amputation of thumb	0.0114
Australia	1995	Amputation of thumb	0.0114
Australia	2000	Amputation of thumb	0.0114
Australia	2005	Amputation of thumb	0.0114
Australia	2010	Amputation of thumb	0.0114
Australia	2017	Amputation of thumb	0.0114
Australia	1990	Amputation of toe/toes	0.0059
Australia	1995	Amputation of toe/toes	0.0059
Australia	2000	Amputation of toe/toes	0.0059
Australia	2005	Amputation of toe/toes	0.0059
Australia	2010	Amputation of toe/toes	0.0059
Australia	2017	Amputation of toe/toes	0.0059
Australia	1990	Burns, <20% total burned surface area without lower airway burns	0.0163
Australia	1995	Burns, <20% total burned surface area without lower airway burns	0.0163
Australia	2000	Burns, <20% total burned surface area without lower airway burns	0.0163
Australia	2005	Burns, <20% total burned surface area without lower airway burns	0.0163
Australia	2010	Burns, <20% total burned surface area without lower airway burns	0.0163
Australia	2017	Burns, <20% total burned surface area without lower airway burns	0.0163
Australia	1990	Burns, >=20% total burned surface area or >= 10% burned surface area if head/neck or hands/wrist involved w/o lower airway burns	0.1346
Australia	1995	Burns, >=20% total burned surface area or >= 10% burned surface area if head/neck or hands/wrist involved w/o lower airway burns	0.1346
Australia	2000	Burns, >=20% total burned surface area or >= 10% burned surface area if head/neck or hands/wrist involved w/o lower airway burns	0.1346
Australia	2005	Burns, >=20% total burned surface area or >= 10% burned surface area if head/neck or hands/wrist involved w/o lower airway burns	0.1346
Australia	2010	Burns, >=20% total burned surface area or >= 10% burned surface area if head/neck or hands/wrist involved w/o lower airway burns	0.1346
Australia	2017	Burns, >=20% total burned surface area or >= 10% burned surface area if head/neck or hands/wrist involved w/o lower airway burns	0.1346
Andorra	1990	Amputation of lower limbs, bilateral	0.1232
Andorra	1995	Amputation of lower limbs, bilateral	0.1232
Andorra	2000	Amputation of lower limbs, bilateral	0.1232
Andorra	2005	Amputation of lower limbs, bilateral	0.1232
Andorra	2010	Amputation of lower limbs, bilateral	0.1232
Andorra	2017	Amputation of lower limbs, bilateral	0.1232
Andorra	1990	Lower airway burns	0.2252
Andorra	1995	Lower airway burns	0.2252
Andorra	2000	Lower airway burns	0.2252
Andorra	2005	Lower airway burns	0.2252
Andorra	2010	Lower airway burns	0.2252
Andorra	2017	Lower airway burns	0.2252
Andorra	1990	Dislocation of hip	0.0159
Andorra	1995	Dislocation of hip	0.0159
Andorra	2000	Dislocation of hip	0.0159
Andorra	2005	Dislocation of hip	0.0159
Andorra	2010	Dislocation of hip	0.0159
Andorra	2017	Dislocation of hip	0.0159
Andorra	1990	Dislocation of knee	0.1134
Andorra	1995	Dislocation of knee	0.1134
Andorra	2000	Dislocation of knee	0.1134
Andorra	2005	Dislocation of knee	0.1134
Andorra	2010	Dislocation of knee	0.1134
Andorra	2017	Dislocation of knee	0.1134
Andorra	1990	Dislocation of shoulder	0.062
Andorra	1995	Dislocation of shoulder	0.062
Andorra	2000	Dislocation of shoulder	0.062
Andorra	2005	Dislocation of shoulder	0.062
Andorra	2010	Dislocation of shoulder	0.062
Andorra	2017	Dislocation of shoulder	0.062
Andorra	1990	Muscle and tendon injuries, including sprains and strains lesser dislocations	0.0075
Andorra	1995	Muscle and tendon injuries, including sprains and strains lesser dislocations	0.0075
Andorra	2000	Muscle and tendon injuries, including sprains and strains lesser dislocations	0.0075
Andorra	2005	Muscle and tendon injuries, including sprains and strains lesser dislocations	0.0075
Andorra	2010	Muscle and tendon injuries, including sprains and strains lesser dislocations	0.0075
Andorra	2017	Muscle and tendon injuries, including sprains and strains lesser dislocations	0.0075
Andorra	1990	Fracture of clavicle, scapula, or humerus	0.0349
Andorra	1995	Fracture of clavicle, scapula, or humerus	0.0349
Andorra	2000	Fracture of clavicle, scapula, or humerus	0.0349
Andorra	2005	Fracture of clavicle, scapula, or humerus	0.0349
Andorra	2010	Fracture of clavicle, scapula, or humerus	0.0349
Andorra	2017	Fracture of clavicle, scapula, or humerus	0.0349
Andorra	1990	Fracture of face bones	0.0669
Andorra	1995	Fracture of face bones	0.0669
Andorra	2000	Fracture of face bones	0.0669
Andorra	2005	Fracture of face bones	0.0669
Andorra	2010	Fracture of face bones	0.0669
Andorra	2017	Fracture of face bones	0.0669
Andorra	1990	Fracture of foot bones except ankle	0.0264
Andorra	1995	Fracture of foot bones except ankle	0.0264
Andorra	2000	Fracture of foot bones except ankle	0.0264
Andorra	2005	Fracture of foot bones except ankle	0.0264
Andorra	2010	Fracture of foot bones except ankle	0.0264
Andorra	2017	Fracture of foot bones except ankle	0.0264
Andorra	1990	Fracture of hand (wrist and other distal part of hand)	0.0138
Andorra	1995	Fracture of hand (wrist and other distal part of hand)	0.0138
Andorra	2000	Fracture of hand (wrist and other distal part of hand)	0.0138
Andorra	2005	Fracture of hand (wrist and other distal part of hand)	0.0138
Andorra	2010	Fracture of hand (wrist and other distal part of hand)	0.0138
Andorra	2017	Fracture of hand (wrist and other distal part of hand)	0.0138
Andorra	1990	Fracture of hip	0.0577
Andorra	1995	Fracture of hip	0.0577
Andorra	2000	Fracture of hip	0.0577
Andorra	2005	Fracture of hip	0.0577
Andorra	2010	Fracture of hip	0.0577
Andorra	2017	Fracture of hip	0.0577
Andorra	1990	Amputation of upper limbs, bilateral	0.0876
Andorra	1995	Amputation of upper limbs, bilateral	0.0876
Andorra	2000	Amputation of upper limbs, bilateral	0.0876
Andorra	2005	Amputation of upper limbs, bilateral	0.0876
Andorra	2010	Amputation of upper limbs, bilateral	0.0876
Andorra	2017	Amputation of upper limbs, bilateral	0.0876
Andorra	1990	Fracture of patella, tibia or fibula, or ankle	0.0554
Andorra	1995	Fracture of patella, tibia or fibula, or ankle	0.0554
Andorra	2000	Fracture of patella, tibia or fibula, or ankle	0.0554
Andorra	2005	Fracture of patella, tibia or fibula, or ankle	0.0554
Andorra	2010	Fracture of patella, tibia or fibula, or ankle	0.0554
Andorra	2017	Fracture of patella, tibia or fibula, or ankle	0.0554
Andorra	1990	Fracture of pelvis	0.1823
Andorra	1995	Fracture of pelvis	0.1823
Andorra	2000	Fracture of pelvis	0.1823
Andorra	2005	Fracture of pelvis	0.1823
Andorra	2010	Fracture of pelvis	0.1823
Andorra	2017	Fracture of pelvis	0.1823
Andorra	1990	Fracture of radius and/or ulna	0.0435
Andorra	1995	Fracture of radius and/or ulna	0.0435
Andorra	2000	Fracture of radius and/or ulna	0.0435
Andorra	2005	Fracture of radius and/or ulna	0.0435
Andorra	2010	Fracture of radius and/or ulna	0.0435
Andorra	2017	Fracture of radius and/or ulna	0.0435
Andorra	1990	Fracture of skull	0.0714
Andorra	1995	Fracture of skull	0.0714
Andorra	2000	Fracture of skull	0.0714
Andorra	2005	Fracture of skull	0.0714
Andorra	2010	Fracture of skull	0.0714
Andorra	2017	Fracture of skull	0.0714
Andorra	1990	Fracture of sternum and/or fracture of one or more ribs	0.1027
Andorra	1995	Fracture of sternum and/or fracture of one or more ribs	0.1027
Andorra	2000	Fracture of sternum and/or fracture of one or more ribs	0.1027
Andorra	2005	Fracture of sternum and/or fracture of one or more ribs	0.1027
Andorra	2010	Fracture of sternum and/or fracture of one or more ribs	0.1027
Andorra	2017	Fracture of sternum and/or fracture of one or more ribs	0.1027
Andorra	1990	Fracture of vertebral column	0.1106
Andorra	1995	Fracture of vertebral column	0.1106
Andorra	2000	Fracture of vertebral column	0.1106
Andorra	2005	Fracture of vertebral column	0.1106
Andorra	2010	Fracture of vertebral column	0.1106
Andorra	2017	Fracture of vertebral column	0.1106
Andorra	1990	Fracture of femur, other than femoral neck	0.042
Andorra	1995	Fracture of femur, other than femoral neck	0.042
Andorra	2000	Fracture of femur, other than femoral neck	0.042
Andorra	2005	Fracture of femur, other than femoral neck	0.042
Andorra	2010	Fracture of femur, other than femoral neck	0.042
Andorra	2017	Fracture of femur, other than femoral neck	0.042
Andorra	1990	Minor TBI	0.1319
Andorra	1995	Minor TBI	0.1319
Andorra	2000	Minor TBI	0.1319
Andorra	2005	Minor TBI	0.1319
Andorra	2010	Minor TBI	0.1319
Andorra	2017	Minor TBI	0.1319
Andorra	1990	Moderate TBI	0.1637
Andorra	1995	Moderate TBI	0.1637
Andorra	2000	Moderate TBI	0.1637
Andorra	2005	Moderate TBI	0.1637
Andorra	2010	Moderate TBI	0.1637
Andorra	2017	Moderate TBI	0.1637
Andorra	1990	Amputation of fingers (excluding thumb)	0.0047
Andorra	1995	Amputation of fingers (excluding thumb)	0.0047
Andorra	2000	Amputation of fingers (excluding thumb)	0.0047
Andorra	2005	Amputation of fingers (excluding thumb)	0.0047
Andorra	2010	Amputation of fingers (excluding thumb)	0.0047
Andorra	2017	Amputation of fingers (excluding thumb)	0.0047
Andorra	1990	Spinal cord lesion at neck level	0.4118
Andorra	1995	Spinal cord lesion at neck level	0.4118
Andorra	2000	Spinal cord lesion at neck level	0.4118
Andorra	2005	Spinal cord lesion at neck level	0.4118
Andorra	2010	Spinal cord lesion at neck level	0.4118
Andorra	2017	Spinal cord lesion at neck level	0.4118
Andorra	1990	Spinal cord lesion below neck level	0.1777
Andorra	1995	Spinal cord lesion below neck level	0.1777
Andorra	2000	Spinal cord lesion below neck level	0.1777
Andorra	2005	Spinal cord lesion below neck level	0.1777
Andorra	2010	Spinal cord lesion below neck level	0.1777
Andorra	2017	Spinal cord lesion below neck level	0.1777
Andorra	1990	Drowning and nonfatal submersion	0.2471
Andorra	1995	Drowning and nonfatal submersion	0.2471
Andorra	2000	Drowning and nonfatal submersion	0.2471
Andorra	2005	Drowning and nonfatal submersion	0.2471
Andorra	2010	Drowning and nonfatal submersion	0.2471
Andorra	2017	Drowning and nonfatal submersion	0.2471
Andorra	1990	Asphyxiation	0.2471
Andorra	1995	Asphyxiation	0.2471
Andorra	2000	Asphyxiation	0.2471
Andorra	2005	Asphyxiation	0.2471
Andorra	2010	Asphyxiation	0.2471
Andorra	2017	Asphyxiation	0.2471
Andorra	1990	Crush injury	0.1325
Andorra	1995	Crush injury	0.1325
Andorra	2000	Crush injury	0.1325
Andorra	2005	Crush injury	0.1325
Andorra	2010	Crush injury	0.1325
Andorra	2017	Crush injury	0.1325
Andorra	1990	Nerve injury	0.1129
Andorra	1995	Nerve injury	0.1129
Andorra	2000	Nerve injury	0.1129
Andorra	2005	Nerve injury	0.1129
Andorra	2010	Nerve injury	0.1129
Andorra	2017	Nerve injury	0.1129
Andorra	1990	Injury to eyes	0.0165
Andorra	1995	Injury to eyes	0.0165
Andorra	2000	Injury to eyes	0.0165
Andorra	2005	Injury to eyes	0.0165
Andorra	2010	Injury to eyes	0.0165
Andorra	2017	Injury to eyes	0.0165
Andorra	1990	Amputation of lower limb, unilateral	0.039
Andorra	1995	Amputation of lower limb, unilateral	0.039
Andorra	2000	Amputation of lower limb, unilateral	0.039
Andorra	2005	Amputation of lower limb, unilateral	0.039
Andorra	2010	Amputation of lower limb, unilateral	0.039
Andorra	2017	Amputation of lower limb, unilateral	0.039
Andorra	1990	Open wound(s)	0.011
Andorra	1995	Open wound(s)	0.011
Andorra	2000	Open wound(s)	0.011
Andorra	2005	Open wound(s)	0.011
Andorra	2010	Open wound(s)	0.011
Andorra	2017	Open wound(s)	0.011
Andorra	1990	Poisoning requiring urgent care	0.1628
Andorra	1995	Poisoning requiring urgent care	0.1628
Andorra	2000	Poisoning requiring urgent care	0.1628
Andorra	2005	Poisoning requiring urgent care	0.1628
Andorra	2010	Poisoning requiring urgent care	0.1628
Andorra	2017	Poisoning requiring urgent care	0.1628
Andorra	1990	Severe chest injury	0.0474
Andorra	1995	Severe chest injury	0.0474
Andorra	2000	Severe chest injury	0.0474
Andorra	2005	Severe chest injury	0.0474
Andorra	2010	Severe chest injury	0.0474
Andorra	2017	Severe chest injury	0.0474
Andorra	1990	Internal hemorrhage in abdomen and pelvis	0.1143
Andorra	1995	Internal hemorrhage in abdomen and pelvis	0.1143
Andorra	2000	Internal hemorrhage in abdomen and pelvis	0.1143
Andorra	2005	Internal hemorrhage in abdomen and pelvis	0.1143
Andorra	2010	Internal hemorrhage in abdomen and pelvis	0.1143
Andorra	2017	Internal hemorrhage in abdomen and pelvis	0.1143
Andorra	1990	Contusion in any part of the body	0.0075
Andorra	1995	Contusion in any part of the body	0.0075
Andorra	2000	Contusion in any part of the body	0.0075
Andorra	2005	Contusion in any part of the body	0.0075
Andorra	2010	Contusion in any part of the body	0.0075
Andorra	2017	Contusion in any part of the body	0.0075
Andorra	1990	Effect of different environmental factors	0.049
Andorra	1995	Effect of different environmental factors	0.049
Andorra	2000	Effect of different environmental factors	0.049
Andorra	2005	Effect of different environmental factors	0.049
Andorra	2010	Effect of different environmental factors	0.049
Andorra	2017	Effect of different environmental factors	0.049
Andorra	1990	Complications following therapeutic procedures	0.0506
Andorra	1995	Complications following therapeutic procedures	0.0506
Andorra	2000	Complications following therapeutic procedures	0.0506
Andorra	2005	Complications following therapeutic procedures	0.0506
Andorra	2010	Complications following therapeutic procedures	0.0506
Andorra	2017	Complications following therapeutic procedures	0.0506
Andorra	1990	Multiple fractures, dislocations, crashes, wounds, sprains, and strains	0.0577
Andorra	1995	Multiple fractures, dislocations, crashes, wounds, sprains, and strains	0.0577
Andorra	2000	Multiple fractures, dislocations, crashes, wounds, sprains, and strains	0.0577
Andorra	2005	Multiple fractures, dislocations, crashes, wounds, sprains, and strains	0.0577
Andorra	2010	Multiple fractures, dislocations, crashes, wounds, sprains, and strains	0.0577
Andorra	2017	Multiple fractures, dislocations, crashes, wounds, sprains, and strains	0.0577
Andorra	1990	Amputation of upper limb, unilateral	0.039
Andorra	1995	Amputation of upper limb, unilateral	0.039
Andorra	2000	Amputation of upper limb, unilateral	0.039
Andorra	2005	Amputation of upper limb, unilateral	0.039
Andorra	2010	Amputation of upper limb, unilateral	0.039
Andorra	2017	Amputation of upper limb, unilateral	0.039
Andorra	1990	Amputation of thumb	0.0114
Andorra	1995	Amputation of thumb	0.0114
Andorra	2000	Amputation of thumb	0.0114
Andorra	2005	Amputation of thumb	0.0114
Andorra	2010	Amputation of thumb	0.0114
Andorra	2017	Amputation of thumb	0.0114
Andorra	1990	Amputation of toe/toes	0.0059
Andorra	1995	Amputation of toe/toes	0.0059
Andorra	2000	Amputation of toe/toes	0.0059
Andorra	2005	Amputation of toe/toes	0.0059
Andorra	2010	Amputation of toe/toes	0.0059
Andorra	2017	Amputation of toe/toes	0.0059
Andorra	1990	Burns, <20% total burned surface area without lower airway burns	0.0163
Andorra	1995	Burns, <20% total burned surface area without lower airway burns	0.0163
Andorra	2000	Burns, <20% total burned surface area without lower airway burns	0.0163
Andorra	2005	Burns, <20% total burned surface area without lower airway burns	0.0163
Andorra	2010	Burns, <20% total burned surface area without lower airway burns	0.0163
Andorra	2017	Burns, <20% total burned surface area without lower airway burns	0.0163
Andorra	1990	Burns, >=20% total burned surface area or >= 10% burned surface area if head/neck or hands/wrist involved w/o lower airway burns	0.1346
Andorra	1995	Burns, >=20% total burned surface area or >= 10% burned surface area if head/neck or hands/wrist involved w/o lower airway burns	0.1346
Andorra	2000	Burns, >=20% total burned surface area or >= 10% burned surface area if head/neck or hands/wrist involved w/o lower airway burns	0.1346
Andorra	2005	Burns, >=20% total burned surface area or >= 10% burned surface area if head/neck or hands/wrist involved w/o lower airway burns	0.1346
Andorra	2010	Burns, >=20% total burned surface area or >= 10% burned surface area if head/neck or hands/wrist involved w/o lower airway burns	0.1346
Andorra	2017	Burns, >=20% total burned surface area or >= 10% burned surface area if head/neck or hands/wrist involved w/o lower airway burns	0.1346
Austria	1990	Amputation of lower limbs, bilateral	0.1232
Austria	1995	Amputation of lower limbs, bilateral	0.1232
Austria	2000	Amputation of lower limbs, bilateral	0.1232
Austria	2005	Amputation of lower limbs, bilateral	0.1232
Austria	2010	Amputation of lower limbs, bilateral	0.1232
Austria	2017	Amputation of lower limbs, bilateral	0.1232
Austria	1990	Lower airway burns	0.2252
Austria	1995	Lower airway burns	0.2252
Austria	2000	Lower airway burns	0.2252
Austria	2005	Lower airway burns	0.2252
Austria	2010	Lower airway burns	0.2252
Austria	2017	Lower airway burns	0.2252
Austria	1990	Dislocation of hip	0.0159
Austria	1995	Dislocation of hip	0.0159
Austria	2000	Dislocation of hip	0.0159
Austria	2005	Dislocation of hip	0.0159
Austria	2010	Dislocation of hip	0.0159
Austria	2017	Dislocation of hip	0.0159
Austria	1990	Dislocation of knee	0.1134
Austria	1995	Dislocation of knee	0.1134
Austria	2000	Dislocation of knee	0.1134
Austria	2005	Dislocation of knee	0.1134
Austria	2010	Dislocation of knee	0.1134
Austria	2017	Dislocation of knee	0.1134
Austria	1990	Dislocation of shoulder	0.062
Austria	1995	Dislocation of shoulder	0.062
Austria	2000	Dislocation of shoulder	0.062
Austria	2005	Dislocation of shoulder	0.062
Austria	2010	Dislocation of shoulder	0.062
Austria	2017	Dislocation of shoulder	0.062
Austria	1990	Muscle and tendon injuries, including sprains and strains lesser dislocations	0.0075
Austria	1995	Muscle and tendon injuries, including sprains and strains lesser dislocations	0.0075
Austria	2000	Muscle and tendon injuries, including sprains and strains lesser dislocations	0.0075
Austria	2005	Muscle and tendon injuries, including sprains and strains lesser dislocations	0.0075
Austria	2010	Muscle and tendon injuries, including sprains and strains lesser dislocations	0.0075
Austria	2017	Muscle and tendon injuries, including sprains and strains lesser dislocations	0.0075
Austria	1990	Fracture of clavicle, scapula, or humerus	0.0349
Austria	1995	Fracture of clavicle, scapula, or humerus	0.0349
Austria	2000	Fracture of clavicle, scapula, or humerus	0.0349
Austria	2005	Fracture of clavicle, scapula, or humerus	0.0349
Austria	2010	Fracture of clavicle, scapula, or humerus	0.0349
Austria	2017	Fracture of clavicle, scapula, or humerus	0.0349
Austria	1990	Fracture of face bones	0.0669
Austria	1995	Fracture of face bones	0.0669
Austria	2000	Fracture of face bones	0.0669
Austria	2005	Fracture of face bones	0.0669
Austria	2010	Fracture of face bones	0.0669
Austria	2017	Fracture of face bones	0.0669
Austria	1990	Fracture of foot bones except ankle	0.0264
Austria	1995	Fracture of foot bones except ankle	0.0264
Austria	2000	Fracture of foot bones except ankle	0.0264
Austria	2005	Fracture of foot bones except ankle	0.0264
Austria	2010	Fracture of foot bones except ankle	0.0264
Austria	2017	Fracture of foot bones except ankle	0.0264
Austria	1990	Fracture of hand (wrist and other distal part of hand)	0.0138
Austria	1995	Fracture of hand (wrist and other distal part of hand)	0.0138
Austria	2000	Fracture of hand (wrist and other distal part of hand)	0.0138
Austria	2005	Fracture of hand (wrist and other distal part of hand)	0.0138
Austria	2010	Fracture of hand (wrist and other distal part of hand)	0.0138
Austria	2017	Fracture of hand (wrist and other distal part of hand)	0.0138
Austria	1990	Fracture of hip	0.0577
Austria	1995	Fracture of hip	0.0577
Austria	2000	Fracture of hip	0.0577
Austria	2005	Fracture of hip	0.0577
Austria	2010	Fracture of hip	0.0577
Austria	2017	Fracture of hip	0.0577
Austria	1990	Amputation of upper limbs, bilateral	0.0876
Austria	1995	Amputation of upper limbs, bilateral	0.0876
Austria	2000	Amputation of upper limbs, bilateral	0.0876
Austria	2005	Amputation of upper limbs, bilateral	0.0876
Austria	2010	Amputation of upper limbs, bilateral	0.0876
Austria	2017	Amputation of upper limbs, bilateral	0.0876
Austria	1990	Fracture of patella, tibia or fibula, or ankle	0.0554
Austria	1995	Fracture of patella, tibia or fibula, or ankle	0.0554
Austria	2000	Fracture of patella, tibia or fibula, or ankle	0.0554
Austria	2005	Fracture of patella, tibia or fibula, or ankle	0.0554
Austria	2010	Fracture of patella, tibia or fibula, or ankle	0.0554
Austria	2017	Fracture of patella, tibia or fibula, or ankle	0.0554
Austria	1990	Fracture of pelvis	0.1823
Austria	1995	Fracture of pelvis	0.1823
Austria	2000	Fracture of pelvis	0.1823
Austria	2005	Fracture of pelvis	0.1823
Austria	2010	Fracture of pelvis	0.1823
Austria	2017	Fracture of pelvis	0.1823
Austria	1990	Fracture of radius and/or ulna	0.0435
Austria	1995	Fracture of radius and/or ulna	0.0435
Austria	2000	Fracture of radius and/or ulna	0.0435
Austria	2005	Fracture of radius and/or ulna	0.0435
Austria	2010	Fracture of radius and/or ulna	0.0435
Austria	2017	Fracture of radius and/or ulna	0.0435
Austria	1990	Fracture of skull	0.0714
Austria	1995	Fracture of skull	0.0714
Austria	2000	Fracture of skull	0.0714
Austria	2005	Fracture of skull	0.0714
Austria	2010	Fracture of skull	0.0714
Austria	2017	Fracture of skull	0.0714
Austria	1990	Fracture of sternum and/or fracture of one or more ribs	0.1027
Austria	1995	Fracture of sternum and/or fracture of one or more ribs	0.1027
Austria	2000	Fracture of sternum and/or fracture of one or more ribs	0.1027
Austria	2005	Fracture of sternum and/or fracture of one or more ribs	0.1027
Austria	2010	Fracture of sternum and/or fracture of one or more ribs	0.1027
Austria	2017	Fracture of sternum and/or fracture of one or more ribs	0.1027
Austria	1990	Fracture of vertebral column	0.1106
Austria	1995	Fracture of vertebral column	0.1106
Austria	2000	Fracture of vertebral column	0.1106
Austria	2005	Fracture of vertebral column	0.1106
Austria	2010	Fracture of vertebral column	0.1106
Austria	2017	Fracture of vertebral column	0.1106
Austria	1990	Fracture of femur, other than femoral neck	0.042
Austria	1995	Fracture of femur, other than femoral neck	0.042
Austria	2000	Fracture of femur, other than femoral neck	0.042
Austria	2005	Fracture of femur, other than femoral neck	0.042
Austria	2010	Fracture of femur, other than femoral neck	0.042
Austria	2017	Fracture of femur, other than femoral neck	0.042
Austria	1990	Minor TBI	0.1319
Austria	1995	Minor TBI	0.1319
Austria	2000	Minor TBI	0.1319

Austria	2005	Minor TBI	0.1319
Austria	2010	Minor TBI	0.1319
Austria	2017	Minor TBI	0.1319
Austria	1990	Moderate TBI	0.1637
Austria	1995	Moderate TBI	0.1637
Austria	2000	Moderate TBI	0.1637
Austria	2005	Moderate TBI	0.1637
Austria	2010	Moderate TBI	0.1637
Austria	2017	Moderate TBI	0.1637
Austria	1990	Amputation of fingers (excluding thumb)	0.0047
Austria	1995	Amputation of fingers (excluding thumb)	0.0047
Austria	2000	Amputation of fingers (excluding thumb)	0.0047
Austria	2005	Amputation of fingers (excluding thumb)	0.0047
Austria	2010	Amputation of fingers (excluding thumb)	0.0047
Austria	2017	Amputation of fingers (excluding thumb)	0.0047
Austria	1990	Spinal cord lesion at neck level	0.4118
Austria	1995	Spinal cord lesion at neck level	0.4118
Austria	2000	Spinal cord lesion at neck level	0.4118
Austria	2005	Spinal cord lesion at neck level	0.4118
Austria	2010	Spinal cord lesion at neck level	0.4118
Austria	2017	Spinal cord lesion at neck level	0.4118
Austria	1990	Spinal cord lesion below neck level	0.1777
Austria	1995	Spinal cord lesion below neck level	0.1777
Austria	2000	Spinal cord lesion below neck level	0.1777
Austria	2005	Spinal cord lesion below neck level	0.1777
Austria	2010	Spinal cord lesion below neck level	0.1777
Austria	2017	Spinal cord lesion below neck level	0.1777
Austria	1990	Drowning and nonfatal submersion	0.2471
Austria	1995	Drowning and nonfatal submersion	0.2471
Austria	2000	Drowning and nonfatal submersion	0.2471
Austria	2005	Drowning and nonfatal submersion	0.2471
Austria	2010	Drowning and nonfatal submersion	0.2471
Austria	2017	Drowning and nonfatal submersion	0.2471
Austria	1990	Asphyxiation	0.2471
Austria	1995	Asphyxiation	0.2471
Austria	2000	Asphyxiation	0.2471
Austria	2005	Asphyxiation	0.2471
Austria	2010	Asphyxiation	0.2471
Austria	2017	Asphyxiation	0.2471
Austria	1990	Crush injury	0.1325
Austria	1995	Crush injury	0.1325
Austria	2000	Crush injury	0.1325
Austria	2005	Crush injury	0.1325
Austria	2010	Crush injury	0.1325
Austria	2017	Crush injury	0.1325
Austria	1990	Nerve injury	0.1129
Austria	1995	Nerve injury	0.1129
Austria	2000	Nerve injury	0.1129
Austria	2005	Nerve injury	0.1129
Austria	2010	Nerve injury	0.1129
Austria	2017	Nerve injury	0.1129
Austria	1990	Injury to eyes	0.0165
Austria	1995	Injury to eyes	0.0165
Austria	2000	Injury to eyes	0.0165
Austria	2005	Injury to eyes	0.0165
Austria	2010	Injury to eyes	0.0165
Austria	2017	Injury to eyes	0.0165
Austria	1990	Amputation of lower limb, unilateral	0.039
Austria	1995	Amputation of lower limb, unilateral	0.039
Austria	2000	Amputation of lower limb, unilateral	0.039
Austria	2005	Amputation of lower limb, unilateral	0.039
Austria	2010	Amputation of lower limb, unilateral	0.039
Austria	2017	Amputation of lower limb, unilateral	0.039
Austria	1990	Open wound(s)	0.011
Austria	1995	Open wound(s)	0.011
Austria	2000	Open wound(s)	0.011
Austria	2005	Open wound(s)	0.011
Austria	2010	Open wound(s)	0.011
Austria	2017	Open wound(s)	0.011
Austria	1990	Poisoning requiring urgent care	0.1628
Austria	1995	Poisoning requiring urgent care	0.1628
Austria	2000	Poisoning requiring urgent care	0.1628
Austria	2005	Poisoning requiring urgent care	0.1628
Austria	2010	Poisoning requiring urgent care	0.1628
Austria	2017	Poisoning requiring urgent care	0.1628
Austria	1990	Severe chest injury	0.0474
Austria	1995	Severe chest injury	0.0474
Austria	2000	Severe chest injury	0.0474
Austria	2005	Severe chest injury	0.0474
Austria	2010	Severe chest injury	0.0474
Austria	2017	Severe chest injury	0.0474
Austria	1990	Internal hemorrhage in abdomen and pelvis	0.1143
Austria	1995	Internal hemorrhage in abdomen and pelvis	0.1143
Austria	2000	Internal hemorrhage in abdomen and pelvis	0.1143
Austria	2005	Internal hemorrhage in abdomen and pelvis	0.1143
Austria	2010	Internal hemorrhage in abdomen and pelvis	0.1143
Austria	2017	Internal hemorrhage in abdomen and pelvis	0.1143
Austria	1990	Contusion in any part of the body	0.0075
Austria	1995	Contusion in any part of the body	0.0075
Austria	2000	Contusion in any part of the body	0.0075
Austria	2005	Contusion in any part of the body	0.0075
Austria	2010	Contusion in any part of the body	0.0075
Austria	2017	Contusion in any part of the body	0.0075
Austria	1990	Effect of different environmental factors	0.049
Austria	1995	Effect of different environmental factors	0.049
Austria	2000	Effect of different environmental factors	0.049
Austria	2005	Effect of different environmental factors	0.049
Austria	2010	Effect of different environmental factors	0.049
Austria	2017	Effect of different environmental factors	0.049
Austria	1990	Complications following therapeutic procedures	0.0506
Austria	1995	Complications following therapeutic procedures	0.0506
Austria	2000	Complications following therapeutic procedures	0.0506
Austria	2005	Complications following therapeutic procedures	0.0506
Austria	2010	Complications following therapeutic procedures	0.0506
Austria	2017	Complications following therapeutic procedures	0.0506
Austria	1990	Multiple fractures, dislocations, crashes, wounds, sprains, and strains	0.0577
Austria	1995	Multiple fractures, dislocations, crashes, wounds, sprains, and strains	0.0577
Austria	2000	Multiple fractures, dislocations, crashes, wounds, sprains, and strains	0.0577
Austria	2005	Multiple fractures, dislocations, crashes, wounds, sprains, and strains	0.0577
Austria	2010	Multiple fractures, dislocations, crashes, wounds, sprains, and strains	0.0577
Austria	2017	Multiple fractures, dislocations, crashes, wounds, sprains, and strains	0.0577
Austria	1990	Amputation of upper limb, unilateral	0.039
Austria	1995	Amputation of upper limb, unilateral	0.039
Austria	2000	Amputation of upper limb, unilateral	0.039
Austria	2005	Amputation of upper limb, unilateral	0.039
Austria	2010	Amputation of upper limb, unilateral	0.039
Austria	2017	Amputation of upper limb, unilateral	0.039
Austria	1990	Amputation of thumb	0.0114
Austria	1995	Amputation of thumb	0.0114
Austria	2000	Amputation of thumb	0.0114
Austria	2005	Amputation of thumb	0.0114
Austria	2010	Amputation of thumb	0.0114
Austria	2017	Amputation of thumb	0.0114
Austria	1990	Amputation of toe/toes	0.0059
Austria	1995	Amputation of toe/toes	0.0059
Austria	2000	Amputation of toe/toes	0.0059
Austria	2005	Amputation of toe/toes	0.0059
Austria	2010	Amputation of toe/toes	0.0059
Austria	2017	Amputation of toe/toes	0.0059
Austria	1990	Burns, <20% total burned surface area without lower airway burns	0.0163
Austria	1995	Burns, <20% total burned surface area without lower airway burns	0.0163
Austria	2000	Burns, <20% total burned surface area without lower airway burns	0.0163
Austria	2005	Burns, <20% total burned surface area without lower airway burns	0.0163
Austria	2010	Burns, <20% total burned surface area without lower airway burns	0.0163
Austria	2017	Burns, <20% total burned surface area without lower airway burns	0.0163
Austria	1990	Burns, >=20% total burned surface area or >= 10% burned surface area if head/neck or hands/wrist involved w/o lower airway burns	0.1346
Austria	1995	Burns, >=20% total burned surface area or >= 10% burned surface area if head/neck or hands/wrist involved w/o lower airway burns	0.1346
Austria	2000	Burns, >=20% total burned surface area or >= 10% burned surface area if head/neck or hands/wrist involved w/o lower airway burns	0.1346
Austria	2005	Burns, >=20% total burned surface area or >= 10% burned surface area if head/neck or hands/wrist involved w/o lower airway burns	0.1346
Austria	2010	Burns, >=20% total burned surface area or >= 10% burned surface area if head/neck or hands/wrist involved w/o lower airway burns	0.1346
Austria	2017	Burns, >=20% total burned surface area or >= 10% burned surface area if head/neck or hands/wrist involved w/o lower airway burns	0.1346
Belgium	1990	Amputation of lower limbs, bilateral	0.1232
Belgium	1995	Amputation of lower limbs, bilateral	0.1232
Belgium	2000	Amputation of lower limbs, bilateral	0.1232
Belgium	2005	Amputation of lower limbs, bilateral	0.1232
Belgium	2010	Amputation of lower limbs, bilateral	0.1232
Belgium	2017	Amputation of lower limbs, bilateral	0.1232
Belgium	1990	Lower airway burns	0.2252
Belgium	1995	Lower airway burns	0.2252
Belgium	2000	Lower airway burns	0.2252
Belgium	2005	Lower airway burns	0.2252
Belgium	2010	Lower airway burns	0.2252
Belgium	2017	Lower airway burns	0.2252
Belgium	1990	Dislocation of hip	0.0159
Belgium	1995	Dislocation of hip	0.0159
Belgium	2000	Dislocation of hip	0.0159
Belgium	2005	Dislocation of hip	0.0159
Belgium	2010	Dislocation of hip	0.0159
Belgium	2017	Dislocation of hip	0.0159
Belgium	1990	Dislocation of knee	0.1134
Belgium	1995	Dislocation of knee	0.1134
Belgium	2000	Dislocation of knee	0.1134
Belgium	2005	Dislocation of knee	0.1134
Belgium	2010	Dislocation of knee	0.1134
Belgium	2017	Dislocation of knee	0.1134
Belgium	1990	Dislocation of shoulder	0.062
Belgium	1995	Dislocation of shoulder	0.062
Belgium	2000	Dislocation of shoulder	0.062
Belgium	2005	Dislocation of shoulder	0.062
Belgium	2010	Dislocation of shoulder	0.062
Belgium	2017	Dislocation of shoulder	0.062
Belgium	1990	Muscle and tendon injuries, including sprains and strains lesser dislocations	0.0075
Belgium	1995	Muscle and tendon injuries, including sprains and strains lesser dislocations	0.0075
Belgium	2000	Muscle and tendon injuries, including sprains and strains lesser dislocations	0.0075
Belgium	2005	Muscle and tendon injuries, including sprains and strains lesser dislocations	0.0075
Belgium	2010	Muscle and tendon injuries, including sprains and strains lesser dislocations	0.0075
Belgium	2017	Muscle and tendon injuries, including sprains and strains lesser dislocations	0.0075
Belgium	1990	Fracture of clavicle, scapula, or humerus	0.0349
Belgium	1995	Fracture of clavicle, scapula, or humerus	0.0349
Belgium	2000	Fracture of clavicle, scapula, or humerus	0.0349
Belgium	2005	Fracture of clavicle, scapula, or humerus	0.0349
Belgium	2010	Fracture of clavicle, scapula, or humerus	0.0349
Belgium	2017	Fracture of clavicle, scapula, or humerus	0.0349
Belgium	1990	Fracture of face bones	0.0669
Belgium	1995	Fracture of face bones	0.0669
Belgium	2000	Fracture of face bones	0.0669
Belgium	2005	Fracture of face bones	0.0669
Belgium	2010	Fracture of face bones	0.0669
Belgium	2017	Fracture of face bones	0.0669
Belgium	1990	Fracture of foot bones except ankle	0.0264
Belgium	1995	Fracture of foot bones except ankle	0.0264
Belgium	2000	Fracture of foot bones except ankle	0.0264
Belgium	2005	Fracture of foot bones except ankle	0.0264
Belgium	2010	Fracture of foot bones except ankle	0.0264
Belgium	2017	Fracture of foot bones except ankle	0.0264
Belgium	1990	Fracture of hand (wrist and other distal part of hand)	0.0138
Belgium	1995	Fracture of hand (wrist and other distal part of hand)	0.0138
Belgium	2000	Fracture of hand (wrist and other distal part of hand)	0.0138
Belgium	2005	Fracture of hand (wrist and other distal part of hand)	0.0138
Belgium	2010	Fracture of hand (wrist and other distal part of hand)	0.0138
Belgium	2017	Fracture of hand (wrist and other distal part of hand)	0.0138
Belgium	1990	Fracture of hip	0.0577
Belgium	1995	Fracture of hip	0.0577
Belgium	2000	Fracture of hip	0.0577
Belgium	2005	Fracture of hip	0.0577
Belgium	2010	Fracture of hip	0.0577
Belgium	2017	Fracture of hip	0.0577
Belgium	1990	Amputation of upper limbs, bilateral	0.0876
Belgium	1995	Amputation of upper limbs, bilateral	0.0876
Belgium	2000	Amputation of upper limbs, bilateral	0.0876
Belgium	2005	Amputation of upper limbs, bilateral	0.0876
Belgium	2010	Amputation of upper limbs, bilateral	0.0876
Belgium	2017	Amputation of upper limbs, bilateral	0.0876
Belgium	1990	Fracture of patella, tibia or fibula, or ankle	0.0554
Belgium	1995	Fracture of patella, tibia or fibula, or ankle	0.0554
Belgium	2000	Fracture of patella, tibia or fibula, or ankle	0.0554
Belgium	2005	Fracture of patella, tibia or fibula, or ankle	0.0554
Belgium	2010	Fracture of patella, tibia or fibula, or ankle	0.0554
Belgium	2017	Fracture of patella, tibia or fibula, or ankle	0.0554
Belgium	1990	Fracture of pelvis	0.1823
Belgium	1995	Fracture of pelvis	0.1823
Belgium	2000	Fracture of pelvis	0.1823
Belgium	2005	Fracture of pelvis	0.1823
Belgium	2010	Fracture of pelvis	0.1823
Belgium	2017	Fracture of pelvis	0.1823
Belgium	1990	Fracture of radius and/or ulna	0.0435
Belgium	1995	Fracture of radius and/or ulna	0.0435
Belgium	2000	Fracture of radius and/or ulna	0.0435
Belgium	2005	Fracture of radius and/or ulna	0.0435
Belgium	2010	Fracture of radius and/or ulna	0.0435
Belgium	2017	Fracture of radius and/or ulna	0.0435
Belgium	1990	Fracture of skull	0.0714
Belgium	1995	Fracture of skull	0.0714
Belgium	2000	Fracture of skull	0.0714
Belgium	2005	Fracture of skull	0.0714
Belgium	2010	Fracture of skull	0.0714
Belgium	2017	Fracture of skull	0.0714
Belgium	1990	Fracture of sternum and/or fracture of one or more ribs	0.1027
Belgium	1995	Fracture of sternum and/or fracture of one or more ribs	0.1027
Belgium	2000	Fracture of sternum and/or fracture of one or more ribs	0.1027
Belgium	2005	Fracture of sternum and/or fracture of one or more ribs	0.1027
Belgium	2010	Fracture of sternum and/or fracture of one or more ribs	0.1027
Belgium	2017	Fracture of sternum and/or fracture of one or more ribs	0.1027
Belgium	1990	Fracture of vertebral column	0.1106
Belgium	1995	Fracture of vertebral column	0.1106
Belgium	2000	Fracture of vertebral column	0.1106
Belgium	2005	Fracture of vertebral column	0.1106
Belgium	2010	Fracture of vertebral column	0.1106
Belgium	2017	Fracture of vertebral column	0.1106
Belgium	1990	Fracture of femur, other than femoral neck	0.042
Belgium	1995	Fracture of femur, other than femoral neck	0.042
Belgium	2000	Fracture of femur, other than femoral neck	0.042
Belgium	2005	Fracture of femur, other than femoral neck	0.042
Belgium	2010	Fracture of femur, other than femoral neck	0.042
Belgium	2017	Fracture of femur, other than femoral neck	0.042
Belgium	1990	Minor TBI	0.1319
Belgium	1995	Minor TBI	0.1319
Belgium	2000	Minor TBI	0.1319
Belgium	2005	Minor TBI	0.1319
Belgium	2010	Minor TBI	0.1319
Belgium	2017	Minor TBI	0.1319
Belgium	1990	Moderate TBI	0.1637
Belgium	1995	Moderate TBI	0.1637
Belgium	2000	Moderate TBI	0.1637
Belgium	2005	Moderate TBI	0.1637
Belgium	2010	Moderate TBI	0.1637
Belgium	2017	Moderate TBI	0.1637
Belgium	1990	Amputation of fingers (excluding thumb)	0.0047
Belgium	1995	Amputation of fingers (excluding thumb)	0.0047
Belgium	2000	Amputation of fingers (excluding thumb)	0.0047
Belgium	2005	Amputation of fingers (excluding thumb)	0.0047
Belgium	2010	Amputation of fingers (excluding thumb)	0.0047
Belgium	2017	Amputation of fingers (excluding thumb)	0.0047
Belgium	1990	Spinal cord lesion at neck level	0.4118
Belgium	1995	Spinal cord lesion at neck level	0.4118
Belgium	2000	Spinal cord lesion at neck level	0.4118
Belgium	2005	Spinal cord lesion at neck level	0.4118
Belgium	2010	Spinal cord lesion at neck level	0.4118
Belgium	2017	Spinal cord lesion at neck level	0.4118
Belgium	1990	Spinal cord lesion below neck level	0.1777
Belgium	1995	Spinal cord lesion below neck level	0.1777
Belgium	2000	Spinal cord lesion below neck level	0.1777
Belgium	2005	Spinal cord lesion below neck level	0.1777
Belgium	2010	Spinal cord lesion below neck level	0.1777
Belgium	2017	Spinal cord lesion below neck level	0.1777
Belgium	1990	Drowning and nonfatal submersion	0.2471
Belgium	1995	Drowning and nonfatal submersion	0.2471
Belgium	2000	Drowning and nonfatal submersion	0.2471
Belgium	2005	Drowning and nonfatal submersion	0.2471
Belgium	2010	Drowning and nonfatal submersion	0.2471
Belgium	2017	Drowning and nonfatal submersion	0.2471
Belgium	1990	Asphyxiation	0.2471
Belgium	1995	Asphyxiation	0.2471
Belgium	2000	Asphyxiation	0.2471
Belgium	2005	Asphyxiation	0.2471
Belgium	2010	Asphyxiation	0.2471
Belgium	2017	Asphyxiation	0.2471
Belgium	1990	Crush injury	0.1325
Belgium	1995	Crush injury	0.1325
Belgium	2000	Crush injury	0.1325
Belgium	2005	Crush injury	0.1325
Belgium	2010	Crush injury	0.1325
Belgium	2017	Crush injury	0.1325
Belgium	1990	Nerve injury	0.1129
Belgium	1995	Nerve injury	0.1129
Belgium	2000	Nerve injury	0.1129
Belgium	2005	Nerve injury	0.1129
Belgium	2010	Nerve injury	0.1129
Belgium	2017	Nerve injury	0.1129
Belgium	1990	Injury to eyes	0.0165
Belgium	1995	Injury to eyes	0.0165
Belgium	2000	Injury to eyes	0.0165
Belgium	2005	Injury to eyes	0.0165
Belgium	2010	Injury to eyes	0.0165
Belgium	2017	Injury to eyes	0.0165
Belgium	1990	Amputation of lower limb, unilateral	0.039
Belgium	1995	Amputation of lower limb, unilateral	0.039
Belgium	2000	Amputation of lower limb, unilateral	0.039
Belgium	2005	Amputation of lower limb, unilateral	0.039
Belgium	2010	Amputation of lower limb, unilateral	0.039
Belgium	2017	Amputation of lower limb, unilateral	0.039
Belgium	1990	Open wound(s)	0.011
Belgium	1995	Open wound(s)	0.011
Belgium	2000	Open wound(s)	0.011
Belgium	2005	Open wound(s)	0.011
Belgium	2010	Open wound(s)	0.011
Belgium	2017	Open wound(s)	0.011
Belgium	1990	Poisoning requiring urgent care	0.1628
Belgium	1995	Poisoning requiring urgent care	0.1628
Belgium	2000	Poisoning requiring urgent care	0.1628
Belgium	2005	Poisoning requiring urgent care	0.1628
Belgium	2010	Poisoning requiring urgent care	0.1628
Belgium	2017	Poisoning requiring urgent care	0.1628
Belgium	1990	Severe chest injury	0.0474
Belgium	1995	Severe chest injury	0.0474
Belgium	2000	Severe chest injury	0.0474
Belgium	2005	Severe chest injury	0.0474
Belgium	2010	Severe chest injury	0.0474
Belgium	2017	Severe chest injury	0.0474
Belgium	1990	Internal hemorrhage in abdomen and pelvis	0.1143
Belgium	1995	Internal hemorrhage in abdomen and pelvis	0.1143
Belgium	2000	Internal hemorrhage in abdomen and pelvis	0.1143
Belgium	2005	Internal hemorrhage in abdomen and pelvis	0.1143
Belgium	2010	Internal hemorrhage in abdomen and pelvis	0.1143
Belgium	2017	Internal hemorrhage in abdomen and pelvis	0.1143
Belgium	1990	Contusion in any part of the body	0.0075
Belgium	1995	Contusion in any part of the body	0.0075
Belgium	2000	Contusion in any part of the body	0.0075
Belgium	2005	Contusion in any part of the body	0.0075
Belgium	2010	Contusion in any part of the body	0.0075
Belgium	2017	Contusion in any part of the body	0.0075
Belgium	1990	Effect of different environmental factors	0.049
Belgium	1995	Effect of different environmental factors	0.049
Belgium	2000	Effect of different environmental factors	0.049
Belgium	2005	Effect of different environmental factors	0.049
Belgium	2010	Effect of different environmental factors	0.049
Belgium	2017	Effect of different environmental factors	0.049
Belgium	1990	Complications following therapeutic procedures	0.0506
Belgium	1995	Complications following therapeutic procedures	0.0506
Belgium	2000	Complications following therapeutic procedures	0.0506
Belgium	2005	Complications following therapeutic procedures	0.0506
Belgium	2010	Complications following therapeutic procedures	0.0506
Belgium	2017	Complications following therapeutic procedures	0.0506
Belgium	1990	Multiple fractures, dislocations, crashes, wounds, sprains, and strains	0.0577
Belgium	1995	Multiple fractures, dislocations, crashes, wounds, sprains, and strains	0.0577
Belgium	2000	Multiple fractures, dislocations, crashes, wounds, sprains, and strains	0.0577
Belgium	2005	Multiple fractures, dislocations, crashes, wounds, sprains, and strains	0.0577
Belgium	2010	Multiple fractures, dislocations, crashes, wounds, sprains, and strains	0.0577
Belgium	2017	Multiple fractures, dislocations, crashes, wounds, sprains, and strains	0.0577
Belgium	1990	Amputation of upper limb, unilateral	0.039
Belgium	1995	Amputation of upper limb, unilateral	0.039
Belgium	2000	Amputation of upper limb, unilateral	0.039
Belgium	2005	Amputation of upper limb, unilateral	0.039
Belgium	2010	Amputation of upper limb, unilateral	0.039
Belgium	2017	Amputation of upper limb, unilateral	0.039
Belgium	1990	Amputation of thumb	0.0114
Belgium	1995	Amputation of thumb	0.0114
Belgium	2000	Amputation of thumb	0.0114
Belgium	2005	Amputation of thumb	0.0114
Belgium	2010	Amputation of thumb	0.0114
Belgium	2017	Amputation of thumb	0.0114
Belgium	1990	Amputation of toe/toes	0.0059
Belgium	1995	Amputation of toe/toes	0.0059
Belgium	2000	Amputation of toe/toes	0.0059
Belgium	2005	Amputation of toe/toes	0.0059
Belgium	2010	Amputation of toe/toes	0.0059
Belgium	2017	Amputation of toe/toes	0.0059
Belgium	1990	Burns, <20% total burned surface area without lower airway burns	0.0163
Belgium	1995	Burns, <20% total burned surface area without lower airway burns	0.0163
Belgium	2000	Burns, <20% total burned surface area without lower airway burns	0.0163
Belgium	2005	Burns, <20% total burned surface area without lower airway burns	0.0163
Belgium	2010	Burns, <20% total burned surface area without lower airway burns	0.0163
Belgium	2017	Burns, <20% total burned surface area without lower airway burns	0.0163
Belgium	1990	Burns, >=20% total burned surface area or >= 10% burned surface area if head/neck or hands/wrist involved w/o lower airway burns	0.1346
Belgium	1995	Burns, >=20% total burned surface area or >= 10% burned surface area if head/neck or hands/wrist involved w/o lower airway burns	0.1346
Belgium	2000	Burns, >=20% total burned surface area or >= 10% burned surface area if head/neck or hands/wrist involved w/o lower airway burns	0.1346
Belgium	2005	Burns, >=20% total burned surface area or >= 10% burned surface area if head/neck or hands/wrist involved w/o lower airway burns	0.1346
Belgium	2010	Burns, >=20% total burned surface area or >= 10% burned surface area if head/neck or hands/wrist involved w/o lower airway burns	0.1346
Belgium	2017	Burns, >=20% total burned surface area or >= 10% burned surface area if head/neck or hands/wrist involved w/o lower airway burns	0.1346
Cyprus	1990	Amputation of lower limbs, bilateral	0.1463
Cyprus	1995	Amputation of lower limbs, bilateral	0.1286
Cyprus	2000	Amputation of lower limbs, bilateral	0.1232
Cyprus	2005	Amputation of lower limbs, bilateral	0.1232
Cyprus	2010	Amputation of lower limbs, bilateral	0.1232
Cyprus	2017	Amputation of lower limbs, bilateral	0.1232
Cyprus	1990	Lower airway burns	0.2252
Cyprus	1995	Lower airway burns	0.2252
Cyprus	2000	Lower airway burns	0.2252
Cyprus	2005	Lower airway burns	0.2252
Cyprus	2010	Lower airway burns	0.2252
Cyprus	2017	Lower airway burns	0.2252
Cyprus	1990	Dislocation of hip	0.0159
Cyprus	1995	Dislocation of hip	0.0159
Cyprus	2000	Dislocation of hip	0.0159
Cyprus	2005	Dislocation of hip	0.0159
Cyprus	2010	Dislocation of hip	0.0159
Cyprus	2017	Dislocation of hip	0.0159
Cyprus	1990	Dislocation of knee	0.1134
Cyprus	1995	Dislocation of knee	0.1134
Cyprus	2000	Dislocation of knee	0.1134
Cyprus	2005	Dislocation of knee	0.1134
Cyprus	2010	Dislocation of knee	0.1134
Cyprus	2017	Dislocation of knee	0.1134
Cyprus	1990	Dislocation of shoulder	0.062
Cyprus	1995	Dislocation of shoulder	0.062
Cyprus	2000	Dislocation of shoulder	0.062
Cyprus	2005	Dislocation of shoulder	0.062
Cyprus	2010	Dislocation of shoulder	0.062
Cyprus	2017	Dislocation of shoulder	0.062
Cyprus	1990	Muscle and tendon injuries, including sprains and strains lesser dislocations	0.0075
Cyprus	1995	Muscle and tendon injuries, including sprains and strains lesser dislocations	0.0075

Cyprus	2000	Muscle and tendon injuries, including sprains and strains lesser dislocations	0.0075
Cyprus	2005	Muscle and tendon injuries, including sprains and strains lesser dislocations	0.0075
Cyprus	2010	Muscle and tendon injuries, including sprains and strains lesser dislocations	0.0075
Cyprus	2017	Muscle and tendon injuries, including sprains and strains lesser dislocations	0.0075
Cyprus	1990	Fracture of clavicle, scapula, or humerus	0.0349
Cyprus	1995	Fracture of clavicle, scapula, or humerus	0.0349
Cyprus	2000	Fracture of clavicle, scapula, or humerus	0.0349
Cyprus	2005	Fracture of clavicle, scapula, or humerus	0.0349
Cyprus	2010	Fracture of clavicle, scapula, or humerus	0.0349
Cyprus	2017	Fracture of clavicle, scapula, or humerus	0.0349
Cyprus	1990	Fracture of face bones	0.0669
Cyprus	1995	Fracture of face bones	0.0669
Cyprus	2000	Fracture of face bones	0.0669
Cyprus	2005	Fracture of face bones	0.0669
Cyprus	2010	Fracture of face bones	0.0669
Cyprus	2017	Fracture of face bones	0.0669
Cyprus	1990	Fracture of foot bones except ankle	0.0264
Cyprus	1995	Fracture of foot bones except ankle	0.0264
Cyprus	2000	Fracture of foot bones except ankle	0.0264
Cyprus	2005	Fracture of foot bones except ankle	0.0264
Cyprus	2010	Fracture of foot bones except ankle	0.0264
Cyprus	2017	Fracture of foot bones except ankle	0.0264
Cyprus	1990	Fracture of hand (wrist and other distal part of hand)	0.0138
Cyprus	1995	Fracture of hand (wrist and other distal part of hand)	0.0138
Cyprus	2000	Fracture of hand (wrist and other distal part of hand)	0.0138
Cyprus	2005	Fracture of hand (wrist and other distal part of hand)	0.0138
Cyprus	2010	Fracture of hand (wrist and other distal part of hand)	0.0138
Cyprus	2017	Fracture of hand (wrist and other distal part of hand)	0.0138
Cyprus	1990	Fracture of hip	0.0882
Cyprus	1995	Fracture of hip	0.0648
Cyprus	2000	Fracture of hip	0.0577
Cyprus	2005	Fracture of hip	0.0577
Cyprus	2010	Fracture of hip	0.0577
Cyprus	2017	Fracture of hip	0.0577
Cyprus	1990	Amputation of upper limbs, bilateral	0.1191
Cyprus	1995	Amputation of upper limbs, bilateral	0.095
Cyprus	2000	Amputation of upper limbs, bilateral	0.0876
Cyprus	2005	Amputation of upper limbs, bilateral	0.0876
Cyprus	2010	Amputation of upper limbs, bilateral	0.0876
Cyprus	2017	Amputation of upper limbs, bilateral	0.0876
Cyprus	1990	Fracture of patella, tibia or fibula, or ankle	0.0554
Cyprus	1995	Fracture of patella, tibia or fibula, or ankle	0.0554
Cyprus	2000	Fracture of patella, tibia or fibula, or ankle	0.0554
Cyprus	2005	Fracture of patella, tibia or fibula, or ankle	0.0554
Cyprus	2010	Fracture of patella, tibia or fibula, or ankle	0.0554
Cyprus	2017	Fracture of patella, tibia or fibula, or ankle	0.0554
Cyprus	1990	Fracture of pelvis	0.1823
Cyprus	1995	Fracture of pelvis	0.1823
Cyprus	2000	Fracture of pelvis	0.1823
Cyprus	2005	Fracture of pelvis	0.1823
Cyprus	2010	Fracture of pelvis	0.1823
Cyprus	2017	Fracture of pelvis	0.1823
Cyprus	1990	Fracture of radius and/or ulna	0.0435
Cyprus	1995	Fracture of radius and/or ulna	0.0435
Cyprus	2000	Fracture of radius and/or ulna	0.0435
Cyprus	2005	Fracture of radius and/or ulna	0.0435
Cyprus	2010	Fracture of radius and/or ulna	0.0435
Cyprus	2017	Fracture of radius and/or ulna	0.0435
Cyprus	1990	Fracture of skull	0.0714
Cyprus	1995	Fracture of skull	0.0714
Cyprus	2000	Fracture of skull	0.0714
Cyprus	2005	Fracture of skull	0.0714
Cyprus	2010	Fracture of skull	0.0714
Cyprus	2017	Fracture of skull	0.0714
Cyprus	1990	Fracture of sternum and/or fracture of one or more ribs	0.1027
Cyprus	1995	Fracture of sternum and/or fracture of one or more ribs	0.1027
Cyprus	2000	Fracture of sternum and/or fracture of one or more ribs	0.1027
Cyprus	2005	Fracture of sternum and/or fracture of one or more ribs	0.1027
Cyprus	2010	Fracture of sternum and/or fracture of one or more ribs	0.1027
Cyprus	2017	Fracture of sternum and/or fracture of one or more ribs	0.1027
Cyprus	1990	Fracture of vertebral column	0.1106
Cyprus	1995	Fracture of vertebral column	0.1106
Cyprus	2000	Fracture of vertebral column	0.1106
Cyprus	2005	Fracture of vertebral column	0.1106
Cyprus	2010	Fracture of vertebral column	0.1106
Cyprus	2017	Fracture of vertebral column	0.1106
Cyprus	1990	Fracture of femur, other than femoral neck	0.042
Cyprus	1995	Fracture of femur, other than femoral neck	0.042
Cyprus	2000	Fracture of femur, other than femoral neck	0.042
Cyprus	2005	Fracture of femur, other than femoral neck	0.042
Cyprus	2010	Fracture of femur, other than femoral neck	0.042
Cyprus	2017	Fracture of femur, other than femoral neck	0.042
Cyprus	1990	Minor TBI	0.1319
Cyprus	1995	Minor TBI	0.1319
Cyprus	2000	Minor TBI	0.1319
Cyprus	2005	Minor TBI	0.1319
Cyprus	2010	Minor TBI	0.1319
Cyprus	2017	Minor TBI	0.1319
Cyprus	1990	Moderate TBI	0.1637
Cyprus	1995	Moderate TBI	0.1637
Cyprus	2000	Moderate TBI	0.1637
Cyprus	2005	Moderate TBI	0.1637
Cyprus	2010	Moderate TBI	0.1637
Cyprus	2017	Moderate TBI	0.1637
Cyprus	1990	Amputation of fingers (excluding thumb)	0.0047
Cyprus	1995	Amputation of fingers (excluding thumb)	0.0047
Cyprus	2000	Amputation of fingers (excluding thumb)	0.0047
Cyprus	2005	Amputation of fingers (excluding thumb)	0.0047
Cyprus	2010	Amputation of fingers (excluding thumb)	0.0047
Cyprus	2017	Amputation of fingers (excluding thumb)	0.0047
Cyprus	1990	Spinal cord lesion at neck level	0.4224
Cyprus	1995	Spinal cord lesion at neck level	0.4143
Cyprus	2000	Spinal cord lesion at neck level	0.4118
Cyprus	2005	Spinal cord lesion at neck level	0.4118
Cyprus	2010	Spinal cord lesion at neck level	0.4118
Cyprus	2017	Spinal cord lesion at neck level	0.4118
Cyprus	1990	Spinal cord lesion below neck level	0.1999
Cyprus	1995	Spinal cord lesion below neck level	0.1829
Cyprus	2000	Spinal cord lesion below neck level	0.1777
Cyprus	2005	Spinal cord lesion below neck level	0.1777
Cyprus	2010	Spinal cord lesion below neck level	0.1777
Cyprus	2017	Spinal cord lesion below neck level	0.1777
Cyprus	1990	Drowning and nonfatal submersion	0.2471
Cyprus	1995	Drowning and nonfatal submersion	0.2471
Cyprus	2000	Drowning and nonfatal submersion	0.2471
Cyprus	2005	Drowning and nonfatal submersion	0.2471
Cyprus	2010	Drowning and nonfatal submersion	0.2471
Cyprus	2017	Drowning and nonfatal submersion	0.2471
Cyprus	1990	Asphyxiation	0.2471
Cyprus	1995	Asphyxiation	0.2471
Cyprus	2000	Asphyxiation	0.2471
Cyprus	2005	Asphyxiation	0.2471
Cyprus	2010	Asphyxiation	0.2471
Cyprus	2017	Asphyxiation	0.2471
Cyprus	1990	Crush injury	0.1325
Cyprus	1995	Crush injury	0.1325
Cyprus	2000	Crush injury	0.1325
Cyprus	2005	Crush injury	0.1325
Cyprus	2010	Crush injury	0.1325
Cyprus	2017	Crush injury	0.1325
Cyprus	1990	Nerve injury	0.1129
Cyprus	1995	Nerve injury	0.1129
Cyprus	2000	Nerve injury	0.1129
Cyprus	2005	Nerve injury	0.1129
Cyprus	2010	Nerve injury	0.1129
Cyprus	2017	Nerve injury	0.1129
Cyprus	1990	Injury to eyes	0.0165
Cyprus	1995	Injury to eyes	0.0165
Cyprus	2000	Injury to eyes	0.0165
Cyprus	2005	Injury to eyes	0.0165
Cyprus	2010	Injury to eyes	0.0165
Cyprus	2017	Injury to eyes	0.0165
Cyprus	1990	Amputation of lower limb, unilateral	0.0508
Cyprus	1995	Amputation of lower limb, unilateral	0.0417
Cyprus	2000	Amputation of lower limb, unilateral	0.039
Cyprus	2005	Amputation of lower limb, unilateral	0.039
Cyprus	2010	Amputation of lower limb, unilateral	0.039
Cyprus	2017	Amputation of lower limb, unilateral	0.039
Cyprus	1990	Open wound(s)	0.011
Cyprus	1995	Open wound(s)	0.011
Cyprus	2000	Open wound(s)	0.011
Cyprus	2005	Open wound(s)	0.011
Cyprus	2010	Open wound(s)	0.011
Cyprus	2017	Open wound(s)	0.011
Cyprus	1990	Poisoning requiring urgent care	0.1628
Cyprus	1995	Poisoning requiring urgent care	0.1628
Cyprus	2000	Poisoning requiring urgent care	0.1628
Cyprus	2005	Poisoning requiring urgent care	0.1628
Cyprus	2010	Poisoning requiring urgent care	0.1628
Cyprus	2017	Poisoning requiring urgent care	0.1628
Cyprus	1990	Severe chest injury	0.0474
Cyprus	1995	Severe chest injury	0.0474
Cyprus	2000	Severe chest injury	0.0474
Cyprus	2005	Severe chest injury	0.0474
Cyprus	2010	Severe chest injury	0.0474
Cyprus	2017	Severe chest injury	0.0474
Cyprus	1990	Internal hemorrhage in abdomen and pelvis	0.1143
Cyprus	1995	Internal hemorrhage in abdomen and pelvis	0.1143
Cyprus	2000	Internal hemorrhage in abdomen and pelvis	0.1143
Cyprus	2005	Internal hemorrhage in abdomen and pelvis	0.1143
Cyprus	2010	Internal hemorrhage in abdomen and pelvis	0.1143
Cyprus	2017	Internal hemorrhage in abdomen and pelvis	0.1143
Cyprus	1990	Contusion in any part of the body	0.0075
Cyprus	1995	Contusion in any part of the body	0.0075
Cyprus	2000	Contusion in any part of the body	0.0075
Cyprus	2005	Contusion in any part of the body	0.0075
Cyprus	2010	Contusion in any part of the body	0.0075
Cyprus	2017	Contusion in any part of the body	0.0075
Cyprus	1990	Effect of different environmental factors	0.049
Cyprus	1995	Effect of different environmental factors	0.049
Cyprus	2000	Effect of different environmental factors	0.049
Cyprus	2005	Effect of different environmental factors	0.049
Cyprus	2010	Effect of different environmental factors	0.049
Cyprus	2017	Effect of different environmental factors	0.049
Cyprus	1990	Complications following therapeutic procedures	0.0506
Cyprus	1995	Complications following therapeutic procedures	0.0506
Cyprus	2000	Complications following therapeutic procedures	0.0506
Cyprus	2005	Complications following therapeutic procedures	0.0506
Cyprus	2010	Complications following therapeutic procedures	0.0506
Cyprus	2017	Complications following therapeutic procedures	0.0506
Cyprus	1990	Multiple fractures, dislocations, crashes, wounds, sprains, and strains	0.0882
Cyprus	1995	Multiple fractures, dislocations, crashes, wounds, sprains, and strains	0.0648
Cyprus	2000	Multiple fractures, dislocations, crashes, wounds, sprains, and strains	0.0577
Cyprus	2005	Multiple fractures, dislocations, crashes, wounds, sprains, and strains	0.0577
Cyprus	2010	Multiple fractures, dislocations, crashes, wounds, sprains, and strains	0.0577
Cyprus	2017	Multiple fractures, dislocations, crashes, wounds, sprains, and strains	0.0577
Cyprus	1990	Amputation of upper limb, unilateral	0.046
Cyprus	1995	Amputation of upper limb, unilateral	0.0406
Cyprus	2000	Amputation of upper limb, unilateral	0.039
Cyprus	2005	Amputation of upper limb, unilateral	0.039
Cyprus	2010	Amputation of upper limb, unilateral	0.039
Cyprus	2017	Amputation of upper limb, unilateral	0.039
Cyprus	1990	Amputation of thumb	0.0114
Cyprus	1995	Amputation of thumb	0.0114
Cyprus	2000	Amputation of thumb	0.0114
Cyprus	2005	Amputation of thumb	0.0114
Cyprus	2010	Amputation of thumb	0.0114
Cyprus	2017	Amputation of thumb	0.0114
Cyprus	1990	Amputation of toe/toes	0.0059
Cyprus	1995	Amputation of toe/toes	0.0059
Cyprus	2000	Amputation of toe/toes	0.0059
Cyprus	2005	Amputation of toe/toes	0.0059
Cyprus	2010	Amputation of toe/toes	0.0059
Cyprus	2017	Amputation of toe/toes	0.0059
Cyprus	1990	Burns, <20% total burned surface area without lower airway burns	0.0163
Cyprus	1995	Burns, <20% total burned surface area without lower airway burns	0.0163
Cyprus	2000	Burns, <20% total burned surface area without lower airway burns	0.0163
Cyprus	2005	Burns, <20% total burned surface area without lower airway burns	0.0163
Cyprus	2010	Burns, <20% total burned surface area without lower airway burns	0.0163
Cyprus	2017	Burns, <20% total burned surface area without lower airway burns	0.0163
Cyprus	1990	Burns, >=20% total burned surface area or >= 10% burned surface area if head/neck or hands/wrist involved w/o lower airway burns	0.163
Cyprus	1995	Burns, >=20% total burned surface area or >= 10% burned surface area if head/neck or hands/wrist involved w/o lower airway burns	0.1412
Cyprus	2000	Burns, >=20% total burned surface area or >= 10% burned surface area if head/neck or hands/wrist involved w/o lower airway burns	0.1346
Cyprus	2005	Burns, >=20% total burned surface area or >= 10% burned surface area if head/neck or hands/wrist involved w/o lower airway burns	0.1346
Cyprus	2010	Burns, >=20% total burned surface area or >= 10% burned surface area if head/neck or hands/wrist involved w/o lower airway burns	0.1346
Cyprus	2017	Burns, >=20% total burned surface area or >= 10% burned surface area if head/neck or hands/wrist involved w/o lower airway burns	0.1346
Denmark	1990	Amputation of lower limbs, bilateral	0.1232
Denmark	1995	Amputation of lower limbs, bilateral	0.1232
Denmark	2000	Amputation of lower limbs, bilateral	0.1232
Denmark	2005	Amputation of lower limbs, bilateral	0.1232
Denmark	2010	Amputation of lower limbs, bilateral	0.1232
Denmark	2017	Amputation of lower limbs, bilateral	0.1232
Denmark	1990	Lower airway burns	0.2252
Denmark	1995	Lower airway burns	0.2252
Denmark	2000	Lower airway burns	0.2252
Denmark	2005	Lower airway burns	0.2252
Denmark	2010	Lower airway burns	0.2252
Denmark	2017	Lower airway burns	0.2252
Denmark	1990	Dislocation of hip	0.0159
Denmark	1995	Dislocation of hip	0.0159
Denmark	2000	Dislocation of hip	0.0159
Denmark	2005	Dislocation of hip	0.0159
Denmark	2010	Dislocation of hip	0.0159
Denmark	2017	Dislocation of hip	0.0159
Denmark	1990	Dislocation of knee	0.1134
Denmark	1995	Dislocation of knee	0.1134
Denmark	2000	Dislocation of knee	0.1134
Denmark	2005	Dislocation of knee	0.1134
Denmark	2010	Dislocation of knee	0.1134
Denmark	2017	Dislocation of knee	0.1134
Denmark	1990	Dislocation of shoulder	0.062
Denmark	1995	Dislocation of shoulder	0.062
Denmark	2000	Dislocation of shoulder	0.062
Denmark	2005	Dislocation of shoulder	0.062
Denmark	2010	Dislocation of shoulder	0.062
Denmark	2017	Dislocation of shoulder	0.062
Denmark	1990	Muscle and tendon injuries, including sprains and strains lesser dislocations	0.0075
Denmark	1995	Muscle and tendon injuries, including sprains and strains lesser dislocations	0.0075
Denmark	2000	Muscle and tendon injuries, including sprains and strains lesser dislocations	0.0075
Denmark	2005	Muscle and tendon injuries, including sprains and strains lesser dislocations	0.0075
Denmark	2010	Muscle and tendon injuries, including sprains and strains lesser dislocations	0.0075
Denmark	2017	Muscle and tendon injuries, including sprains and strains lesser dislocations	0.0075
Denmark	1990	Fracture of clavicle, scapula, or humerus	0.0349
Denmark	1995	Fracture of clavicle, scapula, or humerus	0.0349
Denmark	2000	Fracture of clavicle, scapula, or humerus	0.0349
Denmark	2005	Fracture of clavicle, scapula, or humerus	0.0349
Denmark	2010	Fracture of clavicle, scapula, or humerus	0.0349
Denmark	2017	Fracture of clavicle, scapula, or humerus	0.0349
Denmark	1990	Fracture of face bones	0.0669
Denmark	1995	Fracture of face bones	0.0669
Denmark	2000	Fracture of face bones	0.0669
Denmark	2005	Fracture of face bones	0.0669
Denmark	2010	Fracture of face bones	0.0669
Denmark	2017	Fracture of face bones	0.0669
Denmark	1990	Fracture of foot bones except ankle	0.0264
Denmark	1995	Fracture of foot bones except ankle	0.0264
Denmark	2000	Fracture of foot bones except ankle	0.0264
Denmark	2005	Fracture of foot bones except ankle	0.0264
Denmark	2010	Fracture of foot bones except ankle	0.0264
Denmark	2017	Fracture of foot bones except ankle	0.0264
Denmark	1990	Fracture of hand (wrist and other distal part of hand)	0.0138
Denmark	1995	Fracture of hand (wrist and other distal part of hand)	0.0138
Denmark	2000	Fracture of hand (wrist and other distal part of hand)	0.0138
Denmark	2005	Fracture of hand (wrist and other distal part of hand)	0.0138
Denmark	2010	Fracture of hand (wrist and other distal part of hand)	0.0138
Denmark	2017	Fracture of hand (wrist and other distal part of hand)	0.0138
Denmark	1990	Fracture of hip	0.0577
Denmark	1995	Fracture of hip	0.0577
Denmark	2000	Fracture of hip	0.0577
Denmark	2005	Fracture of hip	0.0577
Denmark	2010	Fracture of hip	0.0577
Denmark	2017	Fracture of hip	0.0577
Denmark	1990	Amputation of upper limbs, bilateral	0.0876
Denmark	1995	Amputation of upper limbs, bilateral	0.0876
Denmark	2000	Amputation of upper limbs, bilateral	0.0876
Denmark	2005	Amputation of upper limbs, bilateral	0.0876
Denmark	2010	Amputation of upper limbs, bilateral	0.0876
Denmark	2017	Amputation of upper limbs, bilateral	0.0876
Denmark	1990	Fracture of patella, tibia or fibula, or ankle	0.0554
Denmark	1995	Fracture of patella, tibia or fibula, or ankle	0.0554
Denmark	2000	Fracture of patella, tibia or fibula, or ankle	0.0554
Denmark	2005	Fracture of patella, tibia or fibula, or ankle	0.0554
Denmark	2010	Fracture of patella, tibia or fibula, or ankle	0.0554
Denmark	2017	Fracture of patella, tibia or fibula, or ankle	0.0554
Denmark	1990	Fracture of pelvis	0.1823
Denmark	1995	Fracture of pelvis	0.1823
Denmark	2000	Fracture of pelvis	0.1823
Denmark	2005	Fracture of pelvis	0.1823
Denmark	2010	Fracture of pelvis	0.1823
Denmark	2017	Fracture of pelvis	0.1823
Denmark	1990	Fracture of radius and/or ulna	0.0435
Denmark	1995	Fracture of radius and/or ulna	0.0435
Denmark	2000	Fracture of radius and/or ulna	0.0435
Denmark	2005	Fracture of radius and/or ulna	0.0435
Denmark	2010	Fracture of radius and/or ulna	0.0435
Denmark	2017	Fracture of radius and/or ulna	0.0435
Denmark	1990	Fracture of skull	0.0714
Denmark	1995	Fracture of skull	0.0714
Denmark	2000	Fracture of skull	0.0714
Denmark	2005	Fracture of skull	0.0714
Denmark	2010	Fracture of skull	0.0714
Denmark	2017	Fracture of skull	0.0714
Denmark	1990	Fracture of sternum and/or fracture of one or more ribs	0.1027
Denmark	1995	Fracture of sternum and/or fracture of one or more ribs	0.1027
Denmark	2000	Fracture of sternum and/or fracture of one or more ribs	0.1027
Denmark	2005	Fracture of sternum and/or fracture of one or more ribs	0.1027
Denmark	2010	Fracture of sternum and/or fracture of one or more ribs	0.1027
Denmark	2017	Fracture of sternum and/or fracture of one or more ribs	0.1027
Denmark	1990	Fracture of vertebral column	0.1106
Denmark	1995	Fracture of vertebral column	0.1106
Denmark	2000	Fracture of vertebral column	0.1106
Denmark	2005	Fracture of vertebral column	0.1106
Denmark	2010	Fracture of vertebral column	0.1106
Denmark	2017	Fracture of vertebral column	0.1106
Denmark	1990	Fracture of femur, other than femoral neck	0.042
Denmark	1995	Fracture of femur, other than femoral neck	0.042
Denmark	2000	Fracture of femur, other than femoral neck	0.042
Denmark	2005	Fracture of femur, other than femoral neck	0.042
Denmark	2010	Fracture of femur, other than femoral neck	0.042
Denmark	2017	Fracture of femur, other than femoral neck	0.042
Denmark	1990	Minor TBI	0.1319
Denmark	1995	Minor TBI	0.1319
Denmark	2000	Minor TBI	0.1319
Denmark	2005	Minor TBI	0.1319
Denmark	2010	Minor TBI	0.1319
Denmark	2017	Minor TBI	0.1319
Denmark	1990	Moderate TBI	0.1637
Denmark	1995	Moderate TBI	0.1637
Denmark	2000	Moderate TBI	0.1637
Denmark	2005	Moderate TBI	0.1637
Denmark	2010	Moderate TBI	0.1637
Denmark	2017	Moderate TBI	0.1637
Denmark	1990	Amputation of fingers (excluding thumb)	0.0047
Denmark	1995	Amputation of fingers (excluding thumb)	0.0047
Denmark	2000	Amputation of fingers (excluding thumb)	0.0047
Denmark	2005	Amputation of fingers (excluding thumb)	0.0047
Denmark	2010	Amputation of fingers (excluding thumb)	0.0047
Denmark	2017	Amputation of fingers (excluding thumb)	0.0047
Denmark	1990	Spinal cord lesion at neck level	0.4118
Denmark	1995	Spinal cord lesion at neck level	0.4118
Denmark	2000	Spinal cord lesion at neck level	0.4118
Denmark	2005	Spinal cord lesion at neck level	0.4118
Denmark	2010	Spinal cord lesion at neck level	0.4118
Denmark	2017	Spinal cord lesion at neck level	0.4118
Denmark	1990	Spinal cord lesion below neck level	0.1777
Denmark	1995	Spinal cord lesion below neck level	0.1777
Denmark	2000	Spinal cord lesion below neck level	0.1777
Denmark	2005	Spinal cord lesion below neck level	0.1777
Denmark	2010	Spinal cord lesion below neck level	0.1777
Denmark	2017	Spinal cord lesion below neck level	0.1777
Denmark	1990	Drowning and nonfatal submersion	0.2471
Denmark	1995	Drowning and nonfatal submersion	0.2471
Denmark	2000	Drowning and nonfatal submersion	0.2471
Denmark	2005	Drowning and nonfatal submersion	0.2471
Denmark	2010	Drowning and nonfatal submersion	0.2471
Denmark	2017	Drowning and nonfatal submersion	0.2471
Denmark	1990	Asphyxiation	0.2471
Denmark	1995	Asphyxiation	0.2471
Denmark	2000	Asphyxiation	0.2471
Denmark	2005	Asphyxiation	0.2471
Denmark	2010	Asphyxiation	0.2471
Denmark	2017	Asphyxiation	0.2471
Denmark	1990	Crush injury	0.1325
Denmark	1995	Crush injury	0.1325
Denmark	2000	Crush injury	0.1325
Denmark	2005	Crush injury	0.1325
Denmark	2010	Crush injury	0.1325
Denmark	2017	Crush injury	0.1325
Denmark	1990	Nerve injury	0.1129
Denmark	1995	Nerve injury	0.1129
Denmark	2000	Nerve injury	0.1129
Denmark	2005	Nerve injury	0.1129
Denmark	2010	Nerve injury	0.1129
Denmark	2017	Nerve injury	0.1129
Denmark	1990	Injury to eyes	0.0165
Denmark	1995	Injury to eyes	0.0165
Denmark	2000	Injury to eyes	0.0165
Denmark	2005	Injury to eyes	0.0165
Denmark	2010	Injury to eyes	0.0165
Denmark	2017	Injury to eyes	0.0165
Denmark	1990	Amputation of lower limb, unilateral	0.039
Denmark	1995	Amputation of lower limb, unilateral	0.039
Denmark	2000	Amputation of lower limb, unilateral	0.039
Denmark	2005	Amputation of lower limb, unilateral	0.039
Denmark	2010	Amputation of lower limb, unilateral	0.039
Denmark	2017	Amputation of lower limb, unilateral	0.039
Denmark	1990	Open wound(s)	0.011
Denmark	1995	Open wound(s)	0.011
Denmark	2000	Open wound(s)	0.011
Denmark	2005	Open wound(s)	0.011
Denmark	2010	Open wound(s)	0.011
Denmark	2017	Open wound(s)	0.011
Denmark	1990	Poisoning requiring urgent care	0.1628
Denmark	1995	Poisoning requiring urgent care	0.1628
Denmark	2000	Poisoning requiring urgent care	0.1628
Denmark	2005	Poisoning requiring urgent care	0.1628
Denmark	2010	Poisoning requiring urgent care	0.1628
Denmark	2017	Poisoning requiring urgent care	0.1628
Denmark	1990	Severe chest injury	0.0474
Denmark	1995	Severe chest injury	0.0474
Denmark	2000	Severe chest injury	0.0474
Denmark	2005	Severe chest injury	0.0474
Denmark	2010	Severe chest injury	0.0474
Denmark	2017	Severe chest injury	0.0474
Denmark	1990	Internal hemorrhage in abdomen and pelvis	0.1143
Denmark	1995	Internal hemorrhage in abdomen and pelvis	0.1143
Denmark	2000	Internal hemorrhage in abdomen and pelvis	0.1143
Denmark	2005	Internal hemorrhage in abdomen and pelvis	0.1143
Denmark	2010	Internal hemorrhage in abdomen and pelvis	0.1143
Denmark	2017	Internal hemorrhage in abdomen and pelvis	0.1143
Denmark	1990	Contusion in any part of the body	0.0075

Denmark	1995	Contusion in any part of the body	0.0075
Denmark	2000	Contusion in any part of the body	0.0075
Denmark	2005	Contusion in any part of the body	0.0075
Denmark	2010	Contusion in any part of the body	0.0075
Denmark	2017	Contusion in any part of the body	0.0075
Denmark	1990	Effect of different environmental factors	0.049
Denmark	1995	Effect of different environmental factors	0.049
Denmark	2000	Effect of different environmental factors	0.049
Denmark	2005	Effect of different environmental factors	0.049
Denmark	2010	Effect of different environmental factors	0.049
Denmark	2017	Effect of different environmental factors	0.049
Denmark	1990	Complications following therapeutic procedures	0.0506
Denmark	1995	Complications following therapeutic procedures	0.0506
Denmark	2000	Complications following therapeutic procedures	0.0506
Denmark	2005	Complications following therapeutic procedures	0.0506
Denmark	2010	Complications following therapeutic procedures	0.0506
Denmark	2017	Complications following therapeutic procedures	0.0506
Denmark	1990	Multiple fractures, dislocations, crashes, wounds, sprains, and strains	0.0577
Denmark	1995	Multiple fractures, dislocations, crashes, wounds, sprains, and strains	0.0577
Denmark	2000	Multiple fractures, dislocations, crashes, wounds, sprains, and strains	0.0577
Denmark	2005	Multiple fractures, dislocations, crashes, wounds, sprains, and strains	0.0577
Denmark	2010	Multiple fractures, dislocations, crashes, wounds, sprains, and strains	0.0577
Denmark	2017	Multiple fractures, dislocations, crashes, wounds, sprains, and strains	0.0577
Denmark	1990	Amputation of upper limb, unilateral	0.039
Denmark	1995	Amputation of upper limb, unilateral	0.039
Denmark	2000	Amputation of upper limb, unilateral	0.039
Denmark	2005	Amputation of upper limb, unilateral	0.039
Denmark	2010	Amputation of upper limb, unilateral	0.039
Denmark	2017	Amputation of upper limb, unilateral	0.039
Denmark	1990	Amputation of thumb	0.0114
Denmark	1995	Amputation of thumb	0.0114
Denmark	2000	Amputation of thumb	0.0114
Denmark	2005	Amputation of thumb	0.0114
Denmark	2010	Amputation of thumb	0.0114
Denmark	2017	Amputation of thumb	0.0114
Denmark	1990	Amputation of toe/toes	0.0059
Denmark	1995	Amputation of toe/toes	0.0059
Denmark	2000	Amputation of toe/toes	0.0059
Denmark	2005	Amputation of toe/toes	0.0059
Denmark	2010	Amputation of toe/toes	0.0059
Denmark	2017	Amputation of toe/toes	0.0059
Denmark	1990	Burns, <20% total burned surface area without lower airway burns	0.0163
Denmark	1995	Burns, <20% total burned surface area without lower airway burns	0.0163
Denmark	2000	Burns, <20% total burned surface area without lower airway burns	0.0163
Denmark	2005	Burns, <20% total burned surface area without lower airway burns	0.0163
Denmark	2010	Burns, <20% total burned surface area without lower airway burns	0.0163
Denmark	2017	Burns, <20% total burned surface area without lower airway burns	0.0163
Denmark	1990	Burns, >=20% total burned surface area or >= 10% burned surface area if head/neck or hands/wrist involved w/o lower airway burns	0.1346
Denmark	1995	Burns, >=20% total burned surface area or >= 10% burned surface area if head/neck or hands/wrist involved w/o lower airway burns	0.1346
Denmark	2000	Burns, >=20% total burned surface area or >= 10% burned surface area if head/neck or hands/wrist involved w/o lower airway burns	0.1346
Denmark	2005	Burns, >=20% total burned surface area or >= 10% burned surface area if head/neck or hands/wrist involved w/o lower airway burns	0.1346
Denmark	2010	Burns, >=20% total burned surface area or >= 10% burned surface area if head/neck or hands/wrist involved w/o lower airway burns	0.1346
Denmark	2017	Burns, >=20% total burned surface area or >= 10% burned surface area if head/neck or hands/wrist involved w/o lower airway burns	0.1346
Finland	1990	Amputation of lower limbs, bilateral	0.1232
Finland	1995	Amputation of lower limbs, bilateral	0.1232
Finland	2000	Amputation of lower limbs, bilateral	0.1232
Finland	2005	Amputation of lower limbs, bilateral	0.1232
Finland	2010	Amputation of lower limbs, bilateral	0.1232
Finland	2017	Amputation of lower limbs, bilateral	0.1232
Finland	1990	Lower airway burns	0.2252
Finland	1995	Lower airway burns	0.2252
Finland	2000	Lower airway burns	0.2252
Finland	2005	Lower airway burns	0.2252
Finland	2010	Lower airway burns	0.2252
Finland	2017	Lower airway burns	0.2252
Finland	1990	Dislocation of hip	0.0159
Finland	1995	Dislocation of hip	0.0159
Finland	2000	Dislocation of hip	0.0159
Finland	2005	Dislocation of hip	0.0159
Finland	2010	Dislocation of hip	0.0159
Finland	2017	Dislocation of hip	0.0159
Finland	1990	Dislocation of knee	0.1134
Finland	1995	Dislocation of knee	0.1134
Finland	2000	Dislocation of knee	0.1134
Finland	2005	Dislocation of knee	0.1134
Finland	2010	Dislocation of knee	0.1134
Finland	2017	Dislocation of knee	0.1134
Finland	1990	Dislocation of shoulder	0.062
Finland	1995	Dislocation of shoulder	0.062
Finland	2000	Dislocation of shoulder	0.062
Finland	2005	Dislocation of shoulder	0.062
Finland	2010	Dislocation of shoulder	0.062
Finland	2017	Dislocation of shoulder	0.062
Finland	1990	Muscle and tendon injuries, including sprains and strains lesser dislocations	0.0075
Finland	1995	Muscle and tendon injuries, including sprains and strains lesser dislocations	0.0075
Finland	2000	Muscle and tendon injuries, including sprains and strains lesser dislocations	0.0075
Finland	2005	Muscle and tendon injuries, including sprains and strains lesser dislocations	0.0075
Finland	2010	Muscle and tendon injuries, including sprains and strains lesser dislocations	0.0075
Finland	2017	Muscle and tendon injuries, including sprains and strains lesser dislocations	0.0075
Finland	1990	Fracture of clavicle, scapula, or humerus	0.0349
Finland	1995	Fracture of clavicle, scapula, or humerus	0.0349
Finland	2000	Fracture of clavicle, scapula, or humerus	0.0349
Finland	2005	Fracture of clavicle, scapula, or humerus	0.0349
Finland	2010	Fracture of clavicle, scapula, or humerus	0.0349
Finland	2017	Fracture of clavicle, scapula, or humerus	0.0349
Finland	1990	Fracture of face bones	0.0669
Finland	1995	Fracture of face bones	0.0669
Finland	2000	Fracture of face bones	0.0669
Finland	2005	Fracture of face bones	0.0669
Finland	2010	Fracture of face bones	0.0669
Finland	2017	Fracture of face bones	0.0669
Finland	1990	Fracture of foot bones except ankle	0.0264
Finland	1995	Fracture of foot bones except ankle	0.0264
Finland	2000	Fracture of foot bones except ankle	0.0264
Finland	2005	Fracture of foot bones except ankle	0.0264
Finland	2010	Fracture of foot bones except ankle	0.0264
Finland	2017	Fracture of foot bones except ankle	0.0264
Finland	1990	Fracture of hand (wrist and other distal part of hand)	0.0138
Finland	1995	Fracture of hand (wrist and other distal part of hand)	0.0138
Finland	2000	Fracture of hand (wrist and other distal part of hand)	0.0138
Finland	2005	Fracture of hand (wrist and other distal part of hand)	0.0138
Finland	2010	Fracture of hand (wrist and other distal part of hand)	0.0138
Finland	2017	Fracture of hand (wrist and other distal part of hand)	0.0138
Finland	1990	Fracture of hip	0.0577
Finland	1995	Fracture of hip	0.0577
Finland	2000	Fracture of hip	0.0577
Finland	2005	Fracture of hip	0.0577
Finland	2010	Fracture of hip	0.0577
Finland	2017	Fracture of hip	0.0577
Finland	1990	Amputation of upper limbs, bilateral	0.0876
Finland	1995	Amputation of upper limbs, bilateral	0.0876
Finland	2000	Amputation of upper limbs, bilateral	0.0876
Finland	2005	Amputation of upper limbs, bilateral	0.0876
Finland	2010	Amputation of upper limbs, bilateral	0.0876
Finland	2017	Amputation of upper limbs, bilateral	0.0876
Finland	1990	Fracture of patella, tibia or fibula, or ankle	0.0554
Finland	1995	Fracture of patella, tibia or fibula, or ankle	0.0554
Finland	2000	Fracture of patella, tibia or fibula, or ankle	0.0554
Finland	2005	Fracture of patella, tibia or fibula, or ankle	0.0554
Finland	2010	Fracture of patella, tibia or fibula, or ankle	0.0554
Finland	2017	Fracture of patella, tibia or fibula, or ankle	0.0554
Finland	1990	Fracture of pelvis	0.1823
Finland	1995	Fracture of pelvis	0.1823
Finland	2000	Fracture of pelvis	0.1823
Finland	2005	Fracture of pelvis	0.1823
Finland	2010	Fracture of pelvis	0.1823
Finland	2017	Fracture of pelvis	0.1823
Finland	1990	Fracture of radius and/or ulna	0.0435
Finland	1995	Fracture of radius and/or ulna	0.0435
Finland	2000	Fracture of radius and/or ulna	0.0435
Finland	2005	Fracture of radius and/or ulna	0.0435
Finland	2010	Fracture of radius and/or ulna	0.0435
Finland	2017	Fracture of radius and/or ulna	0.0435
Finland	1990	Fracture of skull	0.0714
Finland	1995	Fracture of skull	0.0714
Finland	2000	Fracture of skull	0.0714
Finland	2005	Fracture of skull	0.0714
Finland	2010	Fracture of skull	0.0714
Finland	2017	Fracture of skull	0.0714
Finland	1990	Fracture of sternum and/or fracture of one or more ribs	0.1027
Finland	1995	Fracture of sternum and/or fracture of one or more ribs	0.1027
Finland	2000	Fracture of sternum and/or fracture of one or more ribs	0.1027
Finland	2005	Fracture of sternum and/or fracture of one or more ribs	0.1027
Finland	2010	Fracture of sternum and/or fracture of one or more ribs	0.1027
Finland	2017	Fracture of sternum and/or fracture of one or more ribs	0.1027
Finland	1990	Fracture of vertebral column	0.1106
Finland	1995	Fracture of vertebral column	0.1106
Finland	2000	Fracture of vertebral column	0.1106
Finland	2005	Fracture of vertebral column	0.1106
Finland	2010	Fracture of vertebral column	0.1106
Finland	2017	Fracture of vertebral column	0.1106
Finland	1990	Fracture of femur, other than femoral neck	0.042
Finland	1995	Fracture of femur, other than femoral neck	0.042
Finland	2000	Fracture of femur, other than femoral neck	0.042
Finland	2005	Fracture of femur, other than femoral neck	0.042
Finland	2010	Fracture of femur, other than femoral neck	0.042
Finland	2017	Fracture of femur, other than femoral neck	0.042
Finland	1990	Minor TBI	0.1319
Finland	1995	Minor TBI	0.1319
Finland	2000	Minor TBI	0.1319
Finland	2005	Minor TBI	0.1319
Finland	2010	Minor TBI	0.1319
Finland	2017	Minor TBI	0.1319
Finland	1990	Moderate TBI	0.1637
Finland	1995	Moderate TBI	0.1637
Finland	2000	Moderate TBI	0.1637
Finland	2005	Moderate TBI	0.1637
Finland	2010	Moderate TBI	0.1637
Finland	2017	Moderate TBI	0.1637
Finland	1990	Amputation of fingers (excluding thumb)	0.0047
Finland	1995	Amputation of fingers (excluding thumb)	0.0047
Finland	2000	Amputation of fingers (excluding thumb)	0.0047
Finland	2005	Amputation of fingers (excluding thumb)	0.0047
Finland	2010	Amputation of fingers (excluding thumb)	0.0047
Finland	2017	Amputation of fingers (excluding thumb)	0.0047
Finland	1990	Spinal cord lesion at neck level	0.4118
Finland	1995	Spinal cord lesion at neck level	0.4118
Finland	2000	Spinal cord lesion at neck level	0.4118
Finland	2005	Spinal cord lesion at neck level	0.4118
Finland	2010	Spinal cord lesion at neck level	0.4118
Finland	2017	Spinal cord lesion at neck level	0.4118
Finland	1990	Spinal cord lesion below neck level	0.1777
Finland	1995	Spinal cord lesion below neck level	0.1777
Finland	2000	Spinal cord lesion below neck level	0.1777
Finland	2005	Spinal cord lesion below neck level	0.1777
Finland	2010	Spinal cord lesion below neck level	0.1777
Finland	2017	Spinal cord lesion below neck level	0.1777
Finland	1990	Drowning and nonfatal submersion	0.2471
Finland	1995	Drowning and nonfatal submersion	0.2471
Finland	2000	Drowning and nonfatal submersion	0.2471
Finland	2005	Drowning and nonfatal submersion	0.2471
Finland	2010	Drowning and nonfatal submersion	0.2471
Finland	2017	Drowning and nonfatal submersion	0.2471
Finland	1990	Asphyxiation	0.2471
Finland	1995	Asphyxiation	0.2471
Finland	2000	Asphyxiation	0.2471
Finland	2005	Asphyxiation	0.2471
Finland	2010	Asphyxiation	0.2471
Finland	2017	Asphyxiation	0.2471
Finland	1990	Crush injury	0.1325
Finland	1995	Crush injury	0.1325
Finland	2000	Crush injury	0.1325
Finland	2005	Crush injury	0.1325
Finland	2010	Crush injury	0.1325
Finland	2017	Crush injury	0.1325
Finland	1990	Nerve injury	0.1129
Finland	1995	Nerve injury	0.1129
Finland	2000	Nerve injury	0.1129
Finland	2005	Nerve injury	0.1129
Finland	2010	Nerve injury	0.1129
Finland	2017	Nerve injury	0.1129
Finland	1990	Injury to eyes	0.0165
Finland	1995	Injury to eyes	0.0165
Finland	2000	Injury to eyes	0.0165
Finland	2005	Injury to eyes	0.0165
Finland	2010	Injury to eyes	0.0165
Finland	2017	Injury to eyes	0.0165
Finland	1990	Amputation of lower limb, unilateral	0.039
Finland	1995	Amputation of lower limb, unilateral	0.039
Finland	2000	Amputation of lower limb, unilateral	0.039
Finland	2005	Amputation of lower limb, unilateral	0.039
Finland	2010	Amputation of lower limb, unilateral	0.039
Finland	2017	Amputation of lower limb, unilateral	0.039
Finland	1990	Open wound(s)	0.011
Finland	1995	Open wound(s)	0.011
Finland	2000	Open wound(s)	0.011
Finland	2005	Open wound(s)	0.011
Finland	2010	Open wound(s)	0.011
Finland	2017	Open wound(s)	0.011
Finland	1990	Poisoning requiring urgent care	0.1628
Finland	1995	Poisoning requiring urgent care	0.1628
Finland	2000	Poisoning requiring urgent care	0.1628
Finland	2005	Poisoning requiring urgent care	0.1628
Finland	2010	Poisoning requiring urgent care	0.1628
Finland	2017	Poisoning requiring urgent care	0.1628
Finland	1990	Severe chest injury	0.0474
Finland	1995	Severe chest injury	0.0474
Finland	2000	Severe chest injury	0.0474
Finland	2005	Severe chest injury	0.0474
Finland	2010	Severe chest injury	0.0474
Finland	2017	Severe chest injury	0.0474
Finland	1990	Internal hemorrhage in abdomen and pelvis	0.1143
Finland	1995	Internal hemorrhage in abdomen and pelvis	0.1143
Finland	2000	Internal hemorrhage in abdomen and pelvis	0.1143
Finland	2005	Internal hemorrhage in abdomen and pelvis	0.1143
Finland	2010	Internal hemorrhage in abdomen and pelvis	0.1143
Finland	2017	Internal hemorrhage in abdomen and pelvis	0.1143
Finland	1990	Contusion in any part of the body	0.0075
Finland	1995	Contusion in any part of the body	0.0075
Finland	2000	Contusion in any part of the body	0.0075
Finland	2005	Contusion in any part of the body	0.0075
Finland	2010	Contusion in any part of the body	0.0075
Finland	2017	Contusion in any part of the body	0.0075
Finland	1990	Effect of different environmental factors	0.049
Finland	1995	Effect of different environmental factors	0.049
Finland	2000	Effect of different environmental factors	0.049
Finland	2005	Effect of different environmental factors	0.049
Finland	2010	Effect of different environmental factors	0.049
Finland	2017	Effect of different environmental factors	0.049
Finland	1990	Complications following therapeutic procedures	0.0506
Finland	1995	Complications following therapeutic procedures	0.0506
Finland	2000	Complications following therapeutic procedures	0.0506
Finland	2005	Complications following therapeutic procedures	0.0506
Finland	2010	Complications following therapeutic procedures	0.0506
Finland	2017	Complications following therapeutic procedures	0.0506
Finland	1990	Multiple fractures, dislocations, crashes, wounds, sprains, and strains	0.0577
Finland	1995	Multiple fractures, dislocations, crashes, wounds, sprains, and strains	0.0577
Finland	2000	Multiple fractures, dislocations, crashes, wounds, sprains, and strains	0.0577
Finland	2005	Multiple fractures, dislocations, crashes, wounds, sprains, and strains	0.0577
Finland	2010	Multiple fractures, dislocations, crashes, wounds, sprains, and strains	0.0577
Finland	2017	Multiple fractures, dislocations, crashes, wounds, sprains, and strains	0.0577
Finland	1990	Amputation of upper limb, unilateral	0.039
Finland	1995	Amputation of upper limb, unilateral	0.039
Finland	2000	Amputation of upper limb, unilateral	0.039
Finland	2005	Amputation of upper limb, unilateral	0.039
Finland	2010	Amputation of upper limb, unilateral	0.039
Finland	2017	Amputation of upper limb, unilateral	0.039
Finland	1990	Amputation of thumb	0.0114
Finland	1995	Amputation of thumb	0.0114
Finland	2000	Amputation of thumb	0.0114
Finland	2005	Amputation of thumb	0.0114
Finland	2010	Amputation of thumb	0.0114
Finland	2017	Amputation of thumb	0.0114
Finland	1990	Amputation of toe/toes	0.0059
Finland	1995	Amputation of toe/toes	0.0059
Finland	2000	Amputation of toe/toes	0.0059
Finland	2005	Amputation of toe/toes	0.0059
Finland	2010	Amputation of toe/toes	0.0059
Finland	2017	Amputation of toe/toes	0.0059
Finland	1990	Burns, <20% total burned surface area without lower airway burns	0.0163
Finland	1995	Burns, <20% total burned surface area without lower airway burns	0.0163
Finland	2000	Burns, <20% total burned surface area without lower airway burns	0.0163
Finland	2005	Burns, <20% total burned surface area without lower airway burns	0.0163
Finland	2010	Burns, <20% total burned surface area without lower airway burns	0.0163
Finland	2017	Burns, <20% total burned surface area without lower airway burns	0.0163
Finland	1990	Burns, >=20% total burned surface area or >= 10% burned surface area if head/neck or hands/wrist involved w/o lower airway burns	0.1346
Finland	1995	Burns, >=20% total burned surface area or >= 10% burned surface area if head/neck or hands/wrist involved w/o lower airway burns	0.1346
Finland	2000	Burns, >=20% total burned surface area or >= 10% burned surface area if head/neck or hands/wrist involved w/o lower airway burns	0.1346
Finland	2005	Burns, >=20% total burned surface area or >= 10% burned surface area if head/neck or hands/wrist involved w/o lower airway burns	0.1346
Finland	2010	Burns, >=20% total burned surface area or >= 10% burned surface area if head/neck or hands/wrist involved w/o lower airway burns	0.1346
Finland	2017	Burns, >=20% total burned surface area or >= 10% burned surface area if head/neck or hands/wrist involved w/o lower airway burns	0.1346
France	1990	Amputation of lower limbs, bilateral	0.1232
France	1995	Amputation of lower limbs, bilateral	0.1232
France	2000	Amputation of lower limbs, bilateral	0.1232
France	2005	Amputation of lower limbs, bilateral	0.1232
France	2010	Amputation of lower limbs, bilateral	0.1232
France	2017	Amputation of lower limbs, bilateral	0.1232
France	1990	Lower airway burns	0.2252
France	1995	Lower airway burns	0.2252
France	2000	Lower airway burns	0.2252
France	2005	Lower airway burns	0.2252
France	2010	Lower airway burns	0.2252
France	2017	Lower airway burns	0.2252
France	1990	Dislocation of hip	0.0159
France	1995	Dislocation of hip	0.0159
France	2000	Dislocation of hip	0.0159
France	2005	Dislocation of hip	0.0159
France	2010	Dislocation of hip	0.0159
France	2017	Dislocation of hip	0.0159
France	1990	Dislocation of knee	0.1134
France	1995	Dislocation of knee	0.1134
France	2000	Dislocation of knee	0.1134
France	2005	Dislocation of knee	0.1134
France	2010	Dislocation of knee	0.1134
France	2017	Dislocation of knee	0.1134
France	1990	Dislocation of shoulder	0.062
France	1995	Dislocation of shoulder	0.062
France	2000	Dislocation of shoulder	0.062
France	2005	Dislocation of shoulder	0.062
France	2010	Dislocation of shoulder	0.062
France	2017	Dislocation of shoulder	0.062
France	1990	Muscle and tendon injuries, including sprains and strains lesser dislocations	0.0075
France	1995	Muscle and tendon injuries, including sprains and strains lesser dislocations	0.0075
France	2000	Muscle and tendon injuries, including sprains and strains lesser dislocations	0.0075
France	2005	Muscle and tendon injuries, including sprains and strains lesser dislocations	0.0075
France	2010	Muscle and tendon injuries, including sprains and strains lesser dislocations	0.0075
France	2017	Muscle and tendon injuries, including sprains and strains lesser dislocations	0.0075
France	1990	Fracture of clavicle, scapula, or humerus	0.0349
France	1995	Fracture of clavicle, scapula, or humerus	0.0349
France	2000	Fracture of clavicle, scapula, or humerus	0.0349
France	2005	Fracture of clavicle, scapula, or humerus	0.0349
France	2010	Fracture of clavicle, scapula, or humerus	0.0349
France	2017	Fracture of clavicle, scapula, or humerus	0.0349
France	1990	Fracture of face bones	0.0669
France	1995	Fracture of face bones	0.0669
France	2000	Fracture of face bones	0.0669
France	2005	Fracture of face bones	0.0669
France	2010	Fracture of face bones	0.0669
France	2017	Fracture of face bones	0.0669
France	1990	Fracture of foot bones except ankle	0.0264
France	1995	Fracture of foot bones except ankle	0.0264
France	2000	Fracture of foot bones except ankle	0.0264
France	2005	Fracture of foot bones except ankle	0.0264
France	2010	Fracture of foot bones except ankle	0.0264
France	2017	Fracture of foot bones except ankle	0.0264
France	1990	Fracture of hand (wrist and other distal part of hand)	0.0138
France	1995	Fracture of hand (wrist and other distal part of hand)	0.0138
France	2000	Fracture of hand (wrist and other distal part of hand)	0.0138
France	2005	Fracture of hand (wrist and other distal part of hand)	0.0138
France	2010	Fracture of hand (wrist and other distal part of hand)	0.0138
France	2017	Fracture of hand (wrist and other distal part of hand)	0.0138
France	1990	Fracture of hip	0.0577
France	1995	Fracture of hip	0.0577
France	2000	Fracture of hip	0.0577
France	2005	Fracture of hip	0.0577
France	2010	Fracture of hip	0.0577
France	2017	Fracture of hip	0.0577
France	1990	Amputation of upper limbs, bilateral	0.0876
France	1995	Amputation of upper limbs, bilateral	0.0876
France	2000	Amputation of upper limbs, bilateral	0.0876
France	2005	Amputation of upper limbs, bilateral	0.0876
France	2010	Amputation of upper limbs, bilateral	0.0876
France	2017	Amputation of upper limbs, bilateral	0.0876
France	1990	Fracture of patella, tibia or fibula, or ankle	0.0554
France	1995	Fracture of patella, tibia or fibula, or ankle	0.0554
France	2000	Fracture of patella, tibia or fibula, or ankle	0.0554
France	2005	Fracture of patella, tibia or fibula, or ankle	0.0554
France	2010	Fracture of patella, tibia or fibula, or ankle	0.0554
France	2017	Fracture of patella, tibia or fibula, or ankle	0.0554
France	1990	Fracture of pelvis	0.1823
France	1995	Fracture of pelvis	0.1823
France	2000	Fracture of pelvis	0.1823
France	2005	Fracture of pelvis	0.1823
France	2010	Fracture of pelvis	0.1823
France	2017	Fracture of pelvis	0.1823
France	1990	Fracture of radius and/or ulna	0.0435
France	1995	Fracture of radius and/or ulna	0.0435
France	2000	Fracture of radius and/or ulna	0.0435
France	2005	Fracture of radius and/or ulna	0.0435
France	2010	Fracture of radius and/or ulna	0.0435
France	2017	Fracture of radius and/or ulna	0.0435
France	1990	Fracture of skull	0.0714
France	1995	Fracture of skull	0.0714
France	2000	Fracture of skull	0.0714
France	2005	Fracture of skull	0.0714
France	2010	Fracture of skull	0.0714
France	2017	Fracture of skull	0.0714
France	1990	Fracture of sternum and/or fracture of one or more ribs	0.1027
France	1995	Fracture of sternum and/or fracture of one or more ribs	0.1027
France	2000	Fracture of sternum and/or fracture of one or more ribs	0.1027
France	2005	Fracture of sternum and/or fracture of one or more ribs	0.1027
France	2010	Fracture of sternum and/or fracture of one or more ribs	0.1027
France	2017	Fracture of sternum and/or fracture of one or more ribs	0.1027
France	1990	Fracture of vertebral column	0.1106
France	1995	Fracture of vertebral column	0.1106
France	2000	Fracture of vertebral column	0.1106
France	2005	Fracture of vertebral column	0.1106
France	2010	Fracture of vertebral column	0.1106
France	2017	Fracture of vertebral column	0.1106
France	1990	Fracture of femur, other than femoral neck	0.042
France	1995	Fracture of femur, other than femoral neck	0.042
France	2000	Fracture of femur, other than femoral neck	0.042
France	2005	Fracture of femur, other than femoral neck	0.042
France	2010	Fracture of femur, other than femoral neck	0.042
France	2017	Fracture of femur, other than femoral neck	0.042
France	1990	Minor TBI	0.1319
France	1995	Minor TBI	0.1319
France	2000	Minor TBI	0.1319
France	2005	Minor TBI	0.1319
France	2010	Minor TBI	0.1319
France	2017	Minor TBI	0.1319

France	1990	Moderate TBI	0.1637
France	1995	Moderate TBI	0.1637
France	2000	Moderate TBI	0.1637
France	2005	Moderate TBI	0.1637
France	2010	Moderate TBI	0.1637
France	2017	Moderate TBI	0.1637
France	1990	Amputation of fingers (excluding thumb)	0.0047
France	1995	Amputation of fingers (excluding thumb)	0.0047
France	2000	Amputation of fingers (excluding thumb)	0.0047
France	2005	Amputation of fingers (excluding thumb)	0.0047
France	2010	Amputation of fingers (excluding thumb)	0.0047
France	2017	Amputation of fingers (excluding thumb)	0.0047
France	1990	Spinal cord lesion at neck level	0.4118
France	1995	Spinal cord lesion at neck level	0.4118
France	2000	Spinal cord lesion at neck level	0.4118
France	2005	Spinal cord lesion at neck level	0.4118
France	2010	Spinal cord lesion at neck level	0.4118
France	2017	Spinal cord lesion at neck level	0.4118
France	1990	Spinal cord lesion below neck level	0.1777
France	1995	Spinal cord lesion below neck level	0.1777
France	2000	Spinal cord lesion below neck level	0.1777
France	2005	Spinal cord lesion below neck level	0.1777
France	2010	Spinal cord lesion below neck level	0.1777
France	2017	Spinal cord lesion below neck level	0.1777
France	1990	Drowning and nonfatal submersion	0.2471
France	1995	Drowning and nonfatal submersion	0.2471
France	2000	Drowning and nonfatal submersion	0.2471
France	2005	Drowning and nonfatal submersion	0.2471
France	2010	Drowning and nonfatal submersion	0.2471
France	2017	Drowning and nonfatal submersion	0.2471
France	1990	Asphyxiation	0.2471
France	1995	Asphyxiation	0.2471
France	2000	Asphyxiation	0.2471
France	2005	Asphyxiation	0.2471
France	2010	Asphyxiation	0.2471
France	2017	Asphyxiation	0.2471
France	1990	Crush injury	0.1325
France	1995	Crush injury	0.1325
France	2000	Crush injury	0.1325
France	2005	Crush injury	0.1325
France	2010	Crush injury	0.1325
France	2017	Crush injury	0.1325
France	1990	Nerve injury	0.1129
France	1995	Nerve injury	0.1129
France	2000	Nerve injury	0.1129
France	2005	Nerve injury	0.1129
France	2010	Nerve injury	0.1129
France	2017	Nerve injury	0.1129
France	1990	Injury to eyes	0.0165
France	1995	Injury to eyes	0.0165
France	2000	Injury to eyes	0.0165
France	2005	Injury to eyes	0.0165
France	2010	Injury to eyes	0.0165
France	2017	Injury to eyes	0.0165
France	1990	Amputation of lower limb, unilateral	0.039
France	1995	Amputation of lower limb, unilateral	0.039
France	2000	Amputation of lower limb, unilateral	0.039
France	2005	Amputation of lower limb, unilateral	0.039
France	2010	Amputation of lower limb, unilateral	0.039
France	2017	Amputation of lower limb, unilateral	0.039
France	1990	Open wound(s)	0.011
France	1995	Open wound(s)	0.011
France	2000	Open wound(s)	0.011
France	2005	Open wound(s)	0.011
France	2010	Open wound(s)	0.011
France	2017	Open wound(s)	0.011
France	1990	Poisoning requiring urgent care	0.1628
France	1995	Poisoning requiring urgent care	0.1628
France	2000	Poisoning requiring urgent care	0.1628
France	2005	Poisoning requiring urgent care	0.1628
France	2010	Poisoning requiring urgent care	0.1628
France	2017	Poisoning requiring urgent care	0.1628
France	1990	Severe chest injury	0.0474
France	1995	Severe chest injury	0.0474
France	2000	Severe chest injury	0.0474
France	2005	Severe chest injury	0.0474
France	2010	Severe chest injury	0.0474
France	2017	Severe chest injury	0.0474
France	1990	Internal hemorrhage in abdomen and pelvis	0.1143
France	1995	Internal hemorrhage in abdomen and pelvis	0.1143
France	2000	Internal hemorrhage in abdomen and pelvis	0.1143
France	2005	Internal hemorrhage in abdomen and pelvis	0.1143
France	2010	Internal hemorrhage in abdomen and pelvis	0.1143
France	2017	Internal hemorrhage in abdomen and pelvis	0.1143
France	1990	Contusion in any part of the body	0.0075
France	1995	Contusion in any part of the body	0.0075
France	2000	Contusion in any part of the body	0.0075
France	2005	Contusion in any part of the body	0.0075
France	2010	Contusion in any part of the body	0.0075
France	2017	Contusion in any part of the body	0.0075
France	1990	Effect of different environmental factors	0.049
France	1995	Effect of different environmental factors	0.049
France	2000	Effect of different environmental factors	0.049
France	2005	Effect of different environmental factors	0.049
France	2010	Effect of different environmental factors	0.049
France	2017	Effect of different environmental factors	0.049
France	1990	Complications following therapeutic procedures	0.0506
France	1995	Complications following therapeutic procedures	0.0506
France	2000	Complications following therapeutic procedures	0.0506
France	2005	Complications following therapeutic procedures	0.0506
France	2010	Complications following therapeutic procedures	0.0506
France	2017	Complications following therapeutic procedures	0.0506
France	1990	Multiple fractures, dislocations, crashes, wounds, sprains, and strains	0.0577
France	1995	Multiple fractures, dislocations, crashes, wounds, sprains, and strains	0.0577
France	2000	Multiple fractures, dislocations, crashes, wounds, sprains, and strains	0.0577
France	2005	Multiple fractures, dislocations, crashes, wounds, sprains, and strains	0.0577
France	2010	Multiple fractures, dislocations, crashes, wounds, sprains, and strains	0.0577
France	2017	Multiple fractures, dislocations, crashes, wounds, sprains, and strains	0.0577
France	1990	Amputation of upper limb, unilateral	0.039
France	1995	Amputation of upper limb, unilateral	0.039
France	2000	Amputation of upper limb, unilateral	0.039
France	2005	Amputation of upper limb, unilateral	0.039
France	2010	Amputation of upper limb, unilateral	0.039
France	2017	Amputation of upper limb, unilateral	0.039
France	1990	Amputation of thumb	0.0114
France	1995	Amputation of thumb	0.0114
France	2000	Amputation of thumb	0.0114
France	2005	Amputation of thumb	0.0114
France	2010	Amputation of thumb	0.0114
France	2017	Amputation of thumb	0.0114
France	1990	Amputation of toe/toes	0.0059
France	1995	Amputation of toe/toes	0.0059
France	2000	Amputation of toe/toes	0.0059
France	2005	Amputation of toe/toes	0.0059
France	2010	Amputation of toe/toes	0.0059
France	2017	Amputation of toe/toes	0.0059
France	1990	Burns, <20% total burned surface area without lower airway burns	0.0163
France	1995	Burns, <20% total burned surface area without lower airway burns	0.0163
France	2000	Burns, <20% total burned surface area without lower airway burns	0.0163
France	2005	Burns, <20% total burned surface area without lower airway burns	0.0163
France	2010	Burns, <20% total burned surface area without lower airway burns	0.0163
France	2017	Burns, <20% total burned surface area without lower airway burns	0.0163
France	1990	Burns, >=20% total burned surface area or >= 10% burned surface area if head/neck or hands/wrist involved w/o lower airway burns	0.1346
France	1995	Burns, >=20% total burned surface area or >= 10% burned surface area if head/neck or hands/wrist involved w/o lower airway burns	0.1346
France	2000	Burns, >=20% total burned surface area or >= 10% burned surface area if head/neck or hands/wrist involved w/o lower airway burns	0.1346
France	2005	Burns, >=20% total burned surface area or >= 10% burned surface area if head/neck or hands/wrist involved w/o lower airway burns	0.1346
France	2010	Burns, >=20% total burned surface area or >= 10% burned surface area if head/neck or hands/wrist involved w/o lower airway burns	0.1346
France	2017	Burns, >=20% total burned surface area or >= 10% burned surface area if head/neck or hands/wrist involved w/o lower airway burns	0.1346
Germany	1990	Amputation of lower limbs, bilateral	0.1232
Germany	1995	Amputation of lower limbs, bilateral	0.1232
Germany	2000	Amputation of lower limbs, bilateral	0.1232
Germany	2005	Amputation of lower limbs, bilateral	0.1232
Germany	2010	Amputation of lower limbs, bilateral	0.1232
Germany	2017	Amputation of lower limbs, bilateral	0.1232
Germany	1990	Lower airway burns	0.2252
Germany	1995	Lower airway burns	0.2252
Germany	2000	Lower airway burns	0.2252
Germany	2005	Lower airway burns	0.2252
Germany	2010	Lower airway burns	0.2252
Germany	2017	Lower airway burns	0.2252
Germany	1990	Dislocation of hip	0.0159
Germany	1995	Dislocation of hip	0.0159
Germany	2000	Dislocation of hip	0.0159
Germany	2005	Dislocation of hip	0.0159
Germany	2010	Dislocation of hip	0.0159
Germany	2017	Dislocation of hip	0.0159
Germany	1990	Dislocation of knee	0.1134
Germany	1995	Dislocation of knee	0.1134
Germany	2000	Dislocation of knee	0.1134
Germany	2005	Dislocation of knee	0.1134
Germany	2010	Dislocation of knee	0.1134
Germany	2017	Dislocation of knee	0.1134
Germany	1990	Dislocation of shoulder	0.062
Germany	1995	Dislocation of shoulder	0.062
Germany	2000	Dislocation of shoulder	0.062
Germany	2005	Dislocation of shoulder	0.062
Germany	2010	Dislocation of shoulder	0.062
Germany	2017	Dislocation of shoulder	0.062
Germany	1990	Muscle and tendon injuries, including sprains and strains lesser dislocations	0.0075
Germany	1995	Muscle and tendon injuries, including sprains and strains lesser dislocations	0.0075
Germany	2000	Muscle and tendon injuries, including sprains and strains lesser dislocations	0.0075
Germany	2005	Muscle and tendon injuries, including sprains and strains lesser dislocations	0.0075
Germany	2010	Muscle and tendon injuries, including sprains and strains lesser dislocations	0.0075
Germany	2017	Muscle and tendon injuries, including sprains and strains lesser dislocations	0.0075
Germany	1990	Fracture of clavicle, scapula, or humerus	0.0349
Germany	1995	Fracture of clavicle, scapula, or humerus	0.0349
Germany	2000	Fracture of clavicle, scapula, or humerus	0.0349
Germany	2005	Fracture of clavicle, scapula, or humerus	0.0349
Germany	2010	Fracture of clavicle, scapula, or humerus	0.0349
Germany	2017	Fracture of clavicle, scapula, or humerus	0.0349
Germany	1990	Fracture of face bones	0.0669
Germany	1995	Fracture of face bones	0.0669
Germany	2000	Fracture of face bones	0.0669
Germany	2005	Fracture of face bones	0.0669
Germany	2010	Fracture of face bones	0.0669
Germany	2017	Fracture of face bones	0.0669
Germany	1990	Fracture of foot bones except ankle	0.0264
Germany	1995	Fracture of foot bones except ankle	0.0264
Germany	2000	Fracture of foot bones except ankle	0.0264
Germany	2005	Fracture of foot bones except ankle	0.0264
Germany	2010	Fracture of foot bones except ankle	0.0264
Germany	2017	Fracture of foot bones except ankle	0.0264
Germany	1990	Fracture of hand (wrist and other distal part of hand)	0.0138
Germany	1995	Fracture of hand (wrist and other distal part of hand)	0.0138
Germany	2000	Fracture of hand (wrist and other distal part of hand)	0.0138
Germany	2005	Fracture of hand (wrist and other distal part of hand)	0.0138
Germany	2010	Fracture of hand (wrist and other distal part of hand)	0.0138
Germany	2017	Fracture of hand (wrist and other distal part of hand)	0.0138
Germany	1990	Fracture of hip	0.0577
Germany	1995	Fracture of hip	0.0577
Germany	2000	Fracture of hip	0.0577
Germany	2005	Fracture of hip	0.0577
Germany	2010	Fracture of hip	0.0577
Germany	2017	Fracture of hip	0.0577
Germany	1990	Amputation of upper limbs, bilateral	0.0876
Germany	1995	Amputation of upper limbs, bilateral	0.0876
Germany	2000	Amputation of upper limbs, bilateral	0.0876
Germany	2005	Amputation of upper limbs, bilateral	0.0876
Germany	2010	Amputation of upper limbs, bilateral	0.0876
Germany	2017	Amputation of upper limbs, bilateral	0.0876
Germany	1990	Fracture of patella, tibia or fibula, or ankle	0.0554
Germany	1995	Fracture of patella, tibia or fibula, or ankle	0.0554
Germany	2000	Fracture of patella, tibia or fibula, or ankle	0.0554
Germany	2005	Fracture of patella, tibia or fibula, or ankle	0.0554
Germany	2010	Fracture of patella, tibia or fibula, or ankle	0.0554
Germany	2017	Fracture of patella, tibia or fibula, or ankle	0.0554
Germany	1990	Fracture of pelvis	0.1823
Germany	1995	Fracture of pelvis	0.1823
Germany	2000	Fracture of pelvis	0.1823
Germany	2005	Fracture of pelvis	0.1823
Germany	2010	Fracture of pelvis	0.1823
Germany	2017	Fracture of pelvis	0.1823
Germany	1990	Fracture of radius and/or ulna	0.0435
Germany	1995	Fracture of radius and/or ulna	0.0435
Germany	2000	Fracture of radius and/or ulna	0.0435
Germany	2005	Fracture of radius and/or ulna	0.0435
Germany	2010	Fracture of radius and/or ulna	0.0435
Germany	2017	Fracture of radius and/or ulna	0.0435
Germany	1990	Fracture of skull	0.0714
Germany	1995	Fracture of skull	0.0714
Germany	2000	Fracture of skull	0.0714
Germany	2005	Fracture of skull	0.0714
Germany	2010	Fracture of skull	0.0714
Germany	2017	Fracture of skull	0.0714
Germany	1990	Fracture of sternum and/or fracture of one or more ribs	0.1027
Germany	1995	Fracture of sternum and/or fracture of one or more ribs	0.1027
Germany	2000	Fracture of sternum and/or fracture of one or more ribs	0.1027
Germany	2005	Fracture of sternum and/or fracture of one or more ribs	0.1027
Germany	2010	Fracture of sternum and/or fracture of one or more ribs	0.1027
Germany	2017	Fracture of sternum and/or fracture of one or more ribs	0.1027
Germany	1990	Fracture of vertebral column	0.1106
Germany	1995	Fracture of vertebral column	0.1106
Germany	2000	Fracture of vertebral column	0.1106
Germany	2005	Fracture of vertebral column	0.1106
Germany	2010	Fracture of vertebral column	0.1106
Germany	2017	Fracture of vertebral column	0.1106
Germany	1990	Fracture of femur, other than femoral neck	0.042
Germany	1995	Fracture of femur, other than femoral neck	0.042
Germany	2000	Fracture of femur, other than femoral neck	0.042
Germany	2005	Fracture of femur, other than femoral neck	0.042
Germany	2010	Fracture of femur, other than femoral neck	0.042
Germany	2017	Fracture of femur, other than femoral neck	0.042
Germany	1990	Minor TBI	0.1319
Germany	1995	Minor TBI	0.1319
Germany	2000	Minor TBI	0.1319
Germany	2005	Minor TBI	0.1319
Germany	2010	Minor TBI	0.1319
Germany	2017	Minor TBI	0.1319
Germany	1990	Moderate TBI	0.1637
Germany	1995	Moderate TBI	0.1637
Germany	2000	Moderate TBI	0.1637
Germany	2005	Moderate TBI	0.1637
Germany	2010	Moderate TBI	0.1637
Germany	2017	Moderate TBI	0.1637
Germany	1990	Amputation of fingers (excluding thumb)	0.0047
Germany	1995	Amputation of fingers (excluding thumb)	0.0047
Germany	2000	Amputation of fingers (excluding thumb)	0.0047
Germany	2005	Amputation of fingers (excluding thumb)	0.0047
Germany	2010	Amputation of fingers (excluding thumb)	0.0047
Germany	2017	Amputation of fingers (excluding thumb)	0.0047
Germany	1990	Spinal cord lesion at neck level	0.4118
Germany	1995	Spinal cord lesion at neck level	0.4118
Germany	2000	Spinal cord lesion at neck level	0.4118
Germany	2005	Spinal cord lesion at neck level	0.4118
Germany	2010	Spinal cord lesion at neck level	0.4118
Germany	2017	Spinal cord lesion at neck level	0.4118
Germany	1990	Spinal cord lesion below neck level	0.1777
Germany	1995	Spinal cord lesion below neck level	0.1777
Germany	2000	Spinal cord lesion below neck level	0.1777
Germany	2005	Spinal cord lesion below neck level	0.1777
Germany	2010	Spinal cord lesion below neck level	0.1777
Germany	2017	Spinal cord lesion below neck level	0.1777
Germany	1990	Drowning and nonfatal submersion	0.2471
Germany	1995	Drowning and nonfatal submersion	0.2471
Germany	2000	Drowning and nonfatal submersion	0.2471
Germany	2005	Drowning and nonfatal submersion	0.2471
Germany	2010	Drowning and nonfatal submersion	0.2471
Germany	2017	Drowning and nonfatal submersion	0.2471
Germany	1990	Asphyxiation	0.2471
Germany	1995	Asphyxiation	0.2471
Germany	2000	Asphyxiation	0.2471
Germany	2005	Asphyxiation	0.2471
Germany	2010	Asphyxiation	0.2471
Germany	2017	Asphyxiation	0.2471
Germany	1990	Crush injury	0.1325
Germany	1995	Crush injury	0.1325
Germany	2000	Crush injury	0.1325
Germany	2005	Crush injury	0.1325
Germany	2010	Crush injury	0.1325
Germany	2017	Crush injury	0.1325
Germany	1990	Nerve injury	0.1129
Germany	1995	Nerve injury	0.1129
Germany	2000	Nerve injury	0.1129
Germany	2005	Nerve injury	0.1129
Germany	2010	Nerve injury	0.1129
Germany	2017	Nerve injury	0.1129
Germany	1990	Injury to eyes	0.0165
Germany	1995	Injury to eyes	0.0165
Germany	2000	Injury to eyes	0.0165
Germany	2005	Injury to eyes	0.0165
Germany	2010	Injury to eyes	0.0165
Germany	2017	Injury to eyes	0.0165
Germany	1990	Amputation of lower limb, unilateral	0.039
Germany	1995	Amputation of lower limb, unilateral	0.039
Germany	2000	Amputation of lower limb, unilateral	0.039
Germany	2005	Amputation of lower limb, unilateral	0.039
Germany	2010	Amputation of lower limb, unilateral	0.039
Germany	2017	Amputation of lower limb, unilateral	0.039
Germany	1990	Open wound(s)	0.011
Germany	1995	Open wound(s)	0.011
Germany	2000	Open wound(s)	0.011
Germany	2005	Open wound(s)	0.011
Germany	2010	Open wound(s)	0.011
Germany	2017	Open wound(s)	0.011
Germany	1990	Poisoning requiring urgent care	0.1628
Germany	1995	Poisoning requiring urgent care	0.1628
Germany	2000	Poisoning requiring urgent care	0.1628
Germany	2005	Poisoning requiring urgent care	0.1628
Germany	2010	Poisoning requiring urgent care	0.1628
Germany	2017	Poisoning requiring urgent care	0.1628
Germany	1990	Severe chest injury	0.0474
Germany	1995	Severe chest injury	0.0474
Germany	2000	Severe chest injury	0.0474
Germany	2005	Severe chest injury	0.0474
Germany	2010	Severe chest injury	0.0474
Germany	2017	Severe chest injury	0.0474
Germany	1990	Internal hemorrhage in abdomen and pelvis	0.1143
Germany	1995	Internal hemorrhage in abdomen and pelvis	0.1143
Germany	2000	Internal hemorrhage in abdomen and pelvis	0.1143
Germany	2005	Internal hemorrhage in abdomen and pelvis	0.1143
Germany	2010	Internal hemorrhage in abdomen and pelvis	0.1143
Germany	2017	Internal hemorrhage in abdomen and pelvis	0.1143
Germany	1990	Contusion in any part of the body	0.0075
Germany	1995	Contusion in any part of the body	0.0075
Germany	2000	Contusion in any part of the body	0.0075
Germany	2005	Contusion in any part of the body	0.0075
Germany	2010	Contusion in any part of the body	0.0075
Germany	2017	Contusion in any part of the body	0.0075
Germany	1990	Effect of different environmental factors	0.049
Germany	1995	Effect of different environmental factors	0.049
Germany	2000	Effect of different environmental factors	0.049
Germany	2005	Effect of different environmental factors	0.049
Germany	2010	Effect of different environmental factors	0.049
Germany	2017	Effect of different environmental factors	0.049
Germany	1990	Complications following therapeutic procedures	0.0506
Germany	1995	Complications following therapeutic procedures	0.0506
Germany	2000	Complications following therapeutic procedures	0.0506
Germany	2005	Complications following therapeutic procedures	0.0506
Germany	2010	Complications following therapeutic procedures	0.0506
Germany	2017	Complications following therapeutic procedures	0.0506
Germany	1990	Multiple fractures, dislocations, crashes, wounds, sprains, and strains	0.0577
Germany	1995	Multiple fractures, dislocations, crashes, wounds, sprains, and strains	0.0577
Germany	2000	Multiple fractures, dislocations, crashes, wounds, sprains, and strains	0.0577
Germany	2005	Multiple fractures, dislocations, crashes, wounds, sprains, and strains	0.0577
Germany	2010	Multiple fractures, dislocations, crashes, wounds, sprains, and strains	0.0577
Germany	2017	Multiple fractures, dislocations, crashes, wounds, sprains, and strains	0.0577
Germany	1990	Amputation of upper limb, unilateral	0.039
Germany	1995	Amputation of upper limb, unilateral	0.039
Germany	2000	Amputation of upper limb, unilateral	0.039
Germany	2005	Amputation of upper limb, unilateral	0.039
Germany	2010	Amputation of upper limb, unilateral	0.039
Germany	2017	Amputation of upper limb, unilateral	0.039
Germany	1990	Amputation of thumb	0.0114
Germany	1995	Amputation of thumb	0.0114
Germany	2000	Amputation of thumb	0.0114
Germany	2005	Amputation of thumb	0.0114
Germany	2010	Amputation of thumb	0.0114
Germany	2017	Amputation of thumb	0.0114
Germany	1990	Amputation of toe/toes	0.0059
Germany	1995	Amputation of toe/toes	0.0059
Germany	2000	Amputation of toe/toes	0.0059
Germany	2005	Amputation of toe/toes	0.0059
Germany	2010	Amputation of toe/toes	0.0059
Germany	2017	Amputation of toe/toes	0.0059
Germany	1990	Burns, <20% total burned surface area without lower airway burns	0.0163
Germany	1995	Burns, <20% total burned surface area without lower airway burns	0.0163
Germany	2000	Burns, <20% total burned surface area without lower airway burns	0.0163
Germany	2005	Burns, <20% total burned surface area without lower airway burns	0.0163
Germany	2010	Burns, <20% total burned surface area without lower airway burns	0.0163
Germany	2017	Burns, <20% total burned surface area without lower airway burns	0.0163
Germany	1990	Burns, >=20% total burned surface area or >= 10% burned surface area if head/neck or hands/wrist involved w/o lower airway burns	0.1346
Germany	1995	Burns, >=20% total burned surface area or >= 10% burned surface area if head/neck or hands/wrist involved w/o lower airway burns	0.1346
Germany	2000	Burns, >=20% total burned surface area or >= 10% burned surface area if head/neck or hands/wrist involved w/o lower airway burns	0.1346
Germany	2005	Burns, >=20% total burned surface area or >= 10% burned surface area if head/neck or hands/wrist involved w/o lower airway burns	0.1346
Germany	2010	Burns, >=20% total burned surface area or >= 10% burned surface area if head/neck or hands/wrist involved w/o lower airway burns	0.1346
Germany	2017	Burns, >=20% total burned surface area or >= 10% burned surface area if head/neck or hands/wrist involved w/o lower airway burns	0.1346
Greece	1990	Amputation of lower limbs, bilateral	0.1232
Greece	1995	Amputation of lower limbs, bilateral	0.1232
Greece	2000	Amputation of lower limbs, bilateral	0.1232
Greece	2005	Amputation of lower limbs, bilateral	0.1232
Greece	2010	Amputation of lower limbs, bilateral	0.1232
Greece	2017	Amputation of lower limbs, bilateral	0.1232
Greece	1990	Lower airway burns	0.2252
Greece	1995	Lower airway burns	0.2252
Greece	2000	Lower airway burns	0.2252
Greece	2005	Lower airway burns	0.2252
Greece	2010	Lower airway burns	0.2252
Greece	2017	Lower airway burns	0.2252
Greece	1990	Dislocation of hip	0.0159
Greece	1995	Dislocation of hip	0.0159
Greece	2000	Dislocation of hip	0.0159
Greece	2005	Dislocation of hip	0.0159
Greece	2010	Dislocation of hip	0.0159
Greece	2017	Dislocation of hip	0.0159
Greece	1990	Dislocation of knee	0.1134
Greece	1995	Dislocation of knee	0.1134
Greece	2000	Dislocation of knee	0.1134
Greece	2005	Dislocation of knee	0.1134
Greece	2010	Dislocation of knee	0.1134
Greece	2017	Dislocation of knee	0.1134
Greece	1990	Dislocation of shoulder	0.062
Greece	1995	Dislocation of shoulder	0.062
Greece	2000	Dislocation of shoulder	0.062
Greece	2005	Dislocation of shoulder	0.062
Greece	2010	Dislocation of shoulder	0.062
Greece	2017	Dislocation of shoulder	0.062
Greece	1990	Muscle and tendon injuries, including sprains and strains lesser dislocations	0.0075
Greece	1995	Muscle and tendon injuries, including sprains and strains lesser dislocations	0.0075
Greece	2000	Muscle and tendon injuries, including sprains and strains lesser dislocations	0.0075
Greece	2005	Muscle and tendon injuries, including sprains and strains lesser dislocations	0.0075
Greece	2010	Muscle and tendon injuries, including sprains and strains lesser dislocations	0.0075

Greece	2017	Muscle and tendon injuries, including sprains and strains lesser dislocations	0.0075
Greece	1990	Fracture of clavicle, scapula, or humerus	0.0349
Greece	1995	Fracture of clavicle, scapula, or humerus	0.0349
Greece	2000	Fracture of clavicle, scapula, or humerus	0.0349
Greece	2005	Fracture of clavicle, scapula, or humerus	0.0349
Greece	2010	Fracture of clavicle, scapula, or humerus	0.0349
Greece	2017	Fracture of clavicle, scapula, or humerus	0.0349
Greece	1990	Fracture of face bones	0.0669
Greece	1995	Fracture of face bones	0.0669
Greece	2000	Fracture of face bones	0.0669
Greece	2005	Fracture of face bones	0.0669
Greece	2010	Fracture of face bones	0.0669
Greece	2017	Fracture of face bones	0.0669
Greece	1990	Fracture of foot bones except ankle	0.0264
Greece	1995	Fracture of foot bones except ankle	0.0264
Greece	2000	Fracture of foot bones except ankle	0.0264
Greece	2005	Fracture of foot bones except ankle	0.0264
Greece	2010	Fracture of foot bones except ankle	0.0264
Greece	2017	Fracture of foot bones except ankle	0.0264
Greece	1990	Fracture of hand (wrist and other distal part of hand)	0.0138
Greece	1995	Fracture of hand (wrist and other distal part of hand)	0.0138
Greece	2000	Fracture of hand (wrist and other distal part of hand)	0.0138
Greece	2005	Fracture of hand (wrist and other distal part of hand)	0.0138
Greece	2010	Fracture of hand (wrist and other distal part of hand)	0.0138
Greece	2017	Fracture of hand (wrist and other distal part of hand)	0.0138
Greece	1990	Fracture of hip	0.0577
Greece	1995	Fracture of hip	0.0577
Greece	2000	Fracture of hip	0.0577
Greece	2005	Fracture of hip	0.0577
Greece	2010	Fracture of hip	0.0577
Greece	2017	Fracture of hip	0.0577
Greece	1990	Amputation of upper limbs, bilateral	0.0876
Greece	1995	Amputation of upper limbs, bilateral	0.0876
Greece	2000	Amputation of upper limbs, bilateral	0.0876
Greece	2005	Amputation of upper limbs, bilateral	0.0876
Greece	2010	Amputation of upper limbs, bilateral	0.0876
Greece	2017	Amputation of upper limbs, bilateral	0.0876
Greece	1990	Fracture of patella, tibia or fibula, or ankle	0.0554
Greece	1995	Fracture of patella, tibia or fibula, or ankle	0.0554
Greece	2000	Fracture of patella, tibia or fibula, or ankle	0.0554
Greece	2005	Fracture of patella, tibia or fibula, or ankle	0.0554
Greece	2010	Fracture of patella, tibia or fibula, or ankle	0.0554
Greece	2017	Fracture of patella, tibia or fibula, or ankle	0.0554
Greece	1990	Fracture of pelvis	0.1823
Greece	1995	Fracture of pelvis	0.1823
Greece	2000	Fracture of pelvis	0.1823
Greece	2005	Fracture of pelvis	0.1823
Greece	2010	Fracture of pelvis	0.1823
Greece	2017	Fracture of pelvis	0.1823
Greece	1990	Fracture of radius and/or ulna	0.0435
Greece	1995	Fracture of radius and/or ulna	0.0435
Greece	2000	Fracture of radius and/or ulna	0.0435
Greece	2005	Fracture of radius and/or ulna	0.0435
Greece	2010	Fracture of radius and/or ulna	0.0435
Greece	2017	Fracture of radius and/or ulna	0.0435
Greece	1990	Fracture of skull	0.0714
Greece	1995	Fracture of skull	0.0714
Greece	2000	Fracture of skull	0.0714
Greece	2005	Fracture of skull	0.0714
Greece	2010	Fracture of skull	0.0714
Greece	2017	Fracture of skull	0.0714
Greece	1990	Fracture of sternum and/or fracture of one or more ribs	0.1027
Greece	1995	Fracture of sternum and/or fracture of one or more ribs	0.1027
Greece	2000	Fracture of sternum and/or fracture of one or more ribs	0.1027
Greece	2005	Fracture of sternum and/or fracture of one or more ribs	0.1027
Greece	2010	Fracture of sternum and/or fracture of one or more ribs	0.1027
Greece	2017	Fracture of sternum and/or fracture of one or more ribs	0.1027
Greece	1990	Fracture of vertebral column	0.1106
Greece	1995	Fracture of vertebral column	0.1106
Greece	2000	Fracture of vertebral column	0.1106
Greece	2005	Fracture of vertebral column	0.1106
Greece	2010	Fracture of vertebral column	0.1106
Greece	2017	Fracture of vertebral column	0.1106
Greece	1990	Fracture of femur, other than femoral neck	0.042
Greece	1995	Fracture of femur, other than femoral neck	0.042
Greece	2000	Fracture of femur, other than femoral neck	0.042
Greece	2005	Fracture of femur, other than femoral neck	0.042
Greece	2010	Fracture of femur, other than femoral neck	0.042
Greece	2017	Fracture of femur, other than femoral neck	0.042
Greece	1990	Minor TBI	0.1319
Greece	1995	Minor TBI	0.1319
Greece	2000	Minor TBI	0.1319
Greece	2005	Minor TBI	0.1319
Greece	2010	Minor TBI	0.1319
Greece	2017	Minor TBI	0.1319
Greece	1990	Moderate TBI	0.1637
Greece	1995	Moderate TBI	0.1637
Greece	2000	Moderate TBI	0.1637
Greece	2005	Moderate TBI	0.1637
Greece	2010	Moderate TBI	0.1637
Greece	2017	Moderate TBI	0.1637
Greece	1990	Amputation of fingers (excluding thumb)	0.0047
Greece	1995	Amputation of fingers (excluding thumb)	0.0047
Greece	2000	Amputation of fingers (excluding thumb)	0.0047
Greece	2005	Amputation of fingers (excluding thumb)	0.0047
Greece	2010	Amputation of fingers (excluding thumb)	0.0047
Greece	2017	Amputation of fingers (excluding thumb)	0.0047
Greece	1990	Spinal cord lesion at neck level	0.4118
Greece	1995	Spinal cord lesion at neck level	0.4118
Greece	2000	Spinal cord lesion at neck level	0.4118
Greece	2005	Spinal cord lesion at neck level	0.4118
Greece	2010	Spinal cord lesion at neck level	0.4118
Greece	2017	Spinal cord lesion at neck level	0.4118
Greece	1990	Spinal cord lesion below neck level	0.1777
Greece	1995	Spinal cord lesion below neck level	0.1777
Greece	2000	Spinal cord lesion below neck level	0.1777
Greece	2005	Spinal cord lesion below neck level	0.1777
Greece	2010	Spinal cord lesion below neck level	0.1777
Greece	2017	Spinal cord lesion below neck level	0.1777
Greece	1990	Drowning and nonfatal submersion	0.2471
Greece	1995	Drowning and nonfatal submersion	0.2471
Greece	2000	Drowning and nonfatal submersion	0.2471
Greece	2005	Drowning and nonfatal submersion	0.2471
Greece	2010	Drowning and nonfatal submersion	0.2471
Greece	2017	Drowning and nonfatal submersion	0.2471
Greece	1990	Asphyxiation	0.2471
Greece	1995	Asphyxiation	0.2471
Greece	2000	Asphyxiation	0.2471
Greece	2005	Asphyxiation	0.2471
Greece	2010	Asphyxiation	0.2471
Greece	2017	Asphyxiation	0.2471
Greece	1990	Crush injury	0.1325
Greece	1995	Crush injury	0.1325
Greece	2000	Crush injury	0.1325
Greece	2005	Crush injury	0.1325
Greece	2010	Crush injury	0.1325
Greece	2017	Crush injury	0.1325
Greece	1990	Nerve injury	0.1129
Greece	1995	Nerve injury	0.1129
Greece	2000	Nerve injury	0.1129
Greece	2005	Nerve injury	0.1129
Greece	2010	Nerve injury	0.1129
Greece	2017	Nerve injury	0.1129
Greece	1990	Injury to eyes	0.0165
Greece	1995	Injury to eyes	0.0165
Greece	2000	Injury to eyes	0.0165
Greece	2005	Injury to eyes	0.0165
Greece	2010	Injury to eyes	0.0165
Greece	2017	Injury to eyes	0.0165
Greece	1990	Amputation of lower limb, unilateral	0.039
Greece	1995	Amputation of lower limb, unilateral	0.039
Greece	2000	Amputation of lower limb, unilateral	0.039
Greece	2005	Amputation of lower limb, unilateral	0.039
Greece	2010	Amputation of lower limb, unilateral	0.039
Greece	2017	Amputation of lower limb, unilateral	0.039
Greece	1990	Open wound(s)	0.011
Greece	1995	Open wound(s)	0.011
Greece	2000	Open wound(s)	0.011
Greece	2005	Open wound(s)	0.011
Greece	2010	Open wound(s)	0.011
Greece	2017	Open wound(s)	0.011
Greece	1990	Poisoning requiring urgent care	0.1628
Greece	1995	Poisoning requiring urgent care	0.1628
Greece	2000	Poisoning requiring urgent care	0.1628
Greece	2005	Poisoning requiring urgent care	0.1628
Greece	2010	Poisoning requiring urgent care	0.1628
Greece	2017	Poisoning requiring urgent care	0.1628
Greece	1990	Severe chest injury	0.0474
Greece	1995	Severe chest injury	0.0474
Greece	2000	Severe chest injury	0.0474
Greece	2005	Severe chest injury	0.0474
Greece	2010	Severe chest injury	0.0474
Greece	2017	Severe chest injury	0.0474
Greece	1990	Internal hemorrhage in abdomen and pelvis	0.1143
Greece	1995	Internal hemorrhage in abdomen and pelvis	0.1143
Greece	2000	Internal hemorrhage in abdomen and pelvis	0.1143
Greece	2005	Internal hemorrhage in abdomen and pelvis	0.1143
Greece	2010	Internal hemorrhage in abdomen and pelvis	0.1143
Greece	2017	Internal hemorrhage in abdomen and pelvis	0.1143
Greece	1990	Contusion in any part of the body	0.0075
Greece	1995	Contusion in any part of the body	0.0075
Greece	2000	Contusion in any part of the body	0.0075
Greece	2005	Contusion in any part of the body	0.0075
Greece	2010	Contusion in any part of the body	0.0075
Greece	2017	Contusion in any part of the body	0.0075
Greece	1990	Effect of different environmental factors	0.049
Greece	1995	Effect of different environmental factors	0.049
Greece	2000	Effect of different environmental factors	0.049
Greece	2005	Effect of different environmental factors	0.049
Greece	2010	Effect of different environmental factors	0.049
Greece	2017	Effect of different environmental factors	0.049
Greece	1990	Complications following therapeutic procedures	0.0506
Greece	1995	Complications following therapeutic procedures	0.0506
Greece	2000	Complications following therapeutic procedures	0.0506
Greece	2005	Complications following therapeutic procedures	0.0506
Greece	2010	Complications following therapeutic procedures	0.0506
Greece	2017	Complications following therapeutic procedures	0.0506
Greece	1990	Multiple fractures, dislocations, crashes, wounds, sprains, and strains	0.0577
Greece	1995	Multiple fractures, dislocations, crashes, wounds, sprains, and strains	0.0577
Greece	2000	Multiple fractures, dislocations, crashes, wounds, sprains, and strains	0.0577
Greece	2005	Multiple fractures, dislocations, crashes, wounds, sprains, and strains	0.0577
Greece	2010	Multiple fractures, dislocations, crashes, wounds, sprains, and strains	0.0577
Greece	2017	Multiple fractures, dislocations, crashes, wounds, sprains, and strains	0.0577
Greece	1990	Amputation of upper limb, unilateral	0.039
Greece	1995	Amputation of upper limb, unilateral	0.039
Greece	2000	Amputation of upper limb, unilateral	0.039
Greece	2005	Amputation of upper limb, unilateral	0.039
Greece	2010	Amputation of upper limb, unilateral	0.039
Greece	2017	Amputation of upper limb, unilateral	0.039
Greece	1990	Amputation of thumb	0.0114
Greece	1995	Amputation of thumb	0.0114
Greece	2000	Amputation of thumb	0.0114
Greece	2005	Amputation of thumb	0.0114
Greece	2010	Amputation of thumb	0.0114
Greece	2017	Amputation of thumb	0.0114
Greece	1990	Amputation of toe/toes	0.0059
Greece	1995	Amputation of toe/toes	0.0059
Greece	2000	Amputation of toe/toes	0.0059
Greece	2005	Amputation of toe/toes	0.0059
Greece	2010	Amputation of toe/toes	0.0059
Greece	2017	Amputation of toe/toes	0.0059
Greece	1990	Burns, <20% total burned surface area without lower airway burns	0.0163
Greece	1995	Burns, <20% total burned surface area without lower airway burns	0.0163
Greece	2000	Burns, <20% total burned surface area without lower airway burns	0.0163
Greece	2005	Burns, <20% total burned surface area without lower airway burns	0.0163
Greece	2010	Burns, <20% total burned surface area without lower airway burns	0.0163
Greece	2017	Burns, <20% total burned surface area without lower airway burns	0.0163
Greece	1990	Burns, >=20% total burned surface area or >= 10% burned surface area if head/neck or hands/wrist involved w/o lower airway burns	0.1346
Greece	1995	Burns, >=20% total burned surface area or >= 10% burned surface area if head/neck or hands/wrist involved w/o lower airway burns	0.1346
Greece	2000	Burns, >=20% total burned surface area or >= 10% burned surface area if head/neck or hands/wrist involved w/o lower airway burns	0.1346
Greece	2005	Burns, >=20% total burned surface area or >= 10% burned surface area if head/neck or hands/wrist involved w/o lower airway burns	0.1346
Greece	2010	Burns, >=20% total burned surface area or >= 10% burned surface area if head/neck or hands/wrist involved w/o lower airway burns	0.1346
Greece	2017	Burns, >=20% total burned surface area or >= 10% burned surface area if head/neck or hands/wrist involved w/o lower airway burns	0.1346
Iceland	1990	Amputation of lower limbs, bilateral	0.1232
Iceland	1995	Amputation of lower limbs, bilateral	0.1232
Iceland	2000	Amputation of lower limbs, bilateral	0.1232
Iceland	2005	Amputation of lower limbs, bilateral	0.1232
Iceland	2010	Amputation of lower limbs, bilateral	0.1232
Iceland	2017	Amputation of lower limbs, bilateral	0.1232
Iceland	1990	Lower airway burns	0.2252
Iceland	1995	Lower airway burns	0.2252
Iceland	2000	Lower airway burns	0.2252
Iceland	2005	Lower airway burns	0.2252
Iceland	2010	Lower airway burns	0.2252
Iceland	2017	Lower airway burns	0.2252
Iceland	1990	Dislocation of hip	0.0159
Iceland	1995	Dislocation of hip	0.0159
Iceland	2000	Dislocation of hip	0.0159
Iceland	2005	Dislocation of hip	0.0159
Iceland	2010	Dislocation of hip	0.0159
Iceland	2017	Dislocation of hip	0.0159
Iceland	1990	Dislocation of knee	0.1134
Iceland	1995	Dislocation of knee	0.1134
Iceland	2000	Dislocation of knee	0.1134
Iceland	2005	Dislocation of knee	0.1134
Iceland	2010	Dislocation of knee	0.1134
Iceland	2017	Dislocation of knee	0.1134
Iceland	1990	Dislocation of shoulder	0.062
Iceland	1995	Dislocation of shoulder	0.062
Iceland	2000	Dislocation of shoulder	0.062
Iceland	2005	Dislocation of shoulder	0.062
Iceland	2010	Dislocation of shoulder	0.062
Iceland	2017	Dislocation of shoulder	0.062
Iceland	1990	Muscle and tendon injuries, including sprains and strains lesser dislocations	0.0075
Iceland	1995	Muscle and tendon injuries, including sprains and strains lesser dislocations	0.0075
Iceland	2000	Muscle and tendon injuries, including sprains and strains lesser dislocations	0.0075
Iceland	2005	Muscle and tendon injuries, including sprains and strains lesser dislocations	0.0075
Iceland	2010	Muscle and tendon injuries, including sprains and strains lesser dislocations	0.0075
Iceland	2017	Muscle and tendon injuries, including sprains and strains lesser dislocations	0.0075
Iceland	1990	Fracture of clavicle, scapula, or humerus	0.0349
Iceland	1995	Fracture of clavicle, scapula, or humerus	0.0349
Iceland	2000	Fracture of clavicle, scapula, or humerus	0.0349
Iceland	2005	Fracture of clavicle, scapula, or humerus	0.0349
Iceland	2010	Fracture of clavicle, scapula, or humerus	0.0349
Iceland	2017	Fracture of clavicle, scapula, or humerus	0.0349
Iceland	1990	Fracture of face bones	0.0669
Iceland	1995	Fracture of face bones	0.0669
Iceland	2000	Fracture of face bones	0.0669
Iceland	2005	Fracture of face bones	0.0669
Iceland	2010	Fracture of face bones	0.0669
Iceland	2017	Fracture of face bones	0.0669
Iceland	1990	Fracture of foot bones except ankle	0.0264
Iceland	1995	Fracture of foot bones except ankle	0.0264
Iceland	2000	Fracture of foot bones except ankle	0.0264
Iceland	2005	Fracture of foot bones except ankle	0.0264
Iceland	2010	Fracture of foot bones except ankle	0.0264
Iceland	2017	Fracture of foot bones except ankle	0.0264
Iceland	1990	Fracture of hand (wrist and other distal part of hand)	0.0138
Iceland	1995	Fracture of hand (wrist and other distal part of hand)	0.0138
Iceland	2000	Fracture of hand (wrist and other distal part of hand)	0.0138
Iceland	2005	Fracture of hand (wrist and other distal part of hand)	0.0138
Iceland	2010	Fracture of hand (wrist and other distal part of hand)	0.0138
Iceland	2017	Fracture of hand (wrist and other distal part of hand)	0.0138
Iceland	1990	Fracture of hip	0.0577
Iceland	1995	Fracture of hip	0.0577
Iceland	2000	Fracture of hip	0.0577
Iceland	2005	Fracture of hip	0.0577
Iceland	2010	Fracture of hip	0.0577
Iceland	2017	Fracture of hip	0.0577
Iceland	1990	Amputation of upper limbs, bilateral	0.0876
Iceland	1995	Amputation of upper limbs, bilateral	0.0876
Iceland	2000	Amputation of upper limbs, bilateral	0.0876
Iceland	2005	Amputation of upper limbs, bilateral	0.0876
Iceland	2010	Amputation of upper limbs, bilateral	0.0876
Iceland	2017	Amputation of upper limbs, bilateral	0.0876
Iceland	1990	Fracture of patella, tibia or fibula, or ankle	0.0554
Iceland	1995	Fracture of patella, tibia or fibula, or ankle	0.0554
Iceland	2000	Fracture of patella, tibia or fibula, or ankle	0.0554
Iceland	2005	Fracture of patella, tibia or fibula, or ankle	0.0554
Iceland	2010	Fracture of patella, tibia or fibula, or ankle	0.0554
Iceland	2017	Fracture of patella, tibia or fibula, or ankle	0.0554
Iceland	1990	Fracture of pelvis	0.1823
Iceland	1995	Fracture of pelvis	0.1823
Iceland	2000	Fracture of pelvis	0.1823
Iceland	2005	Fracture of pelvis	0.1823
Iceland	2010	Fracture of pelvis	0.1823
Iceland	2017	Fracture of pelvis	0.1823
Iceland	1990	Fracture of radius and/or ulna	0.0435
Iceland	1995	Fracture of radius and/or ulna	0.0435
Iceland	2000	Fracture of radius and/or ulna	0.0435
Iceland	2005	Fracture of radius and/or ulna	0.0435
Iceland	2010	Fracture of radius and/or ulna	0.0435
Iceland	2017	Fracture of radius and/or ulna	0.0435
Iceland	1990	Fracture of skull	0.0714
Iceland	1995	Fracture of skull	0.0714
Iceland	2000	Fracture of skull	0.0714
Iceland	2005	Fracture of skull	0.0714
Iceland	2010	Fracture of skull	0.0714
Iceland	2017	Fracture of skull	0.0714
Iceland	1990	Fracture of sternum and/or fracture of one or more ribs	0.1027
Iceland	1995	Fracture of sternum and/or fracture of one or more ribs	0.1027
Iceland	2000	Fracture of sternum and/or fracture of one or more ribs	0.1027
Iceland	2005	Fracture of sternum and/or fracture of one or more ribs	0.1027
Iceland	2010	Fracture of sternum and/or fracture of one or more ribs	0.1027
Iceland	2017	Fracture of sternum and/or fracture of one or more ribs	0.1027
Iceland	1990	Fracture of vertebral column	0.1106
Iceland	1995	Fracture of vertebral column	0.1106
Iceland	2000	Fracture of vertebral column	0.1106
Iceland	2005	Fracture of vertebral column	0.1106
Iceland	2010	Fracture of vertebral column	0.1106
Iceland	2017	Fracture of vertebral column	0.1106
Iceland	1990	Fracture of femur, other than femoral neck	0.042
Iceland	1995	Fracture of femur, other than femoral neck	0.042
Iceland	2000	Fracture of femur, other than femoral neck	0.042
Iceland	2005	Fracture of femur, other than femoral neck	0.042
Iceland	2010	Fracture of femur, other than femoral neck	0.042
Iceland	2017	Fracture of femur, other than femoral neck	0.042
Iceland	1990	Minor TBI	0.1319
Iceland	1995	Minor TBI	0.1319
Iceland	2000	Minor TBI	0.1319
Iceland	2005	Minor TBI	0.1319
Iceland	2010	Minor TBI	0.1319
Iceland	2017	Minor TBI	0.1319
Iceland	1990	Moderate TBI	0.1637
Iceland	1995	Moderate TBI	0.1637
Iceland	2000	Moderate TBI	0.1637
Iceland	2005	Moderate TBI	0.1637
Iceland	2010	Moderate TBI	0.1637
Iceland	2017	Moderate TBI	0.1637
Iceland	1990	Amputation of fingers (excluding thumb)	0.0047
Iceland	1995	Amputation of fingers (excluding thumb)	0.0047
Iceland	2000	Amputation of fingers (excluding thumb)	0.0047
Iceland	2005	Amputation of fingers (excluding thumb)	0.0047
Iceland	2010	Amputation of fingers (excluding thumb)	0.0047
Iceland	2017	Amputation of fingers (excluding thumb)	0.0047
Iceland	1990	Spinal cord lesion at neck level	0.4118
Iceland	1995	Spinal cord lesion at neck level	0.4118
Iceland	2000	Spinal cord lesion at neck level	0.4118
Iceland	2005	Spinal cord lesion at neck level	0.4118
Iceland	2010	Spinal cord lesion at neck level	0.4118
Iceland	2017	Spinal cord lesion at neck level	0.4118
Iceland	1990	Spinal cord lesion below neck level	0.1777
Iceland	1995	Spinal cord lesion below neck level	0.1777
Iceland	2000	Spinal cord lesion below neck level	0.1777
Iceland	2005	Spinal cord lesion below neck level	0.1777
Iceland	2010	Spinal cord lesion below neck level	0.1777
Iceland	2017	Spinal cord lesion below neck level	0.1777
Iceland	1990	Drowning and nonfatal submersion	0.2471
Iceland	1995	Drowning and nonfatal submersion	0.2471
Iceland	2000	Drowning and nonfatal submersion	0.2471
Iceland	2005	Drowning and nonfatal submersion	0.2471
Iceland	2010	Drowning and nonfatal submersion	0.2471
Iceland	2017	Drowning and nonfatal submersion	0.2471
Iceland	1990	Asphyxiation	0.2471
Iceland	1995	Asphyxiation	0.2471
Iceland	2000	Asphyxiation	0.2471
Iceland	2005	Asphyxiation	0.2471
Iceland	2010	Asphyxiation	0.2471
Iceland	2017	Asphyxiation	0.2471
Iceland	1990	Crush injury	0.1325
Iceland	1995	Crush injury	0.1325
Iceland	2000	Crush injury	0.1325
Iceland	2005	Crush injury	0.1325
Iceland	2010	Crush injury	0.1325
Iceland	2017	Crush injury	0.1325
Iceland	1990	Nerve injury	0.1129
Iceland	1995	Nerve injury	0.1129
Iceland	2000	Nerve injury	0.1129
Iceland	2005	Nerve injury	0.1129
Iceland	2010	Nerve injury	0.1129
Iceland	2017	Nerve injury	0.1129
Iceland	1990	Injury to eyes	0.0165
Iceland	1995	Injury to eyes	0.0165
Iceland	2000	Injury to eyes	0.0165
Iceland	2005	Injury to eyes	0.0165
Iceland	2010	Injury to eyes	0.0165
Iceland	2017	Injury to eyes	0.0165
Iceland	1990	Amputation of lower limb, unilateral	0.039
Iceland	1995	Amputation of lower limb, unilateral	0.039
Iceland	2000	Amputation of lower limb, unilateral	0.039
Iceland	2005	Amputation of lower limb, unilateral	0.039
Iceland	2010	Amputation of lower limb, unilateral	0.039
Iceland	2017	Amputation of lower limb, unilateral	0.039
Iceland	1990	Open wound(s)	0.011
Iceland	1995	Open wound(s)	0.011
Iceland	2000	Open wound(s)	0.011
Iceland	2005	Open wound(s)	0.011
Iceland	2010	Open wound(s)	0.011
Iceland	2017	Open wound(s)	0.011
Iceland	1990	Poisoning requiring urgent care	0.1628
Iceland	1995	Poisoning requiring urgent care	0.1628
Iceland	2000	Poisoning requiring urgent care	0.1628
Iceland	2005	Poisoning requiring urgent care	0.1628
Iceland	2010	Poisoning requiring urgent care	0.1628
Iceland	2017	Poisoning requiring urgent care	0.1628
Iceland	1990	Severe chest injury	0.0474
Iceland	1995	Severe chest injury	0.0474
Iceland	2000	Severe chest injury	0.0474
Iceland	2005	Severe chest injury	0.0474
Iceland	2010	Severe chest injury	0.0474
Iceland	2017	Severe chest injury	0.0474
Iceland	1990	Internal hemorrhage in abdomen and pelvis	0.1143
Iceland	1995	Internal hemorrhage in abdomen and pelvis	0.1143
Iceland	2000	Internal hemorrhage in abdomen and pelvis	0.1143
Iceland	2005	Internal hemorrhage in abdomen and pelvis	0.1143
Iceland	2010	Internal hemorrhage in abdomen and pelvis	0.1143
Iceland	2017	Internal hemorrhage in abdomen and pelvis	0.1143
Iceland	1990	Contusion in any part of the body	0.0075
Iceland	1995	Contusion in any part of the body	0.0075
Iceland	2000	Contusion in any part of the body	0.0075
Iceland	2005	Contusion in any part of the body	0.0075

Iceland	2010	Contusion in any part of the body	0.0075
Iceland	2017	Contusion in any part of the body	0.0075
Iceland	1990	Effect of different environmental factors	0.049
Iceland	1995	Effect of different environmental factors	0.049
Iceland	2000	Effect of different environmental factors	0.049
Iceland	2005	Effect of different environmental factors	0.049
Iceland	2010	Effect of different environmental factors	0.049
Iceland	2017	Effect of different environmental factors	0.049
Iceland	1990	Complications following therapeutic procedures	0.0506
Iceland	1995	Complications following therapeutic procedures	0.0506
Iceland	2000	Complications following therapeutic procedures	0.0506
Iceland	2005	Complications following therapeutic procedures	0.0506
Iceland	2010	Complications following therapeutic procedures	0.0506
Iceland	2017	Complications following therapeutic procedures	0.0506
Iceland	1990	Multiple fractures, dislocations, crashes, wounds, sprains, and strains	0.0577
Iceland	1995	Multiple fractures, dislocations, crashes, wounds, sprains, and strains	0.0577
Iceland	2000	Multiple fractures, dislocations, crashes, wounds, sprains, and strains	0.0577
Iceland	2005	Multiple fractures, dislocations, crashes, wounds, sprains, and strains	0.0577
Iceland	2010	Multiple fractures, dislocations, crashes, wounds, sprains, and strains	0.0577
Iceland	2017	Multiple fractures, dislocations, crashes, wounds, sprains, and strains	0.0577
Iceland	1990	Amputation of upper limb, unilateral	0.039
Iceland	1995	Amputation of upper limb, unilateral	0.039
Iceland	2000	Amputation of upper limb, unilateral	0.039
Iceland	2005	Amputation of upper limb, unilateral	0.039
Iceland	2010	Amputation of upper limb, unilateral	0.039
Iceland	2017	Amputation of upper limb, unilateral	0.039
Iceland	1990	Amputation of thumb	0.0114
Iceland	1995	Amputation of thumb	0.0114
Iceland	2000	Amputation of thumb	0.0114
Iceland	2005	Amputation of thumb	0.0114
Iceland	2010	Amputation of thumb	0.0114
Iceland	2017	Amputation of thumb	0.0114
Iceland	1990	Amputation of toe/toes	0.0059
Iceland	1995	Amputation of toe/toes	0.0059
Iceland	2000	Amputation of toe/toes	0.0059
Iceland	2005	Amputation of toe/toes	0.0059
Iceland	2010	Amputation of toe/toes	0.0059
Iceland	2017	Amputation of toe/toes	0.0059
Iceland	1990	Burns, <20% total burned surface area without lower airway burns	0.0163
Iceland	1995	Burns, <20% total burned surface area without lower airway burns	0.0163
Iceland	2000	Burns, <20% total burned surface area without lower airway burns	0.0163
Iceland	2005	Burns, <20% total burned surface area without lower airway burns	0.0163
Iceland	2010	Burns, <20% total burned surface area without lower airway burns	0.0163
Iceland	2017	Burns, <20% total burned surface area without lower airway burns	0.0163
Iceland	1990	Burns, >=20% total burned surface area or >= 10% burned surface area if head/neck or hands/wrist involved w/o lower airway burns	0.1346
Iceland	1995	Burns, >=20% total burned surface area or >= 10% burned surface area if head/neck or hands/wrist involved w/o lower airway burns	0.1346
Iceland	2000	Burns, >=20% total burned surface area or >= 10% burned surface area if head/neck or hands/wrist involved w/o lower airway burns	0.1346
Iceland	2005	Burns, >=20% total burned surface area or >= 10% burned surface area if head/neck or hands/wrist involved w/o lower airway burns	0.1346
Iceland	2010	Burns, >=20% total burned surface area or >= 10% burned surface area if head/neck or hands/wrist involved w/o lower airway burns	0.1346
Iceland	2017	Burns, >=20% total burned surface area or >= 10% burned surface area if head/neck or hands/wrist involved w/o lower airway burns	0.1346
Ireland	1990	Amputation of lower limbs, bilateral	0.1232
Ireland	1995	Amputation of lower limbs, bilateral	0.1232
Ireland	2000	Amputation of lower limbs, bilateral	0.1232
Ireland	2005	Amputation of lower limbs, bilateral	0.1232
Ireland	2010	Amputation of lower limbs, bilateral	0.1232
Ireland	2017	Amputation of lower limbs, bilateral	0.1232
Ireland	1990	Lower airway burns	0.2252
Ireland	1995	Lower airway burns	0.2252
Ireland	2000	Lower airway burns	0.2252
Ireland	2005	Lower airway burns	0.2252
Ireland	2010	Lower airway burns	0.2252
Ireland	2017	Lower airway burns	0.2252
Ireland	1990	Dislocation of hip	0.0159
Ireland	1995	Dislocation of hip	0.0159
Ireland	2000	Dislocation of hip	0.0159
Ireland	2005	Dislocation of hip	0.0159
Ireland	2010	Dislocation of hip	0.0159
Ireland	2017	Dislocation of hip	0.0159
Ireland	1990	Dislocation of knee	0.1134
Ireland	1995	Dislocation of knee	0.1134
Ireland	2000	Dislocation of knee	0.1134
Ireland	2005	Dislocation of knee	0.1134
Ireland	2010	Dislocation of knee	0.1134
Ireland	2017	Dislocation of knee	0.1134
Ireland	1990	Dislocation of shoulder	0.062
Ireland	1995	Dislocation of shoulder	0.062
Ireland	2000	Dislocation of shoulder	0.062
Ireland	2005	Dislocation of shoulder	0.062
Ireland	2010	Dislocation of shoulder	0.062
Ireland	2017	Dislocation of shoulder	0.062
Ireland	1990	Muscle and tendon injuries, including sprains and strains lesser dislocations	0.0075
Ireland	1995	Muscle and tendon injuries, including sprains and strains lesser dislocations	0.0075
Ireland	2000	Muscle and tendon injuries, including sprains and strains lesser dislocations	0.0075
Ireland	2005	Muscle and tendon injuries, including sprains and strains lesser dislocations	0.0075
Ireland	2010	Muscle and tendon injuries, including sprains and strains lesser dislocations	0.0075
Ireland	2017	Muscle and tendon injuries, including sprains and strains lesser dislocations	0.0075
Ireland	1990	Fracture of clavicle, scapula, or humerus	0.0349
Ireland	1995	Fracture of clavicle, scapula, or humerus	0.0349
Ireland	2000	Fracture of clavicle, scapula, or humerus	0.0349
Ireland	2005	Fracture of clavicle, scapula, or humerus	0.0349
Ireland	2010	Fracture of clavicle, scapula, or humerus	0.0349
Ireland	2017	Fracture of clavicle, scapula, or humerus	0.0349
Ireland	1990	Fracture of face bones	0.0669
Ireland	1995	Fracture of face bones	0.0669
Ireland	2000	Fracture of face bones	0.0669
Ireland	2005	Fracture of face bones	0.0669
Ireland	2010	Fracture of face bones	0.0669
Ireland	2017	Fracture of face bones	0.0669
Ireland	1990	Fracture of foot bones except ankle	0.0264
Ireland	1995	Fracture of foot bones except ankle	0.0264
Ireland	2000	Fracture of foot bones except ankle	0.0264
Ireland	2005	Fracture of foot bones except ankle	0.0264
Ireland	2010	Fracture of foot bones except ankle	0.0264
Ireland	2017	Fracture of foot bones except ankle	0.0264
Ireland	1990	Fracture of hand (wrist and other distal part of hand)	0.0138
Ireland	1995	Fracture of hand (wrist and other distal part of hand)	0.0138
Ireland	2000	Fracture of hand (wrist and other distal part of hand)	0.0138
Ireland	2005	Fracture of hand (wrist and other distal part of hand)	0.0138
Ireland	2010	Fracture of hand (wrist and other distal part of hand)	0.0138
Ireland	2017	Fracture of hand (wrist and other distal part of hand)	0.0138
Ireland	1990	Fracture of hip	0.0577
Ireland	1995	Fracture of hip	0.0577
Ireland	2000	Fracture of hip	0.0577
Ireland	2005	Fracture of hip	0.0577
Ireland	2010	Fracture of hip	0.0577
Ireland	2017	Fracture of hip	0.0577
Ireland	1990	Amputation of upper limbs, bilateral	0.0876
Ireland	1995	Amputation of upper limbs, bilateral	0.0876
Ireland	2000	Amputation of upper limbs, bilateral	0.0876
Ireland	2005	Amputation of upper limbs, bilateral	0.0876
Ireland	2010	Amputation of upper limbs, bilateral	0.0876
Ireland	2017	Amputation of upper limbs, bilateral	0.0876
Ireland	1990	Fracture of patella, tibia or fibula, or ankle	0.0554
Ireland	1995	Fracture of patella, tibia or fibula, or ankle	0.0554
Ireland	2000	Fracture of patella, tibia or fibula, or ankle	0.0554
Ireland	2005	Fracture of patella, tibia or fibula, or ankle	0.0554
Ireland	2010	Fracture of patella, tibia or fibula, or ankle	0.0554
Ireland	2017	Fracture of patella, tibia or fibula, or ankle	0.0554
Ireland	1990	Fracture of pelvis	0.1823
Ireland	1995	Fracture of pelvis	0.1823
Ireland	2000	Fracture of pelvis	0.1823
Ireland	2005	Fracture of pelvis	0.1823
Ireland	2010	Fracture of pelvis	0.1823
Ireland	2017	Fracture of pelvis	0.1823
Ireland	1990	Fracture of radius and/or ulna	0.0435
Ireland	1995	Fracture of radius and/or ulna	0.0435
Ireland	2000	Fracture of radius and/or ulna	0.0435
Ireland	2005	Fracture of radius and/or ulna	0.0435
Ireland	2010	Fracture of radius and/or ulna	0.0435
Ireland	2017	Fracture of radius and/or ulna	0.0435
Ireland	1990	Fracture of skull	0.0714
Ireland	1995	Fracture of skull	0.0714
Ireland	2000	Fracture of skull	0.0714
Ireland	2005	Fracture of skull	0.0714
Ireland	2010	Fracture of skull	0.0714
Ireland	2017	Fracture of skull	0.0714
Ireland	1990	Fracture of sternum and/or fracture of one or more ribs	0.1027
Ireland	1995	Fracture of sternum and/or fracture of one or more ribs	0.1027
Ireland	2000	Fracture of sternum and/or fracture of one or more ribs	0.1027
Ireland	2005	Fracture of sternum and/or fracture of one or more ribs	0.1027
Ireland	2010	Fracture of sternum and/or fracture of one or more ribs	0.1027
Ireland	2017	Fracture of sternum and/or fracture of one or more ribs	0.1027
Ireland	1990	Fracture of vertebral column	0.1106
Ireland	1995	Fracture of vertebral column	0.1106
Ireland	2000	Fracture of vertebral column	0.1106
Ireland	2005	Fracture of vertebral column	0.1106
Ireland	2010	Fracture of vertebral column	0.1106
Ireland	2017	Fracture of vertebral column	0.1106
Ireland	1990	Fracture of femur, other than femoral neck	0.042
Ireland	1995	Fracture of femur, other than femoral neck	0.042
Ireland	2000	Fracture of femur, other than femoral neck	0.042
Ireland	2005	Fracture of femur, other than femoral neck	0.042
Ireland	2010	Fracture of femur, other than femoral neck	0.042
Ireland	2017	Fracture of femur, other than femoral neck	0.042
Ireland	1990	Minor TBI	0.1319
Ireland	1995	Minor TBI	0.1319
Ireland	2000	Minor TBI	0.1319
Ireland	2005	Minor TBI	0.1319
Ireland	2010	Minor TBI	0.1319
Ireland	2017	Minor TBI	0.1319
Ireland	1990	Moderate TBI	0.1637
Ireland	1995	Moderate TBI	0.1637
Ireland	2000	Moderate TBI	0.1637
Ireland	2005	Moderate TBI	0.1637
Ireland	2010	Moderate TBI	0.1637
Ireland	2017	Moderate TBI	0.1637
Ireland	1990	Amputation of fingers (excluding thumb)	0.0047
Ireland	1995	Amputation of fingers (excluding thumb)	0.0047
Ireland	2000	Amputation of fingers (excluding thumb)	0.0047
Ireland	2005	Amputation of fingers (excluding thumb)	0.0047
Ireland	2010	Amputation of fingers (excluding thumb)	0.0047
Ireland	2017	Amputation of fingers (excluding thumb)	0.0047
Ireland	1990	Spinal cord lesion at neck level	0.4118
Ireland	1995	Spinal cord lesion at neck level	0.4118
Ireland	2000	Spinal cord lesion at neck level	0.4118
Ireland	2005	Spinal cord lesion at neck level	0.4118
Ireland	2010	Spinal cord lesion at neck level	0.4118
Ireland	2017	Spinal cord lesion at neck level	0.4118
Ireland	1990	Spinal cord lesion below neck level	0.1777
Ireland	1995	Spinal cord lesion below neck level	0.1777
Ireland	2000	Spinal cord lesion below neck level	0.1777
Ireland	2005	Spinal cord lesion below neck level	0.1777
Ireland	2010	Spinal cord lesion below neck level	0.1777
Ireland	2017	Spinal cord lesion below neck level	0.1777
Ireland	1990	Drowning and nonfatal submersion	0.2471
Ireland	1995	Drowning and nonfatal submersion	0.2471
Ireland	2000	Drowning and nonfatal submersion	0.2471
Ireland	2005	Drowning and nonfatal submersion	0.2471
Ireland	2010	Drowning and nonfatal submersion	0.2471
Ireland	2017	Drowning and nonfatal submersion	0.2471
Ireland	1990	Asphyxiation	0.2471
Ireland	1995	Asphyxiation	0.2471
Ireland	2000	Asphyxiation	0.2471
Ireland	2005	Asphyxiation	0.2471
Ireland	2010	Asphyxiation	0.2471
Ireland	2017	Asphyxiation	0.2471
Ireland	1990	Crush injury	0.1325
Ireland	1995	Crush injury	0.1325
Ireland	2000	Crush injury	0.1325
Ireland	2005	Crush injury	0.1325
Ireland	2010	Crush injury	0.1325
Ireland	2017	Crush injury	0.1325
Ireland	1990	Nerve injury	0.1129
Ireland	1995	Nerve injury	0.1129
Ireland	2000	Nerve injury	0.1129
Ireland	2005	Nerve injury	0.1129
Ireland	2010	Nerve injury	0.1129
Ireland	2017	Nerve injury	0.1129
Ireland	1990	Injury to eyes	0.0165
Ireland	1995	Injury to eyes	0.0165
Ireland	2000	Injury to eyes	0.0165
Ireland	2005	Injury to eyes	0.0165
Ireland	2010	Injury to eyes	0.0165
Ireland	2017	Injury to eyes	0.0165
Ireland	1990	Amputation of lower limb, unilateral	0.039
Ireland	1995	Amputation of lower limb, unilateral	0.039
Ireland	2000	Amputation of lower limb, unilateral	0.039
Ireland	2005	Amputation of lower limb, unilateral	0.039
Ireland	2010	Amputation of lower limb, unilateral	0.039
Ireland	2017	Amputation of lower limb, unilateral	0.039
Ireland	1990	Open wound(s)	0.011
Ireland	1995	Open wound(s)	0.011
Ireland	2000	Open wound(s)	0.011
Ireland	2005	Open wound(s)	0.011
Ireland	2010	Open wound(s)	0.011
Ireland	2017	Open wound(s)	0.011
Ireland	1990	Poisoning requiring urgent care	0.1628
Ireland	1995	Poisoning requiring urgent care	0.1628
Ireland	2000	Poisoning requiring urgent care	0.1628
Ireland	2005	Poisoning requiring urgent care	0.1628
Ireland	2010	Poisoning requiring urgent care	0.1628
Ireland	2017	Poisoning requiring urgent care	0.1628
Ireland	1990	Severe chest injury	0.0474
Ireland	1995	Severe chest injury	0.0474
Ireland	2000	Severe chest injury	0.0474
Ireland	2005	Severe chest injury	0.0474
Ireland	2010	Severe chest injury	0.0474
Ireland	2017	Severe chest injury	0.0474
Ireland	1990	Internal hemorrhage in abdomen and pelvis	0.1143
Ireland	1995	Internal hemorrhage in abdomen and pelvis	0.1143
Ireland	2000	Internal hemorrhage in abdomen and pelvis	0.1143
Ireland	2005	Internal hemorrhage in abdomen and pelvis	0.1143
Ireland	2010	Internal hemorrhage in abdomen and pelvis	0.1143
Ireland	2017	Internal hemorrhage in abdomen and pelvis	0.1143
Ireland	1990	Contusion in any part of the body	0.0075
Ireland	1995	Contusion in any part of the body	0.0075
Ireland	2000	Contusion in any part of the body	0.0075
Ireland	2005	Contusion in any part of the body	0.0075
Ireland	2010	Contusion in any part of the body	0.0075
Ireland	2017	Contusion in any part of the body	0.0075
Ireland	1990	Effect of different environmental factors	0.049
Ireland	1995	Effect of different environmental factors	0.049
Ireland	2000	Effect of different environmental factors	0.049
Ireland	2005	Effect of different environmental factors	0.049
Ireland	2010	Effect of different environmental factors	0.049
Ireland	2017	Effect of different environmental factors	0.049
Ireland	1990	Complications following therapeutic procedures	0.0506
Ireland	1995	Complications following therapeutic procedures	0.0506
Ireland	2000	Complications following therapeutic procedures	0.0506
Ireland	2005	Complications following therapeutic procedures	0.0506
Ireland	2010	Complications following therapeutic procedures	0.0506
Ireland	2017	Complications following therapeutic procedures	0.0506
Ireland	1990	Multiple fractures, dislocations, crashes, wounds, sprains, and strains	0.0577
Ireland	1995	Multiple fractures, dislocations, crashes, wounds, sprains, and strains	0.0577
Ireland	2000	Multiple fractures, dislocations, crashes, wounds, sprains, and strains	0.0577
Ireland	2005	Multiple fractures, dislocations, crashes, wounds, sprains, and strains	0.0577
Ireland	2010	Multiple fractures, dislocations, crashes, wounds, sprains, and strains	0.0577
Ireland	2017	Multiple fractures, dislocations, crashes, wounds, sprains, and strains	0.0577
Ireland	1990	Amputation of upper limb, unilateral	0.039
Ireland	1995	Amputation of upper limb, unilateral	0.039
Ireland	2000	Amputation of upper limb, unilateral	0.039
Ireland	2005	Amputation of upper limb, unilateral	0.039
Ireland	2010	Amputation of upper limb, unilateral	0.039
Ireland	2017	Amputation of upper limb, unilateral	0.039
Ireland	1990	Amputation of thumb	0.0114
Ireland	1995	Amputation of thumb	0.0114
Ireland	2000	Amputation of thumb	0.0114
Ireland	2005	Amputation of thumb	0.0114
Ireland	2010	Amputation of thumb	0.0114
Ireland	2017	Amputation of thumb	0.0114
Ireland	1990	Amputation of toe/toes	0.0059
Ireland	1995	Amputation of toe/toes	0.0059
Ireland	2000	Amputation of toe/toes	0.0059
Ireland	2005	Amputation of toe/toes	0.0059
Ireland	2010	Amputation of toe/toes	0.0059
Ireland	2017	Amputation of toe/toes	0.0059
Ireland	1990	Burns, <20% total burned surface area without lower airway burns	0.0163
Ireland	1995	Burns, <20% total burned surface area without lower airway burns	0.0163
Ireland	2000	Burns, <20% total burned surface area without lower airway burns	0.0163
Ireland	2005	Burns, <20% total burned surface area without lower airway burns	0.0163
Ireland	2010	Burns, <20% total burned surface area without lower airway burns	0.0163
Ireland	2017	Burns, <20% total burned surface area without lower airway burns	0.0163
Ireland	1990	Burns, >=20% total burned surface area or >= 10% burned surface area if head/neck or hands/wrist involved w/o lower airway burns	0.1346
Ireland	1995	Burns, >=20% total burned surface area or >= 10% burned surface area if head/neck or hands/wrist involved w/o lower airway burns	0.1346
Ireland	2000	Burns, >=20% total burned surface area or >= 10% burned surface area if head/neck or hands/wrist involved w/o lower airway burns	0.1346
Ireland	2005	Burns, >=20% total burned surface area or >= 10% burned surface area if head/neck or hands/wrist involved w/o lower airway burns	0.1346
Ireland	2010	Burns, >=20% total burned surface area or >= 10% burned surface area if head/neck or hands/wrist involved w/o lower airway burns	0.1346
Ireland	2017	Burns, >=20% total burned surface area or >= 10% burned surface area if head/neck or hands/wrist involved w/o lower airway burns	0.1346
Israel	1990	Amputation of lower limbs, bilateral	0.1359
Israel	1995	Amputation of lower limbs, bilateral	0.1234
Israel	2000	Amputation of lower limbs, bilateral	0.1232
Israel	2005	Amputation of lower limbs, bilateral	0.1232
Israel	2010	Amputation of lower limbs, bilateral	0.1232
Israel	2017	Amputation of lower limbs, bilateral	0.1232
Israel	1990	Lower airway burns	0.2252
Israel	1995	Lower airway burns	0.2252
Israel	2000	Lower airway burns	0.2252
Israel	2005	Lower airway burns	0.2252
Israel	2010	Lower airway burns	0.2252
Israel	2017	Lower airway burns	0.2252
Israel	1990	Dislocation of hip	0.0159
Israel	1995	Dislocation of hip	0.0159
Israel	2000	Dislocation of hip	0.0159
Israel	2005	Dislocation of hip	0.0159
Israel	2010	Dislocation of hip	0.0159
Israel	2017	Dislocation of hip	0.0159
Israel	1990	Dislocation of knee	0.1134
Israel	1995	Dislocation of knee	0.1134
Israel	2000	Dislocation of knee	0.1134
Israel	2005	Dislocation of knee	0.1134
Israel	2010	Dislocation of knee	0.1134
Israel	2017	Dislocation of knee	0.1134
Israel	1990	Dislocation of shoulder	0.062
Israel	1995	Dislocation of shoulder	0.062
Israel	2000	Dislocation of shoulder	0.062
Israel	2005	Dislocation of shoulder	0.062
Israel	2010	Dislocation of shoulder	0.062
Israel	2017	Dislocation of shoulder	0.062
Israel	1990	Muscle and tendon injuries, including sprains and strains lesser dislocations	0.0075
Israel	1995	Muscle and tendon injuries, including sprains and strains lesser dislocations	0.0075
Israel	2000	Muscle and tendon injuries, including sprains and strains lesser dislocations	0.0075
Israel	2005	Muscle and tendon injuries, including sprains and strains lesser dislocations	0.0075
Israel	2010	Muscle and tendon injuries, including sprains and strains lesser dislocations	0.0075
Israel	2017	Muscle and tendon injuries, including sprains and strains lesser dislocations	0.0075
Israel	1990	Fracture of clavicle, scapula, or humerus	0.0349
Israel	1995	Fracture of clavicle, scapula, or humerus	0.0349
Israel	2000	Fracture of clavicle, scapula, or humerus	0.0349
Israel	2005	Fracture of clavicle, scapula, or humerus	0.0349
Israel	2010	Fracture of clavicle, scapula, or humerus	0.0349
Israel	2017	Fracture of clavicle, scapula, or humerus	0.0349
Israel	1990	Fracture of face bones	0.0669
Israel	1995	Fracture of face bones	0.0669
Israel	2000	Fracture of face bones	0.0669
Israel	2005	Fracture of face bones	0.0669
Israel	2010	Fracture of face bones	0.0669
Israel	2017	Fracture of face bones	0.0669
Israel	1990	Fracture of foot bones except ankle	0.0264
Israel	1995	Fracture of foot bones except ankle	0.0264
Israel	2000	Fracture of foot bones except ankle	0.0264
Israel	2005	Fracture of foot bones except ankle	0.0264
Israel	2010	Fracture of foot bones except ankle	0.0264
Israel	2017	Fracture of foot bones except ankle	0.0264
Israel	1990	Fracture of hand (wrist and other distal part of hand)	0.0138
Israel	1995	Fracture of hand (wrist and other distal part of hand)	0.0138
Israel	2000	Fracture of hand (wrist and other distal part of hand)	0.0138
Israel	2005	Fracture of hand (wrist and other distal part of hand)	0.0138
Israel	2010	Fracture of hand (wrist and other distal part of hand)	0.0138
Israel	2017	Fracture of hand (wrist and other distal part of hand)	0.0138
Israel	1990	Fracture of hip	0.0745
Israel	1995	Fracture of hip	0.0579
Israel	2000	Fracture of hip	0.0577
Israel	2005	Fracture of hip	0.0577
Israel	2010	Fracture of hip	0.0577
Israel	2017	Fracture of hip	0.0577
Israel	1990	Amputation of upper limbs, bilateral	0.105
Israel	1995	Amputation of upper limbs, bilateral	0.0878
Israel	2000	Amputation of upper limbs, bilateral	0.0876
Israel	2005	Amputation of upper limbs, bilateral	0.0876
Israel	2010	Amputation of upper limbs, bilateral	0.0876
Israel	2017	Amputation of upper limbs, bilateral	0.0876
Israel	1990	Fracture of patella, tibia or fibula, or ankle	0.0554
Israel	1995	Fracture of patella, tibia or fibula, or ankle	0.0554
Israel	2000	Fracture of patella, tibia or fibula, or ankle	0.0554
Israel	2005	Fracture of patella, tibia or fibula, or ankle	0.0554
Israel	2010	Fracture of patella, tibia or fibula, or ankle	0.0554
Israel	2017	Fracture of patella, tibia or fibula, or ankle	0.0554
Israel	1990	Fracture of pelvis	0.1823
Israel	1995	Fracture of pelvis	0.1823
Israel	2000	Fracture of pelvis	0.1823
Israel	2005	Fracture of pelvis	0.1823
Israel	2010	Fracture of pelvis	0.1823
Israel	2017	Fracture of pelvis	0.1823
Israel	1990	Fracture of radius and/or ulna	0.0435
Israel	1995	Fracture of radius and/or ulna	0.0435
Israel	2000	Fracture of radius and/or ulna	0.0435
Israel	2005	Fracture of radius and/or ulna	0.0435
Israel	2010	Fracture of radius and/or ulna	0.0435
Israel	2017	Fracture of radius and/or ulna	0.0435
Israel	1990	Fracture of skull	0.0714
Israel	1995	Fracture of skull	0.0714
Israel	2000	Fracture of skull	0.0714
Israel	2005	Fracture of skull	0.0714
Israel	2010	Fracture of skull	0.0714
Israel	2017	Fracture of skull	0.0714
Israel	1990	Fracture of sternum and/or fracture of one or more ribs	0.1027
Israel	1995	Fracture of sternum and/or fracture of one or more ribs	0.1027
Israel	2000	Fracture of sternum and/or fracture of one or more ribs	0.1027
Israel	2005	Fracture of sternum and/or fracture of one or more ribs	0.1027
Israel	2010	Fracture of sternum and/or fracture of one or more ribs	0.1027
Israel	2017	Fracture of sternum and/or fracture of one or more ribs	0.1027
Israel	1990	Fracture of vertebral column	0.1106
Israel	1995	Fracture of vertebral column	0.1106
Israel	2000	Fracture of vertebral column	0.1106
Israel	2005	Fracture of vertebral column	0.1106
Israel	2010	Fracture of vertebral column	0.1106
Israel	2017	Fracture of vertebral column	0.1106
Israel	1990	Fracture of femur, other than femoral neck	0.042
Israel	1995	Fracture of femur, other than femoral neck	0.042
Israel	2000	Fracture of femur, other than femoral neck	0.042
Israel	2005	Fracture of femur, other than femoral neck	0.042
Israel	2010	Fracture of femur, other than femoral neck	0.042
Israel	2017	Fracture of femur, other than femoral neck	0.042
Israel	1990	Minor TBI	0.1319
Israel	1995	Minor TBI	0.1319
Israel	2000	Minor TBI	0.1319
Israel	2005	Minor TBI	0.1319
Israel	2010	Minor TBI	0.1319
Israel	2017	Minor TBI	0.1319
Israel	1990	Moderate TBI	0.1637
Israel	1995	Moderate TBI	0.1637
Israel	2000	Moderate TBI	0.1637

Israel	2005	Moderate TBI	0.1637
Israel	2010	Moderate TBI	0.1637
Israel	2017	Moderate TBI	0.1637
Israel	1990	Amputation of fingers (excluding thumb)	0.0047
Israel	1995	Amputation of fingers (excluding thumb)	0.0047
Israel	2000	Amputation of fingers (excluding thumb)	0.0047
Israel	2005	Amputation of fingers (excluding thumb)	0.0047
Israel	2010	Amputation of fingers (excluding thumb)	0.0047
Israel	2017	Amputation of fingers (excluding thumb)	0.0047
Israel	1990	Spinal cord lesion at neck level	0.4176
Israel	1995	Spinal cord lesion at neck level	0.4119
Israel	2000	Spinal cord lesion at neck level	0.4118
Israel	2005	Spinal cord lesion at neck level	0.4118
Israel	2010	Spinal cord lesion at neck level	0.4118
Israel	2017	Spinal cord lesion at neck level	0.4118
Israel	1990	Spinal cord lesion below neck level	0.1899
Israel	1995	Spinal cord lesion below neck level	0.1778
Israel	2000	Spinal cord lesion below neck level	0.1777
Israel	2005	Spinal cord lesion below neck level	0.1777
Israel	2010	Spinal cord lesion below neck level	0.1777
Israel	2017	Spinal cord lesion below neck level	0.1777
Israel	1990	Drowning and nonfatal submersion	0.2471
Israel	1995	Drowning and nonfatal submersion	0.2471
Israel	2000	Drowning and nonfatal submersion	0.2471
Israel	2005	Drowning and nonfatal submersion	0.2471
Israel	2010	Drowning and nonfatal submersion	0.2471
Israel	2017	Drowning and nonfatal submersion	0.2471
Israel	1990	Asphyxiation	0.2471
Israel	1995	Asphyxiation	0.2471
Israel	2000	Asphyxiation	0.2471
Israel	2005	Asphyxiation	0.2471
Israel	2010	Asphyxiation	0.2471
Israel	2017	Asphyxiation	0.2471
Israel	1990	Crush injury	0.1325
Israel	1995	Crush injury	0.1325
Israel	2000	Crush injury	0.1325
Israel	2005	Crush injury	0.1325
Israel	2010	Crush injury	0.1325
Israel	2017	Crush injury	0.1325
Israel	1990	Nerve injury	0.1129
Israel	1995	Nerve injury	0.1129
Israel	2000	Nerve injury	0.1129
Israel	2005	Nerve injury	0.1129
Israel	2010	Nerve injury	0.1129
Israel	2017	Nerve injury	0.1129
Israel	1990	Injury to eyes	0.0165
Israel	1995	Injury to eyes	0.0165
Israel	2000	Injury to eyes	0.0165
Israel	2005	Injury to eyes	0.0165
Israel	2010	Injury to eyes	0.0165
Israel	2017	Injury to eyes	0.0165
Israel	1990	Amputation of lower limb, unilateral	0.0455
Israel	1995	Amputation of lower limb, unilateral	0.039
Israel	2000	Amputation of lower limb, unilateral	0.039
Israel	2005	Amputation of lower limb, unilateral	0.039
Israel	2010	Amputation of lower limb, unilateral	0.039
Israel	2017	Amputation of lower limb, unilateral	0.039
Israel	1990	Open wound(s)	0.011
Israel	1995	Open wound(s)	0.011
Israel	2000	Open wound(s)	0.011
Israel	2005	Open wound(s)	0.011
Israel	2010	Open wound(s)	0.011
Israel	2017	Open wound(s)	0.011
Israel	1990	Poisoning requiring urgent care	0.1628
Israel	1995	Poisoning requiring urgent care	0.1628
Israel	2000	Poisoning requiring urgent care	0.1628
Israel	2005	Poisoning requiring urgent care	0.1628
Israel	2010	Poisoning requiring urgent care	0.1628
Israel	2017	Poisoning requiring urgent care	0.1628
Israel	1990	Severe chest injury	0.0474
Israel	1995	Severe chest injury	0.0474
Israel	2000	Severe chest injury	0.0474
Israel	2005	Severe chest injury	0.0474
Israel	2010	Severe chest injury	0.0474
Israel	2017	Severe chest injury	0.0474
Israel	1990	Internal hemorrhage in abdomen and pelvis	0.1143
Israel	1995	Internal hemorrhage in abdomen and pelvis	0.1143
Israel	2000	Internal hemorrhage in abdomen and pelvis	0.1143
Israel	2005	Internal hemorrhage in abdomen and pelvis	0.1143
Israel	2010	Internal hemorrhage in abdomen and pelvis	0.1143
Israel	2017	Internal hemorrhage in abdomen and pelvis	0.1143
Israel	1990	Contusion in any part of the body	0.0075
Israel	1995	Contusion in any part of the body	0.0075
Israel	2000	Contusion in any part of the body	0.0075
Israel	2005	Contusion in any part of the body	0.0075
Israel	2010	Contusion in any part of the body	0.0075
Israel	2017	Contusion in any part of the body	0.0075
Israel	1990	Effect of different environmental factors	0.049
Israel	1995	Effect of different environmental factors	0.049
Israel	2000	Effect of different environmental factors	0.049
Israel	2005	Effect of different environmental factors	0.049
Israel	2010	Effect of different environmental factors	0.049
Israel	2017	Effect of different environmental factors	0.049
Israel	1990	Complications following therapeutic procedures	0.0506
Israel	1995	Complications following therapeutic procedures	0.0506
Israel	2000	Complications following therapeutic procedures	0.0506
Israel	2005	Complications following therapeutic procedures	0.0506
Israel	2010	Complications following therapeutic procedures	0.0506
Israel	2017	Complications following therapeutic procedures	0.0506
Israel	1990	Multiple fractures, dislocations, crashes, wounds, sprains, and strains	0.0745
Israel	1995	Multiple fractures, dislocations, crashes, wounds, sprains, and strains	0.0579
Israel	2000	Multiple fractures, dislocations, crashes, wounds, sprains, and strains	0.0577
Israel	2005	Multiple fractures, dislocations, crashes, wounds, sprains, and strains	0.0577
Israel	2010	Multiple fractures, dislocations, crashes, wounds, sprains, and strains	0.0577
Israel	2017	Multiple fractures, dislocations, crashes, wounds, sprains, and strains	0.0577
Israel	1990	Amputation of upper limb, unilateral	0.0428
Israel	1995	Amputation of upper limb, unilateral	0.039
Israel	2000	Amputation of upper limb, unilateral	0.039
Israel	2005	Amputation of upper limb, unilateral	0.039
Israel	2010	Amputation of upper limb, unilateral	0.039
Israel	2017	Amputation of upper limb, unilateral	0.039
Israel	1990	Amputation of thumb	0.0114
Israel	1995	Amputation of thumb	0.0114
Israel	2000	Amputation of thumb	0.0114
Israel	2005	Amputation of thumb	0.0114
Israel	2010	Amputation of thumb	0.0114
Israel	2017	Amputation of thumb	0.0114
Israel	1990	Amputation of toe/toes	0.0059
Israel	1995	Amputation of toe/toes	0.0059
Israel	2000	Amputation of toe/toes	0.0059
Israel	2005	Amputation of toe/toes	0.0059
Israel	2010	Amputation of toe/toes	0.0059
Israel	2017	Amputation of toe/toes	0.0059
Israel	1990	Burns, <20% total burned surface area without lower airway burns	0.0163
Israel	1995	Burns, <20% total burned surface area without lower airway burns	0.0163
Israel	2000	Burns, <20% total burned surface area without lower airway burns	0.0163
Israel	2005	Burns, <20% total burned surface area without lower airway burns	0.0163
Israel	2010	Burns, <20% total burned surface area without lower airway burns	0.0163
Israel	2017	Burns, <20% total burned surface area without lower airway burns	0.0163
Israel	1990	Burns, >=20% total burned surface area or >= 10% burned surface area if head/neck or hands/wrist involved w/o lower airway burns	0.1502
Israel	1995	Burns, >=20% total burned surface area or >= 10% burned surface area if head/neck or hands/wrist involved w/o lower airway burns	0.1347
Israel	2000	Burns, >=20% total burned surface area or >= 10% burned surface area if head/neck or hands/wrist involved w/o lower airway burns	0.1346
Israel	2005	Burns, >=20% total burned surface area or >= 10% burned surface area if head/neck or hands/wrist involved w/o lower airway burns	0.1346
Israel	2010	Burns, >=20% total burned surface area or >= 10% burned surface area if head/neck or hands/wrist involved w/o lower airway burns	0.1346
Israel	2017	Burns, >=20% total burned surface area or >= 10% burned surface area if head/neck or hands/wrist involved w/o lower airway burns	0.1346
Italy	1990	Amputation of lower limbs, bilateral	0.1232
Italy	1995	Amputation of lower limbs, bilateral	0.1232
Italy	2000	Amputation of lower limbs, bilateral	0.1232
Italy	2005	Amputation of lower limbs, bilateral	0.1232
Italy	2010	Amputation of lower limbs, bilateral	0.1232
Italy	2017	Amputation of lower limbs, bilateral	0.1232
Italy	1990	Lower airway burns	0.2252
Italy	1995	Lower airway burns	0.2252
Italy	2000	Lower airway burns	0.2252
Italy	2005	Lower airway burns	0.2252
Italy	2010	Lower airway burns	0.2252
Italy	2017	Lower airway burns	0.2252
Italy	1990	Dislocation of hip	0.0159
Italy	1995	Dislocation of hip	0.0159
Italy	2000	Dislocation of hip	0.0159
Italy	2005	Dislocation of hip	0.0159
Italy	2010	Dislocation of hip	0.0159
Italy	2017	Dislocation of hip	0.0159
Italy	1990	Dislocation of knee	0.1134
Italy	1995	Dislocation of knee	0.1134
Italy	2000	Dislocation of knee	0.1134
Italy	2005	Dislocation of knee	0.1134
Italy	2010	Dislocation of knee	0.1134
Italy	2017	Dislocation of knee	0.1134
Italy	1990	Dislocation of shoulder	0.062
Italy	1995	Dislocation of shoulder	0.062
Italy	2000	Dislocation of shoulder	0.062
Italy	2005	Dislocation of shoulder	0.062
Italy	2010	Dislocation of shoulder	0.062
Italy	2017	Dislocation of shoulder	0.062
Italy	1990	Muscle and tendon injuries, including sprains and strains lesser dislocations	0.0075
Italy	1995	Muscle and tendon injuries, including sprains and strains lesser dislocations	0.0075
Italy	2000	Muscle and tendon injuries, including sprains and strains lesser dislocations	0.0075
Italy	2005	Muscle and tendon injuries, including sprains and strains lesser dislocations	0.0075
Italy	2010	Muscle and tendon injuries, including sprains and strains lesser dislocations	0.0075
Italy	2017	Muscle and tendon injuries, including sprains and strains lesser dislocations	0.0075
Italy	1990	Fracture of clavicle, scapula, or humerus	0.0349
Italy	1995	Fracture of clavicle, scapula, or humerus	0.0349
Italy	2000	Fracture of clavicle, scapula, or humerus	0.0349
Italy	2005	Fracture of clavicle, scapula, or humerus	0.0349
Italy	2010	Fracture of clavicle, scapula, or humerus	0.0349
Italy	2017	Fracture of clavicle, scapula, or humerus	0.0349
Italy	1990	Fracture of face bones	0.0669
Italy	1995	Fracture of face bones	0.0669
Italy	2000	Fracture of face bones	0.0669
Italy	2005	Fracture of face bones	0.0669
Italy	2010	Fracture of face bones	0.0669
Italy	2017	Fracture of face bones	0.0669
Italy	1990	Fracture of foot bones except ankle	0.0264
Italy	1995	Fracture of foot bones except ankle	0.0264
Italy	2000	Fracture of foot bones except ankle	0.0264
Italy	2005	Fracture of foot bones except ankle	0.0264
Italy	2010	Fracture of foot bones except ankle	0.0264
Italy	2017	Fracture of foot bones except ankle	0.0264
Italy	1990	Fracture of hand (wrist and other distal part of hand)	0.0138
Italy	1995	Fracture of hand (wrist and other distal part of hand)	0.0138
Italy	2000	Fracture of hand (wrist and other distal part of hand)	0.0138
Italy	2005	Fracture of hand (wrist and other distal part of hand)	0.0138
Italy	2010	Fracture of hand (wrist and other distal part of hand)	0.0138
Italy	2017	Fracture of hand (wrist and other distal part of hand)	0.0138
Italy	1990	Fracture of hip	0.0577
Italy	1995	Fracture of hip	0.0577
Italy	2000	Fracture of hip	0.0577
Italy	2005	Fracture of hip	0.0577
Italy	2010	Fracture of hip	0.0577
Italy	2017	Fracture of hip	0.0577
Italy	1990	Amputation of upper limbs, bilateral	0.0876
Italy	1995	Amputation of upper limbs, bilateral	0.0876
Italy	2000	Amputation of upper limbs, bilateral	0.0876
Italy	2005	Amputation of upper limbs, bilateral	0.0876
Italy	2010	Amputation of upper limbs, bilateral	0.0876
Italy	2017	Amputation of upper limbs, bilateral	0.0876
Italy	1990	Fracture of patella, tibia or fibula, or ankle	0.0554
Italy	1995	Fracture of patella, tibia or fibula, or ankle	0.0554
Italy	2000	Fracture of patella, tibia or fibula, or ankle	0.0554
Italy	2005	Fracture of patella, tibia or fibula, or ankle	0.0554
Italy	2010	Fracture of patella, tibia or fibula, or ankle	0.0554
Italy	2017	Fracture of patella, tibia or fibula, or ankle	0.0554
Italy	1990	Fracture of pelvis	0.1823
Italy	1995	Fracture of pelvis	0.1823
Italy	2000	Fracture of pelvis	0.1823
Italy	2005	Fracture of pelvis	0.1823
Italy	2010	Fracture of pelvis	0.1823
Italy	2017	Fracture of pelvis	0.1823
Italy	1990	Fracture of radius and/or ulna	0.0435
Italy	1995	Fracture of radius and/or ulna	0.0435
Italy	2000	Fracture of radius and/or ulna	0.0435
Italy	2005	Fracture of radius and/or ulna	0.0435
Italy	2010	Fracture of radius and/or ulna	0.0435
Italy	2017	Fracture of radius and/or ulna	0.0435
Italy	1990	Fracture of skull	0.0714
Italy	1995	Fracture of skull	0.0714
Italy	2000	Fracture of skull	0.0714
Italy	2005	Fracture of skull	0.0714
Italy	2010	Fracture of skull	0.0714
Italy	2017	Fracture of skull	0.0714
Italy	1990	Fracture of sternum and/or fracture of one or more ribs	0.1027
Italy	1995	Fracture of sternum and/or fracture of one or more ribs	0.1027
Italy	2000	Fracture of sternum and/or fracture of one or more ribs	0.1027
Italy	2005	Fracture of sternum and/or fracture of one or more ribs	0.1027
Italy	2010	Fracture of sternum and/or fracture of one or more ribs	0.1027
Italy	2017	Fracture of sternum and/or fracture of one or more ribs	0.1027
Italy	1990	Fracture of vertebral column	0.1106
Italy	1995	Fracture of vertebral column	0.1106
Italy	2000	Fracture of vertebral column	0.1106
Italy	2005	Fracture of vertebral column	0.1106
Italy	2010	Fracture of vertebral column	0.1106
Italy	2017	Fracture of vertebral column	0.1106
Italy	1990	Fracture of femur, other than femoral neck	0.042
Italy	1995	Fracture of femur, other than femoral neck	0.042
Italy	2000	Fracture of femur, other than femoral neck	0.042
Italy	2005	Fracture of femur, other than femoral neck	0.042
Italy	2010	Fracture of femur, other than femoral neck	0.042
Italy	2017	Fracture of femur, other than femoral neck	0.042
Italy	1990	Minor TBI	0.1319
Italy	1995	Minor TBI	0.1319
Italy	2000	Minor TBI	0.1319
Italy	2005	Minor TBI	0.1319
Italy	2010	Minor TBI	0.1319
Italy	2017	Minor TBI	0.1319
Italy	1990	Moderate TBI	0.1637
Italy	1995	Moderate TBI	0.1637
Italy	2000	Moderate TBI	0.1637
Italy	2005	Moderate TBI	0.1637
Italy	2010	Moderate TBI	0.1637
Italy	2017	Moderate TBI	0.1637
Italy	1990	Amputation of fingers (excluding thumb)	0.0047
Italy	1995	Amputation of fingers (excluding thumb)	0.0047
Italy	2000	Amputation of fingers (excluding thumb)	0.0047
Italy	2005	Amputation of fingers (excluding thumb)	0.0047
Italy	2010	Amputation of fingers (excluding thumb)	0.0047
Italy	2017	Amputation of fingers (excluding thumb)	0.0047
Italy	1990	Spinal cord lesion at neck level	0.4118
Italy	1995	Spinal cord lesion at neck level	0.4118
Italy	2000	Spinal cord lesion at neck level	0.4118
Italy	2005	Spinal cord lesion at neck level	0.4118
Italy	2010	Spinal cord lesion at neck level	0.4118
Italy	2017	Spinal cord lesion at neck level	0.4118
Italy	1990	Spinal cord lesion below neck level	0.1777
Italy	1995	Spinal cord lesion below neck level	0.1777
Italy	2000	Spinal cord lesion below neck level	0.1777
Italy	2005	Spinal cord lesion below neck level	0.1777
Italy	2010	Spinal cord lesion below neck level	0.1777
Italy	2017	Spinal cord lesion below neck level	0.1777
Italy	1990	Drowning and nonfatal submersion	0.2471
Italy	1995	Drowning and nonfatal submersion	0.2471
Italy	2000	Drowning and nonfatal submersion	0.2471
Italy	2005	Drowning and nonfatal submersion	0.2471
Italy	2010	Drowning and nonfatal submersion	0.2471
Italy	2017	Drowning and nonfatal submersion	0.2471
Italy	1990	Asphyxiation	0.2471
Italy	1995	Asphyxiation	0.2471
Italy	2000	Asphyxiation	0.2471
Italy	2005	Asphyxiation	0.2471
Italy	2010	Asphyxiation	0.2471
Italy	2017	Asphyxiation	0.2471
Italy	1990	Crush injury	0.1325
Italy	1995	Crush injury	0.1325
Italy	2000	Crush injury	0.1325
Italy	2005	Crush injury	0.1325
Italy	2010	Crush injury	0.1325
Italy	2017	Crush injury	0.1325
Italy	1990	Nerve injury	0.1129
Italy	1995	Nerve injury	0.1129
Italy	2000	Nerve injury	0.1129
Italy	2005	Nerve injury	0.1129
Italy	2010	Nerve injury	0.1129
Italy	2017	Nerve injury	0.1129
Italy	1990	Injury to eyes	0.0165
Italy	1995	Injury to eyes	0.0165
Italy	2000	Injury to eyes	0.0165
Italy	2005	Injury to eyes	0.0165
Italy	2010	Injury to eyes	0.0165
Italy	2017	Injury to eyes	0.0165
Italy	1990	Amputation of lower limb, unilateral	0.039
Italy	1995	Amputation of lower limb, unilateral	0.039
Italy	2000	Amputation of lower limb, unilateral	0.039
Italy	2005	Amputation of lower limb, unilateral	0.039
Italy	2010	Amputation of lower limb, unilateral	0.039
Italy	2017	Amputation of lower limb, unilateral	0.039
Italy	1990	Open wound(s)	0.011
Italy	1995	Open wound(s)	0.011
Italy	2000	Open wound(s)	0.011
Italy	2005	Open wound(s)	0.011
Italy	2010	Open wound(s)	0.011
Italy	2017	Open wound(s)	0.011
Italy	1990	Poisoning requiring urgent care	0.1628
Italy	1995	Poisoning requiring urgent care	0.1628
Italy	2000	Poisoning requiring urgent care	0.1628
Italy	2005	Poisoning requiring urgent care	0.1628
Italy	2010	Poisoning requiring urgent care	0.1628
Italy	2017	Poisoning requiring urgent care	0.1628
Italy	1990	Severe chest injury	0.0474
Italy	1995	Severe chest injury	0.0474
Italy	2000	Severe chest injury	0.0474
Italy	2005	Severe chest injury	0.0474
Italy	2010	Severe chest injury	0.0474
Italy	2017	Severe chest injury	0.0474
Italy	1990	Internal hemorrhage in abdomen and pelvis	0.1143
Italy	1995	Internal hemorrhage in abdomen and pelvis	0.1143
Italy	2000	Internal hemorrhage in abdomen and pelvis	0.1143
Italy	2005	Internal hemorrhage in abdomen and pelvis	0.1143
Italy	2010	Internal hemorrhage in abdomen and pelvis	0.1143
Italy	2017	Internal hemorrhage in abdomen and pelvis	0.1143
Italy	1990	Contusion in any part of the body	0.0075
Italy	1995	Contusion in any part of the body	0.0075
Italy	2000	Contusion in any part of the body	0.0075
Italy	2005	Contusion in any part of the body	0.0075
Italy	2010	Contusion in any part of the body	0.0075
Italy	2017	Contusion in any part of the body	0.0075
Italy	1990	Effect of different environmental factors	0.049
Italy	1995	Effect of different environmental factors	0.049
Italy	2000	Effect of different environmental factors	0.049
Italy	2005	Effect of different environmental factors	0.049
Italy	2010	Effect of different environmental factors	0.049
Italy	2017	Effect of different environmental factors	0.049
Italy	1990	Complications following therapeutic procedures	0.0506
Italy	1995	Complications following therapeutic procedures	0.0506
Italy	2000	Complications following therapeutic procedures	0.0506
Italy	2005	Complications following therapeutic procedures	0.0506
Italy	2010	Complications following therapeutic procedures	0.0506
Italy	2017	Complications following therapeutic procedures	0.0506
Italy	1990	Multiple fractures, dislocations, crashes, wounds, sprains, and strains	0.0577
Italy	1995	Multiple fractures, dislocations, crashes, wounds, sprains, and strains	0.0577
Italy	2000	Multiple fractures, dislocations, crashes, wounds, sprains, and strains	0.0577
Italy	2005	Multiple fractures, dislocations, crashes, wounds, sprains, and strains	0.0577
Italy	2010	Multiple fractures, dislocations, crashes, wounds, sprains, and strains	0.0577
Italy	2017	Multiple fractures, dislocations, crashes, wounds, sprains, and strains	0.0577
Italy	1990	Amputation of upper limb, unilateral	0.039
Italy	1995	Amputation of upper limb, unilateral	0.039
Italy	2000	Amputation of upper limb, unilateral	0.039
Italy	2005	Amputation of upper limb, unilateral	0.039
Italy	2010	Amputation of upper limb, unilateral	0.039
Italy	2017	Amputation of upper limb, unilateral	0.039
Italy	1990	Amputation of thumb	0.0114
Italy	1995	Amputation of thumb	0.0114
Italy	2000	Amputation of thumb	0.0114
Italy	2005	Amputation of thumb	0.0114
Italy	2010	Amputation of thumb	0.0114
Italy	2017	Amputation of thumb	0.0114
Italy	1990	Amputation of toe/toes	0.0059
Italy	1995	Amputation of toe/toes	0.0059
Italy	2000	Amputation of toe/toes	0.0059
Italy	2005	Amputation of toe/toes	0.0059
Italy	2010	Amputation of toe/toes	0.0059
Italy	2017	Amputation of toe/toes	0.0059
Italy	1990	Burns, <20% total burned surface area without lower airway burns	0.0163
Italy	1995	Burns, <20% total burned surface area without lower airway burns	0.0163
Italy	2000	Burns, <20% total burned surface area without lower airway burns	0.0163
Italy	2005	Burns, <20% total burned surface area without lower airway burns	0.0163
Italy	2010	Burns, <20% total burned surface area without lower airway burns	0.0163
Italy	2017	Burns, <20% total burned surface area without lower airway burns	0.0163
Italy	1990	Burns, >=20% total burned surface area or >= 10% burned surface area if head/neck or hands/wrist involved w/o lower airway burns	0.1346
Italy	1995	Burns, >=20% total burned surface area or >= 10% burned surface area if head/neck or hands/wrist involved w/o lower airway burns	0.1346
Italy	2000	Burns, >=20% total burned surface area or >= 10% burned surface area if head/neck or hands/wrist involved w/o lower airway burns	0.1346
Italy	2005	Burns, >=20% total burned surface area or >= 10% burned surface area if head/neck or hands/wrist involved w/o lower airway burns	0.1346
Italy	2010	Burns, >=20% total burned surface area or >= 10% burned surface area if head/neck or hands/wrist involved w/o lower airway burns	0.1346
Italy	2017	Burns, >=20% total burned surface area or >= 10% burned surface area if head/neck or hands/wrist involved w/o lower airway burns	0.1346
Luxembourg	1990	Amputation of lower limbs, bilateral	0.1232
Luxembourg	1995	Amputation of lower limbs, bilateral	0.1232
Luxembourg	2000	Amputation of lower limbs, bilateral	0.1232
Luxembourg	2005	Amputation of lower limbs, bilateral	0.1232
Luxembourg	2010	Amputation of lower limbs, bilateral	0.1232
Luxembourg	2017	Amputation of lower limbs, bilateral	0.1232
Luxembourg	1990	Lower airway burns	0.2252
Luxembourg	1995	Lower airway burns	0.2252
Luxembourg	2000	Lower airway burns	0.2252
Luxembourg	2005	Lower airway burns	0.2252
Luxembourg	2010	Lower airway burns	0.2252
Luxembourg	2017	Lower airway burns	0.2252
Luxembourg	1990	Dislocation of hip	0.0159
Luxembourg	1995	Dislocation of hip	0.0159
Luxembourg	2000	Dislocation of hip	0.0159
Luxembourg	2005	Dislocation of hip	0.0159
Luxembourg	2010	Dislocation of hip	0.0159
Luxembourg	2017	Dislocation of hip	0.0159
Luxembourg	1990	Dislocation of knee	0.1134
Luxembourg	1995	Dislocation of knee	0.1134
Luxembourg	2000	Dislocation of knee	0.1134
Luxembourg	2005	Dislocation of knee	0.1134
Luxembourg	2010	Dislocation of knee	0.1134
Luxembourg	2017	Dislocation of knee	0.1134
Luxembourg	1990	Dislocation of shoulder	0.062
Luxembourg	1995	Dislocation of shoulder	0.062
Luxembourg	2000	Dislocation of shoulder	0.062
Luxembourg	2005	Dislocation of shoulder	0.062
Luxembourg	2010	Dislocation of shoulder	0.062
Luxembourg	2017	Dislocation of shoulder	0.062
Luxembourg	1990	Muscle and tendon injuries, including sprains and strains lesser dislocations	0.0075
Luxembourg	1995	Muscle and tendon injuries, including sprains and strains lesser dislocations	0.0075
Luxembourg	2000	Muscle and tendon injuries, including sprains and strains lesser dislocations	0.0075
Luxembourg	2005	Muscle and tendon injuries, including sprains and strains lesser dislocations	0.0075
Luxembourg	2010	Muscle and tendon injuries, including sprains and strains lesser dislocations	0.0075
Luxembourg	2017	Muscle and tendon injuries, including sprains and strains lesser dislocations	0.0075
Luxembourg	1990	Fracture of clavicle, scapula, or humerus	0.0349
Luxembourg	1995	Fracture of clavicle, scapula, or humerus	0.0349

Luxembourg	2000	Fracture of clavicle, scapula, or humerus	0.0349
Luxembourg	2005	Fracture of clavicle, scapula, or humerus	0.0349
Luxembourg	2010	Fracture of clavicle, scapula, or humerus	0.0349
Luxembourg	2017	Fracture of clavicle, scapula, or humerus	0.0349
Luxembourg	1990	Fracture of face bones	0.0669
Luxembourg	1995	Fracture of face bones	0.0669
Luxembourg	2000	Fracture of face bones	0.0669
Luxembourg	2005	Fracture of face bones	0.0669
Luxembourg	2010	Fracture of face bones	0.0669
Luxembourg	2017	Fracture of face bones	0.0669
Luxembourg	1990	Fracture of foot bones except ankle	0.0264
Luxembourg	1995	Fracture of foot bones except ankle	0.0264
Luxembourg	2000	Fracture of foot bones except ankle	0.0264
Luxembourg	2005	Fracture of foot bones except ankle	0.0264
Luxembourg	2010	Fracture of foot bones except ankle	0.0264
Luxembourg	2017	Fracture of foot bones except ankle	0.0264
Luxembourg	1990	Fracture of hand (wrist and other distal part of hand)	0.0138
Luxembourg	1995	Fracture of hand (wrist and other distal part of hand)	0.0138
Luxembourg	2000	Fracture of hand (wrist and other distal part of hand)	0.0138
Luxembourg	2005	Fracture of hand (wrist and other distal part of hand)	0.0138
Luxembourg	2010	Fracture of hand (wrist and other distal part of hand)	0.0138
Luxembourg	2017	Fracture of hand (wrist and other distal part of hand)	0.0138
Luxembourg	1990	Fracture of hip	0.0577
Luxembourg	1995	Fracture of hip	0.0577
Luxembourg	2000	Fracture of hip	0.0577
Luxembourg	2005	Fracture of hip	0.0577
Luxembourg	2010	Fracture of hip	0.0577
Luxembourg	2017	Fracture of hip	0.0577
Luxembourg	1990	Amputation of upper limbs, bilateral	0.0876
Luxembourg	1995	Amputation of upper limbs, bilateral	0.0876
Luxembourg	2000	Amputation of upper limbs, bilateral	0.0876
Luxembourg	2005	Amputation of upper limbs, bilateral	0.0876
Luxembourg	2010	Amputation of upper limbs, bilateral	0.0876
Luxembourg	2017	Amputation of upper limbs, bilateral	0.0876
Luxembourg	1990	Fracture of patella, tibia or fibula, or ankle	0.0554
Luxembourg	1995	Fracture of patella, tibia or fibula, or ankle	0.0554
Luxembourg	2000	Fracture of patella, tibia or fibula, or ankle	0.0554
Luxembourg	2005	Fracture of patella, tibia or fibula, or ankle	0.0554
Luxembourg	2010	Fracture of patella, tibia or fibula, or ankle	0.0554
Luxembourg	2017	Fracture of patella, tibia or fibula, or ankle	0.0554
Luxembourg	1990	Fracture of pelvis	0.1823
Luxembourg	1995	Fracture of pelvis	0.1823
Luxembourg	2000	Fracture of pelvis	0.1823
Luxembourg	2005	Fracture of pelvis	0.1823
Luxembourg	2010	Fracture of pelvis	0.1823
Luxembourg	2017	Fracture of pelvis	0.1823
Luxembourg	1990	Fracture of radius and/or ulna	0.0435
Luxembourg	1995	Fracture of radius and/or ulna	0.0435
Luxembourg	2000	Fracture of radius and/or ulna	0.0435
Luxembourg	2005	Fracture of radius and/or ulna	0.0435
Luxembourg	2010	Fracture of radius and/or ulna	0.0435
Luxembourg	2017	Fracture of radius and/or ulna	0.0435
Luxembourg	1990	Fracture of skull	0.0714
Luxembourg	1995	Fracture of skull	0.0714
Luxembourg	2000	Fracture of skull	0.0714
Luxembourg	2005	Fracture of skull	0.0714
Luxembourg	2010	Fracture of skull	0.0714
Luxembourg	2017	Fracture of skull	0.0714
Luxembourg	1990	Fracture of sternum and/or fracture of one or more ribs	0.1027
Luxembourg	1995	Fracture of sternum and/or fracture of one or more ribs	0.1027
Luxembourg	2000	Fracture of sternum and/or fracture of one or more ribs	0.1027
Luxembourg	2005	Fracture of sternum and/or fracture of one or more ribs	0.1027
Luxembourg	2010	Fracture of sternum and/or fracture of one or more ribs	0.1027
Luxembourg	2017	Fracture of sternum and/or fracture of one or more ribs	0.1027
Luxembourg	1990	Fracture of vertebral column	0.1106
Luxembourg	1995	Fracture of vertebral column	0.1106
Luxembourg	2000	Fracture of vertebral column	0.1106
Luxembourg	2005	Fracture of vertebral column	0.1106
Luxembourg	2010	Fracture of vertebral column	0.1106
Luxembourg	2017	Fracture of vertebral column	0.1106
Luxembourg	1990	Fracture of femur, other than femoral neck	0.042
Luxembourg	1995	Fracture of femur, other than femoral neck	0.042
Luxembourg	2000	Fracture of femur, other than femoral neck	0.042
Luxembourg	2005	Fracture of femur, other than femoral neck	0.042
Luxembourg	2010	Fracture of femur, other than femoral neck	0.042
Luxembourg	2017	Fracture of femur, other than femoral neck	0.042
Luxembourg	1990	Minor TBI	0.1319
Luxembourg	1995	Minor TBI	0.1319
Luxembourg	2000	Minor TBI	0.1319
Luxembourg	2005	Minor TBI	0.1319
Luxembourg	2010	Minor TBI	0.1319
Luxembourg	2017	Minor TBI	0.1319
Luxembourg	1990	Moderate TBI	0.1637
Luxembourg	1995	Moderate TBI	0.1637
Luxembourg	2000	Moderate TBI	0.1637
Luxembourg	2005	Moderate TBI	0.1637
Luxembourg	2010	Moderate TBI	0.1637
Luxembourg	2017	Moderate TBI	0.1637
Luxembourg	1990	Amputation of fingers (excluding thumb)	0.0047
Luxembourg	1995	Amputation of fingers (excluding thumb)	0.0047
Luxembourg	2000	Amputation of fingers (excluding thumb)	0.0047
Luxembourg	2005	Amputation of fingers (excluding thumb)	0.0047
Luxembourg	2010	Amputation of fingers (excluding thumb)	0.0047
Luxembourg	2017	Amputation of fingers (excluding thumb)	0.0047
Luxembourg	1990	Spinal cord lesion at neck level	0.4118
Luxembourg	1995	Spinal cord lesion at neck level	0.4118
Luxembourg	2000	Spinal cord lesion at neck level	0.4118
Luxembourg	2005	Spinal cord lesion at neck level	0.4118
Luxembourg	2010	Spinal cord lesion at neck level	0.4118
Luxembourg	2017	Spinal cord lesion at neck level	0.4118
Luxembourg	1990	Spinal cord lesion below neck level	0.1777
Luxembourg	1995	Spinal cord lesion below neck level	0.1777
Luxembourg	2000	Spinal cord lesion below neck level	0.1777
Luxembourg	2005	Spinal cord lesion below neck level	0.1777
Luxembourg	2010	Spinal cord lesion below neck level	0.1777
Luxembourg	2017	Spinal cord lesion below neck level	0.1777
Luxembourg	1990	Drowning and nonfatal submersion	0.2471
Luxembourg	1995	Drowning and nonfatal submersion	0.2471
Luxembourg	2000	Drowning and nonfatal submersion	0.2471
Luxembourg	2005	Drowning and nonfatal submersion	0.2471
Luxembourg	2010	Drowning and nonfatal submersion	0.2471
Luxembourg	2017	Drowning and nonfatal submersion	0.2471
Luxembourg	1990	Asphyxiation	0.2471
Luxembourg	1995	Asphyxiation	0.2471
Luxembourg	2000	Asphyxiation	0.2471
Luxembourg	2005	Asphyxiation	0.2471
Luxembourg	2010	Asphyxiation	0.2471
Luxembourg	2017	Asphyxiation	0.2471
Luxembourg	1990	Crush injury	0.1325
Luxembourg	1995	Crush injury	0.1325
Luxembourg	2000	Crush injury	0.1325
Luxembourg	2005	Crush injury	0.1325
Luxembourg	2010	Crush injury	0.1325
Luxembourg	2017	Crush injury	0.1325
Luxembourg	1990	Nerve injury	0.1129
Luxembourg	1995	Nerve injury	0.1129
Luxembourg	2000	Nerve injury	0.1129
Luxembourg	2005	Nerve injury	0.1129
Luxembourg	2010	Nerve injury	0.1129
Luxembourg	2017	Nerve injury	0.1129
Luxembourg	1990	Injury to eyes	0.0165
Luxembourg	1995	Injury to eyes	0.0165
Luxembourg	2000	Injury to eyes	0.0165
Luxembourg	2005	Injury to eyes	0.0165
Luxembourg	2010	Injury to eyes	0.0165
Luxembourg	2017	Injury to eyes	0.0165
Luxembourg	1990	Amputation of lower limb, unilateral	0.039
Luxembourg	1995	Amputation of lower limb, unilateral	0.039
Luxembourg	2000	Amputation of lower limb, unilateral	0.039
Luxembourg	2005	Amputation of lower limb, unilateral	0.039
Luxembourg	2010	Amputation of lower limb, unilateral	0.039
Luxembourg	2017	Amputation of lower limb, unilateral	0.039
Luxembourg	1990	Open wound(s)	0.011
Luxembourg	1995	Open wound(s)	0.011
Luxembourg	2000	Open wound(s)	0.011
Luxembourg	2005	Open wound(s)	0.011
Luxembourg	2010	Open wound(s)	0.011
Luxembourg	2017	Open wound(s)	0.011
Luxembourg	1990	Poisoning requiring urgent care	0.1628
Luxembourg	1995	Poisoning requiring urgent care	0.1628
Luxembourg	2000	Poisoning requiring urgent care	0.1628
Luxembourg	2005	Poisoning requiring urgent care	0.1628
Luxembourg	2010	Poisoning requiring urgent care	0.1628
Luxembourg	2017	Poisoning requiring urgent care	0.1628
Luxembourg	1990	Severe chest injury	0.0474
Luxembourg	1995	Severe chest injury	0.0474
Luxembourg	2000	Severe chest injury	0.0474
Luxembourg	2005	Severe chest injury	0.0474
Luxembourg	2010	Severe chest injury	0.0474
Luxembourg	2017	Severe chest injury	0.0474
Luxembourg	1990	Internal hemorrhage in abdomen and pelvis	0.1143
Luxembourg	1995	Internal hemorrhage in abdomen and pelvis	0.1143
Luxembourg	2000	Internal hemorrhage in abdomen and pelvis	0.1143
Luxembourg	2005	Internal hemorrhage in abdomen and pelvis	0.1143
Luxembourg	2010	Internal hemorrhage in abdomen and pelvis	0.1143
Luxembourg	2017	Internal hemorrhage in abdomen and pelvis	0.1143
Luxembourg	1990	Contusion in any part of the body	0.0075
Luxembourg	1995	Contusion in any part of the body	0.0075
Luxembourg	2000	Contusion in any part of the body	0.0075
Luxembourg	2005	Contusion in any part of the body	0.0075
Luxembourg	2010	Contusion in any part of the body	0.0075
Luxembourg	2017	Contusion in any part of the body	0.0075
Luxembourg	1990	Effect of different environmental factors	0.049
Luxembourg	1995	Effect of different environmental factors	0.049
Luxembourg	2000	Effect of different environmental factors	0.049
Luxembourg	2005	Effect of different environmental factors	0.049
Luxembourg	2010	Effect of different environmental factors	0.049
Luxembourg	2017	Effect of different environmental factors	0.049
Luxembourg	1990	Complications following therapeutic procedures	0.0506
Luxembourg	1995	Complications following therapeutic procedures	0.0506
Luxembourg	2000	Complications following therapeutic procedures	0.0506
Luxembourg	2005	Complications following therapeutic procedures	0.0506
Luxembourg	2010	Complications following therapeutic procedures	0.0506
Luxembourg	2017	Complications following therapeutic procedures	0.0506
Luxembourg	1990	Multiple fractures, dislocations, crashes, wounds, sprains, and strains	0.0577
Luxembourg	1995	Multiple fractures, dislocations, crashes, wounds, sprains, and strains	0.0577
Luxembourg	2000	Multiple fractures, dislocations, crashes, wounds, sprains, and strains	0.0577
Luxembourg	2005	Multiple fractures, dislocations, crashes, wounds, sprains, and strains	0.0577
Luxembourg	2010	Multiple fractures, dislocations, crashes, wounds, sprains, and strains	0.0577
Luxembourg	2017	Multiple fractures, dislocations, crashes, wounds, sprains, and strains	0.0577
Luxembourg	1990	Amputation of upper limb, unilateral	0.039
Luxembourg	1995	Amputation of upper limb, unilateral	0.039
Luxembourg	2000	Amputation of upper limb, unilateral	0.039
Luxembourg	2005	Amputation of upper limb, unilateral	0.039
Luxembourg	2010	Amputation of upper limb, unilateral	0.039
Luxembourg	2017	Amputation of upper limb, unilateral	0.039
Luxembourg	1990	Amputation of thumb	0.0114
Luxembourg	1995	Amputation of thumb	0.0114
Luxembourg	2000	Amputation of thumb	0.0114
Luxembourg	2005	Amputation of thumb	0.0114
Luxembourg	2010	Amputation of thumb	0.0114
Luxembourg	2017	Amputation of thumb	0.0114
Luxembourg	1990	Amputation of toe/toes	0.0059
Luxembourg	1995	Amputation of toe/toes	0.0059
Luxembourg	2000	Amputation of toe/toes	0.0059
Luxembourg	2005	Amputation of toe/toes	0.0059
Luxembourg	2010	Amputation of toe/toes	0.0059
Luxembourg	2017	Amputation of toe/toes	0.0059
Luxembourg	1990	Burns, <20% total burned surface area without lower airway burns	0.0163
Luxembourg	1995	Burns, <20% total burned surface area without lower airway burns	0.0163
Luxembourg	2000	Burns, <20% total burned surface area without lower airway burns	0.0163
Luxembourg	2005	Burns, <20% total burned surface area without lower airway burns	0.0163
Luxembourg	2010	Burns, <20% total burned surface area without lower airway burns	0.0163
Luxembourg	2017	Burns, <20% total burned surface area without lower airway burns	0.0163
Luxembourg	1990	Burns, >=20% total burned surface area or >= 10% burned surface area if head/neck or hands/wrist involved w/o lower airway burns	0.1346
Luxembourg	1995	Burns, >=20% total burned surface area or >= 10% burned surface area if head/neck or hands/wrist involved w/o lower airway burns	0.1346
Luxembourg	2000	Burns, >=20% total burned surface area or >= 10% burned surface area if head/neck or hands/wrist involved w/o lower airway burns	0.1346
Luxembourg	2005	Burns, >=20% total burned surface area or >= 10% burned surface area if head/neck or hands/wrist involved w/o lower airway burns	0.1346
Luxembourg	2010	Burns, >=20% total burned surface area or >= 10% burned surface area if head/neck or hands/wrist involved w/o lower airway burns	0.1346
Luxembourg	2017	Burns, >=20% total burned surface area or >= 10% burned surface area if head/neck or hands/wrist involved w/o lower airway burns	0.1346
Malta	1990	Amputation of lower limbs, bilateral	0.1237
Malta	1995	Amputation of lower limbs, bilateral	0.1232
Malta	2000	Amputation of lower limbs, bilateral	0.1232
Malta	2005	Amputation of lower limbs, bilateral	0.1232
Malta	2010	Amputation of lower limbs, bilateral	0.1232
Malta	2017	Amputation of lower limbs, bilateral	0.1232
Malta	1990	Lower airway burns	0.2252
Malta	1995	Lower airway burns	0.2252
Malta	2000	Lower airway burns	0.2252
Malta	2005	Lower airway burns	0.2252
Malta	2010	Lower airway burns	0.2252
Malta	2017	Lower airway burns	0.2252
Malta	1990	Dislocation of hip	0.0159
Malta	1995	Dislocation of hip	0.0159
Malta	2000	Dislocation of hip	0.0159
Malta	2005	Dislocation of hip	0.0159
Malta	2010	Dislocation of hip	0.0159
Malta	2017	Dislocation of hip	0.0159
Malta	1990	Dislocation of knee	0.1134
Malta	1995	Dislocation of knee	0.1134
Malta	2000	Dislocation of knee	0.1134
Malta	2005	Dislocation of knee	0.1134
Malta	2010	Dislocation of knee	0.1134
Malta	2017	Dislocation of knee	0.1134
Malta	1990	Dislocation of shoulder	0.062
Malta	1995	Dislocation of shoulder	0.062
Malta	2000	Dislocation of shoulder	0.062
Malta	2005	Dislocation of shoulder	0.062
Malta	2010	Dislocation of shoulder	0.062
Malta	2017	Dislocation of shoulder	0.062
Malta	1990	Muscle and tendon injuries, including sprains and strains lesser dislocations	0.0075
Malta	1995	Muscle and tendon injuries, including sprains and strains lesser dislocations	0.0075
Malta	2000	Muscle and tendon injuries, including sprains and strains lesser dislocations	0.0075
Malta	2005	Muscle and tendon injuries, including sprains and strains lesser dislocations	0.0075
Malta	2010	Muscle and tendon injuries, including sprains and strains lesser dislocations	0.0075
Malta	2017	Muscle and tendon injuries, including sprains and strains lesser dislocations	0.0075
Malta	1990	Fracture of clavicle, scapula, or humerus	0.0349
Malta	1995	Fracture of clavicle, scapula, or humerus	0.0349
Malta	2000	Fracture of clavicle, scapula, or humerus	0.0349
Malta	2005	Fracture of clavicle, scapula, or humerus	0.0349
Malta	2010	Fracture of clavicle, scapula, or humerus	0.0349
Malta	2017	Fracture of clavicle, scapula, or humerus	0.0349
Malta	1990	Fracture of face bones	0.0669
Malta	1995	Fracture of face bones	0.0669
Malta	2000	Fracture of face bones	0.0669
Malta	2005	Fracture of face bones	0.0669
Malta	2010	Fracture of face bones	0.0669
Malta	2017	Fracture of face bones	0.0669
Malta	1990	Fracture of foot bones except ankle	0.0264
Malta	1995	Fracture of foot bones except ankle	0.0264
Malta	2000	Fracture of foot bones except ankle	0.0264
Malta	2005	Fracture of foot bones except ankle	0.0264
Malta	2010	Fracture of foot bones except ankle	0.0264
Malta	2017	Fracture of foot bones except ankle	0.0264
Malta	1990	Fracture of hand (wrist and other distal part of hand)	0.0138
Malta	1995	Fracture of hand (wrist and other distal part of hand)	0.0138
Malta	2000	Fracture of hand (wrist and other distal part of hand)	0.0138
Malta	2005	Fracture of hand (wrist and other distal part of hand)	0.0138
Malta	2010	Fracture of hand (wrist and other distal part of hand)	0.0138
Malta	2017	Fracture of hand (wrist and other distal part of hand)	0.0138
Malta	1990	Fracture of hip	0.0583
Malta	1995	Fracture of hip	0.0577
Malta	2000	Fracture of hip	0.0577
Malta	2005	Fracture of hip	0.0577
Malta	2010	Fracture of hip	0.0577
Malta	2017	Fracture of hip	0.0577
Malta	1990	Amputation of upper limbs, bilateral	0.0882
Malta	1995	Amputation of upper limbs, bilateral	0.0876
Malta	2000	Amputation of upper limbs, bilateral	0.0876
Malta	2005	Amputation of upper limbs, bilateral	0.0876
Malta	2010	Amputation of upper limbs, bilateral	0.0876
Malta	2017	Amputation of upper limbs, bilateral	0.0876
Malta	1990	Fracture of patella, tibia or fibula, or ankle	0.0554
Malta	1995	Fracture of patella, tibia or fibula, or ankle	0.0554
Malta	2000	Fracture of patella, tibia or fibula, or ankle	0.0554
Malta	2005	Fracture of patella, tibia or fibula, or ankle	0.0554
Malta	2010	Fracture of patella, tibia or fibula, or ankle	0.0554
Malta	2017	Fracture of patella, tibia or fibula, or ankle	0.0554
Malta	1990	Fracture of pelvis	0.1823
Malta	1995	Fracture of pelvis	0.1823
Malta	2000	Fracture of pelvis	0.1823
Malta	2005	Fracture of pelvis	0.1823
Malta	2010	Fracture of pelvis	0.1823
Malta	2017	Fracture of pelvis	0.1823
Malta	1990	Fracture of radius and/or ulna	0.0435
Malta	1995	Fracture of radius and/or ulna	0.0435
Malta	2000	Fracture of radius and/or ulna	0.0435
Malta	2005	Fracture of radius and/or ulna	0.0435
Malta	2010	Fracture of radius and/or ulna	0.0435
Malta	2017	Fracture of radius and/or ulna	0.0435
Malta	1990	Fracture of skull	0.0714
Malta	1995	Fracture of skull	0.0714
Malta	2000	Fracture of skull	0.0714
Malta	2005	Fracture of skull	0.0714
Malta	2010	Fracture of skull	0.0714
Malta	2017	Fracture of skull	0.0714
Malta	1990	Fracture of sternum and/or fracture of one or more ribs	0.1027
Malta	1995	Fracture of sternum and/or fracture of one or more ribs	0.1027
Malta	2000	Fracture of sternum and/or fracture of one or more ribs	0.1027
Malta	2005	Fracture of sternum and/or fracture of one or more ribs	0.1027
Malta	2010	Fracture of sternum and/or fracture of one or more ribs	0.1027
Malta	2017	Fracture of sternum and/or fracture of one or more ribs	0.1027
Malta	1990	Fracture of vertebral column	0.1106
Malta	1995	Fracture of vertebral column	0.1106
Malta	2000	Fracture of vertebral column	0.1106
Malta	2005	Fracture of vertebral column	0.1106
Malta	2010	Fracture of vertebral column	0.1106
Malta	2017	Fracture of vertebral column	0.1106
Malta	1990	Fracture of femur, other than femoral neck	0.042
Malta	1995	Fracture of femur, other than femoral neck	0.042
Malta	2000	Fracture of femur, other than femoral neck	0.042
Malta	2005	Fracture of femur, other than femoral neck	0.042
Malta	2010	Fracture of femur, other than femoral neck	0.042
Malta	2017	Fracture of femur, other than femoral neck	0.042
Malta	1990	Minor TBI	0.1319
Malta	1995	Minor TBI	0.1319
Malta	2000	Minor TBI	0.1319
Malta	2005	Minor TBI	0.1319
Malta	2010	Minor TBI	0.1319
Malta	2017	Minor TBI	0.1319
Malta	1990	Moderate TBI	0.1637
Malta	1995	Moderate TBI	0.1637
Malta	2000	Moderate TBI	0.1637
Malta	2005	Moderate TBI	0.1637
Malta	2010	Moderate TBI	0.1637
Malta	2017	Moderate TBI	0.1637
Malta	1990	Amputation of fingers (excluding thumb)	0.0047
Malta	1995	Amputation of fingers (excluding thumb)	0.0047
Malta	2000	Amputation of fingers (excluding thumb)	0.0047
Malta	2005	Amputation of fingers (excluding thumb)	0.0047
Malta	2010	Amputation of fingers (excluding thumb)	0.0047
Malta	2017	Amputation of fingers (excluding thumb)	0.0047
Malta	1990	Spinal cord lesion at neck level	0.412
Malta	1995	Spinal cord lesion at neck level	0.4118
Malta	2000	Spinal cord lesion at neck level	0.4118
Malta	2005	Spinal cord lesion at neck level	0.4118
Malta	2010	Spinal cord lesion at neck level	0.4118
Malta	2017	Spinal cord lesion at neck level	0.4118
Malta	1990	Spinal cord lesion below neck level	0.1781
Malta	1995	Spinal cord lesion below neck level	0.1777
Malta	2000	Spinal cord lesion below neck level	0.1777
Malta	2005	Spinal cord lesion below neck level	0.1777
Malta	2010	Spinal cord lesion below neck level	0.1777
Malta	2017	Spinal cord lesion below neck level	0.1777
Malta	1990	Drowning and nonfatal submersion	0.2471
Malta	1995	Drowning and nonfatal submersion	0.2471
Malta	2000	Drowning and nonfatal submersion	0.2471
Malta	2005	Drowning and nonfatal submersion	0.2471
Malta	2010	Drowning and nonfatal submersion	0.2471
Malta	2017	Drowning and nonfatal submersion	0.2471
Malta	1990	Asphyxiation	0.2471
Malta	1995	Asphyxiation	0.2471
Malta	2000	Asphyxiation	0.2471
Malta	2005	Asphyxiation	0.2471
Malta	2010	Asphyxiation	0.2471
Malta	2017	Asphyxiation	0.2471
Malta	1990	Crush injury	0.1325
Malta	1995	Crush injury	0.1325
Malta	2000	Crush injury	0.1325
Malta	2005	Crush injury	0.1325
Malta	2010	Crush injury	0.1325
Malta	2017	Crush injury	0.1325
Malta	1990	Nerve injury	0.1129
Malta	1995	Nerve injury	0.1129
Malta	2000	Nerve injury	0.1129
Malta	2005	Nerve injury	0.1129
Malta	2010	Nerve injury	0.1129
Malta	2017	Nerve injury	0.1129
Malta	1990	Injury to eyes	0.0165
Malta	1995	Injury to eyes	0.0165
Malta	2000	Injury to eyes	0.0165
Malta	2005	Injury to eyes	0.0165
Malta	2010	Injury to eyes	0.0165
Malta	2017	Injury to eyes	0.0165
Malta	1990	Amputation of lower limb, unilateral	0.0392
Malta	1995	Amputation of lower limb, unilateral	0.039
Malta	2000	Amputation of lower limb, unilateral	0.039
Malta	2005	Amputation of lower limb, unilateral	0.039
Malta	2010	Amputation of lower limb, unilateral	0.039
Malta	2017	Amputation of lower limb, unilateral	0.039
Malta	1990	Open wound(s)	0.011
Malta	1995	Open wound(s)	0.011
Malta	2000	Open wound(s)	0.011
Malta	2005	Open wound(s)	0.011
Malta	2010	Open wound(s)	0.011
Malta	2017	Open wound(s)	0.011
Malta	1990	Poisoning requiring urgent care	0.1628
Malta	1995	Poisoning requiring urgent care	0.1628
Malta	2000	Poisoning requiring urgent care	0.1628
Malta	2005	Poisoning requiring urgent care	0.1628
Malta	2010	Poisoning requiring urgent care	0.1628
Malta	2017	Poisoning requiring urgent care	0.1628
Malta	1990	Severe chest injury	0.0474
Malta	1995	Severe chest injury	0.0474
Malta	2000	Severe chest injury	0.0474
Malta	2005	Severe chest injury	0.0474
Malta	2010	Severe chest injury	0.0474
Malta	2017	Severe chest injury	0.0474
Malta	1990	Internal hemorrhage in abdomen and pelvis	0.1143
Malta	1995	Internal hemorrhage in abdomen and pelvis	0.1143
Malta	2000	Internal hemorrhage in abdomen and pelvis	0.1143
Malta	2005	Internal hemorrhage in abdomen and pelvis	0.1143
Malta	2010	Internal hemorrhage in abdomen and pelvis	0.1143
Malta	2017	Internal hemorrhage in abdomen and pelvis	0.1143
Malta	1990	Contusion in any part of the body	0.0075
Malta	1995	Contusion in any part of the body	0.0075
Malta	2000	Contusion in any part of the body	0.0075
Malta	2005	Contusion in any part of the body	0.0075
Malta	2010	Contusion in any part of the body	0.0075
Malta	2017	Contusion in any part of the body	0.0075
Malta	1990	Effect of different environmental factors	0.049

Malta	1995	Effect of different environmental factors	0.049
Malta	2000	Effect of different environmental factors	0.049
Malta	2005	Effect of different environmental factors	0.049
Malta	2010	Effect of different environmental factors	0.049
Malta	2017	Effect of different environmental factors	0.049
Malta	1990	Complications following therapeutic procedures	0.0506
Malta	1995	Complications following therapeutic procedures	0.0506
Malta	2000	Complications following therapeutic procedures	0.0506
Malta	2005	Complications following therapeutic procedures	0.0506
Malta	2010	Complications following therapeutic procedures	0.0506
Malta	2017	Complications following therapeutic procedures	0.0506
Malta	1990	Multiple fractures, dislocations, crashes, wounds, sprains, and strains	0.0583
Malta	1995	Multiple fractures, dislocations, crashes, wounds, sprains, and strains	0.0577
Malta	2000	Multiple fractures, dislocations, crashes, wounds, sprains, and strains	0.0577
Malta	2005	Multiple fractures, dislocations, crashes, wounds, sprains, and strains	0.0577
Malta	2010	Multiple fractures, dislocations, crashes, wounds, sprains, and strains	0.0577
Malta	2017	Multiple fractures, dislocations, crashes, wounds, sprains, and strains	0.0577
Malta	1990	Amputation of upper limb, unilateral	0.0391
Malta	1995	Amputation of upper limb, unilateral	0.039
Malta	2000	Amputation of upper limb, unilateral	0.039
Malta	2005	Amputation of upper limb, unilateral	0.039
Malta	2010	Amputation of upper limb, unilateral	0.039
Malta	2017	Amputation of upper limb, unilateral	0.039
Malta	1990	Amputation of thumb	0.0114
Malta	1995	Amputation of thumb	0.0114
Malta	2000	Amputation of thumb	0.0114
Malta	2005	Amputation of thumb	0.0114
Malta	2010	Amputation of thumb	0.0114
Malta	2017	Amputation of thumb	0.0114
Malta	1990	Amputation of toe/toes	0.0059
Malta	1995	Amputation of toe/toes	0.0059
Malta	2000	Amputation of toe/toes	0.0059
Malta	2005	Amputation of toe/toes	0.0059
Malta	2010	Amputation of toe/toes	0.0059
Malta	2017	Amputation of toe/toes	0.0059
Malta	1990	Burns, <20% total burned surface area without lower airway burns	0.0163
Malta	1995	Burns, <20% total burned surface area without lower airway burns	0.0163
Malta	2000	Burns, <20% total burned surface area without lower airway burns	0.0163
Malta	2005	Burns, <20% total burned surface area without lower airway burns	0.0163
Malta	2010	Burns, <20% total burned surface area without lower airway burns	0.0163
Malta	2017	Burns, <20% total burned surface area without lower airway burns	0.0163
Malta	1990	Burns, >=20% total burned surface area or >= 10% burned surface area if head/neck or hands/wrist involved w/o lower airway burns	0.1351
Malta	1995	Burns, >=20% total burned surface area or >= 10% burned surface area if head/neck or hands/wrist involved w/o lower airway burns	0.1346
Malta	2000	Burns, >=20% total burned surface area or >= 10% burned surface area if head/neck or hands/wrist involved w/o lower airway burns	0.1346
Malta	2005	Burns, >=20% total burned surface area or >= 10% burned surface area if head/neck or hands/wrist involved w/o lower airway burns	0.1346
Malta	2010	Burns, >=20% total burned surface area or >= 10% burned surface area if head/neck or hands/wrist involved w/o lower airway burns	0.1346
Malta	2017	Burns, >=20% total burned surface area or >= 10% burned surface area if head/neck or hands/wrist involved w/o lower airway burns	0.1346
Netherlands	1990	Amputation of lower limbs, bilateral	0.1232
Netherlands	1995	Amputation of lower limbs, bilateral	0.1232
Netherlands	2000	Amputation of lower limbs, bilateral	0.1232
Netherlands	2005	Amputation of lower limbs, bilateral	0.1232
Netherlands	2010	Amputation of lower limbs, bilateral	0.1232
Netherlands	2017	Amputation of lower limbs, bilateral	0.1232
Netherlands	1990	Lower airway burns	0.2252
Netherlands	1995	Lower airway burns	0.2252
Netherlands	2000	Lower airway burns	0.2252
Netherlands	2005	Lower airway burns	0.2252
Netherlands	2010	Lower airway burns	0.2252
Netherlands	2017	Lower airway burns	0.2252
Netherlands	1990	Dislocation of hip	0.0159
Netherlands	1995	Dislocation of hip	0.0159
Netherlands	2000	Dislocation of hip	0.0159
Netherlands	2005	Dislocation of hip	0.0159
Netherlands	2010	Dislocation of hip	0.0159
Netherlands	2017	Dislocation of hip	0.0159
Netherlands	1990	Dislocation of knee	0.1134
Netherlands	1995	Dislocation of knee	0.1134
Netherlands	2000	Dislocation of knee	0.1134
Netherlands	2005	Dislocation of knee	0.1134
Netherlands	2010	Dislocation of knee	0.1134
Netherlands	2017	Dislocation of knee	0.1134
Netherlands	1990	Dislocation of shoulder	0.062
Netherlands	1995	Dislocation of shoulder	0.062
Netherlands	2000	Dislocation of shoulder	0.062
Netherlands	2005	Dislocation of shoulder	0.062
Netherlands	2010	Dislocation of shoulder	0.062
Netherlands	2017	Dislocation of shoulder	0.062
Netherlands	1990	Muscle and tendon injuries, including sprains and strains lesser dislocations	0.0075
Netherlands	1995	Muscle and tendon injuries, including sprains and strains lesser dislocations	0.0075
Netherlands	2000	Muscle and tendon injuries, including sprains and strains lesser dislocations	0.0075
Netherlands	2005	Muscle and tendon injuries, including sprains and strains lesser dislocations	0.0075
Netherlands	2010	Muscle and tendon injuries, including sprains and strains lesser dislocations	0.0075
Netherlands	2017	Muscle and tendon injuries, including sprains and strains lesser dislocations	0.0075
Netherlands	1990	Fracture of clavicle, scapula, or humerus	0.0349
Netherlands	1995	Fracture of clavicle, scapula, or humerus	0.0349
Netherlands	2000	Fracture of clavicle, scapula, or humerus	0.0349
Netherlands	2005	Fracture of clavicle, scapula, or humerus	0.0349
Netherlands	2010	Fracture of clavicle, scapula, or humerus	0.0349
Netherlands	2017	Fracture of clavicle, scapula, or humerus	0.0349
Netherlands	1990	Fracture of face bones	0.0669
Netherlands	1995	Fracture of face bones	0.0669
Netherlands	2000	Fracture of face bones	0.0669
Netherlands	2005	Fracture of face bones	0.0669
Netherlands	2010	Fracture of face bones	0.0669
Netherlands	2017	Fracture of face bones	0.0669
Netherlands	1990	Fracture of foot bones except ankle	0.0264
Netherlands	1995	Fracture of foot bones except ankle	0.0264
Netherlands	2000	Fracture of foot bones except ankle	0.0264
Netherlands	2005	Fracture of foot bones except ankle	0.0264
Netherlands	2010	Fracture of foot bones except ankle	0.0264
Netherlands	2017	Fracture of foot bones except ankle	0.0264
Netherlands	1990	Fracture of hand (wrist and other distal part of hand)	0.0138
Netherlands	1995	Fracture of hand (wrist and other distal part of hand)	0.0138
Netherlands	2000	Fracture of hand (wrist and other distal part of hand)	0.0138
Netherlands	2005	Fracture of hand (wrist and other distal part of hand)	0.0138
Netherlands	2010	Fracture of hand (wrist and other distal part of hand)	0.0138
Netherlands	2017	Fracture of hand (wrist and other distal part of hand)	0.0138
Netherlands	1990	Fracture of hip	0.0577
Netherlands	1995	Fracture of hip	0.0577
Netherlands	2000	Fracture of hip	0.0577
Netherlands	2005	Fracture of hip	0.0577
Netherlands	2010	Fracture of hip	0.0577
Netherlands	2017	Fracture of hip	0.0577
Netherlands	1990	Amputation of upper limbs, bilateral	0.0876
Netherlands	1995	Amputation of upper limbs, bilateral	0.0876
Netherlands	2000	Amputation of upper limbs, bilateral	0.0876
Netherlands	2005	Amputation of upper limbs, bilateral	0.0876
Netherlands	2010	Amputation of upper limbs, bilateral	0.0876
Netherlands	2017	Amputation of upper limbs, bilateral	0.0876
Netherlands	1990	Fracture of patella, tibia or fibula, or ankle	0.0554
Netherlands	1995	Fracture of patella, tibia or fibula, or ankle	0.0554
Netherlands	2000	Fracture of patella, tibia or fibula, or ankle	0.0554
Netherlands	2005	Fracture of patella, tibia or fibula, or ankle	0.0554
Netherlands	2010	Fracture of patella, tibia or fibula, or ankle	0.0554
Netherlands	2017	Fracture of patella, tibia or fibula, or ankle	0.0554
Netherlands	1990	Fracture of pelvis	0.1823
Netherlands	1995	Fracture of pelvis	0.1823
Netherlands	2000	Fracture of pelvis	0.1823
Netherlands	2005	Fracture of pelvis	0.1823
Netherlands	2010	Fracture of pelvis	0.1823
Netherlands	2017	Fracture of pelvis	0.1823
Netherlands	1990	Fracture of radius and/or ulna	0.0435
Netherlands	1995	Fracture of radius and/or ulna	0.0435
Netherlands	2000	Fracture of radius and/or ulna	0.0435
Netherlands	2005	Fracture of radius and/or ulna	0.0435
Netherlands	2010	Fracture of radius and/or ulna	0.0435
Netherlands	2017	Fracture of radius and/or ulna	0.0435
Netherlands	1990	Fracture of skull	0.0714
Netherlands	1995	Fracture of skull	0.0714
Netherlands	2000	Fracture of skull	0.0714
Netherlands	2005	Fracture of skull	0.0714
Netherlands	2010	Fracture of skull	0.0714
Netherlands	2017	Fracture of skull	0.0714
Netherlands	1990	Fracture of sternum and/or fracture of one or more ribs	0.1027
Netherlands	1995	Fracture of sternum and/or fracture of one or more ribs	0.1027
Netherlands	2000	Fracture of sternum and/or fracture of one or more ribs	0.1027
Netherlands	2005	Fracture of sternum and/or fracture of one or more ribs	0.1027
Netherlands	2010	Fracture of sternum and/or fracture of one or more ribs	0.1027
Netherlands	2017	Fracture of sternum and/or fracture of one or more ribs	0.1027
Netherlands	1990	Fracture of vertebral column	0.1106
Netherlands	1995	Fracture of vertebral column	0.1106
Netherlands	2000	Fracture of vertebral column	0.1106
Netherlands	2005	Fracture of vertebral column	0.1106
Netherlands	2010	Fracture of vertebral column	0.1106
Netherlands	2017	Fracture of vertebral column	0.1106
Netherlands	1990	Fracture of femur, other than femoral neck	0.042
Netherlands	1995	Fracture of femur, other than femoral neck	0.042
Netherlands	2000	Fracture of femur, other than femoral neck	0.042
Netherlands	2005	Fracture of femur, other than femoral neck	0.042
Netherlands	2010	Fracture of femur, other than femoral neck	0.042
Netherlands	2017	Fracture of femur, other than femoral neck	0.042
Netherlands	1990	Minor TBI	0.1319
Netherlands	1995	Minor TBI	0.1319
Netherlands	2000	Minor TBI	0.1319
Netherlands	2005	Minor TBI	0.1319
Netherlands	2010	Minor TBI	0.1319
Netherlands	2017	Minor TBI	0.1319
Netherlands	1990	Moderate TBI	0.1637
Netherlands	1995	Moderate TBI	0.1637
Netherlands	2000	Moderate TBI	0.1637
Netherlands	2005	Moderate TBI	0.1637
Netherlands	2010	Moderate TBI	0.1637
Netherlands	2017	Moderate TBI	0.1637
Netherlands	1990	Amputation of fingers (excluding thumb)	0.0047
Netherlands	1995	Amputation of fingers (excluding thumb)	0.0047
Netherlands	2000	Amputation of fingers (excluding thumb)	0.0047
Netherlands	2005	Amputation of fingers (excluding thumb)	0.0047
Netherlands	2010	Amputation of fingers (excluding thumb)	0.0047
Netherlands	2017	Amputation of fingers (excluding thumb)	0.0047
Netherlands	1990	Spinal cord lesion at neck level	0.4118
Netherlands	1995	Spinal cord lesion at neck level	0.4118
Netherlands	2000	Spinal cord lesion at neck level	0.4118
Netherlands	2005	Spinal cord lesion at neck level	0.4118
Netherlands	2010	Spinal cord lesion at neck level	0.4118
Netherlands	2017	Spinal cord lesion at neck level	0.4118
Netherlands	1990	Spinal cord lesion below neck level	0.1777
Netherlands	1995	Spinal cord lesion below neck level	0.1777
Netherlands	2000	Spinal cord lesion below neck level	0.1777
Netherlands	2005	Spinal cord lesion below neck level	0.1777
Netherlands	2010	Spinal cord lesion below neck level	0.1777
Netherlands	2017	Spinal cord lesion below neck level	0.1777
Netherlands	1990	Drowning and nonfatal submersion	0.2471
Netherlands	1995	Drowning and nonfatal submersion	0.2471
Netherlands	2000	Drowning and nonfatal submersion	0.2471
Netherlands	2005	Drowning and nonfatal submersion	0.2471
Netherlands	2010	Drowning and nonfatal submersion	0.2471
Netherlands	2017	Drowning and nonfatal submersion	0.2471
Netherlands	1990	Asphyxiation	0.2471
Netherlands	1995	Asphyxiation	0.2471
Netherlands	2000	Asphyxiation	0.2471
Netherlands	2005	Asphyxiation	0.2471
Netherlands	2010	Asphyxiation	0.2471
Netherlands	2017	Asphyxiation	0.2471
Netherlands	1990	Crush injury	0.1325
Netherlands	1995	Crush injury	0.1325
Netherlands	2000	Crush injury	0.1325
Netherlands	2005	Crush injury	0.1325
Netherlands	2010	Crush injury	0.1325
Netherlands	2017	Crush injury	0.1325
Netherlands	1990	Nerve injury	0.1129
Netherlands	1995	Nerve injury	0.1129
Netherlands	2000	Nerve injury	0.1129
Netherlands	2005	Nerve injury	0.1129
Netherlands	2010	Nerve injury	0.1129
Netherlands	2017	Nerve injury	0.1129
Netherlands	1990	Injury to eyes	0.0165
Netherlands	1995	Injury to eyes	0.0165
Netherlands	2000	Injury to eyes	0.0165
Netherlands	2005	Injury to eyes	0.0165
Netherlands	2010	Injury to eyes	0.0165
Netherlands	2017	Injury to eyes	0.0165
Netherlands	1990	Amputation of lower limb, unilateral	0.039
Netherlands	1995	Amputation of lower limb, unilateral	0.039
Netherlands	2000	Amputation of lower limb, unilateral	0.039
Netherlands	2005	Amputation of lower limb, unilateral	0.039
Netherlands	2010	Amputation of lower limb, unilateral	0.039
Netherlands	2017	Amputation of lower limb, unilateral	0.039
Netherlands	1990	Open wound(s)	0.011
Netherlands	1995	Open wound(s)	0.011
Netherlands	2000	Open wound(s)	0.011
Netherlands	2005	Open wound(s)	0.011
Netherlands	2010	Open wound(s)	0.011
Netherlands	2017	Open wound(s)	0.011
Netherlands	1990	Poisoning requiring urgent care	0.1628
Netherlands	1995	Poisoning requiring urgent care	0.1628
Netherlands	2000	Poisoning requiring urgent care	0.1628
Netherlands	2005	Poisoning requiring urgent care	0.1628
Netherlands	2010	Poisoning requiring urgent care	0.1628
Netherlands	2017	Poisoning requiring urgent care	0.1628
Netherlands	1990	Severe chest injury	0.0474
Netherlands	1995	Severe chest injury	0.0474
Netherlands	2000	Severe chest injury	0.0474
Netherlands	2005	Severe chest injury	0.0474
Netherlands	2010	Severe chest injury	0.0474
Netherlands	2017	Severe chest injury	0.0474
Netherlands	1990	Internal hemorrhage in abdomen and pelvis	0.1143
Netherlands	1995	Internal hemorrhage in abdomen and pelvis	0.1143
Netherlands	2000	Internal hemorrhage in abdomen and pelvis	0.1143
Netherlands	2005	Internal hemorrhage in abdomen and pelvis	0.1143
Netherlands	2010	Internal hemorrhage in abdomen and pelvis	0.1143
Netherlands	2017	Internal hemorrhage in abdomen and pelvis	0.1143
Netherlands	1990	Contusion in any part of the body	0.0075
Netherlands	1995	Contusion in any part of the body	0.0075
Netherlands	2000	Contusion in any part of the body	0.0075
Netherlands	2005	Contusion in any part of the body	0.0075
Netherlands	2010	Contusion in any part of the body	0.0075
Netherlands	2017	Contusion in any part of the body	0.0075
Netherlands	1990	Effect of different environmental factors	0.049
Netherlands	1995	Effect of different environmental factors	0.049
Netherlands	2000	Effect of different environmental factors	0.049
Netherlands	2005	Effect of different environmental factors	0.049
Netherlands	2010	Effect of different environmental factors	0.049
Netherlands	2017	Effect of different environmental factors	0.049
Netherlands	1990	Complications following therapeutic procedures	0.0506
Netherlands	1995	Complications following therapeutic procedures	0.0506
Netherlands	2000	Complications following therapeutic procedures	0.0506
Netherlands	2005	Complications following therapeutic procedures	0.0506
Netherlands	2010	Complications following therapeutic procedures	0.0506
Netherlands	2017	Complications following therapeutic procedures	0.0506
Netherlands	1990	Multiple fractures, dislocations, crashes, wounds, sprains, and strains	0.0577
Netherlands	1995	Multiple fractures, dislocations, crashes, wounds, sprains, and strains	0.0577
Netherlands	2000	Multiple fractures, dislocations, crashes, wounds, sprains, and strains	0.0577
Netherlands	2005	Multiple fractures, dislocations, crashes, wounds, sprains, and strains	0.0577
Netherlands	2010	Multiple fractures, dislocations, crashes, wounds, sprains, and strains	0.0577
Netherlands	2017	Multiple fractures, dislocations, crashes, wounds, sprains, and strains	0.0577
Netherlands	1990	Amputation of upper limb, unilateral	0.039
Netherlands	1995	Amputation of upper limb, unilateral	0.039
Netherlands	2000	Amputation of upper limb, unilateral	0.039
Netherlands	2005	Amputation of upper limb, unilateral	0.039
Netherlands	2010	Amputation of upper limb, unilateral	0.039
Netherlands	2017	Amputation of upper limb, unilateral	0.039
Netherlands	1990	Amputation of thumb	0.0114
Netherlands	1995	Amputation of thumb	0.0114
Netherlands	2000	Amputation of thumb	0.0114
Netherlands	2005	Amputation of thumb	0.0114
Netherlands	2010	Amputation of thumb	0.0114
Netherlands	2017	Amputation of thumb	0.0114
Netherlands	1990	Amputation of toe/toes	0.0059
Netherlands	1995	Amputation of toe/toes	0.0059
Netherlands	2000	Amputation of toe/toes	0.0059
Netherlands	2005	Amputation of toe/toes	0.0059
Netherlands	2010	Amputation of toe/toes	0.0059
Netherlands	2017	Amputation of toe/toes	0.0059
Netherlands	1990	Burns, <20% total burned surface area without lower airway burns	0.0163
Netherlands	1995	Burns, <20% total burned surface area without lower airway burns	0.0163
Netherlands	2000	Burns, <20% total burned surface area without lower airway burns	0.0163
Netherlands	2005	Burns, <20% total burned surface area without lower airway burns	0.0163
Netherlands	2010	Burns, <20% total burned surface area without lower airway burns	0.0163
Netherlands	2017	Burns, <20% total burned surface area without lower airway burns	0.0163
Netherlands	1990	Burns, >=20% total burned surface area or >= 10% burned surface area if head/neck or hands/wrist involved w/o lower airway burns	0.1346
Netherlands	1995	Burns, >=20% total burned surface area or >= 10% burned surface area if head/neck or hands/wrist involved w/o lower airway burns	0.1346
Netherlands	2000	Burns, >=20% total burned surface area or >= 10% burned surface area if head/neck or hands/wrist involved w/o lower airway burns	0.1346
Netherlands	2005	Burns, >=20% total burned surface area or >= 10% burned surface area if head/neck or hands/wrist involved w/o lower airway burns	0.1346
Netherlands	2010	Burns, >=20% total burned surface area or >= 10% burned surface area if head/neck or hands/wrist involved w/o lower airway burns	0.1346
Netherlands	2017	Burns, >=20% total burned surface area or >= 10% burned surface area if head/neck or hands/wrist involved w/o lower airway burns	0.1346
Portugal	1990	Amputation of lower limbs, bilateral	0.15
Portugal	1995	Amputation of lower limbs, bilateral	0.1327
Portugal	2000	Amputation of lower limbs, bilateral	0.1232
Portugal	2005	Amputation of lower limbs, bilateral	0.1232
Portugal	2010	Amputation of lower limbs, bilateral	0.1232
Portugal	2017	Amputation of lower limbs, bilateral	0.1232
Portugal	1990	Lower airway burns	0.2252
Portugal	1995	Lower airway burns	0.2252
Portugal	2000	Lower airway burns	0.2252
Portugal	2005	Lower airway burns	0.2252
Portugal	2010	Lower airway burns	0.2252
Portugal	2017	Lower airway burns	0.2252
Portugal	1990	Dislocation of hip	0.0159
Portugal	1995	Dislocation of hip	0.0159
Portugal	2000	Dislocation of hip	0.0159
Portugal	2005	Dislocation of hip	0.0159
Portugal	2010	Dislocation of hip	0.0159
Portugal	2017	Dislocation of hip	0.0159
Portugal	1990	Dislocation of knee	0.1134
Portugal	1995	Dislocation of knee	0.1134
Portugal	2000	Dislocation of knee	0.1134
Portugal	2005	Dislocation of knee	0.1134
Portugal	2010	Dislocation of knee	0.1134
Portugal	2017	Dislocation of knee	0.1134
Portugal	1990	Dislocation of shoulder	0.062
Portugal	1995	Dislocation of shoulder	0.062
Portugal	2000	Dislocation of shoulder	0.062
Portugal	2005	Dislocation of shoulder	0.062
Portugal	2010	Dislocation of shoulder	0.062
Portugal	2017	Dislocation of shoulder	0.062
Portugal	1990	Muscle and tendon injuries, including sprains and strains lesser dislocations	0.0075
Portugal	1995	Muscle and tendon injuries, including sprains and strains lesser dislocations	0.0075
Portugal	2000	Muscle and tendon injuries, including sprains and strains lesser dislocations	0.0075
Portugal	2005	Muscle and tendon injuries, including sprains and strains lesser dislocations	0.0075
Portugal	2010	Muscle and tendon injuries, including sprains and strains lesser dislocations	0.0075
Portugal	2017	Muscle and tendon injuries, including sprains and strains lesser dislocations	0.0075
Portugal	1990	Fracture of clavicle, scapula, or humerus	0.0349
Portugal	1995	Fracture of clavicle, scapula, or humerus	0.0349
Portugal	2000	Fracture of clavicle, scapula, or humerus	0.0349
Portugal	2005	Fracture of clavicle, scapula, or humerus	0.0349
Portugal	2010	Fracture of clavicle, scapula, or humerus	0.0349
Portugal	2017	Fracture of clavicle, scapula, or humerus	0.0349
Portugal	1990	Fracture of face bones	0.0669
Portugal	1995	Fracture of face bones	0.0669
Portugal	2000	Fracture of face bones	0.0669
Portugal	2005	Fracture of face bones	0.0669
Portugal	2010	Fracture of face bones	0.0669
Portugal	2017	Fracture of face bones	0.0669
Portugal	1990	Fracture of foot bones except ankle	0.0264
Portugal	1995	Fracture of foot bones except ankle	0.0264
Portugal	2000	Fracture of foot bones except ankle	0.0264
Portugal	2005	Fracture of foot bones except ankle	0.0264
Portugal	2010	Fracture of foot bones except ankle	0.0264
Portugal	2017	Fracture of foot bones except ankle	0.0264
Portugal	1990	Fracture of hand (wrist and other distal part of hand)	0.0138
Portugal	1995	Fracture of hand (wrist and other distal part of hand)	0.0138
Portugal	2000	Fracture of hand (wrist and other distal part of hand)	0.0138
Portugal	2005	Fracture of hand (wrist and other distal part of hand)	0.0138
Portugal	2010	Fracture of hand (wrist and other distal part of hand)	0.0138
Portugal	2017	Fracture of hand (wrist and other distal part of hand)	0.0138
Portugal	1990	Fracture of hip	0.093
Portugal	1995	Fracture of hip	0.0701
Portugal	2000	Fracture of hip	0.0577
Portugal	2005	Fracture of hip	0.0577
Portugal	2010	Fracture of hip	0.0577
Portugal	2017	Fracture of hip	0.0577
Portugal	1990	Amputation of upper limbs, bilateral	0.1241
Portugal	1995	Amputation of upper limbs, bilateral	0.1005
Portugal	2000	Amputation of upper limbs, bilateral	0.0876
Portugal	2005	Amputation of upper limbs, bilateral	0.0876
Portugal	2010	Amputation of upper limbs, bilateral	0.0876
Portugal	2017	Amputation of upper limbs, bilateral	0.0876
Portugal	1990	Fracture of patella, tibia or fibula, or ankle	0.0554
Portugal	1995	Fracture of patella, tibia or fibula, or ankle	0.0554
Portugal	2000	Fracture of patella, tibia or fibula, or ankle	0.0554
Portugal	2005	Fracture of patella, tibia or fibula, or ankle	0.0554
Portugal	2010	Fracture of patella, tibia or fibula, or ankle	0.0554
Portugal	2017	Fracture of patella, tibia or fibula, or ankle	0.0554
Portugal	1990	Fracture of pelvis	0.1823
Portugal	1995	Fracture of pelvis	0.1823
Portugal	2000	Fracture of pelvis	0.1823
Portugal	2005	Fracture of pelvis	0.1823
Portugal	2010	Fracture of pelvis	0.1823
Portugal	2017	Fracture of pelvis	0.1823
Portugal	1990	Fracture of radius and/or ulna	0.0435
Portugal	1995	Fracture of radius and/or ulna	0.0435
Portugal	2000	Fracture of radius and/or ulna	0.0435
Portugal	2005	Fracture of radius and/or ulna	0.0435
Portugal	2010	Fracture of radius and/or ulna	0.0435
Portugal	2017	Fracture of radius and/or ulna	0.0435
Portugal	1990	Fracture of skull	0.0714
Portugal	1995	Fracture of skull	0.0714
Portugal	2000	Fracture of skull	0.0714
Portugal	2005	Fracture of skull	0.0714
Portugal	2010	Fracture of skull	0.0714
Portugal	2017	Fracture of skull	0.0714
Portugal	1990	Fracture of sternum and/or fracture of one or more ribs	0.1027
Portugal	1995	Fracture of sternum and/or fracture of one or more ribs	0.1027
Portugal	2000	Fracture of sternum and/or fracture of one or more ribs	0.1027
Portugal	2005	Fracture of sternum and/or fracture of one or more ribs	0.1027
Portugal	2010	Fracture of sternum and/or fracture of one or more ribs	0.1027
Portugal	2017	Fracture of sternum and/or fracture of one or more ribs	0.1027
Portugal	1990	Fracture of vertebral column	0.1106
Portugal	1995	Fracture of vertebral column	0.1106
Portugal	2000	Fracture of vertebral column	0.1106
Portugal	2005	Fracture of vertebral column	0.1106
Portugal	2010	Fracture of vertebral column	0.1106
Portugal	2017	Fracture of vertebral column	0.1106
Portugal	1990	Fracture of femur, other than femoral neck	0.042
Portugal	1995	Fracture of femur, other than femoral neck	0.042
Portugal	2000	Fracture of femur, other than femoral neck	0.042
Portugal	2005	Fracture of femur, other than femoral neck	0.042
Portugal	2010	Fracture of femur, other than femoral neck	0.042
Portugal	2017	Fracture of femur, other than femoral neck	0.042
Portugal	1990	Minor TBI	0.1319
Portugal	1995	Minor TBI	0.1319
Portugal	2000	Minor TBI	0.1319
Portugal	2005	Minor TBI	0.1319
Portugal	2010	Minor TBI	0.1319
Portugal	2017	Minor TBI	0.1319
Portugal	1990	Moderate TBI	0.1637
Portugal	1995	Moderate TBI	0.1637
Portugal	2000	Moderate TBI	0.1637
Portugal	2005	Moderate TBI	0.1637
Portugal	2010	Moderate TBI	0.1637
Portugal	2017	Moderate TBI	0.1637

Portugal	1990	Amputation of fingers (excluding thumb)	0.0047
Portugal	1995	Amputation of fingers (excluding thumb)	0.0047
Portugal	2000	Amputation of fingers (excluding thumb)	0.0047
Portugal	2005	Amputation of fingers (excluding thumb)	0.0047
Portugal	2010	Amputation of fingers (excluding thumb)	0.0047
Portugal	2017	Amputation of fingers (excluding thumb)	0.0047
Portugal	1990	Spinal cord lesion at neck level	0.424
Portugal	1995	Spinal cord lesion at neck level	0.4161
Portugal	2000	Spinal cord lesion at neck level	0.4118
Portugal	2005	Spinal cord lesion at neck level	0.4118
Portugal	2010	Spinal cord lesion at neck level	0.4118
Portugal	2017	Spinal cord lesion at neck level	0.4118
Portugal	1990	Spinal cord lesion below neck level	0.2035
Portugal	1995	Spinal cord lesion below neck level	0.1868
Portugal	2000	Spinal cord lesion below neck level	0.1777
Portugal	2005	Spinal cord lesion below neck level	0.1777
Portugal	2010	Spinal cord lesion below neck level	0.1777
Portugal	2017	Spinal cord lesion below neck level	0.1777
Portugal	1990	Drowning and nonfatal submersion	0.2471
Portugal	1995	Drowning and nonfatal submersion	0.2471
Portugal	2000	Drowning and nonfatal submersion	0.2471
Portugal	2005	Drowning and nonfatal submersion	0.2471
Portugal	2010	Drowning and nonfatal submersion	0.2471
Portugal	2017	Drowning and nonfatal submersion	0.2471
Portugal	1990	Asphyxiation	0.2471
Portugal	1995	Asphyxiation	0.2471
Portugal	2000	Asphyxiation	0.2471
Portugal	2005	Asphyxiation	0.2471
Portugal	2010	Asphyxiation	0.2471
Portugal	2017	Asphyxiation	0.2471
Portugal	1990	Crush injury	0.1325
Portugal	1995	Crush injury	0.1325
Portugal	2000	Crush injury	0.1325
Portugal	2005	Crush injury	0.1325
Portugal	2010	Crush injury	0.1325
Portugal	2017	Crush injury	0.1325
Portugal	1990	Nerve injury	0.1129
Portugal	1995	Nerve injury	0.1129
Portugal	2000	Nerve injury	0.1129
Portugal	2005	Nerve injury	0.1129
Portugal	2010	Nerve injury	0.1129
Portugal	2017	Nerve injury	0.1129
Portugal	1990	Injury to eyes	0.0165
Portugal	1995	Injury to eyes	0.0165
Portugal	2000	Injury to eyes	0.0165
Portugal	2005	Injury to eyes	0.0165
Portugal	2010	Injury to eyes	0.0165
Portugal	2017	Injury to eyes	0.0165
Portugal	1990	Amputation of lower limb, unilateral	0.0527
Portugal	1995	Amputation of lower limb, unilateral	0.0438
Portugal	2000	Amputation of lower limb, unilateral	0.039
Portugal	2005	Amputation of lower limb, unilateral	0.039
Portugal	2010	Amputation of lower limb, unilateral	0.039
Portugal	2017	Amputation of lower limb, unilateral	0.039
Portugal	1990	Open wound(s)	0.011
Portugal	1995	Open wound(s)	0.011
Portugal	2000	Open wound(s)	0.011
Portugal	2005	Open wound(s)	0.011
Portugal	2010	Open wound(s)	0.011
Portugal	2017	Open wound(s)	0.011
Portugal	1990	Poisoning requiring urgent care	0.1628
Portugal	1995	Poisoning requiring urgent care	0.1628
Portugal	2000	Poisoning requiring urgent care	0.1628
Portugal	2005	Poisoning requiring urgent care	0.1628
Portugal	2010	Poisoning requiring urgent care	0.1628
Portugal	2017	Poisoning requiring urgent care	0.1628
Portugal	1990	Severe chest injury	0.0474
Portugal	1995	Severe chest injury	0.0474
Portugal	2000	Severe chest injury	0.0474
Portugal	2005	Severe chest injury	0.0474
Portugal	2010	Severe chest injury	0.0474
Portugal	2017	Severe chest injury	0.0474
Portugal	1990	Internal hemorrhage in abdomen and pelvis	0.1143
Portugal	1995	Internal hemorrhage in abdomen and pelvis	0.1143
Portugal	2000	Internal hemorrhage in abdomen and pelvis	0.1143
Portugal	2005	Internal hemorrhage in abdomen and pelvis	0.1143
Portugal	2010	Internal hemorrhage in abdomen and pelvis	0.1143
Portugal	2017	Internal hemorrhage in abdomen and pelvis	0.1143
Portugal	1990	Contusion in any part of the body	0.0075
Portugal	1995	Contusion in any part of the body	0.0075
Portugal	2000	Contusion in any part of the body	0.0075
Portugal	2005	Contusion in any part of the body	0.0075
Portugal	2010	Contusion in any part of the body	0.0075
Portugal	2017	Contusion in any part of the body	0.0075
Portugal	1990	Effect of different environmental factors	0.049
Portugal	1995	Effect of different environmental factors	0.049
Portugal	2000	Effect of different environmental factors	0.049
Portugal	2005	Effect of different environmental factors	0.049
Portugal	2010	Effect of different environmental factors	0.049
Portugal	2017	Effect of different environmental factors	0.049
Portugal	1990	Complications following therapeutic procedures	0.0506
Portugal	1995	Complications following therapeutic procedures	0.0506
Portugal	2000	Complications following therapeutic procedures	0.0506
Portugal	2005	Complications following therapeutic procedures	0.0506
Portugal	2010	Complications following therapeutic procedures	0.0506
Portugal	2017	Complications following therapeutic procedures	0.0506
Portugal	1990	Multiple fractures, dislocations, crashes, wounds, sprains, and strains	0.093
Portugal	1995	Multiple fractures, dislocations, crashes, wounds, sprains, and strains	0.0701
Portugal	2000	Multiple fractures, dislocations, crashes, wounds, sprains, and strains	0.0577
Portugal	2005	Multiple fractures, dislocations, crashes, wounds, sprains, and strains	0.0577
Portugal	2010	Multiple fractures, dislocations, crashes, wounds, sprains, and strains	0.0577
Portugal	2017	Multiple fractures, dislocations, crashes, wounds, sprains, and strains	0.0577
Portugal	1990	Amputation of upper limb, unilateral	0.0471
Portugal	1995	Amputation of upper limb, unilateral	0.0418
Portugal	2000	Amputation of upper limb, unilateral	0.039
Portugal	2005	Amputation of upper limb, unilateral	0.039
Portugal	2010	Amputation of upper limb, unilateral	0.039
Portugal	2017	Amputation of upper limb, unilateral	0.039
Portugal	1990	Amputation of thumb	0.0114
Portugal	1995	Amputation of thumb	0.0114
Portugal	2000	Amputation of thumb	0.0114
Portugal	2005	Amputation of thumb	0.0114
Portugal	2010	Amputation of thumb	0.0114
Portugal	2017	Amputation of thumb	0.0114
Portugal	1990	Amputation of toe/toes	0.0059
Portugal	1995	Amputation of toe/toes	0.0059
Portugal	2000	Amputation of toe/toes	0.0059
Portugal	2005	Amputation of toe/toes	0.0059
Portugal	2010	Amputation of toe/toes	0.0059
Portugal	2017	Amputation of toe/toes	0.0059
Portugal	1990	Burns, <20% total burned surface area without lower airway burns	0.0163
Portugal	1995	Burns, <20% total burned surface area without lower airway burns	0.0163
Portugal	2000	Burns, <20% total burned surface area without lower airway burns	0.0163
Portugal	2005	Burns, <20% total burned surface area without lower airway burns	0.0163
Portugal	2010	Burns, <20% total burned surface area without lower airway burns	0.0163
Portugal	2017	Burns, <20% total burned surface area without lower airway burns	0.0163
Portugal	1990	Burns, >=20% total burned surface area or >= 10% burned surface area if head/neck or hands/wrist involved w/o lower airway burns	0.1675
Portugal	1995	Burns, >=20% total burned surface area or >= 10% burned surface area if head/neck or hands/wrist involved w/o lower airway burns	0.1462
Portugal	2000	Burns, >=20% total burned surface area or >= 10% burned surface area if head/neck or hands/wrist involved w/o lower airway burns	0.1346
Portugal	2005	Burns, >=20% total burned surface area or >= 10% burned surface area if head/neck or hands/wrist involved w/o lower airway burns	0.1346
Portugal	2010	Burns, >=20% total burned surface area or >= 10% burned surface area if head/neck or hands/wrist involved w/o lower airway burns	0.1346
Portugal	2017	Burns, >=20% total burned surface area or >= 10% burned surface area if head/neck or hands/wrist involved w/o lower airway burns	0.1346
Spain	1990	Amputation of lower limbs, bilateral	0.1232
Spain	1995	Amputation of lower limbs, bilateral	0.1232
Spain	2000	Amputation of lower limbs, bilateral	0.1232
Spain	2005	Amputation of lower limbs, bilateral	0.1232
Spain	2010	Amputation of lower limbs, bilateral	0.1232
Spain	2017	Amputation of lower limbs, bilateral	0.1232
Spain	1990	Lower airway burns	0.2252
Spain	1995	Lower airway burns	0.2252
Spain	2000	Lower airway burns	0.2252
Spain	2005	Lower airway burns	0.2252
Spain	2010	Lower airway burns	0.2252
Spain	2017	Lower airway burns	0.2252
Spain	1990	Dislocation of hip	0.0159
Spain	1995	Dislocation of hip	0.0159
Spain	2000	Dislocation of hip	0.0159
Spain	2005	Dislocation of hip	0.0159
Spain	2010	Dislocation of hip	0.0159
Spain	2017	Dislocation of hip	0.0159
Spain	1990	Dislocation of knee	0.1134
Spain	1995	Dislocation of knee	0.1134
Spain	2000	Dislocation of knee	0.1134
Spain	2005	Dislocation of knee	0.1134
Spain	2010	Dislocation of knee	0.1134
Spain	2017	Dislocation of knee	0.1134
Spain	1990	Dislocation of shoulder	0.062
Spain	1995	Dislocation of shoulder	0.062
Spain	2000	Dislocation of shoulder	0.062
Spain	2005	Dislocation of shoulder	0.062
Spain	2010	Dislocation of shoulder	0.062
Spain	2017	Dislocation of shoulder	0.062
Spain	1990	Muscle and tendon injuries, including sprains and strains lesser dislocations	0.0075
Spain	1995	Muscle and tendon injuries, including sprains and strains lesser dislocations	0.0075
Spain	2000	Muscle and tendon injuries, including sprains and strains lesser dislocations	0.0075
Spain	2005	Muscle and tendon injuries, including sprains and strains lesser dislocations	0.0075
Spain	2010	Muscle and tendon injuries, including sprains and strains lesser dislocations	0.0075
Spain	2017	Muscle and tendon injuries, including sprains and strains lesser dislocations	0.0075
Spain	1990	Fracture of clavicle, scapula, or humerus	0.0349
Spain	1995	Fracture of clavicle, scapula, or humerus	0.0349
Spain	2000	Fracture of clavicle, scapula, or humerus	0.0349
Spain	2005	Fracture of clavicle, scapula, or humerus	0.0349
Spain	2010	Fracture of clavicle, scapula, or humerus	0.0349
Spain	2017	Fracture of clavicle, scapula, or humerus	0.0349
Spain	1990	Fracture of face bones	0.0669
Spain	1995	Fracture of face bones	0.0669
Spain	2000	Fracture of face bones	0.0669
Spain	2005	Fracture of face bones	0.0669
Spain	2010	Fracture of face bones	0.0669
Spain	2017	Fracture of face bones	0.0669
Spain	1990	Fracture of foot bones except ankle	0.0264
Spain	1995	Fracture of foot bones except ankle	0.0264
Spain	2000	Fracture of foot bones except ankle	0.0264
Spain	2005	Fracture of foot bones except ankle	0.0264
Spain	2010	Fracture of foot bones except ankle	0.0264
Spain	2017	Fracture of foot bones except ankle	0.0264
Spain	1990	Fracture of hand (wrist and other distal part of hand)	0.0138
Spain	1995	Fracture of hand (wrist and other distal part of hand)	0.0138
Spain	2000	Fracture of hand (wrist and other distal part of hand)	0.0138
Spain	2005	Fracture of hand (wrist and other distal part of hand)	0.0138
Spain	2010	Fracture of hand (wrist and other distal part of hand)	0.0138
Spain	2017	Fracture of hand (wrist and other distal part of hand)	0.0138
Spain	1990	Fracture of hip	0.0577
Spain	1995	Fracture of hip	0.0577
Spain	2000	Fracture of hip	0.0577
Spain	2005	Fracture of hip	0.0577
Spain	2010	Fracture of hip	0.0577
Spain	2017	Fracture of hip	0.0577
Spain	1990	Amputation of upper limbs, bilateral	0.0876
Spain	1995	Amputation of upper limbs, bilateral	0.0876
Spain	2000	Amputation of upper limbs, bilateral	0.0876
Spain	2005	Amputation of upper limbs, bilateral	0.0876
Spain	2010	Amputation of upper limbs, bilateral	0.0876
Spain	2017	Amputation of upper limbs, bilateral	0.0876
Spain	1990	Fracture of patella, tibia or fibula, or ankle	0.0554
Spain	1995	Fracture of patella, tibia or fibula, or ankle	0.0554
Spain	2000	Fracture of patella, tibia or fibula, or ankle	0.0554
Spain	2005	Fracture of patella, tibia or fibula, or ankle	0.0554
Spain	2010	Fracture of patella, tibia or fibula, or ankle	0.0554
Spain	2017	Fracture of patella, tibia or fibula, or ankle	0.0554
Spain	1990	Fracture of pelvis	0.1823
Spain	1995	Fracture of pelvis	0.1823
Spain	2000	Fracture of pelvis	0.1823
Spain	2005	Fracture of pelvis	0.1823
Spain	2010	Fracture of pelvis	0.1823
Spain	2017	Fracture of pelvis	0.1823
Spain	1990	Fracture of radius and/or ulna	0.0435
Spain	1995	Fracture of radius and/or ulna	0.0435
Spain	2000	Fracture of radius and/or ulna	0.0435
Spain	2005	Fracture of radius and/or ulna	0.0435
Spain	2010	Fracture of radius and/or ulna	0.0435
Spain	2017	Fracture of radius and/or ulna	0.0435
Spain	1990	Fracture of skull	0.0714
Spain	1995	Fracture of skull	0.0714
Spain	2000	Fracture of skull	0.0714
Spain	2005	Fracture of skull	0.0714
Spain	2010	Fracture of skull	0.0714
Spain	2017	Fracture of skull	0.0714
Spain	1990	Fracture of sternum and/or fracture of one or more ribs	0.1027
Spain	1995	Fracture of sternum and/or fracture of one or more ribs	0.1027
Spain	2000	Fracture of sternum and/or fracture of one or more ribs	0.1027
Spain	2005	Fracture of sternum and/or fracture of one or more ribs	0.1027
Spain	2010	Fracture of sternum and/or fracture of one or more ribs	0.1027
Spain	2017	Fracture of sternum and/or fracture of one or more ribs	0.1027
Spain	1990	Fracture of vertebral column	0.1106
Spain	1995	Fracture of vertebral column	0.1106
Spain	2000	Fracture of vertebral column	0.1106
Spain	2005	Fracture of vertebral column	0.1106
Spain	2010	Fracture of vertebral column	0.1106
Spain	2017	Fracture of vertebral column	0.1106
Spain	1990	Fracture of femur, other than femoral neck	0.042
Spain	1995	Fracture of femur, other than femoral neck	0.042
Spain	2000	Fracture of femur, other than femoral neck	0.042
Spain	2005	Fracture of femur, other than femoral neck	0.042
Spain	2010	Fracture of femur, other than femoral neck	0.042
Spain	2017	Fracture of femur, other than femoral neck	0.042
Spain	1990	Minor TBI	0.1319
Spain	1995	Minor TBI	0.1319
Spain	2000	Minor TBI	0.1319
Spain	2005	Minor TBI	0.1319
Spain	2010	Minor TBI	0.1319
Spain	2017	Minor TBI	0.1319
Spain	1990	Moderate TBI	0.1637
Spain	1995	Moderate TBI	0.1637
Spain	2000	Moderate TBI	0.1637
Spain	2005	Moderate TBI	0.1637
Spain	2010	Moderate TBI	0.1637
Spain	2017	Moderate TBI	0.1637
Spain	1990	Amputation of fingers (excluding thumb)	0.0047
Spain	1995	Amputation of fingers (excluding thumb)	0.0047
Spain	2000	Amputation of fingers (excluding thumb)	0.0047
Spain	2005	Amputation of fingers (excluding thumb)	0.0047
Spain	2010	Amputation of fingers (excluding thumb)	0.0047
Spain	2017	Amputation of fingers (excluding thumb)	0.0047
Spain	1990	Spinal cord lesion at neck level	0.4118
Spain	1995	Spinal cord lesion at neck level	0.4118
Spain	2000	Spinal cord lesion at neck level	0.4118
Spain	2005	Spinal cord lesion at neck level	0.4118
Spain	2010	Spinal cord lesion at neck level	0.4118
Spain	2017	Spinal cord lesion at neck level	0.4118
Spain	1990	Spinal cord lesion below neck level	0.1777
Spain	1995	Spinal cord lesion below neck level	0.1777
Spain	2000	Spinal cord lesion below neck level	0.1777
Spain	2005	Spinal cord lesion below neck level	0.1777
Spain	2010	Spinal cord lesion below neck level	0.1777
Spain	2017	Spinal cord lesion below neck level	0.1777
Spain	1990	Drowning and nonfatal submersion	0.2471
Spain	1995	Drowning and nonfatal submersion	0.2471
Spain	2000	Drowning and nonfatal submersion	0.2471
Spain	2005	Drowning and nonfatal submersion	0.2471
Spain	2010	Drowning and nonfatal submersion	0.2471
Spain	2017	Drowning and nonfatal submersion	0.2471
Spain	1990	Asphyxiation	0.2471
Spain	1995	Asphyxiation	0.2471
Spain	2000	Asphyxiation	0.2471
Spain	2005	Asphyxiation	0.2471
Spain	2010	Asphyxiation	0.2471
Spain	2017	Asphyxiation	0.2471
Spain	1990	Crush injury	0.1325
Spain	1995	Crush injury	0.1325
Spain	2000	Crush injury	0.1325
Spain	2005	Crush injury	0.1325
Spain	2010	Crush injury	0.1325
Spain	2017	Crush injury	0.1325
Spain	1990	Nerve injury	0.1129
Spain	1995	Nerve injury	0.1129
Spain	2000	Nerve injury	0.1129
Spain	2005	Nerve injury	0.1129
Spain	2010	Nerve injury	0.1129
Spain	2017	Nerve injury	0.1129
Spain	1990	Injury to eyes	0.0165
Spain	1995	Injury to eyes	0.0165
Spain	2000	Injury to eyes	0.0165
Spain	2005	Injury to eyes	0.0165
Spain	2010	Injury to eyes	0.0165
Spain	2017	Injury to eyes	0.0165
Spain	1990	Amputation of lower limb, unilateral	0.039
Spain	1995	Amputation of lower limb, unilateral	0.039
Spain	2000	Amputation of lower limb, unilateral	0.039
Spain	2005	Amputation of lower limb, unilateral	0.039
Spain	2010	Amputation of lower limb, unilateral	0.039
Spain	2017	Amputation of lower limb, unilateral	0.039
Spain	1990	Open wound(s)	0.011
Spain	1995	Open wound(s)	0.011
Spain	2000	Open wound(s)	0.011
Spain	2005	Open wound(s)	0.011
Spain	2010	Open wound(s)	0.011
Spain	2017	Open wound(s)	0.011
Spain	1990	Poisoning requiring urgent care	0.1628
Spain	1995	Poisoning requiring urgent care	0.1628
Spain	2000	Poisoning requiring urgent care	0.1628
Spain	2005	Poisoning requiring urgent care	0.1628
Spain	2010	Poisoning requiring urgent care	0.1628
Spain	2017	Poisoning requiring urgent care	0.1628
Spain	1990	Severe chest injury	0.0474
Spain	1995	Severe chest injury	0.0474
Spain	2000	Severe chest injury	0.0474
Spain	2005	Severe chest injury	0.0474
Spain	2010	Severe chest injury	0.0474
Spain	2017	Severe chest injury	0.0474
Spain	1990	Internal hemorrhage in abdomen and pelvis	0.1143
Spain	1995	Internal hemorrhage in abdomen and pelvis	0.1143
Spain	2000	Internal hemorrhage in abdomen and pelvis	0.1143
Spain	2005	Internal hemorrhage in abdomen and pelvis	0.1143
Spain	2010	Internal hemorrhage in abdomen and pelvis	0.1143
Spain	2017	Internal hemorrhage in abdomen and pelvis	0.1143
Spain	1990	Contusion in any part of the body	0.0075
Spain	1995	Contusion in any part of the body	0.0075
Spain	2000	Contusion in any part of the body	0.0075
Spain	2005	Contusion in any part of the body	0.0075
Spain	2010	Contusion in any part of the body	0.0075
Spain	2017	Contusion in any part of the body	0.0075
Spain	1990	Effect of different environmental factors	0.049
Spain	1995	Effect of different environmental factors	0.049
Spain	2000	Effect of different environmental factors	0.049
Spain	2005	Effect of different environmental factors	0.049
Spain	2010	Effect of different environmental factors	0.049
Spain	2017	Effect of different environmental factors	0.049
Spain	1990	Complications following therapeutic procedures	0.0506
Spain	1995	Complications following therapeutic procedures	0.0506
Spain	2000	Complications following therapeutic procedures	0.0506
Spain	2005	Complications following therapeutic procedures	0.0506
Spain	2010	Complications following therapeutic procedures	0.0506
Spain	2017	Complications following therapeutic procedures	0.0506
Spain	1990	Multiple fractures, dislocations, crashes, wounds, sprains, and strains	0.0577
Spain	1995	Multiple fractures, dislocations, crashes, wounds, sprains, and strains	0.0577
Spain	2000	Multiple fractures, dislocations, crashes, wounds, sprains, and strains	0.0577
Spain	2005	Multiple fractures, dislocations, crashes, wounds, sprains, and strains	0.0577
Spain	2010	Multiple fractures, dislocations, crashes, wounds, sprains, and strains	0.0577
Spain	2017	Multiple fractures, dislocations, crashes, wounds, sprains, and strains	0.0577
Spain	1990	Amputation of upper limb, unilateral	0.039
Spain	1995	Amputation of upper limb, unilateral	0.039
Spain	2000	Amputation of upper limb, unilateral	0.039
Spain	2005	Amputation of upper limb, unilateral	0.039
Spain	2010	Amputation of upper limb, unilateral	0.039
Spain	2017	Amputation of upper limb, unilateral	0.039
Spain	1990	Amputation of thumb	0.0114
Spain	1995	Amputation of thumb	0.0114
Spain	2000	Amputation of thumb	0.0114
Spain	2005	Amputation of thumb	0.0114
Spain	2010	Amputation of thumb	0.0114
Spain	2017	Amputation of thumb	0.0114
Spain	1990	Amputation of toe/toes	0.0059
Spain	1995	Amputation of toe/toes	0.0059
Spain	2000	Amputation of toe/toes	0.0059
Spain	2005	Amputation of toe/toes	0.0059
Spain	2010	Amputation of toe/toes	0.0059
Spain	2017	Amputation of toe/toes	0.0059
Spain	1990	Burns, <20% total burned surface area without lower airway burns	0.0163
Spain	1995	Burns, <20% total burned surface area without lower airway burns	0.0163
Spain	2000	Burns, <20% total burned surface area without lower airway burns	0.0163
Spain	2005	Burns, <20% total burned surface area without lower airway burns	0.0163
Spain	2010	Burns, <20% total burned surface area without lower airway burns	0.0163
Spain	2017	Burns, <20% total burned surface area without lower airway burns	0.0163
Spain	1990	Burns, >=20% total burned surface area or >= 10% burned surface area if head/neck or hands/wrist involved w/o lower airway burns	0.1346
Spain	1995	Burns, >=20% total burned surface area or >= 10% burned surface area if head/neck or hands/wrist involved w/o lower airway burns	0.1346
Spain	2000	Burns, >=20% total burned surface area or >= 10% burned surface area if head/neck or hands/wrist involved w/o lower airway burns	0.1346
Spain	2005	Burns, >=20% total burned surface area or >= 10% burned surface area if head/neck or hands/wrist involved w/o lower airway burns	0.1346
Spain	2010	Burns, >=20% total burned surface area or >= 10% burned surface area if head/neck or hands/wrist involved w/o lower airway burns	0.1346
Spain	2017	Burns, >=20% total burned surface area or >= 10% burned surface area if head/neck or hands/wrist involved w/o lower airway burns	0.1346
Switzerland	1990	Amputation of lower limbs, bilateral	0.1232
Switzerland	1995	Amputation of lower limbs, bilateral	0.1232
Switzerland	2000	Amputation of lower limbs, bilateral	0.1232
Switzerland	2005	Amputation of lower limbs, bilateral	0.1232
Switzerland	2010	Amputation of lower limbs, bilateral	0.1232
Switzerland	2017	Amputation of lower limbs, bilateral	0.1232
Switzerland	1990	Lower airway burns	0.2252
Switzerland	1995	Lower airway burns	0.2252
Switzerland	2000	Lower airway burns	0.2252
Switzerland	2005	Lower airway burns	0.2252
Switzerland	2010	Lower airway burns	0.2252
Switzerland	2017	Lower airway burns	0.2252
Switzerland	1990	Dislocation of hip	0.0159
Switzerland	1995	Dislocation of hip	0.0159
Switzerland	2000	Dislocation of hip	0.0159
Switzerland	2005	Dislocation of hip	0.0159
Switzerland	2010	Dislocation of hip	0.0159
Switzerland	2017	Dislocation of hip	0.0159
Switzerland	1990	Dislocation of knee	0.1134
Switzerland	1995	Dislocation of knee	0.1134
Switzerland	2000	Dislocation of knee	0.1134
Switzerland	2005	Dislocation of knee	0.1134
Switzerland	2010	Dislocation of knee	0.1134
Switzerland	2017	Dislocation of knee	0.1134
Switzerland	1990	Dislocation of shoulder	0.062
Switzerland	1995	Dislocation of shoulder	0.062
Switzerland	2000	Dislocation of shoulder	0.062
Switzerland	2005	Dislocation of shoulder	0.062
Switzerland	2010	Dislocation of shoulder	0.062
Switzerland	2017	Dislocation of shoulder	0.062
Switzerland	1990	Muscle and tendon injuries, including sprains and strains lesser dislocations	0.0075
Switzerland	1995	Muscle and tendon injuries, including sprains and strains lesser dislocations	0.0075
Switzerland	2000	Muscle and tendon injuries, including sprains and strains lesser dislocations	0.0075
Switzerland	2005	Muscle and tendon injuries, including sprains and strains lesser dislocations	0.0075
Switzerland	2010	Muscle and tendon injuries, including sprains and strains lesser dislocations	0.0075
Switzerland	2017	Muscle and tendon injuries, including sprains and strains lesser dislocations	0.0075
Switzerland	1990	Fracture of clavicle, scapula, or humerus	0.0349
Switzerland	1995	Fracture of clavicle, scapula, or humerus	0.0349
Switzerland	2000	Fracture of clavicle, scapula, or humerus	0.0349
Switzerland	2005	Fracture of clavicle, scapula, or humerus	0.0349
Switzerland	2010	Fracture of clavicle, scapula, or humerus	0.0349

Switzerland	2017 Fracture of clavicle, scapula, or humerus	0.0349
Switzerland	1990 Fracture of face bones	0.0669
Switzerland	1995 Fracture of face bones	0.0669
Switzerland	2000 Fracture of face bones	0.0669
Switzerland	2005 Fracture of face bones	0.0669
Switzerland	2010 Fracture of face bones	0.0669
Switzerland	2017 Fracture of face bones	0.0669
Switzerland	1990 Fracture of foot bones except ankle	0.0264
Switzerland	1995 Fracture of foot bones except ankle	0.0264
Switzerland	2000 Fracture of foot bones except ankle	0.0264
Switzerland	2005 Fracture of foot bones except ankle	0.0264
Switzerland	2010 Fracture of foot bones except ankle	0.0264
Switzerland	2017 Fracture of foot bones except ankle	0.0264
Switzerland	1990 Fracture of hand (wrist and other distal part of hand)	0.0138
Switzerland	1995 Fracture of hand (wrist and other distal part of hand)	0.0138
Switzerland	2000 Fracture of hand (wrist and other distal part of hand)	0.0138
Switzerland	2005 Fracture of hand (wrist and other distal part of hand)	0.0138
Switzerland	2010 Fracture of hand (wrist and other distal part of hand)	0.0138
Switzerland	2017 Fracture of hand (wrist and other distal part of hand)	0.0138
Switzerland	1990 Fracture of hip	0.0577
Switzerland	1995 Fracture of hip	0.0577
Switzerland	2000 Fracture of hip	0.0577
Switzerland	2005 Fracture of hip	0.0577
Switzerland	2010 Fracture of hip	0.0577
Switzerland	2017 Fracture of hip	0.0577
Switzerland	1990 Amputation of upper limbs, bilateral	0.0876
Switzerland	1995 Amputation of upper limbs, bilateral	0.0876
Switzerland	2000 Amputation of upper limbs, bilateral	0.0876
Switzerland	2005 Amputation of upper limbs, bilateral	0.0876
Switzerland	2010 Amputation of upper limbs, bilateral	0.0876
Switzerland	2017 Amputation of upper limbs, bilateral	0.0876
Switzerland	1990 Fracture of patella, tibia or fibula, or ankle	0.0554
Switzerland	1995 Fracture of patella, tibia or fibula, or ankle	0.0554
Switzerland	2000 Fracture of patella, tibia or fibula, or ankle	0.0554
Switzerland	2005 Fracture of patella, tibia or fibula, or ankle	0.0554
Switzerland	2010 Fracture of patella, tibia or fibula, or ankle	0.0554
Switzerland	2017 Fracture of patella, tibia or fibula, or ankle	0.0554
Switzerland	1990 Fracture of pelvis	0.1823
Switzerland	1995 Fracture of pelvis	0.1823
Switzerland	2000 Fracture of pelvis	0.1823
Switzerland	2005 Fracture of pelvis	0.1823
Switzerland	2010 Fracture of pelvis	0.1823
Switzerland	2017 Fracture of pelvis	0.1823
Switzerland	1990 Fracture of radius and/or ulna	0.0435
Switzerland	1995 Fracture of radius and/or ulna	0.0435
Switzerland	2000 Fracture of radius and/or ulna	0.0435
Switzerland	2005 Fracture of radius and/or ulna	0.0435
Switzerland	2010 Fracture of radius and/or ulna	0.0435
Switzerland	2017 Fracture of radius and/or ulna	0.0435
Switzerland	1990 Fracture of skull	0.0714
Switzerland	1995 Fracture of skull	0.0714
Switzerland	2000 Fracture of skull	0.0714
Switzerland	2005 Fracture of skull	0.0714
Switzerland	2010 Fracture of skull	0.0714
Switzerland	2017 Fracture of skull	0.0714
Switzerland	1990 Fracture of sternum and/or fracture of one or more ribs	0.1027
Switzerland	1995 Fracture of sternum and/or fracture of one or more ribs	0.1027
Switzerland	2000 Fracture of sternum and/or fracture of one or more ribs	0.1027
Switzerland	2005 Fracture of sternum and/or fracture of one or more ribs	0.1027
Switzerland	2010 Fracture of sternum and/or fracture of one or more ribs	0.1027
Switzerland	2017 Fracture of sternum and/or fracture of one or more ribs	0.1027
Switzerland	1990 Fracture of vertebral column	0.1106
Switzerland	1995 Fracture of vertebral column	0.1106
Switzerland	2000 Fracture of vertebral column	0.1106
Switzerland	2005 Fracture of vertebral column	0.1106
Switzerland	2010 Fracture of vertebral column	0.1106
Switzerland	2017 Fracture of vertebral column	0.1106
Switzerland	1990 Fracture of femur, other than femoral neck	0.042
Switzerland	1995 Fracture of femur, other than femoral neck	0.042
Switzerland	2000 Fracture of femur, other than femoral neck	0.042
Switzerland	2005 Fracture of femur, other than femoral neck	0.042
Switzerland	2010 Fracture of femur, other than femoral neck	0.042
Switzerland	2017 Fracture of femur, other than femoral neck	0.042
Switzerland	1990 Minor TBI	0.1319
Switzerland	1995 Minor TBI	0.1319
Switzerland	2000 Minor TBI	0.1319
Switzerland	2005 Minor TBI	0.1319
Switzerland	2010 Minor TBI	0.1319
Switzerland	2017 Minor TBI	0.1319
Switzerland	1990 Moderate TBI	0.1637
Switzerland	1995 Moderate TBI	0.1637
Switzerland	2000 Moderate TBI	0.1637
Switzerland	2005 Moderate TBI	0.1637
Switzerland	2010 Moderate TBI	0.1637
Switzerland	2017 Moderate TBI	0.1637
Switzerland	1990 Amputation of fingers (excluding thumb)	0.0047
Switzerland	1995 Amputation of fingers (excluding thumb)	0.0047
Switzerland	2000 Amputation of fingers (excluding thumb)	0.0047
Switzerland	2005 Amputation of fingers (excluding thumb)	0.0047
Switzerland	2010 Amputation of fingers (excluding thumb)	0.0047
Switzerland	2017 Amputation of fingers (excluding thumb)	0.0047
Switzerland	1990 Spinal cord lesion at neck level	0.4118
Switzerland	1995 Spinal cord lesion at neck level	0.4118
Switzerland	2000 Spinal cord lesion at neck level	0.4118
Switzerland	2005 Spinal cord lesion at neck level	0.4118
Switzerland	2010 Spinal cord lesion at neck level	0.4118
Switzerland	2017 Spinal cord lesion at neck level	0.4118
Switzerland	1990 Spinal cord lesion below neck level	0.1777
Switzerland	1995 Spinal cord lesion below neck level	0.1777
Switzerland	2000 Spinal cord lesion below neck level	0.1777
Switzerland	2005 Spinal cord lesion below neck level	0.1777
Switzerland	2010 Spinal cord lesion below neck level	0.1777
Switzerland	2017 Spinal cord lesion below neck level	0.1777
Switzerland	1990 Drowning and nonfatal submersion	0.2471
Switzerland	1995 Drowning and nonfatal submersion	0.2471
Switzerland	2000 Drowning and nonfatal submersion	0.2471
Switzerland	2005 Drowning and nonfatal submersion	0.2471
Switzerland	2010 Drowning and nonfatal submersion	0.2471
Switzerland	2017 Drowning and nonfatal submersion	0.2471
Switzerland	1990 Asphyxiation	0.2471
Switzerland	1995 Asphyxiation	0.2471
Switzerland	2000 Asphyxiation	0.2471
Switzerland	2005 Asphyxiation	0.2471
Switzerland	2010 Asphyxiation	0.2471
Switzerland	2017 Asphyxiation	0.2471
Switzerland	1990 Crush injury	0.1325
Switzerland	1995 Crush injury	0.1325
Switzerland	2000 Crush injury	0.1325
Switzerland	2005 Crush injury	0.1325
Switzerland	2010 Crush injury	0.1325
Switzerland	2017 Crush injury	0.1325
Switzerland	1990 Nerve injury	0.1129
Switzerland	1995 Nerve injury	0.1129
Switzerland	2000 Nerve injury	0.1129
Switzerland	2005 Nerve injury	0.1129
Switzerland	2010 Nerve injury	0.1129
Switzerland	2017 Nerve injury	0.1129
Switzerland	1990 Injury to eyes	0.0165
Switzerland	1995 Injury to eyes	0.0165
Switzerland	2000 Injury to eyes	0.0165
Switzerland	2005 Injury to eyes	0.0165
Switzerland	2010 Injury to eyes	0.0165
Switzerland	2017 Injury to eyes	0.0165
Switzerland	1990 Amputation of lower limb, unilateral	0.039
Switzerland	1995 Amputation of lower limb, unilateral	0.039
Switzerland	2000 Amputation of lower limb, unilateral	0.039
Switzerland	2005 Amputation of lower limb, unilateral	0.039
Switzerland	2010 Amputation of lower limb, unilateral	0.039
Switzerland	2017 Amputation of lower limb, unilateral	0.039
Switzerland	1990 Open wound(s)	0.011
Switzerland	1995 Open wound(s)	0.011
Switzerland	2000 Open wound(s)	0.011
Switzerland	2005 Open wound(s)	0.011
Switzerland	2010 Open wound(s)	0.011
Switzerland	2017 Open wound(s)	0.011
Switzerland	1990 Poisoning requiring urgent care	0.1628
Switzerland	1995 Poisoning requiring urgent care	0.1628
Switzerland	2000 Poisoning requiring urgent care	0.1628
Switzerland	2005 Poisoning requiring urgent care	0.1628
Switzerland	2010 Poisoning requiring urgent care	0.1628
Switzerland	2017 Poisoning requiring urgent care	0.1628
Switzerland	1990 Severe chest injury	0.0474
Switzerland	1995 Severe chest injury	0.0474
Switzerland	2000 Severe chest injury	0.0474
Switzerland	2005 Severe chest injury	0.0474
Switzerland	2010 Severe chest injury	0.0474
Switzerland	2017 Severe chest injury	0.0474
Switzerland	1990 Internal hemorrhage in abdomen and pelvis	0.1143
Switzerland	1995 Internal hemorrhage in abdomen and pelvis	0.1143
Switzerland	2000 Internal hemorrhage in abdomen and pelvis	0.1143
Switzerland	2005 Internal hemorrhage in abdomen and pelvis	0.1143
Switzerland	2010 Internal hemorrhage in abdomen and pelvis	0.1143
Switzerland	2017 Internal hemorrhage in abdomen and pelvis	0.1143
Switzerland	1990 Contusion in any part of the body	0.0075
Switzerland	1995 Contusion in any part of the body	0.0075
Switzerland	2000 Contusion in any part of the body	0.0075
Switzerland	2005 Contusion in any part of the body	0.0075
Switzerland	2010 Contusion in any part of the body	0.0075
Switzerland	2017 Contusion in any part of the body	0.0075
Switzerland	1990 Effect of different environmental factors	0.049
Switzerland	1995 Effect of different environmental factors	0.049
Switzerland	2000 Effect of different environmental factors	0.049
Switzerland	2005 Effect of different environmental factors	0.049
Switzerland	2010 Effect of different environmental factors	0.049
Switzerland	2017 Effect of different environmental factors	0.049
Switzerland	1990 Complications following therapeutic procedures	0.0506
Switzerland	1995 Complications following therapeutic procedures	0.0506
Switzerland	2000 Complications following therapeutic procedures	0.0506
Switzerland	2005 Complications following therapeutic procedures	0.0506
Switzerland	2010 Complications following therapeutic procedures	0.0506
Switzerland	2017 Complications following therapeutic procedures	0.0506
Switzerland	1990 Multiple fractures, dislocations, crashes, wounds, sprains, and strains	0.0577
Switzerland	1995 Multiple fractures, dislocations, crashes, wounds, sprains, and strains	0.0577
Switzerland	2000 Multiple fractures, dislocations, crashes, wounds, sprains, and strains	0.0577
Switzerland	2005 Multiple fractures, dislocations, crashes, wounds, sprains, and strains	0.0577
Switzerland	2010 Multiple fractures, dislocations, crashes, wounds, sprains, and strains	0.0577
Switzerland	2017 Multiple fractures, dislocations, crashes, wounds, sprains, and strains	0.0577
Switzerland	1990 Amputation of upper limb, unilateral	0.039
Switzerland	1995 Amputation of upper limb, unilateral	0.039
Switzerland	2000 Amputation of upper limb, unilateral	0.039
Switzerland	2005 Amputation of upper limb, unilateral	0.039
Switzerland	2010 Amputation of upper limb, unilateral	0.039
Switzerland	2017 Amputation of upper limb, unilateral	0.039
Switzerland	1990 Amputation of thumb	0.0114
Switzerland	1995 Amputation of thumb	0.0114
Switzerland	2000 Amputation of thumb	0.0114
Switzerland	2005 Amputation of thumb	0.0114
Switzerland	2010 Amputation of thumb	0.0114
Switzerland	2017 Amputation of thumb	0.0114
Switzerland	1990 Amputation of toe/toes	0.0059
Switzerland	1995 Amputation of toe/toes	0.0059
Switzerland	2000 Amputation of toe/toes	0.0059
Switzerland	2005 Amputation of toe/toes	0.0059
Switzerland	2010 Amputation of toe/toes	0.0059
Switzerland	2017 Amputation of toe/toes	0.0059
Switzerland	1990 Burns, <20% total burned surface area without lower airway burns	0.0163
Switzerland	1995 Burns, <20% total burned surface area without lower airway burns	0.0163
Switzerland	2000 Burns, <20% total burned surface area without lower airway burns	0.0163
Switzerland	2005 Burns, <20% total burned surface area without lower airway burns	0.0163
Switzerland	2010 Burns, <20% total burned surface area without lower airway burns	0.0163
Switzerland	2017 Burns, <20% total burned surface area without lower airway burns	0.0163
Switzerland	1990 Burns, >=20% total burned surface area or >= 10% burned surface area if head/neck or hands/wrist involved w/o lower airway burns	0.1346
Switzerland	1995 Burns, >=20% total burned surface area or >= 10% burned surface area if head/neck or hands/wrist involved w/o lower airway burns	0.1346
Switzerland	2000 Burns, >=20% total burned surface area or >= 10% burned surface area if head/neck or hands/wrist involved w/o lower airway burns	0.1346
Switzerland	2005 Burns, >=20% total burned surface area or >= 10% burned surface area if head/neck or hands/wrist involved w/o lower airway burns	0.1346
Switzerland	2010 Burns, >=20% total burned surface area or >= 10% burned surface area if head/neck or hands/wrist involved w/o lower airway burns	0.1346
Switzerland	2017 Burns, >=20% total burned surface area or >= 10% burned surface area if head/neck or hands/wrist involved w/o lower airway burns	0.1346
Argentina	1990 Amputation of lower limbs, bilateral	0.1949
Argentina	1995 Amputation of lower limbs, bilateral	0.1793
Argentina	2000 Amputation of lower limbs, bilateral	0.168
Argentina	2005 Amputation of lower limbs, bilateral	0.1631
Argentina	2010 Amputation of lower limbs, bilateral	0.157
Argentina	2017 Amputation of lower limbs, bilateral	0.1452
Argentina	1990 Lower airway burns	0.2252
Argentina	1995 Lower airway burns	0.2252
Argentina	2000 Lower airway burns	0.2252
Argentina	2005 Lower airway burns	0.2252
Argentina	2010 Lower airway burns	0.2252
Argentina	2017 Lower airway burns	0.2252
Argentina	1990 Dislocation of hip	0.0159
Argentina	1995 Dislocation of hip	0.0159
Argentina	2000 Dislocation of hip	0.0159
Argentina	2005 Dislocation of hip	0.0159
Argentina	2010 Dislocation of hip	0.0159
Argentina	2017 Dislocation of hip	0.0159
Argentina	1990 Dislocation of knee	0.1134
Argentina	1995 Dislocation of knee	0.1134
Argentina	2000 Dislocation of knee	0.1134
Argentina	2005 Dislocation of knee	0.1134
Argentina	2010 Dislocation of knee	0.1134
Argentina	2017 Dislocation of knee	0.1134
Argentina	1990 Dislocation of shoulder	0.062
Argentina	1995 Dislocation of shoulder	0.062
Argentina	2000 Dislocation of shoulder	0.062
Argentina	2005 Dislocation of shoulder	0.062
Argentina	2010 Dislocation of shoulder	0.062
Argentina	2017 Dislocation of shoulder	0.062
Argentina	1990 Muscle and tendon injuries, including sprains and strains lesser dislocations	0.0075
Argentina	1995 Muscle and tendon injuries, including sprains and strains lesser dislocations	0.0075
Argentina	2000 Muscle and tendon injuries, including sprains and strains lesser dislocations	0.0075
Argentina	2005 Muscle and tendon injuries, including sprains and strains lesser dislocations	0.0075
Argentina	2010 Muscle and tendon injuries, including sprains and strains lesser dislocations	0.0075
Argentina	2017 Muscle and tendon injuries, including sprains and strains lesser dislocations	0.0075
Argentina	1990 Fracture of clavicle, scapula, or humerus	0.0349
Argentina	1995 Fracture of clavicle, scapula, or humerus	0.0349
Argentina	2000 Fracture of clavicle, scapula, or humerus	0.0349
Argentina	2005 Fracture of clavicle, scapula, or humerus	0.0349
Argentina	2010 Fracture of clavicle, scapula, or humerus	0.0349
Argentina	2017 Fracture of clavicle, scapula, or humerus	0.0349
Argentina	1990 Fracture of face bones	0.0669
Argentina	1995 Fracture of face bones	0.0669
Argentina	2000 Fracture of face bones	0.0669
Argentina	2005 Fracture of face bones	0.0669
Argentina	2010 Fracture of face bones	0.0669
Argentina	2017 Fracture of face bones	0.0669
Argentina	1990 Fracture of foot bones except ankle	0.0264
Argentina	1995 Fracture of foot bones except ankle	0.0264
Argentina	2000 Fracture of foot bones except ankle	0.0264
Argentina	2005 Fracture of foot bones except ankle	0.0264
Argentina	2010 Fracture of foot bones except ankle	0.0264
Argentina	2017 Fracture of foot bones except ankle	0.0264
Argentina	1990 Fracture of hand (wrist and other distal part of hand)	0.0138
Argentina	1995 Fracture of hand (wrist and other distal part of hand)	0.0138
Argentina	2000 Fracture of hand (wrist and other distal part of hand)	0.0138
Argentina	2005 Fracture of hand (wrist and other distal part of hand)	0.0138
Argentina	2010 Fracture of hand (wrist and other distal part of hand)	0.0138
Argentina	2017 Fracture of hand (wrist and other distal part of hand)	0.0138
Argentina	1990 Fracture of hip	0.1524
Argentina	1995 Fracture of hip	0.1318
Argentina	2000 Fracture of hip	0.1169
Argentina	2005 Fracture of hip	0.1103
Argentina	2010 Fracture of hip	0.1023
Argentina	2017 Fracture of hip	0.0867
Argentina	1990 Amputation of upper limbs, bilateral	0.1855
Argentina	1995 Amputation of upper limbs, bilateral	0.1641
Argentina	2000 Amputation of upper limbs, bilateral	0.1488
Argentina	2005 Amputation of upper limbs, bilateral	0.142
Argentina	2010 Amputation of upper limbs, bilateral	0.1338
Argentina	2017 Amputation of upper limbs, bilateral	0.1176
Argentina	1990 Fracture of patella, tibia or fibula, or ankle	0.0554
Argentina	1995 Fracture of patella, tibia or fibula, or ankle	0.0554
Argentina	2000 Fracture of patella, tibia or fibula, or ankle	0.0554
Argentina	2005 Fracture of patella, tibia or fibula, or ankle	0.0554
Argentina	2010 Fracture of patella, tibia or fibula, or ankle	0.0554
Argentina	2017 Fracture of patella, tibia or fibula, or ankle	0.0554
Argentina	1990 Fracture of pelvis	0.1823
Argentina	1995 Fracture of pelvis	0.1823
Argentina	2000 Fracture of pelvis	0.1823
Argentina	2005 Fracture of pelvis	0.1823
Argentina	2010 Fracture of pelvis	0.1823
Argentina	2017 Fracture of pelvis	0.1823
Argentina	1990 Fracture of radius and/or ulna	0.0435
Argentina	1995 Fracture of radius and/or ulna	0.0435
Argentina	2000 Fracture of radius and/or ulna	0.0435
Argentina	2005 Fracture of radius and/or ulna	0.0435
Argentina	2010 Fracture of radius and/or ulna	0.0435
Argentina	2017 Fracture of radius and/or ulna	0.0435
Argentina	1990 Fracture of skull	0.0714
Argentina	1995 Fracture of skull	0.0714
Argentina	2000 Fracture of skull	0.0714
Argentina	2005 Fracture of skull	0.0714
Argentina	2010 Fracture of skull	0.0714
Argentina	2017 Fracture of skull	0.0714
Argentina	1990 Fracture of sternum and/or fracture of one or more ribs	0.1027
Argentina	1995 Fracture of sternum and/or fracture of one or more ribs	0.1027
Argentina	2000 Fracture of sternum and/or fracture of one or more ribs	0.1027
Argentina	2005 Fracture of sternum and/or fracture of one or more ribs	0.1027
Argentina	2010 Fracture of sternum and/or fracture of one or more ribs	0.1027
Argentina	2017 Fracture of sternum and/or fracture of one or more ribs	0.1027
Argentina	1990 Fracture of vertebral column	0.1106
Argentina	1995 Fracture of vertebral column	0.1106
Argentina	2000 Fracture of vertebral column	0.1106
Argentina	2005 Fracture of vertebral column	0.1106
Argentina	2010 Fracture of vertebral column	0.1106
Argentina	2017 Fracture of vertebral column	0.1106
Argentina	1990 Fracture of femur, other than femoral neck	0.042
Argentina	1995 Fracture of femur, other than femoral neck	0.042
Argentina	2000 Fracture of femur, other than femoral neck	0.042
Argentina	2005 Fracture of femur, other than femoral neck	0.042
Argentina	2010 Fracture of femur, other than femoral neck	0.042
Argentina	2017 Fracture of femur, other than femoral neck	0.042
Argentina	1990 Minor TBI	0.1319
Argentina	1995 Minor TBI	0.1319
Argentina	2000 Minor TBI	0.1319
Argentina	2005 Minor TBI	0.1319
Argentina	2010 Minor TBI	0.1319
Argentina	2017 Minor TBI	0.1319
Argentina	1990 Moderate TBI	0.1637
Argentina	1995 Moderate TBI	0.1637
Argentina	2000 Moderate TBI	0.1637
Argentina	2005 Moderate TBI	0.1637
Argentina	2010 Moderate TBI	0.1637
Argentina	2017 Moderate TBI	0.1637
Argentina	1990 Amputation of fingers (excluding thumb)	0.0047
Argentina	1995 Amputation of fingers (excluding thumb)	0.0047
Argentina	2000 Amputation of fingers (excluding thumb)	0.0047
Argentina	2005 Amputation of fingers (excluding thumb)	0.0047
Argentina	2010 Amputation of fingers (excluding thumb)	0.0047
Argentina	2017 Amputation of fingers (excluding thumb)	0.0047
Argentina	1990 Spinal cord lesion at neck level	0.4446
Argentina	1995 Spinal cord lesion at neck level	0.4374
Argentina	2000 Spinal cord lesion at neck level	0.4323
Argentina	2005 Spinal cord lesion at neck level	0.43
Argentina	2010 Spinal cord lesion at neck level	0.4273
Argentina	2017 Spinal cord lesion at neck level	0.4219
Argentina	1990 Spinal cord lesion below neck level	0.2468
Argentina	1995 Spinal cord lesion below neck level	0.2317
Argentina	2000 Spinal cord lesion below neck level	0.2209
Argentina	2005 Spinal cord lesion below neck level	0.2161
Argentina	2010 Spinal cord lesion below neck level	0.2103
Argentina	2017 Spinal cord lesion below neck level	0.1988
Argentina	1990 Drowning and nonfatal submersion	0.2471
Argentina	1995 Drowning and nonfatal submersion	0.2471
Argentina	2000 Drowning and nonfatal submersion	0.2471
Argentina	2005 Drowning and nonfatal submersion	0.2471
Argentina	2010 Drowning and nonfatal submersion	0.2471
Argentina	2017 Drowning and nonfatal submersion	0.2471
Argentina	1990 Asphyxiation	0.2471
Argentina	1995 Asphyxiation	0.2471
Argentina	2000 Asphyxiation	0.2471
Argentina	2005 Asphyxiation	0.2471
Argentina	2010 Asphyxiation	0.2471
Argentina	2017 Asphyxiation	0.2471
Argentina	1990 Crush injury	0.1325
Argentina	1995 Crush injury	0.1325
Argentina	2000 Crush injury	0.1325
Argentina	2005 Crush injury	0.1325
Argentina	2010 Crush injury	0.1325
Argentina	2017 Crush injury	0.1325
Argentina	1990 Nerve injury	0.1129
Argentina	1995 Nerve injury	0.1129
Argentina	2000 Nerve injury	0.1129
Argentina	2005 Nerve injury	0.1129
Argentina	2010 Nerve injury	0.1129
Argentina	2017 Nerve injury	0.1129
Argentina	1990 Injury to eyes	0.0165
Argentina	1995 Injury to eyes	0.0165
Argentina	2000 Injury to eyes	0.0165
Argentina	2005 Injury to eyes	0.0165
Argentina	2010 Injury to eyes	0.0165
Argentina	2017 Injury to eyes	0.0165
Argentina	1990 Amputation of lower limb, unilateral	0.0759
Argentina	1995 Amputation of lower limb, unilateral	0.0678
Argentina	2000 Amputation of lower limb, unilateral	0.062
Argentina	2005 Amputation of lower limb, unilateral	0.0595
Argentina	2010 Amputation of lower limb, unilateral	0.0564
Argentina	2017 Amputation of lower limb, unilateral	0.0503
Argentina	1990 Open wound(s)	0.011
Argentina	1995 Open wound(s)	0.011
Argentina	2000 Open wound(s)	0.011
Argentina	2005 Open wound(s)	0.011
Argentina	2010 Open wound(s)	0.011
Argentina	2017 Open wound(s)	0.011
Argentina	1990 Poisoning requiring urgent care	0.1628
Argentina	1995 Poisoning requiring urgent care	0.1628
Argentina	2000 Poisoning requiring urgent care	0.1628
Argentina	2005 Poisoning requiring urgent care	0.1628
Argentina	2010 Poisoning requiring urgent care	0.1628
Argentina	2017 Poisoning requiring urgent care	0.1628
Argentina	1990 Severe chest injury	0.0474
Argentina	1995 Severe chest injury	0.0474
Argentina	2000 Severe chest injury	0.0474
Argentina	2005 Severe chest injury	0.0474
Argentina	2010 Severe chest injury	0.0474
Argentina	2017 Severe chest injury	0.0474
Argentina	1990 Internal hemorrhage in abdomen and pelvis	0.1143
Argentina	1995 Internal hemorrhage in abdomen and pelvis	0.1143
Argentina	2000 Internal hemorrhage in abdomen and pelvis	0.1143
Argentina	2005 Internal hemorrhage in abdomen and pelvis	0.1143
Argentina	2010 Internal hemorrhage in abdomen and pelvis	0.1143
Argentina	2017 Internal hemorrhage in abdomen and pelvis	0.1143
Argentina	1990 Contusion in any part of the body	0.0075
Argentina	1995 Contusion in any part of the body	0.0075
Argentina	2000 Contusion in any part of the body	0.0075
Argentina	2005 Contusion in any part of the body	0.0075
Argentina	2010 Contusion in any part of the body	0.0075
Argentina	2017 Contusion in any part of the body	0.0075
Argentina	1990 Effect of different environmental factors	0.049
Argentina	1995 Effect of different environmental factors	0.049
Argentina	2000 Effect of different environmental factors	0.049
Argentina	2005 Effect of different environmental factors	0.049

Argentina	2010	Effect of different environmental factors	0.049
Argentina	2017	Effect of different environmental factors	0.049
Argentina	1990	Complications following therapeutic procedures	0.0506
Argentina	1995	Complications following therapeutic procedures	0.0506
Argentina	2000	Complications following therapeutic procedures	0.0506
Argentina	2005	Complications following therapeutic procedures	0.0506
Argentina	2010	Complications following therapeutic procedures	0.0506
Argentina	2017	Complications following therapeutic procedures	0.0506
Argentina	1990	Multiple fractures, dislocations, crashes, wounds, sprains, and strains	0.1524
Argentina	1995	Multiple fractures, dislocations, crashes, wounds, sprains, and strains	0.1318
Argentina	2000	Multiple fractures, dislocations, crashes, wounds, sprains, and strains	0.1169
Argentina	2005	Multiple fractures, dislocations, crashes, wounds, sprains, and strains	0.1103
Argentina	2010	Multiple fractures, dislocations, crashes, wounds, sprains, and strains	0.1023
Argentina	2017	Multiple fractures, dislocations, crashes, wounds, sprains, and strains	0.0867
Argentina	1990	Amputation of upper limb, unilateral	0.0607
Argentina	1995	Amputation of upper limb, unilateral	0.056
Argentina	2000	Amputation of upper limb, unilateral	0.0526
Argentina	2005	Amputation of upper limb, unilateral	0.0511
Argentina	2010	Amputation of upper limb, unilateral	0.0492
Argentina	2017	Amputation of upper limb, unilateral	0.0456
Argentina	1990	Amputation of thumb	0.0114
Argentina	1995	Amputation of thumb	0.0114
Argentina	2000	Amputation of thumb	0.0114
Argentina	2005	Amputation of thumb	0.0114
Argentina	2010	Amputation of thumb	0.0114
Argentina	2017	Amputation of thumb	0.0114
Argentina	1990	Amputation of toe/toes	0.0059
Argentina	1995	Amputation of toe/toes	0.0059
Argentina	2000	Amputation of toe/toes	0.0059
Argentina	2005	Amputation of toe/toes	0.0059
Argentina	2010	Amputation of toe/toes	0.0059
Argentina	2017	Amputation of toe/toes	0.0059
Argentina	1990	Burns, <20% total burned surface area without lower airway burns	0.0163
Argentina	1995	Burns, <20% total burned surface area without lower airway burns	0.0163
Argentina	2000	Burns, <20% total burned surface area without lower airway burns	0.0163
Argentina	2005	Burns, <20% total burned surface area without lower airway burns	0.0163
Argentina	2010	Burns, <20% total burned surface area without lower airway burns	0.0163
Argentina	2017	Burns, <20% total burned surface area without lower airway burns	0.0163
Argentina	1990	Burns, >=20% total burned surface area or >= 10% burned surface area if head/neck or hands/wrist involved w/o lower airway burns	0.2228
Argentina	1995	Burns, >=20% total burned surface area or >= 10% burned surface area if head/neck or hands/wrist involved w/o lower airway burns	0.2035
Argentina	2000	Burns, >=20% total burned surface area or >= 10% burned surface area if head/neck or hands/wrist involved w/o lower airway burns	0.1897
Argentina	2005	Burns, >=20% total burned surface area or >= 10% burned surface area if head/neck or hands/wrist involved w/o lower airway burns	0.1836
Argentina	2010	Burns, >=20% total burned surface area or >= 10% burned surface area if head/neck or hands/wrist involved w/o lower airway burns	0.1762
Argentina	2017	Burns, >=20% total burned surface area or >= 10% burned surface area if head/neck or hands/wrist involved w/o lower airway burns	0.1616
Chile	1990	Amputation of lower limbs, bilateral	0.1855
Chile	1995	Amputation of lower limbs, bilateral	0.1672
Chile	2000	Amputation of lower limbs, bilateral	0.1502
Chile	2005	Amputation of lower limbs, bilateral	0.1371
Chile	2010	Amputation of lower limbs, bilateral	0.1266
Chile	2017	Amputation of lower limbs, bilateral	0.1232
Chile	1990	Lower airway burns	0.2252
Chile	1995	Lower airway burns	0.2252
Chile	2000	Lower airway burns	0.2252
Chile	2005	Lower airway burns	0.2252
Chile	2010	Lower airway burns	0.2252
Chile	2017	Lower airway burns	0.2252
Chile	1990	Dislocation of hip	0.0159
Chile	1995	Dislocation of hip	0.0159
Chile	2000	Dislocation of hip	0.0159
Chile	2005	Dislocation of hip	0.0159
Chile	2010	Dislocation of hip	0.0159
Chile	2017	Dislocation of hip	0.0159
Chile	1990	Dislocation of knee	0.1134
Chile	1995	Dislocation of knee	0.1134
Chile	2000	Dislocation of knee	0.1134
Chile	2005	Dislocation of knee	0.1134
Chile	2010	Dislocation of knee	0.1134
Chile	2017	Dislocation of knee	0.1134
Chile	1990	Dislocation of shoulder	0.062
Chile	1995	Dislocation of shoulder	0.062
Chile	2000	Dislocation of shoulder	0.062
Chile	2005	Dislocation of shoulder	0.062
Chile	2010	Dislocation of shoulder	0.062
Chile	2017	Dislocation of shoulder	0.062
Chile	1990	Muscle and tendon injuries, including sprains and strains lesser dislocations	0.0075
Chile	1995	Muscle and tendon injuries, including sprains and strains lesser dislocations	0.0075
Chile	2000	Muscle and tendon injuries, including sprains and strains lesser dislocations	0.0075
Chile	2005	Muscle and tendon injuries, including sprains and strains lesser dislocations	0.0075
Chile	2010	Muscle and tendon injuries, including sprains and strains lesser dislocations	0.0075
Chile	2017	Muscle and tendon injuries, including sprains and strains lesser dislocations	0.0075
Chile	1990	Fracture of clavicle, scapula, or humerus	0.0349
Chile	1995	Fracture of clavicle, scapula, or humerus	0.0349
Chile	2000	Fracture of clavicle, scapula, or humerus	0.0349
Chile	2005	Fracture of clavicle, scapula, or humerus	0.0349
Chile	2010	Fracture of clavicle, scapula, or humerus	0.0349
Chile	2017	Fracture of clavicle, scapula, or humerus	0.0349
Chile	1990	Fracture of face bones	0.0669
Chile	1995	Fracture of face bones	0.0669
Chile	2000	Fracture of face bones	0.0669
Chile	2005	Fracture of face bones	0.0669
Chile	2010	Fracture of face bones	0.0669
Chile	2017	Fracture of face bones	0.0669
Chile	1990	Fracture of foot bones except ankle	0.0264
Chile	1995	Fracture of foot bones except ankle	0.0264
Chile	2000	Fracture of foot bones except ankle	0.0264
Chile	2005	Fracture of foot bones except ankle	0.0264
Chile	2010	Fracture of foot bones except ankle	0.0264
Chile	2017	Fracture of foot bones except ankle	0.0264
Chile	1990	Fracture of hand (wrist and other distal part of hand)	0.0138
Chile	1995	Fracture of hand (wrist and other distal part of hand)	0.0138
Chile	2000	Fracture of hand (wrist and other distal part of hand)	0.0138
Chile	2005	Fracture of hand (wrist and other distal part of hand)	0.0138
Chile	2010	Fracture of hand (wrist and other distal part of hand)	0.0138
Chile	2017	Fracture of hand (wrist and other distal part of hand)	0.0138
Chile	1990	Fracture of hip	0.14
Chile	1995	Fracture of hip	0.1159
Chile	2000	Fracture of hip	0.0934
Chile	2005	Fracture of hip	0.076
Chile	2010	Fracture of hip	0.0621
Chile	2017	Fracture of hip	0.0577
Chile	1990	Amputation of upper limbs, bilateral	0.1727
Chile	1995	Amputation of upper limbs, bilateral	0.1477
Chile	2000	Amputation of upper limbs, bilateral	0.1245
Chile	2005	Amputation of upper limbs, bilateral	0.1065
Chile	2010	Amputation of upper limbs, bilateral	0.0922
Chile	2017	Amputation of upper limbs, bilateral	0.0876
Chile	1990	Fracture of patella, tibia or fibula, or ankle	0.0554
Chile	1995	Fracture of patella, tibia or fibula, or ankle	0.0554
Chile	2000	Fracture of patella, tibia or fibula, or ankle	0.0554
Chile	2005	Fracture of patella, tibia or fibula, or ankle	0.0554
Chile	2010	Fracture of patella, tibia or fibula, or ankle	0.0554
Chile	2017	Fracture of patella, tibia or fibula, or ankle	0.0554
Chile	1990	Fracture of pelvis	0.1823
Chile	1995	Fracture of pelvis	0.1823
Chile	2000	Fracture of pelvis	0.1823
Chile	2005	Fracture of pelvis	0.1823
Chile	2010	Fracture of pelvis	0.1823
Chile	2017	Fracture of pelvis	0.1823
Chile	1990	Fracture of radius and/or ulna	0.0435
Chile	1995	Fracture of radius and/or ulna	0.0435
Chile	2000	Fracture of radius and/or ulna	0.0435
Chile	2005	Fracture of radius and/or ulna	0.0435
Chile	2010	Fracture of radius and/or ulna	0.0435
Chile	2017	Fracture of radius and/or ulna	0.0435
Chile	1990	Fracture of skull	0.0714
Chile	1995	Fracture of skull	0.0714
Chile	2000	Fracture of skull	0.0714
Chile	2005	Fracture of skull	0.0714
Chile	2010	Fracture of skull	0.0714
Chile	2017	Fracture of skull	0.0714
Chile	1990	Fracture of sternum and/or fracture of one or more ribs	0.1027
Chile	1995	Fracture of sternum and/or fracture of one or more ribs	0.1027
Chile	2000	Fracture of sternum and/or fracture of one or more ribs	0.1027
Chile	2005	Fracture of sternum and/or fracture of one or more ribs	0.1027
Chile	2010	Fracture of sternum and/or fracture of one or more ribs	0.1027
Chile	2017	Fracture of sternum and/or fracture of one or more ribs	0.1027
Chile	1990	Fracture of vertebral column	0.1106
Chile	1995	Fracture of vertebral column	0.1106
Chile	2000	Fracture of vertebral column	0.1106
Chile	2005	Fracture of vertebral column	0.1106
Chile	2010	Fracture of vertebral column	0.1106
Chile	2017	Fracture of vertebral column	0.1106
Chile	1990	Fracture of femur, other than femoral neck	0.042
Chile	1995	Fracture of femur, other than femoral neck	0.042
Chile	2000	Fracture of femur, other than femoral neck	0.042
Chile	2005	Fracture of femur, other than femoral neck	0.042
Chile	2010	Fracture of femur, other than femoral neck	0.042
Chile	2017	Fracture of femur, other than femoral neck	0.042
Chile	1990	Minor TBI	0.1319
Chile	1995	Minor TBI	0.1319
Chile	2000	Minor TBI	0.1319
Chile	2005	Minor TBI	0.1319
Chile	2010	Minor TBI	0.1319
Chile	2017	Minor TBI	0.1319
Chile	1990	Moderate TBI	0.1637
Chile	1995	Moderate TBI	0.1637
Chile	2000	Moderate TBI	0.1637
Chile	2005	Moderate TBI	0.1637
Chile	2010	Moderate TBI	0.1637
Chile	2017	Moderate TBI	0.1637
Chile	1990	Amputation of fingers (excluding thumb)	0.0047
Chile	1995	Amputation of fingers (excluding thumb)	0.0047
Chile	2000	Amputation of fingers (excluding thumb)	0.0047
Chile	2005	Amputation of fingers (excluding thumb)	0.0047
Chile	2010	Amputation of fingers (excluding thumb)	0.0047
Chile	2017	Amputation of fingers (excluding thumb)	0.0047
Chile	1990	Spinal cord lesion at neck level	0.4403
Chile	1995	Spinal cord lesion at neck level	0.4319
Chile	2000	Spinal cord lesion at neck level	0.4242
Chile	2005	Spinal cord lesion at neck level	0.4181
Chile	2010	Spinal cord lesion at neck level	0.4133
Chile	2017	Spinal cord lesion at neck level	0.4118
Chile	1990	Spinal cord lesion below neck level	0.2378
Chile	1995	Spinal cord lesion below neck level	0.2201
Chile	2000	Spinal cord lesion below neck level	0.2037
Chile	2005	Spinal cord lesion below neck level	0.191
Chile	2010	Spinal cord lesion below neck level	0.1809
Chile	2017	Spinal cord lesion below neck level	0.1777
Chile	1990	Drowning and nonfatal submersion	0.2471
Chile	1995	Drowning and nonfatal submersion	0.2471
Chile	2000	Drowning and nonfatal submersion	0.2471
Chile	2005	Drowning and nonfatal submersion	0.2471
Chile	2010	Drowning and nonfatal submersion	0.2471
Chile	2017	Drowning and nonfatal submersion	0.2471
Chile	1990	Asphyxiation	0.2471
Chile	1995	Asphyxiation	0.2471
Chile	2000	Asphyxiation	0.2471
Chile	2005	Asphyxiation	0.2471
Chile	2010	Asphyxiation	0.2471
Chile	2017	Asphyxiation	0.2471
Chile	1990	Crush injury	0.1325
Chile	1995	Crush injury	0.1325
Chile	2000	Crush injury	0.1325
Chile	2005	Crush injury	0.1325
Chile	2010	Crush injury	0.1325
Chile	2017	Crush injury	0.1325
Chile	1990	Nerve injury	0.1129
Chile	1995	Nerve injury	0.1129
Chile	2000	Nerve injury	0.1129
Chile	2005	Nerve injury	0.1129
Chile	2010	Nerve injury	0.1129
Chile	2017	Nerve injury	0.1129
Chile	1990	Injury to eyes	0.0165
Chile	1995	Injury to eyes	0.0165
Chile	2000	Injury to eyes	0.0165
Chile	2005	Injury to eyes	0.0165
Chile	2010	Injury to eyes	0.0165
Chile	2017	Injury to eyes	0.0165
Chile	1990	Amputation of lower limb, unilateral	0.0711
Chile	1995	Amputation of lower limb, unilateral	0.0616
Chile	2000	Amputation of lower limb, unilateral	0.0529
Chile	2005	Amputation of lower limb, unilateral	0.0461
Chile	2010	Amputation of lower limb, unilateral	0.0407
Chile	2017	Amputation of lower limb, unilateral	0.039
Chile	1990	Open wound(s)	0.011
Chile	1995	Open wound(s)	0.011
Chile	2000	Open wound(s)	0.011
Chile	2005	Open wound(s)	0.011
Chile	2010	Open wound(s)	0.011
Chile	2017	Open wound(s)	0.011
Chile	1990	Poisoning requiring urgent care	0.1628
Chile	1995	Poisoning requiring urgent care	0.1628
Chile	2000	Poisoning requiring urgent care	0.1628
Chile	2005	Poisoning requiring urgent care	0.1628
Chile	2010	Poisoning requiring urgent care	0.1628
Chile	2017	Poisoning requiring urgent care	0.1628
Chile	1990	Severe chest injury	0.0474
Chile	1995	Severe chest injury	0.0474
Chile	2000	Severe chest injury	0.0474
Chile	2005	Severe chest injury	0.0474
Chile	2010	Severe chest injury	0.0474
Chile	2017	Severe chest injury	0.0474
Chile	1990	Internal hemorrhage in abdomen and pelvis	0.1143
Chile	1995	Internal hemorrhage in abdomen and pelvis	0.1143
Chile	2000	Internal hemorrhage in abdomen and pelvis	0.1143
Chile	2005	Internal hemorrhage in abdomen and pelvis	0.1143
Chile	2010	Internal hemorrhage in abdomen and pelvis	0.1143
Chile	2017	Internal hemorrhage in abdomen and pelvis	0.1143
Chile	1990	Contusion in any part of the body	0.0075
Chile	1995	Contusion in any part of the body	0.0075
Chile	2000	Contusion in any part of the body	0.0075
Chile	2005	Contusion in any part of the body	0.0075
Chile	2010	Contusion in any part of the body	0.0075
Chile	2017	Contusion in any part of the body	0.0075
Chile	1990	Effect of different environmental factors	0.049
Chile	1995	Effect of different environmental factors	0.049
Chile	2000	Effect of different environmental factors	0.049
Chile	2005	Effect of different environmental factors	0.049
Chile	2010	Effect of different environmental factors	0.049
Chile	2017	Effect of different environmental factors	0.049
Chile	1990	Complications following therapeutic procedures	0.0506
Chile	1995	Complications following therapeutic procedures	0.0506
Chile	2000	Complications following therapeutic procedures	0.0506
Chile	2005	Complications following therapeutic procedures	0.0506
Chile	2010	Complications following therapeutic procedures	0.0506
Chile	2017	Complications following therapeutic procedures	0.0506
Chile	1990	Multiple fractures, dislocations, crashes, wounds, sprains, and strains	0.14
Chile	1995	Multiple fractures, dislocations, crashes, wounds, sprains, and strains	0.1159
Chile	2000	Multiple fractures, dislocations, crashes, wounds, sprains, and strains	0.0934
Chile	2005	Multiple fractures, dislocations, crashes, wounds, sprains, and strains	0.076
Chile	2010	Multiple fractures, dislocations, crashes, wounds, sprains, and strains	0.0621
Chile	2017	Multiple fractures, dislocations, crashes, wounds, sprains, and strains	0.0577
Chile	1990	Amputation of upper limb, unilateral	0.0579
Chile	1995	Amputation of upper limb, unilateral	0.0523
Chile	2000	Amputation of upper limb, unilateral	0.0472
Chile	2005	Amputation of upper limb, unilateral	0.0432
Chile	2010	Amputation of upper limb, unilateral	0.04
Chile	2017	Amputation of upper limb, unilateral	0.039
Chile	1990	Amputation of thumb	0.0114
Chile	1995	Amputation of thumb	0.0114
Chile	2000	Amputation of thumb	0.0114
Chile	2005	Amputation of thumb	0.0114
Chile	2010	Amputation of thumb	0.0114
Chile	2017	Amputation of thumb	0.0114
Chile	1990	Amputation of toe/toes	0.0059
Chile	1995	Amputation of toe/toes	0.0059
Chile	2000	Amputation of toe/toes	0.0059
Chile	2005	Amputation of toe/toes	0.0059
Chile	2010	Amputation of toe/toes	0.0059
Chile	2017	Amputation of toe/toes	0.0059
Chile	1990	Burns, <20% total burned surface area without lower airway burns	0.0163
Chile	1995	Burns, <20% total burned surface area without lower airway burns	0.0163
Chile	2000	Burns, <20% total burned surface area without lower airway burns	0.0163
Chile	2005	Burns, <20% total burned surface area without lower airway burns	0.0163
Chile	2010	Burns, <20% total burned surface area without lower airway burns	0.0163
Chile	2017	Burns, <20% total burned surface area without lower airway burns	0.0163
Chile	1990	Burns, >=20% total burned surface area or >= 10% burned surface area if head/neck or hands/wrist involved w/o lower airway burns	0.2113
Chile	1995	Burns, >=20% total burned surface area or >= 10% burned surface area if head/neck or hands/wrist involved w/o lower airway burns	0.1888
Chile	2000	Burns, >=20% total burned surface area or >= 10% burned surface area if head/neck or hands/wrist involved w/o lower airway burns	0.1678
Chile	2005	Burns, >=20% total burned surface area or >= 10% burned surface area if head/neck or hands/wrist involved w/o lower airway burns	0.1516
Chile	2010	Burns, >=20% total burned surface area or >= 10% burned surface area if head/neck or hands/wrist involved w/o lower airway burns	0.1387
Chile	2017	Burns, >=20% total burned surface area or >= 10% burned surface area if head/neck or hands/wrist involved w/o lower airway burns	0.1346
Uruguay	1990	Amputation of lower limbs, bilateral	0.181
Uruguay	1995	Amputation of lower limbs, bilateral	0.1716
Uruguay	2000	Amputation of lower limbs, bilateral	0.1578
Uruguay	2005	Amputation of lower limbs, bilateral	0.1526
Uruguay	2010	Amputation of lower limbs, bilateral	0.1456
Uruguay	2017	Amputation of lower limbs, bilateral	0.1349
Uruguay	1990	Lower airway burns	0.2252
Uruguay	1995	Lower airway burns	0.2252
Uruguay	2000	Lower airway burns	0.2252
Uruguay	2005	Lower airway burns	0.2252
Uruguay	2010	Lower airway burns	0.2252
Uruguay	2017	Lower airway burns	0.2252
Uruguay	1990	Dislocation of hip	0.0159
Uruguay	1995	Dislocation of hip	0.0159
Uruguay	2000	Dislocation of hip	0.0159
Uruguay	2005	Dislocation of hip	0.0159
Uruguay	2010	Dislocation of hip	0.0159
Uruguay	2017	Dislocation of hip	0.0159
Uruguay	1990	Dislocation of knee	0.1134
Uruguay	1995	Dislocation of knee	0.1134
Uruguay	2000	Dislocation of knee	0.1134
Uruguay	2005	Dislocation of knee	0.1134
Uruguay	2010	Dislocation of knee	0.1134
Uruguay	2017	Dislocation of knee	0.1134
Uruguay	1990	Dislocation of shoulder	0.062
Uruguay	1995	Dislocation of shoulder	0.062
Uruguay	2000	Dislocation of shoulder	0.062
Uruguay	2005	Dislocation of shoulder	0.062
Uruguay	2010	Dislocation of shoulder	0.062
Uruguay	2017	Dislocation of shoulder	0.062
Uruguay	1990	Muscle and tendon injuries, including sprains and strains lesser dislocations	0.0075
Uruguay	1995	Muscle and tendon injuries, including sprains and strains lesser dislocations	0.0075
Uruguay	2000	Muscle and tendon injuries, including sprains and strains lesser dislocations	0.0075
Uruguay	2005	Muscle and tendon injuries, including sprains and strains lesser dislocations	0.0075
Uruguay	2010	Muscle and tendon injuries, including sprains and strains lesser dislocations	0.0075
Uruguay	2017	Muscle and tendon injuries, including sprains and strains lesser dislocations	0.0075
Uruguay	1990	Fracture of clavicle, scapula, or humerus	0.0349
Uruguay	1995	Fracture of clavicle, scapula, or humerus	0.0349
Uruguay	2000	Fracture of clavicle, scapula, or humerus	0.0349
Uruguay	2005	Fracture of clavicle, scapula, or humerus	0.0349
Uruguay	2010	Fracture of clavicle, scapula, or humerus	0.0349
Uruguay	2017	Fracture of clavicle, scapula, or humerus	0.0349
Uruguay	1990	Fracture of face bones	0.0669
Uruguay	1995	Fracture of face bones	0.0669
Uruguay	2000	Fracture of face bones	0.0669
Uruguay	2005	Fracture of face bones	0.0669
Uruguay	2010	Fracture of face bones	0.0669
Uruguay	2017	Fracture of face bones	0.0669
Uruguay	1990	Fracture of foot bones except ankle	0.0264
Uruguay	1995	Fracture of foot bones except ankle	0.0264
Uruguay	2000	Fracture of foot bones except ankle	0.0264
Uruguay	2005	Fracture of foot bones except ankle	0.0264
Uruguay	2010	Fracture of foot bones except ankle	0.0264
Uruguay	2017	Fracture of foot bones except ankle	0.0264
Uruguay	1990	Fracture of hand (wrist and other distal part of hand)	0.0138
Uruguay	1995	Fracture of hand (wrist and other distal part of hand)	0.0138
Uruguay	2000	Fracture of hand (wrist and other distal part of hand)	0.0138
Uruguay	2005	Fracture of hand (wrist and other distal part of hand)	0.0138
Uruguay	2010	Fracture of hand (wrist and other distal part of hand)	0.0138
Uruguay	2017	Fracture of hand (wrist and other distal part of hand)	0.0138
Uruguay	1990	Fracture of hip	0.134
Uruguay	1995	Fracture of hip	0.1216
Uruguay	2000	Fracture of hip	0.1034
Uruguay	2005	Fracture of hip	0.0964
Uruguay	2010	Fracture of hip	0.0872
Uruguay	2017	Fracture of hip	0.073
Uruguay	1990	Amputation of upper limbs, bilateral	0.1665
Uruguay	1995	Amputation of upper limbs, bilateral	0.1536
Uruguay	2000	Amputation of upper limbs, bilateral	0.1348
Uruguay	2005	Amputation of upper limbs, bilateral	0.1277
Uruguay	2010	Amputation of upper limbs, bilateral	0.1181
Uruguay	2017	Amputation of upper limbs, bilateral	0.1035
Uruguay	1990	Fracture of patella, tibia or fibula, or ankle	0.0554
Uruguay	1995	Fracture of patella, tibia or fibula, or ankle	0.0554
Uruguay	2000	Fracture of patella, tibia or fibula, or ankle	0.0554
Uruguay	2005	Fracture of patella, tibia or fibula, or ankle	0.0554
Uruguay	2010	Fracture of patella, tibia or fibula, or ankle	0.0554
Uruguay	2017	Fracture of patella, tibia or fibula, or ankle	0.0554
Uruguay	1990	Fracture of pelvis	0.1823
Uruguay	1995	Fracture of pelvis	0.1823
Uruguay	2000	Fracture of pelvis	0.1823
Uruguay	2005	Fracture of pelvis	0.1823
Uruguay	2010	Fracture of pelvis	0.1823
Uruguay	2017	Fracture of pelvis	0.1823
Uruguay	1990	Fracture of radius and/or ulna	0.0435
Uruguay	1995	Fracture of radius and/or ulna	0.0435
Uruguay	2000	Fracture of radius and/or ulna	0.0435
Uruguay	2005	Fracture of radius and/or ulna	0.0435
Uruguay	2010	Fracture of radius and/or ulna	0.0435
Uruguay	2017	Fracture of radius and/or ulna	0.0435
Uruguay	1990	Fracture of skull	0.0714
Uruguay	1995	Fracture of skull	0.0714
Uruguay	2000	Fracture of skull	0.0714
Uruguay	2005	Fracture of skull	0.0714
Uruguay	2010	Fracture of skull	0.0714
Uruguay	2017	Fracture of skull	0.0714
Uruguay	1990	Fracture of sternum and/or fracture of one or more ribs	0.1027
Uruguay	1995	Fracture of sternum and/or fracture of one or more ribs	0.1027
Uruguay	2000	Fracture of sternum and/or fracture of one or more ribs	0.1027
Uruguay	2005	Fracture of sternum and/or fracture of one or more ribs	0.1027
Uruguay	2010	Fracture of sternum and/or fracture of one or more ribs	0.1027
Uruguay	2017	Fracture of sternum and/or fracture of one or more ribs	0.1027
Uruguay	1990	Fracture of vertebral column	0.1106
Uruguay	1995	Fracture of vertebral column	0.1106
Uruguay	2000	Fracture of vertebral column	0.1106
Uruguay	2005	Fracture of vertebral column	0.1106
Uruguay	2010	Fracture of vertebral column	0.1106
Uruguay	2017	Fracture of vertebral column	0.1106
Uruguay	1990	Fracture of femur, other than femoral neck	0.042
Uruguay	1995	Fracture of femur, other than femoral neck	0.042
Uruguay	2000	Fracture of femur, other than femoral neck	0.042
Uruguay	2005	Fracture of femur, other than femoral neck	0.042
Uruguay	2010	Fracture of femur, other than femoral neck	0.042
Uruguay	2017	Fracture of femur, other than femoral neck	0.042
Uruguay	1990	Minor TBI	0.1319
Uruguay	1995	Minor TBI	0.1319
Uruguay	2000	Minor TBI	0.1319
Uruguay	2005	Minor TBI	0.1319
Uruguay	2010	Minor TBI	0.1319
Uruguay	2017	Minor TBI	0.1319
Uruguay	1990	Moderate TBI	0.1637
Uruguay	1995	Moderate TBI	0.1637
Uruguay	2000	Moderate TBI	0.1637
Uruguay	2005	Moderate TBI	0.1637
Uruguay	2010	Moderate TBI	0.1637
Uruguay	2017	Moderate TBI	0.1637
Uruguay	1990	Amputation of fingers (excluding thumb)	0.0047
Uruguay	1995	Amputation of fingers (excluding thumb)	0.0047
Uruguay	2000	Amputation of fingers (excluding thumb)	0.0047

Uruguay	2005	Amputation of fingers (excluding thumb)	0.0047
Uruguay	2010	Amputation of fingers (excluding thumb)	0.0047
Uruguay	2017	Amputation of fingers (excluding thumb)	0.0047
Uruguay	1990	Spinal cord lesion at neck level	0.4382
Uruguay	1995	Spinal cord lesion at neck level	0.4339
Uruguay	2000	Spinal cord lesion at neck level	0.4276
Uruguay	2005	Spinal cord lesion at neck level	0.4252
Uruguay	2010	Spinal cord lesion at neck level	0.422
Uruguay	2017	Spinal cord lesion at neck level	0.4171
Uruguay	1990	Spinal cord lesion below neck level	0.2334
Uruguay	1995	Spinal cord lesion below neck level	0.2243
Uruguay	2000	Spinal cord lesion below neck level	0.211
Uruguay	2005	Spinal cord lesion below neck level	0.206
Uruguay	2010	Spinal cord lesion below neck level	0.1992
Uruguay	2017	Spinal cord lesion below neck level	0.1889
Uruguay	1990	Drowning and nonfatal submersion	0.2471
Uruguay	1995	Drowning and nonfatal submersion	0.2471
Uruguay	2000	Drowning and nonfatal submersion	0.2471
Uruguay	2005	Drowning and nonfatal submersion	0.2471
Uruguay	2010	Drowning and nonfatal submersion	0.2471
Uruguay	2017	Drowning and nonfatal submersion	0.2471
Uruguay	1990	Asphyxiation	0.2471
Uruguay	1995	Asphyxiation	0.2471
Uruguay	2000	Asphyxiation	0.2471
Uruguay	2005	Asphyxiation	0.2471
Uruguay	2010	Asphyxiation	0.2471
Uruguay	2017	Asphyxiation	0.2471
Uruguay	1990	Crush injury	0.1325
Uruguay	1995	Crush injury	0.1325
Uruguay	2000	Crush injury	0.1325
Uruguay	2005	Crush injury	0.1325
Uruguay	2010	Crush injury	0.1325
Uruguay	2017	Crush injury	0.1325
Uruguay	1990	Nerve injury	0.1129
Uruguay	1995	Nerve injury	0.1129
Uruguay	2000	Nerve injury	0.1129
Uruguay	2005	Nerve injury	0.1129
Uruguay	2010	Nerve injury	0.1129
Uruguay	2017	Nerve injury	0.1129
Uruguay	1990	Injury to eyes	0.0165
Uruguay	1995	Injury to eyes	0.0165
Uruguay	2000	Injury to eyes	0.0165
Uruguay	2005	Injury to eyes	0.0165
Uruguay	2010	Injury to eyes	0.0165
Uruguay	2017	Injury to eyes	0.0165
Uruguay	1990	Amputation of lower limb, unilateral	0.0687
Uruguay	1995	Amputation of lower limb, unilateral	0.0639
Uruguay	2000	Amputation of lower limb, unilateral	0.0568
Uruguay	2005	Amputation of lower limb, unilateral	0.0541
Uruguay	2010	Amputation of lower limb, unilateral	0.0505
Uruguay	2017	Amputation of lower limb, unilateral	0.0449
Uruguay	1990	Open wound(s)	0.011
Uruguay	1995	Open wound(s)	0.011
Uruguay	2000	Open wound(s)	0.011
Uruguay	2005	Open wound(s)	0.011
Uruguay	2010	Open wound(s)	0.011
Uruguay	2017	Open wound(s)	0.011
Uruguay	1990	Poisoning requiring urgent care	0.1628
Uruguay	1995	Poisoning requiring urgent care	0.1628
Uruguay	2000	Poisoning requiring urgent care	0.1628
Uruguay	2005	Poisoning requiring urgent care	0.1628
Uruguay	2010	Poisoning requiring urgent care	0.1628
Uruguay	2017	Poisoning requiring urgent care	0.1628
Uruguay	1990	Severe chest injury	0.0474
Uruguay	1995	Severe chest injury	0.0474
Uruguay	2000	Severe chest injury	0.0474
Uruguay	2005	Severe chest injury	0.0474
Uruguay	2010	Severe chest injury	0.0474
Uruguay	2017	Severe chest injury	0.0474
Uruguay	1990	Internal hemorrhage in abdomen and pelvis	0.1143
Uruguay	1995	Internal hemorrhage in abdomen and pelvis	0.1143
Uruguay	2000	Internal hemorrhage in abdomen and pelvis	0.1143
Uruguay	2005	Internal hemorrhage in abdomen and pelvis	0.1143
Uruguay	2010	Internal hemorrhage in abdomen and pelvis	0.1143
Uruguay	2017	Internal hemorrhage in abdomen and pelvis	0.1143
Uruguay	1990	Contusion in any part of the body	0.0075
Uruguay	1995	Contusion in any part of the body	0.0075
Uruguay	2000	Contusion in any part of the body	0.0075
Uruguay	2005	Contusion in any part of the body	0.0075
Uruguay	2010	Contusion in any part of the body	0.0075
Uruguay	2017	Contusion in any part of the body	0.0075
Uruguay	1990	Effect of different environmental factors	0.049
Uruguay	1995	Effect of different environmental factors	0.049
Uruguay	2000	Effect of different environmental factors	0.049
Uruguay	2005	Effect of different environmental factors	0.049
Uruguay	2010	Effect of different environmental factors	0.049
Uruguay	2017	Effect of different environmental factors	0.049
Uruguay	1990	Complications following therapeutic procedures	0.0506
Uruguay	1995	Complications following therapeutic procedures	0.0506
Uruguay	2000	Complications following therapeutic procedures	0.0506
Uruguay	2005	Complications following therapeutic procedures	0.0506
Uruguay	2010	Complications following therapeutic procedures	0.0506
Uruguay	2017	Complications following therapeutic procedures	0.0506
Uruguay	1990	Multiple fractures, dislocations, crashes, wounds, sprains, and strains	0.134
Uruguay	1995	Multiple fractures, dislocations, crashes, wounds, sprains, and strains	0.1216
Uruguay	2000	Multiple fractures, dislocations, crashes, wounds, sprains, and strains	0.1034
Uruguay	2005	Multiple fractures, dislocations, crashes, wounds, sprains, and strains	0.0964
Uruguay	2010	Multiple fractures, dislocations, crashes, wounds, sprains, and strains	0.0872
Uruguay	2017	Multiple fractures, dislocations, crashes, wounds, sprains, and strains	0.073
Uruguay	1990	Amputation of upper limb, unilateral	0.0565
Uruguay	1995	Amputation of upper limb, unilateral	0.0537
Uruguay	2000	Amputation of upper limb, unilateral	0.0495
Uruguay	2005	Amputation of upper limb, unilateral	0.0479
Uruguay	2010	Amputation of upper limb, unilateral	0.0458
Uruguay	2017	Amputation of upper limb, unilateral	0.0425
Uruguay	1990	Amputation of thumb	0.0114
Uruguay	1995	Amputation of thumb	0.0114
Uruguay	2000	Amputation of thumb	0.0114
Uruguay	2005	Amputation of thumb	0.0114
Uruguay	2010	Amputation of thumb	0.0114
Uruguay	2017	Amputation of thumb	0.0114
Uruguay	1990	Amputation of toe/toes	0.0059
Uruguay	1995	Amputation of toe/toes	0.0059
Uruguay	2000	Amputation of toe/toes	0.0059
Uruguay	2005	Amputation of toe/toes	0.0059
Uruguay	2010	Amputation of toe/toes	0.0059
Uruguay	2017	Amputation of toe/toes	0.0059
Uruguay	1990	Burns, <20% total burned surface area without lower airway burns	0.0163
Uruguay	1995	Burns, <20% total burned surface area without lower airway burns	0.0163
Uruguay	2000	Burns, <20% total burned surface area without lower airway burns	0.0163
Uruguay	2005	Burns, <20% total burned surface area without lower airway burns	0.0163
Uruguay	2010	Burns, <20% total burned surface area without lower airway burns	0.0163
Uruguay	2017	Burns, <20% total burned surface area without lower airway burns	0.0163
Uruguay	1990	Burns, >=20% total burned surface area or >= 10% burned surface area if head/neck or hands/wrist involved w/o lower airway burns	0.2057
Uruguay	1995	Burns, >=20% total burned surface area or >= 10% burned surface area if head/neck or hands/wrist involved w/o lower airway burns	0.1941
Uruguay	2000	Burns, >=20% total burned surface area or >= 10% burned surface area if head/neck or hands/wrist involved w/o lower airway burns	0.1771
Uruguay	2005	Burns, >=20% total burned surface area or >= 10% burned surface area if head/neck or hands/wrist involved w/o lower airway burns	0.1707
Uruguay	2010	Burns, >=20% total burned surface area or >= 10% burned surface area if head/neck or hands/wrist involved w/o lower airway burns	0.1621
Uruguay	2017	Burns, >=20% total burned surface area or >= 10% burned surface area if head/neck or hands/wrist involved w/o lower airway burns	0.1489
Canada	1990	Amputation of lower limbs, bilateral	0.1232
Canada	1995	Amputation of lower limbs, bilateral	0.1232
Canada	2000	Amputation of lower limbs, bilateral	0.1232
Canada	2005	Amputation of lower limbs, bilateral	0.1232
Canada	2010	Amputation of lower limbs, bilateral	0.1232
Canada	2017	Amputation of lower limbs, bilateral	0.1232
Canada	1990	Lower airway burns	0.2252
Canada	1995	Lower airway burns	0.2252
Canada	2000	Lower airway burns	0.2252
Canada	2005	Lower airway burns	0.2252
Canada	2010	Lower airway burns	0.2252
Canada	2017	Lower airway burns	0.2252
Canada	1990	Dislocation of hip	0.0159
Canada	1995	Dislocation of hip	0.0159
Canada	2000	Dislocation of hip	0.0159
Canada	2005	Dislocation of hip	0.0159
Canada	2010	Dislocation of hip	0.0159
Canada	2017	Dislocation of hip	0.0159
Canada	1990	Dislocation of knee	0.1134
Canada	1995	Dislocation of knee	0.1134
Canada	2000	Dislocation of knee	0.1134
Canada	2005	Dislocation of knee	0.1134
Canada	2010	Dislocation of knee	0.1134
Canada	2017	Dislocation of knee	0.1134
Canada	1990	Dislocation of shoulder	0.062
Canada	1995	Dislocation of shoulder	0.062
Canada	2000	Dislocation of shoulder	0.062
Canada	2005	Dislocation of shoulder	0.062
Canada	2010	Dislocation of shoulder	0.062
Canada	2017	Dislocation of shoulder	0.062
Canada	1990	Muscle and tendon injuries, including sprains and strains lesser dislocations	0.0075
Canada	1995	Muscle and tendon injuries, including sprains and strains lesser dislocations	0.0075
Canada	2000	Muscle and tendon injuries, including sprains and strains lesser dislocations	0.0075
Canada	2005	Muscle and tendon injuries, including sprains and strains lesser dislocations	0.0075
Canada	2010	Muscle and tendon injuries, including sprains and strains lesser dislocations	0.0075
Canada	2017	Muscle and tendon injuries, including sprains and strains lesser dislocations	0.0075
Canada	1990	Fracture of clavicle, scapula, or humerus	0.0349
Canada	1995	Fracture of clavicle, scapula, or humerus	0.0349
Canada	2000	Fracture of clavicle, scapula, or humerus	0.0349
Canada	2005	Fracture of clavicle, scapula, or humerus	0.0349
Canada	2010	Fracture of clavicle, scapula, or humerus	0.0349
Canada	2017	Fracture of clavicle, scapula, or humerus	0.0349
Canada	1990	Fracture of face bones	0.0669
Canada	1995	Fracture of face bones	0.0669
Canada	2000	Fracture of face bones	0.0669
Canada	2005	Fracture of face bones	0.0669
Canada	2010	Fracture of face bones	0.0669
Canada	2017	Fracture of face bones	0.0669
Canada	1990	Fracture of foot bones except ankle	0.0264
Canada	1995	Fracture of foot bones except ankle	0.0264
Canada	2000	Fracture of foot bones except ankle	0.0264
Canada	2005	Fracture of foot bones except ankle	0.0264
Canada	2010	Fracture of foot bones except ankle	0.0264
Canada	2017	Fracture of foot bones except ankle	0.0264
Canada	1990	Fracture of hand (wrist and other distal part of hand)	0.0138
Canada	1995	Fracture of hand (wrist and other distal part of hand)	0.0138
Canada	2000	Fracture of hand (wrist and other distal part of hand)	0.0138
Canada	2005	Fracture of hand (wrist and other distal part of hand)	0.0138
Canada	2010	Fracture of hand (wrist and other distal part of hand)	0.0138
Canada	2017	Fracture of hand (wrist and other distal part of hand)	0.0138
Canada	1990	Fracture of hip	0.0577
Canada	1995	Fracture of hip	0.0577
Canada	2000	Fracture of hip	0.0577
Canada	2005	Fracture of hip	0.0577
Canada	2010	Fracture of hip	0.0577
Canada	2017	Fracture of hip	0.0577
Canada	1990	Amputation of upper limbs, bilateral	0.0876
Canada	1995	Amputation of upper limbs, bilateral	0.0876
Canada	2000	Amputation of upper limbs, bilateral	0.0876
Canada	2005	Amputation of upper limbs, bilateral	0.0876
Canada	2010	Amputation of upper limbs, bilateral	0.0876
Canada	2017	Amputation of upper limbs, bilateral	0.0876
Canada	1990	Fracture of patella, tibia or fibula, or ankle	0.0554
Canada	1995	Fracture of patella, tibia or fibula, or ankle	0.0554
Canada	2000	Fracture of patella, tibia or fibula, or ankle	0.0554
Canada	2005	Fracture of patella, tibia or fibula, or ankle	0.0554
Canada	2010	Fracture of patella, tibia or fibula, or ankle	0.0554
Canada	2017	Fracture of patella, tibia or fibula, or ankle	0.0554
Canada	1990	Fracture of pelvis	0.1823
Canada	1995	Fracture of pelvis	0.1823
Canada	2000	Fracture of pelvis	0.1823
Canada	2005	Fracture of pelvis	0.1823
Canada	2010	Fracture of pelvis	0.1823
Canada	2017	Fracture of pelvis	0.1823
Canada	1990	Fracture of radius and/or ulna	0.0435
Canada	1995	Fracture of radius and/or ulna	0.0435
Canada	2000	Fracture of radius and/or ulna	0.0435
Canada	2005	Fracture of radius and/or ulna	0.0435
Canada	2010	Fracture of radius and/or ulna	0.0435
Canada	2017	Fracture of radius and/or ulna	0.0435
Canada	1990	Fracture of skull	0.0714
Canada	1995	Fracture of skull	0.0714
Canada	2000	Fracture of skull	0.0714
Canada	2005	Fracture of skull	0.0714
Canada	2010	Fracture of skull	0.0714
Canada	2017	Fracture of skull	0.0714
Canada	1990	Fracture of sternum and/or fracture of one or more ribs	0.1027
Canada	1995	Fracture of sternum and/or fracture of one or more ribs	0.1027
Canada	2000	Fracture of sternum and/or fracture of one or more ribs	0.1027
Canada	2005	Fracture of sternum and/or fracture of one or more ribs	0.1027
Canada	2010	Fracture of sternum and/or fracture of one or more ribs	0.1027
Canada	2017	Fracture of sternum and/or fracture of one or more ribs	0.1027
Canada	1990	Fracture of vertebral column	0.1106
Canada	1995	Fracture of vertebral column	0.1106
Canada	2000	Fracture of vertebral column	0.1106
Canada	2005	Fracture of vertebral column	0.1106
Canada	2010	Fracture of vertebral column	0.1106
Canada	2017	Fracture of vertebral column	0.1106
Canada	1990	Fracture of femur, other than femoral neck	0.042
Canada	1995	Fracture of femur, other than femoral neck	0.042
Canada	2000	Fracture of femur, other than femoral neck	0.042
Canada	2005	Fracture of femur, other than femoral neck	0.042
Canada	2010	Fracture of femur, other than femoral neck	0.042
Canada	2017	Fracture of femur, other than femoral neck	0.042
Canada	1990	Minor TBI	0.1319
Canada	1995	Minor TBI	0.1319
Canada	2000	Minor TBI	0.1319
Canada	2005	Minor TBI	0.1319
Canada	2010	Minor TBI	0.1319
Canada	2017	Minor TBI	0.1319
Canada	1990	Moderate TBI	0.1637
Canada	1995	Moderate TBI	0.1637
Canada	2000	Moderate TBI	0.1637
Canada	2005	Moderate TBI	0.1637
Canada	2010	Moderate TBI	0.1637
Canada	2017	Moderate TBI	0.1637
Canada	1990	Amputation of fingers (excluding thumb)	0.0047
Canada	1995	Amputation of fingers (excluding thumb)	0.0047
Canada	2000	Amputation of fingers (excluding thumb)	0.0047
Canada	2005	Amputation of fingers (excluding thumb)	0.0047
Canada	2010	Amputation of fingers (excluding thumb)	0.0047
Canada	2017	Amputation of fingers (excluding thumb)	0.0047
Canada	1990	Spinal cord lesion at neck level	0.4118
Canada	1995	Spinal cord lesion at neck level	0.4118
Canada	2000	Spinal cord lesion at neck level	0.4118
Canada	2005	Spinal cord lesion at neck level	0.4118
Canada	2010	Spinal cord lesion at neck level	0.4118
Canada	2017	Spinal cord lesion at neck level	0.4118
Canada	1990	Spinal cord lesion below neck level	0.1777
Canada	1995	Spinal cord lesion below neck level	0.1777
Canada	2000	Spinal cord lesion below neck level	0.1777
Canada	2005	Spinal cord lesion below neck level	0.1777
Canada	2010	Spinal cord lesion below neck level	0.1777
Canada	2017	Spinal cord lesion below neck level	0.1777
Canada	1990	Drowning and nonfatal submersion	0.2471
Canada	1995	Drowning and nonfatal submersion	0.2471
Canada	2000	Drowning and nonfatal submersion	0.2471
Canada	2005	Drowning and nonfatal submersion	0.2471
Canada	2010	Drowning and nonfatal submersion	0.2471
Canada	2017	Drowning and nonfatal submersion	0.2471
Canada	1990	Asphyxiation	0.2471
Canada	1995	Asphyxiation	0.2471
Canada	2000	Asphyxiation	0.2471
Canada	2005	Asphyxiation	0.2471
Canada	2010	Asphyxiation	0.2471
Canada	2017	Asphyxiation	0.2471
Canada	1990	Crush injury	0.1325
Canada	1995	Crush injury	0.1325
Canada	2000	Crush injury	0.1325
Canada	2005	Crush injury	0.1325
Canada	2010	Crush injury	0.1325
Canada	2017	Crush injury	0.1325
Canada	1990	Nerve injury	0.1129
Canada	1995	Nerve injury	0.1129
Canada	2000	Nerve injury	0.1129
Canada	2005	Nerve injury	0.1129
Canada	2010	Nerve injury	0.1129
Canada	2017	Nerve injury	0.1129
Canada	1990	Injury to eyes	0.0165
Canada	1995	Injury to eyes	0.0165
Canada	2000	Injury to eyes	0.0165
Canada	2005	Injury to eyes	0.0165
Canada	2010	Injury to eyes	0.0165
Canada	2017	Injury to eyes	0.0165
Canada	1990	Amputation of lower limb, unilateral	0.039
Canada	1995	Amputation of lower limb, unilateral	0.039
Canada	2000	Amputation of lower limb, unilateral	0.039
Canada	2005	Amputation of lower limb, unilateral	0.039
Canada	2010	Amputation of lower limb, unilateral	0.039
Canada	2017	Amputation of lower limb, unilateral	0.039
Canada	1990	Open wound(s)	0.011
Canada	1995	Open wound(s)	0.011
Canada	2000	Open wound(s)	0.011
Canada	2005	Open wound(s)	0.011
Canada	2010	Open wound(s)	0.011
Canada	2017	Open wound(s)	0.011
Canada	1990	Poisoning requiring urgent care	0.1628
Canada	1995	Poisoning requiring urgent care	0.1628
Canada	2000	Poisoning requiring urgent care	0.1628
Canada	2005	Poisoning requiring urgent care	0.1628
Canada	2010	Poisoning requiring urgent care	0.1628
Canada	2017	Poisoning requiring urgent care	0.1628
Canada	1990	Severe chest injury	0.0474
Canada	1995	Severe chest injury	0.0474
Canada	2000	Severe chest injury	0.0474
Canada	2005	Severe chest injury	0.0474
Canada	2010	Severe chest injury	0.0474
Canada	2017	Severe chest injury	0.0474
Canada	1990	Internal hemorrhage in abdomen and pelvis	0.1143
Canada	1995	Internal hemorrhage in abdomen and pelvis	0.1143
Canada	2000	Internal hemorrhage in abdomen and pelvis	0.1143
Canada	2005	Internal hemorrhage in abdomen and pelvis	0.1143
Canada	2010	Internal hemorrhage in abdomen and pelvis	0.1143
Canada	2017	Internal hemorrhage in abdomen and pelvis	0.1143
Canada	1990	Contusion in any part of the body	0.0075
Canada	1995	Contusion in any part of the body	0.0075
Canada	2000	Contusion in any part of the body	0.0075
Canada	2005	Contusion in any part of the body	0.0075
Canada	2010	Contusion in any part of the body	0.0075
Canada	2017	Contusion in any part of the body	0.0075
Canada	1990	Effect of different environmental factors	0.049
Canada	1995	Effect of different environmental factors	0.049
Canada	2000	Effect of different environmental factors	0.049
Canada	2005	Effect of different environmental factors	0.049
Canada	2010	Effect of different environmental factors	0.049
Canada	2017	Effect of different environmental factors	0.049
Canada	1990	Complications following therapeutic procedures	0.0506
Canada	1995	Complications following therapeutic procedures	0.0506
Canada	2000	Complications following therapeutic procedures	0.0506
Canada	2005	Complications following therapeutic procedures	0.0506
Canada	2010	Complications following therapeutic procedures	0.0506
Canada	2017	Complications following therapeutic procedures	0.0506
Canada	1990	Multiple fractures, dislocations, crashes, wounds, sprains, and strains	0.0577
Canada	1995	Multiple fractures, dislocations, crashes, wounds, sprains, and strains	0.0577
Canada	2000	Multiple fractures, dislocations, crashes, wounds, sprains, and strains	0.0577
Canada	2005	Multiple fractures, dislocations, crashes, wounds, sprains, and strains	0.0577
Canada	2010	Multiple fractures, dislocations, crashes, wounds, sprains, and strains	0.0577
Canada	2017	Multiple fractures, dislocations, crashes, wounds, sprains, and strains	0.0577
Canada	1990	Amputation of upper limb, unilateral	0.039
Canada	1995	Amputation of upper limb, unilateral	0.039
Canada	2000	Amputation of upper limb, unilateral	0.039
Canada	2005	Amputation of upper limb, unilateral	0.039
Canada	2010	Amputation of upper limb, unilateral	0.039
Canada	2017	Amputation of upper limb, unilateral	0.039
Canada	1990	Amputation of thumb	0.0114
Canada	1995	Amputation of thumb	0.0114
Canada	2000	Amputation of thumb	0.0114
Canada	2005	Amputation of thumb	0.0114
Canada	2010	Amputation of thumb	0.0114
Canada	2017	Amputation of thumb	0.0114
Canada	1990	Amputation of toe/toes	0.0059
Canada	1995	Amputation of toe/toes	0.0059
Canada	2000	Amputation of toe/toes	0.0059
Canada	2005	Amputation of toe/toes	0.0059
Canada	2010	Amputation of toe/toes	0.0059
Canada	2017	Amputation of toe/toes	0.0059
Canada	1990	Burns, <20% total burned surface area without lower airway burns	0.0163
Canada	1995	Burns, <20% total burned surface area without lower airway burns	0.0163
Canada	2000	Burns, <20% total burned surface area without lower airway burns	0.0163
Canada	2005	Burns, <20% total burned surface area without lower airway burns	0.0163
Canada	2010	Burns, <20% total burned surface area without lower airway burns	0.0163
Canada	2017	Burns, <20% total burned surface area without lower airway burns	0.0163
Canada	1990	Burns, >=20% total burned surface area or >= 10% burned surface area if head/neck or hands/wrist involved w/o lower airway burns	0.1346
Canada	1995	Burns, >=20% total burned surface area or >= 10% burned surface area if head/neck or hands/wrist involved w/o lower airway burns	0.1346
Canada	2000	Burns, >=20% total burned surface area or >= 10% burned surface area if head/neck or hands/wrist involved w/o lower airway burns	0.1346
Canada	2005	Burns, >=20% total burned surface area or >= 10% burned surface area if head/neck or hands/wrist involved w/o lower airway burns	0.1346
Canada	2010	Burns, >=20% total burned surface area or >= 10% burned surface area if head/neck or hands/wrist involved w/o lower airway burns	0.1346
Canada	2017	Burns, >=20% total burned surface area or >= 10% burned surface area if head/neck or hands/wrist involved w/o lower airway burns	0.1346
Antigua and Barbuda	1990	Amputation of lower limbs, bilateral	0.184
Antigua and Barbuda	1995	Amputation of lower limbs, bilateral	0.1736
Antigua and Barbuda	2000	Amputation of lower limbs, bilateral	0.1645
Antigua and Barbuda	2005	Amputation of lower limbs, bilateral	0.1564
Antigua and Barbuda	2010	Amputation of lower limbs, bilateral	0.1468
Antigua and Barbuda	2017	Amputation of lower limbs, bilateral	0.1396
Antigua and Barbuda	1990	Lower airway burns	0.2252
Antigua and Barbuda	1995	Lower airway burns	0.2252
Antigua and Barbuda	2000	Lower airway burns	0.2252
Antigua and Barbuda	2005	Lower airway burns	0.2252
Antigua and Barbuda	2010	Lower airway burns	0.2252
Antigua and Barbuda	2017	Lower airway burns	0.2252
Antigua and Barbuda	1990	Dislocation of hip	0.0159
Antigua and Barbuda	1995	Dislocation of hip	0.0159
Antigua and Barbuda	2000	Dislocation of hip	0.0159
Antigua and Barbuda	2005	Dislocation of hip	0.0159
Antigua and Barbuda	2010	Dislocation of hip	0.0159
Antigua and Barbuda	2017	Dislocation of hip	0.0159
Antigua and Barbuda	1990	Dislocation of knee	0.1134
Antigua and Barbuda	1995	Dislocation of knee	0.1134
Antigua and Barbuda	2000	Dislocation of knee	0.1134
Antigua and Barbuda	2005	Dislocation of knee	0.1134
Antigua and Barbuda	2010	Dislocation of knee	0.1134
Antigua and Barbuda	2017	Dislocation of knee	0.1134
Antigua and Barbuda	1990	Dislocation of shoulder	0.062
Antigua and Barbuda	1995	Dislocation of shoulder	0.062
Antigua and Barbuda	2000	Dislocation of shoulder	0.062
Antigua and Barbuda	2005	Dislocation of shoulder	0.062
Antigua and Barbuda	2010	Dislocation of shoulder	0.062
Antigua and Barbuda	2017	Dislocation of shoulder	0.062
Antigua and Barbuda	1990	Muscle and tendon injuries, including sprains and strains lesser dislocations	0.0075
Antigua and Barbuda	1995	Muscle and tendon injuries, including sprains and strains lesser dislocations	0.0075
Antigua and Barbuda	2000	Muscle and tendon injuries, including sprains and strains lesser dislocations	0.0075
Antigua and Barbuda	2005	Muscle and tendon injuries, including sprains and strains lesser dislocations	0.0075
Antigua and Barbuda	2010	Muscle and tendon injuries, including sprains and strains lesser dislocations	0.0075
Antigua and Barbuda	2017	Muscle and tendon injuries, including sprains and strains lesser dislocations	0.0075
Antigua and Barbuda	1990	Fracture of clavicle, scapula, or humerus	0.0349
Antigua and Barbuda	1995	Fracture of clavicle, scapula, or humerus	0.0349
Antigua and Barbuda	2000	Fracture of clavicle, scapula, or humerus	0.0349
Antigua and Barbuda	2005	Fracture of clavicle, scapula, or humerus	0.0349
Antigua and Barbuda	2010	Fracture of clavicle, scapula, or humerus	0.0349
Antigua and Barbuda	2017	Fracture of clavicle, scapula, or humerus	0.0349
Antigua and Barbuda	1990	Fracture of face bones	0.0669
Antigua and Barbuda	1995	Fracture of face bones	0.0669

Antigua and Barbuda	2000	Fracture of face bones	0.0669
Antigua and Barbuda	2005	Fracture of face bones	0.0669
Antigua and Barbuda	2010	Fracture of face bones	0.0669
Antigua and Barbuda	2017	Fracture of face bones	0.0669
Antigua and Barbuda	1990	Fracture of foot bones except ankle	0.0264
Antigua and Barbuda	1995	Fracture of foot bones except ankle	0.0264
Antigua and Barbuda	2000	Fracture of foot bones except ankle	0.0264
Antigua and Barbuda	2005	Fracture of foot bones except ankle	0.0264
Antigua and Barbuda	2010	Fracture of foot bones except ankle	0.0264
Antigua and Barbuda	2017	Fracture of foot bones except ankle	0.0264
Antigua and Barbuda	1990	Fracture of hand (wrist and other distal part of hand)	0.0138
Antigua and Barbuda	1995	Fracture of hand (wrist and other distal part of hand)	0.0138
Antigua and Barbuda	2000	Fracture of hand (wrist and other distal part of hand)	0.0138
Antigua and Barbuda	2005	Fracture of hand (wrist and other distal part of hand)	0.0138
Antigua and Barbuda	2010	Fracture of hand (wrist and other distal part of hand)	0.0138
Antigua and Barbuda	2017	Fracture of hand (wrist and other distal part of hand)	0.0138
Antigua and Barbuda	1990	Fracture of hip	0.138
Antigua and Barbuda	1995	Fracture of hip	0.1242
Antigua and Barbuda	2000	Fracture of hip	0.1122
Antigua and Barbuda	2005	Fracture of hip	0.1015
Antigua and Barbuda	2010	Fracture of hip	0.0889
Antigua and Barbuda	2017	Fracture of hip	0.0793
Antigua and Barbuda	1990	Amputation of upper limbs, bilateral	0.1706
Antigua and Barbuda	1995	Amputation of upper limbs, bilateral	0.1564
Antigua and Barbuda	2000	Amputation of upper limbs, bilateral	0.1439
Antigua and Barbuda	2005	Amputation of upper limbs, bilateral	0.1329
Antigua and Barbuda	2010	Amputation of upper limbs, bilateral	0.1198
Antigua and Barbuda	2017	Amputation of upper limbs, bilateral	0.11
Antigua and Barbuda	1990	Fracture of patella, tibia or fibula, or ankle	0.0554
Antigua and Barbuda	1995	Fracture of patella, tibia or fibula, or ankle	0.0554
Antigua and Barbuda	2000	Fracture of patella, tibia or fibula, or ankle	0.0554
Antigua and Barbuda	2005	Fracture of patella, tibia or fibula, or ankle	0.0554
Antigua and Barbuda	2010	Fracture of patella, tibia or fibula, or ankle	0.0554
Antigua and Barbuda	2017	Fracture of patella, tibia or fibula, or ankle	0.0554
Antigua and Barbuda	1990	Fracture of pelvis	0.1823
Antigua and Barbuda	1995	Fracture of pelvis	0.1823
Antigua and Barbuda	2000	Fracture of pelvis	0.1823
Antigua and Barbuda	2005	Fracture of pelvis	0.1823
Antigua and Barbuda	2010	Fracture of pelvis	0.1823
Antigua and Barbuda	2017	Fracture of pelvis	0.1823
Antigua and Barbuda	1990	Fracture of radius and/or ulna	0.0435
Antigua and Barbuda	1995	Fracture of radius and/or ulna	0.0435
Antigua and Barbuda	2000	Fracture of radius and/or ulna	0.0435
Antigua and Barbuda	2005	Fracture of radius and/or ulna	0.0435
Antigua and Barbuda	2010	Fracture of radius and/or ulna	0.0435
Antigua and Barbuda	2017	Fracture of radius and/or ulna	0.0435
Antigua and Barbuda	1990	Fracture of skull	0.0714
Antigua and Barbuda	1995	Fracture of skull	0.0714
Antigua and Barbuda	2000	Fracture of skull	0.0714
Antigua and Barbuda	2005	Fracture of skull	0.0714
Antigua and Barbuda	2010	Fracture of skull	0.0714
Antigua and Barbuda	2017	Fracture of skull	0.0714
Antigua and Barbuda	1990	Fracture of sternum and/or fracture of one or more ribs	0.1027
Antigua and Barbuda	1995	Fracture of sternum and/or fracture of one or more ribs	0.1027
Antigua and Barbuda	2000	Fracture of sternum and/or fracture of one or more ribs	0.1027
Antigua and Barbuda	2005	Fracture of sternum and/or fracture of one or more ribs	0.1027
Antigua and Barbuda	2010	Fracture of sternum and/or fracture of one or more ribs	0.1027
Antigua and Barbuda	2017	Fracture of sternum and/or fracture of one or more ribs	0.1027
Antigua and Barbuda	1990	Fracture of vertebral column	0.1106
Antigua and Barbuda	1995	Fracture of vertebral column	0.1106
Antigua and Barbuda	2000	Fracture of vertebral column	0.1106
Antigua and Barbuda	2005	Fracture of vertebral column	0.1106
Antigua and Barbuda	2010	Fracture of vertebral column	0.1106
Antigua and Barbuda	2017	Fracture of vertebral column	0.1106
Antigua and Barbuda	1990	Fracture of femur, other than femoral neck	0.042
Antigua and Barbuda	1995	Fracture of femur, other than femoral neck	0.042
Antigua and Barbuda	2000	Fracture of femur, other than femoral neck	0.042
Antigua and Barbuda	2005	Fracture of femur, other than femoral neck	0.042
Antigua and Barbuda	2010	Fracture of femur, other than femoral neck	0.042
Antigua and Barbuda	2017	Fracture of femur, other than femoral neck	0.042
Antigua and Barbuda	1990	Minor TBI	0.1319
Antigua and Barbuda	1995	Minor TBI	0.1319
Antigua and Barbuda	2000	Minor TBI	0.1319
Antigua and Barbuda	2005	Minor TBI	0.1319
Antigua and Barbuda	2010	Minor TBI	0.1319
Antigua and Barbuda	2017	Minor TBI	0.1319
Antigua and Barbuda	1990	Moderate TBI	0.1637
Antigua and Barbuda	1995	Moderate TBI	0.1637
Antigua and Barbuda	2000	Moderate TBI	0.1637
Antigua and Barbuda	2005	Moderate TBI	0.1637
Antigua and Barbuda	2010	Moderate TBI	0.1637
Antigua and Barbuda	2017	Moderate TBI	0.1637
Antigua and Barbuda	1990	Amputation of fingers (excluding thumb)	0.0047
Antigua and Barbuda	1995	Amputation of fingers (excluding thumb)	0.0047
Antigua and Barbuda	2000	Amputation of fingers (excluding thumb)	0.0047
Antigua and Barbuda	2005	Amputation of fingers (excluding thumb)	0.0047
Antigua and Barbuda	2010	Amputation of fingers (excluding thumb)	0.0047
Antigua and Barbuda	2017	Amputation of fingers (excluding thumb)	0.0047
Antigua and Barbuda	1990	Spinal cord lesion at neck level	0.4396
Antigua and Barbuda	1995	Spinal cord lesion at neck level	0.4348
Antigua and Barbuda	2000	Spinal cord lesion at neck level	0.4307
Antigua and Barbuda	2005	Spinal cord lesion at neck level	0.427
Antigua and Barbuda	2010	Spinal cord lesion at neck level	0.4226
Antigua and Barbuda	2017	Spinal cord lesion at neck level	0.4193
Antigua and Barbuda	1990	Spinal cord lesion below neck level	0.2363
Antigua and Barbuda	1995	Spinal cord lesion below neck level	0.2262
Antigua and Barbuda	2000	Spinal cord lesion below neck level	0.2174
Antigua and Barbuda	2005	Spinal cord lesion below neck level	0.2096
Antigua and Barbuda	2010	Spinal cord lesion below neck level	0.2004
Antigua and Barbuda	2017	Spinal cord lesion below neck level	0.1935
Antigua and Barbuda	1990	Drowning and nonfatal submersion	0.2471
Antigua and Barbuda	1995	Drowning and nonfatal submersion	0.2471
Antigua and Barbuda	2000	Drowning and nonfatal submersion	0.2471
Antigua and Barbuda	2005	Drowning and nonfatal submersion	0.2471
Antigua and Barbuda	2010	Drowning and nonfatal submersion	0.2471
Antigua and Barbuda	2017	Drowning and nonfatal submersion	0.2471
Antigua and Barbuda	1990	Asphyxiation	0.2471
Antigua and Barbuda	1995	Asphyxiation	0.2471
Antigua and Barbuda	2000	Asphyxiation	0.2471
Antigua and Barbuda	2005	Asphyxiation	0.2471
Antigua and Barbuda	2010	Asphyxiation	0.2471
Antigua and Barbuda	2017	Asphyxiation	0.2471
Antigua and Barbuda	1990	Crush injury	0.1325
Antigua and Barbuda	1995	Crush injury	0.1325
Antigua and Barbuda	2000	Crush injury	0.1325
Antigua and Barbuda	2005	Crush injury	0.1325
Antigua and Barbuda	2010	Crush injury	0.1325
Antigua and Barbuda	2017	Crush injury	0.1325
Antigua and Barbuda	1990	Nerve injury	0.1129
Antigua and Barbuda	1995	Nerve injury	0.1129
Antigua and Barbuda	2000	Nerve injury	0.1129
Antigua and Barbuda	2005	Nerve injury	0.1129
Antigua and Barbuda	2010	Nerve injury	0.1129
Antigua and Barbuda	2017	Nerve injury	0.1129
Antigua and Barbuda	1990	Injury to eyes	0.0165
Antigua and Barbuda	1995	Injury to eyes	0.0165
Antigua and Barbuda	2000	Injury to eyes	0.0165
Antigua and Barbuda	2005	Injury to eyes	0.0165
Antigua and Barbuda	2010	Injury to eyes	0.0165
Antigua and Barbuda	2017	Injury to eyes	0.0165
Antigua and Barbuda	1990	Amputation of lower limb, unilateral	0.0703
Antigua and Barbuda	1995	Amputation of lower limb, unilateral	0.0649
Antigua and Barbuda	2000	Amputation of lower limb, unilateral	0.0602
Antigua and Barbuda	2005	Amputation of lower limb, unilateral	0.056
Antigua and Barbuda	2010	Amputation of lower limb, unilateral	0.0511
Antigua and Barbuda	2017	Amputation of lower limb, unilateral	0.0474
Antigua and Barbuda	1990	Open wound(s)	0.011
Antigua and Barbuda	1995	Open wound(s)	0.011
Antigua and Barbuda	2000	Open wound(s)	0.011
Antigua and Barbuda	2005	Open wound(s)	0.011
Antigua and Barbuda	2010	Open wound(s)	0.011
Antigua and Barbuda	2017	Open wound(s)	0.011
Antigua and Barbuda	1990	Poisoning requiring urgent care	0.1628
Antigua and Barbuda	1995	Poisoning requiring urgent care	0.1628
Antigua and Barbuda	2000	Poisoning requiring urgent care	0.1628
Antigua and Barbuda	2005	Poisoning requiring urgent care	0.1628
Antigua and Barbuda	2010	Poisoning requiring urgent care	0.1628
Antigua and Barbuda	2017	Poisoning requiring urgent care	0.1628
Antigua and Barbuda	1990	Severe chest injury	0.0474
Antigua and Barbuda	1995	Severe chest injury	0.0474
Antigua and Barbuda	2000	Severe chest injury	0.0474
Antigua and Barbuda	2005	Severe chest injury	0.0474
Antigua and Barbuda	2010	Severe chest injury	0.0474
Antigua and Barbuda	2017	Severe chest injury	0.0474
Antigua and Barbuda	1990	Internal hemorrhage in abdomen and pelvis	0.1143
Antigua and Barbuda	1995	Internal hemorrhage in abdomen and pelvis	0.1143
Antigua and Barbuda	2000	Internal hemorrhage in abdomen and pelvis	0.1143
Antigua and Barbuda	2005	Internal hemorrhage in abdomen and pelvis	0.1143
Antigua and Barbuda	2010	Internal hemorrhage in abdomen and pelvis	0.1143
Antigua and Barbuda	2017	Internal hemorrhage in abdomen and pelvis	0.1143
Antigua and Barbuda	1990	Contusion in any part of the body	0.0075
Antigua and Barbuda	1995	Contusion in any part of the body	0.0075
Antigua and Barbuda	2000	Contusion in any part of the body	0.0075
Antigua and Barbuda	2005	Contusion in any part of the body	0.0075
Antigua and Barbuda	2010	Contusion in any part of the body	0.0075
Antigua and Barbuda	2017	Contusion in any part of the body	0.0075
Antigua and Barbuda	1990	Effect of different environmental factors	0.049
Antigua and Barbuda	1995	Effect of different environmental factors	0.049
Antigua and Barbuda	2000	Effect of different environmental factors	0.049
Antigua and Barbuda	2005	Effect of different environmental factors	0.049
Antigua and Barbuda	2010	Effect of different environmental factors	0.049
Antigua and Barbuda	2017	Effect of different environmental factors	0.049
Antigua and Barbuda	1990	Complications following therapeutic procedures	0.0506
Antigua and Barbuda	1995	Complications following therapeutic procedures	0.0506
Antigua and Barbuda	2000	Complications following therapeutic procedures	0.0506
Antigua and Barbuda	2005	Complications following therapeutic procedures	0.0506
Antigua and Barbuda	2010	Complications following therapeutic procedures	0.0506
Antigua and Barbuda	2017	Complications following therapeutic procedures	0.0506
Antigua and Barbuda	1990	Multiple fractures, dislocations, crashes, wounds, sprains, and strains	0.138
Antigua and Barbuda	1995	Multiple fractures, dislocations, crashes, wounds, sprains, and strains	0.1242
Antigua and Barbuda	2000	Multiple fractures, dislocations, crashes, wounds, sprains, and strains	0.1122
Antigua and Barbuda	2005	Multiple fractures, dislocations, crashes, wounds, sprains, and strains	0.1015
Antigua and Barbuda	2010	Multiple fractures, dislocations, crashes, wounds, sprains, and strains	0.0889
Antigua and Barbuda	2017	Multiple fractures, dislocations, crashes, wounds, sprains, and strains	0.0793
Antigua and Barbuda	1990	Amputation of upper limb, unilateral	0.0574
Antigua and Barbuda	1995	Amputation of upper limb, unilateral	0.0543
Antigua and Barbuda	2000	Amputation of upper limb, unilateral	0.0515
Antigua and Barbuda	2005	Amputation of upper limb, unilateral	0.049
Antigua and Barbuda	2010	Amputation of upper limb, unilateral	0.0461
Antigua and Barbuda	2017	Amputation of upper limb, unilateral	0.0439
Antigua and Barbuda	1990	Amputation of thumb	0.0114
Antigua and Barbuda	1995	Amputation of thumb	0.0114
Antigua and Barbuda	2000	Amputation of thumb	0.0114
Antigua and Barbuda	2005	Amputation of thumb	0.0114
Antigua and Barbuda	2010	Amputation of thumb	0.0114
Antigua and Barbuda	2017	Amputation of thumb	0.0114
Antigua and Barbuda	1990	Amputation of toe/toes	0.0059
Antigua and Barbuda	1995	Amputation of toe/toes	0.0059
Antigua and Barbuda	2000	Amputation of toe/toes	0.0059
Antigua and Barbuda	2005	Amputation of toe/toes	0.0059
Antigua and Barbuda	2010	Amputation of toe/toes	0.0059
Antigua and Barbuda	2017	Amputation of toe/toes	0.0059
Antigua and Barbuda	1990	Burns, <20% total burned surface area without lower airway burns	0.0163
Antigua and Barbuda	1995	Burns, <20% total burned surface area without lower airway burns	0.0163
Antigua and Barbuda	2000	Burns, <20% total burned surface area without lower airway burns	0.0163
Antigua and Barbuda	2005	Burns, <20% total burned surface area without lower airway burns	0.0163
Antigua and Barbuda	2010	Burns, <20% total burned surface area without lower airway burns	0.0163
Antigua and Barbuda	2017	Burns, <20% total burned surface area without lower airway burns	0.0163
Antigua and Barbuda	1990	Burns, >=20% total burned surface area or >= 10% burned surface area if head/neck or hands/wrist involved w/o lower airway burns	0.2094
Antigua and Barbuda	1995	Burns, >=20% total burned surface area or >= 10% burned surface area if head/neck or hands/wrist involved w/o lower airway burns	0.1966
Antigua and Barbuda	2000	Burns, >=20% total burned surface area or >= 10% burned surface area if head/neck or hands/wrist involved w/o lower airway burns	0.1853
Antigua and Barbuda	2005	Burns, >=20% total burned surface area or >= 10% burned surface area if head/neck or hands/wrist involved w/o lower airway burns	0.1754
Antigua and Barbuda	2010	Burns, >=20% total burned surface area or >= 10% burned surface area if head/neck or hands/wrist involved w/o lower airway burns	0.1636
Antigua and Barbuda	2017	Burns, >=20% total burned surface area or >= 10% burned surface area if head/neck or hands/wrist involved w/o lower airway burns	0.1547
Commonwealth of the Bahamas	1990	Amputation of lower limbs, bilateral	0.1868
Commonwealth of the Bahamas	1995	Amputation of lower limbs, bilateral	0.1782
Commonwealth of the Bahamas	2000	Amputation of lower limbs, bilateral	0.1623
Commonwealth of the Bahamas	2005	Amputation of lower limbs, bilateral	0.1573
Commonwealth of the Bahamas	2010	Amputation of lower limbs, bilateral	0.153
Commonwealth of the Bahamas	2017	Amputation of lower limbs, bilateral	0.1518
Commonwealth of the Bahamas	1990	Lower airway burns	0.2252
Commonwealth of the Bahamas	1995	Lower airway burns	0.2252
Commonwealth of the Bahamas	2000	Lower airway burns	0.2252
Commonwealth of the Bahamas	2005	Lower airway burns	0.2252
Commonwealth of the Bahamas	2010	Lower airway burns	0.2252
Commonwealth of the Bahamas	2017	Lower airway burns	0.2252
Commonwealth of the Bahamas	1990	Dislocation of hip	0.0159
Commonwealth of the Bahamas	1995	Dislocation of hip	0.0159
Commonwealth of the Bahamas	2000	Dislocation of hip	0.0159
Commonwealth of the Bahamas	2005	Dislocation of hip	0.0159
Commonwealth of the Bahamas	2010	Dislocation of hip	0.0159
Commonwealth of the Bahamas	2017	Dislocation of hip	0.0159
Commonwealth of the Bahamas	1990	Dislocation of knee	0.1134
Commonwealth of the Bahamas	1995	Dislocation of knee	0.1134
Commonwealth of the Bahamas	2000	Dislocation of knee	0.1134
Commonwealth of the Bahamas	2005	Dislocation of knee	0.1134
Commonwealth of the Bahamas	2010	Dislocation of knee	0.1134
Commonwealth of the Bahamas	2017	Dislocation of knee	0.1134
Commonwealth of the Bahamas	1990	Dislocation of shoulder	0.062
Commonwealth of the Bahamas	1995	Dislocation of shoulder	0.062
Commonwealth of the Bahamas	2000	Dislocation of shoulder	0.062
Commonwealth of the Bahamas	2005	Dislocation of shoulder	0.062
Commonwealth of the Bahamas	2010	Dislocation of shoulder	0.062
Commonwealth of the Bahamas	2017	Dislocation of shoulder	0.062
Commonwealth of the Bahamas	1990	Muscle and tendon injuries, including sprains and strains lesser dislocations	0.0075
Commonwealth of the Bahamas	1995	Muscle and tendon injuries, including sprains and strains lesser dislocations	0.0075
Commonwealth of the Bahamas	2000	Muscle and tendon injuries, including sprains and strains lesser dislocations	0.0075
Commonwealth of the Bahamas	2005	Muscle and tendon injuries, including sprains and strains lesser dislocations	0.0075
Commonwealth of the Bahamas	2010	Muscle and tendon injuries, including sprains and strains lesser dislocations	0.0075
Commonwealth of the Bahamas	2017	Muscle and tendon injuries, including sprains and strains lesser dislocations	0.0075
Commonwealth of the Bahamas	1990	Fracture of clavicle, scapula, or humerus	0.0349
Commonwealth of the Bahamas	1995	Fracture of clavicle, scapula, or humerus	0.0349
Commonwealth of the Bahamas	2000	Fracture of clavicle, scapula, or humerus	0.0349
Commonwealth of the Bahamas	2005	Fracture of clavicle, scapula, or humerus	0.0349
Commonwealth of the Bahamas	2010	Fracture of clavicle, scapula, or humerus	0.0349
Commonwealth of the Bahamas	2017	Fracture of clavicle, scapula, or humerus	0.0349
Commonwealth of the Bahamas	1990	Fracture of face bones	0.0669
Commonwealth of the Bahamas	1995	Fracture of face bones	0.0669
Commonwealth of the Bahamas	2000	Fracture of face bones	0.0669
Commonwealth of the Bahamas	2005	Fracture of face bones	0.0669
Commonwealth of the Bahamas	2010	Fracture of face bones	0.0669
Commonwealth of the Bahamas	2017	Fracture of face bones	0.0669
Commonwealth of the Bahamas	1990	Fracture of foot bones except ankle	0.0264
Commonwealth of the Bahamas	1995	Fracture of foot bones except ankle	0.0264
Commonwealth of the Bahamas	2000	Fracture of foot bones except ankle	0.0264
Commonwealth of the Bahamas	2005	Fracture of foot bones except ankle	0.0264
Commonwealth of the Bahamas	2010	Fracture of foot bones except ankle	0.0264
Commonwealth of the Bahamas	2017	Fracture of foot bones except ankle	0.0264
Commonwealth of the Bahamas	1990	Fracture of hand (wrist and other distal part of hand)	0.0138
Commonwealth of the Bahamas	1995	Fracture of hand (wrist and other distal part of hand)	0.0138
Commonwealth of the Bahamas	2000	Fracture of hand (wrist and other distal part of hand)	0.0138
Commonwealth of the Bahamas	2005	Fracture of hand (wrist and other distal part of hand)	0.0138
Commonwealth of the Bahamas	2010	Fracture of hand (wrist and other distal part of hand)	0.0138
Commonwealth of the Bahamas	2017	Fracture of hand (wrist and other distal part of hand)	0.0138
Commonwealth of the Bahamas	1990	Fracture of hip	0.1418
Commonwealth of the Bahamas	1995	Fracture of hip	0.1304
Commonwealth of the Bahamas	2000	Fracture of hip	0.1094
Commonwealth of the Bahamas	2005	Fracture of hip	0.1028
Commonwealth of the Bahamas	2010	Fracture of hip	0.097
Commonwealth of the Bahamas	2017	Fracture of hip	0.0955
Commonwealth of the Bahamas	1990	Amputation of upper limbs, bilateral	0.1745
Commonwealth of the Bahamas	1995	Amputation of upper limbs, bilateral	0.1627
Commonwealth of the Bahamas	2000	Amputation of upper limbs, bilateral	0.141
Commonwealth of the Bahamas	2005	Amputation of upper limbs, bilateral	0.1342
Commonwealth of the Bahamas	2010	Amputation of upper limbs, bilateral	0.1282
Commonwealth of the Bahamas	2017	Amputation of upper limbs, bilateral	0.1267
Commonwealth of the Bahamas	1990	Fracture of patella, tibia or fibula, or ankle	0.0554
Commonwealth of the Bahamas	1995	Fracture of patella, tibia or fibula, or ankle	0.0554
Commonwealth of the Bahamas	2000	Fracture of patella, tibia or fibula, or ankle	0.0554
Commonwealth of the Bahamas	2005	Fracture of patella, tibia or fibula, or ankle	0.0554
Commonwealth of the Bahamas	2010	Fracture of patella, tibia or fibula, or ankle	0.0554
Commonwealth of the Bahamas	2017	Fracture of patella, tibia or fibula, or ankle	0.0554
Commonwealth of the Bahamas	1990	Fracture of pelvis	0.1823
Commonwealth of the Bahamas	1995	Fracture of pelvis	0.1823
Commonwealth of the Bahamas	2000	Fracture of pelvis	0.1823
Commonwealth of the Bahamas	2005	Fracture of pelvis	0.1823
Commonwealth of the Bahamas	2010	Fracture of pelvis	0.1823
Commonwealth of the Bahamas	2017	Fracture of pelvis	0.1823
Commonwealth of the Bahamas	1990	Fracture of radius and/or ulna	0.0435
Commonwealth of the Bahamas	1995	Fracture of radius and/or ulna	0.0435
Commonwealth of the Bahamas	2000	Fracture of radius and/or ulna	0.0435
Commonwealth of the Bahamas	2005	Fracture of radius and/or ulna	0.0435
Commonwealth of the Bahamas	2010	Fracture of radius and/or ulna	0.0435
Commonwealth of the Bahamas	2017	Fracture of radius and/or ulna	0.0435
Commonwealth of the Bahamas	1990	Fracture of skull	0.0714
Commonwealth of the Bahamas	1995	Fracture of skull	0.0714
Commonwealth of the Bahamas	2000	Fracture of skull	0.0714
Commonwealth of the Bahamas	2005	Fracture of skull	0.0714
Commonwealth of the Bahamas	2010	Fracture of skull	0.0714
Commonwealth of the Bahamas	2017	Fracture of skull	0.0714
Commonwealth of the Bahamas	1990	Fracture of sternum and/or fracture of one or more ribs	0.1027
Commonwealth of the Bahamas	1995	Fracture of sternum and/or fracture of one or more ribs	0.1027
Commonwealth of the Bahamas	2000	Fracture of sternum and/or fracture of one or more ribs	0.1027
Commonwealth of the Bahamas	2005	Fracture of sternum and/or fracture of one or more ribs	0.1027
Commonwealth of the Bahamas	2010	Fracture of sternum and/or fracture of one or more ribs	0.1027
Commonwealth of the Bahamas	2017	Fracture of sternum and/or fracture of one or more ribs	0.1027
Commonwealth of the Bahamas	1990	Fracture of vertebral column	0.1106
Commonwealth of the Bahamas	1995	Fracture of vertebral column	0.1106
Commonwealth of the Bahamas	2000	Fracture of vertebral column	0.1106
Commonwealth of the Bahamas	2005	Fracture of vertebral column	0.1106
Commonwealth of the Bahamas	2010	Fracture of vertebral column	0.1106
Commonwealth of the Bahamas	2017	Fracture of vertebral column	0.1106
Commonwealth of the Bahamas	1990	Fracture of femur, other than femoral neck	0.042
Commonwealth of the Bahamas	1995	Fracture of femur, other than femoral neck	0.042
Commonwealth of the Bahamas	2000	Fracture of femur, other than femoral neck	0.042
Commonwealth of the Bahamas	2005	Fracture of femur, other than femoral neck	0.042
Commonwealth of the Bahamas	2010	Fracture of femur, other than femoral neck	0.042
Commonwealth of the Bahamas	2017	Fracture of femur, other than femoral neck	0.042
Commonwealth of the Bahamas	1990	Minor TBI	0.1319
Commonwealth of the Bahamas	1995	Minor TBI	0.1319
Commonwealth of the Bahamas	2000	Minor TBI	0.1319
Commonwealth of the Bahamas	2005	Minor TBI	0.1319
Commonwealth of the Bahamas	2010	Minor TBI	0.1319
Commonwealth of the Bahamas	2017	Minor TBI	0.1319
Commonwealth of the Bahamas	1990	Moderate TBI	0.1637
Commonwealth of the Bahamas	1995	Moderate TBI	0.1637
Commonwealth of the Bahamas	2000	Moderate TBI	0.1637
Commonwealth of the Bahamas	2005	Moderate TBI	0.1637
Commonwealth of the Bahamas	2010	Moderate TBI	0.1637
Commonwealth of the Bahamas	2017	Moderate TBI	0.1637
Commonwealth of the Bahamas	1990	Amputation of fingers (excluding thumb)	0.0047
Commonwealth of the Bahamas	1995	Amputation of fingers (excluding thumb)	0.0047
Commonwealth of the Bahamas	2000	Amputation of fingers (excluding thumb)	0.0047
Commonwealth of the Bahamas	2005	Amputation of fingers (excluding thumb)	0.0047
Commonwealth of the Bahamas	2010	Amputation of fingers (excluding thumb)	0.0047
Commonwealth of the Bahamas	2017	Amputation of fingers (excluding thumb)	0.0047
Commonwealth of the Bahamas	1990	Spinal cord lesion at neck level	0.4409
Commonwealth of the Bahamas	1995	Spinal cord lesion at neck level	0.437
Commonwealth of the Bahamas	2000	Spinal cord lesion at neck level	0.4297
Commonwealth of the Bahamas	2005	Spinal cord lesion at neck level	0.4274
Commonwealth of the Bahamas	2010	Spinal cord lesion at neck level	0.4254
Commonwealth of the Bahamas	2017	Spinal cord lesion at neck level	0.4249
Commonwealth of the Bahamas	1990	Spinal cord lesion below neck level	0.239
Commonwealth of the Bahamas	1995	Spinal cord lesion below neck level	0.2307
Commonwealth of the Bahamas	2000	Spinal cord lesion below neck level	0.2154
Commonwealth of the Bahamas	2005	Spinal cord lesion below neck level	0.2106
Commonwealth of the Bahamas	2010	Spinal cord lesion below neck level	0.2064
Commonwealth of the Bahamas	2017	Spinal cord lesion below neck level	0.2053
Commonwealth of the Bahamas	1990	Drowning and nonfatal submersion	0.2471
Commonwealth of the Bahamas	1995	Drowning and nonfatal submersion	0.2471
Commonwealth of the Bahamas	2000	Drowning and nonfatal submersion	0.2471
Commonwealth of the Bahamas	2005	Drowning and nonfatal submersion	0.2471
Commonwealth of the Bahamas	2010	Drowning and nonfatal submersion	0.2471
Commonwealth of the Bahamas	2017	Drowning and nonfatal submersion	0.2471
Commonwealth of the Bahamas	1990	Asphyxiation	0.2471
Commonwealth of the Bahamas	1995	Asphyxiation	0.2471
Commonwealth of the Bahamas	2000	Asphyxiation	0.2471
Commonwealth of the Bahamas	2005	Asphyxiation	0.2471
Commonwealth of the Bahamas	2010	Asphyxiation	0.2471
Commonwealth of the Bahamas	2017	Asphyxiation	0.2471
Commonwealth of the Bahamas	1990	Crush injury	0.1325
Commonwealth of the Bahamas	1995	Crush injury	0.1325
Commonwealth of the Bahamas	2000	Crush injury	0.1325
Commonwealth of the Bahamas	2005	Crush injury	0.1325
Commonwealth of the Bahamas	2010	Crush injury	0.1325
Commonwealth of the Bahamas	2017	Crush injury	0.1325
Commonwealth of the Bahamas	1990	Nerve injury	0.1129
Commonwealth of the Bahamas	1995	Nerve injury	0.1129
Commonwealth of the Bahamas	2000	Nerve injury	0.1129
Commonwealth of the Bahamas	2005	Nerve injury	0.1129
Commonwealth of the Bahamas	2010	Nerve injury	0.1129
Commonwealth of the Bahamas	2017	Nerve injury	0.1129
Commonwealth of the Bahamas	1990	Injury to eyes	0.0165
Commonwealth of the Bahamas	1995	Injury to eyes	0.0165
Commonwealth of the Bahamas	2000	Injury to eyes	0.0165
Commonwealth of the Bahamas	2005	Injury to eyes	0.0165
Commonwealth of the Bahamas	2010	Injury to eyes	0.0165
Commonwealth of the Bahamas	2017	Injury to eyes	0.0165
Commonwealth of the Bahamas	1990	Amputation of lower limb, unilateral	0.0717
Commonwealth of the Bahamas	1995	Amputation of lower limb, unilateral	0.0673
Commonwealth of the Bahamas	2000	Amputation of lower limb, unilateral	0.0591
Commonwealth of the Bahamas	2005	Amputation of lower limb, unilateral	0.0565
Commonwealth of the Bahamas	2010	Amputation of lower limb, unilateral	0.0543
Commonwealth of the Bahamas	2017	Amputation of lower limb, unilateral	0.0537
Commonwealth of the Bahamas	1990	Open wound(s)	0.011
Commonwealth of the Bahamas	1995	Open wound(s)	0.011
Commonwealth of the Bahamas	2000	Open wound(s)	0.011
Commonwealth of the Bahamas	2005	Open wound(s)	0.011
Commonwealth of the Bahamas	2010	Open wound(s)	0.011
Commonwealth of the Bahamas	2017	Open wound(s)	0.011
Commonwealth of the Bahamas	1990	Poisoning requiring urgent care	0.1628
Commonwealth of the Bahamas	1995	Poisoning requiring urgent care	0.1628
Commonwealth of the Bahamas	2000	Poisoning requiring urgent care	0.1628
Commonwealth of the Bahamas	2005	Poisoning requiring urgent care	0.1628
Commonwealth of the Bahamas	2010	Poisoning requiring urgent care	0.1628
Commonwealth of the Bahamas	2017	Poisoning requiring urgent care	0.1628
Commonwealth of the Bahamas	1990	Severe chest injury	0.0474
Commonwealth of the Bahamas	1995	Severe chest injury	0.0474
Commonwealth of the Bahamas	2000	Severe chest injury	0.0474
Commonwealth of the Bahamas	2005	Severe chest injury	0.0474
Commonwealth of the Bahamas	2010	Severe chest injury	0.0474
Commonwealth of the Bahamas	2017	Severe chest injury	0.0474
Commonwealth of the Bahamas	1990	Internal hemorrhage in abdomen and pelvis	0.1143
Commonwealth of the Bahamas	1995	Internal hemorrhage in abdomen and pelvis	0.1143
Commonwealth of the Bahamas	2000	Internal hemorrhage in abdomen and pelvis	0.1143
Commonwealth of the Bahamas	2005	Internal hemorrhage in abdomen and pelvis	0.1143
Commonwealth of the Bahamas	2010	Internal hemorrhage in abdomen and pelvis	0.1143
Commonwealth of the Bahamas	2017	Internal hemorrhage in abdomen and pelvis	0.1143
Commonwealth of the Bahamas	1990	Contusion in any part of the body	0.0075
Commonwealth of the Bahamas	1995	Contusion in any part of the body	0.0075
Commonwealth of the Bahamas	2000	Contusion in any part of the body	0.0075
Commonwealth of the Bahamas	2005	Contusion in any part of the body	0.0075
Commonwealth of the Bahamas	2010	Contusion in any part of the body	0.0075
Commonwealth of the Bahamas	2017	Contusion in any part of the body	0.0075
Commonwealth of the Bahamas	1990	Effect of different environmental factors	0.049
Commonwealth of the Bahamas	1995	Effect of different environmental factors	0.049
Commonwealth of the Bahamas	2000	Effect of different environmental factors	0.049
Commonwealth of the Bahamas	2005	Effect of different environmental factors	0.049
Commonwealth of the Bahamas	2010	Effect of different environmental factors	0.049
Commonwealth of the Bahamas	2017	Effect of different environmental factors	0.049
Commonwealth of the Bahamas	1990	Complications following therapeutic procedures	0.0506

Commonwealth of the Bahamas	1995	Complications following therapeutic procedures	0.0506
Commonwealth of the Bahamas	2000	Complications following therapeutic procedures	0.0506
Commonwealth of the Bahamas	2005	Complications following therapeutic procedures	0.0506
Commonwealth of the Bahamas	2010	Complications following therapeutic procedures	0.0506
Commonwealth of the Bahamas	2017	Complications following therapeutic procedures	0.0506
Commonwealth of the Bahamas	1990	Multiple fractures, dislocations, crashes, wounds, sprains, and strains	0.1418
Commonwealth of the Bahamas	1995	Multiple fractures, dislocations, crashes, wounds, sprains, and strains	0.1304
Commonwealth of the Bahamas	2000	Multiple fractures, dislocations, crashes, wounds, sprains, and strains	0.1094
Commonwealth of the Bahamas	2005	Multiple fractures, dislocations, crashes, wounds, sprains, and strains	0.1028
Commonwealth of the Bahamas	2010	Multiple fractures, dislocations, crashes, wounds, sprains, and strains	0.097
Commonwealth of the Bahamas	2017	Multiple fractures, dislocations, crashes, wounds, sprains, and strains	0.0955
Commonwealth of the Bahamas	1990	Amputation of upper limb, unilateral	0.0583
Commonwealth of the Bahamas	1995	Amputation of upper limb, unilateral	0.0557
Commonwealth of the Bahamas	2000	Amputation of upper limb, unilateral	0.0508
Commonwealth of the Bahamas	2005	Amputation of upper limb, unilateral	0.0493
Commonwealth of the Bahamas	2010	Amputation of upper limb, unilateral	0.048
Commonwealth of the Bahamas	2017	Amputation of upper limb, unilateral	0.0477
Commonwealth of the Bahamas	1990	Amputation of thumb	0.0114
Commonwealth of the Bahamas	1995	Amputation of thumb	0.0114
Commonwealth of the Bahamas	2000	Amputation of thumb	0.0114
Commonwealth of the Bahamas	2005	Amputation of thumb	0.0114
Commonwealth of the Bahamas	2010	Amputation of thumb	0.0114
Commonwealth of the Bahamas	2017	Amputation of thumb	0.0114
Commonwealth of the Bahamas	1990	Amputation of toe/toes	0.0059
Commonwealth of the Bahamas	1995	Amputation of toe/toes	0.0059
Commonwealth of the Bahamas	2000	Amputation of toe/toes	0.0059
Commonwealth of the Bahamas	2005	Amputation of toe/toes	0.0059
Commonwealth of the Bahamas	2010	Amputation of toe/toes	0.0059
Commonwealth of the Bahamas	2017	Amputation of toe/toes	0.0059
Commonwealth of the Bahamas	1990	Burns, <20% total burned surface area without lower airway burns	0.0163
Commonwealth of the Bahamas	1995	Burns, <20% total burned surface area without lower airway burns	0.0163
Commonwealth of the Bahamas	2000	Burns, <20% total burned surface area without lower airway burns	0.0163
Commonwealth of the Bahamas	2005	Burns, <20% total burned surface area without lower airway burns	0.0163
Commonwealth of the Bahamas	2010	Burns, <20% total burned surface area without lower airway burns	0.0163
Commonwealth of the Bahamas	2017	Burns, <20% total burned surface area without lower airway burns	0.0163
Commonwealth of the Bahamas	1990	Burns, >=20% total burned surface area or >= 10% burned surface area if head/neck or hands/wrist involved w/o lower airway burns	0.2129
Commonwealth of the Bahamas	1995	Burns, >=20% total burned surface area or >= 10% burned surface area if head/neck or hands/wrist involved w/o lower airway burns	0.2023
Commonwealth of the Bahamas	2000	Burns, >=20% total burned surface area or >= 10% burned surface area if head/neck or hands/wrist involved w/o lower airway burns	0.1827
Commonwealth of the Bahamas	2005	Burns, >=20% total burned surface area or >= 10% burned surface area if head/neck or hands/wrist involved w/o lower airway burns	0.1766
Commonwealth of the Bahamas	2010	Burns, >=20% total burned surface area or >= 10% burned surface area if head/neck or hands/wrist involved w/o lower airway burns	0.1712
Commonwealth of the Bahamas	2017	Burns, >=20% total burned surface area or >= 10% burned surface area if head/neck or hands/wrist involved w/o lower airway burns	0.1698
Barbados	1990	Amputation of lower limbs, bilateral	0.1761
Barbados	1995	Amputation of lower limbs, bilateral	0.1597
Barbados	2000	Amputation of lower limbs, bilateral	0.1494
Barbados	2005	Amputation of lower limbs, bilateral	0.1453
Barbados	2010	Amputation of lower limbs, bilateral	0.1381
Barbados	2017	Amputation of lower limbs, bilateral	0.1366
Barbados	1990	Lower airway burns	0.2252
Barbados	1995	Lower airway burns	0.2252
Barbados	2000	Lower airway burns	0.2252
Barbados	2005	Lower airway burns	0.2252
Barbados	2010	Lower airway burns	0.2252
Barbados	2017	Lower airway burns	0.2252
Barbados	1990	Dislocation of hip	0.0159
Barbados	1995	Dislocation of hip	0.0159
Barbados	2000	Dislocation of hip	0.0159
Barbados	2005	Dislocation of hip	0.0159
Barbados	2010	Dislocation of hip	0.0159
Barbados	2017	Dislocation of hip	0.0159
Barbados	1990	Dislocation of knee	0.1134
Barbados	1995	Dislocation of knee	0.1134
Barbados	2000	Dislocation of knee	0.1134
Barbados	2005	Dislocation of knee	0.1134
Barbados	2010	Dislocation of knee	0.1134
Barbados	2017	Dislocation of knee	0.1134
Barbados	1990	Dislocation of shoulder	0.062
Barbados	1995	Dislocation of shoulder	0.062
Barbados	2000	Dislocation of shoulder	0.062
Barbados	2005	Dislocation of shoulder	0.062
Barbados	2010	Dislocation of shoulder	0.062
Barbados	2017	Dislocation of shoulder	0.062
Barbados	1990	Muscle and tendon injuries, including sprains and strains lesser dislocations	0.0075
Barbados	1995	Muscle and tendon injuries, including sprains and strains lesser dislocations	0.0075
Barbados	2000	Muscle and tendon injuries, including sprains and strains lesser dislocations	0.0075
Barbados	2005	Muscle and tendon injuries, including sprains and strains lesser dislocations	0.0075
Barbados	2010	Muscle and tendon injuries, including sprains and strains lesser dislocations	0.0075
Barbados	2017	Muscle and tendon injuries, including sprains and strains lesser dislocations	0.0075
Barbados	1990	Fracture of clavicle, scapula, or humerus	0.0349
Barbados	1995	Fracture of clavicle, scapula, or humerus	0.0349
Barbados	2000	Fracture of clavicle, scapula, or humerus	0.0349
Barbados	2005	Fracture of clavicle, scapula, or humerus	0.0349
Barbados	2010	Fracture of clavicle, scapula, or humerus	0.0349
Barbados	2017	Fracture of clavicle, scapula, or humerus	0.0349
Barbados	1990	Fracture of face bones	0.0669
Barbados	1995	Fracture of face bones	0.0669
Barbados	2000	Fracture of face bones	0.0669
Barbados	2005	Fracture of face bones	0.0669
Barbados	2010	Fracture of face bones	0.0669
Barbados	2017	Fracture of face bones	0.0669
Barbados	1990	Fracture of foot bones except ankle	0.0264
Barbados	1995	Fracture of foot bones except ankle	0.0264
Barbados	2000	Fracture of foot bones except ankle	0.0264
Barbados	2005	Fracture of foot bones except ankle	0.0264
Barbados	2010	Fracture of foot bones except ankle	0.0264
Barbados	2017	Fracture of foot bones except ankle	0.0264
Barbados	1990	Fracture of hand (wrist and other distal part of hand)	0.0138
Barbados	1995	Fracture of hand (wrist and other distal part of hand)	0.0138
Barbados	2000	Fracture of hand (wrist and other distal part of hand)	0.0138
Barbados	2005	Fracture of hand (wrist and other distal part of hand)	0.0138
Barbados	2010	Fracture of hand (wrist and other distal part of hand)	0.0138
Barbados	2017	Fracture of hand (wrist and other distal part of hand)	0.0138
Barbados	1990	Fracture of hip	0.1275
Barbados	1995	Fracture of hip	0.1059
Barbados	2000	Fracture of hip	0.0923
Barbados	2005	Fracture of hip	0.0869
Barbados	2010	Fracture of hip	0.0773
Barbados	2017	Fracture of hip	0.0754
Barbados	1990	Amputation of upper limbs, bilateral	0.1598
Barbados	1995	Amputation of upper limbs, bilateral	0.1375
Barbados	2000	Amputation of upper limbs, bilateral	0.1234
Barbados	2005	Amputation of upper limbs, bilateral	0.1178
Barbados	2010	Amputation of upper limbs, bilateral	0.1079
Barbados	2017	Amputation of upper limbs, bilateral	0.1059
Barbados	1990	Fracture of patella, tibia or fibula, or ankle	0.0554
Barbados	1995	Fracture of patella, tibia or fibula, or ankle	0.0554
Barbados	2000	Fracture of patella, tibia or fibula, or ankle	0.0554
Barbados	2005	Fracture of patella, tibia or fibula, or ankle	0.0554
Barbados	2010	Fracture of patella, tibia or fibula, or ankle	0.0554
Barbados	2017	Fracture of patella, tibia or fibula, or ankle	0.0554
Barbados	1990	Fracture of pelvis	0.1823
Barbados	1995	Fracture of pelvis	0.1823
Barbados	2000	Fracture of pelvis	0.1823
Barbados	2005	Fracture of pelvis	0.1823
Barbados	2010	Fracture of pelvis	0.1823
Barbados	2017	Fracture of pelvis	0.1823
Barbados	1990	Fracture of radius and/or ulna	0.0435
Barbados	1995	Fracture of radius and/or ulna	0.0435
Barbados	2000	Fracture of radius and/or ulna	0.0435
Barbados	2005	Fracture of radius and/or ulna	0.0435
Barbados	2010	Fracture of radius and/or ulna	0.0435
Barbados	2017	Fracture of radius and/or ulna	0.0435
Barbados	1990	Fracture of skull	0.0714
Barbados	1995	Fracture of skull	0.0714
Barbados	2000	Fracture of skull	0.0714
Barbados	2005	Fracture of skull	0.0714
Barbados	2010	Fracture of skull	0.0714
Barbados	2017	Fracture of skull	0.0714
Barbados	1990	Fracture of sternum and/or fracture of one or more ribs	0.1027
Barbados	1995	Fracture of sternum and/or fracture of one or more ribs	0.1027
Barbados	2000	Fracture of sternum and/or fracture of one or more ribs	0.1027
Barbados	2005	Fracture of sternum and/or fracture of one or more ribs	0.1027
Barbados	2010	Fracture of sternum and/or fracture of one or more ribs	0.1027
Barbados	2017	Fracture of sternum and/or fracture of one or more ribs	0.1027
Barbados	1990	Fracture of vertebral column	0.1106
Barbados	1995	Fracture of vertebral column	0.1106
Barbados	2000	Fracture of vertebral column	0.1106
Barbados	2005	Fracture of vertebral column	0.1106
Barbados	2010	Fracture of vertebral column	0.1106
Barbados	2017	Fracture of vertebral column	0.1106
Barbados	1990	Fracture of femur, other than femoral neck	0.042
Barbados	1995	Fracture of femur, other than femoral neck	0.042
Barbados	2000	Fracture of femur, other than femoral neck	0.042
Barbados	2005	Fracture of femur, other than femoral neck	0.042
Barbados	2010	Fracture of femur, other than femoral neck	0.042
Barbados	2017	Fracture of femur, other than femoral neck	0.042
Barbados	1990	Minor TBI	0.1319
Barbados	1995	Minor TBI	0.1319
Barbados	2000	Minor TBI	0.1319
Barbados	2005	Minor TBI	0.1319
Barbados	2010	Minor TBI	0.1319
Barbados	2017	Minor TBI	0.1319
Barbados	1990	Moderate TBI	0.1637
Barbados	1995	Moderate TBI	0.1637
Barbados	2000	Moderate TBI	0.1637
Barbados	2005	Moderate TBI	0.1637
Barbados	2010	Moderate TBI	0.1637
Barbados	2017	Moderate TBI	0.1637
Barbados	1990	Amputation of fingers (excluding thumb)	0.0047
Barbados	1995	Amputation of fingers (excluding thumb)	0.0047
Barbados	2000	Amputation of fingers (excluding thumb)	0.0047
Barbados	2005	Amputation of fingers (excluding thumb)	0.0047
Barbados	2010	Amputation of fingers (excluding thumb)	0.0047
Barbados	2017	Amputation of fingers (excluding thumb)	0.0047
Barbados	1990	Spinal cord lesion at neck level	0.436
Barbados	1995	Spinal cord lesion at neck level	0.4285
Barbados	2000	Spinal cord lesion at neck level	0.4238
Barbados	2005	Spinal cord lesion at neck level	0.4219
Barbados	2010	Spinal cord lesion at neck level	0.4186
Barbados	2017	Spinal cord lesion at neck level	0.4179
Barbados	1990	Spinal cord lesion below neck level	0.2286
Barbados	1995	Spinal cord lesion below neck level	0.2129
Barbados	2000	Spinal cord lesion below neck level	0.2029
Barbados	2005	Spinal cord lesion below neck level	0.199
Barbados	2010	Spinal cord lesion below neck level	0.192
Barbados	2017	Spinal cord lesion below neck level	0.1906
Barbados	1990	Drowning and nonfatal submersion	0.2471
Barbados	1995	Drowning and nonfatal submersion	0.2471
Barbados	2000	Drowning and nonfatal submersion	0.2471
Barbados	2005	Drowning and nonfatal submersion	0.2471
Barbados	2010	Drowning and nonfatal submersion	0.2471
Barbados	2017	Drowning and nonfatal submersion	0.2471
Barbados	1990	Asphyxiation	0.2471
Barbados	1995	Asphyxiation	0.2471
Barbados	2000	Asphyxiation	0.2471
Barbados	2005	Asphyxiation	0.2471
Barbados	2010	Asphyxiation	0.2471
Barbados	2017	Asphyxiation	0.2471
Barbados	1990	Crush injury	0.1325
Barbados	1995	Crush injury	0.1325
Barbados	2000	Crush injury	0.1325
Barbados	2005	Crush injury	0.1325
Barbados	2010	Crush injury	0.1325
Barbados	2017	Crush injury	0.1325
Barbados	1990	Nerve injury	0.1129
Barbados	1995	Nerve injury	0.1129
Barbados	2000	Nerve injury	0.1129
Barbados	2005	Nerve injury	0.1129
Barbados	2010	Nerve injury	0.1129
Barbados	2017	Nerve injury	0.1129
Barbados	1990	Injury to eyes	0.0165
Barbados	1995	Injury to eyes	0.0165
Barbados	2000	Injury to eyes	0.0165
Barbados	2005	Injury to eyes	0.0165
Barbados	2010	Injury to eyes	0.0165
Barbados	2017	Injury to eyes	0.0165
Barbados	1990	Amputation of lower limb, unilateral	0.0662
Barbados	1995	Amputation of lower limb, unilateral	0.0578
Barbados	2000	Amputation of lower limb, unilateral	0.0524
Barbados	2005	Amputation of lower limb, unilateral	0.0503
Barbados	2010	Amputation of lower limb, unilateral	0.0466
Barbados	2017	Amputation of lower limb, unilateral	0.0458
Barbados	1990	Open wound(s)	0.011
Barbados	1995	Open wound(s)	0.011
Barbados	2000	Open wound(s)	0.011
Barbados	2005	Open wound(s)	0.011
Barbados	2010	Open wound(s)	0.011
Barbados	2017	Open wound(s)	0.011
Barbados	1990	Poisoning requiring urgent care	0.1628
Barbados	1995	Poisoning requiring urgent care	0.1628
Barbados	2000	Poisoning requiring urgent care	0.1628
Barbados	2005	Poisoning requiring urgent care	0.1628
Barbados	2010	Poisoning requiring urgent care	0.1628
Barbados	2017	Poisoning requiring urgent care	0.1628
Barbados	1990	Severe chest injury	0.0474
Barbados	1995	Severe chest injury	0.0474
Barbados	2000	Severe chest injury	0.0474
Barbados	2005	Severe chest injury	0.0474
Barbados	2010	Severe chest injury	0.0474
Barbados	2017	Severe chest injury	0.0474
Barbados	1990	Internal hemorrhage in abdomen and pelvis	0.1143
Barbados	1995	Internal hemorrhage in abdomen and pelvis	0.1143
Barbados	2000	Internal hemorrhage in abdomen and pelvis	0.1143
Barbados	2005	Internal hemorrhage in abdomen and pelvis	0.1143
Barbados	2010	Internal hemorrhage in abdomen and pelvis	0.1143
Barbados	2017	Internal hemorrhage in abdomen and pelvis	0.1143
Barbados	1990	Contusion in any part of the body	0.0075
Barbados	1995	Contusion in any part of the body	0.0075
Barbados	2000	Contusion in any part of the body	0.0075
Barbados	2005	Contusion in any part of the body	0.0075
Barbados	2010	Contusion in any part of the body	0.0075
Barbados	2017	Contusion in any part of the body	0.0075
Barbados	1990	Effect of different environmental factors	0.049
Barbados	1995	Effect of different environmental factors	0.049
Barbados	2000	Effect of different environmental factors	0.049
Barbados	2005	Effect of different environmental factors	0.049
Barbados	2010	Effect of different environmental factors	0.049
Barbados	2017	Effect of different environmental factors	0.049
Barbados	1990	Complications following therapeutic procedures	0.0506
Barbados	1995	Complications following therapeutic procedures	0.0506
Barbados	2000	Complications following therapeutic procedures	0.0506
Barbados	2005	Complications following therapeutic procedures	0.0506
Barbados	2010	Complications following therapeutic procedures	0.0506
Barbados	2017	Complications following therapeutic procedures	0.0506
Barbados	1990	Multiple fractures, dislocations, crashes, wounds, sprains, and strains	0.1275
Barbados	1995	Multiple fractures, dislocations, crashes, wounds, sprains, and strains	0.1059
Barbados	2000	Multiple fractures, dislocations, crashes, wounds, sprains, and strains	0.0923
Barbados	2005	Multiple fractures, dislocations, crashes, wounds, sprains, and strains	0.0869
Barbados	2010	Multiple fractures, dislocations, crashes, wounds, sprains, and strains	0.0773
Barbados	2017	Multiple fractures, dislocations, crashes, wounds, sprains, and strains	0.0754
Barbados	1990	Amputation of upper limb, unilateral	0.055
Barbados	1995	Amputation of upper limb, unilateral	0.0501
Barbados	2000	Amputation of upper limb, unilateral	0.0469
Barbados	2005	Amputation of upper limb, unilateral	0.0457
Barbados	2010	Amputation of upper limb, unilateral	0.0435
Barbados	2017	Amputation of upper limb, unilateral	0.043
Barbados	1990	Amputation of thumb	0.0114
Barbados	1995	Amputation of thumb	0.0114
Barbados	2000	Amputation of thumb	0.0114
Barbados	2005	Amputation of thumb	0.0114
Barbados	2010	Amputation of thumb	0.0114
Barbados	2017	Amputation of thumb	0.0114
Barbados	1990	Amputation of toe/toes	0.0059
Barbados	1995	Amputation of toe/toes	0.0059
Barbados	2000	Amputation of toe/toes	0.0059
Barbados	2005	Amputation of toe/toes	0.0059
Barbados	2010	Amputation of toe/toes	0.0059
Barbados	2017	Amputation of toe/toes	0.0059
Barbados	1990	Burns, <20% total burned surface area without lower airway burns	0.0163
Barbados	1995	Burns, <20% total burned surface area without lower airway burns	0.0163
Barbados	2000	Burns, <20% total burned surface area without lower airway burns	0.0163
Barbados	2005	Burns, <20% total burned surface area without lower airway burns	0.0163
Barbados	2010	Burns, <20% total burned surface area without lower airway burns	0.0163
Barbados	2017	Burns, <20% total burned surface area without lower airway burns	0.0163
Barbados	1990	Burns, >=20% total burned surface area or >= 10% burned surface area if head/neck or hands/wrist involved w/o lower airway burns	0.1996
Barbados	1995	Burns, >=20% total burned surface area or >= 10% burned surface area if head/neck or hands/wrist involved w/o lower airway burns	0.1795
Barbados	2000	Burns, >=20% total burned surface area or >= 10% burned surface area if head/neck or hands/wrist involved w/o lower airway burns	0.1668
Barbados	2005	Burns, >=20% total burned surface area or >= 10% burned surface area if head/neck or hands/wrist involved w/o lower airway burns	0.1618
Barbados	2010	Burns, >=20% total burned surface area or >= 10% burned surface area if head/neck or hands/wrist involved w/o lower airway burns	0.1528
Barbados	2017	Burns, >=20% total burned surface area or >= 10% burned surface area if head/neck or hands/wrist involved w/o lower airway burns	0.151
Belize	1990	Amputation of lower limbs, bilateral	0.2192
Belize	1995	Amputation of lower limbs, bilateral	0.2123
Belize	2000	Amputation of lower limbs, bilateral	0.2121
Belize	2005	Amputation of lower limbs, bilateral	0.2034
Belize	2010	Amputation of lower limbs, bilateral	0.195
Belize	2017	Amputation of lower limbs, bilateral	0.1874
Belize	1990	Lower airway burns	0.2252
Belize	1995	Lower airway burns	0.2252
Belize	2000	Lower airway burns	0.2252
Belize	2005	Lower airway burns	0.2252
Belize	2010	Lower airway burns	0.2252
Belize	2017	Lower airway burns	0.2252
Belize	1990	Dislocation of hip	0.0159
Belize	1995	Dislocation of hip	0.0159
Belize	2000	Dislocation of hip	0.0159
Belize	2005	Dislocation of hip	0.0159
Belize	2010	Dislocation of hip	0.0159
Belize	2017	Dislocation of hip	0.0159
Belize	1990	Dislocation of knee	0.1134
Belize	1995	Dislocation of knee	0.1134
Belize	2000	Dislocation of knee	0.1134
Belize	2005	Dislocation of knee	0.1134
Belize	2010	Dislocation of knee	0.1134
Belize	2017	Dislocation of knee	0.1134
Belize	1990	Dislocation of shoulder	0.062
Belize	1995	Dislocation of shoulder	0.062
Belize	2000	Dislocation of shoulder	0.062
Belize	2005	Dislocation of shoulder	0.062
Belize	2010	Dislocation of shoulder	0.062
Belize	2017	Dislocation of shoulder	0.062
Belize	1990	Muscle and tendon injuries, including sprains and strains lesser dislocations	0.0075
Belize	1995	Muscle and tendon injuries, including sprains and strains lesser dislocations	0.0075
Belize	2000	Muscle and tendon injuries, including sprains and strains lesser dislocations	0.0075
Belize	2005	Muscle and tendon injuries, including sprains and strains lesser dislocations	0.0075
Belize	2010	Muscle and tendon injuries, including sprains and strains lesser dislocations	0.0075
Belize	2017	Muscle and tendon injuries, including sprains and strains lesser dislocations	0.0075
Belize	1990	Fracture of clavicle, scapula, or humerus	0.0349
Belize	1995	Fracture of clavicle, scapula, or humerus	0.0349
Belize	2000	Fracture of clavicle, scapula, or humerus	0.0349
Belize	2005	Fracture of clavicle, scapula, or humerus	0.0349
Belize	2010	Fracture of clavicle, scapula, or humerus	0.0349
Belize	2017	Fracture of clavicle, scapula, or humerus	0.0349
Belize	1990	Fracture of face bones	0.0669
Belize	1995	Fracture of face bones	0.0669
Belize	2000	Fracture of face bones	0.0669
Belize	2005	Fracture of face bones	0.0669
Belize	2010	Fracture of face bones	0.0669
Belize	2017	Fracture of face bones	0.0669
Belize	1990	Fracture of foot bones except ankle	0.0264
Belize	1995	Fracture of foot bones except ankle	0.0264
Belize	2000	Fracture of foot bones except ankle	0.0264
Belize	2005	Fracture of foot bones except ankle	0.0264
Belize	2010	Fracture of foot bones except ankle	0.0264
Belize	2017	Fracture of foot bones except ankle	0.0264
Belize	1990	Fracture of hand (wrist and other distal part of hand)	0.0138
Belize	1995	Fracture of hand (wrist and other distal part of hand)	0.0138
Belize	2000	Fracture of hand (wrist and other distal part of hand)	0.0138
Belize	2005	Fracture of hand (wrist and other distal part of hand)	0.0138
Belize	2010	Fracture of hand (wrist and other distal part of hand)	0.0138
Belize	2017	Fracture of hand (wrist and other distal part of hand)	0.0138
Belize	1990	Fracture of hip	0.1846
Belize	1995	Fracture of hip	0.1754
Belize	2000	Fracture of hip	0.1752
Belize	2005	Fracture of hip	0.1637
Belize	2010	Fracture of hip	0.1525
Belize	2017	Fracture of hip	0.1426
Belize	1990	Amputation of upper limbs, bilateral	0.2187
Belize	1995	Amputation of upper limbs, bilateral	0.2092
Belize	2000	Amputation of upper limbs, bilateral	0.209
Belize	2005	Amputation of upper limbs, bilateral	0.1971
Belize	2010	Amputation of upper limbs, bilateral	0.1856
Belize	2017	Amputation of upper limbs, bilateral	0.1753
Belize	1990	Fracture of patella, tibia or fibula, or ankle	0.0554
Belize	1995	Fracture of patella, tibia or fibula, or ankle	0.0554
Belize	2000	Fracture of patella, tibia or fibula, or ankle	0.0554
Belize	2005	Fracture of patella, tibia or fibula, or ankle	0.0554
Belize	2010	Fracture of patella, tibia or fibula, or ankle	0.0554
Belize	2017	Fracture of patella, tibia or fibula, or ankle	0.0554
Belize	1990	Fracture of pelvis	0.1823
Belize	1995	Fracture of pelvis	0.1823
Belize	2000	Fracture of pelvis	0.1823
Belize	2005	Fracture of pelvis	0.1823
Belize	2010	Fracture of pelvis	0.1823
Belize	2017	Fracture of pelvis	0.1823
Belize	1990	Fracture of radius and/or ulna	0.0435
Belize	1995	Fracture of radius and/or ulna	0.0435
Belize	2000	Fracture of radius and/or ulna	0.0435
Belize	2005	Fracture of radius and/or ulna	0.0435
Belize	2010	Fracture of radius and/or ulna	0.0435
Belize	2017	Fracture of radius and/or ulna	0.0435
Belize	1990	Fracture of skull	0.0714
Belize	1995	Fracture of skull	0.0714
Belize	2000	Fracture of skull	0.0714
Belize	2005	Fracture of skull	0.0714
Belize	2010	Fracture of skull	0.0714
Belize	2017	Fracture of skull	0.0714
Belize	1990	Fracture of sternum and/or fracture of one or more ribs	0.1027
Belize	1995	Fracture of sternum and/or fracture of one or more ribs	0.1027
Belize	2000	Fracture of sternum and/or fracture of one or more ribs	0.1027
Belize	2005	Fracture of sternum and/or fracture of one or more ribs	0.1027
Belize	2010	Fracture of sternum and/or fracture of one or more ribs	0.1027
Belize	2017	Fracture of sternum and/or fracture of one or more ribs	0.1027
Belize	1990	Fracture of vertebral column	0.1106
Belize	1995	Fracture of vertebral column	0.1106
Belize	2000	Fracture of vertebral column	0.1106
Belize	2005	Fracture of vertebral column	0.1106
Belize	2010	Fracture of vertebral column	0.1106
Belize	2017	Fracture of vertebral column	0.1106
Belize	1990	Fracture of femur, other than femoral neck	0.042
Belize	1995	Fracture of femur, other than femoral neck	0.042
Belize	2000	Fracture of femur, other than femoral neck	0.042
Belize	2005	Fracture of femur, other than femoral neck	0.042
Belize	2010	Fracture of femur, other than femoral neck	0.042
Belize	2017	Fracture of femur, other than femoral neck	0.042
Belize	1990	Minor TBI	0.1319
Belize	1995	Minor TBI	0.1319
Belize	2000	Minor TBI	0.1319
Belize	2005	Minor TBI	0.1319
Belize	2010	Minor TBI	0.1319
Belize	2017	Minor TBI	0.1319
Belize	1990	Moderate TBI	0.1637
Belize	1995	Moderate TBI	0.1637
Belize	2000	Moderate TBI	0.1637
Belize	2005	Moderate TBI	0.1637
Belize	2010	Moderate TBI	0.1637
Belize	2017	Moderate TBI	0.1637
Belize	1990	Amputation of fingers (excluding thumb)	0.0047
Belize	1995	Amputation of fingers (excluding thumb)	0.0047
Belize	2000	Amputation of fingers (excluding thumb)	0.0047
Belize	2005	Amputation of fingers (excluding thumb)	0.0047
Belize	2010	Amputation of fingers (excluding thumb)	0.0047
Belize	2017	Amputation of fingers (excluding thumb)	0.0047

Belize	1990	Spinal cord lesion at neck level	0.4557
Belize	1995	Spinal cord lesion at neck level	0.4525
Belize	2000	Spinal cord lesion at neck level	0.4525
Belize	2005	Spinal cord lesion at neck level	0.4485
Belize	2010	Spinal cord lesion at neck level	0.4446
Belize	2017	Spinal cord lesion at neck level	0.4412
Belize	1990	Spinal cord lesion below neck level	0.2703
Belize	1995	Spinal cord lesion below neck level	0.2636
Belize	2000	Spinal cord lesion below neck level	0.2635
Belize	2005	Spinal cord lesion below neck level	0.2551
Belize	2010	Spinal cord lesion below neck level	0.2469
Belize	2017	Spinal cord lesion below neck level	0.2396
Belize	1990	Drowning and nonfatal submersion	0.2471
Belize	1995	Drowning and nonfatal submersion	0.2471
Belize	2000	Drowning and nonfatal submersion	0.2471
Belize	2005	Drowning and nonfatal submersion	0.2471
Belize	2010	Drowning and nonfatal submersion	0.2471
Belize	2017	Drowning and nonfatal submersion	0.2471
Belize	1990	Asphyxiation	0.2471
Belize	1995	Asphyxiation	0.2471
Belize	2000	Asphyxiation	0.2471
Belize	2005	Asphyxiation	0.2471
Belize	2010	Asphyxiation	0.2471
Belize	2017	Asphyxiation	0.2471
Belize	1990	Crush injury	0.1325
Belize	1995	Crush injury	0.1325
Belize	2000	Crush injury	0.1325
Belize	2005	Crush injury	0.1325
Belize	2010	Crush injury	0.1325
Belize	2017	Crush injury	0.1325
Belize	1990	Nerve injury	0.1129
Belize	1995	Nerve injury	0.1129
Belize	2000	Nerve injury	0.1129
Belize	2005	Nerve injury	0.1129
Belize	2010	Nerve injury	0.1129
Belize	2017	Nerve injury	0.1129
Belize	1990	Injury to eyes	0.0165
Belize	1995	Injury to eyes	0.0165
Belize	2000	Injury to eyes	0.0165
Belize	2005	Injury to eyes	0.0165
Belize	2010	Injury to eyes	0.0165
Belize	2017	Injury to eyes	0.0165
Belize	1990	Amputation of lower limb, unilateral	0.0884
Belize	1995	Amputation of lower limb, unilateral	0.0848
Belize	2000	Amputation of lower limb, unilateral	0.0848
Belize	2005	Amputation of lower limb, unilateral	0.0803
Belize	2010	Amputation of lower limb, unilateral	0.0759
Belize	2017	Amputation of lower limb, unilateral	0.072
Belize	1990	Open wound(s)	0.011
Belize	1995	Open wound(s)	0.011
Belize	2000	Open wound(s)	0.011
Belize	2005	Open wound(s)	0.011
Belize	2010	Open wound(s)	0.011
Belize	2017	Open wound(s)	0.011
Belize	1990	Poisoning requiring urgent care	0.1628
Belize	1995	Poisoning requiring urgent care	0.1628
Belize	2000	Poisoning requiring urgent care	0.1628
Belize	2005	Poisoning requiring urgent care	0.1628
Belize	2010	Poisoning requiring urgent care	0.1628
Belize	2017	Poisoning requiring urgent care	0.1628
Belize	1990	Severe chest injury	0.0474
Belize	1995	Severe chest injury	0.0474
Belize	2000	Severe chest injury	0.0474
Belize	2005	Severe chest injury	0.0474
Belize	2010	Severe chest injury	0.0474
Belize	2017	Severe chest injury	0.0474
Belize	1990	Internal hemorrhage in abdomen and pelvis	0.1143
Belize	1995	Internal hemorrhage in abdomen and pelvis	0.1143
Belize	2000	Internal hemorrhage in abdomen and pelvis	0.1143
Belize	2005	Internal hemorrhage in abdomen and pelvis	0.1143
Belize	2010	Internal hemorrhage in abdomen and pelvis	0.1143
Belize	2017	Internal hemorrhage in abdomen and pelvis	0.1143
Belize	1990	Contusion in any part of the body	0.0075
Belize	1995	Contusion in any part of the body	0.0075
Belize	2000	Contusion in any part of the body	0.0075
Belize	2005	Contusion in any part of the body	0.0075
Belize	2010	Contusion in any part of the body	0.0075
Belize	2017	Contusion in any part of the body	0.0075
Belize	1990	Effect of different environmental factors	0.049
Belize	1995	Effect of different environmental factors	0.049
Belize	2000	Effect of different environmental factors	0.049
Belize	2005	Effect of different environmental factors	0.049
Belize	2010	Effect of different environmental factors	0.049
Belize	2017	Effect of different environmental factors	0.049
Belize	1990	Complications following therapeutic procedures	0.0506
Belize	1995	Complications following therapeutic procedures	0.0506
Belize	2000	Complications following therapeutic procedures	0.0506
Belize	2005	Complications following therapeutic procedures	0.0506
Belize	2010	Complications following therapeutic procedures	0.0506
Belize	2017	Complications following therapeutic procedures	0.0506
Belize	1990	Multiple fractures, dislocations, crashes, wounds, sprains, and strains	0.1846
Belize	1995	Multiple fractures, dislocations, crashes, wounds, sprains, and strains	0.1754
Belize	2000	Multiple fractures, dislocations, crashes, wounds, sprains, and strains	0.1752
Belize	2005	Multiple fractures, dislocations, crashes, wounds, sprains, and strains	0.1637
Belize	2010	Multiple fractures, dislocations, crashes, wounds, sprains, and strains	0.1525
Belize	2017	Multiple fractures, dislocations, crashes, wounds, sprains, and strains	0.1426
Belize	1990	Amputation of upper limb, unilateral	0.0681
Belize	1995	Amputation of upper limb, unilateral	0.066
Belize	2000	Amputation of upper limb, unilateral	0.066
Belize	2005	Amputation of upper limb, unilateral	0.0633
Belize	2010	Amputation of upper limb, unilateral	0.0607
Belize	2017	Amputation of upper limb, unilateral	0.0585
Belize	1990	Amputation of thumb	0.0114
Belize	1995	Amputation of thumb	0.0114
Belize	2000	Amputation of thumb	0.0114
Belize	2005	Amputation of thumb	0.0114
Belize	2010	Amputation of thumb	0.0114
Belize	2017	Amputation of thumb	0.0114
Belize	1990	Amputation of toe/toes	0.0059
Belize	1995	Amputation of toe/toes	0.0059
Belize	2000	Amputation of toe/toes	0.0059
Belize	2005	Amputation of toe/toes	0.0059
Belize	2010	Amputation of toe/toes	0.0059
Belize	2017	Amputation of toe/toes	0.0059
Belize	1990	Burns, <20% total burned surface area without lower airway burns	0.0163
Belize	1995	Burns, <20% total burned surface area without lower airway burns	0.0163
Belize	2000	Burns, <20% total burned surface area without lower airway burns	0.0163
Belize	2005	Burns, <20% total burned surface area without lower airway burns	0.0163
Belize	2010	Burns, <20% total burned surface area without lower airway burns	0.0163
Belize	2017	Burns, <20% total burned surface area without lower airway burns	0.0163
Belize	1990	Burns, >=20% total burned surface area or >= 10% burned surface area if head/neck or hands/wrist involved w/o lower airway burns	0.2527
Belize	1995	Burns, >=20% total burned surface area or >= 10% burned surface area if head/neck or hands/wrist involved w/o lower airway burns	0.2442
Belize	2000	Burns, >=20% total burned surface area or >= 10% burned surface area if head/neck or hands/wrist involved w/o lower airway burns	0.2441
Belize	2005	Burns, >=20% total burned surface area or >= 10% burned surface area if head/neck or hands/wrist involved w/o lower airway burns	0.2333
Belize	2010	Burns, >=20% total burned surface area or >= 10% burned surface area if head/neck or hands/wrist involved w/o lower airway burns	0.2229
Belize	2017	Burns, >=20% total burned surface area or >= 10% burned surface area if head/neck or hands/wrist involved w/o lower airway burns	0.2136
Cuba	1990	Amputation of lower limbs, bilateral	0.1615
Cuba	1995	Amputation of lower limbs, bilateral	0.1564
Cuba	2000	Amputation of lower limbs, bilateral	0.1494
Cuba	2005	Amputation of lower limbs, bilateral	0.1378
Cuba	2010	Amputation of lower limbs, bilateral	0.1319
Cuba	2017	Amputation of lower limbs, bilateral	0.1233
Cuba	1990	Lower airway burns	0.2252
Cuba	1995	Lower airway burns	0.2252
Cuba	2000	Lower airway burns	0.2252
Cuba	2005	Lower airway burns	0.2252
Cuba	2010	Lower airway burns	0.2252
Cuba	2017	Lower airway burns	0.2252
Cuba	1990	Dislocation of hip	0.0159
Cuba	1995	Dislocation of hip	0.0159
Cuba	2000	Dislocation of hip	0.0159
Cuba	2005	Dislocation of hip	0.0159
Cuba	2010	Dislocation of hip	0.0159
Cuba	2017	Dislocation of hip	0.0159
Cuba	1990	Dislocation of knee	0.1134
Cuba	1995	Dislocation of knee	0.1134
Cuba	2000	Dislocation of knee	0.1134
Cuba	2005	Dislocation of knee	0.1134
Cuba	2010	Dislocation of knee	0.1134
Cuba	2017	Dislocation of knee	0.1134
Cuba	1990	Dislocation of shoulder	0.062
Cuba	1995	Dislocation of shoulder	0.062
Cuba	2000	Dislocation of shoulder	0.062
Cuba	2005	Dislocation of shoulder	0.062
Cuba	2010	Dislocation of shoulder	0.062
Cuba	2017	Dislocation of shoulder	0.062
Cuba	1990	Muscle and tendon injuries, including sprains and strains lesser dislocations	0.0075
Cuba	1995	Muscle and tendon injuries, including sprains and strains lesser dislocations	0.0075
Cuba	2000	Muscle and tendon injuries, including sprains and strains lesser dislocations	0.0075
Cuba	2005	Muscle and tendon injuries, including sprains and strains lesser dislocations	0.0075
Cuba	2010	Muscle and tendon injuries, including sprains and strains lesser dislocations	0.0075
Cuba	2017	Muscle and tendon injuries, including sprains and strains lesser dislocations	0.0075
Cuba	1990	Fracture of clavicle, scapula, or humerus	0.0349
Cuba	1995	Fracture of clavicle, scapula, or humerus	0.0349
Cuba	2000	Fracture of clavicle, scapula, or humerus	0.0349
Cuba	2005	Fracture of clavicle, scapula, or humerus	0.0349
Cuba	2010	Fracture of clavicle, scapula, or humerus	0.0349
Cuba	2017	Fracture of clavicle, scapula, or humerus	0.0349
Cuba	1990	Fracture of face bones	0.0669
Cuba	1995	Fracture of face bones	0.0669
Cuba	2000	Fracture of face bones	0.0669
Cuba	2005	Fracture of face bones	0.0669
Cuba	2010	Fracture of face bones	0.0669
Cuba	2017	Fracture of face bones	0.0669
Cuba	1990	Fracture of foot bones except ankle	0.0264
Cuba	1995	Fracture of foot bones except ankle	0.0264
Cuba	2000	Fracture of foot bones except ankle	0.0264
Cuba	2005	Fracture of foot bones except ankle	0.0264
Cuba	2010	Fracture of foot bones except ankle	0.0264
Cuba	2017	Fracture of foot bones except ankle	0.0264
Cuba	1990	Fracture of hand (wrist and other distal part of hand)	0.0138
Cuba	1995	Fracture of hand (wrist and other distal part of hand)	0.0138
Cuba	2000	Fracture of hand (wrist and other distal part of hand)	0.0138
Cuba	2005	Fracture of hand (wrist and other distal part of hand)	0.0138
Cuba	2010	Fracture of hand (wrist and other distal part of hand)	0.0138
Cuba	2017	Fracture of hand (wrist and other distal part of hand)	0.0138
Cuba	1990	Fracture of hip	0.1082
Cuba	1995	Fracture of hip	0.1015
Cuba	2000	Fracture of hip	0.0923
Cuba	2005	Fracture of hip	0.0769
Cuba	2010	Fracture of hip	0.0691
Cuba	2017	Fracture of hip	0.0577
Cuba	1990	Amputation of upper limbs, bilateral	0.1398
Cuba	1995	Amputation of upper limbs, bilateral	0.1329
Cuba	2000	Amputation of upper limbs, bilateral	0.1234
Cuba	2005	Amputation of upper limbs, bilateral	0.1075
Cuba	2010	Amputation of upper limbs, bilateral	0.0995
Cuba	2017	Amputation of upper limbs, bilateral	0.0876
Cuba	1990	Fracture of patella, tibia or fibula, or ankle	0.0554
Cuba	1995	Fracture of patella, tibia or fibula, or ankle	0.0554
Cuba	2000	Fracture of patella, tibia or fibula, or ankle	0.0554
Cuba	2005	Fracture of patella, tibia or fibula, or ankle	0.0554
Cuba	2010	Fracture of patella, tibia or fibula, or ankle	0.0554
Cuba	2017	Fracture of patella, tibia or fibula, or ankle	0.0554
Cuba	1990	Fracture of pelvis	0.1823
Cuba	1995	Fracture of pelvis	0.1823
Cuba	2000	Fracture of pelvis	0.1823
Cuba	2005	Fracture of pelvis	0.1823
Cuba	2010	Fracture of pelvis	0.1823
Cuba	2017	Fracture of pelvis	0.1823
Cuba	1990	Fracture of radius and/or ulna	0.0435
Cuba	1995	Fracture of radius and/or ulna	0.0435
Cuba	2000	Fracture of radius and/or ulna	0.0435
Cuba	2005	Fracture of radius and/or ulna	0.0435
Cuba	2010	Fracture of radius and/or ulna	0.0435
Cuba	2017	Fracture of radius and/or ulna	0.0435
Cuba	1990	Fracture of skull	0.0714
Cuba	1995	Fracture of skull	0.0714
Cuba	2000	Fracture of skull	0.0714
Cuba	2005	Fracture of skull	0.0714
Cuba	2010	Fracture of skull	0.0714
Cuba	2017	Fracture of skull	0.0714
Cuba	1990	Fracture of sternum and/or fracture of one or more ribs	0.1027
Cuba	1995	Fracture of sternum and/or fracture of one or more ribs	0.1027
Cuba	2000	Fracture of sternum and/or fracture of one or more ribs	0.1027
Cuba	2005	Fracture of sternum and/or fracture of one or more ribs	0.1027
Cuba	2010	Fracture of sternum and/or fracture of one or more ribs	0.1027
Cuba	2017	Fracture of sternum and/or fracture of one or more ribs	0.1027
Cuba	1990	Fracture of vertebral column	0.1106
Cuba	1995	Fracture of vertebral column	0.1106
Cuba	2000	Fracture of vertebral column	0.1106
Cuba	2005	Fracture of vertebral column	0.1106
Cuba	2010	Fracture of vertebral column	0.1106
Cuba	2017	Fracture of vertebral column	0.1106
Cuba	1990	Fracture of femur, other than femoral neck	0.042
Cuba	1995	Fracture of femur, other than femoral neck	0.042
Cuba	2000	Fracture of femur, other than femoral neck	0.042
Cuba	2005	Fracture of femur, other than femoral neck	0.042
Cuba	2010	Fracture of femur, other than femoral neck	0.042
Cuba	2017	Fracture of femur, other than femoral neck	0.042
Cuba	1990	Minor TBI	0.1319
Cuba	1995	Minor TBI	0.1319
Cuba	2000	Minor TBI	0.1319
Cuba	2005	Minor TBI	0.1319
Cuba	2010	Minor TBI	0.1319
Cuba	2017	Minor TBI	0.1319
Cuba	1990	Moderate TBI	0.1637
Cuba	1995	Moderate TBI	0.1637
Cuba	2000	Moderate TBI	0.1637
Cuba	2005	Moderate TBI	0.1637
Cuba	2010	Moderate TBI	0.1637
Cuba	2017	Moderate TBI	0.1637
Cuba	1990	Amputation of fingers (excluding thumb)	0.0047
Cuba	1995	Amputation of fingers (excluding thumb)	0.0047
Cuba	2000	Amputation of fingers (excluding thumb)	0.0047
Cuba	2005	Amputation of fingers (excluding thumb)	0.0047
Cuba	2010	Amputation of fingers (excluding thumb)	0.0047
Cuba	2017	Amputation of fingers (excluding thumb)	0.0047
Cuba	1990	Spinal cord lesion at neck level	0.4293
Cuba	1995	Spinal cord lesion at neck level	0.427
Cuba	2000	Spinal cord lesion at neck level	0.4238
Cuba	2005	Spinal cord lesion at neck level	0.4185
Cuba	2010	Spinal cord lesion at neck level	0.4158
Cuba	2017	Spinal cord lesion at neck level	0.4118
Cuba	1990	Spinal cord lesion below neck level	0.2146
Cuba	1995	Spinal cord lesion below neck level	0.2097
Cuba	2000	Spinal cord lesion below neck level	0.2029
Cuba	2005	Spinal cord lesion below neck level	0.1917
Cuba	2010	Spinal cord lesion below neck level	0.186
Cuba	2017	Spinal cord lesion below neck level	0.1777
Cuba	1990	Drowning and nonfatal submersion	0.2471
Cuba	1995	Drowning and nonfatal submersion	0.2471
Cuba	2000	Drowning and nonfatal submersion	0.2471
Cuba	2005	Drowning and nonfatal submersion	0.2471
Cuba	2010	Drowning and nonfatal submersion	0.2471
Cuba	2017	Drowning and nonfatal submersion	0.2471
Cuba	1990	Asphyxiation	0.2471
Cuba	1995	Asphyxiation	0.2471
Cuba	2000	Asphyxiation	0.2471
Cuba	2005	Asphyxiation	0.2471
Cuba	2010	Asphyxiation	0.2471
Cuba	2017	Asphyxiation	0.2471
Cuba	1990	Crush injury	0.1325
Cuba	1995	Crush injury	0.1325
Cuba	2000	Crush injury	0.1325
Cuba	2005	Crush injury	0.1325
Cuba	2010	Crush injury	0.1325
Cuba	2017	Crush injury	0.1325
Cuba	1990	Nerve injury	0.1129
Cuba	1995	Nerve injury	0.1129
Cuba	2000	Nerve injury	0.1129
Cuba	2005	Nerve injury	0.1129
Cuba	2010	Nerve injury	0.1129
Cuba	2017	Nerve injury	0.1129
Cuba	1990	Injury to eyes	0.0165
Cuba	1995	Injury to eyes	0.0165
Cuba	2000	Injury to eyes	0.0165
Cuba	2005	Injury to eyes	0.0165
Cuba	2010	Injury to eyes	0.0165
Cuba	2017	Injury to eyes	0.0165
Cuba	1990	Amputation of lower limb, unilateral	0.0587
Cuba	1995	Amputation of lower limb, unilateral	0.0561
Cuba	2000	Amputation of lower limb, unilateral	0.0525
Cuba	2005	Amputation of lower limb, unilateral	0.0464
Cuba	2010	Amputation of lower limb, unilateral	0.0434
Cuba	2017	Amputation of lower limb, unilateral	0.039
Cuba	1990	Open wound(s)	0.011
Cuba	1995	Open wound(s)	0.011
Cuba	2000	Open wound(s)	0.011
Cuba	2005	Open wound(s)	0.011
Cuba	2010	Open wound(s)	0.011
Cuba	2017	Open wound(s)	0.011
Cuba	1990	Poisoning requiring urgent care	0.1628
Cuba	1995	Poisoning requiring urgent care	0.1628
Cuba	2000	Poisoning requiring urgent care	0.1628
Cuba	2005	Poisoning requiring urgent care	0.1628
Cuba	2010	Poisoning requiring urgent care	0.1628
Cuba	2017	Poisoning requiring urgent care	0.1628
Cuba	1990	Severe chest injury	0.0474
Cuba	1995	Severe chest injury	0.0474
Cuba	2000	Severe chest injury	0.0474
Cuba	2005	Severe chest injury	0.0474
Cuba	2010	Severe chest injury	0.0474
Cuba	2017	Severe chest injury	0.0474
Cuba	1990	Internal hemorrhage in abdomen and pelvis	0.1143
Cuba	1995	Internal hemorrhage in abdomen and pelvis	0.1143
Cuba	2000	Internal hemorrhage in abdomen and pelvis	0.1143
Cuba	2005	Internal hemorrhage in abdomen and pelvis	0.1143
Cuba	2010	Internal hemorrhage in abdomen and pelvis	0.1143
Cuba	2017	Internal hemorrhage in abdomen and pelvis	0.1143
Cuba	1990	Contusion in any part of the body	0.0075
Cuba	1995	Contusion in any part of the body	0.0075
Cuba	2000	Contusion in any part of the body	0.0075
Cuba	2005	Contusion in any part of the body	0.0075
Cuba	2010	Contusion in any part of the body	0.0075
Cuba	2017	Contusion in any part of the body	0.0075
Cuba	1990	Effect of different environmental factors	0.049
Cuba	1995	Effect of different environmental factors	0.049
Cuba	2000	Effect of different environmental factors	0.049
Cuba	2005	Effect of different environmental factors	0.049
Cuba	2010	Effect of different environmental factors	0.049
Cuba	2017	Effect of different environmental factors	0.049
Cuba	1990	Complications following therapeutic procedures	0.0506
Cuba	1995	Complications following therapeutic procedures	0.0506
Cuba	2000	Complications following therapeutic procedures	0.0506
Cuba	2005	Complications following therapeutic procedures	0.0506
Cuba	2010	Complications following therapeutic procedures	0.0506
Cuba	2017	Complications following therapeutic procedures	0.0506
Cuba	1990	Multiple fractures, dislocations, crashes, wounds, sprains, and strains	0.1082
Cuba	1995	Multiple fractures, dislocations, crashes, wounds, sprains, and strains	0.1015
Cuba	2000	Multiple fractures, dislocations, crashes, wounds, sprains, and strains	0.0923
Cuba	2005	Multiple fractures, dislocations, crashes, wounds, sprains, and strains	0.0769
Cuba	2010	Multiple fractures, dislocations, crashes, wounds, sprains, and strains	0.0691
Cuba	2017	Multiple fractures, dislocations, crashes, wounds, sprains, and strains	0.0577
Cuba	1990	Amputation of upper limb, unilateral	0.0506
Cuba	1995	Amputation of upper limb, unilateral	0.049
Cuba	2000	Amputation of upper limb, unilateral	0.0469
Cuba	2005	Amputation of upper limb, unilateral	0.0434
Cuba	2010	Amputation of upper limb, unilateral	0.0416
Cuba	2017	Amputation of upper limb, unilateral	0.039
Cuba	1990	Amputation of thumb	0.0114
Cuba	1995	Amputation of thumb	0.0114
Cuba	2000	Amputation of thumb	0.0114
Cuba	2005	Amputation of thumb	0.0114
Cuba	2010	Amputation of thumb	0.0114
Cuba	2017	Amputation of thumb	0.0114
Cuba	1990	Amputation of toe/toes	0.0059
Cuba	1995	Amputation of toe/toes	0.0059
Cuba	2000	Amputation of toe/toes	0.0059
Cuba	2005	Amputation of toe/toes	0.0059
Cuba	2010	Amputation of toe/toes	0.0059
Cuba	2017	Amputation of toe/toes	0.0059
Cuba	1990	Burns, <20% total burned surface area without lower airway burns	0.0163
Cuba	1995	Burns, <20% total burned surface area without lower airway burns	0.0163
Cuba	2000	Burns, <20% total burned surface area without lower airway burns	0.0163
Cuba	2005	Burns, <20% total burned surface area without lower airway burns	0.0163
Cuba	2010	Burns, <20% total burned surface area without lower airway burns	0.0163
Cuba	2017	Burns, <20% total burned surface area without lower airway burns	0.0163
Cuba	1990	Burns, >=20% total burned surface area or >= 10% burned surface area if head/neck or hands/wrist involved w/o lower airway burns	0.1816
Cuba	1995	Burns, >=20% total burned surface area or >= 10% burned surface area if head/neck or hands/wrist involved w/o lower airway burns	0.1754
Cuba	2000	Burns, >=20% total burned surface area or >= 10% burned surface area if head/neck or hands/wrist involved w/o lower airway burns	0.1668
Cuba	2005	Burns, >=20% total burned surface area or >= 10% burned surface area if head/neck or hands/wrist involved w/o lower airway burns	0.1524
Cuba	2010	Burns, >=20% total burned surface area or >= 10% burned surface area if head/neck or hands/wrist involved w/o lower airway burns	0.1452
Cuba	2017	Burns, >=20% total burned surface area or >= 10% burned surface area if head/neck or hands/wrist involved w/o lower airway burns	0.1346
Dominica	1990	Amputation of lower limbs, bilateral	0.1997
Dominica	1995	Amputation of lower limbs, bilateral	0.1932
Dominica	2000	Amputation of lower limbs, bilateral	0.1777
Dominica	2005	Amputation of lower limbs, bilateral	0.1701
Dominica	2010	Amputation of lower limbs, bilateral	0.1712
Dominica	2017	Amputation of lower limbs, bilateral	0.1662
Dominica	1990	Lower airway burns	0.2252
Dominica	1995	Lower airway burns	0.2252
Dominica	2000	Lower airway burns	0.2252
Dominica	2005	Lower airway burns	0.2252
Dominica	2010	Lower airway burns	0.2252
Dominica	2017	Lower airway burns	0.2252
Dominica	1990	Dislocation of hip	0.0159
Dominica	1995	Dislocation of hip	0.0159
Dominica	2000	Dislocation of hip	0.0159
Dominica	2005	Dislocation of hip	0.0159
Dominica	2010	Dislocation of hip	0.0159
Dominica	2017	Dislocation of hip	0.0159
Dominica	1990	Dislocation of knee	0.1134
Dominica	1995	Dislocation of knee	0.1134
Dominica	2000	Dislocation of knee	0.1134
Dominica	2005	Dislocation of knee	0.1134
Dominica	2010	Dislocation of knee	0.1134
Dominica	2017	Dislocation of knee	0.1134
Dominica	1990	Dislocation of shoulder	0.062
Dominica	1995	Dislocation of shoulder	0.062
Dominica	2000	Dislocation of shoulder	0.062
Dominica	2005	Dislocation of shoulder	0.062
Dominica	2010	Dislocation of shoulder	0.062
Dominica	2017	Dislocation of shoulder	0.062
Dominica	1990	Muscle and tendon injuries, including sprains and strains lesser dislocations	0.0075
Dominica	1995	Muscle and tendon injuries, including sprains and strains lesser dislocations	0.0075
Dominica	2000	Muscle and tendon injuries, including sprains and strains lesser dislocations	0.0075
Dominica	2005	Muscle and tendon injuries, including sprains and strains lesser dislocations	0.0075
Dominica	2010	Muscle and tendon injuries, including sprains and strains lesser dislocations	0.0075
Dominica	2017	Muscle and tendon injuries, including sprains and strains lesser dislocations	0.0075
Dominica	1990	Fracture of clavicle, scapula, or humerus	0.0349
Dominica	1995	Fracture of clavicle, scapula, or humerus	0.0349
Dominica	2000	Fracture of clavicle, scapula, or humerus	0.0349
Dominica	2005	Fracture of clavicle, scapula, or humerus	0.0349
Dominica	2010	Fracture of clavicle, scapula, or humerus	0.0349
Dominica	2017	Fracture of clavicle, scapula, or humerus	0.0349
Dominica	1990	Fracture of face bones	0.0669
Dominica	1995	Fracture of face bones	0.0669
Dominica	2000	Fracture of face bones	0.0669
Dominica	2005	Fracture of face bones	0.0669
Dominica	2010	Fracture of face bones	0.0669

Dominica	2017	Fracture of face bones	0.0669
Dominica	1990	Fracture of foot bones except ankle	0.0264
Dominica	1995	Fracture of foot bones except ankle	0.0264
Dominica	2000	Fracture of foot bones except ankle	0.0264
Dominica	2005	Fracture of foot bones except ankle	0.0264
Dominica	2010	Fracture of foot bones except ankle	0.0264
Dominica	2017	Fracture of foot bones except ankle	0.0264
Dominica	1990	Fracture of hand (wrist and other distal part of hand)	0.0138
Dominica	1995	Fracture of hand (wrist and other distal part of hand)	0.0138
Dominica	2000	Fracture of hand (wrist and other distal part of hand)	0.0138
Dominica	2005	Fracture of hand (wrist and other distal part of hand)	0.0138
Dominica	2010	Fracture of hand (wrist and other distal part of hand)	0.0138
Dominica	2017	Fracture of hand (wrist and other distal part of hand)	0.0138
Dominica	1990	Fracture of hip	0.1588
Dominica	1995	Fracture of hip	0.1502
Dominica	2000	Fracture of hip	0.1297
Dominica	2005	Fracture of hip	0.1196
Dominica	2010	Fracture of hip	0.1211
Dominica	2017	Fracture of hip	0.1144
Dominica	1990	Amputation of upper limbs, bilateral	0.1921
Dominica	1995	Amputation of upper limbs, bilateral	0.1832
Dominica	2000	Amputation of upper limbs, bilateral	0.162
Dominica	2005	Amputation of upper limbs, bilateral	0.1516
Dominica	2010	Amputation of upper limbs, bilateral	0.1531
Dominica	2017	Amputation of upper limbs, bilateral	0.1462
Dominica	1990	Fracture of patella, tibia or fibula, or ankle	0.0554
Dominica	1995	Fracture of patella, tibia or fibula, or ankle	0.0554
Dominica	2000	Fracture of patella, tibia or fibula, or ankle	0.0554
Dominica	2005	Fracture of patella, tibia or fibula, or ankle	0.0554
Dominica	2010	Fracture of patella, tibia or fibula, or ankle	0.0554
Dominica	2017	Fracture of patella, tibia or fibula, or ankle	0.0554
Dominica	1990	Fracture of pelvis	0.1823
Dominica	1995	Fracture of pelvis	0.1823
Dominica	2000	Fracture of pelvis	0.1823
Dominica	2005	Fracture of pelvis	0.1823
Dominica	2010	Fracture of pelvis	0.1823
Dominica	2017	Fracture of pelvis	0.1823
Dominica	1990	Fracture of radius and/or ulna	0.0435
Dominica	1995	Fracture of radius and/or ulna	0.0435
Dominica	2000	Fracture of radius and/or ulna	0.0435
Dominica	2005	Fracture of radius and/or ulna	0.0435
Dominica	2010	Fracture of radius and/or ulna	0.0435
Dominica	2017	Fracture of radius and/or ulna	0.0435
Dominica	1990	Fracture of skull	0.0714
Dominica	1995	Fracture of skull	0.0714
Dominica	2000	Fracture of skull	0.0714
Dominica	2005	Fracture of skull	0.0714
Dominica	2010	Fracture of skull	0.0714
Dominica	2017	Fracture of skull	0.0714
Dominica	1990	Fracture of sternum and/or fracture of one or more ribs	0.1027
Dominica	1995	Fracture of sternum and/or fracture of one or more ribs	0.1027
Dominica	2000	Fracture of sternum and/or fracture of one or more ribs	0.1027
Dominica	2005	Fracture of sternum and/or fracture of one or more ribs	0.1027
Dominica	2010	Fracture of sternum and/or fracture of one or more ribs	0.1027
Dominica	2017	Fracture of sternum and/or fracture of one or more ribs	0.1027
Dominica	1990	Fracture of vertebral column	0.1106
Dominica	1995	Fracture of vertebral column	0.1106
Dominica	2000	Fracture of vertebral column	0.1106
Dominica	2005	Fracture of vertebral column	0.1106
Dominica	2010	Fracture of vertebral column	0.1106
Dominica	2017	Fracture of vertebral column	0.1106
Dominica	1990	Fracture of femur, other than femoral neck	0.042
Dominica	1995	Fracture of femur, other than femoral neck	0.042
Dominica	2000	Fracture of femur, other than femoral neck	0.042
Dominica	2005	Fracture of femur, other than femoral neck	0.042
Dominica	2010	Fracture of femur, other than femoral neck	0.042
Dominica	2017	Fracture of femur, other than femoral neck	0.042
Dominica	1990	Minor TBI	0.1319
Dominica	1995	Minor TBI	0.1319
Dominica	2000	Minor TBI	0.1319
Dominica	2005	Minor TBI	0.1319
Dominica	2010	Minor TBI	0.1319
Dominica	2017	Minor TBI	0.1319
Dominica	1990	Moderate TBI	0.1637
Dominica	1995	Moderate TBI	0.1637
Dominica	2000	Moderate TBI	0.1637
Dominica	2005	Moderate TBI	0.1637
Dominica	2010	Moderate TBI	0.1637
Dominica	2017	Moderate TBI	0.1637
Dominica	1990	Amputation of fingers (excluding thumb)	0.0047
Dominica	1995	Amputation of fingers (excluding thumb)	0.0047
Dominica	2000	Amputation of fingers (excluding thumb)	0.0047
Dominica	2005	Amputation of fingers (excluding thumb)	0.0047
Dominica	2010	Amputation of fingers (excluding thumb)	0.0047
Dominica	2017	Amputation of fingers (excluding thumb)	0.0047
Dominica	1990	Spinal cord lesion at neck level	0.4468
Dominica	1995	Spinal cord lesion at neck level	0.4438
Dominica	2000	Spinal cord lesion at neck level	0.4367
Dominica	2005	Spinal cord lesion at neck level	0.4332
Dominica	2010	Spinal cord lesion at neck level	0.4337
Dominica	2017	Spinal cord lesion at neck level	0.4314
Dominica	1990	Spinal cord lesion below neck level	0.2515
Dominica	1995	Spinal cord lesion below neck level	0.2452
Dominica	2000	Spinal cord lesion below neck level	0.2302
Dominica	2005	Spinal cord lesion below neck level	0.2229
Dominica	2010	Spinal cord lesion below neck level	0.224
Dominica	2017	Spinal cord lesion below neck level	0.2191
Dominica	1990	Drowning and nonfatal submersion	0.2471
Dominica	1995	Drowning and nonfatal submersion	0.2471
Dominica	2000	Drowning and nonfatal submersion	0.2471
Dominica	2005	Drowning and nonfatal submersion	0.2471
Dominica	2010	Drowning and nonfatal submersion	0.2471
Dominica	2017	Drowning and nonfatal submersion	0.2471
Dominica	1990	Asphyxiation	0.2471
Dominica	1995	Asphyxiation	0.2471
Dominica	2000	Asphyxiation	0.2471
Dominica	2005	Asphyxiation	0.2471
Dominica	2010	Asphyxiation	0.2471
Dominica	2017	Asphyxiation	0.2471
Dominica	1990	Crush injury	0.1325
Dominica	1995	Crush injury	0.1325
Dominica	2000	Crush injury	0.1325
Dominica	2005	Crush injury	0.1325
Dominica	2010	Crush injury	0.1325
Dominica	2017	Crush injury	0.1325
Dominica	1990	Nerve injury	0.1129
Dominica	1995	Nerve injury	0.1129
Dominica	2000	Nerve injury	0.1129
Dominica	2005	Nerve injury	0.1129
Dominica	2010	Nerve injury	0.1129
Dominica	2017	Nerve injury	0.1129
Dominica	1990	Injury to eyes	0.0165
Dominica	1995	Injury to eyes	0.0165
Dominica	2000	Injury to eyes	0.0165
Dominica	2005	Injury to eyes	0.0165
Dominica	2010	Injury to eyes	0.0165
Dominica	2017	Injury to eyes	0.0165
Dominica	1990	Amputation of lower limb, unilateral	0.0784
Dominica	1995	Amputation of lower limb, unilateral	0.075
Dominica	2000	Amputation of lower limb, unilateral	0.067
Dominica	2005	Amputation of lower limb, unilateral	0.0631
Dominica	2010	Amputation of lower limb, unilateral	0.0637
Dominica	2017	Amputation of lower limb, unilateral	0.0611
Dominica	1990	Open wound(s)	0.011
Dominica	1995	Open wound(s)	0.011
Dominica	2000	Open wound(s)	0.011
Dominica	2005	Open wound(s)	0.011
Dominica	2010	Open wound(s)	0.011
Dominica	2017	Open wound(s)	0.011
Dominica	1990	Poisoning requiring urgent care	0.1628
Dominica	1995	Poisoning requiring urgent care	0.1628
Dominica	2000	Poisoning requiring urgent care	0.1628
Dominica	2005	Poisoning requiring urgent care	0.1628
Dominica	2010	Poisoning requiring urgent care	0.1628
Dominica	2017	Poisoning requiring urgent care	0.1628
Dominica	1990	Severe chest injury	0.0474
Dominica	1995	Severe chest injury	0.0474
Dominica	2000	Severe chest injury	0.0474
Dominica	2005	Severe chest injury	0.0474
Dominica	2010	Severe chest injury	0.0474
Dominica	2017	Severe chest injury	0.0474
Dominica	1990	Internal hemorrhage in abdomen and pelvis	0.1143
Dominica	1995	Internal hemorrhage in abdomen and pelvis	0.1143
Dominica	2000	Internal hemorrhage in abdomen and pelvis	0.1143
Dominica	2005	Internal hemorrhage in abdomen and pelvis	0.1143
Dominica	2010	Internal hemorrhage in abdomen and pelvis	0.1143
Dominica	2017	Internal hemorrhage in abdomen and pelvis	0.1143
Dominica	1990	Contusion in any part of the body	0.0075
Dominica	1995	Contusion in any part of the body	0.0075
Dominica	2000	Contusion in any part of the body	0.0075
Dominica	2005	Contusion in any part of the body	0.0075
Dominica	2010	Contusion in any part of the body	0.0075
Dominica	2017	Contusion in any part of the body	0.0075
Dominica	1990	Effect of different environmental factors	0.049
Dominica	1995	Effect of different environmental factors	0.049
Dominica	2000	Effect of different environmental factors	0.049
Dominica	2005	Effect of different environmental factors	0.049
Dominica	2010	Effect of different environmental factors	0.049
Dominica	2017	Effect of different environmental factors	0.049
Dominica	1990	Complications following therapeutic procedures	0.0506
Dominica	1995	Complications following therapeutic procedures	0.0506
Dominica	2000	Complications following therapeutic procedures	0.0506
Dominica	2005	Complications following therapeutic procedures	0.0506
Dominica	2010	Complications following therapeutic procedures	0.0506
Dominica	2017	Complications following therapeutic procedures	0.0506
Dominica	1990	Multiple fractures, dislocations, crashes, wounds, sprains, and strains	0.1588
Dominica	1995	Multiple fractures, dislocations, crashes, wounds, sprains, and strains	0.1502
Dominica	2000	Multiple fractures, dislocations, crashes, wounds, sprains, and strains	0.1297
Dominica	2005	Multiple fractures, dislocations, crashes, wounds, sprains, and strains	0.1196
Dominica	2010	Multiple fractures, dislocations, crashes, wounds, sprains, and strains	0.1211
Dominica	2017	Multiple fractures, dislocations, crashes, wounds, sprains, and strains	0.1144
Dominica	1990	Amputation of upper limb, unilateral	0.0622
Dominica	1995	Amputation of upper limb, unilateral	0.0602
Dominica	2000	Amputation of upper limb, unilateral	0.0555
Dominica	2005	Amputation of upper limb, unilateral	0.0532
Dominica	2010	Amputation of upper limb, unilateral	0.0535
Dominica	2017	Amputation of upper limb, unilateral	0.052
Dominica	1990	Amputation of thumb	0.0114
Dominica	1995	Amputation of thumb	0.0114
Dominica	2000	Amputation of thumb	0.0114
Dominica	2005	Amputation of thumb	0.0114
Dominica	2010	Amputation of thumb	0.0114
Dominica	2017	Amputation of thumb	0.0114
Dominica	1990	Amputation of toe/toes	0.0059
Dominica	1995	Amputation of toe/toes	0.0059
Dominica	2000	Amputation of toe/toes	0.0059
Dominica	2005	Amputation of toe/toes	0.0059
Dominica	2010	Amputation of toe/toes	0.0059
Dominica	2017	Amputation of toe/toes	0.0059
Dominica	1990	Burns, <20% total burned surface area without lower airway burns	0.0163
Dominica	1995	Burns, <20% total burned surface area without lower airway burns	0.0163
Dominica	2000	Burns, <20% total burned surface area without lower airway burns	0.0163
Dominica	2005	Burns, <20% total burned surface area without lower airway burns	0.0163
Dominica	2010	Burns, <20% total burned surface area without lower airway burns	0.0163
Dominica	2017	Burns, <20% total burned surface area without lower airway burns	0.0163
Dominica	1990	Burns, >=20% total burned surface area or >= 10% burned surface area if head/neck or hands/wrist involved w/o lower airway burns	0.2288
Dominica	1995	Burns, >=20% total burned surface area or >= 10% burned surface area if head/neck or hands/wrist involved w/o lower airway burns	0.2207
Dominica	2000	Burns, >=20% total burned surface area or >= 10% burned surface area if head/neck or hands/wrist involved w/o lower airway burns	0.2016
Dominica	2005	Burns, >=20% total burned surface area or >= 10% burned surface area if head/neck or hands/wrist involved w/o lower airway burns	0.1923
Dominica	2010	Burns, >=20% total burned surface area or >= 10% burned surface area if head/neck or hands/wrist involved w/o lower airway burns	0.1936
Dominica	2017	Burns, >=20% total burned surface area or >= 10% burned surface area if head/neck or hands/wrist involved w/o lower airway burns	0.1874
Dominican Republic	1990	Amputation of lower limbs, bilateral	0.2465
Dominican Republic	1995	Amputation of lower limbs, bilateral	0.2188
Dominican Republic	2000	Amputation of lower limbs, bilateral	0.1993
Dominican Republic	2005	Amputation of lower limbs, bilateral	0.1909
Dominican Republic	2010	Amputation of lower limbs, bilateral	0.181
Dominican Republic	2017	Amputation of lower limbs, bilateral	0.168
Dominican Republic	1990	Lower airway burns	0.2252
Dominican Republic	1995	Lower airway burns	0.2252
Dominican Republic	2000	Lower airway burns	0.2252
Dominican Republic	2005	Lower airway burns	0.2252
Dominican Republic	2010	Lower airway burns	0.2252
Dominican Republic	2017	Lower airway burns	0.2252
Dominican Republic	1990	Dislocation of hip	0.0159
Dominican Republic	1995	Dislocation of hip	0.0159
Dominican Republic	2000	Dislocation of hip	0.0159
Dominican Republic	2005	Dislocation of hip	0.0159
Dominican Republic	2010	Dislocation of hip	0.0159
Dominican Republic	2017	Dislocation of hip	0.0159
Dominican Republic	1990	Dislocation of knee	0.1134
Dominican Republic	1995	Dislocation of knee	0.1134
Dominican Republic	2000	Dislocation of knee	0.1134
Dominican Republic	2005	Dislocation of knee	0.1134
Dominican Republic	2010	Dislocation of knee	0.1134
Dominican Republic	2017	Dislocation of knee	0.1134
Dominican Republic	1990	Dislocation of shoulder	0.062
Dominican Republic	1995	Dislocation of shoulder	0.062
Dominican Republic	2000	Dislocation of shoulder	0.062
Dominican Republic	2005	Dislocation of shoulder	0.062
Dominican Republic	2010	Dislocation of shoulder	0.062
Dominican Republic	2017	Dislocation of shoulder	0.062
Dominican Republic	1990	Muscle and tendon injuries, including sprains and strains lesser dislocations	0.0075
Dominican Republic	1995	Muscle and tendon injuries, including sprains and strains lesser dislocations	0.0075
Dominican Republic	2000	Muscle and tendon injuries, including sprains and strains lesser dislocations	0.0075
Dominican Republic	2005	Muscle and tendon injuries, including sprains and strains lesser dislocations	0.0075
Dominican Republic	2010	Muscle and tendon injuries, including sprains and strains lesser dislocations	0.0075
Dominican Republic	2017	Muscle and tendon injuries, including sprains and strains lesser dislocations	0.0075
Dominican Republic	1990	Fracture of clavicle, scapula, or humerus	0.0349
Dominican Republic	1995	Fracture of clavicle, scapula, or humerus	0.0349
Dominican Republic	2000	Fracture of clavicle, scapula, or humerus	0.0349
Dominican Republic	2005	Fracture of clavicle, scapula, or humerus	0.0349
Dominican Republic	2010	Fracture of clavicle, scapula, or humerus	0.0349
Dominican Republic	2017	Fracture of clavicle, scapula, or humerus	0.0349
Dominican Republic	1990	Fracture of face bones	0.0669
Dominican Republic	1995	Fracture of face bones	0.0669
Dominican Republic	2000	Fracture of face bones	0.0669
Dominican Republic	2005	Fracture of face bones	0.0669
Dominican Republic	2010	Fracture of face bones	0.0669
Dominican Republic	2017	Fracture of face bones	0.0669
Dominican Republic	1990	Fracture of foot bones except ankle	0.0264
Dominican Republic	1995	Fracture of foot bones except ankle	0.0264
Dominican Republic	2000	Fracture of foot bones except ankle	0.0264
Dominican Republic	2005	Fracture of foot bones except ankle	0.0264
Dominican Republic	2010	Fracture of foot bones except ankle	0.0264
Dominican Republic	2017	Fracture of foot bones except ankle	0.0264
Dominican Republic	1990	Fracture of hand (wrist and other distal part of hand)	0.0138
Dominican Republic	1995	Fracture of hand (wrist and other distal part of hand)	0.0138
Dominican Republic	2000	Fracture of hand (wrist and other distal part of hand)	0.0138
Dominican Republic	2005	Fracture of hand (wrist and other distal part of hand)	0.0138
Dominican Republic	2010	Fracture of hand (wrist and other distal part of hand)	0.0138
Dominican Republic	2017	Fracture of hand (wrist and other distal part of hand)	0.0138
Dominican Republic	1990	Fracture of hip	0.2207
Dominican Republic	1995	Fracture of hip	0.184
Dominican Republic	2000	Fracture of hip	0.1583
Dominican Republic	2005	Fracture of hip	0.1471
Dominican Republic	2010	Fracture of hip	0.1341
Dominican Republic	2017	Fracture of hip	0.1168
Dominican Republic	1990	Amputation of upper limbs, bilateral	0.2559
Dominican Republic	1995	Amputation of upper limbs, bilateral	0.2181
Dominican Republic	2000	Amputation of upper limbs, bilateral	0.1915
Dominican Republic	2005	Amputation of upper limbs, bilateral	0.18
Dominican Republic	2010	Amputation of upper limbs, bilateral	0.1665
Dominican Republic	2017	Amputation of upper limbs, bilateral	0.1487
Dominican Republic	1990	Fracture of patella, tibia or fibula, or ankle	0.0554
Dominican Republic	1995	Fracture of patella, tibia or fibula, or ankle	0.0554
Dominican Republic	2000	Fracture of patella, tibia or fibula, or ankle	0.0554
Dominican Republic	2005	Fracture of patella, tibia or fibula, or ankle	0.0554
Dominican Republic	2010	Fracture of patella, tibia or fibula, or ankle	0.0554
Dominican Republic	2017	Fracture of patella, tibia or fibula, or ankle	0.0554
Dominican Republic	1990	Fracture of pelvis	0.1823
Dominican Republic	1995	Fracture of pelvis	0.1823
Dominican Republic	2000	Fracture of pelvis	0.1823
Dominican Republic	2005	Fracture of pelvis	0.1823
Dominican Republic	2010	Fracture of pelvis	0.1823
Dominican Republic	2017	Fracture of pelvis	0.1823
Dominican Republic	1990	Fracture of radius and/or ulna	0.0435
Dominican Republic	1995	Fracture of radius and/or ulna	0.0435
Dominican Republic	2000	Fracture of radius and/or ulna	0.0435
Dominican Republic	2005	Fracture of radius and/or ulna	0.0435
Dominican Republic	2010	Fracture of radius and/or ulna	0.0435
Dominican Republic	2017	Fracture of radius and/or ulna	0.0435
Dominican Republic	1990	Fracture of skull	0.0714
Dominican Republic	1995	Fracture of skull	0.0714
Dominican Republic	2000	Fracture of skull	0.0714
Dominican Republic	2005	Fracture of skull	0.0714
Dominican Republic	2010	Fracture of skull	0.0714
Dominican Republic	2017	Fracture of skull	0.0714
Dominican Republic	1990	Fracture of sternum and/or fracture of one or more ribs	0.1027
Dominican Republic	1995	Fracture of sternum and/or fracture of one or more ribs	0.1027
Dominican Republic	2000	Fracture of sternum and/or fracture of one or more ribs	0.1027
Dominican Republic	2005	Fracture of sternum and/or fracture of one or more ribs	0.1027
Dominican Republic	2010	Fracture of sternum and/or fracture of one or more ribs	0.1027
Dominican Republic	2017	Fracture of sternum and/or fracture of one or more ribs	0.1027
Dominican Republic	1990	Fracture of vertebral column	0.1106
Dominican Republic	1995	Fracture of vertebral column	0.1106
Dominican Republic	2000	Fracture of vertebral column	0.1106
Dominican Republic	2005	Fracture of vertebral column	0.1106
Dominican Republic	2010	Fracture of vertebral column	0.1106
Dominican Republic	2017	Fracture of vertebral column	0.1106
Dominican Republic	1990	Fracture of femur, other than femoral neck	0.042
Dominican Republic	1995	Fracture of femur, other than femoral neck	0.042
Dominican Republic	2000	Fracture of femur, other than femoral neck	0.042
Dominican Republic	2005	Fracture of femur, other than femoral neck	0.042
Dominican Republic	2010	Fracture of femur, other than femoral neck	0.042
Dominican Republic	2017	Fracture of femur, other than femoral neck	0.042
Dominican Republic	1990	Minor TBI	0.1319
Dominican Republic	1995	Minor TBI	0.1319
Dominican Republic	2000	Minor TBI	0.1319
Dominican Republic	2005	Minor TBI	0.1319
Dominican Republic	2010	Minor TBI	0.1319
Dominican Republic	2017	Minor TBI	0.1319
Dominican Republic	1990	Moderate TBI	0.1637
Dominican Republic	1995	Moderate TBI	0.1637
Dominican Republic	2000	Moderate TBI	0.1637
Dominican Republic	2005	Moderate TBI	0.1637
Dominican Republic	2010	Moderate TBI	0.1637
Dominican Republic	2017	Moderate TBI	0.1637
Dominican Republic	1990	Amputation of fingers (excluding thumb)	0.0047
Dominican Republic	1995	Amputation of fingers (excluding thumb)	0.0047
Dominican Republic	2000	Amputation of fingers (excluding thumb)	0.0047
Dominican Republic	2005	Amputation of fingers (excluding thumb)	0.0047
Dominican Republic	2010	Amputation of fingers (excluding thumb)	0.0047
Dominican Republic	2017	Amputation of fingers (excluding thumb)	0.0047
Dominican Republic	1990	Spinal cord lesion at neck level	0.4682
Dominican Republic	1995	Spinal cord lesion at neck level	0.4555
Dominican Republic	2000	Spinal cord lesion at neck level	0.4466
Dominican Republic	2005	Spinal cord lesion at neck level	0.4427
Dominican Republic	2010	Spinal cord lesion at neck level	0.4382
Dominican Republic	2017	Spinal cord lesion at neck level	0.4323
Dominican Republic	1990	Spinal cord lesion below neck level	0.2966
Dominican Republic	1995	Spinal cord lesion below neck level	0.2699
Dominican Republic	2000	Spinal cord lesion below neck level	0.2511
Dominican Republic	2005	Spinal cord lesion below neck level	0.2429
Dominican Republic	2010	Spinal cord lesion below neck level	0.2334
Dominican Republic	2017	Spinal cord lesion below neck level	0.2208
Dominican Republic	1990	Drowning and nonfatal submersion	0.2471
Dominican Republic	1995	Drowning and nonfatal submersion	0.2471
Dominican Republic	2000	Drowning and nonfatal submersion	0.2471
Dominican Republic	2005	Drowning and nonfatal submersion	0.2471
Dominican Republic	2010	Drowning and nonfatal submersion	0.2471
Dominican Republic	2017	Drowning and nonfatal submersion	0.2471
Dominican Republic	1990	Asphyxiation	0.2471
Dominican Republic	1995	Asphyxiation	0.2471
Dominican Republic	2000	Asphyxiation	0.2471
Dominican Republic	2005	Asphyxiation	0.2471
Dominican Republic	2010	Asphyxiation	0.2471
Dominican Republic	2017	Asphyxiation	0.2471
Dominican Republic	1990	Crush injury	0.1325
Dominican Republic	1995	Crush injury	0.1325
Dominican Republic	2000	Crush injury	0.1325
Dominican Republic	2005	Crush injury	0.1325
Dominican Republic	2010	Crush injury	0.1325
Dominican Republic	2017	Crush injury	0.1325
Dominican Republic	1990	Nerve injury	0.1129
Dominican Republic	1995	Nerve injury	0.1129
Dominican Republic	2000	Nerve injury	0.1129
Dominican Republic	2005	Nerve injury	0.1129
Dominican Republic	2010	Nerve injury	0.1129
Dominican Republic	2017	Nerve injury	0.1129
Dominican Republic	1990	Injury to eyes	0.0165
Dominican Republic	1995	Injury to eyes	0.0165
Dominican Republic	2000	Injury to eyes	0.0165
Dominican Republic	2005	Injury to eyes	0.0165
Dominican Republic	2010	Injury to eyes	0.0165
Dominican Republic	2017	Injury to eyes	0.0165
Dominican Republic	1990	Amputation of lower limb, unilateral	0.1025
Dominican Republic	1995	Amputation of lower limb, unilateral	0.0882
Dominican Republic	2000	Amputation of lower limb, unilateral	0.0782
Dominican Republic	2005	Amputation of lower limb, unilateral	0.0738
Dominican Republic	2010	Amputation of lower limb, unilateral	0.0687
Dominican Republic	2017	Amputation of lower limb, unilateral	0.062
Dominican Republic	1990	Open wound(s)	0.011
Dominican Republic	1995	Open wound(s)	0.011
Dominican Republic	2000	Open wound(s)	0.011
Dominican Republic	2005	Open wound(s)	0.011
Dominican Republic	2010	Open wound(s)	0.011
Dominican Republic	2017	Open wound(s)	0.011
Dominican Republic	1990	Poisoning requiring urgent care	0.1628
Dominican Republic	1995	Poisoning requiring urgent care	0.1628
Dominican Republic	2000	Poisoning requiring urgent care	0.1628
Dominican Republic	2005	Poisoning requiring urgent care	0.1628
Dominican Republic	2010	Poisoning requiring urgent care	0.1628
Dominican Republic	2017	Poisoning requiring urgent care	0.1628
Dominican Republic	1990	Severe chest injury	0.0474
Dominican Republic	1995	Severe chest injury	0.0474
Dominican Republic	2000	Severe chest injury	0.0474
Dominican Republic	2005	Severe chest injury	0.0474
Dominican Republic	2010	Severe chest injury	0.0474
Dominican Republic	2017	Severe chest injury	0.0474
Dominican Republic	1990	Internal hemorrhage in abdomen and pelvis	0.1143
Dominican Republic	1995	Internal hemorrhage in abdomen and pelvis	0.1143
Dominican Republic	2000	Internal hemorrhage in abdomen and pelvis	0.1143
Dominican Republic	2005	Internal hemorrhage in abdomen and pelvis	0.1143
Dominican Republic	2010	Internal hemorrhage in abdomen and pelvis	0.1143
Dominican Republic	2017	Internal hemorrhage in abdomen and pelvis	0.1143
Dominican Republic	1990	Contusion in any part of the body	0.0075
Dominican Republic	1995	Contusion in any part of the body	0.0075
Dominican Republic	2000	Contusion in any part of the body	0.0075
Dominican Republic	2005	Contusion in any part of the body	0.0075
Dominican Republic	2010	Contusion in any part of the body	0.0075
Dominican Republic	2017	Contusion in any part of the body	0.0075
Dominican Republic	1990	Effect of different environmental factors	0.049
Dominican Republic	1995	Effect of different environmental factors	0.049
Dominican Republic	2000	Effect of different environmental factors	0.049
Dominican Republic	2005	Effect of different environmental factors	0.049
Dominican Republic	2010	Effect of different environmental factors	0.049
Dominican Republic	2017	Effect of different environmental factors	0.049
Dominican Republic	1990	Complications following therapeutic procedures	0.0506
Dominican Republic	1995	Complications following therapeutic procedures	0.0506
Dominican Republic	2000	Complications following therapeutic procedures	0.0506
Dominican Republic	2005	Complications following therapeutic procedures	0.0506

Dominican Republic	2010	Complications following therapeutic procedures	0.0506
Dominican Republic	2017	Complications following therapeutic procedures	0.0506
Dominican Republic	1990	Multiple fractures, dislocations, crashes, wounds, sprains, and strains	0.2207
Dominican Republic	1995	Multiple fractures, dislocations, crashes, wounds, sprains, and strains	0.184
Dominican Republic	2000	Multiple fractures, dislocations, crashes, wounds, sprains, and strains	0.1583
Dominican Republic	2005	Multiple fractures, dislocations, crashes, wounds, sprains, and strains	0.1471
Dominican Republic	2010	Multiple fractures, dislocations, crashes, wounds, sprains, and strains	0.1341
Dominican Republic	2017	Multiple fractures, dislocations, crashes, wounds, sprains, and strains	0.1168
Dominican Republic	1990	Amputation of upper limb, unilateral	0.0764
Dominican Republic	1995	Amputation of upper limb, unilateral	0.068
Dominican Republic	2000	Amputation of upper limb, unilateral	0.0621
Dominican Republic	2005	Amputation of upper limb, unilateral	0.0595
Dominican Republic	2010	Amputation of upper limb, unilateral	0.0565
Dominican Republic	2017	Amputation of upper limb, unilateral	0.0526
Dominican Republic	1990	Amputation of thumb	0.0114
Dominican Republic	1995	Amputation of thumb	0.0114
Dominican Republic	2000	Amputation of thumb	0.0114
Dominican Republic	2005	Amputation of thumb	0.0114
Dominican Republic	2010	Amputation of thumb	0.0114
Dominican Republic	2017	Amputation of thumb	0.0114
Dominican Republic	1990	Amputation of toe/toes	0.0059
Dominican Republic	1995	Amputation of toe/toes	0.0059
Dominican Republic	2000	Amputation of toe/toes	0.0059
Dominican Republic	2005	Amputation of toe/toes	0.0059
Dominican Republic	2010	Amputation of toe/toes	0.0059
Dominican Republic	2017	Amputation of toe/toes	0.0059
Dominican Republic	1990	Burns, <20% total burned surface area without lower airway burns	0.0163
Dominican Republic	1995	Burns, <20% total burned surface area without lower airway burns	0.0163
Dominican Republic	2000	Burns, <20% total burned surface area without lower airway burns	0.0163
Dominican Republic	2005	Burns, <20% total burned surface area without lower airway burns	0.0163
Dominican Republic	2010	Burns, <20% total burned surface area without lower airway burns	0.0163
Dominican Republic	2017	Burns, <20% total burned surface area without lower airway burns	0.0163
Dominican Republic	1990	Burns, >=20% total burned surface area or >= 10% burned surface area if head/neck or hands/wrist involved w/o lower airway burns	0.2863
Dominican Republic	1995	Burns, >=20% total burned surface area or >= 10% burned surface area if head/neck or hands/wrist involved w/o lower airway burns	0.2522
Dominican Republic	2000	Burns, >=20% total burned surface area or >= 10% burned surface area if head/neck or hands/wrist involved w/o lower airway burns	0.2282
Dominican Republic	2005	Burns, >=20% total burned surface area or >= 10% burned surface area if head/neck or hands/wrist involved w/o lower airway burns	0.2178
Dominican Republic	2010	Burns, >=20% total burned surface area or >= 10% burned surface area if head/neck or hands/wrist involved w/o lower airway burns	0.2057
Dominican Republic	2017	Burns, >=20% total burned surface area or >= 10% burned surface area if head/neck or hands/wrist involved w/o lower airway burns	0.1897
Grenada	1990	Amputation of lower limbs, bilateral	0.217
Grenada	1995	Amputation of lower limbs, bilateral	0.2053
Grenada	2000	Amputation of lower limbs, bilateral	0.1967
Grenada	2005	Amputation of lower limbs, bilateral	0.1891
Grenada	2010	Amputation of lower limbs, bilateral	0.1823
Grenada	2017	Amputation of lower limbs, bilateral	0.1775
Grenada	1990	Lower airway burns	0.2252
Grenada	1995	Lower airway burns	0.2252
Grenada	2000	Lower airway burns	0.2252
Grenada	2005	Lower airway burns	0.2252
Grenada	2010	Lower airway burns	0.2252
Grenada	2017	Lower airway burns	0.2252
Grenada	1990	Dislocation of hip	0.0159
Grenada	1995	Dislocation of hip	0.0159
Grenada	2000	Dislocation of hip	0.0159
Grenada	2005	Dislocation of hip	0.0159
Grenada	2010	Dislocation of hip	0.0159
Grenada	2017	Dislocation of hip	0.0159
Grenada	1990	Dislocation of knee	0.1134
Grenada	1995	Dislocation of knee	0.1134
Grenada	2000	Dislocation of knee	0.1134
Grenada	2005	Dislocation of knee	0.1134
Grenada	2010	Dislocation of knee	0.1134
Grenada	2017	Dislocation of knee	0.1134
Grenada	1990	Dislocation of shoulder	0.062
Grenada	1995	Dislocation of shoulder	0.062
Grenada	2000	Dislocation of shoulder	0.062
Grenada	2005	Dislocation of shoulder	0.062
Grenada	2010	Dislocation of shoulder	0.062
Grenada	2017	Dislocation of shoulder	0.062
Grenada	1990	Muscle and tendon injuries, including sprains and strains lesser dislocations	0.0075
Grenada	1995	Muscle and tendon injuries, including sprains and strains lesser dislocations	0.0075
Grenada	2000	Muscle and tendon injuries, including sprains and strains lesser dislocations	0.0075
Grenada	2005	Muscle and tendon injuries, including sprains and strains lesser dislocations	0.0075
Grenada	2010	Muscle and tendon injuries, including sprains and strains lesser dislocations	0.0075
Grenada	2017	Muscle and tendon injuries, including sprains and strains lesser dislocations	0.0075
Grenada	1990	Fracture of clavicle, scapula, or humerus	0.0349
Grenada	1995	Fracture of clavicle, scapula, or humerus	0.0349
Grenada	2000	Fracture of clavicle, scapula, or humerus	0.0349
Grenada	2005	Fracture of clavicle, scapula, or humerus	0.0349
Grenada	2010	Fracture of clavicle, scapula, or humerus	0.0349
Grenada	2017	Fracture of clavicle, scapula, or humerus	0.0349
Grenada	1990	Fracture of face bones	0.0669
Grenada	1995	Fracture of face bones	0.0669
Grenada	2000	Fracture of face bones	0.0669
Grenada	2005	Fracture of face bones	0.0669
Grenada	2010	Fracture of face bones	0.0669
Grenada	2017	Fracture of face bones	0.0669
Grenada	1990	Fracture of foot bones except ankle	0.0264
Grenada	1995	Fracture of foot bones except ankle	0.0264
Grenada	2000	Fracture of foot bones except ankle	0.0264
Grenada	2005	Fracture of foot bones except ankle	0.0264
Grenada	2010	Fracture of foot bones except ankle	0.0264
Grenada	2017	Fracture of foot bones except ankle	0.0264
Grenada	1990	Fracture of hand (wrist and other distal part of hand)	0.0138
Grenada	1995	Fracture of hand (wrist and other distal part of hand)	0.0138
Grenada	2000	Fracture of hand (wrist and other distal part of hand)	0.0138
Grenada	2005	Fracture of hand (wrist and other distal part of hand)	0.0138
Grenada	2010	Fracture of hand (wrist and other distal part of hand)	0.0138
Grenada	2017	Fracture of hand (wrist and other distal part of hand)	0.0138
Grenada	1990	Fracture of hip	0.1816
Grenada	1995	Fracture of hip	0.1662
Grenada	2000	Fracture of hip	0.1549
Grenada	2005	Fracture of hip	0.1447
Grenada	2010	Fracture of hip	0.1358
Grenada	2017	Fracture of hip	0.1295
Grenada	1990	Amputation of upper limbs, bilateral	0.2156
Grenada	1995	Amputation of upper limbs, bilateral	0.1997
Grenada	2000	Amputation of upper limbs, bilateral	0.188
Grenada	2005	Amputation of upper limbs, bilateral	0.1775
Grenada	2010	Amputation of upper limbs, bilateral	0.1683
Grenada	2017	Amputation of upper limbs, bilateral	0.1618
Grenada	1990	Fracture of patella, tibia or fibula, or ankle	0.0554
Grenada	1995	Fracture of patella, tibia or fibula, or ankle	0.0554
Grenada	2000	Fracture of patella, tibia or fibula, or ankle	0.0554
Grenada	2005	Fracture of patella, tibia or fibula, or ankle	0.0554
Grenada	2010	Fracture of patella, tibia or fibula, or ankle	0.0554
Grenada	2017	Fracture of patella, tibia or fibula, or ankle	0.0554
Grenada	1990	Fracture of pelvis	0.1823
Grenada	1995	Fracture of pelvis	0.1823
Grenada	2000	Fracture of pelvis	0.1823
Grenada	2005	Fracture of pelvis	0.1823
Grenada	2010	Fracture of pelvis	0.1823
Grenada	2017	Fracture of pelvis	0.1823
Grenada	1990	Fracture of radius and/or ulna	0.0435
Grenada	1995	Fracture of radius and/or ulna	0.0435
Grenada	2000	Fracture of radius and/or ulna	0.0435
Grenada	2005	Fracture of radius and/or ulna	0.0435
Grenada	2010	Fracture of radius and/or ulna	0.0435
Grenada	2017	Fracture of radius and/or ulna	0.0435
Grenada	1990	Fracture of skull	0.0714
Grenada	1995	Fracture of skull	0.0714
Grenada	2000	Fracture of skull	0.0714
Grenada	2005	Fracture of skull	0.0714
Grenada	2010	Fracture of skull	0.0714
Grenada	2017	Fracture of skull	0.0714
Grenada	1990	Fracture of sternum and/or fracture of one or more ribs	0.1027
Grenada	1995	Fracture of sternum and/or fracture of one or more ribs	0.1027
Grenada	2000	Fracture of sternum and/or fracture of one or more ribs	0.1027
Grenada	2005	Fracture of sternum and/or fracture of one or more ribs	0.1027
Grenada	2010	Fracture of sternum and/or fracture of one or more ribs	0.1027
Grenada	2017	Fracture of sternum and/or fracture of one or more ribs	0.1027
Grenada	1990	Fracture of vertebral column	0.1106
Grenada	1995	Fracture of vertebral column	0.1106
Grenada	2000	Fracture of vertebral column	0.1106
Grenada	2005	Fracture of vertebral column	0.1106
Grenada	2010	Fracture of vertebral column	0.1106
Grenada	2017	Fracture of vertebral column	0.1106
Grenada	1990	Fracture of femur, other than femoral neck	0.042
Grenada	1995	Fracture of femur, other than femoral neck	0.042
Grenada	2000	Fracture of femur, other than femoral neck	0.042
Grenada	2005	Fracture of femur, other than femoral neck	0.042
Grenada	2010	Fracture of femur, other than femoral neck	0.042
Grenada	2017	Fracture of femur, other than femoral neck	0.042
Grenada	1990	Minor TBI	0.1319
Grenada	1995	Minor TBI	0.1319
Grenada	2000	Minor TBI	0.1319
Grenada	2005	Minor TBI	0.1319
Grenada	2010	Minor TBI	0.1319
Grenada	2017	Minor TBI	0.1319
Grenada	1990	Moderate TBI	0.1637
Grenada	1995	Moderate TBI	0.1637
Grenada	2000	Moderate TBI	0.1637
Grenada	2005	Moderate TBI	0.1637
Grenada	2010	Moderate TBI	0.1637
Grenada	2017	Moderate TBI	0.1637
Grenada	1990	Amputation of fingers (excluding thumb)	0.0047
Grenada	1995	Amputation of fingers (excluding thumb)	0.0047
Grenada	2000	Amputation of fingers (excluding thumb)	0.0047
Grenada	2005	Amputation of fingers (excluding thumb)	0.0047
Grenada	2010	Amputation of fingers (excluding thumb)	0.0047
Grenada	2017	Amputation of fingers (excluding thumb)	0.0047
Grenada	1990	Spinal cord lesion at neck level	0.4547
Grenada	1995	Spinal cord lesion at neck level	0.4494
Grenada	2000	Spinal cord lesion at neck level	0.4454
Grenada	2005	Spinal cord lesion at neck level	0.4419
Grenada	2010	Spinal cord lesion at neck level	0.4388
Grenada	2017	Spinal cord lesion at neck level	0.4366
Grenada	1990	Spinal cord lesion below neck level	0.2681
Grenada	1995	Spinal cord lesion below neck level	0.2569
Grenada	2000	Spinal cord lesion below neck level	0.2486
Grenada	2005	Spinal cord lesion below neck level	0.2412
Grenada	2010	Spinal cord lesion below neck level	0.2347
Grenada	2017	Spinal cord lesion below neck level	0.2301
Grenada	1990	Drowning and nonfatal submersion	0.2471
Grenada	1995	Drowning and nonfatal submersion	0.2471
Grenada	2000	Drowning and nonfatal submersion	0.2471
Grenada	2005	Drowning and nonfatal submersion	0.2471
Grenada	2010	Drowning and nonfatal submersion	0.2471
Grenada	2017	Drowning and nonfatal submersion	0.2471
Grenada	1990	Asphyxiation	0.2471
Grenada	1995	Asphyxiation	0.2471
Grenada	2000	Asphyxiation	0.2471
Grenada	2005	Asphyxiation	0.2471
Grenada	2010	Asphyxiation	0.2471
Grenada	2017	Asphyxiation	0.2471
Grenada	1990	Crush injury	0.1325
Grenada	1995	Crush injury	0.1325
Grenada	2000	Crush injury	0.1325
Grenada	2005	Crush injury	0.1325
Grenada	2010	Crush injury	0.1325
Grenada	2017	Crush injury	0.1325
Grenada	1990	Nerve injury	0.1129
Grenada	1995	Nerve injury	0.1129
Grenada	2000	Nerve injury	0.1129
Grenada	2005	Nerve injury	0.1129
Grenada	2010	Nerve injury	0.1129
Grenada	2017	Nerve injury	0.1129
Grenada	1990	Injury to eyes	0.0165
Grenada	1995	Injury to eyes	0.0165
Grenada	2000	Injury to eyes	0.0165
Grenada	2005	Injury to eyes	0.0165
Grenada	2010	Injury to eyes	0.0165
Grenada	2017	Injury to eyes	0.0165
Grenada	1990	Amputation of lower limb, unilateral	0.0873
Grenada	1995	Amputation of lower limb, unilateral	0.0813
Grenada	2000	Amputation of lower limb, unilateral	0.0768
Grenada	2005	Amputation of lower limb, unilateral	0.0729
Grenada	2010	Amputation of lower limb, unilateral	0.0694
Grenada	2017	Amputation of lower limb, unilateral	0.0669
Grenada	1990	Open wound(s)	0.011
Grenada	1995	Open wound(s)	0.011
Grenada	2000	Open wound(s)	0.011
Grenada	2005	Open wound(s)	0.011
Grenada	2010	Open wound(s)	0.011
Grenada	2017	Open wound(s)	0.011
Grenada	1990	Poisoning requiring urgent care	0.1628
Grenada	1995	Poisoning requiring urgent care	0.1628
Grenada	2000	Poisoning requiring urgent care	0.1628
Grenada	2005	Poisoning requiring urgent care	0.1628
Grenada	2010	Poisoning requiring urgent care	0.1628
Grenada	2017	Poisoning requiring urgent care	0.1628
Grenada	1990	Severe chest injury	0.0474
Grenada	1995	Severe chest injury	0.0474
Grenada	2000	Severe chest injury	0.0474
Grenada	2005	Severe chest injury	0.0474
Grenada	2010	Severe chest injury	0.0474
Grenada	2017	Severe chest injury	0.0474
Grenada	1990	Internal hemorrhage in abdomen and pelvis	0.1143
Grenada	1995	Internal hemorrhage in abdomen and pelvis	0.1143
Grenada	2000	Internal hemorrhage in abdomen and pelvis	0.1143
Grenada	2005	Internal hemorrhage in abdomen and pelvis	0.1143
Grenada	2010	Internal hemorrhage in abdomen and pelvis	0.1143
Grenada	2017	Internal hemorrhage in abdomen and pelvis	0.1143
Grenada	1990	Contusion in any part of the body	0.0075
Grenada	1995	Contusion in any part of the body	0.0075
Grenada	2000	Contusion in any part of the body	0.0075
Grenada	2005	Contusion in any part of the body	0.0075
Grenada	2010	Contusion in any part of the body	0.0075
Grenada	2017	Contusion in any part of the body	0.0075
Grenada	1990	Effect of different environmental factors	0.049
Grenada	1995	Effect of different environmental factors	0.049
Grenada	2000	Effect of different environmental factors	0.049
Grenada	2005	Effect of different environmental factors	0.049
Grenada	2010	Effect of different environmental factors	0.049
Grenada	2017	Effect of different environmental factors	0.049
Grenada	1990	Complications following therapeutic procedures	0.0506
Grenada	1995	Complications following therapeutic procedures	0.0506
Grenada	2000	Complications following therapeutic procedures	0.0506
Grenada	2005	Complications following therapeutic procedures	0.0506
Grenada	2010	Complications following therapeutic procedures	0.0506
Grenada	2017	Complications following therapeutic procedures	0.0506
Grenada	1990	Multiple fractures, dislocations, crashes, wounds, sprains, and strains	0.1816
Grenada	1995	Multiple fractures, dislocations, crashes, wounds, sprains, and strains	0.1662
Grenada	2000	Multiple fractures, dislocations, crashes, wounds, sprains, and strains	0.1549
Grenada	2005	Multiple fractures, dislocations, crashes, wounds, sprains, and strains	0.1447
Grenada	2010	Multiple fractures, dislocations, crashes, wounds, sprains, and strains	0.1358
Grenada	2017	Multiple fractures, dislocations, crashes, wounds, sprains, and strains	0.1295
Grenada	1990	Amputation of upper limb, unilateral	0.0674
Grenada	1995	Amputation of upper limb, unilateral	0.0639
Grenada	2000	Amputation of upper limb, unilateral	0.0613
Grenada	2005	Amputation of upper limb, unilateral	0.059
Grenada	2010	Amputation of upper limb, unilateral	0.0569
Grenada	2017	Amputation of upper limb, unilateral	0.0555
Grenada	1990	Amputation of thumb	0.0114
Grenada	1995	Amputation of thumb	0.0114
Grenada	2000	Amputation of thumb	0.0114
Grenada	2005	Amputation of thumb	0.0114
Grenada	2010	Amputation of thumb	0.0114
Grenada	2017	Amputation of thumb	0.0114
Grenada	1990	Amputation of toe/toes	0.0059
Grenada	1995	Amputation of toe/toes	0.0059
Grenada	2000	Amputation of toe/toes	0.0059
Grenada	2005	Amputation of toe/toes	0.0059
Grenada	2010	Amputation of toe/toes	0.0059
Grenada	2017	Amputation of toe/toes	0.0059
Grenada	1990	Burns, <20% total burned surface area without lower airway burns	0.0163
Grenada	1995	Burns, <20% total burned surface area without lower airway burns	0.0163
Grenada	2000	Burns, <20% total burned surface area without lower airway burns	0.0163
Grenada	2005	Burns, <20% total burned surface area without lower airway burns	0.0163
Grenada	2010	Burns, <20% total burned surface area without lower airway burns	0.0163
Grenada	2017	Burns, <20% total burned surface area without lower airway burns	0.0163
Grenada	1990	Burns, >=20% total burned surface area or >= 10% burned surface area if head/neck or hands/wrist involved w/o lower airway burns	0.25
Grenada	1995	Burns, >=20% total burned surface area or >= 10% burned surface area if head/neck or hands/wrist involved w/o lower airway burns	0.2356
Grenada	2000	Burns, >=20% total burned surface area or >= 10% burned surface area if head/neck or hands/wrist involved w/o lower airway burns	0.2251
Grenada	2005	Burns, >=20% total burned surface area or >= 10% burned surface area if head/neck or hands/wrist involved w/o lower airway burns	0.2156
Grenada	2010	Burns, >=20% total burned surface area or >= 10% burned surface area if head/neck or hands/wrist involved w/o lower airway burns	0.2073
Grenada	2017	Burns, >=20% total burned surface area or >= 10% burned surface area if head/neck or hands/wrist involved w/o lower airway burns	0.2014
Guyana	1990	Amputation of lower limbs, bilateral	0.2466
Guyana	1995	Amputation of lower limbs, bilateral	0.2368
Guyana	2000	Amputation of lower limbs, bilateral	0.2301
Guyana	2005	Amputation of lower limbs, bilateral	0.2277
Guyana	2010	Amputation of lower limbs, bilateral	0.2227
Guyana	2017	Amputation of lower limbs, bilateral	0.2064
Guyana	1990	Lower airway burns	0.2252
Guyana	1995	Lower airway burns	0.2252
Guyana	2000	Lower airway burns	0.2252
Guyana	2005	Lower airway burns	0.2252
Guyana	2010	Lower airway burns	0.2252
Guyana	2017	Lower airway burns	0.2252
Guyana	1990	Dislocation of hip	0.0159
Guyana	1995	Dislocation of hip	0.0159
Guyana	2000	Dislocation of hip	0.0159
Guyana	2005	Dislocation of hip	0.0159
Guyana	2010	Dislocation of hip	0.0159
Guyana	2017	Dislocation of hip	0.0159
Guyana	1990	Dislocation of knee	0.1134
Guyana	1995	Dislocation of knee	0.1134
Guyana	2000	Dislocation of knee	0.1134
Guyana	2005	Dislocation of knee	0.1134
Guyana	2010	Dislocation of knee	0.1134
Guyana	2017	Dislocation of knee	0.1134
Guyana	1990	Dislocation of shoulder	0.062
Guyana	1995	Dislocation of shoulder	0.062
Guyana	2000	Dislocation of shoulder	0.062
Guyana	2005	Dislocation of shoulder	0.062
Guyana	2010	Dislocation of shoulder	0.062
Guyana	2017	Dislocation of shoulder	0.062
Guyana	1990	Muscle and tendon injuries, including sprains and strains lesser dislocations	0.0075
Guyana	1995	Muscle and tendon injuries, including sprains and strains lesser dislocations	0.0075
Guyana	2000	Muscle and tendon injuries, including sprains and strains lesser dislocations	0.0075
Guyana	2005	Muscle and tendon injuries, including sprains and strains lesser dislocations	0.0075
Guyana	2010	Muscle and tendon injuries, including sprains and strains lesser dislocations	0.0075
Guyana	2017	Muscle and tendon injuries, including sprains and strains lesser dislocations	0.0075
Guyana	1990	Fracture of clavicle, scapula, or humerus	0.0349
Guyana	1995	Fracture of clavicle, scapula, or humerus	0.0349
Guyana	2000	Fracture of clavicle, scapula, or humerus	0.0349
Guyana	2005	Fracture of clavicle, scapula, or humerus	0.0349
Guyana	2010	Fracture of clavicle, scapula, or humerus	0.0349
Guyana	2017	Fracture of clavicle, scapula, or humerus	0.0349
Guyana	1990	Fracture of face bones	0.0669
Guyana	1995	Fracture of face bones	0.0669
Guyana	2000	Fracture of face bones	0.0669
Guyana	2005	Fracture of face bones	0.0669
Guyana	2010	Fracture of face bones	0.0669
Guyana	2017	Fracture of face bones	0.0669
Guyana	1990	Fracture of foot bones except ankle	0.0264
Guyana	1995	Fracture of foot bones except ankle	0.0264
Guyana	2000	Fracture of foot bones except ankle	0.0264
Guyana	2005	Fracture of foot bones except ankle	0.0264
Guyana	2010	Fracture of foot bones except ankle	0.0264
Guyana	2017	Fracture of foot bones except ankle	0.0264
Guyana	1990	Fracture of hand (wrist and other distal part of hand)	0.0138
Guyana	1995	Fracture of hand (wrist and other distal part of hand)	0.0138
Guyana	2000	Fracture of hand (wrist and other distal part of hand)	0.0138
Guyana	2005	Fracture of hand (wrist and other distal part of hand)	0.0138
Guyana	2010	Fracture of hand (wrist and other distal part of hand)	0.0138
Guyana	2017	Fracture of hand (wrist and other distal part of hand)	0.0138
Guyana	1990	Fracture of hip	0.2208
Guyana	1995	Fracture of hip	0.2078
Guyana	2000	Fracture of hip	0.199
Guyana	2005	Fracture of hip	0.1959
Guyana	2010	Fracture of hip	0.1893
Guyana	2017	Fracture of hip	0.1677
Guyana	1990	Amputation of upper limbs, bilateral	0.2561
Guyana	1995	Amputation of upper limbs, bilateral	0.2427
Guyana	2000	Amputation of upper limbs, bilateral	0.2335
Guyana	2005	Amputation of upper limbs, bilateral	0.2303
Guyana	2010	Amputation of upper limbs, bilateral	0.2235
Guyana	2017	Amputation of upper limbs, bilateral	0.2012
Guyana	1990	Fracture of patella, tibia or fibula, or ankle	0.0554
Guyana	1995	Fracture of patella, tibia or fibula, or ankle	0.0554
Guyana	2000	Fracture of patella, tibia or fibula, or ankle	0.0554
Guyana	2005	Fracture of patella, tibia or fibula, or ankle	0.0554
Guyana	2010	Fracture of patella, tibia or fibula, or ankle	0.0554
Guyana	2017	Fracture of patella, tibia or fibula, or ankle	0.0554
Guyana	1990	Fracture of pelvis	0.1823
Guyana	1995	Fracture of pelvis	0.1823
Guyana	2000	Fracture of pelvis	0.1823
Guyana	2005	Fracture of pelvis	0.1823
Guyana	2010	Fracture of pelvis	0.1823
Guyana	2017	Fracture of pelvis	0.1823
Guyana	1990	Fracture of radius and/or ulna	0.0435
Guyana	1995	Fracture of radius and/or ulna	0.0435
Guyana	2000	Fracture of radius and/or ulna	0.0435
Guyana	2005	Fracture of radius and/or ulna	0.0435
Guyana	2010	Fracture of radius and/or ulna	0.0435
Guyana	2017	Fracture of radius and/or ulna	0.0435
Guyana	1990	Fracture of skull	0.0714
Guyana	1995	Fracture of skull	0.0714
Guyana	2000	Fracture of skull	0.0714
Guyana	2005	Fracture of skull	0.0714
Guyana	2010	Fracture of skull	0.0714
Guyana	2017	Fracture of skull	0.0714
Guyana	1990	Fracture of sternum and/or fracture of one or more ribs	0.1027
Guyana	1995	Fracture of sternum and/or fracture of one or more ribs	0.1027
Guyana	2000	Fracture of sternum and/or fracture of one or more ribs	0.1027
Guyana	2005	Fracture of sternum and/or fracture of one or more ribs	0.1027
Guyana	2010	Fracture of sternum and/or fracture of one or more ribs	0.1027
Guyana	2017	Fracture of sternum and/or fracture of one or more ribs	0.1027
Guyana	1990	Fracture of vertebral column	0.1106
Guyana	1995	Fracture of vertebral column	0.1106
Guyana	2000	Fracture of vertebral column	0.1106
Guyana	2005	Fracture of vertebral column	0.1106
Guyana	2010	Fracture of vertebral column	0.1106
Guyana	2017	Fracture of vertebral column	0.1106
Guyana	1990	Fracture of femur, other than femoral neck	0.042
Guyana	1995	Fracture of femur, other than femoral neck	0.042
Guyana	2000	Fracture of femur, other than femoral neck	0.042
Guyana	2005	Fracture of femur, other than femoral neck	0.042
Guyana	2010	Fracture of femur, other than femoral neck	0.042
Guyana	2017	Fracture of femur, other than femoral neck	0.042
Guyana	1990	Minor TBI	0.1319
Guyana	1995	Minor TBI	0.1319
Guyana	2000	Minor TBI	0.1319
Guyana	2005	Minor TBI	0.1319
Guyana	2010	Minor TBI	0.1319
Guyana	2017	Minor TBI	0.1319
Guyana	1990	Moderate TBI	0.1637
Guyana	1995	Moderate TBI	0.1637
Guyana	2000	Moderate TBI	0.1637
Guyana	2005	Moderate TBI	0.1637
Guyana	2010	Moderate TBI	0.1637
Guyana	2017	Moderate TBI	0.1637
Guyana	1990	Amputation of fingers (excluding thumb)	0.0047
Guyana	1995	Amputation of fingers (excluding thumb)	0.0047
Guyana	2000	Amputation of fingers (excluding thumb)	0.0047
Guyana	2005	Amputation of fingers (excluding thumb)	0.0047
Guyana	2010	Amputation of fingers (excluding thumb)	0.0047
Guyana	2017	Amputation of fingers (excluding thumb)	0.0047
Guyana	1990	Spinal cord lesion at neck level	0.4682
Guyana	1995	Spinal cord lesion at neck level	0.4637
Guyana	2000	Spinal cord lesion at neck level	0.4607

Guyana	2005	Spinal cord lesion at neck level	0.4596
Guyana	2010	Spinal cord lesion at neck level	0.4573
Guyana	2017	Spinal cord lesion at neck level	0.4499
Guyana	1990	Spinal cord lesion below neck level	0.2967
Guyana	1995	Spinal cord lesion below neck level	0.2872
Guyana	2000	Spinal cord lesion below neck level	0.2808
Guyana	2005	Spinal cord lesion below neck level	0.2785
Guyana	2010	Spinal cord lesion below neck level	0.2737
Guyana	2017	Spinal cord lesion below neck level	0.2579
Guyana	1990	Drowning and nonfatal submersion	0.2471
Guyana	1995	Drowning and nonfatal submersion	0.2471
Guyana	2000	Drowning and nonfatal submersion	0.2471
Guyana	2005	Drowning and nonfatal submersion	0.2471
Guyana	2010	Drowning and nonfatal submersion	0.2471
Guyana	2017	Drowning and nonfatal submersion	0.2471
Guyana	1990	Asphyxiation	0.2471
Guyana	1995	Asphyxiation	0.2471
Guyana	2000	Asphyxiation	0.2471
Guyana	2005	Asphyxiation	0.2471
Guyana	2010	Asphyxiation	0.2471
Guyana	2017	Asphyxiation	0.2471
Guyana	1990	Crush injury	0.1325
Guyana	1995	Crush injury	0.1325
Guyana	2000	Crush injury	0.1325
Guyana	2005	Crush injury	0.1325
Guyana	2010	Crush injury	0.1325
Guyana	2017	Crush injury	0.1325
Guyana	1990	Nerve injury	0.1129
Guyana	1995	Nerve injury	0.1129
Guyana	2000	Nerve injury	0.1129
Guyana	2005	Nerve injury	0.1129
Guyana	2010	Nerve injury	0.1129
Guyana	2017	Nerve injury	0.1129
Guyana	1990	Injury to eyes	0.0165
Guyana	1995	Injury to eyes	0.0165
Guyana	2000	Injury to eyes	0.0165
Guyana	2005	Injury to eyes	0.0165
Guyana	2010	Injury to eyes	0.0165
Guyana	2017	Injury to eyes	0.0165
Guyana	1990	Amputation of lower limb, unilateral	0.1025
Guyana	1995	Amputation of lower limb, unilateral	0.0975
Guyana	2000	Amputation of lower limb, unilateral	0.094
Guyana	2005	Amputation of lower limb, unilateral	0.0928
Guyana	2010	Amputation of lower limb, unilateral	0.0902
Guyana	2017	Amputation of lower limb, unilateral	0.0818
Guyana	1990	Open wound(s)	0.011
Guyana	1995	Open wound(s)	0.011
Guyana	2000	Open wound(s)	0.011
Guyana	2005	Open wound(s)	0.011
Guyana	2010	Open wound(s)	0.011
Guyana	2017	Open wound(s)	0.011
Guyana	1990	Poisoning requiring urgent care	0.1628
Guyana	1995	Poisoning requiring urgent care	0.1628
Guyana	2000	Poisoning requiring urgent care	0.1628
Guyana	2005	Poisoning requiring urgent care	0.1628
Guyana	2010	Poisoning requiring urgent care	0.1628
Guyana	2017	Poisoning requiring urgent care	0.1628
Guyana	1990	Severe chest injury	0.0474
Guyana	1995	Severe chest injury	0.0474
Guyana	2000	Severe chest injury	0.0474
Guyana	2005	Severe chest injury	0.0474
Guyana	2010	Severe chest injury	0.0474
Guyana	2017	Severe chest injury	0.0474
Guyana	1990	Internal hemorrhage in abdomen and pelvis	0.1143
Guyana	1995	Internal hemorrhage in abdomen and pelvis	0.1143
Guyana	2000	Internal hemorrhage in abdomen and pelvis	0.1143
Guyana	2005	Internal hemorrhage in abdomen and pelvis	0.1143
Guyana	2010	Internal hemorrhage in abdomen and pelvis	0.1143
Guyana	2017	Internal hemorrhage in abdomen and pelvis	0.1143
Guyana	1990	Contusion in any part of the body	0.0075
Guyana	1995	Contusion in any part of the body	0.0075
Guyana	2000	Contusion in any part of the body	0.0075
Guyana	2005	Contusion in any part of the body	0.0075
Guyana	2010	Contusion in any part of the body	0.0075
Guyana	2017	Contusion in any part of the body	0.0075
Guyana	1990	Effect of different environmental factors	0.049
Guyana	1995	Effect of different environmental factors	0.049
Guyana	2000	Effect of different environmental factors	0.049
Guyana	2005	Effect of different environmental factors	0.049
Guyana	2010	Effect of different environmental factors	0.049
Guyana	2017	Effect of different environmental factors	0.049
Guyana	1990	Complications following therapeutic procedures	0.0506
Guyana	1995	Complications following therapeutic procedures	0.0506
Guyana	2000	Complications following therapeutic procedures	0.0506
Guyana	2005	Complications following therapeutic procedures	0.0506
Guyana	2010	Complications following therapeutic procedures	0.0506
Guyana	2017	Complications following therapeutic procedures	0.0506
Guyana	1990	Multiple fractures, dislocations, crashes, wounds, sprains, and strains	0.2208
Guyana	1995	Multiple fractures, dislocations, crashes, wounds, sprains, and strains	0.2078
Guyana	2000	Multiple fractures, dislocations, crashes, wounds, sprains, and strains	0.199
Guyana	2005	Multiple fractures, dislocations, crashes, wounds, sprains, and strains	0.1959
Guyana	2010	Multiple fractures, dislocations, crashes, wounds, sprains, and strains	0.1893
Guyana	2017	Multiple fractures, dislocations, crashes, wounds, sprains, and strains	0.1677
Guyana	1990	Amputation of upper limb, unilateral	0.0764
Guyana	1995	Amputation of upper limb, unilateral	0.0734
Guyana	2000	Amputation of upper limb, unilateral	0.0714
Guyana	2005	Amputation of upper limb, unilateral	0.0707
Guyana	2010	Amputation of upper limb, unilateral	0.0692
Guyana	2017	Amputation of upper limb, unilateral	0.0642
Guyana	1990	Amputation of thumb	0.0114
Guyana	1995	Amputation of thumb	0.0114
Guyana	2000	Amputation of thumb	0.0114
Guyana	2005	Amputation of thumb	0.0114
Guyana	2010	Amputation of thumb	0.0114
Guyana	2017	Amputation of thumb	0.0114
Guyana	1990	Amputation of toe/toes	0.0059
Guyana	1995	Amputation of toe/toes	0.0059
Guyana	2000	Amputation of toe/toes	0.0059
Guyana	2005	Amputation of toe/toes	0.0059
Guyana	2010	Amputation of toe/toes	0.0059
Guyana	2017	Amputation of toe/toes	0.0059
Guyana	1990	Burns, <20% total burned surface area without lower airway burns	0.0163
Guyana	1995	Burns, <20% total burned surface area without lower airway burns	0.0163
Guyana	2000	Burns, <20% total burned surface area without lower airway burns	0.0163
Guyana	2005	Burns, <20% total burned surface area without lower airway burns	0.0163
Guyana	2010	Burns, <20% total burned surface area without lower airway burns	0.0163
Guyana	2017	Burns, <20% total burned surface area without lower airway burns	0.0163
Guyana	1990	Burns, >=20% total burned surface area or >= 10% burned surface area if head/neck or hands/wrist involved w/o lower airway burns	0.2865
Guyana	1995	Burns, >=20% total burned surface area or >= 10% burned surface area if head/neck or hands/wrist involved w/o lower airway burns	0.2744
Guyana	2000	Burns, >=20% total burned surface area or >= 10% burned surface area if head/neck or hands/wrist involved w/o lower airway burns	0.2661
Guyana	2005	Burns, >=20% total burned surface area or >= 10% burned surface area if head/neck or hands/wrist involved w/o lower airway burns	0.2633
Guyana	2010	Burns, >=20% total burned surface area or >= 10% burned surface area if head/neck or hands/wrist involved w/o lower airway burns	0.2571
Guyana	2017	Burns, >=20% total burned surface area or >= 10% burned surface area if head/neck or hands/wrist involved w/o lower airway burns	0.237
Haiti	1990	Amputation of lower limbs, bilateral	0.319
Haiti	1995	Amputation of lower limbs, bilateral	0.3042
Haiti	2000	Amputation of lower limbs, bilateral	0.2969
Haiti	2005	Amputation of lower limbs, bilateral	0.2856
Haiti	2010	Amputation of lower limbs, bilateral	0.2807
Haiti	2017	Amputation of lower limbs, bilateral	0.2667
Haiti	1990	Lower airway burns	0.2252
Haiti	1995	Lower airway burns	0.2252
Haiti	2000	Lower airway burns	0.2252
Haiti	2005	Lower airway burns	0.2252
Haiti	2010	Lower airway burns	0.2252
Haiti	2017	Lower airway burns	0.2252
Haiti	1990	Dislocation of hip	0.0159
Haiti	1995	Dislocation of hip	0.0159
Haiti	2000	Dislocation of hip	0.0159
Haiti	2005	Dislocation of hip	0.0159
Haiti	2010	Dislocation of hip	0.0159
Haiti	2017	Dislocation of hip	0.0159
Haiti	1990	Dislocation of knee	0.1134
Haiti	1995	Dislocation of knee	0.1134
Haiti	2000	Dislocation of knee	0.1134
Haiti	2005	Dislocation of knee	0.1134
Haiti	2010	Dislocation of knee	0.1134
Haiti	2017	Dislocation of knee	0.1134
Haiti	1990	Dislocation of shoulder	0.062
Haiti	1995	Dislocation of shoulder	0.062
Haiti	2000	Dislocation of shoulder	0.062
Haiti	2005	Dislocation of shoulder	0.062
Haiti	2010	Dislocation of shoulder	0.062
Haiti	2017	Dislocation of shoulder	0.062
Haiti	1990	Muscle and tendon injuries, including sprains and strains lesser dislocations	0.0075
Haiti	1995	Muscle and tendon injuries, including sprains and strains lesser dislocations	0.0075
Haiti	2000	Muscle and tendon injuries, including sprains and strains lesser dislocations	0.0075
Haiti	2005	Muscle and tendon injuries, including sprains and strains lesser dislocations	0.0075
Haiti	2010	Muscle and tendon injuries, including sprains and strains lesser dislocations	0.0075
Haiti	2017	Muscle and tendon injuries, including sprains and strains lesser dislocations	0.0075
Haiti	1990	Fracture of clavicle, scapula, or humerus	0.0349
Haiti	1995	Fracture of clavicle, scapula, or humerus	0.0349
Haiti	2000	Fracture of clavicle, scapula, or humerus	0.0349
Haiti	2005	Fracture of clavicle, scapula, or humerus	0.0349
Haiti	2010	Fracture of clavicle, scapula, or humerus	0.0349
Haiti	2017	Fracture of clavicle, scapula, or humerus	0.0349
Haiti	1990	Fracture of face bones	0.0669
Haiti	1995	Fracture of face bones	0.0669
Haiti	2000	Fracture of face bones	0.0669
Haiti	2005	Fracture of face bones	0.0669
Haiti	2010	Fracture of face bones	0.0669
Haiti	2017	Fracture of face bones	0.0669
Haiti	1990	Fracture of foot bones except ankle	0.0264
Haiti	1995	Fracture of foot bones except ankle	0.0264
Haiti	2000	Fracture of foot bones except ankle	0.0264
Haiti	2005	Fracture of foot bones except ankle	0.0264
Haiti	2010	Fracture of foot bones except ankle	0.0264
Haiti	2017	Fracture of foot bones except ankle	0.0264
Haiti	1990	Fracture of hand (wrist and other distal part of hand)	0.0138
Haiti	1995	Fracture of hand (wrist and other distal part of hand)	0.0138
Haiti	2000	Fracture of hand (wrist and other distal part of hand)	0.0138
Haiti	2005	Fracture of hand (wrist and other distal part of hand)	0.0138
Haiti	2010	Fracture of hand (wrist and other distal part of hand)	0.0138
Haiti	2017	Fracture of hand (wrist and other distal part of hand)	0.0138
Haiti	1990	Fracture of hip	0.3167
Haiti	1995	Fracture of hip	0.2971
Haiti	2000	Fracture of hip	0.2874
Haiti	2005	Fracture of hip	0.2725
Haiti	2010	Fracture of hip	0.266
Haiti	2017	Fracture of hip	0.2474
Haiti	1990	Amputation of upper limbs, bilateral	0.3551
Haiti	1995	Amputation of upper limbs, bilateral	0.3348
Haiti	2000	Amputation of upper limbs, bilateral	0.3249
Haiti	2005	Amputation of upper limbs, bilateral	0.3094
Haiti	2010	Amputation of upper limbs, bilateral	0.3027
Haiti	2017	Amputation of upper limbs, bilateral	0.2836
Haiti	1990	Fracture of patella, tibia or fibula, or ankle	0.0554
Haiti	1995	Fracture of patella, tibia or fibula, or ankle	0.0554
Haiti	2000	Fracture of patella, tibia or fibula, or ankle	0.0554
Haiti	2005	Fracture of patella, tibia or fibula, or ankle	0.0554
Haiti	2010	Fracture of patella, tibia or fibula, or ankle	0.0554
Haiti	2017	Fracture of patella, tibia or fibula, or ankle	0.0554
Haiti	1990	Fracture of pelvis	0.1823
Haiti	1995	Fracture of pelvis	0.1823
Haiti	2000	Fracture of pelvis	0.1823
Haiti	2005	Fracture of pelvis	0.1823
Haiti	2010	Fracture of pelvis	0.1823
Haiti	2017	Fracture of pelvis	0.1823
Haiti	1990	Fracture of radius and/or ulna	0.0435
Haiti	1995	Fracture of radius and/or ulna	0.0435
Haiti	2000	Fracture of radius and/or ulna	0.0435
Haiti	2005	Fracture of radius and/or ulna	0.0435
Haiti	2010	Fracture of radius and/or ulna	0.0435
Haiti	2017	Fracture of radius and/or ulna	0.0435
Haiti	1990	Fracture of skull	0.0714
Haiti	1995	Fracture of skull	0.0714
Haiti	2000	Fracture of skull	0.0714
Haiti	2005	Fracture of skull	0.0714
Haiti	2010	Fracture of skull	0.0714
Haiti	2017	Fracture of skull	0.0714
Haiti	1990	Fracture of sternum and/or fracture of one or more ribs	0.1027
Haiti	1995	Fracture of sternum and/or fracture of one or more ribs	0.1027
Haiti	2000	Fracture of sternum and/or fracture of one or more ribs	0.1027
Haiti	2005	Fracture of sternum and/or fracture of one or more ribs	0.1027
Haiti	2010	Fracture of sternum and/or fracture of one or more ribs	0.1027
Haiti	2017	Fracture of sternum and/or fracture of one or more ribs	0.1027
Haiti	1990	Fracture of vertebral column	0.1106
Haiti	1995	Fracture of vertebral column	0.1106
Haiti	2000	Fracture of vertebral column	0.1106
Haiti	2005	Fracture of vertebral column	0.1106
Haiti	2010	Fracture of vertebral column	0.1106
Haiti	2017	Fracture of vertebral column	0.1106
Haiti	1990	Fracture of femur, other than femoral neck	0.042
Haiti	1995	Fracture of femur, other than femoral neck	0.042
Haiti	2000	Fracture of femur, other than femoral neck	0.042
Haiti	2005	Fracture of femur, other than femoral neck	0.042
Haiti	2010	Fracture of femur, other than femoral neck	0.042
Haiti	2017	Fracture of femur, other than femoral neck	0.042
Haiti	1990	Minor TBI	0.1319
Haiti	1995	Minor TBI	0.1319
Haiti	2000	Minor TBI	0.1319
Haiti	2005	Minor TBI	0.1319
Haiti	2010	Minor TBI	0.1319
Haiti	2017	Minor TBI	0.1319
Haiti	1990	Moderate TBI	0.1637
Haiti	1995	Moderate TBI	0.1637
Haiti	2000	Moderate TBI	0.1637
Haiti	2005	Moderate TBI	0.1637
Haiti	2010	Moderate TBI	0.1637
Haiti	2017	Moderate TBI	0.1637
Haiti	1990	Amputation of fingers (excluding thumb)	0.0047
Haiti	1995	Amputation of fingers (excluding thumb)	0.0047
Haiti	2000	Amputation of fingers (excluding thumb)	0.0047
Haiti	2005	Amputation of fingers (excluding thumb)	0.0047
Haiti	2010	Amputation of fingers (excluding thumb)	0.0047
Haiti	2017	Amputation of fingers (excluding thumb)	0.0047
Haiti	1990	Spinal cord lesion at neck level	0.5014
Haiti	1995	Spinal cord lesion at neck level	0.4946
Haiti	2000	Spinal cord lesion at neck level	0.4913
Haiti	2005	Spinal cord lesion at neck level	0.4861
Haiti	2010	Spinal cord lesion at neck level	0.4839
Haiti	2017	Spinal cord lesion at neck level	0.4774
Haiti	1990	Spinal cord lesion below neck level	0.3667
Haiti	1995	Spinal cord lesion below neck level	0.3524
Haiti	2000	Spinal cord lesion below neck level	0.3453
Haiti	2005	Spinal cord lesion below neck level	0.3344
Haiti	2010	Spinal cord lesion below neck level	0.3297
Haiti	2017	Spinal cord lesion below neck level	0.3161
Haiti	1990	Drowning and nonfatal submersion	0.2471
Haiti	1995	Drowning and nonfatal submersion	0.2471
Haiti	2000	Drowning and nonfatal submersion	0.2471
Haiti	2005	Drowning and nonfatal submersion	0.2471
Haiti	2010	Drowning and nonfatal submersion	0.2471
Haiti	2017	Drowning and nonfatal submersion	0.2471
Haiti	1990	Asphyxiation	0.2471
Haiti	1995	Asphyxiation	0.2471
Haiti	2000	Asphyxiation	0.2471
Haiti	2005	Asphyxiation	0.2471
Haiti	2010	Asphyxiation	0.2471
Haiti	2017	Asphyxiation	0.2471
Haiti	1990	Crush injury	0.1325
Haiti	1995	Crush injury	0.1325
Haiti	2000	Crush injury	0.1325
Haiti	2005	Crush injury	0.1325
Haiti	2010	Crush injury	0.1325
Haiti	2017	Crush injury	0.1325
Haiti	1990	Nerve injury	0.1129
Haiti	1995	Nerve injury	0.1129
Haiti	2000	Nerve injury	0.1129
Haiti	2005	Nerve injury	0.1129
Haiti	2010	Nerve injury	0.1129
Haiti	2017	Nerve injury	0.1129
Haiti	1990	Injury to eyes	0.0165
Haiti	1995	Injury to eyes	0.0165
Haiti	2000	Injury to eyes	0.0165
Haiti	2005	Injury to eyes	0.0165
Haiti	2010	Injury to eyes	0.0165
Haiti	2017	Injury to eyes	0.0165
Haiti	1990	Amputation of lower limb, unilateral	0.1399
Haiti	1995	Amputation of lower limb, unilateral	0.1323
Haiti	2000	Amputation of lower limb, unilateral	0.1285
Haiti	2005	Amputation of lower limb, unilateral	0.1227
Haiti	2010	Amputation of lower limb, unilateral	0.1202
Haiti	2017	Amputation of lower limb, unilateral	0.1129
Haiti	1990	Open wound(s)	0.011
Haiti	1995	Open wound(s)	0.011
Haiti	2000	Open wound(s)	0.011
Haiti	2005	Open wound(s)	0.011
Haiti	2010	Open wound(s)	0.011
Haiti	2017	Open wound(s)	0.011
Haiti	1990	Poisoning requiring urgent care	0.1628
Haiti	1995	Poisoning requiring urgent care	0.1628
Haiti	2000	Poisoning requiring urgent care	0.1628
Haiti	2005	Poisoning requiring urgent care	0.1628
Haiti	2010	Poisoning requiring urgent care	0.1628
Haiti	2017	Poisoning requiring urgent care	0.1628
Haiti	1990	Severe chest injury	0.0474
Haiti	1995	Severe chest injury	0.0474
Haiti	2000	Severe chest injury	0.0474
Haiti	2005	Severe chest injury	0.0474
Haiti	2010	Severe chest injury	0.0474
Haiti	2017	Severe chest injury	0.0474
Haiti	1990	Internal hemorrhage in abdomen and pelvis	0.1143
Haiti	1995	Internal hemorrhage in abdomen and pelvis	0.1143
Haiti	2000	Internal hemorrhage in abdomen and pelvis	0.1143
Haiti	2005	Internal hemorrhage in abdomen and pelvis	0.1143
Haiti	2010	Internal hemorrhage in abdomen and pelvis	0.1143
Haiti	2017	Internal hemorrhage in abdomen and pelvis	0.1143
Haiti	1990	Contusion in any part of the body	0.0075
Haiti	1995	Contusion in any part of the body	0.0075
Haiti	2000	Contusion in any part of the body	0.0075
Haiti	2005	Contusion in any part of the body	0.0075
Haiti	2010	Contusion in any part of the body	0.0075
Haiti	2017	Contusion in any part of the body	0.0075
Haiti	1990	Effect of different environmental factors	0.049
Haiti	1995	Effect of different environmental factors	0.049
Haiti	2000	Effect of different environmental factors	0.049
Haiti	2005	Effect of different environmental factors	0.049
Haiti	2010	Effect of different environmental factors	0.049
Haiti	2017	Effect of different environmental factors	0.049
Haiti	1990	Complications following therapeutic procedures	0.0506
Haiti	1995	Complications following therapeutic procedures	0.0506
Haiti	2000	Complications following therapeutic procedures	0.0506
Haiti	2005	Complications following therapeutic procedures	0.0506
Haiti	2010	Complications following therapeutic procedures	0.0506
Haiti	2017	Complications following therapeutic procedures	0.0506
Haiti	1990	Multiple fractures, dislocations, crashes, wounds, sprains, and strains	0.3167
Haiti	1995	Multiple fractures, dislocations, crashes, wounds, sprains, and strains	0.2971
Haiti	2000	Multiple fractures, dislocations, crashes, wounds, sprains, and strains	0.2874
Haiti	2005	Multiple fractures, dislocations, crashes, wounds, sprains, and strains	0.2725
Haiti	2010	Multiple fractures, dislocations, crashes, wounds, sprains, and strains	0.266
Haiti	2017	Multiple fractures, dislocations, crashes, wounds, sprains, and strains	0.2474
Haiti	1990	Amputation of upper limb, unilateral	0.0984
Haiti	1995	Amputation of upper limb, unilateral	0.0939
Haiti	2000	Amputation of upper limb, unilateral	0.0917
Haiti	2005	Amputation of upper limb, unilateral	0.0883
Haiti	2010	Amputation of upper limb, unilateral	0.0868
Haiti	2017	Amputation of upper limb, unilateral	0.0825
Haiti	1990	Amputation of thumb	0.0114
Haiti	1995	Amputation of thumb	0.0114
Haiti	2000	Amputation of thumb	0.0114
Haiti	2005	Amputation of thumb	0.0114
Haiti	2010	Amputation of thumb	0.0114
Haiti	2017	Amputation of thumb	0.0114
Haiti	1990	Amputation of toe/toes	0.0059
Haiti	1995	Amputation of toe/toes	0.0059
Haiti	2000	Amputation of toe/toes	0.0059
Haiti	2005	Amputation of toe/toes	0.0059
Haiti	2010	Amputation of toe/toes	0.0059
Haiti	2017	Amputation of toe/toes	0.0059
Haiti	1990	Burns, <20% total burned surface area without lower airway burns	0.0163
Haiti	1995	Burns, <20% total burned surface area without lower airway burns	0.0163
Haiti	2000	Burns, <20% total burned surface area without lower airway burns	0.0163
Haiti	2005	Burns, <20% total burned surface area without lower airway burns	0.0163
Haiti	2010	Burns, <20% total burned surface area without lower airway burns	0.0163
Haiti	2017	Burns, <20% total burned surface area without lower airway burns	0.0163
Haiti	1990	Burns, >=20% total burned surface area or >= 10% burned surface area if head/neck or hands/wrist involved w/o lower airway burns	0.3758
Haiti	1995	Burns, >=20% total burned surface area or >= 10% burned surface area if head/neck or hands/wrist involved w/o lower airway burns	0.3575
Haiti	2000	Burns, >=20% total burned surface area or >= 10% burned surface area if head/neck or hands/wrist involved w/o lower airway burns	0.3485
Haiti	2005	Burns, >=20% total burned surface area or >= 10% burned surface area if head/neck or hands/wrist involved w/o lower airway burns	0.3346
Haiti	2010	Burns, >=20% total burned surface area or >= 10% burned surface area if head/neck or hands/wrist involved w/o lower airway burns	0.3286
Haiti	2017	Burns, >=20% total burned surface area or >= 10% burned surface area if head/neck or hands/wrist involved w/o lower airway burns	0.3113
Jamaica	1990	Amputation of lower limbs, bilateral	0.2034
Jamaica	1995	Amputation of lower limbs, bilateral	0.1905
Jamaica	2000	Amputation of lower limbs, bilateral	0.1858
Jamaica	2005	Amputation of lower limbs, bilateral	0.1782
Jamaica	2010	Amputation of lower limbs, bilateral	0.1724
Jamaica	2017	Amputation of lower limbs, bilateral	0.1663
Jamaica	1990	Lower airway burns	0.2252
Jamaica	1995	Lower airway burns	0.2252
Jamaica	2000	Lower airway burns	0.2252
Jamaica	2005	Lower airway burns	0.2252
Jamaica	2010	Lower airway burns	0.2252
Jamaica	2017	Lower airway burns	0.2252
Jamaica	1990	Dislocation of hip	0.0159
Jamaica	1995	Dislocation of hip	0.0159
Jamaica	2000	Dislocation of hip	0.0159
Jamaica	2005	Dislocation of hip	0.0159
Jamaica	2010	Dislocation of hip	0.0159
Jamaica	2017	Dislocation of hip	0.0159
Jamaica	1990	Dislocation of knee	0.1134
Jamaica	1995	Dislocation of knee	0.1134
Jamaica	2000	Dislocation of knee	0.1134
Jamaica	2005	Dislocation of knee	0.1134
Jamaica	2010	Dislocation of knee	0.1134
Jamaica	2017	Dislocation of knee	0.1134
Jamaica	1990	Dislocation of shoulder	0.062
Jamaica	1995	Dislocation of shoulder	0.062
Jamaica	2000	Dislocation of shoulder	0.062
Jamaica	2005	Dislocation of shoulder	0.062
Jamaica	2010	Dislocation of shoulder	0.062
Jamaica	2017	Dislocation of shoulder	0.062
Jamaica	1990	Muscle and tendon injuries, including sprains and strains lesser dislocations	0.0075
Jamaica	1995	Muscle and tendon injuries, including sprains and strains lesser dislocations	0.0075
Jamaica	2000	Muscle and tendon injuries, including sprains and strains lesser dislocations	0.0075
Jamaica	2005	Muscle and tendon injuries, including sprains and strains lesser dislocations	0.0075
Jamaica	2010	Muscle and tendon injuries, including sprains and strains lesser dislocations	0.0075
Jamaica	2017	Muscle and tendon injuries, including sprains and strains lesser dislocations	0.0075
Jamaica	1990	Fracture of clavicle, scapula, or humerus	0.0349
Jamaica	1995	Fracture of clavicle, scapula, or humerus	0.0349
Jamaica	2000	Fracture of clavicle, scapula, or humerus	0.0349
Jamaica	2005	Fracture of clavicle, scapula, or humerus	0.0349
Jamaica	2010	Fracture of clavicle, scapula, or humerus	0.0349
Jamaica	2017	Fracture of clavicle, scapula, or humerus	0.0349
Jamaica	1990	Fracture of face bones	0.0669
Jamaica	1995	Fracture of face bones	0.0669
Jamaica	2000	Fracture of face bones	0.0669
Jamaica	2005	Fracture of face bones	0.0669
Jamaica	2010	Fracture of face bones	0.0669
Jamaica	2017	Fracture of face bones	0.0669
Jamaica	1990	Fracture of foot bones except ankle	0.0264
Jamaica	1995	Fracture of foot bones except ankle	0.0264

Jamaica	2000	Fracture of foot bones except ankle	0.0264
Jamaica	2005	Fracture of foot bones except ankle	0.0264
Jamaica	2010	Fracture of foot bones except ankle	0.0264
Jamaica	2017	Fracture of foot bones except ankle	0.0264
Jamaica	1990	Fracture of hand (wrist and other distal part of hand)	0.0138
Jamaica	1995	Fracture of hand (wrist and other distal part of hand)	0.0138
Jamaica	2000	Fracture of hand (wrist and other distal part of hand)	0.0138
Jamaica	2005	Fracture of hand (wrist and other distal part of hand)	0.0138
Jamaica	2010	Fracture of hand (wrist and other distal part of hand)	0.0138
Jamaica	2017	Fracture of hand (wrist and other distal part of hand)	0.0138
Jamaica	1990	Fracture of hip	0.1637
Jamaica	1995	Fracture of hip	0.1466
Jamaica	2000	Fracture of hip	0.1404
Jamaica	2005	Fracture of hip	0.1304
Jamaica	2010	Fracture of hip	0.1227
Jamaica	2017	Fracture of hip	0.1146
Jamaica	1990	Amputation of upper limbs, bilateral	0.1971
Jamaica	1995	Amputation of upper limbs, bilateral	0.1795
Jamaica	2000	Amputation of upper limbs, bilateral	0.173
Jamaica	2005	Amputation of upper limbs, bilateral	0.1627
Jamaica	2010	Amputation of upper limbs, bilateral	0.1547
Jamaica	2017	Amputation of upper limbs, bilateral	0.1465
Jamaica	1990	Fracture of patella, tibia or fibula, or ankle	0.0554
Jamaica	1995	Fracture of patella, tibia or fibula, or ankle	0.0554
Jamaica	2000	Fracture of patella, tibia or fibula, or ankle	0.0554
Jamaica	2005	Fracture of patella, tibia or fibula, or ankle	0.0554
Jamaica	2010	Fracture of patella, tibia or fibula, or ankle	0.0554
Jamaica	2017	Fracture of patella, tibia or fibula, or ankle	0.0554
Jamaica	1990	Fracture of pelvis	0.1823
Jamaica	1995	Fracture of pelvis	0.1823
Jamaica	2000	Fracture of pelvis	0.1823
Jamaica	2005	Fracture of pelvis	0.1823
Jamaica	2010	Fracture of pelvis	0.1823
Jamaica	2017	Fracture of pelvis	0.1823
Jamaica	1990	Fracture of radius and/or ulna	0.0435
Jamaica	1995	Fracture of radius and/or ulna	0.0435
Jamaica	2000	Fracture of radius and/or ulna	0.0435
Jamaica	2005	Fracture of radius and/or ulna	0.0435
Jamaica	2010	Fracture of radius and/or ulna	0.0435
Jamaica	2017	Fracture of radius and/or ulna	0.0435
Jamaica	1990	Fracture of skull	0.0714
Jamaica	1995	Fracture of skull	0.0714
Jamaica	2000	Fracture of skull	0.0714
Jamaica	2005	Fracture of skull	0.0714
Jamaica	2010	Fracture of skull	0.0714
Jamaica	2017	Fracture of skull	0.0714
Jamaica	1990	Fracture of sternum and/or fracture of one or more ribs	0.1027
Jamaica	1995	Fracture of sternum and/or fracture of one or more ribs	0.1027
Jamaica	2000	Fracture of sternum and/or fracture of one or more ribs	0.1027
Jamaica	2005	Fracture of sternum and/or fracture of one or more ribs	0.1027
Jamaica	2010	Fracture of sternum and/or fracture of one or more ribs	0.1027
Jamaica	2017	Fracture of sternum and/or fracture of one or more ribs	0.1027
Jamaica	1990	Fracture of vertebral column	0.1106
Jamaica	1995	Fracture of vertebral column	0.1106
Jamaica	2000	Fracture of vertebral column	0.1106
Jamaica	2005	Fracture of vertebral column	0.1106
Jamaica	2010	Fracture of vertebral column	0.1106
Jamaica	2017	Fracture of vertebral column	0.1106
Jamaica	1990	Fracture of femur, other than femoral neck	0.042
Jamaica	1995	Fracture of femur, other than femoral neck	0.042
Jamaica	2000	Fracture of femur, other than femoral neck	0.042
Jamaica	2005	Fracture of femur, other than femoral neck	0.042
Jamaica	2010	Fracture of femur, other than femoral neck	0.042
Jamaica	2017	Fracture of femur, other than femoral neck	0.042
Jamaica	1990	Minor TBI	0.1319
Jamaica	1995	Minor TBI	0.1319
Jamaica	2000	Minor TBI	0.1319
Jamaica	2005	Minor TBI	0.1319
Jamaica	2010	Minor TBI	0.1319
Jamaica	2017	Minor TBI	0.1319
Jamaica	1990	Moderate TBI	0.1637
Jamaica	1995	Moderate TBI	0.1637
Jamaica	2000	Moderate TBI	0.1637
Jamaica	2005	Moderate TBI	0.1637
Jamaica	2010	Moderate TBI	0.1637
Jamaica	2017	Moderate TBI	0.1637
Jamaica	1990	Amputation of fingers (excluding thumb)	0.0047
Jamaica	1995	Amputation of fingers (excluding thumb)	0.0047
Jamaica	2000	Amputation of fingers (excluding thumb)	0.0047
Jamaica	2005	Amputation of fingers (excluding thumb)	0.0047
Jamaica	2010	Amputation of fingers (excluding thumb)	0.0047
Jamaica	2017	Amputation of fingers (excluding thumb)	0.0047
Jamaica	1990	Spinal cord lesion at neck level	0.4485
Jamaica	1995	Spinal cord lesion at neck level	0.4426
Jamaica	2000	Spinal cord lesion at neck level	0.4404
Jamaica	2005	Spinal cord lesion at neck level	0.437
Jamaica	2010	Spinal cord lesion at neck level	0.4343
Jamaica	2017	Spinal cord lesion at neck level	0.4315
Jamaica	1990	Spinal cord lesion below neck level	0.2551
Jamaica	1995	Spinal cord lesion below neck level	0.2426
Jamaica	2000	Spinal cord lesion below neck level	0.238
Jamaica	2005	Spinal cord lesion below neck level	0.2307
Jamaica	2010	Spinal cord lesion below neck level	0.2251
Jamaica	2017	Spinal cord lesion below neck level	0.2192
Jamaica	1990	Drowning and nonfatal submersion	0.2471
Jamaica	1995	Drowning and nonfatal submersion	0.2471
Jamaica	2000	Drowning and nonfatal submersion	0.2471
Jamaica	2005	Drowning and nonfatal submersion	0.2471
Jamaica	2010	Drowning and nonfatal submersion	0.2471
Jamaica	2017	Drowning and nonfatal submersion	0.2471
Jamaica	1990	Asphyxiation	0.2471
Jamaica	1995	Asphyxiation	0.2471
Jamaica	2000	Asphyxiation	0.2471
Jamaica	2005	Asphyxiation	0.2471
Jamaica	2010	Asphyxiation	0.2471
Jamaica	2017	Asphyxiation	0.2471
Jamaica	1990	Crush injury	0.1325
Jamaica	1995	Crush injury	0.1325
Jamaica	2000	Crush injury	0.1325
Jamaica	2005	Crush injury	0.1325
Jamaica	2010	Crush injury	0.1325
Jamaica	2017	Crush injury	0.1325
Jamaica	1990	Nerve injury	0.1129
Jamaica	1995	Nerve injury	0.1129
Jamaica	2000	Nerve injury	0.1129
Jamaica	2005	Nerve injury	0.1129
Jamaica	2010	Nerve injury	0.1129
Jamaica	2017	Nerve injury	0.1129
Jamaica	1990	Injury to eyes	0.0165
Jamaica	1995	Injury to eyes	0.0165
Jamaica	2000	Injury to eyes	0.0165
Jamaica	2005	Injury to eyes	0.0165
Jamaica	2010	Injury to eyes	0.0165
Jamaica	2017	Injury to eyes	0.0165
Jamaica	1990	Amputation of lower limb, unilateral	0.0803
Jamaica	1995	Amputation of lower limb, unilateral	0.0736
Jamaica	2000	Amputation of lower limb, unilateral	0.0712
Jamaica	2005	Amputation of lower limb, unilateral	0.0673
Jamaica	2010	Amputation of lower limb, unilateral	0.0643
Jamaica	2017	Amputation of lower limb, unilateral	0.0612
Jamaica	1990	Open wound(s)	0.011
Jamaica	1995	Open wound(s)	0.011
Jamaica	2000	Open wound(s)	0.011
Jamaica	2005	Open wound(s)	0.011
Jamaica	2010	Open wound(s)	0.011
Jamaica	2017	Open wound(s)	0.011
Jamaica	1990	Poisoning requiring urgent care	0.1628
Jamaica	1995	Poisoning requiring urgent care	0.1628
Jamaica	2000	Poisoning requiring urgent care	0.1628
Jamaica	2005	Poisoning requiring urgent care	0.1628
Jamaica	2010	Poisoning requiring urgent care	0.1628
Jamaica	2017	Poisoning requiring urgent care	0.1628
Jamaica	1990	Severe chest injury	0.0474
Jamaica	1995	Severe chest injury	0.0474
Jamaica	2000	Severe chest injury	0.0474
Jamaica	2005	Severe chest injury	0.0474
Jamaica	2010	Severe chest injury	0.0474
Jamaica	2017	Severe chest injury	0.0474
Jamaica	1990	Internal hemorrhage in abdomen and pelvis	0.1143
Jamaica	1995	Internal hemorrhage in abdomen and pelvis	0.1143
Jamaica	2000	Internal hemorrhage in abdomen and pelvis	0.1143
Jamaica	2005	Internal hemorrhage in abdomen and pelvis	0.1143
Jamaica	2010	Internal hemorrhage in abdomen and pelvis	0.1143
Jamaica	2017	Internal hemorrhage in abdomen and pelvis	0.1143
Jamaica	1990	Contusion in any part of the body	0.0075
Jamaica	1995	Contusion in any part of the body	0.0075
Jamaica	2000	Contusion in any part of the body	0.0075
Jamaica	2005	Contusion in any part of the body	0.0075
Jamaica	2010	Contusion in any part of the body	0.0075
Jamaica	2017	Contusion in any part of the body	0.0075
Jamaica	1990	Effect of different environmental factors	0.049
Jamaica	1995	Effect of different environmental factors	0.049
Jamaica	2000	Effect of different environmental factors	0.049
Jamaica	2005	Effect of different environmental factors	0.049
Jamaica	2010	Effect of different environmental factors	0.049
Jamaica	2017	Effect of different environmental factors	0.049
Jamaica	1990	Complications following therapeutic procedures	0.0506
Jamaica	1995	Complications following therapeutic procedures	0.0506
Jamaica	2000	Complications following therapeutic procedures	0.0506
Jamaica	2005	Complications following therapeutic procedures	0.0506
Jamaica	2010	Complications following therapeutic procedures	0.0506
Jamaica	2017	Complications following therapeutic procedures	0.0506
Jamaica	1990	Multiple fractures, dislocations, crashes, wounds, sprains, and strains	0.1637
Jamaica	1995	Multiple fractures, dislocations, crashes, wounds, sprains, and strains	0.1466
Jamaica	2000	Multiple fractures, dislocations, crashes, wounds, sprains, and strains	0.1404
Jamaica	2005	Multiple fractures, dislocations, crashes, wounds, sprains, and strains	0.1304
Jamaica	2010	Multiple fractures, dislocations, crashes, wounds, sprains, and strains	0.1227
Jamaica	2017	Multiple fractures, dislocations, crashes, wounds, sprains, and strains	0.1146
Jamaica	1990	Amputation of upper limb, unilateral	0.0633
Jamaica	1995	Amputation of upper limb, unilateral	0.0594
Jamaica	2000	Amputation of upper limb, unilateral	0.058
Jamaica	2005	Amputation of upper limb, unilateral	0.0557
Jamaica	2010	Amputation of upper limb, unilateral	0.0539
Jamaica	2017	Amputation of upper limb, unilateral	0.0521
Jamaica	1990	Amputation of thumb	0.0114
Jamaica	1995	Amputation of thumb	0.0114
Jamaica	2000	Amputation of thumb	0.0114
Jamaica	2005	Amputation of thumb	0.0114
Jamaica	2010	Amputation of thumb	0.0114
Jamaica	2017	Amputation of thumb	0.0114
Jamaica	1990	Amputation of toe/toes	0.0059
Jamaica	1995	Amputation of toe/toes	0.0059
Jamaica	2000	Amputation of toe/toes	0.0059
Jamaica	2005	Amputation of toe/toes	0.0059
Jamaica	2010	Amputation of toe/toes	0.0059
Jamaica	2017	Amputation of toe/toes	0.0059
Jamaica	1990	Burns, <20% total burned surface area without lower airway burns	0.0163
Jamaica	1995	Burns, <20% total burned surface area without lower airway burns	0.0163
Jamaica	2000	Burns, <20% total burned surface area without lower airway burns	0.0163
Jamaica	2005	Burns, <20% total burned surface area without lower airway burns	0.0163
Jamaica	2010	Burns, <20% total burned surface area without lower airway burns	0.0163
Jamaica	2017	Burns, <20% total burned surface area without lower airway burns	0.0163
Jamaica	1990	Burns, >=20% total burned surface area or >= 10% burned surface area if head/neck or hands/wrist involved w/o lower airway burns	0.2333
Jamaica	1995	Burns, >=20% total burned surface area or >= 10% burned surface area if head/neck or hands/wrist involved w/o lower airway burns	0.2174
Jamaica	2000	Burns, >=20% total burned surface area or >= 10% burned surface area if head/neck or hands/wrist involved w/o lower airway burns	0.2116
Jamaica	2005	Burns, >=20% total burned surface area or >= 10% burned surface area if head/neck or hands/wrist involved w/o lower airway burns	0.2023
Jamaica	2010	Burns, >=20% total burned surface area or >= 10% burned surface area if head/neck or hands/wrist involved w/o lower airway burns	0.1951
Jamaica	2017	Burns, >=20% total burned surface area or >= 10% burned surface area if head/neck or hands/wrist involved w/o lower airway burns	0.1876
Saint Lucia	1990	Amputation of lower limbs, bilateral	0.2114
Saint Lucia	1995	Amputation of lower limbs, bilateral	0.1969
Saint Lucia	2000	Amputation of lower limbs, bilateral	0.1847
Saint Lucia	2005	Amputation of lower limbs, bilateral	0.1768
Saint Lucia	2010	Amputation of lower limbs, bilateral	0.1689
Saint Lucia	2017	Amputation of lower limbs, bilateral	0.1613
Saint Lucia	1990	Lower airway burns	0.2252
Saint Lucia	1995	Lower airway burns	0.2252
Saint Lucia	2000	Lower airway burns	0.2252
Saint Lucia	2005	Lower airway burns	0.2252
Saint Lucia	2010	Lower airway burns	0.2252
Saint Lucia	2017	Lower airway burns	0.2252
Saint Lucia	1990	Dislocation of hip	0.0159
Saint Lucia	1995	Dislocation of hip	0.0159
Saint Lucia	2000	Dislocation of hip	0.0159
Saint Lucia	2005	Dislocation of hip	0.0159
Saint Lucia	2010	Dislocation of hip	0.0159
Saint Lucia	2017	Dislocation of hip	0.0159
Saint Lucia	1990	Dislocation of knee	0.1134
Saint Lucia	1995	Dislocation of knee	0.1134
Saint Lucia	2000	Dislocation of knee	0.1134
Saint Lucia	2005	Dislocation of knee	0.1134
Saint Lucia	2010	Dislocation of knee	0.1134
Saint Lucia	2017	Dislocation of knee	0.1134
Saint Lucia	1990	Dislocation of shoulder	0.062
Saint Lucia	1995	Dislocation of shoulder	0.062
Saint Lucia	2000	Dislocation of shoulder	0.062
Saint Lucia	2005	Dislocation of shoulder	0.062
Saint Lucia	2010	Dislocation of shoulder	0.062
Saint Lucia	2017	Dislocation of shoulder	0.062
Saint Lucia	1990	Muscle and tendon injuries, including sprains and strains lesser dislocations	0.0075
Saint Lucia	1995	Muscle and tendon injuries, including sprains and strains lesser dislocations	0.0075
Saint Lucia	2000	Muscle and tendon injuries, including sprains and strains lesser dislocations	0.0075
Saint Lucia	2005	Muscle and tendon injuries, including sprains and strains lesser dislocations	0.0075
Saint Lucia	2010	Muscle and tendon injuries, including sprains and strains lesser dislocations	0.0075
Saint Lucia	2017	Muscle and tendon injuries, including sprains and strains lesser dislocations	0.0075
Saint Lucia	1990	Fracture of clavicle, scapula, or humerus	0.0349
Saint Lucia	1995	Fracture of clavicle, scapula, or humerus	0.0349
Saint Lucia	2000	Fracture of clavicle, scapula, or humerus	0.0349
Saint Lucia	2005	Fracture of clavicle, scapula, or humerus	0.0349
Saint Lucia	2010	Fracture of clavicle, scapula, or humerus	0.0349
Saint Lucia	2017	Fracture of clavicle, scapula, or humerus	0.0349
Saint Lucia	1990	Fracture of face bones	0.0669
Saint Lucia	1995	Fracture of face bones	0.0669
Saint Lucia	2000	Fracture of face bones	0.0669
Saint Lucia	2005	Fracture of face bones	0.0669
Saint Lucia	2010	Fracture of face bones	0.0669
Saint Lucia	2017	Fracture of face bones	0.0669
Saint Lucia	1990	Fracture of foot bones except ankle	0.0264
Saint Lucia	1995	Fracture of foot bones except ankle	0.0264
Saint Lucia	2000	Fracture of foot bones except ankle	0.0264
Saint Lucia	2005	Fracture of foot bones except ankle	0.0264
Saint Lucia	2010	Fracture of foot bones except ankle	0.0264
Saint Lucia	2017	Fracture of foot bones except ankle	0.0264
Saint Lucia	1990	Fracture of hand (wrist and other distal part of hand)	0.0138
Saint Lucia	1995	Fracture of hand (wrist and other distal part of hand)	0.0138
Saint Lucia	2000	Fracture of hand (wrist and other distal part of hand)	0.0138
Saint Lucia	2005	Fracture of hand (wrist and other distal part of hand)	0.0138
Saint Lucia	2010	Fracture of hand (wrist and other distal part of hand)	0.0138
Saint Lucia	2017	Fracture of hand (wrist and other distal part of hand)	0.0138
Saint Lucia	1990	Fracture of hip	0.1743
Saint Lucia	1995	Fracture of hip	0.1551
Saint Lucia	2000	Fracture of hip	0.1389
Saint Lucia	2005	Fracture of hip	0.1284
Saint Lucia	2010	Fracture of hip	0.1181
Saint Lucia	2017	Fracture of hip	0.108
Saint Lucia	1990	Amputation of upper limbs, bilateral	0.208
Saint Lucia	1995	Amputation of upper limbs, bilateral	0.1882
Saint Lucia	2000	Amputation of upper limbs, bilateral	0.1715
Saint Lucia	2005	Amputation of upper limbs, bilateral	0.1607
Saint Lucia	2010	Amputation of upper limbs, bilateral	0.15
Saint Lucia	2017	Amputation of upper limbs, bilateral	0.1396
Saint Lucia	1990	Fracture of patella, tibia or fibula, or ankle	0.0554
Saint Lucia	1995	Fracture of patella, tibia or fibula, or ankle	0.0554
Saint Lucia	2000	Fracture of patella, tibia or fibula, or ankle	0.0554
Saint Lucia	2005	Fracture of patella, tibia or fibula, or ankle	0.0554
Saint Lucia	2010	Fracture of patella, tibia or fibula, or ankle	0.0554
Saint Lucia	2017	Fracture of patella, tibia or fibula, or ankle	0.0554
Saint Lucia	1990	Fracture of pelvis	0.1823
Saint Lucia	1995	Fracture of pelvis	0.1823
Saint Lucia	2000	Fracture of pelvis	0.1823
Saint Lucia	2005	Fracture of pelvis	0.1823
Saint Lucia	2010	Fracture of pelvis	0.1823
Saint Lucia	2017	Fracture of pelvis	0.1823
Saint Lucia	1990	Fracture of radius and/or ulna	0.0435
Saint Lucia	1995	Fracture of radius and/or ulna	0.0435
Saint Lucia	2000	Fracture of radius and/or ulna	0.0435
Saint Lucia	2005	Fracture of radius and/or ulna	0.0435
Saint Lucia	2010	Fracture of radius and/or ulna	0.0435
Saint Lucia	2017	Fracture of radius and/or ulna	0.0435
Saint Lucia	1990	Fracture of skull	0.0714
Saint Lucia	1995	Fracture of skull	0.0714
Saint Lucia	2000	Fracture of skull	0.0714
Saint Lucia	2005	Fracture of skull	0.0714
Saint Lucia	2010	Fracture of skull	0.0714
Saint Lucia	2017	Fracture of skull	0.0714
Saint Lucia	1990	Fracture of sternum and/or fracture of one or more ribs	0.1027
Saint Lucia	1995	Fracture of sternum and/or fracture of one or more ribs	0.1027
Saint Lucia	2000	Fracture of sternum and/or fracture of one or more ribs	0.1027
Saint Lucia	2005	Fracture of sternum and/or fracture of one or more ribs	0.1027
Saint Lucia	2010	Fracture of sternum and/or fracture of one or more ribs	0.1027
Saint Lucia	2017	Fracture of sternum and/or fracture of one or more ribs	0.1027
Saint Lucia	1990	Fracture of vertebral column	0.1106
Saint Lucia	1995	Fracture of vertebral column	0.1106
Saint Lucia	2000	Fracture of vertebral column	0.1106
Saint Lucia	2005	Fracture of vertebral column	0.1106
Saint Lucia	2010	Fracture of vertebral column	0.1106
Saint Lucia	2017	Fracture of vertebral column	0.1106
Saint Lucia	1990	Fracture of femur, other than femoral neck	0.042
Saint Lucia	1995	Fracture of femur, other than femoral neck	0.042
Saint Lucia	2000	Fracture of femur, other than femoral neck	0.042
Saint Lucia	2005	Fracture of femur, other than femoral neck	0.042
Saint Lucia	2010	Fracture of femur, other than femoral neck	0.042
Saint Lucia	2017	Fracture of femur, other than femoral neck	0.042
Saint Lucia	1990	Minor TBI	0.1319
Saint Lucia	1995	Minor TBI	0.1319
Saint Lucia	2000	Minor TBI	0.1319
Saint Lucia	2005	Minor TBI	0.1319
Saint Lucia	2010	Minor TBI	0.1319
Saint Lucia	2017	Minor TBI	0.1319
Saint Lucia	1990	Moderate TBI	0.1637
Saint Lucia	1995	Moderate TBI	0.1637
Saint Lucia	2000	Moderate TBI	0.1637
Saint Lucia	2005	Moderate TBI	0.1637
Saint Lucia	2010	Moderate TBI	0.1637
Saint Lucia	2017	Moderate TBI	0.1637
Saint Lucia	1990	Amputation of fingers (excluding thumb)	0.0047
Saint Lucia	1995	Amputation of fingers (excluding thumb)	0.0047
Saint Lucia	2000	Amputation of fingers (excluding thumb)	0.0047
Saint Lucia	2005	Amputation of fingers (excluding thumb)	0.0047
Saint Lucia	2010	Amputation of fingers (excluding thumb)	0.0047
Saint Lucia	2017	Amputation of fingers (excluding thumb)	0.0047
Saint Lucia	1990	Spinal cord lesion at neck level	0.4521
Saint Lucia	1995	Spinal cord lesion at neck level	0.4455
Saint Lucia	2000	Spinal cord lesion at neck level	0.4399
Saint Lucia	2005	Spinal cord lesion at neck level	0.4363
Saint Lucia	2010	Spinal cord lesion at neck level	0.4327
Saint Lucia	2017	Spinal cord lesion at neck level	0.4292
Saint Lucia	1990	Spinal cord lesion below neck level	0.2628
Saint Lucia	1995	Spinal cord lesion below neck level	0.2487
Saint Lucia	2000	Spinal cord lesion below neck level	0.237
Saint Lucia	2005	Spinal cord lesion below neck level	0.2293
Saint Lucia	2010	Spinal cord lesion below neck level	0.2217
Saint Lucia	2017	Spinal cord lesion below neck level	0.2144
Saint Lucia	1990	Drowning and nonfatal submersion	0.2471
Saint Lucia	1995	Drowning and nonfatal submersion	0.2471
Saint Lucia	2000	Drowning and nonfatal submersion	0.2471
Saint Lucia	2005	Drowning and nonfatal submersion	0.2471
Saint Lucia	2010	Drowning and nonfatal submersion	0.2471
Saint Lucia	2017	Drowning and nonfatal submersion	0.2471
Saint Lucia	1990	Asphyxiation	0.2471
Saint Lucia	1995	Asphyxiation	0.2471
Saint Lucia	2000	Asphyxiation	0.2471
Saint Lucia	2005	Asphyxiation	0.2471
Saint Lucia	2010	Asphyxiation	0.2471
Saint Lucia	2017	Asphyxiation	0.2471
Saint Lucia	1990	Crush injury	0.1325
Saint Lucia	1995	Crush injury	0.1325
Saint Lucia	2000	Crush injury	0.1325
Saint Lucia	2005	Crush injury	0.1325
Saint Lucia	2010	Crush injury	0.1325
Saint Lucia	2017	Crush injury	0.1325
Saint Lucia	1990	Nerve injury	0.1129
Saint Lucia	1995	Nerve injury	0.1129
Saint Lucia	2000	Nerve injury	0.1129
Saint Lucia	2005	Nerve injury	0.1129
Saint Lucia	2010	Nerve injury	0.1129
Saint Lucia	2017	Nerve injury	0.1129
Saint Lucia	1990	Injury to eyes	0.0165
Saint Lucia	1995	Injury to eyes	0.0165
Saint Lucia	2000	Injury to eyes	0.0165
Saint Lucia	2005	Injury to eyes	0.0165
Saint Lucia	2010	Injury to eyes	0.0165
Saint Lucia	2017	Injury to eyes	0.0165
Saint Lucia	1990	Amputation of lower limb, unilateral	0.0844
Saint Lucia	1995	Amputation of lower limb, unilateral	0.0769
Saint Lucia	2000	Amputation of lower limb, unilateral	0.0706
Saint Lucia	2005	Amputation of lower limb, unilateral	0.0665
Saint Lucia	2010	Amputation of lower limb, unilateral	0.0625
Saint Lucia	2017	Amputation of lower limb, unilateral	0.0586
Saint Lucia	1990	Open wound(s)	0.011
Saint Lucia	1995	Open wound(s)	0.011
Saint Lucia	2000	Open wound(s)	0.011
Saint Lucia	2005	Open wound(s)	0.011
Saint Lucia	2010	Open wound(s)	0.011
Saint Lucia	2017	Open wound(s)	0.011
Saint Lucia	1990	Poisoning requiring urgent care	0.1628
Saint Lucia	1995	Poisoning requiring urgent care	0.1628
Saint Lucia	2000	Poisoning requiring urgent care	0.1628
Saint Lucia	2005	Poisoning requiring urgent care	0.1628
Saint Lucia	2010	Poisoning requiring urgent care	0.1628
Saint Lucia	2017	Poisoning requiring urgent care	0.1628
Saint Lucia	1990	Severe chest injury	0.0474
Saint Lucia	1995	Severe chest injury	0.0474
Saint Lucia	2000	Severe chest injury	0.0474
Saint Lucia	2005	Severe chest injury	0.0474
Saint Lucia	2010	Severe chest injury	0.0474
Saint Lucia	2017	Severe chest injury	0.0474
Saint Lucia	1990	Internal hemorrhage in abdomen and pelvis	0.1143
Saint Lucia	1995	Internal hemorrhage in abdomen and pelvis	0.1143
Saint Lucia	2000	Internal hemorrhage in abdomen and pelvis	0.1143
Saint Lucia	2005	Internal hemorrhage in abdomen and pelvis	0.1143
Saint Lucia	2010	Internal hemorrhage in abdomen and pelvis	0.1143
Saint Lucia	2017	Internal hemorrhage in abdomen and pelvis	0.1143
Saint Lucia	1990	Contusion in any part of the body	0.0075
Saint Lucia	1995	Contusion in any part of the body	0.0075
Saint Lucia	2000	Contusion in any part of the body	0.0075
Saint Lucia	2005	Contusion in any part of the body	0.0075
Saint Lucia	2010	Contusion in any part of the body	0.0075
Saint Lucia	2017	Contusion in any part of the body	0.0075
Saint Lucia	1990	Effect of different environmental factors	0.049
Saint Lucia	1995	Effect of different environmental factors	0.049
Saint Lucia	2000	Effect of different environmental factors	0.049
Saint Lucia	2005	Effect of different environmental factors	0.049
Saint Lucia	2010	Effect of different environmental factors	0.049
Saint Lucia	2017	Effect of different environmental factors	0.049
Saint Lucia	1990	Complications following therapeutic procedures	0.0506
Saint Lucia	1995	Complications following therapeutic procedures	0.0506
Saint Lucia	2000	Complications following therapeutic procedures	0.0506
Saint Lucia	2005	Complications following therapeutic procedures	0.0506
Saint Lucia	2010	Complications following therapeutic procedures	0.0506
Saint Lucia	2017	Complications following therapeutic procedures	0.0506
Saint Lucia	1990	Multiple fractures, dislocations, crashes, wounds, sprains, and strains	0.1743

Saint Lucia	1995	Multiple fractures, dislocations, crashes, wounds, sprains, and strains	0.1551
Saint Lucia	2000	Multiple fractures, dislocations, crashes, wounds, sprains, and strains	0.1389
Saint Lucia	2005	Multiple fractures, dislocations, crashes, wounds, sprains, and strains	0.1284
Saint Lucia	2010	Multiple fractures, dislocations, crashes, wounds, sprains, and strains	0.1181
Saint Lucia	2017	Multiple fractures, dislocations, crashes, wounds, sprains, and strains	0.108
Saint Lucia	1990	Amputation of upper limb, unilateral	0.0657
Saint Lucia	1995	Amputation of upper limb, unilateral	0.0613
Saint Lucia	2000	Amputation of upper limb, unilateral	0.0576
Saint Lucia	2005	Amputation of upper limb, unilateral	0.0552
Saint Lucia	2010	Amputation of upper limb, unilateral	0.0528
Saint Lucia	2017	Amputation of upper limb, unilateral	0.0505
Saint Lucia	1990	Amputation of thumb	0.0114
Saint Lucia	1995	Amputation of thumb	0.0114
Saint Lucia	2000	Amputation of thumb	0.0114
Saint Lucia	2005	Amputation of thumb	0.0114
Saint Lucia	2010	Amputation of thumb	0.0114
Saint Lucia	2017	Amputation of thumb	0.0114
Saint Lucia	1990	Amputation of toe/toes	0.0059
Saint Lucia	1995	Amputation of toe/toes	0.0059
Saint Lucia	2000	Amputation of toe/toes	0.0059
Saint Lucia	2005	Amputation of toe/toes	0.0059
Saint Lucia	2010	Amputation of toe/toes	0.0059
Saint Lucia	2017	Amputation of toe/toes	0.0059
Saint Lucia	1990	Burns, <20% total burned surface area without lower airway burns	0.0163
Saint Lucia	1995	Burns, <20% total burned surface area without lower airway burns	0.0163
Saint Lucia	2000	Burns, <20% total burned surface area without lower airway burns	0.0163
Saint Lucia	2005	Burns, <20% total burned surface area without lower airway burns	0.0163
Saint Lucia	2010	Burns, <20% total burned surface area without lower airway burns	0.0163
Saint Lucia	2017	Burns, <20% total burned surface area without lower airway burns	0.0163
Saint Lucia	1990	Burns, >=20% total burned surface area or >= 10% burned surface area if head/neck or hands/wrist involved w/o lower airway burns	0.2432
Saint Lucia	1995	Burns, >=20% total burned surface area or >= 10% burned surface area if head/neck or hands/wrist involved w/o lower airway burns	0.2252
Saint Lucia	2000	Burns, >=20% total burned surface area or >= 10% burned surface area if head/neck or hands/wrist involved w/o lower airway burns	0.2102
Saint Lucia	2005	Burns, >=20% total burned surface area or >= 10% burned surface area if head/neck or hands/wrist involved w/o lower airway burns	0.2005
Saint Lucia	2010	Burns, >=20% total burned surface area or >= 10% burned surface area if head/neck or hands/wrist involved w/o lower airway burns	0.1908
Saint Lucia	2017	Burns, >=20% total burned surface area or >= 10% burned surface area if head/neck or hands/wrist involved w/o lower airway burns	0.1814
Saint Vincent and the Grenadines	1990	Amputation of lower limbs, bilateral	0.2091
Saint Vincent and the Grenadines	1995	Amputation of lower limbs, bilateral	0.2055
Saint Vincent and the Grenadines	2000	Amputation of lower limbs, bilateral	0.1974
Saint Vincent and the Grenadines	2005	Amputation of lower limbs, bilateral	0.188
Saint Vincent and the Grenadines	2010	Amputation of lower limbs, bilateral	0.1846
Saint Vincent and the Grenadines	2017	Amputation of lower limbs, bilateral	0.1813
Saint Vincent and the Grenadines	1990	Lower airway burns	0.2252
Saint Vincent and the Grenadines	1995	Lower airway burns	0.2252
Saint Vincent and the Grenadines	2000	Lower airway burns	0.2252
Saint Vincent and the Grenadines	2005	Lower airway burns	0.2252
Saint Vincent and the Grenadines	2010	Lower airway burns	0.2252
Saint Vincent and the Grenadines	2017	Lower airway burns	0.2252
Saint Vincent and the Grenadines	1990	Dislocation of hip	0.0159
Saint Vincent and the Grenadines	1995	Dislocation of hip	0.0159
Saint Vincent and the Grenadines	2000	Dislocation of hip	0.0159
Saint Vincent and the Grenadines	2005	Dislocation of hip	0.0159
Saint Vincent and the Grenadines	2010	Dislocation of hip	0.0159
Saint Vincent and the Grenadines	2017	Dislocation of hip	0.0159
Saint Vincent and the Grenadines	1990	Dislocation of knee	0.1134
Saint Vincent and the Grenadines	1995	Dislocation of knee	0.1134
Saint Vincent and the Grenadines	2000	Dislocation of knee	0.1134
Saint Vincent and the Grenadines	2005	Dislocation of knee	0.1134
Saint Vincent and the Grenadines	2010	Dislocation of knee	0.1134
Saint Vincent and the Grenadines	2017	Dislocation of knee	0.1134
Saint Vincent and the Grenadines	1990	Dislocation of shoulder	0.062
Saint Vincent and the Grenadines	1995	Dislocation of shoulder	0.062
Saint Vincent and the Grenadines	2000	Dislocation of shoulder	0.062
Saint Vincent and the Grenadines	2005	Dislocation of shoulder	0.062
Saint Vincent and the Grenadines	2010	Dislocation of shoulder	0.062
Saint Vincent and the Grenadines	2017	Dislocation of shoulder	0.062
Saint Vincent and the Grenadines	1990	Muscle and tendon injuries, including sprains and strains lesser dislocations	0.0075
Saint Vincent and the Grenadines	1995	Muscle and tendon injuries, including sprains and strains lesser dislocations	0.0075
Saint Vincent and the Grenadines	2000	Muscle and tendon injuries, including sprains and strains lesser dislocations	0.0075
Saint Vincent and the Grenadines	2005	Muscle and tendon injuries, including sprains and strains lesser dislocations	0.0075
Saint Vincent and the Grenadines	2010	Muscle and tendon injuries, including sprains and strains lesser dislocations	0.0075
Saint Vincent and the Grenadines	2017	Muscle and tendon injuries, including sprains and strains lesser dislocations	0.0075
Saint Vincent and the Grenadines	1990	Fracture of clavicle, scapula, or humerus	0.0349
Saint Vincent and the Grenadines	1995	Fracture of clavicle, scapula, or humerus	0.0349
Saint Vincent and the Grenadines	2000	Fracture of clavicle, scapula, or humerus	0.0349
Saint Vincent and the Grenadines	2005	Fracture of clavicle, scapula, or humerus	0.0349
Saint Vincent and the Grenadines	2010	Fracture of clavicle, scapula, or humerus	0.0349
Saint Vincent and the Grenadines	2017	Fracture of clavicle, scapula, or humerus	0.0349
Saint Vincent and the Grenadines	1990	Fracture of face bones	0.0669
Saint Vincent and the Grenadines	1995	Fracture of face bones	0.0669
Saint Vincent and the Grenadines	2000	Fracture of face bones	0.0669
Saint Vincent and the Grenadines	2005	Fracture of face bones	0.0669
Saint Vincent and the Grenadines	2010	Fracture of face bones	0.0669
Saint Vincent and the Grenadines	2017	Fracture of face bones	0.0669
Saint Vincent and the Grenadines	1990	Fracture of foot bones except ankle	0.0264
Saint Vincent and the Grenadines	1995	Fracture of foot bones except ankle	0.0264
Saint Vincent and the Grenadines	2000	Fracture of foot bones except ankle	0.0264
Saint Vincent and the Grenadines	2005	Fracture of foot bones except ankle	0.0264
Saint Vincent and the Grenadines	2010	Fracture of foot bones except ankle	0.0264
Saint Vincent and the Grenadines	2017	Fracture of foot bones except ankle	0.0264
Saint Vincent and the Grenadines	1990	Fracture of hand (wrist and other distal part of hand)	0.0138
Saint Vincent and the Grenadines	1995	Fracture of hand (wrist and other distal part of hand)	0.0138
Saint Vincent and the Grenadines	2000	Fracture of hand (wrist and other distal part of hand)	0.0138
Saint Vincent and the Grenadines	2005	Fracture of hand (wrist and other distal part of hand)	0.0138
Saint Vincent and the Grenadines	2010	Fracture of hand (wrist and other distal part of hand)	0.0138
Saint Vincent and the Grenadines	2017	Fracture of hand (wrist and other distal part of hand)	0.0138
Saint Vincent and the Grenadines	1990	Fracture of hip	0.1712
Saint Vincent and the Grenadines	1995	Fracture of hip	0.1664
Saint Vincent and the Grenadines	2000	Fracture of hip	0.1558
Saint Vincent and the Grenadines	2005	Fracture of hip	0.1434
Saint Vincent and the Grenadines	2010	Fracture of hip	0.1388
Saint Vincent and the Grenadines	2017	Fracture of hip	0.1345
Saint Vincent and the Grenadines	1990	Amputation of upper limbs, bilateral	0.2049
Saint Vincent and the Grenadines	1995	Amputation of upper limbs, bilateral	0.1999
Saint Vincent and the Grenadines	2000	Amputation of upper limbs, bilateral	0.1889
Saint Vincent and the Grenadines	2005	Amputation of upper limbs, bilateral	0.1761
Saint Vincent and the Grenadines	2010	Amputation of upper limbs, bilateral	0.1714
Saint Vincent and the Grenadines	2017	Amputation of upper limbs, bilateral	0.167
Saint Vincent and the Grenadines	1990	Fracture of patella, tibia or fibula, or ankle	0.0554
Saint Vincent and the Grenadines	1995	Fracture of patella, tibia or fibula, or ankle	0.0554
Saint Vincent and the Grenadines	2000	Fracture of patella, tibia or fibula, or ankle	0.0554
Saint Vincent and the Grenadines	2005	Fracture of patella, tibia or fibula, or ankle	0.0554
Saint Vincent and the Grenadines	2010	Fracture of patella, tibia or fibula, or ankle	0.0554
Saint Vincent and the Grenadines	2017	Fracture of patella, tibia or fibula, or ankle	0.0554
Saint Vincent and the Grenadines	1990	Fracture of pelvis	0.1823
Saint Vincent and the Grenadines	1995	Fracture of pelvis	0.1823
Saint Vincent and the Grenadines	2000	Fracture of pelvis	0.1823
Saint Vincent and the Grenadines	2005	Fracture of pelvis	0.1823
Saint Vincent and the Grenadines	2010	Fracture of pelvis	0.1823
Saint Vincent and the Grenadines	2017	Fracture of pelvis	0.1823
Saint Vincent and the Grenadines	1990	Fracture of radius and/or ulna	0.0435
Saint Vincent and the Grenadines	1995	Fracture of radius and/or ulna	0.0435
Saint Vincent and the Grenadines	2000	Fracture of radius and/or ulna	0.0435
Saint Vincent and the Grenadines	2005	Fracture of radius and/or ulna	0.0435
Saint Vincent and the Grenadines	2010	Fracture of radius and/or ulna	0.0435
Saint Vincent and the Grenadines	2017	Fracture of radius and/or ulna	0.0435
Saint Vincent and the Grenadines	1990	Fracture of skull	0.0714
Saint Vincent and the Grenadines	1995	Fracture of skull	0.0714
Saint Vincent and the Grenadines	2000	Fracture of skull	0.0714
Saint Vincent and the Grenadines	2005	Fracture of skull	0.0714
Saint Vincent and the Grenadines	2010	Fracture of skull	0.0714
Saint Vincent and the Grenadines	2017	Fracture of skull	0.0714
Saint Vincent and the Grenadines	1990	Fracture of sternum and/or fracture of one or more ribs	0.1027
Saint Vincent and the Grenadines	1995	Fracture of sternum and/or fracture of one or more ribs	0.1027
Saint Vincent and the Grenadines	2000	Fracture of sternum and/or fracture of one or more ribs	0.1027
Saint Vincent and the Grenadines	2005	Fracture of sternum and/or fracture of one or more ribs	0.1027
Saint Vincent and the Grenadines	2010	Fracture of sternum and/or fracture of one or more ribs	0.1027
Saint Vincent and the Grenadines	2017	Fracture of sternum and/or fracture of one or more ribs	0.1027
Saint Vincent and the Grenadines	1990	Fracture of vertebral column	0.1106
Saint Vincent and the Grenadines	1995	Fracture of vertebral column	0.1106
Saint Vincent and the Grenadines	2000	Fracture of vertebral column	0.1106
Saint Vincent and the Grenadines	2005	Fracture of vertebral column	0.1106
Saint Vincent and the Grenadines	2010	Fracture of vertebral column	0.1106
Saint Vincent and the Grenadines	2017	Fracture of vertebral column	0.1106
Saint Vincent and the Grenadines	1990	Fracture of femur, other than femoral neck	0.042
Saint Vincent and the Grenadines	1995	Fracture of femur, other than femoral neck	0.042
Saint Vincent and the Grenadines	2000	Fracture of femur, other than femoral neck	0.042
Saint Vincent and the Grenadines	2005	Fracture of femur, other than femoral neck	0.042
Saint Vincent and the Grenadines	2010	Fracture of femur, other than femoral neck	0.042
Saint Vincent and the Grenadines	2017	Fracture of femur, other than femoral neck	0.042
Saint Vincent and the Grenadines	1990	Minor TBI	0.1319
Saint Vincent and the Grenadines	1995	Minor TBI	0.1319
Saint Vincent and the Grenadines	2000	Minor TBI	0.1319
Saint Vincent and the Grenadines	2005	Minor TBI	0.1319
Saint Vincent and the Grenadines	2010	Minor TBI	0.1319
Saint Vincent and the Grenadines	2017	Minor TBI	0.1319
Saint Vincent and the Grenadines	1990	Moderate TBI	0.1637
Saint Vincent and the Grenadines	1995	Moderate TBI	0.1637
Saint Vincent and the Grenadines	2000	Moderate TBI	0.1637
Saint Vincent and the Grenadines	2005	Moderate TBI	0.1637
Saint Vincent and the Grenadines	2010	Moderate TBI	0.1637
Saint Vincent and the Grenadines	2017	Moderate TBI	0.1637
Saint Vincent and the Grenadines	1990	Amputation of fingers (excluding thumb)	0.0047
Saint Vincent and the Grenadines	1995	Amputation of fingers (excluding thumb)	0.0047
Saint Vincent and the Grenadines	2000	Amputation of fingers (excluding thumb)	0.0047
Saint Vincent and the Grenadines	2005	Amputation of fingers (excluding thumb)	0.0047
Saint Vincent and the Grenadines	2010	Amputation of fingers (excluding thumb)	0.0047
Saint Vincent and the Grenadines	2017	Amputation of fingers (excluding thumb)	0.0047
Saint Vincent and the Grenadines	1990	Spinal cord lesion at neck level	0.4511
Saint Vincent and the Grenadines	1995	Spinal cord lesion at neck level	0.4494
Saint Vincent and the Grenadines	2000	Spinal cord lesion at neck level	0.4457
Saint Vincent and the Grenadines	2005	Spinal cord lesion at neck level	0.4415
Saint Vincent and the Grenadines	2010	Spinal cord lesion at neck level	0.4399
Saint Vincent and the Grenadines	2017	Spinal cord lesion at neck level	0.4384
Saint Vincent and the Grenadines	1990	Spinal cord lesion below neck level	0.2606
Saint Vincent and the Grenadines	1995	Spinal cord lesion below neck level	0.257
Saint Vincent and the Grenadines	2000	Spinal cord lesion below neck level	0.2493
Saint Vincent and the Grenadines	2005	Spinal cord lesion below neck level	0.2402
Saint Vincent and the Grenadines	2010	Spinal cord lesion below neck level	0.2369
Saint Vincent and the Grenadines	2017	Spinal cord lesion below neck level	0.2337
Saint Vincent and the Grenadines	1990	Drowning and nonfatal submersion	0.2471
Saint Vincent and the Grenadines	1995	Drowning and nonfatal submersion	0.2471
Saint Vincent and the Grenadines	2000	Drowning and nonfatal submersion	0.2471
Saint Vincent and the Grenadines	2005	Drowning and nonfatal submersion	0.2471
Saint Vincent and the Grenadines	2010	Drowning and nonfatal submersion	0.2471
Saint Vincent and the Grenadines	2017	Drowning and nonfatal submersion	0.2471
Saint Vincent and the Grenadines	1990	Asphyxiation	0.2471
Saint Vincent and the Grenadines	1995	Asphyxiation	0.2471
Saint Vincent and the Grenadines	2000	Asphyxiation	0.2471
Saint Vincent and the Grenadines	2005	Asphyxiation	0.2471
Saint Vincent and the Grenadines	2010	Asphyxiation	0.2471
Saint Vincent and the Grenadines	2017	Asphyxiation	0.2471
Saint Vincent and the Grenadines	1990	Crush injury	0.1325
Saint Vincent and the Grenadines	1995	Crush injury	0.1325
Saint Vincent and the Grenadines	2000	Crush injury	0.1325
Saint Vincent and the Grenadines	2005	Crush injury	0.1325
Saint Vincent and the Grenadines	2010	Crush injury	0.1325
Saint Vincent and the Grenadines	2017	Crush injury	0.1325
Saint Vincent and the Grenadines	1990	Nerve injury	0.1129
Saint Vincent and the Grenadines	1995	Nerve injury	0.1129
Saint Vincent and the Grenadines	2000	Nerve injury	0.1129
Saint Vincent and the Grenadines	2005	Nerve injury	0.1129
Saint Vincent and the Grenadines	2010	Nerve injury	0.1129
Saint Vincent and the Grenadines	2017	Nerve injury	0.1129
Saint Vincent and the Grenadines	1990	Injury to eyes	0.0165
Saint Vincent and the Grenadines	1995	Injury to eyes	0.0165
Saint Vincent and the Grenadines	2000	Injury to eyes	0.0165
Saint Vincent and the Grenadines	2005	Injury to eyes	0.0165
Saint Vincent and the Grenadines	2010	Injury to eyes	0.0165
Saint Vincent and the Grenadines	2017	Injury to eyes	0.0165
Saint Vincent and the Grenadines	1990	Amputation of lower limb, unilateral	0.0832
Saint Vincent and the Grenadines	1995	Amputation of lower limb, unilateral	0.0813
Saint Vincent and the Grenadines	2000	Amputation of lower limb, unilateral	0.0772
Saint Vincent and the Grenadines	2005	Amputation of lower limb, unilateral	0.0724
Saint Vincent and the Grenadines	2010	Amputation of lower limb, unilateral	0.0706
Saint Vincent and the Grenadines	2017	Amputation of lower limb, unilateral	0.0689
Saint Vincent and the Grenadines	1990	Open wound(s)	0.011
Saint Vincent and the Grenadines	1995	Open wound(s)	0.011
Saint Vincent and the Grenadines	2000	Open wound(s)	0.011
Saint Vincent and the Grenadines	2005	Open wound(s)	0.011
Saint Vincent and the Grenadines	2010	Open wound(s)	0.011
Saint Vincent and the Grenadines	2017	Open wound(s)	0.011
Saint Vincent and the Grenadines	1990	Poisoning requiring urgent care	0.1628
Saint Vincent and the Grenadines	1995	Poisoning requiring urgent care	0.1628
Saint Vincent and the Grenadines	2000	Poisoning requiring urgent care	0.1628
Saint Vincent and the Grenadines	2005	Poisoning requiring urgent care	0.1628
Saint Vincent and the Grenadines	2010	Poisoning requiring urgent care	0.1628
Saint Vincent and the Grenadines	2017	Poisoning requiring urgent care	0.1628
Saint Vincent and the Grenadines	1990	Severe chest injury	0.0474
Saint Vincent and the Grenadines	1995	Severe chest injury	0.0474
Saint Vincent and the Grenadines	2000	Severe chest injury	0.0474
Saint Vincent and the Grenadines	2005	Severe chest injury	0.0474
Saint Vincent and the Grenadines	2010	Severe chest injury	0.0474
Saint Vincent and the Grenadines	2017	Severe chest injury	0.0474
Saint Vincent and the Grenadines	1990	Internal hemorrhage in abdomen and pelvis	0.1143
Saint Vincent and the Grenadines	1995	Internal hemorrhage in abdomen and pelvis	0.1143
Saint Vincent and the Grenadines	2000	Internal hemorrhage in abdomen and pelvis	0.1143
Saint Vincent and the Grenadines	2005	Internal hemorrhage in abdomen and pelvis	0.1143
Saint Vincent and the Grenadines	2010	Internal hemorrhage in abdomen and pelvis	0.1143
Saint Vincent and the Grenadines	2017	Internal hemorrhage in abdomen and pelvis	0.1143
Saint Vincent and the Grenadines	1990	Contusion in any part of the body	0.0075
Saint Vincent and the Grenadines	1995	Contusion in any part of the body	0.0075
Saint Vincent and the Grenadines	2000	Contusion in any part of the body	0.0075
Saint Vincent and the Grenadines	2005	Contusion in any part of the body	0.0075
Saint Vincent and the Grenadines	2010	Contusion in any part of the body	0.0075
Saint Vincent and the Grenadines	2017	Contusion in any part of the body	0.0075
Saint Vincent and the Grenadines	1990	Effect of different environmental factors	0.049
Saint Vincent and the Grenadines	1995	Effect of different environmental factors	0.049
Saint Vincent and the Grenadines	2000	Effect of different environmental factors	0.049
Saint Vincent and the Grenadines	2005	Effect of different environmental factors	0.049
Saint Vincent and the Grenadines	2010	Effect of different environmental factors	0.049
Saint Vincent and the Grenadines	2017	Effect of different environmental factors	0.049
Saint Vincent and the Grenadines	1990	Complications following therapeutic procedures	0.0506
Saint Vincent and the Grenadines	1995	Complications following therapeutic procedures	0.0506
Saint Vincent and the Grenadines	2000	Complications following therapeutic procedures	0.0506
Saint Vincent and the Grenadines	2005	Complications following therapeutic procedures	0.0506
Saint Vincent and the Grenadines	2010	Complications following therapeutic procedures	0.0506
Saint Vincent and the Grenadines	2017	Complications following therapeutic procedures	0.0506
Saint Vincent and the Grenadines	1990	Multiple fractures, dislocations, crashes, wounds, sprains, and strains	0.1712
Saint Vincent and the Grenadines	1995	Multiple fractures, dislocations, crashes, wounds, sprains, and strains	0.1664
Saint Vincent and the Grenadines	2000	Multiple fractures, dislocations, crashes, wounds, sprains, and strains	0.1558
Saint Vincent and the Grenadines	2005	Multiple fractures, dislocations, crashes, wounds, sprains, and strains	0.1434
Saint Vincent and the Grenadines	2010	Multiple fractures, dislocations, crashes, wounds, sprains, and strains	0.1388
Saint Vincent and the Grenadines	2017	Multiple fractures, dislocations, crashes, wounds, sprains, and strains	0.1345
Saint Vincent and the Grenadines	1990	Amputation of upper limb, unilateral	0.065
Saint Vincent and the Grenadines	1995	Amputation of upper limb, unilateral	0.0639
Saint Vincent and the Grenadines	2000	Amputation of upper limb, unilateral	0.0615
Saint Vincent and the Grenadines	2005	Amputation of upper limb, unilateral	0.0586
Saint Vincent and the Grenadines	2010	Amputation of upper limb, unilateral	0.0576
Saint Vincent and the Grenadines	2017	Amputation of upper limb, unilateral	0.0566
Saint Vincent and the Grenadines	1990	Amputation of thumb	0.0114
Saint Vincent and the Grenadines	1995	Amputation of thumb	0.0114
Saint Vincent and the Grenadines	2000	Amputation of thumb	0.0114
Saint Vincent and the Grenadines	2005	Amputation of thumb	0.0114
Saint Vincent and the Grenadines	2010	Amputation of thumb	0.0114
Saint Vincent and the Grenadines	2017	Amputation of thumb	0.0114
Saint Vincent and the Grenadines	1990	Amputation of toe/toes	0.0059
Saint Vincent and the Grenadines	1995	Amputation of toe/toes	0.0059
Saint Vincent and the Grenadines	2000	Amputation of toe/toes	0.0059
Saint Vincent and the Grenadines	2005	Amputation of toe/toes	0.0059
Saint Vincent and the Grenadines	2010	Amputation of toe/toes	0.0059
Saint Vincent and the Grenadines	2017	Amputation of toe/toes	0.0059
Saint Vincent and the Grenadines	1990	Burns, <20% total burned surface area without lower airway burns	0.0163
Saint Vincent and the Grenadines	1995	Burns, <20% total burned surface area without lower airway burns	0.0163
Saint Vincent and the Grenadines	2000	Burns, <20% total burned surface area without lower airway burns	0.0163
Saint Vincent and the Grenadines	2005	Burns, <20% total burned surface area without lower airway burns	0.0163
Saint Vincent and the Grenadines	2010	Burns, <20% total burned surface area without lower airway burns	0.0163
Saint Vincent and the Grenadines	2017	Burns, <20% total burned surface area without lower airway burns	0.0163
Saint Vincent and the Grenadines	1990	Burns, >=20% total burned surface area or >= 10% burned surface area if head/neck or hands/wrist involved w/o lower airway burns	0.2403
Saint Vincent and the Grenadines	1995	Burns, >=20% total burned surface area or >= 10% burned surface area if head/neck or hands/wrist involved w/o lower airway burns	0.2358
Saint Vincent and the Grenadines	2000	Burns, >=20% total burned surface area or >= 10% burned surface area if head/neck or hands/wrist involved w/o lower airway burns	0.2259
Saint Vincent and the Grenadines	2005	Burns, >=20% total burned surface area or >= 10% burned surface area if head/neck or hands/wrist involved w/o lower airway burns	0.2144
Saint Vincent and the Grenadines	2010	Burns, >=20% total burned surface area or >= 10% burned surface area if head/neck or hands/wrist involved w/o lower airway burns	0.2101
Saint Vincent and the Grenadines	2017	Burns, >=20% total burned surface area or >= 10% burned surface area if head/neck or hands/wrist involved w/o lower airway burns	0.2061
Suriname	1990	Amputation of lower limbs, bilateral	0.2347
Suriname	1995	Amputation of lower limbs, bilateral	0.2272
Suriname	2000	Amputation of lower limbs, bilateral	0.2226
Suriname	2005	Amputation of lower limbs, bilateral	0.2161
Suriname	2010	Amputation of lower limbs, bilateral	0.2053
Suriname	2017	Amputation of lower limbs, bilateral	0.1919
Suriname	1990	Lower airway burns	0.2252
Suriname	1995	Lower airway burns	0.2252
Suriname	2000	Lower airway burns	0.2252
Suriname	2005	Lower airway burns	0.2252
Suriname	2010	Lower airway burns	0.2252
Suriname	2017	Lower airway burns	0.2252
Suriname	1990	Dislocation of hip	0.0159
Suriname	1995	Dislocation of hip	0.0159
Suriname	2000	Dislocation of hip	0.0159
Suriname	2005	Dislocation of hip	0.0159
Suriname	2010	Dislocation of hip	0.0159
Suriname	2017	Dislocation of hip	0.0159
Suriname	1990	Dislocation of knee	0.1134
Suriname	1995	Dislocation of knee	0.1134
Suriname	2000	Dislocation of knee	0.1134
Suriname	2005	Dislocation of knee	0.1134
Suriname	2010	Dislocation of knee	0.1134
Suriname	2017	Dislocation of knee	0.1134
Suriname	1990	Dislocation of shoulder	0.062
Suriname	1995	Dislocation of shoulder	0.062
Suriname	2000	Dislocation of shoulder	0.062
Suriname	2005	Dislocation of shoulder	0.062
Suriname	2010	Dislocation of shoulder	0.062
Suriname	2017	Dislocation of shoulder	0.062
Suriname	1990	Muscle and tendon injuries, including sprains and strains lesser dislocations	0.0075
Suriname	1995	Muscle and tendon injuries, including sprains and strains lesser dislocations	0.0075
Suriname	2000	Muscle and tendon injuries, including sprains and strains lesser dislocations	0.0075
Suriname	2005	Muscle and tendon injuries, including sprains and strains lesser dislocations	0.0075
Suriname	2010	Muscle and tendon injuries, including sprains and strains lesser dislocations	0.0075
Suriname	2017	Muscle and tendon injuries, including sprains and strains lesser dislocations	0.0075
Suriname	1990	Fracture of clavicle, scapula, or humerus	0.0349
Suriname	1995	Fracture of clavicle, scapula, or humerus	0.0349
Suriname	2000	Fracture of clavicle, scapula, or humerus	0.0349
Suriname	2005	Fracture of clavicle, scapula, or humerus	0.0349
Suriname	2010	Fracture of clavicle, scapula, or humerus	0.0349
Suriname	2017	Fracture of clavicle, scapula, or humerus	0.0349
Suriname	1990	Fracture of face bones	0.0669
Suriname	1995	Fracture of face bones	0.0669
Suriname	2000	Fracture of face bones	0.0669
Suriname	2005	Fracture of face bones	0.0669
Suriname	2010	Fracture of face bones	0.0669
Suriname	2017	Fracture of face bones	0.0669
Suriname	1990	Fracture of foot bones except ankle	0.0264
Suriname	1995	Fracture of foot bones except ankle	0.0264
Suriname	2000	Fracture of foot bones except ankle	0.0264
Suriname	2005	Fracture of foot bones except ankle	0.0264
Suriname	2010	Fracture of foot bones except ankle	0.0264
Suriname	2017	Fracture of foot bones except ankle	0.0264
Suriname	1990	Fracture of hand (wrist and other distal part of hand)	0.0138
Suriname	1995	Fracture of hand (wrist and other distal part of hand)	0.0138
Suriname	2000	Fracture of hand (wrist and other distal part of hand)	0.0138
Suriname	2005	Fracture of hand (wrist and other distal part of hand)	0.0138
Suriname	2010	Fracture of hand (wrist and other distal part of hand)	0.0138
Suriname	2017	Fracture of hand (wrist and other distal part of hand)	0.0138
Suriname	1990	Fracture of hip	0.205
Suriname	1995	Fracture of hip	0.1952
Suriname	2000	Fracture of hip	0.1891
Suriname	2005	Fracture of hip	0.1805
Suriname	2010	Fracture of hip	0.1663
Suriname	2017	Fracture of hip	0.1485
Suriname	1990	Amputation of upper limbs, bilateral	0.2398
Suriname	1995	Amputation of upper limbs, bilateral	0.2297
Suriname	2000	Amputation of upper limbs, bilateral	0.2234
Suriname	2005	Amputation of upper limbs, bilateral	0.2145
Suriname	2010	Amputation of upper limbs, bilateral	0.1998
Suriname	2017	Amputation of upper limbs, bilateral	0.1814
Suriname	1990	Fracture of patella, tibia or fibula, or ankle	0.0554
Suriname	1995	Fracture of patella, tibia or fibula, or ankle	0.0554
Suriname	2000	Fracture of patella, tibia or fibula, or ankle	0.0554
Suriname	2005	Fracture of patella, tibia or fibula, or ankle	0.0554
Suriname	2010	Fracture of patella, tibia or fibula, or ankle	0.0554
Suriname	2017	Fracture of patella, tibia or fibula, or ankle	0.0554
Suriname	1990	Fracture of pelvis	0.1823
Suriname	1995	Fracture of pelvis	0.1823
Suriname	2000	Fracture of pelvis	0.1823
Suriname	2005	Fracture of pelvis	0.1823
Suriname	2010	Fracture of pelvis	0.1823
Suriname	2017	Fracture of pelvis	0.1823
Suriname	1990	Fracture of radius and/or ulna	0.0435
Suriname	1995	Fracture of radius and/or ulna	0.0435
Suriname	2000	Fracture of radius and/or ulna	0.0435
Suriname	2005	Fracture of radius and/or ulna	0.0435
Suriname	2010	Fracture of radius and/or ulna	0.0435
Suriname	2017	Fracture of radius and/or ulna	0.0435
Suriname	1990	Fracture of skull	0.0714
Suriname	1995	Fracture of skull	0.0714
Suriname	2000	Fracture of skull	0.0714
Suriname	2005	Fracture of skull	0.0714
Suriname	2010	Fracture of skull	0.0714
Suriname	2017	Fracture of skull	0.0714
Suriname	1990	Fracture of sternum and/or fracture of one or more ribs	0.1027
Suriname	1995	Fracture of sternum and/or fracture of one or more ribs	0.1027
Suriname	2000	Fracture of sternum and/or fracture of one or more ribs	0.1027
Suriname	2005	Fracture of sternum and/or fracture of one or more ribs	0.1027
Suriname	2010	Fracture of sternum and/or fracture of one or more ribs	0.1027
Suriname	2017	Fracture of sternum and/or fracture of one or more ribs	0.1027
Suriname	1990	Fracture of vertebral column	0.1106
Suriname	1995	Fracture of vertebral column	0.1106
Suriname	2000	Fracture of vertebral column	0.1106
Suriname	2005	Fracture of vertebral column	0.1106
Suriname	2010	Fracture of vertebral column	0.1106
Suriname	2017	Fracture of vertebral column	0.1106
Suriname	1990	Fracture of femur, other than femoral neck	0.042
Suriname	1995	Fracture of femur, other than femoral neck	0.042
Suriname	2000	Fracture of femur, other than femoral neck	0.042
Suriname	2005	Fracture of femur, other than femoral neck	0.042
Suriname	2010	Fracture of femur, other than femoral neck	0.042
Suriname	2017	Fracture of femur, other than femoral neck	0.042
Suriname	1990	Minor TBI	0.1319
Suriname	1995	Minor TBI	0.1319
Suriname	2000	Minor TBI	0.1319
Suriname	2005	Minor TBI	0.1319
Suriname	2010	Minor TBI	0.1319
Suriname	2017	Minor TBI	0.1319
Suriname	1990	Moderate TBI	0.1637
Suriname	1995	Moderate TBI	0.1637
Suriname	2000	Moderate TBI	0.1637
Suriname	2005	Moderate TBI	0.1637
Suriname	2010	Moderate TBI	0.1637
Suriname	2017	Moderate TBI	0.1637
Suriname	1990	Amputation of fingers (excluding thumb)	0.0047
Suriname	1995	Amputation of fingers (excluding thumb)	0.0047
Suriname	2000	Amputation of fingers (excluding thumb)	0.0047
Suriname	2005	Amputation of fingers (excluding thumb)	0.0047
Suriname	2010	Amputation of fingers (excluding thumb)	0.0047
Suriname	2017	Amputation of fingers (excluding thumb)	0.0047
Suriname	1990	Spinal cord lesion at neck level	0.4628
Suriname	1995	Spinal cord lesion at neck level	0.4584
Suriname	2000	Spinal cord lesion at neck level	0.4573
Suriname	2005	Spinal cord lesion at neck level	0.4543
Suriname	2010	Spinal cord lesion at neck level	0.4484
Suriname	2017	Spinal cord lesion at neck level	0.4432

Suriname	1990	Spinal cord lesion below neck level	0.2852
Suriname	1995	Spinal cord lesion below neck level	0.278
Suriname	2000	Spinal cord lesion below neck level	0.2736
Suriname	2005	Spinal cord lesion below neck level	0.2673
Suriname	2010	Spinal cord lesion below neck level	0.2569
Suriname	2017	Spinal cord lesion below neck level	0.2439
Suriname	1990	Drowning and nonfatal submersion	0.2471
Suriname	1995	Drowning and nonfatal submersion	0.2471
Suriname	2000	Drowning and nonfatal submersion	0.2471
Suriname	2005	Drowning and nonfatal submersion	0.2471
Suriname	2010	Drowning and nonfatal submersion	0.2471
Suriname	2017	Drowning and nonfatal submersion	0.2471
Suriname	1990	Asphyxiation	0.2471
Suriname	1995	Asphyxiation	0.2471
Suriname	2000	Asphyxiation	0.2471
Suriname	2005	Asphyxiation	0.2471
Suriname	2010	Asphyxiation	0.2471
Suriname	2017	Asphyxiation	0.2471
Suriname	1990	Crush injury	0.1325
Suriname	1995	Crush injury	0.1325
Suriname	2000	Crush injury	0.1325
Suriname	2005	Crush injury	0.1325
Suriname	2010	Crush injury	0.1325
Suriname	2017	Crush injury	0.1325
Suriname	1990	Nerve injury	0.1129
Suriname	1995	Nerve injury	0.1129
Suriname	2000	Nerve injury	0.1129
Suriname	2005	Nerve injury	0.1129
Suriname	2010	Nerve injury	0.1129
Suriname	2017	Nerve injury	0.1129
Suriname	1990	Injury to eyes	0.0165
Suriname	1995	Injury to eyes	0.0165
Suriname	2000	Injury to eyes	0.0165
Suriname	2005	Injury to eyes	0.0165
Suriname	2010	Injury to eyes	0.0165
Suriname	2017	Injury to eyes	0.0165
Suriname	1990	Amputation of lower limb, unilateral	0.0964
Suriname	1995	Amputation of lower limb, unilateral	0.0926
Suriname	2000	Amputation of lower limb, unilateral	0.0902
Suriname	2005	Amputation of lower limb, unilateral	0.0868
Suriname	2010	Amputation of lower limb, unilateral	0.0813
Suriname	2017	Amputation of lower limb, unilateral	0.0743
Suriname	1990	Open wound(s)	0.011
Suriname	1995	Open wound(s)	0.011
Suriname	2000	Open wound(s)	0.011
Suriname	2005	Open wound(s)	0.011
Suriname	2010	Open wound(s)	0.011
Suriname	2017	Open wound(s)	0.011
Suriname	1990	Poisoning requiring urgent care	0.1628
Suriname	1995	Poisoning requiring urgent care	0.1628
Suriname	2000	Poisoning requiring urgent care	0.1628
Suriname	2005	Poisoning requiring urgent care	0.1628
Suriname	2010	Poisoning requiring urgent care	0.1628
Suriname	2017	Poisoning requiring urgent care	0.1628
Suriname	1990	Severe chest injury	0.0474
Suriname	1995	Severe chest injury	0.0474
Suriname	2000	Severe chest injury	0.0474
Suriname	2005	Severe chest injury	0.0474
Suriname	2010	Severe chest injury	0.0474
Suriname	2017	Severe chest injury	0.0474
Suriname	1990	Internal hemorrhage in abdomen and pelvis	0.1143
Suriname	1995	Internal hemorrhage in abdomen and pelvis	0.1143
Suriname	2000	Internal hemorrhage in abdomen and pelvis	0.1143
Suriname	2005	Internal hemorrhage in abdomen and pelvis	0.1143
Suriname	2010	Internal hemorrhage in abdomen and pelvis	0.1143
Suriname	2017	Internal hemorrhage in abdomen and pelvis	0.1143
Suriname	1990	Contusion in any part of the body	0.0075
Suriname	1995	Contusion in any part of the body	0.0075
Suriname	2000	Contusion in any part of the body	0.0075
Suriname	2005	Contusion in any part of the body	0.0075
Suriname	2010	Contusion in any part of the body	0.0075
Suriname	2017	Contusion in any part of the body	0.0075
Suriname	1990	Effect of different environmental factors	0.049
Suriname	1995	Effect of different environmental factors	0.049
Suriname	2000	Effect of different environmental factors	0.049
Suriname	2005	Effect of different environmental factors	0.049
Suriname	2010	Effect of different environmental factors	0.049
Suriname	2017	Effect of different environmental factors	0.049
Suriname	1990	Complications following therapeutic procedures	0.0506
Suriname	1995	Complications following therapeutic procedures	0.0506
Suriname	2000	Complications following therapeutic procedures	0.0506
Suriname	2005	Complications following therapeutic procedures	0.0506
Suriname	2010	Complications following therapeutic procedures	0.0506
Suriname	2017	Complications following therapeutic procedures	0.0506
Suriname	1990	Multiple fractures, dislocations, crashes, wounds, sprains, and strains	0.205
Suriname	1995	Multiple fractures, dislocations, crashes, wounds, sprains, and strains	0.1952
Suriname	2000	Multiple fractures, dislocations, crashes, wounds, sprains, and strains	0.1891
Suriname	2005	Multiple fractures, dislocations, crashes, wounds, sprains, and strains	0.1805
Suriname	2010	Multiple fractures, dislocations, crashes, wounds, sprains, and strains	0.1663
Suriname	2017	Multiple fractures, dislocations, crashes, wounds, sprains, and strains	0.1485
Suriname	1990	Amputation of upper limb, unilateral	0.0728
Suriname	1995	Amputation of upper limb, unilateral	0.0705
Suriname	2000	Amputation of upper limb, unilateral	0.0691
Suriname	2005	Amputation of upper limb, unilateral	0.0672
Suriname	2010	Amputation of upper limb, unilateral	0.0639
Suriname	2017	Amputation of upper limb, unilateral	0.0598
Suriname	1990	Amputation of thumb	0.0114
Suriname	1995	Amputation of thumb	0.0114
Suriname	2000	Amputation of thumb	0.0114
Suriname	2005	Amputation of thumb	0.0114
Suriname	2010	Amputation of thumb	0.0114
Suriname	2017	Amputation of thumb	0.0114
Suriname	1990	Amputation of toe/toes	0.0059
Suriname	1995	Amputation of toe/toes	0.0059
Suriname	2000	Amputation of toe/toes	0.0059
Suriname	2005	Amputation of toe/toes	0.0059
Suriname	2010	Amputation of toe/toes	0.0059
Suriname	2017	Amputation of toe/toes	0.0059
Suriname	1990	Burns, <20% total burned surface area without lower airway burns	0.0163
Suriname	1995	Burns, <20% total burned surface area without lower airway burns	0.0163
Suriname	2000	Burns, <20% total burned surface area without lower airway burns	0.0163
Suriname	2005	Burns, <20% total burned surface area without lower airway burns	0.0163
Suriname	2010	Burns, <20% total burned surface area without lower airway burns	0.0163
Suriname	2017	Burns, <20% total burned surface area without lower airway burns	0.0163
Suriname	1990	Burns, >=20% total burned surface area or >= 10% burned surface area if head/neck or hands/wrist involved w/o lower airway burns	0.2718
Suriname	1995	Burns, >=20% total burned surface area or >= 10% burned surface area if head/neck or hands/wrist involved w/o lower airway burns	0.2627
Suriname	2000	Burns, >=20% total burned surface area or >= 10% burned surface area if head/neck or hands/wrist involved w/o lower airway burns	0.257
Suriname	2005	Burns, >=20% total burned surface area or >= 10% burned surface area if head/neck or hands/wrist involved w/o lower airway burns	0.249
Suriname	2010	Burns, >=20% total burned surface area or >= 10% burned surface area if head/neck or hands/wrist involved w/o lower airway burns	0.2357
Suriname	2017	Burns, >=20% total burned surface area or >= 10% burned surface area if head/neck or hands/wrist involved w/o lower airway burns	0.2191
Trinidad and Tobago	1990	Amputation of lower limbs, bilateral	0.2036
Trinidad and Tobago	1995	Amputation of lower limbs, bilateral	0.1967
Trinidad and Tobago	2000	Amputation of lower limbs, bilateral	0.1882
Trinidad and Tobago	2005	Amputation of lower limbs, bilateral	0.1764
Trinidad and Tobago	2010	Amputation of lower limbs, bilateral	0.1664
Trinidad and Tobago	2017	Amputation of lower limbs, bilateral	0.1586
Trinidad and Tobago	1990	Lower airway burns	0.2252
Trinidad and Tobago	1995	Lower airway burns	0.2252
Trinidad and Tobago	2000	Lower airway burns	0.2252
Trinidad and Tobago	2005	Lower airway burns	0.2252
Trinidad and Tobago	2010	Lower airway burns	0.2252
Trinidad and Tobago	2017	Lower airway burns	0.2252
Trinidad and Tobago	1990	Dislocation of hip	0.0159
Trinidad and Tobago	1995	Dislocation of hip	0.0159
Trinidad and Tobago	2000	Dislocation of hip	0.0159
Trinidad and Tobago	2005	Dislocation of hip	0.0159
Trinidad and Tobago	2010	Dislocation of hip	0.0159
Trinidad and Tobago	2017	Dislocation of hip	0.0159
Trinidad and Tobago	1990	Dislocation of knee	0.1134
Trinidad and Tobago	1995	Dislocation of knee	0.1134
Trinidad and Tobago	2000	Dislocation of knee	0.1134
Trinidad and Tobago	2005	Dislocation of knee	0.1134
Trinidad and Tobago	2010	Dislocation of knee	0.1134
Trinidad and Tobago	2017	Dislocation of knee	0.1134
Trinidad and Tobago	1990	Dislocation of shoulder	0.062
Trinidad and Tobago	1995	Dislocation of shoulder	0.062
Trinidad and Tobago	2000	Dislocation of shoulder	0.062
Trinidad and Tobago	2005	Dislocation of shoulder	0.062
Trinidad and Tobago	2010	Dislocation of shoulder	0.062
Trinidad and Tobago	2017	Dislocation of shoulder	0.062
Trinidad and Tobago	1990	Muscle and tendon injuries, including sprains and strains lesser dislocations	0.0075
Trinidad and Tobago	1995	Muscle and tendon injuries, including sprains and strains lesser dislocations	0.0075
Trinidad and Tobago	2000	Muscle and tendon injuries, including sprains and strains lesser dislocations	0.0075
Trinidad and Tobago	2005	Muscle and tendon injuries, including sprains and strains lesser dislocations	0.0075
Trinidad and Tobago	2010	Muscle and tendon injuries, including sprains and strains lesser dislocations	0.0075
Trinidad and Tobago	2017	Muscle and tendon injuries, including sprains and strains lesser dislocations	0.0075
Trinidad and Tobago	1990	Fracture of clavicle, scapula, or humerus	0.0349
Trinidad and Tobago	1995	Fracture of clavicle, scapula, or humerus	0.0349
Trinidad and Tobago	2000	Fracture of clavicle, scapula, or humerus	0.0349
Trinidad and Tobago	2005	Fracture of clavicle, scapula, or humerus	0.0349
Trinidad and Tobago	2010	Fracture of clavicle, scapula, or humerus	0.0349
Trinidad and Tobago	2017	Fracture of clavicle, scapula, or humerus	0.0349
Trinidad and Tobago	1990	Fracture of face bones	0.0669
Trinidad and Tobago	1995	Fracture of face bones	0.0669
Trinidad and Tobago	2000	Fracture of face bones	0.0669
Trinidad and Tobago	2005	Fracture of face bones	0.0669
Trinidad and Tobago	2010	Fracture of face bones	0.0669
Trinidad and Tobago	2017	Fracture of face bones	0.0669
Trinidad and Tobago	1990	Fracture of foot bones except ankle	0.0264
Trinidad and Tobago	1995	Fracture of foot bones except ankle	0.0264
Trinidad and Tobago	2000	Fracture of foot bones except ankle	0.0264
Trinidad and Tobago	2005	Fracture of foot bones except ankle	0.0264
Trinidad and Tobago	2010	Fracture of foot bones except ankle	0.0264
Trinidad and Tobago	2017	Fracture of foot bones except ankle	0.0264
Trinidad and Tobago	1990	Fracture of hand (wrist and other distal part of hand)	0.0138
Trinidad and Tobago	1995	Fracture of hand (wrist and other distal part of hand)	0.0138
Trinidad and Tobago	2000	Fracture of hand (wrist and other distal part of hand)	0.0138
Trinidad and Tobago	2005	Fracture of hand (wrist and other distal part of hand)	0.0138
Trinidad and Tobago	2010	Fracture of hand (wrist and other distal part of hand)	0.0138
Trinidad and Tobago	2017	Fracture of hand (wrist and other distal part of hand)	0.0138
Trinidad and Tobago	1990	Fracture of hip	0.164
Trinidad and Tobago	1995	Fracture of hip	0.1548
Trinidad and Tobago	2000	Fracture of hip	0.1436
Trinidad and Tobago	2005	Fracture of hip	0.128
Trinidad and Tobago	2010	Fracture of hip	0.1148
Trinidad and Tobago	2017	Fracture of hip	0.1057
Trinidad and Tobago	1990	Amputation of upper limbs, bilateral	0.1974
Trinidad and Tobago	1995	Amputation of upper limbs, bilateral	0.1879
Trinidad and Tobago	2000	Amputation of upper limbs, bilateral	0.1763
Trinidad and Tobago	2005	Amputation of upper limbs, bilateral	0.1603
Trinidad and Tobago	2010	Amputation of upper limbs, bilateral	0.1466
Trinidad and Tobago	2017	Amputation of upper limbs, bilateral	0.1372
Trinidad and Tobago	1990	Fracture of patella, tibia or fibula, or ankle	0.0554
Trinidad and Tobago	1995	Fracture of patella, tibia or fibula, or ankle	0.0554
Trinidad and Tobago	2000	Fracture of patella, tibia or fibula, or ankle	0.0554
Trinidad and Tobago	2005	Fracture of patella, tibia or fibula, or ankle	0.0554
Trinidad and Tobago	2010	Fracture of patella, tibia or fibula, or ankle	0.0554
Trinidad and Tobago	2017	Fracture of patella, tibia or fibula, or ankle	0.0554
Trinidad and Tobago	1990	Fracture of pelvis	0.1823
Trinidad and Tobago	1995	Fracture of pelvis	0.1823
Trinidad and Tobago	2000	Fracture of pelvis	0.1823
Trinidad and Tobago	2005	Fracture of pelvis	0.1823
Trinidad and Tobago	2010	Fracture of pelvis	0.1823
Trinidad and Tobago	2017	Fracture of pelvis	0.1823
Trinidad and Tobago	1990	Fracture of radius and/or ulna	0.0435
Trinidad and Tobago	1995	Fracture of radius and/or ulna	0.0435
Trinidad and Tobago	2000	Fracture of radius and/or ulna	0.0435
Trinidad and Tobago	2005	Fracture of radius and/or ulna	0.0435
Trinidad and Tobago	2010	Fracture of radius and/or ulna	0.0435
Trinidad and Tobago	2017	Fracture of radius and/or ulna	0.0435
Trinidad and Tobago	1990	Fracture of skull	0.0714
Trinidad and Tobago	1995	Fracture of skull	0.0714
Trinidad and Tobago	2000	Fracture of skull	0.0714
Trinidad and Tobago	2005	Fracture of skull	0.0714
Trinidad and Tobago	2010	Fracture of skull	0.0714
Trinidad and Tobago	2017	Fracture of skull	0.0714
Trinidad and Tobago	1990	Fracture of sternum and/or fracture of one or more ribs	0.1027
Trinidad and Tobago	1995	Fracture of sternum and/or fracture of one or more ribs	0.1027
Trinidad and Tobago	2000	Fracture of sternum and/or fracture of one or more ribs	0.1027
Trinidad and Tobago	2005	Fracture of sternum and/or fracture of one or more ribs	0.1027
Trinidad and Tobago	2010	Fracture of sternum and/or fracture of one or more ribs	0.1027
Trinidad and Tobago	2017	Fracture of sternum and/or fracture of one or more ribs	0.1027
Trinidad and Tobago	1990	Fracture of vertebral column	0.1106
Trinidad and Tobago	1995	Fracture of vertebral column	0.1106
Trinidad and Tobago	2000	Fracture of vertebral column	0.1106
Trinidad and Tobago	2005	Fracture of vertebral column	0.1106
Trinidad and Tobago	2010	Fracture of vertebral column	0.1106
Trinidad and Tobago	2017	Fracture of vertebral column	0.1106
Trinidad and Tobago	1990	Fracture of femur, other than femoral neck	0.042
Trinidad and Tobago	1995	Fracture of femur, other than femoral neck	0.042
Trinidad and Tobago	2000	Fracture of femur, other than femoral neck	0.042
Trinidad and Tobago	2005	Fracture of femur, other than femoral neck	0.042
Trinidad and Tobago	2010	Fracture of femur, other than femoral neck	0.042
Trinidad and Tobago	2017	Fracture of femur, other than femoral neck	0.042
Trinidad and Tobago	1990	Minor TBI	0.1319
Trinidad and Tobago	1995	Minor TBI	0.1319
Trinidad and Tobago	2000	Minor TBI	0.1319
Trinidad and Tobago	2005	Minor TBI	0.1319
Trinidad and Tobago	2010	Minor TBI	0.1319
Trinidad and Tobago	2017	Minor TBI	0.1319
Trinidad and Tobago	1990	Moderate TBI	0.1637
Trinidad and Tobago	1995	Moderate TBI	0.1637
Trinidad and Tobago	2000	Moderate TBI	0.1637
Trinidad and Tobago	2005	Moderate TBI	0.1637
Trinidad and Tobago	2010	Moderate TBI	0.1637
Trinidad and Tobago	2017	Moderate TBI	0.1637
Trinidad and Tobago	1990	Amputation of fingers (excluding thumb)	0.0047
Trinidad and Tobago	1995	Amputation of fingers (excluding thumb)	0.0047
Trinidad and Tobago	2000	Amputation of fingers (excluding thumb)	0.0047
Trinidad and Tobago	2005	Amputation of fingers (excluding thumb)	0.0047
Trinidad and Tobago	2010	Amputation of fingers (excluding thumb)	0.0047
Trinidad and Tobago	2017	Amputation of fingers (excluding thumb)	0.0047
Trinidad and Tobago	1990	Spinal cord lesion at neck level	0.4486
Trinidad and Tobago	1995	Spinal cord lesion at neck level	0.4454
Trinidad and Tobago	2000	Spinal cord lesion at neck level	0.4415
Trinidad and Tobago	2005	Spinal cord lesion at neck level	0.4361
Trinidad and Tobago	2010	Spinal cord lesion at neck level	0.4316
Trinidad and Tobago	2017	Spinal cord lesion at neck level	0.4284
Trinidad and Tobago	1990	Spinal cord lesion below neck level	0.2552
Trinidad and Tobago	1995	Spinal cord lesion below neck level	0.2486
Trinidad and Tobago	2000	Spinal cord lesion below neck level	0.2404
Trinidad and Tobago	2005	Spinal cord lesion below neck level	0.229
Trinidad and Tobago	2010	Spinal cord lesion below neck level	0.2194
Trinidad and Tobago	2017	Spinal cord lesion below neck level	0.2127
Trinidad and Tobago	1990	Drowning and nonfatal submersion	0.2471
Trinidad and Tobago	1995	Drowning and nonfatal submersion	0.2471
Trinidad and Tobago	2000	Drowning and nonfatal submersion	0.2471
Trinidad and Tobago	2005	Drowning and nonfatal submersion	0.2471
Trinidad and Tobago	2010	Drowning and nonfatal submersion	0.2471
Trinidad and Tobago	2017	Drowning and nonfatal submersion	0.2471
Trinidad and Tobago	1990	Asphyxiation	0.2471
Trinidad and Tobago	1995	Asphyxiation	0.2471
Trinidad and Tobago	2000	Asphyxiation	0.2471
Trinidad and Tobago	2005	Asphyxiation	0.2471
Trinidad and Tobago	2010	Asphyxiation	0.2471
Trinidad and Tobago	2017	Asphyxiation	0.2471
Trinidad and Tobago	1990	Crush injury	0.1325
Trinidad and Tobago	1995	Crush injury	0.1325
Trinidad and Tobago	2000	Crush injury	0.1325
Trinidad and Tobago	2005	Crush injury	0.1325
Trinidad and Tobago	2010	Crush injury	0.1325
Trinidad and Tobago	2017	Crush injury	0.1325
Trinidad and Tobago	1990	Nerve injury	0.1129
Trinidad and Tobago	1995	Nerve injury	0.1129
Trinidad and Tobago	2000	Nerve injury	0.1129
Trinidad and Tobago	2005	Nerve injury	0.1129
Trinidad and Tobago	2010	Nerve injury	0.1129
Trinidad and Tobago	2017	Nerve injury	0.1129
Trinidad and Tobago	1990	Injury to eyes	0.0165
Trinidad and Tobago	1995	Injury to eyes	0.0165
Trinidad and Tobago	2000	Injury to eyes	0.0165
Trinidad and Tobago	2005	Injury to eyes	0.0165
Trinidad and Tobago	2010	Injury to eyes	0.0165
Trinidad and Tobago	2017	Injury to eyes	0.0165
Trinidad and Tobago	1990	Amputation of lower limb, unilateral	0.0804
Trinidad and Tobago	1995	Amputation of lower limb, unilateral	0.0768
Trinidad and Tobago	2000	Amputation of lower limb, unilateral	0.0724
Trinidad and Tobago	2005	Amputation of lower limb, unilateral	0.0664
Trinidad and Tobago	2010	Amputation of lower limb, unilateral	0.0612
Trinidad and Tobago	2017	Amputation of lower limb, unilateral	0.0577
Trinidad and Tobago	1990	Open wound(s)	0.011
Trinidad and Tobago	1995	Open wound(s)	0.011
Trinidad and Tobago	2000	Open wound(s)	0.011
Trinidad and Tobago	2005	Open wound(s)	0.011
Trinidad and Tobago	2010	Open wound(s)	0.011
Trinidad and Tobago	2017	Open wound(s)	0.011
Trinidad and Tobago	1990	Poisoning requiring urgent care	0.1628
Trinidad and Tobago	1995	Poisoning requiring urgent care	0.1628
Trinidad and Tobago	2000	Poisoning requiring urgent care	0.1628
Trinidad and Tobago	2005	Poisoning requiring urgent care	0.1628
Trinidad and Tobago	2010	Poisoning requiring urgent care	0.1628
Trinidad and Tobago	2017	Poisoning requiring urgent care	0.1628
Trinidad and Tobago	1990	Severe chest injury	0.0474
Trinidad and Tobago	1995	Severe chest injury	0.0474
Trinidad and Tobago	2000	Severe chest injury	0.0474
Trinidad and Tobago	2005	Severe chest injury	0.0474
Trinidad and Tobago	2010	Severe chest injury	0.0474
Trinidad and Tobago	2017	Severe chest injury	0.0474
Trinidad and Tobago	1990	Internal hemorrhage in abdomen and pelvis	0.1143
Trinidad and Tobago	1995	Internal hemorrhage in abdomen and pelvis	0.1143
Trinidad and Tobago	2000	Internal hemorrhage in abdomen and pelvis	0.1143
Trinidad and Tobago	2005	Internal hemorrhage in abdomen and pelvis	0.1143
Trinidad and Tobago	2010	Internal hemorrhage in abdomen and pelvis	0.1143
Trinidad and Tobago	2017	Internal hemorrhage in abdomen and pelvis	0.1143
Trinidad and Tobago	1990	Contusion in any part of the body	0.0075
Trinidad and Tobago	1995	Contusion in any part of the body	0.0075
Trinidad and Tobago	2000	Contusion in any part of the body	0.0075
Trinidad and Tobago	2005	Contusion in any part of the body	0.0075
Trinidad and Tobago	2010	Contusion in any part of the body	0.0075
Trinidad and Tobago	2017	Contusion in any part of the body	0.0075
Trinidad and Tobago	1990	Effect of different environmental factors	0.049
Trinidad and Tobago	1995	Effect of different environmental factors	0.049
Trinidad and Tobago	2000	Effect of different environmental factors	0.049
Trinidad and Tobago	2005	Effect of different environmental factors	0.049
Trinidad and Tobago	2010	Effect of different environmental factors	0.049
Trinidad and Tobago	2017	Effect of different environmental factors	0.049
Trinidad and Tobago	1990	Complications following therapeutic procedures	0.0506
Trinidad and Tobago	1995	Complications following therapeutic procedures	0.0506
Trinidad and Tobago	2000	Complications following therapeutic procedures	0.0506
Trinidad and Tobago	2005	Complications following therapeutic procedures	0.0506
Trinidad and Tobago	2010	Complications following therapeutic procedures	0.0506
Trinidad and Tobago	2017	Complications following therapeutic procedures	0.0506
Trinidad and Tobago	1990	Multiple fractures, dislocations, crashes, wounds, sprains, and strains	0.164
Trinidad and Tobago	1995	Multiple fractures, dislocations, crashes, wounds, sprains, and strains	0.1548
Trinidad and Tobago	2000	Multiple fractures, dislocations, crashes, wounds, sprains, and strains	0.1436
Trinidad and Tobago	2005	Multiple fractures, dislocations, crashes, wounds, sprains, and strains	0.128
Trinidad and Tobago	2010	Multiple fractures, dislocations, crashes, wounds, sprains, and strains	0.1148
Trinidad and Tobago	2017	Multiple fractures, dislocations, crashes, wounds, sprains, and strains	0.1057
Trinidad and Tobago	1990	Amputation of upper limb, unilateral	0.0634
Trinidad and Tobago	1995	Amputation of upper limb, unilateral	0.0613
Trinidad and Tobago	2000	Amputation of upper limb, unilateral	0.0587
Trinidad and Tobago	2005	Amputation of upper limb, unilateral	0.0551
Trinidad and Tobago	2010	Amputation of upper limb, unilateral	0.0521
Trinidad and Tobago	2017	Amputation of upper limb, unilateral	0.05
Trinidad and Tobago	1990	Amputation of thumb	0.0114
Trinidad and Tobago	1995	Amputation of thumb	0.0114
Trinidad and Tobago	2000	Amputation of thumb	0.0114
Trinidad and Tobago	2005	Amputation of thumb	0.0114
Trinidad and Tobago	2010	Amputation of thumb	0.0114
Trinidad and Tobago	2017	Amputation of thumb	0.0114
Trinidad and Tobago	1990	Amputation of toe/toes	0.0059
Trinidad and Tobago	1995	Amputation of toe/toes	0.0059
Trinidad and Tobago	2000	Amputation of toe/toes	0.0059
Trinidad and Tobago	2005	Amputation of toe/toes	0.0059
Trinidad and Tobago	2010	Amputation of toe/toes	0.0059
Trinidad and Tobago	2017	Amputation of toe/toes	0.0059
Trinidad and Tobago	1990	Burns, <20% total burned surface area without lower airway burns	0.0163
Trinidad and Tobago	1995	Burns, <20% total burned surface area without lower airway burns	0.0163
Trinidad and Tobago	2000	Burns, <20% total burned surface area without lower airway burns	0.0163
Trinidad and Tobago	2005	Burns, <20% total burned surface area without lower airway burns	0.0163
Trinidad and Tobago	2010	Burns, <20% total burned surface area without lower airway burns	0.0163
Trinidad and Tobago	2017	Burns, <20% total burned surface area without lower airway burns	0.0163
Trinidad and Tobago	1990	Burns, >=20% total burned surface area or >= 10% burned surface area if head/neck or hands/wrist involved w/o lower airway burns	0.2335
Trinidad and Tobago	1995	Burns, >=20% total burned surface area or >= 10% burned surface area if head/neck or hands/wrist involved w/o lower airway burns	0.225
Trinidad and Tobago	2000	Burns, >=20% total burned surface area or >= 10% burned surface area if head/neck or hands/wrist involved w/o lower airway burns	0.2146
Trinidad and Tobago	2005	Burns, >=20% total burned surface area or >= 10% burned surface area if head/neck or hands/wrist involved w/o lower airway burns	0.2001
Trinidad and Tobago	2010	Burns, >=20% total burned surface area or >= 10% burned surface area if head/neck or hands/wrist involved w/o lower airway burns	0.1878
Trinidad and Tobago	2017	Burns, >=20% total burned surface area or >= 10% burned surface area if head/neck or hands/wrist involved w/o lower airway burns	0.1793
Plurinational State of Bolivia	1990	Amputation of lower limbs, bilateral	0.2876
Plurinational State of Bolivia	1995	Amputation of lower limbs, bilateral	0.2702
Plurinational State of Bolivia	2000	Amputation of lower limbs, bilateral	0.253
Plurinational State of Bolivia	2005	Amputation of lower limbs, bilateral	0.2381
Plurinational State of Bolivia	2010	Amputation of lower limbs, bilateral	0.226
Plurinational State of Bolivia	2017	Amputation of lower limbs, bilateral	0.2093
Plurinational State of Bolivia	1990	Lower airway burns	0.2252
Plurinational State of Bolivia	1995	Lower airway burns	0.2252
Plurinational State of Bolivia	2000	Lower airway burns	0.2252
Plurinational State of Bolivia	2005	Lower airway burns	0.2252
Plurinational State of Bolivia	2010	Lower airway burns	0.2252
Plurinational State of Bolivia	2017	Lower airway burns	0.2252
Plurinational State of Bolivia	1990	Dislocation of hip	0.0159
Plurinational State of Bolivia	1995	Dislocation of hip	0.0159
Plurinational State of Bolivia	2000	Dislocation of hip	0.0159
Plurinational State of Bolivia	2005	Dislocation of hip	0.0159
Plurinational State of Bolivia	2010	Dislocation of hip	0.0159
Plurinational State of Bolivia	2017	Dislocation of hip	0.0159
Plurinational State of Bolivia	1990	Dislocation of knee	0.1134
Plurinational State of Bolivia	1995	Dislocation of knee	0.1134
Plurinational State of Bolivia	2000	Dislocation of knee	0.1134
Plurinational State of Bolivia	2005	Dislocation of knee	0.1134
Plurinational State of Bolivia	2010	Dislocation of knee	0.1134
Plurinational State of Bolivia	2017	Dislocation of knee	0.1134
Plurinational State of Bolivia	1990	Dislocation of shoulder	0.062
Plurinational State of Bolivia	1995	Dislocation of shoulder	0.062
Plurinational State of Bolivia	2000	Dislocation of shoulder	0.062
Plurinational State of Bolivia	2005	Dislocation of shoulder	0.062
Plurinational State of Bolivia	2010	Dislocation of shoulder	0.062
Plurinational State of Bolivia	2017	Dislocation of shoulder	0.062
Plurinational State of Bolivia	1990	Muscle and tendon injuries, including sprains and strains lesser dislocations	0.0075
Plurinational State of Bolivia	1995	Muscle and tendon injuries, including sprains and strains lesser dislocations	0.0075
Plurinational State of Bolivia	2000	Muscle and tendon injuries, including sprains and strains lesser dislocations	0.0075
Plurinational State of Bolivia	2005	Muscle and tendon injuries, including sprains and strains lesser dislocations	0.0075
Plurinational State of Bolivia	2010	Muscle and tendon injuries, including sprains and strains lesser dislocations	0.0075
Plurinational State of Bolivia	2017	Muscle and tendon injuries, including sprains and strains lesser dislocations	0.0075
Plurinational State of Bolivia	1990	Fracture of clavicle, scapula, or humerus	0.0349
Plurinational State of Bolivia	1995	Fracture of clavicle, scapula, or humerus	0.0349
Plurinational State of Bolivia	2000	Fracture of clavicle, scapula, or humerus	0.0349
Plurinational State of Bolivia	2005	Fracture of clavicle, scapula, or humerus	0.0349
Plurinational State of Bolivia	2010	Fracture of clavicle, scapula, or humerus	0.0349
Plurinational State of Bolivia	2017	Fracture of clavicle, scapula, or humerus	0.0349
Plurinational State of Bolivia	1990	Fracture of face bones	0.0669
Plurinational State of Bolivia	1995	Fracture of face bones	0.0669
Plurinational State of Bolivia	2000	Fracture of face bones	0.0669
Plurinational State of Bolivia	2005	Fracture of face bones	0.0669
Plurinational State of Bolivia	2010	Fracture of face bones	0.0669
Plurinational State of Bolivia	2017	Fracture of face bones	0.0669
Plurinational State of Bolivia	1990	Fracture of foot bones except ankle	0.0264
Plurinational State of Bolivia	1995	Fracture of foot bones except ankle	0.0264
Plurinational State of Bolivia	2000	Fracture of foot bones except ankle	0.0264
Plurinational State of Bolivia	2005	Fracture of foot bones except ankle	0.0264
Plurinational State of Bolivia	2010	Fracture of foot bones except ankle	0.0264

Plurinational State of Bolivia	2017	Fracture of foot bones except ankle	0.0264
Plurinational State of Bolivia	1990	Fracture of hand (wrist and other distal part of hand)	0.0138
Plurinational State of Bolivia	1995	Fracture of hand (wrist and other distal part of hand)	0.0138
Plurinational State of Bolivia	2000	Fracture of hand (wrist and other distal part of hand)	0.0138
Plurinational State of Bolivia	2005	Fracture of hand (wrist and other distal part of hand)	0.0138
Plurinational State of Bolivia	2010	Fracture of hand (wrist and other distal part of hand)	0.0138
Plurinational State of Bolivia	2017	Fracture of hand (wrist and other distal part of hand)	0.0138
Plurinational State of Bolivia	1990	Fracture of hip	0.2751
Plurinational State of Bolivia	1995	Fracture of hip	0.2521
Plurinational State of Bolivia	2000	Fracture of hip	0.2293
Plurinational State of Bolivia	2005	Fracture of hip	0.2096
Plurinational State of Bolivia	2010	Fracture of hip	0.1935
Plurinational State of Bolivia	2017	Fracture of hip	0.1715
Plurinational State of Bolivia	1990	Amputation of upper limbs, bilateral	0.3121
Plurinational State of Bolivia	1995	Amputation of upper limbs, bilateral	0.2883
Plurinational State of Bolivia	2000	Amputation of upper limbs, bilateral	0.2649
Plurinational State of Bolivia	2005	Amputation of upper limbs, bilateral	0.2445
Plurinational State of Bolivia	2010	Amputation of upper limbs, bilateral	0.2279
Plurinational State of Bolivia	2017	Amputation of upper limbs, bilateral	0.2051
Plurinational State of Bolivia	1990	Fracture of patella, tibia or fibula, or ankle	0.0554
Plurinational State of Bolivia	1995	Fracture of patella, tibia or fibula, or ankle	0.0554
Plurinational State of Bolivia	2000	Fracture of patella, tibia or fibula, or ankle	0.0554
Plurinational State of Bolivia	2005	Fracture of patella, tibia or fibula, or ankle	0.0554
Plurinational State of Bolivia	2010	Fracture of patella, tibia or fibula, or ankle	0.0554
Plurinational State of Bolivia	2017	Fracture of patella, tibia or fibula, or ankle	0.0554
Plurinational State of Bolivia	1990	Fracture of pelvis	0.1823
Plurinational State of Bolivia	1995	Fracture of pelvis	0.1823
Plurinational State of Bolivia	2000	Fracture of pelvis	0.1823
Plurinational State of Bolivia	2005	Fracture of pelvis	0.1823
Plurinational State of Bolivia	2010	Fracture of pelvis	0.1823
Plurinational State of Bolivia	2017	Fracture of pelvis	0.1823
Plurinational State of Bolivia	1990	Fracture of radius and/or ulna	0.0435
Plurinational State of Bolivia	1995	Fracture of radius and/or ulna	0.0435
Plurinational State of Bolivia	2000	Fracture of radius and/or ulna	0.0435
Plurinational State of Bolivia	2005	Fracture of radius and/or ulna	0.0435
Plurinational State of Bolivia	2010	Fracture of radius and/or ulna	0.0435
Plurinational State of Bolivia	2017	Fracture of radius and/or ulna	0.0435
Plurinational State of Bolivia	1990	Fracture of skull	0.0714
Plurinational State of Bolivia	1995	Fracture of skull	0.0714
Plurinational State of Bolivia	2000	Fracture of skull	0.0714
Plurinational State of Bolivia	2005	Fracture of skull	0.0714
Plurinational State of Bolivia	2010	Fracture of skull	0.0714
Plurinational State of Bolivia	2017	Fracture of skull	0.0714
Plurinational State of Bolivia	1990	Fracture of sternum and/or fracture of one or more ribs	0.1027
Plurinational State of Bolivia	1995	Fracture of sternum and/or fracture of one or more ribs	0.1027
Plurinational State of Bolivia	2000	Fracture of sternum and/or fracture of one or more ribs	0.1027
Plurinational State of Bolivia	2005	Fracture of sternum and/or fracture of one or more ribs	0.1027
Plurinational State of Bolivia	2010	Fracture of sternum and/or fracture of one or more ribs	0.1027
Plurinational State of Bolivia	2017	Fracture of sternum and/or fracture of one or more ribs	0.1027
Plurinational State of Bolivia	1990	Fracture of vertebral column	0.1106
Plurinational State of Bolivia	1995	Fracture of vertebral column	0.1106
Plurinational State of Bolivia	2000	Fracture of vertebral column	0.1106
Plurinational State of Bolivia	2005	Fracture of vertebral column	0.1106
Plurinational State of Bolivia	2010	Fracture of vertebral column	0.1106
Plurinational State of Bolivia	2017	Fracture of vertebral column	0.1106
Plurinational State of Bolivia	1990	Fracture of femur, other than femoral neck	0.042
Plurinational State of Bolivia	1995	Fracture of femur, other than femoral neck	0.042
Plurinational State of Bolivia	2000	Fracture of femur, other than femoral neck	0.042
Plurinational State of Bolivia	2005	Fracture of femur, other than femoral neck	0.042
Plurinational State of Bolivia	2010	Fracture of femur, other than femoral neck	0.042
Plurinational State of Bolivia	2017	Fracture of femur, other than femoral neck	0.042
Plurinational State of Bolivia	1990	Minor TBI	0.1319
Plurinational State of Bolivia	1995	Minor TBI	0.1319
Plurinational State of Bolivia	2000	Minor TBI	0.1319
Plurinational State of Bolivia	2005	Minor TBI	0.1319
Plurinational State of Bolivia	2010	Minor TBI	0.1319
Plurinational State of Bolivia	2017	Minor TBI	0.1319
Plurinational State of Bolivia	1990	Moderate TBI	0.1637
Plurinational State of Bolivia	1995	Moderate TBI	0.1637
Plurinational State of Bolivia	2000	Moderate TBI	0.1637
Plurinational State of Bolivia	2005	Moderate TBI	0.1637
Plurinational State of Bolivia	2010	Moderate TBI	0.1637
Plurinational State of Bolivia	2017	Moderate TBI	0.1637
Plurinational State of Bolivia	1990	Amputation of fingers (excluding thumb)	0.0047
Plurinational State of Bolivia	1995	Amputation of fingers (excluding thumb)	0.0047
Plurinational State of Bolivia	2000	Amputation of fingers (excluding thumb)	0.0047
Plurinational State of Bolivia	2005	Amputation of fingers (excluding thumb)	0.0047
Plurinational State of Bolivia	2010	Amputation of fingers (excluding thumb)	0.0047
Plurinational State of Bolivia	2017	Amputation of fingers (excluding thumb)	0.0047
Plurinational State of Bolivia	1990	Spinal cord lesion at neck level	0.487
Plurinational State of Bolivia	1995	Spinal cord lesion at neck level	0.479
Plurinational State of Bolivia	2000	Spinal cord lesion at neck level	0.4712
Plurinational State of Bolivia	2005	Spinal cord lesion at neck level	0.4643
Plurinational State of Bolivia	2010	Spinal cord lesion at neck level	0.4588
Plurinational State of Bolivia	2017	Spinal cord lesion at neck level	0.4512
Plurinational State of Bolivia	1990	Spinal cord lesion below neck level	0.3364
Plurinational State of Bolivia	1995	Spinal cord lesion below neck level	0.3195
Plurinational State of Bolivia	2000	Spinal cord lesion below neck level	0.3029
Plurinational State of Bolivia	2005	Spinal cord lesion below neck level	0.2885
Plurinational State of Bolivia	2010	Spinal cord lesion below neck level	0.2768
Plurinational State of Bolivia	2017	Spinal cord lesion below neck level	0.2607
Plurinational State of Bolivia	1990	Drowning and nonfatal submersion	0.2471
Plurinational State of Bolivia	1995	Drowning and nonfatal submersion	0.2471
Plurinational State of Bolivia	2000	Drowning and nonfatal submersion	0.2471
Plurinational State of Bolivia	2005	Drowning and nonfatal submersion	0.2471
Plurinational State of Bolivia	2010	Drowning and nonfatal submersion	0.2471
Plurinational State of Bolivia	2017	Drowning and nonfatal submersion	0.2471
Plurinational State of Bolivia	1990	Asphyxiation	0.2471
Plurinational State of Bolivia	1995	Asphyxiation	0.2471
Plurinational State of Bolivia	2000	Asphyxiation	0.2471
Plurinational State of Bolivia	2005	Asphyxiation	0.2471
Plurinational State of Bolivia	2010	Asphyxiation	0.2471
Plurinational State of Bolivia	2017	Asphyxiation	0.2471
Plurinational State of Bolivia	1990	Crush injury	0.1325
Plurinational State of Bolivia	1995	Crush injury	0.1325
Plurinational State of Bolivia	2000	Crush injury	0.1325
Plurinational State of Bolivia	2005	Crush injury	0.1325
Plurinational State of Bolivia	2010	Crush injury	0.1325
Plurinational State of Bolivia	2017	Crush injury	0.1325
Plurinational State of Bolivia	1990	Nerve injury	0.1129
Plurinational State of Bolivia	1995	Nerve injury	0.1129
Plurinational State of Bolivia	2000	Nerve injury	0.1129
Plurinational State of Bolivia	2005	Nerve injury	0.1129
Plurinational State of Bolivia	2010	Nerve injury	0.1129
Plurinational State of Bolivia	2017	Nerve injury	0.1129
Plurinational State of Bolivia	1990	Injury to eyes	0.0165
Plurinational State of Bolivia	1995	Injury to eyes	0.0165
Plurinational State of Bolivia	2000	Injury to eyes	0.0165
Plurinational State of Bolivia	2005	Injury to eyes	0.0165
Plurinational State of Bolivia	2010	Injury to eyes	0.0165
Plurinational State of Bolivia	2017	Injury to eyes	0.0165
Plurinational State of Bolivia	1990	Amputation of lower limb, unilateral	0.1237
Plurinational State of Bolivia	1995	Amputation of lower limb, unilateral	0.1147
Plurinational State of Bolivia	2000	Amputation of lower limb, unilateral	0.1059
Plurinational State of Bolivia	2005	Amputation of lower limb, unilateral	0.0982
Plurinational State of Bolivia	2010	Amputation of lower limb, unilateral	0.0919
Plurinational State of Bolivia	2017	Amputation of lower limb, unilateral	0.0833
Plurinational State of Bolivia	1990	Open wound(s)	0.011
Plurinational State of Bolivia	1995	Open wound(s)	0.011
Plurinational State of Bolivia	2000	Open wound(s)	0.011
Plurinational State of Bolivia	2005	Open wound(s)	0.011
Plurinational State of Bolivia	2010	Open wound(s)	0.011
Plurinational State of Bolivia	2017	Open wound(s)	0.011
Plurinational State of Bolivia	1990	Poisoning requiring urgent care	0.1628
Plurinational State of Bolivia	1995	Poisoning requiring urgent care	0.1628
Plurinational State of Bolivia	2000	Poisoning requiring urgent care	0.1628
Plurinational State of Bolivia	2005	Poisoning requiring urgent care	0.1628
Plurinational State of Bolivia	2010	Poisoning requiring urgent care	0.1628
Plurinational State of Bolivia	2017	Poisoning requiring urgent care	0.1628
Plurinational State of Bolivia	1990	Severe chest injury	0.0474
Plurinational State of Bolivia	1995	Severe chest injury	0.0474
Plurinational State of Bolivia	2000	Severe chest injury	0.0474
Plurinational State of Bolivia	2005	Severe chest injury	0.0474
Plurinational State of Bolivia	2010	Severe chest injury	0.0474
Plurinational State of Bolivia	2017	Severe chest injury	0.0474
Plurinational State of Bolivia	1990	Internal hemorrhage in abdomen and pelvis	0.1143
Plurinational State of Bolivia	1995	Internal hemorrhage in abdomen and pelvis	0.1143
Plurinational State of Bolivia	2000	Internal hemorrhage in abdomen and pelvis	0.1143
Plurinational State of Bolivia	2005	Internal hemorrhage in abdomen and pelvis	0.1143
Plurinational State of Bolivia	2010	Internal hemorrhage in abdomen and pelvis	0.1143
Plurinational State of Bolivia	2017	Internal hemorrhage in abdomen and pelvis	0.1143
Plurinational State of Bolivia	1990	Contusion in any part of the body	0.0075
Plurinational State of Bolivia	1995	Contusion in any part of the body	0.0075
Plurinational State of Bolivia	2000	Contusion in any part of the body	0.0075
Plurinational State of Bolivia	2005	Contusion in any part of the body	0.0075
Plurinational State of Bolivia	2010	Contusion in any part of the body	0.0075
Plurinational State of Bolivia	2017	Contusion in any part of the body	0.0075
Plurinational State of Bolivia	1990	Effect of different environmental factors	0.049
Plurinational State of Bolivia	1995	Effect of different environmental factors	0.049
Plurinational State of Bolivia	2000	Effect of different environmental factors	0.049
Plurinational State of Bolivia	2005	Effect of different environmental factors	0.049
Plurinational State of Bolivia	2010	Effect of different environmental factors	0.049
Plurinational State of Bolivia	2017	Effect of different environmental factors	0.049
Plurinational State of Bolivia	1990	Complications following therapeutic procedures	0.0506
Plurinational State of Bolivia	1995	Complications following therapeutic procedures	0.0506
Plurinational State of Bolivia	2000	Complications following therapeutic procedures	0.0506
Plurinational State of Bolivia	2005	Complications following therapeutic procedures	0.0506
Plurinational State of Bolivia	2010	Complications following therapeutic procedures	0.0506
Plurinational State of Bolivia	2017	Complications following therapeutic procedures	0.0506
Plurinational State of Bolivia	1990	Multiple fractures, dislocations, crashes, wounds, sprains, and strains	0.2751
Plurinational State of Bolivia	1995	Multiple fractures, dislocations, crashes, wounds, sprains, and strains	0.2521
Plurinational State of Bolivia	2000	Multiple fractures, dislocations, crashes, wounds, sprains, and strains	0.2293
Plurinational State of Bolivia	2005	Multiple fractures, dislocations, crashes, wounds, sprains, and strains	0.2096
Plurinational State of Bolivia	2010	Multiple fractures, dislocations, crashes, wounds, sprains, and strains	0.1935
Plurinational State of Bolivia	2017	Multiple fractures, dislocations, crashes, wounds, sprains, and strains	0.1715
Plurinational State of Bolivia	1990	Amputation of upper limb, unilateral	0.0889
Plurinational State of Bolivia	1995	Amputation of upper limb, unilateral	0.0836
Plurinational State of Bolivia	2000	Amputation of upper limb, unilateral	0.0784
Plurinational State of Bolivia	2005	Amputation of upper limb, unilateral	0.0738
Plurinational State of Bolivia	2010	Amputation of upper limb, unilateral	0.0702
Plurinational State of Bolivia	2017	Amputation of upper limb, unilateral	0.0651
Plurinational State of Bolivia	1990	Amputation of thumb	0.0114
Plurinational State of Bolivia	1995	Amputation of thumb	0.0114
Plurinational State of Bolivia	2000	Amputation of thumb	0.0114
Plurinational State of Bolivia	2005	Amputation of thumb	0.0114
Plurinational State of Bolivia	2010	Amputation of thumb	0.0114
Plurinational State of Bolivia	2017	Amputation of thumb	0.0114
Plurinational State of Bolivia	1990	Amputation of toe/toes	0.0059
Plurinational State of Bolivia	1995	Amputation of toe/toes	0.0059
Plurinational State of Bolivia	2000	Amputation of toe/toes	0.0059
Plurinational State of Bolivia	2005	Amputation of toe/toes	0.0059
Plurinational State of Bolivia	2010	Amputation of toe/toes	0.0059
Plurinational State of Bolivia	2017	Amputation of toe/toes	0.0059
Plurinational State of Bolivia	1990	Burns, <20% total burned surface area without lower airway burns	0.0163
Plurinational State of Bolivia	1995	Burns, <20% total burned surface area without lower airway burns	0.0163
Plurinational State of Bolivia	2000	Burns, <20% total burned surface area without lower airway burns	0.0163
Plurinational State of Bolivia	2005	Burns, <20% total burned surface area without lower airway burns	0.0163
Plurinational State of Bolivia	2010	Burns, <20% total burned surface area without lower airway burns	0.0163
Plurinational State of Bolivia	2017	Burns, <20% total burned surface area without lower airway burns	0.0163
Plurinational State of Bolivia	1990	Burns, >=20% total burned surface area or >= 10% burned surface area if head/neck or hands/wrist involved w/o lower airway burns	0.337
Plurinational State of Bolivia	1995	Burns, >=20% total burned surface area or >= 10% burned surface area if head/neck or hands/wrist involved w/o lower airway burns	0.3156
Plurinational State of Bolivia	2000	Burns, >=20% total burned surface area or >= 10% burned surface area if head/neck or hands/wrist involved w/o lower airway burns	0.2944
Plurinational State of Bolivia	2005	Burns, >=20% total burned surface area or >= 10% burned surface area if head/neck or hands/wrist involved w/o lower airway burns	0.276
Plurinational State of Bolivia	2010	Burns, >=20% total burned surface area or >= 10% burned surface area if head/neck or hands/wrist involved w/o lower airway burns	0.2611
Plurinational State of Bolivia	2017	Burns, >=20% total burned surface area or >= 10% burned surface area if head/neck or hands/wrist involved w/o lower airway burns	0.2405
Ecuador	1990	Amputation of lower limbs, bilateral	0.2486
Ecuador	1995	Amputation of lower limbs, bilateral	0.2258
Ecuador	2000	Amputation of lower limbs, bilateral	0.204
Ecuador	2005	Amputation of lower limbs, bilateral	0.1893
Ecuador	2010	Amputation of lower limbs, bilateral	0.1792
Ecuador	2017	Amputation of lower limbs, bilateral	0.1648
Ecuador	1990	Lower airway burns	0.2252
Ecuador	1995	Lower airway burns	0.2252
Ecuador	2000	Lower airway burns	0.2252
Ecuador	2005	Lower airway burns	0.2252
Ecuador	2010	Lower airway burns	0.2252
Ecuador	2017	Lower airway burns	0.2252
Ecuador	1990	Dislocation of hip	0.0159
Ecuador	1995	Dislocation of hip	0.0159
Ecuador	2000	Dislocation of hip	0.0159
Ecuador	2005	Dislocation of hip	0.0159
Ecuador	2010	Dislocation of hip	0.0159
Ecuador	2017	Dislocation of hip	0.0159
Ecuador	1990	Dislocation of knee	0.1134
Ecuador	1995	Dislocation of knee	0.1134
Ecuador	2000	Dislocation of knee	0.1134
Ecuador	2005	Dislocation of knee	0.1134
Ecuador	2010	Dislocation of knee	0.1134
Ecuador	2017	Dislocation of knee	0.1134
Ecuador	1990	Dislocation of shoulder	0.062
Ecuador	1995	Dislocation of shoulder	0.062
Ecuador	2000	Dislocation of shoulder	0.062
Ecuador	2005	Dislocation of shoulder	0.062
Ecuador	2010	Dislocation of shoulder	0.062
Ecuador	2017	Dislocation of shoulder	0.062
Ecuador	1990	Muscle and tendon injuries, including sprains and strains lesser dislocations	0.0075
Ecuador	1995	Muscle and tendon injuries, including sprains and strains lesser dislocations	0.0075
Ecuador	2000	Muscle and tendon injuries, including sprains and strains lesser dislocations	0.0075
Ecuador	2005	Muscle and tendon injuries, including sprains and strains lesser dislocations	0.0075
Ecuador	2010	Muscle and tendon injuries, including sprains and strains lesser dislocations	0.0075
Ecuador	2017	Muscle and tendon injuries, including sprains and strains lesser dislocations	0.0075
Ecuador	1990	Fracture of clavicle, scapula, or humerus	0.0349
Ecuador	1995	Fracture of clavicle, scapula, or humerus	0.0349
Ecuador	2000	Fracture of clavicle, scapula, or humerus	0.0349
Ecuador	2005	Fracture of clavicle, scapula, or humerus	0.0349
Ecuador	2010	Fracture of clavicle, scapula, or humerus	0.0349
Ecuador	2017	Fracture of clavicle, scapula, or humerus	0.0349
Ecuador	1990	Fracture of face bones	0.0669
Ecuador	1995	Fracture of face bones	0.0669
Ecuador	2000	Fracture of face bones	0.0669
Ecuador	2005	Fracture of face bones	0.0669
Ecuador	2010	Fracture of face bones	0.0669
Ecuador	2017	Fracture of face bones	0.0669
Ecuador	1990	Fracture of foot bones except ankle	0.0264
Ecuador	1995	Fracture of foot bones except ankle	0.0264
Ecuador	2000	Fracture of foot bones except ankle	0.0264
Ecuador	2005	Fracture of foot bones except ankle	0.0264
Ecuador	2010	Fracture of foot bones except ankle	0.0264
Ecuador	2017	Fracture of foot bones except ankle	0.0264
Ecuador	1990	Fracture of hand (wrist and other distal part of hand)	0.0138
Ecuador	1995	Fracture of hand (wrist and other distal part of hand)	0.0138
Ecuador	2000	Fracture of hand (wrist and other distal part of hand)	0.0138
Ecuador	2005	Fracture of hand (wrist and other distal part of hand)	0.0138
Ecuador	2010	Fracture of hand (wrist and other distal part of hand)	0.0138
Ecuador	2017	Fracture of hand (wrist and other distal part of hand)	0.0138
Ecuador	1990	Fracture of hip	0.2234
Ecuador	1995	Fracture of hip	0.1933
Ecuador	2000	Fracture of hip	0.1645
Ecuador	2005	Fracture of hip	0.145
Ecuador	2010	Fracture of hip	0.1317
Ecuador	2017	Fracture of hip	0.1127
Ecuador	1990	Amputation of upper limbs, bilateral	0.2588
Ecuador	1995	Amputation of upper limbs, bilateral	0.2277
Ecuador	2000	Amputation of upper limbs, bilateral	0.198
Ecuador	2005	Amputation of upper limbs, bilateral	0.1778
Ecuador	2010	Amputation of upper limbs, bilateral	0.1641
Ecuador	2017	Amputation of upper limbs, bilateral	0.1444
Ecuador	1990	Fracture of patella, tibia or fibula, or ankle	0.0554
Ecuador	1995	Fracture of patella, tibia or fibula, or ankle	0.0554
Ecuador	2000	Fracture of patella, tibia or fibula, or ankle	0.0554
Ecuador	2005	Fracture of patella, tibia or fibula, or ankle	0.0554
Ecuador	2010	Fracture of patella, tibia or fibula, or ankle	0.0554
Ecuador	2017	Fracture of patella, tibia or fibula, or ankle	0.0554
Ecuador	1990	Fracture of pelvis	0.1823
Ecuador	1995	Fracture of pelvis	0.1823
Ecuador	2000	Fracture of pelvis	0.1823
Ecuador	2005	Fracture of pelvis	0.1823
Ecuador	2010	Fracture of pelvis	0.1823
Ecuador	2017	Fracture of pelvis	0.1823
Ecuador	1990	Fracture of radius and/or ulna	0.0435
Ecuador	1995	Fracture of radius and/or ulna	0.0435
Ecuador	2000	Fracture of radius and/or ulna	0.0435
Ecuador	2005	Fracture of radius and/or ulna	0.0435
Ecuador	2010	Fracture of radius and/or ulna	0.0435
Ecuador	2017	Fracture of radius and/or ulna	0.0435
Ecuador	1990	Fracture of skull	0.0714
Ecuador	1995	Fracture of skull	0.0714
Ecuador	2000	Fracture of skull	0.0714
Ecuador	2005	Fracture of skull	0.0714
Ecuador	2010	Fracture of skull	0.0714
Ecuador	2017	Fracture of skull	0.0714
Ecuador	1990	Fracture of sternum and/or fracture of one or more ribs	0.1027
Ecuador	1995	Fracture of sternum and/or fracture of one or more ribs	0.1027
Ecuador	2000	Fracture of sternum and/or fracture of one or more ribs	0.1027
Ecuador	2005	Fracture of sternum and/or fracture of one or more ribs	0.1027
Ecuador	2010	Fracture of sternum and/or fracture of one or more ribs	0.1027
Ecuador	2017	Fracture of sternum and/or fracture of one or more ribs	0.1027
Ecuador	1990	Fracture of vertebral column	0.1106
Ecuador	1995	Fracture of vertebral column	0.1106
Ecuador	2000	Fracture of vertebral column	0.1106
Ecuador	2005	Fracture of vertebral column	0.1106
Ecuador	2010	Fracture of vertebral column	0.1106
Ecuador	2017	Fracture of vertebral column	0.1106
Ecuador	1990	Fracture of femur, other than femoral neck	0.042
Ecuador	1995	Fracture of femur, other than femoral neck	0.042
Ecuador	2000	Fracture of femur, other than femoral neck	0.042
Ecuador	2005	Fracture of femur, other than femoral neck	0.042
Ecuador	2010	Fracture of femur, other than femoral neck	0.042
Ecuador	2017	Fracture of femur, other than femoral neck	0.042
Ecuador	1990	Minor TBI	0.1319
Ecuador	1995	Minor TBI	0.1319
Ecuador	2000	Minor TBI	0.1319
Ecuador	2005	Minor TBI	0.1319
Ecuador	2010	Minor TBI	0.1319
Ecuador	2017	Minor TBI	0.1319
Ecuador	1990	Moderate TBI	0.1637
Ecuador	1995	Moderate TBI	0.1637
Ecuador	2000	Moderate TBI	0.1637
Ecuador	2005	Moderate TBI	0.1637
Ecuador	2010	Moderate TBI	0.1637
Ecuador	2017	Moderate TBI	0.1637
Ecuador	1990	Amputation of fingers (excluding thumb)	0.0047
Ecuador	1995	Amputation of fingers (excluding thumb)	0.0047
Ecuador	2000	Amputation of fingers (excluding thumb)	0.0047
Ecuador	2005	Amputation of fingers (excluding thumb)	0.0047
Ecuador	2010	Amputation of fingers (excluding thumb)	0.0047
Ecuador	2017	Amputation of fingers (excluding thumb)	0.0047
Ecuador	1990	Spinal cord lesion at neck level	0.4691
Ecuador	1995	Spinal cord lesion at neck level	0.4587
Ecuador	2000	Spinal cord lesion at neck level	0.4488
Ecuador	2005	Spinal cord lesion at neck level	0.442
Ecuador	2010	Spinal cord lesion at neck level	0.4374
Ecuador	2017	Spinal cord lesion at neck level	0.4308
Ecuador	1990	Spinal cord lesion below neck level	0.2986
Ecuador	1995	Spinal cord lesion below neck level	0.2766
Ecuador	2000	Spinal cord lesion below neck level	0.2556
Ecuador	2005	Spinal cord lesion below neck level	0.2414
Ecuador	2010	Spinal cord lesion below neck level	0.2317
Ecuador	2017	Spinal cord lesion below neck level	0.2178
Ecuador	1990	Drowning and nonfatal submersion	0.2471
Ecuador	1995	Drowning and nonfatal submersion	0.2471
Ecuador	2000	Drowning and nonfatal submersion	0.2471
Ecuador	2005	Drowning and nonfatal submersion	0.2471
Ecuador	2010	Drowning and nonfatal submersion	0.2471
Ecuador	2017	Drowning and nonfatal submersion	0.2471
Ecuador	1990	Asphyxiation	0.2471
Ecuador	1995	Asphyxiation	0.2471
Ecuador	2000	Asphyxiation	0.2471
Ecuador	2005	Asphyxiation	0.2471
Ecuador	2010	Asphyxiation	0.2471
Ecuador	2017	Asphyxiation	0.2471
Ecuador	1990	Crush injury	0.1325
Ecuador	1995	Crush injury	0.1325
Ecuador	2000	Crush injury	0.1325
Ecuador	2005	Crush injury	0.1325
Ecuador	2010	Crush injury	0.1325
Ecuador	2017	Crush injury	0.1325
Ecuador	1990	Nerve injury	0.1129
Ecuador	1995	Nerve injury	0.1129
Ecuador	2000	Nerve injury	0.1129
Ecuador	2005	Nerve injury	0.1129
Ecuador	2010	Nerve injury	0.1129
Ecuador	2017	Nerve injury	0.1129
Ecuador	1990	Injury to eyes	0.0165
Ecuador	1995	Injury to eyes	0.0165
Ecuador	2000	Injury to eyes	0.0165
Ecuador	2005	Injury to eyes	0.0165
Ecuador	2010	Injury to eyes	0.0165
Ecuador	2017	Injury to eyes	0.0165
Ecuador	1990	Amputation of lower limb, unilateral	0.1036
Ecuador	1995	Amputation of lower limb, unilateral	0.0918
Ecuador	2000	Amputation of lower limb, unilateral	0.0806
Ecuador	2005	Amputation of lower limb, unilateral	0.073
Ecuador	2010	Amputation of lower limb, unilateral	0.0678
Ecuador	2017	Amputation of lower limb, unilateral	0.0604
Ecuador	1990	Open wound(s)	0.011
Ecuador	1995	Open wound(s)	0.011
Ecuador	2000	Open wound(s)	0.011
Ecuador	2005	Open wound(s)	0.011
Ecuador	2010	Open wound(s)	0.011
Ecuador	2017	Open wound(s)	0.011
Ecuador	1990	Poisoning requiring urgent care	0.1628
Ecuador	1995	Poisoning requiring urgent care	0.1628
Ecuador	2000	Poisoning requiring urgent care	0.1628
Ecuador	2005	Poisoning requiring urgent care	0.1628
Ecuador	2010	Poisoning requiring urgent care	0.1628
Ecuador	2017	Poisoning requiring urgent care	0.1628
Ecuador	1990	Severe chest injury	0.0474
Ecuador	1995	Severe chest injury	0.0474
Ecuador	2000	Severe chest injury	0.0474
Ecuador	2005	Severe chest injury	0.0474
Ecuador	2010	Severe chest injury	0.0474
Ecuador	2017	Severe chest injury	0.0474
Ecuador	1990	Internal hemorrhage in abdomen and pelvis	0.1143
Ecuador	1995	Internal hemorrhage in abdomen and pelvis	0.1143
Ecuador	2000	Internal hemorrhage in abdomen and pelvis	0.1143
Ecuador	2005	Internal hemorrhage in abdomen and pelvis	0.1143
Ecuador	2010	Internal hemorrhage in abdomen and pelvis	0.1143
Ecuador	2017	Internal hemorrhage in abdomen and pelvis	0.1143
Ecuador	1990	Contusion in any part of the body	0.0075
Ecuador	1995	Contusion in any part of the body	0.0075
Ecuador	2000	Contusion in any part of the body	0.0075
Ecuador	2005	Contusion in any part of the body	0.0075
Ecuador	2010	Contusion in any part of the body	0.0075
Ecuador	2017	Contusion in any part of the body	0.0075
Ecuador	1990	Effect of different environmental factors	0.049
Ecuador	1995	Effect of different environmental factors	0.049
Ecuador	2000	Effect of different environmental factors	0.049
Ecuador	2005	Effect of different environmental factors	0.049
Ecuador	2010	Effect of different environmental factors	0.049
Ecuador	2017	Effect of different environmental factors	0.049
Ecuador	1990	Complications following therapeutic procedures	0.0506
Ecuador	1995	Complications following therapeutic procedures	0.0506
Ecuador	2000	Complications following therapeutic procedures	0.0506
Ecuador	2005	Complications following therapeutic procedures	0.0506
Ecuador	2010	Complications following therapeutic procedures	0.0506
Ecuador	2017	Complications following therapeutic procedures	0.0506
Ecuador	1990	Multiple fractures, dislocations, crashes, wounds, sprains, and strains	0.2234
Ecuador	1995	Multiple fractures, dislocations, crashes, wounds, sprains, and strains	0.1933
Ecuador	2000	Multiple fractures, dislocations, crashes, wounds, sprains, and strains	0.1645
Ecuador	2005	Multiple fractures, dislocations, crashes, wounds, sprains, and strains	0.145

Ecuador	2010	Multiple fractures, dislocations, crashes, wounds, sprains, and strains	0.1317
Ecuador	2017	Multiple fractures, dislocations, crashes, wounds, sprains, and strains	0.1127
Ecuador	1990	Amputation of upper limb, unilateral	0.077
Ecuador	1995	Amputation of upper limb, unilateral	0.0701
Ecuador	2000	Amputation of upper limb, unilateral	0.0635
Ecuador	2005	Amputation of upper limb, unilateral	0.059
Ecuador	2010	Amputation of upper limb, unilateral	0.056
Ecuador	2017	Amputation of upper limb, unilateral	0.0516
Ecuador	1990	Amputation of thumb	0.0114
Ecuador	1995	Amputation of thumb	0.0114
Ecuador	2000	Amputation of thumb	0.0114
Ecuador	2005	Amputation of thumb	0.0114
Ecuador	2010	Amputation of thumb	0.0114
Ecuador	2017	Amputation of thumb	0.0114
Ecuador	1990	Amputation of toe/toes	0.0059
Ecuador	1995	Amputation of toe/toes	0.0059
Ecuador	2000	Amputation of toe/toes	0.0059
Ecuador	2005	Amputation of toe/toes	0.0059
Ecuador	2010	Amputation of toe/toes	0.0059
Ecuador	2017	Amputation of toe/toes	0.0059
Ecuador	1990	Burns, <20% total burned surface area without lower airway burns	0.0163
Ecuador	1995	Burns, <20% total burned surface area without lower airway burns	0.0163
Ecuador	2000	Burns, <20% total burned surface area without lower airway burns	0.0163
Ecuador	2005	Burns, <20% total burned surface area without lower airway burns	0.0163
Ecuador	2010	Burns, <20% total burned surface area without lower airway burns	0.0163
Ecuador	2017	Burns, <20% total burned surface area without lower airway burns	0.0163
Ecuador	1990	Burns, >=20% total burned surface area or >= 10% burned surface area if head/neck or hands/wrist involved w/o lower airway burns	0.2889
Ecuador	1995	Burns, >=20% total burned surface area or >= 10% burned surface area if head/neck or hands/wrist involved w/o lower airway burns	0.2608
Ecuador	2000	Burns, >=20% total burned surface area or >= 10% burned surface area if head/neck or hands/wrist involved w/o lower airway burns	0.2341
Ecuador	2005	Burns, >=20% total burned surface area or >= 10% burned surface area if head/neck or hands/wrist involved w/o lower airway burns	0.2159
Ecuador	2010	Burns, >=20% total burned surface area or >= 10% burned surface area if head/neck or hands/wrist involved w/o lower airway burns	0.2035
Ecuador	2017	Burns, >=20% total burned surface area or >= 10% burned surface area if head/neck or hands/wrist involved w/o lower airway burns	0.1858
Peru	1990	Amputation of lower limbs, bilateral	0.2461
Peru	1995	Amputation of lower limbs, bilateral	0.2315
Peru	2000	Amputation of lower limbs, bilateral	0.2043
Peru	2005	Amputation of lower limbs, bilateral	0.1857
Peru	2010	Amputation of lower limbs, bilateral	0.1761
Peru	2017	Amputation of lower limbs, bilateral	0.1574
Peru	1990	Lower airway burns	0.2252
Peru	1995	Lower airway burns	0.2252
Peru	2000	Lower airway burns	0.2252
Peru	2005	Lower airway burns	0.2252
Peru	2010	Lower airway burns	0.2252
Peru	2017	Lower airway burns	0.2252
Peru	1990	Dislocation of hip	0.0159
Peru	1995	Dislocation of hip	0.0159
Peru	2000	Dislocation of hip	0.0159
Peru	2005	Dislocation of hip	0.0159
Peru	2010	Dislocation of hip	0.0159
Peru	2017	Dislocation of hip	0.0159
Peru	1990	Dislocation of knee	0.1134
Peru	1995	Dislocation of knee	0.1134
Peru	2000	Dislocation of knee	0.1134
Peru	2005	Dislocation of knee	0.1134
Peru	2010	Dislocation of knee	0.1134
Peru	2017	Dislocation of knee	0.1134
Peru	1990	Dislocation of shoulder	0.062
Peru	1995	Dislocation of shoulder	0.062
Peru	2000	Dislocation of shoulder	0.062
Peru	2005	Dislocation of shoulder	0.062
Peru	2010	Dislocation of shoulder	0.062
Peru	2017	Dislocation of shoulder	0.062
Peru	1990	Muscle and tendon injuries, including sprains and strains lesser dislocations	0.0075
Peru	1995	Muscle and tendon injuries, including sprains and strains lesser dislocations	0.0075
Peru	2000	Muscle and tendon injuries, including sprains and strains lesser dislocations	0.0075
Peru	2005	Muscle and tendon injuries, including sprains and strains lesser dislocations	0.0075
Peru	2010	Muscle and tendon injuries, including sprains and strains lesser dislocations	0.0075
Peru	2017	Muscle and tendon injuries, including sprains and strains lesser dislocations	0.0075
Peru	1990	Fracture of clavicle, scapula, or humerus	0.0349
Peru	1995	Fracture of clavicle, scapula, or humerus	0.0349
Peru	2000	Fracture of clavicle, scapula, or humerus	0.0349
Peru	2005	Fracture of clavicle, scapula, or humerus	0.0349
Peru	2010	Fracture of clavicle, scapula, or humerus	0.0349
Peru	2017	Fracture of clavicle, scapula, or humerus	0.0349
Peru	1990	Fracture of face bones	0.0669
Peru	1995	Fracture of face bones	0.0669
Peru	2000	Fracture of face bones	0.0669
Peru	2005	Fracture of face bones	0.0669
Peru	2010	Fracture of face bones	0.0669
Peru	2017	Fracture of face bones	0.0669
Peru	1990	Fracture of foot bones except ankle	0.0264
Peru	1995	Fracture of foot bones except ankle	0.0264
Peru	2000	Fracture of foot bones except ankle	0.0264
Peru	2005	Fracture of foot bones except ankle	0.0264
Peru	2010	Fracture of foot bones except ankle	0.0264
Peru	2017	Fracture of foot bones except ankle	0.0264
Peru	1990	Fracture of hand (wrist and other distal part of hand)	0.0138
Peru	1995	Fracture of hand (wrist and other distal part of hand)	0.0138
Peru	2000	Fracture of hand (wrist and other distal part of hand)	0.0138
Peru	2005	Fracture of hand (wrist and other distal part of hand)	0.0138
Peru	2010	Fracture of hand (wrist and other distal part of hand)	0.0138
Peru	2017	Fracture of hand (wrist and other distal part of hand)	0.0138
Peru	1990	Fracture of hip	0.2202
Peru	1995	Fracture of hip	0.2008
Peru	2000	Fracture of hip	0.1649
Peru	2005	Fracture of hip	0.1403
Peru	2010	Fracture of hip	0.1276
Peru	2017	Fracture of hip	0.1028
Peru	1990	Amputation of upper limbs, bilateral	0.2554
Peru	1995	Amputation of upper limbs, bilateral	0.2354
Peru	2000	Amputation of upper limbs, bilateral	0.1983
Peru	2005	Amputation of upper limbs, bilateral	0.1729
Peru	2010	Amputation of upper limbs, bilateral	0.1599
Peru	2017	Amputation of upper limbs, bilateral	0.1343
Peru	1990	Fracture of patella, tibia or fibula, or ankle	0.0554
Peru	1995	Fracture of patella, tibia or fibula, or ankle	0.0554
Peru	2000	Fracture of patella, tibia or fibula, or ankle	0.0554
Peru	2005	Fracture of patella, tibia or fibula, or ankle	0.0554
Peru	2010	Fracture of patella, tibia or fibula, or ankle	0.0554
Peru	2017	Fracture of patella, tibia or fibula, or ankle	0.0554
Peru	1990	Fracture of pelvis	0.1823
Peru	1995	Fracture of pelvis	0.1823
Peru	2000	Fracture of pelvis	0.1823
Peru	2005	Fracture of pelvis	0.1823
Peru	2010	Fracture of pelvis	0.1823
Peru	2017	Fracture of pelvis	0.1823
Peru	1990	Fracture of radius and/or ulna	0.0435
Peru	1995	Fracture of radius and/or ulna	0.0435
Peru	2000	Fracture of radius and/or ulna	0.0435
Peru	2005	Fracture of radius and/or ulna	0.0435
Peru	2010	Fracture of radius and/or ulna	0.0435
Peru	2017	Fracture of radius and/or ulna	0.0435
Peru	1990	Fracture of skull	0.0714
Peru	1995	Fracture of skull	0.0714
Peru	2000	Fracture of skull	0.0714
Peru	2005	Fracture of skull	0.0714
Peru	2010	Fracture of skull	0.0714
Peru	2017	Fracture of skull	0.0714
Peru	1990	Fracture of sternum and/or fracture of one or more ribs	0.1027
Peru	1995	Fracture of sternum and/or fracture of one or more ribs	0.1027
Peru	2000	Fracture of sternum and/or fracture of one or more ribs	0.1027
Peru	2005	Fracture of sternum and/or fracture of one or more ribs	0.1027
Peru	2010	Fracture of sternum and/or fracture of one or more ribs	0.1027
Peru	2017	Fracture of sternum and/or fracture of one or more ribs	0.1027
Peru	1990	Fracture of vertebral column	0.1106
Peru	1995	Fracture of vertebral column	0.1106
Peru	2000	Fracture of vertebral column	0.1106
Peru	2005	Fracture of vertebral column	0.1106
Peru	2010	Fracture of vertebral column	0.1106
Peru	2017	Fracture of vertebral column	0.1106
Peru	1990	Fracture of femur, other than femoral neck	0.042
Peru	1995	Fracture of femur, other than femoral neck	0.042
Peru	2000	Fracture of femur, other than femoral neck	0.042
Peru	2005	Fracture of femur, other than femoral neck	0.042
Peru	2010	Fracture of femur, other than femoral neck	0.042
Peru	2017	Fracture of femur, other than femoral neck	0.042
Peru	1990	Minor TBI	0.1319
Peru	1995	Minor TBI	0.1319
Peru	2000	Minor TBI	0.1319
Peru	2005	Minor TBI	0.1319
Peru	2010	Minor TBI	0.1319
Peru	2017	Minor TBI	0.1319
Peru	1990	Moderate TBI	0.1637
Peru	1995	Moderate TBI	0.1637
Peru	2000	Moderate TBI	0.1637
Peru	2005	Moderate TBI	0.1637
Peru	2010	Moderate TBI	0.1637
Peru	2017	Moderate TBI	0.1637
Peru	1990	Amputation of fingers (excluding thumb)	0.0047
Peru	1995	Amputation of fingers (excluding thumb)	0.0047
Peru	2000	Amputation of fingers (excluding thumb)	0.0047
Peru	2005	Amputation of fingers (excluding thumb)	0.0047
Peru	2010	Amputation of fingers (excluding thumb)	0.0047
Peru	2017	Amputation of fingers (excluding thumb)	0.0047
Peru	1990	Spinal cord lesion at neck level	0.468
Peru	1995	Spinal cord lesion at neck level	0.4613
Peru	2000	Spinal cord lesion at neck level	0.4489
Peru	2005	Spinal cord lesion at neck level	0.4404
Peru	2010	Spinal cord lesion at neck level	0.436
Peru	2017	Spinal cord lesion at neck level	0.4274
Peru	1990	Spinal cord lesion below neck level	0.2963
Peru	1995	Spinal cord lesion below neck level	0.2821
Peru	2000	Spinal cord lesion below neck level	0.2559
Peru	2005	Spinal cord lesion below neck level	0.238
Peru	2010	Spinal cord lesion below neck level	0.2287
Peru	2017	Spinal cord lesion below neck level	0.2106
Peru	1990	Drowning and nonfatal submersion	0.2471
Peru	1995	Drowning and nonfatal submersion	0.2471
Peru	2000	Drowning and nonfatal submersion	0.2471
Peru	2005	Drowning and nonfatal submersion	0.2471
Peru	2010	Drowning and nonfatal submersion	0.2471
Peru	2017	Drowning and nonfatal submersion	0.2471
Peru	1990	Asphyxiation	0.2471
Peru	1995	Asphyxiation	0.2471
Peru	2000	Asphyxiation	0.2471
Peru	2005	Asphyxiation	0.2471
Peru	2010	Asphyxiation	0.2471
Peru	2017	Asphyxiation	0.2471
Peru	1990	Crush injury	0.1325
Peru	1995	Crush injury	0.1325
Peru	2000	Crush injury	0.1325
Peru	2005	Crush injury	0.1325
Peru	2010	Crush injury	0.1325
Peru	2017	Crush injury	0.1325
Peru	1990	Nerve injury	0.1129
Peru	1995	Nerve injury	0.1129
Peru	2000	Nerve injury	0.1129
Peru	2005	Nerve injury	0.1129
Peru	2010	Nerve injury	0.1129
Peru	2017	Nerve injury	0.1129
Peru	1990	Injury to eyes	0.0165
Peru	1995	Injury to eyes	0.0165
Peru	2000	Injury to eyes	0.0165
Peru	2005	Injury to eyes	0.0165
Peru	2010	Injury to eyes	0.0165
Peru	2017	Injury to eyes	0.0165
Peru	1990	Amputation of lower limb, unilateral	0.1023
Peru	1995	Amputation of lower limb, unilateral	0.0948
Peru	2000	Amputation of lower limb, unilateral	0.0807
Peru	2005	Amputation of lower limb, unilateral	0.0712
Peru	2010	Amputation of lower limb, unilateral	0.0662
Peru	2017	Amputation of lower limb, unilateral	0.0566
Peru	1990	Open wound(s)	0.011
Peru	1995	Open wound(s)	0.011
Peru	2000	Open wound(s)	0.011
Peru	2005	Open wound(s)	0.011
Peru	2010	Open wound(s)	0.011
Peru	2017	Open wound(s)	0.011
Peru	1990	Poisoning requiring urgent care	0.1628
Peru	1995	Poisoning requiring urgent care	0.1628
Peru	2000	Poisoning requiring urgent care	0.1628
Peru	2005	Poisoning requiring urgent care	0.1628
Peru	2010	Poisoning requiring urgent care	0.1628
Peru	2017	Poisoning requiring urgent care	0.1628
Peru	1990	Severe chest injury	0.0474
Peru	1995	Severe chest injury	0.0474
Peru	2000	Severe chest injury	0.0474
Peru	2005	Severe chest injury	0.0474
Peru	2010	Severe chest injury	0.0474
Peru	2017	Severe chest injury	0.0474
Peru	1990	Internal hemorrhage in abdomen and pelvis	0.1143
Peru	1995	Internal hemorrhage in abdomen and pelvis	0.1143
Peru	2000	Internal hemorrhage in abdomen and pelvis	0.1143
Peru	2005	Internal hemorrhage in abdomen and pelvis	0.1143
Peru	2010	Internal hemorrhage in abdomen and pelvis	0.1143
Peru	2017	Internal hemorrhage in abdomen and pelvis	0.1143
Peru	1990	Contusion in any part of the body	0.0075
Peru	1995	Contusion in any part of the body	0.0075
Peru	2000	Contusion in any part of the body	0.0075
Peru	2005	Contusion in any part of the body	0.0075
Peru	2010	Contusion in any part of the body	0.0075
Peru	2017	Contusion in any part of the body	0.0075
Peru	1990	Effect of different environmental factors	0.049
Peru	1995	Effect of different environmental factors	0.049
Peru	2000	Effect of different environmental factors	0.049
Peru	2005	Effect of different environmental factors	0.049
Peru	2010	Effect of different environmental factors	0.049
Peru	2017	Effect of different environmental factors	0.049
Peru	1990	Complications following therapeutic procedures	0.0506
Peru	1995	Complications following therapeutic procedures	0.0506
Peru	2000	Complications following therapeutic procedures	0.0506
Peru	2005	Complications following therapeutic procedures	0.0506
Peru	2010	Complications following therapeutic procedures	0.0506
Peru	2017	Complications following therapeutic procedures	0.0506
Peru	1990	Multiple fractures, dislocations, crashes, wounds, sprains, and strains	0.2202
Peru	1995	Multiple fractures, dislocations, crashes, wounds, sprains, and strains	0.2008
Peru	2000	Multiple fractures, dislocations, crashes, wounds, sprains, and strains	0.1649
Peru	2005	Multiple fractures, dislocations, crashes, wounds, sprains, and strains	0.1403
Peru	2010	Multiple fractures, dislocations, crashes, wounds, sprains, and strains	0.1276
Peru	2017	Multiple fractures, dislocations, crashes, wounds, sprains, and strains	0.1028
Peru	1990	Amputation of upper limb, unilateral	0.0763
Peru	1995	Amputation of upper limb, unilateral	0.0718
Peru	2000	Amputation of upper limb, unilateral	0.0636
Peru	2005	Amputation of upper limb, unilateral	0.0579
Peru	2010	Amputation of upper limb, unilateral	0.055
Peru	2017	Amputation of upper limb, unilateral	0.0493
Peru	1990	Amputation of thumb	0.0114
Peru	1995	Amputation of thumb	0.0114
Peru	2000	Amputation of thumb	0.0114
Peru	2005	Amputation of thumb	0.0114
Peru	2010	Amputation of thumb	0.0114
Peru	2017	Amputation of thumb	0.0114
Peru	1990	Amputation of toe/toes	0.0059
Peru	1995	Amputation of toe/toes	0.0059
Peru	2000	Amputation of toe/toes	0.0059
Peru	2005	Amputation of toe/toes	0.0059
Peru	2010	Amputation of toe/toes	0.0059
Peru	2017	Amputation of toe/toes	0.0059
Peru	1990	Burns, <20% total burned surface area without lower airway burns	0.0163
Peru	1995	Burns, <20% total burned surface area without lower airway burns	0.0163
Peru	2000	Burns, <20% total burned surface area without lower airway burns	0.0163
Peru	2005	Burns, <20% total burned surface area without lower airway burns	0.0163
Peru	2010	Burns, <20% total burned surface area without lower airway burns	0.0163
Peru	2017	Burns, <20% total burned surface area without lower airway burns	0.0163
Peru	1990	Burns, >=20% total burned surface area or >= 10% burned surface area if head/neck or hands/wrist involved w/o lower airway burns	0.2859
Peru	1995	Burns, >=20% total burned surface area or >= 10% burned surface area if head/neck or hands/wrist involved w/o lower airway burns	0.2679
Peru	2000	Burns, >=20% total burned surface area or >= 10% burned surface area if head/neck or hands/wrist involved w/o lower airway burns	0.2344
Peru	2005	Burns, >=20% total burned surface area or >= 10% burned surface area if head/neck or hands/wrist involved w/o lower airway burns	0.2115
Peru	2010	Burns, >=20% total burned surface area or >= 10% burned surface area if head/neck or hands/wrist involved w/o lower airway burns	0.1997
Peru	2017	Burns, >=20% total burned surface area or >= 10% burned surface area if head/neck or hands/wrist involved w/o lower airway burns	0.1766
Colombia	1990	Amputation of lower limbs, bilateral	0.2124
Colombia	1995	Amputation of lower limbs, bilateral	0.1943
Colombia	2000	Amputation of lower limbs, bilateral	0.1815
Colombia	2005	Amputation of lower limbs, bilateral	0.1718
Colombia	2010	Amputation of lower limbs, bilateral	0.1602
Colombia	2017	Amputation of lower limbs, bilateral	0.1434
Colombia	1990	Lower airway burns	0.2252
Colombia	1995	Lower airway burns	0.2252
Colombia	2000	Lower airway burns	0.2252
Colombia	2005	Lower airway burns	0.2252
Colombia	2010	Lower airway burns	0.2252
Colombia	2017	Lower airway burns	0.2252
Colombia	1990	Dislocation of hip	0.0159
Colombia	1995	Dislocation of hip	0.0159
Colombia	2000	Dislocation of hip	0.0159
Colombia	2005	Dislocation of hip	0.0159
Colombia	2010	Dislocation of hip	0.0159
Colombia	2017	Dislocation of hip	0.0159
Colombia	1990	Dislocation of knee	0.1134
Colombia	1995	Dislocation of knee	0.1134
Colombia	2000	Dislocation of knee	0.1134
Colombia	2005	Dislocation of knee	0.1134
Colombia	2010	Dislocation of knee	0.1134
Colombia	2017	Dislocation of knee	0.1134
Colombia	1990	Dislocation of shoulder	0.062
Colombia	1995	Dislocation of shoulder	0.062
Colombia	2000	Dislocation of shoulder	0.062
Colombia	2005	Dislocation of shoulder	0.062
Colombia	2010	Dislocation of shoulder	0.062
Colombia	2017	Dislocation of shoulder	0.062
Colombia	1990	Muscle and tendon injuries, including sprains and strains lesser dislocations	0.0075
Colombia	1995	Muscle and tendon injuries, including sprains and strains lesser dislocations	0.0075
Colombia	2000	Muscle and tendon injuries, including sprains and strains lesser dislocations	0.0075
Colombia	2005	Muscle and tendon injuries, including sprains and strains lesser dislocations	0.0075
Colombia	2010	Muscle and tendon injuries, including sprains and strains lesser dislocations	0.0075
Colombia	2017	Muscle and tendon injuries, including sprains and strains lesser dislocations	0.0075
Colombia	1990	Fracture of clavicle, scapula, or humerus	0.0349
Colombia	1995	Fracture of clavicle, scapula, or humerus	0.0349
Colombia	2000	Fracture of clavicle, scapula, or humerus	0.0349
Colombia	2005	Fracture of clavicle, scapula, or humerus	0.0349
Colombia	2010	Fracture of clavicle, scapula, or humerus	0.0349
Colombia	2017	Fracture of clavicle, scapula, or humerus	0.0349
Colombia	1990	Fracture of face bones	0.0669
Colombia	1995	Fracture of face bones	0.0669
Colombia	2000	Fracture of face bones	0.0669
Colombia	2005	Fracture of face bones	0.0669
Colombia	2010	Fracture of face bones	0.0669
Colombia	2017	Fracture of face bones	0.0669
Colombia	1990	Fracture of foot bones except ankle	0.0264
Colombia	1995	Fracture of foot bones except ankle	0.0264
Colombia	2000	Fracture of foot bones except ankle	0.0264
Colombia	2005	Fracture of foot bones except ankle	0.0264
Colombia	2010	Fracture of foot bones except ankle	0.0264
Colombia	2017	Fracture of foot bones except ankle	0.0264
Colombia	1990	Fracture of hand (wrist and other distal part of hand)	0.0138
Colombia	1995	Fracture of hand (wrist and other distal part of hand)	0.0138
Colombia	2000	Fracture of hand (wrist and other distal part of hand)	0.0138
Colombia	2005	Fracture of hand (wrist and other distal part of hand)	0.0138
Colombia	2010	Fracture of hand (wrist and other distal part of hand)	0.0138
Colombia	2017	Fracture of hand (wrist and other distal part of hand)	0.0138
Colombia	1990	Fracture of hip	0.1755
Colombia	1995	Fracture of hip	0.1517
Colombia	2000	Fracture of hip	0.1347
Colombia	2005	Fracture of hip	0.1218
Colombia	2010	Fracture of hip	0.1066
Colombia	2017	Fracture of hip	0.0844
Colombia	1990	Amputation of upper limbs, bilateral	0.2093
Colombia	1995	Amputation of upper limbs, bilateral	0.1847
Colombia	2000	Amputation of upper limbs, bilateral	0.1672
Colombia	2005	Amputation of upper limbs, bilateral	0.1539
Colombia	2010	Amputation of upper limbs, bilateral	0.1381
Colombia	2017	Amputation of upper limbs, bilateral	0.1152
Colombia	1990	Fracture of patella, tibia or fibula, or ankle	0.0554
Colombia	1995	Fracture of patella, tibia or fibula, or ankle	0.0554
Colombia	2000	Fracture of patella, tibia or fibula, or ankle	0.0554
Colombia	2005	Fracture of patella, tibia or fibula, or ankle	0.0554
Colombia	2010	Fracture of patella, tibia or fibula, or ankle	0.0554
Colombia	2017	Fracture of patella, tibia or fibula, or ankle	0.0554
Colombia	1990	Fracture of pelvis	0.1823
Colombia	1995	Fracture of pelvis	0.1823
Colombia	2000	Fracture of pelvis	0.1823
Colombia	2005	Fracture of pelvis	0.1823
Colombia	2010	Fracture of pelvis	0.1823
Colombia	2017	Fracture of pelvis	0.1823
Colombia	1990	Fracture of radius and/or ulna	0.0435
Colombia	1995	Fracture of radius and/or ulna	0.0435
Colombia	2000	Fracture of radius and/or ulna	0.0435
Colombia	2005	Fracture of radius and/or ulna	0.0435
Colombia	2010	Fracture of radius and/or ulna	0.0435
Colombia	2017	Fracture of radius and/or ulna	0.0435
Colombia	1990	Fracture of skull	0.0714
Colombia	1995	Fracture of skull	0.0714
Colombia	2000	Fracture of skull	0.0714
Colombia	2005	Fracture of skull	0.0714
Colombia	2010	Fracture of skull	0.0714
Colombia	2017	Fracture of skull	0.0714
Colombia	1990	Fracture of sternum and/or fracture of one or more ribs	0.1027
Colombia	1995	Fracture of sternum and/or fracture of one or more ribs	0.1027
Colombia	2000	Fracture of sternum and/or fracture of one or more ribs	0.1027
Colombia	2005	Fracture of sternum and/or fracture of one or more ribs	0.1027
Colombia	2010	Fracture of sternum and/or fracture of one or more ribs	0.1027
Colombia	2017	Fracture of sternum and/or fracture of one or more ribs	0.1027
Colombia	1990	Fracture of vertebral column	0.1106
Colombia	1995	Fracture of vertebral column	0.1106
Colombia	2000	Fracture of vertebral column	0.1106
Colombia	2005	Fracture of vertebral column	0.1106
Colombia	2010	Fracture of vertebral column	0.1106
Colombia	2017	Fracture of vertebral column	0.1106
Colombia	1990	Fracture of femur, other than femoral neck	0.042
Colombia	1995	Fracture of femur, other than femoral neck	0.042
Colombia	2000	Fracture of femur, other than femoral neck	0.042
Colombia	2005	Fracture of femur, other than femoral neck	0.042
Colombia	2010	Fracture of femur, other than femoral neck	0.042
Colombia	2017	Fracture of femur, other than femoral neck	0.042
Colombia	1990	Minor TBI	0.1319
Colombia	1995	Minor TBI	0.1319
Colombia	2000	Minor TBI	0.1319
Colombia	2005	Minor TBI	0.1319
Colombia	2010	Minor TBI	0.1319
Colombia	2017	Minor TBI	0.1319
Colombia	1990	Moderate TBI	0.1637
Colombia	1995	Moderate TBI	0.1637
Colombia	2000	Moderate TBI	0.1637
Colombia	2005	Moderate TBI	0.1637
Colombia	2010	Moderate TBI	0.1637
Colombia	2017	Moderate TBI	0.1637
Colombia	1990	Amputation of fingers (excluding thumb)	0.0047
Colombia	1995	Amputation of fingers (excluding thumb)	0.0047
Colombia	2000	Amputation of fingers (excluding thumb)	0.0047
Colombia	2005	Amputation of fingers (excluding thumb)	0.0047
Colombia	2010	Amputation of fingers (excluding thumb)	0.0047
Colombia	2017	Amputation of fingers (excluding thumb)	0.0047
Colombia	1990	Spinal cord lesion at neck level	0.4526
Colombia	1995	Spinal cord lesion at neck level	0.4443
Colombia	2000	Spinal cord lesion at neck level	0.4385
Colombia	2005	Spinal cord lesion at neck level	0.434
Colombia	2010	Spinal cord lesion at neck level	0.4287
Colombia	2017	Spinal cord lesion at neck level	0.421
Colombia	1990	Spinal cord lesion below neck level	0.2637
Colombia	1995	Spinal cord lesion below neck level	0.2463
Colombia	2000	Spinal cord lesion below neck level	0.2339

Colombia	2005	Spinal cord lesion below neck level	0.2245
Colombia	2010	Spinal cord lesion below neck level	0.2134
Colombia	2017	Spinal cord lesion below neck level	0.1971
Colombia	1990	Drowning and nonfatal submersion	0.2471
Colombia	1995	Drowning and nonfatal submersion	0.2471
Colombia	2000	Drowning and nonfatal submersion	0.2471
Colombia	2005	Drowning and nonfatal submersion	0.2471
Colombia	2010	Drowning and nonfatal submersion	0.2471
Colombia	2017	Drowning and nonfatal submersion	0.2471
Colombia	1990	Asphyxiation	0.2471
Colombia	1995	Asphyxiation	0.2471
Colombia	2000	Asphyxiation	0.2471
Colombia	2005	Asphyxiation	0.2471
Colombia	2010	Asphyxiation	0.2471
Colombia	2017	Asphyxiation	0.2471
Colombia	1990	Crush injury	0.1325
Colombia	1995	Crush injury	0.1325
Colombia	2000	Crush injury	0.1325
Colombia	2005	Crush injury	0.1325
Colombia	2010	Crush injury	0.1325
Colombia	2017	Crush injury	0.1325
Colombia	1990	Nerve injury	0.1129
Colombia	1995	Nerve injury	0.1129
Colombia	2000	Nerve injury	0.1129
Colombia	2005	Nerve injury	0.1129
Colombia	2010	Nerve injury	0.1129
Colombia	2017	Nerve injury	0.1129
Colombia	1990	Injury to eyes	0.0165
Colombia	1995	Injury to eyes	0.0165
Colombia	2000	Injury to eyes	0.0165
Colombia	2005	Injury to eyes	0.0165
Colombia	2010	Injury to eyes	0.0165
Colombia	2017	Injury to eyes	0.0165
Colombia	1990	Amputation of lower limb, unilateral	0.0849
Colombia	1995	Amputation of lower limb, unilateral	0.0756
Colombia	2000	Amputation of lower limb, unilateral	0.069
Colombia	2005	Amputation of lower limb, unilateral	0.064
Colombia	2010	Amputation of lower limb, unilateral	0.058
Colombia	2017	Amputation of lower limb, unilateral	0.0494
Colombia	1990	Open wound(s)	0.011
Colombia	1995	Open wound(s)	0.011
Colombia	2000	Open wound(s)	0.011
Colombia	2005	Open wound(s)	0.011
Colombia	2010	Open wound(s)	0.011
Colombia	2017	Open wound(s)	0.011
Colombia	1990	Poisoning requiring urgent care	0.1628
Colombia	1995	Poisoning requiring urgent care	0.1628
Colombia	2000	Poisoning requiring urgent care	0.1628
Colombia	2005	Poisoning requiring urgent care	0.1628
Colombia	2010	Poisoning requiring urgent care	0.1628
Colombia	2017	Poisoning requiring urgent care	0.1628
Colombia	1990	Severe chest injury	0.0474
Colombia	1995	Severe chest injury	0.0474
Colombia	2000	Severe chest injury	0.0474
Colombia	2005	Severe chest injury	0.0474
Colombia	2010	Severe chest injury	0.0474
Colombia	2017	Severe chest injury	0.0474
Colombia	1990	Internal hemorrhage in abdomen and pelvis	0.1143
Colombia	1995	Internal hemorrhage in abdomen and pelvis	0.1143
Colombia	2000	Internal hemorrhage in abdomen and pelvis	0.1143
Colombia	2005	Internal hemorrhage in abdomen and pelvis	0.1143
Colombia	2010	Internal hemorrhage in abdomen and pelvis	0.1143
Colombia	2017	Internal hemorrhage in abdomen and pelvis	0.1143
Colombia	1990	Contusion in any part of the body	0.0075
Colombia	1995	Contusion in any part of the body	0.0075
Colombia	2000	Contusion in any part of the body	0.0075
Colombia	2005	Contusion in any part of the body	0.0075
Colombia	2010	Contusion in any part of the body	0.0075
Colombia	2017	Contusion in any part of the body	0.0075
Colombia	1990	Effect of different environmental factors	0.049
Colombia	1995	Effect of different environmental factors	0.049
Colombia	2000	Effect of different environmental factors	0.049
Colombia	2005	Effect of different environmental factors	0.049
Colombia	2010	Effect of different environmental factors	0.049
Colombia	2017	Effect of different environmental factors	0.049
Colombia	1990	Complications following therapeutic procedures	0.0506
Colombia	1995	Complications following therapeutic procedures	0.0506
Colombia	2000	Complications following therapeutic procedures	0.0506
Colombia	2005	Complications following therapeutic procedures	0.0506
Colombia	2010	Complications following therapeutic procedures	0.0506
Colombia	2017	Complications following therapeutic procedures	0.0506
Colombia	1990	Multiple fractures, dislocations, crashes, wounds, sprains, and strains	0.1755
Colombia	1995	Multiple fractures, dislocations, crashes, wounds, sprains, and strains	0.1517
Colombia	2000	Multiple fractures, dislocations, crashes, wounds, sprains, and strains	0.1347
Colombia	2005	Multiple fractures, dislocations, crashes, wounds, sprains, and strains	0.1218
Colombia	2010	Multiple fractures, dislocations, crashes, wounds, sprains, and strains	0.1066
Colombia	2017	Multiple fractures, dislocations, crashes, wounds, sprains, and strains	0.0844
Colombia	1990	Amputation of upper limb, unilateral	0.066
Colombia	1995	Amputation of upper limb, unilateral	0.0606
Colombia	2000	Amputation of upper limb, unilateral	0.0567
Colombia	2005	Amputation of upper limb, unilateral	0.0537
Colombia	2010	Amputation of upper limb, unilateral	0.0502
Colombia	2017	Amputation of upper limb, unilateral	0.0451
Colombia	1990	Amputation of thumb	0.0114
Colombia	1995	Amputation of thumb	0.0114
Colombia	2000	Amputation of thumb	0.0114
Colombia	2005	Amputation of thumb	0.0114
Colombia	2010	Amputation of thumb	0.0114
Colombia	2017	Amputation of thumb	0.0114
Colombia	1990	Amputation of toe/toes	0.0059
Colombia	1995	Amputation of toe/toes	0.0059
Colombia	2000	Amputation of toe/toes	0.0059
Colombia	2005	Amputation of toe/toes	0.0059
Colombia	2010	Amputation of toe/toes	0.0059
Colombia	2017	Amputation of toe/toes	0.0059
Colombia	1990	Burns, <20% total burned surface area without lower airway burns	0.0163
Colombia	1995	Burns, <20% total burned surface area without lower airway burns	0.0163
Colombia	2000	Burns, <20% total burned surface area without lower airway burns	0.0163
Colombia	2005	Burns, <20% total burned surface area without lower airway burns	0.0163
Colombia	2010	Burns, <20% total burned surface area without lower airway burns	0.0163
Colombia	2017	Burns, <20% total burned surface area without lower airway burns	0.0163
Colombia	1990	Burns, >=20% total burned surface area or >= 10% burned surface area if head/neck or hands/wrist involved w/o lower airway burns	0.2443
Colombia	1995	Burns, >=20% total burned surface area or >= 10% burned surface area if head/neck or hands/wrist involved w/o lower airway burns	0.2221
Colombia	2000	Burns, >=20% total burned surface area or >= 10% burned surface area if head/neck or hands/wrist involved w/o lower airway burns	0.2063
Colombia	2005	Burns, >=20% total burned surface area or >= 10% burned surface area if head/neck or hands/wrist involved w/o lower airway burns	0.1943
Colombia	2010	Burns, >=20% total burned surface area or >= 10% burned surface area if head/neck or hands/wrist involved w/o lower airway burns	0.1801
Colombia	2017	Burns, >=20% total burned surface area or >= 10% burned surface area if head/neck or hands/wrist involved w/o lower airway burns	0.1594
Costa Rica	1990	Amputation of lower limbs, bilateral	0.1716
Costa Rica	1995	Amputation of lower limbs, bilateral	0.1656
Costa Rica	2000	Amputation of lower limbs, bilateral	0.1578
Costa Rica	2005	Amputation of lower limbs, bilateral	0.1472
Costa Rica	2010	Amputation of lower limbs, bilateral	0.1377
Costa Rica	2017	Amputation of lower limbs, bilateral	0.1267
Costa Rica	1990	Lower airway burns	0.2252
Costa Rica	1995	Lower airway burns	0.2252
Costa Rica	2000	Lower airway burns	0.2252
Costa Rica	2005	Lower airway burns	0.2252
Costa Rica	2010	Lower airway burns	0.2252
Costa Rica	2017	Lower airway burns	0.2252
Costa Rica	1990	Dislocation of hip	0.0159
Costa Rica	1995	Dislocation of hip	0.0159
Costa Rica	2000	Dislocation of hip	0.0159
Costa Rica	2005	Dislocation of hip	0.0159
Costa Rica	2010	Dislocation of hip	0.0159
Costa Rica	2017	Dislocation of hip	0.0159
Costa Rica	1990	Dislocation of knee	0.1134
Costa Rica	1995	Dislocation of knee	0.1134
Costa Rica	2000	Dislocation of knee	0.1134
Costa Rica	2005	Dislocation of knee	0.1134
Costa Rica	2010	Dislocation of knee	0.1134
Costa Rica	2017	Dislocation of knee	0.1134
Costa Rica	1990	Dislocation of shoulder	0.062
Costa Rica	1995	Dislocation of shoulder	0.062
Costa Rica	2000	Dislocation of shoulder	0.062
Costa Rica	2005	Dislocation of shoulder	0.062
Costa Rica	2010	Dislocation of shoulder	0.062
Costa Rica	2017	Dislocation of shoulder	0.062
Costa Rica	1990	Muscle and tendon injuries, including sprains and strains lesser dislocations	0.0075
Costa Rica	1995	Muscle and tendon injuries, including sprains and strains lesser dislocations	0.0075
Costa Rica	2000	Muscle and tendon injuries, including sprains and strains lesser dislocations	0.0075
Costa Rica	2005	Muscle and tendon injuries, including sprains and strains lesser dislocations	0.0075
Costa Rica	2010	Muscle and tendon injuries, including sprains and strains lesser dislocations	0.0075
Costa Rica	2017	Muscle and tendon injuries, including sprains and strains lesser dislocations	0.0075
Costa Rica	1990	Fracture of clavicle, scapula, or humerus	0.0349
Costa Rica	1995	Fracture of clavicle, scapula, or humerus	0.0349
Costa Rica	2000	Fracture of clavicle, scapula, or humerus	0.0349
Costa Rica	2005	Fracture of clavicle, scapula, or humerus	0.0349
Costa Rica	2010	Fracture of clavicle, scapula, or humerus	0.0349
Costa Rica	2017	Fracture of clavicle, scapula, or humerus	0.0349
Costa Rica	1990	Fracture of face bones	0.0669
Costa Rica	1995	Fracture of face bones	0.0669
Costa Rica	2000	Fracture of face bones	0.0669
Costa Rica	2005	Fracture of face bones	0.0669
Costa Rica	2010	Fracture of face bones	0.0669
Costa Rica	2017	Fracture of face bones	0.0669
Costa Rica	1990	Fracture of foot bones except ankle	0.0264
Costa Rica	1995	Fracture of foot bones except ankle	0.0264
Costa Rica	2000	Fracture of foot bones except ankle	0.0264
Costa Rica	2005	Fracture of foot bones except ankle	0.0264
Costa Rica	2010	Fracture of foot bones except ankle	0.0264
Costa Rica	2017	Fracture of foot bones except ankle	0.0264
Costa Rica	1990	Fracture of hand (wrist and other distal part of hand)	0.0138
Costa Rica	1995	Fracture of hand (wrist and other distal part of hand)	0.0138
Costa Rica	2000	Fracture of hand (wrist and other distal part of hand)	0.0138
Costa Rica	2005	Fracture of hand (wrist and other distal part of hand)	0.0138
Costa Rica	2010	Fracture of hand (wrist and other distal part of hand)	0.0138
Costa Rica	2017	Fracture of hand (wrist and other distal part of hand)	0.0138
Costa Rica	1990	Fracture of hip	0.1216
Costa Rica	1995	Fracture of hip	0.1137
Costa Rica	2000	Fracture of hip	0.1034
Costa Rica	2005	Fracture of hip	0.0893
Costa Rica	2010	Fracture of hip	0.0767
Costa Rica	2017	Fracture of hip	0.0622
Costa Rica	1990	Amputation of upper limbs, bilateral	0.1536
Costa Rica	1995	Amputation of upper limbs, bilateral	0.1455
Costa Rica	2000	Amputation of upper limbs, bilateral	0.1349
Costa Rica	2005	Amputation of upper limbs, bilateral	0.1203
Costa Rica	2010	Amputation of upper limbs, bilateral	0.1073
Costa Rica	2017	Amputation of upper limbs, bilateral	0.0923
Costa Rica	1990	Fracture of patella, tibia or fibula, or ankle	0.0554
Costa Rica	1995	Fracture of patella, tibia or fibula, or ankle	0.0554
Costa Rica	2000	Fracture of patella, tibia or fibula, or ankle	0.0554
Costa Rica	2005	Fracture of patella, tibia or fibula, or ankle	0.0554
Costa Rica	2010	Fracture of patella, tibia or fibula, or ankle	0.0554
Costa Rica	2017	Fracture of patella, tibia or fibula, or ankle	0.0554
Costa Rica	1990	Fracture of pelvis	0.1823
Costa Rica	1995	Fracture of pelvis	0.1823
Costa Rica	2000	Fracture of pelvis	0.1823
Costa Rica	2005	Fracture of pelvis	0.1823
Costa Rica	2010	Fracture of pelvis	0.1823
Costa Rica	2017	Fracture of pelvis	0.1823
Costa Rica	1990	Fracture of radius and/or ulna	0.0435
Costa Rica	1995	Fracture of radius and/or ulna	0.0435
Costa Rica	2000	Fracture of radius and/or ulna	0.0435
Costa Rica	2005	Fracture of radius and/or ulna	0.0435
Costa Rica	2010	Fracture of radius and/or ulna	0.0435
Costa Rica	2017	Fracture of radius and/or ulna	0.0435
Costa Rica	1990	Fracture of skull	0.0714
Costa Rica	1995	Fracture of skull	0.0714
Costa Rica	2000	Fracture of skull	0.0714
Costa Rica	2005	Fracture of skull	0.0714
Costa Rica	2010	Fracture of skull	0.0714
Costa Rica	2017	Fracture of skull	0.0714
Costa Rica	1990	Fracture of sternum and/or fracture of one or more ribs	0.1027
Costa Rica	1995	Fracture of sternum and/or fracture of one or more ribs	0.1027
Costa Rica	2000	Fracture of sternum and/or fracture of one or more ribs	0.1027
Costa Rica	2005	Fracture of sternum and/or fracture of one or more ribs	0.1027
Costa Rica	2010	Fracture of sternum and/or fracture of one or more ribs	0.1027
Costa Rica	2017	Fracture of sternum and/or fracture of one or more ribs	0.1027
Costa Rica	1990	Fracture of vertebral column	0.1106
Costa Rica	1995	Fracture of vertebral column	0.1106
Costa Rica	2000	Fracture of vertebral column	0.1106
Costa Rica	2005	Fracture of vertebral column	0.1106
Costa Rica	2010	Fracture of vertebral column	0.1106
Costa Rica	2017	Fracture of vertebral column	0.1106
Costa Rica	1990	Fracture of femur, other than femoral neck	0.042
Costa Rica	1995	Fracture of femur, other than femoral neck	0.042
Costa Rica	2000	Fracture of femur, other than femoral neck	0.042
Costa Rica	2005	Fracture of femur, other than femoral neck	0.042
Costa Rica	2010	Fracture of femur, other than femoral neck	0.042
Costa Rica	2017	Fracture of femur, other than femoral neck	0.042
Costa Rica	1990	Minor TBI	0.1319
Costa Rica	1995	Minor TBI	0.1319
Costa Rica	2000	Minor TBI	0.1319
Costa Rica	2005	Minor TBI	0.1319
Costa Rica	2010	Minor TBI	0.1319
Costa Rica	2017	Minor TBI	0.1319
Costa Rica	1990	Moderate TBI	0.1637
Costa Rica	1995	Moderate TBI	0.1637
Costa Rica	2000	Moderate TBI	0.1637
Costa Rica	2005	Moderate TBI	0.1637
Costa Rica	2010	Moderate TBI	0.1637
Costa Rica	2017	Moderate TBI	0.1637
Costa Rica	1990	Amputation of fingers (excluding thumb)	0.0047
Costa Rica	1995	Amputation of fingers (excluding thumb)	0.0047
Costa Rica	2000	Amputation of fingers (excluding thumb)	0.0047
Costa Rica	2005	Amputation of fingers (excluding thumb)	0.0047
Costa Rica	2010	Amputation of fingers (excluding thumb)	0.0047
Costa Rica	2017	Amputation of fingers (excluding thumb)	0.0047
Costa Rica	1990	Spinal cord lesion at neck level	0.4339
Costa Rica	1995	Spinal cord lesion at neck level	0.4312
Costa Rica	2000	Spinal cord lesion at neck level	0.4276
Costa Rica	2005	Spinal cord lesion at neck level	0.4228
Costa Rica	2010	Spinal cord lesion at neck level	0.4184
Costa Rica	2017	Spinal cord lesion at neck level	0.4134
Costa Rica	1990	Spinal cord lesion below neck level	0.2243
Costa Rica	1995	Spinal cord lesion below neck level	0.2186
Costa Rica	2000	Spinal cord lesion below neck level	0.211
Costa Rica	2005	Spinal cord lesion below neck level	0.2007
Costa Rica	2010	Spinal cord lesion below neck level	0.1916
Costa Rica	2017	Spinal cord lesion below neck level	0.181
Costa Rica	1990	Drowning and nonfatal submersion	0.2471
Costa Rica	1995	Drowning and nonfatal submersion	0.2471
Costa Rica	2000	Drowning and nonfatal submersion	0.2471
Costa Rica	2005	Drowning and nonfatal submersion	0.2471
Costa Rica	2010	Drowning and nonfatal submersion	0.2471
Costa Rica	2017	Drowning and nonfatal submersion	0.2471
Costa Rica	1990	Asphyxiation	0.2471
Costa Rica	1995	Asphyxiation	0.2471
Costa Rica	2000	Asphyxiation	0.2471
Costa Rica	2005	Asphyxiation	0.2471
Costa Rica	2010	Asphyxiation	0.2471
Costa Rica	2017	Asphyxiation	0.2471
Costa Rica	1990	Crush injury	0.1325
Costa Rica	1995	Crush injury	0.1325
Costa Rica	2000	Crush injury	0.1325
Costa Rica	2005	Crush injury	0.1325
Costa Rica	2010	Crush injury	0.1325
Costa Rica	2017	Crush injury	0.1325
Costa Rica	1990	Nerve injury	0.1129
Costa Rica	1995	Nerve injury	0.1129
Costa Rica	2000	Nerve injury	0.1129
Costa Rica	2005	Nerve injury	0.1129
Costa Rica	2010	Nerve injury	0.1129
Costa Rica	2017	Nerve injury	0.1129
Costa Rica	1990	Injury to eyes	0.0165
Costa Rica	1995	Injury to eyes	0.0165
Costa Rica	2000	Injury to eyes	0.0165
Costa Rica	2005	Injury to eyes	0.0165
Costa Rica	2010	Injury to eyes	0.0165
Costa Rica	2017	Injury to eyes	0.0165
Costa Rica	1990	Amputation of lower limb, unilateral	0.0639
Costa Rica	1995	Amputation of lower limb, unilateral	0.0608
Costa Rica	2000	Amputation of lower limb, unilateral	0.0568
Costa Rica	2005	Amputation of lower limb, unilateral	0.0513
Costa Rica	2010	Amputation of lower limb, unilateral	0.0464
Costa Rica	2017	Amputation of lower limb, unilateral	0.0407
Costa Rica	1990	Open wound(s)	0.011
Costa Rica	1995	Open wound(s)	0.011
Costa Rica	2000	Open wound(s)	0.011
Costa Rica	2005	Open wound(s)	0.011
Costa Rica	2010	Open wound(s)	0.011
Costa Rica	2017	Open wound(s)	0.011
Costa Rica	1990	Poisoning requiring urgent care	0.1628
Costa Rica	1995	Poisoning requiring urgent care	0.1628
Costa Rica	2000	Poisoning requiring urgent care	0.1628
Costa Rica	2005	Poisoning requiring urgent care	0.1628
Costa Rica	2010	Poisoning requiring urgent care	0.1628
Costa Rica	2017	Poisoning requiring urgent care	0.1628
Costa Rica	1990	Severe chest injury	0.0474
Costa Rica	1995	Severe chest injury	0.0474
Costa Rica	2000	Severe chest injury	0.0474
Costa Rica	2005	Severe chest injury	0.0474
Costa Rica	2010	Severe chest injury	0.0474
Costa Rica	2017	Severe chest injury	0.0474
Costa Rica	1990	Internal hemorrhage in abdomen and pelvis	0.1143
Costa Rica	1995	Internal hemorrhage in abdomen and pelvis	0.1143
Costa Rica	2000	Internal hemorrhage in abdomen and pelvis	0.1143
Costa Rica	2005	Internal hemorrhage in abdomen and pelvis	0.1143
Costa Rica	2010	Internal hemorrhage in abdomen and pelvis	0.1143
Costa Rica	2017	Internal hemorrhage in abdomen and pelvis	0.1143
Costa Rica	1990	Contusion in any part of the body	0.0075
Costa Rica	1995	Contusion in any part of the body	0.0075
Costa Rica	2000	Contusion in any part of the body	0.0075
Costa Rica	2005	Contusion in any part of the body	0.0075
Costa Rica	2010	Contusion in any part of the body	0.0075
Costa Rica	2017	Contusion in any part of the body	0.0075
Costa Rica	1990	Effect of different environmental factors	0.049
Costa Rica	1995	Effect of different environmental factors	0.049
Costa Rica	2000	Effect of different environmental factors	0.049
Costa Rica	2005	Effect of different environmental factors	0.049
Costa Rica	2010	Effect of different environmental factors	0.049
Costa Rica	2017	Effect of different environmental factors	0.049
Costa Rica	1990	Complications following therapeutic procedures	0.0506
Costa Rica	1995	Complications following therapeutic procedures	0.0506
Costa Rica	2000	Complications following therapeutic procedures	0.0506
Costa Rica	2005	Complications following therapeutic procedures	0.0506
Costa Rica	2010	Complications following therapeutic procedures	0.0506
Costa Rica	2017	Complications following therapeutic procedures	0.0506
Costa Rica	1990	Multiple fractures, dislocations, crashes, wounds, sprains, and strains	0.1216
Costa Rica	1995	Multiple fractures, dislocations, crashes, wounds, sprains, and strains	0.1137
Costa Rica	2000	Multiple fractures, dislocations, crashes, wounds, sprains, and strains	0.1034
Costa Rica	2005	Multiple fractures, dislocations, crashes, wounds, sprains, and strains	0.0893
Costa Rica	2010	Multiple fractures, dislocations, crashes, wounds, sprains, and strains	0.0767
Costa Rica	2017	Multiple fractures, dislocations, crashes, wounds, sprains, and strains	0.0622
Costa Rica	1990	Amputation of upper limb, unilateral	0.0536
Costa Rica	1995	Amputation of upper limb, unilateral	0.0518
Costa Rica	2000	Amputation of upper limb, unilateral	0.0495
Costa Rica	2005	Amputation of upper limb, unilateral	0.0462
Costa Rica	2010	Amputation of upper limb, unilateral	0.0434
Costa Rica	2017	Amputation of upper limb, unilateral	0.04
Costa Rica	1990	Amputation of thumb	0.0114
Costa Rica	1995	Amputation of thumb	0.0114
Costa Rica	2000	Amputation of thumb	0.0114
Costa Rica	2005	Amputation of thumb	0.0114
Costa Rica	2010	Amputation of thumb	0.0114
Costa Rica	2017	Amputation of thumb	0.0114
Costa Rica	1990	Amputation of toe/toes	0.0059
Costa Rica	1995	Amputation of toe/toes	0.0059
Costa Rica	2000	Amputation of toe/toes	0.0059
Costa Rica	2005	Amputation of toe/toes	0.0059
Costa Rica	2010	Amputation of toe/toes	0.0059
Costa Rica	2017	Amputation of toe/toes	0.0059
Costa Rica	1990	Burns, <20% total burned surface area without lower airway burns	0.0163
Costa Rica	1995	Burns, <20% total burned surface area without lower airway burns	0.0163
Costa Rica	2000	Burns, <20% total burned surface area without lower airway burns	0.0163
Costa Rica	2005	Burns, <20% total burned surface area without lower airway burns	0.0163
Costa Rica	2010	Burns, <20% total burned surface area without lower airway burns	0.0163
Costa Rica	2017	Burns, <20% total burned surface area without lower airway burns	0.0163
Costa Rica	1990	Burns, >=20% total burned surface area or >= 10% burned surface area if head/neck or hands/wrist involved w/o lower airway burns	0.1941
Costa Rica	1995	Burns, >=20% total burned surface area or >= 10% burned surface area if head/neck or hands/wrist involved w/o lower airway burns	0.1868
Costa Rica	2000	Burns, >=20% total burned surface area or >= 10% burned surface area if head/neck or hands/wrist involved w/o lower airway burns	0.1772
Costa Rica	2005	Burns, >=20% total burned surface area or >= 10% burned surface area if head/neck or hands/wrist involved w/o lower airway burns	0.164
Costa Rica	2010	Burns, >=20% total burned surface area or >= 10% burned surface area if head/neck or hands/wrist involved w/o lower airway burns	0.1523
Costa Rica	2017	Burns, >=20% total burned surface area or >= 10% burned surface area if head/neck or hands/wrist involved w/o lower airway burns	0.1388
El Salvador	1990	Amputation of lower limbs, bilateral	0.2476
El Salvador	1995	Amputation of lower limbs, bilateral	0.2271
El Salvador	2000	Amputation of lower limbs, bilateral	0.2008
El Salvador	2005	Amputation of lower limbs, bilateral	0.1855
El Salvador	2010	Amputation of lower limbs, bilateral	0.1759
El Salvador	2017	Amputation of lower limbs, bilateral	0.1614
El Salvador	1990	Lower airway burns	0.2252
El Salvador	1995	Lower airway burns	0.2252
El Salvador	2000	Lower airway burns	0.2252
El Salvador	2005	Lower airway burns	0.2252
El Salvador	2010	Lower airway burns	0.2252
El Salvador	2017	Lower airway burns	0.2252
El Salvador	1990	Dislocation of hip	0.0159
El Salvador	1995	Dislocation of hip	0.0159
El Salvador	2000	Dislocation of hip	0.0159
El Salvador	2005	Dislocation of hip	0.0159
El Salvador	2010	Dislocation of hip	0.0159
El Salvador	2017	Dislocation of hip	0.0159
El Salvador	1990	Dislocation of knee	0.1134
El Salvador	1995	Dislocation of knee	0.1134
El Salvador	2000	Dislocation of knee	0.1134
El Salvador	2005	Dislocation of knee	0.1134
El Salvador	2010	Dislocation of knee	0.1134
El Salvador	2017	Dislocation of knee	0.1134
El Salvador	1990	Dislocation of shoulder	0.062
El Salvador	1995	Dislocation of shoulder	0.062
El Salvador	2000	Dislocation of shoulder	0.062
El Salvador	2005	Dislocation of shoulder	0.062
El Salvador	2010	Dislocation of shoulder	0.062
El Salvador	2017	Dislocation of shoulder	0.062
El Salvador	1990	Muscle and tendon injuries, including sprains and strains lesser dislocations	0.0075
El Salvador	1995	Muscle and tendon injuries, including sprains and strains lesser dislocations	0.0075
El Salvador	2000	Muscle and tendon injuries, including sprains and strains lesser dislocations	0.0075
El Salvador	2005	Muscle and tendon injuries, including sprains and strains lesser dislocations	0.0075
El Salvador	2010	Muscle and tendon injuries, including sprains and strains lesser dislocations	0.0075
El Salvador	2017	Muscle and tendon injuries, including sprains and strains lesser dislocations	0.0075
El Salvador	1990	Fracture of clavicle, scapula, or humerus	0.0349
El Salvador	1995	Fracture of clavicle, scapula, or humerus	0.0349
El Salvador	2000	Fracture of clavicle, scapula, or humerus	0.0349
El Salvador	2005	Fracture of clavicle, scapula, or humerus	0.0349
El Salvador	2010	Fracture of clavicle, scapula, or humerus	0.0349
El Salvador	2017	Fracture of clavicle, scapula, or humerus	0.0349
El Salvador	1990	Fracture of face bones	0.0669
El Salvador	1995	Fracture of face bones	0.0669
El Salvador	2000	Fracture of face bones	0.0669
El Salvador	2005	Fracture of face bones	0.0669
El Salvador	2010	Fracture of face bones	0.0669
El Salvador	2017	Fracture of face bones	0.0669
El Salvador	1990	Fracture of foot bones except ankle	0.0264
El Salvador	1995	Fracture of foot bones except ankle	0.0264
El Salvador	2000	Fracture of foot bones except ankle	0.0264
El Salvador	2005	Fracture of foot bones except ankle	0.0264
El Salvador	2010	Fracture of foot bones except ankle	0.0264
El Salvador	2017	Fracture of foot bones except ankle	0.0264
El Salvador	1990	Fracture of hand (wrist and other distal part of hand)	0.0138
El Salvador	1995	Fracture of hand (wrist and other distal part of hand)	0.0138

El Salvador	2000	Fracture of hand (wrist and other distal part of hand)	0.0138
El Salvador	2005	Fracture of hand (wrist and other distal part of hand)	0.0138
El Salvador	2010	Fracture of hand (wrist and other distal part of hand)	0.0138
El Salvador	2017	Fracture of hand (wrist and other distal part of hand)	0.0138
El Salvador	1990	Fracture of hip	0.2222
El Salvador	1995	Fracture of hip	0.1951
El Salvador	2000	Fracture of hip	0.1603
El Salvador	2005	Fracture of hip	0.14
El Salvador	2010	Fracture of hip	0.1273
El Salvador	2017	Fracture of hip	0.1082
El Salvador	1990	Amputation of upper limbs, bilateral	0.2575
El Salvador	1995	Amputation of upper limbs, bilateral	0.2295
El Salvador	2000	Amputation of upper limbs, bilateral	0.1936
El Salvador	2005	Amputation of upper limbs, bilateral	0.1726
El Salvador	2010	Amputation of upper limbs, bilateral	0.1596
El Salvador	2017	Amputation of upper limbs, bilateral	0.1398
El Salvador	1990	Fracture of patella, tibia or fibula, or ankle	0.0554
El Salvador	1995	Fracture of patella, tibia or fibula, or ankle	0.0554
El Salvador	2000	Fracture of patella, tibia or fibula, or ankle	0.0554
El Salvador	2005	Fracture of patella, tibia or fibula, or ankle	0.0554
El Salvador	2010	Fracture of patella, tibia or fibula, or ankle	0.0554
El Salvador	2017	Fracture of patella, tibia or fibula, or ankle	0.0554
El Salvador	1990	Fracture of pelvis	0.1823
El Salvador	1995	Fracture of pelvis	0.1823
El Salvador	2000	Fracture of pelvis	0.1823
El Salvador	2005	Fracture of pelvis	0.1823
El Salvador	2010	Fracture of pelvis	0.1823
El Salvador	2017	Fracture of pelvis	0.1823
El Salvador	1990	Fracture of radius and/or ulna	0.0435
El Salvador	1995	Fracture of radius and/or ulna	0.0435
El Salvador	2000	Fracture of radius and/or ulna	0.0435
El Salvador	2005	Fracture of radius and/or ulna	0.0435
El Salvador	2010	Fracture of radius and/or ulna	0.0435
El Salvador	2017	Fracture of radius and/or ulna	0.0435
El Salvador	1990	Fracture of skull	0.0714
El Salvador	1995	Fracture of skull	0.0714
El Salvador	2000	Fracture of skull	0.0714
El Salvador	2005	Fracture of skull	0.0714
El Salvador	2010	Fracture of skull	0.0714
El Salvador	2017	Fracture of skull	0.0714
El Salvador	1990	Fracture of sternum and/or fracture of one or more ribs	0.1027
El Salvador	1995	Fracture of sternum and/or fracture of one or more ribs	0.1027
El Salvador	2000	Fracture of sternum and/or fracture of one or more ribs	0.1027
El Salvador	2005	Fracture of sternum and/or fracture of one or more ribs	0.1027
El Salvador	2010	Fracture of sternum and/or fracture of one or more ribs	0.1027
El Salvador	2017	Fracture of sternum and/or fracture of one or more ribs	0.1027
El Salvador	1990	Fracture of vertebral column	0.1106
El Salvador	1995	Fracture of vertebral column	0.1106
El Salvador	2000	Fracture of vertebral column	0.1106
El Salvador	2005	Fracture of vertebral column	0.1106
El Salvador	2010	Fracture of vertebral column	0.1106
El Salvador	2017	Fracture of vertebral column	0.1106
El Salvador	1990	Fracture of femur, other than femoral neck	0.042
El Salvador	1995	Fracture of femur, other than femoral neck	0.042
El Salvador	2000	Fracture of femur, other than femoral neck	0.042
El Salvador	2005	Fracture of femur, other than femoral neck	0.042
El Salvador	2010	Fracture of femur, other than femoral neck	0.042
El Salvador	2017	Fracture of femur, other than femoral neck	0.042
El Salvador	1990	Minor TBI	0.1319
El Salvador	1995	Minor TBI	0.1319
El Salvador	2000	Minor TBI	0.1319
El Salvador	2005	Minor TBI	0.1319
El Salvador	2010	Minor TBI	0.1319
El Salvador	2017	Minor TBI	0.1319
El Salvador	1990	Moderate TBI	0.1637
El Salvador	1995	Moderate TBI	0.1637
El Salvador	2000	Moderate TBI	0.1637
El Salvador	2005	Moderate TBI	0.1637
El Salvador	2010	Moderate TBI	0.1637
El Salvador	2017	Moderate TBI	0.1637
El Salvador	1990	Amputation of fingers (excluding thumb)	0.0047
El Salvador	1995	Amputation of fingers (excluding thumb)	0.0047
El Salvador	2000	Amputation of fingers (excluding thumb)	0.0047
El Salvador	2005	Amputation of fingers (excluding thumb)	0.0047
El Salvador	2010	Amputation of fingers (excluding thumb)	0.0047
El Salvador	2017	Amputation of fingers (excluding thumb)	0.0047
El Salvador	1990	Spinal cord lesion at neck level	0.4687
El Salvador	1995	Spinal cord lesion at neck level	0.4593
El Salvador	2000	Spinal cord lesion at neck level	0.4473
El Salvador	2005	Spinal cord lesion at neck level	0.4403
El Salvador	2010	Spinal cord lesion at neck level	0.4359
El Salvador	2017	Spinal cord lesion at neck level	0.4293
El Salvador	1990	Spinal cord lesion below neck level	0.2977
El Salvador	1995	Spinal cord lesion below neck level	0.2779
El Salvador	2000	Spinal cord lesion below neck level	0.2526
El Salvador	2005	Spinal cord lesion below neck level	0.2377
El Salvador	2010	Spinal cord lesion below neck level	0.2285
El Salvador	2017	Spinal cord lesion below neck level	0.2145
El Salvador	1990	Drowning and nonfatal submersion	0.2471
El Salvador	1995	Drowning and nonfatal submersion	0.2471
El Salvador	2000	Drowning and nonfatal submersion	0.2471
El Salvador	2005	Drowning and nonfatal submersion	0.2471
El Salvador	2010	Drowning and nonfatal submersion	0.2471
El Salvador	2017	Drowning and nonfatal submersion	0.2471
El Salvador	1990	Asphyxiation	0.2471
El Salvador	1995	Asphyxiation	0.2471
El Salvador	2000	Asphyxiation	0.2471
El Salvador	2005	Asphyxiation	0.2471
El Salvador	2010	Asphyxiation	0.2471
El Salvador	2017	Asphyxiation	0.2471
El Salvador	1990	Crush injury	0.1325
El Salvador	1995	Crush injury	0.1325
El Salvador	2000	Crush injury	0.1325
El Salvador	2005	Crush injury	0.1325
El Salvador	2010	Crush injury	0.1325
El Salvador	2017	Crush injury	0.1325
El Salvador	1990	Nerve injury	0.1129
El Salvador	1995	Nerve injury	0.1129
El Salvador	2000	Nerve injury	0.1129
El Salvador	2005	Nerve injury	0.1129
El Salvador	2010	Nerve injury	0.1129
El Salvador	2017	Nerve injury	0.1129
El Salvador	1990	Injury to eyes	0.0165
El Salvador	1995	Injury to eyes	0.0165
El Salvador	2000	Injury to eyes	0.0165
El Salvador	2005	Injury to eyes	0.0165
El Salvador	2010	Injury to eyes	0.0165
El Salvador	2017	Injury to eyes	0.0165
El Salvador	1990	Amputation of lower limb, unilateral	0.1031
El Salvador	1995	Amputation of lower limb, unilateral	0.0925
El Salvador	2000	Amputation of lower limb, unilateral	0.079
El Salvador	2005	Amputation of lower limb, unilateral	0.071
El Salvador	2010	Amputation of lower limb, unilateral	0.0661
El Salvador	2017	Amputation of lower limb, unilateral	0.0587
El Salvador	1990	Open wound(s)	0.011
El Salvador	1995	Open wound(s)	0.011
El Salvador	2000	Open wound(s)	0.011
El Salvador	2005	Open wound(s)	0.011
El Salvador	2010	Open wound(s)	0.011
El Salvador	2017	Open wound(s)	0.011
El Salvador	1990	Poisoning requiring urgent care	0.1628
El Salvador	1995	Poisoning requiring urgent care	0.1628
El Salvador	2000	Poisoning requiring urgent care	0.1628
El Salvador	2005	Poisoning requiring urgent care	0.1628
El Salvador	2010	Poisoning requiring urgent care	0.1628
El Salvador	2017	Poisoning requiring urgent care	0.1628
El Salvador	1990	Severe chest injury	0.0474
El Salvador	1995	Severe chest injury	0.0474
El Salvador	2000	Severe chest injury	0.0474
El Salvador	2005	Severe chest injury	0.0474
El Salvador	2010	Severe chest injury	0.0474
El Salvador	2017	Severe chest injury	0.0474
El Salvador	1990	Internal hemorrhage in abdomen and pelvis	0.1143
El Salvador	1995	Internal hemorrhage in abdomen and pelvis	0.1143
El Salvador	2000	Internal hemorrhage in abdomen and pelvis	0.1143
El Salvador	2005	Internal hemorrhage in abdomen and pelvis	0.1143
El Salvador	2010	Internal hemorrhage in abdomen and pelvis	0.1143
El Salvador	2017	Internal hemorrhage in abdomen and pelvis	0.1143
El Salvador	1990	Contusion in any part of the body	0.0075
El Salvador	1995	Contusion in any part of the body	0.0075
El Salvador	2000	Contusion in any part of the body	0.0075
El Salvador	2005	Contusion in any part of the body	0.0075
El Salvador	2010	Contusion in any part of the body	0.0075
El Salvador	2017	Contusion in any part of the body	0.0075
El Salvador	1990	Effect of different environmental factors	0.049
El Salvador	1995	Effect of different environmental factors	0.049
El Salvador	2000	Effect of different environmental factors	0.049
El Salvador	2005	Effect of different environmental factors	0.049
El Salvador	2010	Effect of different environmental factors	0.049
El Salvador	2017	Effect of different environmental factors	0.049
El Salvador	1990	Complications following therapeutic procedures	0.0506
El Salvador	1995	Complications following therapeutic procedures	0.0506
El Salvador	2000	Complications following therapeutic procedures	0.0506
El Salvador	2005	Complications following therapeutic procedures	0.0506
El Salvador	2010	Complications following therapeutic procedures	0.0506
El Salvador	2017	Complications following therapeutic procedures	0.0506
El Salvador	1990	Multiple fractures, dislocations, crashes, wounds, sprains, and strains	0.2222
El Salvador	1995	Multiple fractures, dislocations, crashes, wounds, sprains, and strains	0.1951
El Salvador	2000	Multiple fractures, dislocations, crashes, wounds, sprains, and strains	0.1603
El Salvador	2005	Multiple fractures, dislocations, crashes, wounds, sprains, and strains	0.14
El Salvador	2010	Multiple fractures, dislocations, crashes, wounds, sprains, and strains	0.1273
El Salvador	2017	Multiple fractures, dislocations, crashes, wounds, sprains, and strains	0.1082
El Salvador	1990	Amputation of upper limb, unilateral	0.0767
El Salvador	1995	Amputation of upper limb, unilateral	0.0705
El Salvador	2000	Amputation of upper limb, unilateral	0.0625
El Salvador	2005	Amputation of upper limb, unilateral	0.0579
El Salvador	2010	Amputation of upper limb, unilateral	0.055
El Salvador	2017	Amputation of upper limb, unilateral	0.0506
El Salvador	1990	Amputation of thumb	0.0114
El Salvador	1995	Amputation of thumb	0.0114
El Salvador	2000	Amputation of thumb	0.0114
El Salvador	2005	Amputation of thumb	0.0114
El Salvador	2010	Amputation of thumb	0.0114
El Salvador	2017	Amputation of thumb	0.0114
El Salvador	1990	Amputation of toe/toes	0.0059
El Salvador	1995	Amputation of toe/toes	0.0059
El Salvador	2000	Amputation of toe/toes	0.0059
El Salvador	2005	Amputation of toe/toes	0.0059
El Salvador	2010	Amputation of toe/toes	0.0059
El Salvador	2017	Amputation of toe/toes	0.0059
El Salvador	1990	Burns, <20% total burned surface area without lower airway burns	0.0163
El Salvador	1995	Burns, <20% total burned surface area without lower airway burns	0.0163
El Salvador	2000	Burns, <20% total burned surface area without lower airway burns	0.0163
El Salvador	2005	Burns, <20% total burned surface area without lower airway burns	0.0163
El Salvador	2010	Burns, <20% total burned surface area without lower airway burns	0.0163
El Salvador	2017	Burns, <20% total burned surface area without lower airway burns	0.0163
El Salvador	1990	Burns, >=20% total burned surface area or >= 10% burned surface area if head/neck or hands/wrist involved w/o lower airway burns	0.2878
El Salvador	1995	Burns, >=20% total burned surface area or >= 10% burned surface area if head/neck or hands/wrist involved w/o lower airway burns	0.2625
El Salvador	2000	Burns, >=20% total burned surface area or >= 10% burned surface area if head/neck or hands/wrist involved w/o lower airway burns	0.2301
El Salvador	2005	Burns, >=20% total burned surface area or >= 10% burned surface area if head/neck or hands/wrist involved w/o lower airway burns	0.2112
El Salvador	2010	Burns, >=20% total burned surface area or >= 10% burned surface area if head/neck or hands/wrist involved w/o lower airway burns	0.1995
El Salvador	2017	Burns, >=20% total burned surface area or >= 10% burned surface area if head/neck or hands/wrist involved w/o lower airway burns	0.1816
Guatemala	1990	Amputation of lower limbs, bilateral	0.2737
Guatemala	1995	Amputation of lower limbs, bilateral	0.2575
Guatemala	2000	Amputation of lower limbs, bilateral	0.2342
Guatemala	2005	Amputation of lower limbs, bilateral	0.2196
Guatemala	2010	Amputation of lower limbs, bilateral	0.2117
Guatemala	2017	Amputation of lower limbs, bilateral	0.2009
Guatemala	1990	Lower airway burns	0.2252
Guatemala	1995	Lower airway burns	0.2252
Guatemala	2000	Lower airway burns	0.2252
Guatemala	2005	Lower airway burns	0.2252
Guatemala	2010	Lower airway burns	0.2252
Guatemala	2017	Lower airway burns	0.2252
Guatemala	1990	Dislocation of hip	0.0159
Guatemala	1995	Dislocation of hip	0.0159
Guatemala	2000	Dislocation of hip	0.0159
Guatemala	2005	Dislocation of hip	0.0159
Guatemala	2010	Dislocation of hip	0.0159
Guatemala	2017	Dislocation of hip	0.0159
Guatemala	1990	Dislocation of knee	0.1134
Guatemala	1995	Dislocation of knee	0.1134
Guatemala	2000	Dislocation of knee	0.1134
Guatemala	2005	Dislocation of knee	0.1134
Guatemala	2010	Dislocation of knee	0.1134
Guatemala	2017	Dislocation of knee	0.1134
Guatemala	1990	Dislocation of shoulder	0.062
Guatemala	1995	Dislocation of shoulder	0.062
Guatemala	2000	Dislocation of shoulder	0.062
Guatemala	2005	Dislocation of shoulder	0.062
Guatemala	2010	Dislocation of shoulder	0.062
Guatemala	2017	Dislocation of shoulder	0.062
Guatemala	1990	Muscle and tendon injuries, including sprains and strains lesser dislocations	0.0075
Guatemala	1995	Muscle and tendon injuries, including sprains and strains lesser dislocations	0.0075
Guatemala	2000	Muscle and tendon injuries, including sprains and strains lesser dislocations	0.0075
Guatemala	2005	Muscle and tendon injuries, including sprains and strains lesser dislocations	0.0075
Guatemala	2010	Muscle and tendon injuries, including sprains and strains lesser dislocations	0.0075
Guatemala	2017	Muscle and tendon injuries, including sprains and strains lesser dislocations	0.0075
Guatemala	1990	Fracture of clavicle, scapula, or humerus	0.0349
Guatemala	1995	Fracture of clavicle, scapula, or humerus	0.0349
Guatemala	2000	Fracture of clavicle, scapula, or humerus	0.0349
Guatemala	2005	Fracture of clavicle, scapula, or humerus	0.0349
Guatemala	2010	Fracture of clavicle, scapula, or humerus	0.0349
Guatemala	2017	Fracture of clavicle, scapula, or humerus	0.0349
Guatemala	1990	Fracture of face bones	0.0669
Guatemala	1995	Fracture of face bones	0.0669
Guatemala	2000	Fracture of face bones	0.0669
Guatemala	2005	Fracture of face bones	0.0669
Guatemala	2010	Fracture of face bones	0.0669
Guatemala	2017	Fracture of face bones	0.0669
Guatemala	1990	Fracture of foot bones except ankle	0.0264
Guatemala	1995	Fracture of foot bones except ankle	0.0264
Guatemala	2000	Fracture of foot bones except ankle	0.0264
Guatemala	2005	Fracture of foot bones except ankle	0.0264
Guatemala	2010	Fracture of foot bones except ankle	0.0264
Guatemala	2017	Fracture of foot bones except ankle	0.0264
Guatemala	1990	Fracture of hand (wrist and other distal part of hand)	0.0138
Guatemala	1995	Fracture of hand (wrist and other distal part of hand)	0.0138
Guatemala	2000	Fracture of hand (wrist and other distal part of hand)	0.0138
Guatemala	2005	Fracture of hand (wrist and other distal part of hand)	0.0138
Guatemala	2010	Fracture of hand (wrist and other distal part of hand)	0.0138
Guatemala	2017	Fracture of hand (wrist and other distal part of hand)	0.0138
Guatemala	1990	Fracture of hip	0.2567
Guatemala	1995	Fracture of hip	0.2353
Guatemala	2000	Fracture of hip	0.2044
Guatemala	2005	Fracture of hip	0.1851
Guatemala	2010	Fracture of hip	0.1747
Guatemala	2017	Fracture of hip	0.1603
Guatemala	1990	Amputation of upper limbs, bilateral	0.2932
Guatemala	1995	Amputation of upper limbs, bilateral	0.271
Guatemala	2000	Amputation of upper limbs, bilateral	0.2392
Guatemala	2005	Amputation of upper limbs, bilateral	0.2192
Guatemala	2010	Amputation of upper limbs, bilateral	0.2084
Guatemala	2017	Amputation of upper limbs, bilateral	0.1936
Guatemala	1990	Fracture of patella, tibia or fibula, or ankle	0.0554
Guatemala	1995	Fracture of patella, tibia or fibula, or ankle	0.0554
Guatemala	2000	Fracture of patella, tibia or fibula, or ankle	0.0554
Guatemala	2005	Fracture of patella, tibia or fibula, or ankle	0.0554
Guatemala	2010	Fracture of patella, tibia or fibula, or ankle	0.0554
Guatemala	2017	Fracture of patella, tibia or fibula, or ankle	0.0554
Guatemala	1990	Fracture of pelvis	0.1823
Guatemala	1995	Fracture of pelvis	0.1823
Guatemala	2000	Fracture of pelvis	0.1823
Guatemala	2005	Fracture of pelvis	0.1823
Guatemala	2010	Fracture of pelvis	0.1823
Guatemala	2017	Fracture of pelvis	0.1823
Guatemala	1990	Fracture of radius and/or ulna	0.0435
Guatemala	1995	Fracture of radius and/or ulna	0.0435
Guatemala	2000	Fracture of radius and/or ulna	0.0435
Guatemala	2005	Fracture of radius and/or ulna	0.0435
Guatemala	2010	Fracture of radius and/or ulna	0.0435
Guatemala	2017	Fracture of radius and/or ulna	0.0435
Guatemala	1990	Fracture of skull	0.0714
Guatemala	1995	Fracture of skull	0.0714
Guatemala	2000	Fracture of skull	0.0714
Guatemala	2005	Fracture of skull	0.0714
Guatemala	2010	Fracture of skull	0.0714
Guatemala	2017	Fracture of skull	0.0714
Guatemala	1990	Fracture of sternum and/or fracture of one or more ribs	0.1027
Guatemala	1995	Fracture of sternum and/or fracture of one or more ribs	0.1027
Guatemala	2000	Fracture of sternum and/or fracture of one or more ribs	0.1027
Guatemala	2005	Fracture of sternum and/or fracture of one or more ribs	0.1027
Guatemala	2010	Fracture of sternum and/or fracture of one or more ribs	0.1027
Guatemala	2017	Fracture of sternum and/or fracture of one or more ribs	0.1027
Guatemala	1990	Fracture of vertebral column	0.1106
Guatemala	1995	Fracture of vertebral column	0.1106
Guatemala	2000	Fracture of vertebral column	0.1106
Guatemala	2005	Fracture of vertebral column	0.1106
Guatemala	2010	Fracture of vertebral column	0.1106
Guatemala	2017	Fracture of vertebral column	0.1106
Guatemala	1990	Fracture of femur, other than femoral neck	0.042
Guatemala	1995	Fracture of femur, other than femoral neck	0.042
Guatemala	2000	Fracture of femur, other than femoral neck	0.042
Guatemala	2005	Fracture of femur, other than femoral neck	0.042
Guatemala	2010	Fracture of femur, other than femoral neck	0.042
Guatemala	2017	Fracture of femur, other than femoral neck	0.042
Guatemala	1990	Minor TBI	0.1319
Guatemala	1995	Minor TBI	0.1319
Guatemala	2000	Minor TBI	0.1319
Guatemala	2005	Minor TBI	0.1319
Guatemala	2010	Minor TBI	0.1319
Guatemala	2017	Minor TBI	0.1319
Guatemala	1990	Moderate TBI	0.1637
Guatemala	1995	Moderate TBI	0.1637
Guatemala	2000	Moderate TBI	0.1637
Guatemala	2005	Moderate TBI	0.1637
Guatemala	2010	Moderate TBI	0.1637
Guatemala	2017	Moderate TBI	0.1637
Guatemala	1990	Amputation of fingers (excluding thumb)	0.0047
Guatemala	1995	Amputation of fingers (excluding thumb)	0.0047
Guatemala	2000	Amputation of fingers (excluding thumb)	0.0047
Guatemala	2005	Amputation of fingers (excluding thumb)	0.0047
Guatemala	2010	Amputation of fingers (excluding thumb)	0.0047
Guatemala	2017	Amputation of fingers (excluding thumb)	0.0047
Guatemala	1990	Spinal cord lesion at neck level	0.4807
Guatemala	1995	Spinal cord lesion at neck level	0.4732
Guatemala	2000	Spinal cord lesion at neck level	0.4626
Guatemala	2005	Spinal cord lesion at neck level	0.4559
Guatemala	2010	Spinal cord lesion at neck level	0.4523
Guatemala	2017	Spinal cord lesion at neck level	0.4473
Guatemala	1990	Spinal cord lesion below neck level	0.3229
Guatemala	1995	Spinal cord lesion below neck level	0.3073
Guatemala	2000	Spinal cord lesion below neck level	0.2848
Guatemala	2005	Spinal cord lesion below neck level	0.2707
Guatemala	2010	Spinal cord lesion below neck level	0.263
Guatemala	2017	Spinal cord lesion below neck level	0.2526
Guatemala	1990	Drowning and nonfatal submersion	0.2471
Guatemala	1995	Drowning and nonfatal submersion	0.2471
Guatemala	2000	Drowning and nonfatal submersion	0.2471
Guatemala	2005	Drowning and nonfatal submersion	0.2471
Guatemala	2010	Drowning and nonfatal submersion	0.2471
Guatemala	2017	Drowning and nonfatal submersion	0.2471
Guatemala	1990	Asphyxiation	0.2471
Guatemala	1995	Asphyxiation	0.2471
Guatemala	2000	Asphyxiation	0.2471
Guatemala	2005	Asphyxiation	0.2471
Guatemala	2010	Asphyxiation	0.2471
Guatemala	2017	Asphyxiation	0.2471
Guatemala	1990	Crush injury	0.1325
Guatemala	1995	Crush injury	0.1325
Guatemala	2000	Crush injury	0.1325
Guatemala	2005	Crush injury	0.1325
Guatemala	2010	Crush injury	0.1325
Guatemala	2017	Crush injury	0.1325
Guatemala	1990	Nerve injury	0.1129
Guatemala	1995	Nerve injury	0.1129
Guatemala	2000	Nerve injury	0.1129
Guatemala	2005	Nerve injury	0.1129
Guatemala	2010	Nerve injury	0.1129
Guatemala	2017	Nerve injury	0.1129
Guatemala	1990	Injury to eyes	0.0165
Guatemala	1995	Injury to eyes	0.0165
Guatemala	2000	Injury to eyes	0.0165
Guatemala	2005	Injury to eyes	0.0165
Guatemala	2010	Injury to eyes	0.0165
Guatemala	2017	Injury to eyes	0.0165
Guatemala	1990	Amputation of lower limb, unilateral	0.1165
Guatemala	1995	Amputation of lower limb, unilateral	0.1082
Guatemala	2000	Amputation of lower limb, unilateral	0.0961
Guatemala	2005	Amputation of lower limb, unilateral	0.0886
Guatemala	2010	Amputation of lower limb, unilateral	0.0846
Guatemala	2017	Amputation of lower limb, unilateral	0.079
Guatemala	1990	Open wound(s)	0.011
Guatemala	1995	Open wound(s)	0.011
Guatemala	2000	Open wound(s)	0.011
Guatemala	2005	Open wound(s)	0.011
Guatemala	2010	Open wound(s)	0.011
Guatemala	2017	Open wound(s)	0.011
Guatemala	1990	Poisoning requiring urgent care	0.1628
Guatemala	1995	Poisoning requiring urgent care	0.1628
Guatemala	2000	Poisoning requiring urgent care	0.1628
Guatemala	2005	Poisoning requiring urgent care	0.1628
Guatemala	2010	Poisoning requiring urgent care	0.1628
Guatemala	2017	Poisoning requiring urgent care	0.1628
Guatemala	1990	Severe chest injury	0.0474
Guatemala	1995	Severe chest injury	0.0474
Guatemala	2000	Severe chest injury	0.0474
Guatemala	2005	Severe chest injury	0.0474
Guatemala	2010	Severe chest injury	0.0474
Guatemala	2017	Severe chest injury	0.0474
Guatemala	1990	Internal hemorrhage in abdomen and pelvis	0.1143
Guatemala	1995	Internal hemorrhage in abdomen and pelvis	0.1143
Guatemala	2000	Internal hemorrhage in abdomen and pelvis	0.1143
Guatemala	2005	Internal hemorrhage in abdomen and pelvis	0.1143
Guatemala	2010	Internal hemorrhage in abdomen and pelvis	0.1143
Guatemala	2017	Internal hemorrhage in abdomen and pelvis	0.1143
Guatemala	1990	Contusion in any part of the body	0.0075
Guatemala	1995	Contusion in any part of the body	0.0075
Guatemala	2000	Contusion in any part of the body	0.0075
Guatemala	2005	Contusion in any part of the body	0.0075
Guatemala	2010	Contusion in any part of the body	0.0075
Guatemala	2017	Contusion in any part of the body	0.0075
Guatemala	1990	Effect of different environmental factors	0.049
Guatemala	1995	Effect of different environmental factors	0.049
Guatemala	2000	Effect of different environmental factors	0.049
Guatemala	2005	Effect of different environmental factors	0.049
Guatemala	2010	Effect of different environmental factors	0.049
Guatemala	2017	Effect of different environmental factors	0.049
Guatemala	1990	Complications following therapeutic procedures	0.0506
Guatemala	1995	Complications following therapeutic procedures	0.0506
Guatemala	2000	Complications following therapeutic procedures	0.0506
Guatemala	2005	Complications following therapeutic procedures	0.0506
Guatemala	2010	Complications following therapeutic procedures	0.0506
Guatemala	2017	Complications following therapeutic procedures	0.0506
Guatemala	1990	Multiple fractures, dislocations, crashes, wounds, sprains, and strains	0.2567
Guatemala	1995	Multiple fractures, dislocations, crashes, wounds, sprains, and strains	0.2353
Guatemala	2000	Multiple fractures, dislocations, crashes, wounds, sprains, and strains	0.2044
Guatemala	2005	Multiple fractures, dislocations, crashes, wounds, sprains, and strains	0.1851
Guatemala	2010	Multiple fractures, dislocations, crashes, wounds, sprains, and strains	0.1747
Guatemala	2017	Multiple fractures, dislocations, crashes, wounds, sprains, and strains	0.1603
Guatemala	1990	Amputation of upper limb, unilateral	0.0847

Guatemala	1995	Amputation of upper limb, unilateral	0.0797
Guatemala	2000	Amputation of upper limb, unilateral	0.0726
Guatemala	2005	Amputation of upper limb, unilateral	0.0682
Guatemala	2010	Amputation of upper limb, unilateral	0.0658
Guatemala	2017	Amputation of upper limb, unilateral	0.0625
Guatemala	1990	Amputation of thumb	0.0114
Guatemala	1995	Amputation of thumb	0.0114
Guatemala	2000	Amputation of thumb	0.0114
Guatemala	2005	Amputation of thumb	0.0114
Guatemala	2010	Amputation of thumb	0.0114
Guatemala	2017	Amputation of thumb	0.0114
Guatemala	1990	Amputation of toe/toes	0.0059
Guatemala	1995	Amputation of toe/toes	0.0059
Guatemala	2000	Amputation of toe/toes	0.0059
Guatemala	2005	Amputation of toe/toes	0.0059
Guatemala	2010	Amputation of toe/toes	0.0059
Guatemala	2017	Amputation of toe/toes	0.0059
Guatemala	1990	Burns, <20% total burned surface area without lower airway burns	0.0163
Guatemala	1995	Burns, <20% total burned surface area without lower airway burns	0.0163
Guatemala	2000	Burns, <20% total burned surface area without lower airway burns	0.0163
Guatemala	2005	Burns, <20% total burned surface area without lower airway burns	0.0163
Guatemala	2010	Burns, <20% total burned surface area without lower airway burns	0.0163
Guatemala	2017	Burns, <20% total burned surface area without lower airway burns	0.0163
Guatemala	1990	Burns, >=20% total burned surface area or >= 10% burned surface area if head/neck or hands/wrist involved w/o lower airway burns	0.3199
Guatemala	1995	Burns, >=20% total burned surface area or >= 10% burned surface area if head/neck or hands/wrist involved w/o lower airway burns	0.3
Guatemala	2000	Burns, >=20% total burned surface area or >= 10% burned surface area if head/neck or hands/wrist involved w/o lower airway burns	0.2712
Guatemala	2005	Burns, >=20% total burned surface area or >= 10% burned surface area if head/neck or hands/wrist involved w/o lower airway burns	0.2532
Guatemala	2010	Burns, >=20% total burned surface area or >= 10% burned surface area if head/neck or hands/wrist involved w/o lower airway burns	0.2435
Guatemala	2017	Burns, >=20% total burned surface area or >= 10% burned surface area if head/neck or hands/wrist involved w/o lower airway burns	0.2302
Honduras	1990	Amputation of lower limbs, bilateral	0.2806
Honduras	1995	Amputation of lower limbs, bilateral	0.2579
Honduras	2000	Amputation of lower limbs, bilateral	0.2472
Honduras	2005	Amputation of lower limbs, bilateral	0.2385
Honduras	2010	Amputation of lower limbs, bilateral	0.229
Honduras	2017	Amputation of lower limbs, bilateral	0.2174
Honduras	1990	Lower airway burns	0.2252
Honduras	1995	Lower airway burns	0.2252
Honduras	2000	Lower airway burns	0.2252
Honduras	2005	Lower airway burns	0.2252
Honduras	2010	Lower airway burns	0.2252
Honduras	2017	Lower airway burns	0.2252
Honduras	1990	Dislocation of hip	0.0159
Honduras	1995	Dislocation of hip	0.0159
Honduras	2000	Dislocation of hip	0.0159
Honduras	2005	Dislocation of hip	0.0159
Honduras	2010	Dislocation of hip	0.0159
Honduras	2017	Dislocation of hip	0.0159
Honduras	1990	Dislocation of knee	0.1134
Honduras	1995	Dislocation of knee	0.1134
Honduras	2000	Dislocation of knee	0.1134
Honduras	2005	Dislocation of knee	0.1134
Honduras	2010	Dislocation of knee	0.1134
Honduras	2017	Dislocation of knee	0.1134
Honduras	1990	Dislocation of shoulder	0.062
Honduras	1995	Dislocation of shoulder	0.062
Honduras	2000	Dislocation of shoulder	0.062
Honduras	2005	Dislocation of shoulder	0.062
Honduras	2010	Dislocation of shoulder	0.062
Honduras	2017	Dislocation of shoulder	0.062
Honduras	1990	Muscle and tendon injuries, including sprains and strains lesser dislocations	0.0075
Honduras	1995	Muscle and tendon injuries, including sprains and strains lesser dislocations	0.0075
Honduras	2000	Muscle and tendon injuries, including sprains and strains lesser dislocations	0.0075
Honduras	2005	Muscle and tendon injuries, including sprains and strains lesser dislocations	0.0075
Honduras	2010	Muscle and tendon injuries, including sprains and strains lesser dislocations	0.0075
Honduras	2017	Muscle and tendon injuries, including sprains and strains lesser dislocations	0.0075
Honduras	1990	Fracture of clavicle, scapula, or humerus	0.0349
Honduras	1995	Fracture of clavicle, scapula, or humerus	0.0349
Honduras	2000	Fracture of clavicle, scapula, or humerus	0.0349
Honduras	2005	Fracture of clavicle, scapula, or humerus	0.0349
Honduras	2010	Fracture of clavicle, scapula, or humerus	0.0349
Honduras	2017	Fracture of clavicle, scapula, or humerus	0.0349
Honduras	1990	Fracture of face bones	0.0669
Honduras	1995	Fracture of face bones	0.0669
Honduras	2000	Fracture of face bones	0.0669
Honduras	2005	Fracture of face bones	0.0669
Honduras	2010	Fracture of face bones	0.0669
Honduras	2017	Fracture of face bones	0.0669
Honduras	1990	Fracture of foot bones except ankle	0.0264
Honduras	1995	Fracture of foot bones except ankle	0.0264
Honduras	2000	Fracture of foot bones except ankle	0.0264
Honduras	2005	Fracture of foot bones except ankle	0.0264
Honduras	2010	Fracture of foot bones except ankle	0.0264
Honduras	2017	Fracture of foot bones except ankle	0.0264
Honduras	1990	Fracture of hand (wrist and other distal part of hand)	0.0138
Honduras	1995	Fracture of hand (wrist and other distal part of hand)	0.0138
Honduras	2000	Fracture of hand (wrist and other distal part of hand)	0.0138
Honduras	2005	Fracture of hand (wrist and other distal part of hand)	0.0138
Honduras	2010	Fracture of hand (wrist and other distal part of hand)	0.0138
Honduras	2017	Fracture of hand (wrist and other distal part of hand)	0.0138
Honduras	1990	Fracture of hip	0.2659
Honduras	1995	Fracture of hip	0.2358
Honduras	2000	Fracture of hip	0.2216
Honduras	2005	Fracture of hip	0.2101
Honduras	2010	Fracture of hip	0.1976
Honduras	2017	Fracture of hip	0.1822
Honduras	1990	Amputation of upper limbs, bilateral	0.3026
Honduras	1995	Amputation of upper limbs, bilateral	0.2715
Honduras	2000	Amputation of upper limbs, bilateral	0.2569
Honduras	2005	Amputation of upper limbs, bilateral	0.245
Honduras	2010	Amputation of upper limbs, bilateral	0.2321
Honduras	2017	Amputation of upper limbs, bilateral	0.2162
Honduras	1990	Fracture of patella, tibia or fibula, or ankle	0.0554
Honduras	1995	Fracture of patella, tibia or fibula, or ankle	0.0554
Honduras	2000	Fracture of patella, tibia or fibula, or ankle	0.0554
Honduras	2005	Fracture of patella, tibia or fibula, or ankle	0.0554
Honduras	2010	Fracture of patella, tibia or fibula, or ankle	0.0554
Honduras	2017	Fracture of patella, tibia or fibula, or ankle	0.0554
Honduras	1990	Fracture of pelvis	0.1823
Honduras	1995	Fracture of pelvis	0.1823
Honduras	2000	Fracture of pelvis	0.1823
Honduras	2005	Fracture of pelvis	0.1823
Honduras	2010	Fracture of pelvis	0.1823
Honduras	2017	Fracture of pelvis	0.1823
Honduras	1990	Fracture of radius and/or ulna	0.0435
Honduras	1995	Fracture of radius and/or ulna	0.0435
Honduras	2000	Fracture of radius and/or ulna	0.0435
Honduras	2005	Fracture of radius and/or ulna	0.0435
Honduras	2010	Fracture of radius and/or ulna	0.0435
Honduras	2017	Fracture of radius and/or ulna	0.0435
Honduras	1990	Fracture of skull	0.0714
Honduras	1995	Fracture of skull	0.0714
Honduras	2000	Fracture of skull	0.0714
Honduras	2005	Fracture of skull	0.0714
Honduras	2010	Fracture of skull	0.0714
Honduras	2017	Fracture of skull	0.0714
Honduras	1990	Fracture of sternum and/or fracture of one or more ribs	0.1027
Honduras	1995	Fracture of sternum and/or fracture of one or more ribs	0.1027
Honduras	2000	Fracture of sternum and/or fracture of one or more ribs	0.1027
Honduras	2005	Fracture of sternum and/or fracture of one or more ribs	0.1027
Honduras	2010	Fracture of sternum and/or fracture of one or more ribs	0.1027
Honduras	2017	Fracture of sternum and/or fracture of one or more ribs	0.1027
Honduras	1990	Fracture of vertebral column	0.1106
Honduras	1995	Fracture of vertebral column	0.1106
Honduras	2000	Fracture of vertebral column	0.1106
Honduras	2005	Fracture of vertebral column	0.1106
Honduras	2010	Fracture of vertebral column	0.1106
Honduras	2017	Fracture of vertebral column	0.1106
Honduras	1990	Fracture of femur, other than femoral neck	0.042
Honduras	1995	Fracture of femur, other than femoral neck	0.042
Honduras	2000	Fracture of femur, other than femoral neck	0.042
Honduras	2005	Fracture of femur, other than femoral neck	0.042
Honduras	2010	Fracture of femur, other than femoral neck	0.042
Honduras	2017	Fracture of femur, other than femoral neck	0.042
Honduras	1990	Minor TBI	0.1319
Honduras	1995	Minor TBI	0.1319
Honduras	2000	Minor TBI	0.1319
Honduras	2005	Minor TBI	0.1319
Honduras	2010	Minor TBI	0.1319
Honduras	2017	Minor TBI	0.1319
Honduras	1990	Moderate TBI	0.1637
Honduras	1995	Moderate TBI	0.1637
Honduras	2000	Moderate TBI	0.1637
Honduras	2005	Moderate TBI	0.1637
Honduras	2010	Moderate TBI	0.1637
Honduras	2017	Moderate TBI	0.1637
Honduras	1990	Amputation of fingers (excluding thumb)	0.0047
Honduras	1995	Amputation of fingers (excluding thumb)	0.0047
Honduras	2000	Amputation of fingers (excluding thumb)	0.0047
Honduras	2005	Amputation of fingers (excluding thumb)	0.0047
Honduras	2010	Amputation of fingers (excluding thumb)	0.0047
Honduras	2017	Amputation of fingers (excluding thumb)	0.0047
Honduras	1990	Spinal cord lesion at neck level	0.4838
Honduras	1995	Spinal cord lesion at neck level	0.4734
Honduras	2000	Spinal cord lesion at neck level	0.4685
Honduras	2005	Spinal cord lesion at neck level	0.4645
Honduras	2010	Spinal cord lesion at neck level	0.4602
Honduras	2017	Spinal cord lesion at neck level	0.4549
Honduras	1990	Spinal cord lesion below neck level	0.3296
Honduras	1995	Spinal cord lesion below neck level	0.3077
Honduras	2000	Spinal cord lesion below neck level	0.2973
Honduras	2005	Spinal cord lesion below neck level	0.2889
Honduras	2010	Spinal cord lesion below neck level	0.2797
Honduras	2017	Spinal cord lesion below neck level	0.2685
Honduras	1990	Drowning and nonfatal submersion	0.2471
Honduras	1995	Drowning and nonfatal submersion	0.2471
Honduras	2000	Drowning and nonfatal submersion	0.2471
Honduras	2005	Drowning and nonfatal submersion	0.2471
Honduras	2010	Drowning and nonfatal submersion	0.2471
Honduras	2017	Drowning and nonfatal submersion	0.2471
Honduras	1990	Asphyxiation	0.2471
Honduras	1995	Asphyxiation	0.2471
Honduras	2000	Asphyxiation	0.2471
Honduras	2005	Asphyxiation	0.2471
Honduras	2010	Asphyxiation	0.2471
Honduras	2017	Asphyxiation	0.2471
Honduras	1990	Crush injury	0.1325
Honduras	1995	Crush injury	0.1325
Honduras	2000	Crush injury	0.1325
Honduras	2005	Crush injury	0.1325
Honduras	2010	Crush injury	0.1325
Honduras	2017	Crush injury	0.1325
Honduras	1990	Nerve injury	0.1129
Honduras	1995	Nerve injury	0.1129
Honduras	2000	Nerve injury	0.1129
Honduras	2005	Nerve injury	0.1129
Honduras	2010	Nerve injury	0.1129
Honduras	2017	Nerve injury	0.1129
Honduras	1990	Injury to eyes	0.0165
Honduras	1995	Injury to eyes	0.0165
Honduras	2000	Injury to eyes	0.0165
Honduras	2005	Injury to eyes	0.0165
Honduras	2010	Injury to eyes	0.0165
Honduras	2017	Injury to eyes	0.0165
Honduras	1990	Amputation of lower limb, unilateral	0.1201
Honduras	1995	Amputation of lower limb, unilateral	0.1084
Honduras	2000	Amputation of lower limb, unilateral	0.1028
Honduras	2005	Amputation of lower limb, unilateral	0.0984
Honduras	2010	Amputation of lower limb, unilateral	0.0935
Honduras	2017	Amputation of lower limb, unilateral	0.0875
Honduras	1990	Open wound(s)	0.011
Honduras	1995	Open wound(s)	0.011
Honduras	2000	Open wound(s)	0.011
Honduras	2005	Open wound(s)	0.011
Honduras	2010	Open wound(s)	0.011
Honduras	2017	Open wound(s)	0.011
Honduras	1990	Poisoning requiring urgent care	0.1628
Honduras	1995	Poisoning requiring urgent care	0.1628
Honduras	2000	Poisoning requiring urgent care	0.1628
Honduras	2005	Poisoning requiring urgent care	0.1628
Honduras	2010	Poisoning requiring urgent care	0.1628
Honduras	2017	Poisoning requiring urgent care	0.1628
Honduras	1990	Severe chest injury	0.0474
Honduras	1995	Severe chest injury	0.0474
Honduras	2000	Severe chest injury	0.0474
Honduras	2005	Severe chest injury	0.0474
Honduras	2010	Severe chest injury	0.0474
Honduras	2017	Severe chest injury	0.0474
Honduras	1990	Internal hemorrhage in abdomen and pelvis	0.1143
Honduras	1995	Internal hemorrhage in abdomen and pelvis	0.1143
Honduras	2000	Internal hemorrhage in abdomen and pelvis	0.1143
Honduras	2005	Internal hemorrhage in abdomen and pelvis	0.1143
Honduras	2010	Internal hemorrhage in abdomen and pelvis	0.1143
Honduras	2017	Internal hemorrhage in abdomen and pelvis	0.1143
Honduras	1990	Contusion in any part of the body	0.0075
Honduras	1995	Contusion in any part of the body	0.0075
Honduras	2000	Contusion in any part of the body	0.0075
Honduras	2005	Contusion in any part of the body	0.0075
Honduras	2010	Contusion in any part of the body	0.0075
Honduras	2017	Contusion in any part of the body	0.0075
Honduras	1990	Effect of different environmental factors	0.049
Honduras	1995	Effect of different environmental factors	0.049
Honduras	2000	Effect of different environmental factors	0.049
Honduras	2005	Effect of different environmental factors	0.049
Honduras	2010	Effect of different environmental factors	0.049
Honduras	2017	Effect of different environmental factors	0.049
Honduras	1990	Complications following therapeutic procedures	0.0506
Honduras	1995	Complications following therapeutic procedures	0.0506
Honduras	2000	Complications following therapeutic procedures	0.0506
Honduras	2005	Complications following therapeutic procedures	0.0506
Honduras	2010	Complications following therapeutic procedures	0.0506
Honduras	2017	Complications following therapeutic procedures	0.0506
Honduras	1990	Multiple fractures, dislocations, crashes, wounds, sprains, and strains	0.2659
Honduras	1995	Multiple fractures, dislocations, crashes, wounds, sprains, and strains	0.2358
Honduras	2000	Multiple fractures, dislocations, crashes, wounds, sprains, and strains	0.2216
Honduras	2005	Multiple fractures, dislocations, crashes, wounds, sprains, and strains	0.2101
Honduras	2010	Multiple fractures, dislocations, crashes, wounds, sprains, and strains	0.1976
Honduras	2017	Multiple fractures, dislocations, crashes, wounds, sprains, and strains	0.1822
Honduras	1990	Amputation of upper limb, unilateral	0.0867
Honduras	1995	Amputation of upper limb, unilateral	0.0798
Honduras	2000	Amputation of upper limb, unilateral	0.0766
Honduras	2005	Amputation of upper limb, unilateral	0.0739
Honduras	2010	Amputation of upper limb, unilateral	0.0711
Honduras	2017	Amputation of upper limb, unilateral	0.0675
Honduras	1990	Amputation of thumb	0.0114
Honduras	1995	Amputation of thumb	0.0114
Honduras	2000	Amputation of thumb	0.0114
Honduras	2005	Amputation of thumb	0.0114
Honduras	2010	Amputation of thumb	0.0114
Honduras	2017	Amputation of thumb	0.0114
Honduras	1990	Amputation of toe/toes	0.0059
Honduras	1995	Amputation of toe/toes	0.0059
Honduras	2000	Amputation of toe/toes	0.0059
Honduras	2005	Amputation of toe/toes	0.0059
Honduras	2010	Amputation of toe/toes	0.0059
Honduras	2017	Amputation of toe/toes	0.0059
Honduras	1990	Burns, <20% total burned surface area without lower airway burns	0.0163
Honduras	1995	Burns, <20% total burned surface area without lower airway burns	0.0163
Honduras	2000	Burns, <20% total burned surface area without lower airway burns	0.0163
Honduras	2005	Burns, <20% total burned surface area without lower airway burns	0.0163
Honduras	2010	Burns, <20% total burned surface area without lower airway burns	0.0163
Honduras	2017	Burns, <20% total burned surface area without lower airway burns	0.0163
Honduras	1990	Burns, >=20% total burned surface area or >= 10% burned surface area if head/neck or hands/wrist involved w/o lower airway burns	0.3284
Honduras	1995	Burns, >=20% total burned surface area or >= 10% burned surface area if head/neck or hands/wrist involved w/o lower airway burns	0.3004
Honduras	2000	Burns, >=20% total burned surface area or >= 10% burned surface area if head/neck or hands/wrist involved w/o lower airway burns	0.2872
Honduras	2005	Burns, >=20% total burned surface area or >= 10% burned surface area if head/neck or hands/wrist involved w/o lower airway burns	0.2765
Honduras	2010	Burns, >=20% total burned surface area or >= 10% burned surface area if head/neck or hands/wrist involved w/o lower airway burns	0.2648
Honduras	2017	Burns, >=20% total burned surface area or >= 10% burned surface area if head/neck or hands/wrist involved w/o lower airway burns	0.2505
Nicaragua	1990	Amputation of lower limbs, bilateral	0.231
Nicaragua	1995	Amputation of lower limbs, bilateral	0.2237
Nicaragua	2000	Amputation of lower limbs, bilateral	0.2082
Nicaragua	2005	Amputation of lower limbs, bilateral	0.195
Nicaragua	2010	Amputation of lower limbs, bilateral	0.1822
Nicaragua	2017	Amputation of lower limbs, bilateral	0.1676
Nicaragua	1990	Lower airway burns	0.2252
Nicaragua	1995	Lower airway burns	0.2252
Nicaragua	2000	Lower airway burns	0.2252
Nicaragua	2005	Lower airway burns	0.2252
Nicaragua	2010	Lower airway burns	0.2252
Nicaragua	2017	Lower airway burns	0.2252
Nicaragua	1990	Dislocation of hip	0.0159
Nicaragua	1995	Dislocation of hip	0.0159
Nicaragua	2000	Dislocation of hip	0.0159
Nicaragua	2005	Dislocation of hip	0.0159
Nicaragua	2010	Dislocation of hip	0.0159
Nicaragua	2017	Dislocation of hip	0.0159
Nicaragua	1990	Dislocation of knee	0.1134
Nicaragua	1995	Dislocation of knee	0.1134
Nicaragua	2000	Dislocation of knee	0.1134
Nicaragua	2005	Dislocation of knee	0.1134
Nicaragua	2010	Dislocation of knee	0.1134
Nicaragua	2017	Dislocation of knee	0.1134
Nicaragua	1990	Dislocation of shoulder	0.062
Nicaragua	1995	Dislocation of shoulder	0.062
Nicaragua	2000	Dislocation of shoulder	0.062
Nicaragua	2005	Dislocation of shoulder	0.062
Nicaragua	2010	Dislocation of shoulder	0.062
Nicaragua	2017	Dislocation of shoulder	0.062
Nicaragua	1990	Muscle and tendon injuries, including sprains and strains lesser dislocations	0.0075
Nicaragua	1995	Muscle and tendon injuries, including sprains and strains lesser dislocations	0.0075
Nicaragua	2000	Muscle and tendon injuries, including sprains and strains lesser dislocations	0.0075
Nicaragua	2005	Muscle and tendon injuries, including sprains and strains lesser dislocations	0.0075
Nicaragua	2010	Muscle and tendon injuries, including sprains and strains lesser dislocations	0.0075
Nicaragua	2017	Muscle and tendon injuries, including sprains and strains lesser dislocations	0.0075
Nicaragua	1990	Fracture of clavicle, scapula, or humerus	0.0349
Nicaragua	1995	Fracture of clavicle, scapula, or humerus	0.0349
Nicaragua	2000	Fracture of clavicle, scapula, or humerus	0.0349
Nicaragua	2005	Fracture of clavicle, scapula, or humerus	0.0349
Nicaragua	2010	Fracture of clavicle, scapula, or humerus	0.0349
Nicaragua	2017	Fracture of clavicle, scapula, or humerus	0.0349
Nicaragua	1990	Fracture of face bones	0.0669
Nicaragua	1995	Fracture of face bones	0.0669
Nicaragua	2000	Fracture of face bones	0.0669
Nicaragua	2005	Fracture of face bones	0.0669
Nicaragua	2010	Fracture of face bones	0.0669
Nicaragua	2017	Fracture of face bones	0.0669
Nicaragua	1990	Fracture of foot bones except ankle	0.0264
Nicaragua	1995	Fracture of foot bones except ankle	0.0264
Nicaragua	2000	Fracture of foot bones except ankle	0.0264
Nicaragua	2005	Fracture of foot bones except ankle	0.0264
Nicaragua	2010	Fracture of foot bones except ankle	0.0264
Nicaragua	2017	Fracture of foot bones except ankle	0.0264
Nicaragua	1990	Fracture of hand (wrist and other distal part of hand)	0.0138
Nicaragua	1995	Fracture of hand (wrist and other distal part of hand)	0.0138
Nicaragua	2000	Fracture of hand (wrist and other distal part of hand)	0.0138
Nicaragua	2005	Fracture of hand (wrist and other distal part of hand)	0.0138
Nicaragua	2010	Fracture of hand (wrist and other distal part of hand)	0.0138
Nicaragua	2017	Fracture of hand (wrist and other distal part of hand)	0.0138
Nicaragua	1990	Fracture of hip	0.2002
Nicaragua	1995	Fracture of hip	0.1905
Nicaragua	2000	Fracture of hip	0.1701
Nicaragua	2005	Fracture of hip	0.1525
Nicaragua	2010	Fracture of hip	0.1357
Nicaragua	2017	Fracture of hip	0.1164
Nicaragua	1990	Amputation of upper limbs, bilateral	0.2348
Nicaragua	1995	Amputation of upper limbs, bilateral	0.2248
Nicaragua	2000	Amputation of upper limbs, bilateral	0.2037
Nicaragua	2005	Amputation of upper limbs, bilateral	0.1856
Nicaragua	2010	Amputation of upper limbs, bilateral	0.1682
Nicaragua	2017	Amputation of upper limbs, bilateral	0.1482
Nicaragua	1990	Fracture of patella, tibia or fibula, or ankle	0.0554
Nicaragua	1995	Fracture of patella, tibia or fibula, or ankle	0.0554
Nicaragua	2000	Fracture of patella, tibia or fibula, or ankle	0.0554
Nicaragua	2005	Fracture of patella, tibia or fibula, or ankle	0.0554
Nicaragua	2010	Fracture of patella, tibia or fibula, or ankle	0.0554
Nicaragua	2017	Fracture of patella, tibia or fibula, or ankle	0.0554
Nicaragua	1990	Fracture of pelvis	0.1823
Nicaragua	1995	Fracture of pelvis	0.1823
Nicaragua	2000	Fracture of pelvis	0.1823
Nicaragua	2005	Fracture of pelvis	0.1823
Nicaragua	2010	Fracture of pelvis	0.1823
Nicaragua	2017	Fracture of pelvis	0.1823
Nicaragua	1990	Fracture of radius and/or ulna	0.0435
Nicaragua	1995	Fracture of radius and/or ulna	0.0435
Nicaragua	2000	Fracture of radius and/or ulna	0.0435
Nicaragua	2005	Fracture of radius and/or ulna	0.0435
Nicaragua	2010	Fracture of radius and/or ulna	0.0435
Nicaragua	2017	Fracture of radius and/or ulna	0.0435
Nicaragua	1990	Fracture of skull	0.0714
Nicaragua	1995	Fracture of skull	0.0714
Nicaragua	2000	Fracture of skull	0.0714
Nicaragua	2005	Fracture of skull	0.0714
Nicaragua	2010	Fracture of skull	0.0714
Nicaragua	2017	Fracture of skull	0.0714
Nicaragua	1990	Fracture of sternum and/or fracture of one or more ribs	0.1027
Nicaragua	1995	Fracture of sternum and/or fracture of one or more ribs	0.1027
Nicaragua	2000	Fracture of sternum and/or fracture of one or more ribs	0.1027
Nicaragua	2005	Fracture of sternum and/or fracture of one or more ribs	0.1027
Nicaragua	2010	Fracture of sternum and/or fracture of one or more ribs	0.1027
Nicaragua	2017	Fracture of sternum and/or fracture of one or more ribs	0.1027
Nicaragua	1990	Fracture of vertebral column	0.1106
Nicaragua	1995	Fracture of vertebral column	0.1106
Nicaragua	2000	Fracture of vertebral column	0.1106
Nicaragua	2005	Fracture of vertebral column	0.1106
Nicaragua	2010	Fracture of vertebral column	0.1106
Nicaragua	2017	Fracture of vertebral column	0.1106
Nicaragua	1990	Fracture of femur, other than femoral neck	0.042
Nicaragua	1995	Fracture of femur, other than femoral neck	0.042
Nicaragua	2000	Fracture of femur, other than femoral neck	0.042
Nicaragua	2005	Fracture of femur, other than femoral neck	0.042
Nicaragua	2010	Fracture of femur, other than femoral neck	0.042
Nicaragua	2017	Fracture of femur, other than femoral neck	0.042
Nicaragua	1990	Minor TBI	0.1319
Nicaragua	1995	Minor TBI	0.1319
Nicaragua	2000	Minor TBI	0.1319
Nicaragua	2005	Minor TBI	0.1319
Nicaragua	2010	Minor TBI	0.1319
Nicaragua	2017	Minor TBI	0.1319
Nicaragua	1990	Moderate TBI	0.1637
Nicaragua	1995	Moderate TBI	0.1637
Nicaragua	2000	Moderate TBI	0.1637
Nicaragua	2005	Moderate TBI	0.1637
Nicaragua	2010	Moderate TBI	0.1637
Nicaragua	2017	Moderate TBI	0.1637
Nicaragua	1990	Amputation of fingers (excluding thumb)	0.0047
Nicaragua	1995	Amputation of fingers (excluding thumb)	0.0047
Nicaragua	2000	Amputation of fingers (excluding thumb)	0.0047
Nicaragua	2005	Amputation of fingers (excluding thumb)	0.0047
Nicaragua	2010	Amputation of fingers (excluding thumb)	0.0047
Nicaragua	2017	Amputation of fingers (excluding thumb)	0.0047
Nicaragua	1990	Spinal cord lesion at neck level	0.4611
Nicaragua	1995	Spinal cord lesion at neck level	0.4578
Nicaragua	2000	Spinal cord lesion at neck level	0.4507
Nicaragua	2005	Spinal cord lesion at neck level	0.4446
Nicaragua	2010	Spinal cord lesion at neck level	0.4388
Nicaragua	2017	Spinal cord lesion at neck level	0.4321
Nicaragua	1990	Spinal cord lesion below neck level	0.2817
Nicaragua	1995	Spinal cord lesion below neck level	0.2746
Nicaragua	2000	Spinal cord lesion below neck level	0.2597
Nicaragua	2005	Spinal cord lesion below neck level	0.2469
Nicaragua	2010	Spinal cord lesion below neck level	0.2346
Nicaragua	2017	Spinal cord lesion below neck level	0.2205

Nicaragua	1990	Drowning and nonfatal submersion	0.2471
Nicaragua	1995	Drowning and nonfatal submersion	0.2471
Nicaragua	2000	Drowning and nonfatal submersion	0.2471
Nicaragua	2005	Drowning and nonfatal submersion	0.2471
Nicaragua	2010	Drowning and nonfatal submersion	0.2471
Nicaragua	2017	Drowning and nonfatal submersion	0.2471
Nicaragua	1990	Asphyxiation	0.2471
Nicaragua	1995	Asphyxiation	0.2471
Nicaragua	2000	Asphyxiation	0.2471
Nicaragua	2005	Asphyxiation	0.2471
Nicaragua	2010	Asphyxiation	0.2471
Nicaragua	2017	Asphyxiation	0.2471
Nicaragua	1990	Crush injury	0.1325
Nicaragua	1995	Crush injury	0.1325
Nicaragua	2000	Crush injury	0.1325
Nicaragua	2005	Crush injury	0.1325
Nicaragua	2010	Crush injury	0.1325
Nicaragua	2017	Crush injury	0.1325
Nicaragua	1990	Nerve injury	0.1129
Nicaragua	1995	Nerve injury	0.1129
Nicaragua	2000	Nerve injury	0.1129
Nicaragua	2005	Nerve injury	0.1129
Nicaragua	2010	Nerve injury	0.1129
Nicaragua	2017	Nerve injury	0.1129
Nicaragua	1990	Injury to eyes	0.0165
Nicaragua	1995	Injury to eyes	0.0165
Nicaragua	2000	Injury to eyes	0.0165
Nicaragua	2005	Injury to eyes	0.0165
Nicaragua	2010	Injury to eyes	0.0165
Nicaragua	2017	Injury to eyes	0.0165
Nicaragua	1990	Amputation of lower limb, unilateral	0.0945
Nicaragua	1995	Amputation of lower limb, unilateral	0.0907
Nicaragua	2000	Amputation of lower limb, unilateral	0.0828
Nicaragua	2005	Amputation of lower limb, unilateral	0.0759
Nicaragua	2010	Amputation of lower limb, unilateral	0.0694
Nicaragua	2017	Amputation of lower limb, unilateral	0.0618
Nicaragua	1990	Open wound(s)	0.011
Nicaragua	1995	Open wound(s)	0.011
Nicaragua	2000	Open wound(s)	0.011
Nicaragua	2005	Open wound(s)	0.011
Nicaragua	2010	Open wound(s)	0.011
Nicaragua	2017	Open wound(s)	0.011
Nicaragua	1990	Poisoning requiring urgent care	0.1628
Nicaragua	1995	Poisoning requiring urgent care	0.1628
Nicaragua	2000	Poisoning requiring urgent care	0.1628
Nicaragua	2005	Poisoning requiring urgent care	0.1628
Nicaragua	2010	Poisoning requiring urgent care	0.1628
Nicaragua	2017	Poisoning requiring urgent care	0.1628
Nicaragua	1990	Severe chest injury	0.0474
Nicaragua	1995	Severe chest injury	0.0474
Nicaragua	2000	Severe chest injury	0.0474
Nicaragua	2005	Severe chest injury	0.0474
Nicaragua	2010	Severe chest injury	0.0474
Nicaragua	2017	Severe chest injury	0.0474
Nicaragua	1990	Internal hemorrhage in abdomen and pelvis	0.1143
Nicaragua	1995	Internal hemorrhage in abdomen and pelvis	0.1143
Nicaragua	2000	Internal hemorrhage in abdomen and pelvis	0.1143
Nicaragua	2005	Internal hemorrhage in abdomen and pelvis	0.1143
Nicaragua	2010	Internal hemorrhage in abdomen and pelvis	0.1143
Nicaragua	2017	Internal hemorrhage in abdomen and pelvis	0.1143
Nicaragua	1990	Contusion in any part of the body	0.0075
Nicaragua	1995	Contusion in any part of the body	0.0075
Nicaragua	2000	Contusion in any part of the body	0.0075
Nicaragua	2005	Contusion in any part of the body	0.0075
Nicaragua	2010	Contusion in any part of the body	0.0075
Nicaragua	2017	Contusion in any part of the body	0.0075
Nicaragua	1990	Effect of different environmental factors	0.049
Nicaragua	1995	Effect of different environmental factors	0.049
Nicaragua	2000	Effect of different environmental factors	0.049
Nicaragua	2005	Effect of different environmental factors	0.049
Nicaragua	2010	Effect of different environmental factors	0.049
Nicaragua	2017	Effect of different environmental factors	0.049
Nicaragua	1990	Complications following therapeutic procedures	0.0506
Nicaragua	1995	Complications following therapeutic procedures	0.0506
Nicaragua	2000	Complications following therapeutic procedures	0.0506
Nicaragua	2005	Complications following therapeutic procedures	0.0506
Nicaragua	2010	Complications following therapeutic procedures	0.0506
Nicaragua	2017	Complications following therapeutic procedures	0.0506
Nicaragua	1990	Multiple fractures, dislocations, crashes, wounds, sprains, and strains	0.2002
Nicaragua	1995	Multiple fractures, dislocations, crashes, wounds, sprains, and strains	0.1905
Nicaragua	2000	Multiple fractures, dislocations, crashes, wounds, sprains, and strains	0.1701
Nicaragua	2005	Multiple fractures, dislocations, crashes, wounds, sprains, and strains	0.1525
Nicaragua	2010	Multiple fractures, dislocations, crashes, wounds, sprains, and strains	0.1357
Nicaragua	2017	Multiple fractures, dislocations, crashes, wounds, sprains, and strains	0.1164
Nicaragua	1990	Amputation of upper limb, unilateral	0.0717
Nicaragua	1995	Amputation of upper limb, unilateral	0.0695
Nicaragua	2000	Amputation of upper limb, unilateral	0.0648
Nicaragua	2005	Amputation of upper limb, unilateral	0.0607
Nicaragua	2010	Amputation of upper limb, unilateral	0.0569
Nicaragua	2017	Amputation of upper limb, unilateral	0.0524
Nicaragua	1990	Amputation of thumb	0.0114
Nicaragua	1995	Amputation of thumb	0.0114
Nicaragua	2000	Amputation of thumb	0.0114
Nicaragua	2005	Amputation of thumb	0.0114
Nicaragua	2010	Amputation of thumb	0.0114
Nicaragua	2017	Amputation of thumb	0.0114
Nicaragua	1990	Amputation of toe/toes	0.0059
Nicaragua	1995	Amputation of toe/toes	0.0059
Nicaragua	2000	Amputation of toe/toes	0.0059
Nicaragua	2005	Amputation of toe/toes	0.0059
Nicaragua	2010	Amputation of toe/toes	0.0059
Nicaragua	2017	Amputation of toe/toes	0.0059
Nicaragua	1990	Burns, <20% total burned surface area without lower airway burns	0.0163
Nicaragua	1995	Burns, <20% total burned surface area without lower airway burns	0.0163
Nicaragua	2000	Burns, <20% total burned surface area without lower airway burns	0.0163
Nicaragua	2005	Burns, <20% total burned surface area without lower airway burns	0.0163
Nicaragua	2010	Burns, <20% total burned surface area without lower airway burns	0.0163
Nicaragua	2017	Burns, <20% total burned surface area without lower airway burns	0.0163
Nicaragua	1990	Burns, >=20% total burned surface area or >= 10% burned surface area if head/neck or hands/wrist involved w/o lower airway burns	0.2673
Nicaragua	1995	Burns, >=20% total burned surface area or >= 10% burned surface area if head/neck or hands/wrist involved w/o lower airway burns	0.2583
Nicaragua	2000	Burns, >=20% total burned surface area or >= 10% burned surface area if head/neck or hands/wrist involved w/o lower airway burns	0.2392
Nicaragua	2005	Burns, >=20% total burned surface area or >= 10% burned surface area if head/neck or hands/wrist involved w/o lower airway burns	0.2229
Nicaragua	2010	Burns, >=20% total burned surface area or >= 10% burned surface area if head/neck or hands/wrist involved w/o lower airway burns	0.2072
Nicaragua	2017	Burns, >=20% total burned surface area or >= 10% burned surface area if head/neck or hands/wrist involved w/o lower airway burns	0.1892
Panama	1990	Amputation of lower limbs, bilateral	0.2002
Panama	1995	Amputation of lower limbs, bilateral	0.1834
Panama	2000	Amputation of lower limbs, bilateral	0.1714
Panama	2005	Amputation of lower limbs, bilateral	0.166
Panama	2010	Amputation of lower limbs, bilateral	0.162
Panama	2017	Amputation of lower limbs, bilateral	0.1435
Panama	1990	Lower airway burns	0.2252
Panama	1995	Lower airway burns	0.2252
Panama	2000	Lower airway burns	0.2252
Panama	2005	Lower airway burns	0.2252
Panama	2010	Lower airway burns	0.2252
Panama	2017	Lower airway burns	0.2252
Panama	1990	Dislocation of hip	0.0159
Panama	1995	Dislocation of hip	0.0159
Panama	2000	Dislocation of hip	0.0159
Panama	2005	Dislocation of hip	0.0159
Panama	2010	Dislocation of hip	0.0159
Panama	2017	Dislocation of hip	0.0159
Panama	1990	Dislocation of knee	0.1134
Panama	1995	Dislocation of knee	0.1134
Panama	2000	Dislocation of knee	0.1134
Panama	2005	Dislocation of knee	0.1134
Panama	2010	Dislocation of knee	0.1134
Panama	2017	Dislocation of knee	0.1134
Panama	1990	Dislocation of shoulder	0.062
Panama	1995	Dislocation of shoulder	0.062
Panama	2000	Dislocation of shoulder	0.062
Panama	2005	Dislocation of shoulder	0.062
Panama	2010	Dislocation of shoulder	0.062
Panama	2017	Dislocation of shoulder	0.062
Panama	1990	Muscle and tendon injuries, including sprains and strains lesser dislocations	0.0075
Panama	1995	Muscle and tendon injuries, including sprains and strains lesser dislocations	0.0075
Panama	2000	Muscle and tendon injuries, including sprains and strains lesser dislocations	0.0075
Panama	2005	Muscle and tendon injuries, including sprains and strains lesser dislocations	0.0075
Panama	2010	Muscle and tendon injuries, including sprains and strains lesser dislocations	0.0075
Panama	2017	Muscle and tendon injuries, including sprains and strains lesser dislocations	0.0075
Panama	1990	Fracture of clavicle, scapula, or humerus	0.0349
Panama	1995	Fracture of clavicle, scapula, or humerus	0.0349
Panama	2000	Fracture of clavicle, scapula, or humerus	0.0349
Panama	2005	Fracture of clavicle, scapula, or humerus	0.0349
Panama	2010	Fracture of clavicle, scapula, or humerus	0.0349
Panama	2017	Fracture of clavicle, scapula, or humerus	0.0349
Panama	1990	Fracture of face bones	0.0669
Panama	1995	Fracture of face bones	0.0669
Panama	2000	Fracture of face bones	0.0669
Panama	2005	Fracture of face bones	0.0669
Panama	2010	Fracture of face bones	0.0669
Panama	2017	Fracture of face bones	0.0669
Panama	1990	Fracture of foot bones except ankle	0.0264
Panama	1995	Fracture of foot bones except ankle	0.0264
Panama	2000	Fracture of foot bones except ankle	0.0264
Panama	2005	Fracture of foot bones except ankle	0.0264
Panama	2010	Fracture of foot bones except ankle	0.0264
Panama	2017	Fracture of foot bones except ankle	0.0264
Panama	1990	Fracture of hand (wrist and other distal part of hand)	0.0138
Panama	1995	Fracture of hand (wrist and other distal part of hand)	0.0138
Panama	2000	Fracture of hand (wrist and other distal part of hand)	0.0138
Panama	2005	Fracture of hand (wrist and other distal part of hand)	0.0138
Panama	2010	Fracture of hand (wrist and other distal part of hand)	0.0138
Panama	2017	Fracture of hand (wrist and other distal part of hand)	0.0138
Panama	1990	Fracture of hip	0.1595
Panama	1995	Fracture of hip	0.1372
Panama	2000	Fracture of hip	0.1214
Panama	2005	Fracture of hip	0.1143
Panama	2010	Fracture of hip	0.1089
Panama	2017	Fracture of hip	0.0845
Panama	1990	Amputation of upper limbs, bilateral	0.1927
Panama	1995	Amputation of upper limbs, bilateral	0.1698
Panama	2000	Amputation of upper limbs, bilateral	0.1534
Panama	2005	Amputation of upper limbs, bilateral	0.1461
Panama	2010	Amputation of upper limbs, bilateral	0.1406
Panama	2017	Amputation of upper limbs, bilateral	0.1153
Panama	1990	Fracture of patella, tibia or fibula, or ankle	0.0554
Panama	1995	Fracture of patella, tibia or fibula, or ankle	0.0554
Panama	2000	Fracture of patella, tibia or fibula, or ankle	0.0554
Panama	2005	Fracture of patella, tibia or fibula, or ankle	0.0554
Panama	2010	Fracture of patella, tibia or fibula, or ankle	0.0554
Panama	2017	Fracture of patella, tibia or fibula, or ankle	0.0554
Panama	1990	Fracture of pelvis	0.1823
Panama	1995	Fracture of pelvis	0.1823
Panama	2000	Fracture of pelvis	0.1823
Panama	2005	Fracture of pelvis	0.1823
Panama	2010	Fracture of pelvis	0.1823
Panama	2017	Fracture of pelvis	0.1823
Panama	1990	Fracture of radius and/or ulna	0.0435
Panama	1995	Fracture of radius and/or ulna	0.0435
Panama	2000	Fracture of radius and/or ulna	0.0435
Panama	2005	Fracture of radius and/or ulna	0.0435
Panama	2010	Fracture of radius and/or ulna	0.0435
Panama	2017	Fracture of radius and/or ulna	0.0435
Panama	1990	Fracture of skull	0.0714
Panama	1995	Fracture of skull	0.0714
Panama	2000	Fracture of skull	0.0714
Panama	2005	Fracture of skull	0.0714
Panama	2010	Fracture of skull	0.0714
Panama	2017	Fracture of skull	0.0714
Panama	1990	Fracture of sternum and/or fracture of one or more ribs	0.1027
Panama	1995	Fracture of sternum and/or fracture of one or more ribs	0.1027
Panama	2000	Fracture of sternum and/or fracture of one or more ribs	0.1027
Panama	2005	Fracture of sternum and/or fracture of one or more ribs	0.1027
Panama	2010	Fracture of sternum and/or fracture of one or more ribs	0.1027
Panama	2017	Fracture of sternum and/or fracture of one or more ribs	0.1027
Panama	1990	Fracture of vertebral column	0.1106
Panama	1995	Fracture of vertebral column	0.1106
Panama	2000	Fracture of vertebral column	0.1106
Panama	2005	Fracture of vertebral column	0.1106
Panama	2010	Fracture of vertebral column	0.1106
Panama	2017	Fracture of vertebral column	0.1106
Panama	1990	Fracture of femur, other than femoral neck	0.042
Panama	1995	Fracture of femur, other than femoral neck	0.042
Panama	2000	Fracture of femur, other than femoral neck	0.042
Panama	2005	Fracture of femur, other than femoral neck	0.042
Panama	2010	Fracture of femur, other than femoral neck	0.042
Panama	2017	Fracture of femur, other than femoral neck	0.042
Panama	1990	Minor TBI	0.1319
Panama	1995	Minor TBI	0.1319
Panama	2000	Minor TBI	0.1319
Panama	2005	Minor TBI	0.1319
Panama	2010	Minor TBI	0.1319
Panama	2017	Minor TBI	0.1319
Panama	1990	Moderate TBI	0.1637
Panama	1995	Moderate TBI	0.1637
Panama	2000	Moderate TBI	0.1637
Panama	2005	Moderate TBI	0.1637
Panama	2010	Moderate TBI	0.1637
Panama	2017	Moderate TBI	0.1637
Panama	1990	Amputation of fingers (excluding thumb)	0.0047
Panama	1995	Amputation of fingers (excluding thumb)	0.0047
Panama	2000	Amputation of fingers (excluding thumb)	0.0047
Panama	2005	Amputation of fingers (excluding thumb)	0.0047
Panama	2010	Amputation of fingers (excluding thumb)	0.0047
Panama	2017	Amputation of fingers (excluding thumb)	0.0047
Panama	1990	Spinal cord lesion at neck level	0.447
Panama	1995	Spinal cord lesion at neck level	0.4393
Panama	2000	Spinal cord lesion at neck level	0.4338
Panama	2005	Spinal cord lesion at neck level	0.4314
Panama	2010	Spinal cord lesion at neck level	0.4295
Panama	2017	Spinal cord lesion at neck level	0.4211
Panama	1990	Spinal cord lesion below neck level	0.2519
Panama	1995	Spinal cord lesion below neck level	0.2357
Panama	2000	Spinal cord lesion below neck level	0.2242
Panama	2005	Spinal cord lesion below neck level	0.219
Panama	2010	Spinal cord lesion below neck level	0.2151
Panama	2017	Spinal cord lesion below neck level	0.1972
Panama	1990	Drowning and nonfatal submersion	0.2471
Panama	1995	Drowning and nonfatal submersion	0.2471
Panama	2000	Drowning and nonfatal submersion	0.2471
Panama	2005	Drowning and nonfatal submersion	0.2471
Panama	2010	Drowning and nonfatal submersion	0.2471
Panama	2017	Drowning and nonfatal submersion	0.2471
Panama	1990	Asphyxiation	0.2471
Panama	1995	Asphyxiation	0.2471
Panama	2000	Asphyxiation	0.2471
Panama	2005	Asphyxiation	0.2471
Panama	2010	Asphyxiation	0.2471
Panama	2017	Asphyxiation	0.2471
Panama	1990	Crush injury	0.1325
Panama	1995	Crush injury	0.1325
Panama	2000	Crush injury	0.1325
Panama	2005	Crush injury	0.1325
Panama	2010	Crush injury	0.1325
Panama	2017	Crush injury	0.1325
Panama	1990	Nerve injury	0.1129
Panama	1995	Nerve injury	0.1129
Panama	2000	Nerve injury	0.1129
Panama	2005	Nerve injury	0.1129
Panama	2010	Nerve injury	0.1129
Panama	2017	Nerve injury	0.1129
Panama	1990	Injury to eyes	0.0165
Panama	1995	Injury to eyes	0.0165
Panama	2000	Injury to eyes	0.0165
Panama	2005	Injury to eyes	0.0165
Panama	2010	Injury to eyes	0.0165
Panama	2017	Injury to eyes	0.0165
Panama	1990	Amputation of lower limb, unilateral	0.0786
Panama	1995	Amputation of lower limb, unilateral	0.07
Panama	2000	Amputation of lower limb, unilateral	0.0638
Panama	2005	Amputation of lower limb, unilateral	0.061
Panama	2010	Amputation of lower limb, unilateral	0.0589
Panama	2017	Amputation of lower limb, unilateral	0.0494
Panama	1990	Open wound(s)	0.011
Panama	1995	Open wound(s)	0.011
Panama	2000	Open wound(s)	0.011
Panama	2005	Open wound(s)	0.011
Panama	2010	Open wound(s)	0.011
Panama	2017	Open wound(s)	0.011
Panama	1990	Poisoning requiring urgent care	0.1628
Panama	1995	Poisoning requiring urgent care	0.1628
Panama	2000	Poisoning requiring urgent care	0.1628
Panama	2005	Poisoning requiring urgent care	0.1628
Panama	2010	Poisoning requiring urgent care	0.1628
Panama	2017	Poisoning requiring urgent care	0.1628
Panama	1990	Severe chest injury	0.0474
Panama	1995	Severe chest injury	0.0474
Panama	2000	Severe chest injury	0.0474
Panama	2005	Severe chest injury	0.0474
Panama	2010	Severe chest injury	0.0474
Panama	2017	Severe chest injury	0.0474
Panama	1990	Internal hemorrhage in abdomen and pelvis	0.1143
Panama	1995	Internal hemorrhage in abdomen and pelvis	0.1143
Panama	2000	Internal hemorrhage in abdomen and pelvis	0.1143
Panama	2005	Internal hemorrhage in abdomen and pelvis	0.1143
Panama	2010	Internal hemorrhage in abdomen and pelvis	0.1143
Panama	2017	Internal hemorrhage in abdomen and pelvis	0.1143
Panama	1990	Contusion in any part of the body	0.0075
Panama	1995	Contusion in any part of the body	0.0075
Panama	2000	Contusion in any part of the body	0.0075
Panama	2005	Contusion in any part of the body	0.0075
Panama	2010	Contusion in any part of the body	0.0075
Panama	2017	Contusion in any part of the body	0.0075
Panama	1990	Effect of different environmental factors	0.049
Panama	1995	Effect of different environmental factors	0.049
Panama	2000	Effect of different environmental factors	0.049
Panama	2005	Effect of different environmental factors	0.049
Panama	2010	Effect of different environmental factors	0.049
Panama	2017	Effect of different environmental factors	0.049
Panama	1990	Complications following therapeutic procedures	0.0506
Panama	1995	Complications following therapeutic procedures	0.0506
Panama	2000	Complications following therapeutic procedures	0.0506
Panama	2005	Complications following therapeutic procedures	0.0506
Panama	2010	Complications following therapeutic procedures	0.0506
Panama	2017	Complications following therapeutic procedures	0.0506
Panama	1990	Multiple fractures, dislocations, crashes, wounds, sprains, and strains	0.1595
Panama	1995	Multiple fractures, dislocations, crashes, wounds, sprains, and strains	0.1372
Panama	2000	Multiple fractures, dislocations, crashes, wounds, sprains, and strains	0.1214
Panama	2005	Multiple fractures, dislocations, crashes, wounds, sprains, and strains	0.1143
Panama	2010	Multiple fractures, dislocations, crashes, wounds, sprains, and strains	0.1089
Panama	2017	Multiple fractures, dislocations, crashes, wounds, sprains, and strains	0.0845
Panama	1990	Amputation of upper limb, unilateral	0.0623
Panama	1995	Amputation of upper limb, unilateral	0.0572
Panama	2000	Amputation of upper limb, unilateral	0.0536
Panama	2005	Amputation of upper limb, unilateral	0.052
Panama	2010	Amputation of upper limb, unilateral	0.0507
Panama	2017	Amputation of upper limb, unilateral	0.0451
Panama	1990	Amputation of thumb	0.0114
Panama	1995	Amputation of thumb	0.0114
Panama	2000	Amputation of thumb	0.0114
Panama	2005	Amputation of thumb	0.0114
Panama	2010	Amputation of thumb	0.0114
Panama	2017	Amputation of thumb	0.0114
Panama	1990	Amputation of toe/toes	0.0059
Panama	1995	Amputation of toe/toes	0.0059
Panama	2000	Amputation of toe/toes	0.0059
Panama	2005	Amputation of toe/toes	0.0059
Panama	2010	Amputation of toe/toes	0.0059
Panama	2017	Amputation of toe/toes	0.0059
Panama	1990	Burns, <20% total burned surface area without lower airway burns	0.0163
Panama	1995	Burns, <20% total burned surface area without lower airway burns	0.0163
Panama	2000	Burns, <20% total burned surface area without lower airway burns	0.0163
Panama	2005	Burns, <20% total burned surface area without lower airway burns	0.0163
Panama	2010	Burns, <20% total burned surface area without lower airway burns	0.0163
Panama	2017	Burns, <20% total burned surface area without lower airway burns	0.0163
Panama	1990	Burns, >=20% total burned surface area or >= 10% burned surface area if head/neck or hands/wrist involved w/o lower airway burns	0.2294
Panama	1995	Burns, >=20% total burned surface area or >= 10% burned surface area if head/neck or hands/wrist involved w/o lower airway burns	0.2086
Panama	2000	Burns, >=20% total burned surface area or >= 10% burned surface area if head/neck or hands/wrist involved w/o lower airway burns	0.1939
Panama	2005	Burns, >=20% total burned surface area or >= 10% burned surface area if head/neck or hands/wrist involved w/o lower airway burns	0.1873
Panama	2010	Burns, >=20% total burned surface area or >= 10% burned surface area if head/neck or hands/wrist involved w/o lower airway burns	0.1823
Panama	2017	Burns, >=20% total burned surface area or >= 10% burned surface area if head/neck or hands/wrist involved w/o lower airway burns	0.1595
Bolivarian Republic of Venezuela	1990	Amputation of lower limbs, bilateral	0.2036
Bolivarian Republic of Venezuela	1995	Amputation of lower limbs, bilateral	0.1859
Bolivarian Republic of Venezuela	2000	Amputation of lower limbs, bilateral	0.1739
Bolivarian Republic of Venezuela	2005	Amputation of lower limbs, bilateral	0.1639
Bolivarian Republic of Venezuela	2010	Amputation of lower limbs, bilateral	0.1541
Bolivarian Republic of Venezuela	2017	Amputation of lower limbs, bilateral	0.1472
Bolivarian Republic of Venezuela	1990	Lower airway burns	0.2252
Bolivarian Republic of Venezuela	1995	Lower airway burns	0.2252
Bolivarian Republic of Venezuela	2000	Lower airway burns	0.2252
Bolivarian Republic of Venezuela	2005	Lower airway burns	0.2252
Bolivarian Republic of Venezuela	2010	Lower airway burns	0.2252
Bolivarian Republic of Venezuela	2017	Lower airway burns	0.2252
Bolivarian Republic of Venezuela	1990	Dislocation of hip	0.0159
Bolivarian Republic of Venezuela	1995	Dislocation of hip	0.0159
Bolivarian Republic of Venezuela	2000	Dislocation of hip	0.0159
Bolivarian Republic of Venezuela	2005	Dislocation of hip	0.0159
Bolivarian Republic of Venezuela	2010	Dislocation of hip	0.0159
Bolivarian Republic of Venezuela	2017	Dislocation of hip	0.0159
Bolivarian Republic of Venezuela	1990	Dislocation of knee	0.1134
Bolivarian Republic of Venezuela	1995	Dislocation of knee	0.1134
Bolivarian Republic of Venezuela	2000	Dislocation of knee	0.1134
Bolivarian Republic of Venezuela	2005	Dislocation of knee	0.1134
Bolivarian Republic of Venezuela	2010	Dislocation of knee	0.1134
Bolivarian Republic of Venezuela	2017	Dislocation of knee	0.1134
Bolivarian Republic of Venezuela	1990	Dislocation of shoulder	0.062
Bolivarian Republic of Venezuela	1995	Dislocation of shoulder	0.062
Bolivarian Republic of Venezuela	2000	Dislocation of shoulder	0.062
Bolivarian Republic of Venezuela	2005	Dislocation of shoulder	0.062
Bolivarian Republic of Venezuela	2010	Dislocation of shoulder	0.062
Bolivarian Republic of Venezuela	2017	Dislocation of shoulder	0.062
Bolivarian Republic of Venezuela	1990	Muscle and tendon injuries, including sprains and strains lesser dislocations	0.0075
Bolivarian Republic of Venezuela	1995	Muscle and tendon injuries, including sprains and strains lesser dislocations	0.0075
Bolivarian Republic of Venezuela	2000	Muscle and tendon injuries, including sprains and strains lesser dislocations	0.0075
Bolivarian Republic of Venezuela	2005	Muscle and tendon injuries, including sprains and strains lesser dislocations	0.0075
Bolivarian Republic of Venezuela	2010	Muscle and tendon injuries, including sprains and strains lesser dislocations	0.0075
Bolivarian Republic of Venezuela	2017	Muscle and tendon injuries, including sprains and strains lesser dislocations	0.0075
Bolivarian Republic of Venezuela	1990	Fracture of clavicle, scapula, or humerus	0.0349
Bolivarian Republic of Venezuela	1995	Fracture of clavicle, scapula, or humerus	0.0349
Bolivarian Republic of Venezuela	2000	Fracture of clavicle, scapula, or humerus	0.0349
Bolivarian Republic of Venezuela	2005	Fracture of clavicle, scapula, or humerus	0.0349
Bolivarian Republic of Venezuela	2010	Fracture of clavicle, scapula, or humerus	0.0349
Bolivarian Republic of Venezuela	2017	Fracture of clavicle, scapula, or humerus	0.0349
Bolivarian Republic of Venezuela	1990	Fracture of face bones	0.0669
Bolivarian Republic of Venezuela	1995	Fracture of face bones	0.0669
Bolivarian Republic of Venezuela	2000	Fracture of face bones	0.0669
Bolivarian Republic of Venezuela	2005	Fracture of face bones	0.0669
Bolivarian Republic of Venezuela	2010	Fracture of face bones	0.0669
Bolivarian Republic of Venezuela	2017	Fracture of face bones	0.0669
Bolivarian Republic of Venezuela	1990	Fracture of foot bones except ankle	0.0264
Bolivarian Republic of Venezuela	1995	Fracture of foot bones except ankle	0.0264
Bolivarian Republic of Venezuela	2000	Fracture of foot bones except ankle	0.0264
Bolivarian Republic of Venezuela	2005	Fracture of foot bones except ankle	0.0264
Bolivarian Republic of Venezuela	2010	Fracture of foot bones except ankle	0.0264
Bolivarian Republic of Venezuela	2017	Fracture of foot bones except ankle	0.0264
Bolivarian Republic of Venezuela	1990	Fracture of hand (wrist and other distal part of hand)	0.0138
Bolivarian Republic of Venezuela	1995	Fracture of hand (wrist and other distal part of hand)	0.0138
Bolivarian Republic of Venezuela	2000	Fracture of hand (wrist and other distal part of hand)	0.0138
Bolivarian Republic of Venezuela	2005	Fracture of hand (wrist and other distal part of hand)	0.0138
Bolivarian Republic of Venezuela	2010	Fracture of hand (wrist and other distal part of hand)	0.0138

Bolivarian Republic of Venezuela	2017	Fracture of hand (wrist and other distal part of hand)	0.0138
Bolivarian Republic of Venezuela	1990	Fracture of hip	0.1639
Bolivarian Republic of Venezuela	1995	Fracture of hip	0.1406
Bolivarian Republic of Venezuela	2000	Fracture of hip	0.1247
Bolivarian Republic of Venezuela	2005	Fracture of hip	0.1115
Bolivarian Republic of Venezuela	2010	Fracture of hip	0.0985
Bolivarian Republic of Venezuela	2017	Fracture of hip	0.0893
Bolivarian Republic of Venezuela	1990	Amputation of upper limbs, bilateral	0.1973
Bolivarian Republic of Venezuela	1995	Amputation of upper limbs, bilateral	0.1733
Bolivarian Republic of Venezuela	2000	Amputation of upper limbs, bilateral	0.1568
Bolivarian Republic of Venezuela	2005	Amputation of upper limbs, bilateral	0.1432
Bolivarian Republic of Venezuela	2010	Amputation of upper limbs, bilateral	0.1297
Bolivarian Republic of Venezuela	2017	Amputation of upper limbs, bilateral	0.1203
Bolivarian Republic of Venezuela	1990	Fracture of patella, tibia or fibula, or ankle	0.0554
Bolivarian Republic of Venezuela	1995	Fracture of patella, tibia or fibula, or ankle	0.0554
Bolivarian Republic of Venezuela	2000	Fracture of patella, tibia or fibula, or ankle	0.0554
Bolivarian Republic of Venezuela	2005	Fracture of patella, tibia or fibula, or ankle	0.0554
Bolivarian Republic of Venezuela	2010	Fracture of patella, tibia or fibula, or ankle	0.0554
Bolivarian Republic of Venezuela	2017	Fracture of patella, tibia or fibula, or ankle	0.0554
Bolivarian Republic of Venezuela	1990	Fracture of pelvis	0.1823
Bolivarian Republic of Venezuela	1995	Fracture of pelvis	0.1823
Bolivarian Republic of Venezuela	2000	Fracture of pelvis	0.1823
Bolivarian Republic of Venezuela	2005	Fracture of pelvis	0.1823
Bolivarian Republic of Venezuela	2010	Fracture of pelvis	0.1823
Bolivarian Republic of Venezuela	2017	Fracture of pelvis	0.1823
Bolivarian Republic of Venezuela	1990	Fracture of radius and/or ulna	0.0435
Bolivarian Republic of Venezuela	1995	Fracture of radius and/or ulna	0.0435
Bolivarian Republic of Venezuela	2000	Fracture of radius and/or ulna	0.0435
Bolivarian Republic of Venezuela	2005	Fracture of radius and/or ulna	0.0435
Bolivarian Republic of Venezuela	2010	Fracture of radius and/or ulna	0.0435
Bolivarian Republic of Venezuela	2017	Fracture of radius and/or ulna	0.0435
Bolivarian Republic of Venezuela	1990	Fracture of skull	0.0714
Bolivarian Republic of Venezuela	1995	Fracture of skull	0.0714
Bolivarian Republic of Venezuela	2000	Fracture of skull	0.0714
Bolivarian Republic of Venezuela	2005	Fracture of skull	0.0714
Bolivarian Republic of Venezuela	2010	Fracture of skull	0.0714
Bolivarian Republic of Venezuela	2017	Fracture of skull	0.0714
Bolivarian Republic of Venezuela	1990	Fracture of sternum and/or fracture of one or more ribs	0.1027
Bolivarian Republic of Venezuela	1995	Fracture of sternum and/or fracture of one or more ribs	0.1027
Bolivarian Republic of Venezuela	2000	Fracture of sternum and/or fracture of one or more ribs	0.1027
Bolivarian Republic of Venezuela	2005	Fracture of sternum and/or fracture of one or more ribs	0.1027
Bolivarian Republic of Venezuela	2010	Fracture of sternum and/or fracture of one or more ribs	0.1027
Bolivarian Republic of Venezuela	2017	Fracture of sternum and/or fracture of one or more ribs	0.1027
Bolivarian Republic of Venezuela	1990	Fracture of vertebral column	0.1106
Bolivarian Republic of Venezuela	1995	Fracture of vertebral column	0.1106
Bolivarian Republic of Venezuela	2000	Fracture of vertebral column	0.1106
Bolivarian Republic of Venezuela	2005	Fracture of vertebral column	0.1106
Bolivarian Republic of Venezuela	2010	Fracture of vertebral column	0.1106
Bolivarian Republic of Venezuela	2017	Fracture of vertebral column	0.1106
Bolivarian Republic of Venezuela	1990	Fracture of femur, other than femoral neck	0.042
Bolivarian Republic of Venezuela	1995	Fracture of femur, other than femoral neck	0.042
Bolivarian Republic of Venezuela	2000	Fracture of femur, other than femoral neck	0.042
Bolivarian Republic of Venezuela	2005	Fracture of femur, other than femoral neck	0.042
Bolivarian Republic of Venezuela	2010	Fracture of femur, other than femoral neck	0.042
Bolivarian Republic of Venezuela	2017	Fracture of femur, other than femoral neck	0.042
Bolivarian Republic of Venezuela	1990	Minor TBI	0.1319
Bolivarian Republic of Venezuela	1995	Minor TBI	0.1319
Bolivarian Republic of Venezuela	2000	Minor TBI	0.1319
Bolivarian Republic of Venezuela	2005	Minor TBI	0.1319
Bolivarian Republic of Venezuela	2010	Minor TBI	0.1319
Bolivarian Republic of Venezuela	2017	Minor TBI	0.1319
Bolivarian Republic of Venezuela	1990	Moderate TBI	0.1637
Bolivarian Republic of Venezuela	1995	Moderate TBI	0.1637
Bolivarian Republic of Venezuela	2000	Moderate TBI	0.1637
Bolivarian Republic of Venezuela	2005	Moderate TBI	0.1637
Bolivarian Republic of Venezuela	2010	Moderate TBI	0.1637
Bolivarian Republic of Venezuela	2017	Moderate TBI	0.1637
Bolivarian Republic of Venezuela	1990	Amputation of fingers (excluding thumb)	0.0047
Bolivarian Republic of Venezuela	1995	Amputation of fingers (excluding thumb)	0.0047
Bolivarian Republic of Venezuela	2000	Amputation of fingers (excluding thumb)	0.0047
Bolivarian Republic of Venezuela	2005	Amputation of fingers (excluding thumb)	0.0047
Bolivarian Republic of Venezuela	2010	Amputation of fingers (excluding thumb)	0.0047
Bolivarian Republic of Venezuela	2017	Amputation of fingers (excluding thumb)	0.0047
Bolivarian Republic of Venezuela	1990	Spinal cord lesion at neck level	0.4486
Bolivarian Republic of Venezuela	1995	Spinal cord lesion at neck level	0.4405
Bolivarian Republic of Venezuela	2000	Spinal cord lesion at neck level	0.435
Bolivarian Republic of Venezuela	2005	Spinal cord lesion at neck level	0.4304
Bolivarian Republic of Venezuela	2010	Spinal cord lesion at neck level	0.4259
Bolivarian Republic of Venezuela	2017	Spinal cord lesion at neck level	0.4228
Bolivarian Republic of Venezuela	1990	Spinal cord lesion below neck level	0.2552
Bolivarian Republic of Venezuela	1995	Spinal cord lesion below neck level	0.2382
Bolivarian Republic of Venezuela	2000	Spinal cord lesion below neck level	0.2265
Bolivarian Republic of Venezuela	2005	Spinal cord lesion below neck level	0.2169
Bolivarian Republic of Venezuela	2010	Spinal cord lesion below neck level	0.2074
Bolivarian Republic of Venezuela	2017	Spinal cord lesion below neck level	0.2008
Bolivarian Republic of Venezuela	1990	Drowning and nonfatal submersion	0.2471
Bolivarian Republic of Venezuela	1995	Drowning and nonfatal submersion	0.2471
Bolivarian Republic of Venezuela	2000	Drowning and nonfatal submersion	0.2471
Bolivarian Republic of Venezuela	2005	Drowning and nonfatal submersion	0.2471
Bolivarian Republic of Venezuela	2010	Drowning and nonfatal submersion	0.2471
Bolivarian Republic of Venezuela	2017	Drowning and nonfatal submersion	0.2471
Bolivarian Republic of Venezuela	1990	Asphyxiation	0.2471
Bolivarian Republic of Venezuela	1995	Asphyxiation	0.2471
Bolivarian Republic of Venezuela	2000	Asphyxiation	0.2471
Bolivarian Republic of Venezuela	2005	Asphyxiation	0.2471
Bolivarian Republic of Venezuela	2010	Asphyxiation	0.2471
Bolivarian Republic of Venezuela	2017	Asphyxiation	0.2471
Bolivarian Republic of Venezuela	1990	Crush injury	0.1325
Bolivarian Republic of Venezuela	1995	Crush injury	0.1325
Bolivarian Republic of Venezuela	2000	Crush injury	0.1325
Bolivarian Republic of Venezuela	2005	Crush injury	0.1325
Bolivarian Republic of Venezuela	2010	Crush injury	0.1325
Bolivarian Republic of Venezuela	2017	Crush injury	0.1325
Bolivarian Republic of Venezuela	1990	Nerve injury	0.1129
Bolivarian Republic of Venezuela	1995	Nerve injury	0.1129
Bolivarian Republic of Venezuela	2000	Nerve injury	0.1129
Bolivarian Republic of Venezuela	2005	Nerve injury	0.1129
Bolivarian Republic of Venezuela	2010	Nerve injury	0.1129
Bolivarian Republic of Venezuela	2017	Nerve injury	0.1129
Bolivarian Republic of Venezuela	1990	Injury to eyes	0.0165
Bolivarian Republic of Venezuela	1995	Injury to eyes	0.0165
Bolivarian Republic of Venezuela	2000	Injury to eyes	0.0165
Bolivarian Republic of Venezuela	2005	Injury to eyes	0.0165
Bolivarian Republic of Venezuela	2010	Injury to eyes	0.0165
Bolivarian Republic of Venezuela	2017	Injury to eyes	0.0165
Bolivarian Republic of Venezuela	1990	Amputation of lower limb, unilateral	0.0804
Bolivarian Republic of Venezuela	1995	Amputation of lower limb, unilateral	0.0713
Bolivarian Republic of Venezuela	2000	Amputation of lower limb, unilateral	0.0651
Bolivarian Republic of Venezuela	2005	Amputation of lower limb, unilateral	0.0599
Bolivarian Republic of Venezuela	2010	Amputation of lower limb, unilateral	0.0549
Bolivarian Republic of Venezuela	2017	Amputation of lower limb, unilateral	0.0513
Bolivarian Republic of Venezuela	1990	Open wound(s)	0.011
Bolivarian Republic of Venezuela	1995	Open wound(s)	0.011
Bolivarian Republic of Venezuela	2000	Open wound(s)	0.011
Bolivarian Republic of Venezuela	2005	Open wound(s)	0.011
Bolivarian Republic of Venezuela	2010	Open wound(s)	0.011
Bolivarian Republic of Venezuela	2017	Open wound(s)	0.011
Bolivarian Republic of Venezuela	1990	Poisoning requiring urgent care	0.1628
Bolivarian Republic of Venezuela	1995	Poisoning requiring urgent care	0.1628
Bolivarian Republic of Venezuela	2000	Poisoning requiring urgent care	0.1628
Bolivarian Republic of Venezuela	2005	Poisoning requiring urgent care	0.1628
Bolivarian Republic of Venezuela	2010	Poisoning requiring urgent care	0.1628
Bolivarian Republic of Venezuela	2017	Poisoning requiring urgent care	0.1628
Bolivarian Republic of Venezuela	1990	Severe chest injury	0.0474
Bolivarian Republic of Venezuela	1995	Severe chest injury	0.0474
Bolivarian Republic of Venezuela	2000	Severe chest injury	0.0474
Bolivarian Republic of Venezuela	2005	Severe chest injury	0.0474
Bolivarian Republic of Venezuela	2010	Severe chest injury	0.0474
Bolivarian Republic of Venezuela	2017	Severe chest injury	0.0474
Bolivarian Republic of Venezuela	1990	Internal hemorrhage in abdomen and pelvis	0.1143
Bolivarian Republic of Venezuela	1995	Internal hemorrhage in abdomen and pelvis	0.1143
Bolivarian Republic of Venezuela	2000	Internal hemorrhage in abdomen and pelvis	0.1143
Bolivarian Republic of Venezuela	2005	Internal hemorrhage in abdomen and pelvis	0.1143
Bolivarian Republic of Venezuela	2010	Internal hemorrhage in abdomen and pelvis	0.1143
Bolivarian Republic of Venezuela	2017	Internal hemorrhage in abdomen and pelvis	0.1143
Bolivarian Republic of Venezuela	1990	Contusion in any part of the body	0.0075
Bolivarian Republic of Venezuela	1995	Contusion in any part of the body	0.0075
Bolivarian Republic of Venezuela	2000	Contusion in any part of the body	0.0075
Bolivarian Republic of Venezuela	2005	Contusion in any part of the body	0.0075
Bolivarian Republic of Venezuela	2010	Contusion in any part of the body	0.0075
Bolivarian Republic of Venezuela	2017	Contusion in any part of the body	0.0075
Bolivarian Republic of Venezuela	1990	Effect of different environmental factors	0.049
Bolivarian Republic of Venezuela	1995	Effect of different environmental factors	0.049
Bolivarian Republic of Venezuela	2000	Effect of different environmental factors	0.049
Bolivarian Republic of Venezuela	2005	Effect of different environmental factors	0.049
Bolivarian Republic of Venezuela	2010	Effect of different environmental factors	0.049
Bolivarian Republic of Venezuela	2017	Effect of different environmental factors	0.049
Bolivarian Republic of Venezuela	1990	Complications following therapeutic procedures	0.0506
Bolivarian Republic of Venezuela	1995	Complications following therapeutic procedures	0.0506
Bolivarian Republic of Venezuela	2000	Complications following therapeutic procedures	0.0506
Bolivarian Republic of Venezuela	2005	Complications following therapeutic procedures	0.0506
Bolivarian Republic of Venezuela	2010	Complications following therapeutic procedures	0.0506
Bolivarian Republic of Venezuela	2017	Complications following therapeutic procedures	0.0506
Bolivarian Republic of Venezuela	1990	Multiple fractures, dislocations, crashes, wounds, sprains, and strains	0.1639
Bolivarian Republic of Venezuela	1995	Multiple fractures, dislocations, crashes, wounds, sprains, and strains	0.1406
Bolivarian Republic of Venezuela	2000	Multiple fractures, dislocations, crashes, wounds, sprains, and strains	0.1247
Bolivarian Republic of Venezuela	2005	Multiple fractures, dislocations, crashes, wounds, sprains, and strains	0.1115
Bolivarian Republic of Venezuela	2010	Multiple fractures, dislocations, crashes, wounds, sprains, and strains	0.0985
Bolivarian Republic of Venezuela	2017	Multiple fractures, dislocations, crashes, wounds, sprains, and strains	0.0893
Bolivarian Republic of Venezuela	1990	Amputation of upper limb, unilateral	0.0634
Bolivarian Republic of Venezuela	1995	Amputation of upper limb, unilateral	0.058
Bolivarian Republic of Venezuela	2000	Amputation of upper limb, unilateral	0.0544
Bolivarian Republic of Venezuela	2005	Amputation of upper limb, unilateral	0.0513
Bolivarian Republic of Venezuela	2010	Amputation of upper limb, unilateral	0.0483
Bolivarian Republic of Venezuela	2017	Amputation of upper limb, unilateral	0.0462
Bolivarian Republic of Venezuela	1990	Amputation of thumb	0.0114
Bolivarian Republic of Venezuela	1995	Amputation of thumb	0.0114
Bolivarian Republic of Venezuela	2000	Amputation of thumb	0.0114
Bolivarian Republic of Venezuela	2005	Amputation of thumb	0.0114
Bolivarian Republic of Venezuela	2010	Amputation of thumb	0.0114
Bolivarian Republic of Venezuela	2017	Amputation of thumb	0.0114
Bolivarian Republic of Venezuela	1990	Amputation of toe/toes	0.0059
Bolivarian Republic of Venezuela	1995	Amputation of toe/toes	0.0059
Bolivarian Republic of Venezuela	2000	Amputation of toe/toes	0.0059
Bolivarian Republic of Venezuela	2005	Amputation of toe/toes	0.0059
Bolivarian Republic of Venezuela	2010	Amputation of toe/toes	0.0059
Bolivarian Republic of Venezuela	2017	Amputation of toe/toes	0.0059
Bolivarian Republic of Venezuela	1990	Burns, <20% total burned surface area without lower airway burns	0.0163
Bolivarian Republic of Venezuela	1995	Burns, <20% total burned surface area without lower airway burns	0.0163
Bolivarian Republic of Venezuela	2000	Burns, <20% total burned surface area without lower airway burns	0.0163
Bolivarian Republic of Venezuela	2005	Burns, <20% total burned surface area without lower airway burns	0.0163
Bolivarian Republic of Venezuela	2010	Burns, <20% total burned surface area without lower airway burns	0.0163
Bolivarian Republic of Venezuela	2017	Burns, <20% total burned surface area without lower airway burns	0.0163
Bolivarian Republic of Venezuela	1990	Burns, >=20% total burned surface area or >= 10% burned surface area if head/neck or hands/wrist involved w/o lower airway burns	0.2335
Bolivarian Republic of Venezuela	1995	Burns, >=20% total burned surface area or >= 10% burned surface area if head/neck or hands/wrist involved w/o lower airway burns	0.2118
Bolivarian Republic of Venezuela	2000	Burns, >=20% total burned surface area or >= 10% burned surface area if head/neck or hands/wrist involved w/o lower airway burns	0.1969
Bolivarian Republic of Venezuela	2005	Burns, >=20% total burned surface area or >= 10% burned surface area if head/neck or hands/wrist involved w/o lower airway burns	0.1847
Bolivarian Republic of Venezuela	2010	Burns, >=20% total burned surface area or >= 10% burned surface area if head/neck or hands/wrist involved w/o lower airway burns	0.1725
Bolivarian Republic of Venezuela	2017	Burns, >=20% total burned surface area or >= 10% burned surface area if head/neck or hands/wrist involved w/o lower airway burns	0.164
Paraguay	1990	Amputation of lower limbs, bilateral	0.2306
Paraguay	1995	Amputation of lower limbs, bilateral	0.2157
Paraguay	2000	Amputation of lower limbs, bilateral	0.2079
Paraguay	2005	Amputation of lower limbs, bilateral	0.2018
Paraguay	2010	Amputation of lower limbs, bilateral	0.1954
Paraguay	2017	Amputation of lower limbs, bilateral	0.1825
Paraguay	1990	Lower airway burns	0.2252
Paraguay	1995	Lower airway burns	0.2252
Paraguay	2000	Lower airway burns	0.2252
Paraguay	2005	Lower airway burns	0.2252
Paraguay	2010	Lower airway burns	0.2252
Paraguay	2017	Lower airway burns	0.2252
Paraguay	1990	Dislocation of hip	0.0159
Paraguay	1995	Dislocation of hip	0.0159
Paraguay	2000	Dislocation of hip	0.0159
Paraguay	2005	Dislocation of hip	0.0159
Paraguay	2010	Dislocation of hip	0.0159
Paraguay	2017	Dislocation of hip	0.0159
Paraguay	1990	Dislocation of knee	0.1134
Paraguay	1995	Dislocation of knee	0.1134
Paraguay	2000	Dislocation of knee	0.1134
Paraguay	2005	Dislocation of knee	0.1134
Paraguay	2010	Dislocation of knee	0.1134
Paraguay	2017	Dislocation of knee	0.1134
Paraguay	1990	Dislocation of shoulder	0.062
Paraguay	1995	Dislocation of shoulder	0.062
Paraguay	2000	Dislocation of shoulder	0.062
Paraguay	2005	Dislocation of shoulder	0.062
Paraguay	2010	Dislocation of shoulder	0.062
Paraguay	2017	Dislocation of shoulder	0.062
Paraguay	1990	Muscle and tendon injuries, including sprains and strains lesser dislocations	0.0075
Paraguay	1995	Muscle and tendon injuries, including sprains and strains lesser dislocations	0.0075
Paraguay	2000	Muscle and tendon injuries, including sprains and strains lesser dislocations	0.0075
Paraguay	2005	Muscle and tendon injuries, including sprains and strains lesser dislocations	0.0075
Paraguay	2010	Muscle and tendon injuries, including sprains and strains lesser dislocations	0.0075
Paraguay	2017	Muscle and tendon injuries, including sprains and strains lesser dislocations	0.0075
Paraguay	1990	Fracture of clavicle, scapula, or humerus	0.0349
Paraguay	1995	Fracture of clavicle, scapula, or humerus	0.0349
Paraguay	2000	Fracture of clavicle, scapula, or humerus	0.0349
Paraguay	2005	Fracture of clavicle, scapula, or humerus	0.0349
Paraguay	2010	Fracture of clavicle, scapula, or humerus	0.0349
Paraguay	2017	Fracture of clavicle, scapula, or humerus	0.0349
Paraguay	1990	Fracture of face bones	0.0669
Paraguay	1995	Fracture of face bones	0.0669
Paraguay	2000	Fracture of face bones	0.0669
Paraguay	2005	Fracture of face bones	0.0669
Paraguay	2010	Fracture of face bones	0.0669
Paraguay	2017	Fracture of face bones	0.0669
Paraguay	1990	Fracture of foot bones except ankle	0.0264
Paraguay	1995	Fracture of foot bones except ankle	0.0264
Paraguay	2000	Fracture of foot bones except ankle	0.0264
Paraguay	2005	Fracture of foot bones except ankle	0.0264
Paraguay	2010	Fracture of foot bones except ankle	0.0264
Paraguay	2017	Fracture of foot bones except ankle	0.0264
Paraguay	1990	Fracture of hand (wrist and other distal part of hand)	0.0138
Paraguay	1995	Fracture of hand (wrist and other distal part of hand)	0.0138
Paraguay	2000	Fracture of hand (wrist and other distal part of hand)	0.0138
Paraguay	2005	Fracture of hand (wrist and other distal part of hand)	0.0138
Paraguay	2010	Fracture of hand (wrist and other distal part of hand)	0.0138
Paraguay	2017	Fracture of hand (wrist and other distal part of hand)	0.0138
Paraguay	1990	Fracture of hip	0.1997
Paraguay	1995	Fracture of hip	0.18
Paraguay	2000	Fracture of hip	0.1697
Paraguay	2005	Fracture of hip	0.1616
Paraguay	2010	Fracture of hip	0.1531
Paraguay	2017	Fracture of hip	0.136
Paraguay	1990	Amputation of upper limbs, bilateral	0.2343
Paraguay	1995	Amputation of upper limbs, bilateral	0.2139
Paraguay	2000	Amputation of upper limbs, bilateral	0.2033
Paraguay	2005	Amputation of upper limbs, bilateral	0.195
Paraguay	2010	Amputation of upper limbs, bilateral	0.1862
Paraguay	2017	Amputation of upper limbs, bilateral	0.1685
Paraguay	1990	Fracture of patella, tibia or fibula, or ankle	0.0554
Paraguay	1995	Fracture of patella, tibia or fibula, or ankle	0.0554
Paraguay	2000	Fracture of patella, tibia or fibula, or ankle	0.0554
Paraguay	2005	Fracture of patella, tibia or fibula, or ankle	0.0554
Paraguay	2010	Fracture of patella, tibia or fibula, or ankle	0.0554
Paraguay	2017	Fracture of patella, tibia or fibula, or ankle	0.0554
Paraguay	1990	Fracture of pelvis	0.1823
Paraguay	1995	Fracture of pelvis	0.1823
Paraguay	2000	Fracture of pelvis	0.1823
Paraguay	2005	Fracture of pelvis	0.1823
Paraguay	2010	Fracture of pelvis	0.1823
Paraguay	2017	Fracture of pelvis	0.1823
Paraguay	1990	Fracture of radius and/or ulna	0.0435
Paraguay	1995	Fracture of radius and/or ulna	0.0435
Paraguay	2000	Fracture of radius and/or ulna	0.0435
Paraguay	2005	Fracture of radius and/or ulna	0.0435
Paraguay	2010	Fracture of radius and/or ulna	0.0435
Paraguay	2017	Fracture of radius and/or ulna	0.0435
Paraguay	1990	Fracture of skull	0.0714
Paraguay	1995	Fracture of skull	0.0714
Paraguay	2000	Fracture of skull	0.0714
Paraguay	2005	Fracture of skull	0.0714
Paraguay	2010	Fracture of skull	0.0714
Paraguay	2017	Fracture of skull	0.0714
Paraguay	1990	Fracture of sternum and/or fracture of one or more ribs	0.1027
Paraguay	1995	Fracture of sternum and/or fracture of one or more ribs	0.1027
Paraguay	2000	Fracture of sternum and/or fracture of one or more ribs	0.1027
Paraguay	2005	Fracture of sternum and/or fracture of one or more ribs	0.1027
Paraguay	2010	Fracture of sternum and/or fracture of one or more ribs	0.1027
Paraguay	2017	Fracture of sternum and/or fracture of one or more ribs	0.1027
Paraguay	1990	Fracture of vertebral column	0.1106
Paraguay	1995	Fracture of vertebral column	0.1106
Paraguay	2000	Fracture of vertebral column	0.1106
Paraguay	2005	Fracture of vertebral column	0.1106
Paraguay	2010	Fracture of vertebral column	0.1106
Paraguay	2017	Fracture of vertebral column	0.1106
Paraguay	1990	Fracture of femur, other than femoral neck	0.042
Paraguay	1995	Fracture of femur, other than femoral neck	0.042
Paraguay	2000	Fracture of femur, other than femoral neck	0.042
Paraguay	2005	Fracture of femur, other than femoral neck	0.042
Paraguay	2010	Fracture of femur, other than femoral neck	0.042
Paraguay	2017	Fracture of femur, other than femoral neck	0.042
Paraguay	1990	Minor TBI	0.1319
Paraguay	1995	Minor TBI	0.1319
Paraguay	2000	Minor TBI	0.1319
Paraguay	2005	Minor TBI	0.1319
Paraguay	2010	Minor TBI	0.1319
Paraguay	2017	Minor TBI	0.1319
Paraguay	1990	Moderate TBI	0.1637
Paraguay	1995	Moderate TBI	0.1637
Paraguay	2000	Moderate TBI	0.1637
Paraguay	2005	Moderate TBI	0.1637
Paraguay	2010	Moderate TBI	0.1637
Paraguay	2017	Moderate TBI	0.1637
Paraguay	1990	Amputation of fingers (excluding thumb)	0.0047
Paraguay	1995	Amputation of fingers (excluding thumb)	0.0047
Paraguay	2000	Amputation of fingers (excluding thumb)	0.0047
Paraguay	2005	Amputation of fingers (excluding thumb)	0.0047
Paraguay	2010	Amputation of fingers (excluding thumb)	0.0047
Paraguay	2017	Amputation of fingers (excluding thumb)	0.0047
Paraguay	1990	Spinal cord lesion at neck level	0.4609
Paraguay	1995	Spinal cord lesion at neck level	0.4541
Paraguay	2000	Spinal cord lesion at neck level	0.4505
Paraguay	2005	Spinal cord lesion at neck level	0.4478
Paraguay	2010	Spinal cord lesion at neck level	0.4448
Paraguay	2017	Spinal cord lesion at neck level	0.4389
Paraguay	1990	Spinal cord lesion below neck level	0.2813
Paraguay	1995	Spinal cord lesion below neck level	0.2669
Paraguay	2000	Spinal cord lesion below neck level	0.2584
Paraguay	2005	Spinal cord lesion below neck level	0.2535
Paraguay	2010	Spinal cord lesion below neck level	0.2473
Paraguay	2017	Spinal cord lesion below neck level	0.2349
Paraguay	1990	Drowning and nonfatal submersion	0.2471
Paraguay	1995	Drowning and nonfatal submersion	0.2471
Paraguay	2000	Drowning and nonfatal submersion	0.2471
Paraguay	2005	Drowning and nonfatal submersion	0.2471
Paraguay	2010	Drowning and nonfatal submersion	0.2471
Paraguay	2017	Drowning and nonfatal submersion	0.2471
Paraguay	1990	Asphyxiation	0.2471
Paraguay	1995	Asphyxiation	0.2471
Paraguay	2000	Asphyxiation	0.2471
Paraguay	2005	Asphyxiation	0.2471
Paraguay	2010	Asphyxiation	0.2471
Paraguay	2017	Asphyxiation	0.2471
Paraguay	1990	Crush injury	0.1325
Paraguay	1995	Crush injury	0.1325
Paraguay	2000	Crush injury	0.1325
Paraguay	2005	Crush injury	0.1325
Paraguay	2010	Crush injury	0.1325
Paraguay	2017	Crush injury	0.1325
Paraguay	1990	Nerve injury	0.1129
Paraguay	1995	Nerve injury	0.1129
Paraguay	2000	Nerve injury	0.1129
Paraguay	2005	Nerve injury	0.1129
Paraguay	2010	Nerve injury	0.1129
Paraguay	2017	Nerve injury	0.1129
Paraguay	1990	Injury to eyes	0.0165
Paraguay	1995	Injury to eyes	0.0165
Paraguay	2000	Injury to eyes	0.0165
Paraguay	2005	Injury to eyes	0.0165
Paraguay	2010	Injury to eyes	0.0165
Paraguay	2017	Injury to eyes	0.0165
Paraguay	1990	Amputation of lower limb, unilateral	0.0943
Paraguay	1995	Amputation of lower limb, unilateral	0.0866
Paraguay	2000	Amputation of lower limb, unilateral	0.0826
Paraguay	2005	Amputation of lower limb, unilateral	0.0795
Paraguay	2010	Amputation of lower limb, unilateral	0.0762
Paraguay	2017	Amputation of lower limb, unilateral	0.0695
Paraguay	1990	Open wound(s)	0.011
Paraguay	1995	Open wound(s)	0.011
Paraguay	2000	Open wound(s)	0.011
Paraguay	2005	Open wound(s)	0.011
Paraguay	2010	Open wound(s)	0.011
Paraguay	2017	Open wound(s)	0.011
Paraguay	1990	Poisoning requiring urgent care	0.1628
Paraguay	1995	Poisoning requiring urgent care	0.1628
Paraguay	2000	Poisoning requiring urgent care	0.1628
Paraguay	2005	Poisoning requiring urgent care	0.1628
Paraguay	2010	Poisoning requiring urgent care	0.1628
Paraguay	2017	Poisoning requiring urgent care	0.1628
Paraguay	1990	Severe chest injury	0.0474
Paraguay	1995	Severe chest injury	0.0474
Paraguay	2000	Severe chest injury	0.0474
Paraguay	2005	Severe chest injury	0.0474
Paraguay	2010	Severe chest injury	0.0474
Paraguay	2017	Severe chest injury	0.0474
Paraguay	1990	Internal hemorrhage in abdomen and pelvis	0.1143
Paraguay	1995	Internal hemorrhage in abdomen and pelvis	0.1143
Paraguay	2000	Internal hemorrhage in abdomen and pelvis	0.1143
Paraguay	2005	Internal hemorrhage in abdomen and pelvis	0.1143
Paraguay	2010	Internal hemorrhage in abdomen and pelvis	0.1143
Paraguay	2017	Internal hemorrhage in abdomen and pelvis	0.1143
Paraguay	1990	Contusion in any part of the body	0.0075
Paraguay	1995	Contusion in any part of the body	0.0075
Paraguay	2000	Contusion in any part of the body	0.0075
Paraguay	2005	Contusion in any part of the body	0.0075
Paraguay	2010	Contusion in any part of the body	0.0075
Paraguay	2017	Contusion in any part of the body	0.0075
Paraguay	1990	Effect of different environmental factors	0.049
Paraguay	1995	Effect of different environmental factors	0.049
Paraguay	2000	Effect of different environmental factors	0.049
Paraguay	2005	Effect of different environmental factors	0.049
Paraguay	2010	Effect of different environmental factors	0.049
Paraguay	2017	Effect of different environmental factors	0.049
Paraguay	1990	Complications following therapeutic procedures	0.0506
Paraguay	1995	Complications following therapeutic procedures	0.0506
Paraguay	2000	Complications following therapeutic procedures	0.0506
Paraguay	2005	Complications following therapeutic procedures	0.0506
Paraguay	2010	Complications following therapeutic procedures	0.0506
Paraguay	2017	Complications following therapeutic procedures	0.0506
Paraguay	1990	Multiple fractures, dislocations, crashes, wounds, sprains, and strains	0.1997
Paraguay	1995	Multiple fractures, dislocations, crashes, wounds, sprains, and strains	0.18
Paraguay	2000	Multiple fractures, dislocations, crashes, wounds, sprains, and strains	0.1697
Paraguay	2005	Multiple fractures, dislocations, crashes, wounds, sprains, and strains	0.1616
Paraguay	2010	Multiple fractures, dislocations, crashes, wounds, sprains, and strains	0.1531
Paraguay	2017	Multiple fractures, dislocations, crashes, wounds, sprains, and strains	0.136
Paraguay	1990	Amputation of upper limb, unilateral	0.0716
Paraguay	1995	Amputation of upper limb, unilateral	0.067
Paraguay	2000	Amputation of upper limb, unilateral	0.0647
Paraguay	2005	Amputation of upper limb, unilateral	0.0628

Paraguay	2010	Amputation of upper limb, unilateral	0.0609
Paraguay	2017	Amputation of upper limb, unilateral	0.057
Paraguay	1990	Amputation of thumb	0.0114
Paraguay	1995	Amputation of thumb	0.0114
Paraguay	2000	Amputation of thumb	0.0114
Paraguay	2005	Amputation of thumb	0.0114
Paraguay	2010	Amputation of thumb	0.0114
Paraguay	2017	Amputation of thumb	0.0114
Paraguay	1990	Amputation of toe/toes	0.0059
Paraguay	1995	Amputation of toe/toes	0.0059
Paraguay	2000	Amputation of toe/toes	0.0059
Paraguay	2005	Amputation of toe/toes	0.0059
Paraguay	2010	Amputation of toe/toes	0.0059
Paraguay	2017	Amputation of toe/toes	0.0059
Paraguay	1990	Burns, <20% total burned surface area without lower airway burns	0.0163
Paraguay	1995	Burns, <20% total burned surface area without lower airway burns	0.0163
Paraguay	2000	Burns, <20% total burned surface area without lower airway burns	0.0163
Paraguay	2005	Burns, <20% total burned surface area without lower airway burns	0.0163
Paraguay	2010	Burns, <20% total burned surface area without lower airway burns	0.0163
Paraguay	2017	Burns, <20% total burned surface area without lower airway burns	0.0163
Paraguay	1990	Burns, >=20% total burned surface area or >= 10% burned surface area if head/neck or hands/wrist involved w/o lower airway burns	0.2668
Paraguay	1995	Burns, >=20% total burned surface area or >= 10% burned surface area if head/neck or hands/wrist involved w/o lower airway burns	0.2484
Paraguay	2000	Burns, >=20% total burned surface area or >= 10% burned surface area if head/neck or hands/wrist involved w/o lower airway burns	0.2389
Paraguay	2005	Burns, >=20% total burned surface area or >= 10% burned surface area if head/neck or hands/wrist involved w/o lower airway burns	0.2314
Paraguay	2010	Burns, >=20% total burned surface area or >= 10% burned surface area if head/neck or hands/wrist involved w/o lower airway burns	0.2235
Paraguay	2017	Burns, >=20% total burned surface area or >= 10% burned surface area if head/neck or hands/wrist involved w/o lower airway burns	0.2075
Algeria	1990	Amputation of lower limbs, bilateral	0.2316
Algeria	1995	Amputation of lower limbs, bilateral	0.218
Algeria	2000	Amputation of lower limbs, bilateral	0.2053
Algeria	2005	Amputation of lower limbs, bilateral	0.1898
Algeria	2010	Amputation of lower limbs, bilateral	0.1795
Algeria	2017	Amputation of lower limbs, bilateral	0.1614
Algeria	1990	Lower airway burns	0.2252
Algeria	1995	Lower airway burns	0.2252
Algeria	2000	Lower airway burns	0.2252
Algeria	2005	Lower airway burns	0.2252
Algeria	2010	Lower airway burns	0.2252
Algeria	2017	Lower airway burns	0.2252
Algeria	1990	Dislocation of hip	0.0159
Algeria	1995	Dislocation of hip	0.0159
Algeria	2000	Dislocation of hip	0.0159
Algeria	2005	Dislocation of hip	0.0159
Algeria	2010	Dislocation of hip	0.0159
Algeria	2017	Dislocation of hip	0.0159
Algeria	1990	Dislocation of knee	0.1134
Algeria	1995	Dislocation of knee	0.1134
Algeria	2000	Dislocation of knee	0.1134
Algeria	2005	Dislocation of knee	0.1134
Algeria	2010	Dislocation of knee	0.1134
Algeria	2017	Dislocation of knee	0.1134
Algeria	1990	Dislocation of shoulder	0.062
Algeria	1995	Dislocation of shoulder	0.062
Algeria	2000	Dislocation of shoulder	0.062
Algeria	2005	Dislocation of shoulder	0.062
Algeria	2010	Dislocation of shoulder	0.062
Algeria	2017	Dislocation of shoulder	0.062
Algeria	1990	Muscle and tendon injuries, including sprains and strains lesser dislocations	0.0075
Algeria	1995	Muscle and tendon injuries, including sprains and strains lesser dislocations	0.0075
Algeria	2000	Muscle and tendon injuries, including sprains and strains lesser dislocations	0.0075
Algeria	2005	Muscle and tendon injuries, including sprains and strains lesser dislocations	0.0075
Algeria	2010	Muscle and tendon injuries, including sprains and strains lesser dislocations	0.0075
Algeria	2017	Muscle and tendon injuries, including sprains and strains lesser dislocations	0.0075
Algeria	1990	Fracture of clavicle, scapula, or humerus	0.0349
Algeria	1995	Fracture of clavicle, scapula, or humerus	0.0349
Algeria	2000	Fracture of clavicle, scapula, or humerus	0.0349
Algeria	2005	Fracture of clavicle, scapula, or humerus	0.0349
Algeria	2010	Fracture of clavicle, scapula, or humerus	0.0349
Algeria	2017	Fracture of clavicle, scapula, or humerus	0.0349
Algeria	1990	Fracture of face bones	0.0669
Algeria	1995	Fracture of face bones	0.0669
Algeria	2000	Fracture of face bones	0.0669
Algeria	2005	Fracture of face bones	0.0669
Algeria	2010	Fracture of face bones	0.0669
Algeria	2017	Fracture of face bones	0.0669
Algeria	1990	Fracture of foot bones except ankle	0.0264
Algeria	1995	Fracture of foot bones except ankle	0.0264
Algeria	2000	Fracture of foot bones except ankle	0.0264
Algeria	2005	Fracture of foot bones except ankle	0.0264
Algeria	2010	Fracture of foot bones except ankle	0.0264
Algeria	2017	Fracture of foot bones except ankle	0.0264
Algeria	1990	Fracture of hand (wrist and other distal part of hand)	0.0138
Algeria	1995	Fracture of hand (wrist and other distal part of hand)	0.0138
Algeria	2000	Fracture of hand (wrist and other distal part of hand)	0.0138
Algeria	2005	Fracture of hand (wrist and other distal part of hand)	0.0138
Algeria	2010	Fracture of hand (wrist and other distal part of hand)	0.0138
Algeria	2017	Fracture of hand (wrist and other distal part of hand)	0.0138
Algeria	1990	Fracture of hip	0.2011
Algeria	1995	Fracture of hip	0.183
Algeria	2000	Fracture of hip	0.1661
Algeria	2005	Fracture of hip	0.1457
Algeria	2010	Fracture of hip	0.1321
Algeria	2017	Fracture of hip	0.1082
Algeria	1990	Amputation of upper limbs, bilateral	0.2357
Algeria	1995	Amputation of upper limbs, bilateral	0.2171
Algeria	2000	Amputation of upper limbs, bilateral	0.1996
Algeria	2005	Amputation of upper limbs, bilateral	0.1785
Algeria	2010	Amputation of upper limbs, bilateral	0.1645
Algeria	2017	Amputation of upper limbs, bilateral	0.1398
Algeria	1990	Fracture of patella, tibia or fibula, or ankle	0.0554
Algeria	1995	Fracture of patella, tibia or fibula, or ankle	0.0554
Algeria	2000	Fracture of patella, tibia or fibula, or ankle	0.0554
Algeria	2005	Fracture of patella, tibia or fibula, or ankle	0.0554
Algeria	2010	Fracture of patella, tibia or fibula, or ankle	0.0554
Algeria	2017	Fracture of patella, tibia or fibula, or ankle	0.0554
Algeria	1990	Fracture of pelvis	0.1823
Algeria	1995	Fracture of pelvis	0.1823
Algeria	2000	Fracture of pelvis	0.1823
Algeria	2005	Fracture of pelvis	0.1823
Algeria	2010	Fracture of pelvis	0.1823
Algeria	2017	Fracture of pelvis	0.1823
Algeria	1990	Fracture of radius and/or ulna	0.0435
Algeria	1995	Fracture of radius and/or ulna	0.0435
Algeria	2000	Fracture of radius and/or ulna	0.0435
Algeria	2005	Fracture of radius and/or ulna	0.0435
Algeria	2010	Fracture of radius and/or ulna	0.0435
Algeria	2017	Fracture of radius and/or ulna	0.0435
Algeria	1990	Fracture of skull	0.0714
Algeria	1995	Fracture of skull	0.0714
Algeria	2000	Fracture of skull	0.0714
Algeria	2005	Fracture of skull	0.0714
Algeria	2010	Fracture of skull	0.0714
Algeria	2017	Fracture of skull	0.0714
Algeria	1990	Fracture of sternum and/or fracture of one or more ribs	0.1027
Algeria	1995	Fracture of sternum and/or fracture of one or more ribs	0.1027
Algeria	2000	Fracture of sternum and/or fracture of one or more ribs	0.1027
Algeria	2005	Fracture of sternum and/or fracture of one or more ribs	0.1027
Algeria	2010	Fracture of sternum and/or fracture of one or more ribs	0.1027
Algeria	2017	Fracture of sternum and/or fracture of one or more ribs	0.1027
Algeria	1990	Fracture of vertebral column	0.1106
Algeria	1995	Fracture of vertebral column	0.1106
Algeria	2000	Fracture of vertebral column	0.1106
Algeria	2005	Fracture of vertebral column	0.1106
Algeria	2010	Fracture of vertebral column	0.1106
Algeria	2017	Fracture of vertebral column	0.1106
Algeria	1990	Fracture of femur, other than femoral neck	0.042
Algeria	1995	Fracture of femur, other than femoral neck	0.042
Algeria	2000	Fracture of femur, other than femoral neck	0.042
Algeria	2005	Fracture of femur, other than femoral neck	0.042
Algeria	2010	Fracture of femur, other than femoral neck	0.042
Algeria	2017	Fracture of femur, other than femoral neck	0.042
Algeria	1990	Minor TBI	0.1319
Algeria	1995	Minor TBI	0.1319
Algeria	2000	Minor TBI	0.1319
Algeria	2005	Minor TBI	0.1319
Algeria	2010	Minor TBI	0.1319
Algeria	2017	Minor TBI	0.1319
Algeria	1990	Moderate TBI	0.1637
Algeria	1995	Moderate TBI	0.1637
Algeria	2000	Moderate TBI	0.1637
Algeria	2005	Moderate TBI	0.1637
Algeria	2010	Moderate TBI	0.1637
Algeria	2017	Moderate TBI	0.1637
Algeria	1990	Amputation of fingers (excluding thumb)	0.0047
Algeria	1995	Amputation of fingers (excluding thumb)	0.0047
Algeria	2000	Amputation of fingers (excluding thumb)	0.0047
Algeria	2005	Amputation of fingers (excluding thumb)	0.0047
Algeria	2010	Amputation of fingers (excluding thumb)	0.0047
Algeria	2017	Amputation of fingers (excluding thumb)	0.0047
Algeria	1990	Spinal cord lesion at neck level	0.4614
Algeria	1995	Spinal cord lesion at neck level	0.4552
Algeria	2000	Spinal cord lesion at neck level	0.4493
Algeria	2005	Spinal cord lesion at neck level	0.4423
Algeria	2010	Spinal cord lesion at neck level	0.4376
Algeria	2017	Spinal cord lesion at neck level	0.4293
Algeria	1990	Spinal cord lesion below neck level	0.2823
Algeria	1995	Spinal cord lesion below neck level	0.2692
Algeria	2000	Spinal cord lesion below neck level	0.2568
Algeria	2005	Spinal cord lesion below neck level	0.2419
Algeria	2010	Spinal cord lesion below neck level	0.232
Algeria	2017	Spinal cord lesion below neck level	0.2145
Algeria	1990	Drowning and nonfatal submersion	0.2471
Algeria	1995	Drowning and nonfatal submersion	0.2471
Algeria	2000	Drowning and nonfatal submersion	0.2471
Algeria	2005	Drowning and nonfatal submersion	0.2471
Algeria	2010	Drowning and nonfatal submersion	0.2471
Algeria	2017	Drowning and nonfatal submersion	0.2471
Algeria	1990	Asphyxiation	0.2471
Algeria	1995	Asphyxiation	0.2471
Algeria	2000	Asphyxiation	0.2471
Algeria	2005	Asphyxiation	0.2471
Algeria	2010	Asphyxiation	0.2471
Algeria	2017	Asphyxiation	0.2471
Algeria	1990	Crush injury	0.1325
Algeria	1995	Crush injury	0.1325
Algeria	2000	Crush injury	0.1325
Algeria	2005	Crush injury	0.1325
Algeria	2010	Crush injury	0.1325
Algeria	2017	Crush injury	0.1325
Algeria	1990	Nerve injury	0.1129
Algeria	1995	Nerve injury	0.1129
Algeria	2000	Nerve injury	0.1129
Algeria	2005	Nerve injury	0.1129
Algeria	2010	Nerve injury	0.1129
Algeria	2017	Nerve injury	0.1129
Algeria	1990	Injury to eyes	0.0165
Algeria	1995	Injury to eyes	0.0165
Algeria	2000	Injury to eyes	0.0165
Algeria	2005	Injury to eyes	0.0165
Algeria	2010	Injury to eyes	0.0165
Algeria	2017	Injury to eyes	0.0165
Algeria	1990	Amputation of lower limb, unilateral	0.0948
Algeria	1995	Amputation of lower limb, unilateral	0.0878
Algeria	2000	Amputation of lower limb, unilateral	0.0812
Algeria	2005	Amputation of lower limb, unilateral	0.0733
Algeria	2010	Amputation of lower limb, unilateral	0.068
Algeria	2017	Amputation of lower limb, unilateral	0.0586
Algeria	1990	Open wound(s)	0.011
Algeria	1995	Open wound(s)	0.011
Algeria	2000	Open wound(s)	0.011
Algeria	2005	Open wound(s)	0.011
Algeria	2010	Open wound(s)	0.011
Algeria	2017	Open wound(s)	0.011
Algeria	1990	Poisoning requiring urgent care	0.1628
Algeria	1995	Poisoning requiring urgent care	0.1628
Algeria	2000	Poisoning requiring urgent care	0.1628
Algeria	2005	Poisoning requiring urgent care	0.1628
Algeria	2010	Poisoning requiring urgent care	0.1628
Algeria	2017	Poisoning requiring urgent care	0.1628
Algeria	1990	Severe chest injury	0.0474
Algeria	1995	Severe chest injury	0.0474
Algeria	2000	Severe chest injury	0.0474
Algeria	2005	Severe chest injury	0.0474
Algeria	2010	Severe chest injury	0.0474
Algeria	2017	Severe chest injury	0.0474
Algeria	1990	Internal hemorrhage in abdomen and pelvis	0.1143
Algeria	1995	Internal hemorrhage in abdomen and pelvis	0.1143
Algeria	2000	Internal hemorrhage in abdomen and pelvis	0.1143
Algeria	2005	Internal hemorrhage in abdomen and pelvis	0.1143
Algeria	2010	Internal hemorrhage in abdomen and pelvis	0.1143
Algeria	2017	Internal hemorrhage in abdomen and pelvis	0.1143
Algeria	1990	Contusion in any part of the body	0.0075
Algeria	1995	Contusion in any part of the body	0.0075
Algeria	2000	Contusion in any part of the body	0.0075
Algeria	2005	Contusion in any part of the body	0.0075
Algeria	2010	Contusion in any part of the body	0.0075
Algeria	2017	Contusion in any part of the body	0.0075
Algeria	1990	Effect of different environmental factors	0.049
Algeria	1995	Effect of different environmental factors	0.049
Algeria	2000	Effect of different environmental factors	0.049
Algeria	2005	Effect of different environmental factors	0.049
Algeria	2010	Effect of different environmental factors	0.049
Algeria	2017	Effect of different environmental factors	0.049
Algeria	1990	Complications following therapeutic procedures	0.0506
Algeria	1995	Complications following therapeutic procedures	0.0506
Algeria	2000	Complications following therapeutic procedures	0.0506
Algeria	2005	Complications following therapeutic procedures	0.0506
Algeria	2010	Complications following therapeutic procedures	0.0506
Algeria	2017	Complications following therapeutic procedures	0.0506
Algeria	1990	Multiple fractures, dislocations, crashes, wounds, sprains, and strains	0.2011
Algeria	1995	Multiple fractures, dislocations, crashes, wounds, sprains, and strains	0.183
Algeria	2000	Multiple fractures, dislocations, crashes, wounds, sprains, and strains	0.1661
Algeria	2005	Multiple fractures, dislocations, crashes, wounds, sprains, and strains	0.1457
Algeria	2010	Multiple fractures, dislocations, crashes, wounds, sprains, and strains	0.1321
Algeria	2017	Multiple fractures, dislocations, crashes, wounds, sprains, and strains	0.1082
Algeria	1990	Amputation of upper limb, unilateral	0.0719
Algeria	1995	Amputation of upper limb, unilateral	0.0677
Algeria	2000	Amputation of upper limb, unilateral	0.0639
Algeria	2005	Amputation of upper limb, unilateral	0.0592
Algeria	2010	Amputation of upper limb, unilateral	0.0561
Algeria	2017	Amputation of upper limb, unilateral	0.0506
Algeria	1990	Amputation of thumb	0.0114
Algeria	1995	Amputation of thumb	0.0114
Algeria	2000	Amputation of thumb	0.0114
Algeria	2005	Amputation of thumb	0.0114
Algeria	2010	Amputation of thumb	0.0114
Algeria	2017	Amputation of thumb	0.0114
Algeria	1990	Amputation of toe/toes	0.0059
Algeria	1995	Amputation of toe/toes	0.0059
Algeria	2000	Amputation of toe/toes	0.0059
Algeria	2005	Amputation of toe/toes	0.0059
Algeria	2010	Amputation of toe/toes	0.0059
Algeria	2017	Amputation of toe/toes	0.0059
Algeria	1990	Burns, <20% total burned surface area without lower airway burns	0.0163
Algeria	1995	Burns, <20% total burned surface area without lower airway burns	0.0163
Algeria	2000	Burns, <20% total burned surface area without lower airway burns	0.0163
Algeria	2005	Burns, <20% total burned surface area without lower airway burns	0.0163
Algeria	2010	Burns, <20% total burned surface area without lower airway burns	0.0163
Algeria	2017	Burns, <20% total burned surface area without lower airway burns	0.0163
Algeria	1990	Burns, >=20% total burned surface area or >= 10% burned surface area if head/neck or hands/wrist involved w/o lower airway burns	0.2681
Algeria	1995	Burns, >=20% total burned surface area or >= 10% burned surface area if head/neck or hands/wrist involved w/o lower airway burns	0.2513
Algeria	2000	Burns, >=20% total burned surface area or >= 10% burned surface area if head/neck or hands/wrist involved w/o lower airway burns	0.2356
Algeria	2005	Burns, >=20% total burned surface area or >= 10% burned surface area if head/neck or hands/wrist involved w/o lower airway burns	0.2166
Algeria	2010	Burns, >=20% total burned surface area or >= 10% burned surface area if head/neck or hands/wrist involved w/o lower airway burns	0.2039
Algeria	2017	Burns, >=20% total burned surface area or >= 10% burned surface area if head/neck or hands/wrist involved w/o lower airway burns	0.1816
Bahrain	1990	Amputation of lower limbs, bilateral	0.2081
Bahrain	1995	Amputation of lower limbs, bilateral	0.1944
Bahrain	2000	Amputation of lower limbs, bilateral	0.1767
Bahrain	2005	Amputation of lower limbs, bilateral	0.1633
Bahrain	2010	Amputation of lower limbs, bilateral	0.144
Bahrain	2017	Amputation of lower limbs, bilateral	0.1322
Bahrain	1990	Lower airway burns	0.2252
Bahrain	1995	Lower airway burns	0.2252
Bahrain	2000	Lower airway burns	0.2252
Bahrain	2005	Lower airway burns	0.2252
Bahrain	2010	Lower airway burns	0.2252
Bahrain	2017	Lower airway burns	0.2252
Bahrain	1990	Dislocation of hip	0.0159
Bahrain	1995	Dislocation of hip	0.0159
Bahrain	2000	Dislocation of hip	0.0159
Bahrain	2005	Dislocation of hip	0.0159
Bahrain	2010	Dislocation of hip	0.0159
Bahrain	2017	Dislocation of hip	0.0159
Bahrain	1990	Dislocation of knee	0.1134
Bahrain	1995	Dislocation of knee	0.1134
Bahrain	2000	Dislocation of knee	0.1134
Bahrain	2005	Dislocation of knee	0.1134
Bahrain	2010	Dislocation of knee	0.1134
Bahrain	2017	Dislocation of knee	0.1134
Bahrain	1990	Dislocation of shoulder	0.062
Bahrain	1995	Dislocation of shoulder	0.062
Bahrain	2000	Dislocation of shoulder	0.062
Bahrain	2005	Dislocation of shoulder	0.062
Bahrain	2010	Dislocation of shoulder	0.062
Bahrain	2017	Dislocation of shoulder	0.062
Bahrain	1990	Muscle and tendon injuries, including sprains and strains lesser dislocations	0.0075
Bahrain	1995	Muscle and tendon injuries, including sprains and strains lesser dislocations	0.0075
Bahrain	2000	Muscle and tendon injuries, including sprains and strains lesser dislocations	0.0075
Bahrain	2005	Muscle and tendon injuries, including sprains and strains lesser dislocations	0.0075
Bahrain	2010	Muscle and tendon injuries, including sprains and strains lesser dislocations	0.0075
Bahrain	2017	Muscle and tendon injuries, including sprains and strains lesser dislocations	0.0075
Bahrain	1990	Fracture of clavicle, scapula, or humerus	0.0349
Bahrain	1995	Fracture of clavicle, scapula, or humerus	0.0349
Bahrain	2000	Fracture of clavicle, scapula, or humerus	0.0349
Bahrain	2005	Fracture of clavicle, scapula, or humerus	0.0349
Bahrain	2010	Fracture of clavicle, scapula, or humerus	0.0349
Bahrain	2017	Fracture of clavicle, scapula, or humerus	0.0349
Bahrain	1990	Fracture of face bones	0.0669
Bahrain	1995	Fracture of face bones	0.0669
Bahrain	2000	Fracture of face bones	0.0669
Bahrain	2005	Fracture of face bones	0.0669
Bahrain	2010	Fracture of face bones	0.0669
Bahrain	2017	Fracture of face bones	0.0669
Bahrain	1990	Fracture of foot bones except ankle	0.0264
Bahrain	1995	Fracture of foot bones except ankle	0.0264
Bahrain	2000	Fracture of foot bones except ankle	0.0264
Bahrain	2005	Fracture of foot bones except ankle	0.0264
Bahrain	2010	Fracture of foot bones except ankle	0.0264
Bahrain	2017	Fracture of foot bones except ankle	0.0264
Bahrain	1990	Fracture of hand (wrist and other distal part of hand)	0.0138
Bahrain	1995	Fracture of hand (wrist and other distal part of hand)	0.0138
Bahrain	2000	Fracture of hand (wrist and other distal part of hand)	0.0138
Bahrain	2005	Fracture of hand (wrist and other distal part of hand)	0.0138
Bahrain	2010	Fracture of hand (wrist and other distal part of hand)	0.0138
Bahrain	2017	Fracture of hand (wrist and other distal part of hand)	0.0138
Bahrain	1990	Fracture of hip	0.1699
Bahrain	1995	Fracture of hip	0.1517
Bahrain	2000	Fracture of hip	0.1283
Bahrain	2005	Fracture of hip	0.1106
Bahrain	2010	Fracture of hip	0.0851
Bahrain	2017	Fracture of hip	0.0695
Bahrain	1990	Amputation of upper limbs, bilateral	0.2035
Bahrain	1995	Amputation of upper limbs, bilateral	0.1848
Bahrain	2000	Amputation of upper limbs, bilateral	0.1606
Bahrain	2005	Amputation of upper limbs, bilateral	0.1423
Bahrain	2010	Amputation of upper limbs, bilateral	0.116
Bahrain	2017	Amputation of upper limbs, bilateral	0.0999
Bahrain	1990	Fracture of patella, tibia or fibula, or ankle	0.0554
Bahrain	1995	Fracture of patella, tibia or fibula, or ankle	0.0554
Bahrain	2000	Fracture of patella, tibia or fibula, or ankle	0.0554
Bahrain	2005	Fracture of patella, tibia or fibula, or ankle	0.0554
Bahrain	2010	Fracture of patella, tibia or fibula, or ankle	0.0554
Bahrain	2017	Fracture of patella, tibia or fibula, or ankle	0.0554
Bahrain	1990	Fracture of pelvis	0.1823
Bahrain	1995	Fracture of pelvis	0.1823
Bahrain	2000	Fracture of pelvis	0.1823
Bahrain	2005	Fracture of pelvis	0.1823
Bahrain	2010	Fracture of pelvis	0.1823
Bahrain	2017	Fracture of pelvis	0.1823
Bahrain	1990	Fracture of radius and/or ulna	0.0435
Bahrain	1995	Fracture of radius and/or ulna	0.0435
Bahrain	2000	Fracture of radius and/or ulna	0.0435
Bahrain	2005	Fracture of radius and/or ulna	0.0435
Bahrain	2010	Fracture of radius and/or ulna	0.0435
Bahrain	2017	Fracture of radius and/or ulna	0.0435
Bahrain	1990	Fracture of skull	0.0714
Bahrain	1995	Fracture of skull	0.0714
Bahrain	2000	Fracture of skull	0.0714
Bahrain	2005	Fracture of skull	0.0714
Bahrain	2010	Fracture of skull	0.0714
Bahrain	2017	Fracture of skull	0.0714
Bahrain	1990	Fracture of sternum and/or fracture of one or more ribs	0.1027
Bahrain	1995	Fracture of sternum and/or fracture of one or more ribs	0.1027
Bahrain	2000	Fracture of sternum and/or fracture of one or more ribs	0.1027
Bahrain	2005	Fracture of sternum and/or fracture of one or more ribs	0.1027
Bahrain	2010	Fracture of sternum and/or fracture of one or more ribs	0.1027
Bahrain	2017	Fracture of sternum and/or fracture of one or more ribs	0.1027
Bahrain	1990	Fracture of vertebral column	0.1106
Bahrain	1995	Fracture of vertebral column	0.1106
Bahrain	2000	Fracture of vertebral column	0.1106
Bahrain	2005	Fracture of vertebral column	0.1106
Bahrain	2010	Fracture of vertebral column	0.1106
Bahrain	2017	Fracture of vertebral column	0.1106
Bahrain	1990	Fracture of femur, other than femoral neck	0.042
Bahrain	1995	Fracture of femur, other than femoral neck	0.042
Bahrain	2000	Fracture of femur, other than femoral neck	0.042
Bahrain	2005	Fracture of femur, other than femoral neck	0.042
Bahrain	2010	Fracture of femur, other than femoral neck	0.042
Bahrain	2017	Fracture of femur, other than femoral neck	0.042
Bahrain	1990	Minor TBI	0.1319
Bahrain	1995	Minor TBI	0.1319
Bahrain	2000	Minor TBI	0.1319
Bahrain	2005	Minor TBI	0.1319
Bahrain	2010	Minor TBI	0.1319
Bahrain	2017	Minor TBI	0.1319
Bahrain	1990	Moderate TBI	0.1637
Bahrain	1995	Moderate TBI	0.1637
Bahrain	2000	Moderate TBI	0.1637
Bahrain	2005	Moderate TBI	0.1637
Bahrain	2010	Moderate TBI	0.1637
Bahrain	2017	Moderate TBI	0.1637
Bahrain	1990	Amputation of fingers (excluding thumb)	0.0047
Bahrain	1995	Amputation of fingers (excluding thumb)	0.0047
Bahrain	2000	Amputation of fingers (excluding thumb)	0.0047
Bahrain	2005	Amputation of fingers (excluding thumb)	0.0047
Bahrain	2010	Amputation of fingers (excluding thumb)	0.0047
Bahrain	2017	Amputation of fingers (excluding thumb)	0.0047
Bahrain	1990	Spinal cord lesion at neck level	0.4506
Bahrain	1995	Spinal cord lesion at neck level	0.4443
Bahrain	2000	Spinal cord lesion at neck level	0.4362
Bahrain	2005	Spinal cord lesion at neck level	0.4301
Bahrain	2010	Spinal cord lesion at neck level	0.4213
Bahrain	2017	Spinal cord lesion at neck level	0.4159
Bahrain	1990	Spinal cord lesion below neck level	0.2596
Bahrain	1995	Spinal cord lesion below neck level	0.2463
Bahrain	2000	Spinal cord lesion below neck level	0.2292
Bahrain	2005	Spinal cord lesion below neck level	0.2163
Bahrain	2010	Spinal cord lesion below neck level	0.1977
Bahrain	2017	Spinal cord lesion below neck level	0.1863
Bahrain	1990	Drowning and nonfatal submersion	0.2471
Bahrain	1995	Drowning and nonfatal submersion	0.2471
Bahrain	2000	Drowning and nonfatal submersion	0.2471

Bahrain	2005	Drowning and nonfatal submersion	0.2471
Bahrain	2010	Drowning and nonfatal submersion	0.2471
Bahrain	2017	Drowning and nonfatal submersion	0.2471
Bahrain	1990	Asphyxiation	0.2471
Bahrain	1995	Asphyxiation	0.2471
Bahrain	2000	Asphyxiation	0.2471
Bahrain	2005	Asphyxiation	0.2471
Bahrain	2010	Asphyxiation	0.2471
Bahrain	2017	Asphyxiation	0.2471
Bahrain	1990	Crush injury	0.1325
Bahrain	1995	Crush injury	0.1325
Bahrain	2000	Crush injury	0.1325
Bahrain	2005	Crush injury	0.1325
Bahrain	2010	Crush injury	0.1325
Bahrain	2017	Crush injury	0.1325
Bahrain	1990	Nerve injury	0.1129
Bahrain	1995	Nerve injury	0.1129
Bahrain	2000	Nerve injury	0.1129
Bahrain	2005	Nerve injury	0.1129
Bahrain	2010	Nerve injury	0.1129
Bahrain	2017	Nerve injury	0.1129
Bahrain	1990	Injury to eyes	0.0165
Bahrain	1995	Injury to eyes	0.0165
Bahrain	2000	Injury to eyes	0.0165
Bahrain	2005	Injury to eyes	0.0165
Bahrain	2010	Injury to eyes	0.0165
Bahrain	2017	Injury to eyes	0.0165
Bahrain	1990	Amputation of lower limb, unilateral	0.0827
Bahrain	1995	Amputation of lower limb, unilateral	0.0756
Bahrain	2000	Amputation of lower limb, unilateral	0.0665
Bahrain	2005	Amputation of lower limb, unilateral	0.0596
Bahrain	2010	Amputation of lower limb, unilateral	0.0496
Bahrain	2017	Amputation of lower limb, unilateral	0.0436
Bahrain	1990	Open wound(s)	0.011
Bahrain	1995	Open wound(s)	0.011
Bahrain	2000	Open wound(s)	0.011
Bahrain	2005	Open wound(s)	0.011
Bahrain	2010	Open wound(s)	0.011
Bahrain	2017	Open wound(s)	0.011
Bahrain	1990	Poisoning requiring urgent care	0.1628
Bahrain	1995	Poisoning requiring urgent care	0.1628
Bahrain	2000	Poisoning requiring urgent care	0.1628
Bahrain	2005	Poisoning requiring urgent care	0.1628
Bahrain	2010	Poisoning requiring urgent care	0.1628
Bahrain	2017	Poisoning requiring urgent care	0.1628
Bahrain	1990	Severe chest injury	0.0474
Bahrain	1995	Severe chest injury	0.0474
Bahrain	2000	Severe chest injury	0.0474
Bahrain	2005	Severe chest injury	0.0474
Bahrain	2010	Severe chest injury	0.0474
Bahrain	2017	Severe chest injury	0.0474
Bahrain	1990	Internal hemorrhage in abdomen and pelvis	0.1143
Bahrain	1995	Internal hemorrhage in abdomen and pelvis	0.1143
Bahrain	2000	Internal hemorrhage in abdomen and pelvis	0.1143
Bahrain	2005	Internal hemorrhage in abdomen and pelvis	0.1143
Bahrain	2010	Internal hemorrhage in abdomen and pelvis	0.1143
Bahrain	2017	Internal hemorrhage in abdomen and pelvis	0.1143
Bahrain	1990	Contusion in any part of the body	0.0075
Bahrain	1995	Contusion in any part of the body	0.0075
Bahrain	2000	Contusion in any part of the body	0.0075
Bahrain	2005	Contusion in any part of the body	0.0075
Bahrain	2010	Contusion in any part of the body	0.0075
Bahrain	2017	Contusion in any part of the body	0.0075
Bahrain	1990	Effect of different environmental factors	0.049
Bahrain	1995	Effect of different environmental factors	0.049
Bahrain	2000	Effect of different environmental factors	0.049
Bahrain	2005	Effect of different environmental factors	0.049
Bahrain	2010	Effect of different environmental factors	0.049
Bahrain	2017	Effect of different environmental factors	0.049
Bahrain	1990	Complications following therapeutic procedures	0.0506
Bahrain	1995	Complications following therapeutic procedures	0.0506
Bahrain	2000	Complications following therapeutic procedures	0.0506
Bahrain	2005	Complications following therapeutic procedures	0.0506
Bahrain	2010	Complications following therapeutic procedures	0.0506
Bahrain	2017	Complications following therapeutic procedures	0.0506
Bahrain	1990	Multiple fractures, dislocations, crashes, wounds, sprains, and strains	0.1699
Bahrain	1995	Multiple fractures, dislocations, crashes, wounds, sprains, and strains	0.1517
Bahrain	2000	Multiple fractures, dislocations, crashes, wounds, sprains, and strains	0.1283
Bahrain	2005	Multiple fractures, dislocations, crashes, wounds, sprains, and strains	0.1106
Bahrain	2010	Multiple fractures, dislocations, crashes, wounds, sprains, and strains	0.0851
Bahrain	2017	Multiple fractures, dislocations, crashes, wounds, sprains, and strains	0.0695
Bahrain	1990	Amputation of upper limb, unilateral	0.0647
Bahrain	1995	Amputation of upper limb, unilateral	0.0606
Bahrain	2000	Amputation of upper limb, unilateral	0.0552
Bahrain	2005	Amputation of upper limb, unilateral	0.0511
Bahrain	2010	Amputation of upper limb, unilateral	0.0453
Bahrain	2017	Amputation of upper limb, unilateral	0.0417
Bahrain	1990	Amputation of thumb	0.0114
Bahrain	1995	Amputation of thumb	0.0114
Bahrain	2000	Amputation of thumb	0.0114
Bahrain	2005	Amputation of thumb	0.0114
Bahrain	2010	Amputation of thumb	0.0114
Bahrain	2017	Amputation of thumb	0.0114
Bahrain	1990	Amputation of toe/toes	0.0059
Bahrain	1995	Amputation of toe/toes	0.0059
Bahrain	2000	Amputation of toe/toes	0.0059
Bahrain	2005	Amputation of toe/toes	0.0059
Bahrain	2010	Amputation of toe/toes	0.0059
Bahrain	2017	Amputation of toe/toes	0.0059
Bahrain	1990	Burns, <20% total burned surface area without lower airway burns	0.0163
Bahrain	1995	Burns, <20% total burned surface area without lower airway burns	0.0163
Bahrain	2000	Burns, <20% total burned surface area without lower airway burns	0.0163
Bahrain	2005	Burns, <20% total burned surface area without lower airway burns	0.0163
Bahrain	2010	Burns, <20% total burned surface area without lower airway burns	0.0163
Bahrain	2017	Burns, <20% total burned surface area without lower airway burns	0.0163
Bahrain	1990	Burns, >=20% total burned surface area or >= 10% burned surface area if head/neck or hands/wrist involved w/o lower airway burns	0.2391
Bahrain	1995	Burns, >=20% total burned surface area or >= 10% burned surface area if head/neck or hands/wrist involved w/o lower airway burns	0.2222
Bahrain	2000	Burns, >=20% total burned surface area or >= 10% burned surface area if head/neck or hands/wrist involved w/o lower airway burns	0.2003
Bahrain	2005	Burns, >=20% total burned surface area or >= 10% burned surface area if head/neck or hands/wrist involved w/o lower airway burns	0.1839
Bahrain	2010	Burns, >=20% total burned surface area or >= 10% burned surface area if head/neck or hands/wrist involved w/o lower airway burns	0.1601
Bahrain	2017	Burns, >=20% total burned surface area or >= 10% burned surface area if head/neck or hands/wrist involved w/o lower airway burns	0.1456
Egypt	1990	Amputation of lower limbs, bilateral	0.2608
Egypt	1995	Amputation of lower limbs, bilateral	0.2424
Egypt	2000	Amputation of lower limbs, bilateral	0.2218
Egypt	2005	Amputation of lower limbs, bilateral	0.2075
Egypt	2010	Amputation of lower limbs, bilateral	0.1963
Egypt	2017	Amputation of lower limbs, bilateral	0.1777
Egypt	1990	Lower airway burns	0.2252
Egypt	1995	Lower airway burns	0.2252
Egypt	2000	Lower airway burns	0.2252
Egypt	2005	Lower airway burns	0.2252
Egypt	2010	Lower airway burns	0.2252
Egypt	2017	Lower airway burns	0.2252
Egypt	1990	Dislocation of hip	0.0159
Egypt	1995	Dislocation of hip	0.0159
Egypt	2000	Dislocation of hip	0.0159
Egypt	2005	Dislocation of hip	0.0159
Egypt	2010	Dislocation of hip	0.0159
Egypt	2017	Dislocation of hip	0.0159
Egypt	1990	Dislocation of knee	0.1134
Egypt	1995	Dislocation of knee	0.1134
Egypt	2000	Dislocation of knee	0.1134
Egypt	2005	Dislocation of knee	0.1134
Egypt	2010	Dislocation of knee	0.1134
Egypt	2017	Dislocation of knee	0.1134
Egypt	1990	Dislocation of shoulder	0.062
Egypt	1995	Dislocation of shoulder	0.062
Egypt	2000	Dislocation of shoulder	0.062
Egypt	2005	Dislocation of shoulder	0.062
Egypt	2010	Dislocation of shoulder	0.062
Egypt	2017	Dislocation of shoulder	0.062
Egypt	1990	Muscle and tendon injuries, including sprains and strains lesser dislocations	0.0075
Egypt	1995	Muscle and tendon injuries, including sprains and strains lesser dislocations	0.0075
Egypt	2000	Muscle and tendon injuries, including sprains and strains lesser dislocations	0.0075
Egypt	2005	Muscle and tendon injuries, including sprains and strains lesser dislocations	0.0075
Egypt	2010	Muscle and tendon injuries, including sprains and strains lesser dislocations	0.0075
Egypt	2017	Muscle and tendon injuries, including sprains and strains lesser dislocations	0.0075
Egypt	1990	Fracture of clavicle, scapula, or humerus	0.0349
Egypt	1995	Fracture of clavicle, scapula, or humerus	0.0349
Egypt	2000	Fracture of clavicle, scapula, or humerus	0.0349
Egypt	2005	Fracture of clavicle, scapula, or humerus	0.0349
Egypt	2010	Fracture of clavicle, scapula, or humerus	0.0349
Egypt	2017	Fracture of clavicle, scapula, or humerus	0.0349
Egypt	1990	Fracture of face bones	0.0669
Egypt	1995	Fracture of face bones	0.0669
Egypt	2000	Fracture of face bones	0.0669
Egypt	2005	Fracture of face bones	0.0669
Egypt	2010	Fracture of face bones	0.0669
Egypt	2017	Fracture of face bones	0.0669
Egypt	1990	Fracture of foot bones except ankle	0.0264
Egypt	1995	Fracture of foot bones except ankle	0.0264
Egypt	2000	Fracture of foot bones except ankle	0.0264
Egypt	2005	Fracture of foot bones except ankle	0.0264
Egypt	2010	Fracture of foot bones except ankle	0.0264
Egypt	2017	Fracture of foot bones except ankle	0.0264
Egypt	1990	Fracture of hand (wrist and other distal part of hand)	0.0138
Egypt	1995	Fracture of hand (wrist and other distal part of hand)	0.0138
Egypt	2000	Fracture of hand (wrist and other distal part of hand)	0.0138
Egypt	2005	Fracture of hand (wrist and other distal part of hand)	0.0138
Egypt	2010	Fracture of hand (wrist and other distal part of hand)	0.0138
Egypt	2017	Fracture of hand (wrist and other distal part of hand)	0.0138
Egypt	1990	Fracture of hip	0.2397
Egypt	1995	Fracture of hip	0.2153
Egypt	2000	Fracture of hip	0.1881
Egypt	2005	Fracture of hip	0.1691
Egypt	2010	Fracture of hip	0.1543
Egypt	2017	Fracture of hip	0.1297
Egypt	1990	Amputation of upper limbs, bilateral	0.2755
Egypt	1995	Amputation of upper limbs, bilateral	0.2504
Egypt	2000	Amputation of upper limbs, bilateral	0.2223
Egypt	2005	Amputation of upper limbs, bilateral	0.2027
Egypt	2010	Amputation of upper limbs, bilateral	0.1874
Egypt	2017	Amputation of upper limbs, bilateral	0.162
Egypt	1990	Fracture of patella, tibia or fibula, or ankle	0.0554
Egypt	1995	Fracture of patella, tibia or fibula, or ankle	0.0554
Egypt	2000	Fracture of patella, tibia or fibula, or ankle	0.0554
Egypt	2005	Fracture of patella, tibia or fibula, or ankle	0.0554
Egypt	2010	Fracture of patella, tibia or fibula, or ankle	0.0554
Egypt	2017	Fracture of patella, tibia or fibula, or ankle	0.0554
Egypt	1990	Fracture of pelvis	0.1823
Egypt	1995	Fracture of pelvis	0.1823
Egypt	2000	Fracture of pelvis	0.1823
Egypt	2005	Fracture of pelvis	0.1823
Egypt	2010	Fracture of pelvis	0.1823
Egypt	2017	Fracture of pelvis	0.1823
Egypt	1990	Fracture of radius and/or ulna	0.0435
Egypt	1995	Fracture of radius and/or ulna	0.0435
Egypt	2000	Fracture of radius and/or ulna	0.0435
Egypt	2005	Fracture of radius and/or ulna	0.0435
Egypt	2010	Fracture of radius and/or ulna	0.0435
Egypt	2017	Fracture of radius and/or ulna	0.0435
Egypt	1990	Fracture of skull	0.0714
Egypt	1995	Fracture of skull	0.0714
Egypt	2000	Fracture of skull	0.0714
Egypt	2005	Fracture of skull	0.0714
Egypt	2010	Fracture of skull	0.0714
Egypt	2017	Fracture of skull	0.0714
Egypt	1990	Fracture of sternum and/or fracture of one or more ribs	0.1027
Egypt	1995	Fracture of sternum and/or fracture of one or more ribs	0.1027
Egypt	2000	Fracture of sternum and/or fracture of one or more ribs	0.1027
Egypt	2005	Fracture of sternum and/or fracture of one or more ribs	0.1027
Egypt	2010	Fracture of sternum and/or fracture of one or more ribs	0.1027
Egypt	2017	Fracture of sternum and/or fracture of one or more ribs	0.1027
Egypt	1990	Fracture of vertebral column	0.1106
Egypt	1995	Fracture of vertebral column	0.1106
Egypt	2000	Fracture of vertebral column	0.1106
Egypt	2005	Fracture of vertebral column	0.1106
Egypt	2010	Fracture of vertebral column	0.1106
Egypt	2017	Fracture of vertebral column	0.1106
Egypt	1990	Fracture of femur, other than femoral neck	0.042
Egypt	1995	Fracture of femur, other than femoral neck	0.042
Egypt	2000	Fracture of femur, other than femoral neck	0.042
Egypt	2005	Fracture of femur, other than femoral neck	0.042
Egypt	2010	Fracture of femur, other than femoral neck	0.042
Egypt	2017	Fracture of femur, other than femoral neck	0.042
Egypt	1990	Minor TBI	0.1319
Egypt	1995	Minor TBI	0.1319
Egypt	2000	Minor TBI	0.1319
Egypt	2005	Minor TBI	0.1319
Egypt	2010	Minor TBI	0.1319
Egypt	2017	Minor TBI	0.1319
Egypt	1990	Moderate TBI	0.1637
Egypt	1995	Moderate TBI	0.1637
Egypt	2000	Moderate TBI	0.1637
Egypt	2005	Moderate TBI	0.1637
Egypt	2010	Moderate TBI	0.1637
Egypt	2017	Moderate TBI	0.1637
Egypt	1990	Amputation of fingers (excluding thumb)	0.0047
Egypt	1995	Amputation of fingers (excluding thumb)	0.0047
Egypt	2000	Amputation of fingers (excluding thumb)	0.0047
Egypt	2005	Amputation of fingers (excluding thumb)	0.0047
Egypt	2010	Amputation of fingers (excluding thumb)	0.0047
Egypt	2017	Amputation of fingers (excluding thumb)	0.0047
Egypt	1990	Spinal cord lesion at neck level	0.4747
Egypt	1995	Spinal cord lesion at neck level	0.4663
Egypt	2000	Spinal cord lesion at neck level	0.4569
Egypt	2005	Spinal cord lesion at neck level	0.4503
Egypt	2010	Spinal cord lesion at neck level	0.4452
Egypt	2017	Spinal cord lesion at neck level	0.4367
Egypt	1990	Spinal cord lesion below neck level	0.3105
Egypt	1995	Spinal cord lesion below neck level	0.2927
Egypt	2000	Spinal cord lesion below neck level	0.2728
Egypt	2005	Spinal cord lesion below neck level	0.259
Egypt	2010	Spinal cord lesion below neck level	0.2482
Egypt	2017	Spinal cord lesion below neck level	0.2302
Egypt	1990	Drowning and nonfatal submersion	0.2471
Egypt	1995	Drowning and nonfatal submersion	0.2471
Egypt	2000	Drowning and nonfatal submersion	0.2471
Egypt	2005	Drowning and nonfatal submersion	0.2471
Egypt	2010	Drowning and nonfatal submersion	0.2471
Egypt	2017	Drowning and nonfatal submersion	0.2471
Egypt	1990	Asphyxiation	0.2471
Egypt	1995	Asphyxiation	0.2471
Egypt	2000	Asphyxiation	0.2471
Egypt	2005	Asphyxiation	0.2471
Egypt	2010	Asphyxiation	0.2471
Egypt	2017	Asphyxiation	0.2471
Egypt	1990	Crush injury	0.1325
Egypt	1995	Crush injury	0.1325
Egypt	2000	Crush injury	0.1325
Egypt	2005	Crush injury	0.1325
Egypt	2010	Crush injury	0.1325
Egypt	2017	Crush injury	0.1325
Egypt	1990	Nerve injury	0.1129
Egypt	1995	Nerve injury	0.1129
Egypt	2000	Nerve injury	0.1129
Egypt	2005	Nerve injury	0.1129
Egypt	2010	Nerve injury	0.1129
Egypt	2017	Nerve injury	0.1129
Egypt	1990	Injury to eyes	0.0165
Egypt	1995	Injury to eyes	0.0165
Egypt	2000	Injury to eyes	0.0165
Egypt	2005	Injury to eyes	0.0165
Egypt	2010	Injury to eyes	0.0165
Egypt	2017	Injury to eyes	0.0165
Egypt	1990	Amputation of lower limb, unilateral	0.1099
Egypt	1995	Amputation of lower limb, unilateral	0.1004
Egypt	2000	Amputation of lower limb, unilateral	0.0898
Egypt	2005	Amputation of lower limb, unilateral	0.0824
Egypt	2010	Amputation of lower limb, unilateral	0.0766
Egypt	2017	Amputation of lower limb, unilateral	0.067
Egypt	1990	Open wound(s)	0.011
Egypt	1995	Open wound(s)	0.011
Egypt	2000	Open wound(s)	0.011
Egypt	2005	Open wound(s)	0.011
Egypt	2010	Open wound(s)	0.011
Egypt	2017	Open wound(s)	0.011
Egypt	1990	Poisoning requiring urgent care	0.1628
Egypt	1995	Poisoning requiring urgent care	0.1628
Egypt	2000	Poisoning requiring urgent care	0.1628
Egypt	2005	Poisoning requiring urgent care	0.1628
Egypt	2010	Poisoning requiring urgent care	0.1628
Egypt	2017	Poisoning requiring urgent care	0.1628
Egypt	1990	Severe chest injury	0.0474
Egypt	1995	Severe chest injury	0.0474
Egypt	2000	Severe chest injury	0.0474
Egypt	2005	Severe chest injury	0.0474
Egypt	2010	Severe chest injury	0.0474
Egypt	2017	Severe chest injury	0.0474
Egypt	1990	Internal hemorrhage in abdomen and pelvis	0.1143
Egypt	1995	Internal hemorrhage in abdomen and pelvis	0.1143
Egypt	2000	Internal hemorrhage in abdomen and pelvis	0.1143
Egypt	2005	Internal hemorrhage in abdomen and pelvis	0.1143
Egypt	2010	Internal hemorrhage in abdomen and pelvis	0.1143
Egypt	2017	Internal hemorrhage in abdomen and pelvis	0.1143
Egypt	1990	Contusion in any part of the body	0.0075
Egypt	1995	Contusion in any part of the body	0.0075
Egypt	2000	Contusion in any part of the body	0.0075
Egypt	2005	Contusion in any part of the body	0.0075
Egypt	2010	Contusion in any part of the body	0.0075
Egypt	2017	Contusion in any part of the body	0.0075
Egypt	1990	Effect of different environmental factors	0.049
Egypt	1995	Effect of different environmental factors	0.049
Egypt	2000	Effect of different environmental factors	0.049
Egypt	2005	Effect of different environmental factors	0.049
Egypt	2010	Effect of different environmental factors	0.049
Egypt	2017	Effect of different environmental factors	0.049
Egypt	1990	Complications following therapeutic procedures	0.0506
Egypt	1995	Complications following therapeutic procedures	0.0506
Egypt	2000	Complications following therapeutic procedures	0.0506
Egypt	2005	Complications following therapeutic procedures	0.0506
Egypt	2010	Complications following therapeutic procedures	0.0506
Egypt	2017	Complications following therapeutic procedures	0.0506
Egypt	1990	Multiple fractures, dislocations, crashes, wounds, sprains, and strains	0.2397
Egypt	1995	Multiple fractures, dislocations, crashes, wounds, sprains, and strains	0.2153
Egypt	2000	Multiple fractures, dislocations, crashes, wounds, sprains, and strains	0.1881
Egypt	2005	Multiple fractures, dislocations, crashes, wounds, sprains, and strains	0.1691
Egypt	2010	Multiple fractures, dislocations, crashes, wounds, sprains, and strains	0.1543
Egypt	2017	Multiple fractures, dislocations, crashes, wounds, sprains, and strains	0.1297
Egypt	1990	Amputation of upper limb, unilateral	0.0807
Egypt	1995	Amputation of upper limb, unilateral	0.0751
Egypt	2000	Amputation of upper limb, unilateral	0.0689
Egypt	2005	Amputation of upper limb, unilateral	0.0645
Egypt	2010	Amputation of upper limb, unilateral	0.0611
Egypt	2017	Amputation of upper limb, unilateral	0.0555
Egypt	1990	Amputation of thumb	0.0114
Egypt	1995	Amputation of thumb	0.0114
Egypt	2000	Amputation of thumb	0.0114
Egypt	2005	Amputation of thumb	0.0114
Egypt	2010	Amputation of thumb	0.0114
Egypt	2017	Amputation of thumb	0.0114
Egypt	1990	Amputation of toe/toes	0.0059
Egypt	1995	Amputation of toe/toes	0.0059
Egypt	2000	Amputation of toe/toes	0.0059
Egypt	2005	Amputation of toe/toes	0.0059
Egypt	2010	Amputation of toe/toes	0.0059
Egypt	2017	Amputation of toe/toes	0.0059
Egypt	1990	Burns, <20% total burned surface area without lower airway burns	0.0163
Egypt	1995	Burns, <20% total burned surface area without lower airway burns	0.0163
Egypt	2000	Burns, <20% total burned surface area without lower airway burns	0.0163
Egypt	2005	Burns, <20% total burned surface area without lower airway burns	0.0163
Egypt	2010	Burns, <20% total burned surface area without lower airway burns	0.0163
Egypt	2017	Burns, <20% total burned surface area without lower airway burns	0.0163
Egypt	1990	Burns, >=20% total burned surface area or >= 10% burned surface area if head/neck or hands/wrist involved w/o lower airway burns	0.304
Egypt	1995	Burns, >=20% total burned surface area or >= 10% burned surface area if head/neck or hands/wrist involved w/o lower airway burns	0.2814
Egypt	2000	Burns, >=20% total burned surface area or >= 10% burned surface area if head/neck or hands/wrist involved w/o lower airway burns	0.256
Egypt	2005	Burns, >=20% total burned surface area or >= 10% burned surface area if head/neck or hands/wrist involved w/o lower airway burns	0.2383
Egypt	2010	Burns, >=20% total burned surface area or >= 10% burned surface area if head/neck or hands/wrist involved w/o lower airway burns	0.2245
Egypt	2017	Burns, >=20% total burned surface area or >= 10% burned surface area if head/neck or hands/wrist involved w/o lower airway burns	0.2016
Iraq	1990	Amputation of lower limbs, bilateral	0.233
Iraq	1995	Amputation of lower limbs, bilateral	0.2342
Iraq	2000	Amputation of lower limbs, bilateral	0.2293
Iraq	2005	Amputation of lower limbs, bilateral	0.2205
Iraq	2010	Amputation of lower limbs, bilateral	0.214
Iraq	2017	Amputation of lower limbs, bilateral	0.2017
Iraq	1990	Lower airway burns	0.2252
Iraq	1995	Lower airway burns	0.2252
Iraq	2000	Lower airway burns	0.2252
Iraq	2005	Lower airway burns	0.2252
Iraq	2010	Lower airway burns	0.2252
Iraq	2017	Lower airway burns	0.2252
Iraq	1990	Dislocation of hip	0.0159
Iraq	1995	Dislocation of hip	0.0159
Iraq	2000	Dislocation of hip	0.0159
Iraq	2005	Dislocation of hip	0.0159
Iraq	2010	Dislocation of hip	0.0159
Iraq	2017	Dislocation of hip	0.0159
Iraq	1990	Dislocation of knee	0.1134
Iraq	1995	Dislocation of knee	0.1134
Iraq	2000	Dislocation of knee	0.1134
Iraq	2005	Dislocation of knee	0.1134
Iraq	2010	Dislocation of knee	0.1134
Iraq	2017	Dislocation of knee	0.1134
Iraq	1990	Dislocation of shoulder	0.062
Iraq	1995	Dislocation of shoulder	0.062
Iraq	2000	Dislocation of shoulder	0.062
Iraq	2005	Dislocation of shoulder	0.062
Iraq	2010	Dislocation of shoulder	0.062
Iraq	2017	Dislocation of shoulder	0.062
Iraq	1990	Muscle and tendon injuries, including sprains and strains lesser dislocations	0.0075
Iraq	1995	Muscle and tendon injuries, including sprains and strains lesser dislocations	0.0075
Iraq	2000	Muscle and tendon injuries, including sprains and strains lesser dislocations	0.0075
Iraq	2005	Muscle and tendon injuries, including sprains and strains lesser dislocations	0.0075
Iraq	2010	Muscle and tendon injuries, including sprains and strains lesser dislocations	0.0075
Iraq	2017	Muscle and tendon injuries, including sprains and strains lesser dislocations	0.0075
Iraq	1990	Fracture of clavicle, scapula, or humerus	0.0349
Iraq	1995	Fracture of clavicle, scapula, or humerus	0.0349
Iraq	2000	Fracture of clavicle, scapula, or humerus	0.0349
Iraq	2005	Fracture of clavicle, scapula, or humerus	0.0349
Iraq	2010	Fracture of clavicle, scapula, or humerus	0.0349
Iraq	2017	Fracture of clavicle, scapula, or humerus	0.0349
Iraq	1990	Fracture of face bones	0.0669
Iraq	1995	Fracture of face bones	0.0669
Iraq	2000	Fracture of face bones	0.0669
Iraq	2005	Fracture of face bones	0.0669
Iraq	2010	Fracture of face bones	0.0669
Iraq	2017	Fracture of face bones	0.0669
Iraq	1990	Fracture of foot bones except ankle	0.0264
Iraq	1995	Fracture of foot bones except ankle	0.0264
Iraq	2000	Fracture of foot bones except ankle	0.0264
Iraq	2005	Fracture of foot bones except ankle	0.0264
Iraq	2010	Fracture of foot bones except ankle	0.0264
Iraq	2017	Fracture of foot bones except ankle	0.0264
Iraq	1990	Fracture of hand (wrist and other distal part of hand)	0.0138
Iraq	1995	Fracture of hand (wrist and other distal part of hand)	0.0138
Iraq	2000	Fracture of hand (wrist and other distal part of hand)	0.0138
Iraq	2005	Fracture of hand (wrist and other distal part of hand)	0.0138
Iraq	2010	Fracture of hand (wrist and other distal part of hand)	0.0138
Iraq	2017	Fracture of hand (wrist and other distal part of hand)	0.0138
Iraq	1990	Fracture of hip	0.2028
Iraq	1995	Fracture of hip	0.2044

Iraq	2000	Fracture of hip	0.198
Iraq	2005	Fracture of hip	0.1863
Iraq	2010	Fracture of hip	0.1777
Iraq	2017	Fracture of hip	0.1615
Iraq	1990	Amputation of upper limbs, bilateral	0.2375
Iraq	1995	Amputation of upper limbs, bilateral	0.2392
Iraq	2000	Amputation of upper limbs, bilateral	0.2325
Iraq	2005	Amputation of upper limbs, bilateral	0.2204
Iraq	2010	Amputation of upper limbs, bilateral	0.2116
Iraq	2017	Amputation of upper limbs, bilateral	0.1948
Iraq	1990	Fracture of patella, tibia or fibula, or ankle	0.0554
Iraq	1995	Fracture of patella, tibia or fibula, or ankle	0.0554
Iraq	2000	Fracture of patella, tibia or fibula, or ankle	0.0554
Iraq	2005	Fracture of patella, tibia or fibula, or ankle	0.0554
Iraq	2010	Fracture of patella, tibia or fibula, or ankle	0.0554
Iraq	2017	Fracture of patella, tibia or fibula, or ankle	0.0554
Iraq	1990	Fracture of pelvis	0.1823
Iraq	1995	Fracture of pelvis	0.1823
Iraq	2000	Fracture of pelvis	0.1823
Iraq	2005	Fracture of pelvis	0.1823
Iraq	2010	Fracture of pelvis	0.1823
Iraq	2017	Fracture of pelvis	0.1823
Iraq	1990	Fracture of radius and/or ulna	0.0435
Iraq	1995	Fracture of radius and/or ulna	0.0435
Iraq	2000	Fracture of radius and/or ulna	0.0435
Iraq	2005	Fracture of radius and/or ulna	0.0435
Iraq	2010	Fracture of radius and/or ulna	0.0435
Iraq	2017	Fracture of radius and/or ulna	0.0435
Iraq	1990	Fracture of skull	0.0714
Iraq	1995	Fracture of skull	0.0714
Iraq	2000	Fracture of skull	0.0714
Iraq	2005	Fracture of skull	0.0714
Iraq	2010	Fracture of skull	0.0714
Iraq	2017	Fracture of skull	0.0714
Iraq	1990	Fracture of sternum and/or fracture of one or more ribs	0.1027
Iraq	1995	Fracture of sternum and/or fracture of one or more ribs	0.1027
Iraq	2000	Fracture of sternum and/or fracture of one or more ribs	0.1027
Iraq	2005	Fracture of sternum and/or fracture of one or more ribs	0.1027
Iraq	2010	Fracture of sternum and/or fracture of one or more ribs	0.1027
Iraq	2017	Fracture of sternum and/or fracture of one or more ribs	0.1027
Iraq	1990	Fracture of vertebral column	0.1106
Iraq	1995	Fracture of vertebral column	0.1106
Iraq	2000	Fracture of vertebral column	0.1106
Iraq	2005	Fracture of vertebral column	0.1106
Iraq	2010	Fracture of vertebral column	0.1106
Iraq	2017	Fracture of vertebral column	0.1106
Iraq	1990	Fracture of femur, other than femoral neck	0.042
Iraq	1995	Fracture of femur, other than femoral neck	0.042
Iraq	2000	Fracture of femur, other than femoral neck	0.042
Iraq	2005	Fracture of femur, other than femoral neck	0.042
Iraq	2010	Fracture of femur, other than femoral neck	0.042
Iraq	2017	Fracture of femur, other than femoral neck	0.042
Iraq	1990	Minor TBI	0.1319
Iraq	1995	Minor TBI	0.1319
Iraq	2000	Minor TBI	0.1319
Iraq	2005	Minor TBI	0.1319
Iraq	2010	Minor TBI	0.1319
Iraq	2017	Minor TBI	0.1319
Iraq	1990	Moderate TBI	0.1637
Iraq	1995	Moderate TBI	0.1637
Iraq	2000	Moderate TBI	0.1637
Iraq	2005	Moderate TBI	0.1637
Iraq	2010	Moderate TBI	0.1637
Iraq	2017	Moderate TBI	0.1637
Iraq	1990	Amputation of fingers (excluding thumb)	0.0047
Iraq	1995	Amputation of fingers (excluding thumb)	0.0047
Iraq	2000	Amputation of fingers (excluding thumb)	0.0047
Iraq	2005	Amputation of fingers (excluding thumb)	0.0047
Iraq	2010	Amputation of fingers (excluding thumb)	0.0047
Iraq	2017	Amputation of fingers (excluding thumb)	0.0047
Iraq	1990	Spinal cord lesion at neck level	0.462
Iraq	1995	Spinal cord lesion at neck level	0.4626
Iraq	2000	Spinal cord lesion at neck level	0.4603
Iraq	2005	Spinal cord lesion at neck level	0.4563
Iraq	2010	Spinal cord lesion at neck level	0.4533
Iraq	2017	Spinal cord lesion at neck level	0.4477
Iraq	1990	Spinal cord lesion below neck level	0.2836
Iraq	1995	Spinal cord lesion below neck level	0.2848
Iraq	2000	Spinal cord lesion below neck level	0.2801
Iraq	2005	Spinal cord lesion below neck level	0.2715
Iraq	2010	Spinal cord lesion below neck level	0.2653
Iraq	2017	Spinal cord lesion below neck level	0.2534
Iraq	1990	Drowning and nonfatal submersion	0.2471
Iraq	1995	Drowning and nonfatal submersion	0.2471
Iraq	2000	Drowning and nonfatal submersion	0.2471
Iraq	2005	Drowning and nonfatal submersion	0.2471
Iraq	2010	Drowning and nonfatal submersion	0.2471
Iraq	2017	Drowning and nonfatal submersion	0.2471
Iraq	1990	Asphyxiation	0.2471
Iraq	1995	Asphyxiation	0.2471
Iraq	2000	Asphyxiation	0.2471
Iraq	2005	Asphyxiation	0.2471
Iraq	2010	Asphyxiation	0.2471
Iraq	2017	Asphyxiation	0.2471
Iraq	1990	Crush injury	0.1325
Iraq	1995	Crush injury	0.1325
Iraq	2000	Crush injury	0.1325
Iraq	2005	Crush injury	0.1325
Iraq	2010	Crush injury	0.1325
Iraq	2017	Crush injury	0.1325
Iraq	1990	Nerve injury	0.1129
Iraq	1995	Nerve injury	0.1129
Iraq	2000	Nerve injury	0.1129
Iraq	2005	Nerve injury	0.1129
Iraq	2010	Nerve injury	0.1129
Iraq	2017	Nerve injury	0.1129
Iraq	1990	Injury to eyes	0.0165
Iraq	1995	Injury to eyes	0.0165
Iraq	2000	Injury to eyes	0.0165
Iraq	2005	Injury to eyes	0.0165
Iraq	2010	Injury to eyes	0.0165
Iraq	2017	Injury to eyes	0.0165
Iraq	1990	Amputation of lower limb, unilateral	0.0955
Iraq	1995	Amputation of lower limb, unilateral	0.0962
Iraq	2000	Amputation of lower limb, unilateral	0.0936
Iraq	2005	Amputation of lower limb, unilateral	0.0891
Iraq	2010	Amputation of lower limb, unilateral	0.0857
Iraq	2017	Amputation of lower limb, unilateral	0.0794
Iraq	1990	Open wound(s)	0.011
Iraq	1995	Open wound(s)	0.011
Iraq	2000	Open wound(s)	0.011
Iraq	2005	Open wound(s)	0.011
Iraq	2010	Open wound(s)	0.011
Iraq	2017	Open wound(s)	0.011
Iraq	1990	Poisoning requiring urgent care	0.1628
Iraq	1995	Poisoning requiring urgent care	0.1628
Iraq	2000	Poisoning requiring urgent care	0.1628
Iraq	2005	Poisoning requiring urgent care	0.1628
Iraq	2010	Poisoning requiring urgent care	0.1628
Iraq	2017	Poisoning requiring urgent care	0.1628
Iraq	1990	Severe chest injury	0.0474
Iraq	1995	Severe chest injury	0.0474
Iraq	2000	Severe chest injury	0.0474
Iraq	2005	Severe chest injury	0.0474
Iraq	2010	Severe chest injury	0.0474
Iraq	2017	Severe chest injury	0.0474
Iraq	1990	Internal hemorrhage in abdomen and pelvis	0.1143
Iraq	1995	Internal hemorrhage in abdomen and pelvis	0.1143
Iraq	2000	Internal hemorrhage in abdomen and pelvis	0.1143
Iraq	2005	Internal hemorrhage in abdomen and pelvis	0.1143
Iraq	2010	Internal hemorrhage in abdomen and pelvis	0.1143
Iraq	2017	Internal hemorrhage in abdomen and pelvis	0.1143
Iraq	1990	Contusion in any part of the body	0.0075
Iraq	1995	Contusion in any part of the body	0.0075
Iraq	2000	Contusion in any part of the body	0.0075
Iraq	2005	Contusion in any part of the body	0.0075
Iraq	2010	Contusion in any part of the body	0.0075
Iraq	2017	Contusion in any part of the body	0.0075
Iraq	1990	Effect of different environmental factors	0.049
Iraq	1995	Effect of different environmental factors	0.049
Iraq	2000	Effect of different environmental factors	0.049
Iraq	2005	Effect of different environmental factors	0.049
Iraq	2010	Effect of different environmental factors	0.049
Iraq	2017	Effect of different environmental factors	0.049
Iraq	1990	Complications following therapeutic procedures	0.0506
Iraq	1995	Complications following therapeutic procedures	0.0506
Iraq	2000	Complications following therapeutic procedures	0.0506
Iraq	2005	Complications following therapeutic procedures	0.0506
Iraq	2010	Complications following therapeutic procedures	0.0506
Iraq	2017	Complications following therapeutic procedures	0.0506
Iraq	1990	Multiple fractures, dislocations, crashes, wounds, sprains, and strains	0.2028
Iraq	1995	Multiple fractures, dislocations, crashes, wounds, sprains, and strains	0.2044
Iraq	2000	Multiple fractures, dislocations, crashes, wounds, sprains, and strains	0.198
Iraq	2005	Multiple fractures, dislocations, crashes, wounds, sprains, and strains	0.1863
Iraq	2010	Multiple fractures, dislocations, crashes, wounds, sprains, and strains	0.1777
Iraq	2017	Multiple fractures, dislocations, crashes, wounds, sprains, and strains	0.1615
Iraq	1990	Amputation of upper limb, unilateral	0.0723
Iraq	1995	Amputation of upper limb, unilateral	0.0726
Iraq	2000	Amputation of upper limb, unilateral	0.0712
Iraq	2005	Amputation of upper limb, unilateral	0.0685
Iraq	2010	Amputation of upper limb, unilateral	0.0665
Iraq	2017	Amputation of upper limb, unilateral	0.0628
Iraq	1990	Amputation of thumb	0.0114
Iraq	1995	Amputation of thumb	0.0114
Iraq	2000	Amputation of thumb	0.0114
Iraq	2005	Amputation of thumb	0.0114
Iraq	2010	Amputation of thumb	0.0114
Iraq	2017	Amputation of thumb	0.0114
Iraq	1990	Amputation of toe/toes	0.0059
Iraq	1995	Amputation of toe/toes	0.0059
Iraq	2000	Amputation of toe/toes	0.0059
Iraq	2005	Amputation of toe/toes	0.0059
Iraq	2010	Amputation of toe/toes	0.0059
Iraq	2017	Amputation of toe/toes	0.0059
Iraq	1990	Burns, <20% total burned surface area without lower airway burns	0.0163
Iraq	1995	Burns, <20% total burned surface area without lower airway burns	0.0163
Iraq	2000	Burns, <20% total burned surface area without lower airway burns	0.0163
Iraq	2005	Burns, <20% total burned surface area without lower airway burns	0.0163
Iraq	2010	Burns, <20% total burned surface area without lower airway burns	0.0163
Iraq	2017	Burns, <20% total burned surface area without lower airway burns	0.0163
Iraq	1990	Burns, >=20% total burned surface area or >= 10% burned surface area if head/neck or hands/wrist involved w/o lower airway burns	0.2697
Iraq	1995	Burns, >=20% total burned surface area or >= 10% burned surface area if head/neck or hands/wrist involved w/o lower airway burns	0.2712
Iraq	2000	Burns, >=20% total burned surface area or >= 10% burned surface area if head/neck or hands/wrist involved w/o lower airway burns	0.2652
Iraq	2005	Burns, >=20% total burned surface area or >= 10% burned surface area if head/neck or hands/wrist involved w/o lower airway burns	0.2543
Iraq	2010	Burns, >=20% total burned surface area or >= 10% burned surface area if head/neck or hands/wrist involved w/o lower airway burns	0.2464
Iraq	2017	Burns, >=20% total burned surface area or >= 10% burned surface area if head/neck or hands/wrist involved w/o lower airway burns	0.2312
Jordan	1990	Amputation of lower limbs, bilateral	0.2076
Jordan	1995	Amputation of lower limbs, bilateral	0.1948
Jordan	2000	Amputation of lower limbs, bilateral	0.1801
Jordan	2005	Amputation of lower limbs, bilateral	0.1728
Jordan	2010	Amputation of lower limbs, bilateral	0.1534
Jordan	2017	Amputation of lower limbs, bilateral	0.1374
Jordan	1990	Lower airway burns	0.2252
Jordan	1995	Lower airway burns	0.2252
Jordan	2000	Lower airway burns	0.2252
Jordan	2005	Lower airway burns	0.2252
Jordan	2010	Lower airway burns	0.2252
Jordan	2017	Lower airway burns	0.2252
Jordan	1990	Dislocation of hip	0.0159
Jordan	1995	Dislocation of hip	0.0159
Jordan	2000	Dislocation of hip	0.0159
Jordan	2005	Dislocation of hip	0.0159
Jordan	2010	Dislocation of hip	0.0159
Jordan	2017	Dislocation of hip	0.0159
Jordan	1990	Dislocation of knee	0.1134
Jordan	1995	Dislocation of knee	0.1134
Jordan	2000	Dislocation of knee	0.1134
Jordan	2005	Dislocation of knee	0.1134
Jordan	2010	Dislocation of knee	0.1134
Jordan	2017	Dislocation of knee	0.1134
Jordan	1990	Dislocation of shoulder	0.062
Jordan	1995	Dislocation of shoulder	0.062
Jordan	2000	Dislocation of shoulder	0.062
Jordan	2005	Dislocation of shoulder	0.062
Jordan	2010	Dislocation of shoulder	0.062
Jordan	2017	Dislocation of shoulder	0.062
Jordan	1990	Muscle and tendon injuries, including sprains and strains lesser dislocations	0.0075
Jordan	1995	Muscle and tendon injuries, including sprains and strains lesser dislocations	0.0075
Jordan	2000	Muscle and tendon injuries, including sprains and strains lesser dislocations	0.0075
Jordan	2005	Muscle and tendon injuries, including sprains and strains lesser dislocations	0.0075
Jordan	2010	Muscle and tendon injuries, including sprains and strains lesser dislocations	0.0075
Jordan	2017	Muscle and tendon injuries, including sprains and strains lesser dislocations	0.0075
Jordan	1990	Fracture of clavicle, scapula, or humerus	0.0349
Jordan	1995	Fracture of clavicle, scapula, or humerus	0.0349
Jordan	2000	Fracture of clavicle, scapula, or humerus	0.0349
Jordan	2005	Fracture of clavicle, scapula, or humerus	0.0349
Jordan	2010	Fracture of clavicle, scapula, or humerus	0.0349
Jordan	2017	Fracture of clavicle, scapula, or humerus	0.0349
Jordan	1990	Fracture of face bones	0.0669
Jordan	1995	Fracture of face bones	0.0669
Jordan	2000	Fracture of face bones	0.0669
Jordan	2005	Fracture of face bones	0.0669
Jordan	2010	Fracture of face bones	0.0669
Jordan	2017	Fracture of face bones	0.0669
Jordan	1990	Fracture of foot bones except ankle	0.0264
Jordan	1995	Fracture of foot bones except ankle	0.0264
Jordan	2000	Fracture of foot bones except ankle	0.0264
Jordan	2005	Fracture of foot bones except ankle	0.0264
Jordan	2010	Fracture of foot bones except ankle	0.0264
Jordan	2017	Fracture of foot bones except ankle	0.0264
Jordan	1990	Fracture of hand (wrist and other distal part of hand)	0.0138
Jordan	1995	Fracture of hand (wrist and other distal part of hand)	0.0138
Jordan	2000	Fracture of hand (wrist and other distal part of hand)	0.0138
Jordan	2005	Fracture of hand (wrist and other distal part of hand)	0.0138
Jordan	2010	Fracture of hand (wrist and other distal part of hand)	0.0138
Jordan	2017	Fracture of hand (wrist and other distal part of hand)	0.0138
Jordan	1990	Fracture of hip	0.1693
Jordan	1995	Fracture of hip	0.1523
Jordan	2000	Fracture of hip	0.1329
Jordan	2005	Fracture of hip	0.1232
Jordan	2010	Fracture of hip	0.0976
Jordan	2017	Fracture of hip	0.0764
Jordan	1990	Amputation of upper limbs, bilateral	0.2028
Jordan	1995	Amputation of upper limbs, bilateral	0.1854
Jordan	2000	Amputation of upper limbs, bilateral	0.1653
Jordan	2005	Amputation of upper limbs, bilateral	0.1553
Jordan	2010	Amputation of upper limbs, bilateral	0.1288
Jordan	2017	Amputation of upper limbs, bilateral	0.107
Jordan	1990	Fracture of patella, tibia or fibula, or ankle	0.0554
Jordan	1995	Fracture of patella, tibia or fibula, or ankle	0.0554
Jordan	2000	Fracture of patella, tibia or fibula, or ankle	0.0554
Jordan	2005	Fracture of patella, tibia or fibula, or ankle	0.0554
Jordan	2010	Fracture of patella, tibia or fibula, or ankle	0.0554
Jordan	2017	Fracture of patella, tibia or fibula, or ankle	0.0554
Jordan	1990	Fracture of pelvis	0.1823
Jordan	1995	Fracture of pelvis	0.1823
Jordan	2000	Fracture of pelvis	0.1823
Jordan	2005	Fracture of pelvis	0.1823
Jordan	2010	Fracture of pelvis	0.1823
Jordan	2017	Fracture of pelvis	0.1823
Jordan	1990	Fracture of radius and/or ulna	0.0435
Jordan	1995	Fracture of radius and/or ulna	0.0435
Jordan	2000	Fracture of radius and/or ulna	0.0435
Jordan	2005	Fracture of radius and/or ulna	0.0435
Jordan	2010	Fracture of radius and/or ulna	0.0435
Jordan	2017	Fracture of radius and/or ulna	0.0435
Jordan	1990	Fracture of skull	0.0714
Jordan	1995	Fracture of skull	0.0714
Jordan	2000	Fracture of skull	0.0714
Jordan	2005	Fracture of skull	0.0714
Jordan	2010	Fracture of skull	0.0714
Jordan	2017	Fracture of skull	0.0714
Jordan	1990	Fracture of sternum and/or fracture of one or more ribs	0.1027
Jordan	1995	Fracture of sternum and/or fracture of one or more ribs	0.1027
Jordan	2000	Fracture of sternum and/or fracture of one or more ribs	0.1027
Jordan	2005	Fracture of sternum and/or fracture of one or more ribs	0.1027
Jordan	2010	Fracture of sternum and/or fracture of one or more ribs	0.1027
Jordan	2017	Fracture of sternum and/or fracture of one or more ribs	0.1027
Jordan	1990	Fracture of vertebral column	0.1106
Jordan	1995	Fracture of vertebral column	0.1106
Jordan	2000	Fracture of vertebral column	0.1106
Jordan	2005	Fracture of vertebral column	0.1106
Jordan	2010	Fracture of vertebral column	0.1106
Jordan	2017	Fracture of vertebral column	0.1106
Jordan	1990	Fracture of femur, other than femoral neck	0.042
Jordan	1995	Fracture of femur, other than femoral neck	0.042
Jordan	2000	Fracture of femur, other than femoral neck	0.042
Jordan	2005	Fracture of femur, other than femoral neck	0.042
Jordan	2010	Fracture of femur, other than femoral neck	0.042
Jordan	2017	Fracture of femur, other than femoral neck	0.042
Jordan	1990	Minor TBI	0.1319
Jordan	1995	Minor TBI	0.1319
Jordan	2000	Minor TBI	0.1319
Jordan	2005	Minor TBI	0.1319
Jordan	2010	Minor TBI	0.1319
Jordan	2017	Minor TBI	0.1319
Jordan	1990	Moderate TBI	0.1637
Jordan	1995	Moderate TBI	0.1637
Jordan	2000	Moderate TBI	0.1637
Jordan	2005	Moderate TBI	0.1637
Jordan	2010	Moderate TBI	0.1637
Jordan	2017	Moderate TBI	0.1637
Jordan	1990	Amputation of fingers (excluding thumb)	0.0047
Jordan	1995	Amputation of fingers (excluding thumb)	0.0047
Jordan	2000	Amputation of fingers (excluding thumb)	0.0047
Jordan	2005	Amputation of fingers (excluding thumb)	0.0047
Jordan	2010	Amputation of fingers (excluding thumb)	0.0047
Jordan	2017	Amputation of fingers (excluding thumb)	0.0047
Jordan	1990	Spinal cord lesion at neck level	0.4504
Jordan	1995	Spinal cord lesion at neck level	0.4446
Jordan	2000	Spinal cord lesion at neck level	0.4378
Jordan	2005	Spinal cord lesion at neck level	0.4345
Jordan	2010	Spinal cord lesion at neck level	0.4256
Jordan	2017	Spinal cord lesion at neck level	0.4183
Jordan	1990	Spinal cord lesion below neck level	0.2591
Jordan	1995	Spinal cord lesion below neck level	0.2467
Jordan	2000	Spinal cord lesion below neck level	0.2326
Jordan	2005	Spinal cord lesion below neck level	0.2255
Jordan	2010	Spinal cord lesion below neck level	0.2068
Jordan	2017	Spinal cord lesion below neck level	0.1913
Jordan	1990	Drowning and nonfatal submersion	0.2471
Jordan	1995	Drowning and nonfatal submersion	0.2471
Jordan	2000	Drowning and nonfatal submersion	0.2471
Jordan	2005	Drowning and nonfatal submersion	0.2471
Jordan	2010	Drowning and nonfatal submersion	0.2471
Jordan	2017	Drowning and nonfatal submersion	0.2471
Jordan	1990	Asphyxiation	0.2471
Jordan	1995	Asphyxiation	0.2471
Jordan	2000	Asphyxiation	0.2471
Jordan	2005	Asphyxiation	0.2471
Jordan	2010	Asphyxiation	0.2471
Jordan	2017	Asphyxiation	0.2471
Jordan	1990	Crush injury	0.1325
Jordan	1995	Crush injury	0.1325
Jordan	2000	Crush injury	0.1325
Jordan	2005	Crush injury	0.1325
Jordan	2010	Crush injury	0.1325
Jordan	2017	Crush injury	0.1325
Jordan	1990	Nerve injury	0.1129
Jordan	1995	Nerve injury	0.1129
Jordan	2000	Nerve injury	0.1129
Jordan	2005	Nerve injury	0.1129
Jordan	2010	Nerve injury	0.1129
Jordan	2017	Nerve injury	0.1129
Jordan	1990	Injury to eyes	0.0165
Jordan	1995	Injury to eyes	0.0165
Jordan	2000	Injury to eyes	0.0165
Jordan	2005	Injury to eyes	0.0165
Jordan	2010	Injury to eyes	0.0165
Jordan	2017	Injury to eyes	0.0165
Jordan	1990	Amputation of lower limb, unilateral	0.0825
Jordan	1995	Amputation of lower limb, unilateral	0.0759
Jordan	2000	Amputation of lower limb, unilateral	0.0683
Jordan	2005	Amputation of lower limb, unilateral	0.0645
Jordan	2010	Amputation of lower limb, unilateral	0.0545
Jordan	2017	Amputation of lower limb, unilateral	0.0463
Jordan	1990	Open wound(s)	0.011
Jordan	1995	Open wound(s)	0.011
Jordan	2000	Open wound(s)	0.011
Jordan	2005	Open wound(s)	0.011
Jordan	2010	Open wound(s)	0.011
Jordan	2017	Open wound(s)	0.011
Jordan	1990	Poisoning requiring urgent care	0.1628
Jordan	1995	Poisoning requiring urgent care	0.1628
Jordan	2000	Poisoning requiring urgent care	0.1628
Jordan	2005	Poisoning requiring urgent care	0.1628
Jordan	2010	Poisoning requiring urgent care	0.1628
Jordan	2017	Poisoning requiring urgent care	0.1628
Jordan	1990	Severe chest injury	0.0474
Jordan	1995	Severe chest injury	0.0474
Jordan	2000	Severe chest injury	0.0474
Jordan	2005	Severe chest injury	0.0474
Jordan	2010	Severe chest injury	0.0474
Jordan	2017	Severe chest injury	0.0474
Jordan	1990	Internal hemorrhage in abdomen and pelvis	0.1143
Jordan	1995	Internal hemorrhage in abdomen and pelvis	0.1143
Jordan	2000	Internal hemorrhage in abdomen and pelvis	0.1143
Jordan	2005	Internal hemorrhage in abdomen and pelvis	0.1143
Jordan	2010	Internal hemorrhage in abdomen and pelvis	0.1143
Jordan	2017	Internal hemorrhage in abdomen and pelvis	0.1143
Jordan	1990	Contusion in any part of the body	0.0075
Jordan	1995	Contusion in any part of the body	0.0075
Jordan	2000	Contusion in any part of the body	0.0075
Jordan	2005	Contusion in any part of the body	0.0075
Jordan	2010	Contusion in any part of the body	0.0075
Jordan	2017	Contusion in any part of the body	0.0075
Jordan	1990	Effect of different environmental factors	0.049
Jordan	1995	Effect of different environmental factors	0.049
Jordan	2000	Effect of different environmental factors	0.049
Jordan	2005	Effect of different environmental factors	0.049
Jordan	2010	Effect of different environmental factors	0.049
Jordan	2017	Effect of different environmental factors	0.049
Jordan	1990	Complications following therapeutic procedures	0.0506
Jordan	1995	Complications following therapeutic procedures	0.0506
Jordan	2000	Complications following therapeutic procedures	0.0506
Jordan	2005	Complications following therapeutic procedures	0.0506
Jordan	2010	Complications following therapeutic procedures	0.0506
Jordan	2017	Complications following therapeutic procedures	0.0506
Jordan	1990	Multiple fractures, dislocations, crashes, wounds, sprains, and strains	0.1693
Jordan	1995	Multiple fractures, dislocations, crashes, wounds, sprains, and strains	0.1523
Jordan	2000	Multiple fractures, dislocations, crashes, wounds, sprains, and strains	0.1329
Jordan	2005	Multiple fractures, dislocations, crashes, wounds, sprains, and strains	0.1232
Jordan	2010	Multiple fractures, dislocations, crashes, wounds, sprains, and strains	0.0976
Jordan	2017	Multiple fractures, dislocations, crashes, wounds, sprains, and strains	0.0764
Jordan	1990	Amputation of upper limb, unilateral	0.0646
Jordan	1995	Amputation of upper limb, unilateral	0.0607
Jordan	2000	Amputation of upper limb, unilateral	0.0562
Jordan	2005	Amputation of upper limb, unilateral	0.054
Jordan	2010	Amputation of upper limb, unilateral	0.0481
Jordan	2017	Amputation of upper limb, unilateral	0.0433
Jordan	1990	Amputation of thumb	0.0114

Jordan	1995	Amputation of thumb	0.0114
Jordan	2000	Amputation of thumb	0.0114
Jordan	2005	Amputation of thumb	0.0114
Jordan	2010	Amputation of thumb	0.0114
Jordan	2017	Amputation of thumb	0.0114
Jordan	1990	Amputation of toe/toes	0.0059
Jordan	1995	Amputation of toe/toes	0.0059
Jordan	2000	Amputation of toe/toes	0.0059
Jordan	2005	Amputation of toe/toes	0.0059
Jordan	2010	Amputation of toe/toes	0.0059
Jordan	2017	Amputation of toe/toes	0.0059
Jordan	1990	Burns, <20% total burned surface area without lower airway burns	0.0163
Jordan	1995	Burns, <20% total burned surface area without lower airway burns	0.0163
Jordan	2000	Burns, <20% total burned surface area without lower airway burns	0.0163
Jordan	2005	Burns, <20% total burned surface area without lower airway burns	0.0163
Jordan	2010	Burns, <20% total burned surface area without lower airway burns	0.0163
Jordan	2017	Burns, <20% total burned surface area without lower airway burns	0.0163
Jordan	1990	Burns, >=20% total burned surface area or >= 10% burned surface area if head/neck or hands/wrist involved w/o lower airway burns	0.2385
Jordan	1995	Burns, >=20% total burned surface area or >= 10% burned surface area if head/neck or hands/wrist involved w/o lower airway burns	0.2227
Jordan	2000	Burns, >=20% total burned surface area or >= 10% burned surface area if head/neck or hands/wrist involved w/o lower airway burns	0.2046
Jordan	2005	Burns, >=20% total burned surface area or >= 10% burned surface area if head/neck or hands/wrist involved w/o lower airway burns	0.1956
Jordan	2010	Burns, >=20% total burned surface area or >= 10% burned surface area if head/neck or hands/wrist involved w/o lower airway burns	0.1717
Jordan	2017	Burns, >=20% total burned surface area or >= 10% burned surface area if head/neck or hands/wrist involved w/o lower airway burns	0.152
Kuwait	1990	Amputation of lower limbs, bilateral	0.1504
Kuwait	1995	Amputation of lower limbs, bilateral	0.1493
Kuwait	2000	Amputation of lower limbs, bilateral	0.1374
Kuwait	2005	Amputation of lower limbs, bilateral	0.1339
Kuwait	2010	Amputation of lower limbs, bilateral	0.1233
Kuwait	2017	Amputation of lower limbs, bilateral	0.1232
Kuwait	1990	Lower airway burns	0.2252
Kuwait	1995	Lower airway burns	0.2252
Kuwait	2000	Lower airway burns	0.2252
Kuwait	2005	Lower airway burns	0.2252
Kuwait	2010	Lower airway burns	0.2252
Kuwait	2017	Lower airway burns	0.2252
Kuwait	1990	Dislocation of hip	0.0159
Kuwait	1995	Dislocation of hip	0.0159
Kuwait	2000	Dislocation of hip	0.0159
Kuwait	2005	Dislocation of hip	0.0159
Kuwait	2010	Dislocation of hip	0.0159
Kuwait	2017	Dislocation of hip	0.0159
Kuwait	1990	Dislocation of knee	0.1134
Kuwait	1995	Dislocation of knee	0.1134
Kuwait	2000	Dislocation of knee	0.1134
Kuwait	2005	Dislocation of knee	0.1134
Kuwait	2010	Dislocation of knee	0.1134
Kuwait	2017	Dislocation of knee	0.1134
Kuwait	1990	Dislocation of shoulder	0.062
Kuwait	1995	Dislocation of shoulder	0.062
Kuwait	2000	Dislocation of shoulder	0.062
Kuwait	2005	Dislocation of shoulder	0.062
Kuwait	2010	Dislocation of shoulder	0.062
Kuwait	2017	Dislocation of shoulder	0.062
Kuwait	1990	Muscle and tendon injuries, including sprains and strains lesser dislocations	0.0075
Kuwait	1995	Muscle and tendon injuries, including sprains and strains lesser dislocations	0.0075
Kuwait	2000	Muscle and tendon injuries, including sprains and strains lesser dislocations	0.0075
Kuwait	2005	Muscle and tendon injuries, including sprains and strains lesser dislocations	0.0075
Kuwait	2010	Muscle and tendon injuries, including sprains and strains lesser dislocations	0.0075
Kuwait	2017	Muscle and tendon injuries, including sprains and strains lesser dislocations	0.0075
Kuwait	1990	Fracture of clavicle, scapula, or humerus	0.0349
Kuwait	1995	Fracture of clavicle, scapula, or humerus	0.0349
Kuwait	2000	Fracture of clavicle, scapula, or humerus	0.0349
Kuwait	2005	Fracture of clavicle, scapula, or humerus	0.0349
Kuwait	2010	Fracture of clavicle, scapula, or humerus	0.0349
Kuwait	2017	Fracture of clavicle, scapula, or humerus	0.0349
Kuwait	1990	Fracture of face bones	0.0669
Kuwait	1995	Fracture of face bones	0.0669
Kuwait	2000	Fracture of face bones	0.0669
Kuwait	2005	Fracture of face bones	0.0669
Kuwait	2010	Fracture of face bones	0.0669
Kuwait	2017	Fracture of face bones	0.0669
Kuwait	1990	Fracture of foot bones except ankle	0.0264
Kuwait	1995	Fracture of foot bones except ankle	0.0264
Kuwait	2000	Fracture of foot bones except ankle	0.0264
Kuwait	2005	Fracture of foot bones except ankle	0.0264
Kuwait	2010	Fracture of foot bones except ankle	0.0264
Kuwait	2017	Fracture of foot bones except ankle	0.0264
Kuwait	1990	Fracture of hand (wrist and other distal part of hand)	0.0138
Kuwait	1995	Fracture of hand (wrist and other distal part of hand)	0.0138
Kuwait	2000	Fracture of hand (wrist and other distal part of hand)	0.0138
Kuwait	2005	Fracture of hand (wrist and other distal part of hand)	0.0138
Kuwait	2010	Fracture of hand (wrist and other distal part of hand)	0.0138
Kuwait	2017	Fracture of hand (wrist and other distal part of hand)	0.0138
Kuwait	1990	Fracture of hip	0.0936
Kuwait	1995	Fracture of hip	0.0921
Kuwait	2000	Fracture of hip	0.0764
Kuwait	2005	Fracture of hip	0.0717
Kuwait	2010	Fracture of hip	0.0577
Kuwait	2017	Fracture of hip	0.0577
Kuwait	1990	Amputation of upper limbs, bilateral	0.1248
Kuwait	1995	Amputation of upper limbs, bilateral	0.1231
Kuwait	2000	Amputation of upper limbs, bilateral	0.107
Kuwait	2005	Amputation of upper limbs, bilateral	0.1021
Kuwait	2010	Amputation of upper limbs, bilateral	0.0876
Kuwait	2017	Amputation of upper limbs, bilateral	0.0876
Kuwait	1990	Fracture of patella, tibia or fibula, or ankle	0.0554
Kuwait	1995	Fracture of patella, tibia or fibula, or ankle	0.0554
Kuwait	2000	Fracture of patella, tibia or fibula, or ankle	0.0554
Kuwait	2005	Fracture of patella, tibia or fibula, or ankle	0.0554
Kuwait	2010	Fracture of patella, tibia or fibula, or ankle	0.0554
Kuwait	2017	Fracture of patella, tibia or fibula, or ankle	0.0554
Kuwait	1990	Fracture of pelvis	0.1823
Kuwait	1995	Fracture of pelvis	0.1823
Kuwait	2000	Fracture of pelvis	0.1823
Kuwait	2005	Fracture of pelvis	0.1823
Kuwait	2010	Fracture of pelvis	0.1823
Kuwait	2017	Fracture of pelvis	0.1823
Kuwait	1990	Fracture of radius and/or ulna	0.0435
Kuwait	1995	Fracture of radius and/or ulna	0.0435
Kuwait	2000	Fracture of radius and/or ulna	0.0435
Kuwait	2005	Fracture of radius and/or ulna	0.0435
Kuwait	2010	Fracture of radius and/or ulna	0.0435
Kuwait	2017	Fracture of radius and/or ulna	0.0435
Kuwait	1990	Fracture of skull	0.0714
Kuwait	1995	Fracture of skull	0.0714
Kuwait	2000	Fracture of skull	0.0714
Kuwait	2005	Fracture of skull	0.0714
Kuwait	2010	Fracture of skull	0.0714
Kuwait	2017	Fracture of skull	0.0714
Kuwait	1990	Fracture of sternum and/or fracture of one or more ribs	0.1027
Kuwait	1995	Fracture of sternum and/or fracture of one or more ribs	0.1027
Kuwait	2000	Fracture of sternum and/or fracture of one or more ribs	0.1027
Kuwait	2005	Fracture of sternum and/or fracture of one or more ribs	0.1027
Kuwait	2010	Fracture of sternum and/or fracture of one or more ribs	0.1027
Kuwait	2017	Fracture of sternum and/or fracture of one or more ribs	0.1027
Kuwait	1990	Fracture of vertebral column	0.1106
Kuwait	1995	Fracture of vertebral column	0.1106
Kuwait	2000	Fracture of vertebral column	0.1106
Kuwait	2005	Fracture of vertebral column	0.1106
Kuwait	2010	Fracture of vertebral column	0.1106
Kuwait	2017	Fracture of vertebral column	0.1106
Kuwait	1990	Fracture of femur, other than femoral neck	0.042
Kuwait	1995	Fracture of femur, other than femoral neck	0.042
Kuwait	2000	Fracture of femur, other than femoral neck	0.042
Kuwait	2005	Fracture of femur, other than femoral neck	0.042
Kuwait	2010	Fracture of femur, other than femoral neck	0.042
Kuwait	2017	Fracture of femur, other than femoral neck	0.042
Kuwait	1990	Minor TBI	0.1319
Kuwait	1995	Minor TBI	0.1319
Kuwait	2000	Minor TBI	0.1319
Kuwait	2005	Minor TBI	0.1319
Kuwait	2010	Minor TBI	0.1319
Kuwait	2017	Minor TBI	0.1319
Kuwait	1990	Moderate TBI	0.1637
Kuwait	1995	Moderate TBI	0.1637
Kuwait	2000	Moderate TBI	0.1637
Kuwait	2005	Moderate TBI	0.1637
Kuwait	2010	Moderate TBI	0.1637
Kuwait	2017	Moderate TBI	0.1637
Kuwait	1990	Amputation of fingers (excluding thumb)	0.0047
Kuwait	1995	Amputation of fingers (excluding thumb)	0.0047
Kuwait	2000	Amputation of fingers (excluding thumb)	0.0047
Kuwait	2005	Amputation of fingers (excluding thumb)	0.0047
Kuwait	2010	Amputation of fingers (excluding thumb)	0.0047
Kuwait	2017	Amputation of fingers (excluding thumb)	0.0047
Kuwait	1990	Spinal cord lesion at neck level	0.4243
Kuwait	1995	Spinal cord lesion at neck level	0.4237
Kuwait	2000	Spinal cord lesion at neck level	0.4183
Kuwait	2005	Spinal cord lesion at neck level	0.4167
Kuwait	2010	Spinal cord lesion at neck level	0.4118
Kuwait	2017	Spinal cord lesion at neck level	0.4118
Kuwait	1990	Spinal cord lesion below neck level	0.2039
Kuwait	1995	Spinal cord lesion below neck level	0.2028
Kuwait	2000	Spinal cord lesion below neck level	0.1914
Kuwait	2005	Spinal cord lesion below neck level	0.1879
Kuwait	2010	Spinal cord lesion below neck level	0.1777
Kuwait	2017	Spinal cord lesion below neck level	0.1777
Kuwait	1990	Drowning and nonfatal submersion	0.2471
Kuwait	1995	Drowning and nonfatal submersion	0.2471
Kuwait	2000	Drowning and nonfatal submersion	0.2471
Kuwait	2005	Drowning and nonfatal submersion	0.2471
Kuwait	2010	Drowning and nonfatal submersion	0.2471
Kuwait	2017	Drowning and nonfatal submersion	0.2471
Kuwait	1990	Asphyxiation	0.2471
Kuwait	1995	Asphyxiation	0.2471
Kuwait	2000	Asphyxiation	0.2471
Kuwait	2005	Asphyxiation	0.2471
Kuwait	2010	Asphyxiation	0.2471
Kuwait	2017	Asphyxiation	0.2471
Kuwait	1990	Crush injury	0.1325
Kuwait	1995	Crush injury	0.1325
Kuwait	2000	Crush injury	0.1325
Kuwait	2005	Crush injury	0.1325
Kuwait	2010	Crush injury	0.1325
Kuwait	2017	Crush injury	0.1325
Kuwait	1990	Nerve injury	0.1129
Kuwait	1995	Nerve injury	0.1129
Kuwait	2000	Nerve injury	0.1129
Kuwait	2005	Nerve injury	0.1129
Kuwait	2010	Nerve injury	0.1129
Kuwait	2017	Nerve injury	0.1129
Kuwait	1990	Injury to eyes	0.0165
Kuwait	1995	Injury to eyes	0.0165
Kuwait	2000	Injury to eyes	0.0165
Kuwait	2005	Injury to eyes	0.0165
Kuwait	2010	Injury to eyes	0.0165
Kuwait	2017	Injury to eyes	0.0165
Kuwait	1990	Amputation of lower limb, unilateral	0.053
Kuwait	1995	Amputation of lower limb, unilateral	0.0524
Kuwait	2000	Amputation of lower limb, unilateral	0.0463
Kuwait	2005	Amputation of lower limb, unilateral	0.0444
Kuwait	2010	Amputation of lower limb, unilateral	0.039
Kuwait	2017	Amputation of lower limb, unilateral	0.039
Kuwait	1990	Open wound(s)	0.011
Kuwait	1995	Open wound(s)	0.011
Kuwait	2000	Open wound(s)	0.011
Kuwait	2005	Open wound(s)	0.011
Kuwait	2010	Open wound(s)	0.011
Kuwait	2017	Open wound(s)	0.011
Kuwait	1990	Poisoning requiring urgent care	0.1628
Kuwait	1995	Poisoning requiring urgent care	0.1628
Kuwait	2000	Poisoning requiring urgent care	0.1628
Kuwait	2005	Poisoning requiring urgent care	0.1628
Kuwait	2010	Poisoning requiring urgent care	0.1628
Kuwait	2017	Poisoning requiring urgent care	0.1628
Kuwait	1990	Severe chest injury	0.0474
Kuwait	1995	Severe chest injury	0.0474
Kuwait	2000	Severe chest injury	0.0474
Kuwait	2005	Severe chest injury	0.0474
Kuwait	2010	Severe chest injury	0.0474
Kuwait	2017	Severe chest injury	0.0474
Kuwait	1990	Internal hemorrhage in abdomen and pelvis	0.1143
Kuwait	1995	Internal hemorrhage in abdomen and pelvis	0.1143
Kuwait	2000	Internal hemorrhage in abdomen and pelvis	0.1143
Kuwait	2005	Internal hemorrhage in abdomen and pelvis	0.1143
Kuwait	2010	Internal hemorrhage in abdomen and pelvis	0.1143
Kuwait	2017	Internal hemorrhage in abdomen and pelvis	0.1143
Kuwait	1990	Contusion in any part of the body	0.0075
Kuwait	1995	Contusion in any part of the body	0.0075
Kuwait	2000	Contusion in any part of the body	0.0075
Kuwait	2005	Contusion in any part of the body	0.0075
Kuwait	2010	Contusion in any part of the body	0.0075
Kuwait	2017	Contusion in any part of the body	0.0075
Kuwait	1990	Effect of different environmental factors	0.049
Kuwait	1995	Effect of different environmental factors	0.049
Kuwait	2000	Effect of different environmental factors	0.049
Kuwait	2005	Effect of different environmental factors	0.049
Kuwait	2010	Effect of different environmental factors	0.049
Kuwait	2017	Effect of different environmental factors	0.049
Kuwait	1990	Complications following therapeutic procedures	0.0506
Kuwait	1995	Complications following therapeutic procedures	0.0506
Kuwait	2000	Complications following therapeutic procedures	0.0506
Kuwait	2005	Complications following therapeutic procedures	0.0506
Kuwait	2010	Complications following therapeutic procedures	0.0506
Kuwait	2017	Complications following therapeutic procedures	0.0506
Kuwait	1990	Multiple fractures, dislocations, crashes, wounds, sprains, and strains	0.0936
Kuwait	1995	Multiple fractures, dislocations, crashes, wounds, sprains, and strains	0.0921
Kuwait	2000	Multiple fractures, dislocations, crashes, wounds, sprains, and strains	0.0764
Kuwait	2005	Multiple fractures, dislocations, crashes, wounds, sprains, and strains	0.0717
Kuwait	2010	Multiple fractures, dislocations, crashes, wounds, sprains, and strains	0.0577
Kuwait	2017	Multiple fractures, dislocations, crashes, wounds, sprains, and strains	0.0577
Kuwait	1990	Amputation of upper limb, unilateral	0.0472
Kuwait	1995	Amputation of upper limb, unilateral	0.0469
Kuwait	2000	Amputation of upper limb, unilateral	0.0433
Kuwait	2005	Amputation of upper limb, unilateral	0.0422
Kuwait	2010	Amputation of upper limb, unilateral	0.039
Kuwait	2017	Amputation of upper limb, unilateral	0.039
Kuwait	1990	Amputation of thumb	0.0114
Kuwait	1995	Amputation of thumb	0.0114
Kuwait	2000	Amputation of thumb	0.0114
Kuwait	2005	Amputation of thumb	0.0114
Kuwait	2010	Amputation of thumb	0.0114
Kuwait	2017	Amputation of thumb	0.0114
Kuwait	1990	Amputation of toe/toes	0.0059
Kuwait	1995	Amputation of toe/toes	0.0059
Kuwait	2000	Amputation of toe/toes	0.0059
Kuwait	2005	Amputation of toe/toes	0.0059
Kuwait	2010	Amputation of toe/toes	0.0059
Kuwait	2017	Amputation of toe/toes	0.0059
Kuwait	1990	Burns, <20% total burned surface area without lower airway burns	0.0163
Kuwait	1995	Burns, <20% total burned surface area without lower airway burns	0.0163
Kuwait	2000	Burns, <20% total burned surface area without lower airway burns	0.0163
Kuwait	2005	Burns, <20% total burned surface area without lower airway burns	0.0163
Kuwait	2010	Burns, <20% total burned surface area without lower airway burns	0.0163
Kuwait	2017	Burns, <20% total burned surface area without lower airway burns	0.0163
Kuwait	1990	Burns, >=20% total burned surface area or >= 10% burned surface area if head/neck or hands/wrist involved w/o lower airway burns	0.1681
Kuwait	1995	Burns, >=20% total burned surface area or >= 10% burned surface area if head/neck or hands/wrist involved w/o lower airway burns	0.1666
Kuwait	2000	Burns, >=20% total burned surface area or >= 10% burned surface area if head/neck or hands/wrist involved w/o lower airway burns	0.152
Kuwait	2005	Burns, >=20% total burned surface area or >= 10% burned surface area if head/neck or hands/wrist involved w/o lower airway burns	0.1476
Kuwait	2010	Burns, >=20% total burned surface area or >= 10% burned surface area if head/neck or hands/wrist involved w/o lower airway burns	0.1346
Kuwait	2017	Burns, >=20% total burned surface area or >= 10% burned surface area if head/neck or hands/wrist involved w/o lower airway burns	0.1346
Lebanon	1990	Amputation of lower limbs, bilateral	0.1968
Lebanon	1995	Amputation of lower limbs, bilateral	0.1747
Lebanon	2000	Amputation of lower limbs, bilateral	0.1496
Lebanon	2005	Amputation of lower limbs, bilateral	0.1265
Lebanon	2010	Amputation of lower limbs, bilateral	0.1232
Lebanon	2017	Amputation of lower limbs, bilateral	0.1232
Lebanon	1990	Lower airway burns	0.2252
Lebanon	1995	Lower airway burns	0.2252
Lebanon	2000	Lower airway burns	0.2252
Lebanon	2005	Lower airway burns	0.2252
Lebanon	2010	Lower airway burns	0.2252
Lebanon	2017	Lower airway burns	0.2252
Lebanon	1990	Dislocation of hip	0.0159
Lebanon	1995	Dislocation of hip	0.0159
Lebanon	2000	Dislocation of hip	0.0159
Lebanon	2005	Dislocation of hip	0.0159
Lebanon	2010	Dislocation of hip	0.0159
Lebanon	2017	Dislocation of hip	0.0159
Lebanon	1990	Dislocation of knee	0.1134
Lebanon	1995	Dislocation of knee	0.1134
Lebanon	2000	Dislocation of knee	0.1134
Lebanon	2005	Dislocation of knee	0.1134
Lebanon	2010	Dislocation of knee	0.1134
Lebanon	2017	Dislocation of knee	0.1134
Lebanon	1990	Dislocation of shoulder	0.062
Lebanon	1995	Dislocation of shoulder	0.062
Lebanon	2000	Dislocation of shoulder	0.062
Lebanon	2005	Dislocation of shoulder	0.062
Lebanon	2010	Dislocation of shoulder	0.062
Lebanon	2017	Dislocation of shoulder	0.062
Lebanon	1990	Muscle and tendon injuries, including sprains and strains lesser dislocations	0.0075
Lebanon	1995	Muscle and tendon injuries, including sprains and strains lesser dislocations	0.0075
Lebanon	2000	Muscle and tendon injuries, including sprains and strains lesser dislocations	0.0075
Lebanon	2005	Muscle and tendon injuries, including sprains and strains lesser dislocations	0.0075
Lebanon	2010	Muscle and tendon injuries, including sprains and strains lesser dislocations	0.0075
Lebanon	2017	Muscle and tendon injuries, including sprains and strains lesser dislocations	0.0075
Lebanon	1990	Fracture of clavicle, scapula, or humerus	0.0349
Lebanon	1995	Fracture of clavicle, scapula, or humerus	0.0349
Lebanon	2000	Fracture of clavicle, scapula, or humerus	0.0349
Lebanon	2005	Fracture of clavicle, scapula, or humerus	0.0349
Lebanon	2010	Fracture of clavicle, scapula, or humerus	0.0349
Lebanon	2017	Fracture of clavicle, scapula, or humerus	0.0349
Lebanon	1990	Fracture of face bones	0.0669
Lebanon	1995	Fracture of face bones	0.0669
Lebanon	2000	Fracture of face bones	0.0669
Lebanon	2005	Fracture of face bones	0.0669
Lebanon	2010	Fracture of face bones	0.0669
Lebanon	2017	Fracture of face bones	0.0669
Lebanon	1990	Fracture of foot bones except ankle	0.0264
Lebanon	1995	Fracture of foot bones except ankle	0.0264
Lebanon	2000	Fracture of foot bones except ankle	0.0264
Lebanon	2005	Fracture of foot bones except ankle	0.0264
Lebanon	2010	Fracture of foot bones except ankle	0.0264
Lebanon	2017	Fracture of foot bones except ankle	0.0264
Lebanon	1990	Fracture of hand (wrist and other distal part of hand)	0.0138
Lebanon	1995	Fracture of hand (wrist and other distal part of hand)	0.0138
Lebanon	2000	Fracture of hand (wrist and other distal part of hand)	0.0138
Lebanon	2005	Fracture of hand (wrist and other distal part of hand)	0.0138
Lebanon	2010	Fracture of hand (wrist and other distal part of hand)	0.0138
Lebanon	2017	Fracture of hand (wrist and other distal part of hand)	0.0138
Lebanon	1990	Fracture of hip	0.1549
Lebanon	1995	Fracture of hip	0.1257
Lebanon	2000	Fracture of hip	0.0925
Lebanon	2005	Fracture of hip	0.062
Lebanon	2010	Fracture of hip	0.0577
Lebanon	2017	Fracture of hip	0.0577
Lebanon	1990	Amputation of upper limbs, bilateral	0.188
Lebanon	1995	Amputation of upper limbs, bilateral	0.1579
Lebanon	2000	Amputation of upper limbs, bilateral	0.1236
Lebanon	2005	Amputation of upper limbs, bilateral	0.0921
Lebanon	2010	Amputation of upper limbs, bilateral	0.0876
Lebanon	2017	Amputation of upper limbs, bilateral	0.0876
Lebanon	1990	Fracture of patella, tibia or fibula, or ankle	0.0554
Lebanon	1995	Fracture of patella, tibia or fibula, or ankle	0.0554
Lebanon	2000	Fracture of patella, tibia or fibula, or ankle	0.0554
Lebanon	2005	Fracture of patella, tibia or fibula, or ankle	0.0554
Lebanon	2010	Fracture of patella, tibia or fibula, or ankle	0.0554
Lebanon	2017	Fracture of patella, tibia or fibula, or ankle	0.0554
Lebanon	1990	Fracture of pelvis	0.1823
Lebanon	1995	Fracture of pelvis	0.1823
Lebanon	2000	Fracture of pelvis	0.1823
Lebanon	2005	Fracture of pelvis	0.1823
Lebanon	2010	Fracture of pelvis	0.1823
Lebanon	2017	Fracture of pelvis	0.1823
Lebanon	1990	Fracture of radius and/or ulna	0.0435
Lebanon	1995	Fracture of radius and/or ulna	0.0435
Lebanon	2000	Fracture of radius and/or ulna	0.0435
Lebanon	2005	Fracture of radius and/or ulna	0.0435
Lebanon	2010	Fracture of radius and/or ulna	0.0435
Lebanon	2017	Fracture of radius and/or ulna	0.0435
Lebanon	1990	Fracture of skull	0.0714
Lebanon	1995	Fracture of skull	0.0714
Lebanon	2000	Fracture of skull	0.0714
Lebanon	2005	Fracture of skull	0.0714
Lebanon	2010	Fracture of skull	0.0714
Lebanon	2017	Fracture of skull	0.0714
Lebanon	1990	Fracture of sternum and/or fracture of one or more ribs	0.1027
Lebanon	1995	Fracture of sternum and/or fracture of one or more ribs	0.1027
Lebanon	2000	Fracture of sternum and/or fracture of one or more ribs	0.1027
Lebanon	2005	Fracture of sternum and/or fracture of one or more ribs	0.1027
Lebanon	2010	Fracture of sternum and/or fracture of one or more ribs	0.1027
Lebanon	2017	Fracture of sternum and/or fracture of one or more ribs	0.1027
Lebanon	1990	Fracture of vertebral column	0.1106
Lebanon	1995	Fracture of vertebral column	0.1106
Lebanon	2000	Fracture of vertebral column	0.1106
Lebanon	2005	Fracture of vertebral column	0.1106
Lebanon	2010	Fracture of vertebral column	0.1106
Lebanon	2017	Fracture of vertebral column	0.1106
Lebanon	1990	Fracture of femur, other than femoral neck	0.042
Lebanon	1995	Fracture of femur, other than femoral neck	0.042
Lebanon	2000	Fracture of femur, other than femoral neck	0.042
Lebanon	2005	Fracture of femur, other than femoral neck	0.042
Lebanon	2010	Fracture of femur, other than femoral neck	0.042
Lebanon	2017	Fracture of femur, other than femoral neck	0.042
Lebanon	1990	Minor TBI	0.1319
Lebanon	1995	Minor TBI	0.1319
Lebanon	2000	Minor TBI	0.1319
Lebanon	2005	Minor TBI	0.1319
Lebanon	2010	Minor TBI	0.1319
Lebanon	2017	Minor TBI	0.1319
Lebanon	1990	Moderate TBI	0.1637
Lebanon	1995	Moderate TBI	0.1637
Lebanon	2000	Moderate TBI	0.1637
Lebanon	2005	Moderate TBI	0.1637
Lebanon	2010	Moderate TBI	0.1637
Lebanon	2017	Moderate TBI	0.1637
Lebanon	1990	Amputation of fingers (excluding thumb)	0.0047
Lebanon	1995	Amputation of fingers (excluding thumb)	0.0047
Lebanon	2000	Amputation of fingers (excluding thumb)	0.0047
Lebanon	2005	Amputation of fingers (excluding thumb)	0.0047
Lebanon	2010	Amputation of fingers (excluding thumb)	0.0047
Lebanon	2017	Amputation of fingers (excluding thumb)	0.0047
Lebanon	1990	Spinal cord lesion at neck level	0.4454
Lebanon	1995	Spinal cord lesion at neck level	0.4353
Lebanon	2000	Spinal cord lesion at neck level	0.4239
Lebanon	2005	Spinal cord lesion at neck level	0.4133
Lebanon	2010	Spinal cord lesion at neck level	0.4118
Lebanon	2017	Spinal cord lesion at neck level	0.4118
Lebanon	1990	Spinal cord lesion below neck level	0.2486
Lebanon	1995	Spinal cord lesion below neck level	0.2273
Lebanon	2000	Spinal cord lesion below neck level	0.2031
Lebanon	2005	Spinal cord lesion below neck level	0.1808
Lebanon	2010	Spinal cord lesion below neck level	0.1777
Lebanon	2017	Spinal cord lesion below neck level	0.1777
Lebanon	1990	Drowning and nonfatal submersion	0.2471
Lebanon	1995	Drowning and nonfatal submersion	0.2471
Lebanon	2000	Drowning and nonfatal submersion	0.2471
Lebanon	2005	Drowning and nonfatal submersion	0.2471
Lebanon	2010	Drowning and nonfatal submersion	0.2471
Lebanon	2017	Drowning and nonfatal submersion	0.2471

Lebanon	1990	Asphyxiation	0.2471
Lebanon	1995	Asphyxiation	0.2471
Lebanon	2000	Asphyxiation	0.2471
Lebanon	2005	Asphyxiation	0.2471
Lebanon	2010	Asphyxiation	0.2471
Lebanon	2017	Asphyxiation	0.2471
Lebanon	1990	Crush injury	0.1325
Lebanon	1995	Crush injury	0.1325
Lebanon	2000	Crush injury	0.1325
Lebanon	2005	Crush injury	0.1325
Lebanon	2010	Crush injury	0.1325
Lebanon	2017	Crush injury	0.1325
Lebanon	1990	Nerve injury	0.1129
Lebanon	1995	Nerve injury	0.1129
Lebanon	2000	Nerve injury	0.1129
Lebanon	2005	Nerve injury	0.1129
Lebanon	2010	Nerve injury	0.1129
Lebanon	2017	Nerve injury	0.1129
Lebanon	1990	Injury to eyes	0.0165
Lebanon	1995	Injury to eyes	0.0165
Lebanon	2000	Injury to eyes	0.0165
Lebanon	2005	Injury to eyes	0.0165
Lebanon	2010	Injury to eyes	0.0165
Lebanon	2017	Injury to eyes	0.0165
Lebanon	1990	Amputation of lower limb, unilateral	0.0769
Lebanon	1995	Amputation of lower limb, unilateral	0.0655
Lebanon	2000	Amputation of lower limb, unilateral	0.0525
Lebanon	2005	Amputation of lower limb, unilateral	0.0406
Lebanon	2010	Amputation of lower limb, unilateral	0.039
Lebanon	2017	Amputation of lower limb, unilateral	0.039
Lebanon	1990	Open wound(s)	0.011
Lebanon	1995	Open wound(s)	0.011
Lebanon	2000	Open wound(s)	0.011
Lebanon	2005	Open wound(s)	0.011
Lebanon	2010	Open wound(s)	0.011
Lebanon	2017	Open wound(s)	0.011
Lebanon	1990	Poisoning requiring urgent care	0.1628
Lebanon	1995	Poisoning requiring urgent care	0.1628
Lebanon	2000	Poisoning requiring urgent care	0.1628
Lebanon	2005	Poisoning requiring urgent care	0.1628
Lebanon	2010	Poisoning requiring urgent care	0.1628
Lebanon	2017	Poisoning requiring urgent care	0.1628
Lebanon	1990	Severe chest injury	0.0474
Lebanon	1995	Severe chest injury	0.0474
Lebanon	2000	Severe chest injury	0.0474
Lebanon	2005	Severe chest injury	0.0474
Lebanon	2010	Severe chest injury	0.0474
Lebanon	2017	Severe chest injury	0.0474
Lebanon	1990	Internal hemorrhage in abdomen and pelvis	0.1143
Lebanon	1995	Internal hemorrhage in abdomen and pelvis	0.1143
Lebanon	2000	Internal hemorrhage in abdomen and pelvis	0.1143
Lebanon	2005	Internal hemorrhage in abdomen and pelvis	0.1143
Lebanon	2010	Internal hemorrhage in abdomen and pelvis	0.1143
Lebanon	2017	Internal hemorrhage in abdomen and pelvis	0.1143
Lebanon	1990	Contusion in any part of the body	0.0075
Lebanon	1995	Contusion in any part of the body	0.0075
Lebanon	2000	Contusion in any part of the body	0.0075
Lebanon	2005	Contusion in any part of the body	0.0075
Lebanon	2010	Contusion in any part of the body	0.0075
Lebanon	2017	Contusion in any part of the body	0.0075
Lebanon	1990	Effect of different environmental factors	0.049
Lebanon	1995	Effect of different environmental factors	0.049
Lebanon	2000	Effect of different environmental factors	0.049
Lebanon	2005	Effect of different environmental factors	0.049
Lebanon	2010	Effect of different environmental factors	0.049
Lebanon	2017	Effect of different environmental factors	0.049
Lebanon	1990	Complications following therapeutic procedures	0.0506
Lebanon	1995	Complications following therapeutic procedures	0.0506
Lebanon	2000	Complications following therapeutic procedures	0.0506
Lebanon	2005	Complications following therapeutic procedures	0.0506
Lebanon	2010	Complications following therapeutic procedures	0.0506
Lebanon	2017	Complications following therapeutic procedures	0.0506
Lebanon	1990	Multiple fractures, dislocations, crashes, wounds, sprains, and strains	0.1549
Lebanon	1995	Multiple fractures, dislocations, crashes, wounds, sprains, and strains	0.1257
Lebanon	2000	Multiple fractures, dislocations, crashes, wounds, sprains, and strains	0.0925
Lebanon	2005	Multiple fractures, dislocations, crashes, wounds, sprains, and strains	0.062
Lebanon	2010	Multiple fractures, dislocations, crashes, wounds, sprains, and strains	0.0577
Lebanon	2017	Multiple fractures, dislocations, crashes, wounds, sprains, and strains	0.0577
Lebanon	1990	Amputation of upper limb, unilateral	0.0613
Lebanon	1995	Amputation of upper limb, unilateral	0.0546
Lebanon	2000	Amputation of upper limb, unilateral	0.047
Lebanon	2005	Amputation of upper limb, unilateral	0.04
Lebanon	2010	Amputation of upper limb, unilateral	0.039
Lebanon	2017	Amputation of upper limb, unilateral	0.039
Lebanon	1990	Amputation of thumb	0.0114
Lebanon	1995	Amputation of thumb	0.0114
Lebanon	2000	Amputation of thumb	0.0114
Lebanon	2005	Amputation of thumb	0.0114
Lebanon	2010	Amputation of thumb	0.0114
Lebanon	2017	Amputation of thumb	0.0114
Lebanon	1990	Amputation of toe/toes	0.0059
Lebanon	1995	Amputation of toe/toes	0.0059
Lebanon	2000	Amputation of toe/toes	0.0059
Lebanon	2005	Amputation of toe/toes	0.0059
Lebanon	2010	Amputation of toe/toes	0.0059
Lebanon	2017	Amputation of toe/toes	0.0059
Lebanon	1990	Burns, <20% total burned surface area without lower airway burns	0.0163
Lebanon	1995	Burns, <20% total burned surface area without lower airway burns	0.0163
Lebanon	2000	Burns, <20% total burned surface area without lower airway burns	0.0163
Lebanon	2005	Burns, <20% total burned surface area without lower airway burns	0.0163
Lebanon	2010	Burns, <20% total burned surface area without lower airway burns	0.0163
Lebanon	2017	Burns, <20% total burned surface area without lower airway burns	0.0163
Lebanon	1990	Burns, >=20% total burned surface area or >= 10% burned surface area if head/neck or hands/wrist involved w/o lower airway burns	0.2251
Lebanon	1995	Burns, >=20% total burned surface area or >= 10% burned surface area if head/neck or hands/wrist involved w/o lower airway burns	0.1979
Lebanon	2000	Burns, >=20% total burned surface area or >= 10% burned surface area if head/neck or hands/wrist involved w/o lower airway burns	0.167
Lebanon	2005	Burns, >=20% total burned surface area or >= 10% burned surface area if head/neck or hands/wrist involved w/o lower airway burns	0.1386
Lebanon	2010	Burns, >=20% total burned surface area or >= 10% burned surface area if head/neck or hands/wrist involved w/o lower airway burns	0.1346
Lebanon	2017	Burns, >=20% total burned surface area or >= 10% burned surface area if head/neck or hands/wrist involved w/o lower airway burns	0.1346
Libya	1990	Amputation of lower limbs, bilateral	0.2045
Libya	1995	Amputation of lower limbs, bilateral	0.1917
Libya	2000	Amputation of lower limbs, bilateral	0.1809
Libya	2005	Amputation of lower limbs, bilateral	0.1648
Libya	2010	Amputation of lower limbs, bilateral	0.1406
Libya	2017	Amputation of lower limbs, bilateral	0.1376
Libya	1990	Lower airway burns	0.2252
Libya	1995	Lower airway burns	0.2252
Libya	2000	Lower airway burns	0.2252
Libya	2005	Lower airway burns	0.2252
Libya	2010	Lower airway burns	0.2252
Libya	2017	Lower airway burns	0.2252
Libya	1990	Dislocation of hip	0.0159
Libya	1995	Dislocation of hip	0.0159
Libya	2000	Dislocation of hip	0.0159
Libya	2005	Dislocation of hip	0.0159
Libya	2010	Dislocation of hip	0.0159
Libya	2017	Dislocation of hip	0.0159
Libya	1990	Dislocation of knee	0.1134
Libya	1995	Dislocation of knee	0.1134
Libya	2000	Dislocation of knee	0.1134
Libya	2005	Dislocation of knee	0.1134
Libya	2010	Dislocation of knee	0.1134
Libya	2017	Dislocation of knee	0.1134
Libya	1990	Dislocation of shoulder	0.062
Libya	1995	Dislocation of shoulder	0.062
Libya	2000	Dislocation of shoulder	0.062
Libya	2005	Dislocation of shoulder	0.062
Libya	2010	Dislocation of shoulder	0.062
Libya	2017	Dislocation of shoulder	0.062
Libya	1990	Muscle and tendon injuries, including sprains and strains lesser dislocations	0.0075
Libya	1995	Muscle and tendon injuries, including sprains and strains lesser dislocations	0.0075
Libya	2000	Muscle and tendon injuries, including sprains and strains lesser dislocations	0.0075
Libya	2005	Muscle and tendon injuries, including sprains and strains lesser dislocations	0.0075
Libya	2010	Muscle and tendon injuries, including sprains and strains lesser dislocations	0.0075
Libya	2017	Muscle and tendon injuries, including sprains and strains lesser dislocations	0.0075
Libya	1990	Fracture of clavicle, scapula, or humerus	0.0349
Libya	1995	Fracture of clavicle, scapula, or humerus	0.0349
Libya	2000	Fracture of clavicle, scapula, or humerus	0.0349
Libya	2005	Fracture of clavicle, scapula, or humerus	0.0349
Libya	2010	Fracture of clavicle, scapula, or humerus	0.0349
Libya	2017	Fracture of clavicle, scapula, or humerus	0.0349
Libya	1990	Fracture of face bones	0.0669
Libya	1995	Fracture of face bones	0.0669
Libya	2000	Fracture of face bones	0.0669
Libya	2005	Fracture of face bones	0.0669
Libya	2010	Fracture of face bones	0.0669
Libya	2017	Fracture of face bones	0.0669
Libya	1990	Fracture of foot bones except ankle	0.0264
Libya	1995	Fracture of foot bones except ankle	0.0264
Libya	2000	Fracture of foot bones except ankle	0.0264
Libya	2005	Fracture of foot bones except ankle	0.0264
Libya	2010	Fracture of foot bones except ankle	0.0264
Libya	2017	Fracture of foot bones except ankle	0.0264
Libya	1990	Fracture of hand (wrist and other distal part of hand)	0.0138
Libya	1995	Fracture of hand (wrist and other distal part of hand)	0.0138
Libya	2000	Fracture of hand (wrist and other distal part of hand)	0.0138
Libya	2005	Fracture of hand (wrist and other distal part of hand)	0.0138
Libya	2010	Fracture of hand (wrist and other distal part of hand)	0.0138
Libya	2017	Fracture of hand (wrist and other distal part of hand)	0.0138
Libya	1990	Fracture of hip	0.1652
Libya	1995	Fracture of hip	0.1482
Libya	2000	Fracture of hip	0.134
Libya	2005	Fracture of hip	0.1126
Libya	2010	Fracture of hip	0.0807
Libya	2017	Fracture of hip	0.0766
Libya	1990	Amputation of upper limbs, bilateral	0.1987
Libya	1995	Amputation of upper limbs, bilateral	0.1811
Libya	2000	Amputation of upper limbs, bilateral	0.1664
Libya	2005	Amputation of upper limbs, bilateral	0.1444
Libya	2010	Amputation of upper limbs, bilateral	0.1114
Libya	2017	Amputation of upper limbs, bilateral	0.1072
Libya	1990	Fracture of patella, tibia or fibula, or ankle	0.0554
Libya	1995	Fracture of patella, tibia or fibula, or ankle	0.0554
Libya	2000	Fracture of patella, tibia or fibula, or ankle	0.0554
Libya	2005	Fracture of patella, tibia or fibula, or ankle	0.0554
Libya	2010	Fracture of patella, tibia or fibula, or ankle	0.0554
Libya	2017	Fracture of patella, tibia or fibula, or ankle	0.0554
Libya	1990	Fracture of pelvis	0.1823
Libya	1995	Fracture of pelvis	0.1823
Libya	2000	Fracture of pelvis	0.1823
Libya	2005	Fracture of pelvis	0.1823
Libya	2010	Fracture of pelvis	0.1823
Libya	2017	Fracture of pelvis	0.1823
Libya	1990	Fracture of radius and/or ulna	0.0435
Libya	1995	Fracture of radius and/or ulna	0.0435
Libya	2000	Fracture of radius and/or ulna	0.0435
Libya	2005	Fracture of radius and/or ulna	0.0435
Libya	2010	Fracture of radius and/or ulna	0.0435
Libya	2017	Fracture of radius and/or ulna	0.0435
Libya	1990	Fracture of skull	0.0714
Libya	1995	Fracture of skull	0.0714
Libya	2000	Fracture of skull	0.0714
Libya	2005	Fracture of skull	0.0714
Libya	2010	Fracture of skull	0.0714
Libya	2017	Fracture of skull	0.0714
Libya	1990	Fracture of sternum and/or fracture of one or more ribs	0.1027
Libya	1995	Fracture of sternum and/or fracture of one or more ribs	0.1027
Libya	2000	Fracture of sternum and/or fracture of one or more ribs	0.1027
Libya	2005	Fracture of sternum and/or fracture of one or more ribs	0.1027
Libya	2010	Fracture of sternum and/or fracture of one or more ribs	0.1027
Libya	2017	Fracture of sternum and/or fracture of one or more ribs	0.1027
Libya	1990	Fracture of vertebral column	0.1106
Libya	1995	Fracture of vertebral column	0.1106
Libya	2000	Fracture of vertebral column	0.1106
Libya	2005	Fracture of vertebral column	0.1106
Libya	2010	Fracture of vertebral column	0.1106
Libya	2017	Fracture of vertebral column	0.1106
Libya	1990	Fracture of femur, other than femoral neck	0.042
Libya	1995	Fracture of femur, other than femoral neck	0.042
Libya	2000	Fracture of femur, other than femoral neck	0.042
Libya	2005	Fracture of femur, other than femoral neck	0.042
Libya	2010	Fracture of femur, other than femoral neck	0.042
Libya	2017	Fracture of femur, other than femoral neck	0.042
Libya	1990	Minor TBI	0.1319
Libya	1995	Minor TBI	0.1319
Libya	2000	Minor TBI	0.1319
Libya	2005	Minor TBI	0.1319
Libya	2010	Minor TBI	0.1319
Libya	2017	Minor TBI	0.1319
Libya	1990	Moderate TBI	0.1637
Libya	1995	Moderate TBI	0.1637
Libya	2000	Moderate TBI	0.1637
Libya	2005	Moderate TBI	0.1637
Libya	2010	Moderate TBI	0.1637
Libya	2017	Moderate TBI	0.1637
Libya	1990	Amputation of fingers (excluding thumb)	0.0047
Libya	1995	Amputation of fingers (excluding thumb)	0.0047
Libya	2000	Amputation of fingers (excluding thumb)	0.0047
Libya	2005	Amputation of fingers (excluding thumb)	0.0047
Libya	2010	Amputation of fingers (excluding thumb)	0.0047
Libya	2017	Amputation of fingers (excluding thumb)	0.0047
Libya	1990	Spinal cord lesion at neck level	0.449
Libya	1995	Spinal cord lesion at neck level	0.4431
Libya	2000	Spinal cord lesion at neck level	0.4382
Libya	2005	Spinal cord lesion at neck level	0.4308
Libya	2010	Spinal cord lesion at neck level	0.4198
Libya	2017	Spinal cord lesion at neck level	0.4184
Libya	1990	Spinal cord lesion below neck level	0.2561
Libya	1995	Spinal cord lesion below neck level	0.2437
Libya	2000	Spinal cord lesion below neck level	0.2333
Libya	2005	Spinal cord lesion below neck level	0.2178
Libya	2010	Spinal cord lesion below neck level	0.1945
Libya	2017	Spinal cord lesion below neck level	0.1915
Libya	1990	Drowning and nonfatal submersion	0.2471
Libya	1995	Drowning and nonfatal submersion	0.2471
Libya	2000	Drowning and nonfatal submersion	0.2471
Libya	2005	Drowning and nonfatal submersion	0.2471
Libya	2010	Drowning and nonfatal submersion	0.2471
Libya	2017	Drowning and nonfatal submersion	0.2471
Libya	1990	Asphyxiation	0.2471
Libya	1995	Asphyxiation	0.2471
Libya	2000	Asphyxiation	0.2471
Libya	2005	Asphyxiation	0.2471
Libya	2010	Asphyxiation	0.2471
Libya	2017	Asphyxiation	0.2471
Libya	1990	Crush injury	0.1325
Libya	1995	Crush injury	0.1325
Libya	2000	Crush injury	0.1325
Libya	2005	Crush injury	0.1325
Libya	2010	Crush injury	0.1325
Libya	2017	Crush injury	0.1325
Libya	1990	Nerve injury	0.1129
Libya	1995	Nerve injury	0.1129
Libya	2000	Nerve injury	0.1129
Libya	2005	Nerve injury	0.1129
Libya	2010	Nerve injury	0.1129
Libya	2017	Nerve injury	0.1129
Libya	1990	Injury to eyes	0.0165
Libya	1995	Injury to eyes	0.0165
Libya	2000	Injury to eyes	0.0165
Libya	2005	Injury to eyes	0.0165
Libya	2010	Injury to eyes	0.0165
Libya	2017	Injury to eyes	0.0165
Libya	1990	Amputation of lower limb, unilateral	0.0809
Libya	1995	Amputation of lower limb, unilateral	0.0742
Libya	2000	Amputation of lower limb, unilateral	0.0687
Libya	2005	Amputation of lower limb, unilateral	0.0604
Libya	2010	Amputation of lower limb, unilateral	0.0479
Libya	2017	Amputation of lower limb, unilateral	0.0463
Libya	1990	Open wound(s)	0.011
Libya	1995	Open wound(s)	0.011
Libya	2000	Open wound(s)	0.011
Libya	2005	Open wound(s)	0.011
Libya	2010	Open wound(s)	0.011
Libya	2017	Open wound(s)	0.011
Libya	1990	Poisoning requiring urgent care	0.1628
Libya	1995	Poisoning requiring urgent care	0.1628
Libya	2000	Poisoning requiring urgent care	0.1628
Libya	2005	Poisoning requiring urgent care	0.1628
Libya	2010	Poisoning requiring urgent care	0.1628
Libya	2017	Poisoning requiring urgent care	0.1628
Libya	1990	Severe chest injury	0.0474
Libya	1995	Severe chest injury	0.0474
Libya	2000	Severe chest injury	0.0474
Libya	2005	Severe chest injury	0.0474
Libya	2010	Severe chest injury	0.0474
Libya	2017	Severe chest injury	0.0474
Libya	1990	Internal hemorrhage in abdomen and pelvis	0.1143
Libya	1995	Internal hemorrhage in abdomen and pelvis	0.1143
Libya	2000	Internal hemorrhage in abdomen and pelvis	0.1143
Libya	2005	Internal hemorrhage in abdomen and pelvis	0.1143
Libya	2010	Internal hemorrhage in abdomen and pelvis	0.1143
Libya	2017	Internal hemorrhage in abdomen and pelvis	0.1143
Libya	1990	Contusion in any part of the body	0.0075
Libya	1995	Contusion in any part of the body	0.0075
Libya	2000	Contusion in any part of the body	0.0075
Libya	2005	Contusion in any part of the body	0.0075
Libya	2010	Contusion in any part of the body	0.0075
Libya	2017	Contusion in any part of the body	0.0075
Libya	1990	Effect of different environmental factors	0.049
Libya	1995	Effect of different environmental factors	0.049
Libya	2000	Effect of different environmental factors	0.049
Libya	2005	Effect of different environmental factors	0.049
Libya	2010	Effect of different environmental factors	0.049
Libya	2017	Effect of different environmental factors	0.049
Libya	1990	Complications following therapeutic procedures	0.0506
Libya	1995	Complications following therapeutic procedures	0.0506
Libya	2000	Complications following therapeutic procedures	0.0506
Libya	2005	Complications following therapeutic procedures	0.0506
Libya	2010	Complications following therapeutic procedures	0.0506
Libya	2017	Complications following therapeutic procedures	0.0506
Libya	1990	Multiple fractures, dislocations, crashes, wounds, sprains, and strains	0.1652
Libya	1995	Multiple fractures, dislocations, crashes, wounds, sprains, and strains	0.1482
Libya	2000	Multiple fractures, dislocations, crashes, wounds, sprains, and strains	0.134
Libya	2005	Multiple fractures, dislocations, crashes, wounds, sprains, and strains	0.1126
Libya	2010	Multiple fractures, dislocations, crashes, wounds, sprains, and strains	0.0807
Libya	2017	Multiple fractures, dislocations, crashes, wounds, sprains, and strains	0.0766
Libya	1990	Amputation of upper limb, unilateral	0.0636
Libya	1995	Amputation of upper limb, unilateral	0.0597
Libya	2000	Amputation of upper limb, unilateral	0.0565
Libya	2005	Amputation of upper limb, unilateral	0.0516
Libya	2010	Amputation of upper limb, unilateral	0.0443
Libya	2017	Amputation of upper limb, unilateral	0.0433
Libya	1990	Amputation of thumb	0.0114
Libya	1995	Amputation of thumb	0.0114
Libya	2000	Amputation of thumb	0.0114
Libya	2005	Amputation of thumb	0.0114
Libya	2010	Amputation of thumb	0.0114
Libya	2017	Amputation of thumb	0.0114
Libya	1990	Amputation of toe/toes	0.0059
Libya	1995	Amputation of toe/toes	0.0059
Libya	2000	Amputation of toe/toes	0.0059
Libya	2005	Amputation of toe/toes	0.0059
Libya	2010	Amputation of toe/toes	0.0059
Libya	2017	Amputation of toe/toes	0.0059
Libya	1990	Burns, <20% total burned surface area without lower airway burns	0.0163
Libya	1995	Burns, <20% total burned surface area without lower airway burns	0.0163
Libya	2000	Burns, <20% total burned surface area without lower airway burns	0.0163
Libya	2005	Burns, <20% total burned surface area without lower airway burns	0.0163
Libya	2010	Burns, <20% total burned surface area without lower airway burns	0.0163
Libya	2017	Burns, <20% total burned surface area without lower airway burns	0.0163
Libya	1990	Burns, >=20% total burned surface area or >= 10% burned surface area if head/neck or hands/wrist involved w/o lower airway burns	0.2347
Libya	1995	Burns, >=20% total burned surface area or >= 10% burned surface area if head/neck or hands/wrist involved w/o lower airway burns	0.2189
Libya	2000	Burns, >=20% total burned surface area or >= 10% burned surface area if head/neck or hands/wrist involved w/o lower airway burns	0.2056
Libya	2005	Burns, >=20% total burned surface area or >= 10% burned surface area if head/neck or hands/wrist involved w/o lower airway burns	0.1858
Libya	2010	Burns, >=20% total burned surface area or >= 10% burned surface area if head/neck or hands/wrist involved w/o lower airway burns	0.156
Libya	2017	Burns, >=20% total burned surface area or >= 10% burned surface area if head/neck or hands/wrist involved w/o lower airway burns	0.1522
Morocco	1990	Amputation of lower limbs, bilateral	0.2497
Morocco	1995	Amputation of lower limbs, bilateral	0.2388
Morocco	2000	Amputation of lower limbs, bilateral	0.2258
Morocco	2005	Amputation of lower limbs, bilateral	0.2153
Morocco	2010	Amputation of lower limbs, bilateral	0.2008
Morocco	2017	Amputation of lower limbs, bilateral	0.1795
Morocco	1990	Lower airway burns	0.2252
Morocco	1995	Lower airway burns	0.2252
Morocco	2000	Lower airway burns	0.2252
Morocco	2005	Lower airway burns	0.2252
Morocco	2010	Lower airway burns	0.2252
Morocco	2017	Lower airway burns	0.2252
Morocco	1990	Dislocation of hip	0.0159
Morocco	1995	Dislocation of hip	0.0159
Morocco	2000	Dislocation of hip	0.0159
Morocco	2005	Dislocation of hip	0.0159
Morocco	2010	Dislocation of hip	0.0159
Morocco	2017	Dislocation of hip	0.0159
Morocco	1990	Dislocation of knee	0.1134
Morocco	1995	Dislocation of knee	0.1134
Morocco	2000	Dislocation of knee	0.1134
Morocco	2005	Dislocation of knee	0.1134
Morocco	2010	Dislocation of knee	0.1134
Morocco	2017	Dislocation of knee	0.1134
Morocco	1990	Dislocation of shoulder	0.062
Morocco	1995	Dislocation of shoulder	0.062
Morocco	2000	Dislocation of shoulder	0.062
Morocco	2005	Dislocation of shoulder	0.062
Morocco	2010	Dislocation of shoulder	0.062
Morocco	2017	Dislocation of shoulder	0.062
Morocco	1990	Muscle and tendon injuries, including sprains and strains lesser dislocations	0.0075
Morocco	1995	Muscle and tendon injuries, including sprains and strains lesser dislocations	0.0075
Morocco	2000	Muscle and tendon injuries, including sprains and strains lesser dislocations	0.0075
Morocco	2005	Muscle and tendon injuries, including sprains and strains lesser dislocations	0.0075
Morocco	2010	Muscle and tendon injuries, including sprains and strains lesser dislocations	0.0075
Morocco	2017	Muscle and tendon injuries, including sprains and strains lesser dislocations	0.0075
Morocco	1990	Fracture of clavicle, scapula, or humerus	0.0349
Morocco	1995	Fracture of clavicle, scapula, or humerus	0.0349
Morocco	2000	Fracture of clavicle, scapula, or humerus	0.0349
Morocco	2005	Fracture of clavicle, scapula, or humerus	0.0349
Morocco	2010	Fracture of clavicle, scapula, or humerus	0.0349
Morocco	2017	Fracture of clavicle, scapula, or humerus	0.0349
Morocco	1990	Fracture of face bones	0.0669
Morocco	1995	Fracture of face bones	0.0669
Morocco	2000	Fracture of face bones	0.0669
Morocco	2005	Fracture of face bones	0.0669
Morocco	2010	Fracture of face bones	0.0669
Morocco	2017	Fracture of face bones	0.0669
Morocco	1990	Fracture of foot bones except ankle	0.0264
Morocco	1995	Fracture of foot bones except ankle	0.0264
Morocco	2000	Fracture of foot bones except ankle	0.0264
Morocco	2005	Fracture of foot bones except ankle	0.0264
Morocco	2010	Fracture of foot bones except ankle	0.0264
Morocco	2017	Fracture of foot bones except ankle	0.0264
Morocco	1990	Fracture of hand (wrist and other distal part of hand)	0.0138
Morocco	1995	Fracture of hand (wrist and other distal part of hand)	0.0138
Morocco	2000	Fracture of hand (wrist and other distal part of hand)	0.0138
Morocco	2005	Fracture of hand (wrist and other distal part of hand)	0.0138
Morocco	2010	Fracture of hand (wrist and other distal part of hand)	0.0138
Morocco	2017	Fracture of hand (wrist and other distal part of hand)	0.0138
Morocco	1990	Fracture of hip	0.2249
Morocco	1995	Fracture of hip	0.2105
Morocco	2000	Fracture of hip	0.1934
Morocco	2005	Fracture of hip	0.1795
Morocco	2010	Fracture of hip	0.1602

Morocco	2017	Fracture of hip	0.1321
Morocco	1990	Amputation of upper limbs, bilateral	0.2603
Morocco	1995	Amputation of upper limbs, bilateral	0.2454
Morocco	2000	Amputation of upper limbs, bilateral	0.2277
Morocco	2005	Amputation of upper limbs, bilateral	0.2134
Morocco	2010	Amputation of upper limbs, bilateral	0.1935
Morocco	2017	Amputation of upper limbs, bilateral	0.1644
Morocco	1990	Fracture of patella, tibia or fibula, or ankle	0.0554
Morocco	1995	Fracture of patella, tibia or fibula, or ankle	0.0554
Morocco	2000	Fracture of patella, tibia or fibula, or ankle	0.0554
Morocco	2005	Fracture of patella, tibia or fibula, or ankle	0.0554
Morocco	2010	Fracture of patella, tibia or fibula, or ankle	0.0554
Morocco	2017	Fracture of patella, tibia or fibula, or ankle	0.0554
Morocco	1990	Fracture of pelvis	0.1823
Morocco	1995	Fracture of pelvis	0.1823
Morocco	2000	Fracture of pelvis	0.1823
Morocco	2005	Fracture of pelvis	0.1823
Morocco	2010	Fracture of pelvis	0.1823
Morocco	2017	Fracture of pelvis	0.1823
Morocco	1990	Fracture of radius and/or ulna	0.0435
Morocco	1995	Fracture of radius and/or ulna	0.0435
Morocco	2000	Fracture of radius and/or ulna	0.0435
Morocco	2005	Fracture of radius and/or ulna	0.0435
Morocco	2010	Fracture of radius and/or ulna	0.0435
Morocco	2017	Fracture of radius and/or ulna	0.0435
Morocco	1990	Fracture of skull	0.0714
Morocco	1995	Fracture of skull	0.0714
Morocco	2000	Fracture of skull	0.0714
Morocco	2005	Fracture of skull	0.0714
Morocco	2010	Fracture of skull	0.0714
Morocco	2017	Fracture of skull	0.0714
Morocco	1990	Fracture of sternum and/or fracture of one or more ribs	0.1027
Morocco	1995	Fracture of sternum and/or fracture of one or more ribs	0.1027
Morocco	2000	Fracture of sternum and/or fracture of one or more ribs	0.1027
Morocco	2005	Fracture of sternum and/or fracture of one or more ribs	0.1027
Morocco	2010	Fracture of sternum and/or fracture of one or more ribs	0.1027
Morocco	2017	Fracture of sternum and/or fracture of one or more ribs	0.1027
Morocco	1990	Fracture of vertebral column	0.1106
Morocco	1995	Fracture of vertebral column	0.1106
Morocco	2000	Fracture of vertebral column	0.1106
Morocco	2005	Fracture of vertebral column	0.1106
Morocco	2010	Fracture of vertebral column	0.1106
Morocco	2017	Fracture of vertebral column	0.1106
Morocco	1990	Fracture of femur, other than femoral neck	0.042
Morocco	1995	Fracture of femur, other than femoral neck	0.042
Morocco	2000	Fracture of femur, other than femoral neck	0.042
Morocco	2005	Fracture of femur, other than femoral neck	0.042
Morocco	2010	Fracture of femur, other than femoral neck	0.042
Morocco	2017	Fracture of femur, other than femoral neck	0.042
Morocco	1990	Minor TBI	0.1319
Morocco	1995	Minor TBI	0.1319
Morocco	2000	Minor TBI	0.1319
Morocco	2005	Minor TBI	0.1319
Morocco	2010	Minor TBI	0.1319
Morocco	2017	Minor TBI	0.1319
Morocco	1990	Moderate TBI	0.1637
Morocco	1995	Moderate TBI	0.1637
Morocco	2000	Moderate TBI	0.1637
Morocco	2005	Moderate TBI	0.1637
Morocco	2010	Moderate TBI	0.1637
Morocco	2017	Moderate TBI	0.1637
Morocco	1990	Amputation of fingers (excluding thumb)	0.0047
Morocco	1995	Amputation of fingers (excluding thumb)	0.0047
Morocco	2000	Amputation of fingers (excluding thumb)	0.0047
Morocco	2005	Amputation of fingers (excluding thumb)	0.0047
Morocco	2010	Amputation of fingers (excluding thumb)	0.0047
Morocco	2017	Amputation of fingers (excluding thumb)	0.0047
Morocco	1990	Spinal cord lesion at neck level	0.4697
Morocco	1995	Spinal cord lesion at neck level	0.4647
Morocco	2000	Spinal cord lesion at neck level	0.4587
Morocco	2005	Spinal cord lesion at neck level	0.4539
Morocco	2010	Spinal cord lesion at neck level	0.4473
Morocco	2017	Spinal cord lesion at neck level	0.4375
Morocco	1990	Spinal cord lesion below neck level	0.2997
Morocco	1995	Spinal cord lesion below neck level	0.2892
Morocco	2000	Spinal cord lesion below neck level	0.2767
Morocco	2005	Spinal cord lesion below neck level	0.2666
Morocco	2010	Spinal cord lesion below neck level	0.2525
Morocco	2017	Spinal cord lesion below neck level	0.232
Morocco	1990	Drowning and nonfatal submersion	0.2471
Morocco	1995	Drowning and nonfatal submersion	0.2471
Morocco	2000	Drowning and nonfatal submersion	0.2471
Morocco	2005	Drowning and nonfatal submersion	0.2471
Morocco	2010	Drowning and nonfatal submersion	0.2471
Morocco	2017	Drowning and nonfatal submersion	0.2471
Morocco	1990	Asphyxiation	0.2471
Morocco	1995	Asphyxiation	0.2471
Morocco	2000	Asphyxiation	0.2471
Morocco	2005	Asphyxiation	0.2471
Morocco	2010	Asphyxiation	0.2471
Morocco	2017	Asphyxiation	0.2471
Morocco	1990	Crush injury	0.1325
Morocco	1995	Crush injury	0.1325
Morocco	2000	Crush injury	0.1325
Morocco	2005	Crush injury	0.1325
Morocco	2010	Crush injury	0.1325
Morocco	2017	Crush injury	0.1325
Morocco	1990	Nerve injury	0.1129
Morocco	1995	Nerve injury	0.1129
Morocco	2000	Nerve injury	0.1129
Morocco	2005	Nerve injury	0.1129
Morocco	2010	Nerve injury	0.1129
Morocco	2017	Nerve injury	0.1129
Morocco	1990	Injury to eyes	0.0165
Morocco	1995	Injury to eyes	0.0165
Morocco	2000	Injury to eyes	0.0165
Morocco	2005	Injury to eyes	0.0165
Morocco	2010	Injury to eyes	0.0165
Morocco	2017	Injury to eyes	0.0165
Morocco	1990	Amputation of lower limb, unilateral	0.1041
Morocco	1995	Amputation of lower limb, unilateral	0.0985
Morocco	2000	Amputation of lower limb, unilateral	0.0918
Morocco	2005	Amputation of lower limb, unilateral	0.0864
Morocco	2010	Amputation of lower limb, unilateral	0.0789
Morocco	2017	Amputation of lower limb, unilateral	0.068
Morocco	1990	Open wound(s)	0.011
Morocco	1995	Open wound(s)	0.011
Morocco	2000	Open wound(s)	0.011
Morocco	2005	Open wound(s)	0.011
Morocco	2010	Open wound(s)	0.011
Morocco	2017	Open wound(s)	0.011
Morocco	1990	Poisoning requiring urgent care	0.1628
Morocco	1995	Poisoning requiring urgent care	0.1628
Morocco	2000	Poisoning requiring urgent care	0.1628
Morocco	2005	Poisoning requiring urgent care	0.1628
Morocco	2010	Poisoning requiring urgent care	0.1628
Morocco	2017	Poisoning requiring urgent care	0.1628
Morocco	1990	Severe chest injury	0.0474
Morocco	1995	Severe chest injury	0.0474
Morocco	2000	Severe chest injury	0.0474
Morocco	2005	Severe chest injury	0.0474
Morocco	2010	Severe chest injury	0.0474
Morocco	2017	Severe chest injury	0.0474
Morocco	1990	Internal hemorrhage in abdomen and pelvis	0.1143
Morocco	1995	Internal hemorrhage in abdomen and pelvis	0.1143
Morocco	2000	Internal hemorrhage in abdomen and pelvis	0.1143
Morocco	2005	Internal hemorrhage in abdomen and pelvis	0.1143
Morocco	2010	Internal hemorrhage in abdomen and pelvis	0.1143
Morocco	2017	Internal hemorrhage in abdomen and pelvis	0.1143
Morocco	1990	Contusion in any part of the body	0.0075
Morocco	1995	Contusion in any part of the body	0.0075
Morocco	2000	Contusion in any part of the body	0.0075
Morocco	2005	Contusion in any part of the body	0.0075
Morocco	2010	Contusion in any part of the body	0.0075
Morocco	2017	Contusion in any part of the body	0.0075
Morocco	1990	Effect of different environmental factors	0.049
Morocco	1995	Effect of different environmental factors	0.049
Morocco	2000	Effect of different environmental factors	0.049
Morocco	2005	Effect of different environmental factors	0.049
Morocco	2010	Effect of different environmental factors	0.049
Morocco	2017	Effect of different environmental factors	0.049
Morocco	1990	Complications following therapeutic procedures	0.0506
Morocco	1995	Complications following therapeutic procedures	0.0506
Morocco	2000	Complications following therapeutic procedures	0.0506
Morocco	2005	Complications following therapeutic procedures	0.0506
Morocco	2010	Complications following therapeutic procedures	0.0506
Morocco	2017	Complications following therapeutic procedures	0.0506
Morocco	1990	Multiple fractures, dislocations, crashes, wounds, sprains, and strains	0.2249
Morocco	1995	Multiple fractures, dislocations, crashes, wounds, sprains, and strains	0.2105
Morocco	2000	Multiple fractures, dislocations, crashes, wounds, sprains, and strains	0.1934
Morocco	2005	Multiple fractures, dislocations, crashes, wounds, sprains, and strains	0.1795
Morocco	2010	Multiple fractures, dislocations, crashes, wounds, sprains, and strains	0.1602
Morocco	2017	Multiple fractures, dislocations, crashes, wounds, sprains, and strains	0.1321
Morocco	1990	Amputation of upper limb, unilateral	0.0774
Morocco	1995	Amputation of upper limb, unilateral	0.074
Morocco	2000	Amputation of upper limb, unilateral	0.0701
Morocco	2005	Amputation of upper limb, unilateral	0.0669
Morocco	2010	Amputation of upper limb, unilateral	0.0625
Morocco	2017	Amputation of upper limb, unilateral	0.056
Morocco	1990	Amputation of thumb	0.0114
Morocco	1995	Amputation of thumb	0.0114
Morocco	2000	Amputation of thumb	0.0114
Morocco	2005	Amputation of thumb	0.0114
Morocco	2010	Amputation of thumb	0.0114
Morocco	2017	Amputation of thumb	0.0114
Morocco	1990	Amputation of toe/toes	0.0059
Morocco	1995	Amputation of toe/toes	0.0059
Morocco	2000	Amputation of toe/toes	0.0059
Morocco	2005	Amputation of toe/toes	0.0059
Morocco	2010	Amputation of toe/toes	0.0059
Morocco	2017	Amputation of toe/toes	0.0059
Morocco	1990	Burns, <20% total burned surface area without lower airway burns	0.0163
Morocco	1995	Burns, <20% total burned surface area without lower airway burns	0.0163
Morocco	2000	Burns, <20% total burned surface area without lower airway burns	0.0163
Morocco	2005	Burns, <20% total burned surface area without lower airway burns	0.0163
Morocco	2010	Burns, <20% total burned surface area without lower airway burns	0.0163
Morocco	2017	Burns, <20% total burned surface area without lower airway burns	0.0163
Morocco	1990	Burns, >=20% total burned surface area or >= 10% burned surface area if head/neck or hands/wrist involved w/o lower airway burns	0.2903
Morocco	1995	Burns, >=20% total burned surface area or >= 10% burned surface area if head/neck or hands/wrist involved w/o lower airway burns	0.2769
Morocco	2000	Burns, >=20% total burned surface area or >= 10% burned surface area if head/neck or hands/wrist involved w/o lower airway burns	0.2609
Morocco	2005	Burns, >=20% total burned surface area or >= 10% burned surface area if head/neck or hands/wrist involved w/o lower airway burns	0.248
Morocco	2010	Burns, >=20% total burned surface area or >= 10% burned surface area if head/neck or hands/wrist involved w/o lower airway burns	0.23
Morocco	2017	Burns, >=20% total burned surface area or >= 10% burned surface area if head/neck or hands/wrist involved w/o lower airway burns	0.2038
Palestine	1990	Amputation of lower limbs, bilateral	0.2137
Palestine	1995	Amputation of lower limbs, bilateral	0.2016
Palestine	2000	Amputation of lower limbs, bilateral	0.1931
Palestine	2005	Amputation of lower limbs, bilateral	0.1879
Palestine	2010	Amputation of lower limbs, bilateral	0.1867
Palestine	2017	Amputation of lower limbs, bilateral	0.1797
Palestine	1990	Lower airway burns	0.2252
Palestine	1995	Lower airway burns	0.2252
Palestine	2000	Lower airway burns	0.2252
Palestine	2005	Lower airway burns	0.2252
Palestine	2010	Lower airway burns	0.2252
Palestine	2017	Lower airway burns	0.2252
Palestine	1990	Dislocation of hip	0.0159
Palestine	1995	Dislocation of hip	0.0159
Palestine	2000	Dislocation of hip	0.0159
Palestine	2005	Dislocation of hip	0.0159
Palestine	2010	Dislocation of hip	0.0159
Palestine	2017	Dislocation of hip	0.0159
Palestine	1990	Dislocation of knee	0.1134
Palestine	1995	Dislocation of knee	0.1134
Palestine	2000	Dislocation of knee	0.1134
Palestine	2005	Dislocation of knee	0.1134
Palestine	2010	Dislocation of knee	0.1134
Palestine	2017	Dislocation of knee	0.1134
Palestine	1990	Dislocation of shoulder	0.062
Palestine	1995	Dislocation of shoulder	0.062
Palestine	2000	Dislocation of shoulder	0.062
Palestine	2005	Dislocation of shoulder	0.062
Palestine	2010	Dislocation of shoulder	0.062
Palestine	2017	Dislocation of shoulder	0.062
Palestine	1990	Muscle and tendon injuries, including sprains and strains lesser dislocations	0.0075
Palestine	1995	Muscle and tendon injuries, including sprains and strains lesser dislocations	0.0075
Palestine	2000	Muscle and tendon injuries, including sprains and strains lesser dislocations	0.0075
Palestine	2005	Muscle and tendon injuries, including sprains and strains lesser dislocations	0.0075
Palestine	2010	Muscle and tendon injuries, including sprains and strains lesser dislocations	0.0075
Palestine	2017	Muscle and tendon injuries, including sprains and strains lesser dislocations	0.0075
Palestine	1990	Fracture of clavicle, scapula, or humerus	0.0349
Palestine	1995	Fracture of clavicle, scapula, or humerus	0.0349
Palestine	2000	Fracture of clavicle, scapula, or humerus	0.0349
Palestine	2005	Fracture of clavicle, scapula, or humerus	0.0349
Palestine	2010	Fracture of clavicle, scapula, or humerus	0.0349
Palestine	2017	Fracture of clavicle, scapula, or humerus	0.0349
Palestine	1990	Fracture of face bones	0.0669
Palestine	1995	Fracture of face bones	0.0669
Palestine	2000	Fracture of face bones	0.0669
Palestine	2005	Fracture of face bones	0.0669
Palestine	2010	Fracture of face bones	0.0669
Palestine	2017	Fracture of face bones	0.0669
Palestine	1990	Fracture of foot bones except ankle	0.0264
Palestine	1995	Fracture of foot bones except ankle	0.0264
Palestine	2000	Fracture of foot bones except ankle	0.0264
Palestine	2005	Fracture of foot bones except ankle	0.0264
Palestine	2010	Fracture of foot bones except ankle	0.0264
Palestine	2017	Fracture of foot bones except ankle	0.0264
Palestine	1990	Fracture of hand (wrist and other distal part of hand)	0.0138
Palestine	1995	Fracture of hand (wrist and other distal part of hand)	0.0138
Palestine	2000	Fracture of hand (wrist and other distal part of hand)	0.0138
Palestine	2005	Fracture of hand (wrist and other distal part of hand)	0.0138
Palestine	2010	Fracture of hand (wrist and other distal part of hand)	0.0138
Palestine	2017	Fracture of hand (wrist and other distal part of hand)	0.0138
Palestine	1990	Fracture of hip	0.1774
Palestine	1995	Fracture of hip	0.1614
Palestine	2000	Fracture of hip	0.1501
Palestine	2005	Fracture of hip	0.1432
Palestine	2010	Fracture of hip	0.1416
Palestine	2017	Fracture of hip	0.1324
Palestine	1990	Amputation of upper limbs, bilateral	0.2112
Palestine	1995	Amputation of upper limbs, bilateral	0.1947
Palestine	2000	Amputation of upper limbs, bilateral	0.1831
Palestine	2005	Amputation of upper limbs, bilateral	0.1759
Palestine	2010	Amputation of upper limbs, bilateral	0.1742
Palestine	2017	Amputation of upper limbs, bilateral	0.1648
Palestine	1990	Fracture of patella, tibia or fibula, or ankle	0.0554
Palestine	1995	Fracture of patella, tibia or fibula, or ankle	0.0554
Palestine	2000	Fracture of patella, tibia or fibula, or ankle	0.0554
Palestine	2005	Fracture of patella, tibia or fibula, or ankle	0.0554
Palestine	2010	Fracture of patella, tibia or fibula, or ankle	0.0554
Palestine	2017	Fracture of patella, tibia or fibula, or ankle	0.0554
Palestine	1990	Fracture of pelvis	0.1823
Palestine	1995	Fracture of pelvis	0.1823
Palestine	2000	Fracture of pelvis	0.1823
Palestine	2005	Fracture of pelvis	0.1823
Palestine	2010	Fracture of pelvis	0.1823
Palestine	2017	Fracture of pelvis	0.1823
Palestine	1990	Fracture of radius and/or ulna	0.0435
Palestine	1995	Fracture of radius and/or ulna	0.0435
Palestine	2000	Fracture of radius and/or ulna	0.0435
Palestine	2005	Fracture of radius and/or ulna	0.0435
Palestine	2010	Fracture of radius and/or ulna	0.0435
Palestine	2017	Fracture of radius and/or ulna	0.0435
Palestine	1990	Fracture of skull	0.0714
Palestine	1995	Fracture of skull	0.0714
Palestine	2000	Fracture of skull	0.0714
Palestine	2005	Fracture of skull	0.0714
Palestine	2010	Fracture of skull	0.0714
Palestine	2017	Fracture of skull	0.0714
Palestine	1990	Fracture of sternum and/or fracture of one or more ribs	0.1027
Palestine	1995	Fracture of sternum and/or fracture of one or more ribs	0.1027
Palestine	2000	Fracture of sternum and/or fracture of one or more ribs	0.1027
Palestine	2005	Fracture of sternum and/or fracture of one or more ribs	0.1027
Palestine	2010	Fracture of sternum and/or fracture of one or more ribs	0.1027
Palestine	2017	Fracture of sternum and/or fracture of one or more ribs	0.1027
Palestine	1990	Fracture of vertebral column	0.1106
Palestine	1995	Fracture of vertebral column	0.1106
Palestine	2000	Fracture of vertebral column	0.1106
Palestine	2005	Fracture of vertebral column	0.1106
Palestine	2010	Fracture of vertebral column	0.1106
Palestine	2017	Fracture of vertebral column	0.1106
Palestine	1990	Fracture of femur, other than femoral neck	0.042
Palestine	1995	Fracture of femur, other than femoral neck	0.042
Palestine	2000	Fracture of femur, other than femoral neck	0.042
Palestine	2005	Fracture of femur, other than femoral neck	0.042
Palestine	2010	Fracture of femur, other than femoral neck	0.042
Palestine	2017	Fracture of femur, other than femoral neck	0.042
Palestine	1990	Minor TBI	0.1319
Palestine	1995	Minor TBI	0.1319
Palestine	2000	Minor TBI	0.1319
Palestine	2005	Minor TBI	0.1319
Palestine	2010	Minor TBI	0.1319
Palestine	2017	Minor TBI	0.1319
Palestine	1990	Moderate TBI	0.1637
Palestine	1995	Moderate TBI	0.1637
Palestine	2000	Moderate TBI	0.1637
Palestine	2005	Moderate TBI	0.1637
Palestine	2010	Moderate TBI	0.1637
Palestine	2017	Moderate TBI	0.1637
Palestine	1990	Amputation of fingers (excluding thumb)	0.0047
Palestine	1995	Amputation of fingers (excluding thumb)	0.0047
Palestine	2000	Amputation of fingers (excluding thumb)	0.0047
Palestine	2005	Amputation of fingers (excluding thumb)	0.0047
Palestine	2010	Amputation of fingers (excluding thumb)	0.0047
Palestine	2017	Amputation of fingers (excluding thumb)	0.0047
Palestine	1990	Spinal cord lesion at neck level	0.4532
Palestine	1995	Spinal cord lesion at neck level	0.4477
Palestine	2000	Spinal cord lesion at neck level	0.4438
Palestine	2005	Spinal cord lesion at neck level	0.4414
Palestine	2010	Spinal cord lesion at neck level	0.4408
Palestine	2017	Spinal cord lesion at neck level	0.4376
Palestine	1990	Spinal cord lesion below neck level	0.265
Palestine	1995	Spinal cord lesion below neck level	0.2533
Palestine	2000	Spinal cord lesion below neck level	0.2451
Palestine	2005	Spinal cord lesion below neck level	0.2401
Palestine	2010	Spinal cord lesion below neck level	0.2389
Palestine	2017	Spinal cord lesion below neck level	0.2322
Palestine	1990	Drowning and nonfatal submersion	0.2471
Palestine	1995	Drowning and nonfatal submersion	0.2471
Palestine	2000	Drowning and nonfatal submersion	0.2471
Palestine	2005	Drowning and nonfatal submersion	0.2471
Palestine	2010	Drowning and nonfatal submersion	0.2471
Palestine	2017	Drowning and nonfatal submersion	0.2471
Palestine	1990	Asphyxiation	0.2471
Palestine	1995	Asphyxiation	0.2471
Palestine	2000	Asphyxiation	0.2471
Palestine	2005	Asphyxiation	0.2471
Palestine	2010	Asphyxiation	0.2471
Palestine	2017	Asphyxiation	0.2471
Palestine	1990	Crush injury	0.1325
Palestine	1995	Crush injury	0.1325
Palestine	2000	Crush injury	0.1325
Palestine	2005	Crush injury	0.1325
Palestine	2010	Crush injury	0.1325
Palestine	2017	Crush injury	0.1325
Palestine	1990	Nerve injury	0.1129
Palestine	1995	Nerve injury	0.1129
Palestine	2000	Nerve injury	0.1129
Palestine	2005	Nerve injury	0.1129
Palestine	2010	Nerve injury	0.1129
Palestine	2017	Nerve injury	0.1129
Palestine	1990	Injury to eyes	0.0165
Palestine	1995	Injury to eyes	0.0165
Palestine	2000	Injury to eyes	0.0165
Palestine	2005	Injury to eyes	0.0165
Palestine	2010	Injury to eyes	0.0165
Palestine	2017	Injury to eyes	0.0165
Palestine	1990	Amputation of lower limb, unilateral	0.0856
Palestine	1995	Amputation of lower limb, unilateral	0.0794
Palestine	2000	Amputation of lower limb, unilateral	0.075
Palestine	2005	Amputation of lower limb, unilateral	0.0723
Palestine	2010	Amputation of lower limb, unilateral	0.0717
Palestine	2017	Amputation of lower limb, unilateral	0.0681
Palestine	1990	Open wound(s)	0.011
Palestine	1995	Open wound(s)	0.011
Palestine	2000	Open wound(s)	0.011
Palestine	2005	Open wound(s)	0.011
Palestine	2010	Open wound(s)	0.011
Palestine	2017	Open wound(s)	0.011
Palestine	1990	Poisoning requiring urgent care	0.1628
Palestine	1995	Poisoning requiring urgent care	0.1628
Palestine	2000	Poisoning requiring urgent care	0.1628
Palestine	2005	Poisoning requiring urgent care	0.1628
Palestine	2010	Poisoning requiring urgent care	0.1628
Palestine	2017	Poisoning requiring urgent care	0.1628
Palestine	1990	Severe chest injury	0.0474
Palestine	1995	Severe chest injury	0.0474
Palestine	2000	Severe chest injury	0.0474
Palestine	2005	Severe chest injury	0.0474
Palestine	2010	Severe chest injury	0.0474
Palestine	2017	Severe chest injury	0.0474
Palestine	1990	Internal hemorrhage in abdomen and pelvis	0.1143
Palestine	1995	Internal hemorrhage in abdomen and pelvis	0.1143
Palestine	2000	Internal hemorrhage in abdomen and pelvis	0.1143
Palestine	2005	Internal hemorrhage in abdomen and pelvis	0.1143
Palestine	2010	Internal hemorrhage in abdomen and pelvis	0.1143
Palestine	2017	Internal hemorrhage in abdomen and pelvis	0.1143
Palestine	1990	Contusion in any part of the body	0.0075
Palestine	1995	Contusion in any part of the body	0.0075
Palestine	2000	Contusion in any part of the body	0.0075
Palestine	2005	Contusion in any part of the body	0.0075
Palestine	2010	Contusion in any part of the body	0.0075
Palestine	2017	Contusion in any part of the body	0.0075
Palestine	1990	Effect of different environmental factors	0.049
Palestine	1995	Effect of different environmental factors	0.049
Palestine	2000	Effect of different environmental factors	0.049
Palestine	2005	Effect of different environmental factors	0.049
Palestine	2010	Effect of different environmental factors	0.049
Palestine	2017	Effect of different environmental factors	0.049
Palestine	1990	Complications following therapeutic procedures	0.0506
Palestine	1995	Complications following therapeutic procedures	0.0506
Palestine	2000	Complications following therapeutic procedures	0.0506
Palestine	2005	Complications following therapeutic procedures	0.0506
Palestine	2010	Complications following therapeutic procedures	0.0506
Palestine	2017	Complications following therapeutic procedures	0.0506
Palestine	1990	Multiple fractures, dislocations, crashes, wounds, sprains, and strains	0.1774
Palestine	1995	Multiple fractures, dislocations, crashes, wounds, sprains, and strains	0.1614
Palestine	2000	Multiple fractures, dislocations, crashes, wounds, sprains, and strains	0.1501
Palestine	2005	Multiple fractures, dislocations, crashes, wounds, sprains, and strains	0.1432
Palestine	2010	Multiple fractures, dislocations, crashes, wounds, sprains, and strains	0.1416
Palestine	2017	Multiple fractures, dislocations, crashes, wounds, sprains, and strains	0.1324
Palestine	1990	Amputation of upper limb, unilateral	0.0664
Palestine	1995	Amputation of upper limb, unilateral	0.0628
Palestine	2000	Amputation of upper limb, unilateral	0.0602
Palestine	2005	Amputation of upper limb, unilateral	0.0586
Palestine	2010	Amputation of upper limb, unilateral	0.0582
Palestine	2017	Amputation of upper limb, unilateral	0.0561
Palestine	1990	Amputation of thumb	0.0114
Palestine	1995	Amputation of thumb	0.0114
Palestine	2000	Amputation of thumb	0.0114
Palestine	2005	Amputation of thumb	0.0114

Palestine	2010	Amputation of thumb	0.0114
Palestine	2017	Amputation of thumb	0.0114
Palestine	1990	Amputation of toe/toes	0.0059
Palestine	1995	Amputation of toe/toes	0.0059
Palestine	2000	Amputation of toe/toes	0.0059
Palestine	2005	Amputation of toe/toes	0.0059
Palestine	2010	Amputation of toe/toes	0.0059
Palestine	2017	Amputation of toe/toes	0.0059
Palestine	1990	Burns, <20% total burned surface area without lower airway burns	0.0163
Palestine	1995	Burns, <20% total burned surface area without lower airway burns	0.0163
Palestine	2000	Burns, <20% total burned surface area without lower airway burns	0.0163
Palestine	2005	Burns, <20% total burned surface area without lower airway burns	0.0163
Palestine	2010	Burns, <20% total burned surface area without lower airway burns	0.0163
Palestine	2017	Burns, <20% total burned surface area without lower airway burns	0.0163
Palestine	1990	Burns, >=20% total burned surface area or >= 10% burned surface area if head/neck or hands/wrist involved w/o lower airway burns	0.246
Palestine	1995	Burns, >=20% total burned surface area or >= 10% burned surface area if head/neck or hands/wrist involved w/o lower airway burns	0.2311
Palestine	2000	Burns, >=20% total burned surface area or >= 10% burned surface area if head/neck or hands/wrist involved w/o lower airway burns	0.2206
Palestine	2005	Burns, >=20% total burned surface area or >= 10% burned surface area if head/neck or hands/wrist involved w/o lower airway burns	0.2142
Palestine	2010	Burns, >=20% total burned surface area or >= 10% burned surface area if head/neck or hands/wrist involved w/o lower airway burns	0.2127
Palestine	2017	Burns, >=20% total burned surface area or >= 10% burned surface area if head/neck or hands/wrist involved w/o lower airway burns	0.2041
Oman	1990	Amputation of lower limbs, bilateral	0.1989
Oman	1995	Amputation of lower limbs, bilateral	0.1775
Oman	2000	Amputation of lower limbs, bilateral	0.1619
Oman	2005	Amputation of lower limbs, bilateral	0.1462
Oman	2010	Amputation of lower limbs, bilateral	0.1376
Oman	2017	Amputation of lower limbs, bilateral	0.1233
Oman	1990	Lower airway burns	0.2252
Oman	1995	Lower airway burns	0.2252
Oman	2000	Lower airway burns	0.2252
Oman	2005	Lower airway burns	0.2252
Oman	2010	Lower airway burns	0.2252
Oman	2017	Lower airway burns	0.2252
Oman	1990	Dislocation of hip	0.0159
Oman	1995	Dislocation of hip	0.0159
Oman	2000	Dislocation of hip	0.0159
Oman	2005	Dislocation of hip	0.0159
Oman	2010	Dislocation of hip	0.0159
Oman	2017	Dislocation of hip	0.0159
Oman	1990	Dislocation of knee	0.1134
Oman	1995	Dislocation of knee	0.1134
Oman	2000	Dislocation of knee	0.1134
Oman	2005	Dislocation of knee	0.1134
Oman	2010	Dislocation of knee	0.1134
Oman	2017	Dislocation of knee	0.1134
Oman	1990	Dislocation of shoulder	0.062
Oman	1995	Dislocation of shoulder	0.062
Oman	2000	Dislocation of shoulder	0.062
Oman	2005	Dislocation of shoulder	0.062
Oman	2010	Dislocation of shoulder	0.062
Oman	2017	Dislocation of shoulder	0.062
Oman	1990	Muscle and tendon injuries, including sprains and strains lesser dislocations	0.0075
Oman	1995	Muscle and tendon injuries, including sprains and strains lesser dislocations	0.0075
Oman	2000	Muscle and tendon injuries, including sprains and strains lesser dislocations	0.0075
Oman	2005	Muscle and tendon injuries, including sprains and strains lesser dislocations	0.0075
Oman	2010	Muscle and tendon injuries, including sprains and strains lesser dislocations	0.0075
Oman	2017	Muscle and tendon injuries, including sprains and strains lesser dislocations	0.0075
Oman	1990	Fracture of clavicle, scapula, or humerus	0.0349
Oman	1995	Fracture of clavicle, scapula, or humerus	0.0349
Oman	2000	Fracture of clavicle, scapula, or humerus	0.0349
Oman	2005	Fracture of clavicle, scapula, or humerus	0.0349
Oman	2010	Fracture of clavicle, scapula, or humerus	0.0349
Oman	2017	Fracture of clavicle, scapula, or humerus	0.0349
Oman	1990	Fracture of face bones	0.0669
Oman	1995	Fracture of face bones	0.0669
Oman	2000	Fracture of face bones	0.0669
Oman	2005	Fracture of face bones	0.0669
Oman	2010	Fracture of face bones	0.0669
Oman	2017	Fracture of face bones	0.0669
Oman	1990	Fracture of foot bones except ankle	0.0264
Oman	1995	Fracture of foot bones except ankle	0.0264
Oman	2000	Fracture of foot bones except ankle	0.0264
Oman	2005	Fracture of foot bones except ankle	0.0264
Oman	2010	Fracture of foot bones except ankle	0.0264
Oman	2017	Fracture of foot bones except ankle	0.0264
Oman	1990	Fracture of hand (wrist and other distal part of hand)	0.0138
Oman	1995	Fracture of hand (wrist and other distal part of hand)	0.0138
Oman	2000	Fracture of hand (wrist and other distal part of hand)	0.0138
Oman	2005	Fracture of hand (wrist and other distal part of hand)	0.0138
Oman	2010	Fracture of hand (wrist and other distal part of hand)	0.0138
Oman	2017	Fracture of hand (wrist and other distal part of hand)	0.0138
Oman	1990	Fracture of hip	0.1578
Oman	1995	Fracture of hip	0.1295
Oman	2000	Fracture of hip	0.1089
Oman	2005	Fracture of hip	0.0881
Oman	2010	Fracture of hip	0.0767
Oman	2017	Fracture of hip	0.0577
Oman	1990	Amputation of upper limbs, bilateral	0.191
Oman	1995	Amputation of upper limbs, bilateral	0.1618
Oman	2000	Amputation of upper limbs, bilateral	0.1405
Oman	2005	Amputation of upper limbs, bilateral	0.119
Oman	2010	Amputation of upper limbs, bilateral	0.1073
Oman	2017	Amputation of upper limbs, bilateral	0.0876
Oman	1990	Fracture of patella, tibia or fibula, or ankle	0.0554
Oman	1995	Fracture of patella, tibia or fibula, or ankle	0.0554
Oman	2000	Fracture of patella, tibia or fibula, or ankle	0.0554
Oman	2005	Fracture of patella, tibia or fibula, or ankle	0.0554
Oman	2010	Fracture of patella, tibia or fibula, or ankle	0.0554
Oman	2017	Fracture of patella, tibia or fibula, or ankle	0.0554
Oman	1990	Fracture of pelvis	0.1823
Oman	1995	Fracture of pelvis	0.1823
Oman	2000	Fracture of pelvis	0.1823
Oman	2005	Fracture of pelvis	0.1823
Oman	2010	Fracture of pelvis	0.1823
Oman	2017	Fracture of pelvis	0.1823
Oman	1990	Fracture of radius and/or ulna	0.0435
Oman	1995	Fracture of radius and/or ulna	0.0435
Oman	2000	Fracture of radius and/or ulna	0.0435
Oman	2005	Fracture of radius and/or ulna	0.0435
Oman	2010	Fracture of radius and/or ulna	0.0435
Oman	2017	Fracture of radius and/or ulna	0.0435
Oman	1990	Fracture of skull	0.0714
Oman	1995	Fracture of skull	0.0714
Oman	2000	Fracture of skull	0.0714
Oman	2005	Fracture of skull	0.0714
Oman	2010	Fracture of skull	0.0714
Oman	2017	Fracture of skull	0.0714
Oman	1990	Fracture of sternum and/or fracture of one or more ribs	0.1027
Oman	1995	Fracture of sternum and/or fracture of one or more ribs	0.1027
Oman	2000	Fracture of sternum and/or fracture of one or more ribs	0.1027
Oman	2005	Fracture of sternum and/or fracture of one or more ribs	0.1027
Oman	2010	Fracture of sternum and/or fracture of one or more ribs	0.1027
Oman	2017	Fracture of sternum and/or fracture of one or more ribs	0.1027
Oman	1990	Fracture of vertebral column	0.1106
Oman	1995	Fracture of vertebral column	0.1106
Oman	2000	Fracture of vertebral column	0.1106
Oman	2005	Fracture of vertebral column	0.1106
Oman	2010	Fracture of vertebral column	0.1106
Oman	2017	Fracture of vertebral column	0.1106
Oman	1990	Fracture of femur, other than femoral neck	0.042
Oman	1995	Fracture of femur, other than femoral neck	0.042
Oman	2000	Fracture of femur, other than femoral neck	0.042
Oman	2005	Fracture of femur, other than femoral neck	0.042
Oman	2010	Fracture of femur, other than femoral neck	0.042
Oman	2017	Fracture of femur, other than femoral neck	0.042
Oman	1990	Minor TBI	0.1319
Oman	1995	Minor TBI	0.1319
Oman	2000	Minor TBI	0.1319
Oman	2005	Minor TBI	0.1319
Oman	2010	Minor TBI	0.1319
Oman	2017	Minor TBI	0.1319
Oman	1990	Moderate TBI	0.1637
Oman	1995	Moderate TBI	0.1637
Oman	2000	Moderate TBI	0.1637
Oman	2005	Moderate TBI	0.1637
Oman	2010	Moderate TBI	0.1637
Oman	2017	Moderate TBI	0.1637
Oman	1990	Amputation of fingers (excluding thumb)	0.0047
Oman	1995	Amputation of fingers (excluding thumb)	0.0047
Oman	2000	Amputation of fingers (excluding thumb)	0.0047
Oman	2005	Amputation of fingers (excluding thumb)	0.0047
Oman	2010	Amputation of fingers (excluding thumb)	0.0047
Oman	2017	Amputation of fingers (excluding thumb)	0.0047
Oman	1990	Spinal cord lesion at neck level	0.4464
Oman	1995	Spinal cord lesion at neck level	0.4366
Oman	2000	Spinal cord lesion at neck level	0.4295
Oman	2005	Spinal cord lesion at neck level	0.4223
Oman	2010	Spinal cord lesion at neck level	0.4184
Oman	2017	Spinal cord lesion at neck level	0.4118
Oman	1990	Spinal cord lesion below neck level	0.2507
Oman	1995	Spinal cord lesion below neck level	0.2301
Oman	2000	Spinal cord lesion below neck level	0.215
Oman	2005	Spinal cord lesion below neck level	0.1998
Oman	2010	Spinal cord lesion below neck level	0.1916
Oman	2017	Spinal cord lesion below neck level	0.1777
Oman	1990	Drowning and nonfatal submersion	0.2471
Oman	1995	Drowning and nonfatal submersion	0.2471
Oman	2000	Drowning and nonfatal submersion	0.2471
Oman	2005	Drowning and nonfatal submersion	0.2471
Oman	2010	Drowning and nonfatal submersion	0.2471
Oman	2017	Drowning and nonfatal submersion	0.2471
Oman	1990	Asphyxiation	0.2471
Oman	1995	Asphyxiation	0.2471
Oman	2000	Asphyxiation	0.2471
Oman	2005	Asphyxiation	0.2471
Oman	2010	Asphyxiation	0.2471
Oman	2017	Asphyxiation	0.2471
Oman	1990	Crush injury	0.1325
Oman	1995	Crush injury	0.1325
Oman	2000	Crush injury	0.1325
Oman	2005	Crush injury	0.1325
Oman	2010	Crush injury	0.1325
Oman	2017	Crush injury	0.1325
Oman	1990	Nerve injury	0.1129
Oman	1995	Nerve injury	0.1129
Oman	2000	Nerve injury	0.1129
Oman	2005	Nerve injury	0.1129
Oman	2010	Nerve injury	0.1129
Oman	2017	Nerve injury	0.1129
Oman	1990	Injury to eyes	0.0165
Oman	1995	Injury to eyes	0.0165
Oman	2000	Injury to eyes	0.0165
Oman	2005	Injury to eyes	0.0165
Oman	2010	Injury to eyes	0.0165
Oman	2017	Injury to eyes	0.0165
Oman	1990	Amputation of lower limb, unilateral	0.078
Oman	1995	Amputation of lower limb, unilateral	0.0669
Oman	2000	Amputation of lower limb, unilateral	0.0589
Oman	2005	Amputation of lower limb, unilateral	0.0508
Oman	2010	Amputation of lower limb, unilateral	0.0464
Oman	2017	Amputation of lower limb, unilateral	0.039
Oman	1990	Open wound(s)	0.011
Oman	1995	Open wound(s)	0.011
Oman	2000	Open wound(s)	0.011
Oman	2005	Open wound(s)	0.011
Oman	2010	Open wound(s)	0.011
Oman	2017	Open wound(s)	0.011
Oman	1990	Poisoning requiring urgent care	0.1628
Oman	1995	Poisoning requiring urgent care	0.1628
Oman	2000	Poisoning requiring urgent care	0.1628
Oman	2005	Poisoning requiring urgent care	0.1628
Oman	2010	Poisoning requiring urgent care	0.1628
Oman	2017	Poisoning requiring urgent care	0.1628
Oman	1990	Severe chest injury	0.0474
Oman	1995	Severe chest injury	0.0474
Oman	2000	Severe chest injury	0.0474
Oman	2005	Severe chest injury	0.0474
Oman	2010	Severe chest injury	0.0474
Oman	2017	Severe chest injury	0.0474
Oman	1990	Internal hemorrhage in abdomen and pelvis	0.1143
Oman	1995	Internal hemorrhage in abdomen and pelvis	0.1143
Oman	2000	Internal hemorrhage in abdomen and pelvis	0.1143
Oman	2005	Internal hemorrhage in abdomen and pelvis	0.1143
Oman	2010	Internal hemorrhage in abdomen and pelvis	0.1143
Oman	2017	Internal hemorrhage in abdomen and pelvis	0.1143
Oman	1990	Contusion in any part of the body	0.0075
Oman	1995	Contusion in any part of the body	0.0075
Oman	2000	Contusion in any part of the body	0.0075
Oman	2005	Contusion in any part of the body	0.0075
Oman	2010	Contusion in any part of the body	0.0075
Oman	2017	Contusion in any part of the body	0.0075
Oman	1990	Effect of different environmental factors	0.049
Oman	1995	Effect of different environmental factors	0.049
Oman	2000	Effect of different environmental factors	0.049
Oman	2005	Effect of different environmental factors	0.049
Oman	2010	Effect of different environmental factors	0.049
Oman	2017	Effect of different environmental factors	0.049
Oman	1990	Complications following therapeutic procedures	0.0506
Oman	1995	Complications following therapeutic procedures	0.0506
Oman	2000	Complications following therapeutic procedures	0.0506
Oman	2005	Complications following therapeutic procedures	0.0506
Oman	2010	Complications following therapeutic procedures	0.0506
Oman	2017	Complications following therapeutic procedures	0.0506
Oman	1990	Multiple fractures, dislocations, crashes, wounds, sprains, and strains	0.1578
Oman	1995	Multiple fractures, dislocations, crashes, wounds, sprains, and strains	0.1295
Oman	2000	Multiple fractures, dislocations, crashes, wounds, sprains, and strains	0.1089
Oman	2005	Multiple fractures, dislocations, crashes, wounds, sprains, and strains	0.0881
Oman	2010	Multiple fractures, dislocations, crashes, wounds, sprains, and strains	0.0767
Oman	2017	Multiple fractures, dislocations, crashes, wounds, sprains, and strains	0.0577
Oman	1990	Amputation of upper limb, unilateral	0.0619
Oman	1995	Amputation of upper limb, unilateral	0.0555
Oman	2000	Amputation of upper limb, unilateral	0.0507
Oman	2005	Amputation of upper limb, unilateral	0.0459
Oman	2010	Amputation of upper limb, unilateral	0.0434
Oman	2017	Amputation of upper limb, unilateral	0.039
Oman	1990	Amputation of thumb	0.0114
Oman	1995	Amputation of thumb	0.0114
Oman	2000	Amputation of thumb	0.0114
Oman	2005	Amputation of thumb	0.0114
Oman	2010	Amputation of thumb	0.0114
Oman	2017	Amputation of thumb	0.0114
Oman	1990	Amputation of toe/toes	0.0059
Oman	1995	Amputation of toe/toes	0.0059
Oman	2000	Amputation of toe/toes	0.0059
Oman	2005	Amputation of toe/toes	0.0059
Oman	2010	Amputation of toe/toes	0.0059
Oman	2017	Amputation of toe/toes	0.0059
Oman	1990	Burns, <20% total burned surface area without lower airway burns	0.0163
Oman	1995	Burns, <20% total burned surface area without lower airway burns	0.0163
Oman	2000	Burns, <20% total burned surface area without lower airway burns	0.0163
Oman	2005	Burns, <20% total burned surface area without lower airway burns	0.0163
Oman	2010	Burns, <20% total burned surface area without lower airway burns	0.0163
Oman	2017	Burns, <20% total burned surface area without lower airway burns	0.0163
Oman	1990	Burns, >=20% total burned surface area or >= 10% burned surface area if head/neck or hands/wrist involved w/o lower airway burns	0.2278
Oman	1995	Burns, >=20% total burned surface area or >= 10% burned surface area if head/neck or hands/wrist involved w/o lower airway burns	0.2014
Oman	2000	Burns, >=20% total burned surface area or >= 10% burned surface area if head/neck or hands/wrist involved w/o lower airway burns	0.1822
Oman	2005	Burns, >=20% total burned surface area or >= 10% burned surface area if head/neck or hands/wrist involved w/o lower airway burns	0.1629
Oman	2010	Burns, >=20% total burned surface area or >= 10% burned surface area if head/neck or hands/wrist involved w/o lower airway burns	0.1523
Oman	2017	Burns, >=20% total burned surface area or >= 10% burned surface area if head/neck or hands/wrist involved w/o lower airway burns	0.1346
Qatar	1990	Amputation of lower limbs, bilateral	0.1819
Qatar	1995	Amputation of lower limbs, bilateral	0.1747
Qatar	2000	Amputation of lower limbs, bilateral	0.158
Qatar	2005	Amputation of lower limbs, bilateral	0.1325
Qatar	2010	Amputation of lower limbs, bilateral	0.1232
Qatar	2017	Amputation of lower limbs, bilateral	0.1232
Qatar	1990	Lower airway burns	0.2252
Qatar	1995	Lower airway burns	0.2252
Qatar	2000	Lower airway burns	0.2252
Qatar	2005	Lower airway burns	0.2252
Qatar	2010	Lower airway burns	0.2252
Qatar	2017	Lower airway burns	0.2252
Qatar	1990	Dislocation of hip	0.0159
Qatar	1995	Dislocation of hip	0.0159
Qatar	2000	Dislocation of hip	0.0159
Qatar	2005	Dislocation of hip	0.0159
Qatar	2010	Dislocation of hip	0.0159
Qatar	2017	Dislocation of hip	0.0159
Qatar	1990	Dislocation of knee	0.1134
Qatar	1995	Dislocation of knee	0.1134
Qatar	2000	Dislocation of knee	0.1134
Qatar	2005	Dislocation of knee	0.1134
Qatar	2010	Dislocation of knee	0.1134
Qatar	2017	Dislocation of knee	0.1134
Qatar	1990	Dislocation of shoulder	0.062
Qatar	1995	Dislocation of shoulder	0.062
Qatar	2000	Dislocation of shoulder	0.062
Qatar	2005	Dislocation of shoulder	0.062
Qatar	2010	Dislocation of shoulder	0.062
Qatar	2017	Dislocation of shoulder	0.062
Qatar	1990	Muscle and tendon injuries, including sprains and strains lesser dislocations	0.0075
Qatar	1995	Muscle and tendon injuries, including sprains and strains lesser dislocations	0.0075
Qatar	2000	Muscle and tendon injuries, including sprains and strains lesser dislocations	0.0075
Qatar	2005	Muscle and tendon injuries, including sprains and strains lesser dislocations	0.0075
Qatar	2010	Muscle and tendon injuries, including sprains and strains lesser dislocations	0.0075
Qatar	2017	Muscle and tendon injuries, including sprains and strains lesser dislocations	0.0075
Qatar	1990	Fracture of clavicle, scapula, or humerus	0.0349
Qatar	1995	Fracture of clavicle, scapula, or humerus	0.0349
Qatar	2000	Fracture of clavicle, scapula, or humerus	0.0349
Qatar	2005	Fracture of clavicle, scapula, or humerus	0.0349
Qatar	2010	Fracture of clavicle, scapula, or humerus	0.0349
Qatar	2017	Fracture of clavicle, scapula, or humerus	0.0349
Qatar	1990	Fracture of face bones	0.0669
Qatar	1995	Fracture of face bones	0.0669
Qatar	2000	Fracture of face bones	0.0669
Qatar	2005	Fracture of face bones	0.0669
Qatar	2010	Fracture of face bones	0.0669
Qatar	2017	Fracture of face bones	0.0669
Qatar	1990	Fracture of foot bones except ankle	0.0264
Qatar	1995	Fracture of foot bones except ankle	0.0264
Qatar	2000	Fracture of foot bones except ankle	0.0264
Qatar	2005	Fracture of foot bones except ankle	0.0264
Qatar	2010	Fracture of foot bones except ankle	0.0264
Qatar	2017	Fracture of foot bones except ankle	0.0264
Qatar	1990	Fracture of hand (wrist and other distal part of hand)	0.0138
Qatar	1995	Fracture of hand (wrist and other distal part of hand)	0.0138
Qatar	2000	Fracture of hand (wrist and other distal part of hand)	0.0138
Qatar	2005	Fracture of hand (wrist and other distal part of hand)	0.0138
Qatar	2010	Fracture of hand (wrist and other distal part of hand)	0.0138
Qatar	2017	Fracture of hand (wrist and other distal part of hand)	0.0138
Qatar	1990	Fracture of hip	0.1353
Qatar	1995	Fracture of hip	0.1258
Qatar	2000	Fracture of hip	0.1037
Qatar	2005	Fracture of hip	0.0689
Qatar	2010	Fracture of hip	0.0577
Qatar	2017	Fracture of hip	0.0577
Qatar	1990	Amputation of upper limbs, bilateral	0.1678
Qatar	1995	Amputation of upper limbs, bilateral	0.158
Qatar	2000	Amputation of upper limbs, bilateral	0.1351
Qatar	2005	Amputation of upper limbs, bilateral	0.1003
Qatar	2010	Amputation of upper limbs, bilateral	0.0876
Qatar	2017	Amputation of upper limbs, bilateral	0.0876
Qatar	1990	Fracture of patella, tibia or fibula, or ankle	0.0554
Qatar	1995	Fracture of patella, tibia or fibula, or ankle	0.0554
Qatar	2000	Fracture of patella, tibia or fibula, or ankle	0.0554
Qatar	2005	Fracture of patella, tibia or fibula, or ankle	0.0554
Qatar	2010	Fracture of patella, tibia or fibula, or ankle	0.0554
Qatar	2017	Fracture of patella, tibia or fibula, or ankle	0.0554
Qatar	1990	Fracture of pelvis	0.1823
Qatar	1995	Fracture of pelvis	0.1823
Qatar	2000	Fracture of pelvis	0.1823
Qatar	2005	Fracture of pelvis	0.1823
Qatar	2010	Fracture of pelvis	0.1823
Qatar	2017	Fracture of pelvis	0.1823
Qatar	1990	Fracture of radius and/or ulna	0.0435
Qatar	1995	Fracture of radius and/or ulna	0.0435
Qatar	2000	Fracture of radius and/or ulna	0.0435
Qatar	2005	Fracture of radius and/or ulna	0.0435
Qatar	2010	Fracture of radius and/or ulna	0.0435
Qatar	2017	Fracture of radius and/or ulna	0.0435
Qatar	1990	Fracture of skull	0.0714
Qatar	1995	Fracture of skull	0.0714
Qatar	2000	Fracture of skull	0.0714
Qatar	2005	Fracture of skull	0.0714
Qatar	2010	Fracture of skull	0.0714
Qatar	2017	Fracture of skull	0.0714
Qatar	1990	Fracture of sternum and/or fracture of one or more ribs	0.1027
Qatar	1995	Fracture of sternum and/or fracture of one or more ribs	0.1027
Qatar	2000	Fracture of sternum and/or fracture of one or more ribs	0.1027
Qatar	2005	Fracture of sternum and/or fracture of one or more ribs	0.1027
Qatar	2010	Fracture of sternum and/or fracture of one or more ribs	0.1027
Qatar	2017	Fracture of sternum and/or fracture of one or more ribs	0.1027
Qatar	1990	Fracture of vertebral column	0.1106
Qatar	1995	Fracture of vertebral column	0.1106
Qatar	2000	Fracture of vertebral column	0.1106
Qatar	2005	Fracture of vertebral column	0.1106
Qatar	2010	Fracture of vertebral column	0.1106
Qatar	2017	Fracture of vertebral column	0.1106
Qatar	1990	Fracture of femur, other than femoral neck	0.042
Qatar	1995	Fracture of femur, other than femoral neck	0.042
Qatar	2000	Fracture of femur, other than femoral neck	0.042
Qatar	2005	Fracture of femur, other than femoral neck	0.042
Qatar	2010	Fracture of femur, other than femoral neck	0.042
Qatar	2017	Fracture of femur, other than femoral neck	0.042
Qatar	1990	Minor TBI	0.1319
Qatar	1995	Minor TBI	0.1319
Qatar	2000	Minor TBI	0.1319
Qatar	2005	Minor TBI	0.1319
Qatar	2010	Minor TBI	0.1319
Qatar	2017	Minor TBI	0.1319
Qatar	1990	Moderate TBI	0.1637
Qatar	1995	Moderate TBI	0.1637
Qatar	2000	Moderate TBI	0.1637
Qatar	2005	Moderate TBI	0.1637
Qatar	2010	Moderate TBI	0.1637
Qatar	2017	Moderate TBI	0.1637
Qatar	1990	Amputation of fingers (excluding thumb)	0.0047
Qatar	1995	Amputation of fingers (excluding thumb)	0.0047
Qatar	2000	Amputation of fingers (excluding thumb)	0.0047
Qatar	2005	Amputation of fingers (excluding thumb)	0.0047
Qatar	2010	Amputation of fingers (excluding thumb)	0.0047
Qatar	2017	Amputation of fingers (excluding thumb)	0.0047
Qatar	1990	Spinal cord lesion at neck level	0.4386
Qatar	1995	Spinal cord lesion at neck level	0.4354
Qatar	2000	Spinal cord lesion at neck level	0.4277
Qatar	2005	Spinal cord lesion at neck level	0.4161
Qatar	2010	Spinal cord lesion at neck level	0.4118
Qatar	2017	Spinal cord lesion at neck level	0.4118
Qatar	1990	Spinal cord lesion below neck level	0.2343
Qatar	1995	Spinal cord lesion below neck level	0.2274
Qatar	2000	Spinal cord lesion below neck level	0.2112
Qatar	2005	Spinal cord lesion below neck level	0.1866
Qatar	2010	Spinal cord lesion below neck level	0.1777
Qatar	2017	Spinal cord lesion below neck level	0.1777
Qatar	1990	Drowning and nonfatal submersion	0.2471
Qatar	1995	Drowning and nonfatal submersion	0.2471
Qatar	2000	Drowning and nonfatal submersion	0.2471
Qatar	2005	Drowning and nonfatal submersion	0.2471
Qatar	2010	Drowning and nonfatal submersion	0.2471
Qatar	2017	Drowning and nonfatal submersion	0.2471
Qatar	1990	Asphyxiation	0.2471
Qatar	1995	Asphyxiation	0.2471
Qatar	2000	Asphyxiation	0.2471

Qatar	2005	Asphyxiation	0.2471
Qatar	2010	Asphyxiation	0.2471
Qatar	2017	Asphyxiation	0.2471
Qatar	1990	Crush injury	0.1325
Qatar	1995	Crush injury	0.1325
Qatar	2000	Crush injury	0.1325
Qatar	2005	Crush injury	0.1325
Qatar	2010	Crush injury	0.1325
Qatar	2017	Crush injury	0.1325
Qatar	1990	Nerve injury	0.1129
Qatar	1995	Nerve injury	0.1129
Qatar	2000	Nerve injury	0.1129
Qatar	2005	Nerve injury	0.1129
Qatar	2010	Nerve injury	0.1129
Qatar	2017	Nerve injury	0.1129
Qatar	1990	Injury to eyes	0.0165
Qatar	1995	Injury to eyes	0.0165
Qatar	2000	Injury to eyes	0.0165
Qatar	2005	Injury to eyes	0.0165
Qatar	2010	Injury to eyes	0.0165
Qatar	2017	Injury to eyes	0.0165
Qatar	1990	Amputation of lower limb, unilateral	0.0692
Qatar	1995	Amputation of lower limb, unilateral	0.0655
Qatar	2000	Amputation of lower limb, unilateral	0.0569
Qatar	2005	Amputation of lower limb, unilateral	0.0437
Qatar	2010	Amputation of lower limb, unilateral	0.039
Qatar	2017	Amputation of lower limb, unilateral	0.039
Qatar	1990	Open wound(s)	0.011
Qatar	1995	Open wound(s)	0.011
Qatar	2000	Open wound(s)	0.011
Qatar	2005	Open wound(s)	0.011
Qatar	2010	Open wound(s)	0.011
Qatar	2017	Open wound(s)	0.011
Qatar	1990	Poisoning requiring urgent care	0.1628
Qatar	1995	Poisoning requiring urgent care	0.1628
Qatar	2000	Poisoning requiring urgent care	0.1628
Qatar	2005	Poisoning requiring urgent care	0.1628
Qatar	2010	Poisoning requiring urgent care	0.1628
Qatar	2017	Poisoning requiring urgent care	0.1628
Qatar	1990	Severe chest injury	0.0474
Qatar	1995	Severe chest injury	0.0474
Qatar	2000	Severe chest injury	0.0474
Qatar	2005	Severe chest injury	0.0474
Qatar	2010	Severe chest injury	0.0474
Qatar	2017	Severe chest injury	0.0474
Qatar	1990	Internal hemorrhage in abdomen and pelvis	0.1143
Qatar	1995	Internal hemorrhage in abdomen and pelvis	0.1143
Qatar	2000	Internal hemorrhage in abdomen and pelvis	0.1143
Qatar	2005	Internal hemorrhage in abdomen and pelvis	0.1143
Qatar	2010	Internal hemorrhage in abdomen and pelvis	0.1143
Qatar	2017	Internal hemorrhage in abdomen and pelvis	0.1143
Qatar	1990	Contusion in any part of the body	0.0075
Qatar	1995	Contusion in any part of the body	0.0075
Qatar	2000	Contusion in any part of the body	0.0075
Qatar	2005	Contusion in any part of the body	0.0075
Qatar	2010	Contusion in any part of the body	0.0075
Qatar	2017	Contusion in any part of the body	0.0075
Qatar	1990	Effect of different environmental factors	0.049
Qatar	1995	Effect of different environmental factors	0.049
Qatar	2000	Effect of different environmental factors	0.049
Qatar	2005	Effect of different environmental factors	0.049
Qatar	2010	Effect of different environmental factors	0.049
Qatar	2017	Effect of different environmental factors	0.049
Qatar	1990	Complications following therapeutic procedures	0.0506
Qatar	1995	Complications following therapeutic procedures	0.0506
Qatar	2000	Complications following therapeutic procedures	0.0506
Qatar	2005	Complications following therapeutic procedures	0.0506
Qatar	2010	Complications following therapeutic procedures	0.0506
Qatar	2017	Complications following therapeutic procedures	0.0506
Qatar	1990	Multiple fractures, dislocations, crashes, wounds, sprains, and strains	0.1353
Qatar	1995	Multiple fractures, dislocations, crashes, wounds, sprains, and strains	0.1258
Qatar	2000	Multiple fractures, dislocations, crashes, wounds, sprains, and strains	0.1037
Qatar	2005	Multiple fractures, dislocations, crashes, wounds, sprains, and strains	0.0699
Qatar	2010	Multiple fractures, dislocations, crashes, wounds, sprains, and strains	0.0577
Qatar	2017	Multiple fractures, dislocations, crashes, wounds, sprains, and strains	0.0577
Qatar	1990	Amputation of upper limb, unilateral	0.0568
Qatar	1995	Amputation of upper limb, unilateral	0.0546
Qatar	2000	Amputation of upper limb, unilateral	0.0495
Qatar	2005	Amputation of upper limb, unilateral	0.0418
Qatar	2010	Amputation of upper limb, unilateral	0.039
Qatar	2017	Amputation of upper limb, unilateral	0.039
Qatar	1990	Amputation of thumb	0.0114
Qatar	1995	Amputation of thumb	0.0114
Qatar	2000	Amputation of thumb	0.0114
Qatar	2005	Amputation of thumb	0.0114
Qatar	2010	Amputation of thumb	0.0114
Qatar	2017	Amputation of thumb	0.0114
Qatar	1990	Amputation of toe/toes	0.0059
Qatar	1995	Amputation of toe/toes	0.0059
Qatar	2000	Amputation of toe/toes	0.0059
Qatar	2005	Amputation of toe/toes	0.0059
Qatar	2010	Amputation of toe/toes	0.0059
Qatar	2017	Amputation of toe/toes	0.0059
Qatar	1990	Burns, <20% total burned surface area without lower airway burns	0.0163
Qatar	1995	Burns, <20% total burned surface area without lower airway burns	0.0163
Qatar	2000	Burns, <20% total burned surface area without lower airway burns	0.0163
Qatar	2005	Burns, <20% total burned surface area without lower airway burns	0.0163
Qatar	2010	Burns, <20% total burned surface area without lower airway burns	0.0163
Qatar	2017	Burns, <20% total burned surface area without lower airway burns	0.0163
Qatar	1990	Burns, >=20% total burned surface area or >= 10% burned surface area if head/neck or hands/wrist involved w/o lower airway burns	0.2068
Qatar	1995	Burns, >=20% total burned surface area or >= 10% burned surface area if head/neck or hands/wrist involved w/o lower airway burns	0.198
Qatar	2000	Burns, >=20% total burned surface area or >= 10% burned surface area if head/neck or hands/wrist involved w/o lower airway burns	0.1774
Qatar	2005	Burns, >=20% total burned surface area or >= 10% burned surface area if head/neck or hands/wrist involved w/o lower airway burns	0.146
Qatar	2010	Burns, >=20% total burned surface area or >= 10% burned surface area if head/neck or hands/wrist involved w/o lower airway burns	0.1346
Qatar	2017	Burns, >=20% total burned surface area or >= 10% burned surface area if head/neck or hands/wrist involved w/o lower airway burns	0.1346
Saudi Arabia	1990	Amputation of lower limbs, bilateral	0.2078
Saudi Arabia	1995	Amputation of lower limbs, bilateral	0.1962
Saudi Arabia	2000	Amputation of lower limbs, bilateral	0.1852
Saudi Arabia	2005	Amputation of lower limbs, bilateral	0.1698
Saudi Arabia	2010	Amputation of lower limbs, bilateral	0.1463
Saudi Arabia	2017	Amputation of lower limbs, bilateral	0.1232
Saudi Arabia	1990	Lower airway burns	0.2252
Saudi Arabia	1995	Lower airway burns	0.2252
Saudi Arabia	2000	Lower airway burns	0.2252
Saudi Arabia	2005	Lower airway burns	0.2252
Saudi Arabia	2010	Lower airway burns	0.2252
Saudi Arabia	2017	Lower airway burns	0.2252
Saudi Arabia	1990	Dislocation of hip	0.0159
Saudi Arabia	1995	Dislocation of hip	0.0159
Saudi Arabia	2000	Dislocation of hip	0.0159
Saudi Arabia	2005	Dislocation of hip	0.0159
Saudi Arabia	2010	Dislocation of hip	0.0159
Saudi Arabia	2017	Dislocation of hip	0.0159
Saudi Arabia	1990	Dislocation of knee	0.1134
Saudi Arabia	1995	Dislocation of knee	0.1134
Saudi Arabia	2000	Dislocation of knee	0.1134
Saudi Arabia	2005	Dislocation of knee	0.1134
Saudi Arabia	2010	Dislocation of knee	0.1134
Saudi Arabia	2017	Dislocation of knee	0.1134
Saudi Arabia	1990	Dislocation of shoulder	0.062
Saudi Arabia	1995	Dislocation of shoulder	0.062
Saudi Arabia	2000	Dislocation of shoulder	0.062
Saudi Arabia	2005	Dislocation of shoulder	0.062
Saudi Arabia	2010	Dislocation of shoulder	0.062
Saudi Arabia	2017	Dislocation of shoulder	0.062
Saudi Arabia	1990	Muscle and tendon injuries, including sprains and strains lesser dislocations	0.0075
Saudi Arabia	1995	Muscle and tendon injuries, including sprains and strains lesser dislocations	0.0075
Saudi Arabia	2000	Muscle and tendon injuries, including sprains and strains lesser dislocations	0.0075
Saudi Arabia	2005	Muscle and tendon injuries, including sprains and strains lesser dislocations	0.0075
Saudi Arabia	2010	Muscle and tendon injuries, including sprains and strains lesser dislocations	0.0075
Saudi Arabia	2017	Muscle and tendon injuries, including sprains and strains lesser dislocations	0.0075
Saudi Arabia	1990	Fracture of clavicle, scapula, or humerus	0.0349
Saudi Arabia	1995	Fracture of clavicle, scapula, or humerus	0.0349
Saudi Arabia	2000	Fracture of clavicle, scapula, or humerus	0.0349
Saudi Arabia	2005	Fracture of clavicle, scapula, or humerus	0.0349
Saudi Arabia	2010	Fracture of clavicle, scapula, or humerus	0.0349
Saudi Arabia	2017	Fracture of clavicle, scapula, or humerus	0.0349
Saudi Arabia	1990	Fracture of face bones	0.0669
Saudi Arabia	1995	Fracture of face bones	0.0669
Saudi Arabia	2000	Fracture of face bones	0.0669
Saudi Arabia	2005	Fracture of face bones	0.0669
Saudi Arabia	2010	Fracture of face bones	0.0669
Saudi Arabia	2017	Fracture of face bones	0.0669
Saudi Arabia	1990	Fracture of foot bones except ankle	0.0264
Saudi Arabia	1995	Fracture of foot bones except ankle	0.0264
Saudi Arabia	2000	Fracture of foot bones except ankle	0.0264
Saudi Arabia	2005	Fracture of foot bones except ankle	0.0264
Saudi Arabia	2010	Fracture of foot bones except ankle	0.0264
Saudi Arabia	2017	Fracture of foot bones except ankle	0.0264
Saudi Arabia	1990	Fracture of hand (wrist and other distal part of hand)	0.0138
Saudi Arabia	1995	Fracture of hand (wrist and other distal part of hand)	0.0138
Saudi Arabia	2000	Fracture of hand (wrist and other distal part of hand)	0.0138
Saudi Arabia	2005	Fracture of hand (wrist and other distal part of hand)	0.0138
Saudi Arabia	2010	Fracture of hand (wrist and other distal part of hand)	0.0138
Saudi Arabia	2017	Fracture of hand (wrist and other distal part of hand)	0.0138
Saudi Arabia	1990	Fracture of hip	0.1695
Saudi Arabia	1995	Fracture of hip	0.1542
Saudi Arabia	2000	Fracture of hip	0.1397
Saudi Arabia	2005	Fracture of hip	0.1193
Saudi Arabia	2010	Fracture of hip	0.0882
Saudi Arabia	2017	Fracture of hip	0.0577
Saudi Arabia	1990	Amputation of upper limbs, bilateral	0.2031
Saudi Arabia	1995	Amputation of upper limbs, bilateral	0.1873
Saudi Arabia	2000	Amputation of upper limbs, bilateral	0.1723
Saudi Arabia	2005	Amputation of upper limbs, bilateral	0.1513
Saudi Arabia	2010	Amputation of upper limbs, bilateral	0.1191
Saudi Arabia	2017	Amputation of upper limbs, bilateral	0.0876
Saudi Arabia	1990	Fracture of patella, tibia or fibula, or ankle	0.0554
Saudi Arabia	1995	Fracture of patella, tibia or fibula, or ankle	0.0554
Saudi Arabia	2000	Fracture of patella, tibia or fibula, or ankle	0.0554
Saudi Arabia	2005	Fracture of patella, tibia or fibula, or ankle	0.0554
Saudi Arabia	2010	Fracture of patella, tibia or fibula, or ankle	0.0554
Saudi Arabia	2017	Fracture of patella, tibia or fibula, or ankle	0.0554
Saudi Arabia	1990	Fracture of pelvis	0.1823
Saudi Arabia	1995	Fracture of pelvis	0.1823
Saudi Arabia	2000	Fracture of pelvis	0.1823
Saudi Arabia	2005	Fracture of pelvis	0.1823
Saudi Arabia	2010	Fracture of pelvis	0.1823
Saudi Arabia	2017	Fracture of pelvis	0.1823
Saudi Arabia	1990	Fracture of radius and/or ulna	0.0435
Saudi Arabia	1995	Fracture of radius and/or ulna	0.0435
Saudi Arabia	2000	Fracture of radius and/or ulna	0.0435
Saudi Arabia	2005	Fracture of radius and/or ulna	0.0435
Saudi Arabia	2010	Fracture of radius and/or ulna	0.0435
Saudi Arabia	2017	Fracture of radius and/or ulna	0.0435
Saudi Arabia	1990	Fracture of skull	0.0714
Saudi Arabia	1995	Fracture of skull	0.0714
Saudi Arabia	2000	Fracture of skull	0.0714
Saudi Arabia	2005	Fracture of skull	0.0714
Saudi Arabia	2010	Fracture of skull	0.0714
Saudi Arabia	2017	Fracture of skull	0.0714
Saudi Arabia	1990	Fracture of sternum and/or fracture of one or more ribs	0.1027
Saudi Arabia	1995	Fracture of sternum and/or fracture of one or more ribs	0.1027
Saudi Arabia	2000	Fracture of sternum and/or fracture of one or more ribs	0.1027
Saudi Arabia	2005	Fracture of sternum and/or fracture of one or more ribs	0.1027
Saudi Arabia	2010	Fracture of sternum and/or fracture of one or more ribs	0.1027
Saudi Arabia	2017	Fracture of sternum and/or fracture of one or more ribs	0.1027
Saudi Arabia	1990	Fracture of vertebral column	0.1106
Saudi Arabia	1995	Fracture of vertebral column	0.1106
Saudi Arabia	2000	Fracture of vertebral column	0.1106
Saudi Arabia	2005	Fracture of vertebral column	0.1106
Saudi Arabia	2010	Fracture of vertebral column	0.1106
Saudi Arabia	2017	Fracture of vertebral column	0.1106
Saudi Arabia	1990	Fracture of femur, other than femoral neck	0.042
Saudi Arabia	1995	Fracture of femur, other than femoral neck	0.042
Saudi Arabia	2000	Fracture of femur, other than femoral neck	0.042
Saudi Arabia	2005	Fracture of femur, other than femoral neck	0.042
Saudi Arabia	2010	Fracture of femur, other than femoral neck	0.042
Saudi Arabia	2017	Fracture of femur, other than femoral neck	0.042
Saudi Arabia	1990	Minor TBI	0.1319
Saudi Arabia	1995	Minor TBI	0.1319
Saudi Arabia	2000	Minor TBI	0.1319
Saudi Arabia	2005	Minor TBI	0.1319
Saudi Arabia	2010	Minor TBI	0.1319
Saudi Arabia	2017	Minor TBI	0.1319
Saudi Arabia	1990	Moderate TBI	0.1637
Saudi Arabia	1995	Moderate TBI	0.1637
Saudi Arabia	2000	Moderate TBI	0.1637
Saudi Arabia	2005	Moderate TBI	0.1637
Saudi Arabia	2010	Moderate TBI	0.1637
Saudi Arabia	2017	Moderate TBI	0.1637
Saudi Arabia	1990	Amputation of fingers (excluding thumb)	0.0047
Saudi Arabia	1995	Amputation of fingers (excluding thumb)	0.0047
Saudi Arabia	2000	Amputation of fingers (excluding thumb)	0.0047
Saudi Arabia	2005	Amputation of fingers (excluding thumb)	0.0047
Saudi Arabia	2010	Amputation of fingers (excluding thumb)	0.0047
Saudi Arabia	2017	Amputation of fingers (excluding thumb)	0.0047
Saudi Arabia	1990	Spinal cord lesion at neck level	0.4505
Saudi Arabia	1995	Spinal cord lesion at neck level	0.4452
Saudi Arabia	2000	Spinal cord lesion at neck level	0.4402
Saudi Arabia	2005	Spinal cord lesion at neck level	0.4331
Saudi Arabia	2010	Spinal cord lesion at neck level	0.4224
Saudi Arabia	2017	Spinal cord lesion at neck level	0.4118
Saudi Arabia	1990	Spinal cord lesion below neck level	0.2593
Saudi Arabia	1995	Spinal cord lesion below neck level	0.2481
Saudi Arabia	2000	Spinal cord lesion below neck level	0.2375
Saudi Arabia	2005	Spinal cord lesion below neck level	0.2226
Saudi Arabia	2010	Spinal cord lesion below neck level	0.1999
Saudi Arabia	2017	Spinal cord lesion below neck level	0.1777
Saudi Arabia	1990	Drowning and nonfatal submersion	0.2471
Saudi Arabia	1995	Drowning and nonfatal submersion	0.2471
Saudi Arabia	2000	Drowning and nonfatal submersion	0.2471
Saudi Arabia	2005	Drowning and nonfatal submersion	0.2471
Saudi Arabia	2010	Drowning and nonfatal submersion	0.2471
Saudi Arabia	2017	Drowning and nonfatal submersion	0.2471
[truncated: 12,521,753 more chars]
